# Whole‑exome sequencing reveals Lewis lung carcinoma is a hypermutated *Kras*/*Nras*–mutant cancer with extensive regional mutation clusters in its genome

**DOI:** 10.1038/s41598-023-50703-2

**Published:** 2024-01-02

**Authors:** Quan He, Cuirong Sun, Yuanjiang Pan

**Affiliations:** 1 Department of Chemistry, Zhejiang University, Hangzhou, 310058 Zhejiang China; 2 College of Pharmaceutical Sciences, Zhejiang University, Hangzhou, 310058 Zhejiang China

**Keywords:** Cancer genomics, Lung cancer, Animal disease models, Cancer models

## Abstract

Lewis lung carcinoma (LLC), as a widely used preclinical cancer model, has still not been genetically and genomically characterized. Here, we performed a whole–exome sequencing analysis on the LLC cell line to elucidate its molecular characteristics and etiologies. Our data showed that LLC originated from a male mouse belonging to C57BL/6L (a transitional strain between C57BL/6J and C57BL/6N) and contains substantial somatic SNV and InDel mutations (> 20,000). Extensive regional mutation clusters are present in its genome, which were caused mainly by the mutational processes underlying the SBS1, SBS5, SBS15, SBS17a, and SBS21 signatures during frequent structural rearrangements. Thirty three deleterious mutations are present in 30 cancer genes including *Kras*, *Nras*, *Trp53*, *Dcc*, and *Cacna1d*. *Cdkn2a* and *Cdkn2b* are biallelically deleted from the genome. Five pathways (RTK/RAS, p53, cell cycle, TGFB, and Hippo) are oncogenically deregulated or affected. The major mutational processes in LLC include chromosomal instability, exposure to metabolic mutagens, spontaneous 5–methylcytosine deamination, defective DNA mismatch repair, and reactive oxygen species. Our data also suggest that LLC is a lung cancer similar to human lung adenocarcinoma. This study lays a molecular basis for the more targeted application of LLC in preclinical research.

## Introduction

Lung cancer remains the most deadly cancer and is the leading cause of cancer–related deaths in the world^1,2^. The five-year survival rate for lung cancer is only 15%, and the deaths caused by this disease each year are more than those by breast, colon, prostate, and pancreas cancers together^3^. Therefore, there is an urgent need for developing effective drugs and therapies for lung cancer. In this regard, murine cancer models play an important role. The Lewis lung carcinoma (LLC) model is the only reproducible syngeneic murine model for lung cancer^4^. LLC originated spontaneously in the lung of a C57BL mouse in 1951 and was isolated from the mouse by Dr. Margaret Lewis^5^. Since then, this tumor line has been maintained via subcutaneous or intramuscular transplantation in laboratory settings for a long time^6^. In 1980, a cloned cell line adapted to cell culture was established^7^. To date, this cell line has been used extensively in preclinical studies. The established LLC cell line is anaplastic, highly tumorigenic, and immunologically compatible with the murine system^4,5,8,9^. The corresponding LLC model is syngeneic and can be created on an immune competent murine background such as C57BL, allowing the evaluation of true immune and toxicity responses with respect to tumor growth and cancer therapies^4,8^. In addition, when this model is used orthotopically, the tumor microenvironment can be reliably represented^4^. Therefore, the LLC model is valuable for the testing of chemotherapeutic agents such as vinorelbine and carboplatin^10–12^ and molecularly targeted agents such as sunitinib and erlotinib^13,14^. Upon inoculation of LLC cells, tumors grow rapidly in C57BL mice with high vascularization and metastasize to multiple organs and tissues such as lungs, lymph nodes, liver, myocardium, and pancreas^5,8^. Therefore, this model is also widely used to study tumor angiogenesis and metastasis^4,9^.

According to the statistics of PubMed, the LLC model has served over 5000 published studies since 1956. However, despite over seventy years of experimental usage, this model’s genetic variations and molecular characteristics still remain unknown^4^, which sharply contrasts to the extensive molecular characterization of human lung cancer. As a group, human lung cancer is a highly heterogeneous disease from the standpoint of genetics and genomics. A substantial number of human lung cancer genomes have been sequenced, and somatic mutations, genomic landscapes, molecular features, inter- and intra-tumoral heterogeneity, classifications, and evolutionary paths of human lung cancer have been published^3^. As genetic and genomic data of LLC has not been available, it is unclear how this cancer molecularly correlates to human lung cancer and to what extent it can be used as model. To answer these questions, we utilized the whole-exome sequencing (WES) technique to perform an overdue analysis of the origin, genetic variations, genomic landscape, and etiologies of the established LLC cell line, laying a molecular foundation for its more targeted application in cancer research and drug development.

## Results

### Data quality assessment

DNAs from two vials (LC01 and LC02) of an LLC cell culture sample were extracted and subjected to WES analysis. The quality of extracted DNAs, sequencing, alignment, and variant calling was evaluated (see Supplementary Table S1 online & Figs. S1–S2). The DNA concentrations are 30–80 ng/μL and the total DNA amounts are 2–7 μg, indicating the sufficiency of the extracted DNAs for library construction. The raw reads are 7–50 million and the raw data are 13.5–15.0 gigabytes, indicating successful library construction. The clean reads account for 98% of the raw reads, indicating a high content of high quality reads. The base error rates are 0.03%, and the percentages of bases with the Q_phred_ greater than 20 and 30 are 98% and 94%, respectively, indicating a high base quality for alignment. The GC contents are 47%, within the range of the general exomic GC content (45–55%)^15^. The results about the percentages of properly mapped reads (99%), the capture efficiency (62%), the average on–target sequencing depth (169–178), the exome coverage (99.9%), and the percentages of exome regions read at least 10 times (98.5%) indicate the reliability of alignment. The Ts/Tv ratio (1.7) is consistent with the general Ts/Tv ratio in the mouse genome (1.5–1.7)^16^, indicating a high overall quality of the identified SNVs. The quality score of variant calling is 20–228, providing statistical confidence for variant calling.

### The LLC genome

The normal adjacent tissue cannot be procured for WES analysis of the LLC cell line. By comparing our sequencing data with the previously reported mouse sequencing data (see the “Methods” & Supplementary Tables S2–S3 online), it was found that the LLC-derived C57BL mouse belongs to a transitional unknown subline between C57BL/6J and C57BL/6N. We termed this subline “C57BL/6L”, where “L” denotes “Lewis”. We also found that the C57BL/6L genome is genotypically extremely close to the C57BL/6J genome (see the “Methods”). Therefore, the somatic mutations including single nucleotide variations (SNVs) and short insertions and deletions (InDels) accumulated in LLC from its inception to the present were approximately identified by comparing the sequences of LLC and C57BL/6J. The mutations identified by this comparison can be divided into two categories: the mutations acquired during the development of LLC in the C57BL/6L mouse and the mutations accumulated during its long-term laboratory passaging from 1951 to the present.

The statistics of identified SNVs and InDels as well as mutation burdens are summarized in Table 1 & Supplementary Tables S4–S5. We identified 18,664 SNVs, including 5082 SNVs localized in coding regions. Nearly half of the SNVs in coding regions cause non-synonymous protein changes, including 2201 missense and 63 nonsense mutations. We also identified 2225 InDels, the majority of which are localized in non-coding regions (2127; 95.6%). Nearly two thirds of the InDels localized in coding regions cause non-synonymous protein changes, including 36 frameshift mutations and 25 nonframeshift mutations.

**Table 1:** Statistics of SNVs, InDels, and mutation burdens.

Mutation & TMB	LLC cell
**Total SNVs (Percentage)**	18,664 (100%)
Heterozygous SNVs (Percentage)	11,592 (62.1%)
Homozygous SNVs (Percentage)	7074 (37.9%)
**SNVs in dbSNP (Percentage)**	13,127 (70.3%)
**SNVs in coding regions (Percentage)**	5082 (27.2%)
Synonymous (Percentage)	2654 (14.2%)
Missense (Percentage)	2201 (11.8%)
Stopgains (Percentage)	63 (0.3%)
Stoplosses (Percentage)	0 (0.0%)
Unknowns (Percentage)	164 (0.9%)
**SNVs in non-coding regions (Percentage)**	13,584 (72.8%)
Intronic (Percentage)	10,879 (58.3%)
3′-UTR (Percentage)	657 (3.5%)
5′-UTR (Percentage)	263 (1.4%)
Splicing (Percentage)	145 (0.8%)
Upstream (Percentage)	199 (1.1%)
Downstream (Percentage)	133 (0.7%)
Intergenic (Percentage)	1306 (7.0%)
**Total InDels (Percentage)**	2225 (100%)
Heterozygous InDels (Percentage)	1361 (61.2%)
Homozygous InDels (Percentage)	864 (38.8%)
**InDels in dbSNP (Percentage)**	1412 (63.5%)
**InDels in coding regions (Percentage)**	98 (4.4%)
Frameshift deletions (Percentage)	23 (1.0%)
Frameshift insertions (Percentage)	13 (0.6%)
Nonframeshift deletions (Percentage)	11 (0.5%)
Nonframeshift insertions (Percentage)	14 (0.6%)
Stopgains (Percentage)	0 (0.0%)
Stoplosses (Percentage)	0 (0.0%)
Unknowns (Percentage)	37 (1.7%)
**InDels in non-coding regions (Percentage)**	2127 (95.6%)
Intronic (Percentage)	1738 (78.1%)
3′-UTR (Percentage)	112 (5.0%)
5′-UTR (Percentage)	18 (0.8%)
Splicing (Percentage)	35 (1.6%)
Upstream (Percentage)	26 (1.2%)
Downstream (Percentage)	28 (1.3%)
Intergenic (Percentage)	170 (7.6%)
**Total mutation burden (mut/Mb)**	178.3 ± 3.5
**Mutation burden in coding regions (mut/Mb)**	132.6 ± 0.6
**Mutation burden in non-coding regions (mut/Mb)**	201.1 ± 5.5

The average mutation burden is 178.3 mut per Mb (Table 1). The mutation burden in coding regions (132.6 mut per Mb) is much lower than that in non-coding regions (201.1 mut per Mb), which is in agreement with the notion that purifying selection is stronger in coding regions than in non-coding regions^17^.

**Figure 1:**
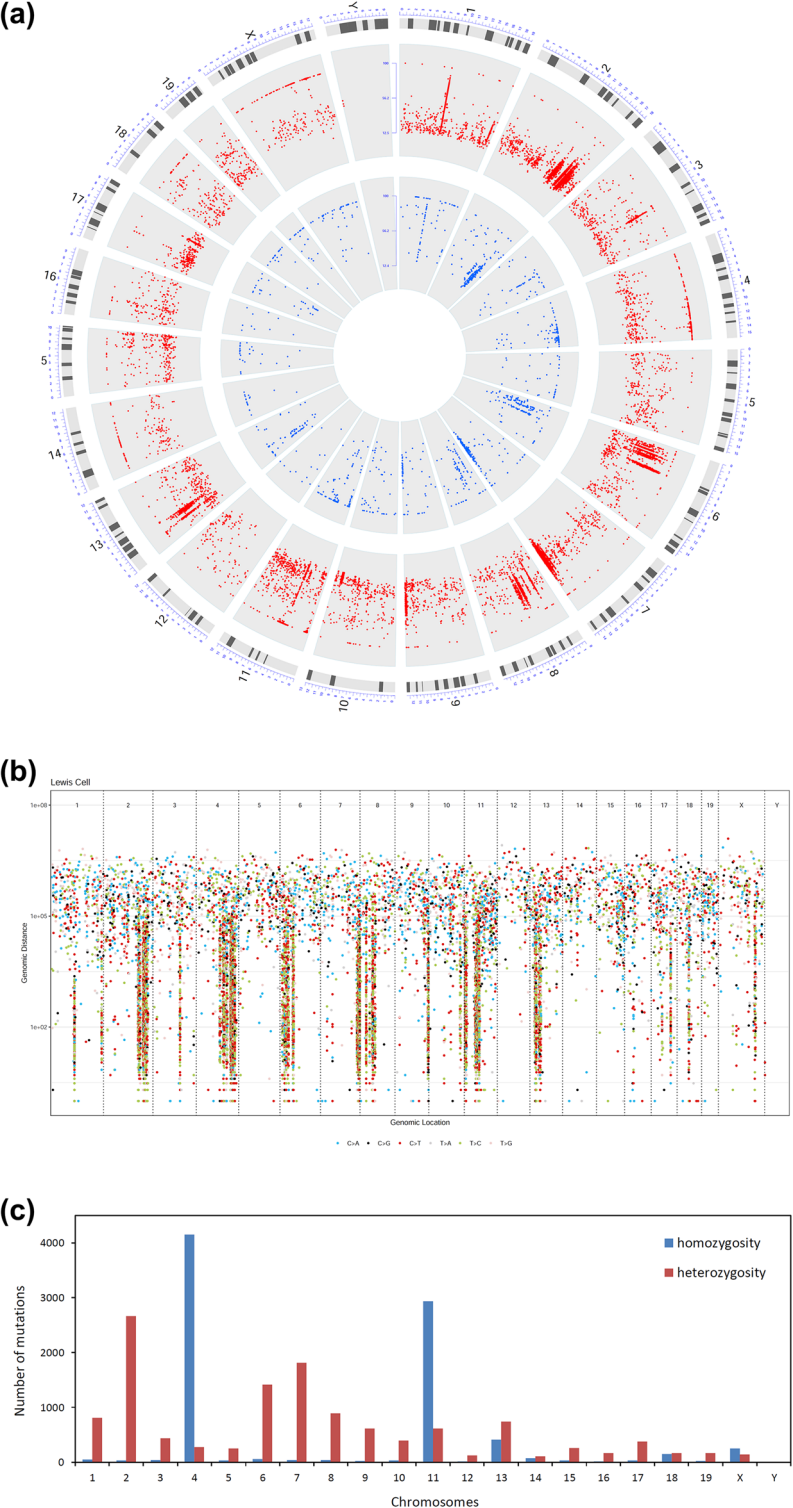
The distribution of the identified SNVs and InDels in the LLC genome. (**a**) Circos diagram in which tracks 1 (outer) and 2 (inner), plotted based on DNA allele frequency, represent SNV and InDel, respectively; (**b**) rainfall plot of the SNV mutations showing extensive regional mutation clusters; (**c**) histogram showing the distribution of homozygous and heterozygous mutations including both SNVs and InDels across 21 chromosomes.

Mutations were detected on chromosome Y (Fig. 1a & see Supplementary Table S4 online), suggesting that LLC originated from a male mouse. Mutations on chromosome X were detected with 100% and 50% allele frequency (Fig. 1a), suggesting that chromosome X in LLC is diploid.

Regional mutation clusters, which we defined as six or more consecutive base substitutions whose intermutation distances are less than or equal to a predefined number (1 kb, 5 kb, and 10 kb), are prevalent in the LLC genome, especially on chromosomes 2, 4, 6, 7, 8, 9, 11, and 13 (Fig. 1b), indicating an uneven mutation distribution within the genome. The regions of regional mutation clusters often colocalize with the regions of somatic structural rearrangements^18^. Thus, the prevalence of regional mutation clusters suggests that LLC could experience frequent structural rearrangements in its genome.

Of the SNVs and InDels identified, approximately 38% (Table 1) are homozygous mutations (100% allele frequency), which can be the result of either a loss of heterozygosity (LOH) onco-transformation or genetic drift in a C57BL mouse followed by inbreeding. Notably, the majority of the homozygous mutations are located on chromosomes 4 and 11 (Fig. 1c) and the regions in which homozygous mutations cluster coincide with the regions of regional mutation clusters (see Supplementary Fig. S3 online), suggesting that the homozygosity of these mutations can be caused by the LOH events occurring during the structural rearrangements.

### Mutation signatures

**Figure 2:**
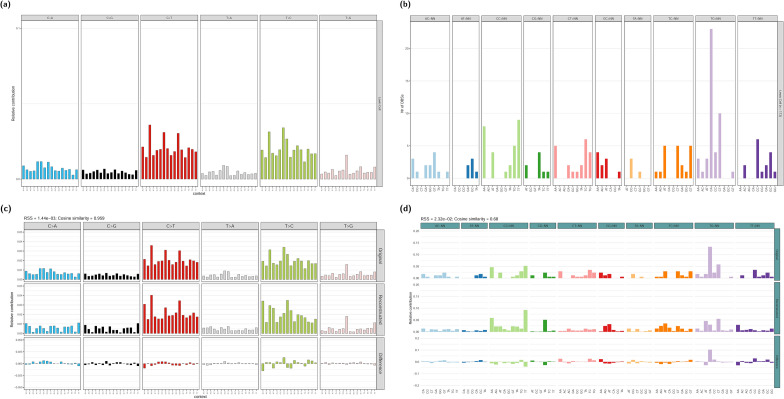
The original and reconstructed mutational profiles of LLC. (**a**) The original SBS profile of LLC in the 96 trinucleotide context; (**b**) the original DBS profiles of LLC; (**c**) the reconstructed SBS profile of LLC using the identified contributing signatures SBS1, SBS5, SBS17a, SBS17b, and SBS21; and (**d**) the reconstructed DBS profile of LLC using the identified contributing signatures DBS2, DBS6, DBS7, DBS9, DBS10, and DBS11.

The single base substitution (SBS) and doublet base substitution (DBS) profiles of LLC are plotted in Fig. 2a, b. Single base substitutions are dominated by C>T (33%) and T>C (32%) mutations (see Supplementary Fig. S4 online). The SBS profile of LLC is similar to the COSMIC signature SBS5 (Cosine similarity: 0.869; see Supplementary Fig. S5 online), indicating that SBS5 is a main component of the SBS profile.

A signature refitting method using the COSMIC signatures system was applied to resolve the contributing signatures (see the “Methods” & Supplementary Table S6 online). As a result, six SBS signatures, namely SBS1, SBS5, SBS17a, SBS17b, SBS15, and SBS21, were found to contribute to the SBS profile of LLC (Table 2). These signatures were used to reconstruct the SBS profile of LLC (Fig. 2c). The cosine similarity between the original and reconstructed SBS profiles is 0.959, indicating that the SBS profile of LLC can be well explained by the signatures identified.

**Table 2:** The signatures identified in the SBS and DBS profiles of LLC.

Mutational profile	Identified signature	Contribution (%)	Etiology (quoted from COSMIC)
SBS profile	SBS1	8.4	Deamination of 5-methylcytosine to thymine
	SBS5	74.0	Putative metabolic mutagen
	SBS17a	4.9	Unknown
	SBS17b	2.0	Unknown
	SBS15	5.1	Defective DNA mismatch repair
	SBS21	5.6	Defective DNA mismatch repair
DBS profile	DBS2	7.8	Exposure to tobacco smoking as well as other endogenous and/or exogenous mutagens
	DBS6	18.6	Unknown
	DBS7	11.2	Defective DNA mismatch repair
	DBS9	19.6	Unknown
	DBS10	15.8	Defective DNA mismatch repair
	DBS11	27.0	Unknown. Possibly related to APOBEC mutagenesis. Reactive oxygen species can also result in CC > TT mutations

We found that SBS1, SBS5, SBS15, SBS17a, and SBS21 contribute to the mutational profiles of both the genomic regions with and without regional mutation clusters, while SBS17b only contributes to the mutational profile of the regions without regional mutation clusters (see Supplementary Table S7 online). It is worth noting that regardless of whether the intermutation distance threshold for regional mutation clusters was set to 1, 5, or 10 kb, the contributions of SBS1, SBS5, and SBS21 to the mutational profile of the regions with regional mutation clusters are higher than their contributions to the mutational profile of the regions without regional mutation clusters, and the contributions of SBS15, SBS17a, and SBS17b to the mutational profile of the regions with regional mutation clusters are lower than their contributions to the mutational profile of the regions without regional mutation clusters (see Supplementary Table S7 online), suggesting that the mutational processes of SBS1, SBS5, and SBS21 have a stronger preference for acting on the regions with frequent structural rearrangements over acting on the regions without frequent structural rearrangements than those of SBS15, SBS17a, and SBS17b.

Similarly, the DBS signatures contributing to the DBS profile of LLC were identified as DBS2, DBS6, DBS7, DBS9, DBS10, and DBS11 (Table 2 & see Supplementary Table S8 online), and the cosine similarity between the original and reconstructed DBS profiles is 0.68 (Fig. 2d), indicating that the DBS profile of LLC is only partially explained by the DBS signatures identified.

### Onco-relevant mutations

We detected 33 deleterious mutations from 30 cancer genes (Table 3), which is at the same level as human lung cancers (1–28 driver events)^19^. Among these mutations, *Kras*^G12C^ and *Nras*^Q61H^ are canonical driver mutations. The canonical tumor suppressor gene (TSG) *Trp53* was found to contain two mutations (*Trp53*^E32X^ and *Trp53*^R334P^), suggesting that *Trp53* has been biallelically inactivated. Two other TSGs, i.e., *Dcc* and *Cacna1d*, also have two deleterious mutations each. In addition, we found the homozygous deletion of *Cdkn2a* and *Cdkn2b* from the LLC genome (see Supplementary Fig. S6 online).

**Table 3:** The deleteriously mutated cancer genes in LLC.

Cancer gene	CHROM	POS	REF	ALT	Amino acid change	Sequencing depth	Mapping quality	Mutation quality	Zygosity	Gene role	Exonic function
*Akap9*	5	3956187	C	T	R171X	93	48	221	Heterozygous	ND*	Stopgain
*Arid1b*	17	5336858	G	T	G1477C	71	49	165	Heterozygous	TSG	Missense
*Bard1*	1	71067145	T	C	D441G	127	49	213	Heterozygous	TSG	Missense
*Cacna1d*	14	30075160	T	A	R1472X	59	47	222	Heterozygous	TSG	Stopgain
*Cacna1d*	14	30196850	T	C	Y166C	46	47	222	Heterozygous	TSG	Missense
*Cant1*	11	118407996	C	A	D314Y	81	49	221	Heterozygous	Fusion	Missense
*Csmd3*	15	47606172	A	G	L3444S	130	48	221	Heterozygous	ND	Missense
*Dcc*	18	71306114	A	AC	P1252fs	65	47	215	Heterozygous	TSG	Frameshift
*Dcc*	18	71384176	C	G	G803R	126	47	221	Heterozygous	TSG	Missense
*Ect2l*	10	18163420	C	G	G422A	141	48	221	Heterozygous	ND	Missense
*Epha3*	16	63611044	A	G	L499P	72	48	222	Heterozygous	TSG	Missense
*Ext2*	2	93704483	C	A	K645N	84	48	222	Heterozygous	TSG	Missense
*Fat4*	3	38889217	G	T	R753I	166	48	176	Heterozygous	TSG	Missense
*Fkbp9*	6	56868836	A	G	S386G	88	48	217	Heterozygous	Oncogene	Missense
*Herpud1*	8	94392420	G	C	W267C	136	48	221	Heterozygous	ND	Missense
*Hsp90ab1*	17	45568996	C	A	K531N	92	49	110	Heterozygous	Oncogene	Missense
*Kras*	6	145246772	C	A	G12C	138	42	221	Heterozygous	Oncogene	Missense
*Myh9*	15	77813135	C	A	W26C	80	47	222	Heterozygous	Oncogene	Missense
*N4bp2*	5	65794557	TC	T	I435fs	141	50	191	Heterozygous	ND	Frameshift
*Nfkb2*	19	46306829	C	T	A68V	139	46	221	Heterozygous	Oncogene	Missense
*Nras*	3	103060272	A	T	Q61H	76	47	221	Heterozygous	Oncogene	Missense
*Prdm1*	10	44446836	C	A	R206M	33	47	203	Heterozygous	TSG	Missense
*Ptprc*	1	138088568	G	T	R682S	275	48	221	Heterozygous	ND	Missense
*Ptprd*	4	76050347	C	T	R919H	162	48	215	Heterozygous	TSG	Missense
*Rgs7*	1	175086218	C	A	S285I	233	49	24	Heterozygous	TSG	Missense
*Sox2*	3	34650888	G	T	R158L	64	47	221	Heterozygous	Oncogene	Missense
*Syk*	13	52640702	G	C	A491P	152	48	221	Heterozygous	Oncogene	Missense
*Tcf3*	10	80412919	T	A	Q576L	20	48	157	Heterozygous	ND	Missense
*Tgfbr2*	9	116131545	C	A	W113L	84	49	89	Heterozygous	TSG	Missense
*Trim33*	3	103341716	T	A	C818X	97	49	166	Heterozygous	TSG	Stopgain
*Trp53*	11	69587267	G	T	E32X	178	47	221	Heterozygous	TSG	Stopgain
*Trp53*	11	69590672	G	C	R334P	147	48	196	Heterozygous	TSG	Missense
*Wnk2*	13	49070977	C	T	R1134Q	84	48	221	Heterozygous	TSG	Missense

*ND, not determined.

**Figure 3:**
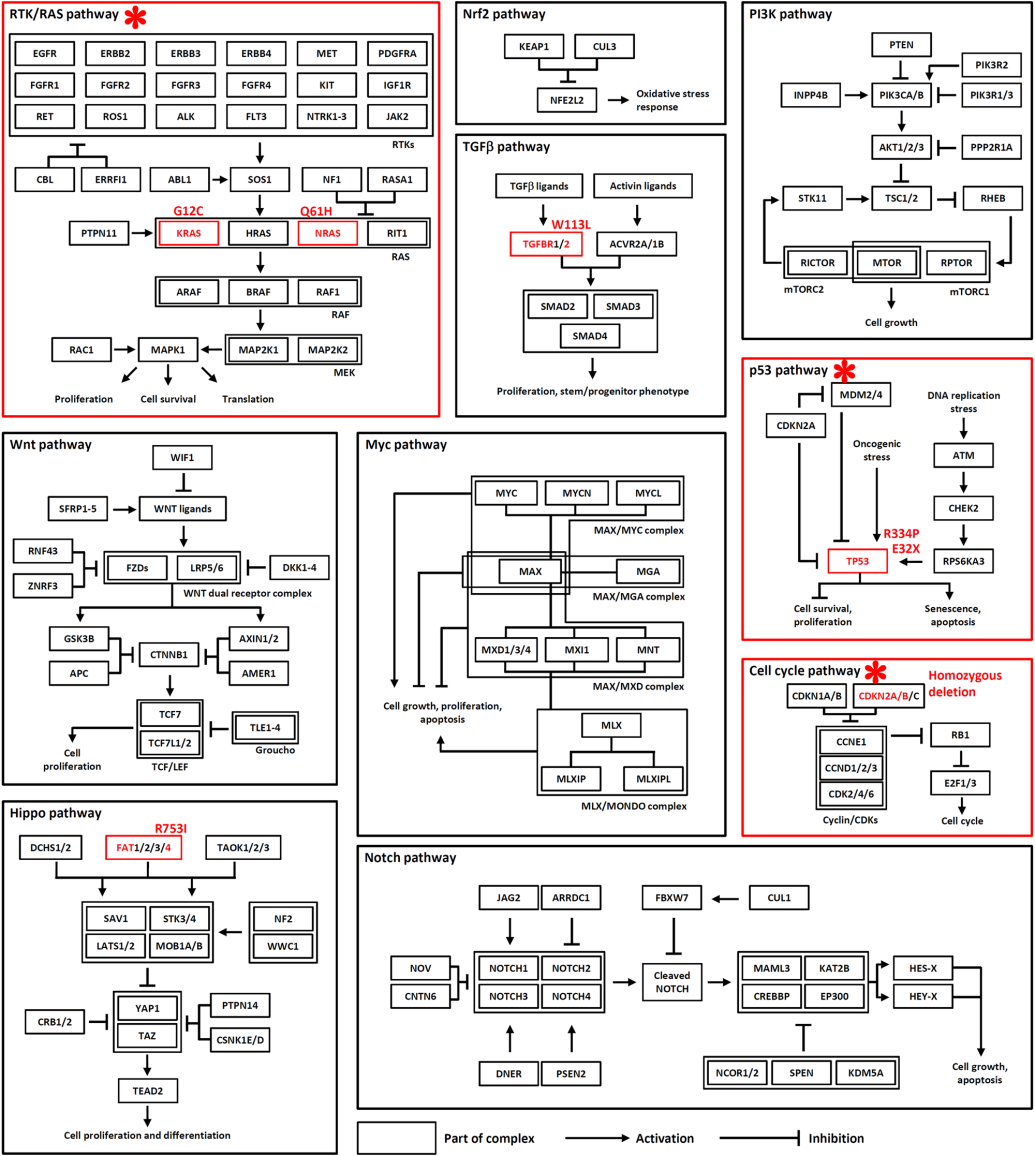
Mapping the identified mutated cancer genes to ten canonical oncogenic pathways. The deregulated oncogenic pathways in LLC are highlighted by a red * sign and a red frame. The oncogenically mutated genes are highlighted in red and their mutation types are indicated next to them. The curated oncogenic pathways are reprinted from Cell, 173, Sanchez–Vega F, Mina M, Armenia J, Chatila WK, Luna A, La KC, et al., Oncogenic signaling pathways in The Cancer Genome Atlas, 321–37.e10, Copyright (2018), with permission from Elsevier.

We mapped the mutated cancer genes identified to ten canonical oncogenic pathways^20^ (Fig. 3) and found that the RTK/RAS, p53, and cell cycle pathways are oncogenically altered. In addition, the TGFB and Hippo pathways can also be oncogenically affected by two TSG mutations *Tgfbr2*^W113L^ and *Fat4*^R753I^, respectively.

Notably, several chromosomal instability (CIN) related genes^21,22^ were found to bear missense and/or nonsense mutations, including *Trp53*^E32X^, *Trp53*^R334P^, *Bub1*^R187K^, *Cenpf*^K1540M^, and *Ttk*^E731X^ (see Supplementary Table S4 online), suggesting checkpoint defect and cell cycle dysregulation in LLC, which play important roles in the development of CIN. This is in agreement with the observation of frequent structural rearrangements indicated by extensive regional mutation clusters across the genome and the diploidy of chromosome X.

Mutations were found in multiple human lung adenocarcinoma (LUAD) related (cancer) genes, including *Kras*^23^, *Nras*^24^, *Csmd3*^25^, *Dcc*^25^, *Epha3*^26^, *Arid1b*^27^, *Arid2*^3^, *Ptprc*^24^, *Ptprd*^28^, *Akap9*^28^, *Setd2*^29^, and *Pask*^29^ (see Supplementary Table S4 online), while we did not find mutations in typical lung squamous cell carcinoma (LUSC) related genes, such as *Mll2*, *Nfel2*, *Pik3ca*, and *Pten*^3^, suggesting that LLC is a lung cancer similar to human LUAD. This is in agreement with Garcia-Sanz’s study which suggests that LLC is a bronchioloalveolar carcinoma (an uncommon LUAD subtype) derived from granular type II pneumocytes^6,30^.

In addition to the above cancer gene mutations, 566 deleterious non–cancer gene mutations, including 527 SNVs and 39 InDels, were identified (see Supplementary Tables S9–S10 online).

## Discussion

Our data indicated that Lewis lung carcinoma is a *Kras*/*Nras*–mutant lung cancer derived from a male C57BL/6L mouse and that it is similar to human LUAD. The high mutation burden observed in LLC (Table 1) is likely due to mutation accumulation during long–term laboratory passaging, suggesting that LLC has become a hypermutated cancer compared to the C57BL/6L mouse from which it was derived. Extensive regional mutation clusters were observed in its genome (Fig. 1b), and mutations in these clusters are contributed mainly by SBS1, SBS5, SBS17a, SBS15, and SBS21 (see Supplementary Table S7 online). Thirty three oncogenic mutations were found in 30 cancer genes (Table 3). *Cdkn2a* and *Cdkn2b* were found to be homozygously deleted (see Supplementary Fig. S6 online). The loss of *Cdkn2a* is often found in human lung cancers including LUAD and LUSC^3^. Oncogenic mutations in *KRAS* and *NRAS* are generally mutually exclusive and rarely observed simultaneously^31–34^, so the co-occurrence of *Kras*^G12C^ and *Nras*^Q61H^ in LLC is interesting and may result in a complex biological outcome that is beneficial for LLC tumorigenesis. TSGs are often genetically recessive and biallelic inactivation is often required to produce an oncogenic phenotype^35^. It is thus not surprising that the *Trp53*, *Dcc*, and *Cacna1d* TSGs contain two deleterious mutations, which suggests the biallelic inactivation of these genes. Further studies are needed for the roles and cooperativity of the identified cancer gene mutations. Accordingly, the RTK/RAS, p53, and cell cycle oncogenic pathways were found to be deregulated and the TGFB and Hippo pathways can be oncogenically affected (Fig. 3). In addition, hundreds of deleterious somatic SNV and InDel mutations were found in non–cancer genes. In consequence, all these cancer and non-cancer gene mutations, to a greater or lesser extent, can be involved in the rewiring of the cellular metabolism and thus determine the biological characteristics of the LLC model used today.

In the identified mutational signatures, SBS5 makes the largest contribution (74.0%) to the SBS profile of LLC (Table 2). The etiology of SBS5 still remains unknown, but it has been proposed to result from exposure to a ubiquitous metabolic mutagen^36,37^. SBS1, which is associated with spontaneous deamination of 5–methylcytosine to thymine, makes the second largest contribution (9.0%) to the SBS profile of LLC. Both SBS5 and SBS1 are clock–like signatures and are present not only in cancer cells but also in normal cells. The high contributions of SBS5 and SBS1 reflects the accumulation of massive SBS5 and SBS1 mutations during the long-term laboratory passaging. The etiologies of SBS15, SBS21, DBS7, and DBS10, which are associated with each other and are often found together in the same sample, are related to DNA mismatch repair (MMR). DBS11, which makes the largest contribution (27.0%) to the DBS profile of LLC (Table 2), is mainly characterized by CC>TT mutations and can be caused by either APOBEC activity or reactive oxygen species. Since SBS2 and SBS13, which are related to APOBEC activity, were not detected in LLC, the CC>TT mutations in LLC were probably caused by reactive oxygen species. DBS2 is predominantly characterized by CC>AA mutations and its etiology is related to exposure to tobacco smoking and other endogenous or exogenous mutagens. Since SBS4, which is related to tobacco smoke mutagens, was not observed in LLC, the DBS2 mutations in LLC were probably caused by endogenous mutagens. The etiologies of SBS17a, SBS17b, DBS6, and DBS9 are still unknown. It is worth noting that SBS17b has zero contribution to the mutational profile of the regions with regional mutation clusters (see Supplementary Table S7 online), suggesting that the mutational process underlying SBS17b does not act on the regions with frequent structural rearrangements, which may provide a new clue to its unknown mechanism and thus deserve further exploration.

Our data also indicated that LLC is chromosomally instable. The mouse genome has an intrinsic instability, prone to chromosomal recombinational events and capable of surviving marked chromosomal reorganizations, which favors the mouse’s adaptation and survival but reduces its lifespan due to the frequent occurrence of mutational events^38^. LLC, as a murine cancer, should inherit this instability. In this study, we found that the CIN-related genes including *Trp53*, *Bub1*, *Cenpf*, and *Ttk* are deleteriously mutated (see Supplementary Table S4 online). In addition, the extensive regional mutation clusters (Fig. 1b) and the diploidy of chromosome X (Fig. 1a) suggest frequent structural rearrangements in the LLC genome, which are probably the results of CIN. The clustering of homozygous mutation on chromosomes 4 and 11 also suggest the presence of CIN, as they positionally coincide with the regional mutation clusters and thus are probably caused by the LOH events during the CIN-induced structural rearrangements (see Supplementary Fig. S3 online). However, SBS2 and SBS13, often observed in human cancers with chromosomal instability^39^ and associated with APOBEC activity, are not detected (Table 2), suggesting the loss or the low level of the APOBEC activity in LLC. Indeed, the Rodentia order has been bereft of APOBEC3A and APOBEC3B like enzymes^40^. Although APOBEC1 enzymes of some species including the mouse have been shown to be DNA mutators in vitro^40^, there is no evidence that APOBEC1 functions as a DNA mutator in vivo. In addition, studies have shown that the expression of APOBEC1 in the mouse lung is low^40^. Therefore, even if the DNA mutator activity of APOBEC1 is present in the mouse lung, it is likely to be low. Taken together, it is possible that there is low or no APOBEC activity in the murine LLC, so that SBS2 and SBS13 are not detected.

Taken together, the major molecular processes that shape the mutational landscape of LLC include: (1) chromosomal instability; (2) spontaneous 5–methylcytosine deamination (SBS1); (3) exposure to metabolic mutagens (SBS5 and DBS2); (4) defective DNA MMR (SBS15, SBS21, DBS7, and DBS10); and (5) reactive oxygen species (DBS11).

## Conclusions

Lewis lung carcinoma as a murine lung cancer model has been widely used in preclinical research for decades, but its mutational landscape remains unknown. This study performed a WES analysis to determine its molecular characteristics and etiologies. Our results showed that LLC is derived from a male C57BL/6L mouse and contains extensive somatic mutations and that this murine cancer is similar to human LUAD. Extensive regional mutation clusters were found in its genome, which were generated mainly by the mutational processes of SBS1, SBS5, SBS15, SBS17a, and SBS21 during the frequent structural rearrangements. Thirty cancer genes including *Kras*, *Nras*, *Trp53*, *Dcc*, and *Cacna1d* are deleteriously mutated, and *Cdkn2a* and *Cdkn2b* are biallelically deleted from the genome. Five pathways (RTK/RAS, p53, cell cycle, TGFB, and Hippo) were found to be oncogenically deregulated or affected. The major mutational processes of LLC include CIN, exposure to endogenous mutagens, spontaneous 5–methylcytosine deamination, defective MMR, and reactive oxygen species.

This study provides a molecular basis for a more targeted application of LLC in preclinical research. For example, our data suggest that LLC can be used as a cancer model for the development of drugs targeting *Kras*/*Nras*-mutant cancers. The RAS genes are known to be difficult therapeutic targets. Furthermore, LLC with its high mutation burden may find a role in the research and development of immunotherapies, as cancers with high mutation burden often respond positively to immunotherapy^41^. In fact, LLC has recently been used to study the synergistic effect of PD–1 blockade and Endostar^42^. With the elucidation of its mutational landscape, the value of LLC in immunotherapy research warrants further exploration.

One of the difficulties in determining the mutational profiles of animal cancer models is the lack of paired normal tissues. As shown by our work, many of these cancer models are derived from inbred animals whose genomes have been sequenced. Thus, accumulated somatic mutations in these cancer models can be identified by comparing their genomes with those of the corresponding animals. In this regard, the WES–based analysis framework established here may be valuable for the genomic characterization of other inbred animal cancer models.

## Methods

### LLC cell sample preparation

The LLC cell line (CSTR:19375.09.3101HUMTCHu205; lot number: 22123) was ordered from the Cell Resource Center of the Shanghai Institute of Biochemistry and Cell Biology (SIBCB). The LLC cell line stored at SIBCB was obtained from the NIH and has been through 5 passages at the SIBCB prior to our order. There is no passage record for the NIH LLC cell line. The ordered LLC cell line was maintained in 75 cm^2^ culture flasks in RPMI–1640 growth medium supplemented with 10% fetal bovine serum (FBS) in a 5% CO_2_ atmosphere at 37 °C. Upon reaching the logarithmic growth phase, the cells were rinsed briefly with phosphate–buffered saline (PBS, pH 7.0) and then trypsinized for four minutes with 0.25% trypsin solution containing 0.53 mM EDTA. After trypsinization, the cells were harvested in the PBS solution and then centrifuged at 800 g for four minutes to obtain the LLC cell samples.

### DNA extraction, quantification and quality assessment

DNA samples were extracted from two vials (LC01 and LC02) of an LLC cell culture sample using the QIAamp DNA FFPE Tissue Kit (Qiagen, Germany). The quality of the isolated genomic DNAs was verified using two methods in combination as follows: (1) DNA degradation and contamination were monitored on 1% agarose gels using the Agilent 5400 DNA/RNA Fragment Analyzer System; (2) DNA concentrations were measured using the Qubit® DNA Assay Kit in Qubit® 2.0 Flurometer (Invitrogen, USA).

### Exome sequencing and data processing

A total amount of 0.4–0.6 μg of DNA per sample at a concentration of > 30 ng/μL was used for sequencing library construction. Sequencing libraries were constructed using the Agilent SureSelect^XT^ Mouse All Exon V1 kit (Agilent Technologies, CA, USA) and index tags were added to each sample. Products were purified using the AMPure XP system (Beckman Coulter, Beverly, USA) and quantified using the Agilent high sensitivity DNA assay on the Agilent Bioanalyzer 2100 system. Clustering of index–coded samples was performed on a cBot Cluster Generation System using the Hiseq PE Cluster Kit (Illumina). After clustering, the prepared DNA libraries were sequenced on an Illumina Hiseq 4000 platform at Novogene (Beijing, China) and the 150 bp paired–end raw reads (raw data) were generated. Sequencing quality was assessed by raw read count, raw data size, clean read percentage, average error rate, average percentage of Q20 and Q30 bases, and GC percentage. The obtained raw data were in FastQ format. Subsequently, paired reads with adapter contamination, a percentage of uncertain bases greater than 10%, or a percentage of low–quality nucleotides greater than 50% (Q_phred_ score < 5) were discarded to obtain the clean (high–quality) reads. The resulting clean reads were mapped to the reference mouse genome (UCSC mm10 for C57BL/6J) using Burrows–Wheeler Aligner (BWA) (version 0.7.8–r455)^43^ and Samblaster (version 0.1.21)^44^ to generate the original mapping results (BAM files). When a paired read was mapped to multiple regions, BWA selected the most likely region. If two or more most likely regions were detected, BWA would choose one at random. Samblaster and Sambamba (version 0.4.7)^45^ were used to mark duplicate reads in the original BAM files. Samtools (version 1.0)^46^ was used to sort the original BAM files to get the sorted BAM files. The sorted BAM files were used for sequencing depth and coverage analysis. Mapping quality was assessed by mapping efficiency, average sequencing depth, and coverage of clean reads.

### Identifying the subline type of the LLC-derived C57BL mouse

The pedigree of the C57BL strain during 1921–1951 and the laboratory passaging of the LLC cancer model during 1951–1980 were summarized as follows^5–7,47–49^: In 1921, the C57BL strain was established; in 1937, the C57BL/6 and C57BL/10 sublines were separated; in 1947, C57BL/10J was established; in 1948, C57BL/6J was established at F24; in 1951, C57BL/6N was established at F32, and LLC was isolated from a C57BL mouse; from 1951 to 1979, LLC was maintained in the laboratory by subcutaneous or intramuscular transplantation; in 1980, the cloned LLC cell line adapted to cell culture was established and stored in cell banks. Therefore, there were three main C57BL sublines, i.e., C57BL/6J, C57BL/10J, and C57BL/6N, by 1951. The C57BL mouse from which LLC was derived may belong to one of these sublines. With the obtained sequencing data, we first investigated whether the LLC was derived from a C57BL/6 or a C57BL/10 mouse. Doran et al. identified 210 missense SNVs that are private only to C57BL/10J^50^. From the loci of these 210 SNVs, we selected 20 loci (one locus per chromosome) where our sequencing data have good read depths and base quality to check whether the bases at these loci of LLC are consistent with those of C57BL/6J or those of C57BL/10J (see Supplementary Table S2 online). It was found that all bases at these loci of LLC are consistent with those of C57BL/6J, indicating that LLC is not derived from a C57BL/10 mouse. Further, we compared LLC with C57BL/6J and C57BL/6N. Simon et al. reported the mutations at 35 genomic loci that distinguish C57BL/6J from C57BL/6N^48^. Therefore, we compared the bases of these loci between LLC, C57BL/6J, and C57BL/6N (see Supplementary Table S3 online). We found that the bases of 14 loci in LLC are identical to those in C57BL/6J, whereas the remaining bases in LLC are identical to those in C57BL/6N, suggesting that the C57BL mouse from which LLC originated probably belongs to a transitional subline (i.e., “C57BL/6L”) between C57BL/6J and C57BL/6N, in which some of the 35 loci are mutated while others remain unchanged. C57BL/6J is an F24 subline established in 1948 and C57BL/6N is an F32 subline established in 1951. Thus, there are seven generations between C57BL/6J and C57BL/6N, and the C57BL/6L subline should belong to one of these generations. All the bases at the abovementioned loci were examined by viewing the sorted BAM files in the Integrative Genomic Viewer (IGV) software (version 2.10.0).

### Identifying SNV and InDel mutations

The somatic SNV and InDel mutations accumulated in LLC from its inception to the present could be identified with very few errors by comparing the sequences of LLC and C57BL/6J, because the number of germline and somatic mutations accumulated from the C57BL/6J generation to the LLC-derived C57BL/6L mouse is small, which could be estimated as the sum of germline mutations accumulated from the C57BL/6J generation to the C57BL/6L generation and somatic mutations accumulated from the conception to death of a C57BL/6L mouse. First, the number of germline mutations accumulated from the C57BL/6J generation to the C57BL/6L generation was estimated by calculating the product of the germline mutation incidence in each basepair in each generation, the number of basepairs in the mouse genome, and the number of generations an inbred strain has undergone. The germline mutation incidence for mouse species has been estimated to be 5.7 × 10^–9^ mutations per basepair per generation^51^. The mouse genome contains 2.5 × 10^9^ basepairs^52^. Therefore, if C57BL/6L belongs to the 7th generation (F31) after C57BL/6J, the number of germline mutations accumulated from C57BL/6J to C57BL/6L was calculated to be approximately 100. Second, the number of somatic mutations accumulated from the conception to death of a healthy C57BL/6L mouse could be estimated by calculating the product of the somatic mutation incidence in each basepair in each mitosis, the number of basepairs in the mouse genome, and the number of somatic mitoses undergone by the mouse during its entire life. The somatic mutation incidence for mice in each basepair in each mitosis has been estimated to be 8.1 × 10^–9^ per basepair per mitosis^51^. The number of somatic mitoses from conception to death could be estimated by calculating the sum of the number of cell divisions during the development in the womb and the number of cell divisions necessary to maintain tissue homeostasis from birth to death. It has been estimated that the embryonic development of a mouse involves approximately 29 mitoses^51^. It has been suggested that Lewis lung carcinoma originates from a pulmonary alveolar cell^6^. The turnover rate of a mammalian pulmonary alveolar cell has been estimated to be 4–5 weeks^53^. The lifespan of a mouse is approximately two years^54^. Thus, the pulmonary alveolar cells should undergo 22–27 mitoses during the entire postnatal life of a mouse. Taken together, the number of somatic mitoses from conception to death of a mouse was estimated to be 51–56. Therefore, the number of the somatic mutations accumulated from conception to death for a C57BL/6L mouse was calculated to be 1030–1130. In total, the sum of germline and somatic mutations accumulated from the C57BL/6J generation to a healthy C57BL/6L mouse was estimated to be 1130–1230, which corresponds to the entire mouse genome (2,500 Mbp). In contrast, the number of mutations (> 20,000, Table 1) identified in LLC using the C57BL/6J genome sequence as a reference sequence corresponds to only a very small portion (100–120 Mbp) of the mouse genome. Therefore, among the > 20,000 mutations, the number of mutations derived from the above estimated 1130–1230 mutations is only 50–54. In other words, the vast majority of the SNV and InDel mutations detected by comparing the sequences of LLC and C57BL/6J are somatic mutations. According to the above argument, we identified the SNVs and InDels by comparing our sequencing data with the mm10 reference sequence using the mpileup and bcftools commands of Samtools (version 1.0)^46^. The filters for SNV/InDel mutation calling were set as follows: QUAL (variant quality) > 20, DP (depth) > 4, and MQ (mapping quality) > 30. ANNOVAR (version 2015Mar22)^55^ was used for functional annotation. The dbSNP142 database^56^ was used to characterize the detected variants. Gene transcript annotation databases, such as Consensus CDS, RefSeq, Ensembl, and UCSC, were used to determine amino acid alteration. SNVs and InDels that were annotated to be located in segmental duplication sequences, interspersed repeats, or low–complexity sequences and thus have low credibility were excluded. Only mutations shared by both LC01 and LC02 were considered. The numbers of the transitions (Ts) and transversions (Tv) in the identified SNVs were counted and Ts/Tv ratios were calculated. Circos plots were made for the detected SNVs and InDels based on their DNA allele frequency using the R package OmicCircos^57^ (Version 1.32.0) and the numbers of homozygous and heterozygous mutations in each of 21 chromosomes were accordingly counted.

### Mutation burden estimation

Mutation burden was defined as the number of mutations detected per megabase of the DNA regions studied. The mutation burden was calculated for exonic, non–exonic, and total regions (consisting of exonic and non–exonic regions), respectively. The mutations include SNVs and InDels. The numbers of bases within the exonic, non–exonic, and total regions of the sorted BAM files were determined using bamdst (version 1.0.9). The SNVs, InDels, and bases with a sequencing depth of at least 10× were used to calculate the mutation burdens. The mutation burden data were expressed as mean ± standard deviation (SD) of two independent samples (LC01 and LC02).

### Mapping human cancer genes to their mouse homologs

Human genes do not always have the same NCBI gene symbols as those of homologous mouse genes. Therefore, we mapped the 723 human cancer genes recorded in the Cancer Gene Census (CGC) database of COSMIC to the homologous mouse genes (see Supplementary Table S11 online). The homologene file was obtained from NCBI (https://ftp.ncbi.nih.gov/pub/HomoloGene/current/, accessed on May 19, 2021). The columns V1, V2, V3, and V4 in the homologene file record the homologene IDs, species IDs (human ID: 9606; mouse ID: 10090), NCBI Entrez gene IDs, and NCBI gene symbols, respectively. The human cancer gene file was obtained from the COSMIC CGC database (https://cancer.sanger.ac.uk/cosmic/census?tier=all#cloverview, accessed on May 20, 2021), which contains the information of 723 identified human cancer genes. Using these downloaded files, the human cancer genes were mapped to the corresponding homologous mouse genes according to their NCBI Entrez gene IDs and homologene IDs. Some human cancer genes, such as *ATF1* and *CYP2C8*, were mapped to multiple homologous mouse genes. A small number of human cancer genes, such as *AKAP9*, *CUX1*, and *ELN*, were mapped to the mouse genes with the same NCBI gene symbols such as *Akap9*, *Cux1*, and *Eln*, because no homologous mouse genes were found in the homologene file. Some human cancer genes, such as *COX6C* and *EIF1AX*, were mapped not only to the homologous mouse genes, but also to the mouse genes with the same NCBI gene symbols. Several human cancer genes, including *FUS*, *MAML2*, and *MUC4*, are not present in the homologene file, but the mouse genes with the same NCBI gene symbols were found in the homologene file. The mouse gene *Ranbp2* is not homologous to the human cancer gene *RANBP2*, but is homologous to the human cancer gene *RGPD3*. Some human cancer genes, such as *CLTCL1* and *DUX4L1*, were not mapped.

### Detecting cancer genes and associated mutations

Using the above human–mouse cancer gene mapping table, mutated cancer genes and associated SNV/InDel mutations present in both LC01 and LC02 were detected. Several types of mutations were excluded from the detected cancer gene mutations because they are likely to be neutral (tolerated) mutations: (1) the mutations present in the dbSNP142 with an associated minor allele frequency (MAF) value of ≥ 0.01 (1%); the MAF values can be found from the Ensembl Genome Browser (http://asia.ensembl.org/index.html); (2) the mutations that were predicted to be neutral or tolerated by the PROVEAN (Protein Variation Effect Analyzer) web server tool (http://provean.jcvi.org/index.php, accessed on January 12, 2022, equivalent to PROVEAN v1.1.3)^58^; this web server tool can provide the PROVEAN and SIFT (Sorting Intolerant from Tolerant) predictions for gene and protein mutations; mutations were considered as “deleterious” if they simultaneously met the following two criteria: their PROVEAN score ≤ – 2.5 and their SIFT score ≤ 0.05; mutations that did not simultaneously meet the above two criteria were considered as “neutral” or “tolerated”.

### Detecting homozygous gene deletion

We used control-FREEC^59^ (version 11.4) to estimate copy number variations (CNVs). Genes with their copy numbers estimated to be zero were then manually examined for their exome-seq reads in the IGV software (version 2.10.0). If no reads are found for a gene, it indicates that this gene is indeed homozygously deleted from the LLC genome.

### Detecting deleterious SNV/InDel mutations in non-cancer genes

The effects of the coding SNVs and InDels of the non–cancer genes on the biological functions of the corresponding proteins were evaluated using the above PROVEAN web server tool. The SNVs and InDels causing the gain or loss of a stop codon and the frameshift InDels predicted by the PROVEAN web server tool or annotated by ANNOVAR were also considered as deleterious mutations. The coding SNVs and InDels recorded in the dbSNP142 with an associated MAF value of ≥ 0.01 were not considered.

### Identifying deregulated oncogenic pathways

Sanchez–Vega et al.^20^ summarized and curated the most frequently altered cancer genes in ten canonical oncogenic pathways. Here, we manually mapped the identified mutated cancer genes to Sanchez–Vega’s results to identify the deregulated oncogenic pathways in LLC.

### Mutation spectrum and mutation signature analysis

The R package MutationalPatterns (version 3.2.0)^60^ was used for mutation spectrum and mutation signature analysis. The genomic distributions of the detected SNVs were visualized as rainfall plots. The SBS and DBS profiles of LLC were plotted. The contributing signatures were resolved and then they were used to reconstruct the SBS and DBS profiles. A signature–adding refitting method based on the algorithm of solving nonnegative least–squares constraints problem was applied with the COSMIC signatures system (v3.2—March 2021) to resolve the likely signatures contributing to the SBS and DBS profiles of LLC. The refitting method, which refits the mutational profile of a tumor sample to a set of COSMIC signatures, was described as follows (using the refitting of the SBS profile of LLC as an example): at the first round, the cosine similarity between each of the 60 SBS signatures and the SBS profile was calculated using the cos_sim_matrix function of MutationalPatterns (see Supplementary Table S6 online, columns A–B). SBS5 showed the highest cosine similarity (0.869), indicating that SBS5 makes the largest contribution to the SBS profile. Thus, at the second round, SBS5 was selected to combine with each of the remaining 59 SBS signatures to create 59 two–signature combinations. Each of these two–signature combinations was used to reconstruct the SBS profile, and the cosine similarity between the original and reconstructed SBS profiles was calculated (see Supplementary Table S6 online, columns C–D). The reconstructed SBS profile by the SBS5 + SBS1 combination has the highest cosine similarity (0.928) to the original SBS profile. Thus, at the third round, the SBS5 + SBS1 combination was selected to combine with each of the remaining 58 signatures, resulting in 58 three–signature combinations. The reconstruction of the SBS profile was performed again, and the reconstructed SBS profile by the SBS5 + SBS1 + SBS17a combination has the highest cosine similarity (0.943) to the original SBS profile (see Supplementary Table S6 online, columns E–F). The above refitting process was performed iteratively until the difference between the highest cosine similarity at the n–signature reconstruction round and that at the (n–1)–signature reconstruction round is less than the cutoff of 0.004. Using this approach, the main signatures contributing to the SBS profile of LLC were identified. This refitting approach was also applied with the cutoff of 0.006 to resolve the signatures contributing to the DBS profile of LLC (see Supplementary Table S8 online). Among the DBS signatures identified, DBS5 was not considered a contributing signature for LLC, because LLC as a spontaneously occurring cancer has not undergone the platinum chemotherapy treatment, which is the mechanism underlying DBS5. This refitting method differs from traditional refitting methods in that traditional refitting methods generally start with a limited number of signatures and a signature–removing strategy is used to identify the signatures involved in a tumor^61^.

### Supplementary Information

Supplementary Information.

## Data Availability

The raw sequence data (FastQ and BAM files) reported in this paper have been deposited in the Genome Sequence Archive (GSA) in National Genomics Data Center, China National Center for Bioinformation/Beijing Institute of Genomics, Chinese Academy of Sciences (GSA accession numbers: CRA008559 for FastQ data; CRA009497 for BAM data) that are publicly accessible at https://ngdc.cncb.ac.cn/gsa. The variation data (VCF files) reported in this paper have been deposited in the Genome Variation Map (GVM) in National Genomics Data Center, China National Center for Bioinformation/Beijing Institute of Genomics, Chinese Academy of Sciences (GVM accession number: GVM000472) that are publicly accessible at https://ngdc.cncb.ac.cn/gvm.
