# Supplementary material for: Whole‑exome sequencing reveals Lewis lung carcinoma is a hypermutated Kras/Nras–mutant cancer with extensive regional mutation clusters in its genome
Source: Sci Rep. 2024 Jan 2;14:100. doi: 10.1038/s41598-023-50703-2 (PMC10762126; doi:10.1038/s41598-023-50703-2)
Supplement: Supplementary file 1 — Supplementary Information. [file 41598_2023_50703_MOESM1_ESM.pdf]

# Whole-exome sequencing reveals Lewis lung carcinoma is a hypermutated *Kras/Nras*-mutant cancer with extensive regional mutation clusters in its genome

Quan He[1], Cuirong Sun[2]* & Yuanjiang Pan[1]*

[1] Department of Chemistry, Zhejiang University, Hangzhou, Zhejiang 310058, China
[2] College of Pharmaceutical Sciences, Zhejiang University, Hangzhou, Zhejiang 310058, China

* Email: suncuirong@zju.edu.cn; cheyjpan@zju.edu.cn

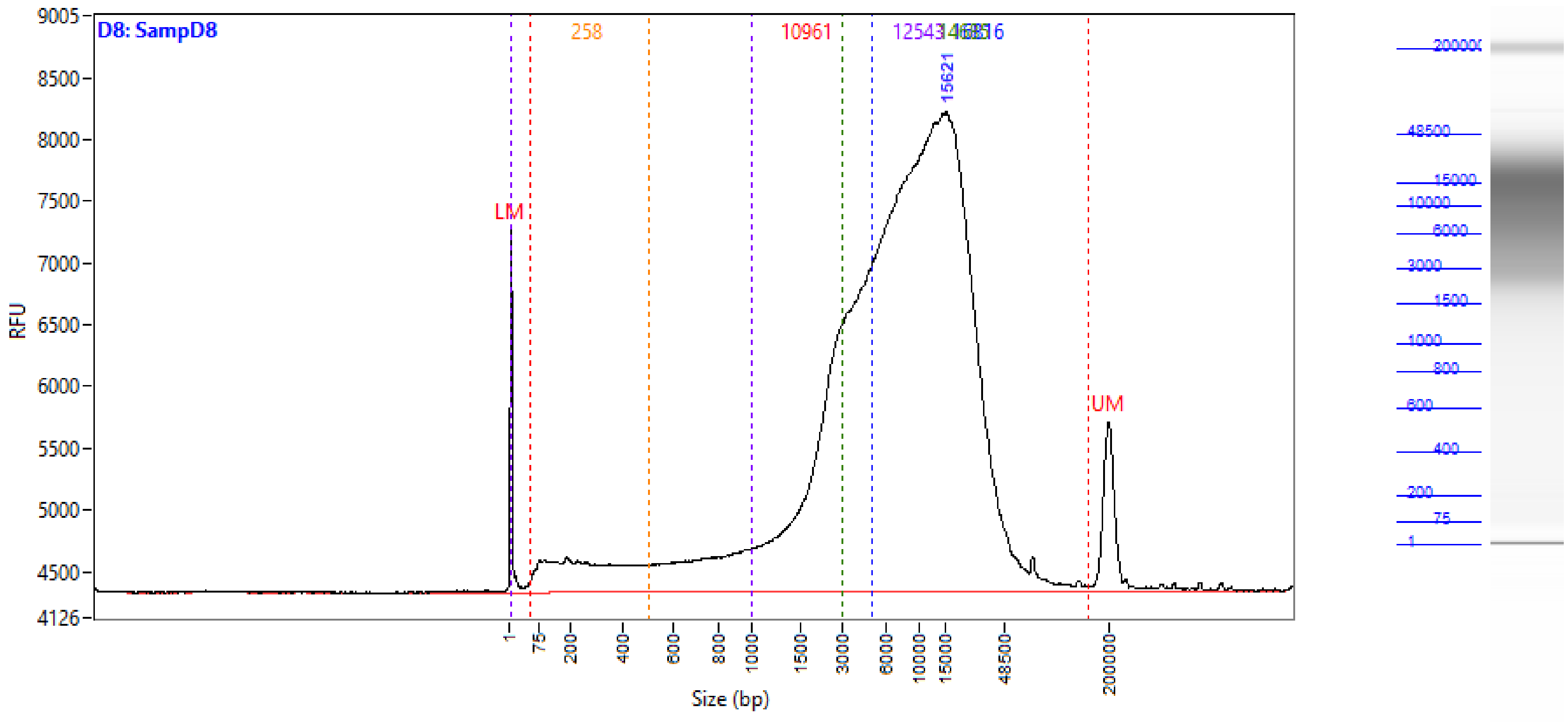


**Supplementary Figure S1.** The fragment analysis of the DNA sample extracted from the LLC cell sample LC01 by Agilent 5400 DNA/RNA Fragment Analyzer.

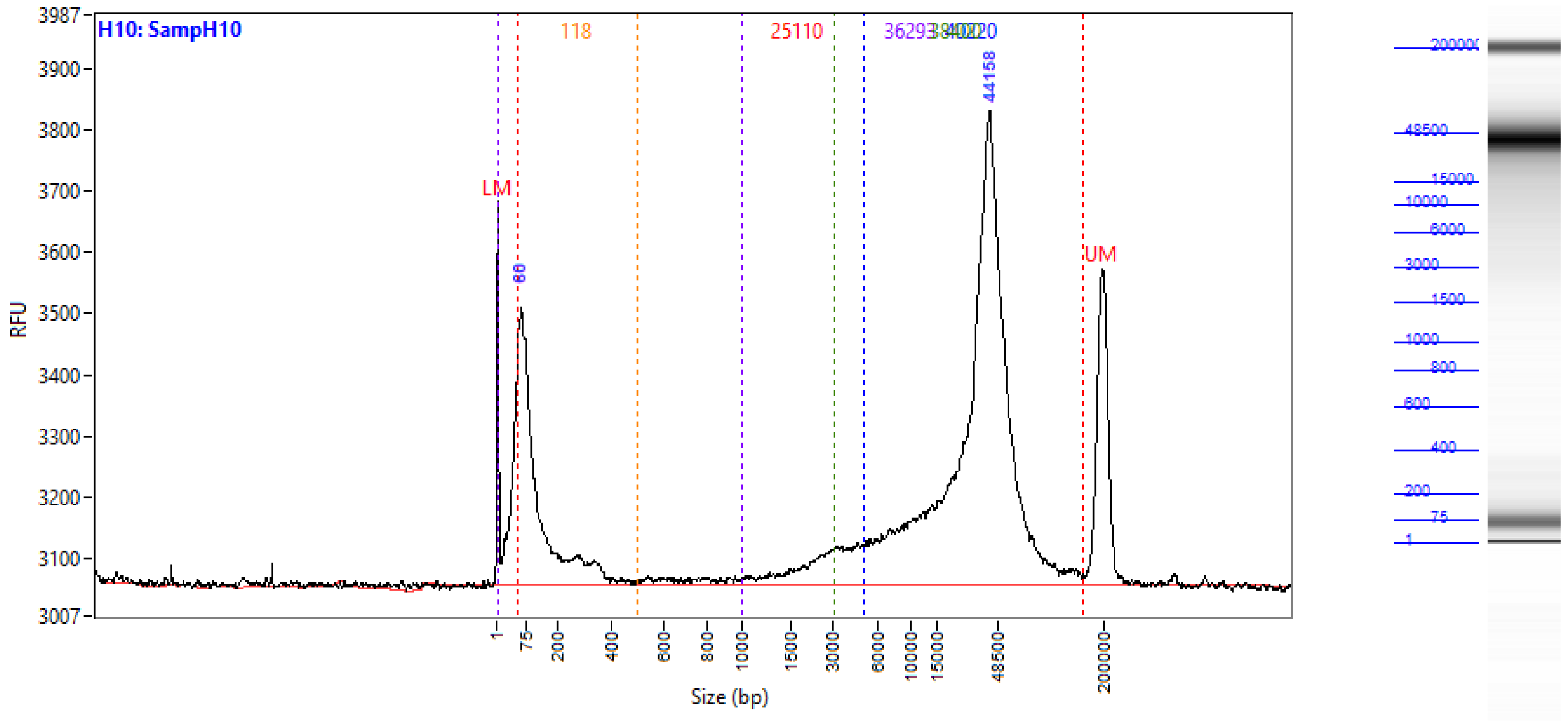


**Supplementary Figure S2.** The fragment analysis of the DNA sample extracted from the LLC cell sample LC02 by Agilent 5400 DNA/RNA Fragment Analyzer. There was slight RNA contamination in the LC02 sample, which did not affect subsequent sequencing analysis.

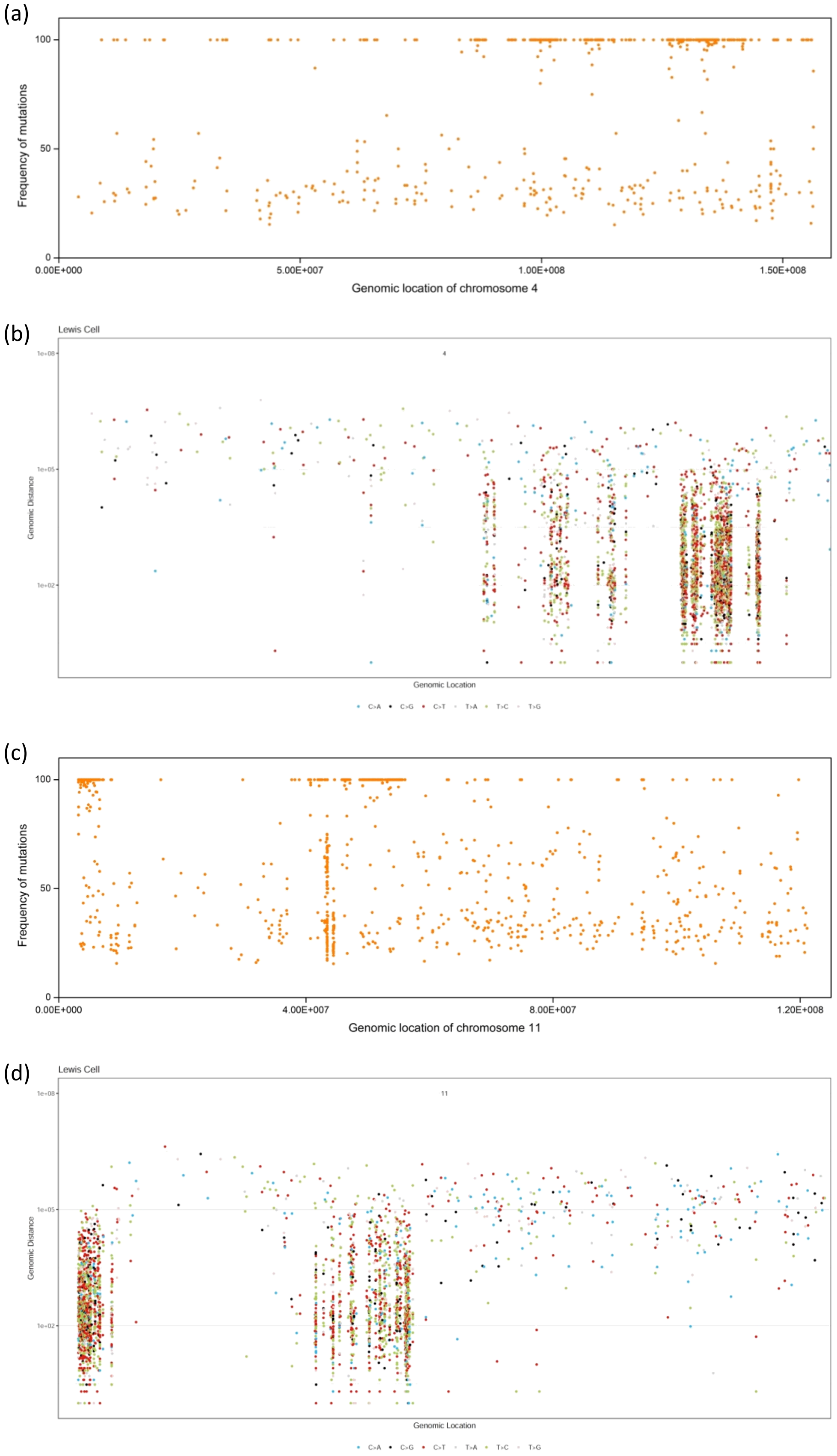


**Supplementary Figure S3.** The coincidence of the regions of homozygous mutation clusters with the regions of regional mutation clusters on chromosomes 4 and 11. (a) Distribution of homozygous and heterozygous mutations on chromosome 4, plotted based on allele frequency; (b) Rainfall plot for SNV

mutations on chromosome 4; (c) Distribution of homozygous and heterozygous mutations on chromosome 11, plotted based on allele frequency; (d) Rainfall plot for SNV mutations on chromosome 11. It could be observed that the regions of homozygous mutation clusters (100% allele frequency) are approximately coincident with the regions of regional mutation clusters on chromosomes 4 and 11.

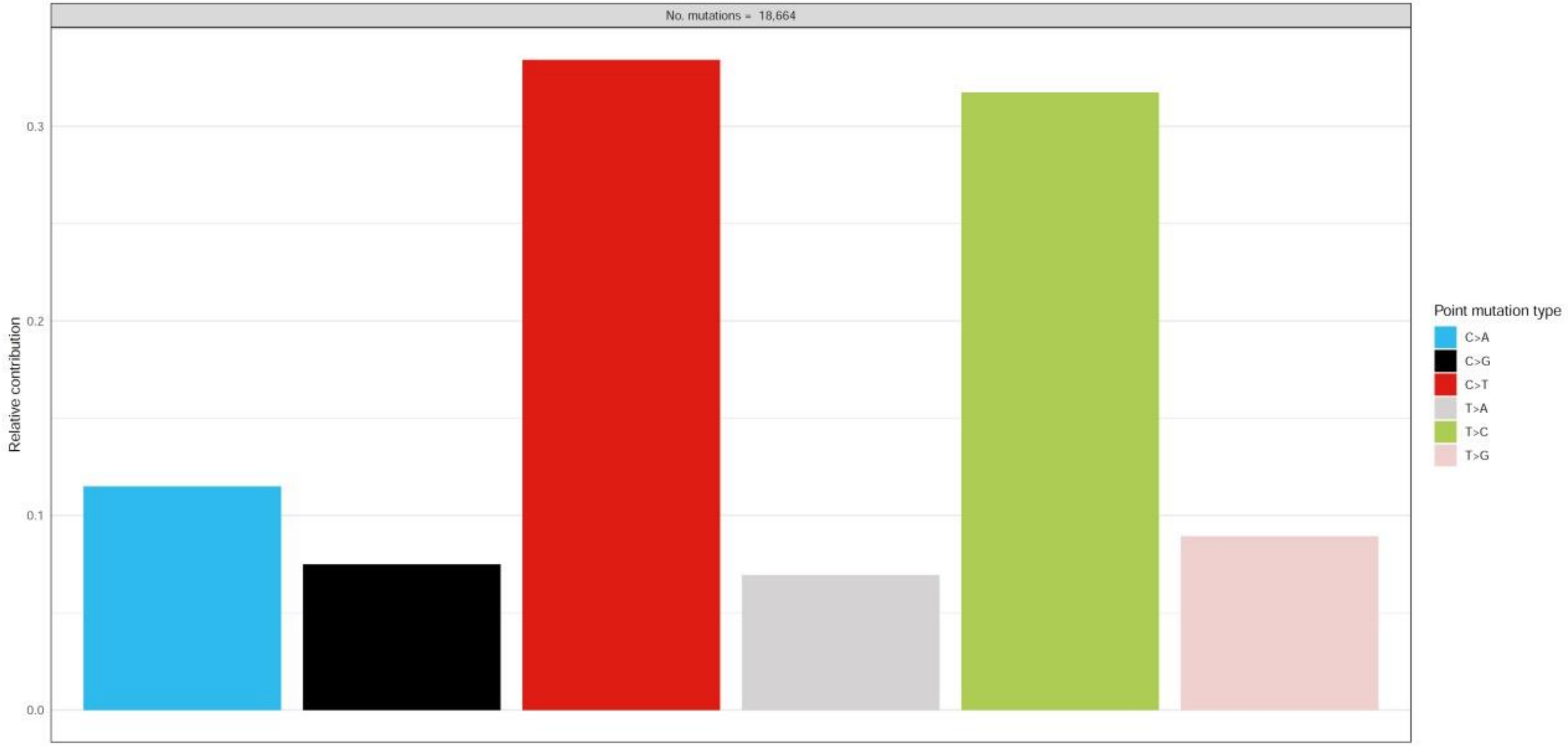


**Supplementary Figure S4.** The mutation spectrum of LLC in the context of 6 base substitutions.

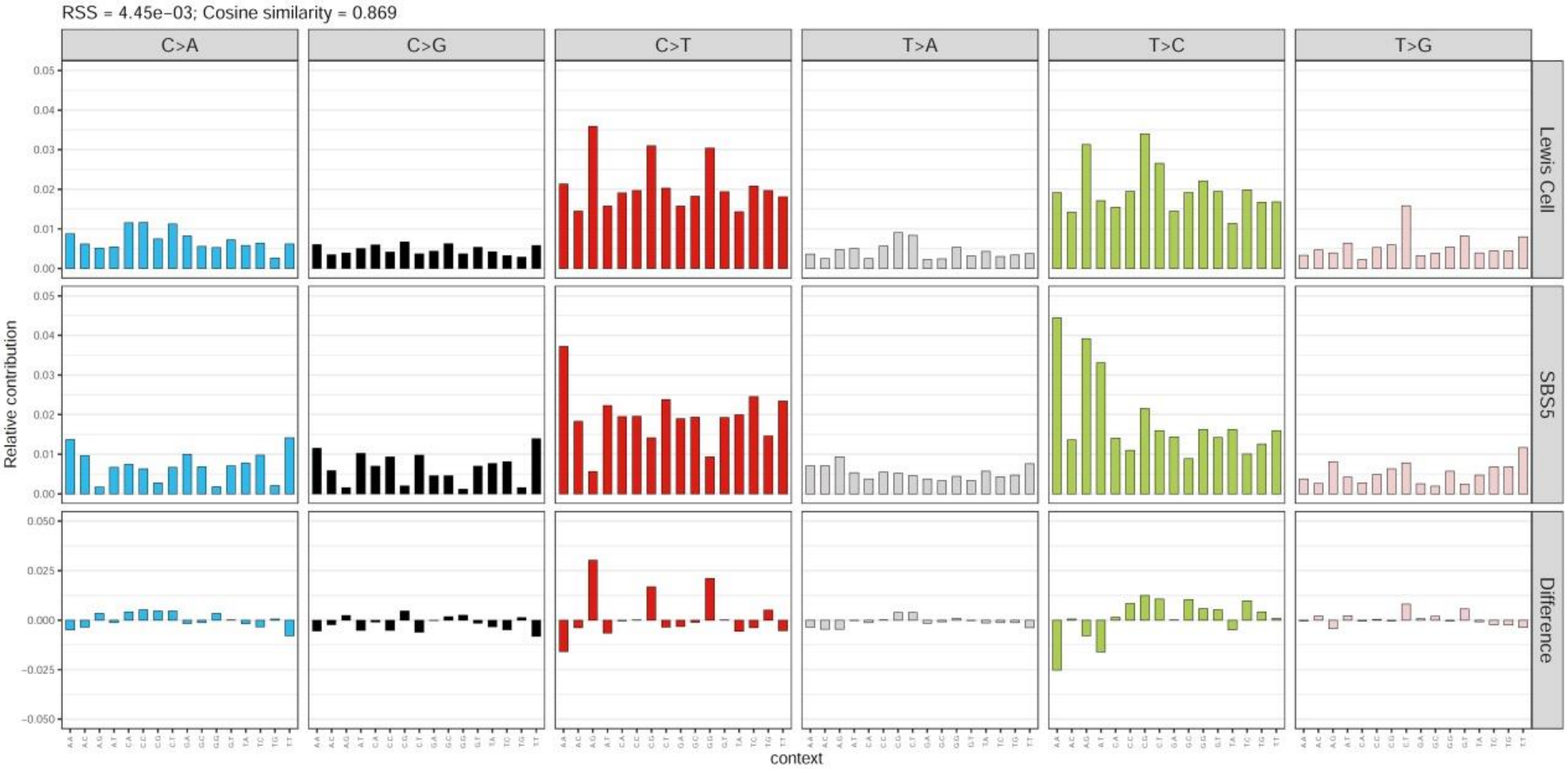


**Supplementary Figure S5.** The cosine similarity between the 96-trinucleotide mutational profiles of LLC and SBS5.

(a)

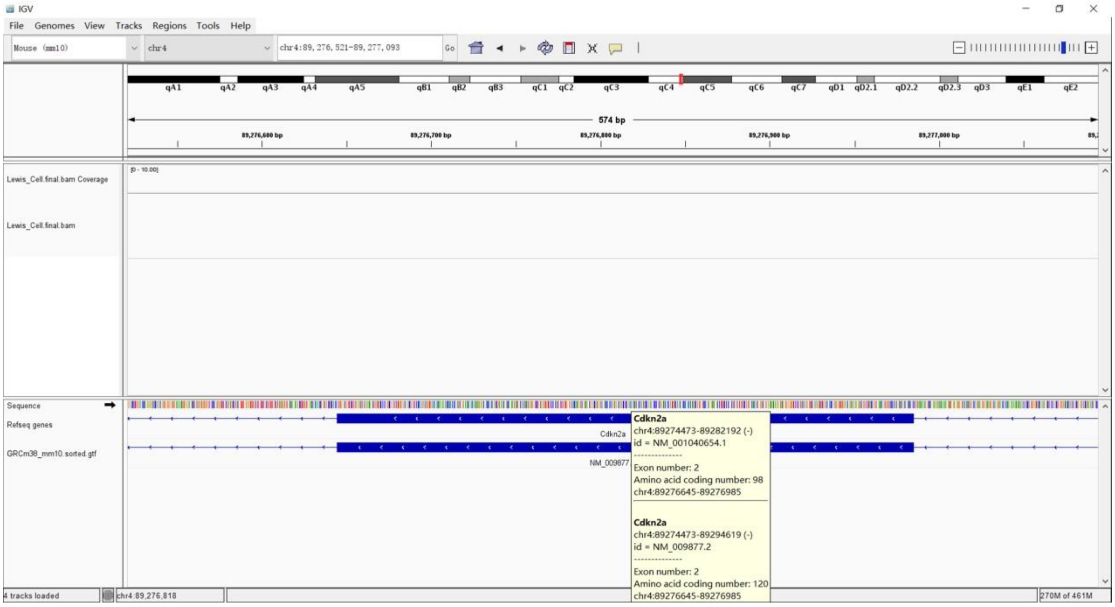


(b)

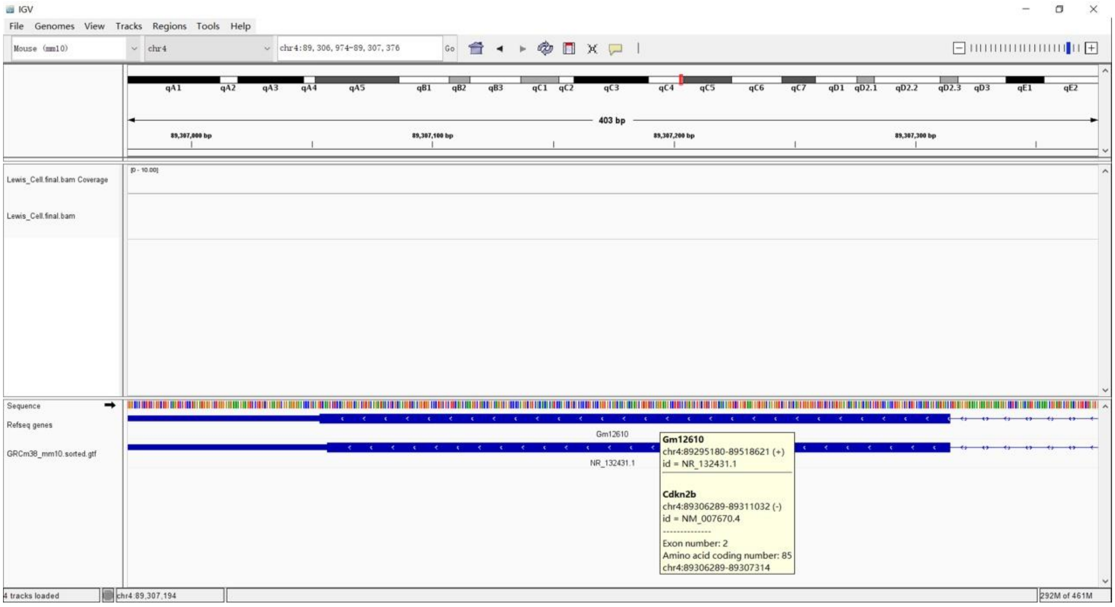


**Supplementary Figure S6.** The IGV view of the genes *Cdkn2a* and *Cdkn2b* for the LLC cell culture sample. No reads were found for *Cdkn2a* (a) and *Cdkn2b* (b). The results indicate that *Cdkn2a* and *Cdkn2b* have been homozygously deleted from the LLC genome.

Supplementary Table S1. The QC data of extracted DNAs, sequencing, alignment, and variant calling

| Sample name | | LC01 | | LC02 |
|---|---|---|---|---|
| Extracted DNAs | Concentration (ng/μL) | 78.1 | | 35 |
| | Total amount (μg) | 6.17 | | 2.76 |
| | DNA quality* | A | | A |
| Sequencing | Lane number | L2 | L1 | L1 |
| | Raw read | 37,993,938 | 7,603,454 | 49,930,666 |
| | Raw data (G) | 13.68 | | 14.98 |
| | Clean read percentage (%) | 98.26 | 98.33 | 97.18 |
| | Error rate (%) | 0.03 | 0.03 | 0.03 |
| | Q20 (%) | 97.99 | 97.99 | 97.95 |
| | Q30 (%) | 94.31 | 94.28 | 94.00 |
| | GC (%) | 46.91 | 46.91 | 46.52 |
| Alignment | Theoretical bases on exome (Mb) | 49.37 | | |
| | Theoretical bases on or near exome (Mb) | 118.25 | | |
| | Total reads (M) | 89.6 | | 97 |
| | Properly mapped reads (M), Percentage | 89.0, 99.3% | | 95.9, 98.8% |
| | Total yield of detected effective bases (Mb) | 13,348.88 | | 14,275.41 |
| | Fraction of detected effective bases on exome | 62.60% | | 61.70% |
| | Fraction of detected effective bases on or near exome | 88.00% | | 86.50% |
| | Average sequencing depth on exome | 169.39 | | 178.47 |
| | Average sequencing depth on or near exome | 99.34 | | 104.42 |
| | Coverage of exome region | 99.80% | | 99.90% |
| | Fraction of exome region covered with at least 100x | 67.70% | | 69.30% |
| | Fraction of exome region covered with at least 50x | 87.10% | | 87.60% |
| | Fraction of exome region covered with at least 20x | 96.20% | | 96.50% |
| | Fraction of exome region covered with at least 10x | 98.40% | | 98.60% |
| | Fraction of exome region covered with at least 4x | 99.40% | | 99.60% |
| Calling | Ts | 15893 | | 16303 |
| | Tv | 9290 | | 9506 |
| | Ts/Tv | 1.71 | | 1.72 |
| | Phred score | 20-228 | | 20-228 |

* The classification for DNA quality is as follows:

Class A: DNA quality meets the quality requirements of library construction and sequencing, and DNA amount meets the needs of one or more times of library construction.

Class C: DNA quality does not fully meet the requirements of library construction and sequencing, but attempts could be made to construct the library using the DNA.

Class D: DNA quality does not meet the requirements of library construction and sequencing at all, and it is not recommended to construct the library using the DNA.

Supplementary Table S2. Comparison of LLC with C57BL/6J and C57BL/10J

| Chromosome | Position | C57BL/6J | C57BL/10J | LLC cell | |
|---|---|---|---|---|---|
| | | Base | Base | Base | Read Depth |
| 1 | 74016171 | C | T | C | 178 |
| 2 | 76814805 | A | G | A | 201 |
| 3 | 88367277 | A | C | A | 47 |
| 4 | 73942623 | T | G | T | 45 |
| 5 | 64286006 | C | T | C | 130 |
| 6 | 34891746 | T | C | T | 11 |
| 7 | 35512008 | A | G | A | 110 |
| 8 | 57583817 | T | C | T | 152 |
| 9 | 106473499 | T | C | T | 90 |
| 10 | 39813838 | G | C | G | 16 |
| 11 | 28915679 | C | T | C | 160 |
| 12 | 104957981 | G | C | G | 20 |
| 13 | 24067645 | C | G | C | 129 |
| 14 | 31379431 | C | T | C | 62 |
| 15 | 75995835 | C | T | C | 63 |
| 16 | 95297591 | C | T | C | 153 |
| 17 | 33645300 | T | C | T | 498 |
| 18 | 82639192 | C | A | C | 53 |
| 19 | 8742352 | C | T | C | 32 |
| X | 157568838 | A | G | A | 50 |

Supplementary Table S3. Comparison of LLC with C57BL/6J and C57BL/6N

| Chromosome | Position | C57BL/6J | C57BL/6N | LLC cell | |
|---|---|---|---|---|---|
| | | Base | Base | Base | Read Depth |
| 1 | 59847167 | G | A | G | 363 |
| 3 | 95734876 | T | C | C | 85 |
| 3 | 96854557 | A | G | G | 269 |
| 4 | 21873684 | C | G | G | 37 |
| 4 | 138221673 | C | T | C | 232 |
| 4 | 140798123 | A | G | A | 132 |
| 4 | 148944359 | T | C | C | 29 |
| 5 | 89775351 | C | T | T | 111 |
| 5 | 96758142 | T | C | C | 120 |
| 5 | 112762721 | C | T | C | 30 |
| 6 | 39400456 | T | A | A | 121 |
| 7 | 3222537 | T | C | C | 110 |
| 7 | 56131292 | G | A | A | 62 |
| 7 | 79111354 | A | C | C | 65 |
| 7 | 102973309 | C | T | C | 127 |
| 7 | 120135179 | G | A | A | 125 |
| 7 | 122167650 | C | T | C | 60 |
| 9 | 25130622 | C | G | C | 145 |
| 10 | 67238174 | T | C | C | 103 |
| 10 | 79169477 | A | G | A | 190 |
| 10 | 88091833 | T | C | T | 89 |
| 11 | 46222615 | G | A | G | 121 |
| 11 | 90480671 | C | T | C | 125 |
| 13 | 21468303 | C | G | G | 19 |
| 13 | 73328436 | A | G | G | 28 |
| 13 | 93063579 | C | G | G | 46 |
| 13 | 64921972 | C | T | C | 161 |
| 14 | 70586204 | G | T | G | 65 |
| 15 | 11336383 | G | T | G | 117 |
| 15 | 77638007 | A | C | C | 138 |
| 16 | 35291544 | G | A | A | 44 |
| 17 | 47400410 | T | C | C | 43 |
| X | 134693042 | C | A | ND* | ND |
| 1 | 139237087 | G | –** | – | 443 |
| 9 | 65280131 | G | – | – | 94 |

* "ND": not detected

** "–": base deleted

# Supplementary Table S4. The SNVs repeatedly detected in both LC01 and LC02

| CHROM | POS | REF | ALT | QUAL | GeneName | Func | ExonicFunc | AAChange | cpgIslandExt | cytoBand | snp142 | homozygosity | heterozygosity |
|---|---|---|---|---|---|---|---|---|---|---|---|---|---|
| chr1 | 3216335 | C | T | 221 | Xkr4 | exonic | missense SNV | Xkr4:NM_001011874:exon3:c.G1631A:p.R544Q | . | 1qA1 | . | 0 | 1 |
| chr1 | 4345005 | C | A | 46.0794 | Rp1 | exonic | missense SNV | Rp1:NM_011283:exon4:c.G5883T:p.M1961I | . | 1qA1 | . | 0 | 1 |
| chr1 | 4348710 | A | G | 154 | Rp1 | exonic | synonymous SNV | Rp1:NM_011283:exon4:c.T2178C:p.N726N | . | 1qA1 | . | 0 | 1 |
| chr1 | 6803090 | A | C | 83 | St18 | exonic | missense SNV | St18:NM_173868:exon6:c.A1048C:p.I350L,St18:NM_001244693:exon7:c.A1048C:p.I350L,St18:NM_00124 | . | 1qA1 | . | 0 | 1 |
| chr1 | 6803184 | T | G | 69 | St18 | intronic | . | . | . | 1qA1 | . | 0 | 1 |
| chr1 | 8855806 | C | G | 118 | Sntg1 | intronic | . | . | . | 1qA2 | . | 0 | 1 |
| chr1 | 8855808 | C | G | 118 | Sntg1 | intronic | . | . | . | 1qA2 | . | 0 | 1 |
| chr1 | 8963082 | G | A | 155 | Sntg1 | intronic | . | . | . | 1qA2 | rs37998313 | 0 | 1 |
| chr1 | 9546463 | G | T | 221 | Rrs1 | exonic | missense SNV | Rrs1:NM_021511:exon1:c.G940T:p.G314W | . | 1qA2 | . | 0 | 1 |
| chr1 | 9549819 | T | C | 228 | Adhfe1 | intronic | . | . | . | 1qA2 | rs30849892 | 1 | 0 |
| chr1 | 9676141 | T | A | 222 | Mybl1 | exonic | missense SNV | Mybl1:NM_001290397:exon10:c.A1293T:p.L431F,Mybl1:NM_008651:exon10:c.A1293T:p.L431F | . | 1qA2 | . | 0 | 1 |
| chr1 | 10033452 | C | G | 89 | Cops5 | intronic | . | . | . | 1qA2 | . | 0 | 1 |
| chr1 | 10719837 | G | T | 215 | Mir467e,Cpa6 | ncRNA_intronic | . | . | . | 1qA2 | . | 0 | 1 |
| chr1 | 11149714 | A | G | 157 | Prex2 | intronic | . | . | . | 1qA2 | . | 0 | 1 |
| chr1 | 11184594 | G | A | 222 | Prex2 | intronic | . | . | . | 1qA2 | . | 0 | 1 |
| chr1 | 11596414 | C | A | 129 | A830018L16Rik | UTR3 | . | . | . | 1qA2 | . | 0 | 1 |
| chr1 | 12817180 | G | T | 73 | Sulf1 | intronic | . | . | . | 1qA3 | . | 0 | 1 |
| chr1 | 12921039 | A | G | 73 | Slco5a1 | exonic | synonymous SNV | Slco5a1:NM_172841:exon4:c.T1425C:p.T475T | . | 1qA3 | . | 0 | 1 |
| chr1 | 13166946 | G | T | 132 | Ncoa2 | exonic | missense SNV | Ncoa2:NM_001302702:exon12:c.C2555A:p.P852H,Ncoa2:NM_008678:exon12:c.C2555A:p.P852H | . | 1qA3 | . | 0 | 1 |
| chr1 | 13579523 | T | G | 113 | Tram1 | exonic | missense SNV | Tram1:NM_028173:exon4:c.A353C:p.K118T | . | 1qA3 | . | 0 | 1 |
| chr1 | 14756499 | C | A | 183 | . | upstream | . | . | . | 1qA3 | . | 0 | 1 |
| chr1 | 14903426 | A | T | 63 | Trpa1 | intronic | . | . | . | 1qA3 | . | 0 | 1 |
| chr1 | 16389985 | C | T | 23.2742 | Stau2 | exonic | missense SNV | Stau2:NM_001111272:exon7:c.G631A:p.V211M,Stau2:NM_025303:exon8:c.G727A:p.V243M | . | 1qA3 | . | 0 | 1 |
| chr1 | 16709379 | C | T | 94 | Ly96 | intronic | . | . | . | 1qA3 | . | 0 | 1 |
| chr1 | 17145629 | C | A | 222 | Gdap1 | intronic | . | . | 328 | 1qA3 | . | 0 | 1 |
| chr1 | 17395318 | A | T | 107 | . | intergenic | . | . | . | 1qA3 | . | 0 | 1 |
| chr1 | 19153582 | T | A | 30.6203 | Tfap2d | intronic | . | . | . | 1qA3 | . | 0 | 1 |
| chr1 | 19153702 | T | C | 111 | Tfap2d | intronic | . | . | . | 1qA3 | . | 0 | 1 |
| chr1 | 19226561 | A | C | 165 | Tfap2b | exonic | missense SNV | Tfap2b:NM_001025305:exon4:c.A673C:p.T225P,Tfap2b:NM_001286340:exon4:c.A727C:p.T243P,Tfap2b:NM | . | 1qA3 | . | 0 | 1 |
| chr1 | 19320419 | A | G | 28.6247 | . | intergenic | . | . | . | 1qA3 | . | 0 | 1 |
| chr1 | 19331306 | T | A | 79 | . | intergenic | . | . | . | 1qA3 | . | 0 | 1 |
| chr1 | 20058690 | G | A | 111 | Pkhd1 | exonic | stopgain | Pkhd1:NM_153179:exon67:c.C11788T:p.Q3930X | . | 1qA3 | . | 0 | 1 |
| chr1 | 20200650 | A | T | 222 | Pkhd1 | exonic | synonymous SNV | Pkhd1:NM_153179:exon58:c.T9678A:p.P3226P | . | 1qA4 | . | 0 | 1 |
| chr1 | 20522705 | A | G | 177 | Pkhd1 | exonic | missense SNV | Pkhd1:NM_153179:exon32:c.T5183C:p.L1728P | . | 1qA4 | . | 0 | 1 |
| chr1 | 20804748 | G | A | 28.2271 | Mcm3 | intronic | . | . | . | 1qA4 | . | 0 | 1 |
| chr1 | 20939207 | T | G | 30.2258 | . | downstream | . | . | . | 1qA4 | . | 0 | 1 |
| chr1 | 21037239 | C | T | 65 | Tram2 | intronic | . | . | . | 1qA4 | . | 0 | 1 |
| chr1 | 21265044 | T | G | 108 | Gsta3 | UTR3 | . | . | . | 1qA4 | . | 0 | 1 |
| chr1 | 22346582 | G | A | 222 | Rims1 | intronic | . | . | . | 1qA5 | rs255919893 | 0 | 1 |
| chr1 | 22805746 | C | A | 55 | . | upstream | . | . | 682 | 1qA5 | . | 0 | 1 |
| chr1 | 24536944 | G | A | 74 | Col19a1 | exonic | stopgain | Col19a1:NM_007733:exon5:c.C316T:p.R106X | . | 1qA5 | . | 0 | 1 |
| chr1 | 26684029 | T | G | 105 | 4931408C20Rik | exonic | missense SNV | 4931408C20Rik:NM_001033764:exon2:c.A2069C:p.N690T | . | 1qA5 | . | 0 | 1 |
| chr1 | 26684265 | G | C | 37.7589 | 4931408C20Rik | exonic | synonymous SNV | 4931408C20Rik:NM_001033764:exon2:c.C1833G:p.G611G | . | 1qA5 | . | 0 | 1 |
| chr1 | 26687400 | A | T | 136 | 4931408C20Rik | exonic | missense SNV | 4931408C20Rik:NM_001033764:exon1:c.T27A:p.N9K | . | 1qA5 | . | 0 | 1 |
| chr1 | 26687547 | A | T | 216 | . | upstream | . | . | . | 1qA5 | . | 0 | 1 |
| chr1 | 32644294 | T | G | 75 | Khdrbs2 | intronic | . | . | . | 1qB | . | 0 | 1 |
| chr1 | 34182595 | G | T | 222 | Dst | exonic | missense SNV | Dst:NM_010081:exon24:c.G7479T:p.L2493F | . | 1qB | . | 0 | 1 |
| chr1 | 34211064 | G | A | 196 | Dst | intronic | . | . | . | 1qB | . | 0 | 1 |
| chr1 | 34499339 | C | A | 222 | Prss39 | exonic | missense SNV | Prss39:NM_009355:exon2:c.C284A:p.A95E | . | 1qB | . | 0 | 1 |
| chr1 | 34588287 | C | A | 115 | Amer3 | exonic | missense SNV | Amer3:NM_213727:exon2:c.C1606A:p.H536N | . | 1qB | . | 0 | 1 |
| chr1 | 34820109 | G | T | 106 | Fam168b | intronic | . | . | . | 1qB | . | 0 | 1 |
| chr1 | 36162072 | C | T | 40.8021 | Uggt1 | intronic | . | . | . | 1qB | . | 0 | 1 |
| chr1 | 36307741 | G | T | 117 | Arid5a | UTR5 | . | . | 597 | 1qB | . | 0 | 1 |
| chr1 | 36782663 | C | A | 68 | Zap70 | UTR3 | . | . | . | 1qB | . | 0 | 1 |
| chr1 | 37257818 | C | A | 60 | Cnga3 | intronic | . | . | . | 1qB | . | 0 | 1 |
| chr1 | 37909104 | A | T | 222 | Lyg2 | intronic | . | . | . | 1qB | . | 0 | 1 |
| chr1 | 37949954 | T | A | 221 | Lyg1 | exonic | missense SNV | Lyg1:NM_027111:exon4:c.A184T:p.R62W | . | 1qB | . | 0 | 1 |
| chr1 | 37990570 | A | G | 228 | Txndc9 | intronic | . | . | . | 1qB | rs46503958 | 1 | 0 |
| chr1 | 38045375 | T | A | 59 | Eif5b | exonic | missense SNV | Eif5b:NM_198303:exon16:c.T2443A:p.S815T | . | 1qB | . | 0 | 1 |
| chr1 | 38406551 | C | G | 145 | Aff3 | intronic | . | . | . | 1qB | . | 0 | 1 |
| chr1 | 39314843 | A | C | 91 | Npas2 | intronic | . | . | . | 1qB | . | 0 | 1 |
| chr1 | 39328270 | G | T | 67 | Npas2 | intronic | . | . | . | 1qB | . | 0 | 1 |
| chr1 | 39385439 | C | A | 45.1512 | Tbc1d8 | intronic | . | . | . | 1qB | . | 0 | 1 |
| chr1 | 40489859 | G | C | 105 | Il18r1 | intronic | . | . | . | 1qB | . | 0 | 1 |
| chr1 | 40781646 | C | A | 135 | Mfsd9 | intronic | . | . | . | 1qB | . | 0 | 1 |
| chr1 | 43152846 | A | T | 70 | Fhl2 | intronic | . | . | . | 1qB | . | 0 | 1 |
| chr1 | 43554179 | T | C | 188 | Nck2 | exonic | missense SNV | Nck2:NM_010879:exon4:c.T545C:p.L182P | 321 | 1qC1.1 | . | 0 | 1 |
| chr1 | 44117362 | T | C | 50 | Kdelc1 | intronic | . | . | . | 1qC1.1 | rs231602733 | 0 | 1 |
| chr1 | 44218337 | T | G | 51 | Mettl21e | intronic | . | . | . | 1qC1.1 | . | 0 | 1 |
| chr1 | 45331700 | C | G | 85 | Col3a1 | exonic | missense SNV | Col3a1:NM_009930:exon18:c.C1214G:p.A405G | . | 1qC1.1 | . | 0 | 1 |
| chr1 | 45383744 | T | G | 101 | Col5a2 | intronic | . | . | . | 1qC1.1 | . | 0 | 1 |
| chr1 | 45422371 | T | G | 58 | Col5a2 | intronic | . | . | . | 1qC1.1 | . | 0 | 1 |
| chr1 | 45520535 | C | A | 92 | . | intergenic | . | . | . | 1qC1.1 | . | 0 | 1 |
| chr1 | 45819811 | A | G | 28.7283 | Wdr75 | intronic | . | . | . | 1qC1.1 | . | 0 | 1 |
| chr1 | 46457415 | A | C | 117 | Dnah7c | intronic | . | . | . | 1qC1.1 | . | 0 | 1 |
| chr1 | 48314753 | G | T | 57 | . | intergenic | . | . | . | 1qC1.1 | . | 0 | 1 |
| chr1 | 50927995 | C | T | 178 | Tmeff2 | exonic | synonymous SNV | Tmeff2:NM_019790:exon1:c.C48T:p.C16C | 1015 | 1qC1.1 | . | 0 | 1 |
| chr1 | 51768456 | G | T | 83 | Myo1b | intronic | . | . | . | 1qC1.1 | . | 0 | 1 |
| chr1 | 52076652 | G | A | 71 | Stat4 | exonic | synonymous SNV | Stat4:NM_011487:exon8:c.G750A:p.P250P,Stat4:NM_001308266:exon9:c.G750A:p.P250P | . | 1qC1.1 | . | 0 | 1 |
| chr1 | 52139155 | C | T | 34.8387 | Stat1 | intronic | . | . | . | 1qC1.1 | . | 0 | 1 |
| chr1 | 52168232 | C | A | 39.0857 | Gls | intronic | . | . | . | 1qC1.1 | . | 0 | 1 |
| chr1 | 52637443 | C | T | 221 | Tmem194b | exonic | missense SNV | Tmem194b:NM_001142647:exon2:c.C173T:p.S58L | . | 1qC1.1 | . | 0 | 1 |
| chr1 | 52790310 | G | A | 103 | Inpp1 | intronic | . | . | . | 1qC1.1 | . | 0 | 1 |
| chr1 | 53062241 | T | G | 153 | Mstn | intronic | . | . | . | 1qC1.1 | . | 0 | 1 |
| chr1 | 54304769 | T | G | 32.5653 | Ccdc150 | intronic | . | . | . | 1qC1.1 | . | 0 | 1 |
| chr1 | 54367814 | C | T | 222 | Ccdc150 | intronic | . | . | . | 1qC1.1 | . | 0 | 1 |
| chr1 | 54492259 | T | C | 60 | Pgap1 | intronic | . | . | . | 1qC1.1 | . | 0 | 1 |
| chr1 | 54524276 | T | G | 175 | Pgap1 | intronic | . | . | . | 1qC1.2 | . | 0 | 1 |
| chr1 | 57938060 | C | T | 121 | Spats2l | intronic | . | . | . | 1qC1.3 | . | 0 | 1 |
| chr1 | 58047250 | G | T | 157 | Aox1 | intronic | . | . | . | 1qC1.3 | . | 0 | 1 |
| chr1 | 58213103 | G | A | 222 | Aox4 | intronic | . | . | . | 1qC1.3 | rs214209342 | 0 | 1 |
| chr1 | 58414420 | T | A | 221 | Clk1 | exonic | missense SNV | Clk1:NM_001042634:exon9:c.A1006T:p.S336C | . | 1qC1.3 | . | 0 | 1 |
| chr1 | 58460985 | C | G | 100 | Nif3l1 | intronic | . | . | . | 1qC1.3 | . | 0 | 1 |
| chr1 | 58475061 | T | C | 29.5699 | Orc2 | intronic | . | . | . | 1qC1.3 | . | 0 | 1 |
| chr1 | 58993044 | A | G | 37.6059 | Stradb | exonic | missense SNV | Stradb:NM_172656:exon10:c.A977G:p.N326S | . | 1qC1.3 | . | 0 | 1 |
| chr1 | 60248408 | T | C | 149 | Nbeal1 | exonic | missense SNV | Nbeal1:NM_173444:exon20:c.T2810C:p.V937A | . | 1qC2 | . | 0 | 1 |
| chr1 | 60434219 | G | A | 49.6522 | Abi2 | intronic | . | . | . | 1qC2 | . | 0 | 1 |
| chr1 | 62276043 | G | T | 46.6549 | Pard3b | intronic | . | . | . | 1qC2 | . | 0 | 1 |
| chr1 | 62479397 | G | C | 147 | Pard3b | intronic | . | . | . | 1qC2 | . | 0 | 1 |
| chr1 | 62771792 | G | T | 221 | Nrp2 | exonic | missense SNV | Nrp2:NM_001077403:exon12:c.G1991T:p.W664L,Nrp2:NM_001077404:exon12:c.G1991T:p.W664L,Nrp2:NM_( | . | 1qC2 | . | 0 | 1 |
| chr1 | 63664982 | A | T | 99 | Dytn | intronic | . | . | . | 1qC2 | . | 0 | 1 |
| chr1 | 63674611 | A | G | 23.9175 | Dytn | intronic | . | . | . | 1qC2 | . | 0 | 1 |
| chr1 | 63768989 | C | A | 55 | 4933402D24Rik | intronic | . | . | . | 1qC2 | . | 0 | 1 |
| chr1 | 64934444 | G | A | 124 | Plekhm3 | intronic | . | . | . | 1qC2 | . | 0 | 1 |
| chr1 | 65082138 | C | T | 29.8474 | Crygb | exonic | missense SNV | Crygb:NM_144761:exon2:c.G29A:p.R10H | . | 1qC2 | . | 0 | 1 |
| chr1 | 65262958 | C | T | 38.1088 | Pikfyve | exonic | synonymous SNV | Pikfyve:NM_001310624:exon32:c.C5007T:p.I1669I,Pikfyve:NM_011086:exon32:c.C4872T:p.I1624I | . | 1qC2 | . | 0 | 1 |
| chr1 | 65311838 | G | A | 99 | Pth2r | intronic | . | . | . | 1qC2 | . | 0 | 1 |
| chr1 | 65928250 | T | C | 60 | Crygf | UTR3 | . | . | . | 1qC3 | . | 0 | 1 |
| chr1 | 66399473 | A | T | 222 | Map2 | exonic | missense SNV | Map2:NM_001310634:exon5:c.A347T:p.H116L,Map2:NM_001039934:exon7:c.A347T:p.H116L,Map2:NM_0086 | . | 1qC3 | . | 0 | 1 |
| chr1 | 66414626 | T | C | 21.3222 | Map2 | exonic | missense SNV | Map2:NM_001310634:exon7:c.T2674C:p.S892P | . | 1qC3 | . | 0 | 1 |
| chr1 | 66525699 | C | G | 121 | Unc80 | exonic | synonymous SNV | Unc80:NM_175510:exon15:c.C2544G:p.T848T | . | 1qC3 | . | 0 | 1 |
| chr1 | 66569954 | C | T | 117 | Unc80 | exonic | synonymous SNV | Unc80:NM_175510:exon23:c.C3801T:p.N1267N | . | 1qC3 | . | 0 | 1 |
| chr1 | 68330395 | C | T | 64 | Erbb4 | intronic | . | . | . | 1qC3 | . | 0 | 1 |
| chr1 | 68370385 | G | T | 222 | Erbb4 | intronic | . | . | . | 1qC3 | . | 0 | 1 |
| chr1 | 71067145 | T | C | 97 | Bard1 | exonic | missense SNV | Bard1:NM_007525:exon5:c.A1322G:p.D441G | . | 1qC3 | . | 0 | 1 |
| chr1 | 71244619 | A | C | 49.0038 | Abca12 | intronic | . | . | . | 1qC3 | . | 0 | 1 |
| chr1 | 71248232 | C | T | 35.8415 | Abca12 | intronic | . | . | . | 1qC3 | . | 0 | 1 |
| chr1 | 71272434 | T | C | 221 | Abca12 | exonic | synonymous SNV | Abca12:NM_175210:exon39:c.A5814G:p.P1938P | . | 1qC3 | . | 0 | 1 |
| chr1 | 71279252 | A | T | 155 | Abca12 | intronic | . | . | . | 1qC3 | . | 0 | 1 |
| chr1 | 71329477 | T | A | 28.9971 | Abca12 | intronic | . | . | . | 1qC3 | . | 0 | 1 |
| chr1 | 71854927 | A | C | 122 | . | intergenic | . | . | . | 1qC3 | . | 0 | 1 |
| chr1 | 72346440 | G | A | 222 | Xrcc5 | intronic | . | . | . | 1qC3 | . | 0 | 1 |
| chr1 | 72651857 | A | G | 51 | Ankar | splicing | . | . | . | 1qC3 | . | 0 | 1 |
| chr1 | 73917726 | G | A | 64 | Tns1 | exonic | missense SNV | Tns1:NM_001289895:exon30:c.C5264T:p.S1755F,Tns1:NM_027884:exon32:c.C5327T:p.S1776F | . | 1qC3 | . | 0 | 1 |
| chr1 | 73993295 | C | T | 84 | Tns1 | intronic | . | . | . | 1qC3 | . | 0 | 1 |
| chr1 | 74470828 | A | C | 108 | Usp37 | intronic | . | . | . | 1qC3 | . | 0 | 1 |
| chr1 | 74562754 | C | T | 87 | Plcd4 | exonic | synonymous SNV | Plcd4:NM_001081456:exon12:c.C1686T:p.R562R,Plcd4:NM_148937:exon13:c.C1782T:p.R594R | . | 1qC3 | . | 0 | 1 |
| chr1 | 74737786 | C | A | 145 | Cyp27a1 | UTR3 | . | . | . | 1qC4 | . | 0 | 1 |
| chr1 | 74900670 | C | T | 84 | Mir375 | ncRNA_exonic | . | . | 429 | 1qC4 | . | 0 | 1 |
| chr1 | 74925010 | C | G | 222 | Ccdc108 | exonic | missense SNV | Ccdc108:NM_001039495:exon11:c.G1778C:p.G593A | . | 1qC4 | . | 0 | 1 |
| chr1 | 74925723 | G | A | 78 | Ccdc108 | intronic | . | . | . | 1qC4 | . | 0 | 1 |
| chr1 | 75262239 | G | C | 22.7475 | Ptprn | intronic | . | . | . | 1qC4 | . | 0 | 1 |
| chr1 | 75278188 | A | G | 179 | Resp18 | intronic | . | . | . | 1qC4 | . | 0 | 1 |
| chr1 | 75486756 | G | A | 228 | Obsl1 | exonic | missense SNV | Obsl1:NM_178884:exon19:c.C5291T:p.T1764M | 238 | 1qC4 | rs31502068 | 1 | 0 |
| chr1 | 75554314 | G | A | 222 | Slc4a3 | intronic | . | . | . | 1qC4 | . | 0 | 1 |
| chr1 | 77066608 | C | A | 222 | . | intergenic | . | . | . | 1qC4 | . | 0 | 1 |
| chr1 | 77382943 | A | C | 62 | Epha4 | exonic | missense SNV | Epha4:NM_007936:exon13:c.T2272G:p.L758V | . | 1qC4 | . | 0 | 1 |
| chr1 | 77511679 | T | G | 153 | Epha4 | splicing | . | . | . | 1qC4 | . | 0 | 1 |
| chr1 | 78967961 | T | C | 93 | . | intergenic | . | . | . | 1qC4 | . | 0 | 1 |
| chr1 | 79801503 | A | G | 139 | Serpine2 | exonic | missense SNV | Serpine2:NM_009255:exon6:c.T938C:p.I313T | . | 1qC4 | . | 0 | 1 |
| chr1 | 79821568 | A | T | 222 | Serpine2 | intronic | . | . | . | 1qC4 | . | 0 | 1 |
| chr1 | 80561082 | A | G | 47.1798 | Dock10 | intronic | . | . | . | 1qC5 | . | 0 | 1 |
| chr1 | 82291875 | A | G | 53 | . | upstream | . | . | . | 1qC5 | . | 0 | 1 |
| chr1 | 82527738 | A | G | 110 | Col4a4 | intronic | . | . | . | 1qC5 | . | 0 | 1 |
| chr1 | 82700408 | T | C | 165 | Col4a3 | exonic | synonymous SNV | Col4a3:NM_007734:exon42:c.T3730C:p.L1244L | . | 1qC5 | . | 0 | 1 |
| chr1 | 83198182 | G | A | 104 | Daw1 | exonic | missense SNV | Daw1:NM_027725:exon7:c.G640A:p.A214T | . | 1qC5 | . | 0 | 1 |
| chr1 | 84384067 | C | T | 47.823 | Dner | intronic | . | . | . | 1qC5 | . | 0 | 1 |
| chr1 | 85061197 | T | C | 146 | . | intergenic | . | . | . | 1qC5 | . | 0 | 1 |
| chr1 | 85061209 | G | T | 47.719 | . | intergenic | . | . | . | 1qC5 | . | 0 | 1 |
| chr1 | 85062374 | G | A | 107 | . | intergenic | . | . | . | 1qC5 | rs47884333 | 0 | 1 |
| chr1 | 85062453 | T | A | 189 | . | intergenic | . | . | . | 1qC5 | rs108162167 | 0 | 1 |
| chr1 | 85062731 | A | C | 222 | . | intergenic | . | . | . | 1qC5 | rs107752361 | 0 | 1 |
| chr1 | 85062795 | G | A | 222 | . | intergenic | . | . | . | 1qC5 | rs48094665 | 0 | 1 |
| chr1 | 85112576 | G | C | 219 | . | intergenic | . | . | . | 1qC5 | rs48445685 | 0 | 1 |
| chr1 | 85112904 | C | T | 222 | . | intergenic | . | . | . | 1qC5 | rs50890065 | 0 | 1 |
| chr1 | 85905368 | G | T | 119 | Itm2c | exonic | missense SNV | Itm2c:NM_022417:exon3:c.G422T:p.G141V | . | 1qC5 | . | 0 | 1 |
| chr1 | 86427378 | T | A | 222 | 1700019O17Rik | exonic | synonymous SNV | 1700019O17Rik:NM_027966:exon1:c.T1008A:p.A336A | . | 1qC5 | . | 0 | 1 |
| chr1 | 86744593 | C | A | 31.7505 | Dis3l2 | intronic | . | . | . | 1qC5 | . | 0 | 1 |
| chr1 | 86973430 | G | T | 69 | Dis3l2 | exonic | stopgain | Dis3l2:NM_153530:exon12:c.G1342T:p.E448X,Dis3l2:NM_001172157:exon13:c.G1384T:p.E462X | . | 1qC5 | . | 0 | 1 |
| chr1 | 87125920 | C | T | 108 | Akp3 | exonic | synonymous SNV | Akp3:NM_007432:exon5:c.C516T:p.H172H | . | 1qC5 | . | 0 | 1 |
| chr1 | 87153179 | C | T | 69 | Ecel1 | exonic | missense SNV | Ecel1:NM_001277925:exon5:c.G1000A:p.D334N,Ecel1:NM_021306:exon5:c.G1000A:p.D334N | . | 1qC5 | . | 0 | 1 |
| chr1 | 87297829 | C | A | 134 | Efhd1 | intronic | . | . | . | 1qC5 | . | 0 | 1 |
| chr1 | 87707886 | G | A | 99 | Inpp5d | intronic | . | . | . | 1qD | . | 0 | 1 |
| chr1 | 87881995 | G | A | 57 | Dgkd | intronic | . | . | . | 1qD | . | 0 | 1 |
| chr1 | 88211922 | A | C | 155 | Ugt1a7c,Ugt1a5,U | intronic | . | . | . | 1qD | rs47567212 | 0 | 1 |
| chr1 | 88211945 | G | A | 189 | Ugt1a7c,Ugt1a5,U | intronic | . | . | . | 1qD | rs50519654 | 0 | 1 |
| chr1 | 88212093 | G | A | 221 | Ugt1a6a,Ugt1a6b,U | exonic | missense SNV | Ugt1a1:NM_201645:exon1:c.G92A:p.R31K | . | 1qD | rs47888099 | 0 | 1 |
| chr1 | 88212141 | G | T | 221 | Ugt1a10,Ugt1a2,U | exonic | missense SNV | Ugt1a1:NM_201645:exon1:c.G140T:p.G47V | . | 1qD | rs49882561 | 0 | 1 |
| chr1 | 88212244 | C | T | 130 | Ugt1a2,Ugt1a10,U | exonic | synonymous SNV | Ugt1a1:NM_201645:exon1:c.C243T:p.F81F | . | 1qD | rs46758900 | 0 | 1 |
| chr1 | 88212317 | T | C | 221 | Ugt1a6b,Ugt1a6a,U | exonic | missense SNV | Ugt1a1:NM_201645:exon1:c.T316C:p.S106P | . | 1qD | rs49282812 | 0 | 1 |
| chr1 | 88212637 | G | A | 146 | Ugt1a10,Ugt1a2,U | exonic | synonymous SNV | Ugt1a1:NM_201645:exon1:c.G636A:p.V212V | . | 1qD | rs48609033 | 0 | 1 |
| chr1 | 88212655 | C | T | 222 | Ugt1a1,Ugt1a7c,U | exonic | synonymous SNV | Ugt1a1:NM_201645:exon1:c.C654T:p.A218A | . | 1qD | rs30305267 | 0 | 1 |
| chr1 | 88212696 | A | T | 115 | Ugt1a6a,Ugt1a6b,U | exonic | missense SNV | Ugt1a1:NM_201645:exon1:c.A695T:p.Y232F | . | 1qD | rs49839149 | 0 | 1 |
| chr1 | 88212730 | A | G | 222 | Ugt1a6b,Ugt1a6a,U | exonic | synonymous SNV | Ugt1a1:NM_201645:exon1:c.A729G:p.E243E | . | 1qD | rs30305266 | 0 | 1 |
| chr1 | 88212740 | C | A | 222 | Ugt1a2,Ugt1a10,U | exonic | missense SNV | Ugt1a1:NM_201645:exon1:c.C739A:p.Q247K | . | 1qD | rs47467070 | 0 | 1 |
| chr1 | 88213019 | C | T | 138 | Ugt1a2,Ugt1a10,U | intronic | . | . | . | 1qD | rs30305264 | 0 | 1 |
| chr1 | 88213057 | A | G | 49.2447 | Ugt1a1,Ugt1a7c,U | intronic | . | . | . | 1qD | rs51452259 | 0 | 1 |
| chr1 | 88214783 | C | G | 108 | Ugt1a2,Ugt1a10,U | intronic | . | . | . | 1qD | rs50534550 | 0 | 1 |
| chr1 | 88214947 | C | A | 20.2136 | Ugt1a6b,Ugt1a6a,U | intronic | . | . | . | 1qD | rs49857111 | 0 | 1 |

| | | | | | | | | | | | | | |
|---|---|---|---|---|---|---|---|---|---|---|---|---|---|
| chr1 | 88214987 | G | A | 221 | Ugt1a2,Ugt1a10,U | intronic | . | . | . | 1qD | rs8258194 | 0 | 1 |
| chr1 | 88215037 | A | G | 222 | Ugt1a6b,Ugt1a6a,U | intronic | . | . | . | 1qD | rs30303367 | 0 | 1 |
| chr1 | 88215088 | T | G | 221 | Ugt1a10,Ugt1a2,U | exonic | synonymous SNV | Ugt1a2:NM_013701:exon2:c.T909G:p.V303V,Ugt1a10:NM_201641:exon2:c.T900G:p.V300V,Ugt1a7c:NM_201 | . | 1qD | rs30303366 | 0 | 1 |
| chr1 | 88215174 | C | T | 56 | Ugt1a1,Ugt1a7c,U | exonic | missense SNV | Ugt1a2:NM_013701:exon2:c.C995T:p.T332M,Ugt1a10:NM_201641:exon2:c.C986T:p.T329M,Ugt1a7c:NM_201 | . | 1qD | rs47208592 | 0 | 1 |
| chr1 | 88215178 | A | T | 132 | Ugt1a1,Ugt1a7c,U | splicing | . | . | . | 1qD | . | 0 | 1 |
| chr1 | 88215188 | T | G | 44.7741 | Ugt1a6a,Ugt1a6b,U | intronic | . | . | . | 1qD | rs48922510 | 0 | 1 |
| chr1 | 88215383 | C | A | 72 | Ugt1a2,Ugt1a10,U | intronic | . | . | . | 1qD | . | 0 | 1 |
| chr1 | 88215606 | C | G | 143 | Ugt1a10,Ugt1a2,U | intronic | . | . | . | 1qD | rs30303364 | 0 | 1 |
| chr1 | 88215740 | A | T | 222 | Ugt1a1,Ugt1a9,Ug | intronic | . | . | . | 1qD | rs50325651 | 0 | 1 |
| chr1 | 88215884 | T | A | 220 | Ugt1a2,Ugt1a10,U | splicing | . | . | . | 1qD | rs8258197 | 0 | 1 |
| chr1 | 88215943 | A | G | 219 | Ugt1a2,Ugt1a10,U | intronic | . | . | . | 1qD | rs51622145 | 0 | 1 |
| chr1 | 88215984 | C | T | 107 | Ugt1a6a,Ugt1a6b,U | intronic | . | . | . | 1qD | rs46479503 | 0 | 1 |
| chr1 | 88216116 | C | T | 221 | Ugt1a6b,Ugt1a6a,U | intronic | . | . | . | 1qD | rs51316825 | 0 | 1 |
| chr1 | 88216213 | C | T | 222 | Ugt1a6b,Ugt1a6a,U | exonic | missense SNV | Ugt1a2:NM_013701:exon4:c.C1160T:p.P387L,Ugt1a10:NM_201641:exon4:c.C1151T:p.P384L,Ugt1a7c:NM_2 | . | 1qD | rs49604602 | 0 | 1 |
| chr1 | 88216261 | G | A | 59 | Ugt1a2,Ugt1a10,U | exonic | missense SNV | Ugt1a2:NM_013701:exon4:c.G1208A:p.R403H,Ugt1a10:NM_201641:exon4:c.G1199A:p.R400H,Ugt1a7c:NM_2 | . | 1qD | rs49274850 | 0 | 1 |
| chr1 | 88216393 | G | T | 222 | Ugt1a10,Ugt1a2,U | intronic | . | . | . | 1qD | rs48400836 | 0 | 1 |
| chr1 | 88216494 | G | A | 131 | Ugt1a2,Ugt1a10,U | intronic | . | . | . | 1qD | rs50397010 | 0 | 1 |
| chr1 | 88216495 | A | C | 131 | Ugt1a6b,Ugt1a6a,U | intronic | . | . | . | 1qD | rs49386843 | 0 | 1 |
| chr1 | 88216566 | T | C | 76 | Ugt1a6a,Ugt1a6b,U | intronic | . | . | . | 1qD | rs46659644 | 0 | 1 |
| chr1 | 88216623 | A | G | 40.8655 | Ugt1a1,Ugt1a9,Ug | intronic | . | . | . | 1qD | rs45681432 | 0 | 1 |
| chr1 | 88217910 | C | T | 88 | Ugt1a10,Ugt1a2,U | intronic | . | . | . | 1qD | rs48412197 | 0 | 1 |
| chr1 | 88217941 | A | G | 101 | Ugt1a2,Ugt1a10,U | intronic | . | . | . | 1qD | rs51823669 | 0 | 1 |
| chr1 | 88218078 | C | A | 189 | Ugt1a2,Ugt1a10,U | intronic | . | . | . | 1qD | rs45719392 | 0 | 1 |
| chr1 | 88218089 | A | G | 221 | Ugt1a6b,Ugt1a6a,U | intronic | . | . | . | 1qD | rs30310343 | 0 | 1 |
| chr1 | 88218098 | C | T | 28.5495 | Ugt1a2,Ugt1a10,U | intronic | . | . | . | 1qD | rs50005353 | 0 | 1 |
| chr1 | 88218146 | C | A | 221 | Ugt1a6a,Ugt1a6b,U | exonic | missense SNV | Ugt1a2:NM_013701:exon5:c.C1324A:p.R442S,Ugt1a10:NM_201641:exon5:c.C1315A:p.R439S,Ugt1a7c:NM_2 | . | 1qD | rs47490282 | 0 | 1 |
| chr1 | 88218240 | A | T | 44.5177 | Ugt1a2,Ugt1a10,U | exonic | missense SNV | Ugt1a2:NM_013701:exon5:c.A1418T:p.H473L,Ugt1a10:NM_201641:exon5:c.A1409T:p.H470L,Ugt1a7c:NM_2 | . | 1qD | rs49647345 | 0 | 1 |
| chr1 | 88218245 | C | T | 222 | Ugt1a5,Ugt1a7c,U | exonic | missense SNV | Ugt1a2:NM_013701:exon5:c.C1423T:p.R475C,Ugt1a10:NM_201641:exon5:c.C1414T:p.R472C,Ugt1a7c:NM_2 | . | 1qD | rs50150817 | 0 | 1 |
| chr1 | 88218279 | A | T | 122 | Ugt1a10,Ugt1a2,U | exonic | missense SNV | Ugt1a2:NM_013701:exon5:c.A1457T:p.Y486F,Ugt1a10:NM_201641:exon5:c.A1448T:p.Y483F,Ugt1a7c:NM_2 | . | 1qD | rs46635214 | 0 | 1 |
| chr1 | 88218283 | C | G | 162 | Ugt1a6a,Ugt1a6b,U | exonic | missense SNV | Ugt1a2:NM_013701:exon5:c.C1461G:p.H487Q,Ugt1a10:NM_201641:exon5:c.C1452G:p.H484Q,Ugt1a7c:NM_2 | . | 1qD | rs47264151 | 0 | 1 |
| chr1 | 88218360 | G | A | 144 | Ugt1a10,Ugt1a2,U | exonic | missense SNV | Ugt1a2:NM_013701:exon5:c.G1538A:p.G513D,Ugt1a10:NM_201641:exon5:c.G1529A:p.G510D,Ugt1a7c:NM_2 | . | 1qD | rs46776285 | 0 | 1 |
| chr1 | 88218388 | G | A | 126 | Ugt1a2,Ugt1a10,U | exonic | synonymous SNV | Ugt1a2:NM_013701:exon5:c.G1566A:p.G522G,Ugt1a10:NM_201641:exon5:c.G1557A:p.G519G,Ugt1a7c:NM_2 | . | 1qD | rs51631235 | 0 | 1 |
| chr1 | 88218738 | C | T | 43.9678 | Ugt1a10,Ugt1a2,U | UTR3 | . | . | . | 1qD | rs30310340 | 0 | 1 |
| chr1 | 88227935 | C | T | 68 | Mroh2a | intronic | . | . | . | 1qD | rs30307490 | 1 | 0 |
| chr1 | 88228001 | T | A | 219 | Mroh2a | intronic | . | . | . | 1qD | rs46364263 | 0 | 1 |
| chr1 | 88228427 | G | A | 221 | Mroh2a | intronic | . | . | . | 1qD | rs47381372 | 0 | 1 |
| chr1 | 88228474 | C | T | 156 | Mroh2a | intronic | . | . | . | 1qD | rs46829270 | 0 | 1 |
| chr1 | 88228498 | G | T | 222 | Mroh2a | intronic | . | . | . | 1qD | rs30307489 | 0 | 1 |
| chr1 | 88228513 | G | C | 184 | Mroh2a | intronic | . | . | . | 1qD | rs47509609 | 0 | 1 |
| chr1 | 88228514 | C | T | 179 | Mroh2a | intronic | . | . | . | 1qD | rs51661379 | 0 | 1 |
| chr1 | 88228602 | G | A | 132 | Mroh2a | intronic | . | . | . | 1qD | rs49734761 | 0 | 1 |
| chr1 | 88230158 | T | C | 106 | Mroh2a | intronic | . | . | . | 1qD | rs51196419 | 0 | 1 |
| chr1 | 88230254 | A | C | 152 | Mroh2a | intronic | . | . | . | 1qD | rs47278076 | 0 | 1 |
| chr1 | 88230368 | C | A | 222 | Mroh2a | exonic | synonymous SNV | Mroh2a:NM_001281466:exon4:c.C288A:p.I96I | . | 1qD | rs46011708 | 0 | 1 |
| chr1 | 88230490 | T | C | 221 | Mroh2a | intronic | . | . | . | 1qD | rs45780079 | 0 | 1 |
| chr1 | 88230576 | A | G | 222 | Mroh2a | intronic | . | . | . | 1qD | rs48988179 | 0 | 1 |
| chr1 | 88230637 | T | C | 222 | Mroh2a | exonic | synonymous SNV | Mroh2a:NM_001281466:exon5:c.T405C:p.F135F | . | 1qD | rs49389668 | 0 | 1 |
| chr1 | 88230650 | G | A | 213 | Mroh2a | exonic | missense SNV | Mroh2a:NM_001281466:exon5:c.G418A:p.V140M | . | 1qD | rs46220231 | 0 | 1 |
| chr1 | 88230667 | A | C | 218 | Mroh2a | exonic | synonymous SNV | Mroh2a:NM_001281466:exon5:c.A435C:p.L145L | . | 1qD | rs48110663 | 0 | 1 |
| chr1 | 88230746 | G | A | 222 | Mroh2a | exonic | missense SNV | Mroh2a:NM_001281466:exon5:c.G514A:p.E172K | . | 1qD | rs108141835 | 0 | 1 |
| chr1 | 88230879 | T | C | 220 | Mroh2a | intronic | . | . | . | 1qD | rs47313177 | 0 | 1 |
| chr1 | 88230896 | T | A | 197 | Mroh2a | intronic | . | . | . | 1qD | rs50348246 | 0 | 1 |
| chr1 | 88230932 | A | C | 126 | Mroh2a | intronic | . | . | . | 1qD | rs49774785 | 0 | 1 |
| chr1 | 88231085 | C | T | 80 | Mroh2a | intronic | . | . | . | 1qD | rs47121784 | 0 | 1 |
| chr1 | 88231995 | C | G | 102 | Mroh2a | intronic | . | . | . | 1qD | rs52651854 | 0 | 1 |
| chr1 | 88232020 | T | G | 149 | Mroh2a | intronic | . | . | . | 1qD | rs52617336 | 0 | 1 |
| chr1 | 88232047 | C | T | 100 | Mroh2a | intronic | . | . | . | 1qD | rs52594762 | 0 | 1 |
| chr1 | 88232086 | A | T | 162 | Mroh2a | intronic | . | . | . | 1qD | rs52607119 | 0 | 1 |
| chr1 | 88232170 | G | A | 105 | Mroh2a | intronic | . | . | . | 1qD | rs50073641 | 0 | 1 |
| chr1 | 88232324 | C | T | 221 | Mroh2a | exonic | missense SNV | Mroh2a:NM_001281466:exon6:c.C637T:p.R213C | . | 1qD | rs49941474 | 0 | 1 |
| chr1 | 88232389 | T | G | 221 | Mroh2a | intronic | . | . | . | 1qD | rs49751518 | 0 | 1 |
| chr1 | 88232419 | A | G | 221 | Mroh2a | intronic | . | . | . | 1qD | rs47522426 | 0 | 1 |
| chr1 | 88233139 | G | T | 62 | Mroh2a | intronic | . | . | . | 1qD | rs48643135 | 0 | 1 |
| chr1 | 88233189 | A | G | 158 | Mroh2a | intronic | . | . | . | 1qD | rs51112296 | 0 | 1 |
| chr1 | 88233303 | C | A | 222 | Mroh2a | intronic | . | . | . | 1qD | rs49424981 | 0 | 1 |
| chr1 | 88233390 | G | A | 43.9557 | Mroh2a | exonic | synonymous SNV | Mroh2a:NM_001281466:exon7:c.G729A:p.T243T | . | 1qD | rs51963635 | 0 | 1 |
| chr1 | 88233417 | C | T | 222 | Mroh2a | exonic | synonymous SNV | Mroh2a:NM_001281466:exon7:c.C756T:p.F252F | . | 1qD | rs50034482 | 0 | 1 |
| chr1 | 88233442 | G | T | 221 | Mroh2a | exonic | missense SNV | Mroh2a:NM_001281466:exon7:c.G781T:p.V261L | . | 1qD | rs47038443 | 0 | 1 |
| chr1 | 88233479 | C | A | 66 | Mroh2a | intronic | . | . | . | 1qD | rs47693451 | 0 | 1 |
| chr1 | 88233480 | A | G | 66 | Mroh2a | intronic | . | . | . | 1qD | rs46767762 | 0 | 1 |
| chr1 | 88233547 | C | A | 171 | Mroh2a | intronic | . | . | . | 1qD | rs45932274 | 0 | 1 |
| chr1 | 88233548 | A | G | 169 | Mroh2a | intronic | . | . | . | 1qD | rs30306583 | 0 | 1 |
| chr1 | 88233860 | C | T | 136 | Mroh2a | intronic | . | . | . | 1qD | rs51585063 | 0 | 1 |
| chr1 | 88233867 | T | G | 162 | Mroh2a | intronic | . | . | . | 1qD | rs49300752 | 0 | 1 |
| chr1 | 88234040 | G | C | 221 | Mroh2a | exonic | synonymous SNV | Mroh2a:NM_001281466:exon8:c.G846C:p.L282L | . | 1qD | rs47387152 | 0 | 1 |
| chr1 | 88234120 | G | A | 221 | Mroh2a | exonic | missense SNV | Mroh2a:NM_001281466:exon8:c.G926A:p.G309D | . | 1qD | . | 0 | 1 |
| chr1 | 88234137 | G | A | 196 | Mroh2a | exonic | missense SNV | Mroh2a:NM_001281466:exon8:c.G943A:p.V315I | . | 1qD | . | 0 | 1 |
| chr1 | 88234200 | G | A | 222 | Mroh2a | intronic | . | . | . | 1qD | rs582632104 | 0 | 1 |
| chr1 | 88234306 | C | T | 86 | Mroh2a | intronic | . | . | . | 1qD | rs47147533 | 0 | 1 |
| chr1 | 88234673 | G | A | 131 | Mroh2a | intronic | . | . | . | 1qD | rs51372928 | 0 | 1 |
| chr1 | 88234724 | C | T | 170 | Mroh2a | intronic | . | . | . | 1qD | rs47927410 | 0 | 1 |
| chr1 | 88234840 | T | C | 221 | Mroh2a | intronic | . | . | . | 1qD | rs47110174 | 0 | 1 |
| chr1 | 88234936 | T | C | 221 | Mroh2a | intronic | . | . | . | 1qD | rs49249967 | 0 | 1 |
| chr1 | 88234981 | G | A | 70 | Mroh2a | exonic | synonymous SNV | Mroh2a:NM_001281466:exon9:c.G963A:p.Q321Q | . | 1qD | rs46732827 | 0 | 1 |
| chr1 | 88235023 | G | A | 222 | Mroh2a | exonic | synonymous SNV | Mroh2a:NM_001281466:exon9:c.G1005A:p.P335P | . | 1qD | rs51100050 | 0 | 1 |
| chr1 | 88235079 | G | A | 180 | Mroh2a | intronic | . | . | . | 1qD | rs49288088 | 0 | 1 |
| chr1 | 88235098 | G | C | 221 | Mroh2a | intronic | . | . | . | 1qD | rs50657360 | 0 | 1 |
| chr1 | 88235114 | G | A | 169 | Mroh2a | intronic | . | . | . | 1qD | rs49604714 | 0 | 1 |
| chr1 | 88235143 | T | C | 109 | Mroh2a | intronic | . | . | . | 1qD | rs46220804 | 0 | 1 |
| chr1 | 88235254 | G | A | 130 | Mroh2a | exonic | missense SNV | Mroh2a:NM_001281466:exon10:c.G1109A:p.C370Y | . | 1qD | rs47868953 | 0 | 1 |
| chr1 | 88235442 | G | A | 221 | Mroh2a | exonic | missense SNV | Mroh2a:NM_001281466:exon11:c.G1193A:p.R398H | . | 1qD | rs51702482 | 0 | 1 |
| chr1 | 88235629 | C | T | 220 | Mroh2a | intronic | . | . | . | 1qD | rs49858676 | 0 | 1 |
| chr1 | 88236665 | T | G | 214 | Mroh2a | exonic | missense SNV | Mroh2a:NM_001281466:exon12:c.T1248G:p.I416M | . | 1qD | rs47887801 | 0 | 1 |
| chr1 | 88236695 | C | G | 222 | Mroh2a | exonic | synonymous SNV | Mroh2a:NM_001281466:exon12:c.C1278G:p.R426R | . | 1qD | rs46995426 | 0 | 1 |
| chr1 | 88236753 | T | C | 222 | Mroh2a | intronic | . | . | . | 1qD | rs45956738 | 0 | 1 |
| chr1 | 88236813 | A | G | 209 | Mroh2a | intronic | . | . | . | 1qD | rs50799947 | 0 | 1 |
| chr1 | 88236938 | A | G | 115 | Mroh2a | intronic | . | . | . | 1qD | rs48827679 | 0 | 1 |
| chr1 | 88236966 | C | G | 109 | Mroh2a | intronic | . | . | . | 1qD | rs51294895 | 0 | 1 |
| chr1 | 88237199 | G | A | 83 | Mroh2a | intronic | . | . | . | 1qD | rs46651061 | 0 | 1 |
| chr1 | 88237279 | A | G | 117 | Mroh2a | intronic | . | . | . | 1qD | rs45633344 | 0 | 1 |
| chr1 | 88237300 | G | A | 88 | Mroh2a | intronic | . | . | . | 1qD | rs49078240 | 0 | 1 |
| chr1 | 88237380 | C | G | 207 | Mroh2a | intronic | . | . | . | 1qD | rs49259097 | 0 | 1 |
| chr1 | 88237457 | T | A | 222 | Mroh2a | intronic | . | . | . | 1qD | rs48101914 | 0 | 1 |
| chr1 | 88237541 | C | A | 222 | Mroh2a | exonic | synonymous SNV | Mroh2a:NM_001281466:exon13:c.C1383A:p.G461G | . | 1qD | rs45963845 | 0 | 1 |
| chr1 | 88237714 | C | T | 222 | Mroh2a | intronic | . | . | . | 1qD | rs51785534 | 0 | 1 |
| chr1 | 88237716 | A | G | 112 | Mroh2a | intronic | . | . | . | 1qD | rs47171996 | 0 | 1 |
| chr1 | 88237749 | C | G | 222 | Mroh2a | intronic | . | . | . | 1qD | rs47600260 | 0 | 1 |
| chr1 | 88237780 | G | A | 90 | Mroh2a | intronic | . | . | . | 1qD | rs50672514 | 0 | 1 |
| chr1 | 88237789 | A | T | 142 | Mroh2a | intronic | . | . | . | 1qD | rs46546338 | 0 | 1 |
| chr1 | 88237800 | T | A | 222 | Mroh2a | intronic | . | . | . | 1qD | rs51520385 | 0 | 1 |
| chr1 | 88237853 | A | C | 222 | Mroh2a | exonic | synonymous SNV | Mroh2a:NM_001281466:exon14:c.A1443C:p.R481R | . | 1qD | rs30306577 | 0 | 1 |
| chr1 | 88237976 | C | T | 222 | Mroh2a | intronic | . | . | . | 1qD | rs47338225 | 0 | 1 |
| chr1 | 88237988 | C | G | 221 | Mroh2a | intronic | . | . | . | 1qD | rs46817305 | 0 | 1 |
| chr1 | 88237998 | T | C | 221 | Mroh2a | intronic | . | . | . | 1qD | rs30306576 | 0 | 1 |
| chr1 | 88238816 | G | A | 123 | Mroh2a | intronic | . | . | . | 1qD | rs51977061 | 0 | 1 |
| chr1 | 88238889 | T | C | 221 | Mroh2a | intronic | . | . | . | 1qD | rs49591749 | 0 | 1 |
| chr1 | 88238947 | A | G | 221 | Mroh2a | exonic | synonymous SNV | Mroh2a:NM_001281466:exon15:c.A1599G:p.A533A | . | 1qD | rs50675978 | 0 | 1 |
| chr1 | 88239006 | A | C | 222 | Mroh2a | exonic | missense SNV | Mroh2a:NM_001281466:exon15:c.A1658C:p.D553A | . | 1qD | rs47431882 | 0 | 1 |
| chr1 | 88239134 | T | C | 221 | Mroh2a | intronic | . | . | . | 1qD | rs49307045 | 0 | 1 |
| chr1 | 88239797 | G | A | 96 | Mroh2a | intronic | . | . | . | 1qD | rs48744281 | 1 | 0 |
| chr1 | 88240029 | A | G | 138 | Mroh2a | intronic | . | . | . | 1qD | rs50711214 | 0 | 1 |
| chr1 | 88240124 | T | C | 222 | Mroh2a | exonic | synonymous SNV | Mroh2a:NM_001281466:exon16:c.T1699C:p.L567L | . | 1qD | rs51719921 | 0 | 1 |
| chr1 | 88240149 | C | T | 222 | Mroh2a | exonic | missense SNV | Mroh2a:NM_001281466:exon16:c.C1724T:p.A575V | . | 1qD | rs48472294 | 0 | 1 |
| chr1 | 88240225 | C | T | 222 | Mroh2a | intronic | . | . | . | 1qD | rs30306575 | 0 | 1 |
| chr1 | 88240342 | T | G | 215 | Mroh2a | intronic | . | . | . | 1qD | rs49533396 | 0 | 1 |
| chr1 | 88240387 | T | C | 133 | Mroh2a | intronic | . | . | . | 1qD | rs30306574 | 0 | 1 |
| chr1 | 88240410 | G | A | 184 | Mroh2a | intronic | . | . | . | 1qD | rs46041707 | 0 | 1 |
| chr1 | 88240507 | G | A | 160 | Mroh2a | intronic | . | . | . | 1qD | rs51030761 | 0 | 1 |
| chr1 | 88240703 | G | A | 185 | Mroh2a | exonic | missense SNV | Mroh2a:NM_001281466:exon17:c.G1759A:p.G587R | . | 1qD | rs46352364 | 0 | 1 |
| chr1 | 88240709 | G | T | 89 | Mroh2a | exonic | missense SNV | Mroh2a:NM_001281466:exon17:c.G1765T:p.G589C | . | 1qD | . | 0 | 1 |
| chr1 | 88240802 | C | T | 221 | Mroh2a | exonic | synonymous SNV | Mroh2a:NM_001281466:exon17:c.C1858T:p.L620L | . | 1qD | rs47373236 | 0 | 1 |
| chr1 | 88240839 | G | A | 221 | Mroh2a | intronic | . | . | . | 1qD | rs51509347 | 0 | 1 |
| chr1 | 88240969 | G | A | 221 | Mroh2a | intronic | . | . | . | 1qD | rs49834198 | 0 | 1 |
| chr1 | 88241093 | C | T | 221 | Mroh2a | exonic | synonymous SNV | Mroh2a:NM_001281466:exon18:c.C1899T:p.N633N | . | 1qD | rs30305673 | 0 | 1 |
| chr1 | 88241160 | G | A | 221 | Mroh2a | intronic | . | . | . | 1qD | rs47087507 | 0 | 1 |
| chr1 | 88241197 | C | T | 190 | Mroh2a | intronic | . | . | . | 1qD | rs47127906 | 0 | 1 |
| chr1 | 88241245 | T | C | 180 | Mroh2a | intronic | . | . | . | 1qD | rs51712461 | 0 | 1 |
| chr1 | 88241325 | C | A | 193 | Mroh2a | intronic | . | . | . | 1qD | rs51368881 | 0 | 1 |
| chr1 | 88241360 | T | C | 94 | Mroh2a | intronic | . | . | . | 1qD | rs45995677 | 0 | 1 |
| chr1 | 88241486 | T | G | 222 | Mroh2a | intronic | . | . | . | 1qD | rs51133357 | 0 | 1 |
| chr1 | 88241661 | G | A | 222 | Mroh2a | intronic | . | . | . | 1qD | rs49704559 | 0 | 1 |
| chr1 | 88241672 | A | G | 222 | Mroh2a | intronic | . | . | . | 1qD | rs46082509 | 0 | 1 |
| chr1 | 88241677 | T | C | 222 | Mroh2a | intronic | . | . | . | 1qD | rs51679286 | 0 | 1 |
| chr1 | 88241797 | G | A | 195 | Mroh2a | intronic | . | . | . | 1qD | rs51706734 | 0 | 1 |
| chr1 | 88241826 | G | T | 140 | Mroh2a | intronic | . | . | . | 1qD | rs47829931 | 0 | 1 |
| chr1 | 88241866 | T | C | 143 | Mroh2a | intronic | . | . | . | 1qD | rs51097550 | 0 | 1 |
| chr1 | 88242108 | T | C | 98 | Mroh2a | intronic | . | . | . | 1qD | rs30305671 | 0 | 1 |
| chr1 | 88242183 | G | T | 154 | Mroh2a | intronic | . | . | . | 1qD | rs30305670 | 0 | 1 |
| chr1 | 88242242 | C | T | 94 | Mroh2a | intronic | . | . | . | 1qD | rs49984831 | 0 | 1 |
| chr1 | 88242417 | G | T | 124 | Mroh2a | exonic | missense SNV | Mroh2a:NM_001281466:exon20:c.G2051T:p.R684M | . | 1qD | rs47723211 | 0 | 1 |
| chr1 | 88242418 | G | T | 189 | Mroh2a | exonic | missense SNV | Mroh2a:NM_001281466:exon20:c.G2052T:p.R684S | . | 1qD | rs49668968 | 0 | 1 |
| chr1 | 88242508 | T | C | 212 | Mroh2a | exonic | synonymous SNV | Mroh2a:NM_001281466:exon20:c.T2142C:p.N714N | . | 1qD | rs30305669 | 0 | 1 |
| chr1 | 88242511 | T | C | 222 | Mroh2a | exonic | synonymous SNV | Mroh2a:NM_001281466:exon20:c.T2145C:p.D715D | . | 1qD | rs30305668 | 0 | 1 |
| chr1 | 88242655 | C | G | 222 | Mroh2a | intronic | . | . | . | 1qD | rs30305667 | 0 | 1 |
| chr1 | 88242760 | C | T | 222 | Mroh2a | exonic | missense SNV | Mroh2a:NM_001281466:exon21:c.C2249T:p.S750L | . | 1qD | rs50641547 | 0 | 1 |
| chr1 | 88242764 | A | G | 222 | Mroh2a | exonic | synonymous SNV | Mroh2a:NM_001281466:exon21:c.A2253G:p.E751E | . | 1qD | rs30305666 | 0 | 1 |
| chr1 | 88242807 | T | G | 215 | Mroh2a | intronic | . | . | . | 1qD | rs51073242 | 0 | 1 |
| chr1 | 88244014 | G | A | 221 | Mroh2a | exonic | synonymous SNV | Mroh2a:NM_001281466:exon22:c.G2364A:p.Q788Q | . | 1qD | rs45714767 | 0 | 1 |
| chr1 | 88244118 | T | C | 222 | Mroh2a | intronic | . | . | . | 1qD | rs50772163 | 0 | 1 |
| chr1 | 88244146 | A | G | 208 | Mroh2a | intronic | . | . | . | 1qD | rs49969874 | 0 | 1 |
| chr1 | 88244780 | C | T | 139 | Mroh2a | intronic | . | . | . | 1qD | rs48284755 | 0 | 1 |
| chr1 | 88244844 | C | T | 221 | Mroh2a | intronic | . | . | . | 1qD | rs46985486 | 0 | 1 |
| chr1 | 88244970 | G | T | 221 | Mroh2a | exonic | missense SNV | Mroh2a:NM_001281466:exon23:c.G2460T:p.M820I | . | 1qD | . | 0 | 1 |
| chr1 | 88245126 | C | T | 220 | Mroh2a | intronic | . | . | . | 1qD | rs47667811 | 0 | 1 |
| chr1 | 88245145 | A | G | 221 | Mroh2a | intronic | . | . | . | 1qD | rs51651816 | 0 | 1 |
| chr1 | 88245187 | T | C | 167 | Mroh2a | intronic | . | . | . | 1qD | rs46408433 | 0 | 1 |
| chr1 | 88245291 | G | T | 132 | Mroh2a | intronic | . | . | . | 1qD | rs47356517 | 0 | 1 |
| chr1 | 88245733 | C | A | 77 | Mroh2a | intronic | . | . | . | 1qD | rs47403049 | 0 | 1 |
| chr1 | 88245743 | C | T | 106 | Mroh2a | intronic | . | . | . | 1qD | rs51968014 | 0 | 1 |
| chr1 | 88245871 | A | G | 185 | Mroh2a | intronic | . | . | . | 1qD | rs46194879 | 0 | 1 |
| chr1 | 88245878 | C | T | 206 | Mroh2a | intronic | . | . | . | 1qD | rs50582628 | 0 | 1 |
| chr1 | 88245949 | G | A | 221 | Mroh2a | intronic | . | . | . | 1qD | rs30304852 | 0 | 1 |
| chr1 | 88245989 | T | C | 221 | Mroh2a | exonic | missense SNV | Mroh2a:NM_001281466:exon24:c.T2558C:p.V853A | . | 1qD | rs48134977 | 0 | 1 |
| chr1 | 88246065 | A | G | 221 | Mroh2a | exonic | synonymous SNV | Mroh2a:NM_001281466:exon24:c.A2634G:p.L878L | . | 1qD | rs50479857 | 0 | 1 |
| chr1 | 88246132 | T | C | 222 | Mroh2a | intronic | . | . | . | 1qD | rs47336759 | 0 | 1 |
| chr1 | 88246156 | C | A | 219 | Mroh2a | intronic | . | . | . | 1qD | rs49267994 | 0 | 1 |
| chr1 | 88246230 | T | G | 222 | Mroh2a | intronic | . | . | . | 1qD | rs30304851 | 0 | 1 |
| chr1 | 88246338 | C | T | 25.8594 | Mroh2a | intronic | . | . | . | 1qD | rs49864288 | 0 | 1 |
| chr1 | 88246977 | A | C | 68 | Mroh2a | intronic | . | . | . | 1qD | rs51660884 | 0 | 1 |
| chr1 | 88247000 | G | A | 130 | Mroh2a | intronic | . | . | . | 1qD | rs48788585 | 0 | 1 |
| chr1 | 88247028 | T | C | 108 | Mroh2a | intronic | . | . | . | 1qD | rs47779264 | 0 | 1 |
| chr1 | 88247123 | T | C | 222 | Mroh2a | intronic | . | . | . | 1qD | rs45887350 | 0 | 1 |
| chr1 | 88247140 | A | C | 222 | Mroh2a | exonic | missense SNV | Mroh2a:NM_001281466:exon25:c.A2641C:p.M881L | . | 1qD | rs30304848 | 0 | 1 |
| chr1 | 88247232 | T | C | 222 | Mroh2a | exonic | synonymous SNV | Mroh2a:NM_001281466:exon25:c.T2733C:p.N911N | . | 1qD | rs46270163 | 0 | 1 |
| chr1 | 88247237 | C | T | 132 | Mroh2a | exonic | missense SNV | Mroh2a:NM_001281466:exon25:c.C2738T:p.S913F | . | 1qD | rs46834053 | 0 | 1 |

| | | | | | | | | | | | | | |
|---|---|---|---|---|---|---|---|---|---|---|---|---|---|
| chr1 | 88247683 | A | G | 178 | Mroh2a | intronic | . | . | . | 1qD | rs46917902 | 0 | 1 |
| chr1 | 88247763 | A | G | 220 | Mroh2a | exonic | missense SNV | Mroh2a:NM_001281466:exon26:c.A2761G:p.T921A | . | 1qD | rs51677514 | 0 | 1 |
| chr1 | 88247852 | T | C | 220 | Mroh2a | intronic | . | . | . | 1qD | rs30304847 | 0 | 1 |
| chr1 | 88247900 | G | A | 221 | Mroh2a | intronic | . | . | . | 1qD | rs48395532 | 0 | 1 |
| chr1 | 88247919 | G | T | 221 | Mroh2a | intronic | . | . | . | 1qD | rs49441570 | 0 | 1 |
| chr1 | 88248429 | A | T | 141 | Mroh2a | intronic | . | . | . | 1qD | rs50263445 | 0 | 1 |
| chr1 | 88248440 | G | A | 162 | Mroh2a | intronic | . | . | . | 1qD | rs50191844 | 0 | 1 |
| chr1 | 88248584 | G | A | 222 | Mroh2a | exonic | synonymous SNV | Mroh2a:NM_001281466:exon27:c.G2874A:p.E958E | . | 1qD | rs49606792 | 0 | 1 |
| chr1 | 88248614 | T | C | 222 | Mroh2a | exonic | synonymous SNV | Mroh2a:NM_001281466:exon27:c.T2904C:p.H968H | . | 1qD | rs50202562 | 0 | 1 |
| chr1 | 88248621 | C | T | 160 | Mroh2a | exonic | missense SNV | Mroh2a:NM_001281466:exon27:c.C2911T:p.L971F | . | 1qD | rs50937237 | 0 | 1 |
| chr1 | 88248816 | T | A | 168 | Mroh2a | intronic | . | . | . | 1qD | rs49835252 | 0 | 1 |
| chr1 | 88248830 | G | T | 135 | Mroh2a | intronic | . | . | . | 1qD | rs46721425 | 0 | 1 |
| chr1 | 88248844 | G | A | 98 | Mroh2a | intronic | . | . | . | 1qD | rs49491131 | 0 | 1 |
| chr1 | 88249457 | T | C | 123 | Mroh2a | intronic | . | . | . | 1qD | rs49268205 | 0 | 1 |
| chr1 | 88249557 | T | A | 222 | Mroh2a | intronic | . | . | . | 1qD | rs48851291 | 0 | 1 |
| chr1 | 88249568 | A | G | 221 | Mroh2a | intronic | . | . | . | 1qD | rs49928501 | 0 | 1 |
| chr1 | 88249700 | T | C | 221 | Mroh2a | splicing | . | . | . | 1qD | rs49354716 | 0 | 1 |
| chr1 | 88249861 | A | G | 221 | Mroh2a | intronic | . | . | . | 1qD | rs50960505 | 0 | 1 |
| chr1 | 88249869 | A | C | 221 | Mroh2a | intronic | . | . | . | 1qD | rs48854013 | 0 | 1 |
| chr1 | 88249970 | C | T | 221 | Mroh2a | intronic | . | . | . | 1qD | rs48055553 | 0 | 1 |
| chr1 | 88250045 | T | C | 222 | Mroh2a | intronic | . | . | . | 1qD | . | 0 | 1 |
| chr1 | 88250101 | T | C | 221 | Mroh2a | intronic | . | . | . | 1qD | rs30303912 | 0 | 1 |
| chr1 | 88250119 | A | G | 41.2767 | Mroh2a | intronic | . | . | . | 1qD | rs47889459 | 0 | 1 |
| chr1 | 88250266 | A | G | 220 | Mroh2a | exonic | synonymous SNV | Mroh2a:NM_001281466:exon29:c.A3072G:p.V1024V | . | 1qD | rs46922901 | 0 | 1 |
| chr1 | 88250393 | G | A | 221 | Mroh2a | intronic | . | . | . | 1qD | rs49466835 | 0 | 1 |
| chr1 | 88250425 | C | T | 222 | Mroh2a | intronic | . | . | . | 1qD | rs49769505 | 0 | 1 |
| chr1 | 88250436 | T | C | 221 | Mroh2a | intronic | . | . | . | 1qD | rs47847431 | 0 | 1 |
| chr1 | 88250492 | C | T | 213 | Mroh2a | intronic | . | . | . | 1qD | rs46623910 | 0 | 1 |
| chr1 | 88250557 | G | C | 155 | Mroh2a | intronic | . | . | . | 1qD | rs48249960 | 0 | 1 |
| chr1 | 88250616 | T | C | 98 | Mroh2a | intronic | . | . | . | 1qD | rs30303911 | 0 | 1 |
| chr1 | 88251195 | A | G | 169 | Mroh2a | intronic | . | . | . | 1qD | rs47026838 | 0 | 1 |
| chr1 | 88251229 | T | C | 222 | Mroh2a | intronic | . | . | . | 1qD | rs48741853 | 0 | 1 |
| chr1 | 88251267 | G | T | 222 | Mroh2a | exonic | missense SNV | Mroh2a:NM_001281466:exon30:c.G3212T:p.C1071F | . | 1qD | rs50826619 | 0 | 1 |
| chr1 | 88251346 | C | T | 222 | Mroh2a | exonic | synonymous SNV | Mroh2a:NM_001281466:exon30:c.C3291T:p.V1097V | . | 1qD | rs50151850 | 0 | 1 |
| chr1 | 88251385 | G | T | 222 | Mroh2a | exonic | missense SNV | Mroh2a:NM_001281466:exon30:c.G3330T:p.E1110D | . | 1qD | rs48746447 | 0 | 1 |
| chr1 | 88251459 | C | A | 222 | Mroh2a | intronic | . | . | . | 1qD | rs49402694 | 0 | 1 |
| chr1 | 88251472 | T | C | 182 | Mroh2a | intronic | . | . | . | 1qD | rs30303909 | 0 | 1 |
| chr1 | 88251479 | C | T | 167 | Mroh2a | intronic | . | . | . | 1qD | rs48272521 | 0 | 1 |
| chr1 | 88251561 | C | T | 62 | Mroh2a | intronic | . | . | . | 1qD | rs30303908 | 0 | 1 |
| chr1 | 88251584 | T | C | 63 | Mroh2a | intronic | . | . | . | 1qD | rs51732114 | 0 | 1 |
| chr1 | 88252313 | A | G | 221 | Mroh2a | intronic | . | . | . | 1qD | rs49686764 | 0 | 1 |
| chr1 | 88252383 | T | C | 221 | Mroh2a | exonic | synonymous SNV | Mroh2a:NM_001281466:exon31:c.T3390C:p.D1130D | . | 1qD | rs48997232 | 0 | 1 |
| chr1 | 88252458 | A | G | 221 | Mroh2a | exonic | synonymous SNV | Mroh2a:NM_001281466:exon31:c.A3465G:p.T1155T | . | 1qD | rs50204196 | 0 | 1 |
| chr1 | 88252477 | C | T | 221 | Mroh2a | exonic | synonymous SNV | Mroh2a:NM_001281466:exon31:c.C3484T:p.L1162L | . | 1qD | rs51522420 | 0 | 1 |
| chr1 | 88252622 | A | G | 221 | Mroh2a | intronic | . | . | . | 1qD | rs46950229 | 0 | 1 |
| chr1 | 88252625 | T | C | 221 | Mroh2a | intronic | . | . | . | 1qD | rs45991953 | 0 | 1 |
| chr1 | 88252669 | C | T | 172 | Mroh2a | intronic | . | . | . | 1qD | rs46710367 | 0 | 1 |
| chr1 | 88254612 | A | G | 22.8054 | Mroh2a | intronic | . | . | . | 1qD | . | 0 | 1 |
| chr1 | 88254676 | C | T | 82 | Mroh2a | intronic | . | . | . | 1qD | rs585910369 | 0 | 1 |
| chr1 | 88254739 | C | T | 149 | Mroh2a | intronic | . | . | . | 1qD | rs108215745 | 0 | 1 |
| chr1 | 88254845 | T | C | 221 | Mroh2a | intronic | . | . | . | 1qD | . | 0 | 1 |
| chr1 | 88254906 | A | C | 79 | Mroh2a | exonic | synonymous SNV | Mroh2a:NM_001281466:exon32:c.A3546C:p.A1182A | . | 1qD | rs48050069 | 0 | 1 |
| chr1 | 88255048 | C | T | 206 | Mroh2a | intronic | . | . | . | 1qD | rs50957006 | 0 | 1 |
| chr1 | 88255050 | G | C | 221 | Mroh2a | intronic | . | . | . | 1qD | . | 0 | 1 |
| chr1 | 88255245 | T | C | 220 | Mroh2a | exonic | synonymous SNV | Mroh2a:NM_001281466:exon33:c.T3813C:p.V1271V | . | 1qD | rs30303027 | 0 | 1 |
| chr1 | 88255315 | G | A | 190 | Mroh2a | intronic | . | . | . | 1qD | rs48530924 | 0 | 1 |
| chr1 | 88255375 | A | C | 45.1577 | Mroh2a | intronic | . | . | . | 1qD | . | 0 | 1 |
| chr1 | 88255409 | A | C | 222 | Mroh2a | intronic | . | . | . | 1qD | . | 0 | 1 |
| chr1 | 88255427 | C | A | 77 | Mroh2a | intronic | . | . | . | 1qD | . | 0 | 1 |
| chr1 | 88255539 | C | A | 83 | Mroh2a | splicing | . | . | . | 1qD | rs49874282 | 0 | 1 |
| chr1 | 88255847 | C | G | 221 | Mroh2a | intronic | . | . | . | 1qD | rs46677296 | 0 | 1 |
| chr1 | 88255894 | A | G | 182 | Mroh2a | intronic | . | . | . | 1qD | rs48372537 | 0 | 1 |
| chr1 | 88255965 | T | C | 147 | Mroh2a | intronic | . | . | . | 1qD | rs50825190 | 0 | 1 |
| chr1 | 88255968 | A | G | 136 | Mroh2a | intronic | . | . | . | 1qD | rs49772798 | 0 | 1 |
| chr1 | 88255977 | T | A | 45.208 | Mroh2a | intronic | . | . | . | 1qD | . | 0 | 1 |
| chr1 | 88256029 | T | A | 221 | Mroh2a | intronic | . | . | . | 1qD | rs49956734 | 0 | 1 |
| chr1 | 88256090 | G | A | 109 | Mroh2a | exonic | synonymous SNV | Mroh2a:NM_001281466:exon35:c.G4023A:p.Q1341Q | . | 1qD | rs50502368 | 0 | 1 |
| chr1 | 88256127 | G | A | 146 | Mroh2a | exonic | missense SNV | Mroh2a:NM_001281466:exon35:c.G4060A:p.E1354K | . | 1qD | rs49915201 | 0 | 1 |
| chr1 | 88256213 | G | A | 190 | Mroh2a | intronic | . | . | . | 1qD | rs51025913 | 0 | 1 |
| chr1 | 88256323 | C | T | 218 | Mroh2a | intronic | . | . | . | 1qD | rs51137176 | 0 | 1 |
| chr1 | 88256324 | C | T | 221 | Mroh2a | intronic | . | . | . | 1qD | rs48153795 | 0 | 1 |
| chr1 | 88256326 | T | G | 134 | Mroh2a | intronic | . | . | . | 1qD | rs51004598 | 0 | 1 |
| chr1 | 88256392 | T | C | 221 | Mroh2a | intronic | . | . | . | 1qD | rs51223356 | 0 | 1 |
| chr1 | 88256468 | T | C | 221 | Mroh2a | exonic | synonymous SNV | Mroh2a:NM_001281466:exon36:c.T4173C:p.G1391G | . | 1qD | rs49242808 | 0 | 1 |
| chr1 | 88256597 | A | G | 221 | Mroh2a | intronic | . | . | . | 1qD | rs51909207 | 0 | 1 |
| chr1 | 88256705 | C | T | 221 | Mroh2a | exonic | stopgain | Mroh2a:NM_001281466:exon37:c.C4309T:p.Q1437X | . | 1qD | rs48861546 | 0 | 1 |
| chr1 | 88256727 | C | T | 221 | Mroh2a | exonic | missense SNV | Mroh2a:NM_001281466:exon37:c.C4331T:p.A1444V | . | 1qD | rs51219523 | 0 | 1 |
| chr1 | 88256798 | G | A | 157 | Mroh2a | exonic | missense SNV | Mroh2a:NM_001281466:exon37:c.G4402A:p.A1468T | . | 1qD | rs30303026 | 0 | 1 |
| chr1 | 88256871 | G | C | 174 | Mroh2a | intronic | . | . | . | 1qD | rs52517967 | 0 | 1 |
| chr1 | 88257463 | G | A | 67 | Mroh2a | intronic | . | . | . | 1qD | . | 0 | 1 |
| chr1 | 88257647 | G | T | 204 | Mroh2a | intronic | . | . | . | 1qD | . | 0 | 1 |
| chr1 | 88257683 | C | T | 221 | Mroh2a | intronic | . | . | . | 1qD | . | 0 | 1 |
| chr1 | 88257739 | T | C | 221 | Mroh2a | exonic | synonymous SNV | Mroh2a:NM_001281466:exon38:c.T4467C:p.F1489F | . | 1qD | rs45916248 | 0 | 1 |
| chr1 | 88257928 | A | G | 220 | Mroh2a | intronic | . | . | . | 1qD | rs48983878 | 0 | 1 |
| chr1 | 88258356 | T | C | 113 | Mroh2a | intronic | . | . | . | 1qD | rs30303024 | 0 | 1 |
| chr1 | 88258376 | G | A | 73 | Mroh2a | intronic | . | . | . | 1qD | . | 0 | 1 |
| chr1 | 88258500 | G | C | 189 | Mroh2a | intronic | . | . | . | 1qD | . | 0 | 1 |
| chr1 | 88258633 | C | T | 214 | Mroh2a | exonic | synonymous SNV | Mroh2a:NM_001281466:exon39:c.C4644T:p.S1548S | . | 1qD | . | 0 | 1 |
| chr1 | 88258723 | A | G | 219 | Mroh2a | intronic | . | . | . | 1qD | rs45783408 | 0 | 1 |
| chr1 | 88258731 | A | G | 222 | Mroh2a | intronic | . | . | . | 1qD | rs47839015 | 0 | 1 |
| chr1 | 88258743 | T | A | 191 | Mroh2a | intronic | . | . | . | 1qD | . | 0 | 1 |
| chr1 | 88258754 | C | G | 185 | Mroh2a | intronic | . | . | . | 1qD | . | 0 | 1 |
| chr1 | 88259369 | C | T | 222 | Mroh2a,Hjurp | UTR3 | . | . | . | 1qD | rs46221671 | 0 | 1 |
| chr1 | 88259370 | C | G | 221 | Hjurp,Mroh2a | UTR3 | . | . | . | 1qD | rs49917120 | 0 | 1 |
| chr1 | 88259436 | G | C | 176 | Hjurp,Mroh2a | UTR3 | . | . | . | 1qD | rs581093463 | 0 | 1 |
| chr1 | 88259567 | A | G | 221 | Hjurp,Mroh2a | exonic | synonymous SNV | Mroh2a:NM_001281466:exon41:c.A4866G:p.K1622K | . | 1qD | rs30301550 | 0 | 1 |
| chr1 | 88259678 | A | G | 221 | Hjurp,Mroh2a | UTR3 | . | . | . | 1qD | rs49400006 | 0 | 1 |
| chr1 | 88259793 | A | G | 220 | Mroh2a,Hjurp | UTR3 | . | . | . | 1qD | rs47144880 | 0 | 1 |
| chr1 | 88260002 | A | G | 222 | Hjurp,Mroh2a | exonic | synonymous SNV | Mroh2a:NM_001281466:exon42:c.A4992G:p.L1664L | . | 1qD | rs50377244 | 0 | 1 |
| chr1 | 88260004 | C | T | 67 | Mroh2a,Hjurp | exonic | missense SNV | Mroh2a:NM_001281466:exon42:c.C4994T:p.A1665V | . | 1qD | . | 0 | 1 |
| chr1 | 88260005 | G | A | 59 | Hjurp,Mroh2a | exonic | synonymous SNV | Mroh2a:NM_001281466:exon42:c.G4995A:p.A1665A | . | 1qD | . | 0 | 1 |
| chr1 | 88264978 | G | A | 92 | Hjurp,6430706D22 | ncRNA_exonic | . | . | . | 1qD | rs49629258 | 0 | 1 |
| chr1 | 88265034 | G | A | 146 | Hjurp,6430706D22 | ncRNA_exonic | . | . | . | 1qD | rs51185680 | 0 | 1 |
| chr1 | 88265122 | G | A | 221 | Hjurp,6430706D22 | ncRNA_exonic | . | . | . | 1qD | rs30301545 | 0 | 1 |
| chr1 | 88265258 | A | G | 221 | A730008H23Rik,643 | exonic | missense SNV | A730008H23Rik:NM_172505:exon5:c.T1699C:p.C567R,Hjurp:NM_198652:exon8:c.T1927C:p.C643R | . | 1qD | rs13472325 | 0 | 1 |
| chr1 | 88265309 | C | G | 222 | A730008H23Rik,643 | exonic | missense SNV | A730008H23Rik:NM_172505:exon5:c.G1648C:p.A550P,Hjurp:NM_198652:exon8:c.G1876C:p.A626P | . | 1qD | rs50378893 | 0 | 1 |
| chr1 | 88265472 | C | T | 221 | Hjurp,6430706D22 | exonic | synonymous SNV | A730008H23Rik:NM_172505:exon5:c.G1485A:p.A495A,Hjurp:NM_198652:exon8:c.G1713A:p.A571A | . | 1qD | rs45762281 | 0 | 1 |
| chr1 | 88265570 | T | A | 221 | Hjurp,A730008H23 | exonic | missense SNV | A730008H23Rik:NM_172505:exon5:c.A1387T:p.S463C,Hjurp:NM_198652:exon8:c.A1615T:p.S539C | . | 1qD | rs30301544 | 0 | 1 |
| chr1 | 88265846 | A | C | 222 | A730008H23Rik,643 | exonic | missense SNV | A730008H23Rik:NM_172505:exon5:c.T1111G:p.S371A,Hjurp:NM_198652:exon8:c.T1339G:p.S447A | . | 1qD | rs49199385 | 0 | 1 |
| chr1 | 88265980 | T | G | 221 | Hjurp,A730008H23 | exonic | missense SNV | A730008H23Rik:NM_172505:exon5:c.A977C:p.Q326P,Hjurp:NM_198652:exon8:c.A1205C:p.Q402P | . | 1qD | rs30300753 | 0 | 1 |
| chr1 | 88266195 | G | A | 220 | A730008H23Rik,643 | exonic | synonymous SNV | A730008H23Rik:NM_172505:exon5:c.C762T:p.H254H,Hjurp:NM_198652:exon8:c.C990T:p.H330H | . | 1qD | rs46656348 | 0 | 1 |
| chr1 | 88266696 | T | C | 221 | A730008H23Rik,643 | ncRNA_intronic | . | . | . | 1qD | rs52012259 | 0 | 1 |
| chr1 | 88267831 | C | A | 55 | 6430706D22Rik,A73 | ncRNA_intronic | . | . | . | 1qD | rs46861852 | 0 | 1 |
| chr1 | 88267879 | T | C | 221 | Hjurp,6430706D22 | ncRNA_intronic | . | . | . | 1qD | rs45950719 | 0 | 1 |
| chr1 | 88269125 | T | C | 170 | A730008H23Rik,643 | ncRNA_intronic | . | . | . | 1qD | rs30300748 | 0 | 1 |
| chr1 | 88269285 | C | T | 221 | Hjurp,A730008H23 | ncRNA_intronic | . | . | . | 1qD | rs30300746 | 0 | 1 |
| chr1 | 88269331 | T | C | 222 | 6430706D22Rik,A73 | exonic | missense SNV | Hjurp:NM_198652:exon6:c.A437G:p.E146G | . | 1qD | rs48809660 | 0 | 1 |
| chr1 | 88269595 | A | T | 181 | Hjurp,A730008H23 | intronic | . | . | . | 1qD | rs30300745 | 0 | 1 |
| chr1 | 88269698 | A | G | 223 | Hjurp,A730008H23 | intronic | . | . | . | 1qD | rs47391518 | 1 | 0 |
| chr1 | 88269747 | G | A | 112 | Hjurp,A730008H23 | intronic | . | . | . | 1qD | rs48616191 | 1 | 0 |
| chr1 | 88269869 | C | T | 120 | A730008H23Rik,Hju | intronic | . | . | . | 1qD | rs45867573 | 1 | 0 |
| chr1 | 88269884 | C | G | 20.0279 | Hjurp,A730008H23 | intronic | . | . | . | 1qD | . | 0 | 1 |
| chr1 | 88270055 | A | G | 109 | A730008H23Rik,Hju | intronic | . | . | . | 1qD | rs46341349 | 0 | 1 |
| chr1 | 88270078 | T | C | 132 | A730008H23Rik,Hju | intronic | . | . | . | 1qD | rs30300744 | 0 | 1 |
| chr1 | 88270169 | A | G | 222 | A730008H23Rik,Hju | intronic | . | . | . | 1qD | rs252030215 | 0 | 1 |
| chr1 | 88270170 | A | G | 222 | A730008H23Rik,Hju | intronic | . | . | . | 1qD | rs46961208 | 0 | 1 |
| chr1 | 88270269 | A | G | 200 | A730008H23Rik,Hju | exonic | missense SNV | Hjurp:NM_198652:exon5:c.T287C:p.L96S | . | 1qD | rs228983144 | 0 | 1 |
| chr1 | 88270274 | C | T | 222 | Hjurp,A730008H23 | exonic | synonymous SNV | Hjurp:NM_198652:exon5:c.G282A:p.T94T | . | 1qD | rs266217461 | 0 | 1 |
| chr1 | 88272649 | G | C | 141 | A730008H23Rik,Hju | intronic | . | . | . | 1qD | rs50347710 | 0 | 1 |
| chr1 | 88272675 | T | C | 171 | A730008H23Rik,Hju | intronic | . | . | . | 1qD | rs46962040 | 0 | 1 |
| chr1 | 88272689 | T | C | 168 | Hjurp,A730008H23 | intronic | . | . | . | 1qD | rs51310927 | 0 | 1 |
| chr1 | 88272931 | A | C | 148 | Hjurp,A730008H23 | intronic | . | . | . | 1qD | rs30299921 | 0 | 1 |
| chr1 | 88272948 | G | A | 173 | Hjurp,A730008H23 | intronic | . | . | . | 1qD | rs48581229 | 0 | 1 |
| chr1 | 88272996 | C | A | 160 | A730008H23Rik,Hju | intronic | . | . | . | 1qD | rs47615153 | 0 | 1 |
| chr1 | 88274747 | G | A | 102 | Hjurp,A730008H23 | intronic | . | . | . | 1qD | rs50613675 | 0 | 1 |
| chr1 | 88274819 | C | G | 134 | A730008H23Rik,Hju | intronic | . | . | . | 1qD | rs30299916 | 0 | 1 |
| chr1 | 88274858 | G | T | 39.9329 | A730008H23Rik,Hju | intronic | . | . | . | 1qD | rs51073193 | 0 | 1 |
| chr1 | 88274928 | G | A | 164 | Hjurp,A730008H23 | intronic | . | . | . | 1qD | rs49167476 | 0 | 1 |
| chr1 | 88274942 | A | G | 221 | A730008H23Rik,Hju | intronic | . | . | . | 1qD | rs50699336 | 0 | 1 |
| chr1 | 88275134 | G | T | 222 | Hjurp,A730008H23 | intronic | . | . | . | 1qD | rs46566500 | 0 | 1 |
| chr1 | 88275143 | G | A | 222 | Hjurp,A730008H23 | intronic | . | . | . | 1qD | rs48860393 | 0 | 1 |
| chr1 | 88275145 | A | G | 222 | A730008H23Rik,Hju | intronic | . | . | . | 1qD | rs49427637 | 0 | 1 |
| chr1 | 88275231 | C | T | 185 | Hjurp,A730008H23 | intronic | . | . | . | 1qD | rs30299033 | 0 | 1 |
| chr1 | 88275363 | C | G | 107 | A730008H23Rik,Hju | intronic | . | . | . | 1qD | rs49293338 | 0 | 1 |
| chr1 | 88277152 | A | G | 176 | Hjurp,A730008H23 | intronic | . | . | . | 1qD | rs51442739 | 0 | 1 |
| chr1 | 88277167 | T | C | 81 | A730008H23Rik,Hju | intronic | . | . | . | 1qD | rs46211197 | 0 | 1 |
| chr1 | 88277238 | T | C | 222 | Hjurp,A730008H23 | exonic | synonymous SNV | A730008H23Rik:NM_172505:exon2:c.A144G:p.L48L,Hjurp:NM_198652:exon2:c.A144G:p.L48L | 491 | 1qD | rs30298182 | 0 | 1 |
| chr1 | 88277304 | T | G | 221 | A730008H23Rik,Hju | intronic | . | . | 491 | 1qD | rs49198881 | 0 | 1 |
| chr1 | 88277413 | A | T | 222 | Hjurp,A730008H23 | exonic | missense SNV | A730008H23Rik:NM_172505:exon1:c.T57A:p.S19R,Hjurp:NM_198652:exon1:c.T57A:p.S19R | 491 | 1qD | rs47427546 | 0 | 1 |
| chr1 | 88277422 | C | A | 176 | Hjurp,A730008H23 | exonic | missense SNV | A730008H23Rik:NM_172505:exon1:c.G48T:p.K16N,Hjurp:NM_198652:exon1:c.G48T:p.K16N | 491 | 1qD | rs30298179 | 0 | 1 |
| chr1 | 88277442 | T | A | 159 | Hjurp,A730008H23 | exonic | missense SNV | A730008H23Rik:NM_172505:exon1:c.A28T:p.R10W,Hjurp:NM_198652:exon1:c.A28T:p.R10W | 491 | 1qD | rs47146764 | 0 | 1 |
| chr1 | 88277475 | A | G | 112 | A730008H23Rik,Hju | UTR5 | . | . | 491 | 1qD | rs31472766 | 0 | 1 |
| chr1 | 88277507 | A | G | 152 | Hjurp,A730008H23 | UTR5 | . | . | 491 | 1qD | rs49303998 | 0 | 1 |
| chr1 | 88277525 | A | G | 146 | A730008H23Rik,Hju | UTR5 | . | . | 491 | 1qD | rs30298178 | 0 | 1 |
| chr1 | 89145813 | G | A | 98 | Sh3bp4 | exonic | synonymous SNV | Sh3bp4:NM_133816:exon4:c.G2382A:p.P794P | . | 1qD | . | 0 | 1 |
| chr1 | 89671214 | A | T | 221 | Agap1 | exonic | missense SNV | Agap1:NM_001037136:exon9:c.A993T:p.K331N,Agap1:NM_178119:exon9:c.A993T:p.K331N | . | 1qD | . | 0 | 1 |
| chr1 | 90810319 | G | T | 88 | Col6a3 | exonic | synonymous SNV | Col6a3:NM_001243009:exon5:c.C1656A:p.P552P,Col6a3:NM_001243008:exon8:c.C3477A:p.P1159P | . | 1qD | . | 0 | 1 |
| chr1 | 91341924 | C | A | 193 | Espnl | exonic | missense SNV | Espnl:NM_001033292:exon7:c.C1181A:p.T394N | . | 1qD | . | 0 | 1 |
| chr1 | 91345650 | C | T | 155 | Espnl | exonic | missense SNV | Espnl:NM_001033292:exon9:c.C2731T:p.R911C | 1360 | 1qD | . | 0 | 1 |
| chr1 | 91412689 | C | A | 145 | Hes6 | intronic | . | . | 1000 | 1qD | . | 0 | 1 |
| chr1 | 91412873 | C | G | 220 | Hes6 | intronic | . | . | 1000 | 1qD | . | 0 | 1 |
| chr1 | 93256141 | C | A | 102 | Sned1 | intronic | . | . | . | 1qD | . | 0 | 1 |
| chr1 | 93262013 | G | C | 61 | Sned1 | intronic | . | . | . | 1qD | . | 0 | 1 |
| chr1 | 93319980 | C | T | 67 | Pask | exonic | missense SNV | Pask:NM_080850:exon12:c.G3232A:p.E1078K | . | 1qD | . | 0 | 1 |
| chr1 | 93419939 | G | A | 22.0644 | Hdlbp | intronic | . | . | . | 1qD | . | 0 | 1 |
| chr1 | 93421826 | C | A | 221 | Hdlbp | intronic | . | . | . | 1qD | . | 0 | 1 |
| chr1 | 93436991 | G | T | 31.6586 | Hdlbp | intronic | . | . | . | 1qD | . | 0 | 1 |
| chr1 | 93620500 | A | G | 189 | Farp2 | exonic | missense SNV | Farp2:NM_145519:exon26:c.A2906G:p.Y969C | . | 1qD | . | 0 | 1 |
| chr1 | 95325147 | T | C | 100 | Fam174a | exonic | missense SNV | Fam174a:NM_026321:exon2:c.T473C:p.V158A | . | 1qD | . | 0 | 1 |
| chr1 | 95498829 | A | C | 151 | . | intergenic | . | . | . | 1qD | . | 0 | 1 |
| chr1 | 95627550 | G | A | 62 | St8sia4 | exonic | stopgain | St8sia4:NM_001159745:exon4:c.C754T:p.R252X,St8sia4:NM_009183:exon4:c.C754T:p.R252X | . | 1qD | . | 0 | 1 |
| chr1 | 96823539 | T | A | 66 | Slco4c1 | intronic | . | . | . | 1qD | . | 0 | 1 |
| chr1 | 96842469 | A | G | 138 | Slco4c1 | exonic | missense SNV | Slco4c1:NM_172658:exon4:c.T869C:p.I290T | . | 1qD | . | 0 | 1 |
| chr1 | 96842755 | A | C | 42.3497 | Slco4c1 | intronic | . | . | . | 1qD | . | 0 | 1 |
| chr1 | 97125506 | C | T | 81 | Slco6c1 | intronic | . | . | . | 1qD | . | 0 | 1 |
| chr1 | 98267578 | C | T | 30.7597 | . | intergenic | . | . | . | 1qD | . | 0 | 1 |
| chr1 | 98497724 | C | T | 90 | Slco6d1 | intronic | . | . | . | 1qD | . | 0 | 1 |
| chr1 | 100282098 | G | T | 33.0221 | Cntnap5b | intronic | . | . | . | 1qE1.1 | . | 0 | 1 |
| chr1 | 100446945 | G | A | 96 | Cntnap5b | intronic | . | . | . | 1qE1.1 | rs262183903 | 0 | 1 |
| chr1 | 100484726 | C | A | 116 | Cntnap5b | UTR3 | . | . | . | 1qE1.1 | . | 0 | 1 |
| chr1 | 102837871 | C | A | 124 | . | intergenic | . | . | . | 1qE1.2 | . | 0 | 1 |
| chr1 | 102837872 | C | A | 99 | . | intergenic | . | . | . | 1qE1.2 | . | 0 | 1 |
| chr1 | 105809387 | G | T | 222 | Tnfrsf11a | exonic | missense SNV | Tnfrsf11a:NM_009399:exon3:c.G218T:p.G73V | . | 1qE2.1 | . | 0 | 1 |
| chr1 | 105813726 | G | T | 92 | Tnfrsf11a | exonic | missense SNV | Tnfrsf11a:NM_009399:exon4:c.G362T:p.W121L | . | 1qE2.1 | . | 0 | 1 |
| chr1 | 106734739 | T | C | 42.0962 | Kdsr | intronic | . | . | . | 1qE2.1 | . | 0 | 1 |

| | | | | | | | | | | | | | |
|---|---|---|---|---|---|---|---|---|---|---|---|---|---|
| chr1 | 106774222 | T | A | 61 | Vps4b | intronic | . | . | . | 1qE2.1 | . | 0 | 1 |
| chr1 | 106777440 | C | T | 60 | Vps4b | exonic | missense SNV | Vps4b:NM_009190:exon9:c.G922A:p.A308T | . | 1qE2.1 | . | 0 | 1 |
| chr1 | 106888632 | C | A | 47.2675 | . | intergenic | . | . | . | 1qE2.1 | . | 0 | 1 |
| chr1 | 106956552 | G | T | 221 | Serpinb12 | exonic | stopgain | Serpinb12:NM_001199213:exon8:c.G1027T:p.E343X,Serpinb12:NM_027971:exon8:c.G1027T:p.E343X | . | 1qE2.1 | . | 0 | 1 |
| chr1 | 107000626 | C | A | 125 | Serpinb13 | exonic | synonymous SNV | Serpinb13:NM_172852:exon8:c.C975A:p.A325A | . | 1qE2.1 | . | 0 | 1 |
| chr1 | 107605905 | T | C | 173 | Serpinb8 | exonic | missense SNV | Serpinb8:NM_001159748:exon6:c.T689C:p.L230P,Serpinb8:NM_011459:exon6:c.T689C:p.L230P | . | 1qE2.1 | . | 0 | 1 |
| chr1 | 112138060 | T | C | 44.6868 | . | intergenic | . | . | . | 1qE2.1 | . | 0 | 1 |
| chr1 | 112138480 | C | A | 86 | . | intergenic | . | . | . | 1qE2.1 | . | 0 | 1 |
| chr1 | 118664615 | A | C | 103 | Tfcp2l1 | intronic | . | . | . | 1qE2.3 | . | 0 | 1 |
| chr1 | 120479188 | T | C | 76 | Marco | intronic | . | . | . | 1qE2.3 | . | 0 | 1 |
| chr1 | 121566237 | A | G | 156 | Ddx18 | intronic | . | . | . | 1qE2.3 | . | 0 | 1 |
| chr1 | 126027595 | T | G | 76 | Nckap5 | exonic | missense SNV | Nckap5:NM_172484:exon10:c.A1219C:p.T407P,Nckap5:NM_001081756:exon12:c.A1423C:p.T475P | . | 1qE3 | . | 0 | 1 |
| chr1 | 127282904 | A | T | 111 | Mgat5 | intronic | . | . | . | 1qE3 | . | 0 | 1 |
| chr1 | 127748775 | G | T | 131 | Acmsd | intronic | . | . | . | 1qE3 | . | 0 | 1 |
| chr1 | 127820596 | A | T | 37.809 | Map3k19 | intronic | . | . | . | 1qE3 | . | 0 | 1 |
| chr1 | 128223318 | G | T | 84 | R3hdm1 | intronic | . | . | . | 1qE3 | . | 0 | 1 |
| chr1 | 128345932 | C | T | 38.7245 | Mcm6 | exonic | missense SNV | Mcm6:NM_001313695:exon8:c.G1160A:p.R387H,Mcm6:NM_008567:exon8:c.G1160A:p.R387H | . | 1qE3 | . | 0 | 1 |
| chr1 | 130743128 | A | C | 81 | AA986860 | exonic | missense SNV | AA986860:NM_177604:exon4:c.A1086C:p.Q362H | . | 1qE4 | . | 0 | 1 |
| chr1 | 130848603 | A | G | 63 | Pigr | exonic | missense SNV | Pigr:NM_011082:exon7:c.A1874G:p.E625G | . | 1qE4 | . | 0 | 1 |
| chr1 | 130848643 | C | T | 163 | Pigr | intronic | . | . | . | 1qE4 | . | 0 | 1 |
| chr1 | 131129591 | G | T | 58 | Dyrk3 | exonic | missense SNV | Dyrk3:NM_145508:exon3:c.C844A:p.H282N | . | 1qE4 | . | 0 | 1 |
| chr1 | 131155676 | T | G | 59 | Eif2d | exonic | synonymous SNV | Eif2d:NM_001136070:exon4:c.T375G:p.P125P,Eif2d:NM_010709:exon4:c.T375G:p.P125P | . | 1qE4 | . | 0 | 1 |
| chr1 | 131804452 | C | A | 22.431 | Pm20d1 | intronic | . | . | . | 1qE4 | . | 0 | 1 |
| chr1 | 132023666 | G | A | 58 | Elk4 | UTR3 | . | . | . | 1qE4 | . | 0 | 1 |
| chr1 | 132331911 | G | T | 222 | Nuak2 | exonic | missense SNV | Nuak2:NM_028778:exon7:c.G1426T:p.G476W,Nuak2:NM_001195025:exon8:c.G1450T:p.G484W | . | 1qE4 | . | 0 | 1 |
| chr1 | 132610421 | T | G | 221 | Nfasc | exonic | missense SNV | Nfasc:NM_001160316:exon12:c.A1481C:p.N494T,Nfasc:NM_001160318:exon13:c.A1430C:p.N477T,Nfasc:N | . | 1qE4 | . | 0 | 1 |
| chr1 | 133365652 | G | T | 197 | Etnk2 | exonic | missense SNV | Etnk2:NM_175443:exon2:c.G332T:p.R111L | . | 1qE4 | . | 0 | 1 |
| chr1 | 133610338 | C | T | 76 | . | upstream | . | . | 683 | 1qE4 | . | 0 | 1 |
| chr1 | 133711631 | C | T | 36.9216 | Atp2b4 | splicing | . | . | . | 1qE4 | . | 0 | 1 |
| chr1 | 134196934 | G | C | 221 | Mybph | intronic | . | . | . | 1qE4 | . | 0 | 1 |
| chr1 | 134234064 | G | T | 78 | Adora1 | exonic | missense SNV | Adora1:NM_001008533:exon1:c.C292A:p.L98M,Adora1:NM_001039510:exon2:c.C292A:p.L98M,Adora1:NM_( | . | 1qE4 | . | 0 | 1 |
| chr1 | 134744598 | C | A | 38.3989 | Syt2 | intronic | . | . | . | 1qE4 | . | 0 | 1 |
| chr1 | 134773266 | C | A | 115 | Ppp1r12b | intronic | . | . | . | 1qE4 | rs47824414 | 1 | 0 |
| chr1 | 135309019 | G | A | 181 | Timm17a | intronic | . | . | . | 1qE4 | . | 0 | 1 |
| chr1 | 136216170 | C | A | 207 | 5730559C18Rik | exonic | missense SNV | 5730559C18Rik:NM_028872:exon9:c.G1527T:p.W509C | . | 1qE4 | . | 0 | 1 |
| chr1 | 136226721 | G | C | 160 | 5730559C18Rik | intronic | . | . | . | 1qE4 | . | 0 | 1 |
| chr1 | 136281297 | G | A | 169 | Camsap2 | exonic | missense SNV | Camsap2:NM_001081360:exon13:c.C2474T:p.S825L | . | 1qE4 | . | 0 | 1 |
| chr1 | 136293237 | T | A | 108 | Camsap2 | intronic | . | . | . | 1qE4 | . | 0 | 1 |
| chr1 | 136495954 | A | T | 221 | Kif14 | exonic | synonymous SNV | Kif14:NM_001287179:exon15:c.A2856T:p.T952T | . | 1qE4 | . | 0 | 1 |
| chr1 | 136945119 | A | G | 34.7496 | Nr5a2 | intronic | . | . | . | 1qE4 | . | 0 | 1 |
| chr1 | 138083971 | C | A | 87 | Ptprc | intronic | . | . | . | 1qE4 | . | 0 | 1 |
| chr1 | 138088568 | G | T | 221 | Ptprc | exonic | missense SNV | Ptprc:NM_001268286:exon16:c.C1555A:p.R519S,Ptprc:NM_011210:exon17:c.C1627A:p.R543S,Ptprc:NM_( | . | 1qE4 | . | 0 | 1 |
| chr1 | 138094403 | T | G | 82 | Ptprc | intronic | . | . | . | 1qE4 | . | 0 | 1 |
| chr1 | 138832508 | G | C | 110 | Lhx9 | intronic | . | . | . | 1qE4 | . | 0 | 1 |
| chr1 | 139169966 | G | T | 221 | Dennd1b | exonic | missense SNV | Dennd1b:NM_181347:exon21:c.G1744T:p.V582L,Dennd1b:NM_001166501:exon22:c.G1969T:p.V657L | . | 1qE4 | . | 0 | 1 |
| chr1 | 139328071 | T | C | 166 | Crb1 | intronic | . | . | . | 1qE4 | . | 0 | 1 |
| chr1 | 139701593 | A | T | 121 | Gm4788 | intronic | . | . | . | 1qF | . | 0 | 1 |
| chr1 | 139878678 | C | T | 151 | . | intergenic | . | . | . | 1qF | . | 0 | 1 |
| chr1 | 140048100 | T | A | 221 | . | intergenic | . | . | . | 1qF | . | 0 | 1 |
| chr1 | 140102784 | T | G | 221 | Cfh | exonic | missense SNV | Cfh:NM_009888:exon16:c.A2565C:p.Q855H | . | 1qF | . | 0 | 1 |
| chr1 | 140427065 | T | C | 56 | Kcnt2 | intronic | . | . | . | 1qF | . | 0 | 1 |
| chr1 | 142125135 | A | G | 228 | . | intergenic | . | . | . | 1qF | rs51106674 | 1 | 0 |
| chr1 | 142125179 | C | G | 228 | . | intergenic | . | . | . | 1qF | rs46274115 | 1 | 0 |
| chr1 | 144222434 | T | C | 44.4754 | . | intergenic | . | . | . | 1qF | . | 0 | 1 |
| chr1 | 146514647 | T | A | 85 | Brinp3 | intronic | . | . | . | 1qF | . | 0 | 1 |
| chr1 | 150658490 | A | G | 222 | Hmcn1 | exonic | missense SNV | Hmcn1:NM_001024720:exon58:c.T9032C:p.L3011P | . | 1qG1 | . | 0 | 1 |
| chr1 | 154204730 | G | A | 82 | Zfp648 | exonic | missense SNV | Zfp648:NM_001204908:exon2:c.G634A:p.D212N | . | 1qG3 | rs248204791 | 0 | 1 |
| chr1 | 154398770 | T | C | 177 | Cacna1e | exonic | missense SNV | Cacna1e:NM_009782:exon47:c.A6445G:p.S2149G | . | 1qG3 | . | 0 | 1 |
| chr1 | 154407991 | A | G | 60 | Cacna1e | intronic | . | . | . | 1qG3 | . | 0 | 1 |
| chr1 | 154471676 | T | G | 177 | Cacna1e | intronic | . | . | . | 1qG3 | . | 0 | 1 |
| chr1 | 154477670 | A | G | 96 | Cacna1e | intronic | . | . | . | 1qG3 | . | 0 | 1 |
| chr1 | 155193030 | G | A | 36.4685 | Stx6 | intronic | . | . | . | 1qG3 | . | 0 | 1 |
| chr1 | 155803715 | A | T | 144 | Qsox1 | exonic | synonymous SNV | Qsox1:NM_001024945:exon2:c.T357A:p.A119A,Qsox1:NM_023268:exon2:c.T357A:p.A119A | . | 1qG3 | . | 0 | 1 |
| chr1 | 156285285 | T | G | 56 | Tdrd5 | intronic | . | . | . | 1qG3 | . | 0 | 1 |
| chr1 | 156323628 | C | G | 39.897 | Nphs2 | intronic | . | . | . | 1qG3 | . | 0 | 1 |
| chr1 | 156674246 | C | T | 218 | Tor3a | exonic | synonymous SNV | Tor3a:NM_023141:exon1:c.G60A:p.Q20Q | 361 | 1qG3 | . | 0 | 1 |
| chr1 | 157156891 | T | A | 104 | Rasal2 | intronic | . | . | . | 1qG3 | . | 0 | 1 |
| chr1 | 157622183 | T | C | 149 | . | intergenic | . | . | . | 1qH1 | . | 0 | 1 |
| chr1 | 158572777 | G | T | 50 | Astn1 | intronic | . | . | . | 1qH1 | . | 0 | 1 |
| chr1 | 158871548 | C | A | 222 | Pappa2 | splicing | . | . | . | 1qH1 | . | 0 | 1 |
| chr1 | 158936392 | C | T | 222 | Pappa2 | exonic | synonymous SNV | Pappa2:NM_001085376:exon2:c.G1548A:p.K516K | . | 1qH1 | . | 0 | 1 |
| chr1 | 159863946 | C | T | 20.3138 | Tnr | intronic | . | . | . | 1qH1 | . | 0 | 1 |
| chr1 | 160195212 | A | G | 70 | . | upstream | . | . | 449 | 1qH2.1 | . | 0 | 1 |
| chr1 | 161053908 | C | A | 53 | Dars2 | intronic | . | . | . | 1qH2.1 | . | 0 | 1 |
| chr1 | 162012120 | G | A | 56 | Dnm3,2810442N19Ri | ncRNA_intronic | . | . | . | 1qH2.1 | . | 0 | 1 |
| chr1 | 162223427 | C | T | 105 | Mir214,Dnm3os,Dnm | ncRNA_exonic | . | . | . | 1qH2.1 | . | 0 | 1 |
| chr1 | 162537239 | C | A | 221 | Mettl13 | exonic | missense SNV | Mettl13:NM_144877:exon6:c.G1570T:p.V524F | . | 1qH2.1 | . | 0 | 1 |
| chr1 | 164062073 | G | T | 166 | . | upstream | . | . | . | 1qH2.2 | . | 0 | 1 |
| chr1 | 164179269 | G | C | 125 | F5 | intronic | . | . | . | 1qH2.2 | . | 0 | 1 |
| chr1 | 164193394 | A | G | 101 | F5 | exonic | missense SNV | F5:NM_007976:exon13:c.A3437G:p.D1146G | . | 1qH2.2 | . | 0 | 1 |
| chr1 | 164194354 | A | C | 73 | F5 | exonic | missense SNV | F5:NM_007976:exon13:c.A4397C:p.K1466T | . | 1qH2.2 | . | 0 | 1 |
| chr1 | 164796705 | C | A | 40.9789 | . | upstream | . | . | . | 1qH2.2 | . | 0 | 1 |
| chr1 | 165153302 | T | G | 109 | Tbx19 | intronic | . | . | . | 1qH2.2 | . | 0 | 1 |
| chr1 | 166226848 | T | A | 154 | Mael | intronic | . | . | . | 1qH2.3 | . | 0 | 1 |
| chr1 | 166235689 | T | G | 121 | Mael | intronic | . | . | . | 1qH2.3 | . | 0 | 1 |
| chr1 | 169956013 | T | G | 222 | Hsd17b7 | exonic | missense SNV | Hsd17b7:NM_010476:exon8:c.A825C:p.K275N | . | 1qH3 | . | 0 | 1 |
| chr1 | 170161201 | T | C | 155 | Uap1 | intronic | . | . | . | 1qH3 | rs3705254 | 1 | 0 |
| chr1 | 170647975 | G | A | 176 | Olfml2b | intronic | . | . | . | 1qH3 | . | 0 | 1 |
| chr1 | 170908705 | C | T | 152 | Fcrlb | exonic | synonymous SNV | Fcrlb:NM_001029984:exon4:c.G501A:p.Q167Q | 258 | 1qH3 | . | 0 | 1 |
| chr1 | 170963519 | G | A | 199 | Fcgr2b | intronic | . | . | . | 1qH3 | . | 0 | 1 |
| chr1 | 170986056 | G | T | 100 | . | intergenic | . | . | . | 1qH3 | . | 0 | 1 |
| chr1 | 171344579 | A | C | 221 | Nit1 | intronic | . | . | . | 1qH3 | rs231787201 | 0 | 1 |
| chr1 | 171345404 | T | A | 139 | Nit1 | intronic | . | . | . | 1qH3 | . | 0 | 1 |
| chr1 | 171357963 | A | G | 222 | Pfdn2 | exonic | synonymous SNV | Pfdn2:NM_011070:exon4:c.A402G:p.A134A | . | 1qH3 | rs31549998 | 0 | 1 |
| chr1 | 171711399 | C | A | 228 | . | intergenic | . | . | . | 1qH3 | rs584342724 | 1 | 0 |
| chr1 | 171885450 | A | C | 159 | Cd84 | intronic | . | . | . | 1qH3 | . | 0 | 1 |
| chr1 | 172009866 | A | C | 86 | Vangl2 | exonic | synonymous SNV | Vangl2:NM_033509:exon4:c.T375G:p.A125A | . | 1qH3 | . | 0 | 1 |
| chr1 | 172279002 | C | T | 221 | Atp1a2 | intronic | . | . | . | 1qH3 | . | 0 | 1 |
| chr1 | 172287240 | A | C | 221 | Atp1a2 | exonic | missense SNV | Atp1a2:NM_178405:exon8:c.T977G:p.I326S | . | 1qH3 | . | 0 | 1 |
| chr1 | 172602364 | A | T | 146 | Fcrl6 | intronic | . | . | . | 1qH3 | . | 0 | 1 |
| chr1 | 172893976 | T | G | 58 | Apcs | UTR3 | . | . | . | 1qH3 | . | 0 | 1 |
| chr1 | 174041979 | C | A | 221 | Olfr433 | exonic | synonymous SNV | Olfr433:NM_146717:exon1:c.C28A:p.R10R | . | 1qH3 | . | 0 | 1 |
| chr1 | 174050677 | G | C | 90 | Olfr432 | exonic | synonymous SNV | Olfr432:NM_146716:exon1:c.G303C:p.L101L | . | 1qH3 | . | 0 | 1 |
| chr1 | 174137529 | T | C | 118 | Olfr424 | exonic | missense SNV | Olfr424:NM_146721:exon1:c.T784C:p.S262P | . | 1qH3 | . | 0 | 1 |
| chr1 | 174158568 | C | T | 53 | . | upstream | . | . | . | 1qH3 | . | 0 | 1 |
| chr1 | 174231428 | C | G | 199 | Spta1 | intronic | . | . | . | 1qH3 | . | 0 | 1 |
| chr1 | 174245688 | A | T | 124 | Spta1 | exonic | missense SNV | Spta1:NM_011465:exon50:c.A6872T:p.E2291V | . | 1qH3 | . | 0 | 1 |
| chr1 | 174249852 | C | A | 42.113 | . | downstream | . | . | . | 1qH3 | . | 0 | 1 |
| chr1 | 174368977 | T | C | 96 | Olfr417 | exonic | missense SNV | Olfr417:NM_207137:exon1:c.T59C:p.L20P | . | 1qH3 | . | 0 | 1 |
| chr1 | 174391177 | C | A | 103 | Olfr248 | exonic | missense SNV | Olfr248:NM_146714:exon1:c.C107A:p.A36D | . | 1qH3 | . | 0 | 1 |
| chr1 | 174502388 | C | A | 60 | Fmn2 | exonic | missense SNV | Fmn2:NM_019445:exon1:c.C343A:p.P115T | 811 | 1qH3 | . | 0 | 1 |
| chr1 | 174537197 | T | A | 49.65 | Fmn2 | exonic | missense SNV | Fmn2:NM_019445:exon2:c.T1699A:p.S567T | . | 1qH3 | . | 0 | 1 |
| chr1 | 174694599 | T | G | 55 | Fmn2 | intronic | . | . | . | 1qH3 | . | 0 | 1 |
| chr1 | 175079276 | T | A | 44.0407 | Rgs7 | intronic | . | . | . | 1qH3 | . | 0 | 1 |
| chr1 | 175086218 | C | A | 114 | Rgs7 | exonic | missense SNV | Rgs7:NM_001199003:exon13:c.G854T:p.S285I,Rgs7:NM_011880:exon13:c.G854T:p.S285I | . | 1qH3 | . | 0 | 1 |
| chr1 | 175755700 | C | A | 123 | Wdr64 | intronic | . | . | . | 1qH3 | . | 0 | 1 |
| chr1 | 175785392 | A | C | 40.9256 | Wdr64 | intronic | . | . | . | 1qH3 | . | 0 | 1 |
| chr1 | 175785654 | A | G | 86 | Wdr64 | intronic | . | . | . | 1qH3 | . | 0 | 1 |
| chr1 | 175808698 | T | C | 222 | Wdr64 | exonic | missense SNV | Wdr64:NM_029453:exon25:c.T2911C:p.S971P | . | 1qH3 | . | 0 | 1 |
| chr1 | 175815318 | T | C | 99 | Wdr64 | exonic | synonymous SNV | Wdr64:NM_029453:exon28:c.T3207C:p.V1069V | . | 1qH3 | rs31487965 | 0 | 1 |
| chr1 | 176780446 | A | G | 23.4859 | Cep170 | intronic | . | . | . | 1qH4 | . | 0 | 1 |
| chr1 | 176919396 | T | A | 112 | Sdccag8 | intronic | . | . | . | 1qH4 | . | 0 | 1 |
| chr1 | 177449706 | G | T | 107 | Zbtb18 | UTR3 | . | . | . | 1qH4 | . | 0 | 1 |
| chr1 | 179786070 | C | A | 39.5973 | Ahctf1 | exonic | missense SNV | Ahctf1:NM_026375:exon11:c.G1494T:p.E498D | . | 1qH4 | . | 0 | 1 |
| chr1 | 180952111 | T | G | 193 | Tmem63a | intronic | . | . | . | 1qH4 | . | 0 | 1 |
| chr1 | 181741317 | G | T | 130 | . | intergenic | . | . | . | 1qH5 | . | 0 | 1 |
| chr1 | 181752169 | G | C | 97 | . | intergenic | . | . | . | 1qH5 | . | 0 | 1 |
| chr1 | 182607732 | G | A | 171 | Capn8 | exonic | missense SNV | Capn8:NM_130890:exon10:c.G1274A:p.G425D | . | 1qH5 | . | 0 | 1 |
| chr1 | 183206081 | G | A | 71 | Disp1 | intronic | . | . | . | 1qH5 | . | 0 | 1 |
| chr1 | 184803294 | G | A | 50 | 1-3月 | intronic | . | . | . | 1qH5 | . | 0 | 1 |
| chr1 | 184810928 | G | T | 69 | 1-3月 | exonic | missense SNV | Marc1:NM_001290273:exon1:c.C202A:p.P68T | 404 | 1qH5 | . | 0 | 1 |
| chr1 | 185456360 | C | T | 193 | Slc30a10 | exonic | synonymous SNV | Slc30a10:NM_001033286:exon2:c.C625T:p.L209L | . | 1qH5 | . | 0 | 1 |
| chr1 | 186970118 | A | C | 202 | D1Pas1 | UTR3 | . | . | . | 1qH5 | . | 0 | 1 |
| chr1 | 188066531 | A | T | 41.3895 | Esrrg | intronic | . | . | . | 1qH5 | . | 0 | 1 |
| chr1 | 188198595 | G | T | 222 | Esrrg | exonic | synonymous SNV | Esrrg:NM_011935:exon6:c.G882T:p.L294L,Esrrg:NM_001243792:exon7:c.G813T:p.L271L | . | 1qH6 | rs37971829 | 0 | 1 |
| chr1 | 188198928 | A | G | 192 | Esrrg | intronic | . | . | . | 1qH6 | rs32220759 | 0 | 1 |
| chr1 | 188318782 | C | T | 70 | Ush2a | intronic | . | . | . | 1qH6 | rs32340460 | 0 | 1 |
| chr1 | 188318888 | C | T | 141 | Ush2a | intronic | . | . | . | 1qH6 | rs32177842 | 0 | 1 |
| chr1 | 188318933 | T | C | 168 | Ush2a | intronic | . | . | . | 1qH6 | rs32490585 | 0 | 1 |
| chr1 | 188318969 | G | T | 188 | Ush2a | exonic | missense SNV | Ush2a:NM_021408:exon3:c.G651T:p.Q217H | . | 1qH6 | rs31751588 | 0 | 1 |
| chr1 | 188319014 | T | G | 221 | Ush2a | exonic | synonymous SNV | Ush2a:NM_021408:exon3:c.T696G:p.T232T | . | 1qH6 | rs31011081 | 0 | 1 |
| chr1 | 188350879 | T | C | 120 | Ush2a | intronic | . | . | . | 1qH6 | rs31810126 | 0 | 1 |
| chr1 | 188357715 | T | C | 56 | Ush2a | intronic | . | . | . | 1qH6 | rs50868625 | 0 | 1 |
| chr1 | 188357929 | G | A | 220 | Ush2a | exonic | missense SNV | Ush2a:NM_021408:exon6:c.G1208A:p.S403N | . | 1qH6 | rs49010670 | 0 | 1 |
| chr1 | 188358012 | A | G | 221 | Ush2a | exonic | missense SNV | Ush2a:NM_021408:exon6:c.A1291G:p.N431D | . | 1qH6 | rs48976549 | 0 | 1 |
| chr1 | 188358091 | C | A | 211 | Ush2a | intronic | . | . | . | 1qH6 | rs48592559 | 0 | 1 |
| chr1 | 188358116 | T | C | 124 | Ush2a | intronic | . | . | . | 1qH6 | rs46617909 | 0 | 1 |
| chr1 | 188358290 | T | C | 20.3719 | Ush2a | intronic | . | . | . | 1qH6 | rs50959078 | 0 | 1 |
| chr1 | 188358361 | G | A | 70 | Ush2a | intronic | . | . | . | 1qH6 | rs51929449 | 0 | 1 |
| chr1 | 188358415 | G | A | 121 | Ush2a | intronic | . | . | . | 1qH6 | rs49948425 | 0 | 1 |
| chr1 | 188358482 | C | T | 222 | Ush2a | intronic | . | . | . | 1qH6 | rs49729365 | 0 | 1 |
| chr1 | 188395637 | C | T | 102 | Ush2a | intronic | . | . | . | 1qH6 | rs32085414 | 0 | 1 |
| chr1 | 188415660 | C | T | 161 | Ush2a | intronic | . | . | . | 1qH6 | rs32326313 | 0 | 1 |
| chr1 | 188415865 | A | C | 222 | Ush2a | splicing | . | . | . | 1qH6 | rs30612870 | 0 | 1 |
| chr1 | 188430755 | T | C | 221 | Ush2a | exonic | synonymous SNV | Ush2a:NM_021408:exon14:c.T3033C:p.C1011C | . | 1qH6 | rs48081307 | 0 | 1 |
| chr1 | 188430814 | C | T | 190 | Ush2a | exonic | missense SNV | Ush2a:NM_021408:exon14:c.C3092T:p.T1031I | . | 1qH6 | rs46431791 | 0 | 1 |
| chr1 | 188430842 | C | T | 29.8635 | Ush2a | exonic | synonymous SNV | Ush2a:NM_021408:exon14:c.C3120T:p.D1040D | . | 1qH6 | rs47442600 | 0 | 1 |
| chr1 | 188436736 | A | G | 62 | Ush2a | intronic | . | . | . | 1qH6 | rs3724371 | 0 | 1 |
| chr1 | 188436809 | C | T | 114 | Ush2a | splicing | . | . | . | 1qH6 | rs3724963 | 0 | 1 |
| chr1 | 188437037 | A | G | 222 | Ush2a | intronic | . | . | . | 1qH6 | rs3726187 | 0 | 1 |
| chr1 | 188437165 | C | A | 94 | Ush2a | intronic | . | . | . | 1qH6 | rs32619345 | 0 | 1 |
| chr1 | 188437185 | C | T | 53 | Ush2a | intronic | . | . | . | 1qH6 | rs30789092 | 0 | 1 |
| chr1 | 188444282 | C | T | 32.1085 | Ush2a | intronic | . | . | . | 1qH6 | rs31534253 | 0 | 1 |
| chr1 | 188444560 | G | A | 221 | Ush2a | exonic | missense SNV | Ush2a:NM_021408:exon17:c.G3920A:p.S1307N | . | 1qH6 | rs13476294 | 0 | 1 |
| chr1 | 188451620 | C | T | 63 | Ush2a | intronic | . | . | . | 1qH6 | rs32329623 | 0 | 1 |
| chr1 | 188466190 | C | T | 221 | Ush2a | exonic | missense SNV | Ush2a:NM_021408:exon20:c.C4450T:p.P1484S | . | 1qH6 | rs50646786 | 0 | 1 |
| chr1 | 188466470 | T | C | 129 | Ush2a | intronic | . | . | . | 1qH6 | rs50049266 | 0 | 1 |
| chr1 | 188626591 | G | A | 92 | Ush2a | exonic | missense SNV | Ush2a:NM_021408:exon32:c.G6313A:p.A2105T | . | 1qH6 | rs48853167 | 0 | 1 |
| chr1 | 188626602 | G | A | 75 | Ush2a | exonic | synonymous SNV | Ush2a:NM_021408:exon32:c.G6324A:p.E2108E | . | 1qH6 | rs51292947 | 0 | 1 |
| chr1 | 188626705 | A | G | 221 | Ush2a | exonic | missense SNV | Ush2a:NM_021408:exon32:c.A6427G:p.T2143A | . | 1qH6 | rs45922548 | 0 | 1 |
| chr1 | 188628447 | C | T | 109 | Ush2a | intronic | . | . | . | 1qH6 | rs213600295 | 0 | 1 |
| chr1 | 188628454 | C | A | 160 | Ush2a | intronic | . | . | . | 1qH6 | rs52625821 | 0 | 1 |
| chr1 | 188628608 | T | C | 222 | Ush2a | exonic | missense SNV | Ush2a:NM_021408:exon33:c.T6524C:p.I2175T | . | 1qH6 | rs50347269 | 0 | 1 |
| chr1 | 188628654 | A | C | 221 | Ush2a | exonic | missense SNV | Ush2a:NM_021408:exon33:c.A6570C:p.K2190N | . | 1qH6 | rs46308824 | 0 | 1 |
| chr1 | 188628696 | A | G | 201 | Ush2a | exonic | synonymous SNV | Ush2a:NM_021408:exon33:c.A6612G:p.L2204L | . | 1qH6 | rs49894839 | 0 | 1 |
| chr1 | 188628750 | T | C | 177 | Ush2a | intronic | . | . | . | 1qH6 | rs50228515 | 0 | 1 |
| chr1 | 188628813 | C | T | 101 | Ush2a | intronic | . | . | . | 1qH6 | rs46972551 | 0 | 1 |
| chr1 | 188628841 | A | G | 44.738 | Ush2a | intronic | . | . | . | 1qH6 | rs51939797 | 0 | 1 |
| chr1 | 188633515 | T | C | 123 | Ush2a | intronic | . | . | . | 1qH6 | rs51706767 | 0 | 1 |
| chr1 | 188633536 | G | A | 135 | Ush2a | intronic | . | . | . | 1qH6 | rs48108451 | 0 | 1 |
| chr1 | 188633634 | G | A | 222 | Ush2a | exonic | synonymous SNV | Ush2a:NM_021408:exon34:c.G6684A:p.E2228E | . | 1qH6 | rs45738922 | 0 | 1 |
| chr1 | 188633717 | A | G | 222 | Ush2a | exonic | missense SNV | Ush2a:NM_021408:exon34:c.A6767G:p.E2256G | . | 1qH6 | rs50987399 | 0 | 1 |
| chr1 | 188648099 | C | T | 77 | Ush2a | intronic | . | . | . | 1qH6 | rs32576017 | 0 | 1 |
| chr1 | 188648166 | G | A | 103 | Ush2a | intronic | . | . | . | 1qH6 | rs242511300 | 0 | 1 |
| chr1 | 188648326 | A | G | 221 | Ush2a | exonic | synonymous SNV | Ush2a:NM_021408:exon35:c.A6831G:p.S2277S | . | 1qH6 | rs51608803 | 0 | 1 |

| | | | | | | | | | | | | | |
|---|---|---|---|---|---|---|---|---|---|---|---|---|---|
| chr1 | 188648565 | G | A | 109 | Ush2a | intronic | . | . | . | 1qH6 | rs31281856 | 0 | 1 |
| chr1 | 188728652 | T | C | 221 | Ush2a | exonic | synonymous SNV | Ush2a:NM_021408:exon40:c.T8109C:p.S2703S | . | 1qH6 | rs45817901 | 0 | 1 |
| chr1 | 188728723 | C | G | 221 | Ush2a | exonic | missense SNV | Ush2a:NM_021408:exon40:c.C8180G:p.A2727G | . | 1qH6 | rs36325126 | 0 | 1 |
| chr1 | 188728737 | G | A | 220 | Ush2a | exonic | missense SNV | Ush2a:NM_021408:exon40:c.G8194A:p.E2732K | . | 1qH6 | rs36501082 | 0 | 1 |
| chr1 | 188728838 | C | T | 90 | Ush2a | intronic | . | . | . | 1qH6 | rs49445209 | 0 | 1 |
| chr1 | 188728851 | C | G | 77 | Ush2a | intronic | . | . | . | 1qH6 | rs38073864 | 0 | 1 |
| chr1 | 188733240 | C | A | 92 | Ush2a | intronic | . | . | . | 1qH6 | rs32510643 | 0 | 1 |
| chr1 | 188733798 | T | A | 222 | Ush2a | intronic | . | . | . | 1qH6 | rs37557812 | 0 | 1 |
| chr1 | 188734688 | T | C | 222 | Ush2a | exonic | missense SNV | Ush2a:NM_021408:exon42:c.T8548C:p.C2850R | . | 1qH6 | rs30983300 | 0 | 1 |
| chr1 | 188734877 | G | A | 222 | Ush2a | intronic | . | . | . | 1qH6 | rs38047130 | 0 | 1 |
| chr1 | 188734903 | G | C | 208 | Ush2a | intronic | . | . | . | 1qH6 | rs38044577 | 0 | 1 |
| chr1 | 188734987 | A | G | 50 | Ush2a | intronic | . | . | . | 1qH6 | rs37129378 | 0 | 1 |
| chr1 | 188850064 | G | C | 222 | Ush2a | exonic | missense SNV | Ush2a:NM_021408:exon57:c.G11323C:p.V3775L | . | 1qH6 | rs38266727 | 0 | 1 |
| chr1 | 188864525 | C | T | 121 | Ush2a | intronic | . | . | . | 1qH6 | rs49615355 | 0 | 1 |
| chr1 | 188864592 | G | A | 134 | Ush2a | exonic | missense SNV | Ush2a:NM_021408:exon59:c.G11528A:p.G3843D | . | 1qH6 | rs240847189 | 0 | 1 |
| chr1 | 188864618 | G | A | 150 | Ush2a | exonic | missense SNV | Ush2a:NM_021408:exon59:c.G11554A:p.V3852I | . | 1qH6 | rs38898903 | 0 | 1 |
| chr1 | 188864766 | T | A | 222 | Ush2a | intronic | . | . | . | 1qH6 | rs38320062 | 0 | 1 |
| chr1 | 188864897 | G | A | 120 | Ush2a | intronic | . | . | . | 1qH6 | rs49040039 | 0 | 1 |
| chr1 | 188906822 | A | T | 46.1159 | Ush2a | intronic | . | . | . | 1qH6 | rs31796348 | 0 | 1 |
| chr1 | 188910656 | G | T | 65 | Ush2a | intronic | . | . | . | 1qH6 | rs31501606 | 0 | 1 |
| chr1 | 188910675 | G | A | 56 | Ush2a | intronic | . | . | . | 1qH6 | rs31855523 | 0 | 1 |
| chr1 | 188911284 | G | A | 221 | Ush2a | exonic | missense SNV | Ush2a:NM_021408:exon62:c.G12842A:p.R4281Q | . | 1qH6 | rs241829660 | 0 | 1 |
| chr1 | 188911297 | T | C | 219 | Ush2a | exonic | synonymous SNV | Ush2a:NM_021408:exon62:c.T12855C:p.G4285G | . | 1qH6 | rs32448008 | 0 | 1 |
| chr1 | 188911479 | T | C | 222 | Ush2a | exonic | missense SNV | Ush2a:NM_021408:exon62:c.T13037C:p.V4346A | . | 1qH6 | rs13476295 | 0 | 1 |
| chr1 | 188911520 | A | G | 186 | Ush2a | exonic | missense SNV | Ush2a:NM_021408:exon62:c.A13078G:p.S4360G | . | 1qH6 | rs31995267 | 0 | 1 |
| chr1 | 188911726 | C | T | 143 | Ush2a | exonic | synonymous SNV | Ush2a:NM_021408:exon62:c.C13284T:p.S4428S | . | 1qH6 | rs31076100 | 0 | 1 |
| chr1 | 188912068 | T | A | 222 | Ush2a | exonic | synonymous SNV | Ush2a:NM_021408:exon62:c.T13626A:p.A4542A | . | 1qH6 | rs32079742 | 0 | 1 |
| chr1 | 188912180 | T | A | 222 | Ush2a | exonic | missense SNV | Ush2a:NM_021408:exon62:c.T13738A:p.S4580T | . | 1qH6 | rs31191866 | 0 | 1 |
| chr1 | 188915883 | T | C | 46.8248 | Ush2a | intronic | . | . | . | 1qH6 | rs36283001 | 0 | 1 |
| chr1 | 188915913 | A | T | 97 | Ush2a | intronic | . | . | . | 1qH6 | rs219715707 | 0 | 1 |
| chr1 | 188915963 | A | G | 180 | Ush2a | intronic | . | . | . | 1qH6 | rs36484305 | 0 | 1 |
| chr1 | 188916373 | T | C | 151 | Ush2a | intronic | . | . | . | 1qH6 | rs36515819 | 0 | 1 |
| chr1 | 188916387 | A | G | 133 | Ush2a | intronic | . | . | . | 1qH6 | rs36313915 | 0 | 1 |
| chr1 | 188944860 | A | C | 222 | Ush2a | intronic | . | . | . | 1qH6 | rs37858928 | 0 | 1 |
| chr1 | 188947125 | C | T | 168 | Ush2a | exonic | synonymous SNV | Ush2a:NM_021408:exon65:c.C14529T:p.P4843P | . | 1qH6 | rs229747116 | 0 | 1 |
| chr1 | 188962785 | C | T | 222 | Ush2a | exonic | missense SNV | Ush2a:NM_021408:exon70:c.C15338T:p.S5113F | . | 1qH6 | rs50354992 | 0 | 1 |
| chr1 | 188997116 | G | A | 222 | Kctd3 | intronic | . | . | . | 1qH6 | rs238189436 | 0 | 1 |
| chr1 | 189340202 | C | T | 158 | Kcnk2 | intronic | . | . | . | 1qH6 | . | 0 | 1 |
| chr1 | 189344196 | A | G | 204 | Kcnk2 | intronic | . | . | . | 1qH6 | . | 0 | 1 |
| chr1 | 189657015 | T | A | 158 | Cenpf | exonic | missense SNV | Cenpf:NM_001081363:exon12:c.A4619T:p.K1540M | . | 1qH6 | . | 0 | 1 |
| chr1 | 189850725 | G | A | 159 | Ptpn14 | exonic | missense SNV | Ptpn14:NM_008976:exon13:c.G1768A:p.V590I | . | 1qH6 | . | 0 | 1 |
| chr1 | 190161446 | T | G | 222 | Prox1 | exonic | missense SNV | Prox1:NM_008937:exon2:c.A801C:p.E267D | . | 1qH6 | . | 0 | 1 |
| chr1 | 191054807 | T | A | 222 | Tatdn3 | intronic | . | . | . | 1qH6 | . | 0 | 1 |
| chr1 | 192980590 | A | C | 217 | Syt14 | intronic | . | . | . | 1qH6 | . | 0 | 1 |
| chr1 | 193324007 | T | A | 77 | Lamb3 | intronic | . | . | . | 1qH6 | . | 0 | 1 |
| chr1 | 193334271 | A | C | 113 | Lamb3 | exonic | missense SNV | Lamb3:NM_001277928:exon15:c.A2059C:p.S687R,Lamb3:NM_008484:exon15:c.A2059C:p.S687R | . | 1qH6 | . | 0 | 1 |
| chr1 | 194712464 | G | C | 140 | Plxna2 | intronic | . | . | . | 1qH6 | . | 0 | 1 |
| chr1 | 195083748 | T | A | 110 | Cd46 | intronic | . | . | . | 1qH6 | . | 0 | 1 |
| chr1 | 195092155 | C | G | 175 | Cd46 | exonic | missense SNV | Cd46:NM_010778:exon1:c.G71C:p.W24S | . | 1qH6 | . | 0 | 1 |
| chr1 | 195151944 | T | A | 43.9904 | Cr2 | intronic | . | . | . | 1qH6 | . | 0 | 1 |
| chr1 | 195166209 | A | C | 228 | Cr2 | intronic | . | . | . | 1qH6 | rs107929253 | 1 | 0 |
| chr10 | 4103245 | G | T | 222 | Mthfd1l | exonic | missense SNV | Mthfd1l:NM_001170785:exon25:c.G2593T:p.V865L,Mthfd1l:NM_001170786:exon25:c.G2593T:p.V865L,Mtl | . | 10qA1 | . | 0 | 1 |
| chr10 | 4966022 | T | A | 121 | Esr1 | intronic | . | . | . | 10qA1 | . | 0 | 1 |
| chr10 | 4998104 | G | T | 121 | Esr1 | intronic | . | . | . | 10qA1 | . | 0 | 1 |
| chr10 | 5123090 | T | A | 222 | Syne1 | exonic | missense SNV | Syne1:NM_001079686:exon24:c.A3734T:p.N1245I | . | 10qA1 | . | 0 | 1 |
| chr10 | 5161513 | C | G | 118 | Syne1 | exonic | missense SNV | Syne1:NM_001079686:exon9:c.G1420C:p.A474P | . | 10qA1 | . | 0 | 1 |
| chr10 | 5215222 | T | G | 110 | . | intergenic | . | . | . | 10qA1 | . | 0 | 1 |
| chr10 | 5215312 | C | G | 221 | . | intergenic | . | . | . | 10qA1 | . | 0 | 1 |
| chr10 | 5227932 | C | A | 205 | . | intergenic | . | . | . | 10qA1 | . | 0 | 1 |
| chr10 | 5360763 | A | T | 222 | Syne1 | exonic | missense SNV | Syne1:NM_153399:exon17:c.T1813A:p.W605R | . | 10qA1 | . | 0 | 1 |
| chr10 | 5425673 | A | C | 221 | Syne1 | exonic | missense SNV | Syne1:NM_153399:exon7:c.T418G:p.F140V | . | 10qA1 | . | 0 | 1 |
| chr10 | 6794700 | A | C | 93 | Oprm1 | intronic | . | . | . | 10qA1 | . | 0 | 1 |
| chr10 | 6823772 | A | C | 194 | Oprm1 | intronic | . | . | . | 10qA1 | . | 0 | 1 |
| chr10 | 7602833 | C | A | 221 | Lrp11 | exonic | stopgain | Lrp11:NM_172784:exon4:c.C870A:p.C290X | . | 10qA1 | . | 0 | 1 |
| chr10 | 7644454 | T | G | 221 | Pcmt1 | intronic | . | . | . | 10qA1 | . | 0 | 1 |
| chr10 | 7862481 | C | G | 221 | Zc3h12d | exonic | missense SNV | Zc3h12d:NM_172785:exon4:c.C550G:p.R184G | . | 10qA1 | . | 0 | 1 |
| chr10 | 8518331 | C | A | 97 | Ust | exonic | synonymous SNV | Ust:NM_177387:exon1:c.G69T:p.G23G | 881 | 10qA1 | . | 0 | 1 |
| chr10 | 8850538 | C | A | 221 | Sash1 | intronic | . | . | . | 10qA1 | . | 0 | 1 |
| chr10 | 12413956 | G | T | 221 | Utrn | exonic | missense SNV | Utrn:NM_011682:exon68:c.C9639A:p.S3213R | . | 10qA1 | . | 0 | 1 |
| chr10 | 12663425 | C | A | 221 | Utrn | exonic | missense SNV | Utrn:NM_011682:exon40:c.G5847T:p.M1949I | . | 10qA1 | . | 0 | 1 |
| chr10 | 13292243 | C | T | 183 | Phactr2 | intronic | . | . | . | 10qA2 | . | 0 | 1 |
| chr10 | 13512585 | C | G | 219 | Fuca2 | intronic | . | . | . | 10qA2 | . | 0 | 1 |
| chr10 | 14408099 | C | A | 79 | Adgrg6 | intronic | . | . | . | 10qA2 | . | 0 | 1 |
| chr10 | 14745942 | T | C | 72 | 1700016L04Rik | ncRNA_intronic | . | . | . | 10qA2 | . | 0 | 1 |
| chr10 | 17843197 | T | A | 126 | Txlnb | exonic | missense SNV | Txlnb:NM_138628:exon10:c.T1775A:p.L592Q | . | 10qA3 | . | 0 | 1 |
| chr10 | 18163420 | C | G | 221 | Ect2l | exonic | missense SNV | Ect2l:NM_001195036:exon10:c.G1265C:p.G422A | . | 10qA3 | . | 0 | 1 |
| chr10 | 18336331 | C | T | 222 | . | intergenic | . | . | . | 10qA3 | . | 0 | 1 |
| chr10 | 18661136 | A | G | 47.7724 | Arfgef3 | intronic | . | . | . | 10qA3 | . | 0 | 1 |
| chr10 | 18853789 | T | G | 134 | Perp | intronic | . | . | . | 10qA3 | . | 0 | 1 |
| chr10 | 19626453 | A | C | 95 | Il22ra2 | intronic | . | . | . | 10qA3 | . | 0 | 1 |
| chr10 | 20108395 | A | T | 131 | Map3k5 | intronic | . | . | . | 10qA3 | . | 0 | 1 |
| chr10 | 20266640 | A | C | 183 | Map7 | intronic | . | . | . | 10qA3 | rs49802724 | 0 | 1 |
| chr10 | 20273505 | A | T | 137 | Map7 | intronic | . | . | . | 10qA3 | . | 0 | 1 |
| chr10 | 20273801 | G | T | 199 | Map7 | exonic | missense SNV | Map7:NM_001198635:exon13:c.G1697T:p.R566L,Map7:NM_008635:exon13:c.G1673T:p.R558L | . | 10qA3 | . | 0 | 1 |
| chr10 | 21382408 | G | A | 159 | Aldh8a1 | exonic | missense SNV | Aldh8a1:NM_178713:exon3:c.G403A:p.G135S | . | 10qA3 | . | 0 | 1 |
| chr10 | 22344506 | T | C | 222 | Raet1b,Raet1c,Rae | intronic | . | . | . | 10qA3 | . | 0 | 1 |
| chr10 | 23138109 | A | T | 148 | Eya4 | intronic | . | . | . | 10qA3 | . | 0 | 1 |
| chr10 | 23824837 | A | G | 197 | Slc18b1 | intronic | . | . | . | 10qA4 | . | 0 | 1 |
| chr10 | 23864234 | C | A | 132 | Vnn3 | intronic | . | . | . | 10qA4 | . | 0 | 1 |
| chr10 | 23900878 | G | T | 222 | Vnn1 | exonic | missense SNV | Vnn1:NM_011704:exon5:c.G1126T:p.D376Y | . | 10qA4 | . | 0 | 1 |
| chr10 | 23921147 | A | G | 221 | Taar1 | exonic | missense SNV | Taar1:NM_053205:exon1:c.A742G:p.K248E | . | 10qA4 | . | 0 | 1 |
| chr10 | 24597676 | T | G | 221 | Ctgf | UTR3 | . | . | . | 10qA4 | . | 0 | 1 |
| chr10 | 24647914 | T | C | 107 | Enpp1 | intronic | . | . | . | 10qA4 | . | 0 | 1 |
| chr10 | 24660371 | G | T | 222 | Enpp1 | intronic | . | . | . | 10qA4 | . | 0 | 1 |
| chr10 | 24665122 | A | G | 64 | Enpp1 | intronic | . | . | . | 10qA4 | . | 0 | 1 |
| chr10 | 24874806 | C | A | 127 | Med23 | intronic | . | . | . | 10qA4 | . | 0 | 1 |
| chr10 | 25478860 | G | A | 66 | Epb41l2 | intronic | . | . | . | 10qA4 | . | 0 | 1 |
| chr10 | 25489173 | C | A | 138 | Epb41l2 | intronic | . | . | . | 10qA4 | . | 0 | 1 |
| chr10 | 25499701 | C | G | 79 | Epb41l2 | intronic | . | . | . | 10qA4 | rs257410748 | 0 | 1 |
| chr10 | 25837162 | C | T | 221 | 4930401C15Rik | ncRNA_intronic | . | . | . | 10qA4 | . | 0 | 1 |
| chr10 | 26984501 | C | A | 222 | Lama2 | exonic | missense SNV | Lama2:NM_008481:exon63:c.G8989T:p.V2997L | . | 10qA4 | . | 0 | 1 |
| chr10 | 27041067 | C | A | 194 | Lama2 | intronic | . | . | . | 10qA4 | . | 0 | 1 |
| chr10 | 27165186 | A | C | 85 | Lama2 | intronic | . | . | . | 10qA4 | . | 0 | 1 |
| chr10 | 27212014 | C | G | 228 | Lama2 | intronic | . | . | . | 10qA4 | rs51751664 | 1 | 0 |
| chr10 | 27231523 | C | A | 205 | Lama2 | intronic | . | . | . | 10qA4 | . | 0 | 1 |
| chr10 | 27422787 | G | A | 201 | Lama2 | intronic | . | . | . | 10qA4 | . | 0 | 1 |
| chr10 | 28383648 | C | T | 222 | Ptprk | exonic | synonymous SNV | Ptprk:NM_008983:exon7:c.C1083T:p.R361R | . | 10qA4 | . | 0 | 1 |
| chr10 | 28923430 | C | G | 110 | 4930519F09Rik | ncRNA_exonic | . | . | . | 10qA4 | . | 0 | 1 |
| chr10 | 29699278 | A | C | 220 | . | intergenic | . | . | 207 | 10qA4 | . | 0 | 1 |
| chr10 | 29781570 | C | A | 129 | . | intergenic | . | . | . | 10qA4 | . | 0 | 1 |
| chr10 | 30547517 | A | C | 111 | Trmt11 | intronic | . | . | . | 10qA4 | . | 0 | 1 |
| chr10 | 31250521 | A | C | 222 | . | intergenic | . | . | . | 10qA4 | . | 0 | 1 |
| chr10 | 31341053 | A | G | 222 | Tpd52l1 | intronic | . | . | . | 10qA4 | . | 0 | 1 |
| chr10 | 31746354 | A | G | 59 | Nkain2 | intronic | . | . | . | 10qA4 | . | 0 | 1 |
| chr10 | 33778372 | A | T | 155 | Gm4794 | intronic | . | . | . | 10qB1 | . | 0 | 1 |
| chr10 | 33879189 | T | A | 222 | Sult3a1 | intronic | . | . | . | 10qB1 | . | 0 | 1 |
| chr10 | 33905741 | G | C | 221 | Rsph4a | exonic | missense SNV | Rsph4a:NM_001162957:exon1:c.G586C:p.V196L | . | 10qB1 | . | 0 | 1 |
| chr10 | 34297707 | A | T | 220 | Tspyl4 | exonic | missense SNV | Tspyl4:NM_030203:exon1:c.A194T:p.E65V | 302 | 10qB1 | . | 0 | 1 |
| chr10 | 34484373 | A | G | 221 | Frk | splicing | . | . | . | 10qB1 | . | 0 | 1 |
| chr10 | 38966072 | G | A | 212 | Lama4 | exonic | missense SNV | Lama4:NM_010681:exon1:c.G118A:p.A40T | . | 10qB1 | . | 0 | 1 |
| chr10 | 39149688 | G | T | 174 | Tube1 | UTR3 | . | . | . | 10qB1 | . | 0 | 1 |
| chr10 | 39158615 | G | T | 35.2594 | Wisp3 | intronic | . | . | . | 10qB1 | . | 0 | 1 |
| chr10 | 39512037 | G | T | 221 | Fyn | intronic | . | . | . | 10qB1 | . | 0 | 1 |
| chr10 | 40133570 | T | C | 210 | Slc16a10 | intronic | . | . | . | 10qB1 | . | 0 | 1 |
| chr10 | 40349226 | A | T | 221 | . | upstream | . | . | 1120 | 10qB1 | . | 0 | 1 |
| chr10 | 42415744 | G | T | 146 | Lace1 | intronic | . | . | . | 10qB2 | rs243207200 | 1 | 0 |
| chr10 | 42698349 | C | T | 146 | Ostm1 | intronic | . | . | . | 10qB2 | . | 0 | 1 |
| chr10 | 43021268 | C | A | 111 | Sobp | exonic | missense SNV | Sobp:NM_175407:exon6:c.G2320T:p.D774Y | 1034 | 10qB2 | . | 0 | 1 |
| chr10 | 43372198 | G | A | 221 | Pdss2 | exonic | missense SNV | Pdss2:NM_001168289:exon4:c.G697A:p.A233T,Pdss2:NM_027772:exon4:c.G697A:p.A233T | . | 10qB2 | . | 0 | 1 |
| chr10 | 43524338 | G | A | 197 | . | downstream | . | . | . | 10qB2 | . | 0 | 1 |
| chr10 | 43822043 | G | A | 221 | . | intergenic | . | . | . | 10qB2 | . | 0 | 1 |
| chr10 | 43921428 | C | T | 221 | Rtn4ip1 | exonic | missense SNV | Rtn4ip1:NM_130892:exon5:c.C662T:p.A221V | . | 10qB2 | . | 0 | 1 |
| chr10 | 43997311 | C | T | 228 | Aim1 | exonic | missense SNV | Aim1:NM_172393:exon2:c.G2678A:p.R893H | . | 10qB2 | rs29356879 | 1 | 0 |
| chr10 | 44170092 | G | C | 222 | . | upstream | . | . | . | 10qB2 | . | 0 | 1 |
| chr10 | 44446836 | C | A | 175 | Prdm1 | exonic | missense SNV | Prdm1:NM_007548:exon4:c.G617T:p.R206M | . | 10qB2 | . | 0 | 1 |
| chr10 | 45006543 | A | G | 221 | . | intergenic | . | . | . | 10qB2 | . | 0 | 1 |
| chr10 | 45346042 | T | G | 111 | Bves | intronic | . | . | . | 10qB2 | . | 0 | 1 |
| chr10 | 49404449 | A | G | 222 | Grik2 | exonic | missense SNV | Grik2:NM_001111268:exon10:c.T1205C:p.I402T,Grik2:NM_010349:exon10:c.T1205C:p.I402T | . | 10qB3 | . | 0 | 1 |
| chr10 | 49404533 | C | A | 111 | Grik2 | intronic | . | . | . | 10qB3 | . | 0 | 1 |
| chr10 | 50645857 | A | C | 222 | Ascc3 | intronic | . | . | . | 10qB3 | . | 0 | 1 |
| chr10 | 50849594 | C | T | 221 | Ascc3 | exonic | stopgain | Ascc3:NM_198007:exon41:c.C6412T:p.R2138X | . | 10qB3 | . | 0 | 1 |
| chr10 | 50984512 | G | T | 100 | Sim1 | UTR3 | . | . | . | 10qB3 | . | 0 | 1 |
| chr10 | 51757117 | A | C | 222 | 4933411G06Rik | ncRNA_exonic | . | . | . | 10qB3 | . | 0 | 1 |
| chr10 | 52022834 | G | C | 81 | Vgll2 | intronic | . | . | 594 | 10qB3 | . | 0 | 1 |
| chr10 | 52077881 | T | C | 222 | Ros1 | exonic | synonymous SNV | Ros1:NM_011282:exon36:c.A5724G:p.G1908G | . | 10qB3 | . | 0 | 1 |
| chr10 | 52144712 | C | A | 200 | Ros1 | intronic | . | . | . | 10qB3 | . | 0 | 1 |
| chr10 | 52161956 | C | T | 222 | Ros1 | intronic | . | . | . | 10qB3 | . | 0 | 1 |
| chr10 | 52391878 | T | G | 29.9819 | Nepn | intronic | . | . | . | 10qB3 | . | 0 | 1 |
| chr10 | 52403853 | G | C | 144 | Nepn | intronic | . | . | . | 10qB3 | . | 0 | 1 |
| chr10 | 53934989 | C | G | 222 | Man1a | exonic | missense SNV | Man1a:NM_008548:exon7:c.G1022C:p.W341S | . | 10qB3 | . | 0 | 1 |
| chr10 | 56497474 | G | A | 208 | . | intergenic | . | . | . | 10qB4 | . | 0 | 1 |
| chr10 | 56497516 | G | A | 165 | . | intergenic | . | . | . | 10qB4 | . | 0 | 1 |
| chr10 | 56497619 | T | C | 222 | . | intergenic | . | . | . | 10qB4 | rs108382269 | 0 | 1 |
| chr10 | 56497622 | C | T | 222 | . | intergenic | . | . | . | 10qB4 | rs108109108 | 0 | 1 |
| chr10 | 56497639 | C | T | 222 | . | intergenic | . | . | . | 10qB4 | . | 0 | 1 |
| chr10 | 56497734 | C | T | 221 | . | intergenic | . | . | . | 10qB4 | . | 0 | 1 |
| chr10 | 56497773 | T | G | 100 | . | intergenic | . | . | . | 10qB4 | . | 0 | 1 |
| chr10 | 56497777 | A | G | 95 | . | intergenic | . | . | . | 10qB4 | rs52629402 | 0 | 1 |
| chr10 | 56656572 | A | T | 222 | . | intergenic | . | . | . | 10qB4 | . | 0 | 1 |
| chr10 | 57736412 | C | A | 25.123 | Pkib | UTR3 | . | . | . | 10qB4 | . | 0 | 1 |
| chr10 | 57807277 | T | G | 128 | Smpdl3a | intronic | . | . | . | 10qB4 | . | 0 | 1 |
| chr10 | 58467390 | T | C | 221 | Ranbp2 | exonic | missense SNV | Ranbp2:NM_011240:exon17:c.T2383C:p.Y795H | . | 10qB4 | . | 0 | 1 |
| chr10 | 58513035 | C | G | 116 | Ccdc138 | intronic | . | . | . | 10qB4 | . | 0 | 1 |
| chr10 | 59427965 | T | C | 80 | Oit3 | exonic | missense SNV | Oit3:NM_010959:exon7:c.A1346G:p.K449R | . | 10qB4 | . | 0 | 1 |
| chr10 | 60294595 | C | T | 99 | Psap | exonic | synonymous SNV | Psap:NM_001146120:exon5:c.C438T:p.A146A,Psap:NM_001146121:exon5:c.C438T:p.A146A,Psap:NM_0011 | . | 10qB4 | . | 0 | 1 |
| chr10 | 60305385 | A | G | 120 | Cdh23 | intronic | . | . | . | 10qB4 | . | 0 | 1 |
| chr10 | 60305432 | T | C | 181 | Cdh23 | intronic | . | . | . | 10qB4 | rs50422567 | 1 | 0 |
| chr10 | 60312166 | T | A | 53 | Cdh23 | intronic | . | . | . | 10qB4 | . | 0 | 1 |
| chr10 | 60317805 | C | G | 111 | Cdh23 | exonic | synonymous SNV | Cdh23:NM_001252635:exon49:c.G6999C:p.L2333L | . | 10qB4 | . | 0 | 1 |
| chr10 | 60376870 | C | A | 222 | Cdh23 | exonic | synonymous SNV | Cdh23:NM_001252635:exon37:c.G4758T:p.R1586R | . | 10qB4 | . | 0 | 1 |
| chr10 | 61249747 | C | T | 65 | Adamts14 | exonic | missense SNV | Adamts14:NM_001081127:exon3:c.G497A:p.R166H | . | 10qB4 | . | 0 | 1 |
| chr10 | 61338995 | G | T | 70 | Pald1 | intronic | . | . | . | 10qB4 | . | 0 | 1 |
| chr10 | 62290475 | G | A | 99 | Hk1 | intronic | . | . | . | 10qB4 | . | 0 | 1 |
| chr10 | 62598342 | C | G | 222 | Ddx21 | exonic | missense SNV | Ddx21:NM_019553:exon2:c.G637C:p.D213H | . | 10qB4 | . | 0 | 1 |
| chr10 | 62780358 | C | A | 130 | Ccar1 | intronic | . | . | . | 10qB4 | . | 0 | 1 |
| chr10 | 62838267 | T | G | 221 | Tet1 | exonic | missense SNV | Tet1:NM_001253857:exon3:c.A4029C:p.E1343D,Tet1:NM_027384:exon3:c.A4029C:p.E1343D | . | 10qB4 | . | 0 | 1 |
| chr10 | 63053878 | C | A | 141 | Pbld2 | exonic | missense SNV | Pbld2:NM_026085:exon7:c.C438A:p.D146E | . | 10qB4 | . | 0 | 1 |
| chr10 | 63118417 | T | G | 221 | Mypn | splicing | . | . | . | 10qB4 | . | 0 | 1 |
| chr10 | 63131073 | G | A | 221 | Mypn | exonic | synonymous SNV | Mypn:NM_182992:exon13:c.C2823T:p.P941P | . | 10qB4 | . | 0 | 1 |
| chr10 | 63820542 | G | A | 221 | Ctnna3 | intronic | . | . | . | 10qB4 | . | 0 | 1 |
| chr10 | 64873355 | G | A | 186 | Ctnna3 | intronic | . | . | . | 10qB5.1 | . | 0 | 1 |
| chr10 | 67021877 | G | C | 210 | Gm31763,Reep3 | exonic | missense SNV | Reep3:NM_001204915:exon6:c.C434G:p.T145R,Reep3:NM_178606:exon6:c.C434G:p.T145R | . | 10qB5.1 | . | 0 | 1 |
| chr10 | 67219470 | C | T | 222 | Jmjd1c | exonic | missense SNV | Jmjd1c:NM_001242396:exon7:c.C986T:p.T329M,Jmjd1c:NM_207221:exon8:c.C1529T:p.T510M | . | 10qB5.1 | . | 0 | 1 |
| chr10 | 67232195 | G | T | 221 | Jmjd1c | intronic | . | . | . | 10qB5.1 | . | 0 | 1 |
| chr10 | 67238174 | T | C | 228 | Jmjd1c | exonic | missense SNV | Jmjd1c:NM_001242396:exon13:c.T5144C:p.L1715P,Jmjd1c:NM_207221:exon14:c.T5684C:p.L1895P | . | 10qB5.1 | rs13480628 | 1 | 0 |

| | | | | | | | | | | | | | |
|---|---|---|---|---|---|---|---|---|---|---|---|---|---|
| chr10 | 68533675 | T | C | 20.4605 | 1700040L02Rik | intronic | . | . | . | 10qB5.2 | . | 0 | 1 |
| chr10 | 69345883 | T | A | 123 | Cdk1 | intronic | . | . | . | 10qB5.3 | . | 0 | 1 |
| chr10 | 70927801 | A | G | 228 | Bicc1 | intronic | . | . | . | 10qB5.3 | rs49419429 | 1 | 0 |
| chr10 | 70940612 | C | A | 218 | Bicc1 | exonic | missense SNV | Bicc1:NM_031397:exon17:c.G2256T:p.E752D | . | 10qB5.3 | . | 0 | 1 |
| chr10 | 70950222 | C | T | 169 | Bicc1 | intronic | . | . | . | 10qB5.3 | . | 0 | 1 |
| chr10 | 71285046 | C | A | 51 | Ube2d1 | intronic | . | . | 861 | 10qB5.3 | . | 0 | 1 |
| chr10 | 71319161 | C | A | 104 | . | intergenic | . | . | . | 10qB5.3 | . | 0 | 1 |
| chr10 | 73947034 | C | A | 172 | Pcdh15 | UTR5 | . | . | . | 10qB5.3 | . | 0 | 1 |
| chr10 | 74317208 | T | A | 203 | Pcdh15 | intronic | . | . | . | 10qB5.3 | . | 0 | 1 |
| chr10 | 74466149 | T | A | 126 | Pcdh15 | intronic | . | . | . | 10qB5.3 | . | 0 | 1 |
| chr10 | 74466392 | C | T | 221 | Pcdh15 | exonic | synonymous SNV | Pcdh15:NM_001142738:exon20:c.C2751T:p.D917D,Pcdh15:NM_001142736:exon21:c.C2862T:p.D954D,Pcdh1 | . | 10qB5.3 | . | 0 | 1 |
| chr10 | 74550335 | T | G | 94 | Pcdh15 | intronic | . | . | . | 10qB5.3 | . | 0 | 1 |
| chr10 | 75352111 | A | G | 162 | Gm5779 | ncRNA_exonic | . | . | . | 10qC1 | . | 0 | 1 |
| chr10 | 75430232 | G | T | 37.4796 | Upb1 | intronic | . | . | . | 10qC1 | . | 0 | 1 |
| chr10 | 75643831 | G | A | 222 | Susd2 | intronic | . | . | . | 10qC1 | . | 0 | 1 |
| chr10 | 75656607 | A | C | 35.4799 | Cabin1 | intronic | . | . | . | 10qC1 | rs225447384 | 0 | 1 |
| chr10 | 75715886 | A | C | 110 | Cabin1 | intronic | . | . | . | 10qC1 | . | 0 | 1 |
| chr10 | 75775084 | C | A | 216 | Gstt3 | intronic | . | . | . | 10qC1 | . | 0 | 1 |
| chr10 | 75937468 | T | C | 228 | Chchd10 | intronic | . | . | . | 10qC1 | rs50869969 | 1 | 0 |
| chr10 | 75940214 | A | C | 112 | Gm867 | intronic | . | . | . | 10qC1 | . | 0 | 1 |
| chr10 | 76147647 | C | T | 221 | Slc5a4a | exonic | synonymous SNV | Slc5a4a:NM_133184:exon1:c.C117T:p.V39V | . | 10qC1 | . | 0 | 1 |
| chr10 | 76427799 | A | G | 192 | Pcnt | intronic | . | . | . | 10qC1 | . | 0 | 1 |
| chr10 | 76507155 | G | A | 107 | Mcm3ap | intronic | . | . | . | 10qC1 | . | 0 | 1 |
| chr10 | 76716963 | C | A | 153 | Col6a1 | intronic | . | . | . | 10qC1 | . | 0 | 1 |
| chr10 | 76789668 | C | A | 222 | Pcbp3 | intronic | . | . | . | 10qC1 | . | 0 | 1 |
| chr10 | 77059925 | G | T | 228 | Col18a1 | exonic | synonymous SNV | Col18a1:NM_001109991:exon33:c.C3393A:p.P1131P,Col18a1:NM_009929:exon34:c.C2757A:p.P919P | . | 10qC1 | rs8244810 | 1 | 0 |
| chr10 | 77265784 | G | A | 174 | Pofut2 | intronic | . | . | 218 | 10qC1 | . | 0 | 1 |
| chr10 | 77836105 | G | C | 221 | Krtap10-10,Tspear | exonic | missense SNV | Krtap10-10:NM_001024709:exon1:c.G107C:p.S36T | . | 10qC1 | . | 0 | 1 |
| chr10 | 77853237 | T | A | 221 | Tspear,Gm10318 | exonic | missense SNV | Gm10318:NM_001162944:exon1:c.T376A:p.S126T | . | 10qC1 | . | 0 | 1 |
| chr10 | 77900883 | G | A | 197 | Lrrc3 | exonic | synonymous SNV | Lrrc3:NM_145152:exon2:c.C718T:p.L240L | . | 10qC1 | . | 0 | 1 |
| chr10 | 77901552 | G | T | 187 | Lrrc3 | exonic | missense SNV | Lrrc3:NM_145152:exon2:c.C49A:p.Q17K | . | 10qC1 | . | 0 | 1 |
| chr10 | 77929938 | G | T | 183 | Trpm2 | exonic | missense SNV | Trpm2:NM_138301:exon20:c.C2672A:p.P891H | . | 10qC1 | . | 0 | 1 |
| chr10 | 78076986 | T | C | 220 | Icosl | intronic | . | . | . | 10qC1 | rs29348417 | 1 | 0 |
| chr10 | 79182122 | A | T | 190 | Vmn2r80 | intronic | . | . | . | 10qC1 | . | 0 | 1 |
| chr10 | 79477933 | T | A | 221 | Vmn2r83 | exonic | missense SNV | Vmn2r83:NM_001104537:exon2:c.T374A:p.L125H | . | 10qC1 | . | 0 | 1 |
| chr10 | 79579794 | A | G | 39.457 | Theg | intronic | . | . | . | 10qC1 | . | 0 | 1 |
| chr10 | 79640323 | G | A | 221 | Odf3l2 | exonic | missense SNV | Odf3l2:NM_001033473:exon4:c.C406T:p.R136C | . | 10qC1 | . | 0 | 1 |
| chr10 | 79726007 | A | G | 53 | Hcn2,Bc1 | ncRNA_intronic | . | . | . | 10qC1 | . | 0 | 1 |
| chr10 | 79967729 | G | T | 129 | Bc1,Wdr18 | ncRNA_intronic | . | . | . | 10qC1 | . | 0 | 1 |
| chr10 | 80136964 | G | A | 104 | Bc1,Cbarp | ncRNA_intronic | . | . | . | 10qC1 | . | 0 | 1 |
| chr10 | 80341706 | G | T | 178 | Bc1,Adamtsl5 | ncRNA_intronic | . | . | 1851 | 10qC1 | . | 0 | 1 |
| chr10 | 80412919 | T | A | 173 | Tcf3 | exonic | missense SNV | Tcf3:NM_001164147:exon18:c.A1727T:p.Q576L,Tcf3:NM_001164148:exon18:c.A1724T:p.Q575L,Tcf3:NM_( | . | 10qC1 | . | 0 | 1 |
| chr10 | 80524519 | C | T | 100 | Atp8b3 | intronic | . | . | . | 10qC1 | . | 0 | 1 |
| chr10 | 80536256 | C | T | 221 | Atp8b3 | exonic | synonymous SNV | Atp8b3:NM_026094:exon3:c.G138A:p.K46K | . | 10qC1 | . | 0 | 1 |
| chr10 | 80667240 | T | C | 141 | Mknk2 | exonic | missense SNV | Mknk2:NM_021462:exon14:c.A1247G:p.D416G | . | 10qC1 | . | 0 | 1 |
| chr10 | 80669632 | C | G | 113 | Mknk2 | exonic | missense SNV | Mknk2:NM_021462:exon7:c.G491C:p.G164A | . | 10qC1 | . | 0 | 1 |
| chr10 | 80689958 | C | G | 222 | Mob3a | exonic | missense SNV | Mob3a:NM_172457:exon3:c.G518C:p.R173P | . | 10qC1 | . | 0 | 1 |
| chr10 | 80721228 | C | A | 188 | Ap3d1 | intronic | . | . | . | 10qC1 | . | 0 | 1 |
| chr10 | 80795365 | A | T | 228 | . | downstream | . | . | . | 10qC1 | rs13459122 | 1 | 0 |
| chr10 | 80907505 | C | T | 92 | Lmnb2 | exonic | synonymous SNV | Lmnb2:NM_010722:exon3:c.G372A:p.Q124Q | . | 10qC1 | . | 0 | 1 |
| chr10 | 81178702 | C | T | 108 | Eef2 | exonic | missense SNV | Eef2:NM_007907:exon4:c.C422T:p.T141I | . | 10qC1 | . | 0 | 1 |
| chr10 | 81178727 | A | G | 162 | Eef2 | exonic | synonymous SNV | Eef2:NM_007907:exon4:c.A447G:p.E149E | . | 10qC1 | rs47730192 | 0 | 1 |
| chr10 | 81178790 | G | A | 131 | Eef2 | exonic | synonymous SNV | Eef2:NM_007907:exon4:c.G510A:p.E170E | . | 10qC1 | . | 0 | 1 |
| chr10 | 81178829 | G | A | 36.4987 | Eef2 | exonic | synonymous SNV | Eef2:NM_007907:exon4:c.G549A:p.E183E | . | 10qC1 | . | 0 | 1 |
| chr10 | 81182816 | G | T | 111 | . | upstream;downstrea | . | . | . | 10qC1 | . | 0 | 1 |
| chr10 | 81183083 | G | A | 221 | Dapk3 | UTR5 | . | . | 309 | 10qC1 | . | 0 | 1 |
| chr10 | 81211901 | C | A | 167 | Atcay | exonic | missense SNV | Atcay:NM_178662:exon8:c.G811T:p.V271F | . | 10qC1 | . | 0 | 1 |
| chr10 | 81246164 | G | A | 154 | Zfr2 | splicing | . | . | . | 10qC1 | . | 0 | 1 |
| chr10 | 81279883 | C | G | 83 | Tjp3 | intronic | . | . | . | 10qC1 | . | 0 | 1 |
| chr10 | 81322726 | A | G | 107 | Cactin | exonic | synonymous SNV | Cactin:NM_027381:exon2:c.A525G:p.Q175Q | 238 | 10qC1 | . | 0 | 1 |
| chr10 | 81467415 | G | T | 221 | Celf5 | intronic | . | . | . | 10qC1 | . | 0 | 1 |
| chr10 | 82431107 | C | A | 221 | . | intergenic | . | . | . | 10qC1 | . | 0 | 1 |
| chr10 | 82664719 | C | T | 216 | Glt8d2 | exonic | missense SNV | Glt8d2:NM_029102:exon4:c.G196A:p.A66T | . | 10qC1 | . | 0 | 1 |
| chr10 | 84309082 | A | T | 222 | . | intergenic | . | . | . | 10qC1 | . | 0 | 1 |
| chr10 | 84696921 | G | A | 221 | Polr3b | intronic | . | . | . | 10qC1 | . | 0 | 1 |
| chr10 | 85388406 | G | A | 86 | Btbd11 | exonic | missense SNV | Btbd11:NM_028709:exon1:c.G1078A:p.V360I | 201 | 10qC1 | . | 0 | 1 |
| chr10 | 85632035 | A | T | 143 | Btbd11 | intronic | . | . | . | 10qC1 | . | 0 | 1 |
| chr10 | 85900994 | T | G | 180 | Prdm4 | exonic | missense SNV | Prdm4:NM_181650:exon9:c.A1781C:p.K594T,Prdm4:NM_001302886:exon10:c.A1760C:p.K587T,Prdm4:NM_0( | . | 10qC1 | . | 0 | 1 |
| chr10 | 85975561 | A | G | 129 | Bpifc | intronic | . | . | . | 10qC1 | . | 0 | 1 |
| chr10 | 86000532 | C | G | 175 | Bpifc | exonic | missense SNV | Bpifc:NM_177772:exon2:c.G108C:p.Q36H | . | 10qC1 | . | 0 | 1 |
| chr10 | 86732380 | G | A | 222 | Ttc41 | intronic | . | . | . | 10qC1 | . | 0 | 1 |
| chr10 | 86969867 | G | T | 221 | Stab2 | exonic | missense SNV | Stab2:NM_138673:exon7:c.C614A:p.P205H | . | 10qC1 | . | 0 | 1 |
| chr10 | 86981074 | C | A | 219 | Stab2 | intronic | . | . | . | 10qC1 | . | 0 | 1 |
| chr10 | 87913391 | T | A | 45.8132 | Igf1 | intronic | . | . | . | 10qC1 | . | 0 | 1 |
| chr10 | 87913392 | C | G | 45.8132 | Igf1 | intronic | . | . | . | 10qC1 | . | 0 | 1 |
| chr10 | 87930781 | T | C | 93 | Igf1 | UTR3 | . | . | . | 10qC1 | . | 0 | 1 |
| chr10 | 88529468 | G | A | 170 | Mybpc1 | exonic | missense SNV | Mybpc1:NM_001252372:exon23:c.C2792T:p.A931V,Mybpc1:NM_175418:exon24:c.C2834T:p.A945V | . | 10qC1 | . | 0 | 1 |
| chr10 | 88585654 | A | C | 220 | Mybpc1 | intronic | . | . | . | 10qC1 | . | 0 | 1 |
| chr10 | 88742209 | C | T | 34.1388 | Arl1 | intronic | . | . | . | 10qC1 | . | 0 | 1 |
| chr10 | 88787476 | G | A | 221 | Utp20 | exonic | missense SNV | Utp20:NM_175158:exon26:c.C3190T:p.P1064S | . | 10qC1 | . | 0 | 1 |
| chr10 | 88791161 | C | A | 83 | Utp20 | exonic | missense SNV | Utp20:NM_175158:exon25:c.G2934T:p.R978S | . | 10qC1 | . | 0 | 1 |
| chr10 | 88971464 | G | A | 221 | Ano4 | intronic | . | . | . | 10qC1 | . | 0 | 1 |
| chr10 | 88972743 | G | A | 103 | Ano4 | exonic | synonymous SNV | Ano4:NM_001277188:exon23:c.C2298T:p.D766D | . | 10qC1 | rs263485885 | 0 | 1 |
| chr10 | 89025167 | C | A | 222 | Ano4 | intronic | . | . | . | 10qC1 | . | 0 | 1 |
| chr10 | 89034903 | A | C | 37.7165 | Ano4 | intronic | . | . | . | 10qC1 | . | 0 | 1 |
| chr10 | 89112937 | G | A | 160 | Ano4 | exonic | stopgain | Ano4:NM_001277188:exon5:c.C310T:p.R104X | . | 10qC1 | . | 0 | 1 |
| chr10 | 89421706 | C | A | 195 | Gas2l3 | intronic | . | . | . | 10qC2 | . | 0 | 1 |
| chr10 | 89811761 | T | G | 37.1791 | Uhrf1bp1l | intronic | . | . | . | 10qC2 | . | 0 | 1 |
| chr10 | 90134768 | C | A | 221 | Anks1b | intronic | . | . | . | 10qC2 | . | 0 | 1 |
| chr10 | 90511121 | C | T | 59 | Anks1b | exonic | unknown | UNKNOWN | . | 10qC2 | . | 0 | 1 |
| chr10 | 90868067 | C | A | 172 | Anks1b | intronic | . | . | . | 10qC2 | . | 0 | 1 |
| chr10 | 90966500 | C | T | 164 | Anks1b | intronic | . | . | . | 10qC2 | . | 0 | 1 |
| chr10 | 91001041 | A | T | 122 | Apaf1 | exonic | stopgain | Apaf1:NM_001042558:exon23:c.T3173A:p.L1058X,Apaf1:NM_001282947:exon23:c.T3140A:p.L1047X,Apaf1 | . | 10qC2 | . | 0 | 1 |
| chr10 | 91096606 | G | A | 221 | Ikbip | exonic | missense SNV | Ikbip:NM_026166:exon3:c.G1111A:p.D371N | . | 10qC2 | . | 0 | 1 |
| chr10 | 92223078 | G | A | 150 | Gm20757 | ncRNA_intronic | . | . | . | 10qC2 | . | 0 | 1 |
| chr10 | 92940305 | A | T | 188 | . | intergenic | . | . | . | 10qC2 | . | 0 | 1 |
| chr10 | 93489433 | G | A | 221 | Hal | intronic | . | . | . | 10qC2 | . | 0 | 1 |
| chr10 | 93499000 | C | T | 221 | Hal | exonic | missense SNV | Hal:NM_010401:exon13:c.C1192T:p.R398C | . | 10qC2 | . | 0 | 1 |
| chr10 | 93707131 | A | G | 42.5022 | Ntn4 | intronic | . | . | . | 10qC2 | . | 0 | 1 |
| chr10 | 94003997 | T | G | 221 | Vezt | exonic | missense SNV | Vezt:NM_001304572:exon3:c.A257C:p.K86T,Vezt:NM_001304575:exon3:c.A257C:p.K86T,Vezt:NM_0013047 | . | 10qC2 | . | 0 | 1 |
| chr10 | 94171215 | C | T | 222 | Nr2c1 | exonic | missense SNV | Nr2c1:NM_011629:exon8:c.C914T:p.T305I | . | 10qC2 | . | 0 | 1 |
| chr10 | 94843660 | G | T | 221 | Plxnc1 | intronic | . | . | . | 10qC2 | . | 0 | 1 |
| chr10 | 94849700 | C | G | 133 | Plxnc1 | intronic | . | . | . | 10qC2 | . | 0 | 1 |
| chr10 | 96026220 | C | T | 174 | Eea1 | intronic | . | . | . | 10qC2 | . | 0 | 1 |
| chr10 | 97693733 | G | A | 176 | Ccer1,Gm10754 | exonic | missense SNV | Ccer1:NM_025724:exon1:c.G257A:p.R86H | . | 10qC3 | . | 0 | 1 |
| chr10 | 99124786 | C | T | 134 | Poc1b | intronic | . | . | . | 10qC3 | . | 0 | 1 |
| chr10 | 99445644 | G | A | 164 | . | intergenic | . | . | . | 10qD1 | . | 0 | 1 |
| chr10 | 100549448 | A | C | 146 | Cep290 | intronic | . | . | . | 10qD1 | . | 0 | 1 |
| chr10 | 100610780 | T | C | 221 | 1700017N19Rik | intronic | . | . | . | 10qD1 | . | 0 | 1 |
| chr10 | 103170541 | G | A | 138 | Lrriq1 | exonic | missense SNV | Lrriq1:NM_001163559:exon16:c.C3722T:p.A1241V | . | 10qD1 | . | 0 | 1 |
| chr10 | 103190038 | A | C | 221 | Lrriq1 | intronic | . | . | . | 10qD1 | . | 0 | 1 |
| chr10 | 103214460 | C | A | 221 | Lrriq1 | exonic | missense SNV | Lrriq1:NM_001163559:exon8:c.G2430T:p.Q810H,Lrriq1:NM_029134:exon8:c.G2430T:p.Q810H | . | 10qD1 | . | 0 | 1 |
| chr10 | 103400236 | A | G | 222 | Slc6a15 | exonic | missense SNV | Slc6a15:NM_001252330:exon6:c.A811G:p.R271G,Slc6a15:NM_175328:exon7:c.A811G:p.R271G | . | 10qD1 | . | 0 | 1 |
| chr10 | 103404823 | A | G | 208 | Slc6a15 | splicing | . | . | . | 10qD1 | . | 0 | 1 |
| chr10 | 103408410 | T | C | 221 | Slc6a15 | intronic | . | . | . | 10qD1 | . | 0 | 1 |
| chr10 | 103418111 | T | C | 222 | Slc6a15 | exonic | missense SNV | Slc6a15:NM_001252330:exon12:c.T1907C:p.V636A,Slc6a15:NM_175328:exon13:c.T1907C:p.V636A | . | 10qD1 | . | 0 | 1 |
| chr10 | 104874371 | A | C | 124 | . | intergenic | . | . | . | 10qD1 | . | 0 | 1 |
| chr10 | 105763443 | C | G | 222 | Mettl25 | exonic | missense SNV | Mettl25:NM_207522:exon12:c.G1720C:p.A574P | . | 10qD1 | . | 0 | 1 |
| chr10 | 106865307 | A | G | 220 | Ppfia2 | exonic | synonymous SNV | Ppfia2:NM_001205341:exon20:c.A2454G:p.Q818Q,Ppfia2:NM_177373:exon20:c.A2451G:p.Q817Q | . | 10qD1 | . | 0 | 1 |
| chr10 | 106928858 | C | T | 125 | Ppfia2 | intronic | . | . | . | 10qD1 | . | 0 | 1 |
| chr10 | 106966883 | C | A | 33.0908 | Acss3 | intronic | . | . | . | 10qD1 | . | 0 | 1 |
| chr10 | 107577125 | G | A | 40.1176 | Ptprq | intronic | . | . | . | 10qD1 | . | 0 | 1 |
| chr10 | 108409626 | T | C | 166 | Pawr | intronic | . | . | . | 10qD1 | . | 0 | 1 |
| chr10 | 109684392 | C | T | 126 | Nav3 | intronic | . | . | . | 10qD1 | . | 0 | 1 |
| chr10 | 111255513 | G | T | 119 | Osbpl8 | intronic | . | . | . | 10qD1 | . | 0 | 1 |
| chr10 | 111291428 | T | G | 222 | Osbpl8 | intronic | . | . | . | 10qD1 | . | 0 | 1 |
| chr10 | 111983201 | C | G | 79 | Krr1 | intronic | . | . | . | 10qD2 | . | 0 | 1 |
| chr10 | 112092385 | T | A | 148 | Glipr1l2 | intronic | . | . | . | 10qD2 | . | 0 | 1 |
| chr10 | 112458344 | T | C | 106 | Kcnc2 | intronic | . | . | . | 10qD2 | . | 0 | 1 |
| chr10 | 114503268 | C | A | 44.3103 | Trhde | intronic | . | . | . | 10qD2 | . | 0 | 1 |
| chr10 | 115079800 | A | G | 189 | Tph2 | exonic | missense SNV | Tph2:NM_173391:exon11:c.T1334C:p.F445S | . | 10qD2 | . | 0 | 1 |
| chr10 | 115079887 | G | A | 134 | Tph2 | intronic | . | . | . | 10qD2 | rs29378222 | 0 | 1 |
| chr10 | 115174159 | A | C | 81 | Tph2 | intronic | . | . | . | 10qD2 | rs29367091 | 0 | 1 |
| chr10 | 115179201 | C | T | 49.1069 | Tph2 | intronic | . | . | . | 10qD2 | rs51190350 | 0 | 1 |
| chr10 | 115179293 | G | T | 122 | Tph2 | intronic | . | . | . | 10qD2 | rs51506751 | 0 | 1 |
| chr10 | 115179662 | T | C | 124 | Tph2 | exonic | synonymous SNV | Tph2:NM_173391:exon3:c.A294G:p.R98R | . | 10qD2 | rs29316839 | 0 | 1 |
| chr10 | 115179670 | T | G | 118 | Tph2 | exonic | synonymous SNV | Tph2:NM_173391:exon3:c.A286C:p.R96R | . | 10qD2 | rs29378914 | 0 | 1 |
| chr10 | 115182813 | T | A | 54 | Tph2 | exonic | synonymous SNV | Tph2:NM_173391:exon2:c.A192T:p.V64V | . | 10qD2 | rs29342684 | 0 | 1 |
| chr10 | 115182816 | C | T | 56 | Tph2 | exonic | synonymous SNV | Tph2:NM_173391:exon2:c.G189A:p.A63A | . | 10qD2 | rs29325093 | 0 | 1 |
| chr10 | 115202452 | T | C | 42.5516 | Tbc1d15 | intronic | . | . | . | 10qD2 | rs29340214 | 0 | 1 |
| chr10 | 115202551 | T | C | 123 | Tbc1d15 | exonic | missense SNV | Tbc1d15:NM_025706:exon16:c.A1756G:p.I586V | . | 10qD2 | rs29344612 | 0 | 1 |
| chr10 | 115202618 | A | G | 125 | Tbc1d15 | intronic | . | . | . | 10qD2 | rs29368309 | 0 | 1 |
| chr10 | 115202792 | A | G | 37.7788 | Tbc1d15 | intronic | . | . | . | 10qD2 | rs29338800 | 0 | 1 |
| chr10 | 115203095 | A | G | 178 | Tbc1d15 | intronic | . | . | . | 10qD2 | rs29342208 | 0 | 1 |
| chr10 | 115203283 | A | G | 35.8997 | Tbc1d15 | intronic | . | . | . | 10qD2 | rs46029361 | 0 | 1 |
| chr10 | 115209530 | G | A | 86 | Tbc1d15 | intronic | . | . | . | 10qD2 | rs29368621 | 0 | 1 |
| chr10 | 115210262 | T | C | 149 | Tbc1d15 | exonic | synonymous SNV | Tbc1d15:NM_025706:exon12:c.A1335G:p.P445P | . | 10qD2 | rs47962279 | 0 | 1 |
| chr10 | 115212659 | T | C | 197 | Tbc1d15 | intronic | . | . | . | 10qD2 | rs48026843 | 0 | 1 |
| chr10 | 115217468 | C | T | 115 | Tbc1d15 | intronic | . | . | . | 10qD2 | rs29372473 | 0 | 1 |
| chr10 | 115217487 | T | C | 124 | Tbc1d15 | intronic | . | . | . | 10qD2 | rs29381586 | 0 | 1 |
| chr10 | 115219051 | T | C | 129 | Tbc1d15 | intronic | . | . | . | 10qD2 | rs29369199 | 0 | 1 |
| chr10 | 115219320 | A | G | 91 | Tbc1d15 | intronic | . | . | . | 10qD2 | rs29345072 | 0 | 1 |
| chr10 | 115220162 | A | G | 113 | Tbc1d15 | intronic | . | . | . | 10qD2 | rs29339973 | 0 | 1 |
| chr10 | 115220525 | T | C | 25.1722 | Tbc1d15 | intronic | . | . | . | 10qD2 | rs29357511 | 0 | 1 |
| chr10 | 115232996 | G | A | 135 | Tbc1d15 | intronic | . | . | . | 10qD2 | rs29371969 | 0 | 1 |
| chr10 | 115233300 | C | T | 69 | Tbc1d15 | intronic | . | . | . | 10qD2 | rs29355619 | 0 | 1 |
| chr10 | 115239257 | A | G | 59 | Tbc1d15 | intronic | . | . | . | 10qD2 | rs29355851 | 0 | 1 |
| chr10 | 115270334 | C | T | 171 | . | intergenic | . | . | . | 10qD2 | . | 0 | 1 |
| chr10 | 115347633 | T | G | 221 | Tmem19 | intronic | . | . | . | 10qD2 | rs29323134 | 0 | 1 |
| chr10 | 115347868 | T | C | 20.6885 | Tmem19 | intronic | . | . | . | 10qD2 | rs29368100 | 0 | 1 |
| chr10 | 115359723 | C | T | 169 | Tmem19 | exonic | missense SNV | Tmem19:NM_133683:exon3:c.G175A:p.V59I | . | 10qD2 | rs223880217 | 0 | 1 |
| chr10 | 115424692 | A | G | 200 | Zfc3h1 | intronic | . | . | . | 10qD2 | . | 0 | 1 |
| chr10 | 115454349 | C | T | 41.0116 | Lgr5 | intronic | . | . | . | 10qD2 | rs49442246 | 0 | 1 |
| chr10 | 115454550 | T | G | 182 | Lgr5 | intronic | . | . | . | 10qD2 | rs235315928 | 0 | 1 |
| chr10 | 115454599 | T | C | 110 | Lgr5 | intronic | . | . | . | 10qD2 | rs261623993 | 0 | 1 |
| chr10 | 115457849 | A | G | 221 | Lgr5 | exonic | synonymous SNV | Lgr5:NM_010195:exon15:c.T1314C:p.P438P | . | 10qD2 | rs253183571 | 0 | 1 |
| chr10 | 115462749 | T | C | 194 | Lgr5 | exonic | synonymous SNV | Lgr5:NM_010195:exon12:c.A1089G:p.L363L | . | 10qD2 | rs50655825 | 0 | 1 |
| chr10 | 115465945 | C | T | 78 | Lgr5 | intronic | . | . | . | 10qD2 | rs47196559 | 0 | 1 |
| chr10 | 115466254 | C | T | 199 | Lgr5 | intronic | . | . | . | 10qD2 | rs47046621 | 0 | 1 |
| chr10 | 115466368 | C | T | 184 | Lgr5 | exonic | synonymous SNV | Lgr5:NM_010195:exon10:c.G972A:p.L324L | . | 10qD2 | rs47340396 | 0 | 1 |
| chr10 | 115475265 | G | A | 202 | Lgr5 | intronic | . | . | . | 10qD2 | rs46048871 | 0 | 1 |
| chr10 | 115502556 | G | A | 40.1341 | Lgr5 | intronic | . | . | . | 10qD2 | rs36553642 | 0 | 1 |
| chr10 | 115520622 | A | G | 212 | Lgr5 | exonic | missense SNV | Lgr5:NM_010195:exon2:c.T268C:p.C90R | . | 10qD2 | rs36674441 | 0 | 1 |
| chr10 | 115817540 | A | C | 134 | Tspan8 | UTR5 | . | . | . | 10qD2 | rs29349889 | 0 | 1 |
| chr10 | 115832972 | A | C | 64 | Tspan8 | intronic | . | . | . | 10qD2 | rs29345876 | 0 | 1 |
| chr10 | 115832999 | G | A | 64 | Tspan8 | intronic | . | . | . | 10qD2 | rs51257360 | 0 | 1 |
| chr10 | 115833164 | A | C | 149 | Tspan8 | intronic | . | . | . | 10qD2 | rs29349964 | 0 | 1 |
| chr10 | 115833226 | T | C | 144 | Tspan8 | exonic | synonymous SNV | Tspan8:NM_146010:exon3:c.T165C:p.A55A,Tspan8:NM_001168679:exon4:c.T165C:p.A55A,Tspan8:NM_0011 | . | 10qD2 | rs29333148 | 0 | 1 |
| chr10 | 115833268 | A | G | 46.3957 | Tspan8 | exonic | synonymous SNV | Tspan8:NM_146010:exon3:c.A207G:p.L69L,Tspan8:NM_001168679:exon4:c.A207G:p.L69L,Tspan8:NM_0011 | . | 10qD2 | rs29351310 | 0 | 1 |
| chr10 | 115833326 | G | A | 67 | Tspan8 | splicing | . | . | . | 10qD2 | rs29339897 | 0 | 1 |
| chr10 | 115835239 | T | C | 205 | Tspan8 | intronic | . | . | . | 10qD2 | rs29348864 | 0 | 1 |
| chr10 | 115849494 | G | T | 80 | Tspan8 | UTR3 | . | . | . | 10qD2 | rs13473211 | 0 | 1 |
| chr10 | 116226183 | G | A | 37.0008 | Ptprr | exonic | synonymous SNV | Ptprr:NM_001161838:exon3:c.G297A:p.T99T,Ptprr:NM_001161839:exon4:c.G297A:p.T99T,Ptprr:NM_0011 | . | 10qD2 | rs244269083 | 0 | 1 |
| chr10 | 116338533 | T | A | 214 | Ptprb | intronic | . | . | . | 10qD2 | . | 0 | 1 |
| chr10 | 116363370 | G | T | 175 | Ptprb | splicing | . | . | . | 10qD2 | . | 0 | 1 |

| | | | | | | | | | | | | | |
|---|---|---|---|---|---|---|---|---|---|---|---|---|---|
| chr10 | 117220310 | T | C | 129 | Yeats4 | intronic | . | . | . | 10qD2 | . | 0 | 1 |
| chr10 | 117766125 | G | T | 201 | Nup107 | intronic | . | . | . | 10qD2 | rs51456382 | 0 | 1 |
| chr10 | 117773094 | T | C | 221 | Nup107 | exonic | synonymous SNV | Nup107:NM_134010:exon14:c.A1215G:p.R405R | . | 10qD2 | rs46985834 | 0 | 1 |
| chr10 | 117785064 | G | T | 221 | Nup107 | exonic | missense SNV | Nup107:NM_134010:exon5:c.C382A:p.H128N | . | 10qD2 | rs47619206 | 0 | 1 |
| chr10 | 117790308 | T | G | 42.0826 | Nup107 | intronic | . | . | . | 10qD2 | . | 0 | 1 |
| chr10 | 118147943 | A | G | 141 | Mdm1 | intronic | . | . | . | 10qD2 | rs29355115 | 0 | 1 |
| chr10 | 118150984 | G | C | 165 | Mdm1 | intronic | . | . | . | 10qD2 | rs29362461 | 0 | 1 |
| chr10 | 118151100 | T | C | 42.3497 | Mdm1 | intronic | . | . | . | 10qD2 | rs29373113 | 0 | 1 |
| chr10 | 118151910 | T | C | 39.3797 | Mdm1 | intronic | . | . | . | 10qD2 | rs29325884 | 0 | 1 |
| chr10 | 118151912 | G | A | 33.3303 | Mdm1 | intronic | . | . | . | 10qD2 | rs29381576 | 0 | 1 |
| chr10 | 118156555 | T | C | 35.563 | Mdm1 | intronic | . | . | . | 10qD2 | rs52029394 | 0 | 1 |
| chr10 | 118156592 | A | G | 48.2414 | Mdm1 | intronic | . | . | . | 10qD2 | rs51240695 | 0 | 1 |
| chr10 | 118156969 | A | G | 139 | Mdm1 | intronic | . | . | . | 10qD2 | rs51446186 | 0 | 1 |
| chr10 | 118157000 | T | A | 141 | Mdm1 | intronic | . | . | . | 10qD2 | rs50140011 | 0 | 1 |
| chr10 | 118157266 | T | C | 157 | Mdm1 | exonic | synonymous SNV | Mdm1:NM_001162905:exon8:c.T975C:p.H325H,Mdm1:NM_010785:exon8:c.T1080C:p.H360H,Mdm1:NM_148922: | . | 10qD2 | rs29356609 | 0 | 1 |
| chr10 | 118158532 | A | G | 221 | Mdm1 | exonic | missense SNV | Mdm1:NM_001162905:exon9:c.A1310G:p.D437G,Mdm1:NM_010785:exon9:c.A1415G:p.D472G,Mdm1:NM_14892 | . | 10qD2 | rs52684110 | 0 | 1 |
| chr10 | 118159722 | G | A | 221 | Mdm1 | exonic | missense SNV | Mdm1:NM_001162905:exon11:c.G1555A:p.A519T,Mdm1:NM_010785:exon11:c.G1660A:p.A554T,Mdm1:NM_148 | . | 10qD2 | . | 0 | 1 |
| chr10 | 118159815 | C | A | 26.4587 | Mdm1 | intronic | . | . | . | 10qD2 | rs29337221 | 0 | 1 |
| chr10 | 118164170 | G | C | 46.9149 | Mdm1 | intronic | . | . | . | 10qD2 | rs48141337 | 0 | 1 |
| chr10 | 118164183 | G | A | 58 | Mdm1 | intronic | . | . | . | 10qD2 | rs46862685 | 0 | 1 |
| chr10 | 118164284 | C | T | 32.3372 | Mdm1 | exonic | synonymous SNV | Mdm1:NM_001162905:exon12:c.C1686T:p.D562D,Mdm1:NM_010785:exon12:c.C1791T:p.D597D,Mdm1:NM_148 | . | 10qD2 | rs51115308 | 0 | 1 |
| chr10 | 118164457 | T | A | 26.5972 | Mdm1 | intronic | . | . | . | 10qD2 | rs218491695 | 0 | 1 |
| chr10 | 118164495 | T | C | 41.6717 | Mdm1 | intronic | . | . | . | 10qD2 | rs48605734 | 0 | 1 |
| chr10 | 118166635 | C | A | 38.3443 | Mdm1 | exonic | synonymous SNV | Mdm1:NM_001162905:exon13:c.C1860A:p.P620P,Mdm1:NM_148922:exon13:c.C1965A:p.P655P,Mdm1:NM_001 | . | 10qD2 | rs29358548 | 0 | 1 |
| chr10 | 118167898 | C | T | 66 | Mdm1 | intronic | . | . | . | 10qD2 | rs51533063 | 0 | 1 |
| chr10 | 118168006 | T | G | 147 | Mdm1 | intronic | . | . | . | 10qD2 | rs3707488 | 0 | 1 |
| chr10 | 119975890 | G | A | 162 | Grip1 | exonic | missense SNV | Grip1:NM_130891:exon7:c.G664A:p.G222R,Grip1:NM_133442:exon7:c.G667A:p.G223R,Grip1:NM_0012772 | . | 10qD2 | . | 0 | 1 |
| chr10 | 120178598 | A | C | 42.0762 | Irak3 | intronic | . | . | . | 10qD2 | . | 0 | 1 |
| chr10 | 121416432 | C | A | 133 | Rassf3 | intronic | . | . | . | 10qD2 | . | 0 | 1 |
| chr10 | 121476055 | C | G | 156 | Rassf3 | exonic | missense SNV | Rassf3:NM_138956:exon1:c.G56C:p.R19T | 1291 | 10qD2 | . | 0 | 1 |
| chr10 | 123002567 | C | G | 115 | Mon2 | intronic | . | . | . | 10qD2 | . | 0 | 1 |
| chr10 | 123008201 | C | G | 221 | Mon2 | exonic | synonymous SNV | Mon2:NM_001163025:exon29:c.G4425C:p.S1475S,Mon2:NM_153395:exon29:c.G4422C:p.S1474S,Mon2:NM_0( | . | 10qD2 | . | 0 | 1 |
| chr10 | 125231193 | A | G | 161 | Slc16a7 | exonic | synonymous SNV | Slc16a7:NM_011391:exon6:c.T576C:p.A192A | . | 10qD3 | . | 0 | 1 |
| chr10 | 127099273 | C | T | 73 | Os9 | intronic | . | . | . | 10qD3 | . | 0 | 1 |
| chr10 | 127183364 | T | C | 209 | Arhgef25 | exonic | missense SNV | Arhgef25:NM_001166413:exon15:c.A1556G:p.N519S,Arhgef25:NM_028027:exon15:c.A1583G:p.N528S | . | 10qD3 | . | 0 | 1 |
| chr10 | 127231634 | C | T | 77 | Kif5a | intronic | . | . | . | 10qD3 | . | 0 | 1 |
| chr10 | 127330196 | C | T | 135 | Gli1 | exonic | missense SNV | Gli1:NM_010296:exon13:c.G3187A:p.G1063S | . | 10qD3 | . | 0 | 1 |
| chr10 | 127337905 | C | A | 228 | Gli1 | intronic | . | . | . | 10qD3 | rs46294478 | 1 | 0 |
| chr10 | 127568615 | C | A | 165 | Lrp1 | exonic | synonymous SNV | Lrp1:NM_008512:exon37:c.G6036T:p.R2012R | . | 10qD3 | . | 0 | 1 |
| chr10 | 127573457 | C | A | 113 | Lrp1 | exonic | missense SNV | Lrp1:NM_008512:exon29:c.G4792T:p.D1598Y | . | 10qD3 | . | 0 | 1 |
| chr10 | 127648014 | G | T | 220 | Stat6 | intronic | . | . | . | 10qD3 | . | 0 | 1 |
| chr10 | 127729382 | C | A | 167 | Tac2 | intronic | . | . | . | 10qD3 | . | 0 | 1 |
| chr10 | 128069247 | T | G | 45.8626 | Ptges3 | exonic | missense SNV | Ptges3:NM_019766:exon3:c.T148G:p.L50V | . | 10qD3 | . | 0 | 1 |
| chr10 | 128126881 | C | T | 32.4694 | Baz2a | UTR3 | . | . | . | 10qD3 | . | 0 | 1 |
| chr10 | 128378037 | G | A | 127 | Ankrd52 | intronic | . | . | . | 10qD3 | . | 0 | 1 |
| chr10 | 128548949 | C | A | 192 | Rpl41 | exonic | missense SNV | Rpl41:NM_018860:exon2:c.G12T:p.K4N | 362 | 10qD3 | . | 0 | 1 |
| chr10 | 128687006 | G | A | 70 | Rab5b | UTR5 | . | . | . | 10qD3 | . | 0 | 1 |
| chr10 | 129260847 | C | A | 101 | . | upstream | . | . | . | 10qD3 | . | 0 | 1 |
| chr10 | 129333122 | C | A | 183 | Olfr781 | exonic | stopgain | Olfr781:NM_146728:exon1:c.C240A:p.Y80X | . | 10qD3 | . | 0 | 1 |
| chr10 | 129571709 | T | G | 222 | . | downstream | . | . | . | 10qD3 | . | 0 | 1 |
| chr10 | 129608197 | A | C | 148 | Olfr796 | exonic | missense SNV | Olfr796:NM_146931:exon1:c.T283G:p.F95V | . | 10qD3 | . | 0 | 1 |
| chr10 | 129682291 | A | T | 158 | Olfr802 | exonic | synonymous SNV | Olfr802:NM_146932:exon1:c.T447A:p.T149T | . | 10qD3 | . | 0 | 1 |
| chr10 | 129704829 | T | G | 105 | . | upstream | . | . | . | 10qD3 | . | 0 | 1 |
| chr10 | 129723042 | A | G | 72 | Olfr805 | exonic | synonymous SNV | Olfr805:NM_146555:exon1:c.T501C:p.C167C | . | 10qD3 | . | 0 | 1 |
| chr10 | 129767757 | A | G | 222 | Olfr808 | exonic | missense SNV | Olfr808:NM_146928:exon1:c.A260G:p.N87S | . | 10qD3 | . | 0 | 1 |
| chr10 | 129945972 | A | G | 208 | Olfr818 | exonic | missense SNV | Olfr818:NM_146777:exon1:c.T89C:p.F30S | . | 10qD3 | . | 0 | 1 |
| chr10 | 130140037 | T | A | 158 | . | intergenic | . | . | . | 10qD3 | . | 0 | 1 |
| chr11 | 3184396 | A | G | 151 | Sfi1 | intronic | . | . | . | 11qA1 | rs245977243 | 1 | 0 |
| chr11 | 3185674 | C | A | 135 | Sfi1 | intronic | . | . | . | 11qA1 | rs253964654 | 1 | 0 |
| chr11 | 3186465 | T | C | 176 | Sfi1 | intronic | . | . | . | 11qA1 | rs228011584 | 1 | 0 |
| chr11 | 3186846 | T | C | 187 | Sfi1 | intronic | . | . | . | 11qA1 | rs255150581 | 1 | 0 |
| chr11 | 3186873 | T | C | 221 | Sfi1 | intronic | . | . | . | 11qA1 | rs212689856 | 1 | 0 |
| chr11 | 3187048 | G | A | 161 | Sfi1 | intronic | . | . | . | 11qA1 | rs229489030 | 0 | 1 |
| chr11 | 3187367 | T | C | 228 | Sfi1 | exonic | missense SNV | Sfi1:NM_030207:exon3:c.A67G:p.T23A | . | 11qA1 | rs236116745 | 1 | 0 |
| chr11 | 3188350 | G | A | 110 | Sfi1 | intronic | . | . | . | 11qA1 | rs217591471 | 1 | 0 |
| chr11 | 3188396 | A | G | 143 | Sfi1 | intronic | . | . | . | 11qA1 | rs228658667 | 1 | 0 |
| chr11 | 3188608 | T | A | 228 | Sfi1 | intronic | . | . | . | 11qA1 | rs239476124 | 1 | 0 |
| chr11 | 3188892 | A | G | 228 | Sfi1 | intronic | . | . | . | 11qA1 | rs213664603 | 1 | 0 |
| chr11 | 3189023 | T | C | 228 | Sfi1 | UTR5 | . | . | . | 11qA1 | rs230250844 | 1 | 0 |
| chr11 | 3189170 | G | T | 157 | Sfi1 | intronic | . | . | . | 11qA1 | . | 1 | 0 |
| chr11 | 3189174 | G | T | 145 | Sfi1 | intronic | . | . | . | 11qA1 | . | 1 | 0 |
| chr11 | 3189324 | A | G | 228 | Sfi1 | intronic | . | . | . | 11qA1 | rs221840149 | 1 | 0 |
| chr11 | 3193245 | A | G | 133 | Sfi1 | intronic | . | . | 702 | 11qA1 | rs214558303 | 1 | 0 |
| chr11 | 3193291 | C | A | 190 | Sfi1 | intronic | . | . | 702 | 11qA1 | rs237761914 | 1 | 0 |
| chr11 | 3193311 | G | A | 196 | Sfi1 | intronic | . | . | 702 | 11qA1 | rs26899969 | 1 | 0 |
| chr11 | 3193726 | A | G | 116 | . | upstream | . | . | . | 11qA1 | rs254399317 | 1 | 0 |
| chr11 | 3193741 | T | A | 118 | . | upstream | . | . | . | 11qA1 | rs221751088 | 1 | 0 |
| chr11 | 3193874 | C | T | 154 | . | upstream | . | . | . | 11qA1 | rs245132889 | 1 | 0 |
| chr11 | 3193875 | C | T | 154 | . | upstream | . | . | . | 11qA1 | rs262378514 | 1 | 0 |
| chr11 | 3194640 | G | A | 68 | . | intergenic | . | . | . | 11qA1 | rs241594142 | 0 | 1 |
| chr11 | 3196463 | C | A | 70 | . | intergenic | . | . | . | 11qA1 | rs244826889 | 1 | 0 |
| chr11 | 3196761 | G | T | 140 | . | intergenic | . | . | . | 11qA1 | rs221986435 | 0 | 1 |
| chr11 | 3198083 | C | A | 107 | . | intergenic | . | . | . | 11qA1 | rs237117907 | 1 | 0 |
| chr11 | 3198283 | A | G | 83 | . | intergenic | . | . | . | 11qA1 | rs218144618 | 1 | 0 |
| chr11 | 3198284 | A | G | 83 | . | intergenic | . | . | . | 11qA1 | rs235334584 | 1 | 0 |
| chr11 | 3215670 | G | A | 228 | Eif4enif1 | exonic | synonymous SNV | Eif4enif1:NM_001166547:exon4:c.G246A:p.L82L,Eif4enif1:NM_001166548:exon4:c.G246A:p.L82L,Eif4e | . | 11qA1 | rs26884360 | 1 | 0 |
| chr11 | 3215767 | T | C | 228 | Eif4enif1 | intronic | . | . | . | 11qA1 | rs26884359 | 1 | 0 |
| chr11 | 3224479 | A | C | 172 | Eif4enif1 | intronic | . | . | . | 11qA1 | rs233031301 | 1 | 0 |
| chr11 | 3227468 | G | A | 228 | Eif4enif1 | exonic | missense SNV | Eif4enif1:NM_001166547:exon8:c.G1034A:p.R345Q,Eif4enif1:NM_001166548:exon8:c.G1034A:p.R345Q,E | . | 11qA1 | rs26884333 | 1 | 0 |
| chr11 | 3227602 | A | G | 228 | Eif4enif1 | intronic | . | . | . | 11qA1 | rs26884332 | 1 | 0 |
| chr11 | 3233809 | C | T | 148 | Eif4enif1 | intronic | . | . | . | 11qA1 | rs26884328 | 1 | 0 |
| chr11 | 3233871 | T | G | 171 | Eif4enif1 | intronic | . | . | . | 11qA1 | rs227088029 | 1 | 0 |
| chr11 | 3235384 | A | G | 173 | Eif4enif1 | intronic | . | . | . | 11qA1 | rs26884326 | 1 | 0 |
| chr11 | 3236128 | A | G | 218 | Eif4enif1 | intronic | . | . | . | 11qA1 | rs26884324 | 1 | 0 |
| chr11 | 3236410 | C | T | 228 | Eif4enif1 | intronic | . | . | . | 11qA1 | rs26884323 | 1 | 0 |
| chr11 | 3239437 | T | C | 228 | Eif4enif1 | exonic | synonymous SNV | Eif4enif1:NM_001166548:exon14:c.T1950C:p.H650H,Eif4enif1:NM_001166549:exon14:c.T1950C:p.H650H | . | 11qA1 | rs228615962 | 1 | 0 |
| chr11 | 3240688 | A | G | 153 | Eif4enif1,Mir347( | ncRNA_intronic | . | . | . | 11qA1 | rs26884317 | 1 | 0 |
| chr11 | 3240928 | C | A | 228 | Eif4enif1,Mir347( | exonic | synonymous SNV | Eif4enif1:NM_001166548:exon15:c.C2032A:p.R678R,Eif4enif1:NM_001166549:exon15:c.C2032A:p.R678R | . | 11qA1 | rs250973697 | 1 | 0 |
| chr11 | 3242491 | G | A | 228 | Mir3470a,Eif4enif | exonic | missense SNV | Eif4enif1:NM_001166548:exon16:c.G2246A:p.S749N,Eif4enif1:NM_001166549:exon16:c.G2246A:p.S749N | . | 11qA1 | rs6164129 | 1 | 0 |
| chr11 | 3242534 | A | G | 228 | Eif4enif1,Mir347( | exonic | synonymous SNV | Eif4enif1:NM_001166548:exon16:c.A2289G:p.G763G,Eif4enif1:NM_001166549:exon16:c.A2289G:p.G763( | . | 11qA1 | rs6177367 | 1 | 0 |
| chr11 | 3243086 | T | C | 171 | Mir3470a,Eif4enif | ncRNA_intronic | . | . | . | 11qA1 | rs230462840 | 1 | 0 |
| chr11 | 3243127 | C | A | 184 | Mir3470a,Eif4enif | ncRNA_intronic | . | . | . | 11qA1 | rs254434782 | 1 | 0 |
| chr11 | 3250128 | T | A | 213 | Drg1,Mir3470a | ncRNA_intronic | . | . | . | 11qA1 | rs229635624 | 1 | 0 |
| chr11 | 3252391 | C | T | 228 | Mir3470a,Drg1 | ncRNA_intronic | . | . | . | 11qA1 | rs26884304 | 1 | 0 |
| chr11 | 3252912 | G | A | 175 | Drg1,Mir3470a | ncRNA_intronic | . | . | . | 11qA1 | rs225640830 | 1 | 0 |
| chr11 | 3254541 | G | A | 228 | Drg1,Mir3470a | exonic | synonymous SNV | Drg1:NM_007879:exon6:c.C684T:p.D228D | . | 11qA1 | rs260558444 | 1 | 0 |
| chr11 | 3256413 | A | G | 93 | Mir3470a,Drg1 | ncRNA_intronic | . | . | . | 11qA1 | rs212211131 | 1 | 0 |
| chr11 | 3262549 | A | G | 228 | Drg1,Mir3470a | exonic | synonymous SNV | Drg1:NM_007879:exon3:c.T282C:p.Y94Y | . | 11qA1 | rs26884294 | 1 | 0 |
| chr11 | 3264783 | G | A | 228 | Mir3470a,Drg1 | exonic | synonymous SNV | Drg1:NM_007879:exon2:c.C150T:p.G50G | . | 11qA1 | rs584573941 | 1 | 0 |
| chr11 | 3290587 | C | T | 228 | Patz1,Mir3470a | ncRNA_intronic | . | . | 1896 | 11qA1 | rs235617749 | 1 | 0 |
| chr11 | 3290832 | G | A | 228 | Mir3470a,Patz1 | exonic | synonymous SNV | Patz1:NM_001253690:exon1:c.G219A:p.Q73Q,Patz1:NM_001253691:exon1:c.G219A:p.Q73Q,Patz1:NM_019 | 1896 | 11qA1 | rs260319367 | 1 | 0 |
| chr11 | 3291576 | G | A | 228 | Mir3470a,Patz1 | exonic | synonymous SNV | Patz1:NM_001253690:exon1:c.G963A:p.L321L,Patz1:NM_001253691:exon1:c.G963A:p.L321L,Patz1:NM_01 | . | 11qA1 | rs257249879 | 1 | 0 |
| chr11 | 3291944 | A | G | 228 | Patz1,Mir3470a | ncRNA_intronic | . | . | . | 11qA1 | . | 1 | 0 |
| chr11 | 3291951 | T | C | 228 | Patz1,Mir3470a | ncRNA_intronic | . | . | . | 11qA1 | . | 1 | 0 |
| chr11 | 3293383 | T | A | 228 | Mir3470a,Patz1 | ncRNA_intronic | . | . | . | 11qA1 | rs232406994 | 1 | 0 |
| chr11 | 3298568 | T | C | 228 | Mir3470a,Patz1 | ncRNA_intronic | . | . | . | 11qA1 | rs26884260 | 1 | 0 |
| chr11 | 3298602 | T | C | 228 | Patz1,Mir3470a | ncRNA_intronic | . | . | . | 11qA1 | rs26884259 | 1 | 0 |
| chr11 | 3298766 | T | C | 228 | Mir3470a,Patz1 | ncRNA_intronic | . | . | . | 11qA1 | rs242102802 | 1 | 0 |
| chr11 | 3298862 | T | G | 133 | Patz1,Mir3470a | ncRNA_intronic | . | . | . | 11qA1 | rs26884257 | 1 | 0 |
| chr11 | 3306534 | T | A | 228 | Patz1,Mir3470a | ncRNA_intronic | . | . | . | 11qA1 | rs220196383 | 1 | 0 |
| chr11 | 3307240 | G | T | 228 | Patz1,Mir3470a | ncRNA_intronic | . | . | . | 11qA1 | rs26884223 | 1 | 0 |
| chr11 | 3307336 | G | A | 228 | Mir3470a,Patz1 | ncRNA_intronic | . | . | . | 11qA1 | rs232637054 | 1 | 0 |
| chr11 | 3330748 | T | A | 187 | Mir3470a,Pik3ip1 | ncRNA_intronic | . | . | . | 11qA1 | rs26884195 | 1 | 0 |
| chr11 | 3331015 | G | C | 228 | Pik3ip1,Mir3470a | ncRNA_intronic | . | . | . | 11qA1 | rs26884194 | 1 | 0 |
| chr11 | 3332365 | C | T | 121 | Mir3470a,Pik3ip1 | ncRNA_intronic | . | . | 680 | 11qA1 | rs26884189 | 1 | 0 |
| chr11 | 3333244 | A | G | 228 | Pik3ip1,Mir3470a | exonic | missense SNV | Pik3ip1:NM_178149:exon4:c.A343G:p.M115V | . | 11qA1 | rs26884185 | 1 | 0 |
| chr11 | 3333271 | C | G | 228 | Pik3ip1,Mir3470a | exonic | missense SNV | Pik3ip1:NM_178149:exon4:c.C370G:p.P124A | . | 11qA1 | rs26884183 | 1 | 0 |
| chr11 | 3346196 | A | G | 228 | Mir3470a,Limk2 | ncRNA_intronic | . | . | . | 11qA1 | rs26884138 | 1 | 0 |
| chr11 | 3346912 | C | G | 170 | Limk2,Mir3470a | ncRNA_intronic | . | . | . | 11qA1 | rs26884135 | 1 | 0 |
| chr11 | 3346943 | G | A | 178 | Mir3470a,Limk2 | ncRNA_intronic | . | . | . | 11qA1 | rs258025009 | 1 | 0 |
| chr11 | 3347459 | G | A | 225 | Mir3470a,Limk2 | ncRNA_intronic | . | . | . | 11qA1 | rs26884131 | 1 | 0 |
| chr11 | 3347889 | T | C | 130 | Limk2,Mir3470a | ncRNA_intronic | . | . | . | 11qA1 | rs215909600 | 1 | 0 |
| chr11 | 3348378 | A | C | 201 | Mir3470a,Limk2 | ncRNA_intronic | . | . | . | 11qA1 | rs26884128 | 1 | 0 |
| chr11 | 3348452 | A | G | 228 | Mir3470a,Limk2 | ncRNA_intronic | . | . | . | 11qA1 | rs26884127 | 1 | 0 |
| chr11 | 3348536 | T | C | 228 | Mir3470a,Limk2 | ncRNA_intronic | . | . | . | 11qA1 | rs26884126 | 1 | 0 |
| chr11 | 3348590 | G | A | 228 | Limk2,Mir3470a | splicing | . | . | . | 11qA1 | rs26884125 | 1 | 0 |
| chr11 | 3348663 | T | C | 228 | Mir3470a,Limk2 | splicing | . | . | . | 11qA1 | rs26884124 | 1 | 0 |
| chr11 | 3350241 | G | T | 180 | Limk2,Mir3470a | ncRNA_intronic | . | . | . | 11qA1 | rs26884122 | 1 | 0 |
| chr11 | 3350265 | A | G | 194 | Mir3470a,Limk2 | ncRNA_intronic | . | . | . | 11qA1 | rs222124701 | 1 | 0 |
| chr11 | 3350516 | T | C | 228 | Mir3470a,Limk2 | ncRNA_intronic | . | . | . | 11qA1 | rs220506361 | 1 | 0 |
| chr11 | 3350696 | A | G | 228 | Mir3470a,Limk2 | exonic | synonymous SNV | Limk2:NM_001034030:exon6:c.T645C:p.N215N,Limk2:NM_173053:exon9:c.T1143C:p.N381N,Limk2:NM_0107 | . | 11qA1 | rs26884121 | 1 | 0 |
| chr11 | 3350780 | G | A | 228 | Mir3470a,Limk2 | ncRNA_intronic | . | . | . | 11qA1 | rs265825140 | 1 | 0 |
| chr11 | 3350801 | T | G | 228 | Limk2,Mir3470a | ncRNA_intronic | . | . | . | 11qA1 | rs226493742 | 1 | 0 |
| chr11 | 3350908 | G | A | 170 | Limk2,Mir3470a | ncRNA_intronic | . | . | . | 11qA1 | rs225176543 | 1 | 0 |
| chr11 | 3352265 | C | T | 228 | Mir3470a,Limk2 | ncRNA_intronic | . | . | . | 11qA1 | rs26884120 | 1 | 0 |
| chr11 | 3352428 | C | T | 228 | Limk2,Mir3470a | ncRNA_intronic | . | . | . | 11qA1 | rs229312949 | 1 | 0 |
| chr11 | 3352729 | G | A | 171 | Mir3470a,Limk2 | ncRNA_intronic | . | . | . | 11qA1 | rs235826246 | 1 | 0 |
| chr11 | 3352737 | C | T | 172 | Mir3470a,Limk2 | ncRNA_intronic | . | . | . | 11qA1 | . | 1 | 0 |
| chr11 | 3352958 | C | T | 228 | Limk2,Mir3470a | ncRNA_intronic | . | . | . | 11qA1 | rs232575214 | 1 | 0 |
| chr11 | 3353331 | G | A | 228 | Mir3470a,Limk2 | exonic | synonymous SNV | Limk2:NM_001034030:exon4:c.C426T:p.C142C,Limk2:NM_173053:exon7:c.C924T:p.C308C,Limk2:NM_0107 | . | 11qA1 | rs26884117 | 1 | 0 |
| chr11 | 3353505 | C | G | 228 | Mir3470a,Limk2 | ncRNA_intronic | . | . | . | 11qA1 | . | 1 | 0 |
| chr11 | 3355174 | G | A | 173 | Mir3470a,Limk2 | ncRNA_intronic | . | . | . | 11qA1 | rs26884115 | 1 | 0 |
| chr11 | 3355458 | T | C | 228 | Mir3470a,Limk2 | exonic | synonymous SNV | Limk2:NM_001034030:exon3:c.A108G:p.A36A,Limk2:NM_173053:exon6:c.A606G:p.A202A,Limk2:NM_01071 | . | 11qA1 | rs26884112 | 1 | 0 |
| chr11 | 3355949 | G | A | 228 | Mir3470a,Limk2 | ncRNA_intronic | . | . | . | 11qA1 | rs26884111 | 1 | 0 |
| chr11 | 3358034 | T | C | 228 | Limk2,Mir3470a | ncRNA_intronic | . | . | . | 11qA1 | rs26884109 | 1 | 0 |
| chr11 | 3360574 | G | A | 228 | Limk2,Mir3470a | exonic | synonymous SNV | Limk2:NM_173053:exon2:c.C66T:p.C22C,Limk2:NM_010718:exon3:c.C129T:p.C43C | . | 11qA1 | rs26884101 | 1 | 0 |
| chr11 | 3360745 | G | A | 220 | Mir3470a,Limk2 | ncRNA_intronic | . | . | . | 11qA1 | rs26884100 | 1 | 0 |
| chr11 | 3370807 | G | A | 162 | Limk2,Mir3470a | ncRNA_intronic | . | . | . | 11qA1 | rs247739309 | 1 | 0 |
| chr11 | 3370821 | G | A | 163 | Limk2,Mir3470a | ncRNA_intronic | . | . | . | 11qA1 | rs263543413 | 1 | 0 |
| chr11 | 3370877 | G | A | 228 | Limk2,Mir3470a | ncRNA_intronic | . | . | . | 11qA1 | rs29431006 | 1 | 0 |
| chr11 | 3392750 | A | G | 157 | Mir3470a,Limk2 | ncRNA_intronic | . | . | . | 11qA1 | rs26929416 | 1 | 0 |
| chr11 | 3393066 | A | G | 228 | Limk2,Mir3470a | ncRNA_intronic | . | . | . | 11qA1 | rs235480100 | 1 | 0 |
| chr11 | 3393073 | T | C | 225 | Mir3470a,Limk2 | ncRNA_intronic | . | . | . | 11qA1 | rs26929415 | 1 | 0 |
| chr11 | 3408974 | T | C | 228 | Mir3470a,Limk2 | ncRNA_intronic | . | . | 537 | 11qA1 | rs26914066 | 1 | 0 |
| chr11 | 3417821 | T | C | 162 | Rnf185,Mir3470a | ncRNA_intronic | . | . | . | 11qA1 | rs585828902 | 1 | 0 |
| chr11 | 3418138 | T | C | 228 | Mir3470a,Rnf185 | ncRNA_intronic | . | . | . | 11qA1 | rs26914048 | 1 | 0 |
| chr11 | 3419649 | G | C | 168 | Mir3470a,Rnf185 | ncRNA_intronic | . | . | . | 11qA1 | rs26914041 | 1 | 0 |
| chr11 | 3419719 | G | A | 186 | Mir3470a,Rnf185 | ncRNA_intronic | . | . | . | 11qA1 | rs26914040 | 1 | 0 |
| chr11 | 3425274 | C | T | 228 | Rnf185,Mir3470a | ncRNA_intronic | . | . | . | 11qA1 | rs232403904 | 1 | 0 |
| chr11 | 3425279 | T | C | 228 | Rnf185,Mir3470a | splicing | . | . | . | 11qA1 | rs248983533 | 1 | 0 |
| chr11 | 3426387 | C | A | 169 | Mir3470a,Rnf185 | ncRNA_intronic | . | . | . | 11qA1 | rs26914027 | 1 | 0 |
| chr11 | 3426711 | G | A | 228 | Mir3470a,Rnf185 | ncRNA_intronic | . | . | . | 11qA1 | rs26914026 | 1 | 0 |
| chr11 | 3428650 | A | C | 142 | Rnf185,Mir3470a | ncRNA_intronic | . | . | . | 11qA1 | rs26914023 | 1 | 0 |
| chr11 | 3432278 | A | G | 228 | Mir3470a,Rnf185 | ncRNA_intronic | . | . | . | 11qA1 | rs26914018 | 1 | 0 |
| chr11 | 3432716 | C | G | 115 | Mir3470a,Rnf185 | ncRNA_intronic | . | . | . | 11qA1 | rs26914017 | 1 | 0 |
| chr11 | 3438044 | G | C | 172 | Rnf185,Mir3470a | ncRNA_intronic | . | . | . | 11qA1 | rs26914002 | 1 | 0 |
| chr11 | 3438194 | A | G | 228 | Mir3470a,Rnf185 | ncRNA_intronic | . | . | . | 11qA1 | rs221745142 | 1 | 0 |
| chr11 | 3438345 | A | G | 199 | Rnf185,Mir3470a | ncRNA_intronic | . | . | . | 11qA1 | rs26913997 | 1 | 0 |
| chr11 | 3438432 | A | C | 70 | Mir3470a,Rnf185 | ncRNA_intronic | . | . | . | 11qA1 | rs249008066 | 1 | 0 |
| chr11 | 3452434 | G | C | 216 | Mir3470a,Rnf185 | ncRNA_intronic | . | . | 504 | 11qA1 | rs26913988 | 1 | 0 |
| chr11 | 3452553 | A | G | 228 | 8430429K09Rik,Rnf | ncRNA_exonic | . | . | 504 | 11qA1 | rs214630373 | 1 | 0 |
| chr11 | 3452621 | C | T | 228 | Rnf185,Mir3470a,8 | ncRNA_exonic | . | . | 504 | 11qA1 | rs243419830 | 1 | 0 |
| chr11 | 3470785 | G | A | 127 | 8430429K09Rik | ncRNA_intronic | . | . | . | 11qA1 | rs241415796 | 1 | 0 |
| chr11 | 3471033 | G | C | 228 | 8430429K09Rik | ncRNA_intronic | . | . | . | 11qA1 | rs585621339 | 1 | 0 |
| chr11 | 3471099 | G | T | 228 | 8430429K09Rik | ncRNA_intronic | . | . | . | 11qA1 | rs227864703 | 1 | 0 |

| | | | | | | | | | | | | | |
|---|---|---|---|---|---|---|---|---|---|---|---|---|---|
| chr11 | 3471293 | T | C | 228 | 8430429K09Rik | ncRNA_intronic | . | . | . | 11qA1 | rs236011130 | 1 | 0 |
| chr11 | 3471484 | G | C | 228 | 8430429K09Rik | ncRNA_exonic | . | . | . | 11qA1 | rs252636255 | 1 | 0 |
| chr11 | 3488318 | G | A | 228 | Pla2g3 | UTR5 | . | . | . | 11qA1 | rs26913957 | 1 | 0 |
| chr11 | 3488690 | A | T | 228 | Pla2g3 | exonic | synonymous SNV | Pla2g3:NM_172791:exon1:c.A339T:p.A113A | . | 11qA1 | rs231590818 | 1 | 0 |
| chr11 | 3488808 | C | T | 228 | Pla2g3 | exonic | synonymous SNV | Pla2g3:NM_172791:exon1:c.C457T:p.L153L | . | 11qA1 | rs26913956 | 1 | 0 |
| chr11 | 3489533 | G | T | 228 | Pla2g3 | intronic | . | . | . | 11qA1 | rs240723692 | 1 | 0 |
| chr11 | 3491169 | A | C | 228 | Pla2g3 | intronic | . | . | . | 11qA1 | rs257057536 | 1 | 0 |
| chr11 | 3491971 | C | T | 228 | Pla2g3 | exonic | synonymous SNV | Pla2g3:NM_172791:exon5:c.C1134T:p.H378H | . | 11qA1 | rs26913950 | 1 | 0 |
| chr11 | 3492071 | A | G | 228 | Pla2g3 | intronic | . | . | . | 11qA1 | rs26913949 | 1 | 0 |
| chr11 | 3492360 | T | C | 228 | Pla2g3 | intronic | . | . | . | 11qA1 | rs215456526 | 1 | 0 |
| chr11 | 3492386 | G | T | 228 | Pla2g3 | intronic | . | . | . | 11qA1 | rs26913947 | 1 | 0 |
| chr11 | 3492946 | T | C | 228 | Pla2g3 | intronic | . | . | . | 11qA1 | rs26913946 | 1 | 0 |
| chr11 | 3493035 | G | A | 228 | Pla2g3 | exonic | missense SNV | Pla2g3:NM_172791:exon7:c.G1367A:p.R456Q | . | 11qA1 | rs26913945 | 1 | 0 |
| chr11 | 3494646 | G | A | 227 | Inpp5j | UTR3 | . | . | . | 11qA1 | rs220145798 | 1 | 0 |
| chr11 | 3494726 | G | A | 228 | Inpp5j | exonic | synonymous SNV | Inpp5j:NM_172439:exon13:c.C2946T:p.D982D | . | 11qA1 | rs217763984 | 1 | 0 |
| chr11 | 3495706 | C | T | 228 | Inpp5j | intronic | . | . | . | 11qA1 | rs26913943 | 1 | 0 |
| chr11 | 3497888 | C | T | 228 | Inpp5j | intronic | . | . | . | 11qA1 | rs26913940 | 1 | 0 |
| chr11 | 3499796 | A | G | 228 | Inpp5j | intronic | . | . | . | 11qA1 | rs26913938 | 1 | 0 |
| chr11 | 3500213 | A | G | 206 | Inpp5j | exonic | synonymous SNV | Inpp5j:NM_172439:exon7:c.T1815C:p.I605I | . | 11qA1 | . | 0 | 1 |
| chr11 | 3500291 | C | T | 228 | Inpp5j | intronic | . | . | . | 11qA1 | rs26913935 | 1 | 0 |
| chr11 | 3500341 | G | A | 228 | Inpp5j | intronic | . | . | . | 11qA1 | rs260106930 | 1 | 0 |
| chr11 | 3500509 | T | C | 228 | Inpp5j | intronic | . | . | . | 11qA1 | rs225948254 | 1 | 0 |
| chr11 | 3500519 | C | T | 228 | Inpp5j | intronic | . | . | . | 11qA1 | rs246026846 | 1 | 0 |
| chr11 | 3501100 | C | G | 228 | Inpp5j | exonic | synonymous SNV | Inpp5j:NM_172439:exon5:c.G1548C:p.P516P | . | 11qA1 | rs26913934 | 1 | 0 |
| chr11 | 3501224 | C | T | 228 | Inpp5j | intronic | . | . | . | 11qA1 | rs231911347 | 1 | 0 |
| chr11 | 3501326 | A | G | 228 | Inpp5j | exonic | synonymous SNV | Inpp5j:NM_172439:exon4:c.T1479C:p.F493F | . | 11qA1 | rs26913933 | 1 | 0 |
| chr11 | 3501374 | A | G | 228 | Inpp5j | exonic | synonymous SNV | Inpp5j:NM_172439:exon4:c.T1431C:p.T477T | . | 11qA1 | rs26913932 | 1 | 0 |
| chr11 | 3501433 | G | A | 228 | Inpp5j | intronic | . | . | . | 11qA1 | rs26913931 | 1 | 0 |
| chr11 | 3501685 | T | C | 228 | Inpp5j | intronic | . | . | . | 11qA1 | rs259995647 | 1 | 0 |
| chr11 | 3502205 | A | G | 228 | Inpp5j | exonic | synonymous SNV | Inpp5j:NM_172439:exon2:c.T1044C:p.S348S | . | 11qA1 | rs259442708 | 1 | 0 |
| chr11 | 3502527 | G | T | 228 | Inpp5j | exonic | missense SNV | Inpp5j:NM_172439:exon2:c.C722A:p.T241N | . | 11qA1 | rs26913930 | 1 | 0 |
| chr11 | 3502887 | G | C | 228 | Inpp5j | exonic | missense SNV | Inpp5j:NM_172439:exon2:c.C362G:p.A121G | . | 11qA1 | rs242126816 | 1 | 0 |
| chr11 | 3502997 | A | G | 228 | Inpp5j | exonic | synonymous SNV | Inpp5j:NM_172439:exon2:c.T252C:p.P84P | . | 11qA1 | rs239376116 | 1 | 0 |
| chr11 | 3503000 | T | C | 228 | Inpp5j | exonic | synonymous SNV | Inpp5j:NM_172439:exon2:c.A249G:p.R83R | . | 11qA1 | rs264142789 | 1 | 0 |
| chr11 | 3503206 | C | A | 228 | Inpp5j | intronic | . | . | . | 11qA1 | rs26913929 | 1 | 0 |
| chr11 | 3504704 | C | T | 228 | Inpp5j | exonic | synonymous SNV | Inpp5j:NM_172439:exon1:c.G45A:p.R15R | . | 11qA1 | rs26913925 | 1 | 0 |
| chr11 | 3515118 | T | C | 228 | Selm | intronic | . | . | . | 11qA1 | rs221023443 | 1 | 0 |
| chr11 | 3516315 | A | G | 228 | Selm | intronic | . | . | . | 11qA1 | rs223524737 | 1 | 0 |
| chr11 | 3516758 | C | A | 228 | Selm | intronic | . | . | . | 11qA1 | rs226549146 | 1 | 0 |
| chr11 | 3516759 | T | A | 228 | Selm | intronic | . | . | . | 11qA1 | rs245674622 | 1 | 0 |
| chr11 | 3521948 | G | A | 228 | Smtn | exonic | synonymous SNV | Smtn:NM_001284427:exon19:c.C2490T:p.F830F,Smtn:NM_013870:exon19:c.C2490T:p.F830F,Smtn:NM_0011 | . | 11qA1 | rs26913908 | 1 | 0 |
| chr11 | 3522323 | C | T | 228 | Smtn | intronic | . | . | . | 11qA1 | . | 1 | 0 |
| chr11 | 3522338 | G | A | 228 | Smtn | intronic | . | . | . | 11qA1 | rs252617089 | 1 | 0 |
| chr11 | 3522889 | C | T | 228 | Smtn | exonic | missense SNV | Smtn:NM_001284427:exon17:c.G2237A:p.R746K,Smtn:NM_013870:exon17:c.G2237A:p.R746K,Smtn:NM_0011 | . | 11qA1 | rs26913906 | 1 | 0 |
| chr11 | 3523070 | G | A | 228 | Smtn | intronic | . | . | . | 11qA1 | rs26913905 | 1 | 0 |
| chr11 | 3524705 | G | C | 130 | Smtn | exonic | missense SNV | Smtn:NM_001284427:exon16:c.C2105G:p.T702S,Smtn:NM_013870:exon16:c.C2105G:p.T702S,Smtn:NM_0011 | . | 11qA1 | . | 1 | 0 |
| chr11 | 3524816 | T | C | 228 | Smtn | intronic | . | . | . | 11qA1 | rs232716221 | 1 | 0 |
| chr11 | 3525078 | C | T | 228 | Smtn | exonic | missense SNV | Smtn:NM_001284427:exon14:c.G1984A:p.G662S,Smtn:NM_013870:exon14:c.G1984A:p.G662S,Smtn:NM_0011 | . | 11qA1 | rs220462511 | 1 | 0 |
| chr11 | 3526147 | G | A | 228 | Smtn | intronic | . | . | . | 11qA1 | rs223593303 | 1 | 0 |
| chr11 | 3526323 | C | T | 228 | Smtn | intronic | . | . | . | 11qA1 | rs230273882 | 1 | 0 |
| chr11 | 3526537 | A | G | 228 | Smtn | intronic | . | . | . | 11qA1 | rs247307610 | 1 | 0 |
| chr11 | 3526626 | A | G | 115 | Smtn | intronic | . | . | . | 11qA1 | rs261714113 | 1 | 0 |
| chr11 | 3527360 | T | C | 161 | Smtn | intronic | . | . | . | 11qA1 | rs222529827 | 1 | 0 |
| chr11 | 3527380 | T | C | 172 | Smtn | intronic | . | . | . | 11qA1 | rs244442630 | 1 | 0 |
| chr11 | 3530079 | A | C | 228 | Smtn | exonic | missense SNV | Smtn:NM_001284427:exon10:c.T1141G:p.S381A,Smtn:NM_013870:exon10:c.T1141G:p.S381A,Smtn:NM_0011 | . | 11qA1 | rs214457759 | 1 | 0 |
| chr11 | 3530512 | A | G | 228 | Smtn | intronic | . | . | . | 11qA1 | rs6347853 | 1 | 0 |
| chr11 | 3530982 | G | A | 228 | Smtn | intronic | . | . | . | 11qA1 | rs256756021 | 1 | 0 |
| chr11 | 3531074 | A | G | 228 | Smtn | exonic | missense SNV | Smtn:NM_001284427:exon8:c.T836C:p.I279T,Smtn:NM_013870:exon8:c.T836C:p.I279T,Smtn:NM_0011592 | . | 11qA1 | rs236968310 | 1 | 0 |
| chr11 | 3531099 | C | T | 228 | Smtn | exonic | missense SNV | Smtn:NM_001284427:exon8:c.G811A:p.E271K,Smtn:NM_013870:exon8:c.G811A:p.E271K,Smtn:NM_0011592 | . | 11qA1 | rs256528014 | 1 | 0 |
| chr11 | 3531172 | T | C | 228 | Smtn | intronic | . | . | . | 11qA1 | rs228483992 | 1 | 0 |
| chr11 | 3531232 | C | G | 228 | Smtn | exonic | synonymous SNV | Smtn:NM_001284427:exon7:c.G762C:p.P254P,Smtn:NM_013870:exon7:c.G762C:p.P254P,Smtn:NM_0011592 | . | 11qA1 | rs232340132 | 1 | 0 |
| chr11 | 3531272 | G | A | 228 | Smtn | exonic | missense SNV | Smtn:NM_001284427:exon7:c.C722T:p.S241L,Smtn:NM_013870:exon7:c.C722T:p.S241L,Smtn:NM_0011592 | . | 11qA1 | rs231230202 | 1 | 0 |
| chr11 | 3531542 | C | T | 228 | Smtn | intronic | . | . | . | 11qA1 | rs213056801 | 1 | 0 |
| chr11 | 3532691 | A | C | 228 | Smtn | intronic | . | . | . | 11qA1 | rs226191487 | 1 | 0 |
| chr11 | 3533224 | G | C | 176 | Smtn | intronic | . | . | . | 11qA1 | rs241399279 | 1 | 0 |
| chr11 | 3533283 | C | T | 228 | Smtn | intronic | . | . | . | 11qA1 | rs251088098 | 1 | 0 |
| chr11 | 3533449 | C | T | 228 | Smtn | exonic | synonymous SNV | Smtn:NM_001284427:exon3:c.G126A:p.L42L,Smtn:NM_013870:exon3:c.G126A:p.L42L,Smtn:NM_001159284: | . | 11qA1 | rs215613874 | 1 | 0 |
| chr11 | 3533561 | C | A | 228 | Smtn | intronic | . | . | . | 11qA1 | rs229691751 | 1 | 0 |
| chr11 | 3533684 | A | G | 213 | Smtn | intronic | . | . | . | 11qA1 | rs262757675 | 1 | 0 |
| chr11 | 3625381 | A | G | 228 | . | intergenic | . | . | . | 11qA1 | rs26913785 | 1 | 0 |
| chr11 | 3625477 | T | A | 228 | . | intergenic | . | . | . | 11qA1 | rs26913784 | 1 | 0 |
| chr11 | 3625532 | A | G | 228 | . | intergenic | . | . | . | 11qA1 | rs26913783 | 1 | 0 |
| chr11 | 3625770 | A | G | 228 | . | intergenic | . | . | . | 11qA1 | rs26913781 | 1 | 0 |
| chr11 | 3625778 | C | T | 228 | . | intergenic | . | . | . | 11qA1 | . | 1 | 0 |
| chr11 | 3628927 | A | C | 228 | . | intergenic | . | . | . | 11qA1 | rs26913778 | 1 | 0 |
| chr11 | 3629088 | T | G | 228 | . | intergenic | . | . | 613 | 11qA1 | rs26913777 | 1 | 0 |
| chr11 | 3629229 | C | G | 228 | . | intergenic | . | . | 613 | 11qA1 | rs26913775 | 1 | 0 |
| chr11 | 3629438 | T | C | 228 | . | intergenic | . | . | 613 | 11qA1 | rs213168310 | 1 | 0 |
| chr11 | 3629715 | T | C | 228 | . | intergenic | . | . | . | 11qA1 | rs26913773 | 1 | 0 |
| chr11 | 3633390 | T | C | 228 | . | intergenic | . | . | . | 11qA1 | rs26897563 | 1 | 0 |
| chr11 | 3633498 | T | C | 228 | . | intergenic | . | . | . | 11qA1 | rs241911499 | 1 | 0 |
| chr11 | 3646361 | C | T | 169 | Tug1 | ncRNA_intronic | . | . | . | 11qA1 | rs242047064 | 1 | 0 |
| chr11 | 3646923 | T | A | 228 | Tug1 | ncRNA_exonic | . | . | . | 11qA1 | rs215375573 | 1 | 0 |
| chr11 | 3649310 | G | A | 228 | . | upstream | . | . | 955 | 11qA1 | rs228016473 | 1 | 0 |
| chr11 | 3649327 | C | G | 228 | . | upstream | . | . | 955 | 11qA1 | . | 1 | 0 |
| chr11 | 3649368 | A | G | 228 | . | upstream | . | . | 955 | 11qA1 | rs265452842 | 1 | 0 |
| chr11 | 3650129 | C | G | 160 | Morc2a | UTR5 | . | . | . | 11qA1 | rs262089870 | 1 | 0 |
| chr11 | 3650131 | G | A | 160 | Morc2a | UTR5 | . | . | . | 11qA1 | rs223769012 | 1 | 0 |
| chr11 | 3658662 | A | G | 228 | Morc2a | intronic | . | . | . | 11qA1 | rs6172814 | 1 | 0 |
| chr11 | 3672548 | C | A | 195 | Morc2a | intronic | . | . | . | 11qA1 | rs26897519 | 1 | 0 |
| chr11 | 3676280 | A | G | 228 | Morc2a | intronic | . | . | . | 11qA1 | rs26897505 | 1 | 0 |
| chr11 | 3676362 | A | C | 213 | Morc2a | intronic | . | . | . | 11qA1 | rs219968804 | 1 | 0 |
| chr11 | 3676433 | C | G | 164 | Morc2a | intronic | . | . | . | 11qA1 | rs26897504 | 1 | 0 |
| chr11 | 3676611 | G | A | 228 | Morc2a | intronic | . | . | . | 11qA1 | rs26897503 | 1 | 0 |
| chr11 | 3678115 | T | C | 228 | Morc2a | intronic | . | . | . | 11qA1 | rs237168295 | 1 | 0 |
| chr11 | 3678417 | T | A | 228 | Morc2a | intronic | . | . | . | 11qA1 | rs29446724 | 1 | 0 |
| chr11 | 3678738 | G | A | 228 | Morc2a | intronic | . | . | . | 11qA1 | rs264165559 | 1 | 0 |
| chr11 | 3679500 | C | T | 169 | Morc2a | intronic | . | . | . | 11qA1 | rs213606987 | 1 | 0 |
| chr11 | 3679652 | T | A | 228 | Morc2a | splicing | . | . | . | 11qA1 | rs220152663 | 1 | 0 |
| chr11 | 3680153 | T | C | 228 | Morc2a | intronic | . | . | . | 11qA1 | rs230481258 | 1 | 0 |
| chr11 | 3680455 | T | C | 228 | Morc2a | intronic | . | . | . | 11qA1 | rs228818422 | 1 | 0 |
| chr11 | 3680539 | A | G | 228 | Morc2a | intronic | . | . | . | 11qA1 | rs253741874 | 1 | 0 |
| chr11 | 3681534 | C | T | 148 | Morc2a | intronic | . | . | . | 11qA1 | rs245740406 | 1 | 0 |
| chr11 | 3683379 | G | A | 173 | Morc2a | intronic | . | . | . | 11qA1 | rs264852011 | 1 | 0 |
| chr11 | 3683459 | C | T | 228 | Morc2a | intronic | . | . | . | 11qA1 | rs228549284 | 1 | 0 |
| chr11 | 3684198 | A | C | 222 | Morc2a | intronic | . | . | . | 11qA1 | rs214084357 | 1 | 0 |
| chr11 | 3684359 | A | G | 170 | Morc2a | intronic | . | . | . | 11qA1 | rs249200559 | 1 | 0 |
| chr11 | 3684848 | G | T | 173 | Morc2a | exonic | missense SNV | Morc2a:NM_198162:exon21:c.G2147T:p.S716I,Morc2a:NM_001159288:exon22:c.G2330T:p.S777I | . | 11qA1 | . | 0 | 1 |
| chr11 | 3684987 | G | C | 228 | Morc2a | intronic | . | . | . | 11qA1 | rs223176880 | 1 | 0 |
| chr11 | 3685437 | A | C | 228 | Morc2a | exonic | synonymous SNV | Morc2a:NM_198162:exon22:c.A2244C:p.R748R,Morc2a:NM_001159288:exon23:c.A2427C:p.R809R | . | 11qA1 | rs258930668 | 1 | 0 |
| chr11 | 3687879 | G | A | 126 | Morc2a | intronic | . | . | . | 11qA1 | rs235935791 | 1 | 0 |
| chr11 | 3688490 | A | C | 163 | Morc2a | intronic | . | . | . | 11qA1 | rs26897502 | 1 | 0 |
| chr11 | 3689125 | G | T | 228 | Morc2a | UTR3 | . | . | . | 11qA1 | rs26897497 | 1 | 0 |
| chr11 | 3689177 | C | G | 228 | Morc2a | UTR3 | . | . | . | 11qA1 | rs26897496 | 1 | 0 |
| chr11 | 3711541 | C | T | 115 | Osbp2 | intronic | . | . | . | 11qA1 | rs248944975 | 1 | 0 |
| chr11 | 3711720 | T | C | 228 | Osbp2 | exonic | synonymous SNV | Osbp2:NM_001302631:exon9:c.A1098G:p.K366K,Osbp2:NM_001302630:exon10:c.A972G:p.K324K,Osbp2:NM_ | . | 11qA1 | rs51752609 | 1 | 0 |
| chr11 | 3711828 | G | A | 228 | Osbp2 | exonic | synonymous SNV | Osbp2:NM_001302631:exon9:c.C990T:p.Y330Y,Osbp2:NM_001302630:exon10:c.C864T:p.Y288Y,Osbp2:NM_1 | . | 11qA1 | rs46061986 | 1 | 0 |
| chr11 | 3711994 | T | C | 228 | Osbp2 | exonic | synonymous SNV | Osbp2:NM_001302631:exon8:c.A912G:p.V304V,Osbp2:NM_001302630:exon9:c.A786G:p.V262V,Osbp2:NM_15 | . | 11qA1 | rs26897444 | 1 | 0 |
| chr11 | 3712258 | A | G | 228 | Osbp2 | intronic | . | . | . | 11qA1 | rs259950189 | 1 | 0 |
| chr11 | 3712266 | G | A | 228 | Osbp2 | intronic | . | . | . | 11qA1 | rs46378859 | 1 | 0 |
| chr11 | 3712289 | C | T | 228 | Osbp2 | intronic | . | . | . | 11qA1 | rs45845454 | 1 | 0 |
| chr11 | 3712674 | C | T | 228 | Osbp2 | intronic | . | . | . | 11qA1 | rs47678488 | 1 | 0 |
| chr11 | 3714720 | T | C | 228 | Osbp2 | splicing | . | . | . | 11qA1 | rs26897428 | 1 | 0 |
| chr11 | 3714783 | G | A | 228 | Osbp2 | intronic | . | . | . | 11qA1 | rs26897427 | 1 | 0 |
| chr11 | 3715056 | C | T | 228 | Osbp2 | intronic | . | . | . | 11qA1 | rs26897425 | 1 | 0 |
| chr11 | 3715437 | T | C | 228 | Osbp2 | intronic | . | . | . | 11qA1 | rs215808257 | 1 | 0 |
| chr11 | 3717093 | T | C | 228 | Osbp2 | splicing | . | . | . | 11qA1 | rs26897414 | 1 | 0 |
| chr11 | 3717273 | A | G | 200 | Osbp2 | intronic | . | . | . | 11qA1 | rs26897413 | 1 | 0 |
| chr11 | 3717698 | A | G | 180 | Osbp2 | intronic | . | . | . | 11qA1 | rs26897412 | 1 | 0 |
| chr11 | 3717894 | T | C | 228 | Osbp2 | exonic | synonymous SNV | Osbp2:NM_152818:exon4:c.A1194G:p.E398E | . | 11qA1 | rs26897410 | 1 | 0 |
| chr11 | 3722047 | C | G | 118 | Osbp2 | intronic | . | . | . | 11qA1 | rs26897388 | 1 | 0 |
| chr11 | 3722167 | T | C | 228 | Osbp2 | UTR5 | . | . | . | 11qA1 | rs26897387 | 1 | 0 |
| chr11 | 3726190 | G | T | 107 | Osbp2 | intronic | . | . | . | 11qA1 | . | 0 | 1 |
| chr11 | 3726264 | G | A | 228 | Osbp2 | intronic | . | . | . | 11qA1 | rs26897365 | 1 | 0 |
| chr11 | 3726421 | A | G | 228 | Osbp2 | exonic | synonymous SNV | Osbp2:NM_152818:exon3:c.T999C:p.N333N | . | 11qA1 | rs244368527 | 1 | 0 |
| chr11 | 3726664 | G | T | 228 | Osbp2 | intronic | . | . | . | 11qA1 | rs26897364 | 1 | 0 |
| chr11 | 3726723 | C | T | 228 | Osbp2 | intronic | . | . | . | 11qA1 | rs26897363 | 1 | 0 |
| chr11 | 3773685 | C | T | 159 | Osbp2 | intronic | . | . | . | 11qA1 | . | 1 | 0 |
| chr11 | 3773731 | G | A | 204 | Osbp2 | intronic | . | . | . | 11qA1 | rs243151590 | 1 | 0 |
| chr11 | 3773764 | A | G | 210 | Osbp2 | intronic | . | . | . | 11qA1 | rs258755695 | 1 | 0 |
| chr11 | 3819713 | A | G | 107 | Osbp2 | intronic | . | . | . | 11qA1 | rs225477574 | 1 | 0 |
| chr11 | 3819781 | A | T | 177 | Osbp2 | intronic | . | . | . | 11qA1 | rs26884613 | 1 | 0 |
| chr11 | 3819795 | C | T | 197 | Osbp2 | intronic | . | . | . | 11qA1 | . | 1 | 0 |
| chr11 | 3819877 | C | T | 228 | Osbp2 | exonic | synonymous SNV | Osbp2:NM_152818:exon2:c.G804A:p.L268L | . | 11qA1 | rs26884612 | 1 | 0 |
| chr11 | 3853593 | A | G | 191 | Osbp2 | intronic | . | . | . | 11qA1 | rs214878176 | 1 | 0 |
| chr11 | 3873363 | C | T | 228 | . | intergenic | . | . | . | 11qA1 | rs217648704 | 1 | 0 |
| chr11 | 3873830 | G | A | 228 | . | intergenic | . | . | . | 11qA1 | . | 1 | 0 |
| chr11 | 3873837 | C | T | 228 | . | intergenic | . | . | . | 11qA1 | . | 1 | 0 |
| chr11 | 3888137 | A | C | 228 | 4921536K21Rik | UTR3 | . | . | . | 11qA1 | rs220997392 | 1 | 0 |
| chr11 | 3888526 | A | G | 101 | 4921536K21Rik | intronic | . | . | . | 11qA1 | rs230801725 | 1 | 0 |
| chr11 | 3889842 | C | T | 160 | 4921536K21Rik | intronic | . | . | . | 11qA1 | . | 1 | 0 |
| chr11 | 3894773 | A | G | 228 | 4921536K21Rik | intronic | . | . | 860 | 11qA1 | rs244099520 | 1 | 0 |
| chr11 | 3894977 | T | C | 228 | 4921536K21Rik | exonic | synonymous SNV | 4921536K21Rik:NM_026150:exon1:c.A111G:p.K37K | 860 | 11qA1 | rs224876929 | 1 | 0 |
| chr11 | 3895055 | C | T | 228 | 4921536K21Rik | exonic | synonymous SNV | 4921536K21Rik:NM_026150:exon1:c.G33A:p.S11S | 860 | 11qA1 | . | 1 | 0 |
| chr11 | 3895136 | T | A | 228 | . | upstream | . | . | 860 | 11qA1 | . | 1 | 0 |
| chr11 | 3895152 | G | A | 228 | . | upstream | . | . | 860 | 11qA1 | . | 1 | 0 |
| chr11 | 3895254 | G | A | 188 | Dusp18 | UTR5 | . | . | 860 | 11qA1 | . | 1 | 0 |
| chr11 | 3896943 | G | A | 158 | Dusp18 | UTR5 | . | . | . | 11qA1 | rs26884523 | 1 | 0 |
| chr11 | 3897008 | T | C | 228 | Dusp18 | UTR5 | . | . | . | 11qA1 | rs26884522 | 1 | 0 |
| chr11 | 3897350 | G | C | 228 | Dusp18 | exonic | synonymous SNV | Dusp18:NM_173745:exon2:c.G339C:p.A113A | . | 11qA1 | rs263708185 | 1 | 0 |
| chr11 | 3897449 | C | T | 228 | Dusp18 | exonic | synonymous SNV | Dusp18:NM_173745:exon2:c.C438T:p.G146G | . | 11qA1 | rs26884520 | 1 | 0 |
| chr11 | 3897687 | G | T | 163 | Dusp18 | UTR3 | . | . | . | 11qA1 | . | 1 | 0 |
| chr11 | 3897828 | G | C | 195 | Dusp18 | UTR3 | . | . | . | 11qA1 | rs238876850 | 1 | 0 |
| chr11 | 3897979 | T | C | 228 | Dusp18 | UTR3 | . | . | . | 11qA1 | rs218581842 | 1 | 0 |
| chr11 | 3898008 | G | A | 228 | Dusp18 | UTR3 | . | . | . | 11qA1 | rs26884519 | 1 | 0 |
| chr11 | 3898050 | G | C | 228 | Dusp18 | UTR3 | . | . | . | 11qA1 | rs26884518 | 1 | 0 |
| chr11 | 3898124 | A | T | 228 | Dusp18 | UTR3 | . | . | . | 11qA1 | rs26884517 | 1 | 0 |
| chr11 | 3898254 | T | C | 228 | Dusp18 | UTR3 | . | . | . | 11qA1 | rs265048694 | 1 | 0 |
| chr11 | 3898339 | C | T | 228 | Dusp18 | UTR3 | . | . | . | 11qA1 | rs26884516 | 1 | 0 |
| chr11 | 3898429 | C | G | 228 | Dusp18 | UTR3 | . | . | . | 11qA1 | rs232641922 | 1 | 0 |
| chr11 | 3898563 | G | A | 168 | Dusp18 | UTR3 | . | . | . | 11qA1 | rs247857792 | 1 | 0 |
| chr11 | 3898932 | T | C | 228 | Dusp18 | UTR3 | . | . | . | 11qA1 | rs26884515 | 1 | 0 |
| chr11 | 3898944 | G | C | 228 | Dusp18 | UTR3 | . | . | . | 11qA1 | rs26884514 | 1 | 0 |
| chr11 | 3907437 | G | A | 169 | Slc35e4 | UTR3 | . | . | . | 11qA1 | rs266095555 | 1 | 0 |
| chr11 | 3907845 | G | A | 190 | Slc35e4 | exonic | synonymous SNV | Slc35e4:NM_153142:exon2:c.C759T:p.R253R | . | 11qA1 | . | 0 | 1 |
| chr11 | 3908172 | A | G | 115 | Slc35e4 | intronic | . | . | . | 11qA1 | rs26884486 | 1 | 0 |
| chr11 | 3913227 | G | A | 228 | Slc35e4 | UTR5 | . | . | . | 11qA1 | rs228198040 | 1 | 0 |
| chr11 | 3917778 | T | C | 228 | Tcn2 | intronic | . | . | . | 11qA1 | rs225750045 | 1 | 0 |
| chr11 | 3919252 | G | A | 228 | Tcn2 | intronic | . | . | . | 11qA1 | . | 1 | 0 |
| chr11 | 3919270 | A | G | 228 | Tcn2 | intronic | . | . | . | 11qA1 | rs254229732 | 1 | 0 |
| chr11 | 3919503 | G | C | 228 | Tcn2 | intronic | . | . | . | 11qA1 | rs252495190 | 1 | 0 |
| chr11 | 3919520 | G | A | 228 | Tcn2 | intronic | . | . | . | 11qA1 | rs212840591 | 1 | 0 |
| chr11 | 3921996 | A | G | 228 | Tcn2 | intronic | . | . | . | 11qA1 | rs226391503 | 1 | 0 |
| chr11 | 3922287 | G | A | 228 | Tcn2 | intronic | . | . | . | 11qA1 | rs215197545 | 1 | 0 |
| chr11 | 3923252 | G | C | 173 | Tcn2 | intronic | . | . | . | 11qA1 | . | 1 | 0 |

| | | | | | | | | | | | | | |
|---|---|---|---|---|---|---|---|---|---|---|---|---|---|
| chr11 | 3923292 | G | A | 209 | Tcn2 | intronic | . | . | . | 11qA1 | rs584744664 | 1 | 0 |
| chr11 | 3923484 | T | C | 228 | Tcn2 | exonic | synonymous SNV | Tcn2:NM_001130458:exon7:c.A861G:p.Q287Q,Tcn2:NM_001130459:exon7:c.A861G:p.Q287Q,Tcn2:NM_0157 | . | 11qA1 | rs264769134 | 1 | 0 |
| chr11 | 3923487 | G | C | 228 | Tcn2 | exonic | missense SNV | Tcn2:NM_001130458:exon7:c.C858G:p.F286L,Tcn2:NM_001130459:exon7:c.C858G:p.F286L,Tcn2:NM_0157 | . | 11qA1 | . | 1 | 0 |
| chr11 | 3923695 | C | T | 228 | Tcn2 | intronic | . | . | . | 11qA1 | rs231345955 | 1 | 0 |
| chr11 | 3923698 | T | C | 228 | Tcn2 | splicing | . | . | . | 11qA1 | rs249243158 | 1 | 0 |
| chr11 | 3924781 | G | A | 166 | Tcn2 | intronic | . | . | . | 11qA1 | . | 1 | 0 |
| chr11 | 3924872 | G | A | 228 | Tcn2 | intronic | . | . | . | 11qA1 | . | 1 | 0 |
| chr11 | 3925209 | A | G | 228 | Tcn2 | intronic | . | . | . | 11qA1 | rs261717390 | 1 | 0 |
| chr11 | 3925288 | T | C | 165 | Tcn2 | intronic | . | . | . | 11qA1 | rs250908972 | 1 | 0 |
| chr11 | 3927542 | A | G | 228 | Tcn2 | exonic | synonymous SNV | Tcn2:NM_001130458:exon3:c.T105C:p.G35G,Tcn2:NM_001130459:exon3:c.T105C:p.G35G,Tcn2:NM_015749: | . | 11qA1 | rs13474404 | 1 | 0 |
| chr11 | 3927591 | A | G | 92 | Tcn2 | intronic | . | . | . | 11qA1 | . | 0 | 1 |
| chr11 | 3937782 | G | A | 133 | . | intergenic | . | . | . | 11qA1 | . | 1 | 0 |
| chr11 | 3937995 | A | G | 228 | . | intergenic | . | . | . | 11qA1 | rs26884469 | 1 | 0 |
| chr11 | 3952253 | A | G | 228 | . | intergenic | . | . | . | 11qA1 | . | 1 | 0 |
| chr11 | 3952396 | T | C | 228 | . | intergenic | . | . | . | 11qA1 | . | 1 | 0 |
| chr11 | 3952749 | T | C | 222 | . | intergenic | . | . | . | 11qA1 | . | 0 | 1 |
| chr11 | 3952805 | C | T | 228 | . | intergenic | . | . | . | 11qA1 | . | 1 | 0 |
| chr11 | 3952819 | C | T | 228 | . | intergenic | . | . | . | 11qA1 | . | 1 | 0 |
| chr11 | 3968470 | G | A | 213 | Pes1 | intronic | . | . | . | 11qA1 | rs6204357 | 1 | 0 |
| chr11 | 3968514 | G | A | 169 | Pes1 | intronic | . | . | . | 11qA1 | rs6204428 | 1 | 0 |
| chr11 | 3969620 | C | T | 228 | Pes1 | intronic | . | . | . | 11qA1 | rs221840632 | 1 | 0 |
| chr11 | 3969694 | C | G | 228 | Pes1 | intronic | . | . | . | 11qA1 | . | 1 | 0 |
| chr11 | 3970813 | G | A | 228 | Pes1 | intronic | . | . | . | 11qA1 | rs239240113 | 1 | 0 |
| chr11 | 3970870 | A | G | 170 | Pes1 | intronic | . | . | . | 11qA1 | . | 1 | 0 |
| chr11 | 3973185 | T | A | 190 | Pes1 | intronic | . | . | . | 11qA1 | rs227798211 | 1 | 0 |
| chr11 | 3973341 | T | C | 228 | Pes1 | exonic | synonymous SNV | Pes1:NM_022889:exon5:c.T513C:p.Y171Y | . | 11qA1 | rs13471074 | 1 | 0 |
| chr11 | 3973380 | C | T | 228 | Pes1 | intronic | . | . | . | 11qA1 | rs236225136 | 1 | 0 |
| chr11 | 3975274 | G | T | 127 | Pes1 | intronic | . | . | . | 11qA1 | rs215377109 | 1 | 0 |
| chr11 | 3975438 | T | C | 228 | Pes1 | intronic | . | . | . | 11qA1 | rs222034911 | 1 | 0 |
| chr11 | 3975522 | T | C | 228 | Pes1 | intronic | . | . | . | 11qA1 | rs221589634 | 1 | 0 |
| chr11 | 3975880 | T | A | 228 | Pes1 | intronic | . | . | . | 11qA1 | rs219845582 | 1 | 0 |
| chr11 | 3976324 | C | T | 228 | Pes1 | exonic | synonymous SNV | Pes1:NM_022889:exon10:c.C946T:p.L316L | . | 11qA1 | . | 1 | 0 |
| chr11 | 3976649 | A | G | 228 | Pes1 | intronic | . | . | . | 11qA1 | rs251528633 | 1 | 0 |
| chr11 | 3976950 | T | C | 228 | Pes1 | intronic | . | . | . | 11qA1 | rs262592059 | 1 | 0 |
| chr11 | 3977319 | C | T | 228 | Pes1 | intronic | . | . | . | 11qA1 | . | 1 | 0 |
| chr11 | 3977325 | A | G | 228 | Pes1 | intronic | . | . | . | 11qA1 | rs260481847 | 1 | 0 |
| chr11 | 3977750 | T | G | 228 | Pes1 | exonic | synonymous SNV | Pes1:NM_022889:exon13:c.T1476G:p.L492L | . | 11qA1 | rs229265621 | 1 | 0 |
| chr11 | 3978188 | T | C | 170 | Pes1 | intronic | . | . | . | 11qA1 | . | 1 | 0 |
| chr11 | 3978199 | A | G | 168 | Pes1 | intronic | . | . | . | 11qA1 | . | 1 | 0 |
| chr11 | 3978665 | G | A | 172 | Pes1 | intronic | . | . | . | 11qA1 | . | 1 | 0 |
| chr11 | 3978919 | T | C | 228 | Pes1 | UTR3 | . | . | . | 11qA1 | rs229031959 | 1 | 0 |
| chr11 | 3979004 | A | G | 228 | Pes1 | UTR3 | . | . | . | 11qA1 | rs258245636 | 1 | 0 |
| chr11 | 3979121 | T | C | 161 | Pes1 | UTR3 | . | . | . | 11qA1 | rs240728993 | 1 | 0 |
| chr11 | 3996897 | C | T | 228 | Gal3st1 | intronic | . | . | . | 11qA1 | rs26884426 | 1 | 0 |
| chr11 | 3996935 | A | G | 228 | Gal3st1 | exonic | synonymous SNV | Gal3st1:NM_001177691:exon2:c.A15G:p.P5P,Gal3st1:NM_001177703:exon2:c.A15G:p.P5P,Gal3st1:NM_01 | . | 11qA1 | rs26884425 | 1 | 0 |
| chr11 | 3996966 | T | C | 228 | Gal3st1 | exonic | synonymous SNV | Gal3st1:NM_001177691:exon2:c.T46C:p.L16L,Gal3st1:NM_001177703:exon2:c.T46C:p.L16L,Gal3st1:NM | . | 11qA1 | rs26884424 | 1 | 0 |
| chr11 | 3997152 | T | C | 228 | Gal3st1 | intronic | . | . | . | 11qA1 | rs52026359 | 1 | 0 |
| chr11 | 3997156 | C | T | 228 | Gal3st1 | intronic | . | . | . | 11qA1 | rs51826440 | 1 | 0 |
| chr11 | 3997953 | C | T | 222 | Gal3st1 | exonic | synonymous SNV | Gal3st1:NM_001177691:exon3:c.C159T:p.S53S,Gal3st1:NM_001177703:exon3:c.C159T:p.S53S,Gal3st1:N | . | 11qA1 | . | 1 | 0 |
| chr11 | 3998232 | T | C | 228 | Gal3st1 | exonic | synonymous SNV | Gal3st1:NM_001177691:exon3:c.T438C:p.Y146Y,Gal3st1:NM_001177703:exon3:c.T438C:p.Y146Y,Gal3st1 | 834 | 11qA1 | . | 1 | 0 |
| chr11 | 3998631 | T | C | 228 | Gal3st1 | exonic | synonymous SNV | Gal3st1:NM_001177691:exon3:c.T837C:p.N279N,Gal3st1:NM_001177703:exon3:c.T837C:p.N279N,Gal3st1 | 834 | 11qA1 | rs26884419 | 1 | 0 |
| chr11 | 3998822 | A | G | 228 | Gal3st1 | exonic | missense SNV | Gal3st1:NM_001177691:exon3:c.A1028G:p.H343R,Gal3st1:NM_001177703:exon3:c.A1028G:p.H343R,Gal3s | . | 11qA1 | rs244003552 | 1 | 0 |
| chr11 | 3998940 | C | T | 228 | Gal3st1 | exonic | synonymous SNV | Gal3st1:NM_001177691:exon3:c.C1146T:p.I382I,Gal3st1:NM_001177703:exon3:c.C1146T:p.I382I,Gal3s | . | 11qA1 | . | 1 | 0 |
| chr11 | 3998946 | A | G | 228 | Gal3st1 | exonic | synonymous SNV | Gal3st1:NM_001177691:exon3:c.A1152G:p.P384P,Gal3st1:NM_001177703:exon3:c.A1152G:p.P384P,Gal3s | . | 11qA1 | rs26884418 | 1 | 0 |
| chr11 | 3999214 | A | G | 158 | Gal3st1 | UTR3 | . | . | . | 11qA1 | rs26884417 | 1 | 0 |
| chr11 | 4002445 | A | G | 228 | . | intergenic | . | . | . | 11qA1 | rs26884407 | 1 | 0 |
| chr11 | 4002691 | C | G | 228 | . | intergenic | . | . | . | 11qA1 | rs6222043 | 1 | 0 |
| chr11 | 4002899 | A | G | 228 | . | intergenic | . | . | . | 11qA1 | rs6235981 | 1 | 0 |
| chr11 | 4002918 | A | G | 228 | . | intergenic | . | . | . | 11qA1 | rs6236015 | 1 | 0 |
| chr11 | 4031735 | G | A | 228 | . | upstream | . | . | . | 11qA1 | . | 1 | 0 |
| chr11 | 4035339 | C | A | 210 | Sec14l4 | intronic | . | . | . | 11qA1 | rs6164746 | 1 | 0 |
| chr11 | 4039611 | T | C | 228 | Sec14l4 | intronic | . | . | . | 11qA1 | rs213210931 | 1 | 0 |
| chr11 | 4040136 | C | T | 228 | Sec14l4 | intronic | . | . | . | 11qA1 | rs26884373 | 1 | 0 |
| chr11 | 4041617 | A | G | 213 | Sec14l4 | intronic | . | . | . | 11qA1 | rs26930949 | 1 | 0 |
| chr11 | 4041643 | T | C | 228 | Sec14l4 | intronic | . | . | . | 11qA1 | rs26930948 | 1 | 0 |
| chr11 | 4041733 | A | G | 228 | Sec14l4 | splicing | . | . | . | 11qA1 | rs26930946 | 1 | 0 |
| chr11 | 4042286 | A | G | 228 | Sec14l4 | intronic | . | . | . | 11qA1 | rs258602531 | 1 | 0 |
| chr11 | 4043057 | A | G | 93 | Sec14l4 | intronic | . | . | . | 11qA1 | rs6202743 | 1 | 0 |
| chr11 | 4043258 | G | C | 228 | Sec14l4 | intronic | . | . | . | 11qA1 | rs6203882 | 1 | 0 |
| chr11 | 4043482 | A | G | 228 | Sec14l4 | intronic | . | . | . | 11qA1 | rs6205074 | 1 | 0 |
| chr11 | 4043531 | C | T | 228 | Sec14l4 | intronic | . | . | . | 11qA1 | rs6205565 | 1 | 0 |
| chr11 | 4043566 | G | T | 228 | Sec14l4 | intronic | . | . | . | 11qA1 | rs6205620 | 1 | 0 |
| chr11 | 4043634 | G | T | 84 | Sec14l4 | intronic | . | . | . | 11qA1 | . | 1 | 0 |
| chr11 | 4043735 | T | C | 228 | Sec14l4 | exonic | synonymous SNV | Sec14l4:NM_146013:exon9:c.T747C:p.D249D | . | 11qA1 | rs26930941 | 1 | 0 |
| chr11 | 4043856 | G | C | 228 | Sec14l4 | exonic | synonymous SNV | Sec14l4:NM_146013:exon10:c.G795C:p.P265P | . | 11qA1 | rs26930940 | 1 | 0 |
| chr11 | 4044018 | T | C | 228 | Sec14l4 | intronic | . | . | . | 11qA1 | . | 1 | 0 |
| chr11 | 4044350 | T | G | 140 | Sec14l4 | intronic | . | . | . | 11qA1 | rs26930939 | 1 | 0 |
| chr11 | 4044371 | T | C | 91 | Sec14l4 | intronic | . | . | . | 11qA1 | rs26930938 | 1 | 0 |
| chr11 | 4046181 | T | A | 228 | Sec14l4 | intronic | . | . | . | 11qA1 | rs221480582 | 1 | 0 |
| chr11 | 4046188 | C | G | 228 | Sec14l4 | intronic | . | . | . | 11qA1 | rs250188365 | 1 | 0 |
| chr11 | 4046322 | A | C | 228 | Sec14l4 | exonic | synonymous SNV | Sec14l4:NM_146013:exon12:c.A1191C:p.L397L | . | 11qA1 | rs26930933 | 1 | 0 |
| chr11 | 4065101 | G | A | 228 | Sec14l3 | intronic | . | . | . | 11qA1 | . | 1 | 0 |
| chr11 | 4065980 | T | C | 175 | Sec14l3 | intronic | . | . | . | 11qA1 | rs236485245 | 1 | 0 |
| chr11 | 4066048 | G | A | 228 | Sec14l3 | intronic | . | . | . | 11qA1 | . | 1 | 0 |
| chr11 | 4066373 | G | A | 228 | Sec14l3 | intronic | . | . | . | 11qA1 | . | 1 | 0 |
| chr11 | 4066461 | C | T | 228 | Sec14l3 | exonic | synonymous SNV | Sec14l3:NM_001029937:exon3:c.C132T:p.A44A | . | 11qA1 | rs265321405 | 1 | 0 |
| chr11 | 4066559 | C | G | 228 | Sec14l3 | intronic | . | . | . | 11qA1 | . | 1 | 0 |
| chr11 | 4067804 | T | C | 155 | Sec14l3 | intronic | . | . | . | 11qA1 | rs222068120 | 1 | 0 |
| chr11 | 4067883 | G | A | 220 | Sec14l3 | intronic | . | . | . | 11qA1 | rs218726856 | 1 | 0 |
| chr11 | 4067967 | G | A | 228 | Sec14l3 | exonic | synonymous SNV | Sec14l3:NM_001029937:exon5:c.G246A:p.K82K | . | 11qA1 | . | 1 | 0 |
| chr11 | 4069092 | G | A | 148 | Sec14l3 | intronic | . | . | . | 11qA1 | rs582270475 | 1 | 0 |
| chr11 | 4069866 | T | A | 160 | Sec14l3 | intronic | . | . | . | 11qA1 | rs213954865 | 1 | 0 |
| chr11 | 4070188 | G | A | 228 | Sec14l3 | intronic | . | . | . | 11qA1 | rs243215460 | 1 | 0 |
| chr11 | 4070242 | A | G | 228 | Sec14l3 | intronic | . | . | . | 11qA1 | rs235550584 | 1 | 0 |
| chr11 | 4071250 | C | T | 198 | Sec14l3 | intronic | . | . | . | 11qA1 | . | 1 | 0 |
| chr11 | 4071565 | T | G | 228 | Sec14l3 | intronic | . | . | . | 11qA1 | rs246728482 | 1 | 0 |
| chr11 | 4074499 | T | A | 120 | Sec14l3 | intronic | . | . | . | 11qA1 | rs258445157 | 1 | 0 |
| chr11 | 4074559 | A | C | 168 | Sec14l3 | intronic | . | . | . | 11qA1 | rs242758772 | 1 | 0 |
| chr11 | 4074607 | T | A | 174 | Sec14l3 | intronic | . | . | . | 11qA1 | rs236559407 | 1 | 0 |
| chr11 | 4075130 | G | A | 228 | Sec14l3 | intronic | . | . | . | 11qA1 | . | 1 | 0 |
| chr11 | 4075136 | C | T | 228 | Sec14l3 | intronic | . | . | . | 11qA1 | . | 1 | 0 |
| chr11 | 4075337 | A | G | 228 | Sec14l3 | intronic | . | . | . | 11qA1 | rs244519850 | 1 | 0 |
| chr11 | 4075493 | C | A | 228 | Sec14l3 | exonic | missense SNV | Sec14l3:NM_001029937:exon11:c.C1016A:p.T339N | . | 11qA1 | . | 1 | 0 |
| chr11 | 4076041 | G | A | 169 | Sec14l3 | intronic | . | . | . | 11qA1 | rs232582271 | 1 | 0 |
| chr11 | 4076424 | A | T | 228 | Sec14l3 | UTR3 | . | . | . | 11qA1 | rs243329315 | 1 | 0 |
| chr11 | 4092076 | A | G | 143 | Mtfp1 | UTR3 | . | . | . | 11qA1 | rs243936747 | 1 | 0 |
| chr11 | 4092080 | C | T | 143 | Mtfp1 | UTR3 | . | . | . | 11qA1 | rs234034295 | 1 | 0 |
| chr11 | 4092223 | A | G | 228 | Mtfp1 | UTR3 | . | . | . | 11qA1 | rs233862237 | 1 | 0 |
| chr11 | 4092338 | A | G | 228 | Mtfp1 | intronic | . | . | . | 11qA1 | rs26913738 | 1 | 0 |
| chr11 | 4092419 | C | T | 145 | Mtfp1 | intronic | . | . | . | 11qA1 | rs246156172 | 1 | 0 |
| chr11 | 4094068 | C | T | 228 | Mtfp1 | intronic | . | . | . | 11qA1 | . | 1 | 0 |
| chr11 | 4094156 | G | A | 209 | Mtfp1 | intronic | . | . | . | 11qA1 | . | 1 | 0 |
| chr11 | 4094659 | C | T | 153 | Mtfp1 | intronic | . | . | . | 11qA1 | rs26913730 | 1 | 0 |
| chr11 | 4098108 | C | T | 194 | Sec14l2 | UTR3 | . | . | . | 11qA1 | rs251450727 | 1 | 0 |
| chr11 | 4098422 | G | A | 228 | Sec14l2 | exonic | synonymous SNV | Sec14l2:NM_144520:exon12:c.C1095T:p.F365F | . | 11qA1 | rs26913724 | 1 | 0 |
| chr11 | 4103201 | T | C | 159 | Sec14l2 | intronic | . | . | . | 11qA1 | rs241201659 | 1 | 0 |
| chr11 | 4103237 | A | C | 169 | Sec14l2 | intronic | . | . | . | 11qA1 | rs262359356 | 1 | 0 |
| chr11 | 4103416 | T | C | 228 | Sec14l2 | exonic | synonymous SNV | Sec14l2:NM_144520:exon11:c.A1053G:p.G351G | . | 11qA1 | rs26913716 | 1 | 0 |
| chr11 | 4103597 | A | G | 228 | Sec14l2 | intronic | . | . | . | 11qA1 | rs232930097 | 1 | 0 |
| chr11 | 4103846 | A | G | 228 | Sec14l2 | intronic | . | . | . | 11qA1 | rs26913715 | 1 | 0 |
| chr11 | 4107884 | A | G | 228 | Sec14l2 | intronic | . | . | . | 11qA1 | rs26913706 | 1 | 0 |
| chr11 | 4107892 | G | A | 228 | Sec14l2 | intronic | . | . | . | 11qA1 | rs257246658 | 1 | 0 |
| chr11 | 4107952 | G | A | 228 | Sec14l2 | intronic | . | . | . | 11qA1 | rs26913705 | 1 | 0 |
| chr11 | 4108051 | G | C | 228 | Sec14l2 | intronic | . | . | . | 11qA1 | . | 1 | 0 |
| chr11 | 4108125 | A | G | 228 | Sec14l2 | intronic | . | . | . | 11qA1 | rs26913703 | 1 | 0 |
| chr11 | 4108413 | A | C | 228 | Sec14l2 | intronic | . | . | . | 11qA1 | . | 1 | 0 |
| chr11 | 4108454 | A | G | 228 | Sec14l2 | intronic | . | . | . | 11qA1 | rs242879646 | 1 | 0 |
| chr11 | 4108555 | A | G | 228 | Sec14l2 | exonic | synonymous SNV | Sec14l2:NM_144520:exon8:c.T606C:p.Y202Y | . | 11qA1 | rs26913702 | 1 | 0 |
| chr11 | 4108735 | A | C | 228 | Sec14l2 | intronic | . | . | . | 11qA1 | rs262401030 | 1 | 0 |
| chr11 | 4108899 | C | A | 228 | Sec14l2 | intronic | . | . | . | 11qA1 | rs26913701 | 1 | 0 |
| chr11 | 4109059 | A | G | 228 | Sec14l2 | intronic | . | . | . | 11qA1 | . | 1 | 0 |
| chr11 | 4109131 | C | G | 128 | Sec14l2 | intronic | . | . | . | 11qA1 | . | 0 | 1 |
| chr11 | 4109386 | C | T | 140 | Sec14l2 | intronic | . | . | . | 11qA1 | . | 1 | 0 |
| chr11 | 4110904 | A | C | 70 | Sec14l2 | intronic | . | . | . | 11qA1 | . | 1 | 0 |
| chr11 | 4110930 | A | C | 163 | Sec14l2 | intronic | . | . | . | 11qA1 | rs258697322 | 1 | 0 |
| chr11 | 4111012 | G | A | 228 | Sec14l2 | intronic | . | . | . | 11qA1 | rs244929818 | 1 | 0 |
| chr11 | 4111196 | T | C | 228 | Sec14l2 | exonic | synonymous SNV | Sec14l2:NM_144520:exon5:c.A264G:p.R88R | . | 11qA1 | . | 1 | 0 |
| chr11 | 4111304 | T | C | 228 | Sec14l2 | intronic | . | . | . | 11qA1 | rs247914787 | 1 | 0 |
| chr11 | 4111873 | G | A | 228 | Sec14l2 | intronic | . | . | . | 11qA1 | . | 1 | 0 |
| chr11 | 4111996 | T | C | 228 | Sec14l2 | intronic | . | . | . | 11qA1 | rs218314801 | 1 | 0 |
| chr11 | 4112055 | C | A | 228 | Sec14l2 | intronic | . | . | . | 11qA1 | rs252793480 | 1 | 0 |
| chr11 | 4112101 | T | C | 166 | Sec14l2 | intronic | . | . | . | 11qA1 | rs223003595 | 1 | 0 |
| chr11 | 4116608 | G | A | 228 | Sec14l2 | intronic | . | . | . | 11qA1 | . | 1 | 0 |
| chr11 | 4116793 | A | G | 228 | Sec14l2 | intronic | . | . | . | 11qA1 | rs224278649 | 1 | 0 |
| chr11 | 4116812 | A | G | 228 | Sec14l2 | intronic | . | . | . | 11qA1 | rs249240480 | 1 | 0 |
| chr11 | 4118563 | G | A | 177 | Sec14l2 | intronic | . | . | 378 | 11qA1 | . | 1 | 0 |
| chr11 | 4118608 | A | G | 228 | Sec14l2 | intronic | . | . | 378 | 11qA1 | rs229688204 | 1 | 0 |
| chr11 | 4118692 | T | C | 228 | Sec14l2 | UTR5 | . | . | 378 | 11qA1 | rs240172527 | 1 | 0 |
| chr11 | 4118732 | G | C | 228 | . | upstream | . | . | 378 | 11qA1 | . | 1 | 0 |
| chr11 | 4129370 | C | T | 228 | . | intergenic | . | . | . | 11qA1 | . | 1 | 0 |
| chr11 | 4130139 | C | G | 228 | . | intergenic | . | . | . | 11qA1 | rs214987000 | 1 | 0 |
| chr11 | 4136419 | C | T | 126 | Rnf215 | intronic | . | . | . | 11qA1 | rs214630701 | 1 | 0 |
| chr11 | 4136565 | T | C | 228 | Rnf215 | exonic | synonymous SNV | Rnf215:NM_027859:exon3:c.T441C:p.R147R | . | 11qA1 | rs221183445 | 1 | 0 |
| chr11 | 4136887 | A | G | 228 | Rnf215 | intronic | . | . | . | 11qA1 | rs265133275 | 1 | 0 |
| chr11 | 4137030 | A | G | 192 | Rnf215 | intronic | . | . | . | 11qA1 | . | 1 | 0 |
| chr11 | 4137407 | C | T | 115 | Rnf215 | intronic | . | . | . | 11qA1 | . | 1 | 0 |
| chr11 | 4139596 | C | T | 78 | Rnf215 | intronic | . | . | . | 11qA1 | rs242153514 | 1 | 0 |
| chr11 | 4139872 | G | T | 228 | Rnf215 | intronic | . | . | . | 11qA1 | rs29434003 | 1 | 0 |
| chr11 | 4140119 | C | T | 228 | Rnf215 | intronic | . | . | . | 11qA1 | . | 1 | 0 |
| chr11 | 4140259 | C | T | 228 | Rnf215 | intronic | . | . | . | 11qA1 | . | 1 | 0 |
| chr11 | 4140645 | C | A | 228 | Rnf215 | UTR3 | . | . | . | 11qA1 | rs254053542 | 1 | 0 |
| chr11 | 4143502 | C | T | 161 | Ccdc157 | UTR3 | . | . | . | 11qA1 | rs26913681 | 1 | 0 |
| chr11 | 4143569 | G | A | 167 | Ccdc157 | UTR3 | . | . | . | 11qA1 | rs29442283 | 1 | 0 |
| chr11 | 4143779 | A | G | 228 | Ccdc157 | exonic | synonymous SNV | Ccdc157:NM_001164620:exon12:c.T2067C:p.N689N,Ccdc157:NM_177616:exon13:c.T1761C:p.N587N | . | 11qA1 | rs29421380 | 1 | 0 |
| chr11 | 4143838 | C | T | 228 | Ccdc157 | exonic | missense SNV | Ccdc157:NM_001164620:exon12:c.G2008A:p.G670S,Ccdc157:NM_177616:exon13:c.G1702A:p.G568S | . | 11qA1 | rs26913680 | 1 | 0 |
| chr11 | 4144143 | G | A | 228 | Ccdc157 | intronic | . | . | . | 11qA1 | rs26913679 | 1 | 0 |
| chr11 | 4144750 | C | T | 228 | Ccdc157 | intronic | . | . | . | 11qA1 | . | 1 | 0 |
| chr11 | 4144752 | C | T | 228 | Ccdc157 | intronic | . | . | . | 11qA1 | . | 1 | 0 |
| chr11 | 4146246 | C | T | 102 | Ccdc157 | exonic | missense SNV | Ccdc157:NM_001164620:exon9:c.G1669A:p.A557T,Ccdc157:NM_177616:exon10:c.G1363A:p.A455T | . | 11qA1 | . | 1 | 0 |
| chr11 | 4146298 | C | T | 152 | Ccdc157 | exonic | synonymous SNV | Ccdc157:NM_001164620:exon9:c.G1617A:p.K539K,Ccdc157:NM_177616:exon10:c.G1311A:p.K437K | . | 11qA1 | rs26913672 | 1 | 0 |
| chr11 | 4146320 | C | T | 169 | Ccdc157 | exonic | missense SNV | Ccdc157:NM_001164620:exon9:c.G1595A:p.R532Q,Ccdc157:NM_177616:exon10:c.G1289A:p.R430Q | . | 11qA1 | . | 1 | 0 |
| chr11 | 4146736 | T | C | 228 | Ccdc157 | intronic | . | . | . | 11qA1 | rs26913671 | 1 | 0 |
| chr11 | 4147526 | G | C | 155 | Ccdc157 | intronic | . | . | . | 11qA1 | rs247392588 | 1 | 0 |
| chr11 | 4147749 | G | A | 228 | Ccdc157 | exonic | synonymous SNV | Ccdc157:NM_001164620:exon6:c.C1095T:p.A365A,Ccdc157:NM_177616:exon7:c.C789T:p.A263A | . | 11qA1 | rs26913667 | 1 | 0 |
| chr11 | 4148353 | T | C | 228 | Ccdc157 | intronic | . | . | . | 11qA1 | rs26913665 | 1 | 0 |
| chr11 | 4148458 | G | A | 228 | Ccdc157 | exonic | synonymous SNV | Ccdc157:NM_001164620:exon5:c.C1047T:p.D349D,Ccdc157:NM_177616:exon6:c.C741T:p.D247D | . | 11qA1 | . | 1 | 0 |
| chr11 | 4150136 | A | G | 207 | Ccdc157 | intronic | . | . | . | 11qA1 | . | 1 | 0 |
| chr11 | 4151788 | A | G | 228 | Ccdc157 | exonic | synonymous SNV | Ccdc157:NM_001164620:exon3:c.T132C:p.D44D | . | 11qA1 | rs251617218 | 1 | 0 |
| chr11 | 4151997 | A | G | 228 | Ccdc157 | intronic | . | . | . | 11qA1 | . | 1 | 0 |
| chr11 | 4166798 | C | T | 169 | Sf3a1 | intronic | . | . | . | 11qA1 | . | 1 | 0 |
| chr11 | 4167523 | A | C | 222 | Sf3a1 | intronic | . | . | . | 11qA1 | . | 1 | 0 |
| chr11 | 4170894 | A | T | 211 | Sf3a1 | intronic | . | . | . | 11qA1 | rs227923910 | 1 | 0 |
| chr11 | 4173004 | A | G | 228 | Sf3a1 | intronic | . | . | . | 11qA1 | rs211998776 | 1 | 0 |
| chr11 | 4173045 | C | A | 228 | Sf3a1 | intronic | . | . | . | 11qA1 | rs26913599 | 1 | 0 |
| chr11 | 4173395 | T | C | 170 | Sf3a1 | intronic | . | . | . | 11qA1 | rs26913596 | 1 | 0 |

| | | | | | | | | | | | | | |
|---|---|---|---|---|---|---|---|---|---|---|---|---|---|
| chr11 | 4174317 | C | T | 228 | Sf3a1 | intronic | . | . | . | 11qA1 | rs26913590 | 1 | 0 |
| chr11 | 4174764 | G | A | 85 | Sf3a1 | intronic | . | . | . | 11qA1 | rs226837665 | 1 | 0 |
| chr11 | 4175564 | T | C | 228 | Sf3a1 | exonic | synonymous SNV | Sf3a1:NM_026175:exon9:c.T1353C:p.D451D | . | 11qA1 | rs26913579 | 1 | 0 |
| chr11 | 4175627 | T | C | 228 | Sf3a1 | intronic | . | . | . | 11qA1 | rs26913578 | 1 | 0 |
| chr11 | 4175646 | A | G | 228 | Sf3a1 | intronic | . | . | . | 11qA1 | rs26913577 | 1 | 0 |
| chr11 | 4176055 | G | A | 151 | Sf3a1 | intronic | . | . | . | 11qA1 | rs26913575 | 1 | 0 |
| chr11 | 4176124 | C | T | 163 | Sf3a1 | intronic | . | . | . | 11qA1 | rs26913573 | 1 | 0 |
| chr11 | 4176181 | T | C | 228 | Sf3a1 | intronic | . | . | . | 11qA1 | rs26913571 | 1 | 0 |
| chr11 | 4176201 | A | G | 228 | Sf3a1 | intronic | . | . | . | 11qA1 | rs26913570 | 1 | 0 |
| chr11 | 4177403 | C | T | 188 | Sf3a1 | intronic | . | . | . | 11qA1 | . | 1 | 0 |
| chr11 | 4177404 | T | C | 195 | Sf3a1 | intronic | . | . | . | 11qA1 | rs253130315 | 1 | 0 |
| chr11 | 4177676 | A | G | 228 | Sf3a1 | intronic | . | . | . | 11qA1 | rs26913561 | 1 | 0 |
| chr11 | 4177731 | G | A | 168 | Sf3a1 | intronic | . | . | . | 11qA1 | rs47246315 | 1 | 0 |
| chr11 | 4177991 | G | A | 102 | Sf3a1 | intronic | . | . | . | 11qA1 | rs26913560 | 1 | 0 |
| chr11 | 4178406 | C | T | 134 | Sf3a1 | intronic | . | . | . | 11qA1 | rs46444220 | 1 | 0 |
| chr11 | 4178957 | C | T | 161 | Sf3a1 | intronic | . | . | . | 11qA1 | rs45944905 | 1 | 0 |
| chr11 | 4179206 | T | C | 228 | Sf3a1 | exonic | synonymous SNV | Sf3a1:NM_026175:exon14:c.T2166C:p.G722G | . | 11qA1 | rs233408049 | 1 | 0 |
| chr11 | 4179233 | C | T | 228 | Sf3a1 | exonic | synonymous SNV | Sf3a1:NM_026175:exon14:c.C2193T:p.L731L | . | 11qA1 | rs46112671 | 1 | 0 |
| chr11 | 4179324 | A | G | 228 | Sf3a1 | intronic | . | . | . | 11qA1 | rs214441956 | 1 | 0 |
| chr11 | 4179546 | G | C | 228 | Sf3a1 | intronic | . | . | . | 11qA1 | rs222649831 | 1 | 0 |
| chr11 | 4179554 | G | A | 228 | Sf3a1 | intronic | . | . | . | 11qA1 | rs252706841 | 1 | 0 |
| chr11 | 4179860 | A | G | 168 | Sf3a1 | intronic | . | . | . | 11qA1 | rs215481797 | 1 | 0 |
| chr11 | 4180324 | G | A | 197 | Sf3a1 | UTR3 | . | . | . | 11qA1 | rs231384906 | 1 | 0 |
| chr11 | 4186886 | C | T | 133 | Tbc1d10a | UTR5 | . | . | 561 | 11qA1 | rs238001216 | 1 | 0 |
| chr11 | 4186925 | A | C | 199 | Tbc1d10a | exonic | missense SNV | Tbc1d10a:NM_134023:exon1:c.A35C:p.E12A | 561 | 11qA1 | rs248746381 | 1 | 0 |
| chr11 | 4187095 | G | A | 228 | Tbc1d10a | exonic | missense SNV | Tbc1d10a:NM_134023:exon1:c.G205A:p.G69S | 561 | 11qA1 | rs261488503 | 1 | 0 |
| chr11 | 4187191 | T | C | 228 | Tbc1d10a | intronic | . | . | 561 | 11qA1 | rs262322014 | 1 | 0 |
| chr11 | 4187225 | A | T | 171 | Tbc1d10a | intronic | . | . | . | 11qA1 | rs256198093 | 1 | 0 |
| chr11 | 4205704 | G | C | 228 | Tbc1d10a | intronic | . | . | . | 11qA1 | rs238662488 | 1 | 0 |
| chr11 | 4209783 | T | C | 212 | Tbc1d10a | intronic | . | . | . | 11qA1 | rs229103131 | 1 | 0 |
| chr11 | 4212014 | A | G | 202 | Tbc1d10a | intronic | . | . | . | 11qA1 | rs240327389 | 1 | 0 |
| chr11 | 4212387 | C | G | 158 | Tbc1d10a | intronic | . | . | . | 11qA1 | rs234637440 | 1 | 0 |
| chr11 | 4212753 | T | C | 131 | Tbc1d10a | intronic | . | . | . | 11qA1 | rs255756109 | 1 | 0 |
| chr11 | 4212769 | C | T | 177 | Tbc1d10a | intronic | . | . | . | 11qA1 | . | 1 | 0 |
| chr11 | 4213165 | T | A | 207 | Tbc1d10a | intronic | . | . | . | 11qA1 | rs230688136 | 1 | 0 |
| chr11 | 4213180 | G | A | 164 | Tbc1d10a | intronic | . | . | . | 11qA1 | rs243685286 | 1 | 0 |
| chr11 | 4214668 | T | A | 228 | Tbc1d10a | intronic | . | . | . | 11qA1 | . | 1 | 0 |
| chr11 | 4214673 | T | G | 228 | Tbc1d10a | intronic | . | . | . | 11qA1 | . | 1 | 0 |
| chr11 | 4214801 | T | C | 228 | Tbc1d10a | exonic | missense SNV | Tbc1d10a:NM_134023:exon9:c.T1151C:p.L384P | . | 11qA1 | rs26913504 | 1 | 0 |
| chr11 | 4214970 | G | A | 228 | Tbc1d10a | exonic | synonymous SNV | Tbc1d10a:NM_134023:exon9:c.G1320A:p.A440A | . | 11qA1 | rs250766754 | 1 | 0 |
| chr11 | 4215044 | G | A | 228 | Tbc1d10a | exonic | missense SNV | Tbc1d10a:NM_134023:exon9:c.G1394A:p.G465D | . | 11qA1 | rs265740524 | 1 | 0 |
| chr11 | 4215190 | G | T | 228 | Tbc1d10a | UTR3 | . | . | . | 11qA1 | rs226960681 | 1 | 0 |
| chr11 | 4215245 | A | G | 163 | Tbc1d10a | UTR3 | . | . | . | 11qA1 | rs225920108 | 1 | 0 |
| chr11 | 4219092 | T | C | 228 | Gats13 | intronic | . | . | . | 11qA1 | rs260920295 | 1 | 0 |
| chr11 | 4220445 | G | C | 228 | Gats13 | exonic | missense SNV | Gats13:NM_028022:exon4:c.G440C:p.G147A | . | 11qA1 | . | 1 | 0 |
| chr11 | 4220823 | C | T | 228 | Gats13 | intronic | . | . | . | 11qA1 | . | 1 | 0 |
| chr11 | 4220863 | T | C | 228 | Gats13 | intronic | . | . | . | 11qA1 | rs246820468 | 1 | 0 |
| chr11 | 4220953 | G | A | 228 | Gats13 | intronic | . | . | . | 11qA1 | rs260308759 | 1 | 0 |
| chr11 | 4220993 | T | A | 202 | Gats13 | intronic | . | . | . | 11qA1 | . | 1 | 0 |
| chr11 | 4221113 | T | C | 228 | Gats13 | intronic | . | . | . | 11qA1 | rs230290372 | 1 | 0 |
| chr11 | 4221398 | T | G | 228 | Gats13 | intronic | . | . | . | 11qA1 | . | 1 | 0 |
| chr11 | 4221528 | G | T | 163 | Gats13 | intronic | . | . | . | 11qA1 | . | 1 | 0 |
| chr11 | 4221535 | C | G | 186 | Gats13 | intronic | . | . | . | 11qA1 | rs222125535 | 1 | 0 |
| chr11 | 4221789 | C | T | 228 | Gats13 | intronic | . | . | . | 11qA1 | rs238202887 | 1 | 0 |
| chr11 | 4221897 | A | G | 228 | Gats13 | exonic | missense SNV | Gats13:NM_028022:exon9:c.A973G:p.S325G | . | 11qA1 | rs254607614 | 1 | 0 |
| chr11 | 4221963 | C | T | 228 | Gats13 | UTR3 | . | . | . | 11qA1 | rs240610347 | 1 | 0 |
| chr11 | 4221970 | A | G | 228 | Gats13 | UTR3 | . | . | . | 11qA1 | rs108124091 | 1 | 0 |
| chr11 | 4230743 | G | A | 202 | . | intergenic | . | . | . | 11qA1 | rs236689227 | 1 | 0 |
| chr11 | 4231017 | T | C | 228 | . | intergenic | . | . | . | 11qA1 | rs26913474 | 1 | 0 |
| chr11 | 4231129 | G | A | 228 | . | intergenic | . | . | . | 11qA1 | rs26913473 | 1 | 0 |
| chr11 | 4231152 | C | T | 228 | . | intergenic | . | . | . | 11qA1 | . | 1 | 0 |
| chr11 | 4238240 | G | A | 162 | Osm | intronic | . | . | . | 11qA1 | . | 1 | 0 |
| chr11 | 4239448 | C | T | 228 | Osm | exonic | synonymous SNV | Osm:NM_001013365:exon3:c.C231T:p.A77A | . | 11qA1 | . | 1 | 0 |
| chr11 | 4239588 | A | G | 228 | Osm | exonic | missense SNV | Osm:NM_001013365:exon3:c.A371G:p.D124G | . | 11qA1 | . | 1 | 0 |
| chr11 | 4240016 | T | C | 228 | Osm | UTR3 | . | . | . | 11qA1 | rs238925561 | 1 | 0 |
| chr11 | 4268401 | G | A | 228 | Lif | intronic | . | . | . | 11qA1 | rs29437903 | 1 | 0 |
| chr11 | 4346426 | C | T | 194 | Hormad2 | UTR3 | . | . | . | 11qA1 | rs50200000 | 1 | 0 |
| chr11 | 4346643 | G | T | 228 | Hormad2 | exonic | missense SNV | Hormad2:NM_029458:exon11:c.C840A:p.H280Q | . | 11qA1 | rs26898799 | 1 | 0 |
| chr11 | 4409152 | G | A | 228 | Hormad2 | exonic | synonymous SNV | Hormad2:NM_029458:exon9:c.C417T:p.S139S | . | 11qA1 | rs26882809 | 1 | 0 |
| chr11 | 4409312 | T | C | 164 | Hormad2 | intronic | . | . | . | 11qA1 | rs26882807 | 1 | 0 |
| chr11 | 4420471 | C | T | 133 | Hormad2 | intronic | . | . | . | 11qA1 | rs26882786 | 1 | 0 |
| chr11 | 4420855 | A | G | 213 | Hormad2 | intronic | . | . | . | 11qA1 | rs26882785 | 1 | 0 |
| chr11 | 4421747 | C | T | 169 | Hormad2 | intronic | . | . | . | 11qA1 | rs26882783 | 1 | 0 |
| chr11 | 4435188 | T | G | 219 | Hormad2 | intronic | . | . | . | 11qA1 | rs257372031 | 1 | 0 |
| chr11 | 4482659 | A | G | 226 | Mtmr3 | UTR3 | . | . | . | 11qA1 | rs26882666 | 1 | 0 |
| chr11 | 4482967 | G | A | 228 | Mtmr3 | intronic | . | . | . | 11qA1 | rs29470514 | 1 | 0 |
| chr11 | 4485149 | T | C | 143 | Mtmr3 | intronic | . | . | . | 11qA1 | rs26882652 | 1 | 0 |
| chr11 | 4485303 | A | G | 228 | Mtmr3 | intronic | . | . | . | 11qA1 | rs26882650 | 1 | 0 |
| chr11 | 4487204 | A | G | 228 | Mtmr3 | intronic | . | . | . | 11qA1 | rs26882644 | 1 | 0 |
| chr11 | 4487489 | G | C | 228 | Mtmr3 | exonic | missense SNV | Mtmr3:NM_028860:exon16:c.C2964G:p.H988Q | . | 11qA1 | rs218968677 | 1 | 0 |
| chr11 | 4487501 | C | T | 228 | Mtmr3 | exonic | synonymous SNV | Mtmr3:NM_028860:exon16:c.G2952A:p.S984S | . | 11qA1 | rs26882641 | 1 | 0 |
| chr11 | 4487855 | T | C | 228 | Mtmr3 | exonic | synonymous SNV | Mtmr3:NM_028860:exon16:c.A2598G:p.P866P | . | 11qA1 | rs26882639 | 1 | 0 |
| chr11 | 4488441 | C | T | 228 | Mtmr3 | exonic | missense SNV | Mtmr3:NM_028860:exon16:c.G2012A:p.R671K | . | 11qA1 | rs26882637 | 1 | 0 |
| chr11 | 4488455 | C | A | 228 | Mtmr3 | exonic | synonymous SNV | Mtmr3:NM_028860:exon16:c.G1998T:p.L666L | . | 11qA1 | rs26882636 | 1 | 0 |
| chr11 | 4489500 | G | A | 224 | Mtmr3 | intronic | . | . | . | 11qA1 | rs26882631 | 1 | 0 |
| chr11 | 4489804 | T | A | 228 | Mtmr3 | intronic | . | . | . | 11qA1 | rs26882630 | 1 | 0 |
| chr11 | 4490964 | T | C | 228 | Mtmr3 | intronic | . | . | . | 11qA1 | rs26882621 | 1 | 0 |
| chr11 | 4491174 | G | C | 228 | Mtmr3 | intronic | . | . | . | 11qA1 | rs26882620 | 1 | 0 |
| chr11 | 4491280 | A | G | 209 | Mtmr3 | intronic | . | . | . | 11qA1 | rs26882619 | 1 | 0 |
| chr11 | 4491621 | A | G | 115 | Mtmr3 | intronic | . | . | . | 11qA1 | rs26882616 | 1 | 0 |
| chr11 | 4492006 | C | T | 228 | Mtmr3 | intronic | . | . | . | 11qA1 | rs26882614 | 1 | 0 |
| chr11 | 4492008 | G | A | 222 | Mtmr3 | intronic | . | . | . | 11qA1 | . | 0 | 1 |
| chr11 | 4492632 | G | A | 200 | Mtmr3 | intronic | . | . | . | 11qA1 | rs26882609 | 1 | 0 |
| chr11 | 4492683 | C | T | 228 | Mtmr3 | intronic | . | . | . | 11qA1 | rs224795156 | 1 | 0 |
| chr11 | 4493005 | C | T | 203 | Mtmr3 | intronic | . | . | . | 11qA1 | . | 1 | 0 |
| chr11 | 4493008 | G | C | 203 | Mtmr3 | intronic | . | . | . | 11qA1 | . | 1 | 0 |
| chr11 | 4497301 | C | A | 228 | Mtmr3 | intronic | . | . | . | 11qA1 | . | 1 | 0 |
| chr11 | 4497541 | A | T | 173 | Mtmr3 | intronic | . | . | . | 11qA1 | rs247785022 | 1 | 0 |
| chr11 | 4497684 | T | C | 196 | Mtmr3 | intronic | . | . | . | 11qA1 | rs218782856 | 1 | 0 |
| chr11 | 4497834 | A | G | 228 | Mtmr3 | intronic | . | . | . | 11qA1 | rs239514635 | 1 | 0 |
| chr11 | 4498132 | C | A | 228 | Mtmr3 | intronic | . | . | . | 11qA1 | rs51252098 | 1 | 0 |
| chr11 | 4498204 | T | A | 169 | Mtmr3 | intronic | . | . | . | 11qA1 | rs226796039 | 1 | 0 |
| chr11 | 4498782 | T | C | 179 | Mtmr3 | intronic | . | . | . | 11qA1 | rs231380206 | 1 | 0 |
| chr11 | 4498844 | G | A | 228 | Mtmr3 | intronic | . | . | . | 11qA1 | rs257813826 | 1 | 0 |
| chr11 | 4498859 | C | T | 228 | Mtmr3 | intronic | . | . | . | 11qA1 | rs213326516 | 1 | 0 |
| chr11 | 4503961 | G | A | 228 | Mtmr3 | intronic | . | . | . | 11qA1 | . | 1 | 0 |
| chr11 | 4503987 | A | G | 228 | Mtmr3 | intronic | . | . | . | 11qA1 | rs584666786 | 1 | 0 |
| chr11 | 4504248 | C | T | 216 | Mtmr3 | intronic | . | . | . | 11qA1 | . | 1 | 0 |
| chr11 | 4507334 | G | A | 155 | Mtmr3 | intronic | . | . | . | 11qA1 | rs26882601 | 1 | 0 |
| chr11 | 4507423 | G | A | 176 | Mtmr3 | intronic | . | . | . | 11qA1 | rs26882600 | 1 | 0 |
| chr11 | 4507470 | A | T | 213 | Mtmr3 | intronic | . | . | . | 11qA1 | rs218021473 | 1 | 0 |
| chr11 | 4507548 | C | G | 228 | Mtmr3 | intronic | . | . | . | 11qA1 | rs26882599 | 1 | 0 |
| chr11 | 4507832 | C | T | 101 | Mtmr3 | intronic | . | . | . | 11qA1 | . | 1 | 0 |
| chr11 | 4521055 | G | A | 169 | Mtmr3 | intronic | . | . | . | 11qA1 | rs26882571 | 1 | 0 |
| chr11 | 4527665 | G | A | 163 | Mtmr3 | intronic | . | . | . | 11qA1 | rs240549413 | 1 | 0 |
| chr11 | 4527756 | C | T | 228 | Mtmr3 | intronic | . | . | . | 11qA1 | rs252937256 | 1 | 0 |
| chr11 | 4527994 | A | G | 228 | Mtmr3 | intronic | . | . | . | 11qA1 | rs248899586 | 1 | 0 |
| chr11 | 4528000 | T | A | 228 | Mtmr3 | intronic | . | . | . | 11qA1 | rs262312680 | 1 | 0 |
| chr11 | 4528303 | C | G | 228 | Mtmr3 | intronic | . | . | . | 11qA1 | rs261320932 | 1 | 0 |
| chr11 | 4528437 | T | A | 228 | Mtmr3 | intronic | . | . | . | 11qA1 | rs232002355 | 1 | 0 |
| chr11 | 4530991 | C | T | 158 | Mtmr3 | intronic | . | . | . | 11qA1 | rs236280768 | 1 | 0 |
| chr11 | 4531182 | T | C | 228 | Mtmr3 | intronic | . | . | . | 11qA1 | . | 1 | 0 |
| chr11 | 4531418 | G | A | 167 | Mtmr3 | intronic | . | . | . | 11qA1 | . | 1 | 0 |
| chr11 | 4531466 | C | T | 138 | Mtmr3 | intronic | . | . | . | 11qA1 | rs223882518 | 1 | 0 |
| chr11 | 4564396 | A | T | 198 | Mtmr3 | intronic | . | . | . | 11qA1 | rs229202387 | 1 | 0 |
| chr11 | 4564519 | A | C | 228 | Mtmr3 | intronic | . | . | . | 11qA1 | rs259683143 | 1 | 0 |
| chr11 | 4564713 | G | A | 145 | Mtmr3 | intronic | . | . | . | 11qA1 | rs26923062 | 1 | 0 |
| chr11 | 4620254 | C | T | 193 | . | downstream | . | . | . | 11qA1 | . | 1 | 0 |
| chr11 | 4620267 | A | G | 161 | . | downstream | . | . | . | 11qA1 | rs26922990 | 1 | 0 |
| chr11 | 4626398 | A | G | 148 | Gm11961 | ncRNA_intronic | . | . | . | 11qA1 | rs26922967 | 1 | 0 |
| chr11 | 4626443 | G | A | 166 | Gm11961 | ncRNA_exonic | . | . | . | 11qA1 | rs26922966 | 1 | 0 |
| chr11 | 4626713 | C | T | 228 | Gm11961 | ncRNA_exonic | . | . | . | 11qA1 | . | 1 | 0 |
| chr11 | 4627674 | C | T | 228 | Gm11961 | ncRNA_intronic | . | . | . | 11qA1 | rs26922960 | 1 | 0 |
| chr11 | 4629887 | C | T | 228 | Gm11961 | ncRNA_intronic | . | . | . | 11qA1 | rs26922953 | 1 | 0 |
| chr11 | 4630207 | A | G | 137 | . | upstream | . | . | . | 11qA1 | . | 1 | 0 |
| chr11 | 4640583 | A | G | 228 | Ascc2 | intronic | . | . | . | 11qA1 | rs26922924 | 1 | 0 |
| chr11 | 4646379 | C | T | 171 | Ascc2 | intronic | . | . | . | 11qA1 | rs26922903 | 1 | 0 |
| chr11 | 4646882 | A | G | 228 | Ascc2 | intronic | . | . | . | 11qA1 | rs26922902 | 1 | 0 |
| chr11 | 4646968 | A | G | 228 | Ascc2 | intronic | . | . | . | 11qA1 | rs26922901 | 1 | 0 |
| chr11 | 4649605 | A | G | 80 | Ascc2 | intronic | . | . | . | 11qA1 | rs26922895 | 1 | 0 |
| chr11 | 4649692 | G | T | 192 | Ascc2 | intronic | . | . | . | 11qA1 | rs259163581 | 1 | 0 |
| chr11 | 4649905 | T | G | 228 | Ascc2 | intronic | . | . | . | 11qA1 | . | 1 | 0 |
| chr11 | 4656239 | G | C | 228 | Ascc2 | intronic | . | . | . | 11qA1 | rs224537355 | 1 | 0 |
| chr11 | 4656344 | C | T | 228 | Ascc2 | intronic | . | . | . | 11qA1 | . | 1 | 0 |
| chr11 | 4658097 | C | A | 200 | Ascc2 | intronic | . | . | . | 11qA1 | . | 1 | 0 |
| chr11 | 4658289 | G | A | 228 | Ascc2 | exonic | synonymous SNV | Ascc2:NM_029291:exon7:c.G684A:p.E228E | . | 11qA1 | rs234821787 | 1 | 0 |
| chr11 | 4658297 | C | G | 228 | Ascc2 | exonic | missense SNV | Ascc2:NM_029291:exon7:c.C692G:p.P231R | . | 11qA1 | rs258303860 | 1 | 0 |
| chr11 | 4658407 | G | A | 228 | Ascc2 | intronic | . | . | . | 11qA1 | . | 1 | 0 |
| chr11 | 4658476 | A | G | 138 | Ascc2 | intronic | . | . | . | 11qA1 | . | 1 | 0 |
| chr11 | 4659423 | C | T | 228 | Ascc2 | intronic | . | . | . | 11qA1 | . | 1 | 0 |
| chr11 | 4664095 | T | C | 219 | Ascc2 | intronic | . | . | . | 11qA1 | rs26922871 | 1 | 0 |
| chr11 | 4668269 | T | A | 228 | Ascc2 | intronic | . | . | . | 11qA1 | rs26922866 | 1 | 0 |
| chr11 | 4668468 | C | T | 228 | Ascc2 | intronic | . | . | . | 11qA1 | rs26922865 | 1 | 0 |
| chr11 | 4668509 | A | G | 228 | Ascc2 | intronic | . | . | . | 11qA1 | rs26922863 | 1 | 0 |
| chr11 | 4668529 | C | T | 228 | Ascc2 | intronic | . | . | . | 11qA1 | rs225706150 | 1 | 0 |
| chr11 | 4668925 | T | C | 228 | Ascc2 | exonic | synonymous SNV | Ascc2:NM_029291:exon12:c.T1149C:p.A383A | . | 11qA1 | rs257656127 | 1 | 0 |
| chr11 | 4668948 | C | T | 228 | Ascc2 | intronic | . | . | . | 11qA1 | rs26922860 | 1 | 0 |
| chr11 | 4670112 | A | G | 170 | Ascc2 | intronic | . | . | . | 11qA1 | rs26922850 | 1 | 0 |
| chr11 | 4670157 | T | C | 183 | Ascc2 | intronic | . | . | . | 11qA1 | rs26922849 | 1 | 0 |
| chr11 | 4670333 | T | G | 222 | Ascc2 | exonic | synonymous SNV | Ascc2:NM_029291:exon13:c.T1218G:p.V406V | . | 11qA1 | rs237322712 | 0 | 1 |
| chr11 | 4670427 | C | G | 228 | Ascc2 | exonic | missense SNV | Ascc2:NM_029291:exon13:c.C1312G:p.P438A | . | 11qA1 | rs26922847 | 1 | 0 |
| chr11 | 4672145 | A | G | 125 | Ascc2 | intronic | . | . | . | 11qA1 | rs247525804 | 1 | 0 |
| chr11 | 4672202 | C | T | 159 | Ascc2 | intronic | . | . | . | 11qA1 | rs212062680 | 1 | 0 |
| chr11 | 4672248 | G | T | 228 | Ascc2 | intronic | . | . | . | 11qA1 | . | 1 | 0 |
| chr11 | 4672315 | C | A | 228 | Ascc2 | exonic | synonymous SNV | Ascc2:NM_029291:exon14:c.C1380A:p.A460A | . | 11qA1 | rs236357260 | 1 | 0 |
| chr11 | 4672541 | C | T | 228 | Ascc2 | intronic | . | . | . | 11qA1 | . | 1 | 0 |
| chr11 | 4672608 | G | A | 216 | Ascc2 | intronic | . | . | . | 11qA1 | rs218944537 | 1 | 0 |
| chr11 | 4673118 | T | C | 160 | Ascc2 | intronic | . | . | . | 11qA1 | rs227483920 | 1 | 0 |
| chr11 | 4673139 | A | G | 164 | Ascc2 | intronic | . | . | . | 11qA1 | rs244950889 | 1 | 0 |
| chr11 | 4673323 | T | C | 228 | Ascc2 | intronic | . | . | . | 11qA1 | rs49161218 | 1 | 0 |
| chr11 | 4673525 | A | C | 228 | Ascc2 | intronic | . | . | . | 11qA1 | rs26922839 | 1 | 0 |
| chr11 | 4679370 | G | C | 228 | Ascc2 | intronic | . | . | . | 11qA1 | rs262162872 | 1 | 0 |
| chr11 | 4679588 | C | T | 228 | Ascc2 | intronic | . | . | . | 11qA1 | . | 1 | 0 |
| chr11 | 4679596 | G | A | 228 | Ascc2 | intronic | . | . | . | 11qA1 | . | 1 | 0 |
| chr11 | 4679959 | T | A | 180 | Ascc2 | intronic | . | . | . | 11qA1 | rs26922830 | 1 | 0 |
| chr11 | 4680332 | C | T | 179 | Ascc2 | intronic | . | . | . | 11qA1 | rs3682937 | 1 | 0 |
| chr11 | 4681365 | T | C | 228 | Ascc2 | intronic | . | . | . | 11qA1 | rs236418206 | 1 | 0 |
| chr11 | 4681372 | T | C | 209 | Ascc2 | intronic | . | . | . | 11qA1 | rs26922827 | 1 | 0 |
| chr11 | 4682900 | T | C | 228 | Ascc2 | intronic | . | . | . | 11qA1 | rs261430935 | 1 | 0 |
| chr11 | 4682934 | A | G | 228 | Ascc2 | exonic | synonymous SNV | Ascc2:NM_029291:exon20:c.A2106G:p.V702V | . | 11qA1 | rs253775160 | 1 | 0 |
| chr11 | 4683159 | G | A | 143 | Ascc2 | UTR3 | . | . | . | 11qA1 | rs26922824 | 1 | 0 |
| chr11 | 4685179 | C | A | 228 | . | intergenic | . | . | . | 11qA1 | rs258304657 | 1 | 0 |
| chr11 | 4685189 | A | G | 228 | . | intergenic | . | . | . | 11qA1 | rs29417086 | 1 | 0 |
| chr11 | 4685216 | A | G | 228 | . | intergenic | . | . | . | 11qA1 | rs29397291 | 1 | 0 |
| chr11 | 4685492 | C | T | 152 | . | intergenic | . | . | . | 11qA1 | rs217713590 | 1 | 0 |

| | | | | | | | | | | | | | |
|---|---|---|---|---|---|---|---|---|---|---|---|---|---|
| chr11 | 4703971 | C | T | 228 | Uqcr10 | intronic | . | . | . | 11qA1 | rs29424821 | 1 | 0 |
| chr11 | 4704112 | T | A | 228 | Uqcr10 | intronic | . | . | . | 11qA1 | . | 1 | 0 |
| chr11 | 4722474 | C | T | 228 | Zmat5 | exonic | synonymous SNV | Zmat5:NM_026015:exon2:c.C90T:p.H30H | . | 11qA1 | rs26922771 | 1 | 0 |
| chr11 | 4728400 | T | G | 160 | Zmat5 | intronic | . | . | . | 11qA1 | rs29438817 | 1 | 0 |
| chr11 | 4728697 | C | T | 228 | Zmat5 | intronic | . | . | . | 11qA1 | rs26902044 | 1 | 0 |
| chr11 | 4728741 | T | G | 228 | Zmat5 | intronic | . | . | . | 11qA1 | rs225024606 | 1 | 0 |
| chr11 | 4728775 | G | T | 195 | Zmat5 | intronic | . | . | . | 11qA1 | rs26902043 | 1 | 0 |
| chr11 | 4730435 | A | G | 204 | Zmat5 | intronic | . | . | . | 11qA1 | rs26902039 | 1 | 0 |
| chr11 | 4731938 | A | G | 127 | Zmat5 | intronic | . | . | . | 11qA1 | rs48448336 | 1 | 0 |
| chr11 | 4732095 | A | G | 228 | Zmat5 | exonic | synonymous SNV | Zmat5:NM_026015:exon5:c.A300G:p.L100L | . | 11qA1 | rs50635022 | 1 | 0 |
| chr11 | 4732209 | A | G | 228 | Zmat5 | intronic | . | . | . | 11qA1 | . | 1 | 0 |
| chr11 | 4732285 | C | T | 190 | Zmat5 | intronic | . | . | . | 11qA1 | . | 1 | 0 |
| chr11 | 4738904 | G | A | 228 | Cabp7 | exonic | synonymous SNV | Cabp7:NM_138948:exon5:c.C565T:p.L189L | . | 11qA1 | rs241308382 | 1 | 0 |
| chr11 | 4739396 | A | C | 221 | Cabp7 | intronic | . | . | . | 11qA1 | . | 1 | 0 |
| chr11 | 4739682 | G | A | 228 | Cabp7 | intronic | . | . | . | 11qA1 | . | 1 | 0 |
| chr11 | 4740753 | G | A | 115 | Cabp7 | intronic | . | . | . | 11qA1 | . | 1 | 0 |
| chr11 | 4768322 | G | A | 228 | Nf2 | intronic | . | . | . | 11qA1 | . | 1 | 0 |
| chr11 | 4768342 | A | C | 228 | Nf2 | intronic | . | . | . | 11qA1 | rs26901939 | 1 | 0 |
| chr11 | 4781999 | G | T | 158 | Nf2 | intronic | . | . | . | 11qA1 | rs238718578 | 1 | 0 |
| chr11 | 4782334 | C | T | 228 | Nf2 | exonic | synonymous SNV | Nf2:NM_001252250:exon15:c.G1593A:p.K531K,Nf2:NM_001252251:exon15:c.G1593A:p.K531K,Nf2:NM_0012 | . | 11qA1 | rs26901894 | 1 | 0 |
| chr11 | 4784418 | G | C | 228 | Nf2 | intronic | . | . | . | 11qA1 | rs255072197 | 1 | 0 |
| chr11 | 4784489 | A | G | 228 | Nf2 | exonic | synonymous SNV | Nf2:NM_001252250:exon14:c.T1521C:p.F507F,Nf2:NM_001252251:exon14:c.T1521C:p.F507F,Nf2:NM_0012 | . | 11qA1 | rs29393599 | 1 | 0 |
| chr11 | 4787273 | A | C | 101 | Nf2 | intronic | . | . | . | 11qA1 | rs26901867 | 1 | 0 |
| chr11 | 4787389 | C | T | 228 | Nf2 | intronic | . | . | . | 11qA1 | rs26901866 | 1 | 0 |
| chr11 | 4787506 | T | G | 228 | Nf2 | exonic | synonymous SNV | Nf2:NM_001252250:exon13:c.A1384C:p.R462R,Nf2:NM_001252251:exon13:c.A1384C:p.R462R,Nf2:NM_0012 | . | 11qA1 | rs6377755 | 1 | 0 |
| chr11 | 4787657 | A | G | 151 | Nf2 | intronic | . | . | . | 11qA1 | rs6378790 | 1 | 0 |
| chr11 | 4789813 | A | G | 228 | Nf2 | exonic | synonymous SNV | Nf2:NM_001252250:exon12:c.T1192C:p.L398L,Nf2:NM_001252251:exon12:c.T1192C:p.L398L,Nf2:NM_0012 | . | 11qA1 | rs45925478 | 1 | 0 |
| chr11 | 4790891 | A | G | 159 | Nf2 | intronic | . | . | . | 11qA1 | rs26901858 | 1 | 0 |
| chr11 | 4791069 | A | G | 228 | Nf2 | intronic | . | . | . | 11qA1 | rs26901857 | 1 | 0 |
| chr11 | 4791220 | A | G | 228 | Nf2 | intronic | . | . | . | 11qA1 | rs26901856 | 1 | 0 |
| chr11 | 4794549 | G | C | 228 | Nf2 | intronic | . | . | . | 11qA1 | rs26901842 | 1 | 0 |
| chr11 | 4794590 | G | A | 228 | Nf2 | intronic | . | . | . | 11qA1 | rs216361759 | 1 | 0 |
| chr11 | 4795097 | T | C | 127 | Nf2 | intronic | . | . | . | 11qA1 | rs26901839 | 1 | 0 |
| chr11 | 4799711 | C | A | 175 | Nf2 | intronic | . | . | . | 11qA1 | rs6222729 | 1 | 0 |
| chr11 | 4799741 | G | A | 194 | Nf2 | intronic | . | . | . | 11qA1 | rs6235960 | 1 | 0 |
| chr11 | 4803395 | G | A | 102 | Nf2 | intronic | . | . | . | 11qA1 | rs26901824 | 1 | 0 |
| chr11 | 4803477 | A | C | 201 | Nf2 | intronic | . | . | . | 11qA1 | rs225013981 | 1 | 0 |
| chr11 | 4803552 | G | A | 228 | Nf2 | intronic | . | . | . | 11qA1 | rs26901823 | 1 | 0 |
| chr11 | 4803626 | C | T | 228 | Nf2 | intronic | . | . | . | 11qA1 | rs262116611 | 1 | 0 |
| chr11 | 4803802 | G | C | 228 | Nf2 | intronic | . | . | . | 11qA1 | rs26901821 | 1 | 0 |
| chr11 | 4806160 | T | C | 228 | Nf2 | intronic | . | . | . | 11qA1 | rs26901813 | 1 | 0 |
| chr11 | 4808197 | G | C | 228 | Nf2 | intronic | . | . | . | 11qA1 | rs26901805 | 1 | 0 |
| chr11 | 4816350 | G | A | 161 | Nf2 | intronic | . | . | . | 11qA1 | rs226844927 | 1 | 0 |
| chr11 | 4816364 | G | A | 150 | Nf2 | intronic | . | . | . | 11qA1 | rs253292896 | 1 | 0 |
| chr11 | 4818252 | G | C | 160 | Nf2 | intronic | . | . | . | 11qA1 | rs26901773 | 1 | 0 |
| chr11 | 4818681 | A | C | 228 | Nf2 | intronic | . | . | . | 11qA1 | rs26901771 | 1 | 0 |
| chr11 | 4818764 | G | C | 228 | Nf2 | intronic | . | . | . | 11qA1 | rs26901769 | 1 | 0 |
| chr11 | 4848757 | C | G | 198 | Nf2 | intronic | . | . | . | 11qA1 | rs238772437 | 1 | 0 |
| chr11 | 4848801 | T | C | 228 | Nf2 | intronic | . | . | 991 | 11qA1 | rs244261856 | 1 | 0 |
| chr11 | 4848992 | A | G | 228 | Nf2 | UTR5 | . | . | 991 | 11qA1 | rs29386533 | 1 | 0 |
| chr11 | 4874069 | C | T | 228 | Nipsnap1 | exonic | missense SNV | Nipsnap1:NM_008698:exon1:c.C64T:p.R22C | . | 11qA1 | rs255795389 | 1 | 0 |
| chr11 | 4882978 | T | A | 228 | Nipsnap1 | intronic | . | . | . | 11qA1 | rs26886059 | 1 | 0 |
| chr11 | 4883055 | A | G | 228 | Nipsnap1 | intronic | . | . | . | 11qA1 | rs26886058 | 1 | 0 |
| chr11 | 4883114 | T | C | 228 | Nipsnap1 | intronic | . | . | . | 11qA1 | rs29388430 | 1 | 0 |
| chr11 | 4883158 | A | G | 228 | Nipsnap1 | intronic | . | . | . | 11qA1 | . | 1 | 0 |
| chr11 | 4883218 | A | G | 228 | Nipsnap1 | intronic | . | . | . | 11qA1 | . | 1 | 0 |
| chr11 | 4883325 | T | A | 222 | Nipsnap1 | intronic | . | . | . | 11qA1 | . | 0 | 1 |
| chr11 | 4883623 | A | G | 228 | Nipsnap1 | intronic | . | . | . | 11qA1 | . | 1 | 0 |
| chr11 | 4884130 | A | G | 228 | Nipsnap1 | intronic | . | . | . | 11qA1 | rs26886057 | 1 | 0 |
| chr11 | 4884170 | A | C | 167 | Nipsnap1 | intronic | . | . | . | 11qA1 | rs253082037 | 1 | 0 |
| chr11 | 4888795 | G | A | 228 | Nipsnap1 | intronic | . | . | . | 11qA1 | rs241093417 | 1 | 0 |
| chr11 | 4888982 | C | G | 228 | Nipsnap1 | intronic | . | . | . | 11qA1 | . | 1 | 0 |
| chr11 | 4889047 | C | T | 228 | Nipsnap1 | intronic | . | . | . | 11qA1 | . | 1 | 0 |
| chr11 | 4889259 | T | C | 228 | Nipsnap1 | intronic | . | . | . | 11qA1 | rs29428906 | 1 | 0 |
| chr11 | 4889514 | C | A | 228 | Nipsnap1 | intronic | . | . | . | 11qA1 | . | 1 | 0 |
| chr11 | 4889724 | A | G | 228 | Nipsnap1 | intronic | . | . | . | 11qA1 | rs234195328 | 1 | 0 |
| chr11 | 4889776 | T | C | 228 | Nipsnap1 | intronic | . | . | . | 11qA1 | rs253765120 | 1 | 0 |
| chr11 | 4889934 | G | C | 228 | Nipsnap1 | exonic | synonymous SNV | Nipsnap1:NM_008698:exon8:c.G654C:p.V218V | . | 11qA1 | rs236595917 | 1 | 0 |
| chr11 | 4891343 | A | G | 228 | Nipsnap1 | intronic | . | . | . | 11qA1 | rs26886052 | 1 | 0 |
| chr11 | 4891518 | G | T | 228 | Nipsnap1 | intronic | . | . | . | 11qA1 | rs108045112 | 1 | 0 |
| chr11 | 4891526 | T | C | 228 | Nipsnap1 | intronic | . | . | . | 11qA1 | rs108077346 | 1 | 0 |
| chr11 | 4899932 | G | C | 228 | Thoc5 | intronic | . | . | . | 11qA1 | rs26886025 | 1 | 0 |
| chr11 | 4899956 | C | G | 228 | Thoc5 | intronic | . | . | . | 11qA1 | rs29478164 | 1 | 0 |
| chr11 | 4900096 | C | T | 141 | Thoc5 | intronic | . | . | . | 11qA1 | rs26886023 | 1 | 0 |
| chr11 | 4900125 | A | G | 108 | Thoc5 | intronic | . | . | . | 11qA1 | rs26886022 | 1 | 0 |
| chr11 | 4901071 | T | C | 228 | Thoc5 | intronic | . | . | . | 11qA1 | rs26886015 | 1 | 0 |
| chr11 | 4901175 | G | A | 228 | Thoc5 | exonic | synonymous SNV | Thoc5:NM_172438:exon3:c.G120A:p.E40E | . | 11qA1 | rs26886014 | 1 | 0 |
| chr11 | 4901293 | C | G | 228 | Thoc5 | exonic | missense SNV | Thoc5:NM_172438:exon3:c.C238G:p.L80V | . | 11qA1 | rs13464073 | 1 | 0 |
| chr11 | 4901367 | A | G | 228 | Thoc5 | intronic | . | . | . | 11qA1 | rs29447671 | 1 | 0 |
| chr11 | 4901419 | A | G | 206 | Thoc5 | intronic | . | . | . | 11qA1 | . | 1 | 0 |
| chr11 | 4902049 | A | G | 228 | Thoc5 | intronic | . | . | . | 11qA1 | rs26886010 | 1 | 0 |
| chr11 | 4902430 | C | T | 228 | Thoc5 | exonic | synonymous SNV | Thoc5:NM_172438:exon5:c.C450T:p.F150F | . | 11qA1 | rs26886008 | 1 | 0 |
| chr11 | 4902464 | G | T | 228 | Thoc5 | intronic | . | . | . | 11qA1 | . | 1 | 0 |
| chr11 | 4903972 | T | C | 188 | Thoc5 | intronic | . | . | . | 11qA1 | rs26886000 | 1 | 0 |
| chr11 | 4905585 | G | T | 228 | Thoc5 | intronic | . | . | . | 11qA1 | rs26885997 | 1 | 0 |
| chr11 | 4905839 | G | A | 211 | Thoc5 | intronic | . | . | . | 11qA1 | rs238486125 | 1 | 0 |
| chr11 | 4910662 | A | G | 228 | Thoc5 | exonic | synonymous SNV | Thoc5:NM_172438:exon8:c.A750G:p.P250P | . | 11qA1 | rs26885988 | 1 | 0 |
| chr11 | 4911389 | T | G | 228 | Thoc5 | intronic | . | . | . | 11qA1 | rs250079636 | 1 | 0 |
| chr11 | 4911392 | C | T | 228 | Thoc5 | intronic | . | . | . | 11qA1 | rs225760166 | 1 | 0 |
| chr11 | 4911577 | C | T | 228 | Thoc5 | intronic | . | . | . | 11qA1 | rs26885985 | 1 | 0 |
| chr11 | 4911680 | A | T | 115 | Thoc5 | intronic | . | . | . | 11qA1 | rs26885984 | 1 | 0 |
| chr11 | 4912512 | C | T | 106 | Thoc5 | intronic | . | . | . | 11qA1 | rs26885980 | 1 | 0 |
| chr11 | 4912618 | A | T | 174 | Thoc5 | intronic | . | . | . | 11qA1 | rs214648254 | 1 | 0 |
| chr11 | 4912659 | C | T | 202 | Thoc5 | intronic | . | . | . | 11qA1 | rs26885979 | 1 | 0 |
| chr11 | 4912714 | T | C | 228 | Thoc5 | intronic | . | . | . | 11qA1 | rs217624092 | 1 | 0 |
| chr11 | 4914023 | A | G | 166 | Thoc5 | intronic | . | . | . | 11qA1 | rs250458558 | 1 | 0 |
| chr11 | 4914530 | G | T | 228 | Thoc5 | exonic | synonymous SNV | Thoc5:NM_172438:exon11:c.G975T:p.R325R | . | 11qA1 | rs258705493 | 1 | 0 |
| chr11 | 4914632 | A | G | 228 | Thoc5 | intronic | . | . | . | 11qA1 | rs26885977 | 1 | 0 |
| chr11 | 4915432 | C | G | 228 | Thoc5 | intronic | . | . | . | 11qA1 | rs220585754 | 1 | 0 |
| chr11 | 4918068 | T | A | 228 | Thoc5 | intronic | . | . | . | 11qA1 | rs26885960 | 1 | 0 |
| chr11 | 4918069 | G | A | 228 | Thoc5 | intronic | . | . | . | 11qA1 | rs234468150 | 1 | 0 |
| chr11 | 4918270 | T | G | 228 | Thoc5 | intronic | . | . | . | 11qA1 | rs585412769 | 1 | 0 |
| chr11 | 4918304 | A | G | 223 | Thoc5 | intronic | . | . | . | 11qA1 | rs261904439 | 1 | 0 |
| chr11 | 4918415 | G | T | 98 | Thoc5 | intronic | . | . | . | 11qA1 | rs26885958 | 1 | 0 |
| chr11 | 4919609 | T | C | 177 | Thoc5 | intronic | . | . | . | 11qA1 | . | 1 | 0 |
| chr11 | 4919865 | C | T | 228 | Thoc5 | intronic | . | . | . | 11qA1 | rs26885949 | 1 | 0 |
| chr11 | 4920200 | C | T | 140 | Thoc5 | intronic | . | . | . | 11qA1 | rs237354503 | 1 | 0 |
| chr11 | 4920205 | A | T | 146 | Thoc5 | intronic | . | . | . | 11qA1 | rs249885915 | 1 | 0 |
| chr11 | 4920307 | G | A | 228 | Thoc5 | intronic | . | . | . | 11qA1 | rs26885944 | 1 | 0 |
| chr11 | 4920373 | C | T | 228 | Thoc5 | splicing | . | . | . | 11qA1 | rs26885943 | 1 | 0 |
| chr11 | 4920633 | G | A | 168 | Thoc5 | intronic | . | . | . | 11qA1 | rs228257631 | 1 | 0 |
| chr11 | 4920684 | G | C | 127 | Thoc5 | intronic | . | . | . | 11qA1 | rs26885941 | 1 | 0 |
| chr11 | 4921701 | A | G | 171 | Thoc5 | intronic | . | . | . | 11qA1 | rs26885936 | 1 | 0 |
| chr11 | 4921767 | C | T | 186 | Thoc5 | intronic | . | . | . | 11qA1 | rs26885935 | 1 | 0 |
| chr11 | 4922008 | A | G | 228 | Thoc5 | exonic | missense SNV | Thoc5:NM_172438:exon16:c.A1576G:p.T526A | . | 11qA1 | rs26885932 | 1 | 0 |
| chr11 | 4922295 | A | G | 228 | Thoc5 | intronic | . | . | . | 11qA1 | rs26885930 | 1 | 0 |
| chr11 | 4924505 | A | G | 145 | Thoc5 | intronic | . | . | . | 11qA1 | rs26885922 | 1 | 0 |
| chr11 | 4924627 | A | G | 228 | Thoc5 | intronic | . | . | . | 11qA1 | rs26885921 | 1 | 0 |
| chr11 | 4924848 | C | T | 228 | Thoc5 | exonic | synonymous SNV | Thoc5:NM_172438:exon18:c.C1782T:p.N594N | . | 11qA1 | rs26885920 | 1 | 0 |
| chr11 | 4925890 | C | T | 110 | Thoc5 | intronic | . | . | . | 11qA1 | rs26885916 | 1 | 0 |
| chr11 | 4928434 | A | G | 152 | Thoc5 | intronic | . | . | . | 11qA1 | rs26885908 | 1 | 0 |
| chr11 | 4939253 | G | T | 202 | Nefh | UTR3 | . | . | . | 11qA1 | rs230927350 | 1 | 0 |
| chr11 | 4939399 | T | A | 228 | Nefh | exonic | missense SNV | Nefh:NM_010904:exon4:c.A3219T:p.E1073D | . | 11qA1 | rs239690638 | 1 | 0 |
| chr11 | 4940002 | T | C | 228 | Nefh | exonic | synonymous SNV | Nefh:NM_010904:exon4:c.A2616G:p.E872E | . | 11qA1 | rs225323237 | 1 | 0 |
| chr11 | 4940151 | G | A | 228 | Nefh | exonic | missense SNV | Nefh:NM_010904:exon4:c.C2467T:p.P823S | . | 11qA1 | rs212425084 | 1 | 0 |
| chr11 | 4940287 | G | C | 228 | Nefh | exonic | synonymous SNV | Nefh:NM_010904:exon4:c.C2331G:p.V777V | . | 11qA1 | rs237644415 | 1 | 0 |
| chr11 | 4940305 | C | G | 228 | Nefh | exonic | missense SNV | Nefh:NM_010904:exon4:c.G2313C:p.E771D | . | 11qA1 | rs214134975 | 1 | 0 |
| chr11 | 4940752 | C | T | 228 | Nefh | exonic | synonymous SNV | Nefh:NM_010904:exon4:c.G1866A:p.E622E | . | 11qA1 | rs258246971 | 1 | 0 |
| chr11 | 4940774 | G | C | 139 | Nefh | exonic | missense SNV | Nefh:NM_010904:exon4:c.C1844G:p.A615G | . | 11qA1 | rs219456007 | 1 | 0 |
| chr11 | 4941071 | C | G | 154 | Nefh | exonic | missense SNV | Nefh:NM_010904:exon4:c.G1547C:p.R516P | . | 11qA1 | rs264560184 | 1 | 0 |
| chr11 | 4941316 | T | A | 228 | Nefh | exonic | synonymous SNV | Nefh:NM_010904:exon4:c.A1302T:p.I434I | . | 11qA1 | rs233940277 | 1 | 0 |
| chr11 | 4944016 | A | T | 228 | Nefh | intronic | . | . | . | 11qA1 | rs29451147 | 1 | 0 |
| chr11 | 4944070 | G | A | 228 | Nefh | intronic | . | . | . | 11qA1 | . | 1 | 0 |
| chr11 | 4945380 | A | G | 228 | Nefh | intronic | . | . | . | 11qA1 | . | 1 | 0 |
| chr11 | 4947856 | A | C | 144 | Ap1b1,Nefh | exonic | missense SNV | Nefh:NM_010904:exon1:c.T79G:p.S27A | 1245 | 11qA1 | rs228331256 | 1 | 0 |
| chr11 | 4947869 | G | A | 135 | Ap1b1,Nefh | exonic | synonymous SNV | Nefh:NM_010904:exon1:c.C66T:p.G22G | 1245 | 11qA1 | rs243716521 | 1 | 0 |
| chr11 | 4947959 | C | T | 163 | Ap1b1,Nefh | UTR5 | . | . | 1245 | 11qA1 | . | 1 | 0 |
| chr11 | 5009474 | A | G | 228 | Ap1b1 | intronic | . | . | . | 11qA1 | rs26916980 | 1 | 0 |
| chr11 | 5009786 | A | G | 156 | Ap1b1 | intronic | . | . | . | 11qA1 | . | 1 | 0 |
| chr11 | 5012796 | G | A | 175 | Ap1b1 | intronic | . | . | . | 11qA1 | rs26916970 | 1 | 0 |
| chr11 | 5012816 | G | A | 188 | Ap1b1 | intronic | . | . | . | 11qA1 | rs45649057 | 1 | 0 |
| chr11 | 5015780 | A | G | 228 | Ap1b1 | intronic | . | . | . | 11qA1 | rs239525745 | 1 | 0 |
| chr11 | 5015846 | A | T | 228 | Ap1b1 | intronic | . | . | . | 11qA1 | rs26916959 | 1 | 0 |
| chr11 | 5015875 | A | G | 228 | Ap1b1 | intronic | . | . | . | 11qA1 | rs26916958 | 1 | 0 |
| chr11 | 5016564 | C | T | 228 | Ap1b1 | intronic | . | . | . | 11qA1 | . | 1 | 0 |
| chr11 | 5016599 | T | C | 228 | Ap1b1 | intronic | . | . | . | 11qA1 | rs3163914 | 1 | 0 |
| chr11 | 5016935 | A | G | 228 | Ap1b1 | intronic | . | . | . | 11qA1 | rs3165897 | 1 | 0 |
| chr11 | 5017839 | G | T | 153 | Ap1b1 | intronic | . | . | . | 11qA1 | . | 1 | 0 |
| chr11 | 5017842 | C | T | 155 | Ap1b1 | intronic | . | . | . | 11qA1 | . | 1 | 0 |
| chr11 | 5018226 | G | A | 228 | Ap1b1 | intronic | . | . | . | 11qA1 | rs217935766 | 1 | 0 |
| chr11 | 5018258 | G | A | 228 | Ap1b1 | intronic | . | . | . | 11qA1 | rs236281221 | 1 | 0 |
| chr11 | 5019177 | C | A | 173 | Ap1b1 | intronic | . | . | . | 11qA1 | rs4228554 | 1 | 0 |
| chr11 | 5019519 | A | C | 228 | Ap1b1 | intronic | . | . | . | 11qA1 | . | 1 | 0 |
| chr11 | 5019540 | C | T | 228 | Ap1b1 | intronic | . | . | . | 11qA1 | . | 1 | 0 |
| chr11 | 5022991 | G | T | 228 | Ap1b1 | intronic | . | . | . | 11qA1 | rs226487687 | 1 | 0 |
| chr11 | 5023016 | T | C | 228 | Ap1b1 | intronic | . | . | . | 11qA1 | rs245695956 | 1 | 0 |
| chr11 | 5023046 | A | G | 228 | Ap1b1 | intronic | . | . | . | 11qA1 | rs213861641 | 1 | 0 |
| chr11 | 5023311 | T | C | 228 | Ap1b1 | intronic | . | . | . | 11qA1 | . | 1 | 0 |
| chr11 | 5023362 | T | G | 170 | Ap1b1 | intronic | . | . | . | 11qA1 | . | 1 | 0 |
| chr11 | 5024242 | T | C | 206 | Ap1b1 | intronic | . | . | . | 11qA1 | . | 1 | 0 |
| chr11 | 5025493 | T | C | 228 | Ap1b1 | intronic | . | . | . | 11qA1 | rs6204700 | 1 | 0 |
| chr11 | 5026220 | G | A | 228 | Ap1b1 | intronic | . | . | . | 11qA1 | rs260946476 | 1 | 0 |
| chr11 | 5026287 | T | G | 228 | Ap1b1 | exonic | synonymous SNV | Ap1b1:NM_001243043:exon11:c.T1314G:p.S438S,Ap1b1:NM_001243044:exon11:c.T1314G:p.S438S,Ap1b1:N | . | 11qA1 | rs13465190 | 1 | 0 |
| chr11 | 5026483 | A | G | 228 | Ap1b1 | intronic | . | . | . | 11qA1 | rs234510782 | 1 | 0 |
| chr11 | 5026495 | C | T | 228 | Ap1b1 | intronic | . | . | . | 11qA1 | rs26916948 | 1 | 0 |
| chr11 | 5030873 | A | G | 228 | Ap1b1 | intronic | . | . | . | 11qA1 | rs26916937 | 1 | 0 |
| chr11 | 5031618 | T | C | 228 | Ap1b1 | intronic | . | . | . | 11qA1 | rs229895904 | 1 | 0 |
| chr11 | 5031622 | C | T | 228 | Ap1b1 | intronic | . | . | . | 11qA1 | . | 1 | 0 |
| chr11 | 5031981 | G | A | 228 | Ap1b1 | intronic | . | . | . | 11qA1 | rs26916931 | 1 | 0 |
| chr11 | 5032027 | A | G | 228 | Ap1b1 | intronic | . | . | . | 11qA1 | rs26916930 | 1 | 0 |
| chr11 | 5032379 | G | T | 228 | Ap1b1 | intronic | . | . | . | 11qA1 | . | 1 | 0 |
| chr11 | 5032706 | G | A | 101 | Ap1b1 | intronic | . | . | . | 11qA1 | rs26916927 | 1 | 0 |
| chr11 | 5033280 | G | C | 228 | Ap1b1 | intronic | . | . | . | 11qA1 | rs26916923 | 1 | 0 |
| chr11 | 5033438 | G | C | 228 | Ap1b1 | intronic | . | . | . | 11qA1 | rs26916922 | 1 | 0 |
| chr11 | 5033661 | A | G | 228 | Ap1b1 | intronic | . | . | . | 11qA1 | rs26916921 | 1 | 0 |
| chr11 | 5033728 | C | G | 228 | Ap1b1 | exonic | synonymous SNV | Ap1b1:NM_001243044:exon15:c.C2040G:p.A680A,Ap1b1:NM_007454:exon15:c.C2040G:p.A680A,Ap1b1:NM_( | . | 11qA1 | rs3165834 | 1 | 0 |
| chr11 | 5033917 | A | C | 228 | Ap1b1 | intronic | . | . | . | 11qA1 | . | 1 | 0 |
| chr11 | 5033925 | C | T | 228 | Ap1b1 | intronic | . | . | . | 11qA1 | . | 1 | 0 |
| chr11 | 5037585 | C | G | 228 | Ap1b1 | intronic | . | . | . | 11qA1 | rs26916900 | 1 | 0 |
| chr11 | 5037597 | G | A | 228 | Ap1b1 | intronic | . | . | . | 11qA1 | rs236880166 | 1 | 0 |
| chr11 | 5039167 | A | G | 228 | Ap1b1 | exonic | synonymous SNV | Ap1b1:NM_001243044:exon17:c.A2343G:p.P781P,Ap1b1:NM_007454:exon17:c.A2343G:p.P781P,Ap1b1:NM_( | . | 11qA1 | rs3163876 | 1 | 0 |
| chr11 | 5039383 | G | A | 228 | Ap1b1 | intronic | . | . | . | 11qA1 | . | 1 | 0 |
| chr11 | 5039398 | T | A | 228 | Ap1b1 | intronic | . | . | . | 11qA1 | rs236219044 | 1 | 0 |
| chr11 | 5039506 | T | C | 228 | Ap1b1 | intronic | . | . | . | 11qA1 | rs3165823 | 1 | 0 |
| chr11 | 5040047 | T | G | 228 | Ap1b1 | intronic | . | . | . | 11qA1 | rs258321778 | 1 | 0 |

| | | | | | | | | | | | | | |
|---|---|---|---|---|---|---|---|---|---|---|---|---|---|
| chr11 | 5040246 | C | T | 228 | Ap1b1 | intronic | . | . | . | 11qA1 | . | 1 | 0 |
| chr11 | 5040820 | C | A | 228 | Ap1b1 | intronic | . | . | . | 11qA1 | . | 1 | 0 |
| chr11 | 5056609 | G | A | 228 | Gas2l1 | UTR3 | . | . | . | 11qA1 | rs228002298 | 1 | 0 |
| chr11 | 5056684 | G | A | 228 | Gas2l1 | exonic | missense SNV | Gas2l1:NM_030228:exon7:c.C1016T:p.P339L | . | 11qA1 | rs265277227 | 1 | 0 |
| chr11 | 5058353 | T | C | 228 | Gas2l1,Rasl10a | exonic | synonymous SNV | Rasl10a:NM_145216:exon1:c.T48C:p.G16G | 590 | 11qA1 | rs257370131 | 1 | 0 |
| chr11 | 5059282 | T | A | 176 | Gas2l1,Rasl10a | intronic | . | . | . | 11qA1 | rs3163862 | 1 | 0 |
| chr11 | 5059327 | G | A | 228 | Gas2l1,Rasl10a | intronic | . | . | . | 11qA1 | . | 1 | 0 |
| chr11 | 5059414 | C | A | 228 | Rasl10a,Gas2l1 | intronic | . | . | . | 11qA1 | . | 1 | 0 |
| chr11 | 5061534 | T | G | 153 | Gas2l1 | exonic | synonymous SNV | Gas2l1:NM_001190408:exon6:c.A1294C:p.R432R,Gas2l1:NM_144560:exon7:c.A1294C:p.R432R | 935 | 11qA1 | rs216005529 | 1 | 0 |
| chr11 | 5061561 | C | T | 177 | Gas2l1 | exonic | missense SNV | Gas2l1:NM_001190408:exon6:c.G1267A:p.A423T,Gas2l1:NM_144560:exon7:c.G1267A:p.A423T | 935 | 11qA1 | . | 1 | 0 |
| chr11 | 5062096 | A | C | 155 | Gas2l1 | intronic | . | . | 935 | 11qA1 | . | 1 | 0 |
| chr11 | 5062253 | G | A | 228 | Gas2l1 | exonic | missense SNV | Gas2l1:NM_001190406:exon4:c.C815T:p.P272L | 935 | 11qA1 | . | 1 | 0 |
| chr11 | 5063832 | A | G | 188 | Gas2l1 | exonic | synonymous SNV | Gas2l1:NM_001190406:exon2:c.T627C:p.D209D,Gas2l1:NM_001190408:exon2:c.T627C:p.D209D,Gas2l1:NM | . | 11qA1 | rs221182980 | 1 | 0 |
| chr11 | 5063937 | A | G | 228 | Gas2l1 | exonic | synonymous SNV | Gas2l1:NM_001190406:exon2:c.T522C:p.V174V,Gas2l1:NM_001190408:exon2:c.T522C:p.V174V,Gas2l1:NM | . | 11qA1 | rs241803802 | 1 | 0 |
| chr11 | 5064258 | G | A | 228 | Gas2l1 | exonic | synonymous SNV | Gas2l1:NM_001190406:exon2:c.C201T:p.N67N,Gas2l1:NM_001190408:exon2:c.C201T:p.N67N,Gas2l1:NM_( | 306 | 11qA1 | . | 1 | 0 |
| chr11 | 5064288 | G | C | 228 | Gas2l1 | exonic | synonymous SNV | Gas2l1:NM_001190406:exon2:c.C171G:p.A57A,Gas2l1:NM_001190408:exon2:c.C171G:p.A57A,Gas2l1:NM_( | . | 11qA1 | . | 1 | 0 |
| chr11 | 5064620 | G | A | 206 | Gas2l1 | intronic | . | . | . | 11qA1 | . | 1 | 0 |
| chr11 | 5070157 | G | C | 228 | Ewsr1 | intronic | . | . | . | 11qA1 | rs3165809 | 1 | 0 |
| chr11 | 5070585 | A | G | 228 | Ewsr1 | intronic | . | . | . | 11qA1 | rs26916886 | 1 | 0 |
| chr11 | 5071091 | C | T | 228 | Ewsr1 | exonic | synonymous SNV | Ewsr1:NM_001283062:exon12:c.G1371A:p.G457G,Ewsr1:NM_001283061:exon14:c.G1485A:p.G495G,Ewsr1:N | . | 11qA1 | rs13460529 | 1 | 0 |
| chr11 | 5071193 | C | T | 228 | Ewsr1 | intronic | . | . | . | 11qA1 | . | 1 | 0 |
| chr11 | 5071490 | G | C | 228 | Ewsr1 | intronic | . | . | . | 11qA1 | rs3163851 | 1 | 0 |
| chr11 | 5071685 | A | C | 228 | Ewsr1 | intronic | . | . | . | 11qA1 | rs227286097 | 1 | 0 |
| chr11 | 5071689 | C | T | 228 | Ewsr1 | intronic | . | . | . | 11qA1 | rs242798715 | 1 | 0 |
| chr11 | 5072560 | A | G | 110 | Ewsr1 | intronic | . | . | . | 11qA1 | rs26916885 | 1 | 0 |
| chr11 | 5082516 | T | C | 228 | Ewsr1 | intronic | . | . | . | 11qA1 | rs3165799 | 1 | 0 |
| chr11 | 5082575 | G | A | 115 | Ewsr1 | intronic | . | . | . | 11qA1 | rs26916873 | 1 | 0 |
| chr11 | 5083487 | T | C | 228 | Ewsr1 | exonic | synonymous SNV | Ewsr1:NM_001283061:exon7:c.A666G:p.Q222Q,Ewsr1:NM_001283062:exon7:c.A666G:p.Q222Q,Ewsr1:NM_0( | . | 11qA1 | rs3163843 | 1 | 0 |
| chr11 | 5087805 | G | A | 146 | Ewsr1 | intronic | . | . | . | 11qA1 | rs217982929 | 1 | 0 |
| chr11 | 5087820 | G | A | 153 | Ewsr1 | intronic | . | . | . | 11qA1 | rs3165795 | 1 | 0 |
| chr11 | 5087925 | C | T | 228 | Ewsr1 | intronic | . | . | . | 11qA1 | rs26916862 | 1 | 0 |
| chr11 | 5087955 | C | A | 201 | Ewsr1 | intronic | . | . | . | 11qA1 | . | 0 | 1 |
| chr11 | 5088084 | T | C | 228 | Ewsr1 | exonic | missense SNV | Ewsr1:NM_001283061:exon5:c.A289G:p.T97A,Ewsr1:NM_001283062:exon5:c.A289G:p.T97A,Ewsr1:NM_0012 | . | 11qA1 | rs26916861 | 1 | 0 |
| chr11 | 5088124 | C | G | 228 | Ewsr1 | exonic | synonymous SNV | Ewsr1:NM_001283061:exon5:c.G249C:p.P83P,Ewsr1:NM_001283062:exon5:c.G249C:p.P83P,Ewsr1:NM_0012 | . | 11qA1 | rs244480083 | 1 | 0 |
| chr11 | 5088299 | G | A | 121 | Ewsr1 | intronic | . | . | . | 11qA1 | rs3165793 | 1 | 0 |
| chr11 | 5091686 | T | A | 228 | Ewsr1 | intronic | . | . | . | 11qA1 | rs26916855 | 1 | 0 |
| chr11 | 5099473 | T | C | 228 | Rhbdd3 | UTR5 | . | . | 1236 | 11qA1 | rs245182621 | 1 | 0 |
| chr11 | 5099840 | C | G | 228 | Rhbdd3 | UTR5 | . | . | . | 11qA1 | rs26916849 | 1 | 0 |
| chr11 | 5099952 | A | G | 228 | Rhbdd3 | intronic | . | . | . | 11qA1 | rs26916848 | 1 | 0 |
| chr11 | 5100005 | G | A | 226 | Rhbdd3 | intronic | . | . | . | 11qA1 | rs263658075 | 1 | 0 |
| chr11 | 5103282 | T | C | 228 | Rhbdd3 | exonic | synonymous SNV | Rhbdd3:NM_001290493:exon4:c.T81C:p.C27C,Rhbdd3:NM_001290491:exon5:c.T81C:p.C27C,Rhbdd3:NM_001 | . | 11qA1 | rs252026635 | 1 | 0 |
| chr11 | 5103363 | C | G | 228 | Rhbdd3 | intronic | . | . | . | 11qA1 | rs26916838 | 1 | 0 |
| chr11 | 5103382 | T | C | 228 | Rhbdd3 | intronic | . | . | . | 11qA1 | rs26916837 | 1 | 0 |
| chr11 | 5103439 | G | A | 228 | Rhbdd3 | intronic | . | . | . | 11qA1 | rs26916836 | 1 | 0 |
| chr11 | 5103915 | G | A | 107 | Rhbdd3 | intronic | . | . | . | 11qA1 | rs3165774 | 1 | 0 |
| chr11 | 5104143 | C | T | 151 | Rhbdd3 | exonic | missense SNV | Rhbdd3:NM_001290493:exon5:c.C271T:p.H91Y,Rhbdd3:NM_001290491:exon6:c.C271T:p.H91Y,Rhbdd3:NM_( | . | 11qA1 | rs26916833 | 1 | 0 |
| chr11 | 5104154 | T | C | 168 | Rhbdd3 | exonic | synonymous SNV | Rhbdd3:NM_001290493:exon5:c.T282C:p.T94T,Rhbdd3:NM_001290491:exon6:c.T282C:p.T94T,Rhbdd3:NM_( | . | 11qA1 | rs26916832 | 1 | 0 |
| chr11 | 5104262 | T | G | 228 | Rhbdd3 | exonic | synonymous SNV | Rhbdd3:NM_001290493:exon5:c.T390G:p.A130A,Rhbdd3:NM_001290491:exon6:c.T390G:p.A130A,Rhbdd3:NM | . | 11qA1 | rs3163832 | 1 | 0 |
| chr11 | 5104364 | C | T | 228 | Rhbdd3 | exonic | synonymous SNV | Rhbdd3:NM_001290493:exon5:c.C492T:p.P164P,Rhbdd3:NM_001290491:exon6:c.C492T:p.P164P,Rhbdd3:NM | . | 11qA1 | rs26916831 | 1 | 0 |
| chr11 | 5105035 | C | T | 126 | Rhbdd3 | intronic | . | . | . | 11qA1 | rs26916828 | 1 | 0 |
| chr11 | 5105282 | G | A | 228 | Rhbdd3 | exonic | missense SNV | Rhbdd3:NM_001290491:exon7:c.G634A:p.A212T | . | 11qA1 | rs26916826 | 1 | 0 |
| chr11 | 5105742 | A | G | 228 | Rhbdd3 | intronic | . | . | . | 11qA1 | rs241197627 | 1 | 0 |
| chr11 | 5105903 | C | A | 228 | Rhbdd3 | exonic | missense SNV | Rhbdd3:NM_001290493:exon8:c.C1084A:p.L362I,Rhbdd3:NM_001290491:exon9:c.C1054A:p.L352I,Rhbdd3: | . | 11qA1 | rs232869727 | 1 | 0 |
| chr11 | 5107029 | T | A | 228 | Emid1 | intronic | . | . | . | 11qA1 | . | 1 | 0 |
| chr11 | 5107088 | C | T | 228 | Emid1 | intronic | . | . | . | 11qA1 | rs240276516 | 1 | 0 |
| chr11 | 5109525 | C | T | 130 | Emid1 | intronic | . | . | . | 11qA1 | . | 1 | 0 |
| chr11 | 5110523 | T | C | 170 | Emid1 | intronic | . | . | . | 11qA1 | rs262825856 | 1 | 0 |
| chr11 | 5110878 | A | G | 211 | Emid1 | intronic | . | . | . | 11qA1 | rs259258325 | 1 | 0 |
| chr11 | 5128884 | A | C | 228 | Emid1 | exonic | missense SNV | Emid1:NM_080595:exon12:c.T1063G:p.L355V | . | 11qA1 | rs212666530 | 1 | 0 |
| chr11 | 5128962 | T | C | 228 | Emid1 | intronic | . | . | . | 11qA1 | rs3165918 | 1 | 0 |
| chr11 | 5129061 | C | T | 195 | Emid1 | exonic | missense SNV | Emid1:NM_080595:exon11:c.G980A:p.G327D | . | 11qA1 | . | 0 | 1 |
| chr11 | 5129172 | T | C | 228 | Emid1 | intronic | . | . | . | 11qA1 | rs6332284 | 1 | 0 |
| chr11 | 5129204 | G | T | 193 | Emid1 | intronic | . | . | . | 11qA1 | . | 1 | 0 |
| chr11 | 5129381 | A | G | 228 | Emid1 | intronic | . | . | . | 11qA1 | rs6333353 | 1 | 0 |
| chr11 | 5129456 | A | G | 228 | Emid1 | exonic | missense SNV | Emid1:NM_080595:exon10:c.T941C:p.M314T | . | 11qA1 | rs6333470 | 1 | 0 |
| chr11 | 5130594 | G | A | 228 | Emid1 | intronic | . | . | . | 11qA1 | . | 1 | 0 |
| chr11 | 5130677 | G | A | 228 | Emid1 | exonic | missense SNV | Emid1:NM_080595:exon8:c.C772T:p.P258S | . | 11qA1 | rs13461673 | 1 | 0 |
| chr11 | 5130811 | A | G | 228 | Emid1 | intronic | . | . | . | 11qA1 | rs249836140 | 1 | 0 |
| chr11 | 5131774 | T | C | 196 | Emid1 | intronic | . | . | . | 11qA1 | rs232700127 | 1 | 0 |
| chr11 | 5131796 | T | C | 202 | Emid1 | intronic | . | . | . | 11qA1 | rs26916822 | 1 | 0 |
| chr11 | 5132136 | T | C | 228 | Emid1 | intronic | . | . | . | 11qA1 | rs29425424 | 1 | 0 |
| chr11 | 5132243 | G | A | 115 | Emid1 | intronic | . | . | . | 11qA1 | rs26916821 | 1 | 0 |
| chr11 | 5133900 | C | T | 156 | Emid1 | intronic | . | . | . | 11qA1 | rs257600699 | 1 | 0 |
| chr11 | 5134118 | G | A | 228 | Emid1 | intronic | . | . | . | 11qA1 | . | 1 | 0 |
| chr11 | 5134491 | A | C | 228 | Emid1 | intronic | . | . | . | 11qA1 | rs227562740 | 1 | 0 |
| chr11 | 5135289 | T | C | 126 | Emid1 | intronic | . | . | . | 11qA1 | rs241409221 | 1 | 0 |
| chr11 | 5135343 | G | A | 183 | Emid1 | intronic | . | . | . | 11qA1 | rs26916817 | 1 | 0 |
| chr11 | 5143757 | G | A | 197 | Emid1 | intronic | . | . | . | 11qA1 | . | 1 | 0 |
| chr11 | 5143813 | G | A | 228 | Emid1 | intronic | . | . | . | 11qA1 | . | 1 | 0 |
| chr11 | 5143900 | T | C | 228 | Emid1 | exonic | synonymous SNV | Emid1:NM_080595:exon3:c.A246G:p.K82K | . | 11qA1 | . | 1 | 0 |
| chr11 | 5144166 | G | A | 171 | Emid1 | intronic | . | . | . | 11qA1 | . | 1 | 0 |
| chr11 | 5152008 | C | T | 56 | Emid1 | intronic | . | . | 575 | 11qA1 | . | 1 | 0 |
| chr11 | 5194860 | T | A | 228 | Kremen1 | UTR3 | . | . | . | 11qA1 | rs218597592 | 1 | 0 |
| chr11 | 5195061 | T | C | 228 | Kremen1 | exonic | synonymous SNV | Kremen1:NM_032396:exon9:c.A1314G:p.G438G | . | 11qA1 | rs265828678 | 1 | 0 |
| chr11 | 5195105 | C | T | 228 | Kremen1 | exonic | missense SNV | Kremen1:NM_032396:exon9:c.G1270A:p.A424T | . | 11qA1 | rs232559254 | 1 | 0 |
| chr11 | 5198129 | T | C | 135 | Kremen1 | intronic | . | . | . | 11qA1 | rs26903854 | 1 | 0 |
| chr11 | 5199686 | C | T | 228 | Kremen1 | exonic | synonymous SNV | Kremen1:NM_032396:exon7:c.G984A:p.E328E | . | 11qA1 | rs242228156 | 1 | 0 |
| chr11 | 5201616 | C | A | 228 | Kremen1 | intronic | . | . | . | 11qA1 | rs229727420 | 1 | 0 |
| chr11 | 5201665 | C | T | 228 | Kremen1 | intronic | . | . | . | 11qA1 | rs249690774 | 1 | 0 |
| chr11 | 5202089 | C | T | 228 | Kremen1 | intronic | . | . | . | 11qA1 | . | 1 | 0 |
| chr11 | 5209875 | G | A | 146 | Kremen1 | intronic | . | . | . | 11qA1 | . | 1 | 0 |
| chr11 | 5209915 | A | G | 228 | Kremen1 | intronic | . | . | . | 11qA1 | rs257546651 | 1 | 0 |
| chr11 | 5210149 | T | C | 228 | Kremen1 | intronic | . | . | . | 11qA1 | rs26903831 | 1 | 0 |
| chr11 | 5210211 | A | G | 227 | Kremen1 | intronic | . | . | . | 11qA1 | rs236326299 | 1 | 0 |
| chr11 | 5210258 | C | T | 150 | Kremen1 | intronic | . | . | . | 11qA1 | rs6165557 | 1 | 0 |
| chr11 | 5215210 | A | C | 153 | Kremen1 | intronic | . | . | . | 11qA1 | . | 1 | 0 |
| chr11 | 5215238 | C | T | 165 | Kremen1 | intronic | . | . | . | 11qA1 | rs26903808 | 1 | 0 |
| chr11 | 5215279 | C | T | 90 | Kremen1 | intronic | . | . | . | 11qA1 | . | 1 | 0 |
| chr11 | 5238532 | C | G | 228 | Kremen1 | intronic | . | . | . | 11qA1 | rs26903736 | 1 | 0 |
| chr11 | 5243718 | C | T | 228 | Kremen1 | intronic | . | . | . | 11qA1 | rs26903730 | 1 | 0 |
| chr11 | 5243991 | C | T | 228 | Kremen1 | intronic | . | . | . | 11qA1 | rs26903729 | 1 | 0 |
| chr11 | 5281282 | A | C | 228 | Znrf3 | exonic | missense SNV | Znrf3:NM_001080924:exon8:c.T2230G:p.S744A,Znrf3:NM_001290501:exon9:c.T1942G:p.S648A | . | 11qA1 | rs259409042 | 1 | 0 |
| chr11 | 5281389 | C | G | 228 | Znrf3 | exonic | missense SNV | Znrf3:NM_001080924:exon8:c.G2123C:p.S708T,Znrf3:NM_001290501:exon9:c.G1835C:p.S612T | . | 11qA1 | rs26903661 | 1 | 0 |
| chr11 | 5282756 | T | A | 115 | Znrf3 | intronic | . | . | . | 11qA1 | rs26903658 | 1 | 0 |
| chr11 | 5338914 | A | C | 181 | Znrf3 | intronic | . | . | . | 11qA1 | rs29468023 | 1 | 0 |
| chr11 | 5462754 | T | C | 126 | . | intergenic | . | . | . | 11qA1 | rs26885214 | 1 | 0 |
| chr11 | 5523317 | A | C | 228 | Xbp1 | intronic | . | . | . | 11qA1 | rs29460761 | 1 | 0 |
| chr11 | 5534147 | T | G | 228 | Ccdc117 | intronic | . | . | . | 11qA1 | rs29410171 | 1 | 0 |
| chr11 | 5534172 | T | C | 93 | Ccdc117 | intronic | . | . | . | 11qA1 | rs29463408 | 1 | 0 |
| chr11 | 5534233 | G | A | 228 | Ccdc117 | intronic | . | . | . | 11qA1 | rs29469833 | 1 | 0 |
| chr11 | 5534672 | A | G | 201 | Ccdc117 | intronic | . | . | . | 11qA1 | rs29418340 | 1 | 0 |
| chr11 | 5564236 | C | T | 228 | . | intergenic | . | . | . | 11qA1 | rs215470299 | 1 | 0 |
| chr11 | 5569790 | G | A | 164 | Ankrd36 | UTR5 | . | . | . | 11qA1 | rs26885131 | 1 | 0 |
| chr11 | 5570848 | G | A | 115 | Ankrd36 | intronic | . | . | . | 11qA1 | rs26885129 | 1 | 0 |
| chr11 | 5574191 | G | T | 228 | Ankrd36 | intronic | . | . | . | 11qA1 | rs29477914 | 1 | 0 |
| chr11 | 5596752 | A | T | 228 | Ankrd36 | exonic | missense SNV | Ankrd36:NM_023816:exon8:c.A932T:p.E311V | . | 11qA1 | rs50244059 | 1 | 0 |
| chr11 | 5607418 | T | C | 228 | Ankrd36 | exonic | missense SNV | Ankrd36:NM_023816:exon10:c.T1178C:p.L393P | . | 11qA1 | rs48033339 | 1 | 0 |
| chr11 | 5607459 | G | T | 228 | Ankrd36 | intronic | . | . | . | 11qA1 | rs26885075 | 1 | 0 |
| chr11 | 5609483 | G | A | 228 | Ankrd36 | intronic | . | . | . | 11qA1 | rs26885070 | 1 | 0 |
| chr11 | 5614729 | A | G | 228 | Ankrd36 | intronic | . | . | . | 11qA1 | rs26885064 | 1 | 0 |
| chr11 | 5615561 | G | T | 228 | Ankrd36 | exonic | missense SNV | Ankrd36:NM_023816:exon13:c.G1394T:p.C465F | . | 11qA1 | rs26885063 | 1 | 0 |
| chr11 | 5618657 | G | C | 165 | Ankrd36 | intronic | . | . | . | 11qA1 | rs26885058 | 1 | 0 |
| chr11 | 5632209 | G | A | 198 | Ankrd36 | exonic | synonymous SNV | Ankrd36:NM_023816:exon18:c.G2763A:p.S921S | . | 11qA1 | . | 0 | 1 |
| chr11 | 5634122 | G | A | 228 | Ankrd36 | intronic | . | . | . | 11qA1 | rs225515589 | 1 | 0 |
| chr11 | 5634123 | A | C | 228 | Ankrd36 | intronic | . | . | . | 11qA1 | rs26885037 | 1 | 0 |
| chr11 | 5634843 | C | T | 127 | Ankrd36 | intronic | . | . | . | 11qA1 | rs26885035 | 1 | 0 |
| chr11 | 5635233 | C | T | 228 | Ankrd36 | exonic | missense SNV | Ankrd36:NM_023816:exon19:c.C2969T:p.A990V | . | 11qA1 | rs26885034 | 1 | 0 |
| chr11 | 5635263 | G | C | 228 | Ankrd36 | exonic | missense SNV | Ankrd36:NM_023816:exon19:c.G2999C:p.R1000T | . | 11qA1 | rs26885033 | 1 | 0 |
| chr11 | 5639213 | G | T | 228 | Ankrd36 | intronic | . | . | . | 11qA1 | rs583778232 | 1 | 0 |
| chr11 | 5639217 | G | T | 228 | Ankrd36 | intronic | . | . | . | 11qA1 | rs586699397 | 1 | 0 |
| chr11 | 5643984 | C | T | 211 | Ankrd36 | intronic | . | . | . | 11qA1 | rs249527163 | 1 | 0 |
| chr11 | 5643993 | G | A | 206 | Ankrd36 | intronic | . | . | . | 11qA1 | rs218741089 | 1 | 0 |
| chr11 | 5644723 | A | G | 164 | Ankrd36 | intronic | . | . | . | 11qA1 | rs240072689 | 1 | 0 |
| chr11 | 5644737 | A | G | 209 | Ankrd36 | intronic | . | . | . | 11qA1 | rs213716496 | 1 | 0 |
| chr11 | 5644759 | G | C | 228 | Ankrd36 | intronic | . | . | . | 11qA1 | rs233119876 | 1 | 0 |
| chr11 | 5646987 | C | T | 228 | Ankrd36 | intronic | . | . | . | 11qA1 | rs214270079 | 1 | 0 |
| chr11 | 5647051 | C | G | 228 | Ankrd36 | intronic | . | . | . | 11qA1 | rs580439800 | 1 | 0 |
| chr11 | 5678342 | C | G | 94 | Ankrd36 | intronic | . | . | . | 11qA1 | rs224983191 | 1 | 0 |
| chr11 | 5689187 | T | C | 228 | Ankrd36 | exonic | synonymous SNV | Ankrd36:NM_023816:exon27:c.T4098C:p.N1366N | . | 11qA1 | rs46048832 | 1 | 0 |
| chr11 | 5707547 | G | A | 228 | Mrps24 | exonic | synonymous SNV | Mrps24:NM_026080:exon2:c.C48T:p.A16A | 492 | 11qA1 | rs13470708 | 1 | 0 |
| chr11 | 5715669 | C | T | 228 | Urgcp | UTR3 | . | . | . | 11qA1 | rs13460445 | 1 | 0 |
| chr11 | 5715680 | A | G | 228 | Urgcp | UTR3 | . | . | . | 11qA1 | rs13460443 | 1 | 0 |
| chr11 | 5715901 | G | A | 228 | Urgcp | exonic | synonymous SNV | Urgcp:NM_001077661:exon6:c.C2436T:p.G812G,Urgcp:NM_178623:exon6:c.C2565T:p.G855G | . | 11qA1 | rs13460448 | 1 | 0 |
| chr11 | 5717551 | G | T | 228 | Urgcp | exonic | synonymous SNV | Urgcp:NM_001077661:exon6:c.C786A:p.R262R,Urgcp:NM_178623:exon6:c.C915A:p.R305R | . | 11qA1 | rs13460447 | 1 | 0 |
| chr11 | 5718231 | G | T | 204 | Urgcp | exonic | missense SNV | Urgcp:NM_001077661:exon6:c.C106A:p.L36M,Urgcp:NM_178623:exon6:c.C235A:p.L79M | . | 11qA1 | rs230468447 | 1 | 0 |
| chr11 | 5722830 | G | A | 228 | Urgcp | exonic | synonymous SNV | Urgcp:NM_001077661:exon5:c.C45T:p.N15N,Urgcp:NM_178623:exon5:c.C174T:p.N58N | . | 11qA1 | rs26885001 | 1 | 0 |
| chr11 | 5722965 | A | C | 228 | Urgcp | intronic | . | . | . | 11qA1 | rs240451691 | 1 | 0 |
| chr11 | 5722991 | T | C | 228 | Urgcp | intronic | . | . | . | 11qA1 | rs255810356 | 1 | 0 |
| chr11 | 5723071 | A | G | 228 | Urgcp | intronic | . | . | . | 11qA1 | rs50356688 | 1 | 0 |
| chr11 | 5723089 | A | G | 228 | Urgcp | intronic | . | . | . | 11qA1 | rs51896253 | 1 | 0 |
| chr11 | 5723330 | A | G | 146 | Urgcp | intronic | . | . | . | 11qA1 | rs227993974 | 1 | 0 |
| chr11 | 5726181 | G | T | 168 | Urgcp | intronic | . | . | . | 11qA1 | rs50938256 | 1 | 0 |
| chr11 | 5726273 | G | A | 228 | Urgcp | intronic | . | . | . | 11qA1 | rs29457570 | 1 | 0 |
| chr11 | 5726667 | T | A | 117 | Urgcp | intronic | . | . | . | 11qA1 | rs49501419 | 1 | 0 |
| chr11 | 5726757 | A | G | 228 | Urgcp | intronic | . | . | . | 11qA1 | rs29441857 | 1 | 0 |
| chr11 | 5727024 | G | A | 123 | Urgcp | intronic | . | . | . | 11qA1 | rs29464691 | 1 | 0 |
| chr11 | 5769257 | C | G | 211 | Ube2d-ps | ncRNA_intronic | . | . | . | 11qA1 | rs26931535 | 1 | 0 |
| chr11 | 5793889 | T | C | 228 | Dbn1 | intronic | . | . | . | 11qA1 | rs33848826 | 1 | 0 |
| chr11 | 5793892 | A | G | 228 | Dbn1 | intronic | . | . | . | 11qA1 | rs29412146 | 1 | 0 |
| chr11 | 5795688 | G | T | 191 | Dbn1 | intronic | . | . | . | 11qA1 | . | 0 | 1 |
| chr11 | 5796538 | A | G | 207 | Dbn1 | intronic | . | . | . | 11qA1 | rs29463450 | 1 | 0 |
| chr11 | 5797531 | G | A | 228 | Dbn1 | intronic | . | . | . | 11qA1 | rs253084195 | 1 | 0 |
| chr11 | 5799381 | T | G | 228 | Dbn1 | intronic | . | . | . | 11qA1 | rs29386557 | 1 | 0 |
| chr11 | 5802094 | C | T | 110 | Pgam2 | intronic | . | . | . | 11qA1 | rs6187057 | 1 | 0 |
| chr11 | 5803105 | A | G | 228 | Pgam2 | exonic | synonymous SNV | Pgam2:NM_018870:exon2:c.T489C:p.A163A | . | 11qA1 | rs13470659 | 1 | 0 |
| chr11 | 5829232 | C | T | 228 | Polm | intronic | . | . | . | 11qA1 | rs26931487 | 1 | 0 |
| chr11 | 5829594 | C | T | 228 | Polm | exonic | synonymous SNV | Polm:NM_017401:exon9:c.G1203A:p.Q401Q | . | 11qA1 | rs244110640 | 1 | 0 |
| chr11 | 5829783 | C | T | 228 | Polm | intronic | . | . | . | 11qA1 | rs26931482 | 1 | 0 |
| chr11 | 5829819 | C | T | 228 | Polm | intronic | . | . | . | 11qA1 | rs26931481 | 1 | 0 |
| chr11 | 5830163 | C | T | 128 | Polm | exonic | synonymous SNV | Polm:NM_017401:exon7:c.G873A:p.R291R | . | 11qA1 | rs26931479 | 1 | 0 |
| chr11 | 5831676 | T | C | 228 | Polm | exonic | synonymous SNV | Polm:NM_017401:exon6:c.A819G:p.T273T | . | 11qA1 | rs47365798 | 1 | 0 |
| chr11 | 5831787 | G | A | 228 | Polm | intronic | . | . | . | 11qA1 | rs26931475 | 1 | 0 |
| chr11 | 5834685 | G | C | 228 | Polm | intronic | . | . | . | 11qA1 | rs26914666 | 1 | 0 |
| chr11 | 5834916 | T | C | 172 | Polm | intronic | . | . | . | 11qA1 | rs26914665 | 1 | 0 |
| chr11 | 5836180 | G | C | 228 | Polm | intronic | . | . | . | 11qA1 | rs26914662 | 1 | 0 |
| chr11 | 5837665 | C | A | 228 | Polm | exonic | synonymous SNV | Polm:NM_017401:exon1:c.G87T:p.P29P | 724 | 11qA1 | rs26914655 | 1 | 0 |
| chr11 | 5837761 | A | G | 228 | Polm | UTR5 | . | . | 724 | 11qA1 | rs26914653 | 1 | 0 |
| chr11 | 5862052 | C | T | 82 | Aebp1 | UTR5 | . | . | 584 | 11qA1 | rs26914619 | 1 | 0 |
| chr11 | 5862115 | G | A | 138 | Aebp1 | UTR5 | . | . | 584 | 11qA1 | rs228548520 | 1 | 0 |
| chr11 | 5862203 | A | G | 228 | Aebp1 | exonic | missense SNV | Aebp1:NM_001291857:exon1:c.A52G:p.T18A,Aebp1:NM_009636:exon1:c.A52G:p.T18A | 584 | 11qA1 | rs258828533 | 1 | 0 |
| chr11 | 5862393 | T | C | 228 | Aebp1 | exonic | missense SNV | Aebp1:NM_001291857:exon1:c.T242C:p.V81A,Aebp1:NM_009636:exon1:c.T242C:p.V81A | . | 11qA1 | rs250492101 | 1 | 0 |
| chr11 | 5862522 | C | G | 183 | Aebp1 | intronic | . | . | . | 11qA1 | rs214833862 | 1 | 0 |
| chr11 | 5862531 | A | G | 149 | Aebp1 | intronic | . | . | . | 11qA1 | rs231185764 | 1 | 0 |
| chr11 | 5863883 | T | C | 135 | Aebp1 | intronic | . | . | . | 11qA1 | rs243092514 | 1 | 0 |
| chr11 | 5864267 | G | C | 186 | Aebp1 | exonic | synonymous SNV | Aebp1:NM_001291857:exon2:c.G576C:p.P192P,Aebp1:NM_009636:exon2:c.G576C:p.P192P | . | 11qA1 | rs258674979 | 1 | 0 |

| | | | | | | | | | | | | | |
|---|---|---|---|---|---|---|---|---|---|---|---|---|---|
| chr11 | 5864290 | A | G | 180 | Aebp1 | intronic | . | . | . | 11qA1 | rs228766705 | 1 | 0 |
| chr11 | 5864728 | G | C | 228 | Aebp1 | intronic | . | . | . | 11qA1 | rs8279651 | 1 | 0 |
| chr11 | 5864933 | A | G | 228 | Aebp1 | intronic | . | . | . | 11qA1 | rs259971237 | 1 | 0 |
| chr11 | 5865154 | A | G | 228 | Aebp1 | intronic | . | . | . | 11qA1 | rs8279656 | 1 | 0 |
| chr11 | 5865322 | A | G | 228 | Aebp1 | intronic | . | . | . | 11qA1 | rs244378694 | 1 | 0 |
| chr11 | 5867166 | C | T | 228 | Aebp1 | intronic | . | . | . | 11qA1 | rs26914611 | 1 | 0 |
| chr11 | 5867239 | A | T | 228 | Aebp1 | intronic | . | . | . | 11qA1 | rs252327101 | 1 | 0 |
| chr11 | 5867814 | T | C | 228 | Aebp1 | intronic | . | . | . | 11qA1 | rs8279689 | 1 | 0 |
| chr11 | 5868525 | T | C | 228 | Aebp1 | exonic | synonymous SNV | Aebp1:NM_001291857:exon13:c.T1512C:p.P504P,Aebp1:NM_009636:exon13:c.T1509C:p.P503P | . | 11qA1 | rs8279701 | 1 | 0 |
| chr11 | 5871155 | G | A | 228 | Aebp1 | intronic | . | . | . | 11qA1 | rs26914608 | 1 | 0 |
| chr11 | 5871386 | G | A | 228 | Aebp1 | exonic | missense SNV | Aebp1:NM_001291857:exon21:c.G2927A:p.R976Q,Aebp1:NM_009636:exon21:c.G2924A:p.R975Q | . | 11qA1 | rs8279706 | 1 | 0 |
| chr11 | 5873659 | T | C | 228 | Pold2 | intronic | . | . | . | 11qA1 | rs29466147 | 1 | 0 |
| chr11 | 5898064 | A | T | 228 | Myl7 | intronic | . | . | . | 11qA1 | rs255216818 | 1 | 0 |
| chr11 | 5898858 | T | C | 228 | . | upstream | . | . | . | 11qA1 | rs26914597 | 1 | 0 |
| chr11 | 5904414 | G | C | 228 | Gck | exonic | synonymous SNV | Gck:NM_001287386:exon7:c.C810G:p.L270L,Gck:NM_010292:exon7:c.C810G:p.L270L | . | 11qA1 | rs26914586 | 1 | 0 |
| chr11 | 5904588 | G | A | 221 | Gck | intronic | . | . | . | 11qA1 | rs33881451 | 0 | 1 |
| chr11 | 5904623 | C | T | 106 | Gck | intronic | . | . | . | 11qA1 | . | 0 | 1 |
| chr11 | 5978181 | A | G | 228 | Camk2b | intronic | . | . | . | 11qA1 | rs26914525 | 1 | 0 |
| chr11 | 5992719 | G | A | 228 | Camk2b | intronic | . | . | . | 11qA1 | rs231991201 | 1 | 0 |
| chr11 | 6015438 | T | C | 228 | Camk2b | intronic | . | . | . | 11qA1 | rs26914469 | 1 | 0 |
| chr11 | 6015487 | T | C | 228 | Camk2b | exonic | synonymous SNV | Camk2b:NM_001174053:exon3:c.A183G:p.E61E,Camk2b:NM_001174054:exon3:c.A183G:p.E61E,Camk2b:NM_( | . | 11qA1 | rs26914468 | 1 | 0 |
| chr11 | 6113369 | G | A | 228 | Nudcd3 | exonic | synonymous SNV | Nudcd3:NM_173748:exon5:c.C855T:p.I285I | . | 11qA1 | rs26899966 | 1 | 0 |
| chr11 | 6171260 | A | T | 145 | Nudcd3 | intronic | . | . | . | 11qA1 | rs29390679 | 1 | 0 |
| chr11 | 6171262 | C | T | 148 | Nudcd3 | intronic | . | . | . | 11qA1 | rs29438713 | 1 | 0 |
| chr11 | 6171270 | A | G | 118 | Nudcd3 | intronic | . | . | . | 11qA1 | rs29428114 | 1 | 0 |
| chr11 | 6172522 | C | T | 159 | Nudcd3,Rps15a-ps( | ncRNA_exonic | . | . | . | 11qA1 | rs243788763 | 1 | 0 |
| chr11 | 6172645 | T | A | 228 | Nudcd3,Rps15a-ps( | ncRNA_exonic | . | . | . | 11qA1 | rs258306046 | 1 | 0 |
| chr11 | 6222264 | C | T | 228 | Npc1l1 | exonic | missense SNV | Npc1l1:NM_207242:exon9:c.G2425A:p.V809I | . | 11qA1 | rs26899830 | 1 | 0 |
| chr11 | 6227981 | T | G | 228 | Npc1l1 | exonic | missense SNV | Npc1l1:NM_207242:exon2:c.A1428C:p.K476N | . | 11qA1 | rs29453506 | 1 | 0 |
| chr11 | 6228656 | C | A | 222 | Npc1l1 | exonic | synonymous SNV | Npc1l1:NM_207242:exon2:c.G753T:p.S251S | . | 11qA1 | . | 0 | 1 |
| chr11 | 6228871 | C | A | 222 | Npc1l1 | exonic | missense SNV | Npc1l1:NM_207242:exon2:c.G538T:p.A180S | . | 11qA1 | . | 0 | 1 |
| chr11 | 6229458 | C | G | 202 | Npc1l1 | intronic | . | . | . | 11qA1 | rs26899813 | 1 | 0 |
| chr11 | 6242452 | C | T | 156 | . | intergenic | . | . | . | 11qA1 | rs47720629 | 1 | 0 |
| chr11 | 6259686 | G | C | 228 | Ddx56 | intronic | . | . | . | 11qA1 | rs26899798 | 1 | 0 |
| chr11 | 6262844 | C | T | 228 | Ddx56 | intronic | . | . | . | 11qA1 | rs225297397 | 1 | 0 |
| chr11 | 6262938 | C | T | 145 | Ddx56 | intronic | . | . | . | 11qA1 | rs217964095 | 1 | 0 |
| chr11 | 6263925 | C | G | 228 | Ddx56 | intronic | . | . | . | 11qA1 | rs26899789 | 1 | 0 |
| chr11 | 6263967 | C | T | 228 | Ddx56 | exonic | synonymous SNV | Ddx56:NM_026538:exon9:c.G1191A:p.L397L | . | 11qA1 | rs263203551 | 1 | 0 |
| chr11 | 6264864 | T | C | 228 | Ddx56 | intronic | . | . | . | 11qA1 | rs26899787 | 1 | 0 |
| chr11 | 6265439 | G | A | 228 | Ddx56 | intronic | . | . | . | 11qA1 | rs253998126 | 1 | 0 |
| chr11 | 6265499 | A | G | 228 | Ddx56 | intronic | . | . | . | 11qA1 | . | 1 | 0 |
| chr11 | 6271753 | C | T | 228 | Tmed4 | exonic | synonymous SNV | Tmed4:NM_134020:exon5:c.G582A:p.R194R | . | 11qA1 | rs26899775 | 1 | 0 |
| chr11 | 6274363 | G | A | 228 | Tmed4 | intronic | . | . | 259 | 11qA1 | rs26899770 | 1 | 0 |
| chr11 | 6274943 | C | T | 228 | . | upstream | . | . | 336 | 11qA1 | rs240288025 | 1 | 0 |
| chr11 | 6314109 | G | A | 209 | Ogdh | intronic | . | . | . | 11qA1 | . | 1 | 0 |
| chr11 | 6334299 | G | A | 101 | Ogdh | intronic | . | . | . | 11qA1 | rs220166502 | 1 | 0 |
| chr11 | 6334315 | T | C | 92 | Ogdh | intronic | . | . | . | 11qA1 | rs236081170 | 1 | 0 |
| chr11 | 6334387 | G | A | 156 | Ogdh | intronic | . | . | . | 11qA1 | rs222030027 | 1 | 0 |
| chr11 | 6338416 | T | C | 184 | Ogdh | intronic | . | . | . | 11qA1 | rs26885847 | 1 | 0 |
| chr11 | 6342362 | C | T | 200 | Ogdh | intronic | . | . | . | 11qA1 | . | 1 | 0 |
| chr11 | 6347124 | T | C | 228 | Ogdh | exonic | synonymous SNV | Ogdh:NM_001252287:exon12:c.T1542C:p.N514N,Ogdh:NM_001252288:exon12:c.T1530C:p.N510N,Ogdh:NM_( | . | 11qA1 | rs26885822 | 1 | 0 |
| chr11 | 6347241 | T | C | 228 | Ogdh | exonic | synonymous SNV | Ogdh:NM_001252287:exon12:c.T1659C:p.P553P,Ogdh:NM_001252288:exon12:c.T1647C:p.P549P,Ogdh:NM_( | . | 11qA1 | rs26885821 | 1 | 0 |
| chr11 | 6348494 | T | C | 228 | Ogdh | intronic | . | . | . | 11qA1 | rs260094824 | 1 | 0 |
| chr11 | 6349025 | C | T | 228 | Ogdh | intronic | . | . | . | 11qA1 | . | 1 | 0 |
| chr11 | 6349067 | T | C | 228 | Ogdh | intronic | . | . | . | 11qA1 | rs26885809 | 1 | 0 |
| chr11 | 6350533 | C | T | 228 | Ogdh | intronic | . | . | . | 11qA1 | . | 1 | 0 |
| chr11 | 6350594 | C | T | 228 | Ogdh | intronic | . | . | . | 11qA1 | rs26885800 | 1 | 0 |
| chr11 | 6350902 | C | G | 151 | Ogdh | intronic | . | . | . | 11qA1 | rs226795113 | 1 | 0 |
| chr11 | 6352333 | C | T | 228 | Ogdh | intronic | . | . | . | 11qA1 | rs3657267 | 1 | 0 |
| chr11 | 6355276 | C | T | 228 | Ogdh | intronic | . | . | . | 11qA1 | . | 1 | 0 |
| chr11 | 6396530 | A | C | 222 | Zmiz2 | intronic | . | . | . | 11qA1 | . | 0 | 1 |
| chr11 | 6398403 | C | T | 228 | Zmiz2 | intronic | . | . | . | 11qA1 | . | 1 | 0 |
| chr11 | 6398446 | A | G | 228 | Zmiz2 | intronic | . | . | . | 11qA1 | rs26885700 | 1 | 0 |
| chr11 | 6399346 | T | C | 168 | Zmiz2 | intronic | . | . | . | 11qA1 | rs26885699 | 1 | 0 |
| chr11 | 6399387 | C | T | 175 | Zmiz2 | intronic | . | . | . | 11qA1 | . | 1 | 0 |
| chr11 | 6399701 | C | T | 228 | Zmiz2 | intronic | . | . | . | 11qA1 | . | 1 | 0 |
| chr11 | 6401401 | A | G | 161 | Zmiz2 | intronic | . | . | . | 11qA1 | rs26885695 | 1 | 0 |
| chr11 | 6402810 | G | A | 228 | Zmiz2 | exonic | synonymous SNV | Zmiz2:NM_001005867:exon15:c.G1839A:p.T613T,Zmiz2:NM_028601:exon15:c.G1935A:p.T645T | . | 11qA1 | . | 1 | 0 |
| chr11 | 6404132 | A | C | 228 | Zmiz2 | exonic | synonymous SNV | Zmiz2:NM_001005867:exon18:c.A2409C:p.L803L,Zmiz2:NM_028601:exon18:c.A2505C:p.L835L | . | 11qA1 | rs29474284 | 1 | 0 |
| chr11 | 6417952 | G | A | 174 | Ppia | intronic | . | . | . | 11qA1 | rs251418272 | 1 | 0 |
| chr11 | 6419000 | T | C | 228 | Ppia | intronic | . | . | . | 11qA1 | rs29430670 | 1 | 0 |
| chr11 | 6419408 | C | T | 228 | Ppia | intronic | . | . | . | 11qA1 | rs29394017 | 1 | 0 |
| chr11 | 6419885 | T | A | 101 | . | downstream | . | . | . | 11qA1 | rs46701142 | 1 | 0 |
| chr11 | 6428107 | T | C | 113 | H2afv | UTR3 | . | . | . | 11qA1 | rs49756795 | 1 | 0 |
| chr11 | 6428198 | C | T | 172 | H2afv | UTR3 | . | . | . | 11qA1 | rs26885652 | 1 | 0 |
| chr11 | 6428299 | G | A | 228 | H2afv | UTR3 | . | . | . | 11qA1 | rs26885650 | 1 | 0 |
| chr11 | 6428306 | C | T | 228 | H2afv | UTR3 | . | . | . | 11qA1 | rs26885649 | 1 | 0 |
| chr11 | 6428640 | T | C | 202 | H2afv | intronic | . | . | . | 11qA1 | rs26885648 | 1 | 0 |
| chr11 | 6428696 | C | G | 124 | H2afv | intronic | . | . | . | 11qA1 | rs26885647 | 1 | 0 |
| chr11 | 6429008 | A | T | 228 | H2afv | intronic | . | . | . | 11qA1 | rs29461512 | 1 | 0 |
| chr11 | 6429208 | A | C | 228 | H2afv | exonic | synonymous SNV | H2afv:NM_029938:exon4:c.T213G:p.G71G | . | 11qA1 | rs26885646 | 1 | 0 |
| chr11 | 6429392 | C | G | 173 | H2afv | intronic | . | . | . | 11qA1 | rs26885644 | 1 | 0 |
| chr11 | 6439342 | T | C | 228 | H2afv | intronic | . | . | . | 11qA1 | rs48135101 | 1 | 0 |
| chr11 | 6446635 | A | T | 165 | . | intergenic | . | . | . | 11qA1 | . | 0 | 1 |
| chr11 | 6446757 | T | C | 101 | . | intergenic | . | . | . | 11qA1 | rs26885630 | 1 | 0 |
| chr11 | 6453118 | C | T | 101 | . | intergenic | . | . | . | 11qA1 | rs240091518 | 1 | 0 |
| chr11 | 6453520 | C | T | 228 | . | intergenic | . | . | . | 11qA1 | . | 1 | 0 |
| chr11 | 6454000 | T | G | 228 | . | intergenic | . | . | . | 11qA1 | rs26885619 | 1 | 0 |
| chr11 | 6468692 | A | T | 228 | Purb | UTR3 | . | . | . | 11qA1 | rs13474585 | 1 | 0 |
| chr11 | 6506472 | T | C | 203 | . | downstream | . | . | . | 11qA1 | rs29399610 | 1 | 0 |
| chr11 | 6508110 | A | G | 228 | Myo1g | intronic | . | . | . | 11qA1 | rs26928842 | 1 | 0 |
| chr11 | 6508361 | C | T | 228 | Myo1g | intronic | . | . | . | 11qA1 | rs26928841 | 1 | 0 |
| chr11 | 6509422 | A | G | 228 | Myo1g | exonic | missense SNV | Myo1g:NM_178440:exon18:c.T2447C:p.I816T | . | 11qA1 | rs26928840 | 1 | 0 |
| chr11 | 6514873 | T | C | 228 | Myo1g | exonic | missense SNV | Myo1g:NM_178440:exon11:c.A1400G:p.H467R | . | 11qA1 | rs6153562 | 1 | 0 |
| chr11 | 6515075 | G | A | 228 | Myo1g | intronic | . | . | . | 11qA1 | rs29444474 | 1 | 0 |
| chr11 | 6516557 | A | G | 167 | Myo1g | intronic | . | . | . | 11qA1 | rs26928806 | 1 | 0 |
| chr11 | 6517069 | C | T | 228 | Myo1g | intronic | . | . | . | 11qA1 | rs26928803 | 1 | 0 |
| chr11 | 6517597 | A | G | 228 | Myo1g | exonic | synonymous SNV | Myo1g:NM_178440:exon5:c.T609C:p.G203G | . | 11qA1 | rs26928802 | 1 | 0 |
| chr11 | 6528210 | G | A | 228 | Gm11974 | ncRNA_intronic | . | . | . | 11qA1 | rs26928786 | 1 | 0 |
| chr11 | 6559668 | A | G | 168 | Ccm2 | intronic | . | . | . | 11qA1 | rs48128442 | 1 | 0 |
| chr11 | 6569648 | C | G | 228 | Ccm2 | intronic | . | . | . | 11qA1 | rs29409545 | 1 | 0 |
| chr11 | 6570178 | T | C | 228 | Ccm2 | intronic | . | . | . | 11qA1 | rs29471184 | 1 | 0 |
| chr11 | 6585069 | G | A | 228 | Ccm2 | exonic | synonymous SNV | Ccm2:NM_001190343:exon3:c.G150A:p.E50E,Ccm2:NM_001190344:exon4:c.G132A:p.E44E,Ccm2:NM_146014: | . | 11qA1 | rs51716912 | 1 | 0 |
| chr11 | 6585156 | T | C | 228 | Ccm2 | exonic | synonymous SNV | Ccm2:NM_001190343:exon3:c.T237C:p.H79H,Ccm2:NM_001190344:exon4:c.T219C:p.H73H,Ccm2:NM_146014: | . | 11qA1 | rs13464281 | 1 | 0 |
| chr11 | 6585308 | C | G | 228 | Ccm2 | intronic | . | . | . | 11qA1 | rs26911332 | 1 | 0 |
| chr11 | 6589612 | C | T | 228 | Ccm2 | intronic | . | . | . | 11qA1 | rs26911311 | 1 | 0 |
| chr11 | 6590738 | A | G | 228 | Ccm2 | intronic | . | . | . | 11qA1 | rs26911299 | 1 | 0 |
| chr11 | 6590768 | T | C | 228 | Ccm2 | exonic | synonymous SNV | Ccm2:NM_001190343:exon5:c.T451C:p.L151L,Ccm2:NM_001190344:exon6:c.T433C:p.L145L,Ccm2:NM_1460] | . | 11qA1 | rs239008698 | 1 | 0 |
| chr11 | 6590909 | C | T | 70 | Ccm2 | intronic | . | . | . | 11qA1 | . | 1 | 0 |
| chr11 | 6590921 | A | G | 228 | Ccm2 | intronic | . | . | . | 11qA1 | rs108846605 | 1 | 0 |
| chr11 | 6590931 | C | A | 228 | Ccm2 | intronic | . | . | . | 11qA1 | rs218784994 | 1 | 0 |
| chr11 | 6592992 | T | C | 228 | Ccm2 | intronic | . | . | . | 11qA1 | rs26911296 | 1 | 0 |
| chr11 | 6593160 | G | A | 228 | Ccm2 | intronic | . | . | . | 11qA1 | rs26911294 | 1 | 0 |
| chr11 | 6593231 | T | C | 228 | Ccm2 | intronic | . | . | . | 11qA1 | rs26911293 | 1 | 0 |
| chr11 | 6593474 | A | G | 228 | Ccm2 | intronic | . | . | . | 11qA1 | rs3725903 | 1 | 0 |
| chr11 | 6593531 | C | T | 228 | Ccm2 | intronic | . | . | . | 11qA1 | rs3726015 | 1 | 0 |
| chr11 | 6593749 | T | C | 228 | Ccm2 | intronic | . | . | . | 11qA1 | rs3667913 | 1 | 0 |
| chr11 | 6593863 | A | G | 192 | Ccm2 | intronic | . | . | . | 11qA1 | rs3668602 | 1 | 0 |
| chr11 | 6593870 | C | A | 177 | Ccm2 | intronic | . | . | . | 11qA1 | rs3668630 | 1 | 0 |
| chr11 | 6594119 | C | T | 228 | Ccm2 | intronic | . | . | . | 11qA1 | rs26911289 | 1 | 0 |
| chr11 | 6594156 | G | C | 228 | Ccm2 | exonic | synonymous SNV | Ccm2:NM_001190343:exon8:c.G777C:p.T259T,Ccm2:NM_001190344:exon9:c.G759C:p.T253T,Ccm2:NM_1460] | . | 11qA1 | rs26911288 | 1 | 0 |
| chr11 | 6594282 | A | G | 228 | Ccm2 | exonic | synonymous SNV | Ccm2:NM_001190343:exon8:c.A903G:p.L301L,Ccm2:NM_001190344:exon9:c.A885G:p.L295L,Ccm2:NM_1460] | . | 11qA1 | rs26911287 | 1 | 0 |
| chr11 | 6596055 | A | G | 228 | Ccm2 | intronic | . | . | . | 11qA1 | rs26911274 | 1 | 0 |
| chr11 | 6596088 | A | G | 228 | Ccm2 | exonic | synonymous SNV | Ccm2:NM_001190343:exon9:c.A915G:p.R305R,Ccm2:NM_001190344:exon10:c.A897G:p.R299R,Ccm2:NM_146( | . | 11qA1 | rs26911273 | 1 | 0 |
| chr11 | 6596193 | T | C | 228 | Ccm2 | exonic | synonymous SNV | Ccm2:NM_001190343:exon9:c.T1020C:p.R340R,Ccm2:NM_001190344:exon10:c.T1002C:p.R334R,Ccm2:NM_14 | . | 11qA1 | rs26911272 | 1 | 0 |
| chr11 | 6596487 | T | C | 228 | Ccm2 | UTR3 | . | . | . | 11qA1 | rs26911269 | 1 | 0 |
| chr11 | 6597847 | A | G | 228 | Nacad | UTR3 | . | . | . | 11qA1 | rs26911254 | 1 | 0 |
| chr11 | 6597873 | C | T | 228 | Nacad | UTR3 | . | . | . | 11qA1 | rs49630926 | 1 | 0 |
| chr11 | 6598831 | C | T | 228 | Nacad | intronic | . | . | . | 11qA1 | rs215437952 | 1 | 0 |
| chr11 | 6599666 | T | A | 228 | Nacad | exonic | missense SNV | Nacad:NM_001081652:exon2:c.A3524T:p.E1175V | . | 11qA1 | rs52061074 | 1 | 0 |
| chr11 | 6599680 | G | A | 228 | Nacad | exonic | synonymous SNV | Nacad:NM_001081652:exon2:c.C3510T:p.P1170P | . | 11qA1 | rs257665051 | 1 | 0 |
| chr11 | 6599922 | C | T | 228 | Nacad | exonic | missense SNV | Nacad:NM_001081652:exon2:c.G3268A:p.A1090T | . | 11qA1 | rs26911247 | 1 | 0 |
| chr11 | 6600007 | C | T | 228 | Nacad | exonic | synonymous SNV | Nacad:NM_001081652:exon2:c.G3183A:p.L1061L | . | 11qA1 | rs243512819 | 1 | 0 |
| chr11 | 6600402 | G | A | 228 | Nacad | exonic | missense SNV | Nacad:NM_001081652:exon2:c.C2788T:p.P930S | . | 11qA1 | rs46119054 | 1 | 0 |
| chr11 | 6600497 | G | A | 217 | Nacad | exonic | missense SNV | Nacad:NM_001081652:exon2:c.C2693T:p.P898L | . | 11qA1 | . | 0 | 1 |
| chr11 | 6600733 | T | C | 228 | Nacad | exonic | synonymous SNV | Nacad:NM_001081652:exon2:c.A2457G:p.P819P | . | 11qA1 | rs47436351 | 1 | 0 |
| chr11 | 6600734 | G | A | 228 | Nacad | exonic | missense SNV | Nacad:NM_001081652:exon2:c.C2456T:p.P819L | . | 11qA1 | rs50563216 | 1 | 0 |
| chr11 | 6600926 | G | C | 222 | Nacad | exonic | missense SNV | Nacad:NM_001081652:exon2:c.C2264G:p.P755R | . | 11qA1 | . | 0 | 1 |
| chr11 | 6601074 | G | A | 228 | Nacad | exonic | missense SNV | Nacad:NM_001081652:exon2:c.C2116T:p.P706S | . | 11qA1 | rs26911240 | 1 | 0 |
| chr11 | 6601622 | T | C | 228 | Nacad | exonic | missense SNV | Nacad:NM_001081652:exon2:c.A1568G:p.N523S | . | 11qA1 | rs51040078 | 1 | 0 |
| chr11 | 6601632 | A | G | 228 | Nacad | exonic | missense SNV | Nacad:NM_001081652:exon2:c.T1558C:p.C520R | . | 11qA1 | rs26911236 | 1 | 0 |
| chr11 | 6602230 | A | G | 228 | Nacad | exonic | synonymous SNV | Nacad:NM_001081652:exon2:c.T960C:p.L320L | . | 11qA1 | rs26911235 | 1 | 0 |
| chr11 | 6616774 | C | T | 228 | Tbrg4 | intronic | . | . | . | 11qA1 | rs50938730 | 1 | 0 |
| chr11 | 6616893 | C | T | 179 | Tbrg4 | intronic | . | . | . | 11qA1 | . | 1 | 0 |
| chr11 | 6617066 | C | T | 161 | Tbrg4 | intronic | . | . | . | 11qA1 | rs233093277 | 1 | 0 |
| chr11 | 6617730 | C | A | 167 | Tbrg4 | intronic | . | . | . | 11qA1 | rs46012643 | 1 | 0 |
| chr11 | 6618045 | C | T | 228 | Tbrg4 | exonic | synonymous SNV | Tbrg4:NM_001130457:exon9:c.G1482A:p.E494E,Tbrg4:NM_134011:exon9:c.G1482A:p.E494E | . | 11qA1 | rs13470631 | 1 | 0 |
| chr11 | 6618619 | C | G | 162 | Tbrg4 | intronic | . | . | . | 11qA1 | . | 0 | 1 |
| chr11 | 6618670 | G | A | 210 | Tbrg4 | intronic | . | . | . | 11qA1 | rs26911203 | 1 | 0 |
| chr11 | 6619248 | T | A | 228 | Tbrg4 | intronic | . | . | . | 11qA1 | rs6289727 | 1 | 0 |
| chr11 | 6621205 | C | T | 210 | Tbrg4 | intronic | . | . | . | 11qA1 | rs51283709 | 1 | 0 |
| chr11 | 6635566 | A | G | 228 | Wap | UTR3 | . | . | . | 11qA1 | rs26911184 | 1 | 0 |
| chr11 | 7063860 | G | C | 105 | Adcy1 | exonic | synonymous SNV | Adcy1:NM_009622:exon1:c.G261C:p.P87P | 555 | 11qA1 | rs217377070 | 1 | 0 |
| chr11 | 7063917 | G | A | 212 | Adcy1 | exonic | synonymous SNV | Adcy1:NM_009622:exon1:c.G318A:p.V106V | 555 | 11qA1 | rs233803259 | 1 | 0 |
| chr11 | 7064262 | G | A | 228 | Adcy1 | intronic | . | . | . | 11qA1 | rs29447766 | 1 | 0 |
| chr11 | 7078690 | G | A | 152 | Adcy1 | intronic | . | . | . | 11qA1 | rs50642728 | 1 | 0 |
| chr11 | 7078867 | A | G | 228 | Adcy1 | intronic | . | . | . | 11qA1 | rs48917096 | 1 | 0 |
| chr11 | 7078892 | G | T | 228 | Adcy1 | exonic | missense SNV | Adcy1:NM_009622:exon2:c.G641T:p.G214V | . | 11qA1 | rs26928486 | 1 | 0 |
| chr11 | 7100265 | G | A | 66 | Adcy1 | intronic | . | . | . | 11qA1 | rs26913394 | 1 | 0 |
| chr11 | 7100526 | A | G | 228 | Adcy1 | intronic | . | . | . | 11qA1 | rs26913391 | 1 | 0 |
| chr11 | 7100739 | G | A | 228 | Adcy1 | intronic | . | . | . | 11qA1 | rs29429100 | 1 | 0 |
| chr11 | 7109122 | A | G | 228 | Adcy1 | intronic | . | . | . | 11qA1 | rs26913340 | 1 | 0 |
| chr11 | 7130166 | G | A | 228 | Adcy1 | intronic | . | . | . | 11qA1 | rs3668599 | 1 | 0 |
| chr11 | 7149967 | C | A | 168 | Adcy1 | exonic | synonymous SNV | Adcy1:NM_009622:exon13:c.C2076A:p.G692G | . | 11qA1 | . | 0 | 1 |
| chr11 | 7161883 | A | G | 152 | Adcy1 | exonic | missense SNV | Adcy1:NM_009622:exon17:c.A2747G:p.E916G | . | 11qA1 | . | 0 | 1 |
| chr11 | 8434338 | T | C | 170 | Tns3 | UTR3 | . | . | . | 11qA1 | rs29452290 | 1 | 0 |
| chr11 | 8434536 | G | A | 228 | Tns3 | exonic | synonymous SNV | Tns3:NM_001083587:exon31:c.C4281T:p.N1427N | . | 11qA1 | rs29442608 | 1 | 0 |
| chr11 | 8434677 | G | A | 228 | Tns3 | intronic | . | . | . | 11qA1 | rs29434082 | 1 | 0 |
| chr11 | 8435459 | A | G | 131 | Tns3 | intronic | . | . | . | 11qA1 | rs230767776 | 1 | 0 |
| chr11 | 8435768 | T | C | 189 | Tns3 | intronic | . | . | . | 11qA1 | . | 0 | 1 |
| chr11 | 8435887 | G | C | 228 | Tns3 | exonic | synonymous SNV | Tns3:NM_001083587:exon29:c.C4110G:p.V1370V | . | 11qA1 | rs26879933 | 1 | 0 |
| chr11 | 8436045 | T | C | 207 | Tns3 | intronic | . | . | . | 11qA1 | rs50847703 | 1 | 0 |
| chr11 | 8437091 | T | C | 228 | Tns3 | intronic | . | . | . | 11qA1 | rs33850833 | 1 | 0 |
| chr11 | 8437300 | G | A | 228 | Tns3 | intronic | . | . | . | 11qA1 | rs48044655 | 1 | 0 |
| chr11 | 8437311 | C | T | 228 | Tns3 | intronic | . | . | . | 11qA1 | rs251427436 | 1 | 0 |
| chr11 | 8437405 | C | A | 226 | Tns3 | intronic | . | . | . | 11qA1 | rs26879928 | 1 | 0 |
| chr11 | 8437468 | A | G | 147 | Tns3 | intronic | . | . | . | 11qA1 | rs224609967 | 1 | 0 |
| chr11 | 8443963 | A | G | 228 | Tns3 | exonic | synonymous SNV | Tns3:NM_001083587:exon27:c.T3885C:p.S1295S | . | 11qA1 | rs252603379 | 1 | 0 |
| chr11 | 8443972 | T | C | 228 | Tns3 | exonic | synonymous SNV | Tns3:NM_001083587:exon27:c.A3876G:p.A1292A | . | 11qA1 | rs212893006 | 1 | 0 |
| chr11 | 8444096 | C | T | 41.8257 | Tns3 | intronic | . | . | . | 11qA1 | . | 0 | 1 |
| chr11 | 8444831 | T | C | 211 | Tns3 | intronic | . | . | . | 11qA1 | rs239495956 | 1 | 0 |
| chr11 | 8444859 | G | T | 55 | Tns3 | intronic | . | . | . | 11qA1 | . | 0 | 1 |
| chr11 | 8444892 | C | T | 228 | Tns3 | intronic | . | . | . | 11qA1 | rs253436920 | 1 | 0 |
| chr11 | 8444953 | C | T | 228 | Tns3 | exonic | synonymous SNV | Tns3:NM_001083587:exon26:c.G3828A:p.P1276P | . | 11qA1 | rs26879903 | 1 | 0 |
| chr11 | 8445075 | G | A | 228 | Tns3 | intronic | . | . | . | 11qA1 | rs243788683 | 1 | 0 |

| | | | | | | | | | | | | | |
|---|---|---|---|---|---|---|---|---|---|---|---|---|---|
| chr11 | 8445804 | C | T | 228 | Tns3 | intronic | . | . | . | 11qA1 | rs587068693 | 1 | 0 |
| chr11 | 8445922 | C | T | 137 | Tns3 | intronic | . | . | . | 11qA1 | rs216013531 | 1 | 0 |
| chr11 | 8445925 | C | T | 140 | Tns3 | intronic | . | . | . | 11qA1 | rs232952152 | 1 | 0 |
| chr11 | 8448407 | C | T | 95 | Tns3 | intronic | . | . | . | 11qA1 | rs26879878 | 1 | 0 |
| chr11 | 8450036 | C | T | 228 | Tns3 | intronic | . | . | . | 11qA1 | rs26879872 | 1 | 0 |
| chr11 | 8450059 | A | C | 228 | Tns3 | intronic | . | . | . | 11qA1 | rs212223596 | 1 | 0 |
| chr11 | 8450267 | C | G | 187 | Tns3 | intronic | . | . | . | 11qA1 | rs26879871 | 1 | 0 |
| chr11 | 8450672 | G | T | 161 | Tns3 | intronic | . | . | . | 11qA1 | rs26926451 | 1 | 0 |
| chr11 | 8450877 | T | G | 228 | Tns3 | exonic | synonymous SNV | Tns3:NM_001083587:exon22:c.A3420C:p.S1140S | . | 11qA1 | rs26926450 | 1 | 0 |
| chr11 | 8450907 | C | T | 228 | Tns3 | exonic | synonymous SNV | Tns3:NM_001083587:exon22:c.G3390A:p.P1130P | . | 11qA1 | rs26926449 | 1 | 0 |
| chr11 | 8450964 | T | A | 228 | Tns3 | exonic | synonymous SNV | Tns3:NM_001083587:exon22:c.A3333T:p.P1111P | . | 11qA1 | rs26926448 | 1 | 0 |
| chr11 | 8451146 | G | T | 228 | Tns3 | exonic | missense SNV | Tns3:NM_001083587:exon22:c.C3151A:p.L1051M | . | 11qA1 | rs51453455 | 1 | 0 |
| chr11 | 8451224 | C | T | 228 | Tns3 | exonic | missense SNV | Tns3:NM_001083587:exon22:c.G3073A:p.V1025I | . | 11qA1 | rs26926444 | 1 | 0 |
| chr11 | 8451234 | T | C | 228 | Tns3 | exonic | synonymous SNV | Tns3:NM_001083587:exon22:c.A3063G:p.T1021T | . | 11qA1 | rs26926443 | 1 | 0 |
| chr11 | 8451357 | G | A | 228 | Tns3 | exonic | synonymous SNV | Tns3:NM_001083587:exon22:c.C2940T:p.P980P | . | 11qA1 | rs26926441 | 1 | 0 |
| chr11 | 8451517 | C | T | 228 | Tns3 | intronic | . | . | . | 11qA1 | rs47559338 | 1 | 0 |
| chr11 | 8451520 | T | C | 228 | Tns3 | intronic | . | . | . | 11qA1 | rs26926440 | 1 | 0 |
| chr11 | 8453023 | T | G | 228 | Tns3 | intronic | . | . | . | 11qA1 | rs51748363 | 1 | 0 |
| chr11 | 8453290 | G | A | 228 | Tns3 | intronic | . | . | . | 11qA1 | rs234020731 | 1 | 0 |
| chr11 | 8453295 | C | T | 228 | Tns3 | intronic | . | . | . | 11qA1 | rs251729154 | 1 | 0 |
| chr11 | 8478423 | A | C | 228 | Tns3 | exonic | missense SNV | Tns3:NM_001083587:exon19:c.T2518G:p.S840A | . | 11qA1 | rs13480859 | 1 | 0 |
| chr11 | 8479452 | A | G | 228 | Tns3 | intronic | . | . | . | 11qA1 | rs26910463 | 1 | 0 |
| chr11 | 8479518 | T | G | 228 | Tns3 | exonic | missense SNV | Tns3:NM_001083587:exon18:c.A2417C:p.E806A | . | 11qA1 | rs26910462 | 1 | 0 |
| chr11 | 8492159 | T | G | 163 | Tns3 | exonic | missense SNV | Tns3:NM_001083587:exon17:c.A2203C:p.S735R | . | 11qA1 | . | 0 | 1 |
| chr11 | 8493369 | A | G | 228 | Tns3 | intronic | . | . | . | 11qA1 | rs26910402 | 1 | 0 |
| chr11 | 8518112 | T | C | 228 | Tns3 | intronic | . | . | . | 11qA1 | rs26910258 | 1 | 0 |
| chr11 | 8532132 | C | A | 85 | Tns3 | intronic | . | . | . | 11qA1 | rs224768184 | 1 | 0 |
| chr11 | 8532542 | A | G | 228 | Tns3 | intronic | . | . | . | 11qA1 | rs26910209 | 1 | 0 |
| chr11 | 8532716 | T | G | 143 | Tns3 | intronic | . | . | . | 11qA1 | rs216647383 | 1 | 0 |
| chr11 | 8538175 | A | G | 213 | Tns3 | intronic | . | . | . | 11qA1 | rs229146603 | 1 | 0 |
| chr11 | 8541006 | C | T | 175 | Tns3 | intronic | . | . | . | 11qA1 | rs245840167 | 1 | 0 |
| chr11 | 8541260 | T | C | 228 | Tns3 | intronic | . | . | . | 11qA1 | rs31010467 | 1 | 0 |
| chr11 | 8545174 | G | A | 228 | Tns3 | intronic | . | . | . | 11qA1 | rs249691119 | 1 | 0 |
| chr11 | 8545193 | T | A | 228 | Tns3 | intronic | . | . | . | 11qA1 | . | 1 | 0 |
| chr11 | 8545295 | A | G | 228 | Tns3 | exonic | synonymous SNV | Tns3:NM_001083587:exon8:c.T258C:p.T86T | . | 11qA1 | rs26895426 | 1 | 0 |
| chr11 | 8545395 | G | A | 228 | Tns3 | intronic | . | . | . | 11qA1 | rs26895425 | 1 | 0 |
| chr11 | 8545447 | A | G | 228 | Tns3 | intronic | . | . | . | 11qA1 | rs224006889 | 1 | 0 |
| chr11 | 8545541 | C | T | 148 | Tns3 | intronic | . | . | . | 11qA1 | rs46209941 | 1 | 0 |
| chr11 | 8547130 | G | A | 181 | Tns3 | intronic | . | . | . | 11qA1 | rs218585251 | 1 | 0 |
| chr11 | 8547452 | T | A | 204 | Tns3 | intronic | . | . | . | 11qA1 | . | 1 | 0 |
| chr11 | 8548803 | C | T | 101 | Tns3 | intronic | . | . | . | 11qA1 | rs237857234 | 1 | 0 |
| chr11 | 8548903 | A | G | 228 | Tns3 | intronic | . | . | . | 11qA1 | rs260062519 | 1 | 0 |
| chr11 | 8548908 | A | C | 228 | Tns3 | intronic | . | . | . | 11qA1 | rs216571352 | 1 | 0 |
| chr11 | 8549100 | G | A | 228 | Tns3 | UTR5 | . | . | . | 11qA1 | rs586454812 | 1 | 0 |
| chr11 | 8549167 | T | C | 228 | Tns3 | intronic | . | . | . | 11qA1 | rs584395191 | 1 | 0 |
| chr11 | 8909979 | C | T | 222 | . | intergenic | . | . | . | 11qA1 | . | 0 | 1 |
| chr11 | 9249595 | C | T | 119 | Abca13 | exonic | missense SNV | Abca13:NM_178259:exon4:c.C293T:p.S98F | . | 11qA1 | . | 0 | 1 |
| chr11 | 9267394 | T | G | 110 | Abca13 | intronic | . | . | . | 11qA1 | . | 0 | 1 |
| chr11 | 9297198 | T | A | 221 | Abca13 | exonic | missense SNV | Abca13:NM_178259:exon18:c.T6944A:p.I2315N | . | 11qA1 | . | 0 | 1 |
| chr11 | 9298926 | T | G | 216 | Abca13 | intronic | . | . | . | 11qA1 | . | 0 | 1 |
| chr11 | 9346014 | C | T | 97 | Abca13 | exonic | synonymous SNV | Abca13:NM_178259:exon28:c.C9978T:p.F3326F | . | 11qA1 | . | 0 | 1 |
| chr11 | 9402002 | C | T | 46.1344 | Abca13 | intronic | . | . | . | 11qA1 | . | 0 | 1 |
| chr11 | 9510689 | T | G | 221 | Abca13 | exonic | missense SNV | Abca13:NM_178259:exon43:c.T12698G:p.L4233R | . | 11qA1 | . | 0 | 1 |
| chr11 | 9567780 | A | C | 222 | Abca13 | exonic | synonymous SNV | Abca13:NM_178259:exon50:c.A13381C:p.R4461R | . | 11qA1 | . | 0 | 1 |
| chr11 | 9622154 | T | G | 71 | Abca13 | intronic | . | . | . | 11qA1 | . | 0 | 1 |
| chr11 | 9636504 | T | C | 110 | Abca13 | intronic | . | . | . | 11qA1 | . | 0 | 1 |
| chr11 | 11261402 | C | A | 158 | Vwc2 | exonic | missense SNV | Vwc2:NM_177033:exon4:c.C857A:p.P286Q | . | 11qA1 | . | 0 | 1 |
| chr11 | 11389271 | A | G | 22.8684 | Zpbp | intronic | . | . | . | 11qA1 | . | 0 | 1 |
| chr11 | 11437654 | A | G | 194 | Zpbp | intronic | . | . | . | 11qA1 | . | 0 | 1 |
| chr11 | 11459881 | T | C | 129 | Zpbp | intronic | . | . | . | 11qA1 | . | 0 | 1 |
| chr11 | 11464571 | G | A | 222 | . | intergenic | . | . | . | 11qA1 | . | 0 | 1 |
| chr11 | 11690457 | C | T | 152 | Ikzf1 | intronic | . | . | . | 11qA1 | . | 0 | 1 |
| chr11 | 11761397 | G | T | 222 | Ikzf1 | intronic | . | . | . | 11qA1 | . | 0 | 1 |
| chr11 | 12309448 | G | T | 62 | Cobl | intronic | . | . | . | 11qA1 | . | 0 | 1 |
| chr11 | 12309572 | C | T | 216 | Cobl | intronic | . | . | . | 11qA1 | . | 0 | 1 |
| chr11 | 12648216 | T | G | 222 | . | intergenic | . | . | . | 11qA1 | . | 0 | 1 |
| chr11 | 16890932 | G | A | 99 | Egfr | intronic | . | . | . | 11qA2 | . | 0 | 1 |
| chr11 | 18884474 | T | G | 222 | Meis1 | exonic | missense SNV | Meis1:NM_001193271:exon11:c.A1027C:p.S343R,Meis1:NM_010789:exon11:c.A1027C:p.S343R | . | 11qA3.1 | . | 0 | 1 |
| chr11 | 19016276 | G | C | 189 | Meis1 | exonic | synonymous SNV | Meis1:NM_001193271:exon2:c.C96G:p.S32S,Meis1:NM_010789:exon2:c.C96G:p.S32S | . | 11qA3.1 | . | 0 | 1 |
| chr11 | 19784077 | C | A | 86 | . | intergenic | . | . | . | 11qA3.1 | . | 0 | 1 |
| chr11 | 22518456 | G | C | 222 | Tmem17 | exonic | missense SNV | Tmem17:NM_153596:exon4:c.G394C:p.D132H | . | 11qA3.2 | . | 0 | 1 |
| chr11 | 23452398 | G | A | 128 | Usp34 | intronic | . | . | . | 11qA3.2 | . | 0 | 1 |
| chr11 | 23649676 | C | A | 222 | Pex13 | intronic | . | . | . | 11qA3.2 | . | 0 | 1 |
| chr11 | 25647140 | T | G | 99 | 5730522E02Rik | ncRNA_intronic | . | . | . | 11qA3.3 | . | 0 | 1 |
| chr11 | 27883270 | T | C | 191 | . | intergenic | . | . | . | 11qA3.3 | . | 0 | 1 |
| chr11 | 29161167 | A | G | 27.6824 | Pnpt1 | intronic | . | . | . | 11qA3.3 | . | 0 | 1 |
| chr11 | 29453122 | A | T | 175 | Ccdc88a | exonic | missense SNV | Ccdc88a:NM_176841:exon10:c.A886T:p.M296L | . | 11qA3.3 | . | 0 | 1 |
| chr11 | 29542638 | A | G | 42.1589 | Mtif2 | intronic | . | . | . | 11qA3.3 | . | 0 | 1 |
| chr11 | 29758761 | T | C | 97 | Eml6 | intronic | . | . | . | 11qA3.3 | rs266214827 | 1 | 0 |
| chr11 | 29758777 | A | G | 144 | Eml6 | intronic | . | . | . | 11qA3.3 | rs222316882 | 1 | 0 |
| chr11 | 30632233 | C | A | 222 | Acyp2 | exonic | missense SNV | Acyp2:NM_029344:exon3:c.G181T:p.G61C | . | 11qA4 | . | 0 | 1 |
| chr11 | 31930066 | G | A | 68 | Cpeb4 | exonic | synonymous SNV | Cpeb4:NM_001290678:exon7:c.G1734A:p.L578L,Cpeb4:NM_001290676:exon8:c.G1785A:p.L595L,Cpeb4:NM | . | 11qA4 | . | 0 | 1 |
| chr11 | 32212033 | C | T | 138 | Rhbdf1 | intronic | . | . | . | 11qA4 | . | 0 | 1 |
| chr11 | 32241764 | C | G | 222 | Nprl3 | intronic | . | . | . | 11qA4 | . | 0 | 1 |
| chr11 | 32598456 | T | C | 167 | Stk10 | exonic | synonymous SNV | Stk10:NM_009288:exon9:c.T1023C:p.T341T | . | 11qA4 | . | 0 | 1 |
| chr11 | 33117810 | T | C | 222 | Fgf18 | exonic | synonymous SNV | Fgf18:NM_008005:exon5:c.A579G:p.T193T | . | 11qA4 | . | 0 | 1 |
| chr11 | 33155881 | A | T | 76 | Npm1 | intronic | . | . | . | 11qA4 | . | 0 | 1 |
| chr11 | 33486051 | T | C | 222 | Ranbp17 | exonic | synonymous SNV | Ranbp17:NM_023146:exon7:c.A627G:p.Q209Q | . | 11qA4 | . | 0 | 1 |
| chr11 | 33970232 | C | A | 221 | Kcnip1,Kcnmb1 | exonic | stopgain | Kcnmb1:NM_031169:exon4:c.C444A:p.Y148X | . | 11qA4 | . | 0 | 1 |
| chr11 | 34080156 | T | C | 221 | Lcp2 | intronic | . | . | . | 11qA4 | . | 0 | 1 |
| chr11 | 34082375 | T | G | 221 | Lcp2 | intronic | . | . | . | 11qA4 | . | 0 | 1 |
| chr11 | 34402248 | A | G | 221 | Fam196b,Dock2 | exonic | missense SNV | Fam196b:NM_001025382:exon2:c.A289G:p.I97V | . | 11qA4 | . | 0 | 1 |
| chr11 | 34402947 | C | A | 222 | Fam196b,Dock2 | exonic | missense SNV | Fam196b:NM_001025382:exon2:c.C988A:p.P330T | . | 11qA4 | . | 0 | 1 |
| chr11 | 34501175 | T | A | 221 | Dock2 | exonic | missense SNV | Dock2:NM_033374:exon25:c.A2549T:p.K850M | . | 11qA4 | . | 0 | 1 |
| chr11 | 34731609 | C | T | 222 | Dock2 | intronic | . | . | . | 11qA4 | . | 0 | 1 |
| chr11 | 35659256 | C | T | 99 | Slit3 | intronic | . | . | . | 11qA4 | . | 0 | 1 |
| chr11 | 35684279 | A | C | 79 | Slit3 | intronic | . | . | . | 11qA4 | . | 0 | 1 |
| chr11 | 35703122 | G | C | 198 | Slit3 | exonic | missense SNV | Slit3:NM_011412:exon35:c.G4138C:p.G1380R | . | 11qA4 | . | 0 | 1 |
| chr11 | 35783472 | G | T | 145 | Pank3 | exonic | missense SNV | Pank3:NM_145962:exon6:c.G961T:p.G321W | . | 11qA4 | . | 0 | 1 |
| chr11 | 35797895 | C | T | 221 | Fbll1 | exonic | synonymous SNV | Fbll1:NM_001004147:exon1:c.G540A:p.L180L | 1164 | 11qA4 | . | 0 | 1 |
| chr11 | 35809197 | C | T | 222 | Rars | exonic | missense SNV | Rars:NM_025936:exon14:c.G1771A:p.D591N | . | 11qA4 | . | 0 | 1 |
| chr11 | 35819763 | C | A | 103 | Rars | intronic | . | . | . | 11qA4 | . | 0 | 1 |
| chr11 | 36008717 | T | A | 222 | Tenm2 | exonic | missense SNV | Tenm2:NM_001290702:exon28:c.A7520T:p.K2507M,Tenm2:NM_011856:exon29:c.A7613T:p.K2538M | . | 11qA4 | . | 0 | 1 |
| chr11 | 36068699 | C | T | 96 | Tenm2 | intronic | . | . | . | 11qA4 | rs47601243 | 0 | 1 |
| chr11 | 36300196 | C | T | 63 | Tenm2 | exonic | missense SNV | Tenm2:NM_001290702:exon4:c.G947A:p.R316Q,Tenm2:NM_011856:exon4:c.G947A:p.R316Q | . | 11qA4 | . | 0 | 1 |
| chr11 | 36864642 | G | A | 194 | Tenm2 | intronic | . | . | . | 11qA5 | rs29415841 | 0 | 1 |
| chr11 | 36865131 | G | C | 59 | Tenm2 | intronic | . | . | . | 11qA5 | rs29451576 | 0 | 1 |
| chr11 | 36943879 | C | A | 122 | Tenm2 | intronic | . | . | . | 11qA5 | rs49098335 | 0 | 1 |
| chr11 | 36944189 | C | T | 102 | Tenm2 | UTR5 | . | . | . | 11qA5 | rs46277559 | 0 | 1 |
| chr11 | 36944198 | T | C | 87 | Tenm2 | UTR5 | . | . | . | 11qA5 | rs50448588 | 0 | 1 |
| chr11 | 37691228 | A | T | 228 | . | intergenic | . | . | . | 11qA5 | rs49560527 | 1 | 0 |
| chr11 | 37691263 | A | G | 228 | . | intergenic | . | . | . | 11qA5 | rs50703311 | 1 | 0 |
| chr11 | 37691466 | A | G | 228 | . | intergenic | . | . | . | 11qA5 | rs46723105 | 1 | 0 |
| chr11 | 37691531 | C | A | 228 | . | intergenic | . | . | . | 11qA5 | rs50934413 | 1 | 0 |
| chr11 | 38219167 | A | G | 228 | . | intergenic | . | . | . | 11qA5 | rs28227421 | 1 | 0 |
| chr11 | 38219371 | G | A | 228 | . | intergenic | . | . | . | 11qA5 | rs27243189 | 1 | 0 |
| chr11 | 38219984 | G | A | 228 | . | intergenic | . | . | . | 11qA5 | rs258646378 | 1 | 0 |
| chr11 | 38923235 | T | C | 178 | . | intergenic | . | . | . | 11qA5 | rs51102153 | 1 | 0 |
| chr11 | 40423265 | A | G | 228 | . | intergenic | . | . | . | 11qA5 | rs51869999 | 1 | 0 |
| chr11 | 40569195 | A | G | 94 | . | intergenic | . | . | . | 11qA5 | . | 1 | 0 |
| chr11 | 40569231 | C | T | 116 | . | intergenic | . | . | . | 11qA5 | . | 1 | 0 |
| chr11 | 40569265 | T | C | 150 | . | intergenic | . | . | . | 11qA5 | rs220330973 | 1 | 0 |
| chr11 | 40569391 | T | A | 197 | . | intergenic | . | . | . | 11qA5 | rs28185558 | 1 | 0 |
| chr11 | 40569583 | C | A | 85 | . | intergenic | . | . | . | 11qA5 | rs28185556 | 1 | 0 |
| chr11 | 40570077 | A | G | 209 | . | intergenic | . | . | . | 11qA5 | rs28185549 | 1 | 0 |
| chr11 | 40570235 | A | G | 228 | . | intergenic | . | . | . | 11qA5 | rs28185548 | 1 | 0 |
| chr11 | 40570391 | A | G | 228 | . | intergenic | . | . | . | 11qA5 | rs28185545 | 1 | 0 |
| chr11 | 40570464 | C | T | 148 | . | intergenic | . | . | . | 11qA5 | rs28185543 | 1 | 0 |
| chr11 | 40679868 | T | C | 150 | Mat2b | UTR3 | . | . | . | 11qA5 | rs29418885 | 1 | 0 |
| chr11 | 40679905 | T | C | 167 | Mat2b | UTR3 | . | . | . | 11qA5 | rs29441882 | 1 | 0 |
| chr11 | 40680021 | G | A | 228 | Mat2b | UTR3 | . | . | . | 11qA5 | rs246825036 | 1 | 0 |
| chr11 | 40680075 | A | G | 228 | Mat2b | exonic | synonymous SNV | Mat2b:NM_001199274:exon7:c.T921C:p.S307S,Mat2b:NM_134017:exon7:c.T954C:p.S318S | . | 11qA5 | rs212985958 | 1 | 0 |
| chr11 | 40680091 | G | A | 228 | Mat2b | exonic | missense SNV | Mat2b:NM_001199274:exon7:c.C905T:p.T302I,Mat2b:NM_134017:exon7:c.C938T:p.T313I | . | 11qA5 | rs230015325 | 1 | 0 |
| chr11 | 40680319 | C | A | 164 | Mat2b | intronic | . | . | . | 11qA5 | rs582359395 | 1 | 0 |
| chr11 | 40680405 | C | A | 228 | Mat2b | intronic | . | . | . | 11qA5 | rs258950680 | 1 | 0 |
| chr11 | 40682383 | C | T | 169 | Mat2b | intronic | . | . | . | 11qA5 | rs29400677 | 1 | 0 |
| chr11 | 40682490 | G | T | 228 | Mat2b | exonic | synonymous SNV | Mat2b:NM_001199274:exon5:c.C661A:p.R221R,Mat2b:NM_134017:exon5:c.C694A:p.R232R | . | 11qA5 | rs246613040 | 1 | 0 |
| chr11 | 40684873 | T | C | 228 | Mat2b | intronic | . | . | . | 11qA5 | rs51803138 | 1 | 0 |
| chr11 | 40684998 | C | T | 81 | Mat2b | intronic | . | . | . | 11qA5 | rs587111275 | 1 | 0 |
| chr11 | 40685189 | C | A | 228 | Mat2b | intronic | . | . | . | 11qA5 | rs255617773 | 1 | 0 |
| chr11 | 40685303 | A | T | 228 | Mat2b | exonic | synonymous SNV | Mat2b:NM_001199274:exon3:c.T264A:p.P88P,Mat2b:NM_134017:exon3:c.T297A:p.P99P | . | 11qA5 | rs13474479 | 1 | 0 |
| chr11 | 40685348 | C | G | 228 | Mat2b | intronic | . | . | . | 11qA5 | rs241895539 | 1 | 0 |
| chr11 | 40687359 | G | C | 228 | Mat2b | intronic | . | . | . | 11qA5 | rs235064886 | 1 | 0 |
| chr11 | 40687393 | A | G | 228 | Mat2b | intronic | . | . | . | 11qA5 | rs214144540 | 1 | 0 |
| chr11 | 40687414 | A | G | 228 | Mat2b | intronic | . | . | . | 11qA5 | rs235114693 | 1 | 0 |
| chr11 | 40694520 | C | G | 135 | Mat2b | intronic | . | . | . | 11qA5 | rs221376603 | 1 | 0 |
| chr11 | 40694665 | A | C | 228 | Mat2b | intronic | . | . | . | 11qA5 | rs249339640 | 1 | 0 |
| chr11 | 40694886 | C | T | 228 | Mat2b | UTR5 | . | . | 227 | 11qA5 | rs238573658 | 1 | 0 |
| chr11 | 40695047 | C | T | 126 | Mat2b | UTR5 | . | . | . | 11qA5 | rs259569478 | 1 | 0 |
| chr11 | 40702878 | G | C | 228 | Hmmr | intronic | . | . | . | 11qA5 | rs249248015 | 1 | 0 |
| chr11 | 40702891 | T | G | 228 | Hmmr | intronic | . | . | . | 11qA5 | rs263198064 | 1 | 0 |
| chr11 | 40703867 | T | C | 228 | Hmmr | exonic | synonymous SNV | Hmmr:NM_013552:exon17:c.A2280G:p.L760L | . | 11qA5 | rs213889821 | 1 | 0 |
| chr11 | 40704003 | G | A | 228 | Hmmr | intronic | . | . | . | 11qA5 | rs229942245 | 1 | 0 |
| chr11 | 40705955 | C | T | 228 | Hmmr | exonic | synonymous SNV | Hmmr:NM_013552:exon16:c.G2148A:p.Q716Q | . | 11qA5 | rs28224097 | 1 | 0 |
| chr11 | 40706039 | T | C | 228 | Hmmr | exonic | synonymous SNV | Hmmr:NM_013552:exon16:c.A2064G:p.K688K | . | 11qA5 | rs28224096 | 1 | 0 |
| chr11 | 40706135 | A | G | 115 | Hmmr | intronic | . | . | . | 11qA5 | rs28224095 | 1 | 0 |
| chr11 | 40706180 | G | A | 228 | Hmmr | intronic | . | . | . | 11qA5 | rs28224094 | 1 | 0 |
| chr11 | 40706208 | G | T | 135 | Hmmr | intronic | . | . | . | 11qA5 | rs28224092 | 1 | 0 |
| chr11 | 40707588 | C | T | 228 | Hmmr | exonic | synonymous SNV | Hmmr:NM_013552:exon14:c.G1911A:p.K637K | . | 11qA5 | . | 1 | 0 |
| chr11 | 40707785 | C | T | 228 | Hmmr | intronic | . | . | . | 11qA5 | rs231073680 | 1 | 0 |
| chr11 | 40709636 | A | G | 228 | Hmmr | intronic | . | . | . | 11qA5 | rs584787510 | 1 | 0 |
| chr11 | 40709821 | A | G | 228 | Hmmr | intronic | . | . | . | 11qA5 | rs28224086 | 1 | 0 |
| chr11 | 40709853 | C | T | 228 | Hmmr | intronic | . | . | . | 11qA5 | rs28224085 | 1 | 0 |
| chr11 | 40710207 | A | G | 170 | Hmmr | exonic | synonymous SNV | Hmmr:NM_013552:exon12:c.T1335C:p.Y445Y | . | 11qA5 | rs229551454 | 0 | 1 |
| chr11 | 40710320 | G | A | 228 | Hmmr | intronic | . | . | . | 11qA5 | rs28224084 | 1 | 0 |
| chr11 | 40714124 | G | A | 228 | Hmmr | exonic | synonymous SNV | Hmmr:NM_013552:exon11:c.C1119T:p.V373V | . | 11qA5 | rs28224065 | 1 | 0 |
| chr11 | 40714209 | A | G | 228 | Hmmr | intronic | . | . | . | 11qA5 | rs28224064 | 1 | 0 |
| chr11 | 40714690 | T | C | 228 | Hmmr | intronic | . | . | . | 11qA5 | rs220076862 | 1 | 0 |
| chr11 | 40714697 | A | C | 228 | Hmmr | intronic | . | . | . | 11qA5 | rs28224063 | 1 | 0 |
| chr11 | 40714798 | C | T | 228 | Hmmr | exonic | synonymous SNV | Hmmr:NM_013552:exon10:c.G963A:p.L321L | . | 11qA5 | rs28224062 | 1 | 0 |
| chr11 | 40715079 | C | T | 204 | Hmmr | intronic | . | . | . | 11qA5 | rs28224061 | 1 | 0 |
| chr11 | 40715257 | T | G | 228 | Hmmr | exonic | synonymous SNV | Hmmr:NM_013552:exon9:c.A885C:p.L295L | . | 11qA5 | rs28224060 | 1 | 0 |
| chr11 | 40721619 | C | T | 228 | Hmmr | intronic | . | . | . | 11qA5 | rs28224046 | 1 | 0 |
| chr11 | 40724983 | T | C | 129 | Hmmr | intronic | . | . | . | 11qA5 | rs224099858 | 1 | 0 |
| chr11 | 40724991 | C | T | 93 | Hmmr | intronic | . | . | . | 11qA5 | rs245925552 | 1 | 0 |
| chr11 | 40728363 | A | C | 210 | Hmmr | intronic | . | . | . | 11qA5 | rs28224034 | 1 | 0 |
| chr11 | 40728516 | G | A | 228 | Hmmr | intronic | . | . | . | 11qA5 | rs248044125 | 1 | 0 |
| chr11 | 40728528 | T | C | 228 | Hmmr | intronic | . | . | . | 11qA5 | rs28224032 | 1 | 0 |
| chr11 | 40729092 | T | C | 133 | Hmmr | intronic | . | . | . | 11qA5 | . | 0 | 1 |
| chr11 | 40729258 | C | T | 228 | Hmmr | intronic | . | . | . | 11qA5 | rs243164153 | 1 | 0 |
| chr11 | 40733137 | A | G | 168 | Hmmr | intronic | . | . | . | 11qA5 | rs29455899 | 1 | 0 |
| chr11 | 40733245 | C | T | 228 | Hmmr | intronic | . | . | . | 11qA5 | rs225367752 | 1 | 0 |
| chr11 | 40733436 | T | C | 228 | Hmmr | UTR5 | . | . | . | 11qA5 | rs46566096 | 1 | 0 |
| chr11 | 40733501 | A | T | 165 | . | upstream | . | . | . | 11qA5 | rs243469422 | 1 | 0 |
| chr11 | 40733523 | C | A | 151 | . | upstream | . | . | . | 11qA5 | rs254239232 | 1 | 0 |

| | | | | | | | | | | | | | |
|---|---|---|---|---|---|---|---|---|---|---|---|---|---|
| chr11 | 40734185 | T | A | 194 | Nudcd2 | intronic | . | . | . | 11qA5 | rs261926986 | 1 | 0 |
| chr11 | 40735916 | C | T | 220 | Nudcd2 | intronic | . | . | . | 11qA5 | rs219094131 | 1 | 0 |
| chr11 | 40735955 | T | C | 228 | Nudcd2 | intronic | . | . | . | 11qA5 | rs237890526 | 1 | 0 |
| chr11 | 40735956 | G | A | 228 | Nudcd2 | intronic | . | . | . | 11qA5 | rs261322140 | 1 | 0 |
| chr11 | 40736111 | T | C | 228 | Nudcd2 | intronic | . | . | . | 11qA5 | rs230792158 | 1 | 0 |
| chr11 | 40736458 | G | A | 228 | Nudcd2 | intronic | . | . | . | 11qA5 | rs245605686 | 1 | 0 |
| chr11 | 40736497 | C | T | 228 | Nudcd2 | exonic | synonymous SNV | Nudcd2:NM_026023:exon3:c.C243T:p.D81D | . | 11qA5 | rs263426333 | 1 | 0 |
| chr11 | 40739292 | C | G | 228 | Nudcd2 | UTR3 | . | . | . | 11qA5 | rs262249236 | 1 | 0 |
| chr11 | 40739312 | G | T | 228 | Nudcd2 | UTR3 | . | . | . | 11qA5 | rs28224024 | 1 | 0 |
| chr11 | 40739382 | C | T | 160 | Nudcd2 | UTR3 | . | . | . | 11qA5 | rs234781403 | 1 | 0 |
| chr11 | 40750737 | T | C | 170 | Ccng1 | UTR3 | . | . | . | 11qA5 | rs223686355 | 1 | 0 |
| chr11 | 40750801 | G | T | 228 | Ccng1 | UTR3 | . | . | . | 11qA5 | . | 1 | 0 |
| chr11 | 40751309 | T | C | 228 | Ccng1 | exonic | synonymous SNV | Ccng1:NM_009831:exon5:c.A645G:p.K215K | . | 11qA5 | rs219744605 | 1 | 0 |
| chr11 | 40751983 | T | C | 209 | Ccng1 | intronic | . | . | . | 11qA5 | rs229816770 | 1 | 0 |
| chr11 | 40752369 | C | A | 228 | Ccng1 | intronic | . | . | . | 11qA5 | rs227267194 | 1 | 0 |
| chr11 | 40752372 | G | C | 228 | Ccng1 | intronic | . | . | . | 11qA5 | rs247499448 | 1 | 0 |
| chr11 | 40753796 | A | G | 228 | Ccng1 | intronic | . | . | . | 11qA5 | rs261136990 | 1 | 0 |
| chr11 | 40754044 | A | C | 228 | Ccng1 | exonic | missense SNV | Ccng1:NM_009831:exon2:c.T25G:p.S9A | . | 11qA5 | . | 1 | 0 |
| chr11 | 40754139 | T | C | 228 | Ccng1 | intronic | . | . | . | 11qA5 | rs256515302 | 1 | 0 |
| chr11 | 40754185 | C | A | 151 | Ccng1 | intronic | . | . | . | 11qA5 | rs223934664 | 1 | 0 |
| chr11 | 41378238 | C | T | 190 | . | intergenic | . | . | . | 11qA5 | rs28222921 | 1 | 0 |
| chr11 | 41913948 | T | A | 222 | Gabrg2 | intronic | . | . | . | 11qA5 | . | 0 | 1 |
| chr11 | 41916146 | C | G | 228 | Gabrg2 | intronic | . | . | . | 11qA5 | rs228235602 | 1 | 0 |
| chr11 | 41916215 | T | A | 162 | Gabrg2 | intronic | . | . | . | 11qA5 | . | 0 | 1 |
| chr11 | 41916497 | C | G | 228 | Gabrg2 | intronic | . | . | . | 11qA5 | rs230643242 | 1 | 0 |
| chr11 | 41916556 | C | T | 228 | Gabrg2 | intronic | . | . | . | 11qA5 | . | 1 | 0 |
| chr11 | 41916673 | T | C | 153 | Gabrg2 | intronic | . | . | . | 11qA5 | rs248561073 | 1 | 0 |
| chr11 | 41920362 | T | C | 228 | Gabrg2 | intronic | . | . | . | 11qA5 | rs214266725 | 1 | 0 |
| chr11 | 41920522 | G | A | 228 | Gabrg2 | exonic | synonymous SNV | Gabrg2:NM_008073:exon7:c.C795T:p.F265F,Gabrg2:NM_177408:exon7:c.C795T:p.F265F | . | 11qA5 | rs215066588 | 1 | 0 |
| chr11 | 41920783 | C | T | 168 | Gabrg2 | intronic | . | . | . | 11qA5 | rs221648871 | 1 | 0 |
| chr11 | 41967399 | T | A | 164 | Gabrg2 | intronic | . | . | . | 11qA5 | rs28242783 | 1 | 0 |
| chr11 | 41967610 | G | A | 228 | Gabrg2 | exonic | synonymous SNV | Gabrg2:NM_008073:exon6:c.C690T:p.D230D,Gabrg2:NM_177408:exon6:c.C690T:p.D230D | . | 11qA5 | rs28242781 | 1 | 0 |
| chr11 | 41967871 | T | A | 148 | Gabrg2 | intronic | . | . | . | 11qA5 | rs28242780 | 1 | 0 |
| chr11 | 41968596 | T | C | 166 | Gabrg2 | intronic | . | . | . | 11qA5 | rs28242778 | 1 | 0 |
| chr11 | 41969020 | C | T | 164 | Gabrg2 | intronic | . | . | . | 11qA5 | rs258610953 | 1 | 0 |
| chr11 | 41971686 | T | C | 228 | Gabrg2 | intronic | . | . | . | 11qA5 | rs29481471 | 1 | 0 |
| chr11 | 41971785 | A | G | 228 | Gabrg2 | exonic | synonymous SNV | Gabrg2:NM_008073:exon4:c.T471C:p.A157A,Gabrg2:NM_177408:exon4:c.T471C:p.A157A | . | 11qA5 | rs28242771 | 1 | 0 |
| chr11 | 42153492 | C | A | 228 | Gabra1 | intronic | . | . | . | 11qA5 | . | 1 | 0 |
| chr11 | 42154649 | A | C | 165 | Gabra1 | intronic | . | . | . | 11qA5 | rs28224545 | 1 | 0 |
| chr11 | 42315304 | T | C | 228 | Gabra6 | intronic | . | . | . | 11qA5 | . | 1 | 0 |
| chr11 | 42320490 | G | A | 221 | Gabra6 | exonic | synonymous SNV | Gabra6:NM_001099641:exon2:c.C51T:p.A17A,Gabra6:NM_008068:exon2:c.C51T:p.A17A | . | 11qA5 | . | 0 | 1 |
| chr11 | 42591686 | C | A | 74 | Gabrb2 | intronic | . | . | . | 11qA5 | . | 0 | 1 |
| chr11 | 42626416 | G | C | 168 | Gabrb2 | intronic | . | . | . | 11qA5 | rs28218745 | 1 | 0 |
| chr11 | 43036720 | G | A | 222 | . | intergenic | . | . | . | 11qA5 | rs28235344 | 0 | 1 |
| chr11 | 43036802 | C | A | 222 | . | intergenic | . | . | . | 11qA5 | rs28235343 | 0 | 1 |
| chr11 | 43036893 | C | T | 221 | . | intergenic | . | . | . | 11qA5 | rs28235342 | 0 | 1 |
| chr11 | 43037107 | C | T | 222 | . | intergenic | . | . | . | 11qA5 | rs28235341 | 0 | 1 |
| chr11 | 43037294 | G | A | 228 | . | intergenic | . | . | . | 11qA5 | rs48272469 | 1 | 0 |
| chr11 | 43151557 | T | C | 228 | Atp10b | intronic | . | . | . | 11qA5 | rs46756065 | 1 | 0 |
| chr11 | 43151725 | A | G | 228 | Atp10b | exonic | synonymous SNV | Atp10b:NM_176999:exon2:c.A111G:p.R37R | . | 11qA5 | rs48010997 | 1 | 0 |
| chr11 | 43151937 | T | C | 228 | Atp10b | intronic | . | . | . | 11qA5 | rs51555280 | 1 | 0 |
| chr11 | 43151938 | G | A | 159 | Atp10b | intronic | . | . | . | 11qA5 | rs47787833 | 0 | 1 |
| chr11 | 43153065 | A | T | 85 | Atp10b | intronic | . | . | . | 11qA5 | rs52601552 | 1 | 0 |
| chr11 | 43153305 | A | G | 228 | Atp10b | exonic | missense SNV | Atp10b:NM_176999:exon3:c.A357G:p.I119M | . | 11qA5 | rs50454054 | 1 | 0 |
| chr11 | 43189548 | C | T | 228 | Atp10b | intronic | . | . | . | 11qA5 | rs215192273 | 1 | 0 |
| chr11 | 43189552 | T | C | 228 | Atp10b | intronic | . | . | . | 11qA5 | rs234428453 | 1 | 0 |
| chr11 | 43194480 | T | C | 228 | Atp10b | intronic | . | . | . | 11qA5 | rs29431584 | 1 | 0 |
| chr11 | 43194528 | G | A | 228 | Atp10b | intronic | . | . | . | 11qA5 | rs29455458 | 1 | 0 |
| chr11 | 43197415 | A | T | 228 | Atp10b | intronic | . | . | . | 11qA5 | rs48842134 | 1 | 0 |
| chr11 | 43197429 | T | C | 228 | Atp10b | intronic | . | . | . | 11qA5 | rs49300923 | 1 | 0 |
| chr11 | 43197601 | A | G | 228 | Atp10b | intronic | . | . | . | 11qA5 | rs29402975 | 1 | 0 |
| chr11 | 43197687 | C | T | 202 | Atp10b | intronic | . | . | . | 11qA5 | rs29411745 | 1 | 0 |
| chr11 | 43197740 | A | G | 161 | Atp10b | intronic | . | . | . | 11qA5 | rs46778736 | 1 | 0 |
| chr11 | 43216324 | T | C | 221 | Atp10b | exonic | synonymous SNV | Atp10b:NM_176999:exon12:c.T2184C:p.A728A | . | 11qA5 | . | 0 | 1 |
| chr11 | 43216755 | A | G | 89 | Atp10b | intronic | . | . | . | 11qA5 | rs29429014 | 0 | 1 |
| chr11 | 43247252 | A | G | 79 | Atp10b | intronic | . | . | . | 11qA5 | rs255936782 | 0 | 1 |
| chr11 | 43247558 | C | T | 221 | Atp10b | intronic | . | . | . | 11qA5 | rs29436060 | 0 | 1 |
| chr11 | 43247572 | A | G | 151 | Atp10b | intronic | . | . | . | 11qA5 | rs29453724 | 0 | 1 |
| chr11 | 43259591 | C | T | 205 | Atp10b | exonic | missense SNV | Atp10b:NM_176999:exon23:c.C4115T:p.T1372I | . | 11qA5 | rs28205210 | 0 | 1 |
| chr11 | 43259639 | G | A | 222 | Atp10b | exonic | missense SNV | Atp10b:NM_176999:exon23:c.G4163A:p.G1388E | . | 11qA5 | rs28205207 | 0 | 1 |
| chr11 | 43420356 | C | T | 222 | Pttg1 | exonic | synonymous SNV | Pttg1:NM_001131054:exon6:c.G573A:p.P191P | . | 11qB1.1 | rs3023336 | 0 | 1 |
| chr11 | 43420436 | T | C | 222 | Pttg1 | UTR3 | . | . | . | 11qB1.1 | rs48071763 | 0 | 1 |
| chr11 | 43420451 | C | A | 228 | Pttg1 | UTR3 | . | . | . | 11qB1.1 | rs3023335 | 1 | 0 |
| chr11 | 43420900 | A | G | 102 | Pttg1 | UTR3 | . | . | . | 11qB1.1 | rs3023331 | 1 | 0 |
| chr11 | 43420906 | A | T | 45.965 | Pttg1 | UTR3 | . | . | . | 11qB1.1 | rs3023330 | 0 | 1 |
| chr11 | 43420978 | T | C | 126 | Pttg1 | UTR3 | . | . | . | 11qB1.1 | rs3023328 | 0 | 1 |
| chr11 | 43421264 | G | A | 211 | Pttg1 | exonic | synonymous SNV | Pttg1:NM_013917:exon4:c.C363T:p.D121D,Pttg1:NM_001131054:exon5:c.C363T:p.D121D | . | 11qB1.1 | rs16821694 | 0 | 1 |
| chr11 | 43421321 | C | G | 181 | Pttg1 | intronic | . | . | . | 11qB1.1 | rs16821695 | 1 | 0 |
| chr11 | 43421323 | T | C | 181 | Pttg1 | intronic | . | . | . | 11qB1.1 | rs16821696 | 1 | 0 |
| chr11 | 43422879 | A | T | 159 | Pttg1 | intronic | . | . | . | 11qB1.1 | rs28183794 | 0 | 1 |
| chr11 | 43422906 | A | G | 110 | Pttg1 | exonic | synonymous SNV | Pttg1:NM_013917:exon3:c.T348C:p.P116P,Pttg1:NM_001131054:exon4:c.T348C:p.P116P | . | 11qB1.1 | rs251627338 | 0 | 1 |
| chr11 | 43424700 | G | A | 221 | Pttg1 | intronic | . | . | . | 11qB1.1 | rs16821680 | 0 | 1 |
| chr11 | 43424761 | G | A | 228 | Pttg1 | exonic | synonymous SNV | Pttg1:NM_013917:exon2:c.C225T:p.G75G,Pttg1:NM_001131054:exon3:c.C225T:p.G75G | . | 11qB1.1 | rs16821681 | 1 | 0 |
| chr11 | 43424829 | C | A | 222 | Pttg1 | exonic | missense SNV | Pttg1:NM_013917:exon2:c.G157T:p.V53L,Pttg1:NM_001131054:exon3:c.G157T:p.V53L | . | 11qB1.1 | rs28183785 | 0 | 1 |
| chr11 | 43425573 | A | C | 125 | Pttg1 | intronic | . | . | . | 11qB1.1 | rs3023321 | 0 | 1 |
| chr11 | 43425589 | T | A | 107 | Pttg1 | exonic | missense SNV | Pttg1:NM_013917:exon1:c.A82T:p.T28S,Pttg1:NM_001131054:exon2:c.A82T:p.T28S | . | 11qB1.1 | rs3023320 | 0 | 1 |
| chr11 | 43425614 | T | A | 89 | Pttg1 | exonic | synonymous SNV | Pttg1:NM_013917:exon1:c.A57T:p.A19A,Pttg1:NM_001131054:exon2:c.A57T:p.A19A | . | 11qB1.1 | rs3023319 | 0 | 1 |
| chr11 | 43425625 | G | T | 228 | Pttg1 | exonic | missense SNV | Pttg1:NM_013917:exon1:c.C46A:p.R16S,Pttg1:NM_001131054:exon2:c.C46A:p.R16S | . | 11qB1.1 | rs3023318 | 1 | 0 |
| chr11 | 43425697 | G | C | 106 | Pttg1 | UTR5 | . | . | . | 11qB1.1 | rs28183784 | 0 | 1 |
| chr11 | 43425720 | G | A | 106 | Pttg1 | UTR5 | . | . | . | 11qB1.1 | rs232012415 | 0 | 1 |
| chr11 | 43425730 | A | G | 109 | Pttg1 | UTR5 | . | . | . | 11qB1.1 | rs28183783 | 0 | 1 |
| chr11 | 43437256 | A | G | 126 | Slu7 | intronic | . | . | . | 11qB1.1 | rs28183747 | 0 | 1 |
| chr11 | 43437258 | G | T | 124 | Slu7 | intronic | . | . | . | 11qB1.1 | rs48026620 | 0 | 1 |
| chr11 | 43437305 | C | T | 142 | Slu7 | intronic | . | . | . | 11qB1.1 | rs28183746 | 0 | 1 |
| chr11 | 43437372 | C | T | 222 | Slu7 | exonic | synonymous SNV | Slu7:NM_198936:exon2:c.C12T:p.A4A,Slu7:NM_148673:exon3:c.C12T:p.A4A | . | 11qB1.1 | rs28183745 | 0 | 1 |
| chr11 | 43437540 | C | G | 228 | Slu7 | intronic | . | . | . | 11qB1.1 | rs28183744 | 1 | 0 |
| chr11 | 43437616 | T | C | 83 | Slu7 | intronic | . | . | . | 11qB1.1 | rs46550024 | 0 | 1 |
| chr11 | 43438048 | G | A | 105 | Slu7 | intronic | . | . | . | 11qB1.1 | rs28183740 | 0 | 1 |
| chr11 | 43438093 | T | C | 193 | Slu7 | intronic | . | . | . | 11qB1.1 | rs28183739 | 0 | 1 |
| chr11 | 43438252 | G | A | 222 | Slu7 | exonic | synonymous SNV | Slu7:NM_198936:exon3:c.G300A:p.E100E,Slu7:NM_148673:exon4:c.G300A:p.E100E | . | 11qB1.1 | rs13471976 | 0 | 1 |
| chr11 | 43438267 | C | T | 222 | Slu7 | exonic | synonymous SNV | Slu7:NM_198936:exon3:c.C315T:p.G105G,Slu7:NM_148673:exon4:c.C315T:p.G105G | . | 11qB1.1 | rs28183738 | 0 | 1 |
| chr11 | 43438387 | A | G | 222 | Slu7 | intronic | . | . | . | 11qB1.1 | rs28183737 | 0 | 1 |
| chr11 | 43438413 | C | T | 95 | Slu7 | intronic | . | . | . | 11qB1.1 | rs233056990 | 0 | 1 |
| chr11 | 43438750 | G | A | 222 | Slu7 | intronic | . | . | . | 11qB1.1 | rs28183736 | 0 | 1 |
| chr11 | 43438755 | A | C | 162 | Slu7 | intronic | . | . | . | 11qB1.1 | rs28183735 | 0 | 1 |
| chr11 | 43438788 | T | C | 171 | Slu7 | intronic | . | . | . | 11qB1.1 | rs260561976 | 0 | 1 |
| chr11 | 43439075 | C | T | 161 | Slu7 | intronic | . | . | . | 11qB1.1 | rs28183730 | 1 | 0 |
| chr11 | 43439095 | G | A | 212 | Slu7 | intronic | . | . | . | 11qB1.1 | . | 0 | 1 |
| chr11 | 43439103 | G | A | 222 | Slu7 | intronic | . | . | . | 11qB1.1 | rs28183729 | 0 | 1 |
| chr11 | 43439124 | T | C | 222 | Slu7 | intronic | . | . | . | 11qB1.1 | rs28183728 | 0 | 1 |
| chr11 | 43439357 | C | T | 221 | Slu7 | intronic | . | . | . | 11qB1.1 | rs28183726 | 0 | 1 |
| chr11 | 43439415 | A | T | 228 | Slu7 | intronic | . | . | . | 11qB1.1 | rs28183723 | 1 | 0 |
| chr11 | 43440554 | A | T | 222 | Slu7 | intronic | . | . | . | 11qB1.1 | rs45673286 | 1 | 0 |
| chr11 | 43440597 | T | C | 73 | Slu7 | intronic | . | . | . | 11qB1.1 | . | 0 | 1 |
| chr11 | 43440619 | G | A | 86 | Slu7 | exonic | synonymous SNV | Slu7:NM_198936:exon6:c.G579A:p.R193R,Slu7:NM_148673:exon7:c.G579A:p.R193R | . | 11qB1.1 | rs28183719 | 0 | 1 |
| chr11 | 43440713 | T | G | 228 | Slu7 | intronic | . | . | . | 11qB1.1 | rs28183718 | 1 | 0 |
| chr11 | 43441133 | C | A | 187 | Slu7 | intronic | . | . | . | 11qB1.1 | rs28183714 | 0 | 1 |
| chr11 | 43441276 | G | C | 209 | Slu7 | intronic | . | . | . | 11qB1.1 | rs28183713 | 0 | 1 |
| chr11 | 43441358 | G | C | 179 | Slu7 | intronic | . | . | . | 11qB1.1 | rs28183712 | 0 | 1 |
| chr11 | 43441381 | T | C | 167 | Slu7 | intronic | . | . | . | 11qB1.1 | . | 0 | 1 |
| chr11 | 43441451 | G | A | 221 | Slu7 | exonic | synonymous SNV | Slu7:NM_198936:exon8:c.G693A:p.K231K,Slu7:NM_148673:exon9:c.G693A:p.K231K | . | 11qB1.1 | rs28183711 | 0 | 1 |
| chr11 | 43441475 | C | T | 194 | Slu7 | exonic | synonymous SNV | Slu7:NM_198936:exon8:c.C717T:p.D239D,Slu7:NM_148673:exon9:c.C717T:p.D239D | . | 11qB1.1 | rs219698094 | 0 | 1 |
| chr11 | 43441610 | G | C | 221 | Slu7 | intronic | . | . | . | 11qB1.1 | rs28183710 | 0 | 1 |
| chr11 | 43441700 | C | A | 222 | Slu7 | intronic | . | . | . | 11qB1.1 | rs28183709 | 0 | 1 |
| chr11 | 43441906 | G | A | 221 | Slu7 | exonic | synonymous SNV | Slu7:NM_198936:exon9:c.G903A:p.G301G,Slu7:NM_148673:exon10:c.G903A:p.G301G | . | 11qB1.1 | rs28183708 | 0 | 1 |
| chr11 | 43441934 | T | A | 221 | Slu7 | intronic | . | . | . | 11qB1.1 | rs29420257 | 0 | 1 |
| chr11 | 43441940 | T | C | 221 | Slu7 | intronic | . | . | . | 11qB1.1 | rs28183707 | 0 | 1 |
| chr11 | 43441993 | G | C | 112 | Slu7 | intronic | . | . | . | 11qB1.1 | . | 0 | 1 |
| chr11 | 43442435 | T | A | 228 | Slu7 | intronic | . | . | . | 11qB1.1 | rs29404882 | 1 | 0 |
| chr11 | 43442528 | A | G | 222 | Slu7 | intronic | . | . | . | 11qB1.1 | rs212119248 | 0 | 1 |
| chr11 | 43442699 | A | T | 99 | Slu7 | intronic | . | . | . | 11qB1.1 | rs215885424 | 0 | 1 |
| chr11 | 43442779 | C | T | 78 | Slu7 | intronic | . | . | . | 11qB1.1 | rs28183706 | 0 | 1 |
| chr11 | 43443313 | C | T | 221 | Slu7 | exonic | synonymous SNV | Slu7:NM_198936:exon12:c.C1165T:p.L389L,Slu7:NM_148673:exon13:c.C1165T:p.L389L | . | 11qB1.1 | rs28183698 | 0 | 1 |
| chr11 | 43444644 | C | T | 65 | Slu7 | intronic | . | . | . | 11qB1.1 | rs28183684 | 0 | 1 |
| chr11 | 43444678 | T | A | 93 | Slu7 | intronic | . | . | . | 11qB1.1 | rs260171380 | 0 | 1 |
| chr11 | 43444858 | G | C | 160 | Slu7 | intronic | . | . | . | 11qB1.1 | rs221025357 | 0 | 1 |
| chr11 | 43445344 | C | T | 43.1322 | Slu7 | intronic | . | . | . | 11qB1.1 | rs224392108 | 0 | 1 |
| chr11 | 43445361 | A | G | 99 | Slu7 | exonic | synonymous SNV | Slu7:NM_198936:exon15:c.A1467G:p.L489L,Slu7:NM_148673:exon16:c.A1467G:p.L489L | . | 11qB1.1 | rs248568908 | 0 | 1 |
| chr11 | 43445938 | C | T | 25.6813 | Slu7 | intronic | . | . | . | 11qB1.1 | rs258631288 | 0 | 1 |
| chr11 | 43445965 | G | C | 69 | Slu7 | intronic | . | . | . | 11qB1.1 | rs28183680 | 0 | 1 |
| chr11 | 43446006 | A | C | 111 | Slu7 | intronic | . | . | . | 11qB1.1 | rs28183679 | 0 | 1 |
| chr11 | 43446158 | C | G | 228 | Slu7 | exonic | synonymous SNV | Slu7:NM_198936:exon16:c.C1725G:p.P575P,Slu7:NM_148673:exon17:c.C1725G:p.P575P | . | 11qB1.1 | rs13471981 | 1 | 0 |
| chr11 | 43463094 | G | T | 40.8655 | . | intergenic | . | . | . | 11qB1.1 | rs29411472 | 0 | 1 |
| chr11 | 43463149 | G | A | 150 | . | intergenic | . | . | . | 11qB1.1 | rs28238029 | 0 | 1 |
| chr11 | 43463244 | G | A | 135 | . | intergenic | . | . | . | 11qB1.1 | rs255001476 | 0 | 1 |
| chr11 | 43463362 | C | T | 228 | . | intergenic | . | . | 259 | 11qB1.1 | rs28238028 | 1 | 0 |
| chr11 | 43463403 | A | G | 228 | . | intergenic | . | . | 259 | 11qB1.1 | rs28238027 | 1 | 0 |
| chr11 | 43463431 | T | C | 228 | . | intergenic | . | . | 259 | 11qB1.1 | rs28238026 | 1 | 0 |
| chr11 | 43480863 | T | C | 167 | C1qtnf2 | intronic | . | . | . | 11qB1.1 | rs49731115 | 1 | 0 |
| chr11 | 43485931 | A | G | 221 | C1qtnf2 | exonic | synonymous SNV | C1qtnf2:NM_026979:exon3:c.A129G:p.L43L | . | 11qB1.1 | . | 0 | 1 |
| chr11 | 43490612 | G | A | 120 | C1qtnf2 | intronic | . | . | . | 11qB1.1 | rs28221097 | 0 | 1 |
| chr11 | 43490636 | C | T | 151 | C1qtnf2 | intronic | . | . | . | 11qB1.1 | rs387521861 | 0 | 1 |
| chr11 | 43490704 | C | T | 222 | C1qtnf2 | intronic | . | . | . | 11qB1.1 | rs234197997 | 0 | 1 |
| chr11 | 43491034 | C | T | 222 | C1qtnf2 | exonic | synonymous SNV | C1qtnf2:NM_026979:exon4:c.C582T:p.G194G | 274 | 11qB1.1 | rs28221096 | 0 | 1 |
| chr11 | 43491130 | G | A | 222 | C1qtnf2 | exonic | synonymous SNV | C1qtnf2:NM_026979:exon4:c.G678A:p.Q226Q | 274 | 11qB1.1 | rs28221094 | 0 | 1 |
| chr11 | 43491435 | T | G | 222 | C1qtnf2 | UTR3 | . | . | . | 11qB1.1 | rs234330776 | 0 | 1 |
| chr11 | 43491454 | C | T | 131 | C1qtnf2 | UTR3 | . | . | . | 11qB1.1 | rs28221093 | 0 | 1 |
| chr11 | 43556689 | G | A | 186 | Ccnjl | exonic | synonymous SNV | Ccnjl:NM_001045530:exon3:c.G198A:p.L66L | . | 11qB1.1 | rs108023783 | 0 | 1 |
| chr11 | 43556816 | T | C | 22.9835 | Ccnjl | intronic | . | . | . | 11qB1.1 | rs247821535 | 0 | 1 |
| chr11 | 43745436 | A | G | 221 | Ttc1 | UTR5 | . | . | . | 11qB1.1 | rs13466130 | 0 | 1 |
| chr11 | 44404169 | A | G | 221 | Il12b | intronic | . | . | . | 11qB1.1 | rs26903002 | 0 | 1 |
| chr11 | 44404222 | A | G | 50 | Il12b | intronic | . | . | . | 11qB1.1 | rs26903001 | 0 | 1 |
| chr11 | 44404224 | C | T | 44.9995 | Il12b | intronic | . | . | . | 11qB1.1 | rs49191872 | 0 | 1 |
| chr11 | 44407710 | G | A | 38.1405 | Il12b | intronic | . | . | . | 11qB1.1 | rs49968995 | 0 | 1 |
| chr11 | 44408089 | C | T | 141 | Il12b | intronic | . | . | . | 11qB1.1 | rs50648860 | 0 | 1 |
| chr11 | 44410164 | T | C | 222 | Il12b | exonic | missense SNV | Il12b:NM_001303244:exon5:c.T506C:p.M169T | . | 11qB1.1 | rs26902972 | 0 | 1 |
| chr11 | 44410821 | A | G | 50 | Il12b | intronic | . | . | . | 11qB1.1 | rs26882564 | 0 | 1 |
| chr11 | 44412527 | T | C | 222 | Il12b | exonic | missense SNV | Il12b:NM_001303244:exon7:c.T880C:p.F294L | . | 11qB1.1 | rs29397656 | 0 | 1 |
| chr11 | 44412613 | A | G | 222 | Il12b | exonic | synonymous SNV | Il12b:NM_001303244:exon7:c.A966G:p.S322S | . | 11qB1.1 | rs26882546 | 0 | 1 |
| chr11 | 44455558 | C | A | 102 | Ublcp1 | UTR3 | . | . | . | 11qB1.1 | rs29393072 | 0 | 1 |
| chr11 | 44456180 | C | T | 84 | Ublcp1 | intronic | . | . | . | 11qB1.1 | rs26882427 | 0 | 1 |
| chr11 | 44456282 | C | T | 134 | Ublcp1 | intronic | . | . | . | 11qB1.1 | rs3089546 | 0 | 1 |
| chr11 | 44456350 | T | C | 221 | Ublcp1 | intronic | . | . | . | 11qB1.1 | rs3023256 | 0 | 1 |
| chr11 | 44456540 | C | A | 61 | Ublcp1 | intronic | . | . | . | 11qB1.1 | rs26882426 | 0 | 1 |
| chr11 | 44460711 | G | A | 221 | Ublcp1 | intronic | . | . | . | 11qB1.1 | rs26882411 | 0 | 1 |
| chr11 | 44460832 | C | T | 203 | Ublcp1 | intronic | . | . | . | 11qB1.1 | rs29421608 | 0 | 1 |
| chr11 | 44460922 | G | A | 94 | Ublcp1 | intronic | . | . | . | 11qB1.1 | rs26882409 | 0 | 1 |
| chr11 | 44461250 | A | G | 53 | Ublcp1 | intronic | . | . | . | 11qB1.1 | rs29417676 | 0 | 1 |
| chr11 | 44463724 | G | A | 163 | Ublcp1 | exonic | synonymous SNV | Ublcp1:NM_024475:exon6:c.C481T:p.L161L | . | 11qB1.1 | rs26882387 | 0 | 1 |
| chr11 | 44463796 | A | G | 94 | Ublcp1 | intronic | . | . | . | 11qB1.1 | rs29426762 | 0 | 1 |
| chr11 | 44465324 | T | C | 128 | Ublcp1 | intronic | . | . | . | 11qB1.1 | rs52024668 | 0 | 1 |
| chr11 | 44465491 | C | T | 221 | Ublcp1 | intronic | . | . | . | 11qB1.1 | rs26882382 | 0 | 1 |
| chr11 | 44465532 | T | C | 221 | Ublcp1 | intronic | . | . | . | 11qB1.1 | rs26882381 | 0 | 1 |
| chr11 | 44465651 | A | G | 221 | Ublcp1 | intronic | . | . | . | 11qB1.1 | rs47566738 | 0 | 1 |
| chr11 | 44466008 | T | A | 78 | Ublcp1 | exonic | synonymous SNV | Ublcp1:NM_024475:exon3:c.A234T:p.R78R | . | 11qB1.1 | rs26882379 | 0 | 1 |

| | | | | | | | | | | | | | |
|---|---|---|---|---|---|---|---|---|---|---|---|---|---|
| chr11 | 44466118 | C | A | 136 | Ublcp1 | intronic | . | . | . | 11qB1.1 | rs26882378 | 0 | 1 |
| chr11 | 44466128 | G | A | 127 | Ublcp1 | intronic | . | . | . | 11qB1.1 | rs26882376 | 0 | 1 |
| chr11 | 44466220 | C | A | 32.3145 | Ublcp1 | intronic | . | . | . | 11qB1.1 | rs49321308 | 0 | 1 |
| chr11 | 44466786 | T | C | 35.311 | Ublcp1 | intronic | . | . | . | 11qB1.1 | rs52281593 | 0 | 1 |
| chr11 | 44466805 | T | C | 46.4414 | Ublcp1 | intronic | . | . | . | 11qB1.1 | rs47080420 | 0 | 1 |
| chr11 | 44466846 | T | C | 128 | Ublcp1 | exonic | synonymous SNV | Ublcp1:NM_024475:exon2:c.A123G:p.E41E | . | 11qB1.1 | rs47487040 | 0 | 1 |
| chr11 | 44467030 | T | C | 212 | Ublcp1 | intronic | . | . | . | 11qB1.1 | rs49676612 | 0 | 1 |
| chr11 | 44467048 | C | T | 205 | Ublcp1 | intronic | . | . | . | 11qB1.1 | rs46002546 | 0 | 1 |
| chr11 | 44524799 | G | A | 95 | Rnf145 | intronic | . | . | . | 11qB1.1 | rs29438431 | 0 | 1 |
| chr11 | 44524818 | A | T | 98 | Rnf145 | intronic | . | . | . | 11qB1.1 | rs29480562 | 0 | 1 |
| chr11 | 44525244 | C | T | 120 | Rnf145 | intronic | . | . | . | 11qB1.1 | rs29419678 | 0 | 1 |
| chr11 | 44531413 | A | T | 107 | Rnf145 | intronic | . | . | . | 11qB1.1 | rs26931217 | 0 | 1 |
| chr11 | 44555077 | G | A | 148 | Rnf145 | intronic | . | . | . | 11qB1.1 | rs26915516 | 0 | 1 |
| chr11 | 44561572 | T | C | 221 | Rnf145 | exonic | synonymous SNV | Rnf145:NM_028862:exon10:c.T1377C:p.R459R | . | 11qB1.1 | rs13464216 | 0 | 1 |
| chr11 | 44561806 | C | T | 181 | Rnf145 | exonic | synonymous SNV | Rnf145:NM_028862:exon10:c.C1611T:p.C537C | . | 11qB1.1 | rs3684482 | 0 | 1 |
| chr11 | 44620540 | G | T | 228 | Ebf1 | exonic | synonymous SNV | Ebf1:NM_001290709:exon2:c.G261T:p.T87T,Ebf1:NM_001290710:exon2:c.G261T:p.T87T,Ebf1:NM_007897: | 311 | 11qB1.1 | rs29438138 | 1 | 0 |
| chr11 | 44621313 | A | C | 228 | Ebf1 | UTR5 | . | . | . | 11qB1.1 | rs29439152 | 1 | 0 |
| chr11 | 44621734 | G | A | 101 | Ebf1 | intronic | . | . | . | 11qB1.1 | rs51040741 | 1 | 0 |
| chr11 | 44621849 | A | T | 228 | Ebf1 | intronic | . | . | . | 11qB1.1 | rs52226804 | 1 | 0 |
| chr11 | 44622025 | G | A | 228 | Ebf1 | intronic | . | . | . | 11qB1.1 | . | 1 | 0 |
| chr11 | 44632956 | A | C | 173 | Ebf1 | intronic | . | . | . | 11qB1.1 | rs29466178 | 1 | 0 |
| chr11 | 44643200 | A | G | 158 | Ebf1 | intronic | . | . | . | 11qB1.1 | rs51001483 | 1 | 0 |
| chr11 | 45813770 | T | A | 228 | F630206G17Rik | ncRNA_intronic | . | . | . | 11qB1.1 | rs221596001 | 1 | 0 |
| chr11 | 45813932 | T | C | 228 | F630206G17Rik | ncRNA_intronic | . | . | . | 11qB1.1 | rs243504182 | 1 | 0 |
| chr11 | 45814004 | C | T | 124 | F630206G17Rik | ncRNA_intronic | . | . | . | 11qB1.1 | rs265382632 | 1 | 0 |
| chr11 | 45887311 | G | A | 195 | Clint1 | intronic | . | . | . | 11qB1.1 | rs225245843 | 1 | 0 |
| chr11 | 45887316 | T | A | 218 | Clint1 | intronic | . | . | . | 11qB1.1 | rs238065914 | 1 | 0 |
| chr11 | 45894141 | A | G | 228 | Clint1 | intronic | . | . | . | 11qB1.1 | rs2020823 | 1 | 0 |
| chr11 | 45894816 | G | A | 157 | Clint1 | intronic | . | . | . | 11qB1.1 | rs230632416 | 1 | 0 |
| chr11 | 45902055 | A | G | 133 | Clint1 | intronic | . | . | . | 11qB1.1 | . | 0 | 1 |
| chr11 | 45902230 | T | C | 228 | Clint1 | exonic | synonymous SNV | Clint1:NM_001045520:exon9:c.T1032C:p.F344F | . | 11qB1.1 | rs13472659 | 1 | 0 |
| chr11 | 45906430 | A | G | 228 | Clint1 | exonic | missense SNV | Clint1:NM_001045520:exon10:c.A1337G:p.N446S | . | 11qB1.1 | rs228082807 | 1 | 0 |
| chr11 | 45984647 | T | G | 228 | Sox30 | intronic | . | . | . | 11qB1.1 | rs29393692 | 1 | 0 |
| chr11 | 45991874 | C | T | 228 | Sox30 | exonic | missense SNV | Sox30:NM_173384:exon4:c.C1730T:p.A577V | . | 11qB1.1 | rs239203276 | 1 | 0 |
| chr11 | 46051997 | C | T | 228 | . | intergenic | . | . | . | 11qB1.1 | rs226827458 | 1 | 0 |
| chr11 | 46056294 | G | T | 170 | Adam19 | intronic | . | . | 331 | 11qB1.1 | rs241363254 | 1 | 0 |
| chr11 | 46093756 | T | C | 209 | Adam19 | intronic | . | . | . | 11qB1.1 | rs215409167 | 1 | 0 |
| chr11 | 46162221 | A | T | 191 | Nipal4 | intronic | . | . | . | 11qB1.1 | rs245399685 | 1 | 0 |
| chr11 | 46221228 | T | C | 228 | Cyfip2 | intronic | . | . | . | 11qB1.1 | rs29466477 | 1 | 0 |
| chr11 | 46238498 | C | T | 228 | Cyfip2,Mir6989,Fn | ncRNA_intronic | . | . | . | 11qB1.1 | rs250166344 | 1 | 0 |
| chr11 | 46238705 | G | C | 228 | Mir6989,Cyfip2,Fn | ncRNA_intronic | . | . | . | 11qB1.1 | rs251944266 | 1 | 0 |
| chr11 | 46239070 | A | C | 228 | Cyfip2,Mir6989,Fn | ncRNA_intronic | . | . | . | 11qB1.1 | rs238037941 | 1 | 0 |
| chr11 | 46239495 | A | C | 228 | Fndc9,Cyfip2,Mir6 | ncRNA_intronic | . | . | . | 11qB1.1 | rs260651266 | 1 | 0 |
| chr11 | 46239819 | A | G | 228 | Fndc9,Cyfip2,Mir6 | ncRNA_intronic | . | . | . | 11qB1.1 | rs238472061 | 1 | 0 |
| chr11 | 46239820 | T | C | 228 | Fndc9,Mir6989,Cy | ncRNA_intronic | . | . | . | 11qB1.1 | rs256788999 | 1 | 0 |
| chr11 | 46240095 | G | A | 228 | Cyfip2,Mir6989 | splicing | . | . | . | 11qB1.1 | rs219207511 | 1 | 0 |
| chr11 | 46242194 | G | A | 228 | Cyfip2,Mir6989 | ncRNA_intronic | . | . | . | 11qB1.1 | rs243338257 | 1 | 0 |
| chr11 | 46244192 | C | T | 228 | Cyfip2,Mir6989 | ncRNA_intronic | . | . | . | 11qB1.1 | rs584858564 | 1 | 0 |
| chr11 | 46247323 | G | T | 116 | Cyfip2,Mir6989 | ncRNA_intronic | . | . | . | 11qB1.1 | rs223268891 | 1 | 0 |
| chr11 | 46247547 | T | C | 228 | Cyfip2,Mir6989 | ncRNA_intronic | . | . | . | 11qB1.1 | rs257999712 | 1 | 0 |
| chr11 | 46247784 | T | C | 228 | Cyfip2,Mir6989 | ncRNA_intronic | . | . | . | 11qB1.1 | rs6273736 | 1 | 0 |
| chr11 | 46247787 | T | G | 228 | Cyfip2,Mir6989 | ncRNA_intronic | . | . | . | 11qB1.1 | rs6273739 | 1 | 0 |
| chr11 | 46249607 | A | G | 187 | Mir6989,Cyfip2 | ncRNA_intronic | . | . | . | 11qB1.1 | rs26951478 | 1 | 0 |
| chr11 | 46250016 | T | C | 126 | Mir6989,Cyfip2 | ncRNA_intronic | . | . | . | 11qB1.1 | rs26951475 | 1 | 0 |
| chr11 | 46261040 | G | A | 228 | Cyfip2,Mir6989 | ncRNA_intronic | . | . | . | 11qB1.1 | rs221647363 | 1 | 0 |
| chr11 | 46261401 | C | T | 136 | Mir6989,Cyfip2 | ncRNA_intronic | . | . | . | 11qB1.1 | . | 0 | 1 |
| chr11 | 46270470 | C | T | 156 | Mir6989,Cyfip2 | ncRNA_intronic | . | . | . | 11qB1.1 | rs222548384 | 1 | 0 |
| chr11 | 46270473 | G | A | 156 | Cyfip2,Mir6989 | ncRNA_intronic | . | . | . | 11qB1.1 | rs238100302 | 1 | 0 |
| chr11 | 46276925 | C | T | 228 | Mir6989,Cyfip2 | ncRNA_intronic | . | . | . | 11qB1.1 | rs255608336 | 1 | 0 |
| chr11 | 46277999 | A | G | 228 | Cyfip2,Mir6989 | ncRNA_intronic | . | . | . | 11qB1.1 | rs234371271 | 1 | 0 |
| chr11 | 46284100 | C | T | 216 | Cyfip2,Mir6989 | ncRNA_intronic | . | . | . | 11qB1.1 | rs234086873 | 1 | 0 |
| chr11 | 46284108 | C | T | 213 | Mir6989,Cyfip2 | ncRNA_intronic | . | . | . | 11qB1.1 | rs264116659 | 1 | 0 |
| chr11 | 46284267 | T | G | 228 | Mir6989,Cyfip2 | ncRNA_intronic | . | . | . | 11qB1.1 | rs223977797 | 1 | 0 |
| chr11 | 46284271 | A | G | 228 | Mir6989,Cyfip2 | ncRNA_intronic | . | . | . | 11qB1.1 | rs249518973 | 1 | 0 |
| chr11 | 46284350 | G | A | 116 | Cyfip2,Mir6989 | ncRNA_intronic | . | . | . | 11qB1.1 | rs28230745 | 1 | 0 |
| chr11 | 46289401 | A | G | 58 | Mir6989,Cyfip2 | ncRNA_intronic | . | . | . | 11qB1.1 | rs242735669 | 1 | 0 |
| chr11 | 46289572 | C | T | 228 | Cyfip2,Mir6989 | ncRNA_intronic | . | . | . | 11qB1.1 | rs28230716 | 1 | 0 |
| chr11 | 46289622 | G | C | 228 | Cyfip2,Mir6989 | ncRNA_intronic | . | . | . | 11qB1.1 | rs28230715 | 1 | 0 |
| chr11 | 46291496 | C | A | 228 | Cyfip2,Mir6989 | ncRNA_intronic | . | . | . | 11qB1.1 | rs263187007 | 1 | 0 |
| chr11 | 46291498 | C | T | 228 | Mir6989,Cyfip2 | ncRNA_intronic | . | . | . | 11qB1.1 | rs241987215 | 1 | 0 |
| chr11 | 46291752 | T | G | 228 | Cyfip2,Mir6989 | ncRNA_intronic | . | . | . | 11qB1.1 | rs28230702 | 1 | 0 |
| chr11 | 46327287 | C | T | 167 | Mir6989,Itk | ncRNA_intronic | . | . | . | 11qB1.1 | rs29469192 | 1 | 0 |
| chr11 | 46331631 | T | C | 154 | Itk,Mir6989 | ncRNA_intronic | . | . | . | 11qB1.1 | rs26951386 | 1 | 0 |
| chr11 | 46331719 | G | A | 169 | Itk,Mir6989 | ncRNA_intronic | . | . | . | 11qB1.1 | rs26951384 | 1 | 0 |
| chr11 | 46331766 | C | T | 228 | Mir6989,Itk | ncRNA_intronic | . | . | . | 11qB1.1 | rs26951383 | 1 | 0 |
| chr11 | 46331812 | T | G | 228 | Mir6989,Itk | ncRNA_intronic | . | . | . | 11qB1.1 | rs26951382 | 1 | 0 |
| chr11 | 46332099 | C | T | 157 | Itk,Mir6989 | ncRNA_intronic | . | . | . | 11qB1.1 | rs26951380 | 1 | 0 |
| chr11 | 46332133 | A | C | 101 | Mir6989,Itk | ncRNA_intronic | . | . | . | 11qB1.1 | rs26951379 | 1 | 0 |
| chr11 | 46336342 | C | T | 228 | Itk,Mir6989 | ncRNA_intronic | . | . | . | 11qB1.1 | rs230073813 | 1 | 0 |
| chr11 | 46336345 | G | A | 228 | Itk,Mir6989 | splicing | . | . | . | 11qB1.1 | rs252115818 | 1 | 0 |
| chr11 | 46336478 | G | A | 228 | Mir6989,Itk | exonic | synonymous SNV | Itk:NM_001281965:exon13:c.C1335T:p.H445H,Itk:NM_001281966:exon13:c.C1326T:p.H442H,Itk:NM_0012 | . | 11qB1.1 | rs262869075 | 1 | 0 |
| chr11 | 46336532 | A | G | 228 | Mir6989,Itk | exonic | synonymous SNV | Itk:NM_001281965:exon13:c.T1281C:p.Y427Y,Itk:NM_001281966:exon13:c.T1272C:p.Y424Y,Itk:NM_0012 | . | 11qB1.1 | rs226701464 | 1 | 0 |
| chr11 | 46336684 | G | A | 162 | Mir6989,Itk | ncRNA_intronic | . | . | . | 11qB1.1 | rs49101319 | 1 | 0 |
| chr11 | 46338037 | G | A | 228 | Itk,Mir6989 | ncRNA_intronic | . | . | . | 11qB1.1 | rs264280133 | 1 | 0 |
| chr11 | 46340494 | C | T | 171 | Mir6989,Itk | ncRNA_intronic | . | . | . | 11qB1.1 | rs29458190 | 1 | 0 |
| chr11 | 46340512 | A | G | 177 | Mir6989,Itk | ncRNA_intronic | . | . | . | 11qB1.1 | rs29468668 | 1 | 0 |
| chr11 | 46340815 | A | G | 228 | Itk,Mir6989 | ncRNA_intronic | . | . | . | 11qB1.1 | rs29450481 | 1 | 0 |
| chr11 | 46340826 | C | A | 228 | Mir6989,Itk | ncRNA_intronic | . | . | . | 11qB1.1 | rs29449003 | 1 | 0 |
| chr11 | 46340892 | A | G | 184 | Mir6989,Itk | ncRNA_intronic | . | . | . | 11qB1.1 | rs29384707 | 1 | 0 |
| chr11 | 46341260 | A | G | 228 | Mir6989,Itk | ncRNA_intronic | . | . | . | 11qB1.1 | rs241691843 | 1 | 0 |
| chr11 | 46342141 | A | G | 98 | Itk,Mir6989 | ncRNA_intronic | . | . | . | 11qB1.1 | rs51822912 | 1 | 0 |
| chr11 | 46342442 | T | C | 228 | Itk,Mir6989 | exonic | synonymous SNV | Itk:NM_001281965:exon9:c.A825G:p.T275T,Itk:NM_001281966:exon9:c.A816G:p.T272T,Itk:NM_0012819 | . | 11qB1.1 | rs50301303 | 1 | 0 |
| chr11 | 46342551 | T | A | 228 | Mir6989,Itk | ncRNA_intronic | . | . | . | 11qB1.1 | rs263477974 | 1 | 0 |
| chr11 | 46344810 | C | T | 228 | Itk,Mir6989 | ncRNA_intronic | . | . | . | 11qB1.1 | rs29421999 | 1 | 0 |
| chr11 | 46354171 | C | A | 106 | Itk,Mir6989 | ncRNA_intronic | . | . | . | 11qB1.1 | rs253601530 | 1 | 0 |
| chr11 | 46355799 | T | C | 228 | Itk,Mir6989 | exonic | synonymous SNV | Itk:NM_001281965:exon6:c.A591G:p.A197A,Itk:NM_001281966:exon6:c.A582G:p.A194A,Itk:NM_0012819 | . | 11qB1.1 | rs29405550 | 1 | 0 |
| chr11 | 46360233 | G | C | 228 | Mir6989,Itk | ncRNA_intronic | . | . | . | 11qB1.1 | rs29457984 | 1 | 0 |
| chr11 | 46360246 | A | G | 228 | Itk,Mir6989 | ncRNA_intronic | . | . | . | 11qB1.1 | rs29430682 | 1 | 0 |
| chr11 | 46360373 | G | A | 228 | Itk,Mir6989 | ncRNA_intronic | . | . | . | 11qB1.1 | rs214499580 | 1 | 0 |
| chr11 | 46366079 | T | C | 228 | Itk,Mir6989 | splicing | . | . | . | 11qB1.1 | rs26951325 | 1 | 0 |
| chr11 | 46407214 | A | G | 228 | Mir6989,Fam71b | exonic | synonymous SNV | Fam71b:NM_001013783:exon2:c.A1344G:p.P448P | . | 11qB1.1 | rs248211491 | 1 | 0 |
| chr11 | 46455094 | G | T | 228 | Mir6989,Havcr2 | ncRNA_intronic | . | . | . | 11qB1.1 | rs231774823 | 1 | 0 |
| chr11 | 46456392 | C | T | 228 | Mir6989,Havcr2 | exonic | synonymous SNV | Havcr2:NM_134250:exon2:c.C195T:p.N65N | . | 11qB1.1 | rs48960716 | 1 | 0 |
| chr11 | 46456476 | C | T | 228 | Mir6989,Havcr2 | exonic | synonymous SNV | Havcr2:NM_134250:exon2:c.C279T:p.D93D | . | 11qB1.1 | rs48144335 | 1 | 0 |
| chr11 | 46459145 | A | G | 228 | Mir6989,Havcr2 | ncRNA_intronic | . | . | . | 11qB1.1 | rs46905390 | 1 | 0 |
| chr11 | 46467096 | T | C | 169 | Mir6989,Havcr2 | ncRNA_intronic | . | . | . | 11qB1.1 | rs29430254 | 1 | 0 |
| chr11 | 46479113 | A | G | 151 | Havcr2,Mir6989 | ncRNA_intronic | . | . | . | 11qB1.1 | rs28230614 | 1 | 0 |
| chr11 | 46526224 | A | G | 228 | Mir6989,Gm12169 | ncRNA_intronic | . | . | . | 11qB1.1 | rs29393403 | 1 | 0 |
| chr11 | 46526337 | G | C | 228 | Mir6989,Gm12169 | ncRNA_intronic | . | . | . | 11qB1.1 | rs29487125 | 1 | 0 |
| chr11 | 46526393 | G | A | 228 | Gm12169,Mir6989 | ncRNA_intronic | . | . | . | 11qB1.1 | rs29435820 | 1 | 0 |
| chr11 | 46528372 | A | G | 228 | Mir6989,Gm12169 | ncRNA_intronic | . | . | . | 11qB1.1 | rs28230504 | 1 | 0 |
| chr11 | 46528416 | C | G | 228 | Mir6989,Gm12169 | exonic | missense SNV | Gm12169:NM_001163357:exon3:c.C58G:p.H20D | . | 11qB1.1 | rs13481009 | 1 | 0 |
| chr11 | 46528591 | A | G | 228 | Gm12169,Mir6989 | exonic | missense SNV | Gm12169:NM_001163357:exon3:c.A233G:p.N78S | . | 11qB1.1 | rs28230503 | 1 | 0 |
| chr11 | 46533189 | A | T | 228 | Mir6989,Gm12169 | ncRNA_intronic | . | . | . | 11qB1.1 | rs28230485 | 1 | 0 |
| chr11 | 46533267 | C | T | 228 | Mir6989,Gm12169 | ncRNA_intronic | . | . | . | 11qB1.1 | rs29443101 | 1 | 0 |
| chr11 | 46535401 | C | T | 183 | Gm12169,Mir6989 | ncRNA_intronic | . | . | . | 11qB1.1 | rs249155204 | 1 | 0 |
| chr11 | 46535418 | G | A | 228 | Mir6989,Gm12169 | ncRNA_intronic | . | . | . | 11qB1.1 | rs28230479 | 1 | 0 |
| chr11 | 46536290 | T | G | 149 | Gm12169,Mir6989 | ncRNA_intronic | . | . | . | 11qB1.1 | rs215156510 | 1 | 0 |
| chr11 | 46536322 | C | T | 228 | Mir6989,Gm12169 | ncRNA_intronic | . | . | . | 11qB1.1 | rs242957282 | 1 | 0 |
| chr11 | 46536583 | A | T | 162 | Mir6989,Gm12169 | ncRNA_intronic | . | . | . | 11qB1.1 | rs219311338 | 1 | 0 |
| chr11 | 46537707 | A | G | 148 | Gm12169,Mir6989 | ncRNA_intronic | . | . | . | 11qB1.1 | rs28230473 | 1 | 0 |
| chr11 | 46537775 | T | C | 228 | Gm12169,Mir6989 | ncRNA_intronic | . | . | . | 11qB1.1 | rs227981516 | 1 | 0 |
| chr11 | 46537813 | T | G | 228 | Mir6989,Gm12169 | ncRNA_intronic | . | . | . | 11qB1.1 | rs28230472 | 1 | 0 |
| chr11 | 46538021 | A | C | 228 | Gm12169,Mir6989 | ncRNA_intronic | . | . | . | 11qB1.1 | rs29423348 | 1 | 0 |
| chr11 | 46538154 | A | G | 169 | Gm12169,Mir6989 | ncRNA_intronic | . | . | . | 11qB1.1 | rs28230471 | 1 | 0 |
| chr11 | 46550397 | C | T | 228 | Gm12171,Mir6989 | exonic | missense SNV | Gm12171:NM_001163353:exon2:c.C68T:p.T23M | . | 11qB1.1 | rs261891805 | 1 | 0 |
| chr11 | 46554257 | G | A | 228 | Mir6989,Gm12171 | exonic | synonymous SNV | Gm12171:NM_001163353:exon3:c.G159A:p.P53P | . | 11qB1.1 | rs47519414 | 1 | 0 |
| chr11 | 46556062 | G | C | 228 | Gm12171,Mir6989 | exonic | synonymous SNV | Gm12171:NM_001163353:exon4:c.G321C:p.A107A | . | 11qB1.1 | rs3708206 | 1 | 0 |
| chr11 | 46625502 | G | T | 206 | Dppa1 | intronic | . | . | . | 11qB1.1 | . | 0 | 1 |
| chr11 | 46625541 | G | A | 228 | Dppa1 | intronic | . | . | . | 11qB1.1 | rs47112354 | 1 | 0 |
| chr11 | 46639304 | T | A | 217 | Gm4926 | ncRNA_intronic | . | . | . | 11qB1.1 | rs28215177 | 1 | 0 |
| chr11 | 46644469 | G | A | 151 | Gm4926 | ncRNA_intronic | . | . | . | 11qB1.1 | rs243179370 | 1 | 0 |
| chr11 | 46644893 | T | C | 228 | Gm4926 | ncRNA_exonic | . | . | . | 11qB1.1 | rs28200162 | 1 | 0 |
| chr11 | 46645041 | A | G | 228 | . | upstream | . | . | . | 11qB1.1 | rs238477192 | 1 | 0 |
| chr11 | 46645059 | T | C | 228 | . | upstream | . | . | . | 11qB1.1 | rs29449526 | 1 | 0 |
| chr11 | 46648572 | G | T | 228 | . | intergenic | . | . | . | 11qB1.1 | rs46041963 | 1 | 0 |
| chr11 | 46670628 | C | T | 108 | Timd2 | UTR3 | . | . | . | 11qB1.1 | rs250666176 | 1 | 0 |
| chr11 | 46670662 | G | A | 125 | Timd2 | UTR3 | . | . | . | 11qB1.1 | rs45791853 | 1 | 0 |
| chr11 | 46670700 | A | G | 152 | Timd2 | UTR3 | . | . | . | 11qB1.1 | rs29456806 | 1 | 0 |
| chr11 | 46670913 | G | A | 228 | Timd2 | exonic | synonymous SNV | Timd2:NM_001161355:exon9:c.C903T:p.P301P,Timd2:NM_001161356:exon9:c.C903T:p.P301P,Timd2:NM_1 | . | 11qB1.1 | rs29450092 | 1 | 0 |
| chr11 | 46671075 | G | A | 228 | Timd2 | intronic | . | . | . | 11qB1.1 | rs258353479 | 1 | 0 |
| chr11 | 46671115 | A | G | 228 | Timd2 | intronic | . | . | . | 11qB1.1 | rs51557713 | 1 | 0 |
| chr11 | 46679488 | C | G | 201 | Timd2 | intronic | . | . | . | 11qB1.1 | rs28200057 | 1 | 0 |
| chr11 | 46679660 | G | T | 228 | Timd2 | exonic | missense SNV | Timd2:NM_001161355:exon5:c.C587A:p.S196Y,Timd2:NM_001161356:exon5:c.C587A:p.S196Y,Timd2:NM_1 | . | 11qB1.1 | rs47459144 | 1 | 0 |
| chr11 | 46679661 | A | C | 228 | Timd2 | exonic | missense SNV | Timd2:NM_001161355:exon5:c.T586G:p.S196A,Timd2:NM_001161356:exon5:c.T586G:p.S196A,Timd2:NM_1 | . | 11qB1.1 | rs51331845 | 1 | 0 |
| chr11 | 46679821 | C | A | 127 | Timd2 | intronic | . | . | . | 11qB1.1 | rs234243917 | 1 | 0 |
| chr11 | 46679825 | A | C | 116 | Timd2 | intronic | . | . | . | 11qB1.1 | rs253945170 | 1 | 0 |
| chr11 | 46683089 | T | C | 212 | Timd2 | intronic | . | . | . | 11qB1.1 | rs29403037 | 1 | 0 |
| chr11 | 46686794 | A | C | 153 | Timd2 | intronic | . | . | . | 11qB1.1 | rs28200007 | 1 | 0 |
| chr11 | 46689324 | T | C | 31.8005 | Timd2 | intronic | . | . | . | 11qB1.1 | . | 0 | 1 |
| chr11 | 46689397 | T | C | 222 | Timd2 | intronic | . | . | . | 11qB1.1 | rs28200001 | 0 | 1 |
| chr11 | 46689459 | T | C | 221 | Timd2 | intronic | . | . | . | 11qB1.1 | rs29431087 | 0 | 1 |
| chr11 | 46689637 | T | C | 228 | Timd2 | intronic | . | . | . | 11qB1.1 | rs28199999 | 1 | 0 |
| chr11 | 46755757 | G | A | 150 | Havcr1 | intronic | . | . | . | 11qB1.1 | rs26994826 | 1 | 0 |
| chr11 | 46755936 | G | C | 228 | Havcr1 | intronic | . | . | . | 11qB1.1 | rs26994825 | 1 | 0 |
| chr11 | 46755981 | T | C | 228 | Havcr1 | intronic | . | . | . | 11qB1.1 | rs252652954 | 1 | 0 |
| chr11 | 46756150 | G | C | 228 | Havcr1 | exonic | missense SNV | Havcr1:NM_134248:exon3:c.G410C:p.R137T,Havcr1:NM_001166631:exon4:c.G410C:p.R137T,Havcr1:NM_0 | . | 11qB1.1 | rs29399302 | 1 | 0 |
| chr11 | 46766618 | C | T | 228 | Havcr1 | intronic | . | . | . | 11qB1.1 | rs47123158 | 1 | 0 |
| chr11 | 46766626 | G | T | 228 | Havcr1 | intronic | . | . | . | 11qB1.1 | rs48726453 | 1 | 0 |
| chr11 | 46766867 | C | T | 169 | Havcr1 | intronic | . | . | . | 11qB1.1 | rs46850191 | 1 | 0 |
| chr11 | 46842120 | G | A | 228 | Timd4 | intronic | . | . | . | 11qB1.1 | rs254794919 | 1 | 0 |
| chr11 | 46842124 | T | G | 228 | Timd4 | intronic | . | . | . | 11qB1.1 | rs223843551 | 1 | 0 |
| chr11 | 46979238 | G | A | 228 | Sgcd | UTR3 | . | . | . | 11qB1.1 | rs26965025 | 1 | 0 |
| chr11 | 46979246 | C | T | 228 | Sgcd | UTR3 | . | . | . | 11qB1.1 | rs259971479 | 1 | 0 |
| chr11 | 46979533 | G | A | 228 | Sgcd | intronic | . | . | . | 11qB1.1 | rs239662953 | 1 | 0 |
| chr11 | 46979534 | A | C | 228 | Sgcd | intronic | . | . | . | 11qB1.1 | rs259046685 | 1 | 0 |
| chr11 | 46980969 | C | T | 228 | Sgcd | intronic | . | . | . | 11qB1.1 | rs26965015 | 1 | 0 |
| chr11 | 47125324 | T | C | 142 | Sgcd | intronic | . | . | . | 11qB1.1 | rs26951171 | 1 | 0 |
| chr11 | 47125358 | C | A | 160 | Sgcd | intronic | . | . | . | 11qB1.1 | rs233200664 | 1 | 0 |
| chr11 | 47125443 | G | A | 212 | Sgcd | intronic | . | . | . | 11qB1.1 | rs260257684 | 1 | 0 |
| chr11 | 47125498 | T | C | 228 | Sgcd | intronic | . | . | . | 11qB1.1 | rs51034496 | 1 | 0 |
| chr11 | 47125705 | C | A | 228 | Sgcd | intronic | . | . | . | 11qB1.1 | rs49262791 | 1 | 0 |
| chr11 | 47125707 | G | A | 228 | Sgcd | intronic | . | . | . | 11qB1.1 | rs47523346 | 1 | 0 |
| chr11 | 47132653 | G | A | 228 | Sgcd | exonic | synonymous SNV | Sgcd:NM_011891:exon4:c.C324T:p.N108N | . | 11qB1.1 | rs26951129 | 1 | 0 |
| chr11 | 47355014 | A | G | 107 | Sgcd | intronic | . | . | . | 11qB1.1 | . | 0 | 1 |
| chr11 | 48734604 | A | G | 228 | . | intergenic | . | . | . | 11qB1.2 | rs220572825 | 1 | 0 |
| chr11 | 48735176 | G | A | 228 | . | intergenic | . | . | . | 11qB1.2 | rs585504349 | 1 | 0 |
| chr11 | 48735316 | A | G | 228 | . | intergenic | . | . | . | 11qB1.2 | rs26974922 | 1 | 0 |
| chr11 | 48735632 | G | A | 228 | . | intergenic | . | . | . | 11qB1.2 | rs265147389 | 1 | 0 |
| chr11 | 48751684 | G | A | 169 | . | intergenic | . | . | . | 11qB1.2 | rs26974915 | 1 | 0 |
| chr11 | 48751751 | A | G | 185 | . | intergenic | . | . | . | 11qB1.2 | rs26974914 | 1 | 0 |
| chr11 | 48752179 | T | G | 228 | . | intergenic | . | . | . | 11qB1.2 | rs26974913 | 1 | 0 |
| chr11 | 48752974 | G | A | 228 | . | intergenic | . | . | . | 11qB1.2 | rs26974912 | 1 | 0 |
| chr11 | 48803098 | T | G | 228 | Gnb2l1 | intronic | . | . | . | 11qB1.2 | rs26974858 | 1 | 0 |
| chr11 | 48803629 | G | A | 228 | Gnb2l1 | intronic | . | . | . | 11qB1.2 | rs6218263 | 1 | 0 |

| | | | | | | | | | | | | | |
|---|---|---|---|---|---|---|---|---|---|---|---|---|---|
| chr11 | 48889444 | G | T | 221 | Gm5431 | exonic | missense SNV | Gm5431:NM_001024230:exon5:c.C650A:p.S217Y | . | 11qB1.2 | . | 0 | 1 |
| chr11 | 49088413 | T | C | 216 | Olfr56,Ifi47 | intronic | . | . | . | 11qB1.2 | rs218086342 | 1 | 0 |
| chr11 | 49088414 | T | C | 216 | Olfr56,Ifi47 | intronic | . | . | . | 11qB1.2 | rs237926845 | 1 | 0 |
| chr11 | 49095438 | G | A | 228 | Ifi47,Olfr56 | exonic | missense SNV | Ifi47:NM_008330:exon2:c.G31A:p.G11S,Ifi47:NM_001271676:exon3:c.G31A:p.G11S,Ifi47:NM_00127167 | . | 11qB1.2 | rs218338425 | 1 | 0 |
| chr11 | 49095454 | G | A | 228 | Olfr56,Ifi47 | exonic | missense SNV | Ifi47:NM_008330:exon2:c.G47A:p.S16N,Ifi47:NM_001271676:exon3:c.G47A:p.S16N,Ifi47:NM_00127167 | . | 11qB1.2 | rs240663886 | 1 | 0 |
| chr11 | 49095589 | T | G | 228 | Olfr56,Ifi47 | exonic | missense SNV | Ifi47:NM_008330:exon2:c.T182G:p.I61S,Ifi47:NM_001271676:exon3:c.T182G:p.I61S,Ifi47:NM_0012716 | . | 11qB1.2 | rs26960149 | 1 | 0 |
| chr11 | 49095837 | G | A | 228 | Olfr56,Ifi47 | exonic | missense SNV | Ifi47:NM_008330:exon2:c.G430A:p.A144T,Ifi47:NM_001271676:exon3:c.G430A:p.A144T,Ifi47:NM_00127 | . | 11qB1.2 | rs26960148 | 1 | 0 |
| chr11 | 49096052 | A | T | 228 | Olfr56,Ifi47 | exonic | synonymous SNV | Ifi47:NM_008330:exon2:c.A645T:p.R215R,Ifi47:NM_001271676:exon3:c.A645T:p.R215R,Ifi47:NM_00127 | . | 11qB1.2 | rs13463986 | 1 | 0 |
| chr11 | 49096098 | T | C | 228 | Olfr56,Ifi47 | exonic | missense SNV | Ifi47:NM_008330:exon2:c.T691C:p.C231R,Ifi47:NM_001271676:exon3:c.T691C:p.C231R,Ifi47:NM_00127 | . | 11qB1.2 | rs26960147 | 1 | 0 |
| chr11 | 49096223 | G | T | 228 | Ifi47,Olfr56 | exonic | synonymous SNV | Ifi47:NM_008330:exon2:c.G816T:p.S272S,Ifi47:NM_001271676:exon3:c.G816T:p.S272S,Ifi47:NM_00127 | . | 11qB1.2 | rs26960145 | 1 | 0 |
| chr11 | 49096268 | C | T | 228 | Ifi47,Olfr56 | exonic | synonymous SNV | Ifi47:NM_008330:exon2:c.C861T:p.I287I,Ifi47:NM_001271676:exon3:c.C861T:p.I287I,Ifi47:NM_00127 | . | 11qB1.2 | rs26960144 | 1 | 0 |
| chr11 | 49096505 | G | A | 228 | Olfr56,Ifi47 | exonic | synonymous SNV | Ifi47:NM_008330:exon2:c.G1098A:p.A366A,Ifi47:NM_001271676:exon3:c.G1098A:p.A366A,Ifi47:NM_001 | . | 11qB1.2 | rs26960143 | 1 | 0 |
| chr11 | 49096809 | G | A | 228 | Olfr56,Ifi47 | UTR3 | . | . | . | 11qB1.2 | rs26960142 | 1 | 0 |
| chr11 | 49097050 | C | G | 228 | Olfr56 | intronic | . | . | . | 11qB1.2 | rs26960141 | 1 | 0 |
| chr11 | 49097091 | C | T | 135 | Olfr56 | intronic | . | . | . | 11qB1.2 | rs26960140 | 1 | 0 |
| chr11 | 49112737 | T | C | 216 | Olfr56 | intronic | . | . | . | 11qB1.2 | rs234736080 | 1 | 0 |
| chr11 | 49112758 | G | C | 228 | Olfr56 | intronic | . | . | . | 11qB1.2 | rs26960091 | 1 | 0 |
| chr11 | 49113050 | G | A | 228 | Olfr1396,Olfr56 | exonic | synonymous SNV | Olfr1396:NM_146337:exon1:c.C675T:p.T225T | . | 11qB1.2 | rs26960090 | 1 | 0 |
| chr11 | 49113163 | A | G | 228 | Olfr56,Olfr1396 | exonic | synonymous SNV | Olfr1396:NM_146337:exon1:c.T562C:p.L188L | . | 11qB1.2 | rs26960089 | 1 | 0 |
| chr11 | 49113380 | C | G | 228 | Olfr1396,Olfr56 | exonic | missense SNV | Olfr1396:NM_146337:exon1:c.G345C:p.L115F | . | 11qB1.2 | rs244912548 | 1 | 0 |
| chr11 | 49113395 | T | C | 228 | Olfr1396,Olfr56 | exonic | synonymous SNV | Olfr1396:NM_146337:exon1:c.A330G:p.G110G | . | 11qB1.2 | rs212478931 | 1 | 0 |
| chr11 | 49134582 | C | T | 221 | Olfr56 | exonic | missense SNV | Olfr56:NM_010999:exon6:c.C293T:p.P98L | . | 11qB1.2 | . | 0 | 1 |
| chr11 | 49135100 | A | C | 228 | Olfr56 | exonic | synonymous SNV | Olfr56:NM_010999:exon6:c.A811C:p.R271R | . | 11qB1.2 | rs219136857 | 1 | 0 |
| chr11 | 49135233 | G | A | 228 | Olfr56 | UTR3 | . | . | . | 11qB1.2 | rs227572565 | 1 | 0 |
| chr11 | 49148729 | G | A | 228 | Olfr1395 | exonic | synonymous SNV | Olfr1395:NM_146877:exon1:c.G471A:p.G157G | . | 11qB1.2 | rs243464348 | 1 | 0 |
| chr11 | 49149035 | A | G | 228 | Olfr1395 | exonic | synonymous SNV | Olfr1395:NM_146877:exon1:c.A777G:p.T259T | . | 11qB1.2 | rs246333385 | 1 | 0 |
| chr11 | 49149155 | C | G | 228 | Olfr1395 | exonic | synonymous SNV | Olfr1395:NM_146877:exon1:c.C897G:p.T299T | . | 11qB1.2 | rs251631367 | 1 | 0 |
| chr11 | 49149166 | G | A | 228 | Olfr1395 | exonic | missense SNV | Olfr1395:NM_146877:exon1:c.G908A:p.R303K | . | 11qB1.2 | rs250539248 | 1 | 0 |
| chr11 | 49149268 | T | C | 228 | . | downstream | . | . | . | 11qB1.2 | rs253569306 | 1 | 0 |
| chr11 | 49159962 | G | T | 173 | . | upstream | . | . | . | 11qB1.2 | rs26960045 | 1 | 0 |
| chr11 | 49159999 | G | A | 228 | . | upstream | . | . | . | 11qB1.2 | rs228009900 | 1 | 0 |
| chr11 | 49160469 | A | G | 228 | Olfr1394 | exonic | missense SNV | Olfr1394:NM_146276:exon1:c.A454G:p.M152V | . | 11qB1.2 | rs220372282 | 1 | 0 |
| chr11 | 49160543 | G | A | 228 | Olfr1394 | exonic | synonymous SNV | Olfr1394:NM_146276:exon1:c.G528A:p.V176V | . | 11qB1.2 | rs248799166 | 1 | 0 |
| chr11 | 49160585 | C | T | 228 | Olfr1394 | exonic | synonymous SNV | Olfr1394:NM_146276:exon1:c.C570T:p.S190S | . | 11qB1.2 | rs26960044 | 1 | 0 |
| chr11 | 49160819 | A | G | 228 | Olfr1394 | exonic | synonymous SNV | Olfr1394:NM_146276:exon1:c.A804G:p.R268R | . | 11qB1.2 | rs26960042 | 1 | 0 |
| chr11 | 49160966 | C | A | 228 | . | downstream | . | . | . | 11qB1.2 | rs226910703 | 1 | 0 |
| chr11 | 49161013 | G | A | 228 | . | downstream | . | . | . | 11qB1.2 | rs264283943 | 1 | 0 |
| chr11 | 49169423 | A | G | 228 | Btnl9 | exonic | synonymous SNV | Btnl9:NM_172793:exon10:c.T1242C:p.A414A | 518 | 11qB1.2 | rs261884777 | 1 | 0 |
| chr11 | 49169453 | G | C | 228 | Btnl9 | exonic | synonymous SNV | Btnl9:NM_172793:exon10:c.C1212G:p.R404R | 518 | 11qB1.2 | rs26960000 | 1 | 0 |
| chr11 | 49173381 | A | G | 228 | Btnl9 | intronic | . | . | . | 11qB1.2 | rs213379234 | 1 | 0 |
| chr11 | 49173383 | G | A | 228 | Btnl9 | intronic | . | . | . | 11qB1.2 | rs238679397 | 1 | 0 |
| chr11 | 49173437 | T | G | 228 | Btnl9 | intronic | . | . | . | 11qB1.2 | rs248823319 | 1 | 0 |
| chr11 | 49173620 | A | G | 171 | Btnl9 | intronic | . | . | . | 11qB1.2 | rs26959984 | 1 | 0 |
| chr11 | 49173667 | A | G | 164 | Btnl9 | intronic | . | . | . | 11qB1.2 | rs26959983 | 1 | 0 |
| chr11 | 49174656 | T | C | 107 | Btnl9 | intronic | . | . | . | 11qB1.2 | rs26959969 | 1 | 0 |
| chr11 | 49174948 | C | T | 137 | Btnl9 | intronic | . | . | . | 11qB1.2 | rs233839334 | 1 | 0 |
| chr11 | 49175468 | A | C | 228 | Btnl9 | intronic | . | . | . | 11qB1.2 | rs26947065 | 1 | 0 |
| chr11 | 49175507 | T | C | 228 | Btnl9 | intronic | . | . | . | 11qB1.2 | rs251382208 | 1 | 0 |
| chr11 | 49175614 | G | A | 228 | Btnl9 | exonic | synonymous SNV | Btnl9:NM_172793:exon5:c.C819T:p.L273L | . | 11qB1.2 | rs221532473 | 1 | 0 |
| chr11 | 49175621 | A | G | 228 | Btnl9 | exonic | missense SNV | Btnl9:NM_172793:exon5:c.T812C:p.I271T | . | 11qB1.2 | rs239921945 | 1 | 0 |
| chr11 | 49175706 | G | A | 228 | Btnl9 | intronic | . | . | . | 11qB1.2 | rs262282292 | 1 | 0 |
| chr11 | 49175965 | C | T | 228 | Btnl9 | intronic | . | . | . | 11qB1.2 | rs231150918 | 1 | 0 |
| chr11 | 49175966 | T | G | 228 | Btnl9 | intronic | . | . | . | 11qB1.2 | rs248289288 | 1 | 0 |
| chr11 | 49176034 | C | T | 228 | Btnl9 | intronic | . | . | . | 11qB1.2 | rs264788457 | 1 | 0 |
| chr11 | 49176035 | T | C | 228 | Btnl9 | intronic | . | . | . | 11qB1.2 | rs228353324 | 1 | 0 |
| chr11 | 49176158 | T | G | 164 | Btnl9 | intronic | . | . | . | 11qB1.2 | rs248604239 | 1 | 0 |
| chr11 | 49178673 | T | G | 228 | Btnl9 | intronic | . | . | . | 11qB1.2 | rs242466636 | 1 | 0 |
| chr11 | 49178719 | A | G | 228 | Btnl9 | exonic | synonymous SNV | Btnl9:NM_172793:exon4:c.T732C:p.I244I | . | 11qB1.2 | rs258750632 | 1 | 0 |
| chr11 | 49178791 | T | C | 228 | Btnl9 | exonic | synonymous SNV | Btnl9:NM_172793:exon4:c.A660G:p.G220G | . | 11qB1.2 | rs26947058 | 1 | 0 |
| chr11 | 49178887 | A | G | 228 | Btnl9 | exonic | synonymous SNV | Btnl9:NM_172793:exon4:c.T564C:p.G188G | . | 11qB1.2 | rs26947057 | 1 | 0 |
| chr11 | 49179109 | T | C | 160 | Btnl9 | intronic | . | . | . | 11qB1.2 | rs26947056 | 1 | 0 |
| chr11 | 49180583 | C | G | 228 | Btnl9 | exonic | missense SNV | Btnl9:NM_172793:exon3:c.G412C:p.D138H | . | 11qB1.2 | rs26947051 | 1 | 0 |
| chr11 | 49180628 | C | G | 228 | Btnl9 | exonic | missense SNV | Btnl9:NM_172793:exon3:c.G367C:p.V123L | . | 11qB1.2 | rs223033985 | 1 | 0 |
| chr11 | 49180629 | T | G | 228 | Btnl9 | exonic | missense SNV | Btnl9:NM_172793:exon3:c.A366C:p.K122N | . | 11qB1.2 | rs26947050 | 1 | 0 |
| chr11 | 49180874 | A | G | 228 | Btnl9 | exonic | synonymous SNV | Btnl9:NM_172793:exon3:c.T121C:p.L41L | . | 11qB1.2 | rs261413206 | 1 | 0 |
| chr11 | 49180908 | C | A | 228 | Btnl9 | intronic | . | . | . | 11qB1.2 | rs222659457 | 1 | 0 |
| chr11 | 49182756 | G | C | 138 | Btnl9 | intronic | . | . | . | 11qB1.2 | rs250347150 | 1 | 0 |
| chr11 | 49182798 | T | A | 169 | Btnl9 | intronic | . | . | . | 11qB1.2 | rs216570269 | 1 | 0 |
| chr11 | 49182806 | C | T | 145 | Btnl9 | intronic | . | . | . | 11qB1.2 | rs236387749 | 1 | 0 |
| chr11 | 49183078 | G | A | 228 | Btnl9 | UTR5 | . | . | . | 11qB1.2 | rs26947046 | 1 | 0 |
| chr11 | 49183214 | A | G | 168 | Btnl9 | intronic | . | . | . | 11qB1.2 | rs26947045 | 1 | 0 |
| chr11 | 49215451 | T | C | 228 | Zfp62 | exonic | missense SNV | Zfp62:NM_009562:exon4:c.T389C:p.L130S,Zfp62:NM_001024846:exon5:c.T368C:p.L123S | . | 11qB1.2 | rs26946979 | 1 | 0 |
| chr11 | 49262068 | C | T | 228 | Mgat1 | UTR3 | . | . | . | 11qB1.2 | rs26946870 | 1 | 0 |
| chr11 | 49279948 | C | A | 158 | . | upstream | . | . | . | 11qB1.2 | rs243895552 | 1 | 0 |
| chr11 | 49279956 | C | T | 163 | . | upstream | . | . | . | 11qB1.2 | rs213677772 | 1 | 0 |
| chr11 | 49280404 | G | C | 228 | Olfr1393 | exonic | synonymous SNV | Olfr1393:NM_146471:exon1:c.G255C:p.L85L | . | 11qB1.2 | rs251870123 | 1 | 0 |
| chr11 | 49280695 | T | C | 228 | Olfr1393 | exonic | synonymous SNV | Olfr1393:NM_146471:exon1:c.T546C:p.V182V | . | 11qB1.2 | rs578722408 | 1 | 0 |
| chr11 | 49281031 | G | T | 228 | Olfr1393 | exonic | missense SNV | Olfr1393:NM_146471:exon1:c.G882T:p.K294N | . | 11qB1.2 | rs219260979 | 1 | 0 |
| chr11 | 49281154 | G | A | 228 | . | downstream | . | . | . | 11qB1.2 | rs238226712 | 1 | 0 |
| chr11 | 49293931 | A | G | 198 | Olfr1392 | exonic | synonymous SNV | Olfr1392:NM_146470:exon1:c.A609G:p.A203A | . | 11qB1.2 | . | 0 | 1 |
| chr11 | 49294204 | A | T | 228 | Olfr1392 | exonic | missense SNV | Olfr1392:NM_146470:exon1:c.A882T:p.K294N | . | 11qB1.2 | . | 1 | 0 |
| chr11 | 49340654 | G | C | 192 | Olfr1390 | exonic | missense SNV | Olfr1390:NM_147065:exon1:c.G121C:p.G41R | . | 11qB1.2 | . | 0 | 1 |
| chr11 | 49430849 | G | T | 228 | Olfr1389 | exonic | synonymous SNV | Olfr1389:NM_147066:exon1:c.G372T:p.A124A | . | 11qB1.2 | rs26976678 | 1 | 0 |
| chr11 | 49443940 | G | A | 228 | Olfr1388 | exonic | missense SNV | Olfr1388:NM_146467:exon1:c.G88A:p.A30T | . | 11qB1.2 | rs26976643 | 1 | 0 |
| chr11 | 49444346 | T | C | 228 | Olfr1388 | exonic | missense SNV | Olfr1388:NM_146467:exon1:c.T494C:p.V165A | . | 11qB1.2 | rs26976642 | 1 | 0 |
| chr11 | 49444508 | A | G | 228 | Olfr1388 | exonic | missense SNV | Olfr1388:NM_146467:exon1:c.A656G:p.H219R | . | 11qB1.2 | rs26976641 | 1 | 0 |
| chr11 | 49459980 | G | A | 228 | Olfr1387 | exonic | synonymous SNV | Olfr1387:NM_146473:exon1:c.G300A:p.Q100Q | . | 11qB1.2 | . | 1 | 0 |
| chr11 | 49514578 | A | G | 228 | Olfr1384 | exonic | synonymous SNV | Olfr1384:NM_146472:exon1:c.A939G:p.R313R | . | 11qB1.2 | rs26976543 | 1 | 0 |
| chr11 | 49625604 | A | G | 228 | Flt4 | intronic | . | . | . | 11qB1.2 | rs26959538 | 1 | 0 |
| chr11 | 49637234 | C | T | 221 | Flt4 | exonic | synonymous SNV | Flt4:NM_008029:exon21:c.C2883T:p.R961R | . | 11qB1.2 | . | 0 | 1 |
| chr11 | 49638091 | C | A | 131 | Flt4 | intronic | . | . | . | 11qB1.2 | . | 0 | 1 |
| chr11 | 49687099 | A | G | 131 | Cnot6 | intronic | . | . | . | 11qB1.2 | . | 1 | 0 |
| chr11 | 49873662 | C | T | 170 | Mapk9 | intronic | . | . | . | 11qB1.3 | rs26996618 | 1 | 0 |
| chr11 | 49874381 | G | A | 228 | Mapk9 | intronic | . | . | . | 11qB1.3 | rs227603188 | 1 | 0 |
| chr11 | 49878560 | A | C | 167 | Mapk9 | intronic | . | . | . | 11qB1.3 | rs239729873 | 1 | 0 |
| chr11 | 49878586 | T | G | 140 | Mapk9 | intronic | . | . | . | 11qB1.3 | rs258599182 | 1 | 0 |
| chr11 | 50052686 | T | G | 196 | Rnf130 | intronic | . | . | . | 11qB1.3 | rs16787288 | 1 | 0 |
| chr11 | 50053061 | G | C | 103 | Rnf130 | intronic | . | . | . | 11qB1.3 | . | 0 | 1 |
| chr11 | 50087603 | A | G | 178 | Rnf130 | intronic | . | . | . | 11qB1.3 | rs26964302 | 1 | 0 |
| chr11 | 50127330 | A | G | 228 | . | intergenic | . | . | . | 11qB1.3 | rs225099043 | 1 | 0 |
| chr11 | 50127459 | A | G | 228 | . | intergenic | . | . | . | 11qB1.3 | rs218930267 | 1 | 0 |
| chr11 | 50135773 | G | C | 137 | Tbc1d9b | intronic | . | . | . | 11qB1.3 | . | 1 | 0 |
| chr11 | 50135924 | T | C | 228 | Tbc1d9b | exonic | missense SNV | Tbc1d9b:NM_001290759:exon2:c.T218C:p.I73T,Tbc1d9b:NM_001290760:exon2:c.T218C:p.I73T,Tbc1d9b:N | . | 11qB1.3 | rs26964190 | 1 | 0 |
| chr11 | 50145287 | G | C | 228 | Tbc1d9b | intronic | . | . | . | 11qB1.3 | . | 1 | 0 |
| chr11 | 50145958 | G | A | 159 | Tbc1d9b | intronic | . | . | . | 11qB1.3 | rs26952165 | 1 | 0 |
| chr11 | 50146410 | A | G | 228 | Tbc1d9b | intronic | . | . | . | 11qB1.3 | rs248807432 | 1 | 0 |
| chr11 | 50146424 | A | G | 228 | Tbc1d9b | intronic | . | . | . | 11qB1.3 | rs254835104 | 1 | 0 |
| chr11 | 50151265 | G | A | 193 | Tbc1d9b | intronic | . | . | . | 11qB1.3 | . | 1 | 0 |
| chr11 | 50151808 | C | T | 223 | Tbc1d9b | intronic | . | . | . | 11qB1.3 | . | 1 | 0 |
| chr11 | 50152628 | C | G | 228 | Tbc1d9b | exonic | synonymous SNV | Tbc1d9b:NM_001290759:exon10:c.C1575G:p.A525A,Tbc1d9b:NM_001290760:exon10:c.C1575G:p.A525A,Tbc | . | 11qB1.3 | . | 1 | 0 |
| chr11 | 50155238 | C | T | 228 | Tbc1d9b | intronic | . | . | . | 11qB1.3 | . | 1 | 0 |
| chr11 | 50156703 | G | C | 228 | Tbc1d9b | intronic | . | . | . | 11qB1.3 | rs231302095 | 1 | 0 |
| chr11 | 50159396 | C | T | 143 | Tbc1d9b | intronic | . | . | . | 11qB1.3 | . | 1 | 0 |
| chr11 | 50159666 | C | A | 228 | Tbc1d9b | exonic | synonymous SNV | Tbc1d9b:NM_001290759:exon15:c.C2439A:p.I813I,Tbc1d9b:NM_001290760:exon15:c.C2442A:p.I814I,Tbc | . | 11qB1.3 | . | 1 | 0 |
| chr11 | 50159730 | G | A | 228 | Tbc1d9b | intronic | . | . | . | 11qB1.3 | . | 1 | 0 |
| chr11 | 50162265 | T | C | 139 | Tbc1d9b | intronic | . | . | . | 11qB1.3 | rs252202555 | 1 | 0 |
| chr11 | 50168576 | G | A | 137 | Tbc1d9b | intronic | . | . | . | 11qB1.3 | . | 1 | 0 |
| chr11 | 50194443 | C | T | 73 | 3010026009Rik | intronic | . | . | . | 11qB1.3 | . | 1 | 0 |
| chr11 | 50207763 | G | A | 228 | Sqstm1 | intronic | . | . | . | 11qB1.3 | . | 1 | 0 |
| chr11 | 50230681 | T | G | 144 | Mgat4b | exonic | synonymous SNV | Mgat4b:NM_145926:exon2:c.T99G:p.G33G | . | 11qB1.3 | . | 0 | 1 |
| chr11 | 50232232 | C | T | 214 | Mgat4b | intronic | . | . | . | 11qB1.3 | . | 1 | 0 |
| chr11 | 50232248 | G | A | 223 | Mgat4b,Mir6919 | ncRNA_exonic | . | . | . | 11qB1.3 | rs242367999 | 1 | 0 |
| chr11 | 50232430 | G | A | 228 | Mgat4b | intronic | . | . | . | 11qB1.3 | . | 1 | 0 |
| chr11 | 50232458 | G | A | 228 | Mgat4b | intronic | . | . | . | 11qB1.3 | . | 1 | 0 |
| chr11 | 50233096 | G | A | 210 | Mgat4b | intronic | . | . | . | 11qB1.3 | . | 0 | 1 |
| chr11 | 50233876 | T | C | 204 | Mgat4b | intronic | . | . | . | 11qB1.3 | rs249643629 | 1 | 0 |
| chr11 | 50263207 | A | G | 101 | Maml1 | intronic | . | . | . | 11qB1.3 | rs26951979 | 1 | 0 |
| chr11 | 50297101 | A | C | 228 | Canx | exonic | missense SNV | Canx:NM_001110499:exon14:c.T1679G:p.V560G,Canx:NM_001110500:exon14:c.T1679G:p.V560G,Canx:NM_( | . | 11qB1.3 | rs13473377 | 1 | 0 |
| chr11 | 50304652 | A | G | 147 | Canx | intronic | . | . | . | 11qB1.3 | rs26951931 | 1 | 0 |
| chr11 | 50307304 | A | G | 151 | Canx | intronic | . | . | . | 11qB1.3 | rs26951929 | 1 | 0 |
| chr11 | 50311963 | T | G | 115 | Canx | intronic | . | . | . | 11qB1.3 | rs217654539 | 1 | 0 |
| chr11 | 50347781 | G | A | 228 | . | intergenic | . | . | . | 11qB1.3 | rs263718762 | 1 | 0 |
| chr11 | 50347893 | G | T | 228 | . | intergenic | . | . | . | 11qB1.3 | rs26951876 | 1 | 0 |
| chr11 | 50395093 | C | A | 195 | Rufy1 | intronic | . | . | . | 11qB1.3 | . | 1 | 0 |
| chr11 | 50414492 | G | A | 228 | Rufy1 | intronic | . | . | . | 11qB1.3 | . | 1 | 0 |
| chr11 | 50419272 | C | T | 131 | Rufy1 | intronic | . | . | . | 11qB1.3 | rs580323471 | 0 | 1 |
| chr11 | 50419751 | A | T | 228 | Rufy1 | intronic | . | . | . | 11qB1.3 | rs221991440 | 1 | 0 |
| chr11 | 50668177 | G | C | 228 | Adamts2 | intronic | . | . | . | 11qB1.3 | rs262303596 | 1 | 0 |
| chr11 | 50668188 | G | C | 228 | Adamts2 | intronic | . | . | . | 11qB1.3 | rs224004220 | 1 | 0 |
| chr11 | 50668260 | T | C | 228 | Adamts2 | intronic | . | . | . | 11qB1.3 | rs248494559 | 1 | 0 |
| chr11 | 50737299 | G | C | 173 | Adamts2 | exonic | synonymous SNV | Adamts2:NM_175643:exon4:c.G825C:p.V275V | . | 11qB1.3 | . | 0 | 1 |
| chr11 | 50756473 | C | T | 151 | Adamts2 | intronic | . | . | . | 11qB1.3 | . | 1 | 0 |
| chr11 | 50756651 | A | G | 228 | Adamts2 | intronic | . | . | . | 11qB1.3 | rs26952258 | 1 | 0 |
| chr11 | 50773089 | A | G | 122 | Adamts2 | intronic | . | . | . | 11qB1.3 | rs26952223 | 1 | 0 |
| chr11 | 50775222 | T | C | 145 | Adamts2 | intronic | . | . | . | 11qB1.3 | rs26952216 | 1 | 0 |
| chr11 | 50775267 | C | T | 160 | Adamts2 | intronic | . | . | . | 11qB1.3 | rs213123086 | 1 | 0 |
| chr11 | 50776041 | T | C | 228 | Adamts2 | intronic | . | . | . | 11qB1.3 | rs26952207 | 1 | 0 |
| chr11 | 50777314 | C | T | 228 | Adamts2 | intronic | . | . | . | 11qB1.3 | rs26952196 | 1 | 0 |
| chr11 | 50779870 | G | A | 228 | Adamts2 | intronic | . | . | . | 11qB1.3 | rs26952185 | 1 | 0 |
| chr11 | 50781873 | C | T | 228 | Adamts2 | exonic | synonymous SNV | Adamts2:NM_175643:exon12:c.C1947T:p.H649H | . | 11qB1.3 | . | 1 | 0 |
| chr11 | 50782011 | G | A | 115 | Adamts2 | intronic | . | . | . | 11qB1.3 | rs252971395 | 1 | 0 |
| chr11 | 50784570 | T | C | 228 | Adamts2 | intronic | . | . | . | 11qB1.3 | rs216167596 | 1 | 0 |
| chr11 | 50785880 | T | C | 160 | Adamts2 | intronic | . | . | . | 11qB1.3 | rs228796369 | 1 | 0 |
| chr11 | 50786501 | C | T | 228 | Adamts2 | intronic | . | . | . | 11qB1.3 | . | 1 | 0 |
| chr11 | 50786505 | T | C | 228 | Adamts2 | intronic | . | . | . | 11qB1.3 | rs258283571 | 1 | 0 |
| chr11 | 50786673 | C | T | 228 | Adamts2 | intronic | . | . | . | 11qB1.3 | rs246181205 | 1 | 0 |
| chr11 | 50786704 | G | A | 228 | Adamts2 | intronic | . | . | . | 11qB1.3 | rs265888097 | 1 | 0 |
| chr11 | 50787286 | A | G | 228 | Adamts2 | exonic | synonymous SNV | Adamts2:NM_175643:exon16:c.A2391G:p.E797E | . | 11qB1.3 | rs254411761 | 1 | 0 |
| chr11 | 50787313 | G | A | 228 | Adamts2 | exonic | synonymous SNV | Adamts2:NM_175643:exon16:c.G2418A:p.T806T | . | 11qB1.3 | . | 1 | 0 |
| chr11 | 50788551 | A | C | 228 | Adamts2 | intronic | . | . | . | 11qB1.3 | rs243653605 | 1 | 0 |
| chr11 | 50788753 | T | C | 228 | Adamts2 | exonic | synonymous SNV | Adamts2:NM_175643:exon17:c.T2559C:p.D853D | . | 11qB1.3 | rs29471547 | 1 | 0 |
| chr11 | 50788935 | G | A | 168 | Adamts2 | intronic | . | . | . | 11qB1.3 | rs29436255 | 1 | 0 |
| chr11 | 50791707 | G | A | 124 | Adamts2 | intronic | . | . | . | 11qB1.3 | rs213190375 | 1 | 0 |
| chr11 | 50791722 | C | T | 171 | Adamts2 | intronic | . | . | . | 11qB1.3 | . | 1 | 0 |
| chr11 | 50791790 | A | T | 228 | Adamts2 | intronic | . | . | . | 11qB1.3 | rs262555443 | 1 | 0 |
| chr11 | 50791855 | A | G | 228 | Adamts2 | exonic | synonymous SNV | Adamts2:NM_175643:exon18:c.A2673G:p.V891V | . | 11qB1.3 | rs224409247 | 1 | 0 |
| chr11 | 50791974 | T | C | 133 | Adamts2 | intronic | . | . | . | 11qB1.3 | rs218757035 | 1 | 0 |
| chr11 | 50792637 | C | T | 211 | Adamts2 | intronic | . | . | . | 11qB1.3 | . | 1 | 0 |
| chr11 | 50792911 | C | A | 119 | Adamts2 | intronic | . | . | . | 11qB1.3 | rs26952180 | 1 | 0 |
| chr11 | 50795095 | T | C | 157 | Adamts2 | intronic | . | . | . | 11qB1.3 | rs6289716 | 1 | 0 |
| chr11 | 50803702 | C | T | 228 | Adamts2 | exonic | synonymous SNV | Adamts2:NM_175643:exon22:c.C3315T:p.D1105D | . | 11qB1.3 | rs26990613 | 1 | 0 |
| chr11 | 50803775 | G | C | 228 | Adamts2 | exonic | missense SNV | Adamts2:NM_175643:exon22:c.G3388C:p.V1130L | . | 11qB1.3 | rs26990612 | 1 | 0 |
| chr11 | 50816153 | G | A | 165 | Zfp354c | intronic | . | . | . | 11qB1.3 | rs29471863 | 1 | 0 |
| chr11 | 50816155 | G | A | 165 | Zfp354c | intronic | . | . | . | 11qB1.3 | rs45669472 | 1 | 0 |
| chr11 | 50817102 | T | C | 228 | Zfp354c | intronic | . | . | . | 11qB1.3 | rs26975522 | 1 | 0 |
| chr11 | 50817379 | A | G | 144 | Zfp354c | intronic | . | . | . | 11qB1.3 | rs29419030 | 1 | 0 |
| chr11 | 50826345 | A | G | 228 | Zfp354c | intronic | . | . | . | 11qB1.3 | rs51365228 | 1 | 0 |
| chr11 | 50833599 | C | A | 190 | Zfp879 | exonic | missense SNV | Zfp879:NM_001290779:exon4:c.G410T:p.G137V,Zfp879:NM_173387:exon5:c.G629T:p.G210V | . | 11qB1.3 | . | 1 | 0 |
| chr11 | 50838549 | A | G | 134 | Zfp879 | intronic | . | . | . | 11qB1.3 | . | 0 | 1 |
| chr11 | 50840475 | G | A | 228 | Zfp879 | intronic | . | . | . | 11qB1.3 | . | 1 | 0 |
| chr11 | 50851650 | C | T | 228 | Grm6 | intronic | . | . | 665 | 11qB1.3 | . | 1 | 0 |
| chr11 | 50853321 | A | C | 228 | Grm6 | exonic | synonymous SNV | Grm6:NM_173372:exon3:c.A618C:p.G206G | . | 11qB1.3 | . | 1 | 0 |
| chr11 | 50853363 | T | C | 228 | Grm6 | exonic | synonymous SNV | Grm6:NM_173372:exon3:c.T660C:p.G220G | . | 11qB1.3 | rs215646678 | 1 | 0 |
| chr11 | 50853520 | G | A | 210 | Grm6 | intronic | . | . | . | 11qB1.3 | . | 1 | 0 |
| chr11 | 50853878 | C | T | 228 | Grm6 | intronic | . | . | . | 11qB1.3 | rs585437677 | 1 | 0 |

| | | | | | | | | | | | | | |
|---|---|---|---|---|---|---|---|---|---|---|---|---|---|
| chr11 | 50853926 | G | A | 228 | Grm6 | intronic | . | . | . | 11qB1.3 | . | 1 | 0 |
| chr11 | 50854118 | A | C | 228 | Grm6 | intronic | . | . | . | 11qB1.3 | rs26975434 | 1 | 0 |
| chr11 | 50855627 | A | G | 228 | Grm6 | intronic | . | . | . | 11qB1.3 | rs26975433 | 1 | 0 |
| chr11 | 50855732 | G | A | 228 | Grm6 | exonic | synonymous SNV | Grm6:NM_173372:exon5:c.G888A:p.L296L | . | 11qB1.3 | . | 1 | 0 |
| chr11 | 50855867 | T | G | 211 | Grm6 | intronic | . | . | . | 11qB1.3 | rs255286519 | 1 | 0 |
| chr11 | 50857018 | T | C | 228 | Grm6 | exonic | synonymous SNV | Grm6:NM_173372:exon6:c.T1056C:p.A352A | . | 11qB1.3 | rs220775875 | 1 | 0 |
| chr11 | 50857473 | A | G | 228 | Grm6 | intronic | . | . | . | 11qB1.3 | rs262144758 | 1 | 0 |
| chr11 | 50859216 | G | A | 228 | Grm6 | intronic | . | . | . | 11qB1.3 | . | 1 | 0 |
| chr11 | 50860027 | T | C | 228 | Grm6 | exonic | synonymous SNV | Grm6:NM_173372:exon9:c.T2016C:p.I672I | 436 | 11qB1.3 | rs26975425 | 1 | 0 |
| chr11 | 50860054 | G | A | 228 | Grm6 | exonic | synonymous SNV | Grm6:NM_173372:exon9:c.G2043A:p.T681T | 436 | 11qB1.3 | rs250082820 | 1 | 0 |
| chr11 | 50923535 | C | T | 228 | Zfp354b | exonic | missense SNV | Zfp354b:NM_013744:exon5:c.G562A:p.E188K | . | 11qB1.3 | rs244554360 | 1 | 0 |
| chr11 | 50929206 | G | A | 167 | Zfp354b | intronic | . | . | . | 11qB1.3 | . | 1 | 0 |
| chr11 | 50942282 | C | T | 228 | . | intergenic | . | . | . | 11qB1.3 | rs584915243 | 1 | 0 |
| chr11 | 50942836 | A | C | 228 | . | intergenic | . | . | . | 11qB1.3 | rs227433690 | 1 | 0 |
| chr11 | 50950805 | C | A | 154 | . | downstream | . | . | . | 11qB1.3 | rs245512778 | 1 | 0 |
| chr11 | 50950838 | A | C | 143 | Prop1 | UTR3 | . | . | . | 11qB1.3 | rs212625988 | 1 | 0 |
| chr11 | 50951067 | A | C | 228 | Prop1 | exonic | missense SNV | Prop1:NM_008936:exon3:c.T511G:p.S171A | . | 11qB1.3 | rs223200207 | 1 | 0 |
| chr11 | 50951114 | C | G | 228 | Prop1 | exonic | missense SNV | Prop1:NM_008936:exon3:c.G464C:p.G155A | . | 11qB1.3 | rs235672595 | 1 | 0 |
| chr11 | 50951334 | G | A | 228 | Prop1 | intronic | . | . | . | 11qB1.3 | . | 1 | 0 |
| chr11 | 50951981 | G | C | 94 | Prop1 | intronic | . | . | . | 11qB1.3 | . | 1 | 0 |
| chr11 | 50968873 | C | T | 175 | . | upstream | . | . | . | 11qB1.3 | . | 1 | 0 |
| chr11 | 50969539 | A | G | 228 | Olfr1378 | exonic | missense SNV | Olfr1378:NM_146910:exon1:c.A520G:p.M174V | . | 11qB1.3 | rs243346931 | 1 | 0 |
| chr11 | 50969654 | C | T | 228 | Olfr1378 | exonic | missense SNV | Olfr1378:NM_146910:exon1:c.C635T:p.T212I | . | 11qB1.3 | . | 1 | 0 |
| chr11 | 50969939 | G | A | 228 | Olfr1378 | exonic | missense SNV | Olfr1378:NM_146910:exon1:c.G920A:p.R307K | . | 11qB1.3 | . | 1 | 0 |
| chr11 | 50984476 | T | A | 110 | . | upstream | . | . | . | 11qB1.3 | rs26962309 | 1 | 0 |
| chr11 | 50984665 | C | T | 228 | . | upstream | . | . | . | 11qB1.3 | rs213592470 | 1 | 0 |
| chr11 | 50984750 | G | A | 228 | Olfr1377 | exonic | synonymous SNV | Olfr1377:NM_146911:exon1:c.G48A:p.G16G | . | 11qB1.3 | rs215325335 | 1 | 0 |
| chr11 | 50984774 | G | A | 228 | Olfr1377 | exonic | synonymous SNV | Olfr1377:NM_146911:exon1:c.G72A:p.E24E | . | 11qB1.3 | rs233659101 | 1 | 0 |
| chr11 | 50984968 | G | T | 228 | Olfr1377 | exonic | missense SNV | Olfr1377:NM_146911:exon1:c.G266T:p.S89I | . | 11qB1.3 | rs582072209 | 1 | 0 |
| chr11 | 50985239 | C | T | 228 | Olfr1377 | exonic | synonymous SNV | Olfr1377:NM_146911:exon1:c.C537T:p.C179C | . | 11qB1.3 | rs26962302 | 1 | 0 |
| chr11 | 50985389 | A | C | 228 | Olfr1377 | exonic | synonymous SNV | Olfr1377:NM_146911:exon1:c.A687C:p.P229P | . | 11qB1.3 | rs26962299 | 1 | 0 |
| chr11 | 50985787 | G | A | 127 | . | downstream | . | . | . | 11qB1.3 | rs213983841 | 1 | 0 |
| chr11 | 51007070 | T | C | 228 | Olfr51 | exonic | missense SNV | Olfr51:NM_146909:exon1:c.T97C:p.W33R | . | 11qB1.3 | . | 1 | 0 |
| chr11 | 51007195 | A | G | 228 | Olfr51 | exonic | synonymous SNV | Olfr51:NM_146909:exon1:c.A222G:p.P74P | . | 11qB1.3 | rs29452660 | 1 | 0 |
| chr11 | 51007466 | C | T | 228 | Olfr51 | exonic | stopgain | Olfr51:NM_146909:exon1:c.C493T:p.R165X | . | 11qB1.3 | rs26962244 | 1 | 0 |
| chr11 | 51007734 | C | T | 228 | Olfr51 | exonic | missense SNV | Olfr51:NM_146909:exon1:c.C761T:p.A254V | . | 11qB1.3 | . | 1 | 0 |
| chr11 | 51007953 | C | T | 228 | . | downstream | . | . | . | 11qB1.3 | rs245834106 | 1 | 0 |
| chr11 | 51059755 | C | T | 153 | Zfp354a | intronic | . | . | . | 11qB1.3 | rs246376981 | 1 | 0 |
| chr11 | 51059992 | A | C | 228 | Zfp354a | intronic | . | . | . | 11qB1.3 | rs29484135 | 1 | 0 |
| chr11 | 51060064 | A | G | 228 | Zfp354a | intronic | . | . | . | 11qB1.3 | rs29426202 | 1 | 0 |
| chr11 | 51060175 | T | C | 228 | Zfp354a | intronic | . | . | . | 11qB1.3 | rs26962168 | 1 | 0 |
| chr11 | 51060865 | G | C | 228 | Zfp354a | exonic | synonymous SNV | Zfp354a:NM_009329:exon3:c.G39C:p.S13S | . | 11qB1.3 | rs249138235 | 1 | 0 |
| chr11 | 51069156 | T | C | 177 | Zfp354a | intronic | . | . | . | 11qB1.3 | rs217237830 | 1 | 0 |
| chr11 | 51069171 | T | C | 228 | Zfp354a | intronic | . | . | . | 11qB1.3 | rs229734205 | 1 | 0 |
| chr11 | 51234096 | T | A | 212 | . | intergenic | . | . | . | 11qB1.3 | . | 0 | 1 |
| chr11 | 51234235 | C | G | 97 | . | intergenic | . | . | . | 11qB1.3 | rs26975245 | 0 | 1 |
| chr11 | 51234259 | C | G | 219 | . | intergenic | . | . | . | 11qB1.3 | rs26975244 | 0 | 1 |
| chr11 | 51234377 | A | G | 228 | . | intergenic | . | . | . | 11qB1.3 | rs29386506 | 1 | 0 |
| chr11 | 51234867 | C | T | 228 | . | intergenic | . | . | . | 11qB1.3 | rs29438295 | 1 | 0 |
| chr11 | 51249601 | T | C | 228 | . | intergenic | . | . | . | 11qB1.3 | rs214127304 | 1 | 0 |
| chr11 | 51249617 | A | G | 228 | . | intergenic | . | . | . | 11qB1.3 | rs26975186 | 1 | 0 |
| chr11 | 51277825 | A | G | 228 | Clk4 | intronic | . | . | . | 11qB1.3 | rs26975095 | 1 | 0 |
| chr11 | 51278033 | C | A | 185 | Clk4 | intronic | . | . | . | 11qB1.3 | . | 1 | 0 |
| chr11 | 51281557 | A | G | 217 | Clk4 | UTR3 | . | . | . | 11qB1.3 | rs13469438 | 1 | 0 |
| chr11 | 51281604 | C | T | 168 | Clk4 | UTR3 | . | . | . | 11qB1.3 | rs13469437 | 1 | 0 |
| chr11 | 51281643 | A | G | 98 | Clk4 | UTR3 | . | . | . | 11qB1.3 | rs247026762 | 1 | 0 |
| chr11 | 51517123 | G | A | 228 | Col23a1 | intronic | . | . | . | 11qB1.3 | rs257461180 | 1 | 0 |
| chr11 | 51517171 | T | C | 228 | Col23a1 | intronic | . | . | . | 11qB1.3 | rs26990910 | 1 | 0 |
| chr11 | 51519410 | C | A | 172 | Col23a1 | intronic | . | . | . | 11qB1.3 | rs260730242 | 1 | 0 |
| chr11 | 51519428 | T | C | 228 | Col23a1 | intronic | . | . | . | 11qB1.3 | rs229306569 | 1 | 0 |
| chr11 | 51519635 | A | G | 228 | Col23a1 | intronic | . | . | . | 11qB1.3 | rs26990900 | 1 | 0 |
| chr11 | 51519664 | T | C | 115 | Col23a1 | intronic | . | . | . | 11qB1.3 | rs243702730 | 1 | 0 |
| chr11 | 51527714 | T | C | 165 | Col23a1 | intronic | . | . | . | 11qB1.3 | rs249050311 | 1 | 0 |
| chr11 | 51527730 | C | T | 176 | Col23a1 | intronic | . | . | . | 11qB1.3 | rs219538905 | 1 | 0 |
| chr11 | 51528053 | A | G | 154 | Col23a1 | intronic | . | . | . | 11qB1.3 | rs248792847 | 1 | 0 |
| chr11 | 51551064 | G | A | 228 | Col23a1 | intronic | . | . | . | 11qB1.3 | rs261767221 | 1 | 0 |
| chr11 | 51557524 | T | C | 146 | Col23a1 | exonic | synonymous SNV | Col23a1:NM_153393:exon9:c.T543C:p.P181P | . | 11qB1.3 | rs250439072 | 1 | 0 |
| chr11 | 51558207 | A | G | 135 | Col23a1 | intronic | . | . | . | 11qB1.3 | rs221457805 | 1 | 0 |
| chr11 | 51561870 | T | C | 228 | Col23a1 | intronic | . | . | . | 11qB1.3 | rs216933016 | 1 | 0 |
| chr11 | 51562794 | G | A | 155 | Col23a1 | intronic | . | . | . | 11qB1.3 | rs238213044 | 1 | 0 |
| chr11 | 51563059 | T | C | 228 | Col23a1 | intronic | . | . | . | 11qB1.3 | rs257930890 | 1 | 0 |
| chr11 | 51563135 | T | C | 205 | Col23a1 | intronic | . | . | . | 11qB1.3 | rs212276066 | 1 | 0 |
| chr11 | 51564494 | C | A | 123 | Col23a1 | intronic | . | . | . | 11qB1.3 | rs229504357 | 1 | 0 |
| chr11 | 51565016 | C | G | 228 | Col23a1 | intronic | . | . | . | 11qB1.3 | rs29395941 | 1 | 0 |
| chr11 | 51565158 | T | C | 228 | Col23a1 | intronic | . | . | . | 11qB1.3 | rs29485489 | 1 | 0 |
| chr11 | 51567716 | C | A | 168 | Col23a1 | intronic | . | . | . | 11qB1.3 | rs262423625 | 1 | 0 |
| chr11 | 51567891 | T | C | 228 | Col23a1 | intronic | . | . | . | 11qB1.3 | rs29397329 | 1 | 0 |
| chr11 | 51567899 | G | C | 228 | Col23a1 | exonic | missense SNV | Col23a1:NM_153393:exon16:c.G862C:p.A288P | . | 11qB1.3 | rs29443384 | 1 | 0 |
| chr11 | 51569259 | A | G | 135 | Col23a1 | intronic | . | . | . | 11qB1.3 | rs264745525 | 1 | 0 |
| chr11 | 51571916 | G | A | 165 | Col23a1 | intronic | . | . | . | 11qB1.3 | rs217901508 | 1 | 0 |
| chr11 | 51572022 | A | G | 228 | Col23a1 | intronic | . | . | . | 11qB1.3 | rs29412895 | 1 | 0 |
| chr11 | 51572503 | T | C | 228 | Col23a1 | exonic | synonymous SNV | Col23a1:NM_153393:exon21:c.T1236C:p.P412P | . | 11qB1.3 | rs242601881 | 1 | 0 |
| chr11 | 51572671 | T | C | 163 | Col23a1 | intronic | . | . | . | 11qB1.3 | rs29440189 | 1 | 0 |
| chr11 | 51572798 | C | A | 228 | Col23a1 | intronic | . | . | . | 11qB1.3 | rs215063724 | 1 | 0 |
| chr11 | 51573811 | T | C | 158 | Col23a1 | intronic | . | . | . | 11qB1.3 | rs29442043 | 1 | 0 |
| chr11 | 51573907 | G | A | 228 | Col23a1 | intronic | . | . | . | 11qB1.3 | rs29384326 | 1 | 0 |
| chr11 | 51574476 | G | T | 197 | Col23a1 | intronic | . | . | . | 11qB1.3 | . | 0 | 1 |
| chr11 | 51574519 | A | G | 228 | Col23a1 | intronic | . | . | . | 11qB1.3 | rs265648851 | 1 | 0 |
| chr11 | 51576666 | G | A | 127 | Col23a1 | intronic | . | . | . | 11qB1.3 | . | 1 | 0 |
| chr11 | 51576945 | A | G | 228 | Col23a1 | intronic | . | . | . | 11qB1.3 | rs52653739 | 1 | 0 |
| chr11 | 51585664 | C | A | 228 | Phykpl | intronic | . | . | . | 11qB1.3 | . | 1 | 0 |
| chr11 | 51593379 | T | A | 164 | Phykpl | intronic | . | . | . | 11qB1.3 | rs26990869 | 1 | 0 |
| chr11 | 51593924 | A | G | 228 | Phykpl | exonic | missense SNV | Phykpl:NM_028398:exon8:c.A710G:p.H237R | . | 11qB1.3 | rs13465869 | 1 | 0 |
| chr11 | 51594120 | C | T | 228 | Phykpl | exonic | synonymous SNV | Phykpl:NM_028398:exon8:c.C906T:p.T302T | . | 11qB1.3 | rs263031735 | 1 | 0 |
| chr11 | 51598112 | C | A | 170 | Phykpl | intronic | . | . | . | 11qB1.3 | . | 1 | 0 |
| chr11 | 51598643 | T | G | 171 | Phykpl | intronic | . | . | . | 11qB1.3 | . | 0 | 1 |
| chr11 | 51602506 | A | C | 144 | Hnrnpab,Phykpl | intronic | . | . | . | 11qB1.3 | rs231754398 | 1 | 0 |
| chr11 | 51602890 | G | C | 228 | Hnrnpab,Phykpl | UTR3 | . | . | . | 11qB1.3 | rs249189010 | 1 | 0 |
| chr11 | 51603001 | A | T | 170 | Hnrnpab,Phykpl | UTR3 | . | . | . | 11qB1.3 | rs51837246 | 1 | 0 |
| chr11 | 51604674 | G | A | 228 | Hnrnpab | exonic | synonymous SNV | Hnrnpab:NM_001048061:exon4:c.C537T:p.F179F,Hnrnpab:NM_010448:exon4:c.C537T:p.F179F | . | 11qB1.3 | rs13460676 | 1 | 0 |
| chr11 | 51620327 | A | G | 158 | Nhp2 | intronic | . | . | . | 11qB1.3 | rs26975854 | 1 | 0 |
| chr11 | 51622271 | T | A | 177 | Nhp2 | intronic | . | . | . | 11qB1.3 | rs26975845 | 1 | 0 |
| chr11 | 51622600 | G | A | 228 | Nhp2 | intronic | . | . | . | 11qB1.3 | rs26975842 | 1 | 0 |
| chr11 | 51622768 | A | G | 101 | Nhp2 | intronic | . | . | . | 11qB1.3 | rs26975840 | 1 | 0 |
| chr11 | 51626751 | T | G | 228 | Rmnd5b | exonic | synonymous SNV | Rmnd5b:NM_025346:exon6:c.A679C:p.R227R | . | 11qB1.3 | rs26975825 | 1 | 0 |
| chr11 | 51627137 | T | C | 228 | Rmnd5b | intronic | . | . | . | 11qB1.3 | rs29435954 | 1 | 0 |
| chr11 | 51627765 | A | G | 228 | Rmnd5b | splicing | . | . | . | 11qB1.3 | rs29412663 | 1 | 0 |
| chr11 | 51627943 | T | C | 228 | Rmnd5b | exonic | synonymous SNV | Rmnd5b:NM_025346:exon3:c.A222G:p.K74K | . | 11qB1.3 | rs26975820 | 1 | 0 |
| chr11 | 51628078 | C | A | 228 | Rmnd5b | intronic | . | . | . | 11qB1.3 | rs29472104 | 1 | 0 |
| chr11 | 51645473 | G | C | 228 | N4bp3 | exonic | synonymous SNV | N4bp3:NM_145974:exon3:c.C531G:p.G177G | . | 11qB1.3 | rs29404022 | 1 | 0 |
| chr11 | 51712346 | A | G | 228 | Sec24a | exonic | synonymous SNV | Sec24a:NM_001290785:exon16:c.T2271C:p.T757T,Sec24a:NM_175255:exon16:c.T2274C:p.T758T | . | 11qB1.3 | rs26975703 | 1 | 0 |
| chr11 | 51817092 | C | T | 228 | Jade2 | exonic | missense SNV | Jade2:NM_199299:exon12:c.G2293A:p.D765N | . | 11qB1.3 | . | 1 | 0 |
| chr11 | 51817473 | C | T | 228 | Jade2 | exonic | missense SNV | Jade2:NM_199299:exon12:c.G1912A:p.A638T | . | 11qB1.3 | . | 1 | 0 |
| chr11 | 51818533 | C | A | 228 | Jade2 | exonic | missense SNV | Jade2:NM_199299:exon11:c.G1648T:p.V550L | . | 11qB1.3 | . | 1 | 0 |
| chr11 | 51818588 | T | C | 228 | Jade2 | exonic | synonymous SNV | Jade2:NM_199299:exon11:c.A1593G:p.S531S | . | 11qB1.3 | rs26975582 | 1 | 0 |
| chr11 | 51825472 | A | G | 228 | Jade2 | intronic | . | . | . | 11qB1.3 | rs242573995 | 1 | 0 |
| chr11 | 51828198 | C | A | 228 | Jade2 | intronic | . | . | . | 11qB1.3 | . | 1 | 0 |
| chr11 | 51828236 | A | C | 228 | Jade2 | intronic | . | . | . | 11qB1.3 | rs237736227 | 1 | 0 |
| chr11 | 51828491 | T | A | 228 | Jade2 | intronic | . | . | . | 11qB1.3 | rs226314651 | 1 | 0 |
| chr11 | 51828525 | T | C | 213 | Jade2 | intronic | . | . | . | 11qB1.3 | rs252153883 | 1 | 0 |
| chr11 | 51830635 | T | C | 228 | Jade2 | intronic | . | . | . | 11qB1.3 | rs217391777 | 1 | 0 |
| chr11 | 51830692 | T | C | 228 | Jade2 | intronic | . | . | . | 11qB1.3 | rs239624269 | 1 | 0 |
| chr11 | 51831019 | G | A | 110 | Jade2 | intronic | . | . | . | 11qB1.3 | rs256330151 | 1 | 0 |
| chr11 | 51831490 | T | G | 228 | Jade2 | intronic | . | . | . | 11qB1.3 | rs26958750 | 1 | 0 |
| chr11 | 51835792 | A | G | 228 | Jade2 | intronic | . | . | . | 11qB1.3 | rs214012295 | 1 | 0 |
| chr11 | 51848702 | T | C | 107 | Jade2 | intronic | . | . | . | 11qB1.3 | . | 1 | 0 |
| chr11 | 51849029 | T | C | 228 | Jade2 | exonic | missense SNV | Jade2:NM_199299:exon2:c.A23G:p.Y8C | . | 11qB1.3 | . | 1 | 0 |
| chr11 | 52052340 | C | A | 102 | Cdk13 | intronic | . | . | . | 11qB1.3 | . | 1 | 0 |
| chr11 | 52098947 | A | G | 228 | Ppp2ca | UTR5 | . | . | 1111 | 11qB1.3 | rs214389458 | 1 | 0 |
| chr11 | 52113007 | G | A | 172 | Ppp2ca | intronic | . | . | . | 11qB1.3 | . | 1 | 0 |
| chr11 | 52118316 | G | T | 135 | Ppp2ca | intronic | . | . | . | 11qB1.3 | . | 1 | 0 |
| chr11 | 52254052 | C | T | 204 | Tcf7 | intronic | . | . | . | 11qB1.3 | rs8243055 | 1 | 0 |
| chr11 | 52256074 | A | G | 178 | Tcf7 | intronic | . | . | . | 11qB1.3 | rs26977605 | 1 | 0 |
| chr11 | 52256415 | T | A | 228 | Tcf7 | intronic | . | . | . | 11qB1.3 | rs8243077 | 1 | 0 |
| chr11 | 52257651 | A | C | 208 | Tcf7 | intronic | . | . | . | 11qB1.3 | rs26977601 | 1 | 0 |
| chr11 | 52257816 | T | G | 228 | Tcf7 | intronic | . | . | . | 11qB1.3 | rs8243092 | 1 | 0 |
| chr11 | 52261502 | T | C | 228 | Tcf7 | intronic | . | . | . | 11qB1.3 | rs8243121 | 1 | 0 |
| chr11 | 52282058 | A | G | 228 | Tcf7 | intronic | . | . | . | 11qB1.3 | rs8243017 | 1 | 0 |
| chr11 | 52282100 | C | A | 228 | Tcf7 | intronic | . | . | . | 11qB1.3 | rs8243015 | 1 | 0 |
| chr11 | 52282158 | G | C | 228 | Tcf7 | exonic | synonymous SNV | Tcf7:NM_009331:exon2:c.C60G:p.S20S,Tcf7:NM_001313981:exon3:c.C60G:p.S20S | . | 11qB1.3 | rs8243014 | 1 | 0 |
| chr11 | 52374340 | C | T | 228 | Vdac1 | exonic | synonymous SNV | Vdac1:NM_011694:exon2:c.C39T:p.S13S | . | 11qB1.3 | rs235354225 | 1 | 0 |
| chr11 | 52374401 | G | T | 228 | Vdac1 | intronic | . | . | . | 11qB1.3 | rs218726426 | 1 | 0 |
| chr11 | 52374746 | T | C | 155 | Vdac1 | intronic | . | . | . | 11qB1.3 | rs47458186 | 1 | 0 |
| chr11 | 52374759 | G | A | 168 | Vdac1 | intronic | . | . | . | 11qB1.3 | rs48399947 | 1 | 0 |
| chr11 | 52374922 | T | C | 228 | Vdac1 | exonic | synonymous SNV | Vdac1:NM_011694:exon3:c.T72C:p.F24F | . | 11qB1.3 | rs47506460 | 1 | 0 |
| chr11 | 52374940 | T | C | 228 | Vdac1 | exonic | synonymous SNV | Vdac1:NM_011694:exon3:c.T90C:p.D30D | . | 11qB1.3 | rs26977510 | 1 | 0 |
| chr11 | 52375030 | C | T | 228 | Vdac1 | intronic | . | . | . | 11qB1.3 | rs26977509 | 1 | 0 |
| chr11 | 52375073 | G | C | 228 | Vdac1 | intronic | . | . | . | 11qB1.3 | rs26977508 | 1 | 0 |
| chr11 | 52376316 | T | G | 215 | Vdac1 | intronic | . | . | . | 11qB1.3 | rs26977505 | 1 | 0 |
| chr11 | 52376373 | A | C | 228 | Vdac1 | intronic | . | . | . | 11qB1.3 | rs262763804 | 1 | 0 |
| chr11 | 52376615 | G | A | 228 | Vdac1 | intronic | . | . | . | 11qB1.3 | rs236657686 | 1 | 0 |
| chr11 | 52376633 | T | C | 228 | Vdac1 | intronic | . | . | . | 11qB1.3 | rs26977503 | 1 | 0 |
| chr11 | 52376692 | G | A | 228 | Vdac1 | exonic | synonymous SNV | Vdac1:NM_011694:exon5:c.G315A:p.P105P | . | 11qB1.3 | rs26977502 | 1 | 0 |
| chr11 | 52376790 | C | T | 228 | Vdac1 | intronic | . | . | . | 11qB1.3 | rs232550675 | 1 | 0 |
| chr11 | 52387231 | C | T | 127 | Vdac1 | intronic | . | . | . | 11qB1.3 | rs29449696 | 1 | 0 |
| chr11 | 52388297 | A | G | 151 | Vdac1 | intronic | . | . | . | 11qB1.3 | rs51978050 | 1 | 0 |
| chr11 | 52396482 | C | T | 228 | 9530068E07Rik | exonic | synonymous SNV | 9530068E07Rik:NM_153117:exon1:c.C15T:p.A5A | 490 | 11qB1.3 | rs26977461 | 1 | 0 |
| chr11 | 52403073 | C | T | 228 | 9530068E07Rik | exonic | missense SNV | 9530068E07Rik:NM_153117:exon2:c.C164T:p.A55V | . | 11qB1.3 | rs13474288 | 1 | 0 |
| chr11 | 52403347 | C | T | 228 | 9530068E07Rik | exonic | synonymous SNV | 9530068E07Rik:NM_153117:exon2:c.C438T:p.Y146Y | . | 11qB1.3 | rs26977458 | 1 | 0 |
| chr11 | 52403443 | G | C | 228 | 9530068E07Rik | exonic | synonymous SNV | 9530068E07Rik:NM_153117:exon2:c.G534C:p.V178V | . | 11qB1.3 | rs26977457 | 1 | 0 |
| chr11 | 52403628 | T | G | 228 | 9530068E07Rik | intronic | . | . | . | 11qB1.3 | rs26977456 | 1 | 0 |
| chr11 | 52403707 | C | T | 175 | 9530068E07Rik | intronic | . | . | . | 11qB1.3 | rs26977455 | 1 | 0 |
| chr11 | 52406729 | T | C | 127 | 9530068E07Rik | intronic | . | . | . | 11qB1.3 | rs26977452 | 1 | 0 |
| chr11 | 52406831 | T | C | 177 | 9530068E07Rik | intronic | . | . | . | 11qB1.3 | rs50322731 | 1 | 0 |
| chr11 | 52406898 | T | C | 228 | 9530068E07Rik | intronic | . | . | . | 11qB1.3 | rs252289330 | 1 | 0 |
| chr11 | 52407257 | A | T | 193 | 9530068E07Rik | UTR3 | . | . | . | 11qB1.3 | rs29474982 | 1 | 0 |
| chr11 | 52814693 | A | G | 228 | Fst14 | intronic | . | . | . | 11qB1.3 | rs247062680 | 1 | 0 |
| chr11 | 52814736 | C | T | 228 | Fst14 | intronic | . | . | . | 11qB1.3 | rs26976009 | 1 | 0 |
| chr11 | 53133989 | A | G | 228 | Fst14 | intronic | . | . | . | 11qB1.3 | rs237633706 | 1 | 0 |
| chr11 | 53134053 | G | A | 228 | Fst14 | exonic | missense SNV | Fst14:NM_177059:exon7:c.G751A:p.E251K | . | 11qB1.3 | rs256806092 | 1 | 0 |
| chr11 | 53134073 | T | C | 228 | Fst14 | exonic | synonymous SNV | Fst14:NM_177059:exon7:c.T771C:p.T257T | . | 11qB1.3 | rs221083520 | 1 | 0 |
| chr11 | 53134279 | T | A | 228 | Fst14 | intronic | . | . | . | 11qB1.3 | rs265998757 | 1 | 0 |
| chr11 | 53148795 | A | G | 228 | Fst14 | intronic | . | . | . | 11qB1.3 | rs258147606 | 1 | 0 |
| chr11 | 53148824 | G | T | 228 | Fst14 | intronic | . | . | . | 11qB1.3 | rs221221958 | 1 | 0 |
| chr11 | 53148826 | C | T | 228 | Fst14 | intronic | . | . | . | 11qB1.3 | rs241099652 | 1 | 0 |
| chr11 | 53149132 | A | G | 121 | Fst14 | intronic | . | . | . | 11qB1.3 | rs236857338 | 1 | 0 |
| chr11 | 53162778 | T | C | 228 | Fst14 | intronic | . | . | . | 11qB1.3 | rs239952827 | 1 | 0 |
| chr11 | 53164288 | A | G | 177 | Fst14 | intronic | . | . | . | 11qB1.3 | rs26968831 | 1 | 0 |
| chr11 | 53168135 | T | A | 228 | Fst14 | intronic | . | . | . | 11qB1.3 | rs26968808 | 1 | 0 |
| chr11 | 53176839 | T | C | 228 | Fst14 | intronic | . | . | . | 11qB1.3 | rs255064153 | 1 | 0 |
| chr11 | 53262480 | T | G | 228 | Hspa4 | intronic | . | . | . | 11qB1.3 | rs229040053 | 1 | 0 |
| chr11 | 53324596 | A | G | 228 | . | upstream | . | . | . | 11qB1.3 | rs26945427 | 1 | 0 |
| chr11 | 53327201 | T | C | 214 | Zcchc10 | intronic | . | . | . | 11qB1.3 | rs26945421 | 1 | 0 |
| chr11 | 53375483 | A | C | 176 | Aff4 | intronic | . | . | . | 11qB1.3 | rs227759556 | 1 | 0 |
| chr11 | 53397716 | G | A | 194 | Aff4 | intronic | . | . | . | 11qB1.3 | rs216793937 | 1 | 0 |

| | | | | | | | | | | | | | |
|---|---|---|---|---|---|---|---|---|---|---|---|---|---|
| chr11 | 53398025 | T | A | 228 | Aff4 | intronic | . | . | . | 11qB1.3 | rs13485267 | 1 | 0 |
| chr11 | 53398145 | C | T | 227 | Aff4 | intronic | . | . | . | 11qB1.3 | rs251125015 | 1 | 0 |
| chr11 | 53407871 | T | A | 228 | Aff4 | intronic | . | . | . | 11qB1.3 | rs230317923 | 1 | 0 |
| chr11 | 53428789 | G | T | 98 | . | downstream | . | . | . | 11qB1.3 | rs216400880 | 1 | 0 |
| chr11 | 53428942 | A | G | 228 | . | downstream | . | . | . | 11qB1.3 | rs26945340 | 1 | 0 |
| chr11 | 53429149 | C | T | 228 | Uqcrq | exonic | synonymous SNV | Uqcrq:NM_025352:exon2:c.G156A:p.P52P | . | 11qB1.3 | rs26945339 | 1 | 0 |
| chr11 | 53430292 | T | C | 208 | Uqcrq | intronic | . | . | . | 11qB1.3 | rs263171345 | 1 | 0 |
| chr11 | 53430404 | G | A | 194 | Uqcrq | intronic | . | . | . | 11qB1.3 | rs26945337 | 1 | 0 |
| chr11 | 53430480 | C | G | 228 | Uqcrq | intronic | . | . | 792 | 11qB1.3 | rs13485326 | 1 | 0 |
| chr11 | 53430529 | C | T | 228 | Uqcrq | intronic | . | . | 792 | 11qB1.3 | rs223276739 | 1 | 0 |
| chr11 | 53430691 | G | A | 228 | Uqcrq | UTR5 | . | . | 792 | 11qB1.3 | rs13495507 | 1 | 0 |
| chr11 | 53433398 | C | T | 228 | Gdf9 | UTR5 | . | . | . | 11qB1.3 | rs236187666 | 1 | 0 |
| chr11 | 53436760 | A | T | 221 | Gdf9 | exonic | missense SNV | Gdf9:NM_008110:exon2:c.A542T:p.K181M | . | 11qB1.3 | . | 0 | 1 |
| chr11 | 53437105 | T | C | 228 | Gdf9 | exonic | missense SNV | Gdf9:NM_008110:exon2:c.T887C:p.I296T | . | 11qB1.3 | rs13495517 | 1 | 0 |
| chr11 | 53437361 | C | T | 228 | Gdf9 | exonic | synonymous SNV | Gdf9:NM_008110:exon2:c.C1143T:p.Y381Y | . | 11qB1.3 | rs13485341 | 1 | 0 |
| chr11 | 53463237 | A | G | 228 | Shroom1 | UTR5 | . | . | . | 11qB1.3 | rs26945319 | 1 | 0 |
| chr11 | 53463367 | C | A | 228 | Shroom1 | exonic | missense SNV | Shroom1:NM_001290789:exon2:c.C113A:p.T38K,Shroom1:NM_027917:exon2:c.C113A:p.T38K | 640 | 11qB1.3 | rs26945318 | 1 | 0 |
| chr11 | 53463746 | C | T | 228 | Shroom1 | exonic | synonymous SNV | Shroom1:NM_001290789:exon2:c.C492T:p.R164R,Shroom1:NM_027917:exon2:c.C492T:p.R164R | 640 | 11qB1.3 | rs26945317 | 1 | 0 |
| chr11 | 53463872 | A | G | 228 | Shroom1 | exonic | synonymous SNV | Shroom1:NM_001290789:exon2:c.A618G:p.L206L,Shroom1:NM_027917:exon2:c.A618G:p.L206L | 640 | 11qB1.3 | rs29382357 | 1 | 0 |
| chr11 | 53464091 | G | A | 228 | Shroom1 | exonic | synonymous SNV | Shroom1:NM_001290789:exon2:c.G837A:p.A279A,Shroom1:NM_027917:exon2:c.G837A:p.A279A | . | 11qB1.3 | rs26945316 | 1 | 0 |
| chr11 | 53464220 | G | A | 228 | Shroom1 | intronic | . | . | . | 11qB1.3 | rs230365320 | 1 | 0 |
| chr11 | 53465424 | G | A | 228 | Shroom1 | intronic | . | . | . | 11qB1.3 | rs226127134 | 1 | 0 |
| chr11 | 53466710 | C | G | 228 | Shroom1 | intronic | . | . | 475 | 11qB1.3 | rs26945314 | 1 | 0 |
| chr11 | 53469837 | T | A | 173 | . | intergenic | . | . | . | 11qB1.3 | rs256654721 | 1 | 0 |
| chr11 | 53470038 | G | T | 228 | . | intergenic | . | . | . | 11qB1.3 | rs233013978 | 1 | 0 |
| chr11 | 53478183 | A | G | 228 | Sowaha | UTR3 | . | . | . | 11qB1.3 | rs218396758 | 1 | 0 |
| chr11 | 53534907 | C | T | 228 | 8-9月 | intronic | . | . | . | 11qB1.3 | rs230642419 | 1 | 0 |
| chr11 | 53535192 | G | A | 228 | 8-9月 | intronic | . | . | . | 11qB1.3 | rs252541341 | 1 | 0 |
| chr11 | 53535239 | C | T | 191 | 8-9月 | intronic | . | . | . | 11qB1.3 | rs13485427 | 1 | 0 |
| chr11 | 53535781 | A | G | 101 | 8-9月 | intronic | . | . | . | 11qB1.3 | rs251310515 | 1 | 0 |
| chr11 | 53536043 | C | T | 228 | 8-9月 | exonic | synonymous SNV | Sept8:NM_001252332:exon5:c.C630T:p.N210N,Sept8:NM_001252333:exon5:c.C624T:p.N208N,Sept8:NM_0 | . | 11qB1.3 | rs263113962 | 1 | 0 |
| chr11 | 53536116 | C | G | 228 | 8-9月 | intronic | . | . | . | 11qB1.3 | rs250107935 | 1 | 0 |
| chr11 | 53536476 | C | T | 135 | 8-9月 | intronic | . | . | . | 11qB1.3 | rs223156183 | 1 | 0 |
| chr11 | 53536573 | T | C | 228 | 8-9月 | intronic | . | . | . | 11qB1.3 | rs241823387 | 1 | 0 |
| chr11 | 53536599 | C | A | 228 | 8-9月 | intronic | . | . | . | 11qB1.3 | rs265847139 | 1 | 0 |
| chr11 | 53536648 | T | C | 228 | 8-9月 | splicing | . | . | . | 11qB1.3 | rs234205560 | 1 | 0 |
| chr11 | 53536872 | G | T | 161 | 8-9月 | intronic | . | . | . | 11qB1.3 | rs257791348 | 1 | 0 |
| chr11 | 53536914 | T | C | 96 | 8-9月 | intronic | . | . | . | 11qB1.3 | rs216169291 | 1 | 0 |
| chr11 | 53537144 | T | C | 228 | 8-9月 | intronic | . | . | . | 11qB1.3 | rs219245248 | 1 | 0 |
| chr11 | 53537329 | G | T | 228 | 8-9月 | intronic | . | . | . | 11qB1.3 | rs231051593 | 1 | 0 |
| chr11 | 53537330 | T | C | 228 | 8-9月 | intronic | . | . | . | 11qB1.3 | rs240279244 | 1 | 0 |
| chr11 | 53537677 | A | G | 148 | 8-9月 | intronic | . | . | . | 11qB1.3 | rs248730104 | 1 | 0 |
| chr11 | 53537696 | T | C | 127 | 8-9月 | intronic | . | . | . | 11qB1.3 | rs212054760 | 1 | 0 |
| chr11 | 53537712 | C | T | 132 | 8-9月 | intronic | . | . | . | 11qB1.3 | rs262893947 | 1 | 0 |
| chr11 | 53537923 | G | C | 228 | 8-9月 | intronic | . | . | . | 11qB1.3 | rs221319913 | 1 | 0 |
| chr11 | 53537934 | T | C | 228 | 8-9月 | intronic | . | . | . | 11qB1.3 | rs237444130 | 1 | 0 |
| chr11 | 53539103 | C | G | 198 | 8-9月 | intronic | . | . | . | 11qB1.3 | rs264637817 | 1 | 0 |
| chr11 | 53539192 | G | A | 228 | 8-9月 | intronic | . | . | . | 11qB1.3 | rs217752811 | 1 | 0 |
| chr11 | 53539200 | C | T | 228 | 8-9月 | intronic | . | . | . | 11qB1.3 | rs239626752 | 1 | 0 |
| chr11 | 53540815 | T | C | 162 | 8-9月 | intronic | . | . | . | 11qB1.3 | rs258592441 | 1 | 0 |
| chr11 | 53540862 | T | C | 173 | 8-9月 | intronic | . | . | . | 11qB1.3 | rs215915947 | 1 | 0 |
| chr11 | 53541333 | C | T | 228 | 8-9月 | UTR3 | . | . | . | 11qB1.3 | rs231425913 | 1 | 0 |
| chr11 | 53541343 | T | C | 228 | 8-9月 | UTR3 | . | . | . | 11qB1.3 | rs13495583 | 1 | 0 |
| chr11 | 53548119 | A | G | 101 | . | intergenic | . | . | . | 11qB1.3 | rs255478760 | 1 | 0 |
| chr11 | 53548203 | T | C | 181 | . | intergenic | . | . | . | 11qB1.3 | rs13495597 | 1 | 0 |
| chr11 | 53548472 | A | G | 226 | . | intergenic | . | . | . | 11qB1.3 | rs13495598 | 1 | 0 |
| chr11 | 53578918 | C | T | 221 | Kif3a | exonic | missense SNV | Kif3a:NM_001290805:exon3:c.C355T:p.L119F,Kif3a:NM_001290806:exon3:c.C355T:p.L119F,Kif3a:NM_00 | . | 11qB1.3 | . | 0 | 1 |
| chr11 | 53579038 | T | C | 228 | Kif3a | intronic | . | . | . | 11qB1.3 | rs13485216 | 1 | 0 |
| chr11 | 53579570 | T | C | 160 | Kif3a | intronic | . | . | . | 11qB1.3 | rs26969159 | 1 | 0 |
| chr11 | 53579720 | T | C | 228 | Kif3a | intronic | . | . | . | 11qB1.3 | rs13495440 | 1 | 0 |
| chr11 | 53583335 | G | T | 193 | Kif3a | exonic | missense SNV | Kif3a:NM_001290805:exon6:c.G725T:p.R242M,Kif3a:NM_001290806:exon6:c.G725T:p.R242M,Kif3a:NM_00 | . | 11qB1.3 | . | 0 | 1 |
| chr11 | 53584178 | T | A | 228 | Kif3a | intronic | . | . | . | 11qB1.3 | rs13495454 | 1 | 0 |
| chr11 | 53584455 | C | T | 228 | Kif3a | intronic | . | . | . | 11qB1.3 | rs26969149 | 1 | 0 |
| chr11 | 53588594 | C | T | 228 | Kif3a | intronic | . | . | . | 11qB1.3 | rs229450340 | 1 | 0 |
| chr11 | 53590743 | G | A | 228 | Kif3a | intronic | . | . | . | 11qB1.3 | rs13485049 | 1 | 0 |
| chr11 | 53593359 | A | G | 228 | Kif3a | intronic | . | . | . | 11qB1.3 | rs13485071 | 1 | 0 |
| chr11 | 53593550 | C | G | 31.936 | Kif3a | intronic | . | . | . | 11qB1.3 | . | 1 | 0 |
| chr11 | 53593589 | A | G | 228 | Kif3a | intronic | . | . | . | 11qB1.3 | rs13485072 | 1 | 0 |
| chr11 | 53593774 | A | G | 228 | Kif3a | intronic | . | . | . | 11qB1.3 | rs13495338 | 1 | 0 |
| chr11 | 53594110 | T | C | 228 | Kif3a | intronic | . | . | . | 11qB1.3 | rs261109015 | 1 | 0 |
| chr11 | 53597803 | G | A | 171 | Kif3a | intronic | . | . | . | 11qB1.3 | rs49466647 | 1 | 0 |
| chr11 | 53598028 | T | C | 228 | Kif3a | intronic | . | . | . | 11qB1.3 | rs49947582 | 1 | 0 |
| chr11 | 53598599 | A | G | 228 | Kif3a | intronic | . | . | . | 11qB1.3 | rs13485089 | 1 | 0 |
| chr11 | 53598607 | A | G | 228 | Kif3a | intronic | . | . | . | 11qB1.3 | rs13485090 | 1 | 0 |
| chr11 | 53598822 | T | C | 228 | Kif3a | exonic | missense SNV | Kif3a:NM_001290805:exon16:c.T2039C:p.M680T,Kif3a:NM_001290806:exon17:c.T2027C:p.M676T,Kif3a:N | . | 11qB1.3 | rs217189586 | 1 | 0 |
| chr11 | 53598946 | T | C | 207 | Kif3a | intronic | . | . | . | 11qB1.3 | rs220690542 | 1 | 0 |
| chr11 | 53598950 | T | C | 132 | Kif3a | intronic | . | . | . | 11qB1.3 | rs579430684 | 1 | 0 |
| chr11 | 53600811 | G | T | 121 | Kif3a | intronic | . | . | . | 11qB1.3 | rs247830010 | 1 | 0 |
| chr11 | 53601098 | G | A | 228 | Kif3a | UTR3 | . | . | . | 11qB1.3 | rs253461334 | 1 | 0 |
| chr11 | 53614071 | A | G | 228 | Il4 | intronic | . | . | . | 11qB1.3 | rs13495387 | 1 | 0 |
| chr11 | 53617115 | C | A | 175 | Il4 | intronic | . | . | . | 11qB1.3 | rs13495396 | 1 | 0 |
| chr11 | 53618682 | G | A | 228 | . | upstream | . | . | . | 11qB1.3 | rs261143691 | 1 | 0 |
| chr11 | 53632146 | G | C | 228 | Il13 | exonic | synonymous SNV | Il13:NM_008355:exon4:c.C315G:p.P105P | . | 11qB1.3 | rs227930415 | 1 | 0 |
| chr11 | 53634563 | T | C | 228 | Il13 | exonic | synonymous SNV | Il13:NM_008355:exon1:c.A72G:p.P24P | . | 11qB1.3 | rs234815160 | 1 | 0 |
| chr11 | 53634569 | C | T | 228 | Il13 | exonic | synonymous SNV | Il13:NM_008355:exon1:c.G66A:p.P22P | . | 11qB1.3 | rs261663273 | 1 | 0 |
| chr11 | 53668319 | T | C | 228 | Rad50 | intronic | . | . | . | 11qB1.3 | rs236593727 | 1 | 0 |
| chr11 | 53679134 | A | G | 228 | Rad50 | intronic | . | . | . | 11qB1.3 | rs13495161 | 1 | 0 |
| chr11 | 53692299 | T | C | 228 | Rad50 | exonic | synonymous SNV | Rad50:NM_009012:exon9:c.A1266G:p.E422E | . | 11qB1.3 | rs13484851 | 1 | 0 |
| chr11 | 53692721 | G | C | 180 | Rad50 | exonic | missense SNV | Rad50:NM_009012:exon8:c.C1232G:p.A411G | . | 11qB1.3 | . | 0 | 1 |
| chr11 | 53698271 | C | A | 228 | Rad50 | exonic | missense SNV | Rad50:NM_009012:exon5:c.G599T:p.G200V | . | 11qB1.3 | rs13484893 | 1 | 0 |
| chr11 | 53701888 | A | G | 228 | Rad50 | intronic | . | . | . | 11qB1.3 | rs13484903 | 1 | 0 |
| chr11 | 53702158 | A | G | 228 | Rad50 | intronic | . | . | . | 11qB1.3 | rs221444966 | 1 | 0 |
| chr11 | 53702181 | T | C | 228 | Rad50 | intronic | . | . | . | 11qB1.3 | rs215937349 | 1 | 0 |
| chr11 | 53706800 | G | T | 136 | Rad50 | intronic | . | . | 699 | 11qB1.3 | rs13496008 | 1 | 0 |
| chr11 | 53720947 | C | T | 228 | Il5 | exonic | synonymous SNV | Il5:NM_010558:exon1:c.C111T:p.S37S | . | 11qB1.3 | rs240927651 | 1 | 0 |
| chr11 | 53722043 | T | C | 96 | Il5 | intronic | . | . | . | 11qB1.3 | rs13484752 | 1 | 0 |
| chr11 | 53723769 | G | A | 228 | Il5 | exonic | missense SNV | Il5:NM_010558:exon3:c.G236A:p.R79H | . | 11qB1.3 | rs261635439 | 1 | 0 |
| chr11 | 53723865 | G | A | 228 | Il5 | intronic | . | . | . | 11qB1.3 | rs224626667 | 1 | 0 |
| chr11 | 53723993 | A | G | 228 | Il5 | exonic | synonymous SNV | Il5:NM_010558:exon4:c.A381G:p.T127T | . | 11qB1.3 | rs26969045 | 1 | 0 |
| chr11 | 53774021 | A | G | 228 | Irf1 | intronic | . | . | . | 11qB1.3 | rs13481028 | 1 | 0 |
| chr11 | 53775801 | G | A | 228 | Irf1 | intronic | . | . | . | 11qB1.3 | rs13495949 | 1 | 0 |
| chr11 | 53966857 | T | A | 85 | Slc22a21 | intronic | . | . | . | 11qB1.3 | rs29383265 | 1 | 0 |
| chr11 | 53967115 | C | T | 228 | Slc22a21 | intronic | . | . | . | 11qB1.3 | rs4228721 | 1 | 0 |
| chr11 | 53967199 | G | A | 228 | Slc22a21 | intronic | . | . | . | 11qB1.3 | rs4228718 | 1 | 0 |
| chr11 | 53967381 | C | G | 228 | Slc22a21 | intronic | . | . | . | 11qB1.3 | rs4228712 | 1 | 0 |
| chr11 | 53979407 | T | G | 228 | Slc22a21 | intronic | . | . | . | 11qB1.3 | rs8261515 | 1 | 0 |
| chr11 | 53979884 | C | G | 228 | Slc22a21 | UTR5 | . | . | 946 | 11qB1.3 | rs29428737 | 1 | 0 |
| chr11 | 53983638 | C | T | 228 | Slc22a4 | exonic | missense SNV | Slc22a4:NM_019687:exon10:c.G1634A:p.S545N | . | 11qB1.3 | rs48450683 | 1 | 0 |
| chr11 | 53991817 | T | C | 171 | Slc22a4 | intronic | . | . | . | 11qB1.3 | rs8260618 | 1 | 0 |
| chr11 | 53991889 | C | G | 222 | Slc22a4 | intronic | . | . | . | 11qB1.3 | rs8260620 | 1 | 0 |
| chr11 | 53996199 | C | T | 228 | Slc22a4 | intronic | . | . | . | 11qB1.3 | rs8264413 | 1 | 0 |
| chr11 | 53997460 | C | T | 228 | Slc22a4 | exonic | synonymous SNV | Slc22a4:NM_019687:exon4:c.G741A:p.P247P | . | 11qB1.3 | rs26982718 | 1 | 0 |
| chr11 | 54005764 | G | A | 110 | Slc22a4 | intronic | . | . | . | 11qB1.3 | rs8264376 | 1 | 0 |
| chr11 | 54054865 | C | T | 157 | . | downstream | . | . | . | 11qB1.3 | rs13495752 | 1 | 0 |
| chr11 | 54055025 | A | G | 228 | Pdlim4 | UTR3 | . | . | . | 11qB1.3 | rs13484322 | 1 | 0 |
| chr11 | 54055305 | G | A | 228 | Pdlim4 | intronic | . | . | . | 11qB1.3 | . | 1 | 0 |
| chr11 | 54055419 | G | C | 228 | Pdlim4 | exonic | synonymous SNV | Pdlim4:NM_019417:exon6:c.C726G:p.L242L | . | 11qB1.3 | . | 1 | 0 |
| chr11 | 54056380 | A | G | 228 | Pdlim4 | intronic | . | . | . | 11qB1.3 | rs260752946 | 1 | 0 |
| chr11 | 54059829 | T | C | 211 | Pdlim4 | intronic | . | . | . | 11qB1.3 | rs13484328 | 1 | 0 |
| chr11 | 54060121 | C | T | 174 | Pdlim4 | intronic | . | . | . | 11qB1.3 | rs219786802 | 1 | 0 |
| chr11 | 54063600 | C | T | 228 | Pdlim4 | intronic | . | . | . | 11qB1.3 | rs245601851 | 1 | 0 |
| chr11 | 54063955 | A | T | 136 | Pdlim4 | intronic | . | . | . | 11qB1.3 | rs235561290 | 1 | 0 |
| chr11 | 54068748 | T | C | 127 | Pdlim4 | intronic | . | . | . | 11qB1.3 | rs13496110 | 1 | 0 |
| chr11 | 54110826 | G | A | 181 | P4ha2 | intronic | . | . | . | 11qB1.3 | rs26982608 | 1 | 0 |
| chr11 | 54111396 | C | T | 228 | P4ha2 | intronic | . | . | . | 11qB1.3 | rs216240655 | 1 | 0 |
| chr11 | 54114319 | G | A | 228 | P4ha2 | intronic | . | . | . | 11qB1.3 | rs26982596 | 1 | 0 |
| chr11 | 54117311 | C | T | 187 | P4ha2 | intronic | . | . | . | 11qB1.3 | rs13495671 | 1 | 0 |
| chr11 | 54118993 | C | T | 169 | P4ha2 | intronic | . | . | . | 11qB1.3 | rs26982587 | 1 | 0 |
| chr11 | 54131151 | G | T | 117 | P4ha2 | intronic | . | . | . | 11qB1.3 | . | 0 | 1 |
| chr11 | 54159178 | C | A | 193 | . | intergenic | . | . | . | 11qB1.3 | rs13496126 | 1 | 0 |
| chr11 | 54159462 | G | A | 228 | . | intergenic | . | . | . | 11qB1.3 | rs13496127 | 1 | 0 |
| chr11 | 54159523 | T | G | 228 | . | intergenic | . | . | . | 11qB1.3 | rs13485536 | 1 | 0 |
| chr11 | 54159903 | C | T | 228 | . | intergenic | . | . | . | 11qB1.3 | rs13502497 | 1 | 0 |
| chr11 | 54159949 | G | A | 228 | . | intergenic | . | . | . | 11qB1.3 | rs13485540 | 1 | 0 |
| chr11 | 54159969 | A | G | 228 | . | intergenic | . | . | . | 11qB1.3 | rs26982495 | 1 | 0 |
| chr11 | 54160118 | A | T | 228 | . | intergenic | . | . | . | 11qB1.3 | rs231475144 | 1 | 0 |
| chr11 | 54160124 | A | C | 228 | . | intergenic | . | . | . | 11qB1.3 | rs13496132 | 1 | 0 |
| chr11 | 54199668 | A | T | 190 | 4933405E24Rik | ncRNA_intronic | . | . | . | 11qB1.3 | . | 0 | 1 |
| chr11 | 54247464 | T | C | 204 | Csf2 | UTR3 | . | . | . | 11qB1.3 | rs29482376 | 1 | 0 |
| chr11 | 54247515 | A | G | 228 | Csf2 | UTR3 | . | . | . | 11qB1.3 | rs13496264 | 1 | 0 |
| chr11 | 54247598 | C | A | 228 | Csf2 | exonic | missense SNV | Csf2:NM_009969:exon4:c.G416T:p.G139V | . | 11qB1.3 | rs26945977 | 1 | 0 |
| chr11 | 54248330 | A | G | 228 | Csf2 | intronic | . | . | . | 11qB1.3 | rs13485928 | 1 | 0 |
| chr11 | 54249464 | C | T | 228 | Csf2 | exonic | synonymous SNV | Csf2:NM_009969:exon1:c.G147A:p.T49T | . | 11qB1.3 | rs13485932 | 1 | 0 |
| chr11 | 54371810 | A | G | 228 | Meikin | exonic | synonymous SNV | Meikin:NM_029105:exon3:c.A291G:p.T97T | . | 11qB1.3 | rs13496487 | 1 | 0 |
| chr11 | 54390341 | A | C | 196 | Meikin | intronic | . | . | . | 11qB1.3 | . | 1 | 0 |
| chr11 | 54561087 | A | T | 126 | Rapgef6 | intronic | . | . | . | 11qB1.3 | rs239230376 | 1 | 0 |
| chr11 | 54610569 | C | T | 148 | Rapgef6 | intronic | . | . | . | 11qB1.3 | rs26959041 | 1 | 0 |
| chr11 | 54620164 | A | T | 228 | Rapgef6 | intronic | . | . | . | 11qB1.3 | . | 1 | 0 |
| chr11 | 54625923 | A | T | 228 | Rapgef6 | exonic | synonymous SNV | Rapgef6:NM_001252497:exon5:c.A564T:p.I188I,Rapgef6:NM_001252498:exon5:c.A564T:p.I188I,Rapgef6 | . | 11qB1.3 | rs26959024 | 1 | 0 |
| chr11 | 54631437 | A | G | 228 | Rapgef6 | intronic | . | . | . | 11qB1.3 | rs219845204 | 1 | 0 |
| chr11 | 54631556 | C | T | 126 | Rapgef6 | intronic | . | . | . | 11qB1.3 | rs26959019 | 1 | 0 |
| chr11 | 54634691 | T | C | 228 | Rapgef6 | intronic | . | . | . | 11qB1.3 | rs26959014 | 1 | 0 |
| chr11 | 54635946 | A | T | 228 | Rapgef6 | intronic | . | . | . | 11qB1.3 | rs26959010 | 1 | 0 |
| chr11 | 54639613 | C | T | 160 | Rapgef6 | intronic | . | . | . | 11qB1.3 | . | 1 | 0 |
| chr11 | 54639711 | T | C | 228 | Rapgef6 | intronic | . | . | . | 11qB1.3 | . | 1 | 0 |
| chr11 | 54649260 | A | G | 228 | Rapgef6 | exonic | synonymous SNV | Rapgef6:NM_001252497:exon12:c.A1719G:p.P573P,Rapgef6:NM_001252494:exon17:c.A2160G:p.P720P,Ra | . | 11qB1.3 | rs26958987 | 1 | 0 |
| chr11 | 54649554 | T | C | 110 | Rapgef6 | intronic | . | . | . | 11qB1.3 | rs26958985 | 1 | 0 |
| chr11 | 54664084 | T | C | 228 | Rapgef6 | exonic | synonymous SNV | Rapgef6:NM_175258:exon20:c.T2946C:p.D982D,Rapgef6:NM_001252494:exon21:c.T2961C:p.D987D,Rapgef | . | 11qB1.3 | rs26958936 | 1 | 0 |
| chr11 | 54740303 | G | A | 228 | Cdc42se2 | UTR5 | . | . | . | 11qB1.3 | rs264490261 | 1 | 0 |
| chr11 | 54746498 | C | T | 228 | Cdc42se2 | intronic | . | . | . | 11qB1.3 | rs585383643 | 1 | 0 |
| chr11 | 54746556 | T | C | 228 | Cdc42se2 | intronic | . | . | . | 11qB1.3 | rs26944577 | 1 | 0 |
| chr11 | 54746747 | A | G | 228 | Cdc42se2 | intronic | . | . | . | 11qB1.3 | rs26944575 | 1 | 0 |
| chr11 | 54746807 | T | C | 228 | Cdc42se2 | intronic | . | . | . | 11qB1.3 | rs236139939 | 1 | 0 |
| chr11 | 54746890 | G | A | 228 | Cdc42se2 | intronic | . | . | . | 11qB1.3 | rs26944574 | 1 | 0 |
| chr11 | 54840929 | C | T | 142 | Lyrm7 | UTR3 | . | . | . | 11qB1.3 | rs260704932 | 1 | 0 |
| chr11 | 54840948 | C | T | 152 | Lyrm7 | UTR3 | . | . | . | 11qB1.3 | rs233826427 | 1 | 0 |
| chr11 | 54841102 | A | C | 228 | Lyrm7 | UTR3 | . | . | . | 11qB1.3 | rs251396447 | 1 | 0 |
| chr11 | 54841112 | A | G | 88 | Lyrm7 | UTR3 | . | . | . | 11qB1.3 | rs262843377 | 1 | 0 |
| chr11 | 54841150 | C | T | 228 | Lyrm7 | exonic | synonymous SNV | Lyrm7:NM_029327:exon5:c.G297A:p.A99A | . | 11qB1.3 | rs214951085 | 1 | 0 |
| chr11 | 54841288 | C | A | 228 | Lyrm7 | intronic | . | . | . | 11qB1.3 | rs240706596 | 1 | 0 |
| chr11 | 54841312 | G | A | 228 | Lyrm7 | intronic | . | . | . | 11qB1.3 | rs26944391 | 1 | 0 |
| chr11 | 54848538 | G | A | 223 | Lyrm7 | intronic | . | . | . | 11qB1.3 | rs232187209 | 1 | 0 |
| chr11 | 54848551 | G | A | 228 | Lyrm7 | intronic | . | . | . | 11qB1.3 | rs26988839 | 1 | 0 |
| chr11 | 54850205 | C | T | 176 | Lyrm7 | intronic | . | . | . | 11qB1.3 | rs233311384 | 1 | 0 |
| chr11 | 54850303 | C | T | 228 | Lyrm7 | intronic | . | . | . | 11qB1.3 | rs26988833 | 1 | 0 |
| chr11 | 54850322 | T | G | 228 | Lyrm7 | intronic | . | . | . | 11qB1.3 | rs26988831 | 1 | 0 |
| chr11 | 54850445 | A | G | 228 | Lyrm7 | intronic | . | . | . | 11qB1.3 | rs213604608 | 1 | 0 |
| chr11 | 54850447 | A | G | 228 | Lyrm7 | intronic | . | . | . | 11qB1.3 | rs232137691 | 1 | 0 |
| chr11 | 54850482 | C | T | 228 | Lyrm7 | intronic | . | . | . | 11qB1.3 | rs26988830 | 1 | 0 |
| chr11 | 54853246 | A | G | 228 | Lyrm7 | exonic | synonymous SNV | Lyrm7:NM_029327:exon2:c.T72C:p.N24N | . | 11qB1.3 | rs26988812 | 1 | 0 |
| chr11 | 54853302 | A | G | 228 | Lyrm7 | splicing | . | . | . | 11qB1.3 | rs26988811 | 1 | 0 |
| chr11 | 54866292 | G | A | 140 | . | upstream | . | . | . | 11qB1.3 | rs213481737 | 1 | 0 |
| chr11 | 54866453 | T | C | 228 | Hint1 | UTR5 | . | . | 649 | 11qB1.3 | rs45849267 | 1 | 0 |
| chr11 | 54866483 | G | A | 228 | Hint1 | UTR5 | . | . | 649 | 11qB1.3 | rs221438445 | 1 | 0 |
| chr11 | 54869833 | G | T | 228 | Hint1 | intronic | . | . | . | 11qB1.3 | rs242658947 | 1 | 0 |

| | | | | | | | | | | | | | |
|---|---|---|---|---|---|---|---|---|---|---|---|---|---|
| chr11 | 54869974 | C | T | 228 | Hint1 | intronic | . | . | . | 11qB1.3 | rs29432911 | 1 | 0 |
| chr11 | 54870281 | C | T | 228 | Hint1 | exonic | synonymous SNV | Hint1:NM_008248:exon3:c.C309T:p.D103D | . | 11qB1.3 | rs234624231 | 1 | 0 |
| chr11 | 54870496 | C | T | 169 | Hint1 | UTR3 | . | . | . | 11qB1.3 | rs212459825 | 1 | 0 |
| chr11 | 54870498 | A | C | 169 | . | downstream | . | . | . | 11qB1.3 | rs235619964 | 1 | 0 |
| chr11 | 54902832 | T | C | 158 | . | upstream | . | . | 261 | 11qB1.3 | rs26974008 | 1 | 0 |
| chr11 | 54902873 | C | A | 172 | Gpx3 | UTR5 | . | . | 261 | 11qB1.3 | rs251587740 | 1 | 0 |
| chr11 | 54903049 | T | C | 228 | Gpx3 | exonic | unknown | UNKNOWN | . | 11qB1.3 | rs220530183 | 1 | 0 |
| chr11 | 54906884 | T | A | 115 | Gpx3 | intronic | . | . | . | 11qB1.3 | rs232800358 | 1 | 0 |
| chr11 | 54908593 | C | T | 228 | Gpx3 | exonic | unknown | UNKNOWN | . | 11qB1.3 | rs230490037 | 1 | 0 |
| chr11 | 54910905 | C | T | 228 | Tnip1 | UTR3 | . | . | . | 11qB1.3 | rs6307427 | 1 | 0 |
| chr11 | 54911074 | G | A | 195 | Tnip1 | UTR3 | . | . | . | 11qB1.3 | rs6308086 | 1 | 0 |
| chr11 | 54911354 | A | G | 228 | Tnip1 | UTR3 | . | . | . | 11qB1.3 | rs253966024 | 1 | 0 |
| chr11 | 54915555 | C | G | 228 | Tnip1 | exonic | synonymous SNV | Tnip1:NM_001199276:exon16:c.G1692C:p.G564G,Tnip1:NM_001271455:exon17:c.G1692C:p.G564G,Tnip1:N | . | 11qB1.3 | rs26973990 | 1 | 0 |
| chr11 | 54916701 | T | C | 180 | Tnip1 | intronic | . | . | . | 11qB1.3 | rs230464543 | 1 | 0 |
| chr11 | 54916749 | C | T | 228 | Tnip1 | intronic | . | . | . | 11qB1.3 | rs26973984 | 1 | 0 |
| chr11 | 54917044 | C | T | 228 | Tnip1 | intronic | . | . | . | 11qB1.3 | rs26973983 | 1 | 0 |
| chr11 | 54917284 | G | A | 221 | Tnip1 | exonic | missense SNV | Tnip1:NM_001199276:exon14:c.C1439T:p.A480V,Tnip1:NM_001271455:exon15:c.C1439T:p.A480V,Tnip1:N | . | 11qB1.3 | rs257625771 | 1 | 0 |
| chr11 | 54917975 | T | C | 228 | Tnip1 | intronic | . | . | . | 11qB1.3 | rs26973974 | 1 | 0 |
| chr11 | 54918116 | G | A | 161 | Tnip1 | intronic | . | . | . | 11qB1.3 | rs26973973 | 1 | 0 |
| chr11 | 54918636 | T | C | 228 | Tnip1 | exonic | synonymous SNV | Tnip1:NM_001199276:exon12:c.A1245G:p.L415L,Tnip1:NM_001271455:exon13:c.A1245G:p.L415L,Tnip1:N | . | 11qB1.3 | rs26973972 | 1 | 0 |
| chr11 | 54918853 | A | G | 228 | Tnip1 | intronic | . | . | . | 11qB1.3 | rs26973971 | 1 | 0 |
| chr11 | 54924036 | A | G | 228 | Tnip1 | intronic | . | . | . | 11qB1.3 | rs29452954 | 1 | 0 |
| chr11 | 54924310 | A | T | 228 | Tnip1 | intronic | . | . | . | 11qB1.3 | rs29461951 | 1 | 0 |
| chr11 | 54924541 | T | C | 135 | Tnip1 | intronic | . | . | . | 11qB1.3 | rs26973964 | 1 | 0 |
| chr11 | 54926756 | G | A | 228 | Tnip1 | exonic | missense SNV | Tnip1:NM_001199276:exon8:c.C764T:p.A255V,Tnip1:NM_001271455:exon9:c.C764T:p.A255V,Tnip1:NM_02 | . | 11qB1.3 | rs26973958 | 1 | 0 |
| chr11 | 54928929 | A | G | 117 | Tnip1 | intronic | . | . | . | 11qB1.3 | rs216732862 | 1 | 0 |
| chr11 | 54928940 | A | G | 128 | Tnip1 | intronic | . | . | . | 11qB1.3 | rs235307541 | 1 | 0 |
| chr11 | 54929206 | C | T | 228 | Tnip1 | exonic | synonymous SNV | Tnip1:NM_001199276:exon7:c.G615A:p.T205T,Tnip1:NM_001271455:exon8:c.G615A:p.T205T,Tnip1:NM_02 | . | 11qB1.3 | rs26973948 | 1 | 0 |
| chr11 | 54929385 | A | G | 165 | Tnip1 | intronic | . | . | . | 11qB1.3 | rs26973946 | 1 | 0 |
| chr11 | 54930887 | A | G | 194 | Tnip1 | intronic | . | . | . | 11qB1.3 | rs26973938 | 1 | 0 |
| chr11 | 54930899 | G | C | 198 | Tnip1 | intronic | . | . | . | 11qB1.3 | rs26973937 | 1 | 0 |
| chr11 | 54933904 | C | G | 115 | Tnip1 | intronic | . | . | . | 11qB1.3 | rs26973923 | 1 | 0 |
| chr11 | 54936377 | T | G | 228 | Tnip1 | intronic | . | . | . | 11qB1.3 | rs226817354 | 1 | 0 |
| chr11 | 54936574 | T | G | 228 | Tnip1 | intronic | . | . | . | 11qB1.3 | rs240702983 | 1 | 0 |
| chr11 | 54937901 | C | T | 228 | Tnip1 | intronic | . | . | . | 11qB1.3 | rs26973910 | 1 | 0 |
| chr11 | 54937958 | A | G | 228 | Tnip1 | exonic | missense SNV | Tnip1:NM_001199276:exon3:c.T223C:p.S75P,Tnip1:NM_001271455:exon4:c.T223C:p.S75P,Tnip1:NM_021 | . | 11qB1.3 | rs26973909 | 1 | 0 |
| chr11 | 54938162 | G | A | 228 | Tnip1 | intronic | . | . | . | 11qB1.3 | rs213382961 | 1 | 0 |
| chr11 | 54939662 | G | A | 228 | Tnip1 | exonic | synonymous SNV | Tnip1:NM_001199276:exon2:c.C51T:p.S17S,Tnip1:NM_001271455:exon3:c.C51T:p.S17S,Tnip1:NM_021327 | . | 11qB1.3 | rs29466274 | 1 | 0 |
| chr11 | 54940095 | A | G | 139 | Tnip1 | intronic | . | . | . | 11qB1.3 | rs29442764 | 1 | 0 |
| chr11 | 54940127 | T | C | 161 | Tnip1 | intronic | . | . | . | 11qB1.3 | rs233057844 | 1 | 0 |
| chr11 | 54940230 | C | T | 228 | Tnip1 | intronic | . | . | . | 11qB1.3 | rs252504455 | 1 | 0 |
| chr11 | 54940273 | T | C | 228 | Tnip1 | intronic | . | . | . | 11qB1.3 | rs48188453 | 1 | 0 |
| chr11 | 54940296 | A | G | 228 | Tnip1 | intronic | . | . | . | 11qB1.3 | rs29387439 | 1 | 0 |
| chr11 | 54940470 | T | C | 228 | Tnip1 | intronic | . | . | . | 11qB1.3 | rs219984336 | 1 | 0 |
| chr11 | 54940681 | C | T | 193 | Tnip1 | intronic | . | . | . | 11qB1.3 | rs257811285 | 1 | 0 |
| chr11 | 54940692 | T | G | 217 | Tnip1 | intronic | . | . | . | 11qB1.3 | rs226778241 | 1 | 0 |
| chr11 | 54940825 | C | G | 202 | Tnip1 | exonic | missense SNV | Tnip1:NM_021327:exon2:c.G22C:p.G8R,Tnip1:NM_001199275:exon3:c.G22C:p.G8R,Tnip1:NM_001271456:e | . | 11qB1.3 | rs51531280 | 1 | 0 |
| chr11 | 54940896 | A | G | 122 | Tnip1 | intronic | . | . | . | 11qB1.3 | rs216877311 | 1 | 0 |
| chr11 | 54947367 | C | T | 141 | Tnip1 | intronic | . | . | . | 11qB1.3 | rs234286360 | 1 | 0 |
| chr11 | 54947619 | A | T | 228 | Tnip1 | intronic | . | . | . | 11qB1.3 | rs26973878 | 1 | 0 |
| chr11 | 54947645 | C | G | 228 | Tnip1 | intronic | . | . | . | 11qB1.3 | rs26973877 | 1 | 0 |
| chr11 | 54947723 | C | T | 228 | Tnip1 | intronic | . | . | . | 11qB1.3 | rs26973876 | 1 | 0 |
| chr11 | 54947845 | T | C | 228 | Tnip1 | intronic | . | . | . | 11qB1.3 | rs224553098 | 1 | 0 |
| chr11 | 54947982 | G | A | 228 | Tnip1 | intronic | . | . | . | 11qB1.3 | rs265135422 | 1 | 0 |
| chr11 | 54947991 | A | G | 228 | Tnip1 | intronic | . | . | . | 11qB1.3 | rs231083786 | 1 | 0 |
| chr11 | 54979595 | T | C | 228 | Anxa6 | intronic | . | . | . | 11qB1.3 | rs230289551 | 1 | 0 |
| chr11 | 54979652 | T | C | 180 | Anxa6 | intronic | . | . | . | 11qB1.3 | rs214604749 | 1 | 0 |
| chr11 | 54981538 | C | T | 228 | Anxa6 | intronic | . | . | . | 11qB1.3 | rs230825018 | 1 | 0 |
| chr11 | 54981543 | C | T | 228 | Anxa6 | intronic | . | . | . | 11qB1.3 | rs249466249 | 1 | 0 |
| chr11 | 54981776 | G | A | 228 | Anxa6 | intronic | . | . | . | 11qB1.3 | rs29400603 | 1 | 0 |
| chr11 | 54981850 | C | T | 228 | Anxa6 | intronic | . | . | . | 11qB1.3 | rs249072681 | 1 | 0 |
| chr11 | 54984932 | T | C | 190 | Anxa6 | intronic | . | . | . | 11qB1.3 | rs47934207 | 1 | 0 |
| chr11 | 54985176 | T | C | 228 | Anxa6 | intronic | . | . | . | 11qB1.3 | rs26973768 | 1 | 0 |
| chr11 | 54985248 | A | C | 228 | Anxa6 | intronic | . | . | . | 11qB1.3 | rs26961467 | 1 | 0 |
| chr11 | 54985321 | G | A | 143 | Anxa6 | intronic | . | . | . | 11qB1.3 | rs26961466 | 1 | 0 |
| chr11 | 54985891 | G | A | 130 | Anxa6 | intronic | . | . | . | 11qB1.3 | rs219763787 | 1 | 0 |
| chr11 | 54985900 | C | A | 177 | Anxa6 | intronic | . | . | . | 11qB1.3 | rs228792703 | 1 | 0 |
| chr11 | 54986024 | C | T | 228 | Anxa6 | intronic | . | . | . | 11qB1.3 | rs214565828 | 1 | 0 |
| chr11 | 54986061 | C | T | 228 | Anxa6 | intronic | . | . | . | 11qB1.3 | rs251407185 | 1 | 0 |
| chr11 | 54986108 | T | C | 228 | Anxa6 | intronic | . | . | . | 11qB1.3 | rs216050534 | 1 | 0 |
| chr11 | 54986235 | C | T | 228 | Anxa6 | intronic | . | . | . | 11qB1.3 | rs264286574 | 1 | 0 |
| chr11 | 54987037 | G | A | 228 | Anxa6 | intronic | . | . | . | 11qB1.3 | rs255815090 | 1 | 0 |
| chr11 | 54991451 | C | T | 228 | Anxa6 | intronic | . | . | . | 11qB1.3 | rs238373380 | 1 | 0 |
| chr11 | 54992283 | T | G | 228 | Anxa6 | splicing | . | . | . | 11qB1.3 | rs257291031 | 1 | 0 |
| chr11 | 54992287 | T | C | 228 | Anxa6 | exonic | synonymous SNV | Anxa6:NM_001110211:exon19:c.A1518G:p.T506T,Anxa6:NM_013472:exon19:c.A1518G:p.T506T | . | 11qB1.3 | rs26961459 | 1 | 0 |
| chr11 | 54994595 | C | T | 228 | Anxa6 | intronic | . | . | . | 11qB1.3 | rs230598504 | 1 | 0 |
| chr11 | 54994605 | A | G | 228 | Anxa6 | intronic | . | . | . | 11qB1.3 | rs251179113 | 1 | 0 |
| chr11 | 54994836 | T | A | 228 | Anxa6 | intronic | . | . | . | 11qB1.3 | rs29411852 | 1 | 0 |
| chr11 | 54994866 | A | C | 228 | Anxa6 | intronic | . | . | . | 11qB1.3 | rs226366975 | 1 | 0 |
| chr11 | 54994945 | G | A | 228 | Anxa6 | exonic | synonymous SNV | Anxa6:NM_001110211:exon16:c.C1173T:p.T391T,Anxa6:NM_013472:exon16:c.C1173T:p.T391T | . | 11qB1.3 | rs245572468 | 1 | 0 |
| chr11 | 54995004 | T | C | 228 | Anxa6 | intronic | . | . | . | 11qB1.3 | rs233314134 | 1 | 0 |
| chr11 | 54995125 | C | T | 137 | Anxa6 | intronic | . | . | . | 11qB1.3 | rs225022062 | 1 | 0 |
| chr11 | 54996206 | T | C | 162 | Anxa6 | intronic | . | . | . | 11qB1.3 | rs26961454 | 1 | 0 |
| chr11 | 54996304 | C | T | 228 | Anxa6 | intronic | . | . | . | 11qB1.3 | rs226092384 | 1 | 0 |
| chr11 | 54996350 | A | G | 228 | Anxa6 | exonic | synonymous SNV | Anxa6:NM_001110211:exon15:c.T1101C:p.A367A,Anxa6:NM_013472:exon15:c.T1101C:p.A367A | . | 11qB1.3 | rs26961453 | 1 | 0 |
| chr11 | 54996470 | G | T | 228 | Anxa6 | intronic | . | . | . | 11qB1.3 | rs256193467 | 1 | 0 |
| chr11 | 54998353 | G | A | 160 | Anxa6 | intronic | . | . | . | 11qB1.3 | rs257218648 | 1 | 0 |
| chr11 | 54998355 | T | C | 162 | Anxa6 | intronic | . | . | . | 11qB1.3 | rs226179304 | 1 | 0 |
| chr11 | 54999621 | T | C | 150 | Anxa6 | intronic | . | . | . | 11qB1.3 | rs26961447 | 1 | 0 |
| chr11 | 54999663 | T | C | 201 | Anxa6 | intronic | . | . | . | 11qB1.3 | rs26961446 | 1 | 0 |
| chr11 | 54999940 | T | C | 228 | Anxa6 | intronic | . | . | . | 11qB1.3 | rs213744502 | 1 | 0 |
| chr11 | 55001328 | T | C | 228 | Anxa6 | exonic | synonymous SNV | Anxa6:NM_001110211:exon12:c.A801G:p.L267L,Anxa6:NM_013472:exon12:c.A801G:p.L267L | . | 11qB1.3 | rs26961436 | 1 | 0 |
| chr11 | 55003825 | A | T | 153 | Anxa6 | intronic | . | . | . | 11qB1.3 | rs223536542 | 1 | 0 |
| chr11 | 55003841 | T | C | 171 | Anxa6 | intronic | . | . | . | 11qB1.3 | rs243161494 | 1 | 0 |
| chr11 | 55004570 | A | C | 104 | Anxa6 | intronic | . | . | . | 11qB1.3 | rs263319669 | 1 | 0 |
| chr11 | 55004674 | C | T | 228 | Anxa6 | exonic | synonymous SNV | Anxa6:NM_001110211:exon10:c.G729A:p.L243L,Anxa6:NM_013472:exon10:c.G729A:p.L243L | . | 11qB1.3 | rs26961430 | 1 | 0 |
| chr11 | 55004996 | T | C | 180 | Anxa6 | intronic | . | . | . | 11qB1.3 | rs26961429 | 1 | 0 |
| chr11 | 55005135 | G | A | 228 | Anxa6 | exonic | synonymous SNV | Anxa6:NM_001110211:exon9:c.C618T:p.R206R,Anxa6:NM_013472:exon9:c.C618T:p.R206R | . | 11qB1.3 | rs26961426 | 1 | 0 |
| chr11 | 55005248 | C | G | 228 | Anxa6 | intronic | . | . | . | 11qB1.3 | rs241112014 | 1 | 0 |
| chr11 | 55005255 | A | G | 228 | Anxa6 | intronic | . | . | . | 11qB1.3 | rs29456711 | 1 | 0 |
| chr11 | 55005806 | A | G | 228 | Anxa6 | exonic | synonymous SNV | Anxa6:NM_001110211:exon8:c.T525C:p.D175D,Anxa6:NM_013472:exon8:c.T525C:p.D175D | . | 11qB1.3 | rs26961422 | 1 | 0 |
| chr11 | 55007795 | G | A | 228 | Anxa6 | intronic | . | . | . | 11qB1.3 | rs237548417 | 1 | 0 |
| chr11 | 55009103 | G | A | 228 | Anxa6 | intronic | . | . | . | 11qB1.3 | rs26961415 | 1 | 0 |
| chr11 | 55010900 | G | C | 228 | Anxa6 | intronic | . | . | . | 11qB1.3 | rs26961403 | 1 | 0 |
| chr11 | 55010944 | A | G | 228 | Anxa6 | intronic | . | . | . | 11qB1.3 | rs26961402 | 1 | 0 |
| chr11 | 55011232 | A | G | 67 | Anxa6 | intronic | . | . | . | 11qB1.3 | rs26961401 | 1 | 0 |
| chr11 | 55011678 | T | C | 228 | Anxa6 | intronic | . | . | . | 11qB1.3 | rs29435510 | 1 | 0 |
| chr11 | 55011694 | G | A | 228 | Anxa6 | intronic | . | . | . | 11qB1.3 | rs29407255 | 1 | 0 |
| chr11 | 55011725 | T | C | 228 | Anxa6 | exonic | missense SNV | Anxa6:NM_001110211:exon4:c.A182G:p.N61S,Anxa6:NM_013472:exon4:c.A182G:p.N61S | . | 11qB1.3 | rs26961399 | 1 | 0 |
| chr11 | 55013775 | T | C | 228 | Anxa6 | intronic | . | . | . | 11qB1.3 | rs51202110 | 1 | 0 |
| chr11 | 55013781 | G | A | 228 | Anxa6 | intronic | . | . | . | 11qB1.3 | rs51511692 | 1 | 0 |
| chr11 | 55014049 | C | T | 158 | Anxa6 | intronic | . | . | . | 11qB1.3 | rs26961382 | 1 | 0 |
| chr11 | 55014065 | C | T | 101 | Anxa6 | intronic | . | . | . | 11qB1.3 | rs26961381 | 1 | 0 |
| chr11 | 55021448 | G | T | 228 | Anxa6 | intronic | . | . | . | 11qB1.3 | rs260118789 | 1 | 0 |
| chr11 | 55021526 | G | C | 228 | Anxa6 | intronic | . | . | . | 11qB1.3 | rs26961327 | 1 | 0 |
| chr11 | 55050418 | A | C | 228 | Ccdc69 | UTR3 | . | . | . | 11qB1.3 | rs26961259 | 1 | 0 |
| chr11 | 55050456 | A | G | 228 | Ccdc69 | UTR3 | . | . | . | 11qB1.3 | rs26961258 | 1 | 0 |
| chr11 | 55050718 | T | C | 208 | Ccdc69 | intronic | . | . | . | 11qB1.3 | rs26961257 | 1 | 0 |
| chr11 | 55051129 | A | G | 228 | Ccdc69 | intronic | . | . | . | 11qB1.3 | rs29410426 | 1 | 0 |
| chr11 | 55051311 | G | A | 228 | Ccdc69 | intronic | . | . | . | 11qB1.3 | rs251742804 | 1 | 0 |
| chr11 | 55051323 | C | A | 228 | Ccdc69 | intronic | . | . | . | 11qB1.3 | rs26961254 | 1 | 0 |
| chr11 | 55052166 | A | T | 89 | Ccdc69 | intronic | . | . | . | 11qB1.3 | . | 0 | 1 |
| chr11 | 55052330 | C | T | 228 | Ccdc69 | intronic | . | . | . | 11qB1.3 | rs244324962 | 1 | 0 |
| chr11 | 55052384 | G | A | 228 | Ccdc69 | exonic | synonymous SNV | Ccdc69:NM_177471:exon5:c.C393T:p.H131H | . | 11qB1.3 | rs26961251 | 1 | 0 |
| chr11 | 55052420 | T | C | 228 | Ccdc69 | exonic | synonymous SNV | Ccdc69:NM_177471:exon5:c.A357G:p.L119L | . | 11qB1.3 | rs26961250 | 1 | 0 |
| chr11 | 55052557 | A | G | 228 | Ccdc69 | intronic | . | . | . | 11qB1.3 | rs26961249 | 1 | 0 |
| chr11 | 55052582 | A | T | 161 | Ccdc69 | intronic | . | . | . | 11qB1.3 | rs26961248 | 1 | 0 |
| chr11 | 55052845 | G | A | 228 | Ccdc69 | intronic | . | . | . | 11qB1.3 | rs261421196 | 1 | 0 |
| chr11 | 55053054 | A | G | 228 | Ccdc69 | intronic | . | . | . | 11qB1.3 | rs26961243 | 1 | 0 |
| chr11 | 55053060 | A | G | 228 | Ccdc69 | intronic | . | . | . | 11qB1.3 | rs231261110 | 1 | 0 |
| chr11 | 55054785 | T | C | 154 | Ccdc69 | intronic | . | . | . | 11qB1.3 | rs26961236 | 1 | 0 |
| chr11 | 55054804 | G | C | 162 | Ccdc69 | intronic | . | . | . | 11qB1.3 | rs26961235 | 1 | 0 |
| chr11 | 55055002 | A | G | 228 | Ccdc69 | exonic | synonymous SNV | Ccdc69:NM_177471:exon3:c.T177C:p.D59D | . | 11qB1.3 | rs248051758 | 1 | 0 |
| chr11 | 55055020 | C | T | 228 | Ccdc69 | exonic | synonymous SNV | Ccdc69:NM_177471:exon3:c.G159A:p.E53E | . | 11qB1.3 | rs252079694 | 1 | 0 |
| chr11 | 55055190 | T | C | 156 | Ccdc69 | intronic | . | . | . | 11qB1.3 | rs26961234 | 1 | 0 |
| chr11 | 55060455 | C | T | 228 | Ccdc69 | intronic | . | . | . | 11qB1.3 | rs26961220 | 1 | 0 |
| chr11 | 55060494 | T | C | 228 | Ccdc69 | exonic | missense SNV | Ccdc69:NM_177471:exon2:c.A100G:p.I34V | . | 11qB1.3 | rs26961219 | 1 | 0 |
| chr11 | 55098305 | C | T | 157 | Gm2a | intronic | . | . | . | 11qB1.3 | rs26961168 | 1 | 0 |
| chr11 | 55103770 | C | A | 228 | Gm2a | intronic | . | . | . | 11qB1.3 | rs243882011 | 1 | 0 |
| chr11 | 55108871 | C | A | 228 | Gm2a | intronic | . | . | . | 11qB1.3 | rs216891979 | 1 | 0 |
| chr11 | 55108978 | C | T | 228 | Gm2a | exonic | synonymous SNV | Gm2a:NM_010299:exon3:c.C324T:p.Y108Y | . | 11qB1.3 | rs243664842 | 1 | 0 |
| chr11 | 55109041 | G | T | 228 | Gm2a | exonic | synonymous SNV | Gm2a:NM_010299:exon3:c.G387T:p.L129L | . | 11qB1.3 | rs238195215 | 1 | 0 |
| chr11 | 55109050 | C | T | 228 | Gm2a | exonic | synonymous SNV | Gm2a:NM_010299:exon3:c.C396T:p.Y132Y | . | 11qB1.3 | rs257931054 | 1 | 0 |
| chr11 | 55109308 | C | G | 228 | Gm2a | intronic | . | . | . | 11qB1.3 | rs263009024 | 1 | 0 |
| chr11 | 55109315 | G | A | 228 | Gm2a | intronic | . | . | . | 11qB1.3 | rs232595036 | 1 | 0 |
| chr11 | 55109428 | A | G | 228 | Gm2a | exonic | synonymous SNV | Gm2a:NM_010299:exon4:c.A477G:p.L159L | . | 11qB1.3 | rs260874289 | 1 | 0 |
| chr11 | 55109628 | C | T | 228 | Gm2a | UTR3 | . | . | . | 11qB1.3 | rs243775269 | 1 | 0 |
| chr11 | 55118100 | T | A | 228 | . | intergenic | . | . | . | 11qB1.3 | rs212861882 | 1 | 0 |
| chr11 | 55118104 | G | C | 228 | . | intergenic | . | . | . | 11qB1.3 | rs238054020 | 1 | 0 |
| chr11 | 55118231 | C | T | 228 | . | intergenic | . | . | . | 11qB1.3 | rs26945239 | 1 | 0 |
| chr11 | 55118368 | G | T | 228 | . | intergenic | . | . | . | 11qB1.3 | rs233502163 | 1 | 0 |
| chr11 | 55118445 | T | C | 228 | . | intergenic | . | . | . | 11qB1.3 | rs234377004 | 1 | 0 |
| chr11 | 55118517 | G | A | 228 | . | intergenic | . | . | . | 11qB1.3 | rs222243238 | 1 | 0 |
| chr11 | 55118809 | C | A | 228 | . | intergenic | . | . | . | 11qB1.3 | rs587148634 | 1 | 0 |
| chr11 | 55123792 | A | G | 157 | . | intergenic | . | . | . | 11qB1.3 | rs26945231 | 1 | 0 |
| chr11 | 55124120 | T | A | 119 | . | downstream | . | . | . | 11qB1.3 | rs580705462 | 1 | 0 |
| chr11 | 55124816 | G | A | 228 | . | downstream | . | . | . | 11qB1.3 | rs264745714 | 1 | 0 |
| chr11 | 55125058 | A | G | 228 | Slc36a3 | exonic | synonymous SNV | Slc36a3:NM_172258:exon11:c.T1269C:p.I423I | . | 11qB1.3 | rs26945225 | 1 | 0 |
| chr11 | 55125520 | T | C | 228 | Slc36a3 | intronic | . | . | . | 11qB1.3 | rs6177747 | 1 | 0 |
| chr11 | 55125667 | T | C | 228 | Slc36a3 | exonic | synonymous SNV | Slc36a3:NM_172258:exon10:c.A1101G:p.R367R | . | 11qB1.3 | rs6178321 | 1 | 0 |
| chr11 | 55125718 | G | A | 228 | Slc36a3 | exonic | synonymous SNV | Slc36a3:NM_172258:exon10:c.C1050T:p.A350A | . | 11qB1.3 | rs26945224 | 1 | 0 |
| chr11 | 55125801 | A | G | 228 | Slc36a3 | intronic | . | . | . | 11qB1.3 | rs6178885 | 1 | 0 |
| chr11 | 55125806 | T | C | 228 | Slc36a3 | intronic | . | . | . | 11qB1.3 | rs6178890 | 1 | 0 |
| chr11 | 55131491 | T | A | 228 | Slc36a3 | intronic | . | . | . | 11qB1.3 | rs587227778 | 1 | 0 |
| chr11 | 55131509 | A | G | 228 | Slc36a3 | intronic | . | . | . | 11qB1.3 | rs583863122 | 1 | 0 |
| chr11 | 55135556 | C | T | 210 | Slc36a3 | intronic | . | . | . | 11qB1.3 | rs217195792 | 1 | 0 |
| chr11 | 55137121 | G | A | 157 | Slc36a3 | intronic | . | . | . | 11qB1.3 | rs26945208 | 1 | 0 |
| chr11 | 55137388 | A | G | 228 | Slc36a3 | intronic | . | . | . | 11qB1.3 | rs26945205 | 1 | 0 |
| chr11 | 55142706 | T | A | 228 | Slc36a3 | intronic | . | . | . | 11qB1.3 | rs251343167 | 1 | 0 |
| chr11 | 55142962 | T | A | 172 | Slc36a3 | intronic | . | . | . | 11qB1.3 | rs247719988 | 1 | 0 |
| chr11 | 55142992 | T | C | 161 | Slc36a3 | intronic | . | . | . | 11qB1.3 | rs223903882 | 1 | 0 |
| chr11 | 55146114 | G | A | 228 | Slc36a3 | intronic | . | . | . | 11qB1.3 | rs222820226 | 1 | 0 |
| chr11 | 55146124 | G | C | 228 | Slc36a3 | intronic | . | . | . | 11qB1.3 | rs238880415 | 1 | 0 |
| chr11 | 55148414 | A | G | 166 | Slc36a3 | intronic | . | . | . | 11qB1.3 | rs243176352 | 1 | 0 |
| chr11 | 55148439 | C | T | 208 | Slc36a3 | intronic | . | . | . | 11qB1.3 | rs26945183 | 1 | 0 |
| chr11 | 55148516 | A | C | 228 | Slc36a3 | intronic | . | . | . | 11qB1.3 | rs245888321 | 1 | 0 |
| chr11 | 55148757 | G | C | 211 | Slc36a3 | intronic | . | . | . | 11qB1.3 | rs233728921 | 1 | 0 |
| chr11 | 55148779 | C | A | 197 | Slc36a3 | intronic | . | . | . | 11qB1.3 | rs582427705 | 1 | 0 |
| chr11 | 55149836 | G | A | 221 | Slc36a3 | intronic | . | . | . | 11qB1.3 | . | 0 | 1 |
| chr11 | 55149962 | A | G | 228 | Slc36a3 | intronic | . | . | . | 11qB1.3 | rs214895921 | 1 | 0 |
| chr11 | 55150940 | G | T | 140 | Slc36a3 | intronic | . | . | . | 11qB1.3 | rs213143826 | 1 | 0 |
| chr11 | 55151410 | T | G | 228 | Slc36a3 | UTR5 | . | . | . | 11qB1.3 | rs26945170 | 1 | 0 |
| chr11 | 55159431 | A | G | 228 | Slc36a2 | exonic | synonymous SNV | Slc36a2:NM_153170:exon10:c.T1350C:p.F450F | . | 11qB1.3 | rs239526696 | 1 | 0 |
| chr11 | 55159439 | T | C | 228 | Slc36a2 | exonic | missense SNV | Slc36a2:NM_153170:exon10:c.A1342G:p.M448V | . | 11qB1.3 | rs26945147 | 1 | 0 |
| chr11 | 55159475 | C | T | 228 | Slc36a2 | exonic | missense SNV | Slc36a2:NM_153170:exon10:c.G1306A:p.V436I | . | 11qB1.3 | rs249694195 | 1 | 0 |

| | | | | | | | | | | | | | |
|---|---|---|---|---|---|---|---|---|---|---|---|---|---|
| chr11 | 55162628 | G | A | 178 | Slc36a2 | intronic | . | . | . | 11qB1.3 | rs228937434 | 1 | 0 |
| chr11 | 55162713 | A | G | 228 | Slc36a2 | exonic | synonymous SNV | Slc36a2:NM_153170:exon9:c.T1098C:p.S366S | . | 11qB1.3 | rs26945125 | 1 | 0 |
| chr11 | 55162776 | G | A | 228 | Slc36a2 | exonic | synonymous SNV | Slc36a2:NM_153170:exon9:c.C1035T:p.I345I | . | 11qB1.3 | rs231015454 | 1 | 0 |
| chr11 | 55162779 | A | G | 228 | Slc36a2 | exonic | synonymous SNV | Slc36a2:NM_153170:exon9:c.T1032C:p.G344G | . | 11qB1.3 | rs26945124 | 1 | 0 |
| chr11 | 55162965 | A | G | 160 | Slc36a2 | intronic | . | . | . | 11qB1.3 | rs26945121 | 1 | 0 |
| chr11 | 55162975 | T | A | 156 | Slc36a2 | intronic | . | . | . | 11qB1.3 | rs262732567 | 1 | 0 |
| chr11 | 55164079 | C | T | 177 | Slc36a2 | intronic | . | . | . | 11qB1.3 | rs26945111 | 1 | 0 |
| chr11 | 55168705 | T | C | 228 | Slc36a2 | intronic | . | . | . | 11qB1.3 | rs255446520 | 1 | 0 |
| chr11 | 55170114 | C | T | 228 | Slc36a2 | intronic | . | . | . | 11qB1.3 | rs260352591 | 1 | 0 |
| chr11 | 55170230 | A | G | 148 | Slc36a2 | intronic | . | . | . | 11qB1.3 | rs250890900 | 1 | 0 |
| chr11 | 55174522 | G | A | 153 | Slc36a2 | intronic | . | . | . | 11qB1.3 | rs233946195 | 1 | 0 |
| chr11 | 55174548 | A | G | 167 | Slc36a2 | intronic | . | . | . | 11qB1.3 | rs252852686 | 1 | 0 |
| chr11 | 55174637 | G | C | 228 | Slc36a2 | intronic | . | . | . | 11qB1.3 | rs241501411 | 1 | 0 |
| chr11 | 55174683 | C | T | 228 | Slc36a2 | exonic | synonymous SNV | Slc36a2:NM_153170:exon5:c.G495A:p.A165A | . | 11qB1.3 | rs222771072 | 1 | 0 |
| chr11 | 55174837 | A | G | 151 | Slc36a2 | intronic | . | . | . | 11qB1.3 | rs242016212 | 1 | 0 |
| chr11 | 55174890 | C | T | 112 | Slc36a2 | intronic | . | . | . | 11qB1.3 | rs249790137 | 1 | 0 |
| chr11 | 55179114 | A | C | 143 | Slc36a2 | intronic | . | . | . | 11qB1.3 | rs265957362 | 1 | 0 |
| chr11 | 55179267 | A | G | 228 | Slc36a2 | intronic | . | . | . | 11qB1.3 | rs231665408 | 1 | 0 |
| chr11 | 55179367 | A | G | 228 | Slc36a2 | exonic | missense SNV | Slc36a2:NM_153170:exon4:c.T383C:p.F128S | . | 11qB1.3 | rs245633497 | 1 | 0 |
| chr11 | 55179441 | G | A | 228 | Slc36a2 | intronic | . | . | . | 11qB1.3 | rs215023228 | 1 | 0 |
| chr11 | 55180577 | T | C | 173 | Slc36a2 | intronic | . | . | . | 11qB1.3 | rs263904416 | 1 | 0 |
| chr11 | 55180739 | G | A | 228 | Slc36a2 | exonic | synonymous SNV | Slc36a2:NM_153170:exon3:c.C297T:p.I99I | . | 11qB1.3 | rs26945086 | 1 | 0 |
| chr11 | 55181428 | G | A | 228 | Slc36a2 | intronic | . | . | . | 11qB1.3 | rs259133870 | 1 | 0 |
| chr11 | 55181468 | A | G | 228 | Slc36a2 | intronic | . | . | . | 11qB1.3 | rs26945081 | 1 | 0 |
| chr11 | 55181498 | T | C | 228 | Slc36a2 | intronic | . | . | . | 11qB1.3 | rs264388361 | 1 | 0 |
| chr11 | 55181581 | G | C | 228 | Slc36a2 | exonic | synonymous SNV | Slc36a2:NM_153170:exon2:c.C204G:p.L68L | . | 11qB1.3 | rs242894328 | 1 | 0 |
| chr11 | 55181754 | C | T | 168 | Slc36a2 | intronic | . | . | . | 11qB1.3 | rs225866674 | 1 | 0 |
| chr11 | 55181765 | T | C | 167 | Slc36a2 | intronic | . | . | . | 11qB1.3 | rs244993215 | 1 | 0 |
| chr11 | 55184784 | C | T | 228 | Slc36a2 | intronic | . | . | . | 11qB1.3 | rs6326722 | 1 | 0 |
| chr11 | 55185047 | A | G | 228 | Slc36a2 | UTR5 | . | . | . | 11qB1.3 | rs6328262 | 1 | 0 |
| chr11 | 55213892 | A | G | 228 | Slc36a1 | intronic | . | . | . | 11qB1.3 | rs26944984 | 1 | 0 |
| chr11 | 55213925 | C | T | 228 | Slc36a1 | intronic | . | . | . | 11qB1.3 | rs246732464 | 1 | 0 |
| chr11 | 55214036 | C | G | 228 | Slc36a1 | exonic | synonymous SNV | Slc36a1:NM_153139:exon2:c.C102G:p.P34P | . | 11qB1.3 | rs223484393 | 1 | 0 |
| chr11 | 55214205 | G | A | 228 | Slc36a1 | intronic | . | . | . | 11qB1.3 | rs26944983 | 1 | 0 |
| chr11 | 55219030 | C | T | 228 | Slc36a1 | exonic | synonymous SNV | Slc36a1:NM_153139:exon3:c.C163T:p.L55L | . | 11qB1.3 | rs243555364 | 1 | 0 |
| chr11 | 55219133 | T | A | 228 | Slc36a1 | intronic | . | . | . | 11qB1.3 | rs212854215 | 1 | 0 |
| chr11 | 55219147 | A | C | 228 | Slc36a1 | intronic | . | . | . | 11qB1.3 | rs238029811 | 1 | 0 |
| chr11 | 55219237 | G | A | 118 | Slc36a1 | intronic | . | . | . | 11qB1.3 | rs26989743 | 1 | 0 |
| chr11 | 55219464 | A | C | 184 | Slc36a1 | intronic | . | . | . | 11qB1.3 | rs246369890 | 1 | 0 |
| chr11 | 55219498 | C | A | 228 | Slc36a1 | intronic | . | . | . | 11qB1.3 | rs26989741 | 1 | 0 |
| chr11 | 55219521 | G | A | 228 | Slc36a1 | intronic | . | . | . | 11qB1.3 | rs26989740 | 1 | 0 |
| chr11 | 55219738 | T | C | 228 | Slc36a1 | intronic | . | . | . | 11qB1.3 | rs51827314 | 1 | 0 |
| chr11 | 55219748 | G | A | 228 | Slc36a1 | intronic | . | . | . | 11qB1.3 | rs26989739 | 1 | 0 |
| chr11 | 55220263 | T | G | 228 | Slc36a1 | intronic | . | . | . | 11qB1.3 | rs26989734 | 1 | 0 |
| chr11 | 55220354 | C | T | 228 | Slc36a1 | intronic | . | . | . | 11qB1.3 | rs26989733 | 1 | 0 |
| chr11 | 55220418 | T | C | 228 | Slc36a1 | exonic | synonymous SNV | Slc36a1:NM_153139:exon5:c.T363C:p.Y121Y | . | 11qB1.3 | rs26989732 | 1 | 0 |
| chr11 | 55221801 | C | G | 137 | Slc36a1 | intronic | . | . | . | 11qB1.3 | rs26989720 | 1 | 0 |
| chr11 | 55222133 | G | A | 228 | Slc36a1 | intronic | . | . | . | 11qB1.3 | rs235028362 | 1 | 0 |
| chr11 | 55222150 | C | A | 228 | Slc36a1 | intronic | . | . | . | 11qB1.3 | rs254570249 | 1 | 0 |
| chr11 | 55223475 | A | G | 170 | Slc36a1 | intronic | . | . | . | 11qB1.3 | rs26989713 | 1 | 0 |
| chr11 | 55223658 | G | A | 228 | Slc36a1 | exonic | synonymous SNV | Slc36a1:NM_153139:exon7:c.G561A:p.P187P | . | 11qB1.3 | rs26989712 | 1 | 0 |
| chr11 | 55223772 | C | T | 228 | Slc36a1 | exonic | synonymous SNV | Slc36a1:NM_153139:exon7:c.C675T:p.I225I | . | 11qB1.3 | rs26989711 | 1 | 0 |
| chr11 | 55223888 | C | T | 228 | Slc36a1 | intronic | . | . | . | 11qB1.3 | rs221245580 | 1 | 0 |
| chr11 | 55223890 | G | T | 228 | Slc36a1 | intronic | . | . | . | 11qB1.3 | rs237753421 | 1 | 0 |
| chr11 | 55225105 | C | T | 228 | Slc36a1 | intronic | . | . | . | 11qB1.3 | rs584930905 | 1 | 0 |
| chr11 | 55226175 | G | A | 228 | Slc36a1 | intronic | . | . | . | 11qB1.3 | rs26989697 | 1 | 0 |
| chr11 | 55226213 | G | T | 228 | Slc36a1 | intronic | . | . | . | 11qB1.3 | rs26989696 | 1 | 0 |
| chr11 | 55226241 | T | C | 170 | Slc36a1 | intronic | . | . | . | 11qB1.3 | rs225336429 | 1 | 0 |
| chr11 | 55228033 | A | G | 88 | Slc36a1 | intronic | . | . | . | 11qB1.3 | rs232273571 | 1 | 0 |
| chr11 | 55228132 | T | C | 228 | Slc36a1 | intronic | . | . | . | 11qB1.3 | rs585422956 | 1 | 0 |
| chr11 | 55228428 | T | A | 228 | Slc36a1 | intronic | . | . | . | 11qB1.3 | rs26989681 | 1 | 0 |
| chr11 | 55232306 | A | G | 114 | Slc36a1 | intronic | . | . | . | 11qB1.3 | rs26974358 | 1 | 0 |
| chr11 | 55232311 | C | A | 125 | Slc36a1 | intronic | . | . | . | 11qB1.3 | rs252261091 | 1 | 0 |
| chr11 | 55251859 | G | A | 176 | Fat2 | UTR3 | . | . | . | 11qB1.3 | rs236308656 | 1 | 0 |
| chr11 | 55251862 | C | A | 179 | Fat2 | UTR3 | . | . | . | 11qB1.3 | rs262498095 | 1 | 0 |
| chr11 | 55252209 | A | G | 228 | Fat2 | exonic | synonymous SNV | Fat2:NM_001029988:exon23:c.T12813C:p.N4271N | . | 11qB1.3 | rs243846382 | 1 | 0 |
| chr11 | 55252302 | G | A | 228 | Fat2 | exonic | synonymous SNV | Fat2:NM_001029988:exon23:c.C12720T:p.P4240P | . | 11qB1.3 | rs261843300 | 1 | 0 |
| chr11 | 55253448 | A | G | 173 | Fat2 | intronic | . | . | . | 11qB1.3 | rs234617711 | 1 | 0 |
| chr11 | 55255890 | A | G | 148 | Fat2 | intronic | . | . | . | 11qB1.3 | rs257461346 | 1 | 0 |
| chr11 | 55256236 | G | A | 228 | Fat2 | exonic | missense SNV | Fat2:NM_001029988:exon21:c.C11933T:p.P3978L | . | 11qB1.3 | rs227722417 | 1 | 0 |
| chr11 | 55257024 | T | C | 228 | Fat2 | intronic | . | . | . | 11qB1.3 | rs51908764 | 1 | 0 |
| chr11 | 55260281 | A | G | 160 | Fat2 | intronic | . | . | . | 11qB1.3 | rs48807859 | 1 | 0 |
| chr11 | 55260784 | G | C | 164 | Fat2 | intronic | . | . | . | 11qB1.3 | rs29456156 | 1 | 0 |
| chr11 | 55262017 | T | C | 228 | Fat2 | intronic | . | . | . | 11qB1.3 | rs26974257 | 1 | 0 |
| chr11 | 55262707 | G | T | 228 | Fat2 | exonic | synonymous SNV | Fat2:NM_001029988:exon18:c.C10678A:p.R3560R | . | 11qB1.3 | rs26974254 | 1 | 0 |
| chr11 | 55262762 | C | T | 228 | Fat2 | exonic | synonymous SNV | Fat2:NM_001029988:exon18:c.G10623A:p.T3541T | . | 11qB1.3 | rs26974253 | 1 | 0 |
| chr11 | 55262930 | T | C | 228 | Fat2 | intronic | . | . | . | 11qB1.3 | rs26974251 | 1 | 0 |
| chr11 | 55262983 | G | A | 203 | Fat2 | intronic | . | . | . | 11qB1.3 | rs26974249 | 1 | 0 |
| chr11 | 55265759 | T | C | 170 | Fat2 | intronic | . | . | . | 11qB1.3 | rs26974238 | 1 | 0 |
| chr11 | 55265946 | T | C | 228 | Fat2 | exonic | synonymous SNV | Fat2:NM_001029988:exon17:c.A10428G:p.V3476V | . | 11qB1.3 | rs26974237 | 1 | 0 |
| chr11 | 55265970 | A | G | 228 | Fat2 | exonic | synonymous SNV | Fat2:NM_001029988:exon17:c.T10404C:p.I3468I | . | 11qB1.3 | rs26974236 | 1 | 0 |
| chr11 | 55265979 | A | G | 228 | Fat2 | exonic | synonymous SNV | Fat2:NM_001029988:exon17:c.T10395C:p.F3465F | . | 11qB1.3 | rs29476868 | 1 | 0 |
| chr11 | 55267380 | G | A | 127 | Fat2 | intronic | . | . | . | 11qB1.3 | rs29486680 | 1 | 0 |
| chr11 | 55267545 | A | G | 228 | Fat2 | intronic | . | . | . | 11qB1.3 | rs26974225 | 1 | 0 |
| chr11 | 55267727 | C | T | 228 | Fat2 | intronic | . | . | . | 11qB1.3 | rs26974224 | 1 | 0 |
| chr11 | 55267762 | T | A | 228 | Fat2 | intronic | . | . | . | 11qB1.3 | rs29472411 | 1 | 0 |
| chr11 | 55267897 | C | T | 175 | Fat2 | intronic | . | . | . | 11qB1.3 | rs26974222 | 1 | 0 |
| chr11 | 55268078 | C | T | 197 | Fat2 | intronic | . | . | . | 11qB1.3 | rs46767241 | 1 | 0 |
| chr11 | 55268082 | G | T | 197 | Fat2 | intronic | . | . | . | 11qB1.3 | rs46183693 | 1 | 0 |
| chr11 | 55268361 | T | C | 228 | Fat2 | intronic | . | . | . | 11qB1.3 | rs29417203 | 1 | 0 |
| chr11 | 55268366 | A | G | 228 | Fat2 | intronic | . | . | . | 11qB1.3 | rs26974221 | 1 | 0 |
| chr11 | 55269398 | A | G | 228 | Fat2 | exonic | synonymous SNV | Fat2:NM_001029988:exon14:c.T9888C:p.S3296S | . | 11qB1.3 | rs29464762 | 1 | 0 |
| chr11 | 55269541 | G | A | 209 | Fat2 | intronic | . | . | . | 11qB1.3 | rs253320215 | 1 | 0 |
| chr11 | 55270015 | A | G | 228 | Fat2 | intronic | . | . | . | 11qB1.3 | rs49237418 | 1 | 0 |
| chr11 | 55270179 | G | A | 228 | Fat2 | exonic | synonymous SNV | Fat2:NM_001029988:exon13:c.C9724T:p.L3242L | . | 11qB1.3 | rs258634937 | 1 | 0 |
| chr11 | 55270182 | A | G | 228 | Fat2 | exonic | missense SNV | Fat2:NM_001029988:exon13:c.T9721C:p.Y3241H | . | 11qB1.3 | rs47873811 | 1 | 0 |
| chr11 | 55273053 | G | A | 228 | Fat2 | exonic | synonymous SNV | Fat2:NM_001029988:exon12:c.C9375T:p.F3125F | . | 11qB1.3 | rs26974212 | 1 | 0 |
| chr11 | 55273140 | T | C | 228 | Fat2 | exonic | synonymous SNV | Fat2:NM_001029988:exon12:c.A9288G:p.S3096S | . | 11qB1.3 | rs26974210 | 1 | 0 |
| chr11 | 55273211 | A | T | 228 | Fat2 | exonic | missense SNV | Fat2:NM_001029988:exon12:c.T9217A:p.S3073T | . | 11qB1.3 | rs26974209 | 1 | 0 |
| chr11 | 55273284 | A | C | 207 | Fat2 | intronic | . | . | . | 11qB1.3 | rs256956548 | 1 | 0 |
| chr11 | 55276247 | C | T | 228 | Fat2 | intronic | . | . | . | 11qB1.3 | rs262591249 | 1 | 0 |
| chr11 | 55278850 | C | T | 228 | Fat2 | intronic | . | . | . | 11qB1.3 | rs26974201 | 1 | 0 |
| chr11 | 55278866 | A | G | 228 | Fat2 | intronic | . | . | . | 11qB1.3 | rs224007867 | 1 | 0 |
| chr11 | 55280976 | G | T | 228 | Fat2 | intronic | . | . | . | 11qB1.3 | rs26974189 | 1 | 0 |
| chr11 | 55281186 | G | A | 228 | Fat2 | exonic | synonymous SNV | Fat2:NM_001029988:exon9:c.C8700T:p.D2900D | . | 11qB1.3 | rs26974188 | 1 | 0 |
| chr11 | 55281360 | A | G | 228 | Fat2 | exonic | synonymous SNV | Fat2:NM_001029988:exon9:c.T8526C:p.P2842P | . | 11qB1.3 | rs26974187 | 1 | 0 |
| chr11 | 55281720 | C | T | 228 | Fat2 | exonic | synonymous SNV | Fat2:NM_001029988:exon9:c.G8166A:p.P2722P | . | 11qB1.3 | rs26974184 | 1 | 0 |
| chr11 | 55281750 | T | C | 228 | Fat2 | exonic | synonymous SNV | Fat2:NM_001029988:exon9:c.A8136G:p.G2712G | . | 11qB1.3 | rs26974183 | 1 | 0 |
| chr11 | 55281927 | T | C | 228 | Fat2 | exonic | synonymous SNV | Fat2:NM_001029988:exon9:c.A7959G:p.L2653L | . | 11qB1.3 | rs26974182 | 1 | 0 |
| chr11 | 55281942 | C | T | 228 | Fat2 | exonic | synonymous SNV | Fat2:NM_001029988:exon9:c.G7944A:p.E2648E | . | 11qB1.3 | rs26974181 | 1 | 0 |
| chr11 | 55281963 | G | A | 228 | Fat2 | exonic | synonymous SNV | Fat2:NM_001029988:exon9:c.C7923T:p.T2641T | . | 11qB1.3 | rs238677627 | 1 | 0 |
| chr11 | 55282217 | T | C | 228 | Fat2 | exonic | missense SNV | Fat2:NM_001029988:exon9:c.A7669G:p.I2557V | . | 11qB1.3 | rs26974177 | 1 | 0 |
| chr11 | 55282758 | A | G | 228 | Fat2 | exonic | synonymous SNV | Fat2:NM_001029988:exon9:c.T7128C:p.P2376P | . | 11qB1.3 | rs241966335 | 1 | 0 |
| chr11 | 55282983 | T | C | 228 | Fat2 | exonic | synonymous SNV | Fat2:NM_001029988:exon9:c.A6903G:p.S2301S | . | 11qB1.3 | rs6234280 | 1 | 0 |
| chr11 | 55283232 | T | G | 228 | Fat2 | exonic | synonymous SNV | Fat2:NM_001029988:exon9:c.A6654C:p.T2218T | . | 11qB1.3 | rs6235430 | 1 | 0 |
| chr11 | 55283325 | G | A | 228 | Fat2 | exonic | synonymous SNV | Fat2:NM_001029988:exon9:c.C6561T:p.Y2187Y | . | 11qB1.3 | rs6235943 | 1 | 0 |
| chr11 | 55283450 | G | T | 228 | Fat2 | exonic | synonymous SNV | Fat2:NM_001029988:exon9:c.C6436A:p.R2146R | . | 11qB1.3 | rs6249469 | 1 | 0 |
| chr11 | 55283946 | G | A | 228 | Fat2 | exonic | synonymous SNV | Fat2:NM_001029988:exon9:c.C5940T:p.N1980N | . | 11qB1.3 | rs26974167 | 1 | 0 |
| chr11 | 55284006 | T | C | 228 | Fat2 | exonic | synonymous SNV | Fat2:NM_001029988:exon9:c.A5880G:p.Q1960Q | . | 11qB1.3 | rs26974166 | 1 | 0 |
| chr11 | 55284389 | T | A | 228 | Fat2 | exonic | missense SNV | Fat2:NM_001029988:exon9:c.A5497T:p.T1833S | . | 11qB1.3 | rs26974164 | 1 | 0 |
| chr11 | 55284648 | A | G | 228 | Fat2 | exonic | synonymous SNV | Fat2:NM_001029988:exon9:c.T5238C:p.V1746V | . | 11qB1.3 | rs26974163 | 1 | 0 |
| chr11 | 55284852 | T | C | 228 | Fat2 | exonic | synonymous SNV | Fat2:NM_001029988:exon9:c.A5034G:p.P1678P | . | 11qB1.3 | rs26974161 | 1 | 0 |
| chr11 | 55284969 | C | T | 228 | Fat2 | exonic | synonymous SNV | Fat2:NM_001029988:exon9:c.G4917A:p.Q1639Q | . | 11qB1.3 | rs26974160 | 1 | 0 |
| chr11 | 55284990 | C | T | 228 | Fat2 | exonic | synonymous SNV | Fat2:NM_001029988:exon9:c.G4896A:p.A1632A | . | 11qB1.3 | rs26974159 | 1 | 0 |
| chr11 | 55285116 | A | G | 228 | Fat2 | intronic | . | . | . | 11qB1.3 | rs247075449 | 1 | 0 |
| chr11 | 55285118 | A | G | 228 | Fat2 | intronic | . | . | . | 11qB1.3 | rs217605818 | 1 | 0 |
| chr11 | 55285747 | A | G | 153 | Fat2 | intronic | . | . | . | 11qB1.3 | rs26974154 | 1 | 0 |
| chr11 | 55285775 | A | T | 170 | Fat2 | intronic | . | . | . | 11qB1.3 | rs26974153 | 1 | 0 |
| chr11 | 55285906 | C | T | 190 | Fat2 | exonic | missense SNV | Fat2:NM_001029988:exon8:c.G4762A:p.A1588T | . | 11qB1.3 | . | 0 | 1 |
| chr11 | 55287689 | T | G | 228 | Fat2 | exonic | missense SNV | Fat2:NM_001029988:exon7:c.A4544C:p.H1515P | . | 11qB1.3 | rs221870337 | 1 | 0 |
| chr11 | 55287697 | C | T | 228 | Fat2 | exonic | synonymous SNV | Fat2:NM_001029988:exon7:c.G4536A:p.L1512L | . | 11qB1.3 | rs26974144 | 1 | 0 |
| chr11 | 55287941 | A | G | 228 | Fat2 | intronic | . | . | . | 11qB1.3 | rs26974143 | 1 | 0 |
| chr11 | 55288077 | A | C | 228 | Fat2 | intronic | . | . | . | 11qB1.3 | rs26974142 | 1 | 0 |
| chr11 | 55289022 | C | T | 165 | Fat2 | intronic | . | . | . | 11qB1.3 | rs26974139 | 1 | 0 |
| chr11 | 55289098 | G | A | 228 | Fat2 | intronic | . | . | . | 11qB1.3 | rs26974138 | 1 | 0 |
| chr11 | 55289175 | C | T | 228 | Fat2 | intronic | . | . | . | 11qB1.3 | rs257582102 | 1 | 0 |
| chr11 | 55289416 | A | G | 228 | Fat2 | intronic | . | . | . | 11qB1.3 | rs240914829 | 1 | 0 |
| chr11 | 55289463 | A | T | 228 | Fat2 | intronic | . | . | . | 11qB1.3 | rs260276035 | 1 | 0 |
| chr11 | 55292160 | G | A | 228 | Fat2 | intronic | . | . | . | 11qB1.3 | rs235039038 | 1 | 0 |
| chr11 | 55296061 | A | G | 228 | Fat2 | intronic | . | . | . | 11qB1.3 | rs216016629 | 1 | 0 |
| chr11 | 55296239 | T | C | 228 | Fat2 | exonic | synonymous SNV | Fat2:NM_001029988:exon4:c.A3780G:p.P1260P | . | 11qB1.3 | rs212024822 | 1 | 0 |
| chr11 | 55296248 | T | G | 228 | Fat2 | exonic | synonymous SNV | Fat2:NM_001029988:exon4:c.A3771C:p.P1257P | . | 11qB1.3 | rs237190193 | 1 | 0 |
| chr11 | 55296368 | A | G | 228 | Fat2 | exonic | synonymous SNV | Fat2:NM_001029988:exon4:c.T3651C:p.N1217N | . | 11qB1.3 | rs219952020 | 1 | 0 |
| chr11 | 55296428 | A | G | 228 | Fat2 | intronic | . | . | . | 11qB1.3 | rs221794177 | 1 | 0 |
| chr11 | 55296587 | C | T | 124 | Fat2 | intronic | . | . | . | 11qB1.3 | rs26974137 | 1 | 0 |
| chr11 | 55298371 | G | A | 160 | Fat2 | intronic | . | . | . | 11qB1.3 | rs231296848 | 1 | 0 |
| chr11 | 55298473 | C | A | 204 | Fat2 | intronic | . | . | . | 11qB1.3 | rs6296416 | 1 | 0 |
| chr11 | 55298538 | G | C | 228 | Fat2 | intronic | . | . | . | 11qB1.3 | rs6296933 | 1 | 0 |
| chr11 | 55298563 | A | G | 228 | Fat2 | exonic | synonymous SNV | Fat2:NM_001029988:exon3:c.T3628C:p.L1210L | . | 11qB1.3 | rs6296995 | 1 | 0 |
| chr11 | 55298693 | C | T | 228 | Fat2 | intronic | . | . | . | 11qB1.3 | rs6297687 | 1 | 0 |
| chr11 | 55298813 | T | C | 97 | Fat2 | intronic | . | . | . | 11qB1.3 | rs6298317 | 1 | 0 |
| chr11 | 55303738 | T | C | 228 | Fat2 | exonic | synonymous SNV | Fat2:NM_001029988:exon2:c.A3474G:p.Q1158Q | . | 11qB1.3 | rs26974123 | 1 | 0 |
| chr11 | 55303831 | A | G | 228 | Fat2 | exonic | synonymous SNV | Fat2:NM_001029988:exon2:c.T3381C:p.V1127V | . | 11qB1.3 | rs224405437 | 1 | 0 |
| chr11 | 55303995 | G | T | 228 | Fat2 | intronic | . | . | . | 11qB1.3 | rs26974122 | 1 | 0 |
| chr11 | 55308885 | T | C | 169 | Fat2 | intronic | . | . | . | 11qB1.3 | rs245469307 | 1 | 0 |
| chr11 | 55309034 | T | A | 228 | Fat2 | exonic | synonymous SNV | Fat2:NM_001029988:exon1:c.A3213T:p.L1071L | . | 11qB1.3 | rs26974118 | 1 | 0 |
| chr11 | 55309230 | C | T | 228 | Fat2 | exonic | missense SNV | Fat2:NM_001029988:exon1:c.G3017A:p.R1006K | . | 11qB1.3 | rs26974117 | 1 | 0 |
| chr11 | 55309394 | A | G | 228 | Fat2 | exonic | synonymous SNV | Fat2:NM_001029988:exon1:c.T2853C:p.D951D | . | 11qB1.3 | rs26974116 | 1 | 0 |
| chr11 | 55309708 | T | C | 228 | Fat2 | exonic | missense SNV | Fat2:NM_001029988:exon1:c.A2539G:p.T847A | . | 11qB1.3 | rs26974114 | 1 | 0 |
| chr11 | 55309799 | T | G | 228 | Fat2 | exonic | missense SNV | Fat2:NM_001029988:exon1:c.A2448C:p.K816N | . | 11qB1.3 | rs29422802 | 1 | 0 |
| chr11 | 55309901 | G | A | 228 | Fat2 | exonic | synonymous SNV | Fat2:NM_001029988:exon1:c.C2346T:p.T782T | . | 11qB1.3 | rs26974113 | 1 | 0 |
| chr11 | 55309997 | T | C | 228 | Fat2 | exonic | synonymous SNV | Fat2:NM_001029988:exon1:c.A2250G:p.L750L | . | 11qB1.3 | rs26974111 | 1 | 0 |
| chr11 | 55310303 | G | A | 228 | Fat2 | exonic | synonymous SNV | Fat2:NM_001029988:exon1:c.C1944T:p.Y648Y | . | 11qB1.3 | rs26974109 | 1 | 0 |
| chr11 | 55310431 | C | T | 228 | Fat2 | exonic | missense SNV | Fat2:NM_001029988:exon1:c.G1816A:p.E606K | . | 11qB1.3 | rs26974106 | 1 | 0 |
| chr11 | 55310501 | C | G | 228 | Fat2 | exonic | synonymous SNV | Fat2:NM_001029988:exon1:c.G1746C:p.S582S | . | 11qB1.3 | rs26974105 | 1 | 0 |
| chr11 | 55310681 | G | A | 228 | Fat2 | exonic | synonymous SNV | Fat2:NM_001029988:exon1:c.C1566T:p.Y522Y | . | 11qB1.3 | rs26974104 | 1 | 0 |
| chr11 | 55311211 | T | C | 228 | Fat2 | exonic | missense SNV | Fat2:NM_001029988:exon1:c.A1036G:p.I346V | . | 11qB1.3 | rs26974101 | 1 | 0 |
| chr11 | 55311212 | G | C | 228 | Fat2 | exonic | synonymous SNV | Fat2:NM_001029988:exon1:c.C1035G:p.S345S | . | 11qB1.3 | rs26974100 | 1 | 0 |
| chr11 | 55311434 | A | G | 228 | Fat2 | exonic | synonymous SNV | Fat2:NM_001029988:exon1:c.T813C:p.I271I | . | 11qB1.3 | rs26974099 | 1 | 0 |
| chr11 | 55312324 | T | C | 228 | . | upstream | . | . | . | 11qB1.3 | rs26974098 | 1 | 0 |
| chr11 | 55395376 | G | A | 151 | Sparc | UTR3 | . | . | . | 11qB1.3 | rs26959846 | 1 | 0 |
| chr11 | 55395390 | T | A | 166 | Sparc | UTR3 | . | . | . | 11qB1.3 | rs249013561 | 1 | 0 |
| chr11 | 55395916 | G | A | 228 | Sparc | exonic | synonymous SNV | Sparc:NM_001290817:exon9:c.C762T:p.R254R,Sparc:NM_009242:exon9:c.C765T:p.R255R | . | 11qB1.3 | rs259731371 | 1 | 0 |
| chr11 | 55398676 | A | G | 228 | Sparc | exonic | synonymous SNV | Sparc:NM_001290817:exon8:c.T600C:p.N200N,Sparc:NM_009242:exon8:c.T603C:p.N201N | . | 11qB1.3 | rs26959835 | 1 | 0 |
| chr11 | 55398799 | G | A | 130 | Sparc | intronic | . | . | . | 11qB1.3 | rs45800836 | 1 | 0 |
| chr11 | 55399026 | G | A | 154 | Sparc | intronic | . | . | . | 11qB1.3 | rs26959832 | 1 | 0 |
| chr11 | 55399294 | A | G | 228 | Sparc | exonic | synonymous SNV | Sparc:NM_001290817:exon7:c.T477C:p.T159T,Sparc:NM_009242:exon7:c.T480C:p.T160T | . | 11qB1.3 | rs26959829 | 1 | 0 |
| chr11 | 55402196 | T | C | 203 | Sparc | intronic | . | . | . | 11qB1.3 | rs235895960 | 1 | 0 |
| chr11 | 55402197 | G | T | 202 | Sparc | intronic | . | . | . | 11qB1.3 | rs261935939 | 1 | 0 |
| chr11 | 55405326 | T | C | 228 | Sparc | intronic | . | . | . | 11qB1.3 | rs47754912 | 1 | 0 |
| chr11 | 55409905 | T | A | 228 | Sparc | intronic | . | . | . | 11qB1.3 | rs26959801 | 1 | 0 |
| chr11 | 55450330 | G | A | 118 | Atox1 | intronic | . | . | . | 11qB1.3 | rs26948554 | 1 | 0 |
| chr11 | 55450475 | T | A | 228 | Atox1 | UTR3 | . | . | . | 11qB1.3 | rs26948552 | 1 | 0 |
| chr11 | 55454838 | A | G | 228 | Atox1 | intronic | . | . | . | 11qB1.3 | rs243421550 | 1 | 0 |
| chr11 | 55454859 | A | G | 228 | Atox1 | intronic | . | . | . | 11qB1.3 | rs227256389 | 1 | 0 |

| | | | | | | | | | | | | | |
|---|---|---|---|---|---|---|---|---|---|---|---|---|---|
| chr11 | 55454996 | C | T | 228 | Atox1 | intronic | . | . | . | 11qB1.3 | rs26948536 | 1 | 0 |
| chr11 | 55455050 | A | G | 228 | Atox1 | intronic | . | . | . | 11qB1.3 | rs26948535 | 1 | 0 |
| chr11 | 55455152 | T | C | 228 | Atox1 | intronic | . | . | . | 11qB1.3 | rs26948534 | 1 | 0 |
| chr11 | 55460841 | C | T | 147 | Atox1 | intronic | . | . | . | 11qB1.3 | rs218616292 | 1 | 0 |
| chr11 | 55461113 | G | A | 228 | Atox1 | UTR5 | . | . | 355 | 11qB1.3 | rs249517579 | 1 | 0 |
| chr11 | 55461225 | C | T | 228 | . | upstream | . | . | 355 | 11qB1.3 | rs260850436 | 1 | 0 |
| chr11 | 55463798 | G | A | 136 | . | intergenic | . | . | . | 11qB1.3 | rs246370860 | 1 | 0 |
| chr11 | 55463853 | A | G | 187 | . | intergenic | . | . | . | 11qB1.3 | rs214344753 | 1 | 0 |
| chr11 | 55463957 | A | G | 166 | . | intergenic | . | . | . | 11qB1.3 | rs212318869 | 1 | 0 |
| chr11 | 55464015 | T | C | 136 | . | intergenic | . | . | . | 11qB1.3 | rs45740117 | 1 | 0 |
| chr11 | 55485380 | C | T | 115 | G3bp1 | intronic | . | . | . | 11qB1.3 | rs26948472 | 1 | 0 |
| chr11 | 55485546 | G | A | 228 | G3bp1 | intronic | . | . | . | 11qB1.3 | rs219362226 | 1 | 0 |
| chr11 | 55485676 | C | T | 228 | G3bp1 | exonic | synonymous SNV | G3bp1:NM_013716:exon2:c.C33T:p.V11V | . | 11qB1.3 | rs236712142 | 1 | 0 |
| chr11 | 55485706 | T | C | 228 | G3bp1 | exonic | synonymous SNV | G3bp1:NM_013716:exon2:c.T63C:p.T21T | . | 11qB1.3 | rs26948471 | 1 | 0 |
| chr11 | 55485817 | G | A | 228 | G3bp1 | intronic | . | . | . | 11qB1.3 | rs26948470 | 1 | 0 |
| chr11 | 55487397 | C | T | 228 | G3bp1 | intronic | . | . | . | 11qB1.3 | rs26948466 | 1 | 0 |
| chr11 | 55487688 | G | C | 173 | G3bp1 | intronic | . | . | . | 11qB1.3 | rs26948465 | 1 | 0 |
| chr11 | 55492068 | T | A | 228 | G3bp1 | intronic | . | . | . | 11qB1.3 | rs214598354 | 1 | 0 |
| chr11 | 55492072 | T | G | 228 | G3bp1 | intronic | . | . | . | 11qB1.3 | rs231616665 | 1 | 0 |
| chr11 | 55493348 | T | C | 200 | G3bp1 | intronic | . | . | . | 11qB1.3 | rs212279076 | 1 | 0 |
| chr11 | 55493394 | T | C | 228 | G3bp1 | intronic | . | . | . | 11qB1.3 | rs243406032 | 1 | 0 |
| chr11 | 55493549 | A | C | 228 | G3bp1 | intronic | . | . | . | 11qB1.3 | rs26948461 | 1 | 0 |
| chr11 | 55493574 | G | A | 228 | G3bp1 | intronic | . | . | . | 11qB1.3 | rs217602576 | 1 | 0 |
| chr11 | 55495148 | A | G | 126 | G3bp1 | intronic | . | . | . | 11qB1.3 | rs235147382 | 1 | 0 |
| chr11 | 55495234 | A | T | 169 | G3bp1 | intronic | . | . | . | 11qB1.3 | rs265780708 | 1 | 0 |
| chr11 | 55495298 | A | G | 208 | G3bp1 | exonic | synonymous SNV | G3bp1:NM_013716:exon7:c.A582G:p.E194E | . | 11qB1.3 | rs227674606 | 1 | 0 |
| chr11 | 55495424 | T | C | 228 | G3bp1 | exonic | synonymous SNV | G3bp1:NM_013716:exon7:c.T708C:p.D236D | . | 11qB1.3 | rs214013212 | 1 | 0 |
| chr11 | 55495495 | T | C | 228 | G3bp1 | intronic | . | . | . | 11qB1.3 | rs225379836 | 1 | 0 |
| chr11 | 55495927 | G | T | 122 | G3bp1 | intronic | . | . | . | 11qB1.3 | rs242206836 | 1 | 0 |
| chr11 | 55496021 | G | T | 194 | G3bp1 | intronic | . | . | . | 11qB1.3 | rs227607825 | 1 | 0 |
| chr11 | 55496090 | C | T | 228 | G3bp1 | intronic | . | . | . | 11qB1.3 | rs239996437 | 1 | 0 |
| chr11 | 55496267 | T | C | 228 | G3bp1 | intronic | . | . | . | 11qB1.3 | rs225320192 | 1 | 0 |
| chr11 | 55496296 | C | T | 228 | G3bp1 | intronic | . | . | . | 11qB1.3 | rs264475012 | 1 | 0 |
| chr11 | 55496365 | G | T | 164 | G3bp1 | intronic | . | . | . | 11qB1.3 | rs258924904 | 1 | 0 |
| chr11 | 55498470 | A | C | 228 | G3bp1 | intronic | . | . | . | 11qB1.3 | rs251350171 | 1 | 0 |
| chr11 | 55499270 | A | G | 131 | G3bp1 | intronic | . | . | . | 11qB1.3 | rs228496119 | 1 | 0 |
| chr11 | 55499420 | C | T | 228 | G3bp1 | intronic | . | . | . | 11qB1.3 | rs247192867 | 1 | 0 |
| chr11 | 55499732 | T | C | 179 | G3bp1 | UTR3 | . | . | . | 11qB1.3 | rs26948456 | 1 | 0 |
| chr11 | 55514828 | A | G | 228 | Glra1 | exonic | synonymous SNV | Glra1:NM_001290821:exon9:c.T1359C:p.D453D,Glra1:NM_020492:exon9:c.T1335C:p.D445D | . | 11qB1.3 | rs237212073 | 1 | 0 |
| chr11 | 55514849 | A | G | 228 | Glra1 | exonic | synonymous SNV | Glra1:NM_001290821:exon9:c.T1338C:p.Y446Y,Glra1:NM_020492:exon9:c.T1314C:p.Y438Y | . | 11qB1.3 | rs26948356 | 1 | 0 |
| chr11 | 55520871 | T | C | 228 | Glra1 | intronic | . | . | . | 11qB1.3 | rs230357873 | 1 | 0 |
| chr11 | 55520887 | T | C | 228 | Glra1 | intronic | . | . | . | 11qB1.3 | rs260610734 | 1 | 0 |
| chr11 | 55521009 | A | G | 228 | Glra1 | exonic | synonymous SNV | Glra1:NM_001290821:exon8:c.T972C:p.S324S,Glra1:NM_020492:exon8:c.T972C:p.S324S | . | 11qB1.3 | rs26948344 | 1 | 0 |
| chr11 | 55521018 | G | A | 228 | Glra1 | exonic | synonymous SNV | Glra1:NM_001290821:exon8:c.C963T:p.F321F,Glra1:NM_020492:exon8:c.C963T:p.F321F | . | 11qB1.3 | rs220868654 | 1 | 0 |
| chr11 | 55527226 | A | G | 169 | Glra1 | intronic | . | . | . | 11qB1.3 | rs253462468 | 1 | 0 |
| chr11 | 55527272 | G | T | 228 | Glra1 | exonic | synonymous SNV | Glra1:NM_001290821:exon7:c.C895A:p.R299R,Glra1:NM_020492:exon7:c.C895A:p.R299R | . | 11qB1.3 | rs257067167 | 1 | 0 |
| chr11 | 55532357 | G | A | 228 | Glra1 | exonic | synonymous SNV | Glra1:NM_001290821:exon6:c.C624T:p.D208D,Glra1:NM_020492:exon6:c.C624T:p.D208D | . | 11qB1.3 | rs26948337 | 1 | 0 |
| chr11 | 55532426 | G | T | 228 | Glra1 | intronic | . | . | . | 11qB1.3 | rs235424098 | 1 | 0 |
| chr11 | 55532615 | T | C | 135 | Glra1 | intronic | . | . | . | 11qB1.3 | rs235012915 | 1 | 0 |
| chr11 | 55536324 | G | A | 228 | Glra1 | exonic | synonymous SNV | Glra1:NM_001290821:exon4:c.C433T:p.L145L,Glra1:NM_020492:exon4:c.C433T:p.L145L | . | 11qB1.3 | rs26948324 | 1 | 0 |
| chr11 | 55536405 | A | G | 228 | Glra1 | exonic | synonymous SNV | Glra1:NM_001290821:exon4:c.T352C:p.L118L,Glra1:NM_020492:exon4:c.T352C:p.L118L | . | 11qB1.3 | rs26948323 | 1 | 0 |
| chr11 | 55566025 | T | A | 178 | Glra1 | intronic | . | . | . | 11qB1.3 | rs26948273 | 1 | 0 |
| chr11 | 55574235 | G | A | 228 | Glra1 | intronic | . | . | . | 11qB1.3 | rs45860477 | 1 | 0 |
| chr11 | 55574458 | A | C | 228 | Glra1 | intronic | . | . | . | 11qB1.3 | rs50935913 | 1 | 0 |
| chr11 | 55607607 | G | T | 228 | Glra1 | intronic | . | . | . | 11qB1.3 | rs26991494 | 1 | 0 |
| chr11 | 55607639 | G | C | 222 | Glra1 | intronic | . | . | . | 11qB1.3 | . | 0 | 1 |
| chr11 | 55607746 | A | T | 228 | Glra1 | UTR5 | . | . | . | 11qB1.3 | rs216993914 | 1 | 0 |
| chr11 | 55607749 | G | T | 228 | Glra1 | UTR5 | . | . | . | 11qB1.3 | rs251650768 | 1 | 0 |
| chr11 | 56026746 | T | C | 151 | Nmur2 | UTR3 | . | . | . | 11qB1.3 | rs26977346 | 1 | 0 |
| chr11 | 56027223 | A | T | 228 | Nmur2 | exonic | synonymous SNV | Nmur2:NM_153079:exon4:c.T939A:p.A313A | . | 11qB1.3 | rs26977340 | 1 | 0 |
| chr11 | 56029415 | G | A | 125 | Nmur2 | intronic | . | . | . | 11qB1.3 | . | 1 | 0 |
| chr11 | 56029580 | T | G | 228 | Nmur2 | exonic | synonymous SNV | Nmur2:NM_153079:exon3:c.A838C:p.R280R | . | 11qB1.3 | rs26977324 | 1 | 0 |
| chr11 | 56029731 | G | T | 228 | Nmur2 | intronic | . | . | . | 11qB1.3 | rs26977323 | 1 | 0 |
| chr11 | 56032588 | T | G | 108 | Nmur2 | intronic | . | . | . | 11qB1.3 | rs247737594 | 1 | 0 |
| chr11 | 56039969 | T | C | 101 | Nmur2 | intronic | . | . | . | 11qB1.3 | rs26977273 | 1 | 0 |
| chr11 | 56040170 | C | T | 228 | Nmur2 | intronic | . | . | . | 11qB1.3 | rs29436742 | 1 | 0 |
| chr11 | 56040278 | T | C | 228 | Nmur2 | exonic | missense SNV | Nmur2:NM_153079:exon1:c.A606G:p.I202M | . | 11qB1.3 | rs29422825 | 1 | 0 |
| chr11 | 56040316 | C | T | 228 | Nmur2 | exonic | missense SNV | Nmur2:NM_153079:exon1:c.G568A:p.V190M | . | 11qB1.3 | rs247623181 | 1 | 0 |
| chr11 | 56040317 | A | G | 228 | Nmur2 | exonic | synonymous SNV | Nmur2:NM_153079:exon1:c.T567C:p.S189S | . | 11qB1.3 | rs26977272 | 1 | 0 |
| chr11 | 56040533 | A | G | 228 | Nmur2 | exonic | synonymous SNV | Nmur2:NM_153079:exon1:c.T351C:p.L117L | . | 11qB1.3 | rs29403798 | 1 | 0 |
| chr11 | 56040686 | T | C | 228 | Nmur2 | exonic | synonymous SNV | Nmur2:NM_153079:exon1:c.A198G:p.Q66Q | . | 11qB1.3 | rs26977271 | 1 | 0 |
| chr11 | 56040752 | A | G | 228 | Nmur2 | exonic | synonymous SNV | Nmur2:NM_153079:exon1:c.T132C:p.Y44Y | . | 11qB1.3 | rs26977270 | 1 | 0 |
| chr11 | 56040927 | A | G | 228 | Nmur2 | UTR5 | . | . | . | 11qB1.3 | rs50896281 | 1 | 0 |
| chr11 | 57513506 | C | T | 204 | Fam114a2 | intronic | . | . | . | 11qB1.3 | . | 0 | 1 |
| chr11 | 58096967 | C | G | 77 | . | intergenic | . | . | . | 11qB1.3 | . | 0 | 1 |
| chr11 | 58097110 | C | T | 193 | . | intergenic | . | . | . | 11qB1.3 | . | 0 | 1 |
| chr11 | 58097276 | G | T | 222 | . | intergenic | . | . | . | 11qB1.3 | . | 0 | 1 |
| chr11 | 58171695 | G | C | 221 | Mrpl22 | exonic | missense SNV | Mrpl22:NM_175001:exon1:c.G10C:p.A4P | . | 11qB1.3 | . | 0 | 1 |
| chr11 | 58222262 | A | C | 222 | Irgm2 | UTR3 | . | . | . | 11qB1.3 | . | 0 | 1 |
| chr11 | 58439982 | G | A | 221 | Gm12253 | exonic | missense SNV | Gm12253:NM_001045542:exon6:c.G566A:p.C189Y | . | 11qB1.3 | . | 0 | 1 |
| chr11 | 58580648 | T | G | 88 | . | upstream | . | . | . | 11qB1.3 | . | 0 | 1 |
| chr11 | 58817086 | T | G | 219 | Olfr313 | exonic | missense SNV | Olfr313:NM_146536:exon1:c.T77G:p.I26R | . | 11qB1.3 | . | 0 | 1 |
| chr11 | 59038918 | C | G | 222 | Obscn | exonic | missense SNV | Obscn:NM_001171512:exon57:c.G15470C:p.R5157P,Obscn:NM_199152:exon64:c.G17429C:p.R5810P | . | 11qB1.3 | . | 0 | 1 |
| chr11 | 59054710 | T | C | 221 | Obscn | intronic | . | . | . | 11qB1.3 | . | 0 | 1 |
| chr11 | 59177309 | G | A | 53 | Gjc2 | exonic | missense SNV | Gjc2:NM_080454:exon2:c.C346T:p.R116C,Gjc2:NM_175452:exon2:c.C346T:p.R116C | 930 | 11qB1.3 | . | 0 | 1 |
| chr11 | 59475091 | G | T | 222 | . | intergenic | . | . | . | 11qB1.3 | . | 0 | 1 |
| chr11 | 59829944 | T | G | 126 | Cops3 | intronic | . | . | . | 11qB1.3 | . | 0 | 1 |
| chr11 | 60493039 | G | A | 221 | Myo15 | exonic | synonymous SNV | Myo15:NM_182698:exon15:c.G1350A:p.K450K,Myo15:NM_001103171:exon16:c.G4911A:p.K1637K,Myo15:NM | . | 11qB2 | . | 0 | 1 |
| chr11 | 60508863 | T | C | 213 | Myo15 | intronic | . | . | . | 11qB2 | . | 0 | 1 |
| chr11 | 60510138 | G | C | 221 | Myo15 | intronic | . | . | . | 11qB2 | . | 0 | 1 |
| chr11 | 61162778 | G | T | 32.683 | Usp22 | intronic | . | . | . | 11qB2 | . | 0 | 1 |
| chr11 | 61213443 | C | G | 117 | Aldh3a1 | intronic | . | . | . | 11qB2 | . | 0 | 1 |
| chr11 | 61506416 | A | C | 221 | B9d1 | splicing | . | . | . | 11qB2 | . | 0 | 1 |
| chr11 | 61673282 | C | T | 222 | Slc5a10 | splicing | . | . | . | 11qB2 | . | 0 | 1 |
| chr11 | 61673284 | G | A | 222 | Slc5a10 | splicing | . | . | . | 11qB2 | . | 0 | 1 |
| chr11 | 61916141 | T | C | 222 | Akap10 | exonic | missense SNV | Akap10:NM_019921:exon3:c.A262G:p.T88A | . | 11qB2 | . | 0 | 1 |
| chr11 | 62249360 | G | T | 221 | Adora2b | exonic | missense SNV | Adora2b:NM_007413:exon1:c.G259T:p.V87L | 947 | 11qB2 | . | 0 | 1 |
| chr11 | 62268684 | T | C | 221 | Zswim7 | intronic | . | . | . | 11qB2 | . | 0 | 1 |
| chr11 | 62750214 | G | A | 221 | . | intergenic | . | . | . | 11qB2 | . | 0 | 1 |
| chr11 | 62836678 | G | C | 143 | Trim16 | splicing | . | . | . | 11qB2 | . | 0 | 1 |
| chr11 | 62873295 | G | T | 51 | Fbxw10 | intronic | . | . | . | 11qB2 | . | 0 | 1 |
| chr11 | 63073481 | T | C | 228 | Mir6354,Tekt3 | exonic | synonymous SNV | Tekt3:NM_027660:exon3:c.T636C:p.H212H | . | 11qB3 | rs6268547 | 1 | 0 |
| chr11 | 63073526 | G | T | 222 | Mir6354,Tekt3 | ncRNA_intronic | . | . | . | 11qB3 | rs51804354 | 0 | 1 |
| chr11 | 63241358 | G | T | 63 | Mir6354 | ncRNA_intronic | . | . | . | 11qB3 | . | 0 | 1 |
| chr11 | 64759592 | A | C | 27.0123 | . | intergenic | . | . | . | 11qB3 | . | 0 | 1 |
| chr11 | 64982597 | T | G | 130 | Elac2 | intronic | . | . | . | 11qB3 | . | 0 | 1 |
| chr11 | 65194954 | C | A | 170 | Myocd | intronic | . | . | . | 11qB3 | . | 0 | 1 |
| chr11 | 65196423 | G | C | 222 | Myocd | intronic | . | . | . | 11qB3 | . | 0 | 1 |
| chr11 | 65205201 | C | T | 48.6696 | Myocd | intronic | . | . | . | 11qB3 | . | 0 | 1 |
| chr11 | 65848150 | A | C | 53 | Dnah9 | intronic | . | . | . | 11qB3 | . | 0 | 1 |
| chr11 | 65955044 | C | A | 221 | Dnah9 | exonic | missense SNV | Dnah9:NM_001099633:exon48:c.G9013T:p.V3005L | . | 11qB3 | . | 0 | 1 |
| chr11 | 66061280 | T | C | 105 | Dnah9 | intronic | . | . | . | 11qB3 | . | 0 | 1 |
| chr11 | 66072643 | A | C | 228 | Dnah9 | intronic | . | . | . | 11qB3 | rs29383516 | 1 | 0 |
| chr11 | 66075144 | A | G | 222 | Dnah9 | exonic | missense SNV | Dnah9:NM_001099633:exon24:c.T5045C:p.L1682P | . | 11qB3 | . | 0 | 1 |
| chr11 | 66159921 | T | G | 222 | Dnah9 | exonic | missense SNV | Dnah9:NM_001099633:exon2:c.A544C:p.T182P | . | 11qB3 | . | 0 | 1 |
| chr11 | 66926006 | G | A | 222 | Gm12298,Pirt | exonic | missense SNV | Pirt:NM_178656:exon2:c.G142A:p.E48K | . | 11qB3 | . | 0 | 1 |
| chr11 | 67038271 | C | A | 221 | Adprm | exonic | missense SNV | Adprm:NM_025510:exon5:c.G893T:p.G298V | . | 11qB3 | . | 0 | 1 |
| chr11 | 67041761 | C | G | 222 | Adprm | exonic | synonymous SNV | Adprm:NM_025510:exon3:c.G321C:p.T107T | . | 11qB3 | . | 0 | 1 |
| chr11 | 67286329 | C | A | 222 | Myh8 | intronic | . | . | . | 11qB3 | . | 0 | 1 |
| chr11 | 67289664 | G | T | 222 | Myh8 | exonic | missense SNV | Myh8:NM_177369:exon16:c.G1725T:p.K575N | . | 11qB3 | . | 0 | 1 |
| chr11 | 67301440 | C | T | 180 | Myh8 | exonic | synonymous SNV | Myh8:NM_177369:exon31:c.C4218T:p.H1406H | . | 11qB3 | . | 0 | 1 |
| chr11 | 67367223 | A | C | 222 | Myh13 | exonic | missense SNV | Myh13:NM_001081250:exon34:c.A5240C:p.N1747T | . | 11qB3 | . | 0 | 1 |
| chr11 | 67695628 | G | A | 177 | Rcvrn | exonic | missense SNV | Rcvrn:NM_009038:exon1:c.G212A:p.R71H | 215 | 11qB3 | . | 0 | 1 |
| chr11 | 67762561 | A | C | 122 | Glp2r | intronic | . | . | . | 11qB3 | . | 0 | 1 |
| chr11 | 67922526 | G | A | 222 | . | upstream | . | . | 1060 | 11qB3 | . | 0 | 1 |
| chr11 | 67922910 | A | G | 221 | . | upstream | . | . | . | 11qB3 | . | 0 | 1 |
| chr11 | 68752000 | C | T | 221 | Myh10 | exonic | missense SNV | Myh10:NM_175260:exon6:c.C692T:p.A231V | . | 11qB3 | . | 0 | 1 |
| chr11 | 68792045 | G | T | 222 | Myh10 | intronic | . | . | . | 11qB3 | . | 0 | 1 |
| chr11 | 68993496 | G | T | 221 | Pfas | intronic | . | . | . | 11qB3 | . | 0 | 1 |
| chr11 | 69100688 | C | A | 194 | Per1 | exonic | missense SNV | Per1:NM_001159367:exon2:c.C47A:p.P16H,Per1:NM_011065:exon2:c.C47A:p.P16H | . | 11qB3 | . | 0 | 1 |
| chr11 | 69164362 | A | C | 222 | Alox12b | exonic | missense SNV | Alox12b:NM_009659:exon8:c.A958C:p.I320L | . | 11qB3 | . | 0 | 1 |
| chr11 | 69169791 | G | T | 189 | Alox12b | UTR3 | . | . | . | 11qB3 | . | 0 | 1 |
| chr11 | 69328648 | G | A | 222 | Kcnab3 | intronic | . | . | . | 11qB3 | . | 0 | 1 |
| chr11 | 69342810 | C | G | 70 | . | downstream | . | . | . | 11qB3 | . | 0 | 1 |
| chr11 | 69342822 | G | A | 66 | . | downstream | . | . | . | 11qB3 | . | 0 | 1 |
| chr11 | 69496703 | A | T | 110 | Dnah2 | intronic | . | . | . | 11qB3 | . | 0 | 1 |
| chr11 | 69587267 | G | T | 222 | Trp53 | exonic | stopgain | Trp53:NM_001127233:exon3:c.G94T:p.E32X,Trp53:NM_011640:exon3:c.G94T:p.E32X | . | 11qB3 | . | 0 | 1 |
| chr11 | 69590672 | G | C | 221 | Trp53 | exonic | missense SNV | Trp53:NM_001127233:exon10:c.G1001C:p.R334P,Trp53:NM_011640:exon10:c.G1001C:p.R334P | . | 11qB3 | . | 0 | 1 |
| chr11 | 69658006 | C | T | 221 | Mpdu1 | exonic | synonymous SNV | Mpdu1:NM_011900:exon4:c.G318A:p.A106A | . | 11qB3 | . | 0 | 1 |
| chr11 | 69758039 | C | T | 60 | Polr2a | intronic | . | . | . | 11qB3 | . | 0 | 1 |
| chr11 | 69856532 | G | A | 222 | Tnk1 | exonic | missense SNV | Tnk1:NM_031880:exon5:c.C272T:p.P91L | . | 11qB3 | . | 0 | 1 |
| chr11 | 69908838 | G | C | 222 | Neurl4 | exonic | missense SNV | Neurl4:NM_001291119:exon17:c.G2966C:p.C989S,Neurl4:NM_001013414:exon18:c.G3038C:p.C1013S,Neur | . | 11qB3 | . | 0 | 1 |
| chr11 | 70030777 | G | A | 80 | Dlg4 | intronic | . | . | . | 11qB3 | . | 0 | 1 |
| chr11 | 70220949 | A | G | 221 | Slc16a13 | UTR5 | . | . | . | 11qB3 | . | 0 | 1 |
| chr11 | 71019149 | C | A | 150 | Derl2 | exonic | missense SNV | Derl2:NM_001291148:exon1:c.G88T:p.A30S,Derl2:NM_033562:exon1:c.G88T:p.A30S | 910 | 11qB4 | . | 0 | 1 |
| chr11 | 71155807 | T | C | 78 | Nlrp1b | intronic | . | . | . | 11qB4 | . | 0 | 1 |
| chr11 | 71161067 | T | C | 221 | Nlrp1b | exonic | missense SNV | Nlrp1b:NM_001162414:exon10:c.A3053G:p.K1018R,Nlrp1b:NM_001040696:exon11:c.A3044G:p.K1015R | . | 11qB4 | . | 0 | 1 |
| chr11 | 71200979 | T | G | 81 | Nlrp1b | intronic | . | . | . | 11qB4 | . | 0 | 1 |
| chr11 | 71228239 | C | T | 52 | Nlrp1b | intronic | . | . | . | 11qB4 | . | 0 | 1 |
| chr11 | 71759836 | G | T | 65 | Wscd1 | UTR5 | . | . | . | 11qB4 | . | 0 | 1 |
| chr11 | 72391622 | T | C | 53 | Smtnl2 | intronic | . | . | . | 11qB4 | . | 0 | 1 |
| chr11 | 72391624 | A | G | 53 | Smtnl2 | intronic | . | . | . | 11qB4 | . | 0 | 1 |
| chr11 | 72728607 | G | T | 128 | Ankfy1 | intronic | . | . | . | 11qB4 | . | 0 | 1 |
| chr11 | 72826003 | T | C | 163 | Zzef1 | intronic | . | . | . | 11qB4 | . | 0 | 1 |
| chr11 | 72876350 | A | C | 103 | Zzef1 | intronic | . | . | . | 11qB4 | . | 0 | 1 |
| chr11 | 73070787 | C | A | 221 | Ncbp3 | intronic | . | . | . | 11qB4 | . | 0 | 1 |
| chr11 | 73160573 | C | A | 35.8817 | P2rx5 | UTR5 | . | . | 336 | 11qB4 | . | 0 | 1 |
| chr11 | 73192955 | T | C | 145 | Ctns | intronic | . | . | . | 11qB4 | . | 0 | 1 |
| chr11 | 73196540 | A | T | 138 | Ctns | intronic | . | . | . | 11qB4 | . | 0 | 1 |
| chr11 | 73225881 | T | A | 125 | . | intergenic | . | . | . | 11qB4 | . | 0 | 1 |
| chr11 | 73344455 | T | A | 58 | Spata22 | intronic | . | . | . | 11qB4 | . | 0 | 1 |
| chr11 | 73354357 | C | A | 221 | Olfr20 | exonic | synonymous SNV | Olfr20:NM_146923:exon2:c.C603A:p.V201V | . | 11qB4 | . | 0 | 1 |
| chr11 | 74239962 | A | G | 221 | . | intergenic | . | . | . | 11qB5 | . | 0 | 1 |
| chr11 | 74298802 | G | A | 221 | . | intergenic | . | . | . | 11qB5 | . | 0 | 1 |
| chr11 | 74323316 | T | C | 53 | . | intergenic | . | . | . | 11qB5 | . | 0 | 1 |
| chr11 | 74364795 | A | C | 221 | Olfr412 | exonic | missense SNV | Olfr412:NM_001011851:exon1:c.A125C:p.N42T | . | 11qB5 | . | 0 | 1 |
| chr11 | 74388890 | G | T | 221 | Rap1gap2 | intronic | . | . | . | 11qB5 | . | 0 | 1 |
| chr11 | 74666421 | A | T | 222 | Cluh | intronic | . | . | . | 11qB5 | . | 0 | 1 |
| chr11 | 74679671 | T | C | 228 | Pafah1b1 | intronic | . | . | . | 11qB5 | rs29439948 | 1 | 0 |
| chr11 | 74816843 | G | C | 89 | Mettl16 | intronic | . | . | . | 11qB5 | . | 0 | 1 |
| chr11 | 74859894 | C | T | 38.1072 | Sgsm2 | exonic | missense SNV | Sgsm2:NM_197943:exon12:c.G1294A:p.G432S | . | 11qB5 | . | 0 | 1 |
| chr11 | 74910383 | T | A | 228 | Srr | intronic | . | . | . | 11qB5 | rs29413824 | 1 | 0 |
| chr11 | 75167660 | T | C | 109 | Hic1 | intronic | . | . | 4020 | 11qB5 | . | 0 | 1 |
| chr11 | 75301303 | C | G | 222 | Rpa1 | UTR3 | . | . | . | 11qB5 | . | 0 | 1 |
| chr11 | 75349963 | C | A | 104 | Smyd4 | intronic | . | . | . | 11qB5 | . | 0 | 1 |
| chr11 | 75503113 | A | G | 74 | Prpf8 | intronic | . | . | . | 11qB5 | . | 0 | 1 |
| chr11 | 75505166 | G | T | 221 | Prpf8 | exonic | missense SNV | Prpf8:NM_138659:exon36:c.G5865T:p.K1955N | . | 11qB5 | . | 0 | 1 |
| chr11 | 75510733 | G | A | 222 | Rilp | exonic | missense SNV | Rilp:NM_001029938:exon3:c.G421A:p.E141K | 605 | 11qB5 | . | 0 | 1 |
| chr11 | 75599909 | G | A | 222 | Gm12338,Pitpna | ncRNA_exonic | . | . | . | 11qB5 | . | 0 | 1 |
| chr11 | 75599919 | G | A | 222 | Gm12338,Pitpna | ncRNA_exonic | . | . | . | 11qB5 | . | 0 | 1 |
| chr11 | 75599997 | G | A | 75 | Gm12338,Pitpna | ncRNA_exonic | . | . | . | 11qB5 | . | 0 | 1 |
| chr11 | 75619700 | T | C | 93 | Pitpna | intronic | . | . | . | 11qB5 | . | 0 | 1 |
| chr11 | 75703441 | C | T | 221 | Crk | exonic | missense SNV | Crk:NM_133656:exon3:c.C853T:p.H285Y | . | 11qB5 | . | 0 | 1 |
| chr11 | 76027551 | C | A | 60 | Fam101b | exonic | synonymous SNV | Fam101b:NM_029658:exon1:c.G87T:p.S29S | 1016 | 11qB5 | . | 0 | 1 |
| chr11 | 76027553 | A | G | 60 | Fam101b | exonic | missense SNV | Fam101b:NM_029658:exon1:c.T85C:p.S29P | 1016 | 11qB5 | . | 0 | 1 |

| | | | | | | | | | | | | | |
|---|---|---|---|---|---|---|---|---|---|---|---|---|---|
| chr11 | 76672338 | C | T | 50 | . | upstream | . | . | . | 11qB5 | . | 0 | 1 |
| chr11 | 76754567 | C | A | 222 | Gosr1 | intronic | . | . | . | 11qB5 | . | 0 | 1 |
| chr11 | 77914889 | C | T | 83 | Gm11190 | ncRNA_exonic | . | . | . | 11qB5 | . | 0 | 1 |
| chr11 | 78195042 | C | G | 113 | Proca1 | intronic | . | . | . | 11qB5 | . | 0 | 1 |
| chr11 | 78201611 | G | A | 134 | Proca1 | intronic | . | . | . | 11qB5 | . | 0 | 1 |
| chr11 | 78222422 | C | A | 221 | Supt6 | exonic | missense SNV | Supt6:NM_009297:exon22:c.G2764T:p.D922Y | . | 11qB5 | . | 0 | 1 |
| chr11 | 78284657 | T | A | 212 | 2610507B11Rik | intronic | . | . | . | 11qB5 | . | 0 | 1 |
| chr11 | 78959808 | G | A | 127 | Nos2 | UTR3 | . | . | . | 11qB5 | . | 0 | 1 |
| chr11 | 79245936 | C | A | 99 | Wsb1 | intronic | . | . | . | 11qB5 | . | 0 | 1 |
| chr11 | 79386583 | T | C | 114 | Nf1 | intronic | . | . | . | 11qB5 | . | 0 | 1 |
| chr11 | 79547114 | G | A | 222 | Nf1 | exonic | synonymous SNV | Nf1:NM_010897:exon42:c.G6297A:p.A2099A | . | 11qB5 | rs28228793 | 0 | 1 |
| chr11 | 79940786 | C | T | 222 | Utp6 | intronic | . | . | . | 11qB5 | . | 0 | 1 |
| chr11 | 80032048 | C | A | 222 | Suz12 | exonic | missense SNV | Suz12:NM_001163018:exon15:c.C2015A:p.S672Y,Suz12:NM_199196:exon16:c.C2084A:p.S695Y | . | 11qB5 | . | 0 | 1 |
| chr11 | 80127547 | T | A | 160 | Atad5 | intronic | . | . | . | 11qB5 | . | 0 | 1 |
| chr11 | 80242629 | G | C | 222 | Rhot1 | exonic | synonymous SNV | Rhot1:NM_001163354:exon10:c.G735C:p.V245V,Rhot1:NM_001163355:exon10:c.G735C:p.V245V,Rhot1:NM | . | 11qB5 | . | 0 | 1 |
| chr11 | 80243557 | A | G | 221 | Rhot1 | intronic | . | . | . | 11qB5 | . | 0 | 1 |
| chr11 | 80388022 | G | A | 166 | Zfp207 | intronic | . | . | . | 11qB5 | . | 0 | 1 |
| chr11 | 80682415 | C | A | 222 | Myo1d | exonic | missense SNV | Myo1d:NM_177390:exon5:c.G585T:p.Q195H | . | 11qB5 | . | 0 | 1 |
| chr11 | 80685834 | C | A | 192 | Myo1d | intronic | . | . | . | 11qB5 | . | 0 | 1 |
| chr11 | 80779765 | C | T | 200 | Myo1d | exonic | missense SNV | Myo1d:NM_177390:exon1:c.G28A:p.G10S | 670 | 11qB5 | . | 0 | 1 |
| chr11 | 80823176 | T | C | 168 | . | intergenic | . | . | . | 11qB5 | . | 0 | 1 |
| chr11 | 80894091 | G | A | 140 | Asic2 | exonic | missense SNV | Asic2:NM_001034013:exon4:c.C868T:p.R290W,Asic2:NM_007384:exon4:c.C1021T:p.R341W | . | 11qB5 | . | 0 | 1 |
| chr11 | 81863706 | A | T | 221 | 5530401A14Rik,Asi | ncRNA_exonic | . | . | . | 11qB5 | . | 0 | 1 |
| chr11 | 82722342 | T | C | 217 | Cct6b | intronic | . | . | . | 11qC | . | 0 | 1 |
| chr11 | 82807407 | T | A | 73 | Rffl | intronic | . | . | . | 11qC | . | 0 | 1 |
| chr11 | 83281825 | A | G | 183 | Slfn14 | exonic | missense SNV | Slfn14:NM_001166028:exon3:c.T1156C:p.F386L | . | 11qC | . | 0 | 1 |
| chr11 | 83779089 | G | A | 46.5932 | Heatr6 | intronic | . | . | . | 11qC | . | 0 | 1 |
| chr11 | 84276448 | G | C | 203 | Acaca | intronic | . | . | . | 11qC | . | 0 | 1 |
| chr11 | 84290161 | T | A | 130 | Acaca | intronic | . | . | . | 11qC | . | 0 | 1 |
| chr11 | 84448947 | C | T | 66 | Aatf | intronic | . | . | . | 11qC | . | 0 | 1 |
| chr11 | 84519457 | G | T | 55 | Lhx1 | UTR3 | . | . | . | 11qC | rs4228957 | 0 | 1 |
| chr11 | 84524016 | C | T | 194 | Lhx1 | exonic | missense SNV | Lhx1:NM_008498:exon1:c.G160A:p.D54N | . | 11qC | . | 0 | 1 |
| chr11 | 84882545 | C | T | 80 | Myo19 | intronic | . | . | . | 11qC | . | 0 | 1 |
| chr11 | 85111504 | C | T | 222 | Usp32 | intronic | . | . | . | 11qC | . | 0 | 1 |
| chr11 | 85509300 | A | G | 132 | Bcas3 | intronic | . | . | . | 11qC | . | 0 | 1 |
| chr11 | 85911374 | G | T | 221 | Tbx4 | intronic | . | . | . | 11qC | . | 0 | 1 |
| chr11 | 85914667 | G | T | 222 | Tbx4 | exonic | missense SNV | Tbx4:NM_172798:exon7:c.G1281T:p.Q427H,Tbx4:NM_011536:exon8:c.G1581T:p.Q527H | . | 11qC | . | 0 | 1 |
| chr11 | 86493190 | T | C | 192 | Mir467c,Rnft1 | exonic | synonymous SNV | Rnft1:NM_029788:exon7:c.T915C:p.T305T | . | 11qC | . | 0 | 1 |
| chr11 | 86726239 | C | A | 151 | Cltc,Mir8115 | ncRNA_intronic | . | . | . | 11qC | . | 0 | 1 |
| chr11 | 86771740 | C | T | 98 | Dhx40,Mir8115 | ncRNA_intronic | . | . | . | 11qC | . | 0 | 1 |
| chr11 | 87045029 | A | T | 103 | Mir8115,Gdpd1 | ncRNA_intronic | . | . | . | 11qC | . | 0 | 1 |
| chr11 | 87177805 | T | G | 101 | Mir8115,Trim37 | ncRNA_intronic | . | . | . | 11qC | . | 0 | 1 |
| chr11 | 87402451 | G | T | 140 | Rad51c,Mir8115 | ncRNA_intronic | . | . | . | 11qC | . | 0 | 1 |
| chr11 | 87404891 | A | C | 124 | Rad51c,Mir8115 | ncRNA_intronic | . | . | . | 11qC | . | 0 | 1 |
| chr11 | 87426892 | A | T | 222 | Tex14,Mir8115 | ncRNA_intronic | . | . | . | 11qC | . | 0 | 1 |
| chr11 | 87542754 | T | G | 61 | Tex14 | intronic | . | . | . | 11qC | . | 0 | 1 |
| chr11 | 87549412 | C | A | 205 | Tex14 | intronic | . | . | . | 11qC | . | 0 | 1 |
| chr11 | 87597166 | G | T | 184 | Mtmr4 | intronic | . | . | . | 11qC | . | 0 | 1 |
| chr11 | 87738311 | C | G | 222 | Supt4a | intronic | . | . | . | 11qC | . | 0 | 1 |
| chr11 | 87874743 | G | A | 153 | Epx | intronic | . | . | . | 11qC | . | 0 | 1 |
| chr11 | 88933507 | A | C | 221 | Scpep1os,Scpep1 | exonic | missense SNV | Scpep1:NM_029023:exon10:c.T935G:p.L312R | . | 11qC | . | 0 | 1 |
| chr11 | 89639580 | T | G | 221 | . | intergenic | . | . | . | 11qC | . | 0 | 1 |
| chr11 | 90001273 | G | A | 221 | Pctp | intronic | . | . | . | 11qC | . | 0 | 1 |
| chr11 | 90370035 | G | T | 152 | Hlf | intronic | . | . | . | 11qC | rs49449401 | 1 | 0 |
| chr11 | 90594719 | G | A | 228 | Stxbp4 | intronic | . | . | . | 11qD | rs29385932 | 1 | 0 |
| chr11 | 90607146 | T | C | 222 | Stxbp4 | exonic | missense SNV | Stxbp4:NM_011505:exon6:c.A326G:p.K109R | . | 11qD | . | 0 | 1 |
| chr11 | 92770261 | A | C | 178 | . | intergenic | . | . | . | 11qD | . | 0 | 1 |
| chr11 | 92793178 | G | A | 67 | . | intergenic | . | . | . | 11qD | . | 0 | 1 |
| chr11 | 94351137 | T | C | 142 | Abcc3 | intronic | . | . | . | 11qD | . | 0 | 1 |
| chr11 | 94361506 | G | A | 66 | Abcc3 | intronic | . | . | . | 11qD | . | 0 | 1 |
| chr11 | 94363796 | T | C | 228 | Abcc3 | exonic | synonymous SNV | Abcc3:NM_029600:exon16:c.A1962G:p.A654A | . | 11qD | rs29458192 | 1 | 0 |
| chr11 | 94418828 | C | A | 142 | Cacna1g | exonic | missense SNV | Cacna1g:NM_001112813:exon26:c.G4857T:p.Q1619H,Cacna1g:NM_001177888:exon26:c.G4836T:p.Q1612H,( | . | 11qD | . | 0 | 1 |
| chr11 | 94443281 | C | A | 99 | Cacna1g | exonic | missense SNV | Cacna1g:NM_001112813:exon11:c.G2533T:p.V845F,Cacna1g:NM_001177888:exon11:c.G2533T:p.V845F,Cac | . | 11qD | . | 0 | 1 |
| chr11 | 94503697 | T | A | 222 | Mycbpap | intronic | . | . | . | 11qD | . | 0 | 1 |
| chr11 | 94635042 | C | T | 222 | Lrrc59 | intronic | . | . | . | 11qD | . | 0 | 1 |
| chr11 | 94638336 | G | T | 115 | Lrrc59 | intronic | . | . | . | 11qD | rs29436909 | 1 | 0 |
| chr11 | 94650632 | T | G | 168 | Eme1 | exonic | synonymous SNV | Eme1:NM_177752:exon2:c.A363C:p.T121T | . | 11qD | . | 0 | 1 |
| chr11 | 94668066 | G | C | 139 | Xylt2 | exonic | missense SNV | Xylt2:NM_145828:exon7:c.C1477G:p.L493V | . | 11qD | . | 0 | 1 |
| chr11 | 94742554 | G | T | 221 | . | intergenic | . | . | . | 11qD | . | 0 | 1 |
| chr11 | 94779155 | T | C | 222 | Tmem92 | intronic | . | . | . | 11qD | . | 0 | 1 |
| chr11 | 96147320 | G | C | 116 | Tt116 | intronic | . | . | . | 11qD | . | 0 | 1 |
| chr11 | 96249015 | C | A | 221 | . | intergenic | . | . | . | 11qD | . | 0 | 1 |
| chr11 | 96287022 | G | A | 69 | Hoxb7 | exonic | missense SNV | Hoxb7:NM_010460:exon1:c.G295A:p.V99M | 772 | 11qD | . | 0 | 1 |
| chr11 | 96336277 | T | C | 79 | Hoxb3 | intronic | . | . | . | 11qD | . | 0 | 1 |
| chr11 | 96336442 | G | A | 221 | Hoxb3 | intronic | . | . | . | 11qD | . | 0 | 1 |
| chr11 | 96943945 | T | A | 101 | Pnpo | UTR5 | . | . | . | 11qD | . | 0 | 1 |
| chr11 | 96963293 | A | C | 147 | D030028A08Rik,Sp2 | ncRNA_intronic | . | . | . | 11qD | . | 0 | 1 |
| chr11 | 97373206 | G | T | 221 | Socs7 | intronic | . | . | . | 11qD | . | 0 | 1 |
| chr11 | 97938341 | G | C | 98 | Plxdc1 | intronic | . | . | . | 11qD | . | 0 | 1 |
| chr11 | 98350954 | G | T | 222 | 1700003D09Rik,Pnm | ncRNA_exonic | . | . | . | 11qD | . | 0 | 1 |
| chr11 | 98378621 | G | T | 64 | Stard3 | intronic | . | . | . | 11qD | . | 0 | 1 |
| chr11 | 98397998 | G | T | 216 | Pgap3 | exonic | synonymous SNV | Pgap3:NM_001033537:exon3:c.C330A:p.A110A | . | 11qD | . | 0 | 1 |
| chr11 | 98453796 | G | C | 190 | Grb7 | exonic | missense SNV | Grb7:NM_010346:exon10:c.G1024C:p.G342R | . | 11qD | . | 0 | 1 |
| chr11 | 98702434 | C | G | 211 | Csf3 | exonic | missense SNV | Csf3:NM_009971:exon4:c.C376G:p.Q126E | . | 11qD | . | 0 | 1 |
| chr11 | 98997746 | T | A | 164 | Top2a | intronic | . | . | . | 11qD | . | 0 | 1 |
| chr11 | 99421693 | A | T | 222 | Krt12 | exonic | missense SNV | Krt12:NM_010661:exon1:c.T524A:p.L175Q | . | 11qD | . | 0 | 1 |
| chr11 | 99437560 | T | G | 30.3309 | Krt20 | intronic | . | . | . | 11qD | . | 0 | 1 |
| chr11 | 99538583 | C | A | 134 | Krt40 | exonic | synonymous SNV | Krt40:NM_001317213:exon4:c.G924T:p.T308T,Krt40:NM_001039666:exon6:c.G1164T:p.T388T | . | 11qD | . | 0 | 1 |
| chr11 | 99539950 | C | A | 222 | Krt40 | exonic | missense SNV | Krt40:NM_001317213:exon3:c.G657T:p.E219D,Krt40:NM_001039666:exon5:c.G897T:p.E299D | . | 11qD | . | 0 | 1 |
| chr11 | 99837840 | C | T | 222 | 2300003K06Rik | exonic | missense SNV | 2300003K06Rik:NM_001195383:exon1:c.G176A:p.C59Y | . | 11qD | . | 0 | 1 |
| chr11 | 99880036 | A | G | 218 | Gm11567 | UTR3 | . | . | . | 11qD | rs254747707 | 0 | 1 |
| chr11 | 99880322 | T | C | 38.9956 | . | downstream | . | . | . | 11qD | . | 0 | 1 |
| chr11 | 99908677 | A | G | 222 | Krtap31-1 | UTR3 | . | . | . | 11qD | . | 0 | 1 |
| chr11 | 99908773 | G | T | 218 | Krtap31-1 | UTR3 | . | . | . | 11qD | . | 0 | 1 |
| chr11 | 99948904 | A | G | 221 | Krtap9-5 | exonic | missense SNV | Krtap9-5:NM_001085527:exon1:c.A430G:p.T144A | . | 11qD | . | 0 | 1 |
| chr11 | 99978446 | G | C | 222 | . | intergenic | . | . | . | 11qD | . | 0 | 1 |
| chr11 | 100016032 | G | C | 165 | Krt33a | exonic | missense SNV | Krt33a:NM_027983:exon1:c.C116G:p.A39G | . | 11qD | . | 0 | 1 |
| chr11 | 100204894 | T | C | 174 | Krt14 | intronic | . | . | . | 11qD | . | 0 | 1 |
| chr11 | 100206942 | A | C | 99 | Krt14 | exonic | missense SNV | Krt14:NM_001313956:exon1:c.T514G:p.F172V,Krt14:NM_016958:exon1:c.T514G:p.F172V | . | 11qD | . | 0 | 1 |
| chr11 | 100231922 | C | A | 142 | . | intergenic | . | . | . | 11qD | . | 0 | 1 |
| chr11 | 100321176 | A | C | 222 | Eif1 | intronic | . | . | . | 11qD | . | 0 | 1 |
| chr11 | 100434545 | C | A | 53 | Nt5c3b | intronic | . | . | . | 11qD | . | 0 | 1 |
| chr11 | 100516021 | C | T | 221 | Acly | splicing | . | . | . | 11qD | . | 0 | 1 |
| chr11 | 100711353 | C | T | 221 | Kat2a | exonic | missense SNV | Kat2a:NM_001038010:exon3:c.G574A:p.V192I,Kat2a:NM_020004:exon3:c.G574A:p.V192I | . | 11qD | . | 0 | 1 |
| chr11 | 101030555 | G | T | 144 | Atp6v0a1 | intronic | . | . | . | 11qD | . | 0 | 1 |
| chr11 | 101038132 | G | T | 221 | Atp6v0a1 | intronic | . | . | . | 11qD | . | 0 | 1 |
| chr11 | 101265235 | G | T | 221 | Wnk4 | intronic | . | . | 294 | 11qD | . | 0 | 1 |
| chr11 | 101277187 | T | C | 177 | Wnk4 | UTR3 | . | . | . | 11qD | . | 0 | 1 |
| chr11 | 101410393 | T | C | 222 | Aarsd1 | exonic | synonymous SNV | Aarsd1:NM_144829:exon8:c.A822G:p.A274A | . | 11qD | . | 0 | 1 |
| chr11 | 101627646 | C | T | 228 | . | upstream | . | . | . | 11qD | . | 1 | 0 |
| chr11 | 101774133 | C | A | 147 | Etv4 | exonic | missense SNV | Etv4:NM_001316365:exon7:c.G496T:p.A166S,Etv4:NM_001316366:exon7:c.G478T:p.A160S,Etv4:NM_0088 | . | 11qD | . | 0 | 1 |
| chr11 | 101981946 | G | C | 70 | Dusp3 | intronic | . | . | . | 11qD | . | 0 | 1 |
| chr11 | 101990939 | A | T | 221 | 1700006E09Rik | intronic | . | . | . | 11qD | . | 0 | 1 |
| chr11 | 102147853 | G | A | 222 | Nags | exonic | synonymous SNV | Nags:NM_145829:exon5:c.G1119A:p.V373V | . | 11qD | . | 0 | 1 |
| chr11 | 102155755 | C | A | 222 | Tmem101 | exonic | missense SNV | Tmem101:NM_029649:exon2:c.G287T:p.G96V | . | 11qD | . | 0 | 1 |
| chr11 | 102460369 | T | G | 221 | Itga2b | intronic | . | . | . | 11qD | . | 0 | 1 |
| chr11 | 103190275 | G | C | 205 | Fmnl1 | intronic | . | . | . | 11qE1 | . | 0 | 1 |
| chr11 | 103340179 | A | T | 111 | Arhgap27 | exonic | missense SNV | Arhgap27:NM_133715:exon2:c.T283A:p.S95T,Arhgap27:NM_001205236:exon4:c.T880A:p.S294T | . | 11qE1 | . | 0 | 1 |
| chr11 | 103494212 | A | T | 112 | . | intergenic | . | . | . | 11qE1 | . | 0 | 1 |
| chr11 | 103494218 | T | A | 221 | . | intergenic | . | . | . | 11qE1 | . | 0 | 1 |
| chr11 | 103683788 | C | A | 222 | Gosr2 | splicing | . | . | . | 11qE1 | . | 0 | 1 |
| chr11 | 103730721 | C | T | 168 | Wnt9b | UTR3 | . | . | . | 11qE1 | . | 0 | 1 |
| chr11 | 103873491 | C | T | 35.4799 | Nsf | intronic | . | . | . | 11qE1 | . | 0 | 1 |
| chr11 | 103910566 | T | C | 221 | Nsf | exonic | missense SNV | Nsf:NM_008740:exon8:c.A622G:p.I208V | . | 11qE1 | . | 0 | 1 |
| chr11 | 103931023 | C | T | 74 | Nsf | intronic | . | . | . | 11qE1 | . | 0 | 1 |
| chr11 | 104295066 | A | T | 146 | Mapt | intronic | . | . | . | 11qE1 | . | 0 | 1 |
| chr11 | 104307618 | G | C | 45.8132 | Mapt | intronic | . | . | . | 11qE1 | . | 0 | 1 |
| chr11 | 104491589 | T | A | 97 | . | intergenic | . | . | . | 11qE1 | . | 0 | 1 |
| chr11 | 104512776 | C | A | 95 | Cdc27 | intronic | . | . | . | 11qE1 | . | 0 | 1 |
| chr11 | 104720542 | T | A | 222 | . | intergenic | . | . | . | 11qE1 | . | 0 | 1 |
| chr11 | 104739374 | C | T | 222 | . | intergenic | . | . | . | 11qE1 | . | 0 | 1 |
| chr11 | 105131523 | G | A | 112 | Mettl2 | intronic | . | . | . | 11qE1 | . | 0 | 1 |
| chr11 | 106284131 | G | T | 78 | Tcam1 | exonic | synonymous SNV | Tcam1:NM_029467:exon4:c.G411T:p.V137V | . | 11qE1 | . | 0 | 1 |
| chr11 | 106300775 | G | T | 222 | Gh | exonic | missense SNV | Gh:NM_008117:exon4:c.C440A:p.A147D | . | 11qE1 | . | 0 | 1 |
| chr11 | 106301337 | G | T | 188 | Gh | intronic | . | . | . | 11qE1 | . | 0 | 1 |
| chr11 | 106812540 | A | G | 222 | Cep95 | intronic | . | . | . | 11qE1 | . | 0 | 1 |
| chr11 | 106996687 | T | C | 221 | Kpna2 | intronic | . | . | . | 11qE1 | . | 0 | 1 |
| chr11 | 107052692 | T | C | 146 | Bptf | intronic | . | . | . | 11qE1 | rs48365903 | 1 | 0 |
| chr11 | 107296352 | C | T | 203 | Pitpnc1 | intronic | . | . | . | 11qE1 | . | 0 | 1 |
| chr11 | 107479603 | C | T | 207 | Psmd12 | exonic | synonymous SNV | Psmd12:NM_025894:exon1:c.C27T:p.A9A | 464 | 11qE1 | . | 0 | 1 |
| chr11 | 107636193 | G | A | 222 | Helz | exonic | missense SNV | Helz:NM_198298:exon19:c.G2506A:p.A836T | . | 11qE1 | . | 0 | 1 |
| chr11 | 107670365 | G | C | 222 | Helz | exonic | missense SNV | Helz:NM_198298:exon28:c.G4252C:p.A1418P | . | 11qE1 | . | 0 | 1 |
| chr11 | 108860162 | A | C | 221 | Cep112 | intronic | . | . | . | 11qE1 | . | 0 | 1 |
| chr11 | 109248356 | C | A | 153 | Rgs9 | intronic | . | . | . | 11qE1 | . | 0 | 1 |
| chr11 | 109281488 | G | C | 221 | Rgs9 | exonic | missense SNV | Rgs9:NM_001165934:exon2:c.C115G:p.Q39E,Rgs9:NM_011268:exon2:c.C115G:p.Q39E | . | 11qE1 | . | 0 | 1 |
| chr11 | 109583961 | C | T | 134 | Wipi1 | intronic | . | . | . | 11qE1 | . | 0 | 1 |
| chr11 | 110060191 | A | G | 122 | Abca8a | intronic | . | . | . | 11qE1 | . | 0 | 1 |
| chr11 | 110108665 | G | A | 221 | Abca9 | exonic | stopgain | Abca9:NM_147220:exon34:c.C4363T:p.Q1455X | . | 11qE1 | . | 0 | 1 |
| chr11 | 110187943 | T | G | 221 | Abca6 | exonic | missense SNV | Abca6:NM_147218:exon29:c.A3720C:p.K1240N | . | 11qE1 | . | 0 | 1 |
| chr11 | 110273201 | G | T | 160 | Abca5 | intronic | . | . | . | 11qE1 | . | 0 | 1 |
| chr11 | 110273253 | C | T | 222 | Abca5 | intronic | . | . | . | 11qE1 | . | 0 | 1 |
| chr11 | 110273255 | A | G | 222 | Abca5 | intronic | . | . | . | 11qE1 | rs216072799 | 0 | 1 |
| chr11 | 110278330 | T | C | 221 | Abca5 | exonic | missense SNV | Abca5:NM_147219:exon32:c.A4085G:p.Y1362C | . | 11qE1 | . | 0 | 1 |
| chr11 | 110492755 | C | A | 175 | Map2k6 | intronic | . | . | . | 11qE2 | . | 0 | 1 |
| chr11 | 111026040 | G | T | 222 | Kcnj16 | UTR3 | . | . | . | 11qE2 | . | 0 | 1 |
| chr11 | 113703240 | G | T | 222 | Cpsf4l | intronic | . | . | . | 11qE2 | . | 0 | 1 |
| chr11 | 113867151 | C | A | 108 | Sdk2 | intronic | . | . | . | 11qE2 | . | 0 | 1 |
| chr11 | 113868067 | G | A | 221 | Sdk2 | exonic | missense SNV | Sdk2:NM_172800:exon11:c.C1330T:p.R444C | . | 11qE2 | . | 0 | 1 |
| chr11 | 114790398 | G | C | 47.9525 | Kif19a | UTR3 | . | . | . | 11qE2 | . | 0 | 1 |
| chr11 | 114791848 | C | T | 196 | Btbd17 | exonic | missense SNV | Btbd17:NM_028055:exon3:c.G1037A:p.S346N | 903 | 11qE2 | . | 0 | 1 |
| chr11 | 114864204 | C | T | 221 | Gprc5c | exonic | missense SNV | Gprc5c:NM_001110337:exon2:c.C706T:p.R236C,Gprc5c:NM_001110338:exon2:c.C706T:p.R236C,Gprc5c:NM | . | 11qE2 | . | 0 | 1 |
| chr11 | 114890403 | G | A | 175 | Cd300a | exonic | synonymous SNV | Cd300a:NM_170758:exon1:c.G39A:p.L13L | . | 11qE2 | . | 0 | 1 |
| chr11 | 114987349 | T | G | 221 | Cd300ld | exonic | missense SNV | Cd300ld:NM_145437:exon2:c.A336C:p.K112N | . | 11qE2 | . | 0 | 1 |
| chr11 | 115001431 | T | C | 215 | AF251705 | intronic | . | . | . | 11qE2 | . | 0 | 1 |
| chr11 | 115120551 | C | A | 23.0242 | Cd300lf,Rab37 | intronic | . | . | . | 11qE2 | . | 0 | 1 |
| chr11 | 115183186 | T | G | 144 | Nat9 | exonic | missense SNV | Nat9:NM_025400:exon7:c.A566C:p.Q189P | . | 11qE2 | . | 0 | 1 |
| chr11 | 115249462 | G | A | 222 | Grin2c | UTR3 | . | . | . | 11qE2 | . | 0 | 1 |
| chr11 | 115446853 | G | A | 222 | Trim80 | intronic | . | . | . | 11qE2 | . | 0 | 1 |
| chr11 | 115882271 | G | A | 222 | . | intergenic | . | . | . | 11qE2 | . | 0 | 1 |
| chr11 | 115883401 | C | A | 222 | . | intergenic | . | . | . | 11qE2 | . | 0 | 1 |
| chr11 | 115935524 | A | C | 156 | Sap30bp | intronic | . | . | . | 11qE2 | . | 0 | 1 |
| chr11 | 116135178 | C | G | 221 | Mrpl38 | intronic | . | . | . | 11qE2 | . | 0 | 1 |
| chr11 | 116147708 | C | T | 72 | Fbf1 | exonic | synonymous SNV | Fbf1:NM_172571:exon24:c.G2586A:p.Q862Q | . | 11qE2 | . | 0 | 1 |
| chr11 | 116160134 | C | G | 222 | Fbf1 | intronic | . | . | . | 11qE2 | . | 0 | 1 |
| chr11 | 116183320 | C | G | 105 | Acox1 | intronic | . | . | . | 11qE2 | . | 0 | 1 |
| chr11 | 116189618 | A | G | 139 | Acox1 | intronic | . | . | . | 11qE2 | . | 0 | 1 |
| chr11 | 116649688 | G | A | 35.8373 | Cygb | intronic | . | . | . | 11qE2 | . | 0 | 1 |
| chr11 | 116842911 | A | T | 120 | Jmjd6 | intronic | . | . | 1375 | 11qE2 | . | 0 | 1 |
| chr11 | 116865956 | G | T | 221 | Mfsd11 | exonic | missense SNV | Mfsd11:NM_178620:exon9:c.G748T:p.G250C | . | 11qE2 | . | 0 | 1 |
| chr11 | 116948437 | C | G | 34.827 | Mgat5b | intronic | . | . | . | 11qE2 | . | 0 | 1 |
| chr11 | 118000046 | T | A | 128 | Pgs1 | intronic | . | . | . | 11qE2 | . | 0 | 1 |
| chr11 | 118036616 | T | G | 38.6781 | Dnah17 | intronic | . | . | . | 11qE2 | . | 0 | 1 |
| chr11 | 118098768 | C | G | 70 | Dnah17 | intronic | . | . | . | 11qE2 | . | 0 | 1 |
| chr11 | 118407996 | C | A | 221 | Cant1 | exonic | missense SNV | Cant1:NM_001267591:exon4:c.G940T:p.D314Y,Cant1:NM_001025617:exon5:c.G940T:p.D314Y,Cant1:NM_0( | . | 11qE2 | . | 0 | 1 |
| chr11 | 118446558 | T | A | 222 | C1qtnf1,Gm11747 | exonic | synonymous SNV | C1qtnf1:NM_001204129:exon3:c.T213A:p.S71S,C1qtnf1:NM_001204130:exon3:c.T213A:p.S71S,C1qtnf1:N | . | 11qE2 | . | 0 | 1 |

| | | | | | | | | | | | | | |
|---|---|---|---|---|---|---|---|---|---|---|---|---|---|
| chr11 | 118485672 | C | T | 76 | Engase | intronic | . | . | . | 11qE2 | rs233531652 | 0 | 1 |
| chr11 | 118503666 | G | T | 114 | Rbfox3 | intronic | . | . | . | 11qE2 | . | 0 | 1 |
| chr11 | 119283776 | C | T | 208 | Gaa | exonic | synonymous SNV | Gaa:NM_001159324:exon17:c.C2448T:p.N816N,Gaa:NM_008064:exon17:c.C2448T:p.N816N | . | 11qE2 | . | 0 | 1 |
| chr11 | 119542649 | C | G | 221 | Nptx1 | exonic | missense SNV | Nptx1:NM_008730:exon5:c.G1123C:p.E375Q | . | 11qE2 | . | 0 | 1 |
| chr11 | 119547567 | C | G | 178 | Nptx1 | exonic | missense SNV | Nptx1:NM_008730:exon1:c.G23C:p.R8P | 2840 | 11qE2 | . | 0 | 1 |
| chr11 | 119657866 | A | C | 107 | Rptor | intronic | . | . | . | 11qE2 | . | 0 | 1 |
| chr11 | 119743785 | T | C | 228 | Rptor | intronic | . | . | . | 11qE2 | rs29415544 | 1 | 0 |
| chr11 | 120478134 | A | T | 79 | Hgs | intronic | . | . | . | 11qE2 | . | 0 | 1 |
| chr11 | 120624630 | C | G | 65 | Sirt7 | intronic | . | . | 618 | 11qE2 | . | 0 | 1 |
| chr11 | 120720664 | G | T | 128 | Lrrc45 | exonic | missense SNV | Lrrc45:NM_153545:exon18:c.G1863T:p.E621D | . | 11qE2 | . | 0 | 1 |
| chr11 | 120919654 | C | T | 177 | Ccdc57 | intronic | . | . | . | 11qE2 | . | 0 | 1 |
| chr11 | 121117825 | A | G | 76 | Tex19.2 | splicing | . | . | . | 11qE2 | . | 0 | 1 |
| chr12 | 3237866 | G | T | 139 | Rab10os | ncRNA intronic | . | . | . | 12qA1.1 | . | 1 | 0 |
| chr12 | 3237867 | T | G | 173 | Rab10os | ncRNA intronic | . | . | . | 12qA1.1 | . | 1 | 0 |
| chr12 | 3644195 | A | G | 160 | Dtnb | intronic | . | . | . | 12qA1.1 | . | 0 | 1 |
| chr12 | 3904075 | T | C | 221 | Dnmt3a | intronic | . | . | . | 12qA1.1 | . | 0 | 1 |
| chr12 | 5018147 | G | A | 220 | Atad2b | intronic | . | . | . | 12qA1.1 | . | 0 | 1 |
| chr12 | 6966745 | A | G | 222 | . | intergenic | . | . | . | 12qA1.1 | . | 0 | 1 |
| chr12 | 7990620 | G | A | 228 | Apob | intronic | . | . | . | 12qA1.1 | rs51514130 | 1 | 0 |
| chr12 | 7995645 | A | C | 221 | Apob | exonic | missense SNV | Apob:NM_009693:exon18:c.A2789C:p.K930T | . | 12qA1.1 | . | 0 | 1 |
| chr12 | 8323787 | C | A | 220 | Hs1bp3 | intronic | . | . | . | 12qA1.1 | . | 0 | 1 |
| chr12 | 8342569 | G | T | 219 | Hs1bp3 | UTR3 | . | . | . | 12qA1.1 | . | 0 | 1 |
| chr12 | 8718960 | T | G | 221 | Pum2 | intronic | . | . | . | 12qA1.1 | . | 0 | 1 |
| chr12 | 16974995 | T | A | 221 | Rock2 | intronic | . | . | . | 12qA1.1 | . | 0 | 1 |
| chr12 | 17547540 | A | C | 218 | Odc1 | intronic | . | . | . | 12qA1.1 | . | 0 | 1 |
| chr12 | 21295130 | G | T | 220 | Cpsf3 | exonic | stopgain | Cpsf3:NM_018813:exon5:c.G442T:p.G148X | . | 12qA1.3 | . | 0 | 1 |
| chr12 | 21295131 | G | T | 220 | Cpsf3 | exonic | missense SNV | Cpsf3:NM_018813:exon5:c.G443T:p.G148V | . | 12qA1.3 | . | 0 | 1 |
| chr12 | 26314079 | C | T | 222 | Rnf144a | exonic | missense SNV | Rnf144a:NM_080563:exon8:c.G688A:p.G230R,Rnf144a:NM_001081977:exon9:c.G688A:p.G230R | . | 12qA2 | . | 0 | 1 |
| chr12 | 27342414 | G | C | 126 | Sox11 | UTR5 | . | . | 2275 | 12qA2 | . | 1 | 0 |
| chr12 | 28657930 | G | T | 196 | Rnaseh1 | intronic | . | . | . | 12qA2 | rs50848289 | 1 | 0 |
| chr12 | 28752004 | G | T | 219 | Tssc1 | exonic | missense SNV | Tssc1:NM_201357:exon1:c.G42T:p.Q14H | 242 | 12qA2 | . | 0 | 1 |
| chr12 | 30125099 | G | C | 133 | Tpo | intronic | . | . | . | 12qA2 | . | 0 | 1 |
| chr12 | 30131784 | C | G | 227 | Tpo | intronic | . | . | . | 12qA2 | . | 1 | 0 |
| chr12 | 31531592 | C | A | 176 | Slc26a4 | intronic | . | . | . | 12qA2 | rs262895565 | 1 | 0 |
| chr12 | 31869777 | C | G | 205 | Cog5 | intronic | . | . | . | 12qA3 | . | 0 | 1 |
| chr12 | 32847088 | A | C | 117 | Nampt | intronic | . | . | . | 12qA3 | . | 0 | 1 |
| chr12 | 34985676 | C | T | 222 | Prps1l1 | exonic | synonymous SNV | Prps1l1:NM_029294:exon1:c.C789T:p.G263G | . | 12qA3 | . | 0 | 1 |
| chr12 | 35500589 | G | A | 222 | Ahr | UTR3 | . | . | . | 12qA3 | . | 0 | 1 |
| chr12 | 35504451 | A | G | 222 | Ahr | exonic | synonymous SNV | Ahr:NM_001314027:exon9:c.T1317C:p.T439T,Ahr:NM_013464:exon10:c.T1668C:p.T556T | . | 12qA3 | . | 0 | 1 |
| chr12 | 38100241 | T | A | 129 | Dgkb | intronic | . | . | . | 12qA3 | . | 0 | 1 |
| chr12 | 38368889 | A | G | 79 | Dgkb | intronic | . | . | . | 12qA3 | . | 0 | 1 |
| chr12 | 43812450 | C | T | 221 | . | intergenic | . | . | . | 12qB1 | . | 0 | 1 |
| chr12 | 46700859 | C | G | 219 | Nova1 | exonic | missense SNV | Nova1:NM_021361:exon5:c.G634C:p.D212H | . | 12qB3 | . | 0 | 1 |
| chr12 | 48047438 | A | C | 163 | . | intergenic | . | . | . | 12qB3 | . | 0 | 1 |
| chr12 | 49391241 | A | T | 45.51 | 3110039M20Rik | ncRNA intronic | . | . | . | 12qB3 | . | 0 | 1 |
| chr12 | 49776280 | G | A | 53 | . | intergenic | . | . | . | 12qB3 | . | 0 | 1 |
| chr12 | 51593772 | C | T | 219 | Coch | exonic | synonymous SNV | Coch:NM_007728:exon2:c.C69T:p.F23F,Coch:NM_001198835:exon3:c.C69T:p.F23F | 943 | 12qB3 | . | 0 | 1 |
| chr12 | 52559977 | T | C | 222 | Arhgap5 | exonic | missense SNV | Arhgap5:NM_009706:exon4:c.T3956C:p.I1319T | . | 12qC1 | . | 0 | 1 |
| chr12 | 52938301 | C | T | 221 | Akap6 | intronic | . | . | . | 12qC1 | . | 0 | 1 |
| chr12 | 53010488 | C | T | 222 | Akap6 | exonic | synonymous SNV | Akap6:NM_198111:exon8:c.C2736T:p.L912L | . | 12qC1 | . | 0 | 1 |
| chr12 | 55820609 | C | T | 21.61 | Ralgapa1 | intronic | . | . | 725 | 12qC1 | . | 0 | 1 |
| chr12 | 56051142 | C | T | 219 | . | intergenic | . | . | . | 12qC1 | . | 0 | 1 |
| chr12 | 56738760 | A | T | 144 | Slc25a21 | intronic | . | . | . | 12qC1 | . | 0 | 1 |
| chr12 | 57576429 | C | A | 220 | Ttc6 | exonic | synonymous SNV | Ttc6:NM_001312644:exon2:c.C613A:p.R205R | 668 | 12qC1 | . | 0 | 1 |
| chr12 | 57705539 | A | G | 219 | Ttc6 | exonic | synonymous SNV | Ttc6:NM_001312644:exon24:c.A4230G:p.A1410A | . | 12qC1 | . | 0 | 1 |
| chr12 | 58268608 | A | G | 72 | Clec14a | exonic | missense SNV | Clec14a:NM_025809:exon1:c.T227C:p.L76P | . | 12qC1 | . | 0 | 1 |
| chr12 | 64877095 | C | A | 220 | . | intergenic | . | . | . | 12qC1 | . | 0 | 1 |
| chr12 | 64943220 | G | T | 74 | Klhl28 | UTR3 | . | . | . | 12qC1 | . | 0 | 1 |
| chr12 | 65021774 | T | G | 215 | Fam179b | UTR3 | . | . | . | 12qC1 | . | 0 | 1 |
| chr12 | 69159156 | C | G | 190 | Rps29 | UTR5 | . | . | 672 | 12qC2 | . | 0 | 1 |
| chr12 | 69701965 | G | A | 64 | L2hgdh | intronic | . | . | . | 12qC2 | . | 0 | 1 |
| chr12 | 72500755 | G | A | 221 | Lrrc9 | exonic | missense SNV | Lrrc9:NM_001142728:exon28:c.G3703A:p.D1235N | . | 12qC3 | . | 0 | 1 |
| chr12 | 73123829 | A | G | 228 | Mnat1 | UTR5 | . | . | . | 12qC3 | rs29193059 | 1 | 0 |
| chr12 | 75888429 | A | G | 193 | Syne2 | intronic | . | . | . | 12qC3 | rs29173065 | 0 | 1 |
| chr12 | 76206979 | G | T | 221 | Tex21 | exonic | synonymous SNV | Tex21:NM_001159532:exon8:c.C1159A:p.R387R,Tex21:NM_019784:exon8:c.C1159A:p.R387R | . | 12qC3 | . | 0 | 1 |
| chr12 | 76822132 | G | A | 220 | Rab15 | intronic | . | . | 680 | 12qC3 | . | 0 | 1 |
| chr12 | 78562866 | G | A | 220 | Gphn | exonic | missense SNV | Gphn:NM_145965:exon10:c.G983A:p.R328K,Gphn:NM_172952:exon11:c.G974A:p.R325K | . | 12qC3 | . | 0 | 1 |
| chr12 | 79054770 | C | A | 183 | Plekhh1 | exonic | missense SNV | Plekhh1:NM_181073:exon7:c.C613A:p.P205T | . | 12qC3 | . | 0 | 1 |
| chr12 | 79236530 | T | C | 228 | Zfyve26 | intronic | . | . | . | 12qC3 | rs29143813 | 1 | 0 |
| chr12 | 80475933 | G | A | 102 | Exd2 | exonic | synonymous SNV | Exd2:NM_133798:exon2:c.G165A:p.Q55Q | . | 12qC3 | . | 0 | 1 |
| chr12 | 81722385 | T | G | 222 | Map3k9 | exonic | missense SNV | Map3k9:NM_001174107:exon11:c.A2888C:p.E963A | . | 12qD1 | . | 0 | 1 |
| chr12 | 81995409 | T | G | 57 | Pcnx | intronic | . | . | . | 12qD1 | . | 0 | 1 |
| chr12 | 83660675 | C | A | 221 | Rbm25 | exonic | missense SNV | Rbm25:NM_027349:exon9:c.C861A:p.H287Q | . | 12qD1 | . | 0 | 1 |
| chr12 | 83674614 | A | G | 163 | Rbm25 | exonic | synonymous SNV | Rbm25:NM_027349:exon15:c.A1953G:p.Q651Q | . | 12qD1 | . | 0 | 1 |
| chr12 | 83777007 | T | G | 222 | Papln | intronic | . | . | . | 12qD1 | rs13481565 | 0 | 1 |
| chr12 | 84109196 | T | C | 221 | Acot6 | exonic | missense SNV | Acot6:NM_172580:exon3:c.T917C:p.L306P | . | 12qD1 | . | 0 | 1 |
| chr12 | 84109417 | G | A | 222 | Acot6 | exonic | missense SNV | Acot6:NM_172580:exon3:c.G1138A:p.V380I | . | 12qD1 | . | 0 | 1 |
| chr12 | 86012767 | A | C | 221 | Ttll5 | exonic | missense SNV | Ttll5:NM_001081423:exon31:c.A3687C:p.K1229N | . | 12qD2 | . | 0 | 1 |
| chr12 | 86161130 | C | T | 132 | Ift43 | intronic | . | . | . | 12qD2 | . | 0 | 1 |
| chr12 | 87069206 | G | A | 222 | Tmem63c | exonic | missense SNV | Tmem63c:NM_172583:exon10:c.G620A:p.G207E | . | 12qD2 | . | 0 | 1 |
| chr12 | 87081860 | T | G | 221 | Tmem63c | exonic | missense SNV | Tmem63c:NM_172583:exon20:c.T1658G:p.L553R | . | 12qD2 | . | 0 | 1 |
| chr12 | 87253011 | T | C | 30.82 | Vipas39 | intronic | . | . | . | 12qD2 | . | 0 | 1 |
| chr12 | 89260299 | G | A | 27.687 | Nrxn3 | exonic | missense SNV | Nrxn3:NM_172544:exon6:c.G703A:p.V235M,Nrxn3:NM_001198587:exon7:c.G1822A:p.V608M | 366 | 12qD3 | . | 0 | 1 |
| chr12 | 89260441 | A | C | 115 | Nrxn3 | exonic | missense SNV | Nrxn3:NM_172544:exon6:c.A845C:p.N282T,Nrxn3:NM_001198587:exon7:c.A1964C:p.N655T | 366 | 12qD3 | . | 0 | 1 |
| chr12 | 90332225 | T | C | 221 | Nrxn3 | exonic | synonymous SNV | Nrxn3:NM_001252074:exon7:c.T1519C:p.L507L,Nrxn3:NM_001198587:exon20:c.T4531C:p.L1511L,Nrxn3:N | . | 12qD3 | . | 0 | 1 |
| chr12 | 90729732 | C | T | 222 | Dio2 | exonic | unknown | UNKNOWN | . | 12qD3 | . | 0 | 1 |
| chr12 | 91740809 | G | T | 222 | Ston2 | intronic | . | . | . | 12qD3 | . | 0 | 1 |
| chr12 | 98275092 | G | A | 222 | Gpr65 | exonic | missense SNV | Gpr65:NM_008152:exon2:c.G3A:p.M1I | . | 12qE | . | 0 | 1 |
| chr12 | 98805387 | G | T | 162 | Eml5 | intronic | . | . | . | 12qE | . | 0 | 1 |
| chr12 | 100332579 | C | G | 115 | Ttc7b | exonic | missense SNV | Ttc7b:NM_001033213:exon18:c.G2107C:p.A703P | . | 12qE | . | 0 | 1 |
| chr12 | 100923332 | C | G | 192 | Ccdc88c | exonic | missense SNV | Ccdc88c:NM_026681:exon26:c.G4451C:p.R1484P | . | 12qE | . | 0 | 1 |
| chr12 | 101065100 | A | G | 222 | Smek1 | intronic | . | . | . | 12qE | . | 0 | 1 |
| chr12 | 101463261 | A | G | 166 | Catsperb | intronic | . | . | . | 12qE | . | 0 | 1 |
| chr12 | 101591361 | G | A | 129 | Catsperb | intronic | . | . | . | 12qE | . | 0 | 1 |
| chr12 | 101690002 | T | G | 126 | Tc2n | intronic | . | . | . | 12qE | . | 0 | 1 |
| chr12 | 101709577 | G | A | 74 | Tc2n | intronic | . | . | . | 12qE | . | 0 | 1 |
| chr12 | 101845146 | G | A | 193 | Trip11 | exonic | missense SNV | Trip11:NM_028446:exon19:c.C5477T:p.T1826I | . | 12qE | . | 0 | 1 |
| chr12 | 102229011 | G | A | 22.5533 | Slc24a4 | intronic | . | . | . | 12qE | . | 0 | 1 |
| chr12 | 102351101 | G | T | 110 | Rin3 | intronic | . | . | . | 12qE | . | 0 | 1 |
| chr12 | 102675324 | C | A | 221 | Itpk1 | intronic | . | . | . | 12qE | . | 0 | 1 |
| chr12 | 102766264 | C | G | 222 | Ubr7 | exonic | missense SNV | Ubr7:NM_025666:exon6:c.C514G:p.P172A | . | 12qE | . | 0 | 1 |
| chr12 | 103047044 | G | C | 221 | Unc79 | exonic | missense SNV | Unc79:NM_001081017:exon9:c.G1052C:p.G351A | . | 12qE | . | 0 | 1 |
| chr12 | 103088397 | A | G | 110 | Unc79 | intronic | . | . | . | 12qE | . | 0 | 1 |
| chr12 | 103125420 | T | G | 121 | Unc79 | intronic | . | . | . | 12qE | . | 0 | 1 |
| chr12 | 103165557 | T | C | 222 | Unc79 | intronic | . | . | . | 12qE | . | 0 | 1 |
| chr12 | 103408087 | A | G | 123 | Ddx24 | UTR3 | . | . | . | 12qE | . | 0 | 1 |
| chr12 | 103601731 | A | C | 97 | Ppp4r4 | intronic | . | . | . | 12qE | . | 0 | 1 |
| chr12 | 103672403 | G | T | 222 | Serpina16 | intronic | . | . | . | 12qE | . | 0 | 1 |
| chr12 | 104116553 | T | G | 222 | Serpina3a | exonic | missense SNV | Serpina3a:NM_001167705:exon2:c.T584G:p.L195R | . | 12qE | . | 0 | 1 |
| chr12 | 104119683 | C | A | 222 | Serpina3a | exonic | stopgain | Serpina3a:NM_028740:exon3:c.C396A:p.Y132X,Serpina3a:NM_001167705:exon4:c.C966A:p.Y322X | . | 12qE | . | 0 | 1 |
| chr12 | 104152160 | C | T | 87 | Serpina3c | intronic | . | . | . | 12qE | . | 0 | 1 |
| chr12 | 104174473 | A | T | 196 | . | intergenic | . | . | . | 12qE | . | 0 | 1 |
| chr12 | 104315048 | A | G | 222 | Serpina3j | exonic | missense SNV | Serpina3j:NM_001101472:exon1:c.A479G:p.H160R | . | 12qE | . | 0 | 1 |
| chr12 | 104392684 | T | C | 222 | Serpina3m | exonic | synonymous SNV | Serpina3m:NM_009253:exon4:c.T954C:p.T318T | . | 12qE | . | 0 | 1 |
| chr12 | 104692412 | C | T | 222 | Dicer1 | exonic | synonymous SNV | Dicer1:NM_148948:exon25:c.G5370A:p.K1790K | . | 12qE | . | 0 | 1 |
| chr12 | 104696893 | G | A | 221 | Dicer1 | exonic | synonymous SNV | Dicer1:NM_148948:exon23:c.C4428T:p.G1476G | . | 12qE | . | 0 | 1 |
| chr12 | 105644433 | A | G | 201 | Atg2b | intronic | . | . | . | 12qE | . | 0 | 1 |
| chr12 | 106036733 | T | C | 191 | Vrk1 | intronic | . | . | . | 12qE | . | 0 | 1 |
| chr12 | 106459365 | C | G | 37.9885 | . | intergenic | . | . | . | 12qF1 | . | 0 | 1 |
| chr12 | 107917232 | C | A | 222 | Bcl11b | exonic | missense SNV | Bcl11b:NM_001286343:exon2:c.G241T:p.A81S,Bcl11b:NM_021399:exon3:c.G607T:p.A203S,Bcl11b:NM_001 | 2010 | 12qF1 | . | 0 | 1 |
| chr12 | 108157639 | T | A | 144 | Setd3 | intronic | . | . | . | 12qF1 | . | 0 | 1 |
| chr12 | 108357339 | C | G | 54 | Cyp46a1 | intronic | . | . | . | 12qF1 | . | 0 | 1 |
| chr12 | 108714168 | A | G | 145 | . | intergenic | . | . | . | 12qF1 | . | 0 | 1 |
| chr12 | 109549677 | G | A | 156 | Meg3 | ncRNA exonic | . | . | . | 12qF1 | . | 0 | 1 |
| chr12 | 109713751 | G | A | 46.1437 | . | downstream | . | . | . | 12qF1 | . | 0 | 1 |
| chr12 | 110654970 | G | T | 222 | Dync1h1 | intronic | . | . | . | 12qF1 | . | 0 | 1 |
| chr12 | 111321407 | T | C | 228 | Cdc42bpb | intronic | . | . | . | 12qF1 | rs29179635 | 1 | 0 |
| chr12 | 111485167 | G | A | 165 | Gm266 | UTR3 | . | . | 851 | 12qF1 | . | 0 | 1 |
| chr12 | 111671141 | A | C | 106 | Ckb | intronic | . | . | 1972 | 12qF1 | . | 0 | 1 |
| chr12 | 111858250 | C | T | 222 | Ppp1r13b | exonic | missense SNV | Ppp1r13b:NM_011625:exon4:c.G314A:p.S105N | . | 12qF1 | . | 0 | 1 |
| chr12 | 112122845 | C | A | 45.9261 | Aspg | intronic | . | . | . | 12qF1 | . | 0 | 1 |
| chr12 | 113546565 | C | A | 124 | . | downstream | . | . | . | 12qF1 | . | 0 | 1 |
| chr12 | 113896240 | T | G | 89 | . | intergenic | . | . | . | 12qF1 | . | 0 | 1 |
| chr12 | 113897117 | T | G | 124 | . | intergenic | . | . | . | 12qF1 | . | 0 | 1 |
| chr12 | 114366445 | A | T | 123 | . | intergenic | . | . | . | 12qF1 | . | 0 | 1 |
| chr12 | 114462330 | T | C | 222 | . | intergenic | . | . | . | 12qF2 | . | 0 | 1 |
| chr12 | 114630848 | C | A | 122 | . | intergenic | . | . | . | 12qF2 | . | 0 | 1 |
| chr12 | 114945874 | A | C | 217 | . | intergenic | . | . | . | 12qF2 | . | 0 | 1 |
| chr12 | 114991151 | C | T | 222 | . | intergenic | . | . | . | 12qF2 | . | 0 | 1 |
| chr12 | 116137381 | C | G | 222 | Vipr2 | intronic | . | . | . | 12qF2 | . | 0 | 1 |
| chr12 | 117151066 | T | A | 222 | Gm10421,Ptprn2 | ncRNA exonic | . | . | . | 12qF2 | . | 0 | 1 |
| chr12 | 118140523 | C | T | 195 | Dnah11 | intronic | . | . | . | 12qF2 | . | 0 | 1 |
| chr12 | 118299049 | G | C | 222 | Sp4 | exonic | missense SNV | Sp4:NM_001166385:exon3:c.C1261G:p.Q421E,Sp4:NM_009239:exon3:c.C1261G:p.Q421E | . | 12qF2 | . | 0 | 1 |
| chr12 | 118873096 | A | C | 32.4022 | Abcb5 | intronic | . | . | . | 12qF2 | . | 0 | 1 |
| chr12 | 118874879 | T | A | 152 | Abcb5 | intronic | . | . | . | 12qF2 | rs236853964 | 0 | 1 |
| chr12 | 119877233 | A | C | 128 | . | intergenic | . | . | . | 12qF2 | . | 0 | 1 |
| chr13 | 3803933 | G | A | 197 | Calml3 | exonic | stopgain | Calml3:NM_027416:exon1:c.C271T:p.R91X | . | 13qA1 | . | 0 | 1 |
| chr13 | 3928052 | T | A | 90 | Tuba13 | intronic | . | . | . | 13qA1 | . | 0 | 1 |
| chr13 | 4197642 | C | A | 100 | Akr1c13 | intronic | . | . | . | 13qA1 | . | 0 | 1 |
| chr13 | 4275795 | C | A | 221 | Akr1c12 | exonic | stopgain | Akr1c12:NM_013777:exon3:c.G298T:p.E100X | . | 13qA1 | . | 0 | 1 |
| chr13 | 4382574 | C | A | 137 | . | intergenic | . | . | . | 13qA1 | . | 0 | 1 |
| chr13 | 4457126 | A | T | 222 | Akr1c6 | intronic | . | . | . | 13qA1 | . | 0 | 1 |
| chr13 | 4607274 | G | A | 126 | Akr1e1 | intronic | . | . | . | 13qA1 | . | 0 | 1 |
| chr13 | 6570690 | G | C | 222 | Pitrm1 | exonic | missense SNV | Pitrm1:NM_145131:exon20:c.G2324C:p.C775S | . | 13qA1 | . | 0 | 1 |
| chr13 | 8569551 | A | G | 89 | Adarb2 | intronic | . | . | . | 13qA1 | . | 0 | 1 |
| chr13 | 9596460 | A | C | 222 | Dip2c | intronic | . | . | . | 13qA1 | . | 0 | 1 |
| chr13 | 9698815 | C | T | 101 | Zmynd11 | intronic | . | . | . | 13qA1 | . | 0 | 1 |
| chr13 | 9878146 | T | C | 221 | Chrm3 | exonic | missense SNV | Chrm3:NM_033269:exon5:c.A853G:p.S285G | . | 13qA1 | . | 0 | 1 |
| chr13 | 11584016 | C | T | 111 | Ryr2 | intronic | . | . | . | 13qA1 | . | 0 | 1 |
| chr13 | 11588039 | G | C | 141 | Ryr2 | intronic | . | . | . | 13qA1 | . | 0 | 1 |
| chr13 | 11605183 | G | T | 86 | Ryr2 | exonic | missense SNV | Ryr2:NM_023868:exon85:c.C11527A:p.Q3843K | . | 13qA1 | . | 0 | 1 |
| chr13 | 11621167 | G | T | 130 | Ryr2 | intronic | . | . | . | 13qA1 | . | 0 | 1 |
| chr13 | 11716923 | T | C | 221 | Ryr2 | intronic | . | . | . | 13qA1 | . | 0 | 1 |
| chr13 | 11721555 | A | G | 109 | Ryr2 | intronic | . | . | . | 13qA1 | . | 0 | 1 |
| chr13 | 11811078 | G | T | 109 | Ryr2 | intronic | . | . | . | 13qA1 | . | 0 | 1 |
| chr13 | 11940710 | T | C | 154 | Ryr2 | intronic | . | . | . | 13qA1 | . | 0 | 1 |
| chr13 | 12378595 | A | T | 221 | . | intergenic | . | . | . | 13qA1 | . | 0 | 1 |
| chr13 | 12421021 | G | C | 149 | Heatr1 | intronic | . | . | . | 13qA1 | . | 0 | 1 |
| chr13 | 12448603 | A | C | 170 | Lgals8 | intronic | . | . | . | 13qA1 | rs49775858 | 1 | 0 |
| chr13 | 13232921 | G | T | 51 | . | intergenic | . | . | 255 | 13qA1 | . | 0 | 1 |
| chr13 | 13710618 | G | C | 95 | Lyst | intronic | . | . | . | 13qA1 | . | 0 | 1 |
| chr13 | 14316151 | C | T | 221 | Hecw1 | exonic | missense SNV | Hecw1:NM_001081348:exon10:c.G2255A:p.S752N | . | 13qA1 | . | 0 | 1 |
| chr13 | 14636940 | C | T | 210 | AW209491 | exonic | missense SNV | AW209491:NM_001104646:exon2:c.C377T:p.A126V,AW209491:NM_134067:exon2:c.C377T:p.A126V | . | 13qA1 | . | 0 | 1 |
| chr13 | 15118907 | A | C | 204 | . | intergenic | . | . | . | 13qA1 | . | 0 | 1 |
| chr13 | 15725309 | G | A | 222 | Gli3 | exonic | missense SNV | Gli3:NM_008130:exon15:c.G3280A:p.D1094N | . | 13qA1 | . | 0 | 1 |
| chr13 | 16976108 | A | G | 228 | Sugct | intronic | . | . | . | 13qA2 | rs45753332 | 1 | 0 |
| chr13 | 16976217 | A | G | 228 | Sugct | exonic | synonymous SNV | Sugct:NM_138654:exon13:c.T1087C:p.L363L | . | 13qA2 | rs45872465 | 1 | 0 |
| chr13 | 16976322 | G | T | 206 | Sugct | intronic | . | . | . | 13qA2 | rs49968542 | 1 | 0 |
| chr13 | 17252518 | G | A | 228 | Sugct | exonic | synonymous SNV | Sugct:NM_138654:exon12:c.C1056T:p.S352S | . | 13qA2 | rs13481710 | 1 | 0 |
| chr13 | 17252740 | G | T | 158 | Sugct | intronic | . | . | . | 13qA2 | rs29573258 | 1 | 0 |
| chr13 | 17644601 | T | C | 168 | Sugct | intronic | . | . | . | 13qA2 | rs50491137 | 1 | 0 |
| chr13 | 17644744 | G | A | 228 | Sugct | intronic | . | . | . | 13qA2 | rs51038051 | 1 | 0 |
| chr13 | 17644763 | A | G | 228 | Sugct | intronic | . | . | . | 13qA2 | rs216763607 | 1 | 0 |
| chr13 | 17644925 | T | G | 228 | Sugct | intronic | . | . | . | 13qA2 | rs46980153 | 1 | 0 |
| chr13 | 17644944 | T | C | 228 | Sugct | intronic | . | . | . | 13qA2 | rs48746993 | 1 | 0 |

| | | | | | | | | | | | | | |
|---|---|---|---|---|---|---|---|---|---|---|---|---|---|
| chr13 | 17671605 | G | T | 178 | Sugct | intronic | . | . | . | 13qA2 | rs48061876 | 1 | 0 |
| chr13 | 17671678 | C | T | 228 | Sugct | intronic | . | . | . | 13qA2 | rs45882590 | 1 | 0 |
| chr13 | 17671806 | T | C | 228 | Sugct | intronic | . | . | . | 13qA2 | rs48300048 | 1 | 0 |
| chr13 | 17671848 | A | G | 228 | Sugct | intronic | . | . | . | 13qA2 | rs45891166 | 1 | 0 |
| chr13 | 17671901 | T | A | 159 | Sugct | intronic | . | . | . | 13qA2 | rs47735592 | 1 | 0 |
| chr13 | 17672339 | A | T | 168 | Sugct | intronic | . | . | . | 13qA2 | rs48005810 | 1 | 0 |
| chr13 | 17672628 | A | G | 228 | Sugct | intronic | . | . | . | 13qA2 | rs48188659 | 1 | 0 |
| chr13 | 17697067 | T | C | 228 | Mplkip | UTR3 | . | . | . | 13qA2 | rs48036909 | 1 | 0 |
| chr13 | 17718130 | A | G | 228 | Cdk13 | exonic | synonymous SNV | Cdk13:NM_001081058:exon14:c.T4467C:p.Y1489Y,Cdk13:NM_027118:exon14:c.T4287C:p.Y1429Y | . | 13qA2 | rs29772415 | 1 | 0 |
| chr13 | 17719185 | T | C | 172 | Cdk13 | intronic | . | . | . | 13qA2 | rs29495745 | 1 | 0 |
| chr13 | 17738755 | C | A | 170 | Cdk13 | intronic | . | . | . | 13qA2 | rs45929579 | 1 | 0 |
| chr13 | 17751643 | T | C | 228 | Cdk13 | intronic | . | . | . | 13qA2 | rs29565252 | 1 | 0 |
| chr13 | 17763147 | G | A | 228 | Cdk13 | exonic | synonymous SNV | Cdk13:NM_001081058:exon5:c.C2310T:p.I770I,Cdk13:NM_027118:exon5:c.C2310T:p.I770I | . | 13qA2 | rs29251859 | 1 | 0 |
| chr13 | 17764816 | A | G | 168 | Cdk13 | intronic | . | . | . | 13qA2 | rs46259880 | 1 | 0 |
| chr13 | 17765180 | T | G | 228 | Cdk13 | intronic | . | . | . | 13qA2 | rs29227549 | 1 | 0 |
| chr13 | 17821047 | C | T | 228 | . | intergenic | . | . | . | 13qA2 | rs254736417 | 1 | 0 |
| chr13 | 17821634 | G | C | 228 | . | intergenic | . | . | . | 13qA2 | rs13481711 | 1 | 0 |
| chr13 | 18038827 | G | A | 200 | . | intergenic | . | . | . | 13qA2 | rs29231807 | 1 | 0 |
| chr13 | 18129278 | G | A | 228 | Pou6f2 | intronic | . | . | . | 13qA2 | rs29874739 | 1 | 0 |
| chr13 | 18138982 | T | A | 228 | Pou6f2 | intronic | . | . | . | 13qA2 | rs30062315 | 1 | 0 |
| chr13 | 18139879 | C | T | 112 | Pou6f2 | intronic | . | . | . | 13qA2 | rs29252676 | 1 | 0 |
| chr13 | 18151875 | T | A | 183 | Pou6f2 | intronic | . | . | . | 13qA2 | rs46363497 | 1 | 0 |
| chr13 | 18239429 | C | A | 193 | Pou6f2 | intronic | . | . | . | 13qA2 | rs48668484 | 1 | 0 |
| chr13 | 18239483 | G | T | 228 | Pou6f2 | exonic | missense SNV | Pou6f2:NM_175006:exon3:c.C706A:p.P236T | . | 13qA2 | rs50906382 | 1 | 0 |
| chr13 | 18239726 | G | A | 124 | Pou6f2 | exonic | missense SNV | Pou6f2:NM_175006:exon3:c.C463T:p.P155S | . | 13qA2 | rs29821949 | 1 | 0 |
| chr13 | 18378725 | C | T | 163 | Pou6f2 | intronic | . | . | . | 13qA2 | rs29956525 | 1 | 0 |
| chr13 | 18381984 | G | A | 228 | Pou6f2 | exonic | missense SNV | Pou6f2:NM_175006:exon1:c.C56T:p.A19V | . | 13qA2 | rs29234524 | 1 | 0 |
| chr13 | 18381995 | G | C | 228 | Pou6f2 | exonic | missense SNV | Pou6f2:NM_175006:exon1:c.C45G:p.D15E | . | 13qA2 | rs29250924 | 1 | 0 |
| chr13 | 18382016 | C | T | 228 | Pou6f2 | exonic | synonymous SNV | Pou6f2:NM_175006:exon1:c.G24A:p.L8L | . | 13qA2 | rs47846974 | 1 | 0 |
| chr13 | 18717103 | A | G | 137 | . | upstream | . | . | . | 13qA2 | rs51567373 | 1 | 0 |
| chr13 | 18717178 | T | A | 212 | . | upstream | . | . | . | 13qA2 | rs29225604 | 1 | 0 |
| chr13 | 18717483 | G | T | 170 | Vps41 | intronic | . | . | . | 13qA2 | rs29493953 | 1 | 0 |
| chr13 | 18717545 | T | C | 128 | Vps41 | intronic | . | . | . | 13qA2 | rs29709501 | 1 | 0 |
| chr13 | 18739430 | T | A | 228 | Vps41 | intronic | . | . | . | 13qA2 | rs49062514 | 1 | 0 |
| chr13 | 18739605 | A | G | 228 | Vps41 | intronic | . | . | . | 13qA2 | rs29730115 | 1 | 0 |
| chr13 | 18745070 | G | A | 182 | Vps41 | intronic | . | . | . | 13qA2 | rs29247820 | 1 | 0 |
| chr13 | 18745355 | A | C | 228 | Vps41 | intronic | . | . | . | 13qA2 | rs29528107 | 1 | 0 |
| chr13 | 18791484 | G | A | 213 | Vps41 | intronic | . | . | . | 13qA2 | rs29730947 | 1 | 0 |
| chr13 | 18810542 | C | T | 228 | Vps41 | exonic | synonymous SNV | Vps41:NM_172120:exon8:c.C558T:p.A186A | . | 13qA2 | rs13473673 | 1 | 0 |
| chr13 | 18811617 | C | T | 228 | Vps41 | exonic | synonymous SNV | Vps41:NM_172120:exon9:c.C618T:p.P206P | . | 13qA2 | rs13473676 | 1 | 0 |
| chr13 | 18811790 | G | A | 228 | Vps41 | intronic | . | . | . | 13qA2 | rs29224879 | 1 | 0 |
| chr13 | 18814116 | G | A | 227 | Vps41 | intronic | . | . | . | 13qA2 | rs29802516 | 1 | 0 |
| chr13 | 18814268 | T | G | 185 | Vps41 | exonic | missense SNV | Vps41:NM_172120:exon10:c.T756G:p.D252E | . | 13qA2 | . | 0 | 1 |
| chr13 | 18827453 | C | T | 169 | Vps41 | intronic | . | . | . | 13qA2 | rs29550956 | 1 | 0 |
| chr13 | 18829080 | A | C | 170 | Vps41 | intronic | . | . | . | 13qA2 | rs29553606 | 1 | 0 |
| chr13 | 18838678 | C | T | 228 | Vps41 | exonic | synonymous SNV | Vps41:NM_172120:exon15:c.C1209T:p.H403H | . | 13qA2 | rs50639951 | 1 | 0 |
| chr13 | 18838699 | C | T | 228 | Vps41 | exonic | synonymous SNV | Vps41:NM_172120:exon15:c.C1230T:p.Y410Y | . | 13qA2 | rs50939212 | 1 | 0 |
| chr13 | 18838838 | C | T | 173 | Vps41 | intronic | . | . | . | 13qA2 | rs52321794 | 1 | 0 |
| chr13 | 18839496 | A | G | 170 | Vps41 | intronic | . | . | . | 13qA2 | rs50676936 | 1 | 0 |
| chr13 | 18839854 | A | G | 168 | Vps41 | intronic | . | . | . | 13qA2 | rs47964094 | 1 | 0 |
| chr13 | 18841144 | T | C | 228 | Vps41 | intronic | . | . | . | 13qA2 | rs51133968 | 1 | 0 |
| chr13 | 18841360 | G | A | 163 | Vps41 | intronic | . | . | . | 13qA2 | rs48409468 | 1 | 0 |
| chr13 | 18841361 | A | T | 136 | Vps41 | intronic | . | . | . | 13qA2 | rs50457863 | 1 | 0 |
| chr13 | 18841952 | G | A | 140 | Vps41 | intronic | . | . | . | 13qA2 | rs47748667 | 1 | 0 |
| chr13 | 18842027 | C | T | 180 | Vps41 | intronic | . | . | . | 13qA2 | rs47868162 | 1 | 0 |
| chr13 | 18842030 | T | A | 180 | Vps41 | intronic | . | . | . | 13qA2 | rs46188481 | 1 | 0 |
| chr13 | 18842215 | G | A | 228 | Vps41 | exonic | synonymous SNV | Vps41:NM_172120:exon18:c.G1482A:p.Q494Q | . | 13qA2 | rs51286363 | 1 | 0 |
| chr13 | 18842320 | A | G | 228 | Vps41 | intronic | . | . | . | 13qA2 | rs49550479 | 1 | 0 |
| chr13 | 18845694 | T | C | 139 | Vps41 | intronic | . | . | . | 13qA2 | rs30031038 | 1 | 0 |
| chr13 | 18846173 | A | G | 131 | Vps41 | intronic | . | . | . | 13qA2 | rs48879692 | 1 | 0 |
| chr13 | 18848843 | A | G | 228 | Vps41 | intronic | . | . | . | 13qA2 | rs29866899 | 1 | 0 |
| chr13 | 18852250 | C | T | 195 | Vps41 | intronic | . | . | . | 13qA2 | rs29494096 | 1 | 0 |
| chr13 | 18852324 | C | T | 228 | Vps41 | intronic | . | . | . | 13qA2 | rs47455449 | 1 | 0 |
| chr13 | 18862395 | T | C | 179 | Vps41 | intronic | . | . | . | 13qA2 | rs29249555 | 1 | 0 |
| chr13 | 18862548 | A | G | 228 | Vps41 | exonic | synonymous SNV | Vps41:NM_172120:exon26:c.A2268G:p.R756R | . | 13qA2 | rs29226497 | 1 | 0 |
| chr13 | 18864131 | G | A | 228 | Vps41 | intronic | . | . | . | 13qA2 | rs13481714 | 1 | 0 |
| chr13 | 19094910 | T | C | 228 | Amph | intronic | . | . | . | 13qA2 | rs29902408 | 1 | 0 |
| chr13 | 19099669 | T | C | 101 | Amph | intronic | . | . | . | 13qA2 | rs46781517 | 1 | 0 |
| chr13 | 19099775 | A | C | 184 | Amph | intronic | . | . | . | 13qA2 | rs46843049 | 1 | 0 |
| chr13 | 19099836 | G | A | 228 | Amph | intronic | . | . | . | 13qA2 | rs46669644 | 1 | 0 |
| chr13 | 19100035 | T | C | 228 | Amph | intronic | . | . | . | 13qA2 | rs30058618 | 1 | 0 |
| chr13 | 19100075 | A | G | 179 | Amph | intronic | . | . | . | 13qA2 | rs29513753 | 1 | 0 |
| chr13 | 19100484 | A | G | 228 | Amph | intronic | . | . | . | 13qA2 | rs29632668 | 1 | 0 |
| chr13 | 19102809 | T | C | 228 | Amph | intronic | . | . | . | 13qA2 | rs6289991 | 1 | 0 |
| chr13 | 19103038 | G | A | 228 | Amph | intronic | . | . | . | 13qA2 | rs6291498 | 1 | 0 |
| chr13 | 19104568 | C | T | 101 | Amph | intronic | . | . | . | 13qA2 | rs52165763 | 1 | 0 |
| chr13 | 19137584 | A | C | 168 | Amph | exonic | missense SNV | Amph:NM_001289546:exon18:c.A1444C:p.T482P,Amph:NM_175007:exon18:c.A1432C:p.T478P | . | 13qA2 | . | 0 | 1 |
| chr13 | 19137782 | A | T | 228 | Amph | intronic | . | . | . | 13qA2 | rs30031112 | 1 | 0 |
| chr13 | 19139021 | G | A | 74 | Amph | intronic | . | . | . | 13qA2 | . | 0 | 1 |
| chr13 | 19149537 | T | C | 166 | Amph | intronic | . | . | . | 13qA2 | rs48018646 | 1 | 0 |
| chr13 | 19149870 | G | A | 228 | Amph | UTR3 | . | . | . | 13qA2 | rs51720604 | 1 | 0 |
| chr13 | 19185244 | C | G | 228 | . | intergenic | . | . | . | 13qA2 | rs51933963 | 1 | 0 |
| chr13 | 19339866 | T | A | 146 | . | intergenic | . | . | . | 13qA2 | rs47686781 | 1 | 0 |
| chr13 | 19339971 | T | C | 228 | . | intergenic | . | . | . | 13qA2 | rs242549477 | 1 | 0 |
| chr13 | 19340006 | T | C | 228 | . | intergenic | . | . | . | 13qA2 | rs47371136 | 1 | 0 |
| chr13 | 19340109 | A | G | 228 | . | intergenic | . | . | . | 13qA2 | rs215793963 | 1 | 0 |
| chr13 | 19340196 | A | C | 228 | . | intergenic | . | . | . | 13qA2 | rs232370900 | 1 | 0 |
| chr13 | 19340507 | C | G | 228 | . | intergenic | . | . | . | 13qA2 | rs255824559 | 1 | 0 |
| chr13 | 19340508 | C | T | 228 | . | intergenic | . | . | . | 13qA2 | rs230969831 | 1 | 0 |
| chr13 | 19342085 | T | C | 228 | . | intergenic | . | . | . | 13qA2 | rs29238859 | 1 | 0 |
| chr13 | 19342293 | C | A | 228 | . | intergenic | . | . | . | 13qA2 | rs29571275 | 1 | 0 |
| chr13 | 19342422 | A | T | 167 | . | intergenic | . | . | . | 13qA2 | rs29825124 | 1 | 0 |
| chr13 | 19344674 | G | A | 228 | . | intergenic | . | . | . | 13qA2 | rs47703809 | 1 | 0 |
| chr13 | 19344973 | T | C | 228 | . | intergenic | . | . | . | 13qA2 | rs29959972 | 1 | 0 |
| chr13 | 19367677 | A | G | 228 | Stard3nl | intronic | . | . | . | 13qA2 | rs46466427 | 1 | 0 |
| chr13 | 19367678 | T | C | 228 | Stard3nl | intronic | . | . | . | 13qA2 | rs47720097 | 1 | 0 |
| chr13 | 19370083 | G | C | 145 | Stard3nl | intronic | . | . | . | 13qA2 | rs49861983 | 1 | 0 |
| chr13 | 19370137 | T | C | 160 | Stard3nl | intronic | . | . | . | 13qA2 | rs48939168 | 1 | 0 |
| chr13 | 19370215 | G | A | 228 | Stard3nl | intronic | . | . | . | 13qA2 | rs46379797 | 1 | 0 |
| chr13 | 19370240 | A | G | 228 | Stard3nl | intronic | . | . | . | 13qA2 | rs49290638 | 1 | 0 |
| chr13 | 19370338 | T | C | 228 | Stard3nl | exonic | synonymous SNV | Stard3nl:NM_024270:exon6:c.A465G:p.V155V | . | 13qA2 | rs13461691 | 1 | 0 |
| chr13 | 19370416 | T | C | 228 | Stard3nl | intronic | . | . | . | 13qA2 | rs47891079 | 1 | 0 |
| chr13 | 19370419 | G | T | 228 | Stard3nl | intronic | . | . | . | 13qA2 | rs48625391 | 1 | 0 |
| chr13 | 19370561 | T | C | 228 | Stard3nl | intronic | . | . | . | 13qA2 | rs49169855 | 1 | 0 |
| chr13 | 19370623 | T | A | 228 | Stard3nl | intronic | . | . | . | 13qA2 | rs6207940 | 1 | 0 |
| chr13 | 19372673 | G | A | 228 | Stard3nl | intronic | . | . | . | 13qA2 | rs49357169 | 1 | 0 |
| chr13 | 19373102 | C | T | 228 | Stard3nl | intronic | . | . | . | 13qA2 | rs46172905 | 1 | 0 |
| chr13 | 19373248 | G | T | 220 | Stard3nl | intronic | . | . | . | 13qA2 | rs52645535 | 1 | 0 |
| chr13 | 19526186 | C | G | 228 | . | intergenic | . | . | . | 13qA2 | rs49700432 | 1 | 0 |
| chr13 | 19526211 | T | C | 184 | . | intergenic | . | . | . | 13qA2 | rs49238170 | 1 | 0 |
| chr13 | 19623489 | T | G | 228 | Sfrp4 | exonic | synonymous SNV | Sfrp4:NM_016687:exon1:c.T57G:p.V19V | . | 13qA2 | rs37492835 | 1 | 0 |
| chr13 | 19624011 | T | A | 153 | Sfrp4 | intronic | . | . | . | 13qA2 | rs37246612 | 1 | 0 |
| chr13 | 19624856 | T | C | 166 | Sfrp4 | intronic | . | . | . | 13qA2 | rs37307723 | 1 | 0 |
| chr13 | 19625155 | T | C | 228 | Sfrp4 | intronic | . | . | . | 13qA2 | rs47476880 | 1 | 0 |
| chr13 | 19627266 | T | C | 228 | Sfrp4 | exonic | synonymous SNV | Sfrp4:NM_016687:exon4:c.T753C:p.H251H | . | 13qA2 | rs38131344 | 1 | 0 |
| chr13 | 19627415 | C | T | 211 | Sfrp4 | intronic | . | . | . | 13qA2 | rs37729070 | 1 | 0 |
| chr13 | 19627460 | G | A | 160 | Sfrp4 | intronic | . | . | . | 13qA2 | rs38667430 | 1 | 0 |
| chr13 | 19632117 | T | G | 228 | Sfrp4 | intronic | . | . | . | 13qA2 | rs39195157 | 1 | 0 |
| chr13 | 19632370 | C | T | 206 | Sfrp4 | exonic | synonymous SNV | Sfrp4:NM_016687:exon6:c.C1047T:p.S349S | . | 13qA2 | . | 0 | 1 |
| chr13 | 19632451 | T | C | 228 | Sfrp4 | UTR3 | . | . | . | 13qA2 | rs37403064 | 1 | 0 |
| chr13 | 19645383 | C | A | 110 | Nme8 | intronic | . | . | . | 13qA2 | rs213645103 | 1 | 0 |
| chr13 | 19650742 | C | T | 228 | Nme8 | UTR3 | . | . | . | 13qA2 | rs36575965 | 1 | 0 |
| chr13 | 19652154 | T | G | 166 | Nme8 | intronic | . | . | . | 13qA2 | rs36504585 | 1 | 0 |
| chr13 | 19652243 | A | C | 152 | Nme8 | exonic | missense SNV | Nme8:NM_181591:exon15:c.T1479G:p.I493M | . | 13qA2 | . | 0 | 1 |
| chr13 | 19658174 | T | C | 155 | Nme8 | intronic | . | . | . | 13qA2 | rs37032275 | 1 | 0 |
| chr13 | 19658692 | T | C | 228 | Nme8 | intronic | . | . | . | 13qA2 | rs36520619 | 1 | 0 |
| chr13 | 19660228 | A | C | 121 | Nme8 | intronic | . | . | . | 13qA2 | rs36344517 | 1 | 0 |
| chr13 | 19660246 | T | C | 130 | Nme8 | intronic | . | . | . | 13qA2 | rs36968900 | 1 | 0 |
| chr13 | 19660415 | A | T | 228 | Nme8 | intronic | . | . | . | 13qA2 | rs36977649 | 1 | 0 |
| chr13 | 19660787 | A | G | 170 | Nme8 | intronic | . | . | . | 13qA2 | rs36382710 | 1 | 0 |
| chr13 | 19729312 | A | G | 222 | A530099J19Rik | exonic | missense SNV | A530099J19Rik:NM_175688:exon2:c.T569C:p.I190T | . | 13qA2 | . | 0 | 1 |
| chr13 | 19751747 | C | A | 221 | Gpr141 | exonic | missense SNV | Gpr141:NM_181754:exon5:c.G857T:p.G286V | . | 13qA2 | . | 0 | 1 |
| chr13 | 20185389 | T | C | 228 | Elmo1 | UTR5 | . | . | . | 13qA2 | rs29963345 | 1 | 0 |
| chr13 | 20207997 | T | C | 178 | Elmo1 | intronic | . | . | . | 13qA2 | rs37836944 | 1 | 0 |
| chr13 | 20207998 | G | A | 76 | Elmo1 | intronic | . | . | . | 13qA2 | . | 0 | 1 |
| chr13 | 20241339 | G | T | 86 | Elmo1 | intronic | . | . | . | 13qA2 | rs49566875 | 1 | 0 |
| chr13 | 20241351 | T | A | 94 | Elmo1 | intronic | . | . | . | 13qA2 | rs265501424 | 1 | 0 |
| chr13 | 20241491 | G | A | 220 | Elmo1 | intronic | . | . | . | 13qA2 | rs36998335 | 1 | 0 |
| chr13 | 20241641 | T | C | 228 | Elmo1 | intronic | . | . | . | 13qA2 | rs36793769 | 1 | 0 |
| chr13 | 20251187 | G | A | 228 | Elmo1 | intronic | . | . | . | 13qA2 | rs29762588 | 1 | 0 |
| chr13 | 20251372 | G | A | 135 | Elmo1 | intronic | . | . | . | 13qA2 | rs37024316 | 1 | 0 |
| chr13 | 20261346 | A | G | 91 | Elmo1 | intronic | . | . | . | 13qA2 | . | 0 | 1 |
| chr13 | 20274607 | C | T | 228 | Elmo1 | exonic | synonymous SNV | Elmo1:NM_080288:exon8:c.C537T:p.A179A | . | 13qA2 | rs36929683 | 1 | 0 |
| chr13 | 20280677 | A | G | 197 | Elmo1 | intronic | . | . | . | 13qA2 | rs30000136 | 1 | 0 |
| chr13 | 20282801 | A | G | 228 | Elmo1 | intronic | . | . | . | 13qA2 | rs30090910 | 1 | 0 |
| chr13 | 20283014 | G | A | 228 | Elmo1 | intronic | . | . | . | 13qA2 | rs29743711 | 1 | 0 |
| chr13 | 20286459 | T | C | 135 | Elmo1 | intronic | . | . | . | 13qA2 | rs49387319 | 1 | 0 |
| chr13 | 20286878 | T | C | 228 | Elmo1 | intronic | . | . | . | 13qA2 | rs47496635 | 1 | 0 |
| chr13 | 20286886 | G | A | 100 | Elmo1 | intronic | . | . | . | 13qA2 | . | 0 | 1 |
| chr13 | 20287820 | G | T | 228 | Elmo1 | intronic | . | . | . | 13qA2 | rs37137424 | 1 | 0 |
| chr13 | 20287821 | G | A | 228 | Elmo1 | intronic | . | . | . | 13qA2 | rs38865469 | 1 | 0 |
| chr13 | 20290290 | C | T | 228 | Elmo1 | intronic | . | . | . | 13qA2 | rs29713946 | 1 | 0 |
| chr13 | 20290561 | G | T | 228 | Elmo1 | intronic | . | . | . | 13qA2 | rs36288215 | 1 | 0 |
| chr13 | 20449313 | A | G | 167 | Elmo1 | intronic | . | . | . | 13qA2 | rs29526809 | 1 | 0 |
| chr13 | 20449398 | T | C | 228 | Elmo1 | intronic | . | . | . | 13qA2 | rs29531006 | 1 | 0 |
| chr13 | 20449406 | A | G | 228 | Elmo1 | intronic | . | . | . | 13qA2 | rs29756104 | 1 | 0 |
| chr13 | 20449492 | T | C | 228 | Elmo1 | exonic | synonymous SNV | Elmo1:NM_080288:exon16:c.T1323C:p.F441F | . | 13qA2 | rs13481720 | 1 | 0 |
| chr13 | 20564616 | A | G | 228 | Elmo1 | intronic | . | . | . | 13qA2 | rs13481721 | 1 | 0 |
| chr13 | 20564675 | C | T | 228 | Elmo1 | intronic | . | . | . | 13qA2 | rs29246123 | 1 | 0 |
| chr13 | 20572165 | A | T | 55 | Elmo1 | intronic | . | . | . | 13qA2 | . | 0 | 1 |
| chr13 | 20572225 | G | C | 179 | Elmo1 | intronic | . | . | . | 13qA2 | rs48580217 | 1 | 0 |
| chr13 | 20582156 | G | A | 228 | Elmo1 | intronic | . | . | . | 13qA2 | rs51434973 | 1 | 0 |
| chr13 | 20582193 | G | T | 228 | Elmo1 | intronic | . | . | . | 13qA2 | rs108636434 | 1 | 0 |
| chr13 | 20582361 | A | G | 228 | Elmo1 | intronic | . | . | . | 13qA2 | rs52151564 | 1 | 0 |
| chr13 | 20589571 | G | A | 228 | Elmo1 | intronic | . | . | . | 13qA2 | rs29495053 | 1 | 0 |
| chr13 | 20600159 | T | C | 228 | Elmo1 | intronic | . | . | . | 13qA2 | rs49391300 | 1 | 0 |
| chr13 | 20600411 | C | A | 168 | Elmo1 | intronic | . | . | . | 13qA2 | rs47984109 | 1 | 0 |
| chr13 | 20600426 | A | G | 161 | Elmo1 | intronic | . | . | . | 13qA2 | rs48285114 | 1 | 0 |
| chr13 | 20604984 | C | T | 182 | Elmo1 | intronic | . | . | . | 13qA2 | rs29722981 | 1 | 0 |
| chr13 | 20605218 | G | C | 228 | Elmo1 | exonic | synonymous SNV | Elmo1:NM_198093:exon7:c.G663C:p.L221L,Elmo1:NM_080288:exon22:c.G2103C:p.L701L | . | 13qA2 | rs48216607 | 1 | 0 |
| chr13 | 20704190 | G | A | 166 | . | intergenic | . | . | . | 13qA2 | rs29236682 | 1 | 0 |
| chr13 | 20704374 | T | C | 228 | . | intergenic | . | . | . | 13qA2 | rs47191692 | 1 | 0 |
| chr13 | 20704416 | C | T | 228 | . | intergenic | . | . | . | 13qA2 | rs46259332 | 1 | 0 |
| chr13 | 20704560 | A | C | 222 | . | intergenic | . | . | . | 13qA2 | . | 0 | 1 |
| chr13 | 20704761 | A | G | 228 | . | intergenic | . | . | . | 13qA2 | rs29883566 | 1 | 0 |
| chr13 | 20704787 | A | C | 228 | . | intergenic | . | . | . | 13qA2 | rs29237153 | 1 | 0 |
| chr13 | 20705001 | T | C | 228 | . | intergenic | . | . | . | 13qA2 | rs29874055 | 1 | 0 |
| chr13 | 20705242 | G | A | 228 | . | intergenic | . | . | . | 13qA2 | rs49999984 | 1 | 0 |
| chr13 | 20705342 | C | T | 228 | . | intergenic | . | . | . | 13qA2 | rs29971895 | 1 | 0 |
| chr13 | 20748508 | G | A | 228 | . | intergenic | . | . | . | 13qA2 | rs29250572 | 1 | 0 |
| chr13 | 20794175 | A | G | 85 | Aoah | UTR5 | . | . | . | 13qA2 | rs48134333 | 1 | 0 |
| chr13 | 20794329 | C | T | 228 | Aoah | UTR5 | . | . | . | 13qA2 | rs238654278 | 1 | 0 |
| chr13 | 20794376 | T | C | 228 | Aoah | UTR5 | . | . | . | 13qA2 | rs33834812 | 1 | 0 |
| chr13 | 20794546 | T | C | 228 | Aoah | intronic | . | . | . | 13qA2 | rs46204645 | 1 | 0 |
| chr13 | 20816401 | A | C | 207 | Aoah | intronic | . | . | . | 13qA2 | rs29596418 | 1 | 0 |

| | | | | | | | | | | | | | |
|---|---|---|---|---|---|---|---|---|---|---|---|---|---|
| chr13 | 20816479 | T | C | 228 | Aoah | intronic | . | . | . | 13qA2 | rs29596421 | 1 | 0 |
| chr13 | 20816640 | T | G | 228 | Aoah | intronic | . | . | . | 13qA2 | rs29597449 | 1 | 0 |
| chr13 | 20827925 | T | C | 228 | Aoah | intronic | . | . | . | 13qA2 | rs29594295 | 1 | 0 |
| chr13 | 20827929 | C | T | 228 | Aoah | intronic | . | . | . | 13qA2 | rs50877993 | 1 | 0 |
| chr13 | 20828063 | A | C | 228 | Aoah | intronic | . | . | . | 13qA2 | rs46014002 | 1 | 0 |
| chr13 | 20828103 | A | T | 228 | Aoah | intronic | . | . | . | 13qA2 | rs29594297 | 1 | 0 |
| chr13 | 20840112 | G | A | 228 | Aoah | exonic | synonymous SNV | Aoah:NM_001281854:exon3:c.G237A:p.K79K,Aoah:NM_012054:exon3:c.G237A:p.K79K | . | 13qA2 | rs29956301 | 1 | 0 |
| chr13 | 20851058 | T | C | 228 | Aoah | intronic | . | . | . | 13qA2 | rs29585225 | 1 | 0 |
| chr13 | 20851241 | A | C | 228 | Aoah | intronic | . | . | . | 13qA2 | rs29226068 | 1 | 0 |
| chr13 | 20851524 | T | C | 210 | Aoah | intronic | . | . | . | 13qA2 | rs29925481 | 1 | 0 |
| chr13 | 20900364 | C | T | 228 | Aoah | intronic | . | . | . | 13qA2 | rs29619822 | 1 | 0 |
| chr13 | 20900461 | C | T | 228 | Aoah | intronic | . | . | . | 13qA2 | rs29596964 | 1 | 0 |
| chr13 | 20900608 | A | C | 228 | Aoah | intronic | . | . | . | 13qA2 | rs213975412 | 1 | 0 |
| chr13 | 20905668 | C | G | 228 | Aoah | exonic | missense SNV | Aoah:NM_012054:exon6:c.C455G:p.P152R | . | 13qA2 | rs13481722 | 1 | 0 |
| chr13 | 20909935 | T | C | 170 | Aoah | intronic | . | . | . | 13qA2 | rs29596587 | 1 | 0 |
| chr13 | 20909938 | A | G | 170 | Aoah | intronic | . | . | . | 13qA2 | rs226283408 | 1 | 0 |
| chr13 | 20910096 | A | G | 228 | Aoah | exonic | missense SNV | Aoah:NM_012054:exon7:c.A550G:p.I184V | . | 13qA2 | rs29597526 | 1 | 0 |
| chr13 | 20910224 | T | C | 228 | Aoah | intronic | . | . | . | 13qA2 | rs48621369 | 1 | 0 |
| chr13 | 20910250 | A | T | 150 | Aoah | intronic | . | . | . | 13qA2 | rs50753859 | 1 | 0 |
| chr13 | 20911031 | G | A | 228 | Aoah | intronic | . | . | . | 13qA2 | rs29599384 | 1 | 0 |
| chr13 | 20911330 | A | G | 184 | Aoah | intronic | . | . | . | 13qA2 | rs47734429 | 1 | 0 |
| chr13 | 20911332 | G | A | 186 | Aoah | intronic | . | . | . | 13qA2 | rs29599387 | 1 | 0 |
| chr13 | 20911906 | C | T | 228 | Aoah | intronic | . | . | . | 13qA2 | rs29592477 | 1 | 0 |
| chr13 | 20979672 | A | T | 66 | Aoah | intronic | . | . | . | 13qA2 | . | 0 | 1 |
| chr13 | 20979739 | A | G | 101 | Aoah | intronic | . | . | . | 13qA2 | rs3664984 | 1 | 0 |
| chr13 | 20994844 | A | T | 132 | Aoah | intronic | . | . | . | 13qA2 | rs29532209 | 1 | 0 |
| chr13 | 20999720 | C | T | 115 | Aoah | intronic | . | . | . | 13qA2 | rs29602302 | 1 | 0 |
| chr13 | 20999943 | C | T | 228 | Aoah | exonic | synonymous SNV | Aoah:NM_012054:exon16:c.C1176T:p.Y392Y | . | 13qA2 | rs29603402 | 1 | 0 |
| chr13 | 20999967 | A | G | 228 | Aoah | exonic | synonymous SNV | Aoah:NM_012054:exon16:c.A1200G:p.T400T | . | 13qA2 | rs29604564 | 1 | 0 |
| chr13 | 21000066 | T | C | 228 | Aoah | exonic | synonymous SNV | Aoah:NM_012054:exon16:c.T1299C:p.P433P | . | 13qA2 | rs29604566 | 1 | 0 |
| chr13 | 21000095 | T | C | 228 | Aoah | intronic | . | . | . | 13qA2 | rs29604569 | 1 | 0 |
| chr13 | 21002570 | A | C | 228 | Aoah | intronic | . | . | . | 13qA2 | rs50785815 | 1 | 0 |
| chr13 | 21002652 | C | T | 228 | Aoah | splicing | . | . | . | 13qA2 | rs29602953 | 1 | 0 |
| chr13 | 21003092 | A | G | 163 | Aoah | intronic | . | . | . | 13qA2 | rs29233617 | 1 | 0 |
| chr13 | 21003116 | T | C | 113 | Aoah | intronic | . | . | . | 13qA2 | rs29538279 | 1 | 0 |
| chr13 | 21004835 | G | A | 152 | Aoah | intronic | . | . | . | 13qA2 | rs29530889 | 1 | 0 |
| chr13 | 21005012 | C | A | 228 | Aoah | intronic | . | . | . | 13qA2 | rs29600923 | 1 | 0 |
| chr13 | 21005093 | C | T | 228 | Aoah | exonic | synonymous SNV | Aoah:NM_012054:exon19:c.C1485T:p.F495F | . | 13qA2 | rs29601866 | 1 | 0 |
| chr13 | 21005186 | A | G | 173 | Aoah | intronic | . | . | . | 13qA2 | rs48030806 | 1 | 0 |
| chr13 | 21072872 | A | T | 221 | Olfr1370 | exonic | missense SNV | Olfr1370:NM_146535:exon1:c.T428A:p.L143H | . | 13qA2 | . | 0 | 1 |
| chr13 | 21073323 | T | C | 228 | . | upstream | . | . | . | 13qA2 | rs29602388 | 1 | 0 |
| chr13 | 21183718 | C | T | 228 | Trim27 | intronic | . | . | . | 13qA2 | rs29614216 | 1 | 0 |
| chr13 | 21191046 | A | G | 164 | Trim27 | intronic | . | . | . | 13qA2 | rs29492857 | 1 | 0 |
| chr13 | 21192184 | C | G | 228 | Trim27 | exonic | missense SNV | Trim27:NM_009054:exon8:c.C1101G:p.I367M | . | 13qA2 | rs13462756 | 1 | 0 |
| chr13 | 21192622 | G | A | 228 | Trim27 | exonic | synonymous SNV | Trim27:NM_009054:exon8:c.G1539A:p.P513P | . | 13qA2 | rs13462758 | 1 | 0 |
| chr13 | 21287149 | C | A | 101 | Gpx5 | UTR3 | . | . | . | 13qA3.1 | rs29246456 | 1 | 0 |
| chr13 | 21287455 | T | C | 228 | Gpx5 | exonic | missense SNV | Gpx5:NM_010343:exon5:c.A577G:p.I193V | . | 13qA3.1 | rs29571920 | 1 | 0 |
| chr13 | 21288956 | G | A | 228 | Gpx5 | intronic | . | . | . | 13qA3.1 | rs3023381 | 1 | 0 |
| chr13 | 21291191 | A | C | 170 | Gpx5 | intronic | . | . | . | 13qA3.1 | rs8266897 | 1 | 0 |
| chr13 | 21291450 | A | G | 228 | Gpx5 | exonic | synonymous SNV | Gpx5:NM_010343:exon2:c.T105C:p.D35D | . | 13qA3.1 | rs8266896 | 1 | 0 |
| chr13 | 21296200 | A | G | 153 | . | intergenic | . | . | . | 13qA3.1 | rs29644974 | 1 | 0 |
| chr13 | 21296316 | C | A | 228 | . | intergenic | . | . | . | 13qA3.1 | rs29644980 | 1 | 0 |
| chr13 | 21296717 | A | T | 228 | . | intergenic | . | . | . | 13qA3.1 | rs29646009 | 1 | 0 |
| chr13 | 21312194 | A | G | 228 | . | upstream | . | . | . | 13qA3.1 | rs50707431 | 1 | 0 |
| chr13 | 21312195 | C | G | 228 | . | upstream | . | . | . | 13qA3.1 | rs48693557 | 1 | 0 |
| chr13 | 21313715 | A | G | 228 | Gpx6 | exonic | missense SNV | Gpx6:NM_145451:exon2:c.A155G:p.E52G | . | 13qA3.1 | rs13471770 | 1 | 0 |
| chr13 | 21316820 | G | C | 138 | Gpx6 | intronic | . | . | . | 13qA3.1 | rs50120291 | 1 | 0 |
| chr13 | 21316832 | G | C | 147 | Gpx6 | intronic | . | . | . | 13qA3.1 | rs45684472 | 1 | 0 |
| chr13 | 21316848 | C | T | 164 | Gpx6 | intronic | . | . | . | 13qA3.1 | rs48560105 | 1 | 0 |
| chr13 | 21317302 | A | G | 160 | Gpx6 | intronic | . | . | . | 13qA3.1 | rs29646759 | 1 | 0 |
| chr13 | 21317548 | G | A | 228 | Gpx6 | intronic | . | . | . | 13qA3.1 | rs29646762 | 1 | 0 |
| chr13 | 21317562 | A | G | 228 | Gpx6 | intronic | . | . | . | 13qA3.1 | rs29647485 | 1 | 0 |
| chr13 | 21318855 | G | A | 228 | Gpx6 | intronic | . | . | . | 13qA3.1 | rs29252031 | 1 | 0 |
| chr13 | 21318943 | G | A | 228 | Gpx6 | exonic | missense SNV | Gpx6:NM_145451:exon5:c.G526A:p.V176I | . | 13qA3.1 | rs29640160 | 1 | 0 |
| chr13 | 21319100 | T | C | 228 | Gpx6 | UTR3 | . | . | . | 13qA3.1 | rs29493501 | 1 | 0 |
| chr13 | 21363814 | G | A | 228 | Zscan12 | exonic | synonymous SNV | Zscan12:NM_016684:exon2:c.G165A:p.Q55Q | . | 13qA3.1 | rs29553490 | 1 | 0 |
| chr13 | 21363850 | G | C | 228 | Zscan12 | exonic | synonymous SNV | Zscan12:NM_016684:exon2:c.G201C:p.L67L | . | 13qA3.1 | rs29248720 | 1 | 0 |
| chr13 | 21366554 | G | C | 228 | Zscan12 | intronic | . | . | . | 13qA3.1 | rs29232733 | 1 | 0 |
| chr13 | 21366761 | A | G | 228 | Zscan12 | intronic | . | . | . | 13qA3.1 | rs218449383 | 1 | 0 |
| chr13 | 21368541 | G | T | 228 | Zscan12 | intronic | . | . | . | 13qA3.1 | rs29240394 | 1 | 0 |
| chr13 | 21380927 | G | A | 228 | . | intergenic | . | . | . | 13qA3.1 | rs29642972 | 1 | 0 |
| chr13 | 21380978 | T | C | 228 | . | intergenic | . | . | . | 13qA3.1 | rs29640084 | 1 | 0 |
| chr13 | 21381061 | G | A | 228 | . | intergenic | . | . | . | 13qA3.1 | rs29549983 | 1 | 0 |
| chr13 | 21381091 | A | G | 228 | . | intergenic | . | . | . | 13qA3.1 | rs29513751 | 1 | 0 |
| chr13 | 21394573 | C | T | 228 | Zkscan3 | exonic | missense SNV | Zkscan3:NM_001145778:exon4:c.G574A:p.V192M,Zkscan3:NM_023685:exon4:c.G574A:p.V192M | . | 13qA3.1 | rs13481724 | 1 | 0 |
| chr13 | 21396181 | G | A | 83 | Zkscan3 | exonic | synonymous SNV | Zkscan3:NM_001145778:exon2:c.C336T:p.R112R,Zkscan3:NM_023685:exon2:c.C336T:p.R112R | . | 13qA3.1 | rs49262219 | 0 | 1 |
| chr13 | 21396379 | C | T | 228 | Zkscan3 | exonic | synonymous SNV | Zkscan3:NM_001145778:exon2:c.G138A:p.G46G,Zkscan3:NM_023685:exon2:c.G138A:p.G46G | . | 13qA3.1 | rs29571387 | 1 | 0 |
| chr13 | 21396423 | C | A | 228 | Zkscan3 | exonic | missense SNV | Zkscan3:NM_001145778:exon2:c.G94T:p.A32S,Zkscan3:NM_023685:exon2:c.G94T:p.A32S | . | 13qA3.1 | rs13463504 | 1 | 0 |
| chr13 | 21423407 | G | A | 228 | Pgbd1 | exonic | stopgain | Pgbd1:NM_001012311:exon4:c.C505T:p.Q169X,Pgbd1:NM_001162919:exon5:c.C616T:p.Q206X,Pgbd1:NM_0 | . | 13qA3.1 | rs29635983 | 1 | 0 |
| chr13 | 21423575 | C | T | 228 | Pgbd1 | exonic | missense SNV | Pgbd1:NM_001012311:exon4:c.G337A:p.G113R,Pgbd1:NM_001162919:exon5:c.G448A:p.G150R,Pgbd1:NM_0 | . | 13qA3.1 | rs45832095 | 1 | 0 |
| chr13 | 21423663 | T | A | 198 | Pgbd1 | intronic | . | . | . | 13qA3.1 | rs48698748 | 1 | 0 |
| chr13 | 21428046 | C | A | 43.9904 | Pgbd1 | intronic | . | . | . | 13qA3.1 | . | 0 | 1 |
| chr13 | 21433570 | A | C | 228 | Pgbd1 | intronic | . | . | . | 13qA3.1 | rs29566794 | 1 | 0 |
| chr13 | 21448302 | T | C | 228 | Zscan26 | intronic | . | . | . | 13qA3.1 | rs107856699 | 1 | 0 |
| chr13 | 21468217 | C | T | 228 | Nkap1 | exonic | synonymous SNV | Nkap1:NM_025719:exon1:c.G225A:p.P75P | 437 | 13qA3.1 | rs29225530 | 1 | 0 |
| chr13 | 21468301 | A | C | 228 | Nkap1 | exonic | synonymous SNV | Nkap1:NM_025719:exon1:c.T141G:p.G47G | 437 | 13qA3.1 | rs46727751 | 1 | 0 |
| chr13 | 21468303 | C | G | 228 | Nkap1 | exonic | missense SNV | Nkap1:NM_025719:exon1:c.G139C:p.G47R | 437 | 13qA3.1 | rs29909970 | 1 | 0 |
| chr13 | 21479134 | G | A | 228 | Zkscan4 | exonic | synonymous SNV | Zkscan4:NM_001039115:exon1:c.G42A:p.R14R | . | 13qA3.1 | rs29531749 | 1 | 0 |
| chr13 | 21479136 | C | T | 228 | Zkscan4 | exonic | missense SNV | Zkscan4:NM_001039115:exon1:c.C44T:p.S15F | . | 13qA3.1 | rs29995268 | 1 | 0 |
| chr13 | 21479284 | A | G | 183 | Zkscan4 | exonic | synonymous SNV | Zkscan4:NM_001039115:exon1:c.A192G:p.G64G | . | 13qA3.1 | rs29870449 | 1 | 0 |
| chr13 | 21479661 | A | T | 156 | Zkscan4 | intronic | . | . | . | 13qA3.1 | rs29238906 | 1 | 0 |
| chr13 | 21483991 | C | T | 228 | Zkscan4 | exonic | missense SNV | Zkscan4:NM_001039115:exon3:c.C698T:p.A233V | . | 13qA3.1 | rs29248966 | 1 | 0 |
| chr13 | 21484011 | T | C | 228 | Zkscan4 | exonic | synonymous SNV | Zkscan4:NM_001039115:exon3:c.T718C:p.L240L | . | 13qA3.1 | rs29928496 | 1 | 0 |
| chr13 | 21484838 | A | G | 228 | Zkscan4 | UTR3 | . | . | . | 13qA3.1 | rs29649787 | 1 | 0 |
| chr13 | 21496543 | G | A | 217 | . | intergenic | . | . | . | 13qA3.1 | . | 0 | 1 |
| chr13 | 21496657 | T | C | 228 | . | intergenic | . | . | . | 13qA3.1 | rs49285556 | 1 | 0 |
| chr13 | 21496769 | T | C | 165 | . | intergenic | . | . | . | 13qA3.1 | rs47113736 | 1 | 0 |
| chr13 | 21522197 | C | G | 228 | Zkscan8 | intronic | . | . | . | 13qA3.1 | rs29225094 | 1 | 0 |
| chr13 | 21537269 | A | T | 228 | Olfr1366 | exonic | synonymous SNV | Olfr1366:NM_146283:exon1:c.T735A:p.S245S | . | 13qA3.1 | rs48002367 | 1 | 0 |
| chr13 | 21537934 | G | C | 228 | Olfr1366 | exonic | missense SNV | Olfr1366:NM_146283:exon1:c.C70G:p.L24V | . | 13qA3.1 | rs29867622 | 1 | 0 |
| chr13 | 21555138 | C | G | 228 | Olfr1535 | exonic | missense SNV | Olfr1535:NM_207572:exon1:c.G883C:p.A295P | . | 13qA3.1 | rs29586811 | 1 | 0 |
| chr13 | 21555177 | T | C | 228 | Olfr1535 | exonic | missense SNV | Olfr1535:NM_207572:exon1:c.A844G:p.M282V | . | 13qA3.1 | rs29925096 | 1 | 0 |
| chr13 | 21555427 | C | A | 222 | Olfr1535 | exonic | missense SNV | Olfr1535:NM_207572:exon1:c.G594T:p.K198N | . | 13qA3.1 | . | 0 | 1 |
| chr13 | 21555798 | C | T | 228 | Olfr1535 | exonic | missense SNV | Olfr1535:NM_207572:exon1:c.G223A:p.A75T | . | 13qA3.1 | rs29250996 | 1 | 0 |
| chr13 | 21555859 | G | A | 228 | Olfr1535 | exonic | synonymous SNV | Olfr1535:NM_207572:exon1:c.C162T:p.H54H | . | 13qA3.1 | rs47046887 | 1 | 0 |
| chr13 | 21555961 | T | C | 228 | Olfr1535 | exonic | synonymous SNV | Olfr1535:NM_207572:exon1:c.A60G:p.R20R | . | 13qA3.1 | rs47783274 | 1 | 0 |
| chr13 | 21573747 | C | T | 228 | Olfr1364 | exonic | synonymous SNV | Olfr1364:NM_146540:exon1:c.G708A:p.S236S | . | 13qA3.1 | rs47233408 | 1 | 0 |
| chr13 | 21574439 | T | C | 228 | Olfr1364 | exonic | missense SNV | Olfr1364:NM_146540:exon1:c.A16G:p.I6V | . | 13qA3.1 | rs29229223 | 1 | 0 |
| chr13 | 21574528 | C | T | 133 | . | upstream | . | . | . | 13qA3.1 | . | 0 | 1 |
| chr13 | 21611200 | A | G | 228 | Olfr1362 | exonic | synonymous SNV | Olfr1362:NM_146744:exon1:c.T768C:p.I256I | . | 13qA3.1 | rs29249095 | 1 | 0 |
| chr13 | 21611427 | G | T | 228 | Olfr1362 | exonic | missense SNV | Olfr1362:NM_146744:exon1:c.C541A:p.L181M | . | 13qA3.1 | rs29636805 | 1 | 0 |
| chr13 | 21611431 | A | G | 228 | Olfr1362 | exonic | synonymous SNV | Olfr1362:NM_146744:exon1:c.T537C:p.C179C | . | 13qA3.1 | rs29550024 | 1 | 0 |
| chr13 | 21611469 | G | A | 228 | Olfr1362 | exonic | missense SNV | Olfr1362:NM_146744:exon1:c.C499T:p.P167S | . | 13qA3.1 | rs29240018 | 1 | 0 |
| chr13 | 21611635 | C | T | 228 | Olfr1362 | exonic | synonymous SNV | Olfr1362:NM_146744:exon1:c.G333A:p.E111E | . | 13qA3.1 | rs3699596 | 1 | 0 |
| chr13 | 21611674 | C | T | 228 | Olfr1362 | exonic | synonymous SNV | Olfr1362:NM_146744:exon1:c.G294A:p.V98V | . | 13qA3.1 | rs3700094 | 1 | 0 |
| chr13 | 21612040 | C | T | 228 | . | upstream | . | . | . | 13qA3.1 | rs48338171 | 1 | 0 |
| chr13 | 21638744 | A | G | 228 | Olfr11 | exonic | missense SNV | Olfr11:NM_146542:exon1:c.T778C:p.F260L | . | 13qA3.1 | rs29238491 | 1 | 0 |
| chr13 | 21659298 | C | T | 228 | Olfr1361 | exonic | synonymous SNV | Olfr1361:NM_146541:exon1:c.G24A:p.K8K | . | 13qA3.1 | rs29224558 | 1 | 0 |
| chr13 | 21717512 | T | A | 228 | . | upstream;downstrea | . | . | . | 13qA3.1 | rs29224337 | 1 | 0 |
| chr13 | 21754455 | C | G | 228 | Hist1h2bn | exonic | synonymous SNV | Hist1h2bn:NM_178201:exon1:c.C333G:p.A111A | 328 | 13qA3.1 | rs29809119 | 1 | 0 |
| chr13 | 21754476 | T | C | 228 | Hist1h2bn | exonic | synonymous SNV | Hist1h2bn:NM_178201:exon1:c.T354C:p.A118A | . | 13qA3.1 | rs29926197 | 1 | 0 |
| chr13 | 21787407 | T | A | 228 | . | upstream | . | . | 999 | 13qA3.1 | rs29859100 | 1 | 0 |
| chr13 | 22006991 | G | A | 221 | Prss16 | intronic | . | . | 460 | 13qA3.1 | rs16806831 | 0 | 1 |
| chr13 | 22035373 | C | G | 127 | Hist1h2ah | exonic | synonymous SNV | Hist1h2ah:NM_175659:exon1:c.G180C:p.T60T | 995 | 13qA3.1 | rs29784588 | 0 | 1 |
| chr13 | 22036650 | T | C | 79 | . | downstream | . | . | . | 13qA3.1 | rs51411174 | 0 | 1 |
| chr13 | 22040962 | T | C | 204 | Hist1h4i | UTR3 | . | . | . | 13qA3.1 | rs46798802 | 0 | 1 |
| chr13 | 22041137 | G | A | 131 | Hist1h4i | exonic | synonymous SNV | Hist1h4i:NM_175656:exon1:c.C187T:p.L63L | 444 | 13qA3.1 | . | 0 | 1 |
| chr13 | 22041270 | G | A | 222 | Hist1h4i | exonic | synonymous SNV | Hist1h4i:NM_175656:exon1:c.C54T:p.R18R | 444 | 13qA3.1 | rs29233899 | 0 | 1 |
| chr13 | 22042778 | G | C | 221 | Hist1h2ag | exonic | synonymous SNV | Hist1h2ag:NM_178186:exon1:c.C150G:p.V50V | 979 | 13qA3.1 | rs228319559 | 0 | 1 |
| chr13 | 22043286 | C | G | 222 | Hist1h2bj | exonic | synonymous SNV | Hist1h2bj:NM_178198:exon1:c.C57G:p.V19V | 979 | 13qA3.1 | rs52062387 | 0 | 1 |
| chr13 | 22043355 | C | G | 222 | Hist1h2bj | exonic | synonymous SNV | Hist1h2bj:NM_178198:exon1:c.C126G:p.V42V | 979 | 13qA3.1 | rs29516829 | 0 | 1 |
| chr13 | 22043583 | C | T | 221 | Hist1h2bj | exonic | synonymous SNV | Hist1h2bj:NM_178198:exon1:c.C354T:p.A118A | 979 | 13qA3.1 | rs30059312 | 0 | 1 |
| chr13 | 22083458 | A | G | 109 | . | intergenic | . | . | . | 13qA3.1 | rs52594477 | 0 | 1 |
| chr13 | 22083468 | C | T | 113 | . | intergenic | . | . | . | 13qA3.1 | rs52593635 | 0 | 1 |
| chr13 | 22083569 | A | G | 177 | . | intergenic | . | . | . | 13qA3.1 | rs47383366 | 0 | 1 |
| chr13 | 22083631 | T | C | 221 | . | intergenic | . | . | . | 13qA3.1 | rs49319784 | 0 | 1 |
| chr13 | 22083721 | T | C | 71 | . | intergenic | . | . | . | 13qA3.1 | rs47981640 | 0 | 1 |
| chr13 | 22164082 | C | G | 221 | . | intergenic | . | . | . | 13qA3.1 | rs13481727 | 0 | 1 |
| chr13 | 22164740 | T | G | 148 | . | intergenic | . | . | . | 13qA3.1 | . | 0 | 1 |
| chr13 | 22164976 | T | A | 155 | . | intergenic | . | . | . | 13qA3.1 | rs29703474 | 0 | 1 |
| chr13 | 22178533 | T | C | 79 | . | downstream | . | . | . | 13qA3.1 | rs29534954 | 0 | 1 |
| chr13 | 22219822 | G | T | 222 | . | upstream | . | . | . | 13qA3.1 | rs47934098 | 0 | 1 |
| chr13 | 22219951 | A | G | 112 | . | upstream | . | . | . | 13qA3.1 | rs29701548 | 0 | 1 |
| chr13 | 22244725 | T | C | 222 | Vmn1r194 | exonic | synonymous SNV | Vmn1r194:NM_001080972:exon1:c.T511C:p.L171L | . | 13qA3.1 | rs29243866 | 0 | 1 |
| chr13 | 22278382 | C | A | 222 | Vmn1r195 | exonic | synonymous SNV | Vmn1r195:NM_134223:exon1:c.C21A:p.I7I | . | 13qA3.1 | rs29699224 | 0 | 1 |
| chr13 | 22278588 | G | T | 221 | Vmn1r195 | exonic | missense SNV | Vmn1r195:NM_134223:exon1:c.G227T:p.G76V | . | 13qA3.1 | rs29699227 | 0 | 1 |
| chr13 | 22278710 | T | G | 222 | Vmn1r195 | exonic | missense SNV | Vmn1r195:NM_134223:exon1:c.T349G:p.F117V | . | 13qA3.1 | rs29550627 | 0 | 1 |
| chr13 | 22278861 | A | G | 222 | Vmn1r195 | exonic | missense SNV | Vmn1r195:NM_134223:exon1:c.A500G:p.E167G | . | 13qA3.1 | rs29250470 | 0 | 1 |
| chr13 | 22282414 | G | A | 222 | . | intergenic | . | . | . | 13qA3.1 | rs29513795 | 0 | 1 |
| chr13 | 22282470 | A | G | 222 | . | intergenic | . | . | . | 13qA3.1 | rs29496713 | 0 | 1 |
| chr13 | 22282822 | A | G | 222 | . | intergenic | . | . | . | 13qA3.1 | rs29571296 | 0 | 1 |
| chr13 | 22283412 | A | G | 200 | . | intergenic | . | . | . | 13qA3.1 | rs29226079 | 0 | 1 |
| chr13 | 22293297 | T | C | 222 | Vmn1r196 | exonic | synonymous SNV | Vmn1r196:NM_001167541:exon1:c.T105C:p.C35C | . | 13qA3.1 | rs29497058 | 0 | 1 |
| chr13 | 22293617 | T | C | 221 | Vmn1r196 | exonic | missense SNV | Vmn1r196:NM_001167541:exon1:c.T425C:p.M142T | . | 13qA3.1 | rs29702737 | 0 | 1 |
| chr13 | 22293883 | C | A | 222 | Vmn1r196 | exonic | missense SNV | Vmn1r196:NM_001167541:exon1:c.C691A:p.L231M | . | 13qA3.1 | rs29549040 | 0 | 1 |
| chr13 | 22354444 | T | A | 222 | Vmn1r198 | exonic | synonymous SNV | Vmn1r198:NM_134220:exon1:c.T99A:p.T33T | . | 13qA3.1 | . | 0 | 1 |
| chr13 | 22450141 | A | T | 221 | . | intergenic | . | . | . | 13qA3.1 | . | 0 | 1 |
| chr13 | 22556411 | A | G | 222 | Vmn1r204 | exonic | missense SNV | Vmn1r204:NM_001045544:exon1:c.A211G:p.S71G | . | 13qA3.1 | . | 0 | 1 |
| chr13 | 22883681 | T | C | 222 | Vmn1r212 | exonic | missense SNV | Vmn1r212:NM_134241:exon1:c.A481G:p.S161G | . | 13qA3.1 | . | 0 | 1 |
| chr13 | 23076508 | T | G | 221 | Vmn1r215 | exonic | synonymous SNV | Vmn1r215:NM_134219:exon1:c.T717G:p.L239L | . | 13qA3.1 | . | 0 | 1 |
| chr13 | 23099448 | C | A | 222 | Vmn1r216 | exonic | synonymous SNV | Vmn1r216:NM_134245:exon1:c.C300A:p.T100T | . | 13qA3.1 | . | 0 | 1 |
| chr13 | 23137455 | A | C | 222 | . | downstream | . | . | . | 13qA3.1 | rs29241727 | 0 | 1 |
| chr13 | 23163101 | T | C | 222 | Vmn1r219 | exonic | synonymous SNV | Vmn1r219:NM_134238:exon1:c.T459C:p.G153G | . | 13qA3.1 | . | 0 | 1 |
| chr13 | 23531625 | G | C | 59 | . | downstream | . | . | 689 | 13qA3.1 | . | 0 | 1 |
| chr13 | 23561078 | T | C | 122 | . | downstream | . | . | . | 13qA3.1 | rs265458141 | 0 | 1 |
| chr13 | 23561128 | C | T | 94 | . | downstream | . | . | . | 13qA3.1 | rs29788715 | 0 | 1 |
| chr13 | 23561144 | A | G | 42.727 | . | downstream | . | . | . | 13qA3.1 | rs47010390 | 0 | 1 |
| chr13 | 23561637 | T | C | 99 | . | downstream | . | . | . | 13qA3.1 | rs579289764 | 0 | 1 |
| chr13 | 23562026 | G | T | 221 | Hist1h3e | exonic | missense SNV | Hist1h3e:NM_178205:exon1:c.C340A:p.H114N | 606 | 13qA3.1 | . | 0 | 1 |
| chr13 | 23576526 | A | G | 222 | . | downstream | . | . | . | 13qA3.1 | rs48613662 | 0 | 1 |
| chr13 | 23683768 | G | C | 222 | Hist1h2ac | exonic | synonymous SNV | Hist1h2ac:NM_178189:exon1:c.C150G:p.V50V | 991 | 13qA3.1 | rs262770003 | 0 | 1 |
| chr13 | 23683954 | C | A | 222 | Hist1h2ac | UTR5 | . | . | 991 | 13qA3.1 | rs29794947 | 0 | 1 |
| chr13 | 23684288 | G | C | 222 | Hist1h2bc | exonic | synonymous SNV | Hist1h2bc:NM_001290380:exon1:c.G57C:p.V19V,Hist1h2bc:NM_023422:exon1:c.G57C:p.V19V | 991 | 13qA3.1 | rs29494151 | 0 | 1 |
| chr13 | 23684372 | G | A | 222 | Hist1h2bc | exonic | synonymous SNV | Hist1h2bc:NM_001290380:exon1:c.G141A:p.K47K,Hist1h2bc:NM_023422:exon1:c.G141A:p.K47K | 991 | 13qA3.1 | rs29565525 | 0 | 1 |
| chr13 | 23684390 | C | G | 222 | Hist1h2bc | exonic | synonymous SNV | Hist1h2bc:NM_001290380:exon1:c.C159G:p.T53T,Hist1h2bc:NM_023422:exon1:c.C159G:p.T53T | 991 | 13qA3.1 | rs30042716 | 0 | 1 |
| chr13 | 23684698 | G | C | 221 | Hist1h2bc | intronic | . | . | . | 13qA3.1 | rs29237917 | 0 | 1 |
| chr13 | 23696111 | T | C | 221 | Hist1h1t | exonic | synonymous SNV | Hist1h1t:NM_010377:exon1:c.T246C:p.R82R | . | 13qA3.1 | rs29804938 | 0 | 1 |
| chr13 | 23696285 | G | C | 221 | Hist1h1t | exonic | synonymous SNV | Hist1h1t:NM_010377:exon1:c.G420C:p.S140S | . | 13qA3.1 | rs3678815 | 0 | 1 |
| chr13 | 23696447 | A | G | 221 | Hist1h1t | exonic | synonymous SNV | Hist1h1t:NM_010377:exon1:c.A582G:p.K194K | . | 13qA3.1 | rs3679575 | 0 | 1 |
| chr13 | 23696795 | T | C | 222 | . | downstream | . | . | . | 13qA3.1 | rs29867406 | 0 | 1 |
| chr13 | 23697932 | G | T | 92 | . | downstream | . | . | . | 13qA3.1 | rs3717050 | 0 | 1 |
| chr13 | 23698378 | A | G | 176 | Hist1h4c | exonic | synonymous SNV | Hist1h4c:NM_178208:exon1:c.T72C:p.R24R | 285 | 13qA3.1 | rs29530495 | 0 | 1 |
| chr13 | 23698435 | A | G | 222 | Hist1h4c | exonic | synonymous SNV | Hist1h4c:NM_178208:exon1:c.T15C:p.G5G | . | 13qA3.1 | rs29511226 | 0 | 1 |
| chr13 | 23704193 | T | G | 78 | Hfe | UTR3 | . | . | . | 13qA3.1 | rs48106349 | 0 | 1 |
| chr13 | 23704259 | A | G | 129 | Hfe | UTR3 | . | . | . | 13qA3.1 | rs29796256 | 0 | 1 |

| | | | | | | | | | | | | | |
|---|---|---|---|---|---|---|---|---|---|---|---|---|---|
| chr13 | 23704509 | G | C | 222 | Hfe | intronic | . | . | . | 13qA3.1 | rs16795040 | 0 | 1 |
| chr13 | 23705464 | G | A | 85 | Hfe | intronic | . | . | . | 13qA3.1 | rs8267105 | 0 | 1 |
| chr13 | 23705667 | C | T | 222 | Hfe | exonic | synonymous SNV | Hfe:NM_010424:exon5:c.G996A:p.L332L | . | 13qA3.1 | rs8267100 | 0 | 1 |
| chr13 | 23705684 | C | T | 203 | Hfe | exonic | missense SNV | Hfe:NM_010424:exon5:c.G979A:p.V327I | . | 13qA3.1 | rs8267099 | 0 | 1 |
| chr13 | 23705691 | T | C | 156 | Hfe | exonic | synonymous SNV | Hfe:NM_010424:exon5:c.A972G:p.G324G | . | 13qA3.1 | rs8267098 | 0 | 1 |
| chr13 | 23705706 | G | A | 134 | Hfe | exonic | synonymous SNV | Hfe:NM_010424:exon5:c.C957T:p.I319I | . | 13qA3.1 | rs29926195 | 0 | 1 |
| chr13 | 23705904 | T | C | 214 | Hfe | exonic | synonymous SNV | Hfe:NM_010424:exon4:c.A885G:p.Q295Q | . | 13qA3.1 | . | 0 | 1 |
| chr13 | 23706069 | C | T | 222 | Hfe | exonic | synonymous SNV | Hfe:NM_010424:exon4:c.G720A:p.L240L | . | 13qA3.1 | rs8267032 | 0 | 1 |
| chr13 | 23707154 | C | T | 158 | Hfe | intronic | . | . | . | 13qA3.1 | rs262611023 | 0 | 1 |
| chr13 | 23707156 | A | G | 123 | Hfe | intronic | . | . | . | 13qA3.1 | rs8267086 | 0 | 1 |
| chr13 | 23728759 | A | G | 146 | . | intergenic | . | . | . | 13qA3.1 | rs6234938 | 0 | 1 |
| chr13 | 23729489 | C | T | 175 | . | intergenic | . | . | . | 13qA3.1 | rs29800779 | 1 | 0 |
| chr13 | 23729706 | T | C | 98 | . | intergenic | . | . | . | 13qA3.1 | rs48009157 | 0 | 1 |
| chr13 | 23729839 | G | A | 116 | . | intergenic | . | . | . | 13qA3.1 | rs51960563 | 0 | 1 |
| chr13 | 23738875 | C | T | 87 | Hist1h1c | exonic | synonymous SNV | Hist1h1c:NM_015786:exon1:c.C27T:p.P9P | 632 | 13qA3.1 | rs47949963 | 0 | 1 |
| chr13 | 23739148 | T | C | 221 | Hist1h1c | exonic | synonymous SNV | Hist1h1c:NM_015786:exon1:c.T300C:p.G100G | 632 | 13qA3.1 | rs13472732 | 0 | 1 |
| chr13 | 23739937 | G | A | 222 | . | downstream | . | . | . | 13qA3.1 | rs29550396 | 0 | 1 |
| chr13 | 23739978 | A | G | 222 | . | downstream | . | . | . | 13qA3.1 | rs29807133 | 0 | 1 |
| chr13 | 23740006 | C | T | 222 | . | downstream | . | . | . | 13qA3.1 | rs29515163 | 0 | 1 |
| chr13 | 23740086 | G | T | 216 | . | downstream | . | . | . | 13qA3.1 | . | 0 | 1 |
| chr13 | 23740239 | G | T | 222 | . | downstream | . | . | . | 13qA3.1 | rs29564394 | 0 | 1 |
| chr13 | 23745140 | T | C | 216 | Hist1h3c | exonic | missense SNV | Hist1h3c:NM_175653:exon1:c.A358G:p.I120V | 391 | 13qA3.1 | . | 0 | 1 |
| chr13 | 23745423 | G | A | 222 | Hist1h3c | exonic | synonymous SNV | Hist1h3c:NM_175653:exon1:c.C75T:p.A25A | 391 | 13qA3.1 | rs29725362 | 0 | 1 |
| chr13 | 23745426 | T | C | 222 | Hist1h3c | exonic | synonymous SNV | Hist1h3c:NM_175653:exon1:c.A72G:p.K24K | 391 | 13qA3.1 | rs29238313 | 0 | 1 |
| chr13 | 23746079 | T | C | 127 | . | upstream | . | . | . | 13qA3.1 | rs29556998 | 0 | 1 |
| chr13 | 23746908 | T | C | 222 | Hist1h2bb | exonic | synonymous SNV | Hist1h2bb:NM_175664:exon1:c.T114C:p.Y38Y | 268 | 13qA3.1 | rs29574070 | 0 | 1 |
| chr13 | 23747007 | C | T | 222 | Hist1h2bb | exonic | synonymous SNV | Hist1h2bb:NM_175664:exon1:c.C213T:p.F71F | 268 | 13qA3.1 | rs29929903 | 0 | 1 |
| chr13 | 23747028 | G | T | 222 | Hist1h2bb | exonic | synonymous SNV | Hist1h2bb:NM_175664:exon1:c.G234T:p.A78A | 268 | 13qA3.1 | rs29242433 | 0 | 1 |
| chr13 | 23751216 | A | G | 222 | Hist1h2ab | exonic | synonymous SNV | Hist1h2ab:NM_175660:exon1:c.A84G:p.V28V | 329 | 13qA3.1 | rs29963821 | 0 | 1 |
| chr13 | 23751228 | T | C | 213 | Hist1h2ab | exonic | synonymous SNV | Hist1h2ab:NM_175660:exon1:c.T96C:p.H32H | 329 | 13qA3.1 | rs29760794 | 0 | 1 |
| chr13 | 23751294 | C | T | 222 | Hist1h2ab | exonic | synonymous SNV | Hist1h2ab:NM_175660:exon1:c.C162T:p.A54A | 329 | 13qA3.1 | rs29252112 | 0 | 1 |
| chr13 | 23751339 | C | T | 220 | Hist1h2ab | exonic | synonymous SNV | Hist1h2ab:NM_175660:exon1:c.C207T:p.N69N | 329 | 13qA3.1 | rs29784377 | 0 | 1 |
| chr13 | 23752406 | C | T | 222 | Hist1h3b | exonic | synonymous SNV | Hist1h3b:NM_178203:exon1:c.C27T:p.R9R | 389 | 13qA3.1 | rs237663563 | 0 | 1 |
| chr13 | 23752418 | C | T | 222 | Hist1h3b | exonic | synonymous SNV | Hist1h3b:NM_178203:exon1:c.C39T:p.G13G | 389 | 13qA3.1 | rs255914766 | 0 | 1 |
| chr13 | 23752433 | C | T | 222 | Hist1h3b | exonic | synonymous SNV | Hist1h3b:NM_178203:exon1:c.C54T:p.R18R | 389 | 13qA3.1 | rs220106724 | 0 | 1 |
| chr13 | 23752646 | C | T | 193 | Hist1h3b | exonic | synonymous SNV | Hist1h3b:NM_178203:exon1:c.C267T:p.A89A | 389 | 13qA3.1 | rs29569832 | 0 | 1 |
| chr13 | 23752988 | G | C | 74 | . | downstream | . | . | . | 13qA3.1 | rs29803220 | 0 | 1 |
| chr13 | 23756485 | G | A | 222 | . | upstream | . | . | . | 13qA3.1 | rs29776213 | 0 | 1 |
| chr13 | 23756787 | C | T | 222 | . | upstream | . | . | . | 13qA3.1 | rs29587948 | 0 | 1 |
| chr13 | 23756951 | A | T | 222 | Hist1h4b | UTR5 | . | . | . | 13qA3.1 | rs29571337 | 0 | 1 |
| chr13 | 23757113 | C | G | 222 | Hist1h4b | exonic | synonymous SNV | Hist1h4b:NM_178193:exon1:c.C93G:p.T31T | 295 | 13qA3.1 | rs29877665 | 0 | 1 |
| chr13 | 23757146 | G | A | 222 | Hist1h4b | exonic | synonymous SNV | Hist1h4b:NM_178193:exon1:c.G126A:p.G42G | 295 | 13qA3.1 | rs46858096 | 0 | 1 |
| chr13 | 23757340 | A | G | 222 | Hist1h4b | UTR3 | . | . | . | 13qA3.1 | rs29235858 | 0 | 1 |
| chr13 | 23757445 | G | C | 188 | . | downstream | . | . | . | 13qA3.1 | rs30095839 | 0 | 1 |
| chr13 | 23760690 | T | C | 33.5001 | . | downstream | . | . | . | 13qA3.1 | . | 0 | 1 |
| chr13 | 23760760 | G | A | 147 | . | downstream | . | . | . | 13qA3.1 | rs29799803 | 0 | 1 |
| chr13 | 23761742 | A | C | 78 | . | upstream;downstrea | . | . | 1497 | 13qA3.1 | rs29801849 | 0 | 1 |
| chr13 | 23761832 | T | C | 155 | . | upstream;downstrea | . | . | 1497 | 13qA3.1 | rs29803178 | 0 | 1 |
| chr13 | 23762315 | G | A | 68 | Hist1h3a | exonic | synonymous SNV | Hist1h3a:NM_013550:exon1:c.C39T:p.G13G | 1497 | 13qA3.1 | rs47172452 | 0 | 1 |
| chr13 | 23762375 | T | G | 212 | Hist1h3a | UTR5 | . | . | . | 13qA3.1 | rs29804060 | 0 | 1 |
| chr13 | 23763713 | A | G | 222 | Hist1h1a | UTR5 | . | . | . | 13qA3.1 | rs29684453 | 0 | 1 |
| chr13 | 23782591 | C | T | 222 | Trim38 | exonic | missense SNV | Trim38:NM_001029935:exon1:c.C20T:p.T7M | . | 13qA3.1 | rs29798558 | 0 | 1 |
| chr13 | 23786038 | G | A | 195 | Trim38 | intronic | . | . | . | 13qA3.1 | rs29760759 | 0 | 1 |
| chr13 | 23788369 | G | A | 190 | Trim38 | exonic | synonymous SNV | Trim38:NM_001029935:exon3:c.G672A:p.T224T | . | 13qA3.1 | rs29806034 | 0 | 1 |
| chr13 | 23791130 | T | G | 222 | Trim38 | exonic | missense SNV | Trim38:NM_001029935:exon6:c.T1051G:p.Y351D | . | 13qA3.1 | . | 0 | 1 |
| chr13 | 23817668 | C | A | 182 | Slc17a2 | intronic | . | . | . | 13qA3.1 | . | 0 | 1 |
| chr13 | 23842509 | G | A | 222 | Slc17a3 | exonic | synonymous SNV | Slc17a3:NM_134069:exon2:c.G60A:p.T20T | . | 13qA3.1 | rs13475360 | 0 | 1 |
| chr13 | 23842550 | A | G | 222 | Slc17a3 | intronic | . | . | . | 13qA3.1 | rs29231730 | 0 | 1 |
| chr13 | 23842638 | T | G | 123 | Slc17a3 | intronic | . | . | . | 13qA3.1 | rs47145563 | 0 | 1 |
| chr13 | 23845440 | A | C | 222 | Slc17a3 | exonic | synonymous SNV | Slc17a3:NM_001164743:exon2:c.A87C:p.A29A,Slc17a3:NM_134069:exon3:c.A195C:p.A65A | . | 13qA3.1 | rs3719900 | 0 | 1 |
| chr13 | 23845557 | G | A | 222 | Slc17a3 | intronic | . | . | . | 13qA3.1 | rs3720584 | 0 | 1 |
| chr13 | 23845570 | T | C | 221 | Slc17a3 | intronic | . | . | . | 13qA3.1 | rs3720606 | 0 | 1 |
| chr13 | 23846100 | A | G | 132 | Slc17a3 | intronic | . | . | . | 13qA3.1 | rs30074223 | 0 | 1 |
| chr13 | 23846385 | G | A | 222 | Slc17a3 | exonic | missense SNV | Slc17a3:NM_001164743:exon3:c.G370A:p.A124T,Slc17a3:NM_134069:exon4:c.G478A:p.A160T | . | 13qA3.1 | rs29551960 | 0 | 1 |
| chr13 | 23846831 | T | C | 221 | Slc17a3 | intronic | . | . | . | 13qA3.1 | rs29249336 | 0 | 1 |
| chr13 | 23846985 | A | G | 221 | Slc17a3 | intronic | . | . | . | 13qA3.1 | rs29568704 | 0 | 1 |
| chr13 | 23852226 | G | A | 124 | Slc17a3 | intronic | . | . | . | 13qA3.1 | rs48687953 | 0 | 1 |
| chr13 | 23852305 | C | T | 222 | Slc17a3 | intronic | . | . | . | 13qA3.1 | rs47325922 | 0 | 1 |
| chr13 | 23852306 | A | G | 222 | Slc17a3 | intronic | . | . | . | 13qA3.1 | rs46196909 | 0 | 1 |
| chr13 | 23852485 | A | G | 222 | Slc17a3 | intronic | . | . | . | 13qA3.1 | rs29815980 | 0 | 1 |
| chr13 | 23856350 | G | A | 108 | Slc17a3 | intronic | . | . | . | 13qA3.1 | rs29909424 | 0 | 1 |
| chr13 | 23856356 | G | A | 100 | Slc17a3 | intronic | . | . | . | 13qA3.1 | rs49858942 | 0 | 1 |
| chr13 | 23856617 | G | A | 222 | Slc17a3 | intronic | . | . | . | 13qA3.1 | rs29225499 | 0 | 1 |
| chr13 | 23857092 | T | C | 222 | Slc17a3 | intronic | . | . | . | 13qA3.1 | rs29516565 | 0 | 1 |
| chr13 | 23857236 | A | G | 222 | Slc17a3 | intronic | . | . | . | 13qA3.1 | rs29896839 | 0 | 1 |
| chr13 | 23858259 | T | C | 108 | Slc17a3 | UTR3 | . | . | . | 13qA3.1 | rs29568400 | 0 | 1 |
| chr13 | 23875654 | C | A | 222 | Slc17a1 | exonic | missense SNV | Slc17a1:NM_001170638:exon4:c.C367A:p.L123I,Slc17a1:NM_009198:exon4:c.C367A:p.L123I | . | 13qA3.1 | rs13462956 | 0 | 1 |
| chr13 | 23933956 | A | T | 221 | Hist1h2ba | exonic | missense SNV | Hist1h2ba:NM_175663:exon1:c.T201A:p.F67L | . | 13qA3.1 | . | 0 | 1 |
| chr13 | 23959507 | A | G | 35.7175 | Scgn | intronic | . | . | . | 13qA3.1 | . | 0 | 1 |
| chr13 | 23959707 | A | G | 221 | Scgn | exonic | missense SNV | Scgn:NM_145399:exon10:c.T673C:p.F225L | . | 13qA3.1 | . | 0 | 1 |
| chr13 | 23978977 | C | T | 100 | Scgn | intronic | . | . | . | 13qA3.1 | rs219202462 | 0 | 1 |
| chr13 | 24041811 | C | T | 221 | Lrrc16a | intronic | . | . | . | 13qA3.1 | . | 0 | 1 |
| chr13 | 24164800 | C | T | 168 | Lrrc16a | splicing | . | . | . | 13qA3.1 | . | 0 | 1 |
| chr13 | 24320638 | G | A | 75 | . | intergenic | . | . | . | 13qA3.1 | . | 0 | 1 |
| chr13 | 24689319 | G | A | 85 | Fam65b | intronic | . | . | . | 13qA3.1 | . | 0 | 1 |
| chr13 | 24831425 | G | T | 199 | Acot13 | UTR5 | . | . | 793 | 13qA3.1 | . | 0 | 1 |
| chr13 | 24883734 | C | A | 181 | D130043K22Rik | intronic | . | . | . | 13qA3.1 | . | 0 | 1 |
| chr13 | 27202664 | A | C | 72 | Prl3c1 | intronic | . | . | . | 13qA3.1 | . | 0 | 1 |
| chr13 | 27351017 | G | T | 160 | Prl8a2 | exonic | stopgain | Prl8a2:NM_001289919:exon4:c.G271T:p.E91X,Prl8a2:NM_010088:exon4:c.G274T:p.E92X | . | 13qA3.1 | . | 0 | 1 |
| chr13 | 27434107 | A | G | 181 | Prl8a6 | intronic | . | . | . | 13qA3.1 | . | 0 | 1 |
| chr13 | 27558414 | C | T | 55 | Prl8a9 | intronic | . | . | . | 13qA3.1 | . | 0 | 1 |
| chr13 | 27610426 | G | T | 79 | Prl7b1 | intronic | . | . | . | 13qA3.1 | . | 0 | 1 |
| chr13 | 27635674 | G | T | 221 | Prl7a1 | intronic | . | . | . | 13qA3.1 | . | 0 | 1 |
| chr13 | 27714488 | G | A | 222 | Prl7d1 | exonic | missense SNV | Prl7d1:NM_011120:exon2:c.C40T:p.L14F | . | 13qA3.1 | . | 0 | 1 |
| chr13 | 30194622 | G | A | 221 | Mboat1 | exonic | synonymous SNV | Mboat1:NM_153546:exon2:c.G183A:p.E61E | . | 13qA3.2 | . | 0 | 1 |
| chr13 | 30817878 | G | A | 168 | Exoc2 | intronic | . | . | . | 13qA3.2 | rs29957831 | 0 | 1 |
| chr13 | 30820518 | A | G | 222 | Exoc2 | intronic | . | . | . | 13qA3.2 | rs3712759 | 0 | 1 |
| chr13 | 30820684 | A | G | 222 | Exoc2 | intronic | . | . | . | 13qA3.2 | . | 0 | 1 |
| chr13 | 30875179 | T | C | 163 | Exoc2 | intronic | . | . | . | 13qA3.2 | rs29909379 | 0 | 1 |
| chr13 | 30876643 | A | C | 199 | Exoc2 | intronic | . | . | . | 13qA3.2 | rs29955323 | 0 | 1 |
| chr13 | 30935282 | A | C | 32.2355 | Exoc2 | intronic | . | . | . | 13qA3.2 | . | 0 | 1 |
| chr13 | 30935287 | A | T | 28.9904 | Exoc2 | intronic | . | . | . | 13qA3.2 | . | 0 | 1 |
| chr13 | 30938371 | G | A | 165 | Exoc2 | intronic | . | . | . | 13qA3.2 | . | 0 | 1 |
| chr13 | 30975949 | C | T | 222 | . | intergenic | . | . | . | 13qA3.2 | rs29908841 | 0 | 1 |
| chr13 | 30976201 | G | A | 222 | . | intergenic | . | . | . | 13qA3.2 | rs29530787 | 0 | 1 |
| chr13 | 31884165 | A | C | 206 | Gmds | intronic | . | . | . | 13qA3.2 | rs29908717 | 0 | 1 |
| chr13 | 31885017 | A | T | 222 | Gmds | intronic | . | . | . | 13qA3.2 | rs29929601 | 0 | 1 |
| chr13 | 32100325 | C | A | 222 | Gmds | intronic | . | . | . | 13qA3.2 | rs29494333 | 0 | 1 |
| chr13 | 32100555 | T | C | 222 | Gmds | intronic | . | . | . | 13qA3.2 | rs29613643 | 0 | 1 |
| chr13 | 32126906 | T | G | 128 | Gmds | intronic | . | . | . | 13qA3.2 | rs46189992 | 0 | 1 |
| chr13 | 32126975 | C | T | 222 | Gmds | intronic | . | . | . | 13qA3.2 | rs51243204 | 0 | 1 |
| chr13 | 32127028 | A | G | 222 | Gmds | exonic | synonymous SNV | Gmds:NM_146041:exon6:c.T615C:p.I205I | . | 13qA3.2 | rs50233276 | 0 | 1 |
| chr13 | 32127076 | A | G | 222 | Gmds | exonic | synonymous SNV | Gmds:NM_146041:exon6:c.T567C:p.I189I | . | 13qA3.2 | rs29910532 | 0 | 1 |
| chr13 | 32127190 | C | T | 131 | Gmds | intronic | . | . | . | 13qA3.2 | rs50413031 | 0 | 1 |
| chr13 | 32127241 | C | G | 124 | Gmds | intronic | . | . | . | 13qA3.2 | rs49413221 | 0 | 1 |
| chr13 | 32127277 | C | A | 57 | Gmds | intronic | . | . | . | 13qA3.2 | rs46877571 | 0 | 1 |
| chr13 | 32127759 | T | C | 100 | Gmds | intronic | . | . | . | 13qA3.2 | rs108873198 | 0 | 1 |
| chr13 | 32127766 | G | A | 121 | Gmds | intronic | . | . | . | 13qA3.2 | rs107773454 | 0 | 1 |
| chr13 | 32127878 | C | T | 221 | Gmds | intronic | . | . | . | 13qA3.2 | rs260727319 | 0 | 1 |
| chr13 | 32128132 | A | C | 221 | Gmds | intronic | . | . | . | 13qA3.2 | rs29910529 | 0 | 1 |
| chr13 | 32128182 | G | C | 221 | Gmds | intronic | . | . | . | 13qA3.2 | rs47972755 | 0 | 1 |
| chr13 | 32224878 | T | C | 117 | Gmds | intronic | . | . | . | 13qA3.2 | rs29908664 | 0 | 1 |
| chr13 | 32234321 | C | G | 221 | Gmds | intronic | . | . | . | 13qA3.2 | rs29229306 | 0 | 1 |
| chr13 | 32724588 | C | T | 123 | Mylk4 | intronic | . | . | . | 13qA3.2 | rs29768269 | 0 | 1 |
| chr13 | 32968567 | G | A | 222 | Serpinb6b | exonic | missense SNV | Serpinb6b:NM_011454:exon2:c.G58A:p.G20S | . | 13qA3.2 | . | 0 | 1 |
| chr13 | 33006641 | G | A | 222 | Serpinb9 | exonic | synonymous SNV | Serpinb9:NM_009256:exon2:c.G99A:p.A33A | . | 13qA3.2 | rs13468085 | 0 | 1 |
| chr13 | 33029365 | T | C | 98 | Serpinb9b | intronic | . | . | . | 13qA3.2 | rs29895272 | 0 | 1 |
| chr13 | 33029374 | G | A | 98 | Serpinb9b | intronic | . | . | . | 13qA3.2 | rs29529992 | 0 | 1 |
| chr13 | 33029399 | C | A | 132 | Serpinb9b | intronic | . | . | . | 13qA3.2 | rs29547073 | 0 | 1 |
| chr13 | 33029486 | G | A | 222 | Serpinb9b | exonic | missense SNV | Serpinb9b:NM_011452:exon2:c.G25A:p.G9S | . | 13qA3.2 | rs29828974 | 0 | 1 |
| chr13 | 33033068 | A | T | 221 | Serpinb9b | intronic | . | . | . | 13qA3.2 | rs29633633 | 0 | 1 |
| chr13 | 33033110 | A | G | 221 | Serpinb9b | intronic | . | . | . | 13qA3.2 | rs29706599 | 0 | 1 |
| chr13 | 33033741 | C | T | 222 | Serpinb9b | intronic | . | . | . | 13qA3.2 | rs29638369 | 0 | 1 |
| chr13 | 33035377 | T | C | 132 | Serpinb9b | intronic | . | . | . | 13qA3.2 | rs29729267 | 0 | 1 |
| chr13 | 33035472 | C | T | 222 | Serpinb9b | intronic | . | . | . | 13qA3.2 | rs29900117 | 0 | 1 |
| chr13 | 33035686 | C | G | 222 | Serpinb9b | intronic | . | . | . | 13qA3.2 | rs29856714 | 0 | 1 |
| chr13 | 33038067 | T | C | 222 | Serpinb9b | exonic | synonymous SNV | Serpinb9b:NM_011452:exon6:c.T663C:p.Y221Y | . | 13qA3.2 | rs13481748 | 0 | 1 |
| chr13 | 33038118 | C | T | 222 | Serpinb9b | exonic | synonymous SNV | Serpinb9b:NM_011452:exon6:c.C714T:p.D238D | . | 13qA3.2 | rs29225264 | 0 | 1 |
| chr13 | 33040019 | C | T | 221 | Serpinb9b | UTR3 | . | . | . | 13qA3.2 | rs29515136 | 0 | 1 |
| chr13 | 33090283 | G | T | 118 | Serpinb1b | intronic | . | . | . | 13qA3.3 | rs30053869 | 0 | 1 |
| chr13 | 33091577 | C | T | 221 | Serpinb1b | intronic | . | . | . | 13qA3.3 | rs29932708 | 0 | 1 |
| chr13 | 33121191 | T | C | 221 | . | intergenic | . | . | . | 13qA3.3 | rs29241068 | 0 | 1 |
| chr13 | 33154194 | T | C | 43.9428 | Serpinb9c | intronic | . | . | . | 13qA3.3 | rs29494422 | 0 | 1 |
| chr13 | 33154374 | C | T | 222 | Serpinb9c | intronic | . | . | . | 13qA3.3 | rs29614199 | 0 | 1 |
| chr13 | 33154620 | C | A | 221 | Serpinb9c | intronic | . | . | . | 13qA3.3 | rs29570078 | 0 | 1 |
| chr13 | 33156252 | T | C | 176 | Serpinb9c | intronic | . | . | . | 13qA3.3 | rs29948996 | 0 | 1 |
| chr13 | 33156465 | G | A | 222 | Serpinb9c | exonic | missense SNV | Serpinb9c:NM_001164524:exon4:c.C352T:p.P118S,Serpinb9c:NM_011453:exon4:c.C268T:p.P90S | . | 13qA3.3 | rs29833021 | 0 | 1 |
| chr13 | 33156585 | G | A | 198 | Serpinb9c | intronic | . | . | . | 13qA3.3 | rs29676627 | 0 | 1 |
| chr13 | 33833253 | A | C | 221 | Serpinb6e | exonic | synonymous SNV | Serpinb6e:NM_001045535:exon7:c.T783G:p.T261T | . | 13qA3.3 | . | 0 | 1 |
| chr13 | 33922775 | G | A | 222 | Serpinb6a | intronic | . | . | . | 13qA3.3 | rs29759975 | 0 | 1 |
| chr13 | 33935751 | T | C | 102 | Serpinb6a | UTR5 | . | . | 296 | 13qA3.3 | rs29589161 | 0 | 1 |
| chr13 | 33982290 | A | G | 127 | Serpinb6a,Nqo2 | intronic | . | . | . | 13qA3.3 | rs29223931 | 0 | 1 |
| chr13 | 34028126 | C | T | 221 | Ripk1 | exonic | missense SNV | Ripk1:NM_009068:exon9:c.C1418T:p.T473I | . | 13qA3.3 | rs37678806 | 0 | 1 |
| chr13 | 34044185 | T | A | 32.818 | Bphl | intronic | . | . | . | 13qA3.3 | rs30003335 | 0 | 1 |
| chr13 | 34044216 | G | A | 92 | Bphl | intronic | . | . | . | 13qA3.3 | rs29237445 | 0 | 1 |
| chr13 | 34050147 | G | A | 98 | Bphl | intronic | . | . | . | 13qA3.3 | rs29680037 | 0 | 1 |
| chr13 | 34050175 | G | A | 129 | Bphl | intronic | . | . | . | 13qA3.3 | rs29975939 | 0 | 1 |
| chr13 | 34050337 | G | A | 222 | Bphl | exonic | synonymous SNV | Bphl:NM_026512:exon4:c.G435A:p.A145A | . | 13qA3.3 | rs13462066 | 0 | 1 |
| chr13 | 34050464 | T | C | 130 | Bphl | intronic | . | . | . | 13qA3.3 | rs29583768 | 0 | 1 |
| chr13 | 34050478 | A | G | 122 | Bphl | intronic | . | . | . | 13qA3.3 | rs29910113 | 0 | 1 |
| chr13 | 34060679 | G | C | 217 | Bphl | intronic | . | . | . | 13qA3.3 | rs29232160 | 0 | 1 |
| chr13 | 34060685 | G | A | 191 | Bphl | intronic | . | . | . | 13qA3.3 | rs29240605 | 0 | 1 |
| chr13 | 34060699 | G | A | 71 | Bphl | intronic | . | . | . | 13qA3.3 | rs29236098 | 0 | 1 |
| chr13 | 34064091 | A | G | 222 | Bphl | intronic | . | . | . | 13qA3.3 | rs29536784 | 0 | 1 |
| chr13 | 34064099 | C | A | 222 | Bphl | intronic | . | . | . | 13qA3.3 | rs29493434 | 0 | 1 |
| chr13 | 34073896 | T | C | 121 | Bphl | UTR3 | . | . | . | 13qA3.3 | rs29495974 | 0 | 1 |
| chr13 | 34074654 | C | T | 222 | Tubb2a | exonic | synonymous SNV | Tubb2a:NM_009450:exon4:c.G1152A:p.Q384Q | . | 13qA3.3 | rs13465251 | 0 | 1 |
| chr13 | 34076447 | A | T | 222 | Tubb2a | intronic | . | . | . | 13qA3.3 | rs30047033 | 0 | 1 |
| chr13 | 34076870 | G | C | 90 | Tubb2a | intronic | . | . | . | 13qA3.3 | rs29240956 | 0 | 1 |
| chr13 | 34078096 | T | G | 79 | . | upstream | . | . | 464 | 13qA3.3 | rs29230568 | 0 | 1 |
| chr13 | 34089013 | A | C | 222 | 4930447K03Rik | ncRNA_exonic | . | . | . | 13qA3.3 | rs29227551 | 0 | 1 |
| chr13 | 34089139 | T | G | 161 | 4930447K03Rik | ncRNA_intronic | . | . | . | 13qA3.3 | rs29712422 | 0 | 1 |
| chr13 | 34099141 | A | C | 201 | 4930447K03Rik | ncRNA_intronic | . | . | . | 13qA3.3 | rs52205848 | 0 | 1 |
| chr13 | 34127648 | A | G | 112 | Tubb2b | exonic | synonymous SNV | Tubb2b:NM_023716:exon4:c.T1161C:p.A387A | . | 13qA3.3 | rs29676938 | 0 | 1 |
| chr13 | 34192148 | G | T | 222 | Slc22a23 | intronic | . | . | . | 13qA3.3 | rs29232070 | 0 | 1 |
| chr13 | 34192283 | A | G | 222 | Slc22a23 | splicing | . | . | . | 13qA3.3 | rs36952145 | 0 | 1 |
| chr13 | 34193316 | A | G | 221 | Slc22a23 | intronic | . | . | . | 13qA3.3 | rs29495245 | 0 | 1 |
| chr13 | 34193346 | G | A | 221 | Slc22a23 | intronic | . | . | . | 13qA3.3 | rs29247199 | 0 | 1 |
| chr13 | 34195570 | A | G | 65 | Slc22a23 | intronic | . | . | . | 13qA3.3 | rs37553120 | 0 | 1 |
| chr13 | 34197610 | A | G | 93 | Slc22a23 | intronic | . | . | . | 13qA3.3 | rs37129787 | 0 | 1 |
| chr13 | 34197631 | A | G | 120 | Slc22a23 | intronic | . | . | . | 13qA3.3 | rs36913257 | 0 | 1 |
| chr13 | 34197765 | T | A | 221 | Slc22a23 | intronic | . | . | . | 13qA3.3 | rs47135371 | 0 | 1 |
| chr13 | 34197822 | A | G | 221 | Slc22a23 | exonic | synonymous SNV | Slc22a23:NM_001033167:exon6:c.T1278C:p.I426I | . | 13qA3.3 | rs36928607 | 0 | 1 |
| chr13 | 34197930 | A | G | 221 | Slc22a23 | intronic | . | . | . | 13qA3.3 | rs36391878 | 0 | 1 |
| chr13 | 34198051 | C | G | 123 | Slc22a23 | intronic | . | . | . | 13qA3.3 | rs37184832 | 0 | 1 |

| | | | | | | | | | | | | | |
|---|---|---|---|---|---|---|---|---|---|---|---|---|---|
| chr13 | 34204095 | G | C | 221 | Slc22a23 | intronic | . | . | . | 13qA3.3 | rs29757741 | 0 | 1 |
| chr13 | 34932219 | A | G | 79 | 4933417A18Rik | intronic | . | . | . | 13qA3.3 | rs29713123 | 0 | 1 |
| chr13 | 34940673 | A | C | 158 | 4933417A18Rik | intronic | . | . | . | 13qA3.3 | rs29495306 | 0 | 1 |
| chr13 | 34940696 | G | A | 192 | 4933417A18Rik | intronic | . | . | . | 13qA3.3 | rs29246785 | 0 | 1 |
| chr13 | 34940855 | A | G | 222 | 4933417A18Rik | intronic | . | . | . | 13qA3.3 | rs29223704 | 0 | 1 |
| chr13 | 34940867 | C | T | 222 | 4933417A18Rik | intronic | . | . | . | 13qA3.3 | rs29241644 | 0 | 1 |
| chr13 | 34940880 | T | C | 214 | 4933417A18Rik | intronic | . | . | . | 13qA3.3 | rs29250803 | 0 | 1 |
| chr13 | 34940959 | A | G | 221 | 4933417A18Rik | intronic | . | . | . | 13qA3.3 | rs38640576 | 0 | 1 |
| chr13 | 34941097 | T | C | 221 | 4933417A18Rik | intronic | . | . | . | 13qA3.3 | rs29226925 | 0 | 1 |
| chr13 | 34941292 | G | C | 222 | 4933417A18Rik | intronic | . | . | . | 13qA3.3 | rs29666533 | 0 | 1 |
| chr13 | 34941423 | T | C | 222 | 4933417A18Rik | intronic | . | . | . | 13qA3.3 | rs29856289 | 0 | 1 |
| chr13 | 34941865 | G | A | 222 | 4933417A18Rik | intronic | . | . | . | 13qA3.3 | rs29910106 | 0 | 1 |
| chr13 | 34941905 | G | A | 222 | 4933417A18Rik | intronic | . | . | . | 13qA3.3 | rs51880205 | 0 | 1 |
| chr13 | 34942003 | A | G | 142 | 4933417A18Rik | intronic | . | . | . | 13qA3.3 | rs30095470 | 0 | 1 |
| chr13 | 34947124 | C | T | 222 | Eci3,4933417A18Ri | intronic | . | . | . | 13qA3.3 | rs48003744 | 0 | 1 |
| chr13 | 34948533 | C | A | 142 | 4933417A18Rik,Eci | intronic | . | . | . | 13qA3.3 | rs29527452 | 0 | 1 |
| chr13 | 34948715 | A | G | 222 | Eci3,4933417A18Ri | intronic | . | . | . | 13qA3.3 | rs29589466 | 0 | 1 |
| chr13 | 34948716 | T | C | 222 | 4933417A18Rik,Eci | intronic | . | . | . | 13qA3.3 | rs29249042 | 0 | 1 |
| chr13 | 34951425 | T | C | 53 | 4933417A18Rik,Eci | intronic | . | . | . | 13qA3.3 | rs48483401 | 0 | 1 |
| chr13 | 34951533 | G | A | 188 | Eci3,4933417A18Ri | intronic | . | . | . | 13qA3.3 | rs49029303 | 0 | 1 |
| chr13 | 34951564 | G | C | 207 | 4933417A18Rik,Eci | intronic | . | . | . | 13qA3.3 | rs47809480 | 0 | 1 |
| chr13 | 34952761 | G | A | 128 | Eci3,4933417A18Ri | intronic | . | . | . | 13qA3.3 | rs29509164 | 0 | 1 |
| chr13 | 34956880 | T | C | 222 | Eci3 | exonic | synonymous SNV | Eci3:NM_026947:exon4:c.A318G:p.S106S | . | 13qA3.3 | rs38195829 | 0 | 1 |
| chr13 | 34959637 | C | T | 53 | Eci3 | intronic | . | . | . | 13qA3.3 | rs29659554 | 0 | 1 |
| chr13 | 34959845 | T | G | 222 | Eci3 | intronic | . | . | . | 13qA3.3 | rs29553271 | 0 | 1 |
| chr13 | 34959922 | T | C | 222 | Eci3 | exonic | synonymous SNV | Eci3:NM_026947:exon3:c.A219G:p.T73T | . | 13qA3.3 | rs29557937 | 0 | 1 |
| chr13 | 34960545 | T | G | 222 | Eci3 | intronic | . | . | . | 13qA3.3 | rs30091625 | 0 | 1 |
| chr13 | 34978871 | C | A | 222 | Eci2 | intronic | . | . | . | 13qA3.3 | rs29737074 | 0 | 1 |
| chr13 | 34990158 | T | C | 222 | Eci2 | intronic | . | . | . | 13qA3.3 | rs37018629 | 0 | 1 |
| chr13 | 34990235 | A | G | 222 | Eci2 | exonic | synonymous SNV | Eci2:NM_001110331:exon4:c.T441C:p.G147G,Eci2:NM_011868:exon4:c.T342C:p.G114G | . | 13qA3.3 | rs13464613 | 0 | 1 |
| chr13 | 34990272 | C | T | 222 | Eci2 | exonic | missense SNV | Eci2:NM_001110331:exon4:c.G404A:p.R135Q,Eci2:NM_011868:exon4:c.G305A:p.R102Q | . | 13qA3.3 | rs13464612 | 0 | 1 |
| chr13 | 34994203 | C | A | 112 | . | upstream | . | . | . | 13qA3.3 | . | 0 | 1 |
| chr13 | 35669835 | C | A | 222 | Cdyl | UTR5 | . | . | . | 13qA3.3 | . | 0 | 1 |
| chr13 | 35856892 | G | A | 221 | Cdyl | exonic | synonymous SNV | Cdyl:NM_001123386:exon3:c.G921A:p.S307S,Cdyl:NM_009881:exon6:c.G1068A:p.S356S | . | 13qA3.3 | rs29511720 | 0 | 1 |
| chr13 | 35858090 | G | A | 222 | Cdyl | exonic | synonymous SNV | Cdyl:NM_001123386:exon4:c.G963A:p.V321V,Cdyl:NM_009881:exon7:c.G1110A:p.V370V | . | 13qA3.3 | rs29633566 | 0 | 1 |
| chr13 | 35858204 | A | G | 222 | Cdyl | exonic | synonymous SNV | Cdyl:NM_001123386:exon4:c.A1077G:p.T359T,Cdyl:NM_009881:exon7:c.A1224G:p.T408T | . | 13qA3.3 | rs29250793 | 0 | 1 |
| chr13 | 35871577 | G | A | 222 | Cdyl | splicing | . | . | . | 13qA3.3 | . | 0 | 1 |
| chr13 | 35871858 | C | T | 112 | Cdyl | intronic | . | . | . | 13qA3.3 | rs29586335 | 0 | 1 |
| chr13 | 35872346 | G | A | 137 | Cdyl | intronic | . | . | . | 13qA3.3 | rs29570631 | 0 | 1 |
| chr13 | 35896597 | C | G | 87 | Rpp40 | UTR3 | . | . | . | 13qA3.3 | rs29638546 | 0 | 1 |
| chr13 | 35896630 | C | T | 139 | Rpp40 | UTR3 | . | . | . | 13qA3.3 | rs29248521 | 0 | 1 |
| chr13 | 35896650 | G | A | 168 | Rpp40 | UTR3 | . | . | . | 13qA3.3 | rs48995021 | 0 | 1 |
| chr13 | 35896664 | A | C | 148 | Rpp40 | UTR3 | . | . | . | 13qA3.3 | rs216505742 | 0 | 1 |
| chr13 | 35896796 | A | G | 222 | Rpp40 | exonic | synonymous SNV | Rpp40:NM_145938:exon8:c.T1023C:p.F341F | . | 13qA3.3 | rs45796477 | 0 | 1 |
| chr13 | 35896856 | G | A | 222 | Rpp40 | exonic | synonymous SNV | Rpp40:NM_145938:exon8:c.C963T:p.P321P | . | 13qA3.3 | rs51881984 | 0 | 1 |
| chr13 | 35901268 | T | C | 147 | Rpp40 | intronic | . | . | . | 13qA3.3 | rs47944878 | 0 | 1 |
| chr13 | 35901285 | C | T | 167 | Rpp40 | intronic | . | . | . | 13qA3.3 | rs47063298 | 0 | 1 |
| chr13 | 35901311 | G | T | 200 | Rpp40 | exonic | missense SNV | Rpp40:NM_145938:exon5:c.C554A:p.P185H | . | 13qA3.3 | rs47545937 | 0 | 1 |
| chr13 | 35901606 | T | A | 111 | Rpp40 | intronic | . | . | . | 13qA3.3 | rs48770924 | 0 | 1 |
| chr13 | 35901641 | T | C | 79 | Rpp40 | intronic | . | . | . | 13qA3.3 | rs49707531 | 0 | 1 |
| chr13 | 35901737 | T | A | 79 | Rpp40 | intronic | . | . | . | 13qA3.3 | rs30089049 | 0 | 1 |
| chr13 | 35901881 | A | G | 120 | Rpp40 | intronic | . | . | . | 13qA3.3 | rs51625640 | 0 | 1 |
| chr13 | 35901961 | G | A | 190 | Rpp40 | intronic | . | . | . | 13qA3.3 | rs48207853 | 0 | 1 |
| chr13 | 35902065 | G | A | 222 | Rpp40 | exonic | synonymous SNV | Rpp40:NM_145938:exon4:c.C381T:p.T127T | . | 13qA3.3 | rs51928072 | 0 | 1 |
| chr13 | 35902089 | T | C | 222 | Rpp40 | exonic | synonymous SNV | Rpp40:NM_145938:exon4:c.A357G:p.L119L | . | 13qA3.3 | rs46842573 | 0 | 1 |
| chr13 | 35902215 | A | G | 133 | Rpp40 | intronic | . | . | . | 13qA3.3 | rs49175446 | 0 | 1 |
| chr13 | 35903617 | C | A | 96 | Rpp40 | intronic | . | . | . | 13qA3.3 | rs578456077 | 0 | 1 |
| chr13 | 35903779 | T | C | 58 | Rpp40 | intronic | . | . | . | 13qA3.3 | rs51351780 | 0 | 1 |
| chr13 | 35904259 | C | T | 56 | Rpp40 | intronic | . | . | . | 13qA3.3 | rs47366513 | 0 | 1 |
| chr13 | 35904313 | T | C | 105 | Rpp40 | intronic | . | . | . | 13qA3.3 | rs50928275 | 0 | 1 |
| chr13 | 35904444 | C | T | 222 | Rpp40 | exonic | synonymous SNV | Rpp40:NM_145938:exon2:c.G243A:p.E81E | . | 13qA3.3 | rs52041415 | 0 | 1 |
| chr13 | 35904514 | C | T | 221 | Rpp40 | exonic | missense SNV | Rpp40:NM_145938:exon2:c.G173A:p.S58N | . | 13qA3.3 | rs48794703 | 0 | 1 |
| chr13 | 35904534 | A | C | 221 | Rpp40 | exonic | synonymous SNV | Rpp40:NM_145938:exon2:c.T153G:p.L51L | . | 13qA3.3 | rs50397510 | 0 | 1 |
| chr13 | 35904696 | T | C | 82 | Rpp40 | intronic | . | . | . | 13qA3.3 | rs47828441 | 0 | 1 |
| chr13 | 35906006 | T | C | 90 | Rpp40 | intronic | . | . | . | 13qA3.3 | rs45791728 | 0 | 1 |
| chr13 | 35906092 | G | A | 95 | Rpp40 | intronic | . | . | 229 | 13qA3.3 | rs45798680 | 0 | 1 |
| chr13 | 35906143 | A | G | 222 | Rpp40 | intronic | . | . | 229 | 13qA3.3 | rs46817745 | 0 | 1 |
| chr13 | 35906147 | G | A | 222 | Rpp40 | intronic | . | . | 229 | 13qA3.3 | rs47762001 | 0 | 1 |
| chr13 | 35906306 | C | T | 222 | Rpp40 | exonic | synonymous SNV | Rpp40:NM_145938:exon1:c.G21A:p.L7L | 229 | 13qA3.3 | rs50589664 | 0 | 1 |
| chr13 | 35906357 | C | T | 104 | . | upstream | . | . | . | 13qA3.3 | rs52238754 | 0 | 1 |
| chr13 | 35968739 | T | C | 221 | Ppp1r3g | exonic | synonymous SNV | Ppp1r3g:NM_029628:exon2:c.T141C:p.D47D | 1309 | 13qA3.3 | rs51615520 | 0 | 1 |
| chr13 | 35968755 | C | A | 221 | Ppp1r3g | exonic | synonymous SNV | Ppp1r3g:NM_029628:exon2:c.C157A:p.R53R | 1309 | 13qA3.3 | rs29506908 | 0 | 1 |
| chr13 | 35969127 | C | T | 93 | Ppp1r3g | exonic | missense SNV | Ppp1r3g:NM_029628:exon2:c.C529T:p.P177S | 1309 | 13qA3.3 | rs29548464 | 0 | 1 |
| chr13 | 35969135 | T | C | 76 | Ppp1r3g | exonic | synonymous SNV | Ppp1r3g:NM_029628:exon2:c.T537C:p.P179P | 1309 | 13qA3.3 | rs29509995 | 0 | 1 |
| chr13 | 35969282 | A | G | 130 | Ppp1r3g | exonic | synonymous SNV | Ppp1r3g:NM_029628:exon2:c.A684G:p.R228R | 1309 | 13qA3.3 | rs29234216 | 0 | 1 |
| chr13 | 35969294 | C | T | 144 | Ppp1r3g | exonic | synonymous SNV | Ppp1r3g:NM_029628:exon2:c.C696T:p.C232C | 1309 | 13qA3.3 | rs29230808 | 0 | 1 |
| chr13 | 35969363 | T | C | 129 | Ppp1r3g | exonic | synonymous SNV | Ppp1r3g:NM_029628:exon2:c.T765C:p.A255A | 1309 | 13qA3.3 | rs107928465 | 0 | 1 |
| chr13 | 35969382 | G | C | 160 | Ppp1r3g | exonic | missense SNV | Ppp1r3g:NM_029628:exon2:c.G784C:p.V262L | 1309 | 13qA3.3 | rs29723776 | 0 | 1 |
| chr13 | 35969450 | C | G | 222 | Ppp1r3g | exonic | synonymous SNV | Ppp1r3g:NM_029628:exon2:c.C852G:p.P284P | 1309 | 13qA3.3 | rs29782636 | 0 | 1 |
| chr13 | 35969681 | T | A | 221 | Ppp1r3g | UTR3 | . | . | . | 13qA3.3 | rs29871136 | 0 | 1 |
| chr13 | 35969810 | T | C | 222 | Ppp1r3g | UTR3 | . | . | . | 13qA3.3 | rs29685566 | 0 | 1 |
| chr13 | 35969838 | C | G | 222 | Ppp1r3g | UTR3 | . | . | . | 13qA3.3 | rs29533693 | 0 | 1 |
| chr13 | 35970173 | T | A | 222 | Ppp1r3g | UTR3 | . | . | . | 13qA3.3 | rs29584068 | 0 | 1 |
| chr13 | 35970195 | A | G | 222 | Ppp1r3g | UTR3 | . | . | . | 13qA3.3 | rs29243368 | 0 | 1 |
| chr13 | 35970390 | A | G | 222 | . | downstream | . | . | . | 13qA3.3 | rs29246312 | 0 | 1 |
| chr13 | 35970394 | G | T | 221 | . | downstream | . | . | . | 13qA3.3 | rs29531297 | 0 | 1 |
| chr13 | 35992537 | T | C | 47.4658 | Lyrm4 | intronic | . | . | . | 13qA3.3 | rs29527012 | 0 | 1 |
| chr13 | 35992828 | A | G | 221 | Lyrm4 | intronic | . | . | . | 13qA3.3 | rs29778856 | 0 | 1 |
| chr13 | 36204318 | T | A | 86 | Fars2 | intronic | . | . | . | 13qA3.3 | rs38560769 | 0 | 1 |
| chr13 | 36204932 | C | T | 210 | Fars2 | exonic | missense SNV | Fars2:NM_024274:exon2:c.C403T:p.H135Y | . | 13qA3.3 | . | 0 | 1 |
| chr13 | 36537340 | C | G | 222 | Fars2 | exonic | synonymous SNV | Fars2:NM_001039189:exon6:c.C561G:p.R187R,Fars2:NM_024274:exon7:c.C1257G:p.R419R | . | 13qA3.3 | rs29493388 | 0 | 1 |
| chr13 | 36877760 | G | T | 221 | F13a1 | exonic | synonymous SNV | F13a1:NM_001166391:exon14:c.C2028A:p.P676P,F13a1:NM_028784:exon14:c.C2028A:p.P676P | . | 13qA3.3 | . | 0 | 1 |
| chr13 | 36972100 | T | C | 221 | F13a1 | intronic | . | . | . | 13qA3.3 | rs6200652 | 0 | 1 |
| chr13 | 37930403 | A | G | 222 | Rreb1 | exonic | synonymous SNV | Rreb1:NM_001177869:exon9:c.A1737G:p.Q579Q,Rreb1:NM_001039188:exon10:c.A1737G:p.Q579Q,Rreb1:NM | . | 13qA3.3 | rs29496537 | 0 | 1 |
| chr13 | 37947744 | G | A | 103 | Rreb1 | intronic | . | . | . | 13qA3.3 | . | 0 | 1 |
| chr13 | 37982876 | T | A | 94 | Ssr1 | intronic | . | . | . | 13qA3.3 | rs6345744 | 0 | 1 |
| chr13 | 37985067 | T | C | 91 | Ssr1 | intronic | . | . | . | 13qA3.3 | rs29243418 | 0 | 1 |
| chr13 | 37985875 | T | C | 188 | Ssr1 | intronic | . | . | . | 13qA3.3 | rs29999513 | 0 | 1 |
| chr13 | 37985880 | A | T | 189 | Ssr1 | intronic | . | . | . | 13qA3.3 | rs29511058 | 0 | 1 |
| chr13 | 37987559 | T | C | 221 | Ssr1 | exonic | synonymous SNV | Ssr1:NM_025965:exon4:c.A501G:p.R167R | . | 13qA3.3 | rs13459140 | 0 | 1 |
| chr13 | 37989425 | C | T | 151 | Ssr1 | intronic | . | . | . | 13qA3.3 | rs29238126 | 0 | 1 |
| chr13 | 37989466 | G | A | 222 | Ssr1 | intronic | . | . | . | 13qA3.3 | rs50495950 | 0 | 1 |
| chr13 | 37992161 | T | A | 164 | Ssr1 | intronic | . | . | . | 13qA3.3 | rs51248909 | 0 | 1 |
| chr13 | 38005989 | A | G | 141 | . | downstream | . | . | . | 13qA3.3 | rs29514161 | 0 | 1 |
| chr13 | 38008174 | G | T | 115 | Cage1 | intronic | . | . | . | 13qA3.3 | rs29947635 | 0 | 1 |
| chr13 | 38012225 | G | T | 80 | Cage1 | intronic | . | . | . | 13qA3.3 | rs29513136 | 0 | 1 |
| chr13 | 38015561 | C | T | 129 | Cage1 | intronic | . | . | . | 13qA3.3 | rs29226397 | 0 | 1 |
| chr13 | 38016586 | G | A | 91 | Cage1 | intronic | . | . | . | 13qA3.3 | rs50610903 | 0 | 1 |
| chr13 | 38016604 | G | T | 92 | Cage1 | intronic | . | . | . | 13qA3.3 | rs49627035 | 0 | 1 |
| chr13 | 38022272 | C | T | 222 | Cage1 | intronic | . | . | . | 13qA3.3 | rs29494969 | 0 | 1 |
| chr13 | 38023049 | G | A | 221 | Cage1 | exonic | synonymous SNV | Cage1:NM_027724:exon5:c.C1137T:p.N379N | . | 13qA3.3 | rs29532147 | 0 | 1 |
| chr13 | 38023322 | C | T | 222 | Cage1 | exonic | synonymous SNV | Cage1:NM_027724:exon5:c.G864A:p.G288G | . | 13qA3.3 | rs49736271 | 0 | 1 |
| chr13 | 38023327 | T | C | 222 | Cage1 | exonic | missense SNV | Cage1:NM_027724:exon5:c.A859G:p.S287G | . | 13qA3.3 | rs47194029 | 0 | 1 |
| chr13 | 38023370 | G | A | 222 | Cage1 | exonic | synonymous SNV | Cage1:NM_027724:exon5:c.C816T:p.L272L | . | 13qA3.3 | rs36544769 | 0 | 1 |
| chr13 | 38023518 | G | A | 208 | Cage1 | intronic | . | . | . | 13qA3.3 | rs37594671 | 0 | 1 |
| chr13 | 38023546 | A | G | 80 | Cage1 | intronic | . | . | . | 13qA3.3 | rs48648978 | 0 | 1 |
| chr13 | 38023551 | C | A | 72 | Cage1 | intronic | . | . | . | 13qA3.3 | rs48463121 | 0 | 1 |
| chr13 | 38023613 | C | G | 45.987 | Cage1 | intronic | . | . | . | 13qA3.3 | rs36751727 | 0 | 1 |
| chr13 | 38025276 | A | G | 222 | Cage1 | intronic | . | . | . | 13qA3.3 | rs29229106 | 0 | 1 |
| chr13 | 38025436 | G | C | 222 | Cage1 | exonic | missense SNV | Cage1:NM_027724:exon4:c.C564G:p.F188L | . | 13qA3.3 | rs29225702 | 0 | 1 |
| chr13 | 38025454 | C | G | 222 | Cage1 | exonic | synonymous SNV | Cage1:NM_027724:exon4:c.G546C:p.P182P | . | 13qA3.3 | rs29736920 | 0 | 1 |
| chr13 | 38025487 | T | C | 222 | Cage1 | exonic | synonymous SNV | Cage1:NM_027724:exon4:c.A513G:p.L171L | . | 13qA3.3 | rs30060311 | 0 | 1 |
| chr13 | 38025616 | T | C | 222 | Cage1 | exonic | synonymous SNV | Cage1:NM_027724:exon4:c.A384G:p.V128V | . | 13qA3.3 | rs29726321 | 0 | 1 |
| chr13 | 38025622 | G | A | 222 | Cage1 | exonic | synonymous SNV | Cage1:NM_027724:exon4:c.C378T:p.S126S | . | 13qA3.3 | rs48646608 | 0 | 1 |
| chr13 | 38025749 | C | T | 222 | Cage1 | exonic | missense SNV | Cage1:NM_027724:exon4:c.G251A:p.R84K | . | 13qA3.3 | rs30030730 | 0 | 1 |
| chr13 | 38027884 | C | A | 35.4799 | Cage1 | intronic | . | . | . | 13qA3.3 | rs38723075 | 0 | 1 |
| chr13 | 38027996 | T | G | 167 | Cage1 | intronic | . | . | . | 13qA3.3 | rs39484963 | 0 | 1 |
| chr13 | 38028137 | C | T | 221 | Cage1 | exonic | synonymous SNV | Cage1:NM_027724:exon3:c.G162A:p.E54E | . | 13qA3.3 | rs37693159 | 0 | 1 |
| chr13 | 38028146 | C | T | 99 | Cage1 | exonic | synonymous SNV | Cage1:NM_027724:exon3:c.G153A:p.T51T | . | 13qA3.3 | rs36671401 | 0 | 1 |
| chr13 | 38028154 | T | C | 74 | Cage1 | exonic | missense SNV | Cage1:NM_027724:exon3:c.A145G:p.T49A | . | 13qA3.3 | rs46212189 | 0 | 1 |
| chr13 | 38028304 | C | A | 88 | Cage1 | intronic | . | . | . | 13qA3.3 | rs37422596 | 0 | 1 |
| chr13 | 38028329 | C | T | 23.7306 | Cage1 | intronic | . | . | . | 13qA3.3 | rs36280753 | 0 | 1 |
| chr13 | 38037396 | T | G | 222 | Riok1 | UTR5 | . | . | 711 | 13qA3.3 | rs29732669 | 0 | 1 |
| chr13 | 38037673 | A | G | 131 | Riok1 | intronic | . | . | . | 13qA3.3 | rs29570814 | 0 | 1 |
| chr13 | 38040002 | C | T | 132 | Riok1 | intronic | . | . | . | 13qA3.3 | rs36677618 | 0 | 1 |
| chr13 | 38040003 | G | A | 141 | Riok1 | intronic | . | . | . | 13qA3.3 | rs227172924 | 0 | 1 |
| chr13 | 38040086 | C | G | 222 | Riok1 | exonic | missense SNV | Riok1:NM_024242:exon2:c.C85G:p.L29V | . | 13qA3.3 | rs36417508 | 0 | 1 |
| chr13 | 38040133 | C | T | 222 | Riok1 | exonic | synonymous SNV | Riok1:NM_024242:exon2:c.C132T:p.L44L | . | 13qA3.3 | rs37432548 | 0 | 1 |
| chr13 | 38042908 | T | G | 97 | Riok1 | intronic | . | . | . | 13qA3.3 | rs36461275 | 0 | 1 |
| chr13 | 38042961 | A | C | 128 | Riok1 | intronic | . | . | . | 13qA3.3 | rs39001264 | 0 | 1 |
| chr13 | 38042969 | C | T | 127 | Riok1 | intronic | . | . | . | 13qA3.3 | rs50664120 | 0 | 1 |
| chr13 | 38043231 | T | C | 51 | Riok1 | intronic | . | . | . | 13qA3.3 | rs45764740 | 0 | 1 |
| chr13 | 38044018 | C | G | 219 | Riok1 | intronic | . | . | . | 13qA3.3 | rs49389295 | 0 | 1 |
| chr13 | 38044020 | C | T | 220 | Riok1 | intronic | . | . | . | 13qA3.3 | rs46232745 | 0 | 1 |
| chr13 | 38045206 | C | T | 222 | Riok1 | intronic | . | . | . | 13qA3.3 | rs37340266 | 0 | 1 |
| chr13 | 38045407 | C | T | 221 | Riok1 | intronic | . | . | . | 13qA3.3 | rs36936119 | 0 | 1 |
| chr13 | 38045452 | C | T | 129 | Riok1 | intronic | . | . | . | 13qA3.3 | rs50100630 | 0 | 1 |
| chr13 | 38045488 | G | A | 107 | Riok1 | intronic | . | . | . | 13qA3.3 | rs36614940 | 0 | 1 |
| chr13 | 38047161 | C | T | 125 | Riok1 | intronic | . | . | . | 13qA3.3 | rs29567979 | 0 | 1 |
| chr13 | 38047200 | G | A | 221 | Riok1 | intronic | . | . | . | 13qA3.3 | rs29527611 | 0 | 1 |
| chr13 | 38047428 | A | G | 222 | Riok1 | intronic | . | . | . | 13qA3.3 | rs29493006 | 0 | 1 |
| chr13 | 38049174 | A | C | 222 | Riok1 | intronic | . | . | . | 13qA3.3 | rs49484206 | 0 | 1 |
| chr13 | 38049228 | T | C | 222 | Riok1 | intronic | . | . | . | 13qA3.3 | rs49105543 | 0 | 1 |
| chr13 | 38049280 | A | C | 106 | Riok1 | intronic | . | . | . | 13qA3.3 | rs38150815 | 0 | 1 |
| chr13 | 38050163 | G | A | 221 | Riok1 | intronic | . | . | . | 13qA3.3 | rs37459095 | 0 | 1 |
| chr13 | 38050326 | A | G | 222 | Riok1 | intronic | . | . | . | 13qA3.3 | rs37810886 | 0 | 1 |
| chr13 | 38050656 | A | G | 101 | Riok1 | intronic | . | . | . | 13qA3.3 | rs29234418 | 0 | 1 |
| chr13 | 38050902 | G | T | 222 | Riok1 | exonic | missense SNV | Riok1:NM_024242:exon10:c.G946T:p.D316Y | . | 13qA3.3 | . | 0 | 1 |
| chr13 | 38050904 | C | T | 222 | Riok1 | exonic | synonymous SNV | Riok1:NM_024242:exon10:c.C948T:p.D316D | . | 13qA3.3 | rs30018482 | 0 | 1 |
| chr13 | 38051029 | T | C | 221 | Riok1 | intronic | . | . | . | 13qA3.3 | rs29547068 | 0 | 1 |
| chr13 | 38051076 | A | G | 191 | Riok1 | intronic | . | . | . | 13qA3.3 | rs29231686 | 0 | 1 |
| chr13 | 38051139 | T | C | 96 | Riok1 | intronic | . | . | . | 13qA3.3 | rs29948529 | 0 | 1 |
| chr13 | 38052219 | A | C | 130 | Riok1 | intronic | . | . | . | 13qA3.3 | rs29866019 | 0 | 1 |
| chr13 | 38052264 | T | G | 221 | Riok1 | intronic | . | . | . | 13qA3.3 | rs29528989 | 0 | 1 |
| chr13 | 38052724 | C | T | 105 | Riok1 | intronic | . | . | . | 13qA3.3 | rs36761659 | 0 | 1 |
| chr13 | 38052770 | C | T | 127 | Riok1 | intronic | . | . | . | 13qA3.3 | rs39618426 | 0 | 1 |
| chr13 | 38052991 | T | C | 222 | Riok1 | intronic | . | . | . | 13qA3.3 | rs36881594 | 0 | 1 |
| chr13 | 38053042 | A | G | 218 | Riok1 | intronic | . | . | . | 13qA3.3 | rs51921591 | 0 | 1 |
| chr13 | 38053074 | A | G | 127 | Riok1 | intronic | . | . | . | 13qA3.3 | rs36423535 | 0 | 1 |
| chr13 | 38055922 | T | C | 68 | Riok1 | intronic | . | . | . | 13qA3.3 | rs50259043 | 0 | 1 |
| chr13 | 38056272 | C | T | 222 | Riok1 | intronic | . | . | . | 13qA3.3 | rs36505315 | 0 | 1 |
| chr13 | 38057043 | A | G | 221 | Riok1 | intronic | . | . | . | 13qA3.3 | rs29638262 | 0 | 1 |
| chr13 | 38057048 | C | A | 221 | Riok1 | intronic | . | . | . | 13qA3.3 | rs29569038 | 0 | 1 |
| chr13 | 38058931 | C | T | 110 | Riok1 | intronic | . | . | . | 13qA3.3 | rs36911849 | 0 | 1 |
| chr13 | 38059875 | G | A | 169 | Riok1 | intronic | . | . | . | 13qA3.3 | rs36289856 | 0 | 1 |
| chr13 | 38060077 | T | C | 222 | Riok1 | exonic | synonymous SNV | Riok1:NM_024242:exon16:c.T1578C:p.P526P | . | 13qA3.3 | rs13460905 | 0 | 1 |
| chr13 | 38060081 | A | G | 222 | Riok1 | exonic | missense SNV | Riok1:NM_024242:exon16:c.A1582G:p.I528V | . | 13qA3.3 | rs36982157 | 0 | 1 |
| chr13 | 38060234 | T | C | 65 | Riok1 | intronic | . | . | . | 13qA3.3 | rs51545958 | 0 | 1 |
| chr13 | 38060241 | T | G | 57 | Riok1 | intronic | . | . | . | 13qA3.3 | rs46835283 | 0 | 1 |
| chr13 | 38060699 | C | T | 54 | Riok1 | intronic | . | . | . | 13qA3.3 | rs37785256 | 0 | 1 |
| chr13 | 38167370 | A | G | 129 | Dsp | intronic | . | . | . | 13qA3.3 | rs250037830 | 0 | 1 |
| chr13 | 38167488 | G | A | 221 | Dsp | intronic | . | . | . | 13qA3.3 | rs221828898 | 0 | 1 |
| chr13 | 38167804 | G | C | 167 | Dsp | intronic | . | . | . | 13qA3.3 | rs50239556 | 0 | 1 |
| chr13 | 38168672 | A | G | 221 | Dsp | exonic | synonymous SNV | Dsp:NM_023842:exon4:c.A525G:p.R175R | . | 13qA3.3 | rs29683495 | 0 | 1 |
| chr13 | 38168863 | G | A | 126 | Dsp | intronic | . | . | . | 13qA3.3 | rs29863868 | 0 | 1 |

| | | | | | | | | | | | | |
|---|---|---|---|---|---|---|---|---|---|---|---|---|
| chr13 | 38173573 | C | T | 112 | Dsp | intronic | . | . | . | 13qA3.3 | rs29554348 | 0 | 1 |
| chr13 | 38178846 | A | T | 123 | Dsp | intronic | . | . | . | 13qA3.3 | rs29921685 | 0 | 1 |
| chr13 | 38180940 | C | T | 128 | Dsp | intronic | . | . | . | 13qA3.3 | rs29514462 | 0 | 1 |
| chr13 | 38182174 | C | A | 222 | Dsp | intronic | . | . | . | 13qA3.3 | rs29242110 | 0 | 1 |
| chr13 | 38182958 | G | A | 112 | Dsp | intronic | . | . | . | 13qA3.3 | rs48220322 | 0 | 1 |
| chr13 | 38184211 | C | T | 201 | Dsp | intronic | . | . | . | 13qA3.3 | rs37854581 | 0 | 1 |
| chr13 | 38184245 | C | T | 159 | Dsp | intronic | . | . | . | 13qA3.3 | rs36704267 | 0 | 1 |
| chr13 | 38185058 | T | C | 222 | Dsp | intronic | . | . | . | 13qA3.3 | rs29851912 | 0 | 1 |
| chr13 | 38185654 | A | G | 222 | Dsp | exonic | synonymous SNV | Dsp:NM_023842:exon17:c.A2415G:p.E805E | . | 13qA3.3 | rs30072412 | 0 | 1 |
| chr13 | 38188587 | C | T | 222 | Dsp | intronic | . | . | . | 13qA3.3 | rs29623695 | 0 | 1 |
| chr13 | 38191736 | A | G | 222 | Dsp | exonic | missense SNV | Dsp:NM_023842:exon23:c.A3496G:p.S1166G | . | 13qA3.3 | rs29528874 | 0 | 1 |
| chr13 | 38192134 | G | A | 222 | Dsp | exonic | synonymous SNV | Dsp:NM_023842:exon23:c.G3894A:p.Q1298Q | . | 13qA3.3 | rs37648139 | 0 | 1 |
| chr13 | 38193004 | G | A | 222 | Dsp | exonic | synonymous SNV | Dsp:NM_023842:exon23:c.G4764A:p.E1588E | . | 13qA3.3 | rs29570429 | 0 | 1 |
| chr13 | 38193103 | C | T | 222 | Dsp | exonic | synonymous SNV | Dsp:NM_023842:exon23:c.C4863T:p.A1621A | . | 13qA3.3 | rs29532071 | 0 | 1 |
| chr13 | 38194929 | T | C | 221 | Dsp | exonic | synonymous SNV | Dsp:NM_023842:exon24:c.T5649C:p.Y1883Y | . | 13qA3.3 | rs30040329 | 0 | 1 |
| chr13 | 38195184 | A | G | 115 | Dsp | exonic | synonymous SNV | Dsp:NM_023842:exon24:c.A5904G:p.E1968E | . | 13qA3.3 | rs29225159 | 0 | 1 |
| chr13 | 38196576 | A | T | 222 | Dsp | exonic | synonymous SNV | Dsp:NM_023842:exon24:c.A7296T:p.T2432T | . | 13qA3.3 | rs29227733 | 0 | 1 |
| chr13 | 38196871 | T | C | 222 | Dsp | exonic | synonymous SNV | Dsp:NM_023842:exon24:c.T7591C:p.L2531L | . | 13qA3.3 | rs29553038 | 0 | 1 |
| chr13 | 38197164 | A | G | 222 | Dsp | exonic | synonymous SNV | Dsp:NM_023842:exon24:c.A7884G:p.S2628S | . | 13qA3.3 | rs38023879 | 0 | 1 |
| chr13 | 38197470 | T | G | 222 | Dsp | exonic | synonymous SNV | Dsp:NM_023842:exon24:c.T8190G:p.L2730L | . | 13qA3.3 | rs29495022 | 0 | 1 |
| chr13 | 38205194 | G | A | 79 | Snrnp48 | intronic | . | . | 575 | 13qA3.3 | rs29226777 | 0 | 1 |
| chr13 | 38210856 | T | C | 77 | Snrnp48 | intronic | . | . | . | 13qA3.3 | rs29233410 | 0 | 1 |
| chr13 | 38216310 | A | C | 176 | Snrnp48 | intronic | . | . | . | 13qA3.3 | rs29241434 | 0 | 1 |
| chr13 | 38220794 | T | C | 222 | Snrnp48 | intronic | . | . | . | 13qA3.3 | rs29714297 | 0 | 1 |
| chr13 | 38221037 | T | C | 128 | Snrnp48 | intronic | . | . | . | 13qA3.3 | rs29908003 | 0 | 1 |
| chr13 | 38612219 | C | T | 79 | Bloc1s5 | intronic | . | . | . | 13qA3.3 | rs52121187 | 0 | 1 |
| chr13 | 38656313 | A | T | 107 | Eef1e1 | intronic | . | . | . | 13qA3.3 | rs30027563 | 0 | 1 |
| chr13 | 38940020 | C | T | 220 | Slc35b3 | intronic | . | . | . | 13qA3.3 | . | 0 | 1 |
| chr13 | 40001737 | A | G | 186 | . | downstream | . | . | . | 13qA3.3 | rs29910770 | 1 | 0 |
| chr13 | 40002076 | A | G | 169 | Ofcc1 | intronic | . | . | . | 13qA3.3 | rs36648600 | 1 | 0 |
| chr13 | 40004052 | A | G | 228 | Ofcc1 | exonic | missense SNV | Ofcc1:NM_172143:exon18:c.T2669C:p.I890T | . | 13qA3.3 | rs36854285 | 1 | 0 |
| chr13 | 40015544 | A | G | 228 | Ofcc1 | intronic | . | . | . | 13qA3.3 | rs233450140 | 1 | 0 |
| chr13 | 40142790 | C | T | 228 | Ofcc1 | exonic | missense SNV | Ofcc1:NM_172143:exon12:c.G1567A:p.V523M | . | 13qA3.3 | rs29534538 | 1 | 0 |
| chr13 | 40180514 | G | T | 222 | Ofcc1 | exonic | missense SNV | Ofcc1:NM_172143:exon9:c.C1100A:p.P367H | . | 13qA3.3 | . | 0 | 1 |
| chr13 | 40206992 | A | G | 101 | Ofcc1 | intronic | . | . | . | 13qA3.3 | rs3655601 | 1 | 0 |
| chr13 | 40214610 | G | C | 228 | Ofcc1 | intronic | . | . | . | 13qA3.3 | rs48707441 | 1 | 0 |
| chr13 | 40214624 | G | A | 228 | Ofcc1 | intronic | . | . | . | 13qA3.3 | rs48911187 | 1 | 0 |
| chr13 | 40255353 | G | A | 164 | Ofcc1 | intronic | . | . | . | 13qA3.3 | rs52003830 | 1 | 0 |
| chr13 | 40255360 | A | G | 165 | Ofcc1 | intronic | . | . | . | 13qA3.3 | rs48492957 | 1 | 0 |
| chr13 | 40255495 | G | A | 228 | Ofcc1 | exonic | synonymous SNV | Ofcc1:NM_172143:exon5:c.C453T:p.D151D | . | 13qA3.3 | rs51720053 | 1 | 0 |
| chr13 | 40255511 | G | A | 228 | Ofcc1 | exonic | missense SNV | Ofcc1:NM_172143:exon5:c.C437T:p.P146L | . | 13qA3.3 | rs51182441 | 1 | 0 |
| chr13 | 40255682 | C | T | 228 | Ofcc1 | intronic | . | . | . | 13qA3.3 | rs50722583 | 1 | 0 |
| chr13 | 40255710 | A | G | 153 | Ofcc1 | intronic | . | . | . | 13qA3.3 | rs51089490 | 1 | 0 |
| chr13 | 40263428 | G | T | 191 | Ofcc1 | intronic | . | . | . | 13qA3.3 | rs29908730 | 1 | 0 |
| chr13 | 40263461 | C | T | 175 | Ofcc1 | intronic | . | . | . | 13qA3.3 | rs29587467 | 1 | 0 |
| chr13 | 40263825 | T | C | 126 | Ofcc1 | intronic | . | . | . | 13qA3.3 | rs29677737 | 1 | 0 |
| chr13 | 40279987 | G | A | 172 | Ofcc1 | intronic | . | . | . | 13qA3.3 | rs29725251 | 1 | 0 |
| chr13 | 40280169 | T | G | 228 | Ofcc1 | intronic | . | . | . | 13qA3.3 | rs29245362 | 1 | 0 |
| chr13 | 40280181 | G | A | 228 | Ofcc1 | intronic | . | . | . | 13qA3.3 | rs29249084 | 1 | 0 |
| chr13 | 40280532 | G | T | 228 | Ofcc1 | exonic | missense SNV | Ofcc1:NM_172143:exon3:c.C96A:p.D32E | . | 13qA3.3 | rs29496686 | 1 | 0 |
| chr13 | 40287839 | C | G | 130 | Ofcc1 | intronic | . | . | . | 13qA3.3 | . | 0 | 1 |
| chr13 | 40496782 | A | T | 228 | . | intergenic | . | . | . | 13qA3.3 | rs29779946 | 1 | 0 |
| chr13 | 40497009 | C | A | 228 | . | intergenic | . | . | . | 13qA3.3 | rs47099526 | 1 | 0 |
| chr13 | 40497128 | C | T | 228 | . | intergenic | . | . | . | 13qA3.3 | rs47389182 | 1 | 0 |
| chr13 | 40705864 | T | C | 228 | . | intergenic | . | . | . | 13qA3.3 | rs48468040 | 1 | 0 |
| chr13 | 40706458 | T | C | 228 | . | intergenic | . | . | . | 13qA3.3 | rs29223679 | 1 | 0 |
| chr13 | 40707172 | T | C | 228 | . | intergenic | . | . | . | 13qA3.3 | rs246230135 | 1 | 0 |
| chr13 | 40721291 | G | A | 228 | Tfap2a | exonic | synonymous SNV | Tfap2a:NM_001122948:exon5:c.C843T:p.T281T,Tfap2a:NM_011547:exon5:c.C861T:p.T287T,Tfap2a:NM_0 | . | 13qA3.3 | rs29241396 | 1 | 0 |
| chr13 | 40729893 | T | A | 191 | Gm26688,Tfap2a | ncRNA_intronic | . | . | . | 13qA3.3 | rs29914277 | 1 | 0 |
| chr13 | 40860764 | A | C | 228 | Gcnt2 | exonic | missense SNV | Gcnt2:NM_023887:exon1:c.A410C:p.E137A | . | 13qA3.3 | rs29231768 | 1 | 0 |
| chr13 | 40860852 | A | G | 228 | Gcnt2 | exonic | synonymous SNV | Gcnt2:NM_023887:exon1:c.A498G:p.Q166Q | . | 13qA3.3 | rs30077976 | 1 | 0 |
| chr13 | 40958356 | G | C | 228 | Gcnt2 | UTR3 | . | . | . | 13qA3.3 | rs29231478 | 1 | 0 |
| chr13 | 41102757 | C | T | 228 | Gcm2 | exonic | synonymous SNV | Gcm2:NM_008104:exon5:c.G1515A:p.X505X | . | 13qA3.3 | rs46332386 | 1 | 0 |
| chr13 | 41102959 | A | G | 228 | Gcm2 | exonic | missense SNV | Gcm2:NM_008104:exon5:c.T1313C:p.V438A | . | 13qA3.3 | rs49352972 | 1 | 0 |
| chr13 | 41103010 | A | T | 222 | Gcm2 | exonic | missense SNV | Gcm2:NM_008104:exon5:c.T1262A:p.M421K | . | 13qA3.3 | . | 0 | 1 |
| chr13 | 41103202 | A | G | 228 | Gcm2 | exonic | missense SNV | Gcm2:NM_008104:exon5:c.T1070C:p.L357S | . | 13qA3.3 | rs46560615 | 1 | 0 |
| chr13 | 41103382 | T | A | 228 | Gcm2 | exonic | missense SNV | Gcm2:NM_008104:exon5:c.A890T:p.E297V | . | 13qA3.3 | rs47635650 | 1 | 0 |
| chr13 | 41103663 | T | C | 228 | Gcm2 | exonic | synonymous SNV | Gcm2:NM_008104:exon5:c.A609G:p.G203G | . | 13qA3.3 | rs46207061 | 1 | 0 |
| chr13 | 41104556 | T | A | 228 | Gcm2 | intronic | . | . | . | 13qA3.3 | rs51787075 | 1 | 0 |
| chr13 | 41104579 | C | T | 228 | Gcm2 | exonic | missense SNV | Gcm2:NM_008104:exon4:c.G572A:p.G191E | . | 13qA3.3 | rs47338163 | 1 | 0 |
| chr13 | 41104729 | G | T | 228 | Gcm2 | intronic | . | . | . | 13qA3.3 | rs29911003 | 1 | 0 |
| chr13 | 41104743 | A | C | 222 | Gcm2 | intronic | . | . | . | 13qA3.3 | . | 0 | 1 |
| chr13 | 41104746 | G | A | 228 | Gcm2 | intronic | . | . | . | 13qA3.3 | rs29821307 | 1 | 0 |
| chr13 | 41105336 | T | A | 216 | Gcm2 | intronic | . | . | . | 13qA3.3 | rs46356971 | 1 | 0 |
| chr13 | 41105498 | T | C | 228 | Gcm2 | exonic | synonymous SNV | Gcm2:NM_008104:exon3:c.A369G:p.S123S | . | 13qA3.3 | rs49269737 | 1 | 0 |
| chr13 | 41105638 | T | C | 228 | Gcm2 | intronic | . | . | . | 13qA3.3 | rs49686586 | 1 | 0 |
| chr13 | 41105731 | A | G | 228 | Gcm2 | exonic | synonymous SNV | Gcm2:NM_008104:exon2:c.T261C:p.C87C | . | 13qA3.3 | rs47146760 | 1 | 0 |
| chr13 | 41105917 | A | G | 228 | Gcm2 | intronic | . | . | . | 13qA3.3 | rs49482553 | 1 | 0 |
| chr13 | 41105965 | G | A | 228 | Gcm2 | intronic | . | . | . | 13qA3.3 | rs50682365 | 1 | 0 |
| chr13 | 41110007 | T | C | 228 | . | upstream | . | . | . | 13qA3.3 | rs52207933 | 1 | 0 |
| chr13 | 41135161 | A | G | 228 | . | intergenic | . | . | . | 13qA3.3 | rs29568218 | 1 | 0 |
| chr13 | 41138803 | C | T | 228 | . | intergenic | . | . | . | 13qA3.3 | rs49673746 | 1 | 0 |
| chr13 | 41141569 | A | G | 175 | . | intergenic | . | . | . | 13qA3.3 | rs29572123 | 1 | 0 |
| chr13 | 41141770 | G | T | 221 | . | intergenic | . | . | . | 13qA3.3 | . | 0 | 1 |
| chr13 | 41143498 | A | G | 228 | . | intergenic | . | . | . | 13qA3.3 | rs48024104 | 1 | 0 |
| chr13 | 41145911 | G | T | 167 | . | intergenic | . | . | . | 13qA3.3 | rs29553956 | 1 | 0 |
| chr13 | 41828344 | G | C | 143 | Adtrp | exonic | synonymous SNV | Adtrp:NM_175417:exon2:c.C48G:p.L16L | . | 13qA4 | . | 0 | 1 |
| chr13 | 43168760 | C | A | 222 | Tbc1d7 | intronic | . | . | . | 13qA4 | . | 0 | 1 |
| chr13 | 43549439 | C | A | 211 | Mcur1 | intronic | . | . | . | 13qA4 | . | 0 | 1 |
| chr13 | 45078551 | C | G | 208 | . | intergenic | . | . | 568 | 13qA5 | . | 0 | 1 |
| chr13 | 45516917 | G | T | 141 | Gmpr | intronic | . | . | . | 13qA5 | . | 0 | 1 |
| chr13 | 45520866 | T | G | 222 | Gmpr | exonic | missense SNV | Gmpr:NM_025508:exon4:c.T306G:p.S102R | . | 13qA5 | . | 0 | 1 |
| chr13 | 46177522 | T | A | 221 | . | intergenic | . | . | . | 13qA5 | . | 0 | 1 |
| chr13 | 46285360 | C | A | 73 | Stmnd1 | intronic | . | . | . | 13qA5 | . | 0 | 1 |
| chr13 | 46285366 | G | C | 63 | Stmnd1 | intronic | . | . | . | 13qA5 | . | 0 | 1 |
| chr13 | 46684115 | A | C | 222 | Nup153 | exonic | synonymous SNV | Nup153:NM_175749:exon19:c.T3630G:p.P1210P | . | 13qA5 | . | 0 | 1 |
| chr13 | 46704525 | A | G | 122 | Nup153 | intronic | . | . | . | 13qA5 | . | 0 | 1 |
| chr13 | 47010817 | G | A | 198 | . | intergenic | . | . | . | 13qA5 | . | 0 | 1 |
| chr13 | 47014669 | G | C | 54 | Nhlrc1 | exonic | missense SNV | Nhlrc1:NM_175340:exon1:c.C111G:p.H37Q | 756 | 13qA5 | . | 0 | 1 |
| chr13 | 47073565 | G | T | 34.9773 | Kdm1b | intronic | . | . | . | 13qA5 | . | 0 | 1 |
| chr13 | 48312617 | A | T | 147 | . | intergenic | . | . | . | 13qA5 | . | 0 | 1 |
| chr13 | 48967395 | G | C | 208 | Fam120a | exonic | missense SNV | Fam120a:NM_001033268:exon1:c.C434G:p.A145G | 1278 | 13qA5 | . | 0 | 1 |
| chr13 | 49070977 | C | T | 221 | Wnk2 | exonic | missense SNV | Wnk2:NM_001290311:exon15:c.G3401A:p.R1134Q,Wnk2:NM_001290313:exon15:c.G3401A:p.R1134Q,Wnk2:NM | . | 13qA5 | . | 0 | 1 |
| chr13 | 49264926 | C | T | 103 | Fgd3 | intronic | . | . | . | 13qA5 | . | 0 | 1 |
| chr13 | 49732376 | A | T | 202 | Iars | exonic | missense SNV | Iars:NM_172015:exon33:c.A3590T:p.E1197V | . | 13qA5 | . | 0 | 1 |
| chr13 | 50950326 | A | G | 87 | . | intergenic | . | . | . | 13qA5 | . | 0 | 1 |
| chr13 | 50950808 | T | G | 221 | . | intergenic | . | . | . | 13qA5 | . | 0 | 1 |
| chr13 | 51149105 | A | T | 221 | Spin1 | exonic | missense SNV | Spin1:NM_011462:exon4:c.A667T:p.I223F,Spin1:NM_001283030:exon5:c.A658T:p.I220F,Spin1:NM_00128 | . | 13qA5 | . | 0 | 1 |
| chr13 | 51422029 | A | G | 221 | S1pr3 | UTR3 | . | . | . | 13qA5 | . | 0 | 1 |
| chr13 | 52507689 | C | G | 217 | Diras2 | exonic | missense SNV | Diras2:NM_001024474:exon2:c.G581C:p.G194A | . | 13qA5 | . | 0 | 1 |
| chr13 | 52624784 | A | C | 221 | Syk | intronic | . | . | . | 13qA5 | . | 0 | 1 |
| chr13 | 52640702 | G | C | 221 | Syk | exonic | missense SNV | Syk:NM_001198977:exon11:c.G1471C:p.A491P,Syk:NM_011518:exon11:c.G1471C:p.A491P | . | 13qA5 | . | 0 | 1 |
| chr13 | 52838072 | A | T | 222 | Auh | intronic | . | . | . | 13qB1 | . | 0 | 1 |
| chr13 | 53117204 | C | T | 228 | Ror2 | exonic | missense SNV | Ror2:NM_013846:exon7:c.G1114A:p.V372M | . | 13qB1 | rs46672326 | 1 | 0 |
| chr13 | 54214792 | C | T | 178 | Hrh2 | exonic | synonymous SNV | Hrh2:NM_001010973:exon2:c.C786T:p.D262D,Hrh2:NM_008286:exon2:c.C786T:p.D262D | . | 13qB1 | . | 0 | 1 |
| chr13 | 54537088 | A | C | 221 | Simc1 | exonic | missense SNV | Simc1:NM_176987:exon5:c.A3142C:p.T1048P | . | 13qB1 | . | 0 | 1 |
| chr13 | 54585249 | C | T | 222 | Nop16 | UTR3 | . | . | . | 13qB1 | . | 0 | 1 |
| chr13 | 55105590 | C | A | 91 | Zfp346 | intronic | . | . | 532 | 13qB1 | . | 0 | 1 |
| chr13 | 55412527 | C | T | 45.5477 | Slc34a1 | intronic | . | . | . | 13qB1 | . | 0 | 1 |
| chr13 | 55419990 | C | A | 138 | F12 | intronic | . | . | . | 13qB1 | . | 0 | 1 |
| chr13 | 55670214 | T | A | 222 | Ddx46 | exonic | missense SNV | Ddx46:NM_001282055:exon18:c.T2297A:p.I766K | . | 13qB1 | . | 0 | 1 |
| chr13 | 56449090 | G | A | 146 | Slc25a48 | intronic | . | . | . | 13qB1 | . | 0 | 1 |
| chr13 | 56625473 | A | T | 128 | Tgfbi | intronic | . | . | . | 13qB1 | . | 0 | 1 |
| chr13 | 56895578 | G | A | 221 | Trpc7 | UTR5 | . | . | . | 13qB1 | . | 0 | 1 |
| chr13 | 56895680 | G | A | 90 | Trpc7 | UTR5 | . | . | . | 13qB1 | . | 0 | 1 |
| chr13 | 56895681 | A | T | 90 | Trpc7 | UTR5 | . | . | . | 13qB1 | . | 0 | 1 |
| chr13 | 58328746 | C | T | 146 | Kif27 | intronic | . | . | . | 13qB1 | . | 0 | 1 |
| chr13 | 59718819 | T | G | 26.3722 | Spata31d1b | exonic | synonymous SNV | Spata31d1b:NM_001167593:exon4:c.T3780G:p.P1260P | . | 13qB2 | . | 0 | 1 |
| chr13 | 59755815 | C | T | 62 | Isca1 | UTR3 | . | . | . | 13qB2 | rs107904884 | 0 | 1 |
| chr13 | 60730664 | C | T | 54 | Dapk1 | intronic | . | . | . | 13qB2 | . | 0 | 1 |
| chr13 | 60771821 | C | T | 183 | . | intergenic | . | . | . | 13qB2 | . | 0 | 1 |
| chr13 | 60782599 | T | C | 222 | BC051665 | intronic | . | . | . | 13qB2 | . | 0 | 1 |
| chr13 | 60798558 | G | A | 222 | Ctsll3 | exonic | missense SNV | Ctsll3:NM_027344:exon8:c.C938T:p.A313V | . | 13qB2 | . | 0 | 1 |
| chr13 | 60853380 | T | C | 222 | 4930486L24Rik | intronic | . | . | . | 13qB2 | . | 0 | 1 |
| chr13 | 60935565 | C | A | 222 | Ctla2a | exonic | synonymous SNV | Ctla2a:NM_001145799:exon3:c.G192T:p.L64L,Ctla2a:NM_007796:exon3:c.G264T:p.L88L | . | 13qB2 | . | 0 | 1 |
| chr13 | 60935885 | T | G | 221 | Ctla2a | intronic | . | . | . | 13qB2 | . | 0 | 1 |
| chr13 | 60939904 | T | C | 221 | Tpbpa | intronic | . | . | . | 13qB2 | . | 0 | 1 |
| chr13 | 61035464 | A | G | 131 | Ctsq | intronic | . | . | . | 13qB2 | . | 0 | 1 |
| chr13 | 61202124 | T | G | 193 | Cts6 | intronic | . | . | . | 13qB2 | . | 0 | 1 |
| chr13 | 61354836 | A | G | 104 | Cts7 | intronic | . | . | . | 13qB2 | . | 0 | 1 |
| chr13 | 61356382 | A | G | 221 | Cts7 | exonic | missense SNV | Cts7:NM_019539:exon4:c.T283C:p.S95P | . | 13qB2 | . | 0 | 1 |
| chr13 | 61532242 | G | T | 214 | . | intergenic | . | . | . | 13qB2 | . | 0 | 1 |
| chr13 | 61666405 | G | T | 220 | . | intergenic | . | . | . | 13qB2 | . | 0 | 1 |
| chr13 | 63539151 | C | T | 199 | Ptch1 | intronic | . | . | . | 13qB3 | rs580947499 | 0 | 1 |
| chr13 | 63563879 | G | T | 59 | Ptch1 | intronic | . | . | 2942 | 13qB3 | . | 0 | 1 |
| chr13 | 63834651 | A | C | 221 | Ercc6l2 | exonic | missense SNV | Ercc6l2:NM_001013608:exon4:c.A644C:p.D215A,Ercc6l2:NM_023507:exon4:c.A644C:p.D215A | . | 13qB3 | . | 0 | 1 |
| chr13 | 64068337 | A | G | 221 | Hsd17b3 | intronic | . | . | . | 13qB3 | . | 0 | 1 |
| chr13 | 64432632 | G | T | 71 | Cdk20 | exonic | missense SNV | Cdk20:NM_053180:exon1:c.G37T:p.G13C | 535 | 13qB3 | . | 0 | 1 |
| chr13 | 64858670 | C | A | 157 | Cntnap3 | exonic | synonymous SNV | Cntnap3:NM_001081129:exon3:c.G327T:p.V109V | . | 13qB3 | . | 0 | 1 |
| chr13 | 64928750 | A | T | 222 | . | intergenic | . | . | . | 13qB3 | . | 0 | 1 |
| chr13 | 64928852 | C | T | 222 | . | intergenic | . | . | . | 13qB3 | . | 0 | 1 |
| chr13 | 65034528 | T | C | 222 | Spata31d1c | intronic | . | . | . | 13qB3 | . | 0 | 1 |
| chr13 | 65066682 | T | C | 221 | Hiatl1 | exonic | synonymous SNV | Hiatl1:NM_133680:exon12:c.A1455G:p.P485P,Hiatl1:NM_001083901:exon13:c.A1455G:p.P485P | . | 13qB3 | rs29494131 | 0 | 1 |
| chr13 | 65125899 | C | A | 145 | . | intergenic | . | . | . | 13qB3 | . | 0 | 1 |
| chr13 | 66007874 | T | G | 222 | . | intergenic | . | . | . | 13qB3 | . | 0 | 1 |
| chr13 | 67661390 | G | A | 92 | . | intergenic | . | . | . | 13qB3 | . | 0 | 1 |
| chr13 | 68668433 | A | C | 157 | Adcy2 | intronic | . | . | . | 13qB3 | . | 0 | 1 |
| chr13 | 68709953 | A | C | 222 | Adcy2 | intronic | . | . | . | 13qB3 | . | 0 | 1 |
| chr13 | 68732264 | C | A | 221 | Adcy2 | intronic | . | . | . | 13qB3 | . | 0 | 1 |
| chr13 | 68982360 | G | A | 215 | Adcy2 | exonic | missense SNV | Adcy2:NM_153534:exon2:c.C407T:p.S136F | . | 13qB3 | . | 0 | 1 |
| chr13 | 69633775 | A | G | 222 | Nsun2 | exonic | missense SNV | Nsun2:NM_145354:exon19:c.A2198G:p.K733R | . | 13qC1 | . | 0 | 1 |
| chr13 | 69665055 | T | A | 104 | . | intergenic | . | . | . | 13qC1 | . | 0 | 1 |
| chr13 | 70007386 | G | T | 222 | 1700084F23Rik | ncRNA_intronic | . | . | . | 13qC1 | . | 0 | 1 |
| chr13 | 70772094 | G | T | 221 | Adamts16 | exonic | missense SNV | Adamts16:NM_172053:exon13:c.C1906A:p.Q636K | . | 13qC1 | . | 0 | 1 |
| chr13 | 70791759 | A | C | 222 | Adamts16 | intronic | . | . | . | 13qC1 | . | 0 | 1 |
| chr13 | 70884067 | A | G | 221 | 8030423J24Rik | exonic | missense SNV | 8030423J24Rik:NM_029873:exon3:c.A257G:p.D86G | . | 13qC1 | . | 0 | 1 |
| chr13 | 71959050 | C | T | 221 | Irx1 | intronic | . | . | . | 13qC1 | . | 0 | 1 |
| chr13 | 71960110 | T | C | 216 | Irx1 | exonic | missense SNV | Irx1:NM_010573:exon2:c.A452G:p.K151R | 902 | 13qC1 | . | 0 | 1 |
| chr13 | 72350439 | C | A | 214 | . | intergenic | . | . | . | 13qC1 | . | 0 | 1 |
| chr13 | 72630674 | G | A | 222 | Irx2 | exonic | missense SNV | Irx2:NM_010574:exon2:c.G356A:p.R119H | 3674 | 13qC1 | . | 0 | 1 |
| chr13 | 72630777 | T | C | 228 | Irx2 | exonic | synonymous SNV | Irx2:NM_010574:exon2:c.T459C:p.I153I | 3674 | 13qC1 | rs29957076 | 1 | 0 |
| chr13 | 72631717 | G | A | 69 | Irx2 | exonic | synonymous SNV | Irx2:NM_010574:exon3:c.G1119A:p.S373S | 3674 | 13qC1 | . | 0 | 1 |
| chr13 | 73328436 | A | G | 228 | Ndufs6 | exonic | missense SNV | Ndufs6:NM_010888:exon1:c.T11C:p.V4A | 357 | 13qC1 | rs50934341 | 1 | 0 |
| chr13 | 73568121 | A | G | 205 | Slc6a3 | intronic | . | . | . | 13qC1 | . | 0 | 1 |
| chr13 | 73568238 | C | T | 221 | Slc6a3 | exonic | synonymous SNV | Slc6a3:NM_010020:exon11:c.C1449T:p.I483I | . | 13qC1 | . | 0 | 1 |
| chr13 | 73568288 | A | C | 221 | Slc6a3 | splicing | . | . | . | 13qC1 | . | 0 | 1 |
| chr13 | 73809871 | C | G | 169 | Slc12a7 | intronic | . | . | . | 13qC1 | . | 0 | 1 |
| chr13 | 74213907 | G | A | 150 | Ahrr | exonic | missense SNV | Ahrr:NM_001317351:exon10:c.C1472T:p.A491V,Ahrr:NM_009644:exon11:c.C1856T:p.A619V | . | 13qC1 | . | 0 | 1 |
| chr13 | 74356883 | A | G | 202 | Ccdc127 | exonic | synonymous SNV | Ccdc127:NM_001168659:exon3:c.A549G:p.R183R,Ccdc127:NM_024201:exon3:c.A549G:p.R183R | . | 13qC1 | . | 0 | 1 |
| chr13 | 74674469 | G | T | 114 | Erap1 | intronic | . | . | . | 13qC1 | . | 0 | 1 |
| chr13 | 74748892 | C | G | 222 | Cast | intronic | . | . | . | 13qC1 | . | 0 | 1 |
| chr13 | 74864745 | C | A | 174 | . | intergenic | . | . | . | 13qC1 | . | 0 | 1 |
| chr13 | 75515683 | C | T | 147 | . | intergenic | . | . | . | 13qC1 | . | 0 | 1 |
| chr13 | 76008262 | T | C | 199 | Rfesd | exonic | synonymous SNV | Rfesd:NM_001131068:exon2:c.A24G:p.Q8Q,Rfesd:NM_001131069:exon2:c.A24G:p.Q8Q,Rfesd:NM_178916: | . | 13qC1 | . | 0 | 1 |
| chr13 | 76759669 | A | C | 86 | Mctp1 | intronic | . | . | . | 13qC1 | . | 0 | 1 |

| | | | | | | | | | | | | | |
|---|---|---|---|---|---|---|---|---|---|---|---|---|---|
| chr13 | 81374425 | G | A | 104 | Adgrv1 | intronic | . | . | . | 13qC3 | . | 0 | 1 |
| chr13 | 81413095 | T | G | 222 | Adgrv1 | exonic | synonymous SNV | Adgrv1:NM_054053:exon76:c.A16299C:p.T5433T | . | 13qC3 | . | 0 | 1 |
| chr13 | 81439555 | T | G | 221 | Adgrv1 | exonic | missense SNV | Adgrv1:NM_054053:exon67:c.A13496C:p.K4499T | . | 13qC3 | . | 0 | 1 |
| chr13 | 81448977 | A | T | 179 | Adgrv1 | intronic | . | . | . | 13qC3 | . | 0 | 1 |
| chr13 | 81505986 | C | T | 222 | Adgrv1 | intronic | . | . | . | 13qC3 | . | 0 | 1 |
| chr13 | 81787082 | C | T | 92 | Cetn3 | intronic | . | . | . | 13qC3 | . | 0 | 1 |
| chr13 | 83735841 | A | G | 121 | . | downstream | . | . | . | 13qC3 | . | 0 | 1 |
| chr13 | 88977938 | A | C | 45.6129 | Edil3 | intronic | . | . | . | 13qC3 | . | 0 | 1 |
| chr13 | 89577873 | C | A | 214 | Hapln1 | intronic | . | . | . | 13qC3 | . | 0 | 1 |
| chr13 | 89678154 | A | T | 221 | Vcan | exonic | missense SNV | Vcan:NM_001134475:exon10:c.T1433A:p.V478E,Vcan:NM_001134474:exon11:c.T4313A:p.V1438E,Vcan:NM | . | 13qC3 | . | 0 | 1 |
| chr13 | 89691025 | C | A | 222 | Vcan | exonic | synonymous SNV | Vcan:NM_019389:exon7:c.G3519T:p.T1173T,Vcan:NM_001081249:exon8:c.G6399T:p.T2133T | . | 13qC3 | . | 0 | 1 |
| chr13 | 90923559 | G | A | 179 | Rps23 | intronic | . | . | 561 | 13qC3 | . | 0 | 1 |
| chr13 | 91564670 | C | A | 94 | Ssbp2 | exonic | missense SNV | Ssbp2:NM_024186:exon4:c.C258A:p.S86R,Ssbp2:NM_024272:exon4:c.C258A:p.S86R | . | 13qC3 | . | 0 | 1 |
| chr13 | 91770877 | T | G | 221 | Acot12 | exonic | synonymous SNV | Acot12:NM_028790:exon7:c.T690G:p.S230S | . | 13qC3 | . | 0 | 1 |
| chr13 | 91783109 | A | C | 222 | Acot12 | intronic | . | . | . | 13qC3 | . | 0 | 1 |
| chr13 | 92031041 | G | A | 130 | . | intergenic | . | . | . | 13qC3 | rs224294184 | 0 | 1 |
| chr13 | 92450385 | G | A | 107 | Fam151b | intronic | . | . | . | 13qC3 | . | 0 | 1 |
| chr13 | 92519690 | C | G | 28.9904 | Zfyve16 | intronic | . | . | . | 13qC3 | . | 0 | 1 |
| chr13 | 93063579 | C | G | 228 | Cmya5 | exonic | missense SNV | Cmya5:NM_023821:exon9:c.G10240C:p.A3414P | . | 13qC3 | rs46190260 | 1 | 0 |
| chr13 | 93387622 | A | T | 222 | Homer1 | exonic | missense SNV | Homer1:NM_001284189:exon6:c.A626T:p.K209M,Homer1:NM_152134:exon6:c.A674T:p.K225M,Homer1:NM_14 | . | 13qC3 | . | 0 | 1 |
| chr13 | 93627222 | G | A | 179 | Bhmt | intronic | . | . | . | 13qC3 | . | 0 | 1 |
| chr13 | 93708648 | T | C | 221 | Dmgdh,AW495222 | ncRNA_intronic | . | . | . | 13qC3 | . | 0 | 1 |
| chr13 | 95019124 | T | C | 87 | Wdr41 | intronic | . | . | . | 13qD1 | . | 0 | 1 |
| chr13 | 95049800 | T | C | 79 | Pde8b | intronic | . | . | . | 13qD1 | . | 0 | 1 |
| chr13 | 95353956 | A | G | 59 | Aggf1 | exonic | missense SNV | Aggf1:NM_025630:exon12:c.T1829C:p.V610A | . | 13qD1 | . | 0 | 1 |
| chr13 | 95721296 | C | T | 37.7165 | Iqgap2 | intronic | . | . | . | 13qD1 | . | 0 | 1 |
| chr13 | 95721566 | T | C | 222 | Iqgap2 | exonic | synonymous SNV | Iqgap2:NM_027711:exon11:c.A1179G:p.Q393Q | . | 13qD1 | . | 0 | 1 |
| chr13 | 95986060 | A | T | 214 | Sv2c | intronic | . | . | . | 13qD1 | . | 0 | 1 |
| chr13 | 96082383 | G | T | 222 | Sv2c | intronic | . | . | . | 13qD1 | . | 0 | 1 |
| chr13 | 97149331 | G | A | 222 | Gfm2 | exonic | synonymous SNV | Gfm2:NM_001146043:exon6:c.G327A:p.V109V,Gfm2:NM_001271463:exon6:c.G327A:p.V109V,Gfm2:NM_0012 | . | 13qD1 | . | 0 | 1 |
| chr13 | 97760524 | C | T | 221 | . | intergenic | . | . | . | 13qD1 | . | 0 | 1 |
| chr13 | 98305517 | C | T | 221 | . | intergenic | . | . | . | 13qD1 | . | 0 | 1 |
| chr13 | 98306966 | C | G | 221 | . | intergenic | . | . | . | 13qD1 | . | 0 | 1 |
| chr13 | 98310303 | T | G | 110 | Btf3 | intronic | . | . | . | 13qD1 | . | 0 | 1 |
| chr13 | 98355074 | C | T | 95 | Foxd1 | exonic | missense SNV | Foxd1:NM_008242:exon1:c.C455T:p.T152M | 1897 | 13qD1 | . | 0 | 1 |
| chr13 | 98356241 | C | T | 181 | Foxd1 | UTR3 | . | . | . | 13qD1 | . | 0 | 1 |
| chr13 | 98356467 | G | T | 123 | Foxd1 | UTR3 | . | . | . | 13qD1 | . | 0 | 1 |
| chr13 | 98402501 | C | A | 222 | . | intergenic | . | . | . | 13qD1 | . | 0 | 1 |
| chr13 | 98613338 | C | A | 167 | . | intergenic | . | . | 249 | 13qD1 | . | 0 | 1 |
| chr13 | 100055387 | C | G | 70 | Bdp1 | intronic | . | . | . | 13qD1 | . | 0 | 1 |
| chr13 | 100126919 | G | A | 222 | Smn1 | exonic | missense SNV | Smn1:NM_011420:exon3:c.G194A:p.G65D | . | 13qD1 | . | 0 | 1 |
| chr13 | 100152046 | A | G | 44.0638 | Naip2 | intronic | . | . | . | 13qD1 | . | 0 | 1 |
| chr13 | 100155200 | A | G | 196 | Naip2 | intronic | . | . | . | 13qD1 | rs3144873 | 0 | 1 |
| chr13 | 100415936 | G | A | 222 | Naip1 | intronic | . | . | . | 13qD1 | . | 0 | 1 |
| chr13 | 100432487 | A | G | 222 | Naip1 | exonic | missense SNV | Naip1:NM_008670:exon8:c.T935C:p.I312T | . | 13qD1 | . | 0 | 1 |
| chr13 | 100664134 | G | T | 156 | Ak6 | exonic | missense SNV | Ak6:NM_027592:exon4:c.G264T:p.W88C | . | 13qD1 | . | 0 | 1 |
| chr13 | 100717365 | C | T | 94 | Cdk7 | intronic | . | . | . | 13qD1 | . | 0 | 1 |
| chr13 | 100809265 | T | A | 220 | Slc30a5 | exonic | synonymous SNV | Slc30a5:NM_022885:exon13:c.A1578T:p.G526G | . | 13qD1 | . | 0 | 1 |
| chr13 | 100909786 | C | A | 221 | . | intergenic | . | . | . | 13qD1 | . | 0 | 1 |
| chr13 | 101349147 | C | G | 222 | . | intergenic | . | . | . | 13qD1 | . | 0 | 1 |
| chr13 | 102704981 | G | T | 221 | Cd180 | exonic | synonymous SNV | Cd180:NM_008533:exon3:c.G534T:p.L178L | . | 13qD1 | . | 0 | 1 |
| chr13 | 102769621 | C | A | 60 | Mast4 | intronic | . | . | . | 13qD1 | . | 0 | 1 |
| chr13 | 103758227 | T | A | 222 | Srek1 | exonic | synonymous SNV | Srek1:NM_172592:exon5:c.A621T:p.S207S | . | 13qD1 | . | 0 | 1 |
| chr13 | 104807787 | A | C | 222 | Cwc27 | exonic | missense SNV | Cwc27:NM_026072:exon3:c.T185G:p.I62R | . | 13qD1 | . | 0 | 1 |
| chr13 | 104967121 | T | C | 222 | Rgs7bp | exonic | synonymous SNV | Rgs7bp:NM_029879:exon4:c.A471G:p.E157E | . | 13qD1 | . | 0 | 1 |
| chr13 | 105249612 | T | C | 184 | Rnf180 | exonic | missense SNV | Rnf180:NM_027934:exon4:c.A1190G:p.Q397R | . | 13qD1 | . | 0 | 1 |
| chr13 | 106795581 | G | A | 133 | Ipo11 | UTR3 | . | . | . | 13qD2.1 | . | 0 | 1 |
| chr13 | 106833772 | G | A | 118 | Ipo11 | intronic | . | . | . | 13qD2.1 | rs49185152 | 0 | 1 |
| chr13 | 107414370 | A | C | 221 | . | intergenic | . | . | . | 13qD2.1 | . | 0 | 1 |
| chr13 | 108166535 | G | T | 221 | Ercc8 | intronic | . | . | . | 13qD2.1 | . | 0 | 1 |
| chr13 | 108310169 | C | A | 222 | . | intergenic | . | . | . | 13qD2.1 | . | 0 | 1 |
| chr13 | 109324681 | C | T | 96 | Pde4d | intronic | . | . | . | 13qD2.1 | . | 0 | 1 |
| chr13 | 109939166 | C | G | 169 | Pde4d | intronic | . | . | . | 13qD2.1 | . | 0 | 1 |
| chr13 | 109955552 | G | T | 148 | Pde4d | UTR3 | . | . | . | 13qD2.1 | . | 0 | 1 |
| chr13 | 110354151 | G | A | 221 | Gapt | UTR5 | . | . | . | 13qD2.1 | . | 0 | 1 |
| chr13 | 111593497 | T | A | 221 | . | intergenic | . | . | . | 13qD2.2 | . | 0 | 1 |
| chr13 | 112533761 | C | A | 222 | Il31ra | exonic | missense SNV | Il31ra:NM_139299:exon11:c.G1092T:p.Q364H | . | 13qD2.2 | . | 0 | 1 |
| chr13 | 112884818 | C | T | 31.9786 | Skiv2l2 | intronic | . | . | . | 13qD2.2 | . | 0 | 1 |
| chr13 | 112965179 | A | G | 201 | Dhx29 | intronic | . | . | . | 13qD2.2 | . | 0 | 1 |
| chr13 | 112994255 | C | A | 209 | Mcidas | intronic | . | . | 928 | 13qD2.2 | . | 0 | 1 |
| chr13 | 114611423 | G | A | 222 | 4930544M13Rik | ncRNA_exonic | . | . | . | 13qD2.2 | . | 0 | 1 |
| chr13 | 114965767 | C | T | 205 | Itga1 | intronic | . | . | . | 13qD2.2 | . | 0 | 1 |
| chr13 | 114975444 | G | T | 131 | Itga1 | intronic | . | . | . | 13qD2.2 | . | 0 | 1 |
| chr13 | 115007049 | C | A | 222 | Itga1 | exonic | missense SNV | Itga1:NM_001033228:exon9:c.G945T:p.R315S | . | 13qD2.2 | . | 0 | 1 |
| chr13 | 115030114 | G | C | 221 | Itga1 | exonic | missense SNV | Itga1:NM_001033228:exon6:c.C546G:p.S182R | . | 13qD2.2 | . | 0 | 1 |
| chr13 | 115101558 | A | C | 145 | Itga1 | UTR5 | . | . | . | 13qD2.2 | . | 0 | 1 |
| chr13 | 116303351 | C | T | 92 | Isl1 | intronic | . | . | . | 13qD2.2 | . | 0 | 1 |
| chr13 | 118384642 | C | T | 222 | Mrps30 | exonic | missense SNV | Mrps30:NM_021556:exon3:c.G760A:p.E254K | . | 13qD2.3 | . | 0 | 1 |
| chr13 | 119394874 | C | T | 222 | Nnt | intronic | . | . | . | 13qD2.3 | . | 0 | 1 |
| chr13 | 119404796 | A | C | 105 | Nnt | intronic | . | . | . | 13qD2.3 | . | 0 | 1 |
| chr13 | 119409056 | T | A | 120 | Nnt | UTR5 | . | . | 352 | 13qD2.3 | . | 0 | 1 |
| chr13 | 119440671 | G | T | 132 | Paip1 | intronic | . | . | . | 13qD2.3 | . | 0 | 1 |
| chr13 | 119457151 | G | T | 148 | Paip1 | UTR3 | . | . | . | 13qD2.3 | rs108777436 | 1 | 0 |
| chr13 | 119477802 | G | A | 221 | 4833420G17Rik | exonic | missense SNV | 4833420G17Rik:NM_001113550:exon9:c.G1445A:p.G482D,4833420G17Rik:NM_026127:exon10:c.G1445A:p.( | . | 13qD2.3 | . | 0 | 1 |
| chr13 | 119488435 | C | T | 121 | Gm7120 | intronic | . | . | 1149 | 13qD2.3 | rs241964556 | 0 | 1 |
| chr14 | 7756873 | G | A | 69 | . | intergenic | . | . | . | 14qA1 | . | 0 | 1 |
| chr14 | 8172871 | C | G | 228 | Pdhb | intronic | . | . | 681 | 14qA1 | . | 1 | 0 |
| chr14 | 8518558 | C | A | 90 | 4930452B06Rik | intronic | . | . | . | 14qA1 | . | 0 | 1 |
| chr14 | 12037616 | C | T | 89 | Ptprg | intronic | . | . | . | 14qA1 | . | 0 | 1 |
| chr14 | 12142611 | G | T | 162 | Ptprg | intronic | . | . | . | 14qA1 | . | 0 | 1 |
| chr14 | 12219893 | G | T | 129 | Ptprg | intronic | . | . | . | 14qA1 | . | 0 | 1 |
| chr14 | 12237136 | T | C | 228 | Ptprg | exonic | missense SNV | Ptprg:NM_008981:exon29:c.T4154C:p.L1385P | . | 14qA1 | . | 1 | 0 |
| chr14 | 12517718 | T | C | 228 | Cadps | intronic | . | . | . | 14qA1 | . | 1 | 0 |
| chr14 | 12597366 | T | G | 190 | Cadps | splicing | . | . | . | 14qA1 | . | 0 | 1 |
| chr14 | 13608901 | C | T | 44.8751 | Synpr | intronic | . | . | . | 14qA1 | . | 0 | 1 |
| chr14 | 13954487 | T | A | 216 | Thoc7 | intronic | . | . | . | 14qA1 | . | 0 | 1 |
| chr14 | 14198456 | G | A | 189 | . | intergenic | . | . | . | 14qA1 | . | 0 | 1 |
| chr14 | 14773295 | T | C | 222 | Slc4a7 | exonic | missense SNV | Slc4a7:NM_001033270:exon17:c.T2264C:p.I755T | . | 14qA1 | . | 0 | 1 |
| chr14 | 16818876 | C | T | 204 | Rarb | UTR5 | . | . | . | 14qA2 | . | 0 | 1 |
| chr14 | 18100041 | T | G | 187 | . | intergenic | . | . | . | 14qA2 | . | 0 | 1 |
| chr14 | 19797106 | A | C | 178 | Nid2 | intronic | . | . | . | 14qA3 | . | 0 | 1 |
| chr14 | 20141957 | C | G | 228 | Kcnk5 | exonic | missense SNV | Kcnk5:NM_021542:exon5:c.G1135C:p.G379R | . | 14qA3 | . | 1 | 0 |
| chr14 | 20855930 | C | G | 222 | . | intergenic | . | . | . | 14qA3 | . | 0 | 1 |
| chr14 | 20995117 | A | G | 228 | Vcl | intronic | . | . | . | 14qA3 | rs108073956 | 1 | 0 |
| chr14 | 23039740 | G | A | 228 | 1700112E06Rik,Gml | ncRNA_exonic | . | . | . | 14qA3 | . | 1 | 0 |
| chr14 | 23337027 | T | C | 222 | Kcnma1 | exonic | synonymous SNV | Kcnma1:NM_001253360:exon23:c.A2760G:p.S920S,Kcnma1:NM_001253367:exon23:c.A2760G:p.S920S,Kcnma | . | 14qA3 | rs212708808 | 0 | 1 |
| chr14 | 23462906 | T | C | 83 | Kcnma1 | intronic | . | . | . | 14qA3 | . | 0 | 1 |
| chr14 | 24157841 | G | C | 167 | Dlg5 | exonic | missense SNV | Dlg5:NM_027726:exon14:c.C3227G:p.T1076S,Dlg5:NM_001163513:exon15:c.C3296G:p.T1099S | . | 14qA3 | . | 0 | 1 |
| chr14 | 24474397 | G | T | 216 | Polr3a | intronic | . | . | . | 14qA3 | . | 1 | 0 |
| chr14 | 24474398 | G | T | 225 | Polr3a | intronic | . | . | . | 14qA3 | . | 1 | 0 |
| chr14 | 26592183 | T | G | 228 | Dennd6a | intronic | . | . | . | 14qA3 | . | 1 | 0 |
| chr14 | 26723887 | G | C | 228 | . | intergenic | . | . | . | 14qA3 | . | 1 | 0 |
| chr14 | 27211461 | C | T | 40.901 | Arhgef3 | intronic | . | . | . | 14qA3 | . | 0 | 1 |
| chr14 | 27551568 | T | A | 95 | . | intergenic | . | . | . | 14qA3 | . | 0 | 1 |
| chr14 | 29217622 | C | T | 222 | Cacna2d3 | intronic | . | . | . | 14qA3 | . | 0 | 1 |
| chr14 | 29396413 | A | T | 222 | Cacna2d3 | intronic | . | . | . | 14qA3 | . | 0 | 1 |
| chr14 | 30000482 | C | A | 204 | Il17rb | intronic | . | . | . | 14qB | . | 0 | 1 |
| chr14 | 30075160 | T | A | 222 | Cacna1d | exonic | stopgain | Cacna1d:NM_001083616:exon35:c.A4414T:p.R1472X,Cacna1d:NM_028981:exon35:c.A4480T:p.R1494X,Cacn | . | 14qB | . | 0 | 1 |
| chr14 | 30096863 | G | T | 204 | Cacna1d | intronic | . | . | . | 14qB | . | 1 | 0 |
| chr14 | 30196850 | T | C | 222 | Cacna1d | exonic | missense SNV | Cacna1d:NM_001083616:exon4:c.A497G:p.Y166C,Cacna1d:NM_001302637:exon4:c.A497G:p.Y166C,Cacna1d | . | 14qB | . | 0 | 1 |
| chr14 | 30765989 | T | C | 222 | Sfmbt1 | UTR5 | . | . | . | 14qB | . | 0 | 1 |
| chr14 | 31025374 | G | A | 44.2097 | Pbrm1 | intronic | . | . | . | 14qB | . | 0 | 1 |
| chr14 | 31268945 | G | C | 222 | Dnah1 | intronic | . | . | . | 14qB | . | 0 | 1 |
| chr14 | 31479869 | T | C | 228 | Mettl6 | intronic | . | . | . | 14qB | . | 1 | 0 |
| chr14 | 31506533 | G | C | 222 | Eaf1 | intronic | . | . | . | 14qB | . | 0 | 1 |
| chr14 | 31545070 | G | A | 222 | Colq | exonic | missense SNV | Colq:NM_009937:exon7:c.C512T:p.P171L | . | 14qB | . | 0 | 1 |
| chr14 | 31622831 | C | A | 228 | Hacl1 | intronic | . | . | . | 14qB | . | 1 | 0 |
| chr14 | 32056094 | G | T | 98 | Galnt15 | intronic | . | . | . | 14qB | . | 0 | 1 |
| chr14 | 32660026 | G | A | 222 | 3425401B19Rik | exonic | synonymous SNV | 3425401B19Rik:NM_001195097:exon4:c.C3981T:p.S1327S | . | 14qB | . | 0 | 1 |
| chr14 | 33012877 | T | C | 222 | Lrrc18,Wdfy4 | intronic | . | . | . | 14qB | . | 0 | 1 |
| chr14 | 33018610 | T | G | 222 | Wdfy4 | intronic | . | . | . | 14qB | . | 0 | 1 |
| chr14 | 33639327 | T | G | 228 | Ptpn20 | UTR3 | . | . | . | 14qB | . | 1 | 0 |
| chr14 | 33932117 | C | T | 140 | Gdf10 | exonic | missense SNV | Gdf10:NM_145741:exon2:c.C580T:p.R194C | . | 14qB | . | 0 | 1 |
| chr14 | 33955005 | T | A | 228 | Rbp3 | exonic | synonymous SNV | Rbp3:NM_015745:exon1:c.T909A:p.P303P | . | 14qB | . | 1 | 0 |
| chr14 | 34093480 | A | C | 228 | Anxa8,A630023A22R | intronic | . | . | . | 14qB | . | 1 | 0 |
| chr14 | 34594782 | G | T | 228 | Opn4 | exonic | missense SNV | Opn4:NM_001128599:exon7:c.C1049A:p.T350N,Opn4:NM_013887:exon7:c.C1049A:p.T350N | . | 14qB | . | 1 | 0 |
| chr14 | 34597417 | G | C | 228 | Opn4 | exonic | missense SNV | Opn4:NM_001128599:exon3:c.C364G:p.Q122E,Opn4:NM_013887:exon3:c.C364G:p.Q122E | . | 14qB | . | 1 | 0 |
| chr14 | 37023950 | C | G | 139 | . | intergenic | . | . | . | 14qB | . | 0 | 1 |
| chr14 | 40842416 | A | C | 222 | Sh2d4b | exonic | missense SNV | Sh2d4b:NM_177816:exon5:c.T815G:p.L272R | . | 14qB | . | 0 | 1 |
| chr14 | 41158929 | T | G | 215 | Mbl1 | UTR3 | . | . | . | 14qB | . | 0 | 1 |
| chr14 | 44853496 | G | A | 188 | Ptgdr | intronic | . | . | . | 14qC1 | . | 0 | 1 |
| chr14 | 44989243 | A | G | 202 | Ptger2 | exonic | synonymous SNV | Ptger2:NM_008964:exon1:c.A279G:p.S93S | 618 | 14qC1 | . | 0 | 1 |
| chr14 | 45629795 | C | G | 228 | Ddhd1 | intronic | . | . | . | 14qC1 | . | 1 | 0 |
| chr14 | 47568390 | C | A | 193 | Atg14 | exonic | missense SNV | Atg14:NM_172599:exon1:c.G17T:p.G6V | . | 14qC1 | . | 1 | 0 |
| chr14 | 48409311 | T | A | 228 | . | intergenic | . | . | . | 14qC1 | . | 1 | 0 |
| chr14 | 48793408 | A | T | 228 | . | downstream | . | . | . | 14qC1 | . | 1 | 0 |
| chr14 | 49318484 | T | C | 25.8241 | Slc35f4 | intronic | . | . | . | 14qC1 | . | 0 | 1 |
| chr14 | 49319007 | T | C | 228 | Slc35f4 | splicing | . | . | . | 14qC1 | . | 1 | 0 |
| chr14 | 49689280 | T | G | 179 | 3632451006Rik | exonic | missense SNV | 3632451006Rik:NM_026142:exon6:c.A2111C:p.K704T | . | 14qC1 | . | 0 | 1 |
| chr14 | 49751276 | T | G | 164 | 3632451006Rik | intronic | . | . | . | 14qC1 | . | 0 | 1 |
| chr14 | 50035401 | T | G | 222 | Olfr725 | exonic | missense SNV | Olfr725:NM_146317:exon1:c.A1C:p.M1L | . | 14qC1 | . | 0 | 1 |
| chr14 | 50148357 | A | C | 228 | Olfr729 | exonic | missense SNV | Olfr729:NM_146278:exon1:c.T516G:p.N172K | . | 14qC1 | . | 1 | 0 |
| chr14 | 50618431 | T | C | 222 | Olfr744 | exonic | missense SNV | Olfr744:NM_001011738:exon1:c.T208C:p.F70L | . | 14qC1 | . | 0 | 1 |
| chr14 | 51314602 | C | T | 222 | . | intergenic | . | . | . | 14qC1 | . | 0 | 1 |
| chr14 | 52136538 | A | C | 228 | Rpgrip1 | intronic | . | . | . | 14qC2 | . | 1 | 0 |
| chr14 | 52170715 | C | T | 129 | Supt16 | intronic | . | . | . | 14qC2 | . | 0 | 1 |
| chr14 | 52232817 | C | A | 215 | Chd8 | exonic | missense SNV | Chd8:NM_201637:exon3:c.G1335T:p.K445N | . | 14qC2 | . | 0 | 1 |
| chr14 | 52427764 | T | C | 162 | . | intergenic | . | . | . | 14qC2 | . | 1 | 0 |
| chr14 | 53777193 | G | A | 222 | . | intergenic | . | . | . | 14qC2 | . | 0 | 1 |
| chr14 | 53845933 | A | G | 208 | . | intergenic | . | . | . | 14qC2 | rs50834680 | 1 | 0 |
| chr14 | 53876195 | C | T | 165 | . | intergenic | . | . | . | 14qC2 | rs3156911 | 0 | 1 |
| chr14 | 54173829 | G | C | 222 | . | intergenic | . | . | . | 14qC2 | . | 0 | 1 |
| chr14 | 54180357 | G | A | 160 | . | intergenic | . | . | . | 14qC2 | . | 1 | 0 |
| chr14 | 54436507 | G | T | 193 | Mmp14 | intronic | . | . | . | 14qC2 | . | 1 | 0 |
| chr14 | 54627424 | C | A | 222 | Psmb11 | UTR3 | . | . | . | 14qC2 | . | 0 | 1 |
| chr14 | 54644095 | T | C | 110 | Acin1 | exonic | synonymous SNV | Acin1:NM_001242605:exon10:c.A1122G:p.E374E,Acin1:NM_001242606:exon10:c.A1083G:p.E361E,Acin1:N | . | 14qC2 | . | 1 | 0 |
| chr14 | 54729444 | A | G | 141 | Slc7a8 | intronic | . | . | . | 14qC2 | . | 0 | 1 |
| chr14 | 54941962 | C | A | 228 | Myh6 | UTR3 | . | . | . | 14qC2 | . | 1 | 0 |
| chr14 | 54946609 | G | A | 60 | Myh6 | intronic | . | . | . | 14qC2 | . | 0 | 1 |
| chr14 | 54978405 | C | T | 112 | Myh7 | intronic | . | . | . | 14qC3 | . | 1 | 0 |
| chr14 | 54978407 | C | G | 112 | Myh7 | intronic | . | . | . | 14qC3 | . | 1 | 0 |
| chr14 | 55065201 | G | A | 222 | Zfhx2 | exonic | synonymous SNV | Zfhx2:NM_001039198:exon9:c.C5325T:p.L1775L | . | 14qC3 | . | 0 | 1 |
| chr14 | 55494343 | C | T | 222 | Lrrc16b | intronic | . | . | . | 14qC3 | . | 0 | 1 |
| chr14 | 55504585 | G | A | 228 | Lrrc16b | exonic | missense SNV | Lrrc16b:NM_001024645:exon34:c.G3511A:p.D1171N | . | 14qC3 | . | 1 | 0 |
| chr14 | 55709094 | T | C | 222 | Tgm1 | intronic | . | . | . | 14qC3 | . | 0 | 1 |
| chr14 | 55887723 | C | A | 222 | Khnyn | exonic | synonymous SNV | Khnyn:NM_027143:exon4:c.C1338A:p.L446L | . | 14qC3 | . | 0 | 1 |
| chr14 | 56376326 | T | G | 222 | Atp12a | intronic | . | . | . | 14qC3 | . | 0 | 1 |
| chr14 | 56516524 | T | C | 199 | Rnf17 | intronic | . | . | . | 14qC3 | . | 0 | 1 |
| chr14 | 57598032 | C | G | 228 | Xpo4 | exonic | missense SNV | Xpo4:NM_020506:exon14:c.G1831C:p.V611L | . | 14qC3 | . | 1 | 0 |
| chr14 | 57694191 | C | A | 135 | Lats2 | intronic | . | . | . | 14qC3 | . | 1 | 0 |
| chr14 | 58049581 | G | A | 212 | . | intergenic | . | . | . | 14qC3 | . | 0 | 1 |
| chr14 | 59178302 | A | C | 179 | Gm5142 | intronic | . | . | . | 14qC3 | . | 0 | 1 |
| chr14 | 60141460 | G | C | 228 | . | intergenic | . | . | . | 14qD1 | . | 1 | 0 |
| chr14 | 60801974 | T | G | 123 | Mipep | intronic | . | . | . | 14qD1 | . | 0 | 1 |
| chr14 | 61208353 | G | A | 222 | Sacs | intronic | . | . | . | 14qD1 | . | 0 | 1 |

| | | | | | | | | | | | | | |
|---|---|---|---|---|---|---|---|---|---|---|---|---|---|
| chr14 | 61611635 | T | G | 201 | Kcnrg,Dleu2 | ncRNA_intronic | . | . | . | 14qD1 | . | 0 | 1 |
| chr14 | 62448140 | A | G | 176 | Gucy1b2 | intronic | . | . | . | 14qD1 | . | 0 | 1 |
| chr14 | 62587764 | G | A | 222 | Fam124a | exonic | missense SNV | Fam124a:NM_001243857:exon3:c.G706A:p.V236M | . | 14qD1 | . | 0 | 1 |
| chr14 | 63177982 | A | G | 126 | Fdft1 | UTR5 | . | . | 469 | 14qD1 | . | 1 | 0 |
| chr14 | 64095079 | C | T | 228 | Prss51 | intronic | . | . | . | 14qD1 | . | 1 | 0 |
| chr14 | 64897145 | C | A | 228 | Hmbox1 | intronic | . | . | . | 14qD1 | . | 1 | 0 |
| chr14 | 66355733 | T | C | 222 | Stmn4 | intronic | . | . | . | 14qD1 | . | 0 | 1 |
| chr14 | 66968046 | G | A | 118 | . | intergenic | . | . | . | 14qD1 | . | 0 | 1 |
| chr14 | 67038764 | A | G | 131 | Ppp2r2a | intronic | . | . | . | 14qD1 | . | 0 | 1 |
| chr14 | 67429716 | T | C | 222 | Ebf2 | UTR3 | . | . | . | 14qD1 | . | 0 | 1 |
| chr14 | 68514857 | G | A | 222 | Adam7 | exonic | missense SNV | Adam7:NM_007402:exon12:c.C1177T:p.P393S | . | 14qD1 | . | 0 | 1 |
| chr14 | 68642150 | A | C | 222 | Adam28 | exonic | missense SNV | Adam28:NM_001048175:exon6:c.T448G:p.L150V,Adam28:NM_010082:exon6:c.T448G:p.L150V,Adam28:NM_18 | . | 14qD1 | . | 0 | 1 |
| chr14 | 70461658 | G | T | 88 | Phyhip | intronic | . | . | . | 14qD2 | . | 0 | 1 |
| chr14 | 70585824 | C | A | 228 | Fam160b2 | intronic | . | . | . | 14qD2 | . | 1 | 0 |
| chr14 | 70585825 | A | T | 228 | Fam160b2 | intronic | . | . | . | 14qD2 | . | 1 | 0 |
| chr14 | 73029181 | T | A | 67 | Cysltr2 | UTR3 | . | . | . | 14qD3 | . | 0 | 1 |
| chr14 | 75592093 | C | A | 131 | Spert | intronic | . | . | . | 14qD3 | . | 1 | 0 |
| chr14 | 76110883 | T | G | 228 | . | upstream | . | . | 710 | 14qD3 | . | 1 | 0 |
| chr14 | 76126392 | G | T | 222 | Nufip1 | intronic | . | . | . | 14qD3 | rs230381305 | 0 | 1 |
| chr14 | 77963463 | C | T | 98 | Epsti1 | intronic | . | . | . | 14qD3 | . | 0 | 1 |
| chr14 | 79020820 | A | T | 80 | Vwa8 | intronic | . | . | . | 14qD3 | . | 0 | 1 |
| chr14 | 79768935 | C | G | 216 | Pcdh8 | exonic | synonymous SNV | Pcdh8:NM_001042726:exon1:c.G2187C:p.R729R,Pcdh8:NM_021543:exon1:c.G2187C:p.R729R | 2966 | 14qD3 | . | 0 | 1 |
| chr14 | 80021650 | G | T | 228 | Olfm4 | exonic | missense SNV | Olfm4:NM_001030294:exon7:c.G1337T:p.W446L | . | 14qD3 | . | 1 | 0 |
| chr14 | 86866528 | C | T | 228 | Diaph3 | exonic | missense SNV | Diaph3:NM_019670:exon21:c.G2419A:p.E807K | . | 14qE1 | . | 1 | 0 |
| chr14 | 88468506 | C | G | 228 | Pcdh20 | exonic | missense SNV | Pcdh20:NM_178685:exon3:c.G1357C:p.A453P | . | 14qE1 | . | 1 | 0 |
| chr14 | 88468512 | G | T | 85 | Pcdh20 | exonic | missense SNV | Pcdh20:NM_178685:exon3:c.C1351A:p.P451T | . | 14qE1 | . | 1 | 0 |
| chr14 | 88469564 | T | G | 222 | Pcdh20 | exonic | missense SNV | Pcdh20:NM_178685:exon3:c.A299C:p.N100T | . | 14qE1 | . | 0 | 1 |
| chr14 | 95339633 | A | G | 218 | . | intergenic | . | . | . | 14qE2.1 | rs223733892 | 0 | 1 |
| chr14 | 95420889 | C | A | 156 | . | intergenic | . | . | . | 14qE2.1 | rs46337357 | 0 | 1 |
| chr14 | 96201174 | A | C | 134 | Klhl1 | intronic | . | . | . | 14qE2.1 | . | 0 | 1 |
| chr14 | 98519621 | T | A | 80 | . | intergenic | . | . | . | 14qE2.1 | . | 0 | 1 |
| chr14 | 99060333 | G | A | 222 | Bora | intronic | . | . | . | 14qE2.2 | . | 0 | 1 |
| chr14 | 99366042 | C | A | 228 | . | intergenic | . | . | . | 14qE2.2 | . | 1 | 0 |
| chr14 | 101462996 | G | A | 222 | Tbc1d4 | exonic | synonymous SNV | Tbc1d4:NM_001081278:exon12:c.C2307T:p.D769D | . | 14qE2.3 | rs261877387 | 0 | 1 |
| chr14 | 103054937 | G | C | 222 | Irg1 | exonic | missense SNV | Irg1:NM_008392:exon5:c.G896C:p.R299T | . | 14qE2.3 | . | 0 | 1 |
| chr14 | 103120570 | T | C | 170 | Mycbp2 | exonic | missense SNV | Mycbp2:NM_207215:exon82:c.A13787G:p.Y4596C | . | 14qE2.3 | . | 0 | 1 |
| chr14 | 103227682 | C | T | 106 | Mycbp2 | intronic | . | . | . | 14qE2.3 | . | 0 | 1 |
| chr14 | 103529323 | A | C | 28.2335 | Scel | intronic | . | . | . | 14qE2.3 | . | 0 | 1 |
| chr14 | 103610757 | C | T | 222 | Scel | exonic | stopgain | Scel:NM_022886:exon30:c.C1873T:p.Q625X | . | 14qE2.3 | . | 0 | 1 |
| chr14 | 103702685 | C | G | 133 | Slain1 | exonic | missense SNV | Slain1:NM_198014:exon6:c.C1511G:p.S504C | . | 14qE2.3 | . | 0 | 1 |
| chr14 | 109078024 | G | T | 159 | . | intergenic | . | . | . | 14qE3 | . | 0 | 1 |
| chr14 | 110556494 | C | T | 187 | . | intergenic | . | . | . | 14qE3 | . | 0 | 1 |
| chr14 | 110751467 | A | G | 222 | Slitrk6 | exonic | synonymous SNV | Slitrk6:NM_175499:exon2:c.T807C:p.T269T | . | 14qE3 | . | 0 | 1 |
| chr14 | 111680643 | T | C | 222 | Slitrk5 | exonic | synonymous SNV | Slitrk5:NM_198865:exon2:c.T1698C:p.D566D | . | 14qE4 | . | 0 | 1 |
| chr14 | 115044260 | C | T | 222 | Mir20a | ncRNA_exonic | . | . | . | 14qE4 | . | 0 | 1 |
| chr14 | 118596645 | G | A | 222 | Abcc4 | intronic | . | . | . | 14qE4 | . | 0 | 1 |
| chr14 | 118975174 | A | C | 228 | Dnajc3 | exonic | missense SNV | Dnajc3:NM_008929:exon10:c.A1118C:p.D373A | . | 14qE4 | . | 1 | 0 |
| chr14 | 119044433 | C | T | 162 | Uggt2 | intronic | . | . | . | 14qE4 | rs49518016 | 0 | 1 |
| chr14 | 120503449 | A | T | 101 | Rap2a | intronic | . | . | . | 14qE4 | . | 0 | 1 |
| chr14 | 121337267 | C | T | 184 | Stk24 | intronic | . | . | . | 14qE5 | . | 1 | 0 |
| chr14 | 121583770 | A | G | 222 | Dock9 | exonic | synonymous SNV | Dock9:NM_001081039:exon40:c.T4329C:p.N1443N,Dock9:NM_001128307:exon40:c.T4305C:p.N1435N,Dock9 | . | 14qE5 | . | 0 | 1 |
| chr14 | 121586138 | C | A | 228 | Dock9 | intronic | . | . | . | 14qE5 | . | 1 | 0 |
| chr14 | 121911570 | A | T | 228 | Gpr18,Ubac2 | UTR3 | . | . | . | 14qE5 | . | 1 | 0 |
| chr14 | 122463751 | A | G | 132 | Zic5 | intronic | . | . | 1857 | 14qE5 | rs263558720 | 1 | 0 |
| chr14 | 124398705 | G | A | 109 | Fgf14 | intronic | . | . | . | 14qE5 | . | 0 | 1 |
| chr15 | 4782085 | A | G | 115 | C6 | intronic | . | . | . | 15qA1 | . | 0 | 1 |
| chr15 | 4899113 | T | G | 139 | Mroh2b | UTR5 | . | . | . | 15qA1 | . | 0 | 1 |
| chr15 | 4944597 | A | G | 124 | Mroh2b | intronic | . | . | . | 15qA1 | . | 0 | 1 |
| chr15 | 5059333 | T | C | 73 | C7 | intronic | . | . | . | 15qA1 | . | 0 | 1 |
| chr15 | 5243781 | C | G | 193 | Ptger4 | UTR5 | . | . | . | 15qA1 | . | 0 | 1 |
| chr15 | 5951852 | G | C | 220 | . | intergenic | . | . | . | 15qA1 | . | 0 | 1 |
| chr15 | 6483485 | C | T | 156 | C9 | intronic | . | . | . | 15qA1 | . | 0 | 1 |
| chr15 | 6660811 | A | C | 222 | Fyb | intronic | . | . | . | 15qA1 | . | 0 | 1 |
| chr15 | 7231770 | T | G | 221 | Egflam | splicing | . | . | . | 15qA1 | . | 0 | 1 |
| chr15 | 8222482 | A | C | 74 | 2410089E03Rik | intronic | . | . | . | 15qA1 | . | 0 | 1 |
| chr15 | 8350920 | T | A | 222 | Nipbl | exonic | missense SNV | Nipbl:NM_027707:exon10:c.A2387T:p.E796V,Nipbl:NM_201232:exon10:c.A2387T:p.E796V | . | 15qA1 | . | 0 | 1 |
| chr15 | 8529688 | G | C | 48.7322 | . | intergenic | . | . | . | 15qA1 | rs231263453 | 0 | 1 |
| chr15 | 9306703 | A | C | 57 | Ugt3a1 | intronic | . | . | . | 15qA1 | . | 0 | 1 |
| chr15 | 9310410 | T | A | 68 | Ugt3a1 | intronic | . | . | . | 15qA1 | . | 0 | 1 |
| chr15 | 9361850 | G | T | 221 | Ugt3a2 | exonic | missense SNV | Ugt3a2:NM_144845:exon4:c.G711T:p.L237F | . | 15qA1 | . | 0 | 1 |
| chr15 | 9717529 | G | A | 220 | Spef2 | exonic | synonymous SNV | Spef2:NM_001305044:exon6:c.C729T:p.D243D,Spef2:NM_177123:exon6:c.C729T:p.D243D,Spef2:NM_0013( | . | 15qA1 | rs227077617 | 0 | 1 |
| chr15 | 10461218 | C | A | 148 | Dnajc21 | exonic | missense SNV | Dnajc21:NM_030046:exon5:c.G645T:p.K215N | . | 15qA1 | . | 0 | 1 |
| chr15 | 10589462 | C | A | 95 | Rai14 | intronic | . | . | . | 15qA1 | . | 0 | 1 |
| chr15 | 12368008 | C | T | 116 | Pdzd2 | exonic | missense SNV | Pdzd2:NM_001081064:exon22:c.G7894A:p.V2632I | . | 15qA1 | . | 0 | 1 |
| chr15 | 12410844 | C | G | 168 | Pdzd2 | intronic | . | . | . | 15qA1 | . | 0 | 1 |
| chr15 | 12410914 | A | G | 228 | Pdzd2 | intronic | . | . | . | 15qA1 | rs31945760 | 1 | 0 |
| chr15 | 12810938 | G | A | 193 | 6030458C11Rik | intronic | . | . | . | 15qA1 | . | 0 | 1 |
| chr15 | 12901246 | T | G | 148 | Drosha | intronic | . | . | . | 15qA1 | . | 0 | 1 |
| chr15 | 13044453 | G | T | 82 | Cdh6 | intronic | . | . | . | 15qA1 | . | 0 | 1 |
| chr15 | 13051261 | T | C | 200 | Cdh6 | intronic | . | . | . | 15qA1 | . | 0 | 1 |
| chr15 | 13714262 | C | A | 41.2341 | . | intergenic | . | . | . | 15qA1 | . | 0 | 1 |
| chr15 | 16830780 | C | T | 52 | Cdh9 | intronic | . | . | . | 15qA2 | . | 0 | 1 |
| chr15 | 19013495 | C | T | 105 | Cdh10 | exonic | missense SNV | Cdh10:NM_001316758:exon12:c.C2180T:p.A727V,Cdh10:NM_009865:exon12:c.C2180T:p.A727V | . | 15qA2 | . | 0 | 1 |
| chr15 | 19455460 | T | G | 164 | . | intergenic | . | . | . | 15qA2 | . | 0 | 1 |
| chr15 | 19749575 | T | C | 81 | . | intergenic | . | . | . | 15qA2 | . | 0 | 1 |
| chr15 | 20665796 | C | A | 222 | Acot10 | exonic | synonymous SNV | Acot10:NM_022816:exon1:c.G858T:p.V286V | . | 15qA2 | . | 0 | 1 |
| chr15 | 20666732 | C | T | 79 | Acot10 | UTR5 | . | . | . | 15qA2 | . | 0 | 1 |
| chr15 | 21480292 | T | G | 214 | Cdh12 | exonic | missense SNV | Cdh12:NM_001008420:exon4:c.T593G:p.V198G | . | 15qA2 | . | 0 | 1 |
| chr15 | 21578525 | T | G | 211 | Cdh12 | exonic | missense SNV | Cdh12:NM_001008420:exon10:c.T1611G:p.N537K | . | 15qA2 | . | 0 | 1 |
| chr15 | 23436225 | A | G | 89 | Cdh18 | intronic | . | . | . | 15qA2 | . | 0 | 1 |
| chr15 | 23490029 | T | G | 104 | . | intergenic | . | . | . | 15qA2 | . | 0 | 1 |
| chr15 | 25726287 | G | T | 124 | Myo10 | intronic | . | . | . | 15qB1 | . | 0 | 1 |
| chr15 | 25786010 | A | G | 53 | Myo10 | exonic | missense SNV | Myo10:NM_019472:exon27:c.A3776G:p.E1259G | . | 15qB1 | . | 0 | 1 |
| chr15 | 25895163 | A | G | 221 | Fam134b | intronic | . | . | . | 15qB1 | . | 0 | 1 |
| chr15 | 26409043 | G | A | 108 | 11-3月 | intronic | . | . | . | 15qB1 | rs37058400 | 0 | 1 |
| chr15 | 27466986 | C | T | 38.7172 | Ank | UTR5 | . | . | 1079 | 15qB1 | . | 0 | 1 |
| chr15 | 27623343 | G | A | 164 | Otulin | exonic | synonymous SNV | Otulin:NM_001013792:exon2:c.C168T:p.Y56Y | . | 15qB1 | . | 0 | 1 |
| chr15 | 27828011 | A | G | 105 | Trio | intronic | . | . | . | 15qB1 | . | 0 | 1 |
| chr15 | 31655303 | T | G | 135 | . | intergenic | . | . | . | 15qB2 | . | 0 | 1 |
| chr15 | 32641065 | C | A | 80 | Sema5a | exonic | missense SNV | Sema5a:NM_009154:exon16:c.C2024A:p.A675D | . | 15qB3.1 | . | 0 | 1 |
| chr15 | 34316413 | T | G | 36.8999 | Matn2 | intronic | . | . | . | 15qB3.1 | . | 0 | 1 |
| chr15 | 35439111 | A | G | 61 | Vps13b | intronic | . | . | . | 15qB3.1 | . | 0 | 1 |
| chr15 | 35646468 | A | G | 59 | Vps13b | intronic | . | . | . | 15qB3.1 | . | 0 | 1 |
| chr15 | 35879946 | G | A | 222 | Vps13b | exonic | missense SNV | Vps13b:NM_177151:exon50:c.G9166A:p.G3056R | . | 15qB3.1 | . | 0 | 1 |
| chr15 | 36048806 | T | A | 159 | Rgs22 | exonic | missense SNV | Rgs22:NM_001195748:exon18:c.A2699T:p.K900I | . | 15qB3.1 | . | 0 | 1 |
| chr15 | 36247608 | C | G | 221 | Rnf19a | intronic | . | . | . | 15qB3.1 | rs32537887 | 1 | 0 |
| chr15 | 37358497 | T | C | 181 | Grhl2 | intronic | . | . | . | 15qB3.1 | . | 0 | 1 |
| chr15 | 37868232 | C | T | 221 | . | intergenic | . | . | . | 15qB3.1 | . | 0 | 1 |
| chr15 | 38009379 | T | C | 172 | Ubr5 | intronic | . | . | . | 15qB3.1 | . | 0 | 1 |
| chr15 | 39437533 | T | C | 157 | Rims2 | exonic | missense SNV | Rims2:NM_001256382:exon4:c.T1235C:p.M412T,Rims2:NM_001256383:exon4:c.T1235C:p.M412T,Rims2:NM_ | . | 15qB3.1 | . | 0 | 1 |
| chr15 | 39478581 | T | C | 228 | Rims2 | exonic | synonymous SNV | Rims2:NM_001256382:exon13:c.T2665C:p.L889L,Rims2:NM_001256383:exon13:c.T2665C:p.L889L,Rims2:N | . | 15qB3.1 | rs46838335 | 1 | 0 |
| chr15 | 39534703 | T | A | 132 | Rims2 | intronic | . | . | . | 15qB3.1 | . | 0 | 1 |
| chr15 | 39760443 | T | C | 221 | Dcstamp | UTR3 | . | . | . | 15qB3.1 | . | 0 | 1 |
| chr15 | 42496109 | A | C | 80 | Angpt1 | intronic | . | . | . | 15qB3.1 | . | 0 | 1 |
| chr15 | 42523522 | C | A | 221 | Angpt1 | exonic | synonymous SNV | Angpt1:NM_001286062:exon2:c.G435T:p.L145L,Angpt1:NM_009640:exon2:c.G435T:p.L145L | . | 15qB3.1 | . | 0 | 1 |
| chr15 | 42523523 | A | G | 221 | Angpt1 | exonic | missense SNV | Angpt1:NM_001286062:exon2:c.T434C:p.L145P,Angpt1:NM_009640:exon2:c.T434C:p.L145P | . | 15qB3.1 | . | 0 | 1 |
| chr15 | 43866761 | C | A | 222 | Tmem74 | exonic | missense SNV | Tmem74:NM_175502:exon2:c.G885T:p.L295F | . | 15qB3.2 | . | 0 | 1 |
| chr15 | 44547419 | T | A | 116 | Pkhd1l1 | exonic | missense SNV | Pkhd1l1:NM_138674:exon49:c.T8321A:p.V2774D | . | 15qB3.2 | . | 0 | 1 |
| chr15 | 44568294 | C | T | 221 | Pkhd1l1 | exonic | missense SNV | Pkhd1l1:NM_138674:exon61:c.C10043T:p.A3348V | . | 15qB3.2 | . | 0 | 1 |
| chr15 | 44636744 | T | G | 159 | Ebag9 | intronic | . | . | . | 15qB3.2 | . | 0 | 1 |
| chr15 | 44719153 | A | T | 42.487 | Sybu | intronic | . | . | . | 15qB3.2 | . | 0 | 1 |
| chr15 | 44787502 | T | C | 222 | Sybu | intronic | . | . | . | 15qB3.2 | . | 0 | 1 |
| chr15 | 45962634 | C | A | 110 | . | intergenic | . | . | . | 15qB3.3 | . | 0 | 1 |
| chr15 | 47422443 | C | A | 220 | . | intergenic | . | . | . | 15qB3.3 | . | 0 | 1 |
| chr15 | 47587133 | G | A | 47.7371 | Csmd3 | intronic | . | . | . | 15qB3.3 | . | 0 | 1 |
| chr15 | 47606172 | A | G | 199 | Csmd3 | exonic | missense SNV | Csmd3:NM_001081391:exon65:c.T10331C:p.L3444S | . | 15qB3.3 | . | 0 | 1 |
| chr15 | 47659308 | C | G | 45.5975 | Csmd3 | intronic | . | . | . | 15qB3.3 | . | 0 | 1 |
| chr15 | 47733411 | T | A | 142 | Csmd3 | exonic | synonymous SNV | Csmd3:NM_001081391:exon37:c.A6030T:p.S2010S | . | 15qB3.3 | . | 0 | 1 |
| chr15 | 47899839 | C | T | 221 | Csmd3 | exonic | synonymous SNV | Csmd3:NM_001081391:exon24:c.G3912A:p.T1304T | . | 15qB3.3 | . | 0 | 1 |
| chr15 | 52259756 | T | A | 202 | . | intergenic | . | . | . | 15qC | . | 0 | 1 |
| chr15 | 52306740 | A | C | 170 | Slc30a8 | intronic | . | . | . | 15qC | . | 0 | 1 |
| chr15 | 54839691 | C | T | 25.7587 | Enpp2 | intronic | . | . | . | 15qD1 | . | 0 | 1 |
| chr15 | 54845693 | T | G | 52 | Enpp2 | intronic | . | . | . | 15qD1 | . | 0 | 1 |
| chr15 | 54863919 | C | A | 221 | Enpp2 | intronic | . | . | . | 15qD1 | . | 0 | 1 |
| chr15 | 55038095 | T | C | 34.0536 | Taf2 | intronic | . | . | . | 15qD1 | . | 0 | 1 |
| chr15 | 55427853 | C | G | 48.0863 | Col14a1 | intronic | . | . | . | 15qD1 | . | 0 | 1 |
| chr15 | 55448231 | T | C | 142 | Col14a1 | exonic | missense SNV | Col14a1:NM_181277:exon30:c.T3674C:p.L1225P | . | 15qD1 | . | 0 | 1 |
| chr15 | 55567267 | G | A | 33.2445 | Mtbp | exonic | missense SNV | Mtbp:NM_001168250:exon7:c.G719A:p.R240Q,Mtbp:NM_134092:exon7:c.G719A:p.R240Q | . | 15qD1 | . | 0 | 1 |
| chr15 | 55620664 | C | T | 67 | Mtbp | exonic | synonymous SNV | Mtbp:NM_134092:exon20:c.C2508T:p.H836H | . | 15qD1 | . | 0 | 1 |
| chr15 | 56783072 | T | G | 222 | . | intergenic | . | . | . | 15qD1 | . | 0 | 1 |
| chr15 | 57879932 | T | C | 153 | Derl1 | intronic | . | . | . | 15qD1 | . | 0 | 1 |
| chr15 | 58132222 | C | T | 46.8884 | Atad2 | exonic | synonymous SNV | Atad2:NM_027435:exon2:c.G288A:p.E96E | . | 15qD1 | . | 0 | 1 |
| chr15 | 58192211 | C | A | 228 | Fbxo32 | intronic | . | . | . | 15qD1 | rs48688146 | 1 | 0 |
| chr15 | 59317862 | T | C | 171 | Sqle | exonic | missense SNV | Sqle:NM_009270:exon2:c.T352C:p.F118L | . | 15qD1 | . | 0 | 1 |
| chr15 | 59342173 | A | C | 137 | E430025E21Rik | intronic | . | . | . | 15qD1 | . | 0 | 1 |
| chr15 | 60830835 | T | C | 221 | 9930014A18Rik | ncRNA_exonic | . | . | . | 15qD1 | . | 0 | 1 |
| chr15 | 60919663 | T | C | 183 | A1bg | exonic | missense SNV | A1bg:NM_001081067:exon5:c.A923G:p.K308R | . | 15qD1 | . | 0 | 1 |
| chr15 | 61967108 | G | T | 138 | . | intergenic | . | . | . | 15qD1 | . | 1 | 0 |
| chr15 | 63943083 | T | A | 74 | Fam49b | intronic | . | . | . | 15qD1 | . | 0 | 1 |
| chr15 | 64094446 | C | T | 221 | Asap1 | exonic | missense SNV | Asap1:NM_001276462:exon26:c.G2740A:p.E914K,Asap1:NM_001276463:exon26:c.G2704A:p.E902K,Asap1:N | . | 15qD1 | . | 0 | 1 |
| chr15 | 64099470 | G | A | 228 | Asap1 | intronic | . | . | . | 15qD1 | rs31624599 | 1 | 0 |
| chr15 | 64737540 | A | C | 134 | Adcy8 | exonic | missense SNV | Adcy8:NM_001291903:exon11:c.T2429G:p.L810W,Adcy8:NM_009623:exon12:c.T2519G:p.L840W | . | 15qD1 | . | 0 | 1 |
| chr15 | 64920533 | T | C | 222 | Adcy8 | exonic | synonymous SNV | Adcy8:NM_001291903:exon1:c.A573G:p.K191K,Adcy8:NM_009623:exon1:c.A573G:p.K191K | . | 15qD1 | . | 0 | 1 |
| chr15 | 64920857 | G | T | 118 | Adcy8 | exonic | missense SNV | Adcy8:NM_001291903:exon1:c.C249A:p.S83R,Adcy8:NM_009623:exon1:c.C249A:p.S83R | 1950 | 15qD1 | . | 0 | 1 |
| chr15 | 66541578 | T | A | 222 | Tmem71 | exonic | missense SNV | Tmem71:NM_172514:exon6:c.A644T:p.H215L | . | 15qD2 | . | 0 | 1 |
| chr15 | 68178622 | C | G | 108 | Zfat | intronic | . | . | . | 15qD2 | . | 0 | 1 |
| chr15 | 68184465 | G | A | 101 | Zfat | exonic | synonymous SNV | Zfat:NM_001145888:exon5:c.C741T:p.Y247Y,Zfat:NM_198644:exon5:c.C720T:p.Y240Y | . | 15qD2 | . | 0 | 1 |
| chr15 | 69017542 | C | T | 221 | Khdrbs3 | intronic | . | . | . | 15qD3 | . | 0 | 1 |
| chr15 | 71479036 | T | C | 177 | Fam135b | exonic | synonymous SNV | Fam135b:NM_177819:exon10:c.A990G:p.Q330Q | . | 15qD3 | . | 0 | 1 |
| chr15 | 71906972 | G | C | 76 | Col22a1 | intronic | . | . | . | 15qD3 | . | 0 | 1 |
| chr15 | 71922741 | T | C | 63 | Col22a1 | intronic | . | . | . | 15qD3 | . | 0 | 1 |
| chr15 | 72512996 | A | G | 190 | Kcnk9 | exonic | missense SNV | Kcnk9:NM_001033876:exon2:c.T332C:p.M111T | . | 15qD3 | . | 0 | 1 |
| chr15 | 74572732 | T | C | 159 | Adgrb1 | intronic | . | . | . | 15qD3 | . | 0 | 1 |
| chr15 | 74733058 | C | A | 212 | Lypd2 | exonic | synonymous SNV | Lypd2:NM_026671:exon2:c.G87T:p.A29A | . | 15qD3 | . | 0 | 1 |
| chr15 | 74799623 | C | G | 123 | Ly6k | intronic | . | . | . | 15qD3 | . | 0 | 1 |
| chr15 | 75596839 | G | A | 80 | Gpihbp1 | intronic | . | . | . | 15qD3 | . | 0 | 1 |
| chr15 | 75779471 | G | A | 221 | Zc3h3 | intronic | . | . | . | 15qD3 | . | 0 | 1 |
| chr15 | 75902776 | C | T | 92 | Eef1d | exonic | missense SNV | Eef1d:NM_029663:exon3:c.G1033A:p.V345I | . | 15qD3 | . | 0 | 1 |
| chr15 | 75912737 | C | T | 200 | Tigd5 | UTR3 | . | . | . | 15qD3 | . | 0 | 1 |
| chr15 | 75926133 | G | C | 221 | Tsta3 | exonic | stopgain | Tsta3:NM_031201:exon7:c.C659G:p.S220X | . | 15qD3 | . | 0 | 1 |
| chr15 | 75995985 | C | A | 221 | Mapk15 | intronic | . | . | . | 15qD3 | . | 0 | 1 |
| chr15 | 76001827 | T | A | 220 | Fam83h | UTR3 | . | . | . | 15qD3 | . | 0 | 1 |
| chr15 | 76064972 | G | C | 158 | Scrib | exonic | missense SNV | Scrib:NM_001310542:exon11:c.C1211G:p.A404G,Scrib:NM_001310543:exon11:c.C1211G:p.A404G,Scrib:N | . | 15qD3 | . | 0 | 1 |
| chr15 | 76102557 | G | A | 222 | Eppk1 | exonic | stopgain | Eppk1:NM_144848:exon2:c.C10123T:p.R3375X | . | 15qD3 | . | 0 | 1 |
| chr15 | 76173947 | C | G | 222 | Plec | exonic | missense SNV | Plec:NM_011117:exon32:c.G11942C:p.G3981A,Plec:NM_201385:exon32:c.G11828C:p.G3943A,Plec:NM_201 | . | 15qD3 | . | 0 | 1 |
| chr15 | 76178371 | C | A | 156 | Plec | exonic | missense SNV | Plec:NM_011117:exon32:c.G7518T:p.Q2506H,Plec:NM_201385:exon32:c.G7404T:p.Q2468H,Plec:NM_20138 | . | 15qD3 | . | 0 | 1 |
| chr15 | 76183578 | T | G | 198 | Plec | exonic | missense SNV | Plec:NM_011117:exon27:c.A3779C:p.Q1260P,Plec:NM_201385:exon27:c.A3665C:p.Q1222P,Plec:NM_20138 | 263 | 15qD3 | . | 0 | 1 |
| chr15 | 76284150 | C | A | 222 | Spatc1 | exonic | synonymous SNV | Spatc1:NM_028852:exon2:c.C808A:p.R270R | . | 15qD3 | . | 0 | 1 |
| chr15 | 76285075 | C | A | 143 | Spatc1 | intronic | . | . | . | 15qD3 | . | 0 | 1 |
| chr15 | 76614464 | A | C | 221 | Slc39a4 | intronic | . | . | . | 15qD3 | . | 0 | 1 |
| chr15 | 76722641 | C | T | 170 | C030006K11Rik | exonic | missense SNV | C030006K11Rik:NM_145472:exon3:c.G554A:p.G185E | 797 | 15qD3 | . | 0 | 1 |
| chr15 | 77813135 | C | A | 221 | Myh9 | exonic | missense SNV | Myh9:NM_022410:exon2:c.G78T:p.W26C | . | 15qE1 | . | 0 | 1 |
| chr15 | 78255684 | G | T | 82 | Ncf4 | intronic | . | . | . | 15qE1 | . | 0 | 1 |
| chr15 | 78386228 | G | T | 60 | Tex33 | exonic | synonymous SNV | Tex33:NM_001163613:exon2:c.C264A:p.S88S,Tex33:NM_001163612:exon3:c.C339A:p.S113S,Tex33:NM_028 | . | 15qE1 | . | 0 | 1 |
| chr15 | 78602709 | C | A | 218 | Cyth4 | exonic | missense SNV | Cyth4:NM_028195:exon2:c.C36A:p.S12R | . | 15qE1 | . | 0 | 1 |

| | | | | | | | | | | | | | |
|---|---|---|---|---|---|---|---|---|---|---|---|---|---|
| chr15 | 78672061 | G | A | 96 | Elfn2 | exonic | missense SNV | Elfn2:NM_183141:exon2:c.C2285T:p.P762L | 709 | 15qE1 | . | 0 | 1 |
| chr15 | 78776422 | T | C | 23.3612 | Card10 | UTR3 | . | . | . | 15qE1 | rs46048048 | 0 | 1 |
| chr15 | 78776444 | C | T | 31.6409 | Card10 | UTR3 | . | . | . | 15qE1 | rs51100240 | 0 | 1 |
| chr15 | 78777474 | C | G | 222 | Card10 | exonic | missense SNV | Card10:NM_130859:exon20:c.G2966C:p.R989P | . | 15qE1 | . | 0 | 1 |
| chr15 | 79031110 | C | T | 70 | Gcat | exonic | synonymous SNV | Gcat:NM_001161712:exon1:c.C177T:p.G59G,Gcat:NM_013847:exon1:c.C177T:p.G59G | 572 | 15qE1 | . | 0 | 1 |
| chr15 | 79081888 | C | T | 26.66 | Eif3l | exonic | synonymous SNV | Eif3l:NM_145139:exon7:c.C540T:p.N180N | . | 15qE1 | . | 0 | 1 |
| chr15 | 79156535 | C | A | 221 | Sox10 | exonic | missense SNV | Sox10:NM_011437:exon4:c.G804T:p.K268N | . | 15qE1 | . | 0 | 1 |
| chr15 | 79239868 | T | A | 154 | Pick1 | intronic | . | . | . | 15qE1 | . | 0 | 1 |
| chr15 | 79419920 | T | C | 98 | Csnk1e | UTR3 | . | . | . | 15qE1 | . | 0 | 1 |
| chr15 | 79429952 | T | C | 96 | Csnk1e | intronic | . | . | . | 15qE1 | rs50415458 | 1 | 0 |
| chr15 | 79524922 | G | A | 222 | Kdelr3 | exonic | missense SNV | Kdelr3:NM_134090:exon3:c.G311A:p.G104D | . | 15qE1 | . | 0 | 1 |
| chr15 | 81208795 | C | A | 106 | . | intergenic | . | . | . | 15qE1 | . | 0 | 1 |
| chr15 | 81466333 | G | A | 118 | Rbx1 | UTR5 | . | . | 760 | 15qE1 | . | 0 | 1 |
| chr15 | 81499417 | G | C | 182 | . | intergenic | . | . | . | 15qE1 | . | 0 | 1 |
| chr15 | 81668701 | G | A | 220 | L3mbtl2 | exonic | missense SNV | L3mbtl2:NM_001289711:exon3:c.G334A:p.V112I,L3mbtl2:NM_001289712:exon3:c.G334A:p.V112I,L3mbtl2 | . | 15qE1 | . | 0 | 1 |
| chr15 | 81782980 | G | A | 121 | Zc3h7b | intronic | . | . | . | 15qE1 | . | 0 | 1 |
| chr15 | 82041782 | A | G | 190 | Nhp2l1 | UTR3 | . | . | . | 15qE1 | . | 0 | 1 |
| chr15 | 82086393 | T | A | 189 | Mei1 | exonic | synonymous SNV | Mei1:NM_001310435:exon5:c.T117A:p.R39R,Mei1:NM_028897:exon11:c.T1239A:p.R413R | . | 15qE1 | . | 0 | 1 |
| chr15 | 82091520 | G | T | 48.227 | Mei1 | intronic | . | . | . | 15qE1 | . | 0 | 1 |
| chr15 | 82194912 | T | A | 222 | Srebf2 | splicing | . | . | . | 15qE1 | . | 0 | 1 |
| chr15 | 82330537 | C | A | 86 | Naga | intronic | . | . | . | 15qE1 | . | 0 | 1 |
| chr15 | 82443778 | G | A | 42.2006 | . | intergenic | . | . | . | 15qE1 | rs237943998 | 0 | 1 |
| chr15 | 82565317 | A | G | 143 | . | intergenic | . | . | . | 15qE1 | . | 0 | 1 |
| chr15 | 82654527 | A | G | 221 | . | intergenic | . | . | . | 15qE1 | . | 0 | 1 |
| chr15 | 83149689 | C | G | 64 | Rnu12 | ncRNA_exonic | . | . | . | 15qE1 | . | 0 | 1 |
| chr15 | 83162107 | T | C | 228 | Cyb5r3 | intronic | . | . | . | 15qE1 | rs46345941 | 1 | 0 |
| chr15 | 83334737 | T | G | 155 | Arfgap3 | intronic | . | . | . | 15qE1 | . | 0 | 1 |
| chr15 | 83379156 | G | C | 203 | Pacsin2 | exonic | missense SNV | Pacsin2:NM_001159509:exon10:c.C1191G:p.D397E,Pacsin2:NM_001159510:exon10:c.C1191G:p.D397E,Pac | . | 15qE1 | . | 0 | 1 |
| chr15 | 83542935 | C | A | 123 | Bik | intronic | . | . | . | 15qE1 | . | 0 | 1 |
| chr15 | 84086591 | A | C | 221 | Sult4a1 | intronic | . | . | . | 15qE2 | . | 0 | 1 |
| chr15 | 84113125 | G | T | 221 | Pnpla5 | intronic | . | . | . | 15qE2 | . | 0 | 1 |
| chr15 | 84297915 | G | T | 55 | Parvb | intronic | . | . | . | 15qE2 | . | 0 | 1 |
| chr15 | 84960865 | T | C | 79 | 5031439G07Rik | intronic | . | . | . | 15qE2 | . | 0 | 1 |
| chr15 | 85905307 | G | T | 134 | Celsr1 | intronic | . | . | . | 15qE2 | . | 0 | 1 |
| chr15 | 85922170 | C | G | 87 | Celsr1 | exonic | synonymous SNV | Celsr1:NM_009886:exon21:c.G7086C:p.L2362L | . | 15qE2 | . | 0 | 1 |
| chr15 | 85926935 | G | A | 70 | Celsr1 | exonic | missense SNV | Celsr1:NM_009886:exon17:c.C6386T:p.A2129V | . | 15qE2 | . | 0 | 1 |
| chr15 | 85939177 | C | A | 133 | Celsr1 | exonic | missense SNV | Celsr1:NM_009886:exon8:c.G5029T:p.G1677C | . | 15qE2 | . | 0 | 1 |
| chr15 | 87681463 | C | A | 221 | Fam19a5 | intronic | . | . | . | 15qE3 | . | 0 | 1 |
| chr15 | 88965469 | G | A | 222 | Mlc1 | exonic | synonymous SNV | Mlc1:NM_133241:exon8:c.C642T:p.S214S | . | 15qE3 | . | 0 | 1 |
| chr15 | 88994719 | A | G | 216 | Mov10l1 | intronic | . | . | . | 15qE3 | rs31549680 | 1 | 0 |
| chr15 | 89145950 | C | T | 221 | Mapk11 | intronic | . | . | . | 15qE3 | . | 0 | 1 |
| chr15 | 89157528 | C | A | 221 | Plxnb2 | exonic | missense SNV | Plxnb2:NM_001284506:exon33:c.G5072T:p.R1691L,Plxnb2:NM_001159521:exon34:c.G5072T:p.R1691L,Plx | . | 15qE3 | . | 0 | 1 |
| chr15 | 89305918 | G | A | 222 | Sbf1 | intronic | . | . | . | 15qE3 | . | 0 | 1 |
| chr15 | 89307112 | A | C | 157 | Sbf1 | intronic | . | . | . | 15qE3 | . | 0 | 1 |
| chr15 | 89569384 | G | A | 220 | Acr | exonic | missense SNV | Acr:NM_001205049:exon2:c.G125A:p.R42Q,Acr:NM_013455:exon2:c.G125A:p.R42Q | . | 15qE3 | . | 0 | 1 |
| chr15 | 89584099 | C | G | 221 | Rabl2 | intronic | . | . | . | 15qE3 | . | 0 | 1 |
| chr15 | 89782603 | G | T | 79 | Syt10 | exonic | missense SNV | Syt10:NM_018803:exon7:c.C1544A:p.S515Y | . | 15qE3 | . | 0 | 1 |
| chr15 | 90541455 | C | T | 165 | Cpne8 | intronic | . | . | . | 15qE3 | . | 0 | 1 |
| chr15 | 90679191 | T | A | 205 | Cpne8 | exonic | synonymous SNV | Cpne8:NM_001033851:exon1:c.A114T:p.R38R,Cpne8:NM_025815:exon1:c.A114T:p.R38R | 1093 | 15qE3 | . | 0 | 1 |
| chr15 | 90898409 | T | C | 162 | Spn-ps | ncRNA_exonic | . | . | . | 15qE3 | . | 0 | 1 |
| chr15 | 90937049 | C | A | 103 | Kif21a | intronic | . | . | . | 15qE3 | . | 0 | 1 |
| chr15 | 90959865 | A | G | 78 | Kif21a | intronic | . | . | . | 15qE3 | . | 0 | 1 |
| chr15 | 90968645 | C | G | 222 | Kif21a | intronic | . | . | . | 15qE3 | . | 0 | 1 |
| chr15 | 91343894 | C | A | 222 | Slc2a13 | intronic | . | . | . | 15qE3 | . | 0 | 1 |
| chr15 | 91752527 | G | T | 20.0052 | Lrrk2 | intronic | . | . | . | 15qE3 | . | 0 | 1 |
| chr15 | 91844280 | C | A | 153 | Smgc,Muc19 | intronic | . | . | . | 15qE3 | . | 0 | 1 |
| chr15 | 91909167 | A | G | 80 | Muc19 | exonic | synonymous SNV | Muc19:NM_207243:exon34:c.A6693G:p.T2231T | . | 15qE3 | . | 0 | 1 |
| chr15 | 91909288 | A | G | 69 | Muc19 | exonic | missense SNV | Muc19:NM_207243:exon34:c.A6814G:p.T2272A | . | 15qE3 | . | 0 | 1 |
| chr15 | 92317930 | C | A | 222 | Cntn1 | exonic | missense SNV | Cntn1:NM_001159647:exon23:c.C2843A:p.P948Q,Cntn1:NM_001159648:exon23:c.C2843A:p.P948Q,Cntn1:N | . | 15qE3 | . | 0 | 1 |
| chr15 | 93390912 | A | C | 65 | Zcrb1 | intronic | . | . | . | 15qE3 | . | 0 | 1 |
| chr15 | 94280253 | C | A | 25.7522 | Adamts20 | intronic | . | . | . | 15qE3 | . | 0 | 1 |
| chr15 | 94523722 | T | C | 58 | Pus7l | intronic | . | . | . | 15qE3 | . | 0 | 1 |
| chr15 | 95148814 | A | G | 143 | LOC100043315 | ncRNA_intronic | . | . | . | 15qE3 | . | 0 | 1 |
| chr15 | 95229032 | C | T | 222 | Nell2 | intronic | . | . | 423 | 15qE3 | . | 0 | 1 |
| chr15 | 95654748 | G | T | 127 | Dbx2 | exonic | missense SNV | Dbx2:NM_207533:exon1:c.C15A:p.D5E | . | 15qE3 | . | 0 | 1 |
| chr15 | 96188586 | C | T | 107 | Gm4371 | ncRNA_intronic | . | . | . | 15qF1 | . | 0 | 1 |
| chr15 | 96371363 | A | G | 222 | Arid2 | exonic | missense SNV | Arid2:NM_175251:exon15:c.A3356G:p.Q1119R | . | 15qF1 | . | 0 | 1 |
| chr15 | 96692641 | C | A | 221 | Slc38a2 | intronic | . | . | . | 15qF1 | . | 0 | 1 |
| chr15 | 96998536 | A | C | 221 | Slc38a4 | exonic | synonymous SNV | Slc38a4:NM_027052:exon15:c.T1449G:p.A483A | . | 15qF1 | . | 0 | 1 |
| chr15 | 97383917 | G | T | 136 | Pced1b | intronic | . | . | . | 15qF1 | . | 0 | 1 |
| chr15 | 97754414 | A | T | 112 | Rapgef3 | intronic | . | . | . | 15qF1 | . | 0 | 1 |
| chr15 | 97756786 | G | T | 129 | Rapgef3 | intronic | . | . | . | 15qF1 | . | 0 | 1 |
| chr15 | 97977716 | C | T | 221 | Col2a1 | intronic | . | . | . | 15qF1 | . | 0 | 1 |
| chr15 | 97984016 | T | C | 222 | Col2a1 | splicing | . | . | . | 15qF1 | . | 0 | 1 |
| chr15 | 97985991 | C | A | 38.9725 | Col2a1 | intronic | . | . | . | 15qF1 | . | 0 | 1 |
| chr15 | 97994697 | A | T | 222 | Col2a1 | intronic | . | . | . | 15qF1 | . | 0 | 1 |
| chr15 | 98122643 | G | A | 221 | Pfkm | exonic | missense SNV | Pfkm:NM_021514:exon7:c.G652A:p.V218I,Pfkm:NM_001163487:exon8:c.G652A:p.V218I,Pfkm:NM_0011634{ | . | 15qF1 | . | 0 | 1 |
| chr15 | 98720812 | G | A | 184 | Ccdc65 | intronic | . | . | . | 15qF1 | . | 0 | 1 |
| chr15 | 98726275 | C | G | 111 | Fkbp11 | intronic | . | . | . | 15qF1 | . | 0 | 1 |
| chr15 | 98813679 | C | A | 221 | Prkag1 | exonic | missense SNV | Prkag1:NM_016781:exon11:c.G803T:p.R268L | . | 15qF1 | . | 0 | 1 |
| chr15 | 98849043 | C | A | 115 | Kmt2d | intronic | . | . | . | 15qF1 | . | 0 | 1 |
| chr15 | 98857920 | C | A | 221 | Kmt2d | intronic | . | . | . | 15qF1 | . | 0 | 1 |
| chr15 | 98864620 | C | T | 221 | Kmt2d | intronic | . | . | . | 15qF1 | . | 0 | 1 |
| chr15 | 99055687 | C | T | 100 | Prph | exonic | synonymous SNV | Prph:NM_001163588:exon1:c.C481T:p.L161L,Prph:NM_001163589:exon1:c.C481T:p.L161L,Prph:NM_0136 | 675 | 15qF1 | . | 0 | 1 |
| chr15 | 99081279 | G | T | 115 | Troap | intronic | . | . | . | 15qF1 | . | 0 | 1 |
| chr15 | 99173554 | A | T | 221 | Spats2 | intronic | . | . | . | 15qF1 | . | 0 | 1 |
| chr15 | 99278934 | C | A | 220 | Fam186b | intronic | . | . | . | 15qF1 | . | 0 | 1 |
| chr15 | 99309159 | A | G | 151 | Prpf40b | exonic | missense SNV | Prpf40b:NM_018786:exon16:c.A1457G:p.E486G | . | 15qF1 | . | 0 | 1 |
| chr15 | 99315559 | A | C | 198 | Prpf40b | exonic | missense SNV | Prpf40b:NM_018786:exon22:c.A2129C:p.H710P | . | 15qF1 | . | 0 | 1 |
| chr15 | 99427490 | C | T | 113 | Nckap5l | exonic | stopgain | Nckap5l:NM_001001884:exon8:c.G1131A:p.W377X | . | 15qF1 | . | 0 | 1 |
| chr15 | 99592747 | C | A | 67 | Aqp5 | exonic | missense SNV | Aqp5:NM_009701:exon2:c.C449A:p.T150K | . | 15qF1 | . | 0 | 1 |
| chr15 | 99694749 | C | T | 222 | Asic1 | exonic | missense SNV | Asic1:NM_001289791:exon2:c.C713T:p.P238L,Asic1:NM_009597:exon4:c.C614T:p.P205L | . | 15qF1 | . | 0 | 1 |
| chr15 | 99694750 | A | T | 221 | Asic1 | exonic | synonymous SNV | Asic1:NM_001289791:exon2:c.A714T:p.P238P,Asic1:NM_009597:exon4:c.A615T:p.P205P | . | 15qF1 | . | 0 | 1 |
| chr15 | 99796714 | C | T | 68 | Lima1 | intronic | . | . | . | 15qF1 | . | 0 | 1 |
| chr15 | 100157105 | G | T | 135 | Mir3473a,Dip2b | ncRNA_intronic | . | . | . | 15qF1 | . | 0 | 1 |
| chr15 | 100183054 | T | C | 192 | Dip2b,Mir3473a | exonic | missense SNV | Dip2b:NM_172819:exon17:c.T1874C:p.V625A,Dip2b:NM_001159361:exon22:c.T2576C:p.V859A | . | 15qF1 | . | 0 | 1 |
| chr15 | 100335260 | G | C | 185 | Mettl7a3 | exonic | missense SNV | Mettl7a3:NM_001081471:exon1:c.G331C:p.V111L | . | 15qF1 | . | 0 | 1 |
| chr15 | 100484409 | C | T | 222 | Csrnp2 | intronic | . | . | . | 15qF1 | . | 0 | 1 |
| chr15 | 100583299 | C | G | 143 | Pou6f1 | exonic | missense SNV | Pou6f1:NM_010127:exon3:c.G178C:p.A60P,Pou6f1:NM_001304965:exon8:c.G1003C:p.A335P | . | 15qF1 | . | 0 | 1 |
| chr15 | 100587886 | G | A | 44.6348 | Pou6f1 | exonic | synonymous SNV | Pou6f1:NM_001304965:exon4:c.C261T:p.S87S | . | 15qF1 | . | 0 | 1 |
| chr15 | 100656387 | A | T | 221 | Bin2 | intronic | . | . | . | 15qF1 | . | 0 | 1 |
| chr15 | 100972954 | G | T | 187 | Scn8a | exonic | missense SNV | Scn8a:NM_011323:exon9:c.G1335T:p.E445D,Scn8a:NM_001077499:exon10:c.G1335T:p.E445D | . | 15qF1 | . | 0 | 1 |
| chr15 | 100973040 | G | T | 95 | Scn8a | intronic | . | . | . | 15qF1 | . | 0 | 1 |
| chr15 | 101018415 | A | C | 222 | Scn8a | exonic | missense SNV | Scn8a:NM_011323:exon19:c.A3704C:p.K1235T,Scn8a:NM_001077499:exon20:c.A3704C:p.K1235T | . | 15qF1 | . | 0 | 1 |
| chr15 | 101119168 | T | C | 155 | Ankrd33 | exonic | missense SNV | Ankrd33:NM_144790:exon4:c.T461C:p.L154P | . | 15qF1 | . | 0 | 1 |
| chr15 | 101119899 | T | A | 177 | Ankrd33 | UTR3 | . | . | . | 15qF1 | . | 0 | 1 |
| chr15 | 101287068 | G | A | 140 | Atg101 | exonic | missense SNV | Atg101:NM_026566:exon2:c.G70A:p.V24M | . | 15qF1 | . | 0 | 1 |
| chr15 | 101453039 | G | T | 222 | Krt85,1700011A15R | intronic | . | . | . | 15qF2 | . | 0 | 1 |
| chr15 | 101473902 | G | T | 221 | Krt85,Krt86 | exonic | missense SNV | Krt86:NM_010667:exon1:c.G352T:p.A118S | . | 15qF2 | . | 0 | 1 |
| chr15 | 101486217 | C | A | 120 | Krt85,5430421N21R | intronic | . | . | . | 15qF2 | . | 0 | 1 |
| chr15 | 101541579 | G | T | 93 | Krt82 | UTR3 | . | . | . | 15qF2 | . | 0 | 1 |
| chr15 | 101545365 | T | C | 79 | Krt82 | intronic | . | . | . | 15qF2 | . | 0 | 1 |
| chr15 | 101546228 | T | C | 221 | Krt82 | intronic | . | . | . | 15qF2 | . | 0 | 1 |
| chr15 | 101627694 | G | T | 222 | Gm5414 | exonic | synonymous SNV | Gm5414:NM_001003670:exon1:c.C495A:p.S165S | . | 15qF2 | . | 0 | 1 |
| chr15 | 101800740 | G | A | 107 | Krt73 | exonic | missense SNV | Krt73:NM_212485:exon2:c.C631T:p.R211W | . | 15qF2 | . | 0 | 1 |
| chr15 | 101920480 | G | A | 190 | Krt4 | intronic | . | . | . | 15qF2 | . | 0 | 1 |
| chr15 | 102073895 | T | C | 108 | Eif4b | UTR5 | . | . | 1031 | 15qF2 | . | 0 | 1 |
| chr15 | 102163114 | A | G | 58 | Soat2 | UTR3 | . | . | . | 15qF2 | . | 0 | 1 |
| chr15 | 102187746 | C | A | 140 | Csad | intronic | . | . | . | 15qF2 | . | 0 | 1 |
| chr15 | 102302802 | G | T | 222 | Espl1 | intronic | . | . | . | 15qF3 | . | 0 | 1 |
| chr15 | 102709125 | G | A | 222 | Calcoco1 | exonic | missense SNV | Calcoco1:NM_026192:exon13:c.C1742T:p.P581L | . | 15qF3 | . | 0 | 1 |
| chr15 | 103034583 | C | A | 40.1476 | Hoxc4 | UTR5 | . | . | . | 15qF3 | . | 0 | 1 |
| chr15 | 103439771 | C | T | 180 | BC048502 | intronic | . | . | . | 15qF3 | . | 0 | 1 |
| chr15 | 103526483 | C | G | 131 | Pde1b | intronic | . | . | . | 15qF3 | . | 0 | 1 |
| chr16 | 3730785 | T | A | 222 | . | intergenic | . | . | . | 16qA1 | . | 0 | 1 |
| chr16 | 4044562 | C | G | 221 | Trap1 | intronic | . | . | . | 16qA1 | . | 0 | 1 |
| chr16 | 4096265 | C | G | 203 | Crebbp | intronic | . | . | . | 16qA1 | . | 0 | 1 |
| chr16 | 4230947 | T | A | 222 | Gm5766 | ncRNA_exonic | . | . | . | 16qA1 | . | 0 | 1 |
| chr16 | 4633305 | G | A | 222 | Coro7 | exonic | missense SNV | Coro7:NM_030205:exon16:c.C1519T:p.R507C | . | 16qA1 | . | 0 | 1 |
| chr16 | 4922606 | G | A | 218 | Mgrn1 | exonic | missense SNV | Mgrn1:NM_001252437:exon10:c.G811A:p.E271K,Mgrn1:NM_029657:exon10:c.G808A:p.E270K | . | 16qA1 | . | 0 | 1 |
| chr16 | 4946120 | C | A | 221 | Anks3 | exonic | missense SNV | Anks3:NM_028301:exon11:c.G1341T:p.E447D | . | 16qA1 | . | 0 | 1 |
| chr16 | 7285536 | G | A | 117 | Rbfox1 | intronic | . | . | . | 16qA1 | . | 0 | 1 |
| chr16 | 7330412 | G | A | 221 | Rbfox1 | exonic | missense SNV | Rbfox1:NM_183188:exon8:c.G784A:p.G262S,Rbfox1:NM_021477:exon12:c.G724A:p.G242S | . | 16qA1 | . | 0 | 1 |
| chr16 | 8487324 | G | T | 172 | Mettl22 | intronic | . | . | . | 16qA1 | . | 0 | 1 |
| chr16 | 8996461 | T | C | 228 | . | intergenic | . | . | . | 16qA1 | rs108816945 | 1 | 0 |
| chr16 | 10513358 | A | G | 222 | Ciita | intronic | . | . | . | 16qA1 | . | 0 | 1 |
| chr16 | 10542490 | C | G | 222 | Dexi | exonic | synonymous SNV | Dexi:NM_021428:exon1:c.G201C:p.L67L | 729 | 16qA1 | . | 0 | 1 |
| chr16 | 11134119 | G | A | 22.9835 | Txndc11 | intronic | . | . | 892 | 16qA1 | . | 0 | 1 |
| chr16 | 11162678 | G | A | 228 | Zc3h7a | splicing | . | . | . | 16qA1 | rs49821199 | 1 | 0 |
| chr16 | 13652852 | G | T | 51 | Parn | intronic | . | . | . | 16qA1 | . | 0 | 1 |
| chr16 | 13780765 | C | T | 60 | Rrn3 | exonic | synonymous SNV | Rrn3:NM_001039521:exon1:c.C9T:p.A3A | 389 | 16qA1 | . | 0 | 1 |
| chr16 | 13879705 | C | G | 124 | Pdxdc1 | intronic | . | . | . | 16qA1 | . | 0 | 1 |
| chr16 | 14232457 | C | T | 107 | Myh11 | intronic | . | . | . | 16qA1 | . | 0 | 1 |
| chr16 | 14233397 | T | C | 161 | Myh11 | intronic | . | . | . | 16qA1 | rs48122048 | 1 | 0 |
| chr16 | 14906574 | G | T | 37.1918 | . | upstream | . | . | . | 16qA1 | . | 0 | 1 |
| chr16 | 14916781 | T | A | 147 | Efcab1 | intronic | . | . | . | 16qA1 | . | 0 | 1 |
| chr16 | 16314431 | G | A | 221 | Dnm1l | intronic | . | . | . | 16qA2 | . | 0 | 1 |
| chr16 | 17153551 | A | C | 221 | Ube2l3 | UTR3 | . | . | . | 16qA3 | . | 0 | 1 |
| chr16 | 17499584 | A | G | 173 | Aifm3 | exonic | missense SNV | Aifm3:NM_001291070:exon2:c.A68G:p.E23G,Aifm3:NM_175178:exon4:c.A68G:p.E23G | . | 16qA3 | . | 0 | 1 |
| chr16 | 18076140 | C | A | 147 | Prodh | intronic | . | . | . | 16qA3 | . | 0 | 1 |
| chr16 | 18620804 | G | A | 164 | Gp1bb | exonic | missense SNV | Gp1bb:NM_001001999:exon1:c.C563T:p.A188V,Gp1bb:NM_010327:exon2:c.C539T:p.A180V | 1072 | 16qA3 | . | 0 | 1 |
| chr16 | 18624048 | C | T | 62 | 5-9月 | intronic | . | . | . | 16qA3 | . | 0 | 1 |
| chr16 | 19486977 | C | G | 221 | Olfr166 | exonic | missense SNV | Olfr166:NM_147068:exon1:c.C138G:p.H46Q | . | 16qA3 | . | 0 | 1 |
| chr16 | 20231952 | A | C | 222 | Yeats2 | UTR3 | . | . | . | 16qA3 | . | 0 | 1 |
| chr16 | 20369215 | G | T | 158 | Abcc5 | intronic | . | . | . | 16qA3 | . | 0 | 1 |
| chr16 | 20655380 | G | T | 222 | Psmd2 | exonic | missense SNV | Psmd2:NM_134101:exon6:c.G832T:p.V278L | . | 16qB1 | . | 0 | 1 |
| chr16 | 20656834 | A | G | 222 | Psmd2 | intronic | . | . | . | 16qB1 | . | 0 | 1 |
| chr16 | 21220350 | G | T | 168 | Ephb3 | intronic | . | . | . | 16qB1 | . | 0 | 1 |
| chr16 | 21220351 | G | T | 166 | Ephb3 | intronic | . | . | . | 16qB1 | . | 0 | 1 |
| chr16 | 21221156 | G | A | 157 | Ephb3 | intronic | . | . | . | 16qB1 | . | 0 | 1 |
| chr16 | 22079325 | C | A | 132 | Igf2bp2 | intronic | . | . | . | 16qB1 | . | 0 | 1 |
| chr16 | 22244349 | C | G | 222 | . | intergenic | . | . | . | 16qB1 | . | 0 | 1 |
| chr16 | 22869164 | G | C | 222 | Dnajb11 | exonic | missense SNV | Dnajb11:NM_001190804:exon6:c.G643C:p.G215R,Dnajb11:NM_001190805:exon6:c.G643C:p.G215R,Dnajb11 | . | 16qB1 | . | 0 | 1 |
| chr16 | 22896626 | C | A | 221 | Ahsg | intronic | . | . | . | 16qB1 | . | 0 | 1 |
| chr16 | 23429241 | G | A | 222 | Rtp1 | exonic | synonymous SNV | Rtp1:NM_001004151:exon1:c.G63A:p.T21T | . | 16qB1 | . | 0 | 1 |
| chr16 | 25174789 | G | A | 80 | . | intergenic | . | . | . | 16qB1 | . | 0 | 1 |
| chr16 | 26473935 | C | T | 25.7522 | Cldn16 | intronic | . | . | . | 16qB2 | rs256316425 | 0 | 1 |
| chr16 | 28827930 | G | A | 221 | Mb21d2 | exonic | missense SNV | Mb21d2:NM_177718:exon2:c.C1291T:p.R431W | . | 16qB2 | . | 0 | 1 |
| chr16 | 29263258 | C | G | 127 | Atp13a5 | intronic | . | . | . | 16qB2 | . | 0 | 1 |
| chr16 | 29263578 | C | T | 99 | Atp13a5 | intronic | . | . | . | 16qB2 | . | 0 | 1 |
| chr16 | 29263581 | C | G | 93 | Atp13a5 | intronic | . | . | . | 16qB2 | . | 0 | 1 |
| chr16 | 29277590 | G | T | 222 | Atp13a5 | intronic | . | . | . | 16qB2 | . | 0 | 1 |
| chr16 | 29413482 | C | A | 46.7978 | Atp13a4 | intronic | . | . | . | 16qB2 | . | 0 | 1 |
| chr16 | 29413484 | C | A | 46.7978 | Atp13a4 | intronic | . | . | . | 16qB2 | . | 0 | 1 |
| chr16 | 29418749 | C | A | 112 | Atp13a4 | intronic | . | . | . | 16qB2 | . | 0 | 1 |
| chr16 | 29472092 | T | C | 199 | Atp13a4 | intronic | . | . | . | 16qB2 | . | 0 | 1 |
| chr16 | 30199905 | T | G | 146 | . | intergenic | . | . | . | 16qB2 | . | 0 | 1 |
| chr16 | 30321925 | A | T | 221 | Atp13a3 | intronic | . | . | . | 16qB2 | . | 0 | 1 |
| chr16 | 30582582 | C | T | 221 | Lsg1 | exonic | missense SNV | Lsg1:NM_178069:exon3:c.G296A:p.R99K | . | 16qB2 | . | 0 | 1 |
| chr16 | 30601753 | G | C | 194 | Fam43a | exonic | missense SNV | Fam43a:NM_177632:exon1:c.G1154C:p.G385A | . | 16qB2 | . | 0 | 1 |
| chr16 | 31888124 | T | C | 222 | Mfi2 | exonic | synonymous SNV | Mfi2:NM_013900:exon8:c.T915C:p.H305H | . | 16qB2 | rs46174544 | 0 | 1 |
| chr16 | 32332440 | G | C | 228 | Ubxn7 | intronic | . | . | 863 | 16qB3 | rs46286023 | 1 | 0 |
| chr16 | 32451615 | T | G | 156 | Pcyt1a | intronic | . | . | . | 16qB3 | rs257082658 | 0 | 1 |
| chr16 | 32506425 | C | A | 222 | Zdhhc19 | exonic | missense SNV | Zdhhc19:NM_199309:exon8:c.C848A:p.P283Q | . | 16qB3 | . | 0 | 1 |
| chr16 | 32591443 | T | C | 210 | . | intergenic | . | . | . | 16qB3 | . | 0 | 1 |

| | | | | | | | | | | | | | |
|---|---|---|---|---|---|---|---|---|---|---|---|---|---|
| chr16 | 32591653 | G | T | 221 | . | intergenic | . | . | . | 16qB3 | . | 0 | 1 |
| chr16 | 32752446 | A | G | 45.0822 | Muc4 | exonic | missense SNV | Muc4:NM_080457:exon2:c.A2323G:p.R775G | . | 16qB3 | . | 0 | 1 |
| chr16 | 32752449 | C | T | 64 | Muc4 | exonic | missense SNV | Muc4:NM_080457:exon2:c.C2326T:p.H776Y | . | 16qB3 | . | 0 | 1 |
| chr16 | 32752456 | T | C | 146 | Muc4 | exonic | missense SNV | Muc4:NM_080457:exon2:c.T2333C:p.I778T | . | 16qB3 | rs581186642 | 0 | 1 |
| chr16 | 32752533 | A | G | 228 | Muc4 | exonic | missense SNV | Muc4:NM_080457:exon2:c.A2410G:p.T804A | . | 16qB3 | rs225618688 | 1 | 0 |
| chr16 | 32752889 | C | T | 87 | Muc4 | exonic | synonymous SNV | Muc4:NM_080457:exon2:c.C2766T:p.N922N | . | 16qB3 | rs586419889 | 0 | 1 |
| chr16 | 32752905 | G | A | 25.5481 | Muc4 | exonic | missense SNV | Muc4:NM_080457:exon2:c.G2782A:p.A928T | . | 16qB3 | . | 0 | 1 |
| chr16 | 32753802 | T | C | 127 | Muc4 | exonic | missense SNV | Muc4:NM_080457:exon3:c.T3677C:p.I1226T | . | 16qB3 | . | 0 | 1 |
| chr16 | 32754333 | T | A | 228 | Muc4 | exonic | synonymous SNV | Muc4:NM_080457:exon4:c.T4206A:p.A1402A | . | 16qB3 | rs47751160 | 1 | 0 |
| chr16 | 32754529 | C | A | 173 | Muc4 | exonic | missense SNV | Muc4:NM_080457:exon4:c.C4402A:p.H1468N | . | 16qB3 | . | 0 | 1 |
| chr16 | 32754535 | C | T | 86 | Muc4 | exonic | missense SNV | Muc4:NM_080457:exon4:c.C4408T:p.P1470S | . | 16qB3 | . | 0 | 1 |
| chr16 | 32754885 | C | T | 80 | Muc4 | exonic | synonymous SNV | Muc4:NM_080457:exon4:c.C4758T:p.G1586G | . | 16qB3 | rs580697147 | 0 | 1 |
| chr16 | 32755053 | C | A | 65 | Muc4 | exonic | synonymous SNV | Muc4:NM_080457:exon4:c.C4926A:p.S1642S | . | 16qB3 | . | 0 | 1 |
| chr16 | 32755059 | C | T | 51 | Muc4 | exonic | synonymous SNV | Muc4:NM_080457:exon4:c.C4932T:p.N1644N | . | 16qB3 | rs48965780 | 0 | 1 |
| chr16 | 32755148 | C | G | 137 | Muc4 | exonic | missense SNV | Muc4:NM_080457:exon4:c.C5021G:p.T1674S | . | 16qB3 | rs586118638 | 0 | 1 |
| chr16 | 32755717 | G | C | 228 | Muc4 | exonic | missense SNV | Muc4:NM_080457:exon4:c.G5590C:p.E1864Q | . | 16qB3 | rs47254141 | 1 | 0 |
| chr16 | 32778812 | A | G | 221 | Muc4,Muc20 | exonic | missense SNV | Muc4:NM_080457:exon24:c.A9811G:p.I3271V | . | 16qB3 | . | 0 | 1 |
| chr16 | 33080887 | A | G | 167 | Lmln | intronic | . | . | . | 16qB3 | . | 0 | 1 |
| chr16 | 34212635 | C | G | 55 | Kalrn | intronic | . | . | . | 16qB3 | . | 0 | 1 |
| chr16 | 34226928 | C | A | 212 | Kalrn | intronic | . | . | . | 16qB3 | . | 0 | 1 |
| chr16 | 34709406 | C | T | 114 | Ccdc14 | intronic | . | . | . | 16qB3 | . | 0 | 1 |
| chr16 | 34938757 | A | T | 121 | 1700119H24Rik,Mvl | ncRNA intronic | . | . | . | 16qB3 | . | 0 | 1 |
| chr16 | 34938758 | G | T | 125 | 1700119H24Rik,Mvl | ncRNA intronic | . | . | . | 16qB3 | . | 0 | 1 |
| chr16 | 35253319 | T | G | 222 | Adcy5 | intronic | . | . | . | 16qB3 | . | 0 | 1 |
| chr16 | 35291544 | G | A | 124 | Adcy5 | exonic | missense SNV | Adcy5:NM_001012765:exon15:c.G2770A:p.V924M | . | 16qB3 | rs258334795 | 0 | 1 |
| chr16 | 35622748 | C | G | 222 | Sema5b | exonic | missense SNV | Sema5b:NM_013661:exon2:c.C122G:p.T41R | . | 16qB3 | . | 0 | 1 |
| chr16 | 35662428 | G | C | 193 | Sema5b | intronic | . | . | . | 16qB3 | . | 0 | 1 |
| chr16 | 35719251 | A | G | 221 | Dirc2 | intronic | . | . | . | 16qB3 | rs52693926 | 0 | 1 |
| chr16 | 36455060 | T | C | 23.0118 | Stfa3 | intronic | . | . | . | 16qB3 | . | 0 | 1 |
| chr16 | 36455358 | G | A | 221 | Stfa3 | exonic | stopgain | Stfa3:NM_025288:exon1:c.C7T:p.Q3X | . | 16qB3 | . | 0 | 1 |
| chr16 | 36722601 | G | T | 222 | Ildr1 | exonic | missense SNV | Ildr1:NM_001285791:exon6:c.G1362T:p.E454D,Ildr1:NM_001285788:exon7:c.G1494T:p.E498D,Ildr1:NM | . | 16qB3 | . | 0 | 1 |
| chr16 | 36752935 | A | C | 167 | Slc15a2 | intronic | . | . | . | 16qB3 | . | 0 | 1 |
| chr16 | 36772445 | A | G | 228 | Slc15a2 | exonic | missense SNV | Slc15a2:NM_001145899:exon6:c.T629C:p.M210T,Slc15a2:NM_021301:exon6:c.T629C:p.M210T | . | 16qB3 | rs50751634 | 1 | 0 |
| chr16 | 36772472 | G | T | 221 | Slc15a2 | exonic | missense SNV | Slc15a2:NM_001145899:exon6:c.C602A:p.S201Y,Slc15a2:NM_021301:exon6:c.C602A:p.S201Y | . | 16qB3 | . | 0 | 1 |
| chr16 | 36843254 | A | C | 98 | Iqcb1,Eaf2 | intronic | . | . | . | 16qB3 | . | 0 | 1 |
| chr16 | 36847771 | G | C | 122 | Iqcb1,Eaf2 | intronic | . | . | . | 16qB3 | . | 0 | 1 |
| chr16 | 36962754 | C | A | 221 | Hcls1 | UTR3 | . | . | . | 16qB3 | . | 0 | 1 |
| chr16 | 37092951 | A | T | 183 | Polq | exonic | missense SNV | Polq:NM_001159369:exon23:c.A6544T:p.S2182C,Polq:NM_029977:exon28:c.A7381T:p.S2461C | . | 16qB3 | . | 0 | 1 |
| chr16 | 37121955 | T | G | 205 | Stxbp5l | intronic | . | . | . | 16qB3 | . | 0 | 1 |
| chr16 | 37631611 | A | T | 127 | Hgd | intronic | . | . | . | 16qB3 | . | 0 | 1 |
| chr16 | 37815818 | C | T | 221 | Fstl1 | intronic | . | . | . | 16qB3 | . | 0 | 1 |
| chr16 | 38184475 | C | T | 221 | Gsk3b | exonic | missense SNV | Gsk3b:NM_019827:exon4:c.C407T:p.P136L | . | 16qB3 | . | 0 | 1 |
| chr16 | 38252754 | A | G | 228 | Nr1i2 | intronic | . | . | . | 16qB3 | rs4174481 | 1 | 0 |
| chr16 | 38310929 | C | A | 221 | Maats1 | intronic | . | . | . | 16qB3 | . | 0 | 1 |
| chr16 | 38712556 | G | A | 21.7264 | Arhgap31 | intronic | . | . | . | 16qB4 | . | 0 | 1 |
| chr16 | 38828915 | C | T | 74 | . | upstream | . | . | . | 16qB4 | . | 0 | 1 |
| chr16 | 40071571 | A | T | 98 | . | intergenic | . | . | . | 16qB4 | . | 0 | 1 |
| chr16 | 44370861 | G | C | 221 | Spice1 | exonic | synonymous SNV | Spice1:NM_144550:exon10:c.G1077C:p.T359T | . | 16qB4 | . | 0 | 1 |
| chr16 | 44419224 | C | T | 221 | Cfap44 | exonic | missense SNV | Cfap44:NM_001033247:exon14:c.C1633T:p.R545W | . | 16qB4 | . | 0 | 1 |
| chr16 | 44500443 | T | C | 222 | Boc | exonic | missense SNV | Boc:NM_172506:exon6:c.A853G:p.S285G | . | 16qB4 | . | 0 | 1 |
| chr16 | 44501852 | G | C | 221 | Boc | intronic | . | . | . | 16qB4 | . | 0 | 1 |
| chr16 | 45392524 | C | T | 44.1949 | Cd200 | intronic | . | . | . | 16qB5 | . | 0 | 1 |
| chr16 | 46154710 | G | T | 221 | Gm4737 | exonic | synonymous SNV | Gm4737:NM_001304528:exon1:c.C303A:p.A101A | . | 16qB5 | . | 0 | 1 |
| chr16 | 48412183 | T | A | 100 | Gm5485 | ncRNA intronic | . | . | . | 16qB5 | . | 0 | 1 |
| chr16 | 48514467 | A | C | 51 | Morc1 | intronic | . | . | . | 16qB5 | . | 0 | 1 |
| chr16 | 48740367 | C | G | 113 | Trat1 | intronic | . | . | . | 16qB5 | . | 0 | 1 |
| chr16 | 49369854 | G | A | 177 | . | intergenic | . | . | . | 16qB5 | . | 0 | 1 |
| chr16 | 49412498 | A | C | 222 | . | intergenic | . | . | . | 16qB5 | . | 0 | 1 |
| chr16 | 49884207 | T | C | 195 | Cd47 | exonic | missense SNV | Cd47:NM_010581:exon4:c.T500C:p.F167S | . | 16qB5 | . | 0 | 1 |
| chr16 | 50111189 | C | A | 222 | . | intergenic | . | . | . | 16qB5 | . | 0 | 1 |
| chr16 | 52288888 | G | T | 221 | Alcam | intronic | . | . | . | 16qB5 | . | 0 | 1 |
| chr16 | 52291066 | C | A | 222 | Alcam | intronic | . | . | . | 16qB5 | . | 0 | 1 |
| chr16 | 53984931 | A | G | 221 | . | intergenic | . | . | . | 16qC1.1 | . | 0 | 1 |
| chr16 | 53985438 | A | C | 94 | . | intergenic | . | . | . | 16qC1.1 | . | 0 | 1 |
| chr16 | 53985969 | A | C | 221 | . | intergenic | . | . | . | 16qC1.1 | . | 0 | 1 |
| chr16 | 55865709 | A | G | 228 | Nxpe3 | intronic | . | . | . | 16qC1.1 | rs107935613 | 1 | 0 |
| chr16 | 56588744 | T | A | 222 | Abi3bp | splicing | . | . | . | 16qC1.1 | . | 0 | 1 |
| chr16 | 59050538 | C | T | 189 | . | intergenic | . | . | . | 16qC1.3 | . | 0 | 1 |
| chr16 | 59345216 | A | C | 222 | Olfr206 | exonic | missense SNV | Olfr206:NM_146991:exon1:c.T484G:p.L162V | . | 16qC1.3 | . | 0 | 1 |
| chr16 | 59915956 | T | C | 222 | Epha6 | exonic | synonymous SNV | Epha6:NM_007938:exon11:c.A2214G:p.E738E | . | 16qC1.3 | . | 0 | 1 |
| chr16 | 63546008 | G | A | 119 | Epha3 | UTR3 | . | . | . | 16qC1.3 | . | 0 | 1 |
| chr16 | 63546276 | G | A | 222 | Epha3 | intronic | . | . | . | 16qC1.3 | . | 0 | 1 |
| chr16 | 63611044 | A | G | 222 | Epha3 | exonic | missense SNV | Epha3:NM_010140:exon7:c.T1496C:p.L499P | . | 16qC1.3 | . | 0 | 1 |
| chr16 | 63612745 | T | G | 155 | Epha3 | intronic | . | . | . | 16qC1.3 | . | 0 | 1 |
| chr16 | 64858581 | A | G | 198 | Cggbp1 | UTR3 | . | . | . | 16qC1.3 | . | 0 | 1 |
| chr16 | 64859009 | G | A | 221 | Cggbp1 | UTR3 | . | . | . | 16qC1.3 | . | 0 | 1 |
| chr16 | 64918103 | C | T | 209 | . | intergenic | . | . | . | 16qC1.3 | . | 0 | 1 |
| chr16 | 64918356 | C | G | 221 | . | intergenic | . | . | . | 16qC1.3 | . | 0 | 1 |
| chr16 | 64918357 | G | A | 221 | . | intergenic | . | . | . | 16qC1.3 | . | 0 | 1 |
| chr16 | 65523467 | C | T | 173 | Pou1f1 | intronic | . | . | . | 16qC1.3 | . | 0 | 1 |
| chr16 | 66747273 | T | C | 222 | Cadm2 | exonic | missense SNV | Cadm2:NM_001145977:exon7:c.A869G:p.D290G,Cadm2:NM_178721:exon8:c.A896G:p.D299G | . | 16qC1.3 | . | 0 | 1 |
| chr16 | 66771814 | G | T | 80 | Cadm2 | intronic | . | . | . | 16qC1.3 | rs107931692 | 0 | 1 |
| chr16 | 70568804 | T | G | 118 | Gbe1 | intronic | . | . | . | 16qC2 | . | 0 | 1 |
| chr16 | 72970099 | T | C | 182 | Robo1 | intronic | . | . | . | 16qC3.1 | . | 0 | 1 |
| chr16 | 72970348 | C | T | 222 | Robo1 | exonic | synonymous SNV | Robo1:NM_019413:exon9:c.C1407T:p.G469G | . | 16qC3.1 | . | 0 | 1 |
| chr16 | 72977599 | G | A | 206 | Robo1 | intronic | . | . | . | 16qC3.1 | . | 0 | 1 |
| chr16 | 73035973 | G | A | 179 | Robo1 | intronic | . | . | . | 16qC3.1 | . | 0 | 1 |
| chr16 | 74016068 | C | T | 163 | Robo2 | intronic | . | . | . | 16qC3.1 | . | 0 | 1 |
| chr16 | 78359054 | G | A | 110 | Cxadr | intronic | . | . | . | 16qC3.1 | . | 0 | 1 |
| chr16 | 79090850 | G | A | 222 | Tmprss15 | exonic | missense SNV | Tmprss15:NM_008941:exon1:c.C68T:p.A23V,Tmprss15:NM_178855:exon1:c.C68T:p.A23V | . | 16qC3.2 | . | 0 | 1 |
| chr16 | 81483434 | G | A | 177 | Ncam2 | intronic | . | . | . | 16qC3.3 | . | 0 | 1 |
| chr16 | 84730777 | T | A | 221 | Mrpl39 | intronic | . | . | . | 16qC3.3 | . | 0 | 1 |
| chr16 | 84809476 | A | C | 222 | Jam2 | exonic | missense SNV | Jam2:NM_023844:exon4:c.A377C:p.K126T | . | 16qC3.3 | rs48761727 | 0 | 1 |
| chr16 | 84809524 | C | T | 222 | Jam2 | intronic | . | . | . | 16qC3.3 | rs51892012 | 0 | 1 |
| chr16 | 87458520 | C | G | 112 | Usp16 | intronic | . | . | . | 16qC3.3 | rs261428282 | 0 | 1 |
| chr16 | 87940637 | A | G | 221 | Grik1 | exonic | missense SNV | Grik1:NM_010348:exon12:c.T1768C:p.Y590H,Grik1:NM_146072:exon12:c.T1768C:p.Y590H | . | 16qC3.3 | . | 0 | 1 |
| chr16 | 87940785 | T | A | 38.9797 | Grik1 | intronic | . | . | . | 16qC3.3 | . | 0 | 1 |
| chr16 | 88289696 | G | A | 38.3136 | Grik1 | exonic | missense SNV | Grik1:NM_010348:exon1:c.C7T:p.R3C,Grik1:NM_146072:exon1:c.C7T:p.R3C | 429 | 16qC3.3 | . | 0 | 1 |
| chr16 | 88874252 | T | C | 114 | Krtap19-2 | exonic | missense SNV | Krtap19-2:NM_130870:exon1:c.A4G:p.S2G | . | 16qC3.3 | . | 0 | 1 |
| chr16 | 89047906 | T | G | 222 | . | upstream | . | . | . | 16qC3.3 | . | 0 | 1 |
| chr16 | 89396662 | A | T | 194 | . | intergenic | . | . | . | 16qC3.3 | . | 0 | 1 |
| chr16 | 89804813 | T | G | 221 | Tiam1 | splicing | . | . | . | 16qC3.3 | . | 0 | 1 |
| chr16 | 89884952 | C | A | 190 | Tiam1 | exonic | synonymous SNV | Tiam1:NM_001145886:exon5:c.G1137T:p.A379A,Tiam1:NM_009384:exon5:c.G1137T:p.A379A | 460 | 16qC3.3 | . | 0 | 1 |
| chr16 | 90753561 | C | G | 190 | Urb1 | exonic | missense SNV | Urb1:NM_029497:exon38:c.G6402C:p.Q2134H | . | 16qC3.3 | . | 0 | 1 |
| chr16 | 90915204 | G | A | 165 | . | intergenic | . | . | . | 16qC3.3 | . | 0 | 1 |
| chr16 | 91539585 | G | A | 221 | . | intergenic | . | . | . | 16qC3.3 | . | 0 | 1 |
| chr16 | 93585380 | C | T | 221 | Setd4 | intronic | . | . | . | 16qC4 | . | 0 | 1 |
| chr16 | 93844174 | C | G | 118 | Morc3 | intronic | . | . | . | 16qC4 | . | 0 | 1 |
| chr16 | 94426673 | G | C | 149 | Ttc3 | intronic | . | . | . | 16qC4 | . | 0 | 1 |
| chr16 | 95102514 | T | G | 188 | . | intergenic | . | . | . | 16qC4 | . | 0 | 1 |
| chr16 | 97408426 | A | G | 221 | Bace2 | exonic | missense SNV | Bace2:NM_019517:exon3:c.A539G:p.E180G | . | 16qC4 | . | 0 | 1 |
| chr16 | 97747214 | G | T | 186 | Ripk4 | intronic | . | . | . | 16qC4 | . | 0 | 1 |
| chr16 | 97748190 | G | T | 222 | Ripk4 | intronic | . | . | . | 16qC4 | . | 0 | 1 |
| chr16 | 97922212 | T | A | 60 | C2cd2 | exonic | missense SNV | C2cd2:NM_174847:exon1:c.A104T:p.K35M | 666 | 16qC4 | . | 0 | 1 |
| chr17 | 3190165 | C | A | 41.8001 | Scaf8 | exonic | missense SNV | Scaf8:NM_134123:exon16:c.C1811A:p.A604D | . | 17qA1 | . | 0 | 1 |
| chr17 | 3555059 | C | G | 154 | Tfb1m | exonic | missense SNV | Tfb1m:NM_146074:exon2:c.G158C:p.S53T | . | 17qA1 | . | 0 | 1 |
| chr17 | 3665647 | C | A | 106 | Nox3 | intronic | . | . | . | 17qA1 | . | 0 | 1 |
| chr17 | 5336858 | G | T | 213 | Arid1b | exonic | missense SNV | Arid1b:NM_001085355:exon18:c.G4429T:p.G1477C | . | 17qA1 | . | 0 | 1 |
| chr17 | 5753113 | G | A | 186 | Zdhhc14 | UTR3 | . | . | . | 17qA1 | . | 0 | 1 |
| chr17 | 8173039 | A | G | 145 | Fgfr1op | intronic | . | . | . | 17qA1 | . | 0 | 1 |
| chr17 | 8396929 | G | A | 104 | T2 | intronic | . | . | . | 17qA1 | . | 0 | 1 |
| chr17 | 8438530 | T | C | 59 | T | intronic | . | . | . | 17qA1 | . | 0 | 1 |
| chr17 | 8850256 | G | A | 125 | Pde10a | intronic | . | . | . | 17qA1 | . | 0 | 1 |
| chr17 | 8949401 | G | T | 221 | Pde10a | exonic | missense SNV | Pde10a:NM_011866:exon11:c.G907T:p.D303Y,Pde10a:NM_001290707:exon12:c.G1069T:p.D357Y | . | 17qA1 | . | 0 | 1 |
| chr17 | 8997205 | G | C | 108 | 1700010I14Rik | intronic | . | . | . | 17qA1 | . | 0 | 1 |
| chr17 | 9007720 | C | G | 124 | 1700010I14Rik | intronic | . | . | . | 17qA1 | . | 0 | 1 |
| chr17 | 9008244 | G | A | 124 | 1700010I14Rik | UTR3 | . | . | . | 17qA1 | . | 0 | 1 |
| chr17 | 9323118 | T | A | 157 | . | intergenic | . | . | . | 17qA1 | . | 0 | 1 |
| chr17 | 10597385 | C | G | 228 | Pacrg | intronic | . | . | . | 17qA1 | rs108311698 | 1 | 0 |
| chr17 | 12418936 | C | T | 178 | Plg | intronic | . | . | . | 17qA1 | . | 0 | 1 |
| chr17 | 12960747 | T | C | 32.6139 | . | upstream | . | . | . | 17qA1 | . | 0 | 1 |
| chr17 | 12960748 | G | T | 34.7483 | . | upstream | . | . | . | 17qA1 | . | 0 | 1 |
| chr17 | 12975400 | T | C | 222 | Wtap | exonic | synonymous SNV | Wtap:NM_001113532:exon6:c.A348G:p.A116A,Wtap:NM_001113533:exon6:c.A348G:p.A116A,Wtap:NM_17539 | . | 17qA1 | rs29539522 | 0 | 1 |
| chr17 | 15730967 | A | T | 191 | Chd1 | exonic | missense SNV | Chd1:NM_007690:exon7:c.A803T:p.E268V | 234 | 17qA2 | . | 0 | 1 |
| chr17 | 15739622 | A | G | 185 | Chd1 | exonic | missense SNV | Chd1:NM_007690:exon15:c.A2102G:p.E701G | . | 17qA2 | . | 0 | 1 |
| chr17 | 17457376 | C | A | 64 | Lix1 | exonic | missense SNV | Lix1:NM_025681:exon6:c.C832A:p.L278M | . | 17qA3.2 | . | 0 | 1 |
| chr17 | 17500935 | G | A | 185 | . | intergenic | . | . | . | 17qA3.2 | . | 0 | 1 |
| chr17 | 17657916 | G | A | 191 | . | intergenic | . | . | . | 17qA3.2 | . | 0 | 1 |
| chr17 | 19065100 | T | G | 87 | Vmn2r98 | intronic | . | . | . | 17qA3.2 | . | 0 | 1 |
| chr17 | 19277367 | G | T | 96 | . | intergenic | . | . | . | 17qA3.2 | . | 0 | 1 |
| chr17 | 19394053 | C | G | 144 | Vmn2r99 | exonic | synonymous SNV | Vmn2r99:NM_001104551:exon6:c.C2034G:p.V678V | . | 17qA3.2 | . | 0 | 1 |
| chr17 | 19521990 | A | G | 221 | Vmn2r100 | exonic | missense SNV | Vmn2r100:NM_001104562:exon3:c.A625G:p.M209V | . | 17qA3.2 | . | 0 | 1 |
| chr17 | 19868617 | C | G | 83 | . | intergenic | . | . | . | 17qA3.2 | . | 0 | 1 |
| chr17 | 20029712 | A | C | 109 | Vmn2r104 | exonic | missense SNV | Vmn2r104:NM_001104566:exon6:c.T2296G:p.F766V | . | 17qA3.2 | . | 0 | 1 |
| chr17 | 20113998 | T | A | 191 | . | intergenic | . | . | . | 17qA3.2 | . | 0 | 1 |
| chr17 | 20183144 | G | A | 221 | . | upstream | . | . | . | 17qA3.2 | . | 0 | 1 |
| chr17 | 20209167 | A | G | 56 | Vmn2r105 | intronic | . | . | . | 17qA3.2 | . | 0 | 1 |
| chr17 | 20574192 | G | T | 158 | Vmn2r110 | exonic | missense SNV | Vmn2r110:NM_001104572:exon6:c.C2214A:p.N738K | . | 17qA3.2 | . | 0 | 1 |
| chr17 | 20814424 | C | T | 73 | . | upstream | . | . | . | 17qA3.2 | . | 0 | 1 |
| chr17 | 20960888 | A | G | 106 | Ppp2r1a | intronic | . | . | . | 17qA3.2 | . | 0 | 1 |
| chr17 | 21287694 | A | C | 221 | . | downstream | . | . | . | 17qA3.2 | . | 0 | 1 |
| chr17 | 21456329 | G | A | 164 | Zfp51 | exonic | missense SNV | Zfp51:NM_001308323:exon3:c.G61A:p.E21K,Zfp51:NM_009558:exon3:c.G61A:p.E21K | . | 17qA3.2 | . | 0 | 1 |
| chr17 | 23662096 | G | A | 222 | Ccdc64b | intronic | . | . | . | 17qA3.3 | . | 0 | 1 |
| chr17 | 23760851 | C | T | 115 | Flywch1 | exonic | synonymous SNV | Flywch1:NM_153791:exon4:c.G948A:p.R316R | . | 17qA3.3 | . | 0 | 1 |
| chr17 | 23761170 | C | G | 152 | Flywch1 | intronic | . | . | . | 17qA3.3 | . | 0 | 1 |
| chr17 | 24285848 | T | G | 193 | Abca17 | intronic | . | . | . | 17qA3.3 | . | 0 | 1 |
| chr17 | 24509238 | T | A | 52 | Traf7 | UTR3 | . | . | . | 17qA3.3 | . | 0 | 1 |
| chr17 | 24513995 | A | G | 222 | Traf7 | intronic | . | . | . | 17qA3.3 | rs33146442 | 0 | 1 |
| chr17 | 24630811 | C | A | 61 | Tsc2 | intronic | . | . | . | 17qA3.3 | . | 0 | 1 |
| chr17 | 24687842 | C | T | 145 | Syngr3 | intronic | . | . | . | 17qA3.3 | . | 0 | 1 |
| chr17 | 24945470 | C | T | 39.8295 | Hn1l | intronic | . | . | . | 17qA3.3 | . | 0 | 1 |
| chr17 | 25372602 | C | T | 221 | Tpsg1 | exonic | synonymous SNV | Tpsg1:NM_012034:exon2:c.C175T:p.L59L | . | 17qA3.3 | . | 0 | 1 |
| chr17 | 25375243 | T | A | 178 | Cacna1h | exonic | missense SNV | Cacna1h:NM_021415:exon35:c.A6940T:p.T2314S | . | 17qA3.3 | . | 0 | 1 |
| chr17 | 25721086 | T | C | 221 | Chtf18 | exonic | synonymous SNV | Chtf18:NM_145409:exon16:c.A2130G:p.R710R | . | 17qA3.3 | . | 0 | 1 |
| chr17 | 25845757 | C | A | 93 | Wdr90 | intronic | . | . | . | 17qA3.3 | . | 0 | 1 |
| chr17 | 26093882 | T | C | 221 | Nme4 | exonic | missense SNV | Nme4:NM_019731:exon3:c.A301G:p.S101G | . | 17qA3.3 | . | 0 | 1 |
| chr17 | 26098957 | A | G | 199 | . | intergenic | . | . | . | 17qA3.3 | rs583342138 | 0 | 1 |
| chr17 | 26099063 | T | C | 222 | . | intergenic | . | . | . | 17qA3.3 | rs580095245 | 0 | 1 |
| chr17 | 26099127 | C | T | 222 | . | intergenic | . | . | . | 17qA3.3 | rs584073073 | 0 | 1 |
| chr17 | 26192711 | T | C | 108 | Axin1 | intronic | . | . | . | 17qA3.3 | . | 0 | 1 |
| chr17 | 26762145 | A | G | 100 | Crebrf | intronic | . | . | . | 17qA3.3 | . | 0 | 1 |
| chr17 | 27035944 | G | A | 61 | Ggnbp1 | intronic | . | . | . | 17qA3.3 | . | 0 | 1 |
| chr17 | 27119540 | T | G | 80 | Itpr3 | intronic | . | . | . | 17qA3.3 | . | 0 | 1 |
| chr17 | 27438722 | G | T | 222 | Grm4 | exonic | synonymous SNV | Grm4:NM_001013385:exon6:c.C1309A:p.R437R,Grm4:NM_001291045:exon6:c.C1309A:p.R437R | . | 17qA3.3 | rs579343735 | 0 | 1 |
| chr17 | 27622526 | T | A | 150 | Nudt3 | intronic | . | . | . | 17qA3.3 | rs48751876 | 0 | 1 |
| chr17 | 27632447 | T | C | 30.9252 | Rps10 | intronic | . | . | . | 17qA3.3 | . | 0 | 1 |
| chr17 | 28662056 | A | C | 189 | Slc26a8 | intronic | . | . | . | 17qA3.3 | . | 0 | 1 |
| chr17 | 28908235 | C | G | 52 | 4930539E08Rik | intronic | . | . | . | 17qA3.3 | . | 0 | 1 |
| chr17 | 29038714 | T | C | 75 | Srsf3 | intronic | . | . | . | 17qA3.3 | . | 0 | 1 |
| chr17 | 29137171 | T | A | 109 | Rab44 | intronic | . | . | . | 17qA3.3 | . | 0 | 1 |
| chr17 | 29526500 | A | G | 222 | Tmem217 | exonic | synonymous SNV | Tmem217:NM_001162902:exon2:c.T255C:p.Y85Y,Tmem217:NM_001162901:exon3:c.T255C:p.Y85Y | . | 17qA3.3 | rs33442265 | 0 | 1 |
| chr17 | 29840042 | G | T | 129 | Mdga1 | intronic | . | . | . | 17qA3.3 | . | 0 | 1 |
| chr17 | 29850666 | C | T | 183 | Mdga1 | exonic | synonymous SNV | Mdga1:NM_001081160:exon4:c.G405A:p.T135T | . | 17qA3.3 | . | 0 | 1 |
| chr17 | 30649761 | A | T | 190 | Dnah8 | exonic | missense SNV | Dnah8:NM_013811:exon7:c.A1168T:p.M390L | . | 17qA3.3 | . | 0 | 1 |
| chr17 | 30677238 | G | T | 92 | Dnah8 | exonic | synonymous SNV | Dnah8:NM_013811:exon18:c.G2625T:p.L875L | . | 17qA3.3 | . | 0 | 1 |

| | | | | | | | | | | | | | |
|---|---|---|---|---|---|---|---|---|---|---|---|---|---|
| chr17 | 30683658 | T | A | 83 | Dnah8 | exonic | missense SNV | Dnah8:NM_013811:exon19:c.T2689A:p.C897S | . | 17qA3.3 | . | 0 | 1 |
| chr17 | 30724985 | C | T | 32.6968 | Dnah8 | intronic | . | . | . | 17qA3.3 | . | 0 | 1 |
| chr17 | 31197689 | C | A | 168 | Tmprss3 | exonic | synonymous SNV | Tmprss3:NM_080727:exon1:c.G6T:p.G2G,Tmprss3:NM_001163776:exon2:c.G72T:p.G24G | . | 17qA3.3 | . | 0 | 1 |
| chr17 | 31340823 | G | T | 88 | Slc37a1 | intronic | . | . | . | 17qA3.3 | . | 0 | 1 |
| chr17 | 31613372 | C | A | 102 | Cbs | intronic | . | . | . | 17qB1 | . | 0 | 1 |
| chr17 | 32198392 | C | T | 76 | Brd4 | exonic | synonymous SNV | Brd4:NM_001286630:exon18:c.G3858A:p.E1286E,Brd4:NM_020508:exon18:c.G3855A:p.E1285E | . | 17qB1 | . | 0 | 1 |
| chr17 | 32198405 | G | A | 103 | Brd4 | exonic | missense SNV | Brd4:NM_001286630:exon18:c.C3845T:p.A1282V,Brd4:NM_020508:exon18:c.C3842T:p.A1281V | . | 17qB1 | . | 0 | 1 |
| chr17 | 32201512 | G | C | 99 | Brd4 | exonic | missense SNV | Brd4:NM_001286630:exon15:c.C2671G:p.P891A,Brd4:NM_020508:exon15:c.C2668G:p.P890A | . | 17qB1 | . | 0 | 1 |
| chr17 | 32213153 | T | A | 129 | Brd4 | intronic | . | . | . | 17qB1 | . | 0 | 1 |
| chr17 | 32359413 | T | A | 190 | Wiz | exonic | missense SNV | Wiz:NM_212438:exon4:c.A1097T:p.H366L,Wiz:NM_011717:exon5:c.A1097T:p.H366L | . | 17qB1 | . | 0 | 1 |
| chr17 | 32403549 | C | T | 23.8931 | A530088E08Rik,Ras | ncRNA_exonic | . | . | . | 17qB1 | . | 0 | 1 |
| chr17 | 32674277 | C | T | 93 | Cyp4f40 | exonic | synonymous SNV | Cyp4f40:NM_001101588:exon8:c.C1081T:p.L361L | . | 17qB1 | . | 0 | 1 |
| chr17 | 32692313 | T | A | 56 | Cyp4f15 | intronic | . | . | . | 17qB1 | . | 0 | 1 |
| chr17 | 33136344 | T | C | 221 | Morc2b | exonic | missense SNV | Morc2b:NM_177719:exon2:c.A2453G:p.K818R | . | 17qB1 | . | 0 | 1 |
| chr17 | 33549402 | G | A | 164 | Adamts10 | intronic | . | . | . | 17qB1 | . | 0 | 1 |
| chr17 | 33585616 | C | T | 110 | Myo1f | exonic | synonymous SNV | Myo1f:NM_053214:exon13:c.C1293T:p.I431I | . | 17qB1 | . | 0 | 1 |
| chr17 | 33646516 | G | A | 149 | Hnrnpm | exonic | stopgain | Hnrnpm:NM_029804:exon16:c.C2170T:p.R724X,Hnrnpm:NM_001109913:exon17:c.C2053T:p.R685X | . | 17qB1 | . | 0 | 1 |
| chr17 | 33917189 | G | T | 221 | Zbtb22 | exonic | missense SNV | Zbtb22:NM_020625:exon2:c.G307T:p.G103C | . | 17qB1 | . | 0 | 1 |
| chr17 | 33918594 | T | C | 203 | Zbtb22 | exonic | missense SNV | Zbtb22:NM_020625:exon2:c.T1712C:p.V571A | . | 17qB1 | . | 0 | 1 |
| chr17 | 34056934 | G | C | 150 | Col11a2 | exonic | missense SNV | Col11a2:NM_009926:exon32:c.G2288C:p.R763P,Col11a2:NM_001317722:exon33:c.G2483C:p.R828P | . | 17qB1 | . | 0 | 1 |
| chr17 | 34057562 | C | A | 72 | Col11a2 | intronic | . | . | . | 17qB1 | . | 0 | 1 |
| chr17 | 34265053 | C | A | 148 | H2-Ab1 | intronic | . | . | 585 | 17qB1 | . | 0 | 1 |
| chr17 | 34267613 | G | T | 107 | H2-Ab1 | exonic | missense SNV | H2-Ab1:NM_207105:exon3:c.G645T:p.E215D | . | 17qB1 | . | 0 | 1 |
| chr17 | 34497448 | A | C | 129 | . | upstream | . | . | . | 17qB1 | . | 0 | 1 |
| chr17 | 34517434 | T | C | 100 | . | upstream | . | . | . | 17qB1 | . | 0 | 1 |
| chr17 | 35029117 | C | A | 116 | Msh5 | intronic | . | . | . | 17qB1 | . | 0 | 1 |
| chr17 | 35204666 | C | T | 120 | Lta | splicing | . | . | . | 17qB1 | . | 0 | 1 |
| chr17 | 35513141 | C | A | 89 | Tcf19 | UTR3 | . | . | . | 17qB1 | . | 0 | 1 |
| chr17 | 35568867 | T | A | 135 | 2300002M23Rik | UTR3 | . | . | . | 17qB1 | . | 0 | 1 |
| chr17 | 35637501 | G | A | 149 | Dpcr1 | exonic | missense SNV | Dpcr1:NM_001033366:exon2:c.C1205T:p.P402L | . | 17qB1 | . | 0 | 1 |
| chr17 | 35895622 | A | G | 104 | 2310061I04Rik | intronic | . | . | . | 17qB1 | . | 0 | 1 |
| chr17 | 35909567 | C | T | 56 | Atat1 | intronic | . | . | . | 17qB1 | . | 0 | 1 |
| chr17 | 36226071 | A | G | 83 | . | intergenic | . | . | . | 17qB1 | . | 0 | 1 |
| chr17 | 36269131 | T | C | 188 | Trim39 | intronic | . | . | . | 17qB1 | . | 0 | 1 |
| chr17 | 36672266 | C | A | 159 | . | upstream | . | . | . | 17qB1 | . | 0 | 1 |
| chr17 | 36965205 | A | G | 122 | Znrd1as | exonic | synonymous SNV | Znrd1as:NM_029602:exon3:c.A468G:p.E156E | . | 17qB1 | . | 0 | 1 |
| chr17 | 36978191 | G | A | 129 | 2410137M14Rik | intronic | . | . | . | 17qB1 | . | 0 | 1 |
| chr17 | 36979037 | G | A | 94 | 2410137M14Rik | intronic | . | . | . | 17qB1 | . | 0 | 1 |
| chr17 | 37270164 | C | G | 163 | . | upstream | . | . | . | 17qB1 | . | 0 | 1 |
| chr17 | 37313437 | T | G | 89 | Olfr102 | UTR3 | . | . | . | 17qB1 | . | 0 | 1 |
| chr17 | 37406505 | C | A | 221 | Olfr107 | UTR3 | . | . | . | 17qB1 | . | 0 | 1 |
| chr17 | 37499425 | C | A | 86 | Olfr110 | exonic | missense SNV | Olfr110:NM_146328:exon2:c.C773A:p.T258K | . | 17qB1 | . | 0 | 1 |
| chr17 | 37772432 | T | A | 163 | Olfr122 | exonic | missense SNV | Olfr122:NM_146288:exon2:c.T778A:p.S260T | . | 17qB1 | . | 0 | 1 |
| chr17 | 38067987 | T | A | 172 | Olfr130 | exonic | missense SNV | Olfr130:NM_146487:exon1:c.T815A:p.F272Y | . | 17qB1 | . | 0 | 1 |
| chr17 | 38082482 | A | G | 125 | Olfr131 | exonic | synonymous SNV | Olfr131:NM_146867:exon1:c.T495C:p.T165T | . | 17qB1 | . | 0 | 1 |
| chr17 | 38082669 | A | G | 221 | Olfr131 | exonic | missense SNV | Olfr131:NM_146867:exon1:c.T308C:p.L103P | . | 17qB1 | . | 0 | 1 |
| chr17 | 38082847 | T | A | 141 | Olfr131 | exonic | missense SNV | Olfr131:NM_146867:exon1:c.A130T:p.N44Y | . | 17qB1 | . | 0 | 1 |
| chr17 | 39843289 | C | T | 228 | Rn45s | ncRNA_exonic | . | . | 3178 | 17qB1 | rs108514044 | 1 | 0 |
| chr17 | 39843367 | G | C | 115 | Rn45s | ncRNA_exonic | . | . | 3178 | 17qB1 | rs51196648 | 0 | 1 |
| chr17 | 39843540 | C | G | 88 | Rn45s | ncRNA_exonic | . | . | 3178 | 17qB1 | rs8256199 | 0 | 1 |
| chr17 | 39843657 | T | G | 96 | Rn45s | ncRNA_exonic | . | . | 3178 | 17qB1 | rs108423881 | 0 | 1 |
| chr17 | 39843824 | C | G | 224 | Rn45s | ncRNA_exonic | . | . | 3178 | 17qB1 | rs108722263 | 1 | 0 |
| chr17 | 39843842 | T | C | 216 | Rn45s | ncRNA_exonic | . | . | 3178 | 17qB1 | rs108507302 | 1 | 0 |
| chr17 | 39844227 | A | G | 228 | Rn45s | ncRNA_exonic | . | . | 3178 | 17qB1 | rs108187150 | 1 | 0 |
| chr17 | 39844265 | C | A | 222 | Rn45s | ncRNA_exonic | . | . | 3178 | 17qB1 | rs13473251 | 0 | 1 |
| chr17 | 39844355 | G | C | 228 | Rn45s | ncRNA_exonic | . | . | 3178 | 17qB1 | rs108721547 | 1 | 0 |
| chr17 | 39844434 | G | A | 228 | Rn45s | ncRNA_exonic | . | . | 3178 | 17qB1 | rs108537318 | 1 | 0 |
| chr17 | 39844458 | T | C | 228 | Rn45s | ncRNA_exonic | . | . | 3178 | 17qB1 | rs108634309 | 1 | 0 |
| chr17 | 39844684 | C | G | 228 | Rn45s | ncRNA_exonic | . | . | 3178 | 17qB1 | rs107948916 | 1 | 0 |
| chr17 | 39844879 | T | C | 127 | Rn45s | ncRNA_exonic | . | . | 3178 | 17qB1 | rs244710099 | 0 | 1 |
| chr17 | 39844919 | T | C | 129 | Rn45s | ncRNA_exonic | . | . | 3178 | 17qB1 | rs108084908 | 0 | 1 |
| chr17 | 39845013 | T | G | 161 | Rn45s | ncRNA_exonic | . | . | 3178 | 17qB1 | . | 0 | 1 |
| chr17 | 39845049 | T | C | 228 | Rn45s | ncRNA_exonic | . | . | 3178 | 17qB1 | rs108225550 | 1 | 0 |
| chr17 | 39845219 | A | C | 228 | Rn45s | ncRNA_exonic | . | . | 3178 | 17qB1 | rs108846804 | 1 | 0 |
| chr17 | 39845224 | T | G | 228 | Rn45s | ncRNA_exonic | . | . | 3178 | 17qB1 | rs107674267 | 1 | 0 |
| chr17 | 39845580 | C | T | 228 | Rn45s | ncRNA_exonic | . | . | 3178 | 17qB1 | rs108374714 | 1 | 0 |
| chr17 | 39845719 | G | A | 119 | Rn45s | ncRNA_exonic | . | . | 3178 | 17qB1 | . | 0 | 1 |
| chr17 | 39845749 | A | C | 228 | Rn45s | ncRNA_exonic | . | . | 3178 | 17qB1 | rs108289589 | 1 | 0 |
| chr17 | 39845863 | C | T | 142 | Rn45s | ncRNA_exonic | . | . | 3178 | 17qB1 | . | 0 | 1 |
| chr17 | 39846004 | C | T | 106 | Rn45s | ncRNA_exonic | . | . | 3178 | 17qB1 | . | 0 | 1 |
| chr17 | 39846349 | C | T | 228 | Rn45s | ncRNA_exonic | . | . | . | 17qB1 | rs108093966 | 1 | 0 |
| chr17 | 39848233 | T | C | 215 | Rn45s | ncRNA_exonic | . | . | 603 | 17qB1 | rs107624959 | 0 | 1 |
| chr17 | 39848455 | G | T | 228 | Rn45s | ncRNA_exonic | . | . | 603 | 17qB1 | rs51883994 | 1 | 0 |
| chr17 | 39848652 | T | C | 178 | Rn45s | ncRNA_exonic | . | . | 603 | 17qB1 | rs107810024 | 0 | 1 |
| chr17 | 40786025 | C | T | 89 | Crisp2 | intronic | . | . | . | 17qB2 | . | 0 | 1 |
| chr17 | 40831127 | C | T | 55 | Rhag | intronic | . | . | . | 17qB2 | . | 0 | 1 |
| chr17 | 40880568 | A | G | 103 | . | upstream | . | . | . | 17qB2 | . | 0 | 1 |
| chr17 | 42834132 | C | T | 221 | Cd2ap | intronic | . | . | . | 17qB3 | rs33594712 | 0 | 1 |
| chr17 | 43038382 | G | A | 31.7231 | Tnfrsf21 | intronic | . | . | . | 17qB3 | . | 0 | 1 |
| chr17 | 43085421 | A | C | 154 | Tnfrsf21 | exonic | missense SNV | Tnfrsf21:NM_178589:exon5:c.A1595C:p.N532T | . | 17qB3 | . | 0 | 1 |
| chr17 | 43291102 | G | A | 176 | Adgrf1 | exonic | missense SNV | Adgrf1:NM_133776:exon3:c.G55A:p.D19N | . | 17qB3 | . | 0 | 1 |
| chr17 | 43293304 | G | T | 221 | Adgrf1 | intronic | . | . | . | 17qB3 | . | 0 | 1 |
| chr17 | 43440221 | C | T | 194 | Adgrf5 | intronic | . | . | . | 17qB3 | . | 0 | 1 |
| chr17 | 43440248 | A | G | 81 | Adgrf5 | intronic | . | . | . | 17qB3 | . | 0 | 1 |
| chr17 | 43478979 | G | C | 181 | Mep1a | exonic | missense SNV | Mep1a:NM_008585:exon11:c.C1478G:p.S493C | . | 17qB3 | . | 0 | 1 |
| chr17 | 45465856 | C | T | 136 | Spats1 | intronic | . | . | . | 17qB3 | . | 0 | 1 |
| chr17 | 45568996 | C | A | 130 | Hsp90ab1 | exonic | missense SNV | Hsp90ab1:NM_008302:exon9:c.G1593T:p.K531N | . | 17qB3 | . | 0 | 1 |
| chr17 | 46247959 | G | A | 68 | Polr1c | exonic | missense SNV | Polr1c:NM_009085:exon1:c.C5T:p.A2V | 659 | 17qC | . | 0 | 1 |
| chr17 | 46433797 | G | T | 69 | Slc22a7 | intronic | . | . | . | 17qC | . | 0 | 1 |
| chr17 | 46525062 | G | A | 134 | Cul9 | splicing | . | . | . | 17qC | . | 0 | 1 |
| chr17 | 46527928 | G | A | 113 | Cul9 | exonic | missense SNV | Cul9:NM_001081335:exon11:c.C2780T:p.S927L | . | 17qC | . | 0 | 1 |
| chr17 | 46653132 | C | T | 31.713 | Cul7 | exonic | synonymous SNV | Cul7:NM_025611:exon4:c.C1134T:p.Y378Y | 213 | 17qC | . | 0 | 1 |
| chr17 | 47016981 | G | A | 31.8881 | . | intergenic | . | . | . | 17qC | . | 0 | 1 |
| chr17 | 47353956 | T | C | 110 | Trerf1 | intronic | . | . | . | 17qC | . | 0 | 1 |
| chr17 | 47353958 | G | C | 110 | Trerf1 | intronic | . | . | . | 17qC | . | 0 | 1 |
| chr17 | 47400410 | T | C | 228 | Guca1a | exonic | missense SNV | Guca1a:NM_008189:exon1:c.A10G:p.I4V | . | 17qC | rs33079196 | 1 | 0 |
| chr17 | 47733741 | A | C | 219 | Pgc | intronic | . | . | . | 17qC | . | 0 | 1 |
| chr17 | 49096684 | T | G | 112 | Lrfn2 | exonic | missense SNV | Lrfn2:NM_027452:exon3:c.T1834G:p.F612V | . | 17qC | . | 0 | 1 |
| chr17 | 49463894 | C | A | 169 | Daam2 | intronic | . | . | . | 17qC | . | 0 | 1 |
| chr17 | 49904089 | T | A | 221 | Kif6 | intronic | . | . | . | 17qC | . | 0 | 1 |
| chr17 | 50288793 | A | T | 88 | Dazl | intronic | . | . | . | 17qC | . | 0 | 1 |
| chr17 | 53421913 | A | C | 216 | Efhb | exonic | missense SNV | Efhb:NM_172497:exon9:c.T1747G:p.F583V | . | 17qC | . | 0 | 1 |
| chr17 | 53831844 | T | C | 211 | Sult1c2 | exonic | missense SNV | Sult1c2:NM_026935:exon7:c.A704G:p.E235G | . | 17qC | . | 0 | 1 |
| chr17 | 53866660 | C | A | 165 | . | intergenic | . | . | . | 17qC | . | 0 | 1 |
| chr17 | 53969891 | T | C | 48.2414 | Sult1c1 | intronic | . | . | . | 17qC | . | 0 | 1 |
| chr17 | 54507634 | A | G | 42.0498 | . | intergenic | . | . | . | 17qC | . | 0 | 1 |
| chr17 | 55608051 | C | A | 190 | Vmn2r118 | exonic | stopgain | Vmn2r118:NM_001104582:exon5:c.G1594T:p.E532X | . | 17qC | . | 0 | 1 |
| chr17 | 56286584 | G | A | 174 | Plin3 | exonic | synonymous SNV | Plin3:NM_025836:exon3:c.C129T:p.S43S | . | 17qD | rs256863977 | 0 | 1 |
| chr17 | 56318358 | A | G | 160 | Uhrf1 | exonic | missense SNV | Uhrf1:NM_001111078:exon13:c.A1804G:p.T602A,Uhrf1:NM_001111079:exon13:c.A1780G:p.T594A,Uhrf1:N | . | 17qD | . | 0 | 1 |
| chr17 | 56511624 | C | A | 145 | Znrf4 | exonic | missense SNV | Znrf4:NM_011483:exon1:c.G682T:p.A228S | 1169 | 17qD | . | 0 | 1 |
| chr17 | 56619293 | C | A | 170 | Lonp1 | exonic | missense SNV | Lonp1:NM_028782:exon8:c.G1213T:p.A405S | . | 17qD | . | 0 | 1 |
| chr17 | 56861696 | G | T | 186 | Acsbg2 | intronic | . | . | . | 17qD | . | 0 | 1 |
| chr17 | 57024155 | C | T | 114 | Khsrp | exonic | synonymous SNV | Khsrp:NM_010613:exon13:c.G1347A:p.K449K | . | 17qD | . | 0 | 1 |
| chr17 | 57064974 | C | T | 37.9715 | Crb3 | intronic | . | . | . | 17qD | . | 0 | 1 |
| chr17 | 57219033 | C | A | 100 | C3 | intronic | . | . | . | 17qD | . | 0 | 1 |
| chr17 | 57406912 | C | T | 135 | Adgre1 | exonic | missense SNV | Adgre1:NM_010130:exon6:c.C596T:p.T199M | . | 17qD | . | 0 | 1 |
| chr17 | 57407090 | A | G | 26.7158 | Adgre1 | intronic | . | . | . | 17qD | . | 0 | 1 |
| chr17 | 57411013 | T | A | 158 | Adgre1 | exonic | missense SNV | Adgre1:NM_010130:exon9:c.T1024A:p.S342T | . | 17qD | . | 0 | 1 |
| chr17 | 57509545 | A | C | 207 | Vmn2r120 | exonic | synonymous SNV | Vmn2r120:NM_001104591:exon7:c.T1809G:p.S603S | . | 17qD | . | 0 | 1 |
| chr17 | 57521984 | T | C | 157 | Vmn2r120 | exonic | missense SNV | Vmn2r120:NM_001104591:exon6:c.A1610G:p.K537R | . | 17qD | . | 0 | 1 |
| chr17 | 57839114 | G | T | 122 | Cntnap5c | intronic | . | . | . | 17qD | . | 0 | 1 |
| chr17 | 58313811 | A | T | 221 | Cntnap5c | exonic | missense SNV | Cntnap5c:NM_001081653:exon18:c.A2884T:p.S962C | . | 17qD | . | 0 | 1 |
| chr17 | 58314048 | T | G | 113 | Cntnap5c | intronic | . | . | . | 17qD | . | 0 | 1 |
| chr17 | 58330391 | A | G | 182 | Cntnap5c | intronic | . | . | . | 17qD | . | 0 | 1 |
| chr17 | 58885774 | C | A | 46.0743 | Pdzph1 | intronic | . | . | . | 17qD | . | 0 | 1 |
| chr17 | 60491086 | A | C | 213 | . | intergenic | . | . | . | 17qE1.1 | rs243401119 | 0 | 1 |
| chr17 | 66555783 | G | A | 99 | Themis3 | exonic | synonymous SNV | Themis3:NM_028998:exon4:c.C1179T:p.H393H | . | 17qE1.1 | rs48710637 | 0 | 1 |
| chr17 | 66920266 | A | C | 98 | Ptprm | exonic | synonymous SNV | Ptprm:NM_008984:exon12:c.T1887G:p.R629R | . | 17qE1.1 | . | 0 | 1 |
| chr17 | 67716837 | T | G | 222 | Lama1 | exonic | missense SNV | Lama1:NM_008480:exon2:c.T226G:p.C76G | . | 17qE1.1 | . | 0 | 1 |
| chr17 | 67752883 | G | A | 228 | Lama1 | exonic | missense SNV | Lama1:NM_008480:exon14:c.G1966A:p.D656N | . | 17qE1.1 | rs33387924 | 1 | 0 |
| chr17 | 68461300 | T | G | 63 | L3mbtl4 | intronic | . | . | . | 17qE1.2 | . | 0 | 1 |
| chr17 | 69274908 | G | A | 222 | Epb4113 | intronic | . | . | . | 17qE1.3 | . | 0 | 1 |
| chr17 | 70875937 | C | T | 151 | . | intergenic | . | . | . | 17qE1.3 | rs49711941 | 0 | 1 |
| chr17 | 70926247 | T | A | 105 | . | intergenic | . | . | . | 17qE1.3 | rs49587537 | 0 | 1 |
| chr17 | 70929073 | C | G | 95 | . | intergenic | . | . | . | 17qE1.3 | rs48918194 | 0 | 1 |
| chr17 | 70929094 | A | G | 84 | . | intergenic | . | . | . | 17qE1.3 | rs51130961 | 0 | 1 |
| chr17 | 70929328 | A | G | 187 | . | intergenic | . | . | . | 17qE1.3 | rs46376673 | 0 | 1 |
| chr17 | 70929426 | G | A | 96 | . | intergenic | . | . | . | 17qE1.3 | rs33699695 | 0 | 1 |
| chr17 | 70929470 | G | T | 80 | . | intergenic | . | . | . | 17qE1.3 | rs33180221 | 0 | 1 |
| chr17 | 70975523 | T | C | 194 | Myl12b | exonic | synonymous SNV | Myl12b:NM_023402:exon3:c.A282G:p.L94L | . | 17qE1.3 | rs13472620 | 0 | 1 |
| chr17 | 70976903 | C | T | 34.759 | Myl12b | intronic | . | . | . | 17qE1.3 | rs48471220 | 0 | 1 |
| chr17 | 70994511 | C | A | 37.8012 | Myl12a | UTR3 | . | . | . | 17qE1.3 | rs33485829 | 0 | 1 |
| chr17 | 70994557 | C | T | 85 | Myl12a | UTR3 | . | . | . | 17qE1.3 | rs33246108 | 0 | 1 |
| chr17 | 70994717 | C | T | 169 | Myl12a | exonic | synonymous SNV | Myl12a:NM_026064:exon4:c.G414A:p.E138E | . | 17qE1.3 | rs33204141 | 0 | 1 |
| chr17 | 70994813 | A | C | 168 | Myl12a | intronic | . | . | . | 17qE1.3 | rs33579967 | 0 | 1 |
| chr17 | 70994865 | C | T | 69 | Myl12a | intronic | . | . | . | 17qE1.3 | rs29537144 | 0 | 1 |
| chr17 | 70996126 | A | G | 138 | Myl12a | exonic | synonymous SNV | Myl12a:NM_026064:exon3:c.T330C:p.F110F | . | 17qE1.3 | rs33445047 | 0 | 1 |
| chr17 | 70996581 | C | T | 22.7466 | Myl12a | intronic | . | . | . | 17qE1.3 | rs29526705 | 0 | 1 |
| chr17 | 71061101 | T | A | 70 | Myom1 | intronic | . | . | . | 17qE1.3 | rs33549156 | 0 | 1 |
| chr17 | 71084290 | C | A | 218 | Myom1 | exonic | synonymous SNV | Myom1:NM_001083934:exon18:c.C2499A:p.S833S,Myom1:NM_010867:exon19:c.C2793A:p.S931S | . | 17qE1.3 | . | 0 | 1 |
| chr17 | 71229217 | C | G | 30.7725 | Lpin2 | intronic | . | . | . | 17qE1.3 | rs33353309 | 0 | 1 |
| chr17 | 71230472 | C | T | 194 | Lpin2 | intronic | . | . | . | 17qE1.3 | rs33380324 | 0 | 1 |
| chr17 | 71231429 | T | C | 221 | Lpin2 | exonic | synonymous SNV | Lpin2:NM_001164885:exon7:c.T1183C:p.L395L,Lpin2:NM_022882:exon7:c.T1069C:p.L357L | . | 17qE1.3 | rs33494149 | 0 | 1 |
| chr17 | 71238615 | C | T | 221 | Lpin2 | exonic | synonymous SNV | Lpin2:NM_001164885:exon10:c.C1569T:p.F523F,Lpin2:NM_022882:exon10:c.C1455T:p.F485F | . | 17qE1.3 | rs29516721 | 0 | 1 |
| chr17 | 71246539 | G | A | 156 | Lpin2 | intronic | . | . | . | 17qE1.3 | rs50800673 | 0 | 1 |
| chr17 | 71247152 | A | G | 53 | Lpin2 | UTR3 | . | . | . | 17qE1.3 | rs33581727 | 0 | 1 |
| chr17 | 71252855 | A | G | 60 | Emilin2 | exonic | synonymous SNV | Emilin2:NM_145158:exon8:c.T3126C:p.N1042N | . | 17qE1.3 | rs29521214 | 0 | 1 |
| chr17 | 71254993 | G | A | 41.0596 | Emilin2 | intronic | . | . | . | 17qE1.3 | rs49668646 | 0 | 1 |
| chr17 | 71255035 | A | G | 73 | Emilin2 | intronic | . | . | . | 17qE1.3 | rs46865778 | 0 | 1 |
| chr17 | 71255144 | A | G | 136 | Emilin2 | exonic | synonymous SNV | Emilin2:NM_145158:exon7:c.T2862C:p.D954D | . | 17qE1.3 | rs51155070 | 0 | 1 |
| chr17 | 71255233 | T | C | 120 | Emilin2 | exonic | missense SNV | Emilin2:NM_145158:exon7:c.A2773G:p.T925A | . | 17qE1.3 | rs49743770 | 0 | 1 |
| chr17 | 71273280 | C | T | 47.0873 | Emilin2 | intronic | . | . | . | 17qE1.3 | rs33493484 | 0 | 1 |
| chr17 | 71273483 | C | T | 187 | Emilin2 | exonic | synonymous SNV | Emilin2:NM_145158:exon4:c.G2247A:p.S749S | . | 17qE1.3 | rs33462737 | 0 | 1 |
| chr17 | 71273507 | C | T | 140 | Emilin2 | exonic | synonymous SNV | Emilin2:NM_145158:exon4:c.G2223A:p.R741R | . | 17qE1.3 | rs33532749 | 0 | 1 |
| chr17 | 71273745 | G | A | 184 | Emilin2 | exonic | missense SNV | Emilin2:NM_145158:exon4:c.C1985T:p.A662V | . | 17qE1.3 | rs33550769 | 0 | 1 |
| chr17 | 71274018 | A | C | 168 | Emilin2 | exonic | missense SNV | Emilin2:NM_145158:exon4:c.T1712G:p.V571G | . | 17qE1.3 | rs33068573 | 0 | 1 |
| chr17 | 71274275 | G | A | 115 | Emilin2 | exonic | synonymous SNV | Emilin2:NM_145158:exon4:c.C1455T:p.N485N | . | 17qE1.3 | rs29517252 | 0 | 1 |
| chr17 | 71274283 | T | C | 105 | Emilin2 | exonic | missense SNV | Emilin2:NM_145158:exon4:c.A1447G:p.T483A | . | 17qE1.3 | rs33256687 | 0 | 1 |
| chr17 | 71274320 | C | T | 128 | Emilin2 | exonic | synonymous SNV | Emilin2:NM_145158:exon4:c.G1410A:p.E470E | . | 17qE1.3 | rs33557530 | 0 | 1 |
| chr17 | 71274482 | C | T | 221 | Emilin2 | exonic | synonymous SNV | Emilin2:NM_145158:exon4:c.G1248A:p.A416A | . | 17qE1.3 | rs48802843 | 0 | 1 |
| chr17 | 71274749 | G | A | 142 | Emilin2 | exonic | synonymous SNV | Emilin2:NM_145158:exon4:c.C981T:p.I327I | . | 17qE1.3 | rs29523982 | 0 | 1 |
| chr17 | 71274884 | A | G | 124 | Emilin2 | exonic | synonymous SNV | Emilin2:NM_145158:exon4:c.T846C:p.S282S | . | 17qE1.3 | rs46556153 | 0 | 1 |
| chr17 | 71274957 | C | G | 89 | Emilin2 | exonic | missense SNV | Emilin2:NM_145158:exon4:c.G773C:p.S258T | . | 17qE1.3 | rs47512214 | 0 | 1 |
| chr17 | 71280710 | A | G | 101 | Emilin2 | exonic | missense SNV | Emilin2:NM_145158:exon3:c.T406C:p.S136P | . | 17qE1.3 | rs49041973 | 0 | 1 |
| chr17 | 71280900 | G | A | 82 | Emilin2 | intronic | . | . | . | 17qE1.3 | rs46630229 | 0 | 1 |
| chr17 | 71310376 | C | T | 173 | Emilin2 | intronic | . | . | . | 17qE1.3 | rs29506451 | 0 | 1 |
| chr17 | 71348857 | A | C | 140 | Smchd1 | intronic | . | . | . | 17qE1.3 | rs33079067 | 0 | 1 |
| chr17 | 71349063 | T | C | 108 | Smchd1 | intronic | . | . | . | 17qE1.3 | rs49314203 | 0 | 1 |
| chr17 | 71349069 | T | G | 72 | Smchd1 | intronic | . | . | . | 17qE1.3 | rs45800826 | 0 | 1 |
| chr17 | 71349523 | G | A | 65 | Smchd1 | intronic | . | . | . | 17qE1.3 | rs46502531 | 0 | 1 |
| chr17 | 71358465 | A | T | 164 | Smchd1 | intronic | . | . | . | 17qE1.3 | rs33706653 | 0 | 1 |
| chr17 | 71361983 | T | C | 110 | Smchd1 | intronic | . | . | . | 17qE1.3 | rs47300826 | 0 | 1 |
| chr17 | 71362890 | T | A | 56 | Smchd1 | intronic | . | . | . | 17qE1.3 | rs51452115 | 0 | 1 |
| chr17 | 71364952 | T | C | 59 | Smchd1 | intronic | . | . | . | 17qE1.3 | rs33581198 | 0 | 1 |
| chr17 | 71365123 | G | A | 120 | Smchd1 | exonic | synonymous SNV | Smchd1:NM_028887:exon39:c.C4935T:p.F1645F | . | 17qE1.3 | rs33345565 | 0 | 1 |

| | | | | | | | | | | | | | |
|---|---|---|---|---|---|---|---|---|---|---|---|---|---|
| chr17 | 71365231 | C | T | 90 | Smchd1 | intronic | . | . | . | 17qE1.3 | rs33670131 | 0 | 1 |
| chr17 | 71365253 | G | A | 113 | Smchd1 | intronic | . | . | . | 17qE1.3 | rs33651997 | 0 | 1 |
| chr17 | 71369719 | T | C | 86 | Smchd1 | intronic | . | . | . | 17qE1.3 | rs33286138 | 0 | 1 |
| chr17 | 71376856 | G | T | 61 | Smchd1 | intronic | . | . | . | 17qE1.3 | rs33314236 | 0 | 1 |
| chr17 | 71379405 | G | A | 89 | Smchd1 | intronic | . | . | . | 17qE1.3 | rs33308468 | 0 | 1 |
| chr17 | 71380414 | T | A | 39.3192 | Smchd1 | intronic | . | . | . | 17qE1.3 | rs29538067 | 0 | 1 |
| chr17 | 71386881 | A | G | 69 | Smchd1 | intronic | . | . | . | 17qE1.3 | rs33654133 | 0 | 1 |
| chr17 | 71415748 | G | A | 59 | Smchd1 | intronic | . | . | . | 17qE1.3 | rs29499777 | 0 | 1 |
| chr17 | 71425983 | T | A | 55 | Smchd1 | intronic | . | . | . | 17qE1.3 | rs33423471 | 0 | 1 |
| chr17 | 71431058 | C | T | 50 | Smchd1 | intronic | . | . | . | 17qE1.3 | rs33583958 | 0 | 1 |
| chr17 | 71459346 | T | C | 221 | Smchd1 | intronic | . | . | . | 17qE1.3 | . | 0 | 1 |
| chr17 | 71556381 | A | T | 221 | Spdya | exonic | missense SNV | Spdya:NM_001142631:exon2:c.A124T:p.S42C,Spdya:NM_029254:exon2:c.A124T:p.S42C | . | 17qE1.3 | rs49839657 | 0 | 1 |
| chr17 | 71598091 | T | G | 33.8436 | Trmt61b | ncRNA_exonic | . | . | . | 17qE1.3 | rs33376532 | 0 | 1 |
| chr17 | 71598252 | C | A | 157 | Trmt61b | ncRNA_exonic | . | . | . | 17qE1.3 | rs51089184 | 0 | 1 |
| chr17 | 71598696 | G | A | 203 | Trmt61b | ncRNA_exonic | . | . | 584 | 17qE1.3 | rs47853137 | 0 | 1 |
| chr17 | 71616464 | G | A | 102 | Wdr43 | exonic | synonymous SNV | Wdr43:NM_175639:exon1:c.G204A:p.A68A | 946 | 17qE1.3 | rs13472145 | 0 | 1 |
| chr17 | 71616503 | G | A | 64 | Wdr43 | intronic | . | . | 946 | 17qE1.3 | rs233169605 | 0 | 1 |
| chr17 | 71625266 | G | A | 33.7791 | Wdr43 | intronic | . | . | . | 17qE1.3 | rs49108943 | 0 | 1 |
| chr17 | 71625382 | G | A | 46.6587 | Wdr43 | exonic | synonymous SNV | Wdr43:NM_175639:exon2:c.G255A:p.S85S | . | 17qE1.3 | rs13472146 | 0 | 1 |
| chr17 | 71630123 | C | G | 150 | Wdr43 | intronic | . | . | . | 17qE1.3 | rs33713256 | 0 | 1 |
| chr17 | 71631215 | T | C | 64 | Wdr43 | intronic | . | . | . | 17qE1.3 | rs50514540 | 0 | 1 |
| chr17 | 71631218 | C | A | 43.404 | Wdr43 | intronic | . | . | . | 17qE1.3 | rs226031081 | 0 | 1 |
| chr17 | 71631313 | G | A | 212 | Wdr43,Snord92 | ncRNA_exonic | . | . | . | 17qE1.3 | rs48564915 | 0 | 1 |
| chr17 | 71631921 | G | C | 103 | Wdr43 | intronic | . | . | . | 17qE1.3 | rs50385747 | 0 | 1 |
| chr17 | 71632150 | T | C | 162 | Wdr43 | intronic | . | . | . | 17qE1.3 | rs48610141 | 0 | 1 |
| chr17 | 71632179 | T | A | 126 | Wdr43 | intronic | . | . | . | 17qE1.3 | rs46722111 | 0 | 1 |
| chr17 | 71633416 | A | G | 52 | Wdr43 | intronic | . | . | . | 17qE1.3 | rs49815889 | 0 | 1 |
| chr17 | 71633607 | C | T | 69 | Wdr43 | intronic | . | . | . | 17qE1.3 | rs33583051 | 0 | 1 |
| chr17 | 71633791 | A | G | 21.0511 | Wdr43 | intronic | . | . | . | 17qE1.3 | rs47230317 | 0 | 1 |
| chr17 | 71636487 | G | A | 66 | Wdr43 | intronic | . | . | . | 17qE1.3 | rs50130227 | 0 | 1 |
| chr17 | 71636699 | G | A | 139 | Wdr43 | intronic | . | . | . | 17qE1.3 | rs50616973 | 0 | 1 |
| chr17 | 71640949 | T | C | 161 | Wdr43,Snord53 | ncRNA_exonic | . | . | . | 17qE1.3 | rs13483094 | 0 | 1 |
| chr17 | 71641414 | C | A | 153 | Wdr43 | exonic | missense SNV | Wdr43:NM_175639:exon10:c.C1298A:p.T433N | . | 17qE1.3 | rs13472147 | 0 | 1 |
| chr17 | 71641498 | G | A | 98 | Wdr43 | intronic | . | . | . | 17qE1.3 | rs260051443 | 0 | 1 |
| chr17 | 71641530 | G | T | 89 | Wdr43 | intronic | . | . | . | 17qE1.3 | rs107984233 | 0 | 1 |
| chr17 | 71648628 | G | T | 95 | Wdr43 | intronic | . | . | . | 17qE1.3 | rs49602941 | 0 | 1 |
| chr17 | 71649036 | A | C | 41.9238 | Wdr43 | intronic | . | . | . | 17qE1.3 | rs46306891 | 0 | 1 |
| chr17 | 71657412 | G | A | 215 | Wdr43 | intronic | . | . | . | 17qE1.3 | rs51434850 | 0 | 1 |
| chr17 | 71685176 | T | G | 139 | Fam179a | exonic | synonymous SNV | Fam179a:NM_177087:exon2:c.T12G:p.R4R | . | 17qE1.3 | rs221972449 | 0 | 1 |
| chr17 | 71689010 | C | A | 78 | Fam179a | intronic | . | . | . | 17qE1.3 | rs51721635 | 0 | 1 |
| chr17 | 71689033 | C | T | 98 | Fam179a | intronic | . | . | . | 17qE1.3 | rs218248363 | 0 | 1 |
| chr17 | 71691469 | A | G | 98 | Fam179a | exonic | synonymous SNV | Fam179a:NM_177087:exon6:c.A666G:p.L222L | . | 17qE1.3 | rs48352888 | 0 | 1 |
| chr17 | 71695741 | C | T | 136 | Fam179a | exonic | missense SNV | Fam179a:NM_177087:exon7:c.C878T:p.P293L | . | 17qE1.3 | rs45998295 | 0 | 1 |
| chr17 | 71695790 | C | T | 21.8402 | Fam179a | intronic | . | . | . | 17qE1.3 | rs48275494 | 0 | 1 |
| chr17 | 71697861 | G | A | 169 | Fam179a | exonic | synonymous SNV | Fam179a:NM_177087:exon8:c.G909A:p.K303K | . | 17qE1.3 | rs29501902 | 0 | 1 |
| chr17 | 71697914 | T | G | 138 | Fam179a | exonic | missense SNV | Fam179a:NM_177087:exon8:c.T962G:p.F321C | . | 17qE1.3 | rs51842469 | 0 | 1 |
| chr17 | 71700874 | C | T | 175 | Fam179a | intronic | . | . | . | 17qE1.3 | rs46778241 | 0 | 1 |
| chr17 | 71705699 | C | T | 42.0847 | Fam179a | intronic | . | . | . | 17qE1.3 | rs217540694 | 0 | 1 |
| chr17 | 71707143 | T | C | 100 | Fam179a | exonic | missense SNV | Fam179a:NM_177087:exon13:c.T1685C:p.V562A | . | 17qE1.3 | rs46351261 | 0 | 1 |
| chr17 | 71716455 | G | A | 222 | Fam179a | exonic | missense SNV | Fam179a:NM_177087:exon17:c.G2305A:p.V769I | . | 17qE1.3 | rs6382778 | 0 | 1 |
| chr17 | 71716528 | A | G | 182 | Fam179a | intronic | . | . | . | 17qE1.3 | rs6383315 | 0 | 1 |
| chr17 | 71744391 | A | G | 57 | BC027072 | UTR3 | . | . | . | 17qE1.3 | rs29503526 | 0 | 1 |
| chr17 | 71806915 | C | T | 64 | Clip4 | intronic | . | . | . | 17qE1.3 | rs47229568 | 0 | 1 |
| chr17 | 71824796 | C | T | 181 | Clip4 | exonic | synonymous SNV | Clip4:NM_001271483:exon9:c.C1128T:p.V376V,Clip4:NM_001271484:exon9:c.C1128T:p.V376V,Clip4:NM_ | . | 17qE1.3 | rs51116352 | 0 | 1 |
| chr17 | 71824809 | T | G | 174 | Clip4 | exonic | missense SNV | Clip4:NM_001271483:exon9:c.T1141G:p.F381V,Clip4:NM_001271484:exon9:c.T1141G:p.F381V,Clip4:NM_ | . | 17qE1.3 | rs47337114 | 0 | 1 |
| chr17 | 71824948 | C | T | 58 | Clip4 | intronic | . | . | . | 17qE1.3 | rs51936089 | 0 | 1 |
| chr17 | 71831085 | A | T | 84 | Clip4 | intronic | . | . | . | 17qE1.3 | rs48407015 | 0 | 1 |
| chr17 | 71831247 | A | G | 178 | Clip4 | exonic | missense SNV | Clip4:NM_175378:exon11:c.A1298G:p.H433R,Clip4:NM_001271483:exon12:c.A1439G:p.H480R,Clip4:NM_( | . | 17qE1.3 | rs50568716 | 0 | 1 |
| chr17 | 71831284 | C | T | 164 | Clip4 | exonic | synonymous SNV | Clip4:NM_175378:exon11:c.C1335T:p.V445V,Clip4:NM_001271483:exon12:c.C1476T:p.V492V,Clip4:NM_( | . | 17qE1.3 | rs46323714 | 0 | 1 |
| chr17 | 71831391 | A | G | 95 | Clip4 | intronic | . | . | . | 17qE1.3 | rs255423784 | 0 | 1 |
| chr17 | 71834230 | T | C | 65 | Clip4 | exonic | synonymous SNV | Clip4:NM_175378:exon12:c.T1437C:p.D479D,Clip4:NM_001271483:exon13:c.T1578C:p.D526D,Clip4:NM_( | . | 17qE1.3 | rs51931722 | 0 | 1 |
| chr17 | 71849745 | C | A | 75 | Clip4 | intronic | . | . | . | 17qE1.3 | rs33084539 | 0 | 1 |
| chr17 | 71856435 | T | C | 193 | Clip4 | exonic | synonymous SNV | Clip4:NM_030179:exon16:c.T2001C:p.A667A | . | 17qE1.3 | rs50534818 | 0 | 1 |
| chr17 | 71856510 | C | T | 131 | Clip4 | exonic | synonymous SNV | Clip4:NM_030179:exon16:c.C2076T:p.R692R | . | 17qE1.3 | rs30876251 | 0 | 1 |
| chr17 | 71869389 | C | T | 81 | Alk | UTR3 | . | . | . | 17qE1.3 | rs251558121 | 0 | 1 |
| chr17 | 71869547 | A | G | 89 | Alk | exonic | synonymous SNV | Alk:NM_007439:exon29:c.T4761C:p.N1587N | . | 17qE1.3 | rs50949764 | 0 | 1 |
| chr17 | 71874275 | C | G | 156 | Alk | intronic | . | . | . | 17qE1.3 | rs29502736 | 0 | 1 |
| chr17 | 71880812 | G | A | 175 | Alk | exonic | synonymous SNV | Alk:NM_007439:exon26:c.C3933T:p.T1311T | . | 17qE1.3 | rs257209362 | 0 | 1 |
| chr17 | 71885215 | T | C | 70 | Alk | intronic | . | . | . | 17qE1.3 | rs49006562 | 0 | 1 |
| chr17 | 71890876 | C | A | 48.2489 | Alk | intronic | . | . | . | 17qE1.3 | rs48956761 | 0 | 1 |
| chr17 | 71894035 | C | A | 112 | Alk | intronic | . | . | . | 17qE1.3 | rs255436953 | 0 | 1 |
| chr17 | 71897974 | G | T | 205 | Alk | exonic | missense SNV | Alk:NM_007439:exon20:c.C3202A:p.Q1068K | . | 17qE1.3 | . | 0 | 1 |
| chr17 | 71904445 | T | C | 44.8887 | Alk | intronic | . | . | . | 17qE1.3 | rs48217220 | 0 | 1 |
| chr17 | 71911636 | C | T | 221 | Alk | splicing | . | . | . | 17qE1.3 | rs48833573 | 0 | 1 |
| chr17 | 71911693 | C | A | 137 | Alk | intronic | . | . | . | 17qE1.3 | . | 0 | 1 |
| chr17 | 71911698 | C | T | 202 | Alk | intronic | . | . | . | 17qE1.3 | rs29535434 | 0 | 1 |
| chr17 | 71921966 | G | C | 30.7337 | Alk | exonic | synonymous SNV | Alk:NM_007439:exon12:c.C2103G:p.T701T | . | 17qE1.3 | rs48882645 | 0 | 1 |
| chr17 | 71922073 | G | C | 34.7769 | Alk | intronic | . | . | . | 17qE1.3 | rs251412651 | 0 | 1 |
| chr17 | 71922119 | T | G | 33.0725 | Alk | intronic | . | . | . | 17qE1.3 | rs50614637 | 0 | 1 |
| chr17 | 71949674 | T | C | 61 | Alk | intronic | . | . | . | 17qE1.3 | rs51410058 | 0 | 1 |
| chr17 | 71949741 | A | G | 142 | Alk | intronic | . | . | . | 17qE1.3 | rs46813610 | 0 | 1 |
| chr17 | 71950060 | C | T | 146 | Alk | intronic | . | . | . | 17qE1.3 | rs46208161 | 0 | 1 |
| chr17 | 71967563 | A | G | 197 | Alk | exonic | synonymous SNV | Alk:NM_007439:exon9:c.T1752C:p.T584T | . | 17qE1.3 | rs50129041 | 0 | 1 |
| chr17 | 71992336 | G | T | 62 | Alk | intronic | . | . | . | 17qE1.3 | . | 0 | 1 |
| chr17 | 73367158 | G | A | 183 | Capn13 | intronic | . | . | . | 17qE2 | . | 0 | 1 |
| chr17 | 73382878 | T | A | 222 | Capn13 | exonic | missense SNV | Capn13:NM_001033444:exon2:c.A137T:p.K46M | . | 17qE2 | . | 0 | 1 |
| chr17 | 73512199 | T | G | 145 | Galnt14 | intronic | . | . | . | 17qE2 | . | 0 | 1 |
| chr17 | 73905937 | A | C | 157 | Xdh | intronic | . | . | . | 17qE2 | . | 0 | 1 |
| chr17 | 74412153 | A | G | 52 | Slc30a6 | intronic | . | . | . | 17qE2 | . | 0 | 1 |
| chr17 | 74548761 | A | T | 87 | Birc6 | exonic | missense SNV | Birc6:NM_007566:exon3:c.A565T:p.T189S | . | 17qE2 | . | 0 | 1 |
| chr17 | 75290061 | C | T | 130 | Ltbp1 | exonic | synonymous SNV | Ltbp1:NM_206958:exon12:c.C1686T:p.H562H,Ltbp1:NM_019919:exon16:c.C2640T:p.H880H | . | 17qE2 | . | 0 | 1 |
| chr17 | 78625049 | C | T | 221 | Vit | exonic | synonymous SNV | Vit:NM_001197028:exon12:c.C1518T:p.A506A,Vit:NM_028813:exon13:c.C1584T:p.A528A | . | 17qE3 | . | 0 | 1 |
| chr17 | 78687348 | G | C | 28.9904 | Strn | intronic | . | . | . | 17qE3 | . | 0 | 1 |
| chr17 | 78687359 | G | T | 30.2758 | Strn | intronic | . | . | . | 17qE3 | . | 0 | 1 |
| chr17 | 78831323 | A | G | 69 | Heatr5b | intronic | . | . | . | 17qE3 | . | 0 | 1 |
| chr17 | 78937218 | A | T | 148 | Ndufaf7 | intronic | . | . | 619 | 17qE3 | . | 0 | 1 |
| chr17 | 78943230 | A | T | 191 | Ndufaf7 | intronic | . | . | . | 17qE3 | . | 0 | 1 |
| chr17 | 79715722 | C | T | 219 | . | upstream | . | . | 421 | 17qE3 | . | 0 | 1 |
| chr17 | 80349279 | G | A | 78 | Arhgef33 | intronic | . | . | . | 17qE3 | . | 0 | 1 |
| chr17 | 80535359 | C | A | 103 | Cdk14 | intronic | . | . | . | 17qE3 | . | 0 | 1 |
| chr17 | 83441468 | C | A | 190 | Eml4 | intronic | . | . | . | 17qE4 | . | 0 | 1 |
| chr17 | 83738252 | C | G | 99 | Mta3 | intronic | . | . | . | 17qE4 | rs49499677 | 0 | 1 |
| chr17 | 83738303 | A | G | 124 | Mta3 | intronic | . | . | . | 17qE4 | rs52003502 | 0 | 1 |
| chr17 | 83738483 | T | G | 221 | Mta3 | intronic | . | . | . | 17qE4 | . | 0 | 1 |
| chr17 | 84563887 | T | C | 151 | Plekhh2 | exonic | missense SNV | Plekhh2:NM_177606:exon7:c.T572C:p.F191S | . | 17qE4 | . | 0 | 1 |
| chr17 | 85202659 | T | A | 25.4743 | Camkmt | intronic | . | . | . | 17qE4 | . | 0 | 1 |
| chr17 | 86824413 | C | A | 82 | Epas1 | exonic | synonymous SNV | Epas1:NM_010137:exon9:c.C1041A:p.I347I | . | 17qE4 | . | 0 | 1 |
| chr17 | 88009006 | C | T | 115 | Fbxo11 | splicing | . | . | . | 17qE4 | . | 0 | 1 |
| chr17 | 88582400 | G | A | 221 | Ppp1r21 | intronic | . | . | . | 17qE4 | . | 0 | 1 |
| chr17 | 88671430 | T | C | 221 | Gtf2a1l | intronic | . | . | . | 17qE4 | . | 0 | 1 |
| chr17 | 88711707 | G | A | 139 | Gtf2a1l | intronic | . | . | . | 17qE4 | . | 0 | 1 |
| chr17 | 88755599 | T | C | 78 | Lhcgr | exonic | missense SNV | Lhcgr:NM_013582:exon8:c.A644G:p.E215G | . | 17qE4 | . | 0 | 1 |
| chr17 | 88985740 | G | T | 130 | Fshr | exonic | synonymous SNV | Fshr:NM_013523:exon10:c.C1509A:p.P503P | . | 17qE5 | . | 0 | 1 |
| chr17 | 88986320 | T | C | 221 | Fshr | exonic | missense SNV | Fshr:NM_013523:exon10:c.A929G:p.Q310R | . | 17qE5 | . | 0 | 1 |
| chr17 | 89009370 | G | A | 151 | Fshr | intronic | . | . | . | 17qE5 | . | 0 | 1 |
| chr17 | 90702075 | T | C | 221 | Nrxn1 | intronic | . | . | . | 17qE5 | . | 0 | 1 |
| chr17 | 94608303 | C | A | 195 | . | intergenic | . | . | 328 | 17qE5 | . | 0 | 1 |
| chr18 | 3267895 | C | A | 221 | Crem | UTR3 | . | . | . | 18qA1 | . | 0 | 1 |
| chr18 | 3430873 | G | C | 189 | Cul2 | intronic | . | . | . | 18qA1 | . | 0 | 1 |
| chr18 | 4052723 | C | T | 45.7618 | . | intergenic | . | . | 213 | 18qA1 | rs228643517 | 0 | 1 |
| chr18 | 4673614 | G | T | 168 | 9430020K01Rik | exonic | missense SNV | 9430020K01Rik:NM_001081963:exon3:c.G1375T:p.G459C | . | 18qA1 | . | 0 | 1 |
| chr18 | 6026739 | A | G | 222 | Arhgap12 | intronic | . | . | . | 18qA1 | . | 0 | 1 |
| chr18 | 7444112 | C | T | 187 | Mpp7 | intronic | . | . | . | 18qA1 | . | 0 | 1 |
| chr18 | 7910758 | G | A | 222 | Wac | exonic | missense SNV | Wac:NM_001282093:exon7:c.G718A:p.A240T,Wac:NM_153085:exon7:c.G853A:p.A285T | . | 18qA1 | . | 0 | 1 |
| chr18 | 8227411 | T | C | 81 | . | intergenic | . | . | . | 18qA1 | . | 0 | 1 |
| chr18 | 8227436 | C | A | 82 | . | intergenic | . | . | . | 18qA1 | . | 0 | 1 |
| chr18 | 8227975 | T | G | 132 | . | intergenic | . | . | . | 18qA1 | . | 0 | 1 |
| chr18 | 9280885 | A | T | 221 | Gjd4 | exonic | synonymous SNV | Gjd4:NM_153086:exon2:c.T192A:p.V64V | . | 18qA1 | . | 0 | 1 |
| chr18 | 9345460 | C | T | 222 | Ccny | exonic | missense SNV | Ccny:NM_026484:exon6:c.G413A:p.G138E | . | 18qA1 | . | 0 | 1 |
| chr18 | 10129531 | T | C | 47.4933 | Rock1 | intronic | . | . | . | 18qA1 | . | 0 | 1 |
| chr18 | 10647828 | C | T | 143 | Abhd3 | exonic | missense SNV | Abhd3:NM_134130:exon8:c.G955A:p.D319N | . | 18qA1 | . | 0 | 1 |
| chr18 | 10792953 | G | T | 212 | Mib1 | exonic | missense SNV | Mib1:NM_144860:exon13:c.G1888T:p.D630Y | . | 18qA1 | . | 0 | 1 |
| chr18 | 10811830 | A | C | 76 | Mib1 | intronic | . | . | . | 18qA1 | . | 0 | 1 |
| chr18 | 12189722 | A | G | 120 | 3110002H16Rik,Nnd | UTR3 | . | . | . | 18qA1 | . | 0 | 1 |
| chr18 | 12472243 | A | T | 220 | Lama3 | exonic | missense SNV | Lama3:NM_010680:exon27:c.A3253T:p.S1085C | . | 18qA1 | . | 0 | 1 |
| chr18 | 12519677 | G | C | 115 | Lama3 | intronic | . | . | . | 18qA1 | . | 1 | 0 |
| chr18 | 14651458 | C | G | 23.0085 | Ss18 | intronic | . | . | . | 18qA1 | . | 0 | 1 |
| chr18 | 14804517 | A | G | 219 | Taf4b | exonic | missense SNV | Taf4b:NM_001100449:exon4:c.A646G:p.N216D | . | 18qA1 | . | 0 | 1 |
| chr18 | 15062628 | A | C | 222 | Kctd1 | exonic | missense SNV | Kctd1:NM_001142731:exon1:c.T937G:p.F313V | 2483 | 18qA1 | . | 0 | 1 |
| chr18 | 15151371 | C | T | 222 | Kctd1 | UTR5 | . | . | . | 18qA1 | . | 0 | 1 |
| chr18 | 15452718 | T | G | 222 | Chst9 | exonic | missense SNV | Chst9:NM_199055:exon4:c.A787C:p.K263Q | . | 18qA1 | . | 0 | 1 |
| chr18 | 15717817 | T | G | 168 | Chst9 | intronic | . | . | . | 18qA1 | . | 0 | 1 |
| chr18 | 15833229 | C | T | 78 | . | intergenic | . | . | . | 18qA1 | . | 0 | 1 |
| chr18 | 19989667 | T | G | 89 | Dsc3 | exonic | missense SNV | Dsc3:NM_001291809:exon3:c.A199C:p.S67R,Dsc3:NM_007882:exon3:c.A199C:p.S67R | . | 18qA2 | . | 0 | 1 |
| chr18 | 20521515 | T | A | 222 | Dsg3 | exonic | missense SNV | Dsg3:NM_030596:exon4:c.T275A:p.I92N | . | 18qA2 | . | 0 | 1 |
| chr18 | 20538648 | C | T | 109 | Dsg3 | intronic | . | . | . | 18qA2 | . | 0 | 1 |
| chr18 | 20573629 | C | T | 34.4877 | Dsg2 | intronic | . | . | . | 18qA2 | . | 0 | 1 |
| chr18 | 20592096 | G | A | 24.6117 | Dsg2 | intronic | . | . | . | 18qA2 | . | 0 | 1 |
| chr18 | 21093416 | A | T | 221 | Mep1b | exonic | missense SNV | Mep1b:NM_008586:exon11:c.A1334T:p.Q445L | . | 18qA2 | . | 0 | 1 |
| chr18 | 21093417 | G | T | 221 | Mep1b | exonic | missense SNV | Mep1b:NM_008586:exon11:c.G1335T:p.Q445H | . | 18qA2 | . | 0 | 1 |
| chr18 | 21168640 | T | A | 82 | Garem | intronic | . | . | . | 18qA2 | . | 0 | 1 |
| chr18 | 22120658 | T | C | 36.5623 | Ccdc178 | exonic | missense SNV | Ccdc178:NM_027616:exon9:c.A523G:p.S175G | . | 18qA2 | . | 0 | 1 |
| chr18 | 22521857 | A | C | 220 | Asxl3 | exonic | missense SNV | Asxl3:NM_001167777:exon13:c.A2923C:p.K975Q | . | 18qA2 | . | 0 | 1 |
| chr18 | 22770946 | T | G | 95 | Nol4 | intronic | . | . | . | 18qA2 | . | 0 | 1 |
| chr18 | 23646746 | C | T | 32.7293 | Dtna | intronic | . | . | . | 18qA2 | . | 0 | 1 |
| chr18 | 23651522 | G | T | 128 | Dtna | exonic | synonymous SNV | Dtna:NM_001285817:exon18:c.G1905T:p.P635P,Dtna:NM_001285808:exon19:c.G1926T:p.P642P,Dtna:NM_( | . | 18qA2 | . | 0 | 1 |
| chr18 | 23877760 | G | A | 110 | Mapre2 | intronic | . | . | . | 18qA2 | . | 1 | 0 |
| chr18 | 24429199 | G | A | 85 | . | upstream | . | . | . | 18qA2 | . | 0 | 1 |
| chr18 | 24670453 | T | A | 177 | Mocos | intronic | . | . | . | 18qA2 | . | 0 | 1 |
| chr18 | 25400123 | G | A | 23.5292 | AW554918 | intronic | . | . | . | 18qA2 | . | 0 | 1 |
| chr18 | 25753541 | G | A | 185 | Celf4 | exonic | synonymous SNV | Celf4:NM_001146292:exon1:c.C87T:p.S29S,Celf4:NM_001146293:exon1:c.C87T:p.S29S,Celf4:NM_00114( | 478 | 18qA2 | . | 0 | 1 |
| chr18 | 30330160 | G | A | 222 | Pik3c3 | intronic | . | . | . | 18qB1 | . | 0 | 1 |
| chr18 | 30347847 | A | G | 35.7952 | Pik3c3 | UTR3 | . | . | . | 18qB1 | . | 0 | 1 |
| chr18 | 30459029 | G | T | 92 | . | intergenic | . | . | . | 18qB1 | . | 0 | 1 |
| chr18 | 31967874 | G | C | 137 | Myo7b | intronic | . | . | . | 18qB1 | . | 0 | 1 |
| chr18 | 31990224 | G | A | 222 | Myo7b | exonic | missense SNV | Myo7b:NM_032394:exon17:c.C2059T:p.R687C | . | 18qB1 | . | 0 | 1 |
| chr18 | 32125331 | G | T | 200 | Proc | intronic | . | . | . | 18qB1 | . | 1 | 0 |
| chr18 | 32133148 | A | G | 130 | Proc | exonic | synonymous SNV | Proc:NM_001042767:exon6:c.T531C:p.T177T,Proc:NM_001313938:exon6:c.T531C:p.T177T,Proc:NM_0089: | . | 18qB1 | . | 0 | 1 |
| chr18 | 33129618 | G | T | 89 | Camk4 | intronic | . | . | . | 18qB1 | . | 0 | 1 |
| chr18 | 33176018 | A | G | 93 | Camk4 | intronic | . | . | . | 18qB1 | . | 0 | 1 |
| chr18 | 34497819 | A | C | 29.3273 | Fam13b | intronic | . | . | . | 18qB1 | . | 0 | 1 |
| chr18 | 34641490 | G | C | 221 | Cdc23 | exonic | missense SNV | Cdc23:NM_178347:exon7:c.C815G:p.A272G | . | 18qB1 | . | 0 | 1 |
| chr18 | 34796919 | C | A | 192 | Kdm3b | intronic | . | . | . | 18qB1 | . | 0 | 1 |
| chr18 | 35616105 | A | T | 76 | Slc23a1,Paip2 | intronic | . | . | . | 18qB2 | . | 0 | 1 |
| chr18 | 35648222 | C | A | 170 | Mzb1 | intronic | . | . | . | 18qB2 | . | 0 | 1 |
| chr18 | 36038501 | T | C | 116 | Nrg2 | intronic | . | . | . | 18qB2 | . | 0 | 1 |
| chr18 | 36613184 | C | T | 193 | Ankhd1 | exonic | synonymous SNV | Ankhd1:NM_175375:exon12:c.C1971T:p.L657L | . | 18qB2 | . | 0 | 1 |
| chr18 | 36795128 | T | C | 175 | Zmat2 | exonic | missense SNV | Zmat2:NM_025594:exon3:c.T200C:p.V67A | . | 18qB2 | . | 0 | 1 |
| chr18 | 36939766 | C | T | 220 | Pcdha1,Pcdha2 | exonic | missense SNV | Pcdha2:NM_198117:exon1:c.C449T:p.S150L | . | 18qB2 | . | 0 | 1 |
| chr18 | 36940885 | T | C | 220 | Pcdha2,Pcdha1 | exonic | missense SNV | Pcdha2:NM_198117:exon1:c.T1568C:p.L523P | . | 18qB2 | . | 0 | 1 |
| chr18 | 36969890 | T | A | 221 | Gm38666,Pcdha5,Gm | exonic | missense SNV | Gm38666:NM_001301256:exon1:c.T2162A:p.L721Q,Pcdha6:NM_007767:exon1:c.T2135A:p.L712Q | . | 18qB2 | . | 0 | 1 |
| chr18 | 36980345 | G | A | 119 | Pcdha7-g,Pcdha3,I | intronic | . | . | 309 | 18qB2 | . | 0 | 1 |
| chr18 | 36980710 | C | G | 83 | Gm38666,Gm37013,I | intronic | . | . | . | 18qB2 | . | 0 | 1 |
| chr18 | 37006866 | G | A | 222 | Pcdha2,Pcdha6,Pcd | exonic | missense SNV | Pcdha10:NM_009961:exon1:c.G1547A:p.G516D | 276 | 18qB2 | . | 0 | 1 |
| chr18 | 37092263 | C | T | 221 | Pcdha6,Pcdhac1,Pc | exonic | stopgain | Pcdhac1:NM_001003671:exon1:c.C2128T:p.Q710X | . | 18qB2 | . | 0 | 1 |

| | | | | | | | | | | | | | |
|---|---|---|---|---|---|---|---|---|---|---|---|---|---|
| chr18 | 37145824 | T | A | 210 | Pcdha3,Pcdhac2,Pc | exonic | missense SNV | Pcdhac2:NM_001003672:exon1:c.T1856A:p.L619H | . | 18qB3 | . | 0 | 1 |
| chr18 | 37303723 | C | T | 221 | Gm38666,Pcdha7-g, | UTR3 | . | . | . | 18qB3 | . | 0 | 1 |
| chr18 | 37309267 | G | C | 221 | Pcdha7-g,Gm37013, | exonic | missense SNV | Pcdhb4:NM_053129:exon1:c.G1629C:p.Q543H | 433 | 18qB3 | . | 0 | 1 |
| chr18 | 37449739 | C | A | 221 | Pcdha7-g,Gm37013, | exonic | missense SNV | Pcdhb14:NM_053139:exon1:c.C1897A:p.R633S | 284 | 18qB3 | . | 0 | 1 |
| chr18 | 37474912 | A | C | 176 | Gm38666,Gm37013,I | exonic | missense SNV | Pcdhb15:NM_053140:exon1:c.A1196C:p.N399T | . | 18qB3 | . | 0 | 1 |
| chr18 | 37662735 | T | C | 222 | Pcdhga1,Pcdha7-g, | exonic | missense SNV | Pcdhga1:NM_033584:exon1:c.T791C:p.V264A | . | 18qB3 | . | 0 | 1 |
| chr18 | 37674800 | G | T | 219 | Gm37013,Pcdhga3,I | exonic | missense SNV | Pcdhga3:NM_033586:exon1:c.G305T:p.S102I | . | 18qB3 | . | 0 | 1 |
| chr18 | 37681065 | T | G | 221 | Gm38666,Pcdhga2,I | exonic | missense SNV | Pcdhgb1:NM_033574:exon1:c.T608G:p.M203R | . | 18qB3 | . | 0 | 1 |
| chr18 | 37900666 | A | C | 222 | Diaph1 | exonic | missense SNV | Diaph1:NM_007858:exon7:c.T776G:p.I259S,Diaph1:NM_001305980:exon8:c.T803G:p.I268S,Diaph1:NM_00 | . | 18qB3 | . | 0 | 1 |
| chr18 | 37957570 | C | A | 143 | Fchsd1,Rell2 | UTR3 | . | . | . | 18qB3 | . | 0 | 1 |
| chr18 | 37969181 | A | T | 101 | Fchsd1 | intronic | . | . | . | 18qB3 | . | 0 | 1 |
| chr18 | 39227553 | G | T | 111 | Arhgap26 | intronic | . | . | . | 18qB3 | . | 0 | 1 |
| chr18 | 41998710 | C | G | 43.0889 | Grxcr2 | exonic | missense SNV | Grxcr2:NM_001033426:exon1:c.G293C:p.G98A | . | 18qB3 | . | 0 | 1 |
| chr18 | 42338563 | A | T | 105 | Rbm27 | UTR3 | . | . | . | 18qB3 | . | 0 | 1 |
| chr18 | 42578405 | C | A | 221 | Gpr151 | exonic | missense SNV | Gpr151:NM_181543:exon1:c.G1207T:p.A403S | . | 18qB3 | . | 0 | 1 |
| chr18 | 42898545 | G | C | 136 | Ppp2r2b | intronic | . | . | . | 18qB3 | rs30155617 | 1 | 0 |
| chr18 | 43059181 | A | G | 228 | Ppp2r2b | exonic | synonymous SNV | Ppp2r2b:NM_028392:exon1:c.T60C:p.S20S | . | 18qB3 | rs244933869 | 1 | 0 |
| chr18 | 43171204 | A | G | 228 | . | intergenic | . | . | . | 18qB3 | rs31895854 | 1 | 0 |
| chr18 | 43171738 | C | G | 228 | . | intergenic | . | . | . | 18qB3 | rs31894892 | 1 | 0 |
| chr18 | 43171897 | G | C | 228 | . | intergenic | . | . | . | 18qB3 | rs240511914 | 1 | 0 |
| chr18 | 43171967 | C | T | 191 | . | intergenic | . | . | . | 18qB3 | rs31894890 | 1 | 0 |
| chr18 | 43211925 | C | T | 228 | Stk32a | UTR5 | . | . | . | 18qB3 | rs31897774 | 1 | 0 |
| chr18 | 43212171 | G | A | 155 | Stk32a | intronic | . | . | . | 18qB3 | rs31897013 | 1 | 0 |
| chr18 | 43212197 | G | A | 126 | Stk32a | intronic | . | . | . | 18qB3 | rs29967110 | 1 | 0 |
| chr18 | 43243205 | G | A | 228 | Stk32a | intronic | . | . | . | 18qB3 | rs31900327 | 1 | 0 |
| chr18 | 43297335 | G | A | 168 | Stk32a | intronic | . | . | . | 18qB3 | rs31895837 | 1 | 0 |
| chr18 | 43304928 | A | G | 228 | Stk32a | intronic | . | . | . | 18qB3 | rs30307939 | 1 | 0 |
| chr18 | 43304973 | C | T | 228 | Stk32a | intronic | . | . | . | 18qB3 | rs31899486 | 1 | 0 |
| chr18 | 43304993 | G | C | 228 | Stk32a | intronic | . | . | . | 18qB3 | rs29560034 | 1 | 0 |
| chr18 | 43310208 | A | G | 146 | Stk32a | intronic | . | . | . | 18qB3 | rs47848273 | 1 | 0 |
| chr18 | 43310615 | G | T | 228 | Stk32a | intronic | . | . | . | 18qB3 | rs108840599 | 1 | 0 |
| chr18 | 43313251 | C | T | 90 | Stk32a | intronic | . | . | . | 18qB3 | rs46393180 | 1 | 0 |
| chr18 | 43313544 | A | G | 228 | Stk32a | intronic | . | . | . | 18qB3 | rs240848334 | 1 | 0 |
| chr18 | 43313545 | A | G | 228 | Stk32a | intronic | . | . | . | 18qB3 | rs46113681 | 1 | 0 |
| chr18 | 43314085 | C | T | 147 | Stk32a | intronic | . | . | . | 18qB3 | rs261726284 | 1 | 0 |
| chr18 | 43315052 | C | A | 228 | Stk32a | splicing | . | . | . | 18qB3 | rs52526861 | 1 | 0 |
| chr18 | 43315211 | G | A | 228 | Stk32a | UTR3 | . | . | . | 18qB3 | rs51089198 | 1 | 0 |
| chr18 | 43315217 | G | C | 228 | Stk32a | UTR3 | . | . | . | 18qB3 | rs51179481 | 1 | 0 |
| chr18 | 43326036 | C | A | 153 | Dpysl3 | intronic | . | . | . | 18qB3 | rs30074648 | 1 | 0 |
| chr18 | 43328231 | T | C | 163 | Dpysl3 | intronic | . | . | . | 18qB3 | rs29768843 | 1 | 0 |
| chr18 | 43328400 | G | A | 94 | Dpysl3 | exonic | synonymous SNV | Dpysl3:NM_001136086:exon12:c.C1380T:p.T460T,Dpysl3:NM_001291455:exon12:c.C1725T:p.T575T,Dpysl | . | 18qB3 | . | 0 | 1 |
| chr18 | 43332732 | C | T | 137 | Dpysl3 | intronic | . | . | . | 18qB3 | rs30017924 | 1 | 0 |
| chr18 | 43358214 | G | A | 228 | Dpysl3 | intronic | . | . | . | 18qB3 | rs50614855 | 1 | 0 |
| chr18 | 43358218 | G | A | 228 | Dpysl3 | intronic | . | . | . | 18qB3 | rs50113569 | 1 | 0 |
| chr18 | 43358391 | G | A | 228 | Dpysl3 | exonic | synonymous SNV | Dpysl3:NM_001136086:exon4:c.C315T:p.H105H,Dpysl3:NM_001291455:exon4:c.C660T:p.H220H,Dpysl3:NM | . | 18qB3 | rs13459338 | 1 | 0 |
| chr18 | 43360947 | T | C | 228 | Dpysl3 | intronic | . | . | . | 18qB3 | rs50389007 | 1 | 0 |
| chr18 | 43361328 | A | C | 105 | Dpysl3 | intronic | . | . | . | 18qB3 | rs49950601 | 1 | 0 |
| chr18 | 43363469 | A | G | 228 | Dpysl3 | intronic | . | . | . | 18qB3 | rs31894549 | 1 | 0 |
| chr18 | 43363476 | G | T | 228 | Dpysl3 | intronic | . | . | . | 18qB3 | rs48757111 | 1 | 0 |
| chr18 | 43363483 | G | A | 228 | Dpysl3 | intronic | . | . | . | 18qB3 | rs31894548 | 1 | 0 |
| chr18 | 43363669 | T | C | 228 | Dpysl3 | intronic | . | . | . | 18qB3 | rs31894547 | 1 | 0 |
| chr18 | 43363681 | C | T | 170 | Dpysl3 | intronic | . | . | . | 18qB3 | . | 0 | 1 |
| chr18 | 43373014 | C | A | 228 | Dpysl3 | intronic | . | . | . | 18qB3 | rs29823352 | 1 | 0 |
| chr18 | 43438267 | G | C | 228 | Dpysl3 | UTR5 | . | . | 560 | 18qB3 | . | 1 | 0 |
| chr18 | 43549344 | T | C | 125 | Jakmip2 | intronic | . | . | . | 18qB3 | rs45898475 | 1 | 0 |
| chr18 | 43549528 | G | A | 228 | Jakmip2 | intronic | . | . | . | 18qB3 | rs46340538 | 1 | 0 |
| chr18 | 43552396 | A | T | 132 | Jakmip2 | intronic | . | . | . | 18qB3 | rs48772483 | 1 | 0 |
| chr18 | 43556458 | C | A | 228 | Jakmip2 | intronic | . | . | . | 18qB3 | rs51242414 | 1 | 0 |
| chr18 | 43556604 | A | G | 228 | Jakmip2 | intronic | . | . | . | 18qB3 | rs50762065 | 1 | 0 |
| chr18 | 43558983 | C | T | 208 | Jakmip2 | intronic | . | . | . | 18qB3 | rs46340883 | 1 | 0 |
| chr18 | 43558991 | T | C | 71 | Jakmip2 | intronic | . | . | . | 18qB3 | . | 0 | 1 |
| chr18 | 43565798 | G | A | 228 | Jakmip2 | intronic | . | . | . | 18qB3 | rs218710232 | 1 | 0 |
| chr18 | 43568023 | C | T | 228 | Jakmip2 | intronic | . | . | . | 18qB3 | . | 1 | 0 |
| chr18 | 43568137 | C | T | 228 | Jakmip2 | intronic | . | . | . | 18qB3 | rs47906283 | 1 | 0 |
| chr18 | 43575530 | A | G | 228 | Jakmip2 | exonic | synonymous SNV | Jakmip2:NM_001163637:exon5:c.T867C:p.A289A | . | 18qB3 | rs47440485 | 1 | 0 |
| chr18 | 43575725 | C | T | 101 | Jakmip2 | intronic | . | . | . | 18qB3 | rs51613435 | 1 | 0 |
| chr18 | 43581584 | G | A | 101 | Jakmip2 | intronic | . | . | . | 18qB3 | rs45690415 | 1 | 0 |
| chr18 | 43590469 | G | T | 228 | Jakmip2 | intronic | . | . | . | 18qB3 | rs49113417 | 1 | 0 |
| chr18 | 43733571 | A | G | 43.8762 | Spink1 | exonic | synonymous SNV | Spink1:NM_009258:exon3:c.T150C:p.T50T | . | 18qB3 | . | 0 | 1 |
| chr18 | 43735322 | C | A | 228 | Spink1 | intronic | . | . | . | 18qB3 | rs31954657 | 1 | 0 |
| chr18 | 43764350 | G | T | 219 | Scgb3a2 | UTR5 | . | . | . | 18qB3 | . | 0 | 1 |
| chr18 | 43764394 | C | A | 228 | Scgb3a2 | UTR5 | . | . | . | 18qB3 | rs31950448 | 1 | 0 |
| chr18 | 43766701 | C | T | 228 | Scgb3a2 | splicing | . | . | . | 18qB3 | rs31947910 | 1 | 0 |
| chr18 | 43766808 | C | T | 228 | Scgb3a2 | exonic | synonymous SNV | Scgb3a2:NM_001289643:exon2:c.C160T:p.L54L,Scgb3a2:NM_001289644:exon2:c.C160T:p.L54L | . | 18qB3 | rs31947909 | 1 | 0 |
| chr18 | 43781005 | T | C | 153 | Gm94 | intronic | . | . | . | 18qB3 | rs29859194 | 1 | 0 |
| chr18 | 43781044 | G | A | 168 | Gm94 | intronic | . | . | . | 18qB3 | rs256393809 | 1 | 0 |
| chr18 | 43784636 | G | A | 228 | Gm94 | exonic | synonymous SNV | Gm94:NM_001033280:exon2:c.C163T:p.L55L | . | 18qB3 | rs255047412 | 1 | 0 |
| chr18 | 43784814 | G | A | 161 | Gm94 | intronic | . | . | . | 18qB3 | rs223861163 | 1 | 0 |
| chr18 | 43792552 | G | T | 107 | Gm94 | intronic | . | . | . | 18qB3 | rs257932674 | 1 | 0 |
| chr18 | 43792767 | G | A | 228 | Gm94 | intronic | . | . | . | 18qB3 | rs29669436 | 1 | 0 |
| chr18 | 43792825 | A | C | 228 | Gm94 | exonic | synonymous SNV | Gm94:NM_001033280:exon1:c.T18G:p.L6L | . | 18qB3 | rs31947700 | 1 | 0 |
| chr18 | 43792894 | A | G | 228 | . | upstream | . | . | . | 18qB3 | rs30005637 | 1 | 0 |
| chr18 | 43963092 | C | T | 157 | . | upstream | . | . | . | 18qB3 | rs30212422 | 1 | 0 |
| chr18 | 43963287 | A | G | 228 | Spink5 | UTR5 | . | . | . | 18qB3 | rs30057194 | 1 | 0 |
| chr18 | 43964364 | T | C | 204 | Spink5 | intronic | . | . | . | 18qB3 | rs30064245 | 1 | 0 |
| chr18 | 43964462 | C | A | 228 | Spink5 | exonic | synonymous SNV | Spink5:NM_001081180:exon2:c.C75A:p.G25G | . | 18qB3 | rs50199661 | 1 | 0 |
| chr18 | 43969078 | A | G | 135 | Spink5 | intronic | . | . | . | 18qB3 | rs29818330 | 1 | 0 |
| chr18 | 43977566 | A | G | 208 | Spink5 | intronic | . | . | . | 18qB3 | rs31946265 | 1 | 0 |
| chr18 | 43977750 | T | C | 228 | Spink5 | exonic | synonymous SNV | Spink5:NM_001081180:exon5:c.T387C:p.S129S | . | 18qB3 | rs6291107 | 1 | 0 |
| chr18 | 43989681 | T | A | 164 | Spink5 | intronic | . | . | . | 18qB3 | rs29551931 | 1 | 0 |
| chr18 | 44009773 | T | C | 65 | Spink5 | intronic | . | . | . | 18qB3 | . | 0 | 1 |
| chr18 | 44018613 | A | C | 109 | Spink5 | intronic | . | . | . | 18qB3 | . | 0 | 1 |
| chr18 | 44018737 | A | G | 228 | Spink5 | exonic | synonymous SNV | Spink5:NM_001081180:exon31:c.A2982G:p.G994G | . | 18qB3 | rs29625803 | 1 | 0 |
| chr18 | 44020976 | T | A | 163 | Spink5 | UTR3 | . | . | . | 18qB3 | . | 0 | 1 |
| chr18 | 44029581 | T | C | 219 | Spink14 | exonic | missense SNV | Spink14:NM_001039218:exon3:c.T101C:p.L34P | . | 18qB3 | . | 0 | 1 |
| chr18 | 44082129 | A | T | 126 | Spink6 | intronic | . | . | . | 18qB3 | . | 0 | 1 |
| chr18 | 44192551 | T | G | 179 | Spink11 | intronic | . | . | . | 18qB3 | . | 0 | 1 |
| chr18 | 44337083 | A | C | 228 | Myot | exonic | synonymous SNV | Myot:NM_001033621:exon2:c.A126C:p.I42I | . | 18qB3 | rs13465640 | 1 | 0 |
| chr18 | 44344330 | A | C | 45.0769 | Myot | intronic | . | . | . | 18qB3 | . | 0 | 1 |
| chr18 | 44405970 | A | T | 115 | Dcp2 | intronic | . | . | . | 18qB3 | . | 1 | 0 |
| chr18 | 44417494 | A | G | 228 | Dcp2 | intronic | . | . | . | 18qB3 | rs46864382 | 1 | 0 |
| chr18 | 44429902 | G | A | 228 | Mcc | exonic | synonymous SNV | Mcc:NM_001085374:exon17:c.C2472T:p.N824N,Mcc:NM_001085373:exon19:c.C2997T:p.N999N | . | 18qB3 | rs31934217 | 1 | 0 |
| chr18 | 44442836 | A | G | 228 | Mcc | intronic | . | . | . | 18qB3 | rs31935154 | 1 | 0 |
| chr18 | 44491378 | G | T | 135 | Mcc | intronic | . | . | . | 18qB3 | . | 0 | 1 |
| chr18 | 44661599 | T | C | 118 | Mcc | intronic | . | . | . | 18qB3 | rs30216300 | 1 | 0 |
| chr18 | 44676271 | C | A | 168 | Mcc,A930012L18Rik | ncRNA_exonic | . | . | . | 18qB3 | rs6228548 | 1 | 0 |
| chr18 | 44759430 | G | A | 135 | Mcc | intronic | . | . | . | 18qB3 | rs51624090 | 1 | 0 |
| chr18 | 44841027 | A | T | 33.5205 | Ythdc2 | intronic | . | . | . | 18qB3 | . | 0 | 1 |
| chr18 | 44841379 | C | T | 228 | Ythdc2 | exonic | synonymous SNV | Ythdc2:NM_001163013:exon7:c.C1107T:p.N369N | . | 18qB3 | rs48420762 | 1 | 0 |
| chr18 | 44858173 | A | G | 228 | Ythdc2 | intronic | . | . | . | 18qB3 | rs264333543 | 1 | 0 |
| chr18 | 44884955 | C | A | 162 | Ythdc2 | intronic | . | . | . | 18qB3 | rs218546662 | 1 | 0 |
| chr18 | 45070138 | A | G | 165 | . | intergenic | . | . | . | 18qB3 | rs250955286 | 1 | 0 |
| chr18 | 45070365 | C | T | 228 | . | intergenic | . | . | . | 18qB3 | rs583041063 | 1 | 0 |
| chr18 | 45070865 | G | A | 228 | . | intergenic | . | . | . | 18qB3 | rs31925049 | 1 | 0 |
| chr18 | 45090135 | C | A | 116 | . | intergenic | . | . | . | 18qB3 | rs235585415 | 1 | 0 |
| chr18 | 45146875 | G | A | 136 | . | intergenic | . | . | . | 18qB3 | rs31912587 | 1 | 0 |
| chr18 | 45146964 | G | C | 228 | . | intergenic | . | . | 205 | 18qB3 | rs31911893 | 1 | 0 |
| chr18 | 45285419 | G | A | 183 | Kcnn2 | intronic | . | . | . | 18qB3 | rs29677341 | 1 | 0 |
| chr18 | 45285961 | A | G | 228 | Kcnn2 | intronic | . | . | . | 18qB3 | rs29921308 | 1 | 0 |
| chr18 | 45285971 | C | T | 228 | Kcnn2 | intronic | . | . | . | 18qB3 | rs29624880 | 1 | 0 |
| chr18 | 45418756 | T | C | 159 | Kcnn2 | intronic | . | . | . | 18qB3 | rs31967709 | 1 | 0 |
| chr18 | 45418878 | C | G | 228 | Kcnn2 | intronic | . | . | . | 18qB3 | rs31967708 | 1 | 0 |
| chr18 | 45418923 | G | T | 228 | Kcnn2 | intronic | . | . | . | 18qB3 | rs31967707 | 1 | 0 |
| chr18 | 45505862 | T | C | 88 | Kcnn2 | intronic | . | . | . | 18qB3 | . | 0 | 1 |
| chr18 | 45506134 | G | C | 103 | Kcnn2 | intronic | . | . | . | 18qB3 | . | 1 | 0 |
| chr18 | 45506173 | G | A | 139 | Kcnn2 | intronic | . | . | . | 18qB3 | . | 1 | 0 |
| chr18 | 45560490 | G | A | 68 | Kcnn2 | exonic | missense SNV | Kcnn2:NM_080465:exon1:c.G337A:p.G113S,Kcnn2:NM_001312905:exon4:c.G1162A:p.G388S | 1328 | 18qB3 | . | 0 | 1 |
| chr18 | 45655073 | G | A | 228 | Kcnn2 | intronic | . | . | . | 18qC | rs29808041 | 1 | 0 |
| chr18 | 45655151 | A | G | 228 | Kcnn2 | intronic | . | . | . | 18qC | rs29580954 | 1 | 0 |
| chr18 | 45655210 | A | G | 228 | Kcnn2 | exonic | synonymous SNV | Kcnn2:NM_080465:exon4:c.A999G:p.Q333Q,Kcnn2:NM_001312905:exon7:c.A1824G:p.Q608Q | . | 18qC | rs31966874 | 1 | 0 |
| chr18 | 45655430 | T | C | 228 | Kcnn2 | intronic | . | . | . | 18qC | rs31966033 | 1 | 0 |
| chr18 | 45685310 | G | A | 228 | A330093E20Rik,Kcr | exonic | synonymous SNV | Kcnn2:NM_080465:exon8:c.G1473A:p.L491L,Kcnn2:NM_001312905:exon11:c.G2298A:p.L766L | . | 18qC | rs30217102 | 1 | 0 |
| chr18 | 45685572 | A | C | 202 | Kcnn2,A330093E20F | ncRNA_intronic | . | . | . | 18qC | . | 0 | 1 |
| chr18 | 46169448 | C | T | 228 | Trim36 | intronic | . | . | . | 18qC | . | 1 | 0 |
| chr18 | 46212209 | C | A | 39.528 | Trim36 | intronic | . | . | 303 | 18qC | . | 0 | 1 |
| chr18 | 46560825 | G | T | 48.149 | Ticam2 | exonic | missense SNV | Ticam2:NM_173394:exon3:c.C194A:p.A65E | . | 18qC | . | 0 | 1 |
| chr18 | 46560826 | C | T | 74 | Ticam2 | exonic | missense SNV | Ticam2:NM_173394:exon3:c.G193A:p.A65T | . | 18qC | . | 0 | 1 |
| chr18 | 46942150 | C | A | 220 | . | intergenic | . | . | . | 18qC | . | 0 | 1 |
| chr18 | 48047829 | A | C | 72 | Eno1b,G630055G22F | exonic | missense SNV | Eno1b:NM_001025388:exon2:c.A1073C:p.K358T | . | 18qC | . | 0 | 1 |
| chr18 | 49846618 | T | C | 222 | Dmxl1 | exonic | missense SNV | Dmxl1:NM_001081371:exon4:c.T319C:p.F107L | . | 18qD1 | . | 0 | 1 |
| chr18 | 49867926 | A | C | 222 | Dmxl1 | intronic | . | . | . | 18qD1 | . | 0 | 1 |
| chr18 | 49894793 | G | T | 101 | Dmxl1 | intronic | . | . | . | 18qD1 | . | 1 | 0 |
| chr18 | 49917500 | G | C | 66 | Dmxl1 | intronic | . | . | . | 18qD1 | . | 0 | 1 |
| chr18 | 49934757 | G | C | 228 | Dmxl1 | intronic | . | . | . | 18qD1 | rs108293061 | 1 | 0 |
| chr18 | 50144902 | C | G | 74 | Hsd17b4 | intronic | . | . | . | 18qD1 | . | 0 | 1 |
| chr18 | 50220345 | T | A | 228 | . | intergenic | . | . | . | 18qD1 | rs51047014 | 1 | 0 |
| chr18 | 52488353 | A | C | 168 | Srfbp1 | exonic | missense SNV | Srfbp1:NM_026040:exon6:c.A485C:p.K162T | . | 18qD1 | rs265206682 | 0 | 1 |
| chr18 | 52488399 | G | C | 222 | Srfbp1 | exonic | missense SNV | Srfbp1:NM_026040:exon6:c.G531C:p.R177S | . | 18qD1 | rs48137619 | 0 | 1 |
| chr18 | 52488518 | G | A | 222 | Srfbp1 | exonic | missense SNV | Srfbp1:NM_026040:exon6:c.G650A:p.R217K | . | 18qD1 | rs46726272 | 0 | 1 |
| chr18 | 52488614 | A | T | 222 | Srfbp1 | exonic | missense SNV | Srfbp1:NM_026040:exon6:c.A746T:p.E249V | . | 18qD1 | rs49667517 | 0 | 1 |
| chr18 | 52490494 | T | C | 221 | Srfbp1 | exonic | missense SNV | Srfbp1:NM_026040:exon8:c.T1192C:p.Y398H | . | 18qD1 | rs50025930 | 0 | 1 |
| chr18 | 52490692 | A | T | 222 | Srfbp1 | UTR3 | . | . | . | 18qD1 | rs258771142 | 0 | 1 |
| chr18 | 52490786 | T | A | 113 | . | downstream | . | . | . | 18qD1 | . | 0 | 1 |
| chr18 | 52490874 | G | A | 43.9904 | . | downstream | . | . | . | 18qD1 | rs387845807 | 0 | 1 |
| chr18 | 53218447 | A | C | 220 | Snx2 | intronic | . | . | . | 18qD1 | . | 0 | 1 |
| chr18 | 53406844 | T | A | 191 | Ppic | UTR3 | . | . | . | 18qD1 | . | 0 | 1 |
| chr18 | 55745328 | T | A | 81 | . | intergenic | . | . | . | 18qD3 | . | 0 | 1 |
| chr18 | 56474168 | A | G | 190 | Gramd3 | intronic | . | . | . | 18qD3 | . | 0 | 1 |
| chr18 | 58027833 | G | A | 137 | Fbn2 | exonic | synonymous SNV | Fbn2:NM_010181:exon56:c.C7101T:p.S2367S | . | 18qD3 | . | 0 | 1 |
| chr18 | 58037885 | T | C | 228 | Fbn2 | intronic | . | . | . | 18qD3 | rs46801469 | 1 | 0 |
| chr18 | 59072357 | A | G | 222 | A730017C20Rik | exonic | synonymous SNV | A730017C20Rik:NM_173759:exon2:c.A342G:p.S114S,A730017C20Rik:NM_001167925:exon3:c.A393G:p.S131 | . | 18qD3 | . | 0 | 1 |
| chr18 | 59408972 | C | T | 34.8945 | Chsy3 | exonic | missense SNV | Chsy3:NM_001081328:exon3:c.C1181T:p.T394M | . | 18qD3 | . | 0 | 1 |
| chr18 | 59409031 | C | T | 179 | Chsy3 | exonic | missense SNV | Chsy3:NM_001081328:exon3:c.C1240T:p.R414C | . | 18qD3 | . | 0 | 1 |
| chr18 | 60426608 | G | A | 137 | . | intergenic | . | . | . | 18qD3 | . | 0 | 1 |
| chr18 | 60602823 | C | A | 67 | Synpo | exonic | missense SNV | Synpo:NM_001109975:exon2:c.G1333T:p.A445S,Synpo:NM_177340:exon2:c.G1333T:p.A445S | . | 18qD3 | . | 0 | 1 |
| chr18 | 60776437 | A | T | 222 | Rps14 | exonic | missense SNV | Rps14:NM_020600:exon2:c.A35T:p.E12V | . | 18qE1 | . | 0 | 1 |
| chr18 | 60776440 | A | T | 222 | Rps14 | exonic | missense SNV | Rps14:NM_020600:exon2:c.A38T:p.Q13L | . | 18qE1 | . | 0 | 1 |
| chr18 | 61522617 | T | C | 228 | Arhgef37 | exonic | missense SNV | Arhgef37:NM_177828:exon3:c.A269G:p.Q90R | . | 18qE1 | . | 1 | 0 |
| chr18 | 61819871 | G | T | 228 | Ablim3 | exonic | missense SNV | Ablim3:NM_198649:exon12:c.C1121A:p.P374H,Ablim3:NM_001164491:exon13:c.C1121A:p.P374H | . | 18qE1 | . | 1 | 0 |
| chr18 | 62506724 | T | G | 222 | Fbxo38 | exonic | missense SNV | Fbxo38:NM_134136:exon19:c.A3068C:p.K1023T | . | 18qE1 | . | 0 | 1 |
| chr18 | 62614717 | G | A | 164 | Spink13,Spink10 | intronic | . | . | . | 18qE1 | . | 1 | 0 |
| chr18 | 62650927 | A | C | 81 | Spink10,Spink13 | splicing | . | . | . | 18qE1 | . | 0 | 1 |
| chr18 | 63042532 | A | G | 221 | Piezo2 | exonic | missense SNV | Piezo2:NM_001039485:exon39:c.T5777C:p.M1926T | . | 18qE1 | . | 0 | 1 |
| chr18 | 63544382 | C | A | 228 | . | intergenic | . | . | . | 18qE1 | . | 1 | 0 |
| chr18 | 63738720 | T | A | 222 | Wdr7 | intronic | . | . | . | 18qE1 | . | 0 | 1 |
| chr18 | 64428602 | T | C | 102 | . | intergenic | . | . | 329 | 18qE1 | . | 0 | 1 |
| chr18 | 64539428 | C | A | 30.1627 | Atp8b1 | intronic | . | . | . | 18qE1 | . | 0 | 1 |
| chr18 | 65074695 | G | A | 120 | Nedd4l | intronic | . | . | . | 18qE1 | rs37922811 | 0 | 1 |
| chr18 | 65685411 | G | A | 153 | Zfp532 | intronic | . | . | . | 18qE1 | . | 1 | 0 |
| chr18 | 66085017 | C | A | 62 | Ccbe1 | intronic | . | . | . | 18qE1 | . | 0 | 1 |
| chr18 | 67318636 | C | T | 206 | Impa2 | UTR3 | . | . | . | 18qE1 | . | 0 | 1 |
| chr18 | 67850762 | G | T | 222 | Cep192 | intronic | . | . | . | 18qE1 | rs108431865 | 0 | 1 |
| chr18 | 68254634 | G | C | 130 | Ldlrad4 | UTR3 | . | . | . | 18qE2 | . | 0 | 1 |
| chr18 | 70242236 | C | A | 91 | Dynap | intronic | . | . | . | 18qE2 | . | 0 | 1 |
| chr18 | 71306052 | G | A | 220 | Dcc | exonic | missense SNV | Dcc:NM_007831:exon26:c.C3818T:p.P1273L | . | 18qE2 | . | 0 | 1 |

| | | | | | | | | | | | | | |
|---|---|---|---|---|---|---|---|---|---|---|---|---|---|
| chr18 | 71378485 | T | A | 74 | Dcc | intronic | . | . | . | 18qE2 | . | 0 | 1 |
| chr18 | 71378486 | C | T | 66 | Dcc | intronic | . | . | . | 18qE2 | . | 0 | 1 |
| chr18 | 71384176 | C | G | 220 | Dcc | exonic | missense SNV | Dcc:NM_007831:exon16:c.G2407C:p.G803R | . | 18qE2 | . | 0 | 1 |
| chr18 | 71420109 | T | G | 43.4258 | Dcc | intronic | . | . | . | 18qE2 | . | 0 | 1 |
| chr18 | 71808936 | A | T | 109 | Dcc | intronic | . | . | . | 18qE2 | . | 0 | 1 |
| chr18 | 72350482 | G | C | 168 | Dcc | UTR5 | . | . | . | 18qE2 | . | 0 | 1 |
| chr18 | 74283236 | T | C | 159 | Cfap53 | exonic | synonymous SNV | Cfap53:NM_028948:exon1:c.T39C:p.F13F | . | 18qE2 | . | 0 | 1 |
| chr18 | 74654223 | G | T | 129 | Myo5b | intronic | . | . | . | 18qE2 | . | 0 | 1 |
| chr18 | 74947223 | G | A | 228 | Lipg | intronic | . | . | . | 18qE2 | . | 1 | 0 |
| chr18 | 76014480 | C | A | 222 | Zbtb7c | intronic | . | . | . | 18qE3 | . | 0 | 1 |
| chr18 | 77462719 | A | T | 170 | Rnf165 | intronic | . | . | . | 18qE3 | rs29727158 | 1 | 0 |
| chr18 | 77462720 | G | A | 169 | Rnf165 | intronic | . | . | . | 18qE3 | rs29782008 | 1 | 0 |
| chr18 | 77466527 | C | T | 102 | Rnf165 | intronic | . | . | . | 18qE3 | rs30120085 | 1 | 0 |
| chr18 | 77466865 | C | T | 228 | Rnf165 | intronic | . | . | . | 18qE3 | rs30173086 | 1 | 0 |
| chr18 | 77475459 | T | C | 228 | Rnf165 | intronic | . | . | . | 18qE3 | rs52308334 | 1 | 0 |
| chr18 | 77475579 | T | C | 228 | Rnf165 | exonic | missense SNV | Rnf165:NM_001164504:exon3:c.A383G:p.N128S | . | 18qE3 | rs38117271 | 1 | 0 |
| chr18 | 77484499 | A | C | 223 | Rnf165 | intronic | . | . | . | 18qE3 | rs47404530 | 1 | 0 |
| chr18 | 77513617 | C | T | 176 | Rnf165 | intronic | . | . | . | 18qE3 | rs29557948 | 1 | 0 |
| chr18 | 77618377 | G | T | 205 | . | intergenic | . | . | . | 18qE3 | rs36870845 | 1 | 0 |
| chr18 | 77715211 | T | C | 157 | 4930465K10Rik | ncRNA_exonic | . | . | . | 18qE3 | rs49542901 | 1 | 0 |
| chr18 | 77870173 | G | T | 156 | Pstpip2 | intronic | . | . | . | 18qE3 | . | 1 | 0 |
| chr18 | 77874445 | A | C | 228 | Pstpip2 | intronic | . | . | . | 18qE3 | rs47094517 | 1 | 0 |
| chr18 | 77981019 | G | A | 112 | Epg5 | exonic | synonymous SNV | Epg5:NM_001195633:exon18:c.G3300A:p.R1100R | . | 18qE3 | . | 0 | 1 |
| chr18 | 77986475 | A | G | 221 | Epg5 | exonic | missense SNV | Epg5:NM_001195633:exon23:c.A4052G:p.E1351G | . | 18qE3 | . | 0 | 1 |
| chr18 | 78859832 | T | C | 222 | Setbp1 | exonic | missense SNV | Setbp1:NM_053099:exon4:c.A619G:p.S207G | . | 18qE3 | . | 0 | 1 |
| chr18 | 79110857 | T | G | 222 | . | intergenic | . | . | . | 18qE3 | . | 0 | 1 |
| chr18 | 80972247 | G | C | 171 | Sall3 | exonic | missense SNV | Sall3:NM_178280:exon2:c.C2465G:p.S822W | 561 | 18qE3 | . | 0 | 1 |
| chr18 | 82579025 | C | T | 132 | Mbp | exonic | synonymous SNV | Mbp:NM_001025258:exon4:c.C399T:p.D133D,Mbp:NM_001025254:exon5:c.C477T:p.D159D,Mbp:NM_0010252 | . | 18qE3 | . | 0 | 1 |
| chr18 | 83064471 | T | A | 228 | . | intergenic | . | . | . | 18qE3 | . | 1 | 0 |
| chr18 | 83483936 | G | T | 171 | . | intergenic | . | . | . | 18qE3 | . | 1 | 0 |
| chr18 | 84632011 | T | C | 222 | Cndp1 | exonic | missense SNV | Cndp1:NM_177450:exon6:c.A556G:p.M186V | . | 18qE4 | . | 0 | 1 |
| chr18 | 84636349 | T | G | 60 | Cndp1 | intronic | . | . | . | 18qE4 | . | 0 | 1 |
| chr18 | 84712510 | C | G | 159 | . | intergenic | . | . | . | 18qE4 | . | 0 | 1 |
| chr18 | 84851304 | T | A | 77 | . | upstream | . | . | . | 18qE4 | . | 0 | 1 |
| chr18 | 84947472 | G | A | 163 | Fbxo15,Timm21 | UTR3 | . | . | . | 18qE4 | rs29882288 | 1 | 0 |
| chr18 | 89207401 | C | A | 117 | Cd226 | intronic | . | . | . | 18qE4 | . | 0 | 1 |
| chr18 | 89560229 | A | G | 30.2213 | Dok6 | intronic | . | . | . | 18qE4 | . | 0 | 1 |
| chr19 | 3357964 | G | T | 43.9771 | Cpt1a | intronic | . | . | . | 19qA | . | 0 | 1 |
| chr19 | 3394078 | C | A | 212 | Mt15 | intronic | . | . | . | 19qA | . | 0 | 1 |
| chr19 | 3394079 | A | T | 215 | Mt15 | intronic | . | . | . | 19qA | . | 0 | 1 |
| chr19 | 3804327 | G | T | 83 | Suv420h1 | intronic | . | . | . | 19qA | . | 0 | 1 |
| chr19 | 3889088 | G | T | 222 | Chka | exonic | stopgain | Chka:NM_013490:exon7:c.G928T:p.E310X,Chka:NM_001271496:exon8:c.G982T:p.E328X | . | 19qA | . | 0 | 1 |
| chr19 | 3918821 | C | T | 221 | Aldh3b1 | exonic | missense SNV | Aldh3b1:NM_026316:exon7:c.G731A:p.C244Y | . | 19qA | . | 0 | 1 |
| chr19 | 3979373 | C | A | 143 | Aldh3b2 | exonic | missense SNV | Aldh3b2:NM_001177438:exon7:c.C745A:p.R249S | . | 19qA | . | 0 | 1 |
| chr19 | 4082658 | A | T | 81 | Cabp2 | UTR5 | . | . | . | 19qA | . | 0 | 1 |
| chr19 | 4085027 | C | A | 114 | Cabp2 | intronic | . | . | . | 19qA | . | 0 | 1 |
| chr19 | 4157146 | T | A | 119 | Rps6kb2 | exonic | missense SNV | Rps6kb2:NM_021485:exon15:c.A1322T:p.E441V | . | 19qA | . | 0 | 1 |
| chr19 | 4190007 | G | T | 186 | Tbc1d10c | intronic | . | . | . | 19qA | . | 0 | 1 |
| chr19 | 4270744 | C | A | 222 | Ankrd13d | intronic | . | . | . | 19qA | . | 0 | 1 |
| chr19 | 4294986 | C | A | 103 | Adrbk1 | intronic | . | . | . | 19qA | . | 0 | 1 |
| chr19 | 4350166 | A | C | 222 | Kdm2a | intronic | . | . | . | 19qA | . | 0 | 1 |
| chr19 | 4867823 | T | A | 121 | Actn3 | exonic | missense SNV | Actn3:NM_013456:exon10:c.A1115T:p.K372M | 225 | 19qA | . | 0 | 1 |
| chr19 | 4932948 | A | T | 103 | Peli3 | intronic | . | . | . | 19qA | . | 0 | 1 |
| chr19 | 5275064 | C | A | 221 | Sf3b2 | exonic | synonymous SNV | Sf3b2:NM_030109:exon19:c.G2379T:p.T793T | . | 19qA | . | 0 | 1 |
| chr19 | 5348517 | C | A | 222 | Cst6 | exonic | synonymous SNV | Cst6:NM_028623:exon2:c.G246T:p.V82V | . | 19qA | . | 0 | 1 |
| chr19 | 5455103 | T | C | 222 | Fosl1,Ccdc85b | exonic | missense SNV | Fosl1:NM_010235:exon4:c.T617C:p.L206P | . | 19qA | . | 0 | 1 |
| chr19 | 5483956 | T | A | 222 | Mus81 | exonic | missense SNV | Mus81:NM_027877:exon12:c.A1246T:p.T416S | . | 19qA | . | 0 | 1 |
| chr19 | 5716603 | C | G | 196 | Ehbp1l1 | intronic | . | . | . | 19qA | . | 0 | 1 |
| chr19 | 5880408 | A | T | 222 | Slc25a45 | intronic | . | . | . | 19qA | . | 0 | 1 |
| chr19 | 5902620 | G | C | 222 | Dpf2 | intronic | . | . | . | 19qA | . | 0 | 1 |
| chr19 | 6081778 | T | C | 76 | Zfpl1 | intronic | . | . | . | 19qA | . | 0 | 1 |
| chr19 | 6084008 | T | C | 222 | Zfpl1 | exonic | missense SNV | Zfpl1:NM_024231:exon3:c.A155G:p.N52S | . | 19qA | . | 0 | 1 |
| chr19 | 6223358 | G | T | 86 | 1700123I01Rik | intronic | . | . | . | 19qA | . | 0 | 1 |
| chr19 | 6938705 | G | A | 64 | Gpr137 | intronic | . | . | . | 19qA | . | 0 | 1 |
| chr19 | 7458559 | A | G | 157 | Rtn3 | intronic | . | . | . | 19qA | rs30989704 | 1 | 0 |
| chr19 | 8965125 | G | C | 189 | Tut1 | exonic | synonymous SNV | Tut1:NM_197993:exon9:c.G1575C:p.V525V | . | 19qA | . | 0 | 1 |
| chr19 | 9216620 | C | T | 221 | . | intergenic | . | . | . | 19qA | . | 0 | 1 |
| chr19 | 9883158 | G | C | 209 | Incenp | intronic | . | . | . | 19qA | . | 0 | 1 |
| chr19 | 9893668 | G | A | 221 | Incenp | exonic | missense SNV | Incenp:NM_016692:exon4:c.C596T:p.S199L | . | 19qA | . | 0 | 1 |
| chr19 | 10033812 | T | G | 222 | Rab3il1 | splicing | . | . | . | 19qA | . | 0 | 1 |
| chr19 | 10479754 | C | T | 44.2299 | Ppp1r32 | intronic | . | . | . | 19qA | . | 0 | 1 |
| chr19 | 10627168 | A | T | 221 | Ddb1 | exonic | missense SNV | Ddb1:NM_015735:exon23:c.A2875T:p.I959F | . | 19qA | . | 0 | 1 |
| chr19 | 10967028 | C | A | 213 | Ms4a10 | intronic | . | . | . | 19qA | . | 0 | 1 |
| chr19 | 10967270 | C | T | 74 | Ms4a10 | intronic | . | . | . | 19qA | . | 0 | 1 |
| chr19 | 11068452 | A | T | 221 | Ms4a8a | exonic | missense SNV | Ms4a8a:NM_022430:exon6:c.T691A:p.F231I | . | 19qA | . | 0 | 1 |
| chr19 | 11091773 | T | A | 221 | . | intergenic | . | . | . | 19qA | . | 0 | 1 |
| chr19 | 11321688 | T | A | 40.9685 | Ms4a7 | intronic | . | . | . | 19qA | . | 0 | 1 |
| chr19 | 11449833 | A | C | 222 | Ms4a4b | intronic | . | . | . | 19qA | . | 0 | 1 |
| chr19 | 11450291 | A | G | 143 | Ms4a4b | intronic | . | . | . | 19qA | rs31222360 | 1 | 0 |
| chr19 | 11635637 | G | T | 127 | Ms4a3 | intronic | . | . | . | 19qA | . | 0 | 1 |
| chr19 | 11639339 | A | G | 175 | Ms4a3 | intronic | . | . | . | 19qA | . | 0 | 1 |
| chr19 | 11896334 | C | T | 222 | Olfr1420 | exonic | synonymous SNV | Olfr1420:NM_146410:exon1:c.C312T:p.F104F | . | 19qA | . | 0 | 1 |
| chr19 | 12035617 | G | A | 84 | . | downstream | . | . | . | 19qA | . | 0 | 1 |
| chr19 | 12299125 | C | T | 222 | Olfr1436,Mir466f- | exonic | synonymous SNV | Olfr1436:NM_146687:exon1:c.G6A:p.E2E | . | 19qA | . | 0 | 1 |
| chr19 | 12524977 | A | C | 221 | . | intergenic | . | . | . | 19qA | . | 0 | 1 |
| chr19 | 12648010 | C | T | 175 | Glyat | exonic | missense SNV | Glyat:NM_145935:exon4:c.C197T:p.T66I | . | 19qA | . | 0 | 1 |
| chr19 | 12650238 | T | G | 128 | Glyat | intronic | . | . | . | 19qA | . | 0 | 1 |
| chr19 | 12650239 | C | A | 127 | Glyat | intronic | . | . | . | 19qA | . | 0 | 1 |
| chr19 | 12883734 | C | A | 88 | . | upstream | . | . | . | 19qA | . | 0 | 1 |
| chr19 | 13313570 | A | T | 221 | Olfr1465 | exonic | synonymous SNV | Olfr1465:NM_001011841:exon1:c.T714A:p.T238T | . | 19qA | . | 0 | 1 |
| chr19 | 13794645 | A | C | 116 | . | downstream | . | . | . | 19qA | . | 0 | 1 |
| chr19 | 13888003 | T | G | 201 | Olfr1504 | exonic | missense SNV | Olfr1504:NM_146634:exon1:c.A206C:p.D69A | . | 19qA | . | 0 | 1 |
| chr19 | 13888060 | C | T | 203 | Olfr1504 | exonic | missense SNV | Olfr1504:NM_146634:exon1:c.G149A:p.S50N | . | 19qA | . | 0 | 1 |
| chr19 | 14960362 | A | T | 222 | . | intergenic | . | . | . | 19qA | . | 0 | 1 |
| chr19 | 15362747 | C | T | 124 | . | intergenic | . | . | . | 19qA | . | 0 | 1 |
| chr19 | 16780786 | C | T | 222 | Vps13a | UTR5 | . | . | 984 | 19qB | . | 0 | 1 |
| chr19 | 17474941 | G | C | 222 | Pcsk5 | exonic | missense SNV | Pcsk5:NM_001190483:exon26:c.C3199G:p.R1067G | . | 19qB | . | 0 | 1 |
| chr19 | 17714707 | G | T | 208 | Pcsk5 | intronic | . | . | . | 19qB | . | 0 | 1 |
| chr19 | 17836992 | C | T | 222 | Pcsk5 | exonic | missense SNV | Pcsk5:NM_001163144:exon1:c.G161A:p.R54H,Pcsk5:NM_001190483:exon1:c.G161A:p.R54H | 878 | 19qB | . | 0 | 1 |
| chr19 | 18802120 | G | T | 222 | Trpm6 | intronic | . | . | . | 19qB | . | 0 | 1 |
| chr19 | 20709288 | T | G | 192 | Aldh1a7 | intronic | . | . | . | 19qB | . | 0 | 1 |
| chr19 | 20715002 | T | G | 37.7788 | Aldh1a7 | intronic | . | . | . | 19qB | . | 0 | 1 |
| chr19 | 20941152 | C | A | 124 | Tmc1 | intronic | . | . | . | 19qB | . | 0 | 1 |
| chr19 | 21419350 | T | G | 184 | Gda | intronic | . | . | . | 19qB | . | 0 | 1 |
| chr19 | 21597894 | T | C | 79 | 1110059E24Rik | UTR3 | . | . | . | 19qB | rs13462794 | 0 | 1 |
| chr19 | 21598274 | T | C | 221 | 1110059E24Rik | exonic | synonymous SNV | 1110059E24Rik:NM_025423:exon4:c.A384G:p.R128R | . | 19qB | rs13462795 | 0 | 1 |
| chr19 | 21847861 | G | T | 221 | Tmem2 | intronic | . | . | . | 19qB | . | 0 | 1 |
| chr19 | 22987192 | C | A | 222 | Trpm3 | exonic | missense SNV | Trpm3:NM_001035243:exon24:c.C3984A:p.S1328R,Trpm3:NM_001035239:exon25:c.C4050A:p.S1350R,Trpm3 | . | 19qB | . | 0 | 1 |
| chr19 | 23164859 | T | G | 221 | Klf9 | exonic | missense SNV | Klf9:NM_010638:exon2:c.T682G:p.F228V | . | 19qB | . | 0 | 1 |
| chr19 | 23353556 | C | G | 193 | Mamdc2 | intronic | . | . | . | 19qB | . | 0 | 1 |
| chr19 | 23933821 | A | G | 222 | Apba1 | intronic | . | . | . | 19qB | . | 0 | 1 |
| chr19 | 24100728 | A | G | 187 | Tjp2 | exonic | missense SNV | Tjp2:NM_001198985:exon19:c.T2869C:p.S957P,Tjp2:NM_011597:exon19:c.T2800C:p.S934P | . | 19qB | . | 0 | 1 |
| chr19 | 24132843 | C | G | 167 | Tjp2 | intronic | . | . | . | 19qB | . | 0 | 1 |
| chr19 | 24397066 | G | A | 222 | Pip5k1b | exonic | synonymous SNV | Pip5k1b:NM_008846:exon4:c.C150T:p.R50R | . | 19qB | rs223685267 | 0 | 1 |
| chr19 | 24397261 | T | C | 203 | Pip5k1b | intronic | . | . | . | 19qB | . | 0 | 1 |
| chr19 | 24709167 | C | A | 222 | Pgm5 | intronic | . | . | . | 19qB | . | 0 | 1 |
| chr19 | 25094234 | C | G | 86 | Dock8 | intronic | . | . | . | 19qB | . | 0 | 1 |
| chr19 | 25127306 | A | C | 185 | Dock8 | intronic | . | . | . | 19qB | . | 0 | 1 |
| chr19 | 25154277 | C | A | 214 | Dock8 | exonic | missense SNV | Dock8:NM_028785:exon27:c.C3370A:p.P1124T | . | 19qB | . | 0 | 1 |
| chr19 | 25200723 | A | G | 56 | Dock8 | intronic | . | . | . | 19qB | . | 0 | 1 |
| chr19 | 25378629 | G | T | 214 | Kank1 | intronic | . | . | . | 19qB | . | 0 | 1 |
| chr19 | 25560065 | G | T | 184 | Dmrt1 | intronic | . | . | . | 19qB | . | 0 | 1 |
| chr19 | 26619916 | C | A | 222 | Smarca2 | exonic | missense SNV | Smarca2:NM_011416:exon2:c.C197A:p.P66Q | . | 19qC1 | . | 0 | 1 |
| chr19 | 26630796 | C | T | 222 | Smarca2 | exonic | missense SNV | Smarca2:NM_011416:exon4:c.C482T:p.P161L | . | 19qC1 | rs227946743 | 0 | 1 |
| chr19 | 26698568 | G | T | 222 | Smarca2 | intronic | . | . | . | 19qC1 | . | 0 | 1 |
| chr19 | 27238825 | G | C | 221 | Vldlr | exonic | missense SNV | Vldlr:NM_001161420:exon7:c.G1045C:p.D349H,Vldlr:NM_013703:exon7:c.G1045C:p.D349H | . | 19qC1 | . | 0 | 1 |
| chr19 | 27242903 | A | C | 136 | Vldlr | intronic | . | . | . | 19qC1 | . | 0 | 1 |
| chr19 | 27323702 | G | C | 219 | Kcnv2 | exonic | missense SNV | Kcnv2:NM_183179:exon1:c.G952C:p.V318L | . | 19qC1 | . | 0 | 1 |
| chr19 | 28299098 | C | T | 121 | Glis3 | intronic | . | . | . | 19qC1 | . | 0 | 1 |
| chr19 | 28531433 | C | T | 222 | Glis3 | exonic | missense SNV | Glis3:NM_001305671:exon3:c.G322A:p.G108S,Glis3:NM_175459:exon4:c.G1150A:p.G384S | . | 19qC1 | . | 0 | 1 |
| chr19 | 29579868 | A | T | 222 | Ric1 | exonic | missense SNV | Ric1:NM_001081319:exon12:c.A1352T:p.E451V | . | 19qC1 | . | 0 | 1 |
| chr19 | 29700125 | G | T | 221 | Mlana | exonic | missense SNV | Mlana:NM_029993:exon2:c.G26T:p.G9V | . | 19qC1 | . | 0 | 1 |
| chr19 | 29949844 | A | G | 89 | Il33 | intronic | . | . | . | 19qC1 | . | 0 | 1 |
| chr19 | 29955215 | C | T | 221 | Il33 | exonic | missense SNV | Il33:NM_001164724:exon6:c.C475T:p.R159C,Il33:NM_133775:exon6:c.C475T:p.R159C | . | 19qC1 | . | 0 | 1 |
| chr19 | 30581750 | T | C | 49.9427 | Prkg1 | intronic | . | . | . | 19qC1 | . | 0 | 1 |
| chr19 | 30724202 | A | G | 222 | Prkg1 | exonic | missense SNV | Prkg1:NM_001013833:exon8:c.T914C:p.V305A,Prkg1:NM_011160:exon8:c.T959C:p.V320A | . | 19qC1 | . | 0 | 1 |
| chr19 | 32074952 | C | T | 110 | Asah2 | intronic | . | . | . | 19qC1 | . | 0 | 1 |
| chr19 | 32466171 | C | T | 222 | . | intergenic | . | . | . | 19qC1 | . | 0 | 1 |
| chr19 | 32639403 | T | C | 117 | Papss2 | intronic | . | . | . | 19qC1 | . | 0 | 1 |
| chr19 | 33673782 | C | A | 169 | . | intergenic | . | . | . | 19qC1 | . | 0 | 1 |
| chr19 | 34234133 | A | G | 222 | Stambpl1 | exonic | missense SNV | Stambpl1:NM_029682:exon6:c.A593G:p.D198G | . | 19qC1 | . | 0 | 1 |
| chr19 | 34629807 | G | A | 222 | Ifit1bl2 | UTR5 | . | . | . | 19qC1 | . | 0 | 1 |
| chr19 | 34929701 | T | A | 162 | Kif20b | exonic | synonymous SNV | Kif20b:NM_183046:exon5:c.T369A:p.T123T | . | 19qC1 | . | 0 | 1 |
| chr19 | 36085198 | G | A | 222 | Rpp30 | intronic | . | . | . | 19qC2 | . | 0 | 1 |
| chr19 | 36991068 | A | G | 109 | Btaf1 | intronic | . | . | . | 19qC2 | . | 0 | 1 |
| chr19 | 37009777 | C | T | 222 | Btaf1 | intronic | . | . | . | 19qC2 | rs36425992 | 0 | 1 |
| chr19 | 37723100 | G | T | 221 | . | intergenic | . | . | . | 19qC2 | . | 0 | 1 |
| chr19 | 38175668 | C | A | 222 | Pde6c | exonic | missense SNV | Pde6c:NM_001170959:exon16:c.C1969A:p.Q657K,Pde6c:NM_033614:exon16:c.C1969A:p.Q657K | . | 19qC2 | . | 0 | 1 |
| chr19 | 38298202 | A | C | 78 | Lgi1 | intronic | . | . | . | 19qC3 | . | 0 | 1 |
| chr19 | 38305806 | A | G | 95 | Lgi1 | exonic | missense SNV | Lgi1:NM_020278:exon8:c.A1025G:p.D342G | . | 19qC3 | . | 0 | 1 |
| chr19 | 38701717 | A | C | 133 | Plce1 | intronic | . | . | . | 19qC3 | . | 0 | 1 |
| chr19 | 39460424 | A | G | 221 | Cyp2c38 | intronic | . | . | . | 19qC3 | . | 0 | 1 |
| chr19 | 40607647 | T | G | 222 | Tctn3 | exonic | synonymous SNV | Tctn3:NM_026260:exon8:c.A915C:p.T305T | . | 19qC3 | . | 0 | 1 |
| chr19 | 41077073 | G | T | 221 | Opalin | UTR5 | . | . | . | 19qC3 | . | 0 | 1 |
| chr19 | 41459768 | A | C | 221 | . | intergenic | . | . | . | 19qC3 | . | 0 | 1 |
| chr19 | 41957197 | T | G | 221 | Mms19 | exonic | missense SNV | Mms19:NM_028152:exon10:c.A793C:p.I265L | . | 19qC3 | . | 0 | 1 |
| chr19 | 42160582 | G | A | 96 | . | intergenic | . | . | . | 19qC3 | . | 0 | 1 |
| chr19 | 42323742 | C | A | 148 | Crtac1 | exonic | synonymous SNV | Crtac1:NM_145123:exon4:c.G531T:p.A177A | . | 19qC3 | . | 0 | 1 |
| chr19 | 42563045 | G | T | 221 | R3hcc1l | exonic | missense SNV | R3hcc1l:NM_177464:exon2:c.G480T:p.W160C | . | 19qC3 | . | 0 | 1 |
| chr19 | 42931790 | C | A | 56 | Hpse2 | intronic | . | . | . | 19qC3 | . | 0 | 1 |
| chr19 | 43294472 | C | A | 88 | Hpse2 | intronic | . | . | . | 19qC3 | . | 0 | 1 |
| chr19 | 43465295 | C | A | 221 | Cnnm1 | intronic | . | . | . | 19qC3 | . | 0 | 1 |
| chr19 | 43472392 | A | G | 222 | Cnnm1 | exonic | missense SNV | Cnnm1:NM_031396:exon6:c.A2144G:p.K715R | . | 19qC3 | . | 0 | 1 |
| chr19 | 43491686 | C | G | 137 | Cnnm1 | intronic | . | . | . | 19qC3 | . | 0 | 1 |
| chr19 | 43507516 | C | A | 185 | Got1 | intronic | . | . | . | 19qC3 | . | 0 | 1 |
| chr19 | 43612561 | G | T | 158 | Nkx2-3 | exonic | stopgain | Nkx2-3:NM_008699:exon1:c.G61T:p.E21X | . | 19qC3 | . | 0 | 1 |
| chr19 | 43753001 | G | T | 154 | . | upstream | . | . | 668 | 19qC3 | . | 0 | 1 |
| chr19 | 43796781 | C | T | 99 | Abcc2 | intronic | . | . | . | 19qC3 | . | 0 | 1 |
| chr19 | 43810361 | C | T | 134 | Abcc2 | intronic | . | . | . | 19qC3 | . | 0 | 1 |
| chr19 | 44191866 | A | G | 48.7322 | Pkd2l1 | intronic | . | . | . | 19qC3 | . | 0 | 1 |
| chr19 | 44302862 | G | A | 101 | Scd2 | intronic | . | . | . | 19qC3 | . | 0 | 1 |
| chr19 | 44796419 | C | A | 164 | Pax2 | intronic | . | . | . | 19qC3 | . | 0 | 1 |
| chr19 | 45742494 | G | A | 135 | Fgf8 | exonic | missense SNV | Fgf8:NM_010205:exon2:c.C91T:p.R31W | 499 | 19qC3 | . | 0 | 1 |
| chr19 | 45966363 | G | A | 104 | 9130011E15Rik | intronic | . | . | . | 19qC3 | . | 0 | 1 |
| chr19 | 46285613 | C | A | 219 | Gbf1 | intronic | . | . | . | 19qC3 | . | 0 | 1 |
| chr19 | 46306829 | C | T | 222 | Nfkb2 | exonic | missense SNV | Nfkb2:NM_001177370:exon4:c.C203T:p.A68V,Nfkb2:NM_019408:exon4:c.C203T:p.A68V,Nfkb2:NM_001177 | . | 19qC3 | . | 0 | 1 |
| chr19 | 46308188 | G | A | 222 | Nfkb2 | exonic | missense SNV | Nfkb2:NM_001177370:exon9:c.G782A:p.R261Q,Nfkb2:NM_019408:exon9:c.G782A:p.R261Q,Nfkb2:NM_0011 | . | 19qC3 | . | 0 | 1 |
| chr19 | 47826081 | G | A | 157 | Cfap43 | intronic | . | . | . | 19qD1 | . | 0 | 1 |
| chr19 | 47944162 | G | T | 222 | Cfap58 | exonic | missense SNV | Cfap58:NM_001163267:exon3:c.G364T:p.A122S | . | 19qD1 | . | 0 | 1 |
| chr19 | 47962324 | C | G | 107 | Cfap58 | intronic | . | . | . | 19qD1 | . | 0 | 1 |
| chr19 | 48026818 | C | T | 222 | Cfap58 | exonic | synonymous SNV | Cfap58:NM_001163267:exon16:c.C2355T:p.R785R | . | 19qD1 | . | 0 | 1 |
| chr19 | 48764316 | A | C | 221 | Sorcs3 | intronic | . | . | . | 19qD1 | . | 0 | 1 |
| chr19 | 48796651 | G | T | 47.3152 | Sorcs3 | intronic | . | . | . | 19qD1 | . | 0 | 1 |
| chr19 | 50190163 | G | T | 221 | Sorcs1 | exonic | synonymous SNV | Sorcs1:NM_001252501:exon19:c.C2520A:p.G840G,Sorcs1:NM_001290356:exon19:c.C2520A:p.G840G,Sorcs | . | 19qD1 | . | 0 | 1 |
| chr19 | 53384694 | T | A | 189 | Smndc1 | intronic | . | . | . | 19qD2 | . | 0 | 1 |

| | | | | | | | | | | | | | |
|---|---|---|---|---|---|---|---|---|---|---|---|---|---|
| chr19 | 53618895 | G | T | 175 | Smc3 | intronic | . | . | . | 19qD2 | rs48699043 | 1 | 0 |
| chr19 | 53618897 | A | G | 183 | Smc3 | intronic | . | . | . | 19qD2 | rs46734564 | 1 | 0 |
| chr19 | 53853597 | G | C | 221 | Rbm20 | exonic | missense SNV | Rbm20:NM_001170847:exon12:c.G3329C:p.W1110S | . | 19qD2 | . | 0 | 1 |
| chr19 | 53944472 | A | T | 152 | Shoc2,Bbip1 | UTR5 | . | . | 1746 | 19qD2 | rs47782010 | 1 | 0 |
| chr19 | 55097719 | G | A | 126 | Gpam | intronic | . | . | . | 19qD2 | . | 0 | 1 |
| chr19 | 55222112 | C | A | 104 | Gucy2g | intronic | . | . | . | 19qD2 | . | 0 | 1 |
| chr19 | 55225976 | G | C | 222 | Gucy2g | exonic | synonymous SNV | Gucy2g:NM_001081076:exon9:c.C1719G:p.P573P | . | 19qD2 | . | 0 | 1 |
| chr19 | 55235455 | C | G | 201 | Gucy2g | intronic | . | . | . | 19qD2 | . | 0 | 1 |
| chr19 | 56311137 | G | A | 222 | Habp2 | exonic | missense SNV | Habp2:NM_146101:exon5:c.G355A:p.G119S | . | 19qD2 | . | 0 | 1 |
| chr19 | 56319282 | T | G | 128 | Habp2 | intronic | . | . | . | 19qD2 | . | 0 | 1 |
| chr19 | 56434031 | C | T | 136 | Casp7 | intronic | . | . | . | 19qD2 | . | 0 | 1 |
| chr19 | 56436878 | C | G | 222 | Casp7 | intronic | . | . | . | 19qD2 | . | 0 | 1 |
| chr19 | 56797529 | C | T | 190 | Ccdc186 | exonic | synonymous SNV | Ccdc186:NM_170757:exon11:c.G2073A:p.Q691Q | . | 19qD2 | . | 0 | 1 |
| chr19 | 56881065 | G | T | 75 | Vwa2 | intronic | . | . | . | 19qD2 | . | 0 | 1 |
| chr19 | 57691626 | T | G | 89 | Atrnl1 | intronic | . | . | . | 19qD2 | . | 0 | 1 |
| chr19 | 58730294 | T | G | 175 | Pnliprp1 | intronic | . | . | . | 19qD2 | . | 0 | 1 |
| chr19 | 58771455 | A | C | 222 | Pnliprp2 | exonic | missense SNV | Pnliprp2:NM_011128:exon9:c.A1097C:p.N366T | . | 19qD2 | . | 0 | 1 |
| chr19 | 59035030 | C | T | 221 | Shtn1 | intronic | . | . | . | 19qD3 | . | 0 | 1 |
| chr19 | 60079570 | A | G | 222 | . | intergenic | . | . | . | 19qD3 | . | 0 | 1 |
| chr19 | 60467618 | C | T | 222 | Prlhr | exonic | missense SNV | Prlhr:NM_201615:exon1:c.G509A:p.R170H | 686 | 19qD3 | . | 0 | 1 |
| chr19 | 61090152 | G | T | 221 | Zfp950,Grk5 | intronic | . | . | . | 19qD3 | . | 0 | 1 |
| chr2 | 3408947 | A | G | 35.4733 | . | downstream | . | . | . | 2qA1 | . | 0 | 1 |
| chr2 | 3437035 | T | C | 222 | Dclre1c | exonic | missense SNV | Dclre1c:NM_001302674:exon4:c.T23C:p.L8P,Dclre1c:NM_001110214:exon6:c.T409C:p.F137L,Dclre1c:NM | . | 2qA1 | . | 0 | 1 |
| chr2 | 4643617 | C | T | 165 | Prpf18 | intronic | . | . | . | 2qA1 | . | 0 | 1 |
| chr2 | 4834452 | C | G | 221 | . | intergenic | . | . | . | 2qA1 | . | 0 | 1 |
| chr2 | 5856074 | T | G | 221 | Nudt5 | intronic | . | . | . | 2qA1 | . | 0 | 1 |
| chr2 | 5867956 | A | G | 160 | Nudt5 | intronic | . | . | . | 2qA1 | . | 0 | 1 |
| chr2 | 6113840 | A | C | 222 | Proser2 | intronic | . | . | . | 2qA1 | . | 0 | 1 |
| chr2 | 6133593 | T | A | 222 | Gm10857 | ncRNA_exonic | . | . | . | 2qA1 | . | 0 | 1 |
| chr2 | 6608113 | T | G | 75 | Celf2 | intronic | . | . | . | 2qA1 | . | 0 | 1 |
| chr2 | 9883460 | T | G | 23.4659 | 4930412O13Rik,923 | ncRNA_intronic | . | . | . | 2qA1 | . | 0 | 1 |
| chr2 | 10106683 | G | A | 218 | Itih2 | exonic | synonymous SNV | Itih2:NM_010582:exon12:c.C1443T:p.Y481Y | . | 2qA1 | . | 0 | 1 |
| chr2 | 10127804 | T | C | 222 | Itih2 | exonic | missense SNV | Itih2:NM_010582:exon3:c.A175G:p.S59G | . | 2qA1 | . | 0 | 1 |
| chr2 | 10129092 | G | A | 47.238 | Itih2 | intronic | . | . | . | 2qA1 | rs225615265 | 0 | 1 |
| chr2 | 10130552 | A | G | 87 | Itih2 | exonic | missense SNV | Itih2:NM_010582:exon1:c.T26C:p.V9A | . | 2qA1 | . | 0 | 1 |
| chr2 | 10153749 | G | A | 170 | Itih5 | exonic | synonymous SNV | Itih5:NM_172471:exon1:c.G84A:p.S28S | . | 2qA1 | . | 0 | 1 |
| chr2 | 10185721 | A | T | 157 | Itih5 | intronic | . | . | . | 2qA1 | . | 0 | 1 |
| chr2 | 10504635 | G | C | 76 | Mir297a-3,Sfmbt2 | ncRNA_intronic | . | . | . | 2qA1 | . | 0 | 1 |
| chr2 | 10894405 | T | C | 83 | . | intergenic | . | . | . | 2qA1 | . | 0 | 1 |
| chr2 | 11246503 | G | C | 55 | Prkcq | intronic | . | . | . | 2qA1 | . | 0 | 1 |
| chr2 | 11757309 | C | G | 98 | Fbxo18 | intronic | . | . | . | 2qA1 | . | 0 | 1 |
| chr2 | 11778820 | G | A | 214 | Ankrd16 | exonic | missense SNV | Ankrd16:NM_177268:exon1:c.G232A:p.A78T | 270 | 2qA1 | . | 0 | 1 |
| chr2 | 12351028 | T | G | 221 | Fam188a | intronic | . | . | . | 2qA1 | . | 0 | 1 |
| chr2 | 13580129 | G | C | 222 | Vim | exonic | missense SNV | Vim:NM_011701:exon6:c.G1120C:p.E374Q | . | 2qA1 | . | 0 | 1 |
| chr2 | 14292358 | T | G | 96 | Mrc1 | intronic | . | . | . | 2qA2 | . | 0 | 1 |
| chr2 | 14319317 | C | G | 222 | Mrc1 | exonic | missense SNV | Mrc1:NM_008625:exon24:c.C3464G:p.A1155G | . | 2qA2 | . | 0 | 1 |
| chr2 | 14692150 | A | G | 69 | Cacnb2 | intronic | . | . | . | 2qA2 | . | 0 | 1 |
| chr2 | 14692235 | A | G | 138 | Cacnb2 | intronic | . | . | . | 2qA2 | rs27132664 | 0 | 1 |
| chr2 | 14692316 | A | G | 184 | Cacnb2 | intronic | . | . | . | 2qA2 | . | 0 | 1 |
| chr2 | 14971628 | C | A | 222 | Cacnb2 | exonic | missense SNV | Cacnb2:NM_001252533:exon7:c.C709A:p.L237M,Cacnb2:NM_001309519:exon7:c.C727A:p.L243M,Cacnb2:NM | . | 2qA2 | . | 0 | 1 |
| chr2 | 15127846 | T | C | 222 | . | intergenic | . | . | . | 2qA2 | . | 0 | 1 |
| chr2 | 16074557 | A | C | 78 | Malrd1 | intronic | . | . | . | 2qA2 | . | 0 | 1 |
| chr2 | 16638553 | A | G | 24.2924 | Plxdc2 | intronic | . | . | . | 2qA3 | . | 0 | 1 |
| chr2 | 16660997 | A | G | 72 | Plxdc2 | intronic | . | . | . | 2qA3 | . | 0 | 1 |
| chr2 | 16751447 | A | G | 75 | Plxdc2 | UTR3 | . | . | . | 2qA3 | . | 0 | 1 |
| chr2 | 17382970 | A | C | 222 | Nebl | intronic | . | . | . | 2qA3 | . | 0 | 1 |
| chr2 | 19238904 | C | A | 23.9133 | Armc3 | intronic | . | . | . | 2qA3 | . | 0 | 1 |
| chr2 | 19659469 | A | G | 222 | Otud1 | UTR3 | . | . | . | 2qA3 | . | 0 | 1 |
| chr2 | 20008171 | A | T | 106 | . | intergenic | . | . | . | 2qA3 | . | 0 | 1 |
| chr2 | 22266184 | T | A | 222 | Myo3a | intronic | . | . | . | 2qA3 | . | 0 | 1 |
| chr2 | 22544697 | G | A | 142 | . | intergenic | . | . | . | 2qA3 | rs254109034 | 1 | 0 |
| chr2 | 22668402 | C | A | 183 | Gad2 | intronic | . | . | . | 2qA3 | . | 0 | 1 |
| chr2 | 22685217 | G | A | 179 | Gad2 | intronic | . | . | . | 2qA3 | . | 0 | 1 |
| chr2 | 23121174 | A | C | 96 | Mast1 | intronic | . | . | . | 2qA3 | . | 0 | 1 |
| chr2 | 23133614 | C | A | 190 | Mast1 | exonic | missense SNV | Mast1:NM_025979:exon8:c.G1096T:p.D366Y | . | 2qA3 | . | 0 | 1 |
| chr2 | 23172955 | G | T | 221 | Yme1l1 | splicing | . | . | . | 2qA3 | . | 0 | 1 |
| chr2 | 23194914 | G | A | 207 | Yme1l1 | intronic | . | . | . | 2qA3 | . | 0 | 1 |
| chr2 | 23399853 | C | A | 222 | Nxph2 | exonic | missense SNV | Nxph2:NM_008752:exon2:c.C216A:p.D72E | . | 2qA3 | . | 0 | 1 |
| chr2 | 24048607 | C | T | 182 | Hnmt | intronic | . | . | . | 2qA3 | . | 0 | 1 |
| chr2 | 24959009 | A | T | 157 | Zmynd19 | exonic | missense SNV | Zmynd19:NM_026021:exon6:c.A632T:p.H211L,Zmynd19:NM_147179:exon6:c.A515T:p.H172L | . | 2qA3 | . | 0 | 1 |
| chr2 | 25212936 | G | T | 63 | 4933433C11Rik | UTR3 | . | . | . | 2qA3 | . | 0 | 1 |
| chr2 | 25230454 | C | T | 142 | Slc34a3 | intronic | . | . | . | 2qA3 | . | 0 | 1 |
| chr2 | 25295905 | C | G | 185 | Grin1os,Grin1 | exonic | missense SNV | Grin1:NM_001177657:exon18:c.G2476C:p.A826P,Grin1:NM_008169:exon18:c.G2476C:p.A826P,Grin1:NM_( | . | 2qA3 | . | 0 | 1 |
| chr2 | 25338141 | C | T | 221 | Man1b1 | exonic | missense SNV | Man1b1:NM_001029983:exon4:c.C371T:p.P124L | . | 2qA3 | . | 0 | 1 |
| chr2 | 25354288 | G | C | 221 | Dpp7 | exonic | synonymous SNV | Dpp7:NM_031843:exon8:c.C906G:p.G302G | . | 2qA3 | . | 0 | 1 |
| chr2 | 25354293 | C | T | 221 | Dpp7 | exonic | missense SNV | Dpp7:NM_031843:exon8:c.G901A:p.V301M | . | 2qA3 | . | 0 | 1 |
| chr2 | 25376286 | G | A | 221 | Sapcd2 | intronic | . | . | . | 2qA3 | . | 0 | 1 |
| chr2 | 25558044 | G | T | 147 | Edf1 | intronic | . | . | 483 | 2qA3 | . | 0 | 1 |
| chr2 | 25566300 | C | T | 222 | Mamdc4 | exonic | missense SNV | Mamdc4:NM_001081199:exon18:c.G2182A:p.V728I | . | 2qA3 | . | 0 | 1 |
| chr2 | 25657305 | C | A | 103 | . | upstream | . | . | . | 2qA3 | . | 0 | 1 |
| chr2 | 25911105 | G | C | 86 | Kcnt1 | exonic | missense SNV | Kcnt1:NM_001302351:exon28:c.G3152C:p.G1051A,Kcnt1:NM_001145403:exon29:c.G3134C:p.G1045A,Kcnt1 | . | 2qA3 | . | 0 | 1 |
| chr2 | 26423812 | G | T | 107 | Sec16a | intronic | . | . | . | 2qA3 | . | 0 | 1 |
| chr2 | 26425547 | C | T | 222 | Sec16a | exonic | synonymous SNV | Sec16a:NM_153125:exon15:c.G5277A:p.L1759L | . | 2qA3 | . | 0 | 1 |
| chr2 | 26431226 | G | A | 222 | Sec16a | exonic | missense SNV | Sec16a:NM_153125:exon5:c.C3853T:p.P1285S | . | 2qA3 | . | 0 | 1 |
| chr2 | 27062177 | G | T | 222 | Tmem8c | UTR3 | . | . | . | 2qA3 | . | 0 | 1 |
| chr2 | 27196622 | C | A | 148 | Sardh | intronic | . | . | . | 2qA3 | . | 0 | 1 |
| chr2 | 29198810 | T | C | 222 | Ntng2 | splicing | . | . | . | 2qB | . | 0 | 1 |
| chr2 | 29939664 | G | T | 86 | Gle1 | intronic | . | . | . | 2qB | . | 0 | 1 |
| chr2 | 30237565 | G | A | 112 | 1700084E18Rik | ncRNA_exonic | . | . | 1054 | 2qB | . | 0 | 1 |
| chr2 | 30384138 | G | A | 203 | Fam73b | UTR3 | . | . | . | 2qB | . | 0 | 1 |
| chr2 | 31388350 | T | A | 145 | . | intergenic | . | . | . | 2qB | . | 0 | 1 |
| chr2 | 31518573 | A | G | 119 | Ass1,Gm5424 | ncRNA_intronic | . | . | . | 2qB | . | 0 | 1 |
| chr2 | 31801379 | T | G | 85 | Abl1 | exonic | missense SNV | Abl1:NM_001112703:exon11:c.T2909G:p.V970G,Abl1:NM_001283045:exon11:c.T2837G:p.V946G,Abl1:NM_( | . | 2qB | . | 0 | 1 |
| chr2 | 31912192 | G | A | 202 | Lamc3 | intronic | . | . | . | 2qB | . | 0 | 1 |
| chr2 | 32069661 | C | A | 206 | Fam78a | exonic | missense SNV | Fam78a:NM_175511:exon2:c.G436T:p.G146W | . | 2qB | . | 0 | 1 |
| chr2 | 32236815 | C | T | 222 | Pomt1 | intronic | . | . | 529 | 2qB | . | 0 | 1 |
| chr2 | 32298536 | C | G | 196 | Golga2 | intronic | . | . | . | 2qB | . | 0 | 1 |
| chr2 | 32416979 | C | T | 86 | Slc25a25 | intronic | . | . | . | 2qB | . | 0 | 1 |
| chr2 | 32708150 | A | G | 221 | Cdk9 | exonic | missense SNV | Cdk9:NM_130860:exon7:c.T893C:p.L298P | 215 | 2qB | . | 0 | 1 |
| chr2 | 32762764 | T | C | 222 | Ttc16 | exonic | synonymous SNV | Ttc16:NM_001290566:exon14:c.A1881G:p.Q627Q,Ttc16:NM_001290564:exon15:c.A1881G:p.Q627Q,Ttc16:N | . | 2qB | . | 0 | 1 |
| chr2 | 32776419 | G | T | 214 | Ptrh1 | exonic | missense SNV | Ptrh1:NM_178595:exon2:c.G182T:p.R61L | . | 2qB | . | 0 | 1 |
| chr2 | 32940318 | C | T | 78 | Lrsam1 | exonic | synonymous SNV | Lrsam1:NM_199302:exon16:c.G1182A:p.T394T | . | 2qB | . | 0 | 1 |
| chr2 | 32961654 | C | A | 109 | . | upstream | . | . | 568 | 2qB | . | 0 | 1 |
| chr2 | 34780713 | C | A | 190 | Rabepk | exonic | missense SNV | Rabepk:NM_145522:exon7:c.G734T:p.G245V | . | 2qB | . | 0 | 1 |
| chr2 | 35183994 | A | C | 170 | Rab14 | intronic | . | . | . | 2qB | rs33002315 | 1 | 0 |
| chr2 | 35326684 | C | A | 40.901 | Stom | intronic | . | . | . | 2qB | . | 0 | 1 |
| chr2 | 35709827 | C | G | 221 | Dab2ip | exonic | missense SNV | Dab2ip:NM_001001602:exon4:c.C430G:p.R144G,Dab2ip:NM_001290639:exon4:c.C253G:p.R85G,Dab2ip:NM | . | 2qB | . | 0 | 1 |
| chr2 | 35795274 | T | C | 150 | Ttll11 | intronic | . | . | . | 2qB | . | 0 | 1 |
| chr2 | 36453114 | C | G | 178 | Olfr340 | exonic | missense SNV | Olfr340:NM_146951:exon1:c.C528G:p.H176Q | . | 2qB | . | 0 | 1 |
| chr2 | 36479409 | C | T | 221 | Olfr341 | exonic | synonymous SNV | Olfr341:NM_146950:exon1:c.G720A:p.T240T | . | 2qB | rs222690171 | 0 | 1 |
| chr2 | 36787530 | T | C | 154 | . | downstream | . | . | . | 2qB | . | 0 | 1 |
| chr2 | 36859074 | T | C | 82 | . | downstream | . | . | . | 2qB | . | 0 | 1 |
| chr2 | 36907545 | C | G | 187 | Olfr354 | exonic | missense SNV | Olfr354:NM_146939:exon1:c.C598G:p.L200V | . | 2qB | . | 0 | 1 |
| chr2 | 36907686 | C | A | 222 | Olfr354 | exonic | missense SNV | Olfr354:NM_146939:exon1:c.C739A:p.H247N | . | 2qB | . | 0 | 1 |
| chr2 | 37106414 | G | T | 89 | . | upstream | . | . | . | 2qB | . | 0 | 1 |
| chr2 | 37201667 | G | A | 221 | Olfr365 | exonic | missense SNV | Olfr365:NM_146662:exon1:c.G425A:p.R142H | . | 2qB | . | 0 | 1 |
| chr2 | 37332274 | C | T | 222 | Olfr368 | exonic | synonymous SNV | Olfr368:NM_146374:exon1:c.C526T:p.L176L | . | 2qB | . | 0 | 1 |
| chr2 | 37332430 | G | A | 221 | Olfr368 | exonic | missense SNV | Olfr368:NM_146374:exon1:c.G682A:p.A228T | . | 2qB | . | 0 | 1 |
| chr2 | 37469630 | C | G | 202 | Rabgap1 | intronic | . | . | . | 2qB | . | 0 | 1 |
| chr2 | 37624722 | C | G | 79 | Strbp | intronic | . | . | . | 2qB | . | 0 | 1 |
| chr2 | 37790000 | A | G | 178 | Crb2 | intronic | . | . | . | 2qB | rs46336157 | 1 | 0 |
| chr2 | 38784202 | C | T | 91 | Nr6a1 | intronic | . | . | . | 2qB | . | 0 | 1 |
| chr2 | 39041433 | C | G | 222 | Golga1 | intronic | . | . | . | 2qB | . | 0 | 1 |
| chr2 | 39121224 | T | A | 221 | Scai | intronic | . | . | . | 2qB | rs264686766 | 0 | 1 |
| chr2 | 40750960 | C | A | 222 | Lrp1b | exonic | missense SNV | Lrp1b:NM_053011:exon67:c.G10471T:p.D3491Y | . | 2qB | . | 0 | 1 |
| chr2 | 40750967 | A | G | 222 | Lrp1b | exonic | synonymous SNV | Lrp1b:NM_053011:exon67:c.T10464C:p.C3488C | . | 2qB | . | 0 | 1 |
| chr2 | 40851912 | G | A | 47.2298 | Lrp1b | intronic | . | . | . | 2qB | . | 0 | 1 |
| chr2 | 40903521 | A | T | 222 | Lrp1b | intronic | . | . | . | 2qB | . | 0 | 1 |
| chr2 | 40927120 | C | T | 97 | Lrp1b | intronic | . | . | . | 2qB | . | 0 | 1 |
| chr2 | 40959150 | T | A | 43.2894 | Lrp1b | intronic | . | . | . | 2qB | . | 0 | 1 |
| chr2 | 41408787 | G | T | 94 | Lrp1b | intronic | . | . | . | 2qB | . | 0 | 1 |
| chr2 | 41458473 | C | T | 222 | Lrp1b | intronic | . | . | . | 2qB | . | 0 | 1 |
| chr2 | 41476821 | G | T | 215 | Lrp1b | intronic | . | . | . | 2qB | . | 0 | 1 |
| chr2 | 41728533 | T | C | 221 | Lrp1b | intronic | . | . | . | 2qB | . | 0 | 1 |
| chr2 | 41987375 | C | T | 29.7643 | Lrp1b | intronic | . | . | . | 2qB | rs108290206 | 0 | 1 |
| chr2 | 41987400 | G | A | 95 | Lrp1b | intronic | . | . | . | 2qB | rs108154238 | 0 | 1 |
| chr2 | 41987592 | C | T | 222 | Lrp1b | intronic | . | . | . | 2qB | rs47735788 | 0 | 1 |
| chr2 | 41987599 | C | A | 222 | Lrp1b | intronic | . | . | . | 2qB | rs46212367 | 0 | 1 |
| chr2 | 41987758 | G | A | 222 | Lrp1b | intronic | . | . | . | 2qB | rs45814573 | 0 | 1 |
| chr2 | 41987776 | G | T | 202 | Lrp1b | intronic | . | . | . | 2qB | rs49965331 | 0 | 1 |
| chr2 | 41987795 | T | C | 115 | Lrp1b | intronic | . | . | . | 2qB | rs47454070 | 0 | 1 |
| chr2 | 43671415 | A | G | 222 | Kynu | splicing | . | . | . | 2qB | . | 0 | 1 |
| chr2 | 43681234 | T | A | 95 | Kynu | intronic | . | . | . | 2qB | . | 0 | 1 |
| chr2 | 44988487 | T | C | 122 | Zeb2 | UTR3 | . | . | . | 2qB | . | 0 | 1 |
| chr2 | 48926930 | C | A | 24.0395 | Orc4 | intronic | . | . | . | 2qC1.1 | . | 0 | 1 |
| chr2 | 49535341 | C | T | 221 | Epc2 | exonic | synonymous SNV | Epc2:NM_172663:exon8:c.C1227T:p.Y409Y | . | 2qC1.1 | . | 0 | 1 |
| chr2 | 49694879 | G | T | 222 | Kif5c | intronic | . | . | . | 2qC1.1 | . | 0 | 1 |
| chr2 | 52093735 | A | G | 100 | Rif1 | intronic | . | . | . | 2qC1.1 | . | 0 | 1 |
| chr2 | 52105295 | G | T | 94 | Rif1 | intronic | . | . | . | 2qC1.1 | . | 0 | 1 |
| chr2 | 52108627 | T | C | 130 | Rif1 | intronic | . | . | . | 2qC1.1 | . | 0 | 1 |
| chr2 | 52245162 | C | A | 91 | Neb | intronic | . | . | . | 2qC1.1 | rs260075320 | 0 | 1 |
| chr2 | 52270372 | A | C | 222 | Neb | intronic | . | . | . | 2qC1.1 | . | 0 | 1 |
| chr2 | 53398284 | T | C | 119 | . | intergenic | . | . | . | 2qC1.1 | . | 0 | 1 |
| chr2 | 53398404 | C | T | 112 | . | intergenic | . | . | . | 2qC1.1 | . | 0 | 1 |
| chr2 | 53696276 | C | T | 107 | . | intergenic | . | . | . | 2qC1.1 | . | 0 | 1 |
| chr2 | 55098642 | T | A | 222 | Galnt13 | exonic | missense SNV | Galnt13:NM_173030:exon12:c.T1475A:p.I492N | . | 2qC1.1 | . | 0 | 1 |
| chr2 | 55437166 | G | C | 125 | Kcnj3 | UTR5 | . | . | 262 | 2qC1.1 | . | 0 | 1 |
| chr2 | 59348078 | C | T | 85 | Pkp4 | intronic | . | . | . | 2qC1.1 | . | 0 | 1 |
| chr2 | 59564418 | C | T | 180 | . | intergenic | . | . | . | 2qC1.1 | . | 0 | 1 |
| chr2 | 59942895 | C | T | 222 | Baz2b | intronic | . | . | . | 2qC1.1 | . | 0 | 1 |
| chr2 | 60673522 | C | A | 222 | Itgb6 | UTR5 | . | . | . | 2qC1.2 | . | 0 | 1 |
| chr2 | 62234647 | A | C | 58 | Slc4a10 | intronic | . | . | . | 2qC1.3 | . | 0 | 1 |
| chr2 | 62268447 | G | A | 222 | Slc4a10 | intronic | . | . | . | 2qC1.3 | . | 0 | 1 |
| chr2 | 62599023 | T | A | 47.3828 | Ifih1 | intronic | . | . | . | 2qC1.3 | . | 0 | 1 |
| chr2 | 62606239 | A | T | 222 | Ifih1 | exonic | missense SNV | Ifih1:NM_001164477:exon9:c.T1839A:p.D613E,Ifih1:NM_027835:exon10:c.T1986A:p.D662E | . | 2qC1.3 | . | 0 | 1 |
| chr2 | 65126140 | T | C | 222 | Cobll1 | intronic | . | . | . | 2qC1.3 | . | 0 | 1 |
| chr2 | 65520813 | C | A | 121 | Scn3a | intronic | . | . | . | 2qC1.3 | . | 0 | 1 |
| chr2 | 65525948 | C | T | 222 | Scn3a | intronic | . | . | . | 2qC1.3 | . | 0 | 1 |
| chr2 | 65713593 | G | T | 87 | Scn2a1 | intronic | . | . | . | 2qC1.3 | . | 0 | 1 |
| chr2 | 66289283 | A | C | 51 | Scn1a | intronic | . | . | . | 2qC1.3 | . | 0 | 1 |
| chr2 | 66550268 | T | G | 42.6729 | Scn9a | intronic | . | . | . | 2qC1.3 | . | 0 | 1 |
| chr2 | 67184720 | T | G | 221 | Gm1322 | exonic | missense SNV | Gm1322:NM_001033477:exon2:c.T284G:p.L95R | . | 2qC1.3 | . | 0 | 1 |
| chr2 | 67184781 | T | G | 221 | Gm1322 | exonic | missense SNV | Gm1322:NM_001033477:exon2:c.T345G:p.I115M | . | 2qC1.3 | . | 0 | 1 |
| chr2 | 67507049 | A | T | 124 | Xirp2 | exonic | missense SNV | Xirp2:NM_001024618:exon6:c.A361T:p.I121F,Xirp2:NM_001083919:exon6:c.A361T:p.I121F | . | 2qC1.3 | . | 0 | 1 |
| chr2 | 67524682 | G | A | 173 | Xirp2 | intronic | . | . | . | 2qC1.3 | rs29720088 | 1 | 0 |
| chr2 | 68119547 | A | C | 222 | B3galt1 | UTR3 | . | . | . | 2qC1.3 | . | 0 | 1 |
| chr2 | 69144812 | G | A | 221 | Nostrin | exonic | missense SNV | Nostrin:NM_181547:exon2:c.G70A:p.G24R | . | 2qC2 | . | 0 | 1 |
| chr2 | 69220024 | T | G | 197 | G6pc2 | UTR5 | . | . | . | 2qC2 | . | 0 | 1 |
| chr2 | 69394406 | T | C | 222 | Dhrs9 | exonic | missense SNV | Dhrs9:NM_175512:exon3:c.T437C:p.L146P | . | 2qC2 | . | 0 | 1 |
| chr2 | 69425643 | G | T | 187 | Lrp2 | UTR3 | . | . | . | 2qC2 | . | 0 | 1 |
| chr2 | 69702682 | C | G | 221 | Fastkd1 | exonic | missense SNV | Fastkd1:NM_177244:exon7:c.G1340C:p.W447S | . | 2qC2 | . | 0 | 1 |
| chr2 | 69867589 | T | C | 212 | Ssb | intronic | . | . | . | 2qC2 | . | 0 | 1 |
| chr2 | 69954341 | C | T | 211 | Ubr3 | exonic | missense SNV | Ubr3:NM_001303033:exon17:c.C2404T:p.H802Y,Ubr3:NM_177783:exon17:c.C2404T:p.H802Y | . | 2qC2 | . | 0 | 1 |
| chr2 | 70016339 | G | T | 221 | Ubr3 | splicing | . | . | . | 2qC2 | . | 0 | 1 |
| chr2 | 70289423 | T | C | 221 | Myo3b | exonic | synonymous SNV | Myo3b:NM_177376:exon26:c.T2910C:p.I970I | . | 2qC2 | . | 0 | 1 |

| | | | | | | | | | | | | | |
|---|---|---|---|---|---|---|---|---|---|---|---|---|---|
| chr2 | 71816568 | T | C | 136 | Mir5098,Itga6 | ncRNA_intronic | . | . | . | 2qC3 | . | 0 | 1 |
| chr2 | 73348363 | C | A | 174 | Gpr155 | intronic | . | . | . | 2qC3 | . | 0 | 1 |
| chr2 | 73959807 | G | T | 221 | . | intergenic | . | . | . | 2qC3 | . | 0 | 1 |
| chr2 | 74659164 | T | C | 100 | Evx2 | exonic | missense SNV | Evx2:NM_007967:exon1:c.A256G:p.S86G | 319 | 2qC3 | . | 0 | 1 |
| chr2 | 76027971 | T | C | 149 | Pde11a | intronic | . | . | . | 2qC3 | . | 0 | 1 |
| chr2 | 76075889 | T | C | 157 | Pde11a | intronic | . | . | . | 2qC3 | . | 0 | 1 |
| chr2 | 76137268 | C | T | 161 | Pde11a | intronic | . | . | . | 2qC3 | . | 0 | 1 |
| chr2 | 76370257 | G | A | 221 | Rbm45 | exonic | synonymous SNV | Rbm45:NM_153405:exon1:c.G129A:p.E43E | 441 | 2qC3 | . | 0 | 1 |
| chr2 | 76378701 | A | T | 221 | Rbm45 | exonic | missense SNV | Rbm45:NM_153405:exon7:c.A1013T:p.Q338L | . | 2qC3 | . | 0 | 1 |
| chr2 | 76549861 | C | A | 221 | Osbpl6 | intronic | . | . | . | 2qC3 | . | 0 | 1 |
| chr2 | 76715625 | T | G | 221 | Ttn | exonic | missense SNV | Ttn:NM_028004:exon183:c.A72462C:p.Q24154H,Ttn:NM_011652:exon304:c.A92205C:p.Q30735H | . | 2qC3 | . | 0 | 1 |
| chr2 | 76728341 | G | A | 124 | Ttn | exonic | missense SNV | Ttn:NM_028004:exon164:c.C64214T:p.S21405F,Ttn:NM_011652:exon285:c.C83957T:p.S27986F | . | 2qC3 | . | 0 | 1 |
| chr2 | 76755128 | G | A | 82 | Ttn | intronic | . | . | . | 2qC3 | . | 0 | 1 |
| chr2 | 76786746 | C | A | 202 | Ttn | exonic | missense SNV | Ttn:NM_028004:exon100:c.G24292T:p.V8098F,Ttn:NM_011652:exon221:c.G44035T:p.V14679F | . | 2qC3 | . | 0 | 1 |
| chr2 | 76798966 | A | C | 221 | Ttn | exonic | missense SNV | Ttn:NM_028004:exon74:c.T18046G:p.F6016V,Ttn:NM_011652:exon195:c.T37789G:p.F12597V | . | 2qC3 | . | 0 | 1 |
| chr2 | 76800540 | T | C | 221 | Ttn | exonic | synonymous SNV | Ttn:NM_028004:exon71:c.A17538G:p.Q5846Q,Ttn:NM_011652:exon192:c.A37281G:p.Q12427Q | . | 2qC3 | . | 0 | 1 |
| chr2 | 76890949 | C | G | 221 | Ttn | exonic | missense SNV | Ttn:NM_011652:exon70:c.G17611C:p.V5871L | . | 2qC3 | . | 0 | 1 |
| chr2 | 76942245 | C | A | 212 | Ttn | exonic | missense SNV | Ttn:NM_028004:exon31:c.G7405T:p.A2469S,Ttn:NM_011652:exon32:c.G7543T:p.A2515S | . | 2qC3 | . | 0 | 1 |
| chr2 | 76953137 | G | A | 221 | Ttn | exonic | missense SNV | Ttn:NM_028004:exon15:c.C2510T:p.P837L,Ttn:NM_011652:exon16:c.C2648T:p.P883L | . | 2qC3 | . | 0 | 1 |
| chr2 | 76953182 | C | A | 221 | Ttn | exonic | missense SNV | Ttn:NM_028004:exon15:c.G2465T:p.G822V,Ttn:NM_011652:exon16:c.G2603T:p.G868V | . | 2qC3 | . | 0 | 1 |
| chr2 | 77042613 | G | A | 221 | Ccdc141 | exonic | stopgain | Ccdc141:NM_001025576:exon15:c.C2224T:p.Q742X | . | 2qC3 | . | 0 | 1 |
| chr2 | 77192711 | A | G | 116 | Sestd1 | intronic | . | . | . | 2qC3 | . | 0 | 1 |
| chr2 | 77908210 | T | C | 222 | Cwc22 | intronic | . | . | . | 2qC3 | . | 0 | 1 |
| chr2 | 78513058 | G | T | 185 | . | intergenic | . | . | 528 | 2qC3 | . | 0 | 1 |
| chr2 | 78657728 | G | A | 22.983 | . | intergenic | . | . | . | 2qC3 | . | 0 | 1 |
| chr2 | 79303462 | A | C | 103 | Itga4 | intronic | . | . | . | 2qC3 | . | 0 | 1 |
| chr2 | 80345196 | G | T | 96 | Dnajc10 | intronic | . | . | . | 2qC3 | . | 0 | 1 |
| chr2 | 80540580 | T | G | 221 | Nckap1 | exonic | missense SNV | Nckap1:NM_016965:exon11:c.A1031C:p.K344T,Nckap1:NM_001290745:exon12:c.A1049C:p.K350T | . | 2qC3 | . | 0 | 1 |
| chr2 | 80644257 | C | T | 221 | Nup35 | exonic | missense SNV | Nup35:NM_001190179:exon3:c.C266T:p.T89M,Nup35:NM_027091:exon3:c.C317T:p.T106M | . | 2qC3 | . | 0 | 1 |
| chr2 | 82257756 | C | T | 212 | Zfp804a | exonic | missense SNV | Zfp804a:NM_175513:exon4:c.C1928T:p.A643V | . | 2qD | . | 0 | 1 |
| chr2 | 82978087 | G | T | 215 | . | intergenic | . | . | . | 2qD | . | 0 | 1 |
| chr2 | 82993470 | G | A | 222 | . | intergenic | . | . | . | 2qD | . | 0 | 1 |
| chr2 | 84621073 | A | G | 104 | Ctnnd1 | intronic | . | . | . | 2qD | . | 0 | 1 |
| chr2 | 84852567 | T | G | 20.1941 | Slc43a1 | intronic | . | . | . | 2qD | . | 0 | 1 |
| chr2 | 84860089 | G | A | 221 | Slc43a1 | splicing | . | . | . | 2qD | . | 0 | 1 |
| chr2 | 85438785 | C | A | 196 | Olfr995 | exonic | synonymous SNV | Olfr995:NM_146434:exon1:c.G372T:p.V124V | . | 2qD | . | 0 | 1 |
| chr2 | 85494249 | T | G | 188 | 4833423E24Rik | intronic | . | . | . | 2qD | . | 0 | 1 |
| chr2 | 85721786 | G | A | 221 | Olfr1009 | exonic | missense SNV | Olfr1009:NM_146572:exon1:c.G380A:p.C127Y | . | 2qD | . | 0 | 1 |
| chr2 | 85785931 | T | C | 222 | Olfr1015 | exonic | missense SNV | Olfr1015:NM_146571:exon1:c.T419C:p.L140P | . | 2qD | . | 0 | 1 |
| chr2 | 85886980 | A | G | 222 | Olfr1023 | exonic | missense SNV | Olfr1023:NM_146587:exon1:c.A179G:p.Y60C | . | 2qD | . | 0 | 1 |
| chr2 | 85931989 | T | C | 23.0134 | . | intergenic | . | . | . | 2qD | . | 0 | 1 |
| chr2 | 86146319 | T | A | 125 | Olfr1040 | exonic | missense SNV | Olfr1040:NM_207561:exon1:c.A414T:p.K138N | . | 2qD | . | 0 | 1 |
| chr2 | 86162158 | A | T | 212 | Olfr1043 | exonic | missense SNV | Olfr1043:NM_146577:exon1:c.T790A:p.S264T | . | 2qD | . | 0 | 1 |
| chr2 | 86171903 | C | T | 93 | . | upstream | . | . | . | 2qD | . | 0 | 1 |
| chr2 | 86228480 | A | T | 221 | Olfr1047 | exonic | missense SNV | Olfr1047:NM_147012:exon1:c.T490A:p.F164I | . | 2qD | . | 0 | 1 |
| chr2 | 86347480 | A | T | 222 | Olfr1055 | exonic | synonymous SNV | Olfr1055:NM_001310551:exon1:c.T285A:p.I95I,Olfr1055:NM_147021:exon1:c.T285A:p.I95I | . | 2qD | . | 0 | 1 |
| chr2 | 86786596 | T | G | 221 | Olfr1093 | exonic | missense SNV | Olfr1093:NM_146366:exon1:c.T865G:p.F289V | . | 2qD | . | 0 | 1 |
| chr2 | 86829098 | C | A | 222 | Olfr1094 | exonic | synonymous SNV | Olfr1094:NM_146365:exon1:c.C345A:p.L115L | . | 2qD | . | 0 | 1 |
| chr2 | 86890383 | G | A | 174 | Olfr1097 | exonic | missense SNV | Olfr1097:NM_146843:exon2:c.C791T:p.T264M | . | 2qD | . | 0 | 1 |
| chr2 | 86890667 | G | T | 221 | Olfr1097 | exonic | stopgain | Olfr1097:NM_146843:exon2:c.C507A:p.C169X | . | 2qD | . | 0 | 1 |
| chr2 | 86978860 | A | T | 53 | . | upstream | . | . | . | 2qD | . | 0 | 1 |
| chr2 | 87021522 | C | T | 127 | . | downstream | . | . | . | 2qD | . | 0 | 1 |
| chr2 | 87072087 | C | A | 221 | Olfr1107 | exonic | missense SNV | Olfr1107:NM_146844:exon1:c.G46T:p.G16C | . | 2qD | . | 0 | 1 |
| chr2 | 87308957 | A | C | 221 | Olfr1118 | exonic | missense SNV | Olfr1118:NM_207632:exon1:c.A167C:p.N56T | . | 2qD | . | 0 | 1 |
| chr2 | 87508909 | T | C | 163 | Prame16 | intronic | . | . | . | 2qD | . | 0 | 1 |
| chr2 | 87544998 | G | A | 209 | Olfr1128 | exonic | missense SNV | Olfr1128:NM_146349:exon1:c.C545T:p.P182L | . | 2qD | . | 0 | 1 |
| chr2 | 87645495 | C | G | 221 | Olfr1133 | exonic | synonymous SNV | Olfr1133:NM_146351:exon1:c.G627C:p.G209G | . | 2qD | . | 0 | 1 |
| chr2 | 87782742 | C | A | 222 | Olfr152 | exonic | missense SNV | Olfr152:NM_146646:exon1:c.C207A:p.N69K | . | 2qD | . | 0 | 1 |
| chr2 | 87896440 | G | T | 221 | Olfr1153 | exonic | missense SNV | Olfr1153:NM_146640:exon1:c.G264T:p.M88I | . | 2qD | . | 0 | 1 |
| chr2 | 87973784 | T | A | 222 | Olfr74 | exonic | stopgain | Olfr74:NM_054091:exon1:c.A880T:p.R294X | . | 2qD | . | 0 | 1 |
| chr2 | 88402240 | G | T | 221 | Olfr1179 | exonic | synonymous SNV | Olfr1179:NM_146917:exon1:c.C693A:p.R231R | . | 2qD | . | 0 | 1 |
| chr2 | 88487543 | T | C | 221 | Olfr1184 | exonic | synonymous SNV | Olfr1184:NM_146823:exon1:c.T810C:p.A270A | . | 2qD | rs254566253 | 0 | 1 |
| chr2 | 88795291 | A | C | 222 | Olfr1201 | exonic | missense SNV | Olfr1201:NM_146895:exon1:c.A908C:p.K303T | . | 2qE1 | . | 0 | 1 |
| chr2 | 88831888 | C | A | 221 | Olfr1205 | exonic | missense SNV | Olfr1205:NM_146896:exon2:c.C770A:p.A257E | . | 2qE1 | . | 0 | 1 |
| chr2 | 88973586 | C | T | 220 | Olfr1213 | exonic | missense SNV | Olfr1213:NM_146898:exon3:c.G304A:p.A102T | . | 2qE1 | . | 0 | 1 |
| chr2 | 89170858 | G | A | 221 | Olfr1225 | exonic | missense SNV | Olfr1225:NM_146891:exon1:c.C353T:p.A118V | . | 2qE1 | . | 0 | 1 |
| chr2 | 89527514 | G | T | 162 | Olfr1243 | exonic | missense SNV | Olfr1243:NM_146969:exon1:c.C895A:p.L299I | . | 2qE1 | . | 0 | 1 |
| chr2 | 89930073 | A | T | 221 | Olfr1258 | exonic | missense SNV | Olfr1258:NM_146978:exon1:c.A263T:p.K88M | . | 2qE1 | . | 0 | 1 |
| chr2 | 90149447 | A | G | 221 | Olfr1270 | exonic | synonymous SNV | Olfr1270:NM_146985:exon1:c.T558C:p.A186A | . | 2qE1 | . | 0 | 1 |
| chr2 | 90708807 | T | G | 221 | Nup160 | intronic | . | . | . | 2qE1 | . | 0 | 1 |
| chr2 | 90745538 | G | T | 111 | Fnbp4 | exonic | missense SNV | Fnbp4:NM_018828:exon1:c.G82T:p.G28W | 804 | 2qE1 | . | 0 | 1 |
| chr2 | 90775906 | T | C | 102 | Fnbp4 | exonic | synonymous SNV | Fnbp4:NM_018828:exon13:c.T2169C:p.G723G | . | 2qE1 | . | 0 | 1 |
| chr2 | 90804654 | C | T | 194 | Agbl2 | intronic | . | . | . | 2qE1 | . | 0 | 1 |
| chr2 | 91043128 | T | A | 179 | Rapsn | intronic | . | . | . | 2qE1 | . | 0 | 1 |
| chr2 | 91114438 | A | G | 200 | Spi1 | intronic | . | . | . | 2qE1 | . | 0 | 1 |
| chr2 | 91134664 | G | A | 177 | Mybpc3 | intronic | . | . | . | 2qE1 | . | 0 | 1 |
| chr2 | 91158375 | C | T | 204 | Madd | intronic | . | . | . | 2qE1 | . | 0 | 1 |
| chr2 | 91260258 | G | T | 162 | Pacsin3 | exonic | missense SNV | Pacsin3:NM_001289677:exon2:c.G51T:p.W17C,Pacsin3:NM_001289678:exon3:c.G51T:p.W17C,Pacsin3:NM | . | 2qE1 | . | 0 | 1 |
| chr2 | 91632926 | C | G | 217 | F2 | intronic | . | . | . | 2qE1 | . | 0 | 1 |
| chr2 | 91689582 | T | A | 110 | Atg13 | intronic | . | . | . | 2qE1 | . | 0 | 1 |
| chr2 | 91938427 | C | T | 221 | Dgkz | intronic | . | . | . | 2qE1 | . | 0 | 1 |
| chr2 | 91943889 | T | C | 168 | Dgkz | intronic | . | . | . | 2qE1 | . | 0 | 1 |
| chr2 | 91946759 | C | T | 221 | Dgkz | intronic | . | . | . | 2qE1 | . | 0 | 1 |
| chr2 | 92023914 | G | C | 207 | Creb3l1 | intronic | . | . | . | 2qE1 | . | 0 | 1 |
| chr2 | 92348824 | G | C | 184 | Phf21a | intronic | . | . | . | 2qE1 | . | 0 | 1 |
| chr2 | 92383358 | T | G | 222 | 1700029I15Rik | intronic | . | . | . | 2qE1 | . | 0 | 1 |
| chr2 | 93198421 | C | A | 221 | Trp53i11 | intronic | . | . | . | 2qE1 | . | 0 | 1 |
| chr2 | 93222876 | A | G | 191 | Tspan18 | intronic | . | . | . | 2qE1 | . | 0 | 1 |
| chr2 | 93430205 | C | A | 222 | Cd82 | exonic | missense SNV | Cd82:NM_001271430:exon4:c.G187T:p.G63C,Cd82:NM_001271431:exon4:c.G187T:p.G63C,Cd82:NM_001136( | . | 2qE1 | . | 0 | 1 |
| chr2 | 93704483 | C | A | 169 | Ext2 | exonic | missense SNV | Ext2:NM_010163:exon12:c.G1935T:p.K645N | . | 2qE1 | . | 0 | 1 |
| chr2 | 93704680 | C | T | 222 | Ext2 | intronic | . | . | . | 2qE1 | . | 0 | 1 |
| chr2 | 94371851 | G | A | 183 | Ttc17 | exonic | missense SNV | Ttc17:NM_183106:exon9:c.C1105T:p.H369Y | . | 2qE1 | . | 0 | 1 |
| chr2 | 94374647 | T | A | 222 | Ttc17 | exonic | missense SNV | Ttc17:NM_183106:exon8:c.A929T:p.E310V | . | 2qE1 | . | 0 | 1 |
| chr2 | 97630154 | G | A | 222 | Lrrc4c | exonic | missense SNV | Lrrc4c:NM_001289744:exon2:c.G1124A:p.C375Y,Lrrc4c:NM_178725:exon3:c.G1124A:p.C375Y,Lrrc4c:NM | . | 2qE1 | . | 0 | 1 |
| chr2 | 98291580 | A | C | 38.7365 | . | intergenic | . | . | . | 2qE1 | . | 0 | 1 |
| chr2 | 101630020 | C | A | 221 | Rag2 | exonic | missense SNV | Rag2:NM_009020:exon3:c.C674A:p.A225D | . | 2qE2 | . | 0 | 1 |
| chr2 | 102550088 | G | C | 220 | Pamr1 | UTR5 | . | . | . | 2qE2 | . | 0 | 1 |
| chr2 | 104154819 | C | G | 133 | D430041D05Rik | intronic | . | . | . | 2qE2 | . | 0 | 1 |
| chr2 | 105388965 | C | A | 93 | Rcn1 | intronic | . | . | . | 2qE3 | . | 0 | 1 |
| chr2 | 105554148 | A | G | 228 | Pax6os1 | ncRNA_intronic | . | . | . | 2qE3 | rs29521762 | 1 | 0 |
| chr2 | 106377892 | A | G | 222 | . | intergenic | . | . | . | 2qE3 | . | 0 | 1 |
| chr2 | 106383992 | C | A | 124 | . | intergenic | . | . | . | 2qE3 | . | 0 | 1 |
| chr2 | 106965668 | T | A | 221 | Arl14ep | intronic | . | . | . | 2qE3 | . | 0 | 1 |
| chr2 | 107011076 | C | T | 76 | . | intergenic | . | . | . | 2qE3 | . | 0 | 1 |
| chr2 | 110681730 | T | C | 153 | Ano3 | intronic | . | . | . | 2qE3 | . | 0 | 1 |
| chr2 | 110884810 | T | C | 118 | Ano3 | intronic | . | . | . | 2qE3 | rs259596235 | 0 | 1 |
| chr2 | 111306496 | G | A | 222 | Olfr1279 | exonic | missense SNV | Olfr1279:NM_146393:exon1:c.G296A:p.C99Y | . | 2qE3 | . | 0 | 1 |
| chr2 | 111479278 | A | T | 222 | Olfr1288 | exonic | missense SNV | Olfr1288:NM_146400:exon1:c.A493T:p.N165Y | . | 2qE3 | . | 0 | 1 |
| chr2 | 111912471 | A | C | 222 | Olfr1306 | exonic | missense SNV | Olfr1306:NM_001011803:exon1:c.T458G:p.L153R | . | 2qE3 | . | 0 | 1 |
| chr2 | 112251769 | C | G | 222 | Nutm1 | exonic | missense SNV | Nutm1:NM_172521:exon4:c.G961C:p.G321R | . | 2qE3 | . | 0 | 1 |
| chr2 | 112775683 | A | C | 222 | Ryr3 | exonic | missense SNV | Ryr3:NM_177652:exon47:c.T7231G:p.L2411V | . | 2qE3 | . | 0 | 1 |
| chr2 | 112833641 | T | C | 222 | Ryr3 | exonic | synonymous SNV | Ryr3:NM_177652:exon35:c.A5364G:p.K1788K | . | 2qE3 | . | 0 | 1 |
| chr2 | 113291110 | A | C | 89 | Mir1954,Tmco5b | ncRNA_intronic | . | . | . | 2qE4 | . | 0 | 1 |
| chr2 | 113363872 | C | G | 150 | Mir1954,Fmn1 | ncRNA_intronic | . | . | . | 2qE4 | . | 0 | 1 |
| chr2 | 115867216 | T | A | 156 | Meis2 | intronic | . | . | . | 2qE4 | . | 0 | 1 |
| chr2 | 118600012 | G | T | 111 | Bub1b | intronic | . | . | . | 2qE5 | . | 0 | 1 |
| chr2 | 119026937 | A | C | 81 | Ccdc32 | intronic | . | . | . | 2qE5 | rs46130980 | 1 | 0 |
| chr2 | 119069722 | C | T | 221 | Casc5 | exonic | synonymous SNV | Casc5:NM_029617:exon8:c.C1903T:p.L635L | . | 2qE5 | . | 0 | 1 |
| chr2 | 119127222 | A | G | 150 | Rad51 | intronic | . | . | . | 2qE5 | . | 0 | 1 |
| chr2 | 119172005 | C | T | 221 | Gchfr | exonic | synonymous SNV | Gchfr:NM_177157:exon3:c.C195T:p.F65F | . | 2qE5 | . | 0 | 1 |
| chr2 | 119921259 | T | A | 108 | Mga | intronic | . | . | . | 2qE5 | . | 0 | 1 |
| chr2 | 120012703 | G | C | 222 | Mapkbp1 | exonic | missense SNV | Mapkbp1:NM_011941:exon6:c.G612C:p.W204C | . | 2qE5 | . | 0 | 1 |
| chr2 | 120170237 | T | C | 222 | Pla2g4e | intronic | . | . | . | 2qE5 | . | 0 | 1 |
| chr2 | 120171468 | G | C | 181 | Pla2g4e | intronic | . | . | . | 2qE5 | . | 0 | 1 |
| chr2 | 120268904 | G | C | 194 | Pla2g4d | exonic | missense SNV | Pla2g4d:NM_001024137:exon19:c.C2116G:p.R706G | . | 2qE5 | . | 0 | 1 |
| chr2 | 120311224 | G | T | 71 | Pla2g4f | intronic | . | . | . | 2qE5 | . | 0 | 1 |
| chr2 | 120325621 | C | A | 100 | Vps39 | intronic | . | . | . | 2qE5 | . | 0 | 1 |
| chr2 | 120374078 | C | G | 56 | Tmem87a | intronic | . | . | . | 2qE5 | . | 0 | 1 |
| chr2 | 120435442 | G | T | 221 | Ganc | intronic | . | . | . | 2qE5 | . | 0 | 1 |
| chr2 | 120444364 | C | T | 35.0822 | Ganc | intronic | . | . | . | 2qE5 | . | 0 | 1 |
| chr2 | 120494886 | G | A | 190 | Capn3 | exonic | missense SNV | Capn3:NM_001109761:exon10:c.G1265A:p.R422H,Capn3:NM_001177799:exon10:c.G1325A:p.R442H,Capn3:N | . | 2qE5 | . | 0 | 1 |
| chr2 | 120666683 | A | T | 58 | . | intergenic | . | . | . | 2qE5 | . | 0 | 1 |
| chr2 | 120699574 | G | T | 221 | . | intergenic | . | . | . | 2qE5 | . | 0 | 1 |
| chr2 | 120705960 | G | T | 132 | . | intergenic | . | . | . | 2qE5 | . | 0 | 1 |
| chr2 | 120720842 | G | A | 221 | Cdan1 | intronic | . | . | . | 2qE5 | . | 0 | 1 |
| chr2 | 120979192 | G | T | 176 | Tmem62 | exonic | missense SNV | Tmem62:NM_175285:exon3:c.G354T:p.W118C | . | 2qE5 | . | 0 | 1 |
| chr2 | 121576349 | C | T | 222 | Frmd5 | exonic | missense SNV | Frmd5:NM_172673:exon5:c.G400A:p.A134T | . | 2qE5 | . | 0 | 1 |
| chr2 | 122248944 | G | T | 219 | Sord | exonic | missense SNV | Sord:NM_146126:exon3:c.G246T:p.L82F | . | 2qE5 | . | 0 | 1 |
| chr2 | 122291983 | C | T | 204 | Duox2 | intronic | . | . | . | 2qE5 | . | 0 | 1 |
| chr2 | 124664635 | G | T | 216 | Sema6d | exonic | stopgain | Sema6d:NM_172537:exon17:c.G2362T:p.E788X,Sema6d:NM_199238:exon17:c.G2323T:p.E775X,Sema6d:NM_1 | . | 2qF1 | . | 0 | 1 |
| chr2 | 125186789 | C | A | 221 | Slc12a1 | intronic | . | . | . | 2qF1 | . | 0 | 1 |
| chr2 | 125397942 | C | G | 221 | Fbn1 | intronic | . | . | . | 2qF1 | . | 0 | 1 |
| chr2 | 125587736 | C | A | 71 | Cep152 | intronic | . | . | . | 2qF1 | . | 0 | 1 |
| chr2 | 126424373 | G | A | 61 | Atp8b4 | intronic | . | . | . | 2qF1 | . | 0 | 1 |
| chr2 | 126840591 | G | A | 108 | Trpm7 | intronic | . | . | . | 2qF1 | . | 0 | 1 |
| chr2 | 127140482 | C | G | 203 | Itpripl1 | UTR3 | . | . | . | 2qF1 | . | 0 | 1 |
| chr2 | 127226622 | G | T | 25.7522 | Snrnp200 | intronic | . | . | . | 2qF1 | . | 0 | 1 |
| chr2 | 127244747 | T | G | 80 | Ciao1 | intronic | . | . | . | 2qF1 | . | 0 | 1 |
| chr2 | 127510759 | T | C | 67 | Kcnip3 | intronic | . | . | . | 2qF1 | rs27434814 | 0 | 1 |
| chr2 | 127510791 | C | T | 135 | Kcnip3 | intronic | . | . | . | 2qF1 | rs47217030 | 0 | 1 |
| chr2 | 127510969 | T | C | 222 | Kcnip3 | exonic | missense SNV | Kcnip3:NM_019789:exon2:c.A41G:p.N14S | . | 2qF1 | rs51749766 | 0 | 1 |
| chr2 | 127510977 | T | C | 222 | Kcnip3 | exonic | missense SNV | Kcnip3:NM_001291005:exon2:c.A121G:p.R41G | . | 2qF1 | rs27434812 | 0 | 1 |
| chr2 | 127511101 | C | A | 125 | Kcnip3 | intronic | . | . | . | 2qF1 | rs51095997 | 0 | 1 |
| chr2 | 127512212 | T | C | 32.1299 | Kcnip3 | intronic | . | . | . | 2qF1 | rs45792786 | 0 | 1 |
| chr2 | 127512226 | A | G | 23.7881 | Kcnip3 | intronic | . | . | . | 2qF1 | rs50012419 | 0 | 1 |
| chr2 | 127512317 | T | C | 221 | Kcnip3 | intronic | . | . | . | 2qF1 | rs46495933 | 0 | 1 |
| chr2 | 127521086 | C | T | 130 | Kcnip3 | intronic | . | . | . | 2qF1 | rs32840599 | 0 | 1 |
| chr2 | 127528504 | C | A | 58 | Prom2 | UTR3 | . | . | . | 2qF1 | rs50625253 | 0 | 1 |
| chr2 | 127528725 | T | C | 107 | Prom2 | intronic | . | . | . | 2qF1 | rs27434722 | 0 | 1 |
| chr2 | 127528727 | A | G | 103 | Prom2 | intronic | . | . | . | 2qF1 | rs47286647 | 0 | 1 |
| chr2 | 127528933 | T | C | 176 | Prom2 | intronic | . | . | . | 2qF1 | rs27434720 | 0 | 1 |
| chr2 | 127528981 | C | T | 84 | Prom2 | intronic | . | . | . | 2qF1 | rs27434719 | 0 | 1 |
| chr2 | 127529049 | A | T | 33.9379 | Prom2 | intronic | . | . | . | 2qF1 | rs51275675 | 0 | 1 |
| chr2 | 127529063 | G | A | 43.6958 | Prom2 | intronic | . | . | . | 2qF1 | rs49923142 | 0 | 1 |
| chr2 | 127529161 | C | T | 221 | Prom2 | exonic | synonymous SNV | Prom2:NM_138750:exon22:c.G2415A:p.K805K,Prom2:NM_178047:exon22:c.G2415A:p.K805K | . | 2qF1 | rs27434718 | 0 | 1 |
| chr2 | 127529313 | T | C | 198 | Prom2 | intronic | . | . | . | 2qF1 | rs47411911 | 0 | 1 |
| chr2 | 127529314 | A | G | 196 | Prom2 | intronic | . | . | . | 2qF1 | rs47998663 | 0 | 1 |
| chr2 | 127529386 | T | C | 222 | Prom2 | intronic | . | . | . | 2qF1 | rs48022029 | 0 | 1 |
| chr2 | 127529898 | A | C | 46.8246 | Prom2 | intronic | . | . | . | 2qF1 | rs27434714 | 0 | 1 |
| chr2 | 127529931 | G | C | 55 | Prom2 | intronic | . | . | . | 2qF1 | rs27434713 | 0 | 1 |
| chr2 | 127529994 | A | G | 108 | Prom2 | intronic | . | . | . | 2qF1 | rs50805654 | 0 | 1 |
| chr2 | 127530056 | T | C | 201 | Prom2 | intronic | . | . | . | 2qF1 | rs27434712 | 0 | 1 |
| chr2 | 127530279 | G | A | 222 | Prom2 | intronic | . | . | . | 2qF1 | rs45722446 | 0 | 1 |
| chr2 | 127530334 | T | C | 221 | Prom2 | intronic | . | . | . | 2qF1 | rs47167110 | 0 | 1 |
| chr2 | 127530412 | A | G | 222 | Prom2 | exonic | synonymous SNV | Prom2:NM_138750:exon19:c.T2130C:p.T710T,Prom2:NM_178047:exon19:c.T2130C:p.T710T | . | 2qF1 | rs27434711 | 0 | 1 |
| chr2 | 127530550 | A | C | 203 | Prom2 | intronic | . | . | . | 2qF1 | rs27434710 | 0 | 1 |
| chr2 | 127531169 | C | T | 67 | Prom2 | exonic | missense SNV | Prom2:NM_138750:exon17:c.G1979A:p.R660Q,Prom2:NM_178047:exon17:c.G1979A:p.R660Q | . | 2qF1 | rs33360083 | 0 | 1 |
| chr2 | 127532979 | T | C | 222 | Prom2 | intronic | . | . | . | 2qF1 | rs3685281 | 0 | 1 |
| chr2 | 127534939 | T | A | 222 | Prom2 | intronic | . | . | . | 2qF1 | rs27434693 | 0 | 1 |
| chr2 | 127535064 | G | A | 222 | Prom2 | exonic | synonymous SNV | Prom2:NM_138750:exon13:c.C1629T:p.S543S,Prom2:NM_178047:exon13:c.C1629T:p.S543S | . | 2qF1 | rs27434692 | 0 | 1 |
| chr2 | 127590747 | T | C | 222 | Mrps5 | exonic | synonymous SNV | Mrps5:NM_029963:exon2:c.T111C:p.T37T | . | 2qF1 | rs13471332 | 0 | 1 |
| chr2 | 127592113 | T | C | 70 | Mrps5 | intronic | . | . | . | 2qF1 | rs29499550 | 0 | 1 |
| chr2 | 127690066 | C | T | 222 | . | intergenic | . | . | . | 2qF1 | rs28284030 | 0 | 1 |
| chr2 | 127690198 | A | G | 128 | . | intergenic | . | . | . | 2qF1 | rs28284028 | 0 | 1 |
| chr2 | 127690240 | A | T | 86 | . | intergenic | . | . | . | 2qF1 | rs28284026 | 0 | 1 |

| | | | | | | | | | | | | | |
|---|---|---|---|---|---|---|---|---|---|---|---|---|---|
| chr2 | 127705592 | T | C | 42.2333 | Mal1 | UTR3 | . | . | . | 2qF1 | rs51111466 | 0 | 1 |
| chr2 | 127705837 | A | G | 222 | Mal1 | UTR3 | . | . | . | 2qF1 | rs28283985 | 0 | 1 |
| chr2 | 127706073 | C | T | 118 | Mal1 | intronic | . | . | . | 2qF1 | rs49339697 | 0 | 1 |
| chr2 | 127708808 | C | G | 222 | Mal1 | intronic | . | . | . | 2qF1 | rs28283972 | 0 | 1 |
| chr2 | 127708929 | G | A | 222 | Mal1 | exonic | synonymous SNV | Mal1:NM_145532:exon3:c.C310T:p.L104L | . | 2qF1 | rs47523056 | 0 | 1 |
| chr2 | 127712395 | G | T | 221 | Mal1 | exonic | missense SNV | Mal1:NM_145532:exon2:c.C153A:p.H51Q | . | 2qF1 | . | 0 | 1 |
| chr2 | 127740757 | G | T | 66 | Nphp1 | UTR3 | . | . | . | 2qF1 | rs33279962 | 0 | 1 |
| chr2 | 127743175 | T | A | 221 | Nphp1 | intronic | . | . | . | 2qF1 | rs28269162 | 0 | 1 |
| chr2 | 127747821 | C | T | 98 | Nphp1 | intronic | . | . | . | 2qF1 | rs29517953 | 0 | 1 |
| chr2 | 127754041 | A | G | 185 | Nphp1 | exonic | synonymous SNV | Nphp1:NM_001291012:exon16:c.T1500C:p.S500S,Nphp1:NM_001291013:exon16:c.T1497C:p.S499S,Nphp1:N | . | 2qF1 | rs28269120 | 0 | 1 |
| chr2 | 127754122 | C | T | 68 | Nphp1 | intronic | . | . | . | 2qF1 | rs28269118 | 0 | 1 |
| chr2 | 127754145 | T | C | 69 | Nphp1 | intronic | . | . | . | 2qF1 | rs28269117 | 0 | 1 |
| chr2 | 127756271 | A | T | 172 | Nphp1 | intronic | . | . | . | 2qF1 | rs28269103 | 0 | 1 |
| chr2 | 127768345 | C | A | 157 | Nphp1 | intronic | . | . | . | 2qF1 | rs33425104 | 0 | 1 |
| chr2 | 127769437 | G | A | 66 | Nphp1 | intronic | . | . | . | 2qF1 | rs29770020 | 0 | 1 |
| chr2 | 127769468 | T | C | 73 | Nphp1 | intronic | . | . | . | 2qF1 | rs29674668 | 0 | 1 |
| chr2 | 127769497 | A | G | 82 | Nphp1 | intronic | . | . | . | 2qF1 | rs33304283 | 0 | 1 |
| chr2 | 127772245 | C | T | 134 | Nphp1 | intronic | . | . | . | 2qF1 | rs49111860 | 0 | 1 |
| chr2 | 127777252 | A | G | 222 | Nphp1 | intronic | . | . | . | 2qF1 | rs51475102 | 0 | 1 |
| chr2 | 127777295 | C | T | 221 | Nphp1 | exonic | synonymous SNV | Nphp1:NM_001291012:exon4:c.G303A:p.V101V,Nphp1:NM_001291013:exon4:c.G303A:p.V101V,Nphp1:NM_01 | . | 2qF1 | rs33243757 | 0 | 1 |
| chr2 | 127779038 | G | A | 107 | Nphp1 | intronic | . | . | . | 2qF1 | rs46723302 | 0 | 1 |
| chr2 | 127779892 | C | T | 66 | Nphp1 | intronic | . | . | . | 2qF1 | rs52714014 | 0 | 1 |
| chr2 | 127780165 | C | T | 222 | Nphp1 | intronic | . | . | . | 2qF1 | rs50115066 | 0 | 1 |
| chr2 | 127780196 | T | C | 222 | Nphp1 | intronic | . | . | . | 2qF1 | rs47816622 | 0 | 1 |
| chr2 | 127802800 | T | C | 46.7885 | Bub1 | intronic | . | . | . | 2qF1 | rs6360100 | 0 | 1 |
| chr2 | 127803270 | G | T | 106 | Bub1 | intronic | . | . | . | 2qF1 | rs28269085 | 0 | 1 |
| chr2 | 127804771 | T | A | 222 | Bub1 | intronic | . | . | . | 2qF1 | rs33404050 | 0 | 1 |
| chr2 | 127804854 | A | G | 222 | Bub1 | exonic | synonymous SNV | Bub1:NM_001113179:exon21:c.T2505C:p.N835N,Bub1:NM_009772:exon21:c.T2502C:p.N834N | . | 2qF1 | rs29781674 | 0 | 1 |
| chr2 | 127806197 | C | T | 170 | Bub1 | intronic | . | . | . | 2qF1 | rs33455445 | 0 | 1 |
| chr2 | 127806341 | G | A | 222 | Bub1 | exonic | synonymous SNV | Bub1:NM_001113179:exon19:c.C2157T:p.D719D,Bub1:NM_009772:exon19:c.C2154T:p.D718D | . | 2qF1 | rs33732346 | 0 | 1 |
| chr2 | 127809071 | T | A | 222 | Bub1 | intronic | . | . | . | 2qF1 | rs33188156 | 0 | 1 |
| chr2 | 127810792 | A | G | 221 | Bub1 | intronic | . | . | . | 2qF1 | rs49090744 | 0 | 1 |
| chr2 | 127810809 | G | A | 221 | Bub1 | intronic | . | . | . | 2qF1 | rs46114461 | 0 | 1 |
| chr2 | 127814031 | C | A | 147 | Bub1 | intronic | . | . | . | 2qF1 | rs29620690 | 0 | 1 |
| chr2 | 127817159 | T | C | 81 | Bub1 | intronic | . | . | . | 2qF1 | rs29969266 | 0 | 1 |
| chr2 | 127819169 | A | G | 221 | Bub1 | intronic | . | . | . | 2qF1 | . | 0 | 1 |
| chr2 | 127823891 | G | A | 128 | Bub1 | intronic | . | . | . | 2qF1 | rs28269041 | 0 | 1 |
| chr2 | 127823966 | C | T | 222 | Bub1 | exonic | missense SNV | Bub1:NM_001113179:exon6:c.G560A:p.R187K,Bub1:NM_009772:exon6:c.G560A:p.R187K | . | 2qF1 | rs28269040 | 0 | 1 |
| chr2 | 127825010 | A | T | 116 | Bub1 | intronic | . | . | . | 2qF1 | rs51142374 | 0 | 1 |
| chr2 | 127825384 | C | T | 74 | Bub1 | intronic | . | . | . | 2qF1 | rs28269038 | 0 | 1 |
| chr2 | 127827483 | A | G | 221 | Bub1 | exonic | synonymous SNV | Bub1:NM_001113179:exon4:c.T240C:p.S80S,Bub1:NM_009772:exon4:c.T240C:p.S80S | . | 2qF1 | rs13471505 | 0 | 1 |
| chr2 | 127829886 | A | G | 92 | Bub1 | intronic | . | . | . | 2qF1 | rs46756201 | 0 | 1 |
| chr2 | 128423611 | T | C | 120 | Gm14005 | ncRNA_intronic | . | . | . | 2qF1 | rs27411108 | 0 | 1 |
| chr2 | 128424219 | C | T | 140 | Gm14005 | ncRNA_intronic | . | . | . | 2qF1 | rs27411105 | 0 | 1 |
| chr2 | 128424457 | A | G | 222 | Gm14005 | ncRNA_intronic | . | . | . | 2qF1 | rs33330427 | 0 | 1 |
| chr2 | 128558002 | T | C | 222 | . | intergenic | . | . | . | 2qF1 | rs6325084 | 0 | 1 |
| chr2 | 128592804 | C | T | 222 | Spdye4c | intronic | . | . | . | 2qF1 | . | 0 | 1 |
| chr2 | 128593732 | A | C | 131 | Spdye4c | intronic | . | . | . | 2qF1 | rs27432704 | 0 | 1 |
| chr2 | 128749955 | A | G | 158 | Mertk | intronic | . | . | . | 2qF1 | rs27446492 | 0 | 1 |
| chr2 | 128750035 | A | G | 222 | Mertk | intronic | . | . | . | 2qF1 | rs27446491 | 0 | 1 |
| chr2 | 129038129 | T | C | 184 | . | intergenic | . | . | . | 2qF1 | rs27448965 | 0 | 1 |
| chr2 | 129038284 | C | A | 221 | . | intergenic | . | . | . | 2qF1 | rs27448964 | 0 | 1 |
| chr2 | 129038340 | G | A | 221 | . | intergenic | . | . | . | 2qF1 | rs27448963 | 0 | 1 |
| chr2 | 129038628 | A | G | 222 | . | intergenic | . | . | . | 2qF1 | rs27448962 | 0 | 1 |
| chr2 | 129038757 | A | G | 222 | . | intergenic | . | . | . | 2qF1 | rs27448961 | 0 | 1 |
| chr2 | 129039006 | A | G | 222 | . | intergenic | . | . | 707 | 2qF1 | rs32979474 | 0 | 1 |
| chr2 | 129039487 | T | G | 208 | . | intergenic | . | . | 707 | 2qF1 | rs27448960 | 0 | 1 |
| chr2 | 129039581 | C | T | 202 | . | intergenic | . | . | . | 2qF1 | rs27448959 | 0 | 1 |
| chr2 | 129040897 | T | C | 197 | . | intergenic | . | . | . | 2qF1 | rs27448957 | 0 | 1 |
| chr2 | 129040968 | A | G | 104 | . | intergenic | . | . | . | 2qF1 | rs27448956 | 0 | 1 |
| chr2 | 129102947 | G | A | 222 | Polr1b | intronic | . | . | . | 2qF1 | rs47662182 | 0 | 1 |
| chr2 | 129103094 | T | C | 222 | Polr1b | exonic | synonymous SNV | Polr1b:NM_009086:exon2:c.T309C:p.C103C | . | 2qF1 | rs45674576 | 0 | 1 |
| chr2 | 129103776 | A | G | 222 | Polr1b | intronic | . | . | . | 2qF1 | rs51963275 | 0 | 1 |
| chr2 | 129103798 | A | G | 222 | Polr1b | intronic | . | . | . | 2qF1 | rs27448809 | 0 | 1 |
| chr2 | 129103841 | G | A | 133 | Polr1b | intronic | . | . | . | 2qF1 | rs27448808 | 0 | 1 |
| chr2 | 129105064 | C | T | 44.9713 | Polr1b | intronic | . | . | . | 2qF1 | rs47792184 | 0 | 1 |
| chr2 | 129105163 | G | T | 144 | Polr1b | intronic | . | . | . | 2qF1 | rs48079245 | 0 | 1 |
| chr2 | 129105191 | T | C | 222 | Polr1b | intronic | . | . | . | 2qF1 | rs46499472 | 0 | 1 |
| chr2 | 129105500 | T | G | 50 | Polr1b | intronic | . | . | . | 2qF1 | rs46574518 | 0 | 1 |
| chr2 | 129108165 | C | T | 222 | Polr1b | intronic | . | . | . | 2qF1 | rs27448795 | 0 | 1 |
| chr2 | 129108325 | T | C | 35.4733 | Polr1b | intronic | . | . | . | 2qF1 | rs46267817 | 0 | 1 |
| chr2 | 129109078 | A | G | 36.5376 | Polr1b | intronic | . | . | . | 2qF1 | rs49591525 | 0 | 1 |
| chr2 | 129109150 | T | C | 146 | Polr1b | intronic | . | . | . | 2qF1 | rs27448793 | 0 | 1 |
| chr2 | 129109176 | G | A | 175 | Polr1b | intronic | . | . | . | 2qF1 | rs251146289 | 0 | 1 |
| chr2 | 129110237 | T | C | 30.7784 | Polr1b | intronic | . | . | . | 2qF1 | rs33760585 | 0 | 1 |
| chr2 | 129110239 | A | G | 30.8036 | Polr1b | intronic | . | . | . | 2qF1 | rs33059706 | 0 | 1 |
| chr2 | 129113691 | T | G | 222 | Polr1b | exonic | synonymous SNV | Polr1b:NM_009086:exon9:c.T1521G:p.P507P | . | 2qF1 | rs248669083 | 0 | 1 |
| chr2 | 129115882 | G | T | 171 | Polr1b | intronic | . | . | . | 2qF1 | rs27448762 | 0 | 1 |
| chr2 | 129117737 | G | T | 43.9904 | Polr1b | intronic | . | . | . | 2qF1 | rs27448753 | 0 | 1 |
| chr2 | 129118269 | C | T | 25.6681 | Polr1b | intronic | . | . | . | 2qF1 | rs27448749 | 0 | 1 |
| chr2 | 129119162 | C | T | 221 | Polr1b | exonic | synonymous SNV | Polr1b:NM_009086:exon12:c.C2040T:p.N680N | . | 2qF1 | rs27448743 | 0 | 1 |
| chr2 | 129125893 | A | G | 222 | Polr1b | exonic | missense SNV | Polr1b:NM_009086:exon15:c.A3205G:p.M1069V | 273 | 2qF1 | rs27448705 | 0 | 1 |
| chr2 | 129126197 | C | T | 138 | Polr1b | UTR3 | . | . | . | 2qF1 | rs27448701 | 0 | 1 |
| chr2 | 129225525 | A | G | 221 | A730036I17Rik | ncRNA_exonic | . | . | . | 2qF1 | rs27415056 | 0 | 1 |
| chr2 | 129268972 | G | C | 63 | Ckap2l | UTR3 | . | . | . | 2qF1 | rs27414806 | 0 | 1 |
| chr2 | 129269065 | T | C | 185 | Ckap2l | exonic | synonymous SNV | Ckap2l:NM_181589:exon9:c.A2211G:p.T737T | . | 2qF1 | rs27414805 | 0 | 1 |
| chr2 | 129269206 | T | A | 222 | Ckap2l | exonic | synonymous SNV | Ckap2l:NM_181589:exon9:c.A2070T:p.S690S | . | 2qF1 | rs27414804 | 0 | 1 |
| chr2 | 129270544 | A | T | 175 | Ckap2l | intronic | . | . | . | 2qF1 | rs32830488 | 0 | 1 |
| chr2 | 129270608 | T | G | 222 | Ckap2l | exonic | missense SNV | Ckap2l:NM_181589:exon8:c.A1979C:p.H660P | . | 2qF1 | rs27414792 | 0 | 1 |
| chr2 | 129270635 | C | T | 222 | Ckap2l | exonic | missense SNV | Ckap2l:NM_181589:exon8:c.G1952A:p.R651Q | . | 2qF1 | rs27414791 | 0 | 1 |
| chr2 | 129270820 | A | C | 222 | Ckap2l | intronic | . | . | . | 2qF1 | rs27414790 | 0 | 1 |
| chr2 | 129270874 | A | G | 183 | Ckap2l | intronic | . | . | . | 2qF1 | rs27414789 | 0 | 1 |
| chr2 | 129272585 | A | C | 221 | Ckap2l | intronic | . | . | . | 2qF1 | rs27414778 | 0 | 1 |
| chr2 | 129275233 | C | T | 94 | Ckap2l | intronic | . | . | . | 2qF1 | rs3719265 | 0 | 1 |
| chr2 | 129275389 | T | A | 222 | Ckap2l | intronic | . | . | . | 2qF1 | rs3720402 | 0 | 1 |
| chr2 | 129275681 | G | A | 222 | Ckap2l | intronic | . | . | . | 2qF1 | rs27462848 | 0 | 1 |
| chr2 | 129282051 | T | C | 149 | Ckap2l | exonic | synonymous SNV | Ckap2l:NM_181589:exon5:c.A1449G:p.R483R | . | 2qF1 | rs29574166 | 0 | 1 |
| chr2 | 129284684 | C | A | 48.7322 | Ckap2l | intronic | . | . | . | 2qF1 | rs27462803 | 0 | 1 |
| chr2 | 129285045 | G | A | 221 | Ckap2l | exonic | synonymous SNV | Ckap2l:NM_181589:exon4:c.C1212T:p.Y404Y | . | 2qF1 | rs33106605 | 0 | 1 |
| chr2 | 129285258 | T | C | 221 | Ckap2l | exonic | synonymous SNV | Ckap2l:NM_181589:exon4:c.A999G:p.Q333Q | . | 2qF1 | rs27462802 | 0 | 1 |
| chr2 | 129285380 | T | C | 221 | Ckap2l | exonic | missense SNV | Ckap2l:NM_181589:exon4:c.A877G:p.M293V | . | 2qF1 | rs27462801 | 0 | 1 |
| chr2 | 129285509 | T | C | 221 | Ckap2l | exonic | missense SNV | Ckap2l:NM_181589:exon4:c.A748G:p.T250A | . | 2qF1 | rs27462800 | 0 | 1 |
| chr2 | 129285530 | T | C | 221 | Ckap2l | exonic | missense SNV | Ckap2l:NM_181589:exon4:c.A727G:p.I243V | . | 2qF1 | rs13471578 | 0 | 1 |
| chr2 | 129285755 | G | A | 221 | Ckap2l | exonic | missense SNV | Ckap2l:NM_181589:exon4:c.C502T:p.H168Y | . | 2qF1 | rs13471576 | 0 | 1 |
| chr2 | 129285874 | A | G | 222 | Ckap2l | exonic | missense SNV | Ckap2l:NM_181589:exon4:c.T383C:p.F128S | . | 2qF1 | rs13471577 | 0 | 1 |
| chr2 | 129296106 | T | C | 24.0799 | Ckap2l | intronic | . | . | . | 2qF1 | rs27462775 | 0 | 1 |
| chr2 | 129296272 | C | A | 204 | Ckap2l | intronic | . | . | . | 2qF1 | rs247902322 | 0 | 1 |
| chr2 | 129296473 | T | C | 152 | Ckap2l | intronic | . | . | . | 2qF1 | rs3673784 | 0 | 1 |
| chr2 | 129296548 | C | G | 72 | Ckap2l | intronic | . | . | . | 2qF1 | rs27462771 | 0 | 1 |
| chr2 | 129296975 | C | A | 138 | Ckap2l | intronic | . | . | . | 2qF1 | rs256344788 | 0 | 1 |
| chr2 | 129297009 | G | A | 161 | Ckap2l | intronic | . | . | 318 | 2qF1 | rs27462768 | 0 | 1 |
| chr2 | 129302900 | T | C | 222 | Gm14023,Il1a | exonic | synonymous SNV | Il1a:NM_010554:exon6:c.A597G:p.Q199Q | . | 2qF1 | rs33300494 | 0 | 1 |
| chr2 | 129302960 | G | A | 222 | Il1a,Gm14023 | exonic | synonymous SNV | Il1a:NM_010554:exon6:c.C537T:p.D179D | . | 2qF1 | rs27447740 | 0 | 1 |
| chr2 | 129306612 | C | T | 222 | Gm14023,Il1a | exonic | synonymous SNV | Il1a:NM_010554:exon4:c.G177A:p.T59T | . | 2qF1 | rs27447731 | 0 | 1 |
| chr2 | 129379160 | C | T | 174 | . | intergenic | . | . | . | 2qF1 | . | 0 | 1 |
| chr2 | 129459440 | A | G | 222 | F830045P16Rik | intronic | . | . | . | 2qF1 | rs213756738 | 0 | 1 |
| chr2 | 129460303 | G | T | 221 | F830045P16Rik | intronic | . | . | . | 2qF1 | rs50672544 | 0 | 1 |
| chr2 | 129536295 | T | C | 99 | F830045P16Rik | intronic | . | . | . | 2qF1 | rs29719915 | 0 | 1 |
| chr2 | 129536628 | A | T | 58 | . | upstream | . | . | . | 2qF1 | rs29521845 | 0 | 1 |
| chr2 | 129608789 | T | C | 69 | Sirpa | intronic | . | . | . | 2qF1 | rs51431074 | 0 | 1 |
| chr2 | 129608794 | T | A | 73 | Sirpa | intronic | . | . | . | 2qF1 | rs27448101 | 0 | 1 |
| chr2 | 129609295 | T | A | 160 | Sirpa | intronic | . | . | . | 2qF1 | rs51942147 | 0 | 1 |
| chr2 | 129609344 | G | A | 105 | Sirpa | intronic | . | . | . | 2qF1 | rs27448093 | 0 | 1 |
| chr2 | 129615275 | T | G | 70 | Sirpa | intronic | . | . | . | 2qF1 | rs27433051 | 0 | 1 |
| chr2 | 129615588 | C | A | 222 | Sirpa | exonic | missense SNV | Sirpa:NM_001291019:exon3:c.C581A:p.P194H,Sirpa:NM_007547:exon3:c.C581A:p.P194H,Sirpa:NM_0012[illegible] | . | 2qF1 | rs32957782 | 0 | 1 |
| chr2 | 129615677 | A | C | 222 | Sirpa | exonic | missense SNV | Sirpa:NM_001291019:exon3:c.A670C:p.N224H,Sirpa:NM_007547:exon3:c.A670C:p.N224H,Sirpa:NM_0012[illegible] | . | 2qF1 | rs27433049 | 0 | 1 |
| chr2 | 129615819 | G | C | 222 | Sirpa | intronic | . | . | . | 2qF1 | rs27433048 | 0 | 1 |
| chr2 | 129615853 | A | C | 154 | Sirpa | intronic | . | . | . | 2qF1 | rs27433047 | 0 | 1 |
| chr2 | 129616039 | C | T | 88 | Sirpa | intronic | . | . | . | 2qF1 | rs223907541 | 0 | 1 |
| chr2 | 129618298 | T | A | 86 | Sirpa | intronic | . | . | . | 2qF1 | rs29557734 | 0 | 1 |
| chr2 | 129618299 | T | C | 83 | Sirpa | intronic | . | . | . | 2qF1 | rs33227751 | 0 | 1 |
| chr2 | 129618457 | A | G | 221 | Sirpa | exonic | missense SNV | Sirpa:NM_001291022:exon2:c.A83G:p.D28G,Sirpa:NM_001177647:exon3:c.A440G:p.D147G,Sirpa:NM_0012 | . | 2qF1 | rs27433024 | 0 | 1 |
| chr2 | 129618723 | T | C | 23.516 | Sirpa | intronic | . | . | . | 2qF1 | rs27433022 | 0 | 1 |
| chr2 | 129627773 | C | A | 110 | Sirpa | intronic | . | . | . | 2qF1 | rs29817344 | 0 | 1 |
| chr2 | 129628093 | A | C | 123 | Sirpa | intronic | . | . | . | 2qF1 | rs32852380 | 0 | 1 |
| chr2 | 129689679 | C | T | 136 | Pdyn | intronic | . | . | . | 2qF1 | . | 0 | 1 |
| chr2 | 129827739 | A | T | 180 | Stk35 | intronic | . | . | . | 2qF1 | rs27271363 | 0 | 1 |
| chr2 | 130026587 | A | C | 79 | Tgm3 | intronic | . | . | . | 2qF1 | rs29863737 | 0 | 1 |
| chr2 | 130026593 | T | C | 86 | Tgm3 | intronic | . | . | . | 2qF1 | rs29720282 | 0 | 1 |
| chr2 | 130030049 | T | C | 222 | Tgm3 | intronic | . | . | . | 2qF1 | rs238299153 | 0 | 1 |
| chr2 | 130037628 | T | C | 222 | Tgm3 | intronic | . | . | . | 2qF1 | rs27289446 | 0 | 1 |
| chr2 | 130038315 | G | C | 168 | Tgm3 | intronic | . | . | . | 2qF1 | rs27289442 | 0 | 1 |
| chr2 | 130041741 | C | T | 86 | Tgm3 | intronic | . | . | . | 2qF1 | rs27289408 | 0 | 1 |
| chr2 | 130041812 | C | T | 218 | Tgm3 | exonic | synonymous SNV | Tgm3:NM_009374:exon10:c.C1389T:p.N463N | . | 2qF1 | rs51008079 | 0 | 1 |
| chr2 | 130041965 | A | G | 222 | Tgm3 | exonic | synonymous SNV | Tgm3:NM_009374:exon10:c.A1542G:p.T514T | . | 2qF1 | rs27289406 | 0 | 1 |
| chr2 | 130042224 | C | T | 107 | Tgm3 | intronic | . | . | . | 2qF1 | rs27289405 | 0 | 1 |
| chr2 | 130042259 | G | T | 76 | Tgm3 | intronic | . | . | . | 2qF1 | rs27289404 | 0 | 1 |
| chr2 | 130047539 | G | A | 70 | Tgm3 | intronic | . | . | . | 2qF1 | rs27273163 | 0 | 1 |
| chr2 | 130047856 | G | T | 222 | Tgm3 | intronic | . | . | . | 2qF1 | rs27273161 | 0 | 1 |
| chr2 | 130047930 | A | C | 83 | Tgm3 | intronic | . | . | . | 2qF1 | rs27273160 | 0 | 1 |
| chr2 | 130100838 | T | C | 222 | AU015228 | exonic | synonymous SNV | AU015228:NM_001033197:exon1:c.T315C:p.Y105Y | . | 2qF1 | rs27273006 | 0 | 1 |
| chr2 | 130101042 | C | T | 222 | AU015228 | exonic | synonymous SNV | AU015228:NM_001033197:exon1:c.C519T:p.A173A | . | 2qF1 | rs27273004 | 0 | 1 |
| chr2 | 130101076 | G | T | 221 | AU015228 | exonic | stopgain | AU015228:NM_001033197:exon1:c.G553T:p.E185X | . | 2qF1 | . | 0 | 1 |
| chr2 | 130102444 | A | G | 222 | AU015228 | UTR3 | . | . | . | 2qF1 | rs27272995 | 0 | 1 |
| chr2 | 130102548 | C | T | 222 | AU015228 | UTR3 | . | . | . | 2qF1 | rs27272994 | 0 | 1 |
| chr2 | 130103011 | A | G | 222 | AU015228 | UTR3 | . | . | . | 2qF1 | rs4223486 | 0 | 1 |
| chr2 | 130123317 | A | T | 222 | Tgm6 | UTR5 | . | . | . | 2qF1 | rs27272872 | 0 | 1 |
| chr2 | 130141400 | T | A | 150 | Tgm6 | intronic | . | . | . | 2qF1 | . | 0 | 1 |
| chr2 | 130141967 | G | A | 222 | Tgm6 | intronic | . | . | . | 2qF1 | rs27258389 | 0 | 1 |
| chr2 | 130143126 | T | C | 136 | Tgm6 | intronic | . | . | . | 2qF1 | rs27258387 | 0 | 1 |
| chr2 | 130143415 | A | G | 222 | Tgm6 | exonic | synonymous SNV | Tgm6:NM_001289749:exon7:c.A468G:p.E156E,Tgm6:NM_001289748:exon9:c.A468G:p.E156E,Tgm6:NM_1777[illegible] | . | 2qF1 | rs27258386 | 0 | 1 |
| chr2 | 130143652 | A | G | 221 | Tgm6 | intronic | . | . | . | 2qF1 | rs27258384 | 0 | 1 |
| chr2 | 130143759 | T | C | 111 | Tgm6 | intronic | . | . | . | 2qF1 | rs27258381 | 0 | 1 |
| chr2 | 130145116 | G | A | 115 | Tgm6 | exonic | synonymous SNV | Tgm6:NM_001289749:exon8:c.G834A:p.V278V,Tgm6:NM_001289748:exon10:c.G834A:p.V278V,Tgm6:NM_177[illegible] | . | 2qF1 | rs29521606 | 0 | 1 |
| chr2 | 130145125 | A | G | 115 | Tgm6 | exonic | synonymous SNV | Tgm6:NM_001289749:exon8:c.A843G:p.P281P,Tgm6:NM_001289748:exon10:c.A843G:p.P281P,Tgm6:NM_177[illegible] | . | 2qF1 | rs27258366 | 0 | 1 |
| chr2 | 130145239 | T | C | 159 | Tgm6 | exonic | synonymous SNV | Tgm6:NM_001289749:exon8:c.T957C:p.P319P,Tgm6:NM_001289748:exon10:c.T957C:p.P319P,Tgm6:NM_177[illegible] | . | 2qF1 | rs27258364 | 0 | 1 |
| chr2 | 130151275 | A | G | 195 | Tgm6 | exonic | synonymous SNV | Tgm6:NM_001289749:exon9:c.A1011G:p.E337E,Tgm6:NM_001289748:exon11:c.A1011G:p.E337E,Tgm6:NM_17 | . | 2qF1 | rs27258324 | 0 | 1 |
| chr2 | 130151374 | C | T | 221 | Tgm6 | exonic | synonymous SNV | Tgm6:NM_001289749:exon9:c.C1110T:p.G370G,Tgm6:NM_001289748:exon11:c.C1110T:p.G370G,Tgm6:NM_17 | . | 2qF1 | rs27258323 | 0 | 1 |
| chr2 | 130151627 | G | A | 190 | Tgm6 | intronic | . | . | . | 2qF1 | rs50843155 | 0 | 1 |
| chr2 | 130151721 | T | C | 222 | Tgm6 | exonic | synonymous SNV | Tgm6:NM_001289749:exon10:c.T1210C:p.L404L,Tgm6:NM_001289748:exon12:c.T1210C:p.L404L,Tgm6:NM_1 | . | 2qF1 | rs27258318 | 0 | 1 |
| chr2 | 130151857 | G | A | 222 | Tgm6 | intronic | . | . | . | 2qF1 | rs27258316 | 0 | 1 |
| chr2 | 130151964 | C | T | 78 | Tgm6 | intronic | . | . | . | 2qF1 | rs27258315 | 0 | 1 |
| chr2 | 130152884 | G | A | 57 | Tgm6 | intronic | . | . | . | 2qF1 | rs3715413 | 0 | 1 |
| chr2 | 130152923 | A | T | 101 | Tgm6 | intronic | . | . | . | 2qF1 | rs3715943 | 0 | 1 |
| chr2 | 130152949 | T | C | 98 | Tgm6 | intronic | . | . | . | 2qF1 | rs3715981 | 0 | 1 |
| chr2 | 130172069 | A | G | 136 | Snrpb | intronic | . | . | . | 2qF1 | rs27258248 | 0 | 1 |
| chr2 | 130235083 | G | A | 56 | Tmc2 | intronic | . | . | . | 2qF1 | rs229381196 | 0 | 1 |
| chr2 | 130360151 | C | A | 221 | Ebf4 | intronic | . | . | . | 2qF1 | rs29516612 | 0 | 1 |
| chr2 | 130394115 | T | G | 222 | Cpxm1 | exonic | synonymous SNV | Cpxm1:NM_019696:exon9:c.A1126C:p.R376R | . | 2qF1 | rs13460789 | 0 | 1 |
| chr2 | 130394963 | G | A | 221 | Cpxm1 | exonic | missense SNV | Cpxm1:NM_019696:exon6:c.C758T:p.A253V | . | 2qF1 | rs27260717 | 0 | 1 |
| chr2 | 130396012 | A | G | 180 | Cpxm1 | intronic | . | . | . | 2qF1 | rs257757418 | 0 | 1 |
| chr2 | 130405541 | G | C | 222 | 1700020A23Rik | UTR5 | . | . | . | 2qF1 | rs27260679 | 0 | 1 |
| chr2 | 130405759 | C | T | 222 | 1700020A23Rik | intronic | . | . | . | 2qF1 | rs27260677 | 0 | 1 |
| chr2 | 130419720 | G | A | 222 | Pced1a | exonic | missense SNV | Pced1a:NM_178762:exon6:c.C1012T:p.P338S,Pced1a:NM_001114541:exon7:c.C1012T:p.P338S | . | 2qF1 | rs33645896 | 0 | 1 |
| chr2 | 130437820 | G | C | 221 | Vps16 | intronic | . | . | . | 2qF1 | rs27260572 | 0 | 1 |

| | | | | | | | | | | | | | |
|---|---|---|---|---|---|---|---|---|---|---|---|---|---|
| chr2 | 130438441 | A | G | 222 | Vps16 | exonic | synonymous SNV | Vps16:NM_030559:exon5:c.A504G:p.P168P | . | 2qF1 | rs27244067 | 0 | 1 |
| chr2 | 130438571 | C | A | 221 | Vps16 | exonic | missense SNV | Vps16:NM_030559:exon6:c.C517A:p.L173M | . | 2qF1 | rs27244065 | 0 | 1 |
| chr2 | 130439371 | C | T | 173 | Vps16 | intronic | . | . | . | 2qF1 | rs27244059 | 0 | 1 |
| chr2 | 130439594 | C | T | 221 | Vps16 | intronic | . | . | . | 2qF1 | rs27244058 | 0 | 1 |
| chr2 | 130473425 | G | T | 126 | Ptpra | intronic | . | . | . | 2qF1 | rs27243946 | 0 | 1 |
| chr2 | 130473532 | C | T | 187 | Ptpra | intronic | . | . | . | 2qF1 | rs228206866 | 0 | 1 |
| chr2 | 130473606 | T | C | 25.001 | Ptpra | intronic | . | . | . | 2qF1 | rs243309809 | 0 | 1 |
| chr2 | 130473891 | T | C | 52 | Ptpra | intronic | . | . | . | 2qF1 | rs254789226 | 0 | 1 |
| chr2 | 130473901 | C | A | 42.2507 | Ptpra | intronic | . | . | . | 2qF1 | rs263283949 | 0 | 1 |
| chr2 | 130473908 | G | A | 63 | Ptpra | intronic | . | . | . | 2qF1 | rs226342272 | 0 | 1 |
| chr2 | 130504361 | C | T | 144 | Ptpra | intronic | . | . | . | 2qF1 | rs27243852 | 0 | 1 |
| chr2 | 130532302 | A | G | 87 | Ptpra | intronic | . | . | . | 2qF1 | rs27292397 | 0 | 1 |
| chr2 | 130533224 | C | T | 211 | Ptpra | intronic | . | . | . | 2qF1 | rs27292389 | 0 | 1 |
| chr2 | 130539605 | G | A | 182 | Ptpra | intronic | . | . | . | 2qF1 | rs27275552 | 0 | 1 |
| chr2 | 130540369 | G | A | 86 | Ptpra | intronic | . | . | . | 2qF1 | rs27275545 | 0 | 1 |
| chr2 | 130540735 | G | A | 123 | Ptpra | intronic | . | . | . | 2qF1 | rs27275544 | 0 | 1 |
| chr2 | 130541361 | A | G | 222 | Ptpra | exonic | synonymous SNV | Ptpra:NM_001163688:exon13:c.A1368G:p.E456E,Ptpra:NM_008980:exon14:c.A1476G:p.E492E | . | 2qF1 | rs13474861 | 0 | 1 |
| chr2 | 130541379 | C | T | 222 | Ptpra | exonic | synonymous SNV | Ptpra:NM_001163688:exon13:c.C1386T:p.Y462Y,Ptpra:NM_008980:exon14:c.C1494T:p.Y498Y | . | 2qF1 | rs13459166 | 0 | 1 |
| chr2 | 130581117 | G | A | 106 | Avp | exonic | synonymous SNV | Avp:NM_009732:exon2:c.C273T:p.S91S | 590 | 2qF1 | rs33251255 | 0 | 1 |
| chr2 | 130635523 | A | G | 204 | Lzts3 | intronic | . | . | . | 2qF1 | rs33244645 | 0 | 1 |
| chr2 | 130686956 | A | G | 221 | Slc4a11 | exonic | missense SNV | Slc4a11:NM_001081162:exon14:c.T1372C:p.S458P | . | 2qF1 | . | 0 | 1 |
| chr2 | 130717259 | G | T | 221 | 4930402H24Rik | splicing | . | . | . | 2qF1 | . | 0 | 1 |
| chr2 | 130960227 | T | C | 99 | Atrn | intronic | . | . | . | 2qF1 | . | 0 | 1 |
| chr2 | 131070694 | A | G | 79 | Siglec1 | intronic | . | . | . | 2qF1 | rs50129760 | 0 | 1 |
| chr2 | 131077989 | C | T | 222 | Siglec1 | exonic | missense SNV | Siglec1:NM_011426:exon10:c.G2621A:p.G874E | . | 2qF1 | . | 0 | 1 |
| chr2 | 131083298 | C | A | 94 | Siglec1 | exonic | synonymous SNV | Siglec1:NM_011426:exon5:c.G1242T:p.V414V | . | 2qF1 | . | 0 | 1 |
| chr2 | 131847648 | C | A | 165 | 5330413P13Rik | ncRNA_exonic | . | . | . | 2qF1 | rs33065313 | 0 | 1 |
| chr2 | 131996322 | A | C | 137 | Rassf2 | UTR3 | . | . | . | 2qF2 | rs27274997 | 0 | 1 |
| chr2 | 132000286 | G | T | 53 | Rassf2 | intronic | . | . | . | 2qF2 | rs47579752 | 0 | 1 |
| chr2 | 132000376 | G | A | 96 | Rassf2 | intronic | . | . | . | 2qF2 | rs49455994 | 0 | 1 |
| chr2 | 132000413 | C | T | 124 | Rassf2 | intronic | . | . | . | 2qF2 | rs27258746 | 0 | 1 |
| chr2 | 132000572 | C | T | 222 | Rassf2 | intronic | . | . | . | 2qF2 | rs27258744 | 0 | 1 |
| chr2 | 132001493 | A | G | 27.0604 | Rassf2 | intronic | . | . | . | 2qF2 | rs27258740 | 0 | 1 |
| chr2 | 132001640 | G | A | 115 | Rassf2 | intronic | . | . | . | 2qF2 | rs33721088 | 0 | 1 |
| chr2 | 132001649 | A | G | 130 | Rassf2 | intronic | . | . | . | 2qF2 | rs32899887 | 0 | 1 |
| chr2 | 132001653 | C | T | 128 | Rassf2 | intronic | . | . | . | 2qF2 | rs32878507 | 0 | 1 |
| chr2 | 132001921 | C | T | 35.4799 | Rassf2 | intronic | . | . | . | 2qF2 | rs27258738 | 0 | 1 |
| chr2 | 132004149 | C | A | 110 | Rassf2 | intronic | . | . | . | 2qF2 | rs27258723 | 0 | 1 |
| chr2 | 132005289 | G | A | 221 | Rassf2 | intronic | . | . | . | 2qF2 | rs27258718 | 0 | 1 |
| chr2 | 132012763 | C | T | 78 | Rassf2 | intronic | . | . | . | 2qF2 | rs29726848 | 0 | 1 |
| chr2 | 132062224 | G | A | 75 | Slc23a2 | intronic | . | . | . | 2qF2 | . | 0 | 1 |
| chr2 | 132240277 | T | C | 56 | Tmem230 | UTR3 | . | . | . | 2qF2 | rs27261422 | 0 | 1 |
| chr2 | 132240516 | G | A | 222 | Tmem230 | exonic | synonymous SNV | Tmem230:NM_001141971:exon4:c.C333T:p.S111S,Tmem230:NM_027478:exon4:c.C333T:p.S111S | . | 2qF2 | rs27261421 | 0 | 1 |
| chr2 | 132240748 | G | A | 222 | Tmem230 | intronic | . | . | . | 2qF2 | rs107855871 | 0 | 1 |
| chr2 | 132240789 | A | T | 118 | Tmem230 | intronic | . | . | . | 2qF2 | rs27261420 | 0 | 1 |
| chr2 | 132240833 | C | T | 68 | Tmem230 | intronic | . | . | . | 2qF2 | rs223160479 | 0 | 1 |
| chr2 | 132243937 | T | C | 222 | Tmem230 | intronic | . | . | . | 2qF2 | rs27261417 | 0 | 1 |
| chr2 | 132246170 | T | C | 154 | Tmem230 | intronic | . | . | . | 2qF2 | rs33553931 | 0 | 1 |
| chr2 | 132249722 | C | T | 171 | Pcna | intronic | . | . | . | 2qF2 | rs46317482 | 0 | 1 |
| chr2 | 132249739 | A | G | 104 | Pcna | intronic | . | . | . | 2qF2 | rs47805852 | 0 | 1 |
| chr2 | 132249781 | C | T | 89 | Pcna | exonic | synonymous SNV | Pcna:NM_011045:exon5:c.G699A:p.V233V | . | 2qF2 | rs13461896 | 0 | 1 |
| chr2 | 132249862 | C | A | 221 | Pcna | exonic | synonymous SNV | Pcna:NM_011045:exon5:c.G618T:p.T206T | . | 2qF2 | . | 0 | 1 |
| chr2 | 132250040 | C | T | 118 | Pcna | intronic | . | . | . | 2qF2 | rs27261386 | 0 | 1 |
| chr2 | 132250087 | T | C | 69 | Pcna | intronic | . | . | . | 2qF2 | rs27261385 | 0 | 1 |
| chr2 | 132250105 | T | C | 31.5111 | Pcna | intronic | . | . | . | 2qF2 | rs29577137 | 0 | 1 |
| chr2 | 132251204 | C | T | 172 | Pcna | intronic | . | . | . | 2qF2 | rs29515417 | 0 | 1 |
| chr2 | 132251325 | C | T | 221 | Pcna | exonic | synonymous SNV | Pcna:NM_011045:exon4:c.G501A:p.V167V | . | 2qF2 | rs13461897 | 0 | 1 |
| chr2 | 132251446 | C | T | 221 | Pcna | intronic | . | . | . | 2qF2 | rs253611393 | 0 | 1 |
| chr2 | 132251530 | T | C | 221 | Pcna | intronic | . | . | . | 2qF2 | rs211770244 | 0 | 1 |
| chr2 | 132251625 | C | T | 213 | Pcna | intronic | . | . | . | 2qF2 | rs229011799 | 0 | 1 |
| chr2 | 132251637 | G | C | 221 | Pcna | intronic | . | . | . | 2qF2 | rs246540536 | 0 | 1 |
| chr2 | 132253127 | C | A | 117 | Pcna | UTR5 | . | . | 973 | 2qF2 | rs13461900 | 0 | 1 |
| chr2 | 132263294 | G | C | 87 | Cds2 | UTR5 | . | . | 799 | 2qF2 | rs33229126 | 0 | 1 |
| chr2 | 132263365 | G | C | 144 | Cds2 | UTR5 | . | . | 799 | 2qF2 | rs214210526 | 0 | 1 |
| chr2 | 132263415 | C | A | 109 | Cds2 | UTR5 | . | . | 799 | 2qF2 | rs33740137 | 0 | 1 |
| chr2 | 132263554 | A | G | 184 | Cds2 | intronic | . | . | 799 | 2qF2 | rs33659723 | 0 | 1 |
| chr2 | 132263571 | C | T | 177 | Cds2 | intronic | . | . | 799 | 2qF2 | rs33292536 | 0 | 1 |
| chr2 | 132293467 | T | C | 222 | Cds2 | intronic | . | . | . | 2qF2 | rs27261270 | 0 | 1 |
| chr2 | 132297080 | C | T | 96 | Cds2 | intronic | . | . | . | 2qF2 | rs246335196 | 0 | 1 |
| chr2 | 132297085 | G | A | 93 | Cds2 | intronic | . | . | . | 2qF2 | rs265501770 | 0 | 1 |
| chr2 | 132297162 | G | A | 167 | Cds2 | intronic | . | . | . | 2qF2 | rs27261238 | 0 | 1 |
| chr2 | 132297174 | C | T | 182 | Cds2 | intronic | . | . | . | 2qF2 | rs27261237 | 0 | 1 |
| chr2 | 132297264 | G | A | 221 | Cds2 | exonic | synonymous SNV | Cds2:NM_001291039:exon5:c.G375A:p.E125E,Cds2:NM_138651:exon5:c.G426A:p.E142E | . | 2qF2 | rs3691322 | 0 | 1 |
| chr2 | 132297537 | T | C | 152 | Cds2 | intronic | . | . | . | 2qF2 | rs49460284 | 0 | 1 |
| chr2 | 132297543 | A | G | 163 | Cds2 | intronic | . | . | . | 2qF2 | rs27261235 | 0 | 1 |
| chr2 | 132297587 | G | A | 106 | Cds2 | intronic | . | . | . | 2qF2 | rs27261234 | 0 | 1 |
| chr2 | 132298386 | A | G | 27.8075 | Cds2 | intronic | . | . | . | 2qF2 | rs51797633 | 0 | 1 |
| chr2 | 132298393 | G | T | 33.8921 | Cds2 | intronic | . | . | . | 2qF2 | rs50839062 | 0 | 1 |
| chr2 | 132298448 | G | A | 222 | Cds2 | intronic | . | . | . | 2qF2 | rs27261223 | 0 | 1 |
| chr2 | 132298613 | T | G | 221 | Cds2 | intronic | . | . | . | 2qF2 | rs27261219 | 0 | 1 |
| chr2 | 132298623 | A | G | 180 | Cds2 | intronic | . | . | . | 2qF2 | rs27261218 | 0 | 1 |
| chr2 | 132300450 | C | A | 61 | Cds2 | intronic | . | . | . | 2qF2 | rs27261200 | 0 | 1 |
| chr2 | 132300458 | C | T | 57 | Cds2 | intronic | . | . | . | 2qF2 | rs212504715 | 0 | 1 |
| chr2 | 132300482 | G | A | 95 | Cds2 | intronic | . | . | . | 2qF2 | rs227754648 | 0 | 1 |
| chr2 | 132302034 | G | T | 214 | Cds2 | intronic | . | . | . | 2qF2 | rs29672736 | 0 | 1 |
| chr2 | 132305294 | T | C | 169 | Cds2 | UTR3 | . | . | . | 2qF2 | rs27261187 | 0 | 1 |
| chr2 | 132341141 | C | T | 85 | . | intergenic | . | . | . | 2qF2 | rs27245504 | 0 | 1 |
| chr2 | 132381683 | G | C | 222 | Prokr2 | UTR5 | . | . | . | 2qF2 | rs27245326 | 0 | 1 |
| chr2 | 132530565 | C | T | 222 | Gpcpd1 | exonic | synonymous SNV | Gpcpd1:NM_027096:exon18:c.G1464A:p.V488V,Gpcpd1:NM_001291050:exon19:c.G1464A:p.V488V,Gpcpd1:N | . | 2qF2 | rs27255665 | 0 | 1 |
| chr2 | 132535614 | T | C | 108 | Gpcpd1 | intronic | . | . | . | 2qF2 | rs27255662 | 0 | 1 |
| chr2 | 132540231 | C | T | 146 | Gpcpd1 | intronic | . | . | . | 2qF2 | rs27255659 | 0 | 1 |
| chr2 | 132540430 | T | G | 222 | Gpcpd1 | intronic | . | . | . | 2qF2 | rs27255658 | 0 | 1 |
| chr2 | 132553969 | T | A | 72 | Gpcpd1 | exonic | missense SNV | Gpcpd1:NM_001042672:exon7:c.A461T:p.K154I,Gpcpd1:NM_028802:exon7:c.A461T:p.K154I | . | 2qF2 | . | 0 | 1 |
| chr2 | 132662247 | G | C | 62 | . | intergenic | . | . | . | 2qF2 | rs250716327 | 0 | 1 |
| chr2 | 132662255 | G | C | 40.7869 | . | intergenic | . | . | . | 2qF2 | rs222816125 | 0 | 1 |
| chr2 | 132662353 | T | A | 48.4736 | . | intergenic | . | . | . | 2qF2 | rs245389045 | 0 | 1 |
| chr2 | 132805583 | T | C | 222 | Trmt6 | exonic | synonymous SNV | Trmt6:NM_175113:exon11:c.A1332G:p.L444L | . | 2qF2 | rs6288723 | 0 | 1 |
| chr2 | 132806567 | C | T | 53 | Trmt6 | intronic | . | . | . | 2qF2 | rs29822761 | 0 | 1 |
| chr2 | 132808682 | T | C | 221 | Trmt6 | exonic | synonymous SNV | Trmt6:NM_175113:exon7:c.A1005G:p.E335E | . | 2qF2 | rs33014982 | 0 | 1 |
| chr2 | 132808839 | C | T | 222 | Trmt6 | exonic | missense SNV | Trmt6:NM_175113:exon7:c.G848A:p.S283N | . | 2qF2 | rs27259617 | 0 | 1 |
| chr2 | 132810001 | C | T | 222 | Trmt6 | intronic | . | . | . | 2qF2 | rs29820290 | 0 | 1 |
| chr2 | 132810068 | C | T | 166 | Trmt6 | intronic | . | . | . | 2qF2 | rs27259606 | 0 | 1 |
| chr2 | 132810276 | C | T | 213 | Trmt6 | exonic | missense SNV | Trmt6:NM_175113:exon5:c.G475A:p.V159I | . | 2qF2 | rs27259605 | 0 | 1 |
| chr2 | 132810404 | T | C | 160 | Trmt6 | intronic | . | . | . | 2qF2 | rs29958832 | 0 | 1 |
| chr2 | 132810455 | T | C | 109 | Trmt6 | exonic | synonymous SNV | Trmt6:NM_175113:exon4:c.A423G:p.E141E | . | 2qF2 | rs33411683 | 0 | 1 |
| chr2 | 132810929 | A | C | 131 | Trmt6 | intronic | . | . | . | 2qF2 | rs33013682 | 0 | 1 |
| chr2 | 132810937 | T | C | 127 | Trmt6 | intronic | . | . | . | 2qF2 | rs27259602 | 0 | 1 |
| chr2 | 132827259 | G | T | 94 | Mcm8 | intronic | . | . | . | 2qF2 | rs27259523 | 0 | 1 |
| chr2 | 132827961 | C | T | 148 | Mcm8 | exonic | synonymous SNV | Mcm8:NM_025676:exon9:c.C954T:p.F318F,Mcm8:NM_001291054:exon10:c.C1038T:p.F346F | . | 2qF2 | rs27259520 | 0 | 1 |
| chr2 | 132831535 | T | C | 149 | Mcm8 | intronic | . | . | . | 2qF2 | rs29773732 | 0 | 1 |
| chr2 | 132831705 | A | G | 154 | Mcm8 | intronic | . | . | . | 2qF2 | rs29861565 | 0 | 1 |
| chr2 | 132831763 | T | C | 59 | Mcm8 | intronic | . | . | . | 2qF2 | rs32947256 | 0 | 1 |
| chr2 | 132834889 | T | C | 222 | Mcm8 | intronic | . | . | . | 2qF2 | rs27259478 | 0 | 1 |
| chr2 | 132838335 | C | T | 128 | Mcm8 | intronic | . | . | . | 2qF2 | rs29927890 | 0 | 1 |
| chr2 | 132838938 | C | A | 222 | Mcm8 | intronic | . | . | . | 2qF2 | rs33653739 | 0 | 1 |
| chr2 | 132839000 | G | A | 94 | Mcm8 | intronic | . | . | . | 2qF2 | rs27259459 | 0 | 1 |
| chr2 | 132842593 | T | C | 210 | Mcm8 | intronic | . | . | . | 2qF2 | rs33732767 | 0 | 1 |
| chr2 | 132843336 | G | A | 124 | Mcm8 | intronic | . | . | . | 2qF2 | rs33320096 | 0 | 1 |
| chr2 | 132843694 | T | C | 108 | Mcm8 | UTR3 | . | . | . | 2qF2 | rs27259447 | 0 | 1 |
| chr2 | 132863923 | A | T | 51 | Crls1 | intronic | . | . | . | 2qF2 | rs27259397 | 0 | 1 |
| chr2 | 132865703 | G | A | 39.0029 | Crls1 | intronic | . | . | . | 2qF2 | . | 0 | 1 |
| chr2 | 132869753 | G | A | 145 | Lrrn4 | exonic | synonymous SNV | Lrrn4:NM_177303:exon5:c.C2149T:p.L717L | . | 2qF2 | rs27259369 | 0 | 1 |
| chr2 | 132870724 | T | C | 221 | Lrrn4 | exonic | missense SNV | Lrrn4:NM_177303:exon5:c.A1178G:p.E393G | . | 2qF2 | rs27245866 | 0 | 1 |
| chr2 | 132879743 | G | C | 120 | Lrrn4 | exonic | synonymous SNV | Lrrn4:NM_177303:exon2:c.C153G:p.T51T | 511 | 2qF2 | rs237786435 | 0 | 1 |
| chr2 | 132879757 | C | G | 135 | Lrrn4 | exonic | missense SNV | Lrrn4:NM_177303:exon2:c.G139C:p.D47H | 511 | 2qF2 | rs249086375 | 0 | 1 |
| chr2 | 132906500 | A | G | 60 | Fermt1 | UTR3 | . | . | . | 2qF2 | rs27245726 | 0 | 1 |
| chr2 | 132906535 | T | C | 84 | Fermt1 | UTR3 | . | . | . | 2qF2 | rs27245725 | 0 | 1 |
| chr2 | 132906680 | G | A | 222 | Fermt1 | exonic | synonymous SNV | Fermt1:NM_198029:exon15:c.C2022T:p.G674G | . | 2qF2 | rs27245724 | 0 | 1 |
| chr2 | 132906713 | C | T | 222 | Fermt1 | exonic | synonymous SNV | Fermt1:NM_198029:exon15:c.G1989A:p.T663T | . | 2qF2 | rs27245723 | 0 | 1 |
| chr2 | 132911282 | C | A | 221 | Fermt1 | exonic | missense SNV | Fermt1:NM_198029:exon14:c.G1756T:p.A586S | . | 2qF2 | rs27245702 | 0 | 1 |
| chr2 | 132915262 | G | C | 222 | Fermt1 | exonic | synonymous SNV | Fermt1:NM_198029:exon13:c.C1647G:p.V549V | . | 2qF2 | rs27245681 | 0 | 1 |
| chr2 | 132915378 | C | T | 222 | Fermt1 | intronic | . | . | . | 2qF2 | rs27245679 | 0 | 1 |
| chr2 | 132915390 | T | C | 156 | Fermt1 | intronic | . | . | . | 2qF2 | rs27245678 | 0 | 1 |
| chr2 | 132915406 | A | T | 117 | Fermt1 | intronic | . | . | . | 2qF2 | rs27245677 | 0 | 1 |
| chr2 | 132915930 | A | G | 221 | Fermt1 | exonic | missense SNV | Fermt1:NM_198029:exon12:c.T1564C:p.C522R | . | 2qF2 | rs27245668 | 0 | 1 |
| chr2 | 132916045 | C | T | 221 | Fermt1 | exonic | synonymous SNV | Fermt1:NM_198029:exon12:c.G1449A:p.Q483Q | . | 2qF2 | rs27245667 | 0 | 1 |
| chr2 | 132916066 | A | G | 213 | Fermt1 | exonic | synonymous SNV | Fermt1:NM_198029:exon12:c.T1428C:p.T476T | . | 2qF2 | rs27245666 | 0 | 1 |
| chr2 | 132916172 | T | C | 160 | Fermt1 | intronic | . | . | . | 2qF2 | rs27245665 | 0 | 1 |
| chr2 | 132917469 | C | T | 190 | Fermt1 | exonic | missense SNV | Fermt1:NM_198029:exon11:c.G1366A:p.D456N | . | 2qF2 | rs27245656 | 0 | 1 |
| chr2 | 132918634 | T | C | 222 | Fermt1 | splicing | . | . | . | 2qF2 | rs27245650 | 0 | 1 |
| chr2 | 132918724 | T | C | 222 | Fermt1 | intronic | . | . | . | 2qF2 | rs27245649 | 0 | 1 |
| chr2 | 132924862 | C | A | 112 | Fermt1 | intronic | . | . | . | 2qF2 | rs27245608 | 0 | 1 |
| chr2 | 132924949 | G | A | 222 | Fermt1 | intronic | . | . | . | 2qF2 | rs27245605 | 0 | 1 |
| chr2 | 132925898 | C | T | 96 | Fermt1 | intronic | . | . | . | 2qF2 | rs47657213 | 0 | 1 |
| chr2 | 132925914 | T | C | 97 | Fermt1 | intronic | . | . | . | 2qF2 | rs45889029 | 0 | 1 |
| chr2 | 132935180 | G | A | 54 | Fermt1 | intronic | . | . | . | 2qF2 | rs27245584 | 0 | 1 |
| chr2 | 132939118 | C | T | 25.7587 | Fermt1 | intronic | . | . | . | 2qF2 | rs27284959 | 0 | 1 |
| chr2 | 132939337 | G | A | 222 | Fermt1 | exonic | synonymous SNV | Fermt1:NM_198029:exon3:c.C330T:p.R110R | . | 2qF2 | rs27284957 | 0 | 1 |
| chr2 | 132939558 | C | T | 159 | Fermt1 | intronic | . | . | . | 2qF2 | rs27284956 | 0 | 1 |
| chr2 | 132939587 | T | C | 124 | Fermt1 | intronic | . | . | . | 2qF2 | rs27284955 | 0 | 1 |
| chr2 | 133200251 | G | A | 222 | . | intergenic | . | . | . | 2qF2 | rs241245361 | 0 | 1 |
| chr2 | 133560936 | A | C | 221 | Bmp2 | exonic | missense SNV | Bmp2:NM_007553:exon3:c.A406C:p.S136R | . | 2qF2 | . | 0 | 1 |
| chr2 | 134987893 | A | T | 29.783 | Plcb1 | intronic | . | . | . | 2qF3 | rs247038431 | 0 | 1 |
| chr2 | 134988228 | T | C | 222 | Plcb1 | intronic | . | . | . | 2qF3 | rs27253225 | 0 | 1 |
| chr2 | 134988262 | A | C | 203 | Plcb1 | intronic | . | . | . | 2qF3 | . | 0 | 1 |
| chr2 | 135220509 | T | A | 37.228 | Plcb1 | intronic | . | . | . | 2qF3 | . | 0 | 1 |
| chr2 | 135325562 | A | T | 176 | Plcb1 | intronic | . | . | . | 2qF3 | rs49647543 | 0 | 1 |
| chr2 | 135325565 | A | C | 166 | Plcb1 | intronic | . | . | . | 2qF3 | rs27270339 | 0 | 1 |
| chr2 | 135325736 | A | T | 222 | Plcb1 | exonic | synonymous SNV | Plcb1:NM_001145830:exon14:c.A1449T:p.S483S,Plcb1:NM_019677:exon14:c.A1449T:p.S483S | . | 2qF3 | rs49372054 | 0 | 1 |
| chr2 | 135330072 | A | G | 50 | Plcb1 | intronic | . | . | . | 2qF3 | rs27270298 | 0 | 1 |
| chr2 | 135330090 | A | G | 82 | Plcb1 | intronic | . | . | . | 2qF3 | rs48634267 | 0 | 1 |
| chr2 | 135330109 | T | C | 90 | Plcb1 | intronic | . | . | . | 2qF3 | rs27270297 | 0 | 1 |
| chr2 | 135330239 | T | A | 222 | Plcb1 | intronic | . | . | . | 2qF3 | rs27270295 | 0 | 1 |
| chr2 | 135330371 | A | G | 222 | Plcb1 | splicing | . | . | . | 2qF3 | rs27270294 | 0 | 1 |
| chr2 | 135330419 | G | A | 213 | Plcb1 | intronic | . | . | . | 2qF3 | rs108624284 | 0 | 1 |
| chr2 | 135330423 | G | A | 216 | Plcb1 | intronic | . | . | . | 2qF3 | rs108344906 | 0 | 1 |
| chr2 | 135333502 | T | C | 90 | Plcb1 | intronic | . | . | . | 2qF3 | rs27270268 | 0 | 1 |
| chr2 | 135337557 | A | T | 222 | Plcb1 | exonic | synonymous SNV | Plcb1:NM_001145830:exon18:c.A1884T:p.T628T,Plcb1:NM_019677:exon18:c.A1884T:p.T628T | . | 2qF3 | rs243815003 | 0 | 1 |
| chr2 | 135337667 | A | G | 192 | Plcb1 | intronic | . | . | . | 2qF3 | rs265040696 | 0 | 1 |
| chr2 | 135340582 | A | G | 177 | Plcb1 | intronic | . | . | . | 2qF3 | rs27270229 | 0 | 1 |
| chr2 | 135346205 | A | G | 141 | Plcb1 | intronic | . | . | . | 2qF3 | rs27270204 | 0 | 1 |
| chr2 | 135346379 | T | C | 222 | Plcb1 | exonic | synonymous SNV | Plcb1:NM_001145830:exon22:c.T2391C:p.D797D,Plcb1:NM_019677:exon22:c.T2391C:p.D797D | . | 2qF3 | rs27270201 | 0 | 1 |
| chr2 | 135346515 | T | C | 89 | Plcb1 | intronic | . | . | . | 2qF3 | rs27270200 | 0 | 1 |
| chr2 | 135347302 | G | C | 138 | Plcb1 | intronic | . | . | . | 2qF3 | rs27270194 | 0 | 1 |
| chr2 | 135359563 | T | G | 154 | Plcb1 | intronic | . | . | . | 2qF3 | rs27270179 | 0 | 1 |
| chr2 | 135359847 | C | A | 108 | Plcb1 | intronic | . | . | . | 2qF3 | rs256791138 | 0 | 1 |
| chr2 | 135359872 | A | G | 90 | Plcb1 | intronic | . | . | . | 2qF3 | rs220632566 | 0 | 1 |
| chr2 | 135362242 | C | T | 105 | Plcb1 | intronic | . | . | . | 2qF3 | rs47959203 | 0 | 1 |
| chr2 | 135362558 | G | A | 148 | Plcb1 | intronic | . | . | . | 2qF3 | rs252940036 | 0 | 1 |
| chr2 | 135362588 | C | A | 103 | Plcb1 | intronic | . | . | . | 2qF3 | rs216489705 | 0 | 1 |
| chr2 | 135364787 | C | T | 64 | Plcb1 | exonic | synonymous SNV | Plcb1:NM_001145830:exon26:c.C2730T:p.I910I,Plcb1:NM_019677:exon26:c.C2730T:p.I910I | . | 2qF3 | rs27256066 | 0 | 1 |
| chr2 | 135364886 | G | A | 175 | Plcb1 | exonic | synonymous SNV | Plcb1:NM_001145830:exon26:c.G2829A:p.E943E,Plcb1:NM_019677:exon26:c.G2829A:p.E943E | . | 2qF3 | rs27256064 | 0 | 1 |
| chr2 | 135364904 | G | A | 172 | Plcb1 | exonic | synonymous SNV | Plcb1:NM_001145830:exon26:c.G2847A:p.T949T,Plcb1:NM_019677:exon26:c.G2847A:p.T949T | . | 2qF3 | rs27256063 | 0 | 1 |
| chr2 | 135365083 | C | T | 63 | Plcb1 | intronic | . | . | . | 2qF3 | rs27256062 | 0 | 1 |
| chr2 | 135370329 | C | T | 102 | Plcb1 | intronic | . | . | . | 2qF3 | rs33185680 | 0 | 1 |

| | | | | | | | | | | | | | |
|---|---|---|---|---|---|---|---|---|---|---|---|---|---|
| chr2 | 135370405 | G | C | 169 | Plcb1 | intronic | . | . | . | 2qF3 | rs33203789 | 0 | 1 |
| chr2 | 135399121 | A | G | 60 | Plcb1 | intronic | . | . | . | 2qF3 | rs237885226 | 0 | 1 |
| chr2 | 135583139 | G | A | 179 | 9630028H03Rik | ncRNA_exonic | . | . | . | 2qF3 | rs27254388 | 0 | 1 |
| chr2 | 135583205 | C | T | 221 | 9630028H03Rik | ncRNA_exonic | . | . | . | 2qF3 | rs27254387 | 0 | 1 |
| chr2 | 135583411 | T | G | 38.8595 | . | upstream | . | . | . | 2qF3 | . | 0 | 1 |
| chr2 | 135954941 | A | C | 212 | Plcb4 | exonic | missense SNV | Plcb4:NM_013829:exon16:c.A1265C:p.K422T | . | 2qF3 | . | 0 | 1 |
| chr2 | 135971848 | G | A | 222 | Plcb4 | exonic | synonymous SNV | Plcb4:NM_013829:exon24:c.G2106A:p.T702T | . | 2qF3 | rs27283718 | 0 | 1 |
| chr2 | 135994241 | G | T | 163 | Plcb4 | intronic | . | . | . | 2qF3 | . | 0 | 1 |
| chr2 | 136502112 | A | G | 123 | . | intergenic | . | . | . | 2qF3 | rs27257014 | 0 | 1 |
| chr2 | 136502223 | A | T | 121 | . | intergenic | . | . | . | 2qF3 | rs259628703 | 0 | 1 |
| chr2 | 136502231 | G | C | 95 | . | intergenic | . | . | . | 2qF3 | rs27257013 | 0 | 1 |
| chr2 | 136502267 | G | C | 114 | . | intergenic | . | . | . | 2qF3 | rs27257012 | 0 | 1 |
| chr2 | 136502294 | C | T | 164 | . | intergenic | . | . | . | 2qF3 | rs27257011 | 0 | 1 |
| chr2 | 136502512 | C | A | 151 | . | intergenic | . | . | . | 2qF3 | rs27257010 | 0 | 1 |
| chr2 | 136549726 | A | G | 222 | Ankef1 | exonic | synonymous SNV | Ankef1:NM_175667:exon7:c.A834G:p.K278K | . | 2qF3 | rs27240921 | 0 | 1 |
| chr2 | 136550308 | C | A | 221 | Ankef1 | exonic | missense SNV | Ankef1:NM_175667:exon7:c.C1416A:p.H472Q | . | 2qF3 | rs220088492 | 0 | 1 |
| chr2 | 137092737 | A | G | 94 | Jag1 | intronic | . | . | . | 2qF3 | . | 0 | 1 |
| chr2 | 139537483 | A | G | 135 | Sptlc3 | intronic | . | . | . | 2qF3 | . | 0 | 1 |
| chr2 | 139625709 | C | A | 145 | Sptlc3 | intronic | . | . | . | 2qF3 | . | 0 | 1 |
| chr2 | 139977755 | C | A | 222 | Tasp1 | intronic | . | . | . | 2qF3 | . | 0 | 1 |
| chr2 | 140073377 | G | T | 222 | . | intergenic | . | . | . | 2qF3 | . | 0 | 1 |
| chr2 | 140120593 | A | T | 186 | Esf1 | UTR3 | . | . | . | 2qF3 | . | 0 | 1 |
| chr2 | 140420174 | T | G | 221 | Macrod2 | UTR3 | . | . | . | 2qF3 | . | 0 | 1 |
| chr2 | 141232039 | G | T | 222 | Macrod2 | intronic | . | . | . | 2qF3 | . | 0 | 1 |
| chr2 | 142317803 | A | G | 72 | Macrod2 | intronic | . | . | . | 2qG1 | . | 0 | 1 |
| chr2 | 143955149 | C | G | 180 | Rrbp1 | exonic | missense SNV | Rrbp1:NM_024281:exon13:c.G3382C:p.A1128P | . | 2qG1 | . | 0 | 1 |
| chr2 | 145850939 | A | G | 48.5369 | Rin2 | intronic | . | . | . | 2qG1 | rs27272513 | 1 | 0 |
| chr2 | 145851321 | G | A | 216 | Rin2 | intronic | . | . | . | 2qG1 | rs27272512 | 0 | 1 |
| chr2 | 145851364 | A | G | 107 | Rin2 | intronic | . | . | . | 2qG1 | rs27272511 | 0 | 1 |
| chr2 | 145860030 | G | A | 98 | Rin2 | exonic | synonymous SNV | Rin2:NM_028724:exon9:c.G510A:p.S170S | . | 2qG1 | rs233827346 | 0 | 1 |
| chr2 | 145860047 | T | C | 151 | Rin2 | exonic | missense SNV | Rin2:NM_028724:exon9:c.T527C:p.L176P | . | 2qG1 | rs262755024 | 0 | 1 |
| chr2 | 145860258 | G | C | 222 | Rin2 | exonic | synonymous SNV | Rin2:NM_028724:exon9:c.G738C:p.P246P | . | 2qG1 | rs27272478 | 0 | 1 |
| chr2 | 145860540 | G | C | 157 | Rin2 | exonic | missense SNV | Rin2:NM_028724:exon9:c.G1020C:p.R340S | 227 | 2qG1 | rs29574918 | 0 | 1 |
| chr2 | 145860666 | G | A | 182 | Rin2 | exonic | synonymous SNV | Rin2:NM_028724:exon9:c.G1146A:p.Q382Q | 227 | 2qG1 | rs33125419 | 0 | 1 |
| chr2 | 145860777 | T | C | 202 | Rin2 | exonic | synonymous SNV | Rin2:NM_028724:exon9:c.T1257C:p.A419A | . | 2qG1 | rs13463944 | 0 | 1 |
| chr2 | 145861188 | T | A | 166 | Rin2 | intronic | . | . | . | 2qG1 | rs33423966 | 0 | 1 |
| chr2 | 145861194 | T | C | 128 | Rin2 | intronic | . | . | . | 2qG1 | rs32825383 | 0 | 1 |
| chr2 | 145861202 | A | G | 142 | Rin2 | intronic | . | . | . | 2qG1 | rs33653742 | 0 | 1 |
| chr2 | 145878694 | G | T | 42.1649 | Rin2 | intronic | . | . | . | 2qG1 | rs27272411 | 0 | 1 |
| chr2 | 145878719 | A | G | 55 | Rin2 | intronic | . | . | . | 2qG1 | rs27272410 | 0 | 1 |
| chr2 | 145879002 | G | A | 221 | Rin2 | intronic | . | . | . | 2qG1 | rs3690107 | 0 | 1 |
| chr2 | 145879029 | G | A | 207 | Rin2 | intronic | . | . | . | 2qG1 | rs3690613 | 0 | 1 |
| chr2 | 145883254 | C | T | 198 | Rin2 | intronic | . | . | . | 2qG1 | rs3698945 | 0 | 1 |
| chr2 | 145883491 | C | T | 159 | Rin2 | intronic | . | . | . | 2qG1 | rs27272381 | 0 | 1 |
| chr2 | 145885423 | T | C | 82 | Rin2 | intronic | . | . | . | 2qG1 | rs234769216 | 0 | 1 |
| chr2 | 145885525 | T | C | 129 | Rin2 | intronic | . | . | . | 2qG1 | rs27272370 | 0 | 1 |
| chr2 | 145885713 | A | G | 222 | Rin2 | exonic | synonymous SNV | Rin2:NM_028724:exon13:c.A2367G:p.K789K | . | 2qG1 | rs27272369 | 0 | 1 |
| chr2 | 145903384 | T | C | 179 | Naa20 | intronic | . | . | 463 | 2qG1 | rs33065773 | 0 | 1 |
| chr2 | 145912373 | G | A | 221 | Naa20 | intronic | . | . | . | 2qG1 | rs29546270 | 0 | 1 |
| chr2 | 145912415 | G | A | 221 | Naa20 | exonic | synonymous SNV | Naa20:NM_001141965:exon4:c.G192A:p.S64S,Naa20:NM_026425:exon4:c.G222A:p.S74S | . | 2qG1 | rs13472638 | 0 | 1 |
| chr2 | 145912550 | A | T | 221 | Naa20 | intronic | . | . | . | 2qG1 | rs33752739 | 0 | 1 |
| chr2 | 145918307 | A | G | 70 | Crnkl1 | UTR3 | . | . | . | 2qG1 | rs27272334 | 0 | 1 |
| chr2 | 145918553 | A | G | 221 | Crnkl1 | exonic | synonymous SNV | Crnkl1:NM_025820:exon14:c.T1944C:p.D648D | . | 2qG1 | rs27272333 | 0 | 1 |
| chr2 | 145918624 | C | A | 135 | Crnkl1 | intronic | . | . | . | 2qG1 | rs27272332 | 0 | 1 |
| chr2 | 145919652 | C | A | 166 | Crnkl1 | intronic | . | . | . | 2qG1 | rs27272326 | 0 | 1 |
| chr2 | 145920059 | A | G | 153 | Crnkl1 | intronic | . | . | . | 2qG1 | rs27272324 | 0 | 1 |
| chr2 | 145920806 | T | C | 120 | Crnkl1 | intronic | . | . | . | 2qG1 | rs27272323 | 0 | 1 |
| chr2 | 145921850 | C | T | 221 | Crnkl1 | exonic | synonymous SNV | Crnkl1:NM_025820:exon11:c.G1467A:p.E489E | . | 2qG1 | rs27272319 | 0 | 1 |
| chr2 | 145922066 | G | C | 222 | Crnkl1 | intronic | . | . | . | 2qG1 | rs27272318 | 0 | 1 |
| chr2 | 145923928 | C | T | 222 | Crnkl1 | splicing | . | . | . | 2qG1 | rs27272317 | 0 | 1 |
| chr2 | 145924916 | C | G | 130 | Crnkl1 | intronic | . | . | . | 2qG1 | rs27272315 | 0 | 1 |
| chr2 | 145926589 | C | T | 23.4901 | Crnkl1 | intronic | . | . | . | 2qG1 | rs27272313 | 0 | 1 |
| chr2 | 145926628 | T | C | 74 | Crnkl1 | intronic | . | . | . | 2qG1 | rs27272312 | 0 | 1 |
| chr2 | 145926722 | G | C | 168 | Crnkl1 | intronic | . | . | . | 2qG1 | rs27272311 | 0 | 1 |
| chr2 | 145928028 | A | T | 82 | Crnkl1 | intronic | . | . | . | 2qG1 | rs27272306 | 0 | 1 |
| chr2 | 145930420 | G | A | 90 | Crnkl1 | intronic | . | . | . | 2qG1 | rs27272297 | 0 | 1 |
| chr2 | 145930447 | G | A | 91 | Crnkl1 | intronic | . | . | . | 2qG1 | rs27272296 | 0 | 1 |
| chr2 | 145931305 | A | C | 221 | Crnkl1 | exonic | synonymous SNV | Crnkl1:NM_025820:exon4:c.T420G:p.R140R | . | 2qG1 | rs13466733 | 0 | 1 |
| chr2 | 145931553 | T | C | 103 | Crnkl1 | intronic | . | . | . | 2qG1 | rs33700658 | 0 | 1 |
| chr2 | 145931574 | T | C | 107 | Crnkl1 | intronic | . | . | . | 2qG1 | rs27272295 | 0 | 1 |
| chr2 | 145932187 | T | C | 113 | Crnkl1 | intronic | . | . | . | 2qG1 | rs27272290 | 0 | 1 |
| chr2 | 145932212 | T | G | 118 | Crnkl1 | intronic | . | . | . | 2qG1 | rs27272289 | 0 | 1 |
| chr2 | 145932704 | A | G | 95 | Crnkl1 | intronic | . | . | . | 2qG1 | rs27272285 | 0 | 1 |
| chr2 | 145932719 | C | T | 117 | Crnkl1 | intronic | . | . | . | 2qG1 | rs33444520 | 0 | 1 |
| chr2 | 145934670 | G | A | 78 | Crnkl1 | UTR5 | . | . | 725 | 2qG1 | rs27272281 | 0 | 1 |
| chr2 | 145939935 | C | T | 222 | Cfap61 | exonic | synonymous SNV | Cfap61:NM_175280:exon2:c.C87T:p.N29N | . | 2qG1 | rs27257867 | 0 | 1 |
| chr2 | 145946990 | T | C | 200 | Cfap61 | intronic | . | . | . | 2qG1 | rs27257833 | 0 | 1 |
| chr2 | 145947004 | T | C | 222 | Cfap61 | intronic | . | . | . | 2qG1 | rs27257832 | 0 | 1 |
| chr2 | 145947232 | C | T | 222 | Cfap61 | intronic | . | . | . | 2qG1 | rs27257831 | 0 | 1 |
| chr2 | 145947348 | A | G | 111 | Cfap61 | intronic | . | . | . | 2qG1 | rs27257830 | 0 | 1 |
| chr2 | 145947435 | T | C | 40.8655 | Cfap61 | intronic | . | . | . | 2qG1 | rs27257829 | 0 | 1 |
| chr2 | 145949900 | G | A | 86 | Cfap61 | intronic | . | . | . | 2qG1 | rs33166597 | 0 | 1 |
| chr2 | 145949916 | C | T | 99 | Cfap61 | splicing | . | . | . | 2qG1 | rs51652665 | 0 | 1 |
| chr2 | 145950568 | C | A | 115 | Cfap61 | intronic | . | . | . | 2qG1 | rs29969729 | 0 | 1 |
| chr2 | 145950703 | G | C | 222 | Cfap61 | intronic | . | . | . | 2qG1 | rs33728739 | 0 | 1 |
| chr2 | 145951152 | T | C | 222 | Cfap61 | exonic | synonymous SNV | Cfap61:NM_175280:exon6:c.T534C:p.N178N | . | 2qG1 | rs27257810 | 0 | 1 |
| chr2 | 145963489 | T | C | 222 | Cfap61 | exonic | missense SNV | Cfap61:NM_175280:exon7:c.T758C:p.F253S | . | 2qG1 | rs27257758 | 0 | 1 |
| chr2 | 145973293 | T | C | 72 | . | intergenic | . | . | . | 2qG1 | rs27257724 | 0 | 1 |
| chr2 | 145973417 | G | T | 190 | . | intergenic | . | . | . | 2qG1 | rs249424346 | 0 | 1 |
| chr2 | 145973450 | T | C | 196 | . | intergenic | . | . | . | 2qG1 | rs27257723 | 0 | 1 |
| chr2 | 145973647 | T | C | 222 | . | intergenic | . | . | . | 2qG1 | rs27257722 | 0 | 1 |
| chr2 | 145973717 | C | T | 136 | . | intergenic | . | . | . | 2qG1 | rs27257721 | 0 | 1 |
| chr2 | 146001664 | A | G | 56 | . | intergenic | . | . | . | 2qG1 | rs46882712 | 0 | 1 |
| chr2 | 146001796 | A | G | 191 | . | intergenic | . | . | . | 2qG1 | rs264409639 | 0 | 1 |
| chr2 | 146008747 | G | A | 100 | . | intergenic | . | . | . | 2qG1 | rs29866454 | 0 | 1 |
| chr2 | 146009070 | G | A | 104 | . | intergenic | . | . | . | 2qG1 | rs29505856 | 0 | 1 |
| chr2 | 146011937 | C | T | 98 | . | intergenic | . | . | . | 2qG1 | rs260685667 | 0 | 1 |
| chr2 | 146012047 | C | A | 220 | . | intergenic | . | . | . | 2qG1 | rs228327853 | 0 | 1 |
| chr2 | 146012119 | A | T | 222 | . | intergenic | . | . | . | 2qG1 | rs246838119 | 0 | 1 |
| chr2 | 146012301 | C | G | 222 | . | intergenic | . | . | . | 2qG1 | rs29573590 | 0 | 1 |
| chr2 | 146012309 | C | A | 222 | . | intergenic | . | . | . | 2qG1 | rs32827570 | 0 | 1 |
| chr2 | 146014151 | A | C | 90 | . | intergenic | . | . | . | 2qG1 | rs27257689 | 0 | 1 |
| chr2 | 146014216 | C | T | 116 | . | intergenic | . | . | . | 2qG1 | rs108611180 | 0 | 1 |
| chr2 | 146014398 | T | A | 79 | . | intergenic | . | . | . | 2qG1 | rs27257688 | 0 | 1 |
| chr2 | 146014431 | C | T | 72 | . | intergenic | . | . | . | 2qG1 | rs27257687 | 0 | 1 |
| chr2 | 146047083 | T | A | 31.9949 | . | intergenic | . | . | . | 2qG1 | rs27257624 | 0 | 1 |
| chr2 | 146047094 | C | T | 56 | . | intergenic | . | . | . | 2qG1 | rs27257623 | 0 | 1 |
| chr2 | 146047231 | G | T | 195 | . | intergenic | . | . | . | 2qG1 | rs27257622 | 0 | 1 |
| chr2 | 146047261 | A | G | 221 | . | intergenic | . | . | . | 2qG1 | rs27257621 | 0 | 1 |
| chr2 | 146047323 | A | G | 148 | . | intergenic | . | . | . | 2qG1 | rs27257620 | 0 | 1 |
| chr2 | 146047370 | G | C | 134 | . | intergenic | . | . | . | 2qG1 | rs27257619 | 0 | 1 |
| chr2 | 146047435 | A | G | 91 | . | intergenic | . | . | . | 2qG1 | rs27257618 | 0 | 1 |
| chr2 | 146077268 | T | A | 46.8331 | . | intergenic | . | . | . | 2qG1 | rs27242780 | 0 | 1 |
| chr2 | 146077509 | A | C | 64 | . | intergenic | . | . | . | 2qG1 | rs27242778 | 0 | 1 |
| chr2 | 146095468 | G | A | 128 | . | intergenic | . | . | . | 2qG1 | rs46437329 | 0 | 1 |
| chr2 | 146095718 | A | T | 221 | . | intergenic | . | . | . | 2qG1 | . | 0 | 1 |
| chr2 | 146095845 | C | G | 120 | . | intergenic | . | . | . | 2qG1 | rs258917928 | 0 | 1 |
| chr2 | 146101802 | G | C | 90 | . | intergenic | . | . | . | 2qG1 | rs27242680 | 0 | 1 |
| chr2 | 146101830 | G | A | 139 | . | intergenic | . | . | . | 2qG1 | rs27242679 | 0 | 1 |
| chr2 | 146102136 | A | G | 112 | . | intergenic | . | . | . | 2qG1 | rs32994647 | 0 | 1 |
| chr2 | 146102143 | T | C | 118 | . | intergenic | . | . | . | 2qG1 | rs223426661 | 0 | 1 |
| chr2 | 146102168 | C | G | 36.2889 | . | intergenic | . | . | . | 2qG1 | rs241904190 | 0 | 1 |
| chr2 | 146109772 | C | T | 43.3247 | . | intergenic | . | . | . | 2qG1 | rs50516191 | 0 | 1 |
| chr2 | 146109845 | T | C | 161 | . | intergenic | . | . | . | 2qG1 | rs27242646 | 0 | 1 |
| chr2 | 146110033 | T | A | 222 | . | intergenic | . | . | . | 2qG1 | . | 0 | 1 |
| chr2 | 146110110 | A | G | 221 | . | intergenic | . | . | . | 2qG1 | rs27242644 | 0 | 1 |
| chr2 | 146110199 | A | T | 131 | . | intergenic | . | . | . | 2qG1 | rs27242643 | 0 | 1 |
| chr2 | 146120285 | T | C | 45.459 | . | intergenic | . | . | . | 2qG1 | rs27242587 | 0 | 1 |
| chr2 | 146120478 | A | G | 222 | . | intergenic | . | . | . | 2qG1 | rs27242584 | 0 | 1 |
| chr2 | 146143501 | A | C | 106 | . | intergenic | . | . | . | 2qG1 | rs27272062 | 0 | 1 |
| chr2 | 146145341 | C | T | 180 | . | intergenic | . | . | . | 2qG1 | rs46059456 | 0 | 1 |
| chr2 | 146145364 | C | A | 210 | . | intergenic | . | . | . | 2qG1 | rs48742257 | 0 | 1 |
| chr2 | 146145485 | G | A | 134 | . | intergenic | . | . | . | 2qG1 | rs47322701 | 0 | 1 |
| chr2 | 146145500 | T | C | 105 | . | intergenic | . | . | . | 2qG1 | rs45675494 | 0 | 1 |
| chr2 | 146145512 | A | C | 104 | . | intergenic | . | . | . | 2qG1 | rs51373775 | 0 | 1 |
| chr2 | 146153492 | C | A | 70 | . | intergenic | . | . | . | 2qG1 | rs47623348 | 0 | 1 |
| chr2 | 146153754 | T | C | 221 | . | intergenic | . | . | . | 2qG1 | . | 0 | 1 |
| chr2 | 146153964 | G | A | 141 | . | intergenic | . | . | . | 2qG1 | rs47023300 | 0 | 1 |
| chr2 | 146154000 | G | A | 78 | . | intergenic | . | . | . | 2qG1 | rs51728519 | 0 | 1 |
| chr2 | 146200740 | C | T | 97 | . | intergenic | . | . | . | 2qG1 | rs27278825 | 0 | 1 |
| chr2 | 146214521 | C | T | 81 | . | intergenic | . | . | . | 2qG1 | rs33616190 | 0 | 1 |
| chr2 | 146214876 | T | G | 98 | . | intergenic | . | . | . | 2qG1 | rs29911470 | 0 | 1 |
| chr2 | 146248032 | A | G | 111 | Ralgapa2 | intronic | . | . | . | 2qG1 | rs48788400 | 0 | 1 |
| chr2 | 146248296 | C | T | 222 | Ralgapa2 | intronic | . | . | . | 2qG1 | rs27247169 | 0 | 1 |
| chr2 | 146248380 | C | T | 135 | Ralgapa2 | intronic | . | . | . | 2qG1 | rs27247168 | 0 | 1 |
| chr2 | 146258849 | C | T | 194 | Ralgapa2 | intronic | . | . | . | 2qG1 | rs27247128 | 0 | 1 |
| chr2 | 146260378 | C | G | 221 | Ralgapa2,A930019I | exonic | missense SNV | Ralgapa2:NM_001033348:exon39:c.G5716C:p.V1906L | . | 2qG1 | rs27247125 | 0 | 1 |
| chr2 | 146260523 | A | C | 221 | Ralgapa2,A930019I | ncRNA_intronic | . | . | . | 2qG1 | rs27247124 | 0 | 1 |
| chr2 | 146314976 | T | C | 221 | Ralgapa2 | exonic | synonymous SNV | Ralgapa2:NM_001033348:exon38:c.A5553G:p.T1851T | . | 2qG1 | rs27291461 | 0 | 1 |
| chr2 | 146315081 | G | A | 89 | Ralgapa2 | intronic | . | . | . | 2qG1 | rs27291460 | 0 | 1 |
| chr2 | 146315177 | T | C | 68 | Ralgapa2 | intronic | . | . | . | 2qG1 | rs33186858 | 0 | 1 |
| chr2 | 146333212 | C | T | 47.2951 | Ralgapa2 | intronic | . | . | . | 2qG1 | rs27291414 | 0 | 1 |
| chr2 | 146342300 | T | G | 222 | Ralgapa2 | exonic | missense SNV | Ralgapa2:NM_001033348:exon33:c.A4645C:p.N1549H | . | 2qG1 | rs27291367 | 0 | 1 |
| chr2 | 146342325 | T | C | 222 | Ralgapa2 | exonic | synonymous SNV | Ralgapa2:NM_001033348:exon33:c.A4620G:p.T1540T | . | 2qG1 | rs27291366 | 0 | 1 |
| chr2 | 146342804 | A | G | 203 | Ralgapa2 | intronic | . | . | . | 2qG1 | rs27291365 | 0 | 1 |
| chr2 | 146342830 | T | G | 92 | Ralgapa2 | intronic | . | . | . | 2qG1 | rs33532709 | 0 | 1 |
| chr2 | 146345208 | T | G | 56 | Ralgapa2 | intronic | . | . | . | 2qG1 | rs33056644 | 0 | 1 |
| chr2 | 146345301 | A | G | 144 | Ralgapa2 | intronic | . | . | . | 2qG1 | rs27291343 | 0 | 1 |
| chr2 | 146345543 | G | C | 221 | Ralgapa2 | exonic | synonymous SNV | Ralgapa2:NM_001033348:exon32:c.C4038G:p.S1346S | . | 2qG1 | rs29722729 | 0 | 1 |
| chr2 | 146345616 | A | G | 193 | Ralgapa2 | intronic | . | . | . | 2qG1 | rs27291341 | 0 | 1 |
| chr2 | 146345647 | G | A | 131 | Ralgapa2 | intronic | . | . | . | 2qG1 | rs27291340 | 0 | 1 |
| chr2 | 146346652 | T | C | 162 | Ralgapa2 | intronic | . | . | . | 2qG1 | rs33273686 | 0 | 1 |
| chr2 | 146346664 | A | C | 184 | Ralgapa2 | intronic | . | . | . | 2qG1 | rs32970094 | 0 | 1 |
| chr2 | 146347259 | G | T | 222 | Ralgapa2 | intronic | . | . | . | 2qG1 | rs33760942 | 0 | 1 |
| chr2 | 146347292 | A | T | 112 | Ralgapa2 | intronic | . | . | . | 2qG1 | rs27291337 | 0 | 1 |
| chr2 | 146347306 | A | T | 84 | Ralgapa2 | intronic | . | . | . | 2qG1 | rs27291336 | 0 | 1 |
| chr2 | 146348463 | G | C | 176 | Ralgapa2 | exonic | synonymous SNV | Ralgapa2:NM_001033348:exon29:c.C3789G:p.P1263P | . | 2qG1 | rs33685840 | 0 | 1 |
| chr2 | 146348622 | A | G | 105 | Ralgapa2 | intronic | . | . | . | 2qG1 | rs33387162 | 0 | 1 |
| chr2 | 146348626 | C | A | 113 | Ralgapa2 | intronic | . | . | . | 2qG1 | rs32919459 | 0 | 1 |
| chr2 | 146353127 | A | G | 146 | Ralgapa2 | intronic | . | . | . | 2qG1 | rs52261125 | 0 | 1 |
| chr2 | 146353129 | A | C | 168 | Ralgapa2 | intronic | . | . | . | 2qG1 | rs49852146 | 0 | 1 |
| chr2 | 146353437 | T | C | 23.5286 | Ralgapa2 | intronic | . | . | . | 2qG1 | rs27291301 | 0 | 1 |
| chr2 | 146353447 | G | C | 42.3497 | Ralgapa2 | intronic | . | . | . | 2qG1 | rs46174965 | 0 | 1 |
| chr2 | 146358211 | T | A | 127 | Ralgapa2 | intronic | . | . | . | 2qG1 | rs27291287 | 0 | 1 |
| chr2 | 146361626 | C | G | 137 | Ralgapa2 | intronic | . | . | . | 2qG1 | rs27291271 | 0 | 1 |
| chr2 | 146387165 | C | T | 222 | Ralgapa2 | intronic | . | . | . | 2qG1 | rs27291201 | 0 | 1 |
| chr2 | 146387712 | C | T | 222 | Ralgapa2 | intronic | . | . | . | 2qG1 | rs29577344 | 0 | 1 |
| chr2 | 146388827 | G | A | 59 | Ralgapa2 | intronic | . | . | . | 2qG1 | rs27291198 | 0 | 1 |
| chr2 | 146405187 | G | A | 90 | Ralgapa2 | intronic | . | . | . | 2qG1 | rs27291169 | 0 | 1 |
| chr2 | 146410192 | T | G | 158 | Ralgapa2 | exonic | missense SNV | Ralgapa2:NM_001033348:exon19:c.A2531C:p.H844P | . | 2qG1 | rs257482641 | 0 | 1 |
| chr2 | 146410193 | G | A | 162 | Ralgapa2 | exonic | missense SNV | Ralgapa2:NM_001033348:exon19:c.C2530T:p.H844Y | . | 2qG1 | rs216921401 | 0 | 1 |
| chr2 | 146410194 | A | G | 151 | Ralgapa2 | exonic | synonymous SNV | Ralgapa2:NM_001033348:exon19:c.T2529C:p.T843T | . | 2qG1 | rs237955486 | 0 | 1 |
| chr2 | 146410257 | T | G | 221 | Ralgapa2 | exonic | missense SNV | Ralgapa2:NM_001033348:exon19:c.A2466C:p.E822D | . | 2qG1 | . | 0 | 1 |
| chr2 | 146411959 | C | T | 130 | Ralgapa2 | intronic | . | . | . | 2qG1 | rs27343136 | 0 | 1 |
| chr2 | 146412888 | A | G | 103 | Ralgapa2 | intronic | . | . | . | 2qG1 | rs29628068 | 0 | 1 |

| | | | | | | | | | | | | | |
|---|---|---|---|---|---|---|---|---|---|---|---|---|---|
| chr2 | 146421729 | T | G | 222 | Ralgapa2 | exonic | synonymous SNV | Ralgapa2:NM_001033348:exon16:c.A2067C:p.T689T | . | 2qG1 | rs27343092 | 0 | 1 |
| chr2 | 146421752 | T | C | 202 | Ralgapa2 | exonic | missense SNV | Ralgapa2:NM_001033348:exon16:c.A2044G:p.I682V | . | 2qG1 | rs48111556 | 0 | 1 |
| chr2 | 146421756 | A | C | 203 | Ralgapa2 | exonic | synonymous SNV | Ralgapa2:NM_001033348:exon16:c.T2040G:p.G680G | . | 2qG1 | rs47901317 | 0 | 1 |
| chr2 | 146424520 | A | G | 130 | Ralgapa2 | intronic | . | . | . | 2qG1 | rs48301884 | 0 | 1 |
| chr2 | 146424699 | G | C | 222 | Ralgapa2 | exonic | synonymous SNV | Ralgapa2:NM_001033348:exon15:c.C1956G:p.A652A | . | 2qG1 | rs29917152 | 0 | 1 |
| chr2 | 146424914 | A | G | 204 | Ralgapa2 | intronic | . | . | . | 2qG1 | rs33119613 | 0 | 1 |
| chr2 | 146431769 | T | C | 194 | Ralgapa2 | intronic | . | . | . | 2qG1 | rs33115501 | 0 | 1 |
| chr2 | 146432009 | C | T | 221 | Ralgapa2 | intronic | . | . | . | 2qG1 | rs46696532 | 0 | 1 |
| chr2 | 146435825 | A | G | 221 | Ralgapa2 | intronic | . | . | . | 2qG1 | rs33088455 | 0 | 1 |
| chr2 | 146435901 | T | C | 212 | Ralgapa2 | exonic | missense SNV | Ralgapa2:NM_001033348:exon11:c.A1339G:p.K447E | . | 2qG1 | rs33700655 | 0 | 1 |
| chr2 | 146435908 | T | C | 215 | Ralgapa2 | exonic | synonymous SNV | Ralgapa2:NM_001033348:exon11:c.A1332G:p.E444E | . | 2qG1 | rs29541136 | 0 | 1 |
| chr2 | 146436142 | C | T | 75 | Ralgapa2 | intronic | . | . | . | 2qG1 | rs33717155 | 0 | 1 |
| chr2 | 146443772 | G | A | 222 | Ralgapa2 | intronic | . | . | . | 2qG1 | rs231004673 | 0 | 1 |
| chr2 | 146449173 | G | C | 89 | Ralgapa2 | intronic | . | . | . | 2qG1 | rs27327457 | 0 | 1 |
| chr2 | 146449199 | A | G | 81 | Ralgapa2 | intronic | . | . | . | 2qG1 | rs27327456 | 0 | 1 |
| chr2 | 146449238 | T | C | 84 | Ralgapa2 | exonic | synonymous SNV | Ralgapa2:NM_001033348:exon8:c.A777G:p.K259K | . | 2qG1 | rs27327455 | 0 | 1 |
| chr2 | 146450291 | A | T | 99 | Ralgapa2 | intronic | . | . | . | 2qG1 | rs33747402 | 0 | 1 |
| chr2 | 146451723 | T | C | 222 | Ralgapa2 | exonic | synonymous SNV | Ralgapa2:NM_001033348:exon6:c.A510G:p.T170T | . | 2qG1 | rs27327451 | 0 | 1 |
| chr2 | 146451936 | C | A | 32.508 | Ralgapa2 | intronic | . | . | . | 2qG1 | rs27327450 | 0 | 1 |
| chr2 | 146460673 | A | C | 145 | Ralgapa2 | intronic | . | . | . | 2qG1 | rs27327421 | 0 | 1 |
| chr2 | 146479556 | G | A | 87 | Ralgapa2 | intronic | . | . | . | 2qG1 | rs32832751 | 0 | 1 |
| chr2 | 146485300 | C | G | 100 | Ralgapa2 | intronic | . | . | . | 2qG1 | rs27327328 | 0 | 1 |
| chr2 | 146754691 | G | A | 185 | . | intergenic | . | . | . | 2qG1 | rs387590245 | 0 | 1 |
| chr2 | 146754853 | T | C | 188 | . | intergenic | . | . | . | 2qG1 | rs583625239 | 0 | 1 |
| chr2 | 146754892 | C | T | 116 | . | intergenic | . | . | . | 2qG1 | rs579770047 | 0 | 1 |
| chr2 | 146754925 | C | T | 121 | . | intergenic | . | . | . | 2qG1 | rs582985344 | 0 | 1 |
| chr2 | 146754996 | G | A | 204 | . | intergenic | . | . | . | 2qG1 | rs51188464 | 0 | 1 |
| chr2 | 146755131 | G | A | 222 | . | intergenic | . | . | . | 2qG1 | rs33175904 | 0 | 1 |
| chr2 | 146755167 | G | A | 222 | . | intergenic | . | . | . | 2qG1 | rs32859870 | 0 | 1 |
| chr2 | 146755251 | G | A | 221 | . | intergenic | . | . | . | 2qG1 | rs33075403 | 0 | 1 |
| chr2 | 146755382 | G | A | 189 | . | intergenic | . | . | . | 2qG1 | . | 0 | 1 |
| chr2 | 146755593 | A | G | 222 | . | intergenic | . | . | . | 2qG1 | rs29543245 | 0 | 1 |
| chr2 | 146755692 | C | T | 214 | . | intergenic | . | . | . | 2qG1 | rs32820596 | 0 | 1 |
| chr2 | 146755714 | C | T | 214 | . | intergenic | . | . | . | 2qG1 | rs33285875 | 0 | 1 |
| chr2 | 146755818 | G | T | 161 | . | intergenic | . | . | . | 2qG1 | rs27290877 | 0 | 1 |
| chr2 | 146860964 | A | G | 84 | Kiz | intronic | . | . | . | 2qG1 | rs27328568 | 0 | 1 |
| chr2 | 146870615 | C | T | 70 | Kiz | intronic | . | . | . | 2qG1 | rs27328524 | 0 | 1 |
| chr2 | 146870824 | T | C | 222 | Kiz | exonic | missense SNV | Kiz:NM_001033298:exon4:c.T407C:p.M136T | . | 2qG1 | rs27328523 | 0 | 1 |
| chr2 | 146871029 | T | C | 51 | Kiz | intronic | . | . | . | 2qG1 | rs27328522 | 0 | 1 |
| chr2 | 146882234 | G | A | 167 | Kiz | intronic | . | . | . | 2qG1 | rs33284521 | 0 | 1 |
| chr2 | 146882249 | A | C | 162 | Kiz | intronic | . | . | . | 2qG1 | rs32896047 | 0 | 1 |
| chr2 | 146882390 | G | T | 222 | Kiz | intronic | . | . | . | 2qG1 | rs27328470 | 0 | 1 |
| chr2 | 146882701 | G | A | 222 | Kiz | intronic | . | . | . | 2qG1 | rs33730524 | 0 | 1 |
| chr2 | 146882961 | A | T | 132 | Kiz | intronic | . | . | . | 2qG1 | rs32922096 | 0 | 1 |
| chr2 | 146889252 | G | A | 222 | Kiz | exonic | missense SNV | Kiz:NM_001033298:exon5:c.G661A:p.G221S | . | 2qG1 | rs27328442 | 0 | 1 |
| chr2 | 146889700 | T | C | 222 | Kiz | intronic | . | . | . | 2qG1 | rs27328440 | 0 | 1 |
| chr2 | 146889741 | A | T | 221 | Kiz | intronic | . | . | . | 2qG1 | rs50082129 | 0 | 1 |
| chr2 | 146890886 | A | T | 56 | Kiz | intronic | . | . | . | 2qG1 | rs29719005 | 0 | 1 |
| chr2 | 146890893 | A | G | 56 | Kiz | intronic | . | . | . | 2qG1 | rs33140270 | 0 | 1 |
| chr2 | 146891029 | A | T | 119 | Kiz | exonic | missense SNV | Kiz:NM_001033298:exon6:c.A1124T:p.H375L | . | 2qG1 | rs27328439 | 0 | 1 |
| chr2 | 146891078 | A | G | 209 | Kiz | exonic | synonymous SNV | Kiz:NM_001033298:exon6:c.A1173G:p.T391T | . | 2qG1 | rs27328438 | 0 | 1 |
| chr2 | 146935673 | T | A | 111 | Kiz | intronic | . | . | . | 2qG2 | rs29570985 | 0 | 1 |
| chr2 | 146935906 | T | C | 222 | Kiz | intronic | . | . | . | 2qG2 | rs27313957 | 0 | 1 |
| chr2 | 146943066 | T | C | 146 | Kiz | intronic | . | . | . | 2qG2 | rs27313916 | 0 | 1 |
| chr2 | 146952857 | T | G | 97 | Kiz | intronic | . | . | . | 2qG2 | rs27313870 | 0 | 1 |
| chr2 | 146953098 | T | G | 221 | Kiz | intronic | . | . | . | 2qG2 | rs29671417 | 0 | 1 |
| chr2 | 146953141 | T | G | 135 | Kiz | intronic | . | . | . | 2qG2 | rs27313869 | 0 | 1 |
| chr2 | 146956091 | A | G | 112 | Kiz | intronic | . | . | . | 2qG2 | rs27313847 | 0 | 1 |
| chr2 | 146969310 | T | C | 51 | Kiz | intronic | . | . | . | 2qG2 | rs47705643 | 0 | 1 |
| chr2 | 146969456 | C | T | 208 | Kiz | intronic | . | . | . | 2qG2 | rs27313796 | 0 | 1 |
| chr2 | 146969473 | C | T | 222 | Kiz | intronic | . | . | . | 2qG2 | rs48048480 | 0 | 1 |
| chr2 | 146969661 | T | C | 222 | Kiz | intronic | . | . | . | 2qG2 | rs27313794 | 0 | 1 |
| chr2 | 146969685 | C | G | 195 | Kiz | intronic | . | . | . | 2qG2 | rs27313793 | 0 | 1 |
| chr2 | 146969735 | G | T | 58 | Kiz | intronic | . | . | . | 2qG2 | rs27313792 | 0 | 1 |
| chr2 | 146969917 | G | A | 222 | Kiz | intronic | . | . | . | 2qG2 | rs228328141 | 0 | 1 |
| chr2 | 146970003 | C | T | 221 | Kiz | exonic | synonymous SNV | Kiz:NM_001033298:exon14:c.C2031T:p.A677A | . | 2qG2 | rs27313790 | 0 | 1 |
| chr2 | 146970071 | G | C | 221 | Kiz | UTR3 | . | . | . | 2qG2 | rs47056380 | 0 | 1 |
| chr2 | 146970159 | C | G | 114 | . | downstream | . | . | . | 2qG2 | rs27313789 | 0 | 1 |
| chr2 | 147024423 | C | T | 151 | Xrn2 | intronic | . | . | . | 2qG2 | rs27295066 | 0 | 1 |
| chr2 | 147025070 | T | C | 51 | Xrn2 | intronic | . | . | . | 2qG2 | rs27295064 | 0 | 1 |
| chr2 | 147025380 | A | G | 95 | Xrn2 | intronic | . | . | . | 2qG2 | rs217082705 | 0 | 1 |
| chr2 | 147025385 | T | C | 119 | Xrn2 | intronic | . | . | . | 2qG2 | rs237792435 | 0 | 1 |
| chr2 | 147026254 | G | A | 99 | Xrn2 | intronic | . | . | . | 2qG2 | rs27295057 | 0 | 1 |
| chr2 | 147026347 | A | G | 221 | Xrn2 | intronic | . | . | . | 2qG2 | rs27295056 | 0 | 1 |
| chr2 | 147028100 | A | C | 140 | Xrn2 | exonic | synonymous SNV | Xrn2:NM_011917:exon9:c.A708C:p.L236L | . | 2qG2 | rs13471168 | 0 | 1 |
| chr2 | 147029644 | T | A | 221 | Xrn2 | intronic | . | . | . | 2qG2 | rs27295043 | 0 | 1 |
| chr2 | 147029708 | A | G | 221 | Xrn2 | intronic | . | . | . | 2qG2 | rs27295042 | 0 | 1 |
| chr2 | 147036582 | G | T | 151 | Xrn2 | intronic | . | . | . | 2qG2 | rs33383411 | 0 | 1 |
| chr2 | 147036932 | G | A | 120 | Xrn2 | intronic | . | . | . | 2qG2 | rs33516217 | 0 | 1 |
| chr2 | 147040684 | T | G | 90 | Xrn2 | intronic | . | . | . | 2qG2 | rs33193493 | 0 | 1 |
| chr2 | 147040733 | G | A | 111 | Xrn2 | intronic | . | . | . | 2qG2 | rs29858508 | 0 | 1 |
| chr2 | 147041073 | A | T | 92 | Xrn2 | intronic | . | . | . | 2qG2 | rs33661616 | 0 | 1 |
| chr2 | 147042820 | G | A | 222 | Xrn2 | intronic | . | . | . | 2qG2 | rs29499660 | 0 | 1 |
| chr2 | 147047864 | A | G | 104 | Xrn2 | intronic | . | . | . | 2qG2 | rs27295014 | 0 | 1 |
| chr2 | 147049144 | A | G | 95 | Xrn2 | intronic | . | . | . | 2qG2 | rs27295012 | 0 | 1 |
| chr2 | 147049169 | T | G | 109 | Xrn2 | intronic | . | . | . | 2qG2 | rs27295011 | 0 | 1 |
| chr2 | 147049412 | A | G | 222 | Xrn2 | intronic | . | . | . | 2qG2 | rs27295010 | 0 | 1 |
| chr2 | 147049425 | T | C | 222 | Xrn2 | intronic | . | . | . | 2qG2 | rs27295009 | 0 | 1 |
| chr2 | 147050012 | C | T | 221 | Xrn2 | exonic | synonymous SNV | Xrn2:NM_011917:exon23:c.C2148T:p.H716H | . | 2qG2 | rs13471171 | 0 | 1 |
| chr2 | 147050177 | C | A | 85 | Xrn2 | intronic | . | . | . | 2qG2 | rs33595079 | 0 | 1 |
| chr2 | 147051608 | T | G | 120 | Xrn2 | intronic | . | . | . | 2qG2 | rs27295006 | 0 | 1 |
| chr2 | 147051701 | A | G | 222 | Xrn2 | intronic | . | . | . | 2qG2 | rs27295005 | 0 | 1 |
| chr2 | 147061641 | A | G | 163 | Xrn2 | intronic | . | . | . | 2qG2 | rs27294982 | 0 | 1 |
| chr2 | 147063187 | T | A | 183 | Xrn2 | intronic | . | . | . | 2qG2 | rs27294977 | 0 | 1 |
| chr2 | 147063407 | C | T | 219 | Xrn2 | intronic | . | . | . | 2qG2 | rs29817683 | 0 | 1 |
| chr2 | 147068724 | T | C | 59 | Xrn2 | intronic | . | . | . | 2qG2 | rs27294951 | 0 | 1 |
| chr2 | 147068748 | C | T | 175 | Xrn2 | intronic | . | . | . | 2qG2 | rs27294950 | 0 | 1 |
| chr2 | 147068783 | G | A | 190 | Xrn2 | intronic | . | . | . | 2qG2 | rs27294949 | 0 | 1 |
| chr2 | 147074710 | G | A | 124 | Xrn2 | intronic | . | . | . | 2qG2 | rs27294932 | 0 | 1 |
| chr2 | 147075141 | A | G | 118 | Xrn2 | intronic | . | . | . | 2qG2 | rs257949862 | 0 | 1 |
| chr2 | 147075211 | T | G | 76 | Xrn2 | intronic | . | . | . | 2qG2 | rs27294931 | 0 | 1 |
| chr2 | 147077295 | G | A | 70 | Xrn2 | intronic | . | . | . | 2qG2 | rs33417691 | 0 | 1 |
| chr2 | 147077711 | C | T | 39.3265 | Xrn2 | UTR3 | . | . | . | 2qG2 | rs13471170 | 0 | 1 |
| chr2 | 147177589 | A | G | 72 | Nkx2-2 | UTR3 | . | . | . | 2qG2 | rs29773728 | 0 | 1 |
| chr2 | 147177682 | A | C | 107 | Nkx2-2 | UTR3 | . | . | . | 2qG2 | rs50425189 | 0 | 1 |
| chr2 | 147177691 | G | A | 137 | Nkx2-2 | UTR3 | . | . | . | 2qG2 | rs33339130 | 0 | 1 |
| chr2 | 147177772 | C | T | 222 | Nkx2-2 | exonic | synonymous SNV | Nkx2-2:NM_001077632:exon3:c.G744A:p.Q248Q | . | 2qG2 | rs33122231 | 0 | 1 |
| chr2 | 147177861 | G | C | 222 | Nkx2-2 | exonic | missense SNV | Nkx2-2:NM_001077632:exon3:c.C655G:p.Q219E | . | 2qG2 | rs33639760 | 0 | 1 |
| chr2 | 147178001 | T | C | 222 | Nkx2-2 | intronic | . | . | . | 2qG2 | rs33592874 | 0 | 1 |
| chr2 | 147184212 | C | T | 101 | Nkx2-2os,Nkx2-2 | exonic | missense SNV | Nkx2-2:NM_010919:exon2:c.G605A:p.R202H | 644 | 2qG2 | . | 0 | 1 |
| chr2 | 147187686 | T | C | 139 | Nkx2-2os,6430503I | ncRNA_intronic | . | . | . | 2qG2 | rs27342825 | 0 | 1 |
| chr2 | 147187993 | A | T | 147 | 6430503K07Rik,Nk: | ncRNA_intronic | . | . | . | 2qG2 | rs33187305 | 0 | 1 |
| chr2 | 147188061 | T | C | 189 | 6430503K07Rik,Nk: | ncRNA_exonic | . | . | 261 | 2qG2 | rs27342823 | 0 | 1 |
| chr2 | 147188112 | C | T | 184 | 6430503K07Rik,Nk: | ncRNA_exonic | . | . | 261 | 2qG2 | rs29544755 | 0 | 1 |
| chr2 | 147280580 | G | T | 23.5017 | Nkx2-2os | ncRNA_intronic | . | . | . | 2qG2 | . | 0 | 1 |
| chr2 | 147280631 | T | G | 164 | Nkx2-2os | ncRNA_intronic | . | . | . | 2qG2 | rs29567706 | 0 | 1 |
| chr2 | 147280707 | A | G | 204 | Nkx2-2os | ncRNA_intronic | . | . | . | 2qG2 | rs27313608 | 0 | 1 |
| chr2 | 147280738 | T | C | 221 | Nkx2-2os | ncRNA_intronic | . | . | . | 2qG2 | rs47334000 | 0 | 1 |
| chr2 | 147280748 | C | T | 221 | Nkx2-2os | ncRNA_intronic | . | . | . | 2qG2 | rs32984489 | 0 | 1 |
| chr2 | 147280876 | A | T | 222 | Nkx2-2os | ncRNA_intronic | . | . | . | 2qG2 | rs49202636 | 0 | 1 |
| chr2 | 147289741 | A | G | 114 | Nkx2-2os | ncRNA_intronic | . | . | . | 2qG2 | rs27313577 | 0 | 1 |
| chr2 | 147290103 | A | G | 221 | Nkx2-2os | ncRNA_intronic | . | . | . | 2qG2 | rs32901169 | 0 | 1 |
| chr2 | 147290265 | A | G | 222 | Nkx2-2os | ncRNA_intronic | . | . | . | 2qG2 | rs33569553 | 0 | 1 |
| chr2 | 147290437 | A | G | 222 | Nkx2-2os | ncRNA_intronic | . | . | . | 2qG2 | rs6165851 | 0 | 1 |
| chr2 | 147290588 | G | A | 222 | Nkx2-2os | ncRNA_intronic | . | . | . | 2qG2 | rs3669927 | 0 | 1 |
| chr2 | 147290661 | A | G | 221 | Nkx2-2os | ncRNA_intronic | . | . | . | 2qG2 | rs3670080 | 0 | 1 |
| chr2 | 147290721 | C | T | 222 | Nkx2-2os | ncRNA_intronic | . | . | . | 2qG2 | rs3684827 | 0 | 1 |
| chr2 | 147290892 | C | T | 154 | Nkx2-2os | ncRNA_intronic | . | . | . | 2qG2 | rs3686009 | 0 | 1 |
| chr2 | 147290985 | C | T | 58 | Nkx2-2os | ncRNA_intronic | . | . | . | 2qG2 | rs33004544 | 0 | 1 |
| chr2 | 147331471 | A | G | 67 | Nkx2-2os | ncRNA_exonic | . | . | . | 2qG2 | rs4140021 | 0 | 1 |
| chr2 | 147331759 | A | G | 222 | . | downstream | . | . | . | 2qG2 | rs3719269 | 0 | 1 |
| chr2 | 147331784 | G | T | 184 | . | downstream | . | . | . | 2qG2 | rs3719727 | 0 | 1 |
| chr2 | 147331791 | T | A | 136 | . | downstream | . | . | . | 2qG2 | rs3719741 | 0 | 1 |
| chr2 | 147366148 | C | T | 221 | Pax1 | exonic | synonymous SNV | Pax1:NM_008780:exon2:c.C675T:p.Y225Y | 1498 | 2qG2 | rs33275121 | 0 | 1 |
| chr2 | 147366373 | G | A | 222 | Pax1 | intronic | . | . | 1498 | 2qG2 | rs48509440 | 0 | 1 |
| chr2 | 147366425 | A | G | 221 | Pax1 | intronic | . | . | . | 2qG2 | rs47789120 | 0 | 1 |
| chr2 | 147367783 | C | T | 79 | Pax1 | intronic | . | . | . | 2qG2 | rs33146224 | 0 | 1 |
| chr2 | 147367887 | T | C | 68 | Pax1 | exonic | synonymous SNV | Pax1:NM_008780:exon3:c.T900C:p.A300A | . | 2qG2 | rs239478444 | 0 | 1 |
| chr2 | 147367932 | G | C | 137 | Pax1 | exonic | synonymous SNV | Pax1:NM_008780:exon3:c.G945C:p.A315A | . | 2qG2 | rs265233657 | 0 | 1 |
| chr2 | 147367947 | G | A | 165 | Pax1 | exonic | synonymous SNV | Pax1:NM_008780:exon3:c.G960A:p.A320A | . | 2qG2 | rs222335723 | 0 | 1 |
| chr2 | 147402977 | G | A | 67 | . | intergenic | . | . | . | 2qG2 | rs238987009 | 0 | 1 |
| chr2 | 147403123 | C | G | 222 | . | intergenic | . | . | . | 2qG2 | rs27294501 | 0 | 1 |
| chr2 | 147403201 | A | G | 181 | . | intergenic | . | . | . | 2qG2 | rs27294500 | 0 | 1 |
| chr2 | 147403211 | G | A | 86 | . | intergenic | . | . | . | 2qG2 | rs27294499 | 0 | 1 |
| chr2 | 147642923 | C | G | 57 | . | intergenic | . | . | . | 2qG2 | rs46487672 | 0 | 1 |
| chr2 | 147642927 | T | C | 64 | . | intergenic | . | . | . | 2qG2 | rs215050832 | 0 | 1 |
| chr2 | 147642931 | A | C | 64 | . | intergenic | . | . | . | 2qG2 | rs49159298 | 0 | 1 |
| chr2 | 147643033 | A | G | 57 | . | intergenic | . | . | . | 2qG2 | rs49309559 | 0 | 1 |
| chr2 | 147643038 | A | G | 35.6855 | . | intergenic | . | . | . | 2qG2 | rs51486652 | 0 | 1 |
| chr2 | 147643059 | G | A | 83 | . | intergenic | . | . | . | 2qG2 | rs27340718 | 0 | 1 |
| chr2 | 147643142 | T | C | 73 | . | intergenic | . | . | . | 2qG2 | rs27340716 | 0 | 1 |
| chr2 | 147710536 | C | T | 67 | . | upstream | . | . | . | 2qG2 | rs48429337 | 0 | 1 |
| chr2 | 147710812 | A | G | 222 | A530006G24Rik | ncRNA_exonic | . | . | . | 2qG2 | rs32970021 | 0 | 1 |
| chr2 | 147716631 | C | T | 221 | A530006G24Rik | ncRNA_exonic | . | . | . | 2qG2 | rs33289670 | 0 | 1 |
| chr2 | 147716641 | G | A | 211 | A530006G24Rik | ncRNA_exonic | . | . | . | 2qG2 | rs32854684 | 0 | 1 |
| chr2 | 148043781 | G | A | 201 | Foxa2 | exonic | synonymous SNV | Foxa2:NM_001291065:exon2:c.C1131T:p.Y377Y,Foxa2:NM_001291067:exon2:c.C1023T:p.Y341Y,Foxa2:NM | . | 2qG3 | rs28265650 | 0 | 1 |
| chr2 | 148045830 | G | C | 102 | Foxa2 | exonic | synonymous SNV | Foxa2:NM_001291065:exon1:c.C84G:p.P28P,Foxa2:NM_010446:exon2:c.C66G:p.P22P | 3089 | 2qG3 | rs28265644 | 0 | 1 |
| chr2 | 148686701 | C | T | 103 | Gzf1 | intronic | . | . | . | 2qG3 | rs262942118 | 0 | 1 |
| chr2 | 148750865 | C | T | 194 | Cst11 | UTR5 | . | . | . | 2qG3 | rs28210847 | 0 | 1 |
| chr2 | 148750915 | C | T | 222 | Cst11 | UTR5 | . | . | . | 2qG3 | rs28210845 | 0 | 1 |
| chr2 | 148751076 | T | C | 222 | Cst11 | exonic | synonymous SNV | Cst11:NM_177655:exon2:c.T150C:p.F50F | . | 2qG3 | rs28210844 | 0 | 1 |
| chr2 | 148751097 | C | T | 147 | Cst11 | exonic | synonymous SNV | Cst11:NM_177655:exon2:c.C171T:p.N57N | . | 2qG3 | rs28210843 | 0 | 1 |
| chr2 | 148751173 | G | A | 33.9308 | Cst11 | intronic | . | . | . | 2qG3 | rs28210842 | 0 | 1 |
| chr2 | 148754437 | A | C | 99 | Cst11 | intronic | . | . | . | 2qG3 | rs28210806 | 0 | 1 |
| chr2 | 148754477 | A | G | 113 | Cst11 | intronic | . | . | . | 2qG3 | rs32831670 | 0 | 1 |
| chr2 | 148754583 | A | G | 222 | Cst11 | intronic | . | . | . | 2qG3 | rs28210805 | 0 | 1 |
| chr2 | 148754674 | C | T | 222 | Cst11 | exonic | missense SNV | Cst11:NM_177655:exon3:c.C296T:p.T99M | . | 2qG3 | rs28210804 | 0 | 1 |
| chr2 | 148755253 | C | T | 139 | Cst11 | intronic | . | . | . | 2qG3 | rs46050425 | 0 | 1 |
| chr2 | 148768625 | T | G | 212 | Cst11 | UTR3 | . | . | . | 2qG3 | rs49875805 | 0 | 1 |
| chr2 | 148768653 | A | G | 222 | Cst11 | UTR3 | . | . | . | 2qG3 | rs48447714 | 0 | 1 |
| chr2 | 148770276 | A | G | 118 | Cst11 | intronic | . | . | . | 2qG3 | rs47905983 | 0 | 1 |
| chr2 | 148770295 | C | G | 129 | Cst11 | intronic | . | . | . | 2qG3 | rs28210758 | 0 | 1 |
| chr2 | 148770491 | T | C | 222 | Cst11 | splicing | . | . | . | 2qG3 | rs29958538 | 0 | 1 |
| chr2 | 148770533 | T | C | 178 | Cst11 | intronic | . | . | . | 2qG3 | rs28210757 | 0 | 1 |
| chr2 | 148771236 | G | A | 220 | Cst11 | exonic | synonymous SNV | Cst11:NM_030059:exon1:c.C168T:p.N56N | . | 2qG3 | rs33355303 | 0 | 1 |
| chr2 | 148771394 | C | T | 175 | Cst11 | exonic | missense SNV | Cst11:NM_030059:exon1:c.G10A:p.G4R | . | 2qG3 | rs28210752 | 0 | 1 |
| chr2 | 148782036 | C | A | 134 | 8030411F24Rik | UTR5 | . | . | . | 2qG3 | rs29773611 | 0 | 1 |
| chr2 | 148782107 | C | T | 191 | 8030411F24Rik | UTR5 | . | . | . | 2qG3 | rs13476828 | 0 | 1 |
| chr2 | 148782390 | A | G | 155 | 8030411F24Rik | intronic | . | . | . | 2qG3 | rs28210680 | 0 | 1 |
| chr2 | 148783183 | A | G | 54 | 8030411F24Rik | intronic | . | . | . | 2qG3 | rs6293314 | 0 | 1 |
| chr2 | 148783198 | T | G | 66 | 8030411F24Rik | intronic | . | . | . | 2qG3 | rs6293335 | 0 | 1 |
| chr2 | 148783228 | A | G | 95 | 8030411F24Rik | intronic | . | . | . | 2qG3 | rs6293751 | 0 | 1 |
| chr2 | 148783338 | C | T | 222 | 8030411F24Rik | intronic | . | . | . | 2qG3 | rs6294346 | 0 | 1 |
| chr2 | 148783480 | C | A | 201 | 8030411F24Rik | intronic | . | . | . | 2qG3 | rs27296055 | 0 | 1 |
| chr2 | 148783577 | T | C | 51 | 8030411F24Rik | intronic | . | . | . | 2qG3 | rs27296054 | 0 | 1 |

| | | | | | | | | | | | | | |
|---|---|---|---|---|---|---|---|---|---|---|---|---|---|
| chr2 | 148783603 | T | C | 37.2844 | 8030411F24Rik | intronic | . | . | . | 2qG3 | rs27296053 | 0 | 1 |
| chr2 | 148783622 | A | C | 20.7239 | 8030411F24Rik | intronic | . | . | . | 2qG3 | rs52513800 | 0 | 1 |
| chr2 | 148785888 | G | A | 222 | 8030411F24Rik | UTR3 | . | . | . | 2qG3 | rs27296048 | 0 | 1 |
| chr2 | 148789702 | C | A | 128 | Cst12 | intronic | . | . | . | 2qG3 | rs27296001 | 0 | 1 |
| chr2 | 148793352 | G | A | 222 | Cst12 | UTR3 | . | . | . | 2qG3 | rs27277343 | 0 | 1 |
| chr2 | 148799939 | C | T | 48.5427 | Cst8 | UTR5 | . | . | . | 2qG3 | rs27277284 | 0 | 1 |
| chr2 | 148799941 | T | A | 49.5282 | Cst8 | UTR5 | . | . | . | 2qG3 | rs48685711 | 0 | 1 |
| chr2 | 148800256 | A | G | 222 | Cst8 | intronic | . | . | . | 2qG3 | rs27277283 | 0 | 1 |
| chr2 | 148804444 | C | T | 73 | Cst8 | intronic | . | . | . | 2qG3 | rs27277255 | 0 | 1 |
| chr2 | 148804470 | C | T | 53 | Cst8 | intronic | . | . | . | 2qG3 | rs49678777 | 0 | 1 |
| chr2 | 148804559 | A | G | 182 | Cst8 | intronic | . | . | . | 2qG3 | rs27277254 | 0 | 1 |
| chr2 | 148804704 | G | A | 182 | Cst8 | intronic | . | . | . | 2qG3 | rs27277253 | 0 | 1 |
| chr2 | 148804721 | A | G | 121 | Cst8 | intronic | . | . | . | 2qG3 | rs27277252 | 0 | 1 |
| chr2 | 148804753 | T | C | 82 | Cst8 | intronic | . | . | . | 2qG3 | rs27277251 | 0 | 1 |
| chr2 | 148805283 | C | T | 57 | Cst8 | intronic | . | . | . | 2qG3 | rs51018323 | 0 | 1 |
| chr2 | 148805289 | A | G | 62 | Cst8 | intronic | . | . | . | 2qG3 | rs27277243 | 0 | 1 |
| chr2 | 148805292 | C | A | 58 | Cst8 | intronic | . | . | . | 2qG3 | rs50899643 | 0 | 1 |
| chr2 | 148805542 | C | T | 222 | Cst8 | UTR3 | . | . | . | 2qG3 | rs27277240 | 0 | 1 |
| chr2 | 148805619 | T | C | 62 | . | downstream | . | . | . | 2qG3 | rs29820032 | 0 | 1 |
| chr2 | 148823003 | A | G | 93 | Cst13 | UTR5 | . | . | . | 2qG3 | rs32864267 | 0 | 1 |
| chr2 | 148828026 | T | G | 38.405 | Cst13 | intronic | . | . | . | 2qG3 | rs27277118 | 0 | 1 |
| chr2 | 148828066 | C | T | 123 | Cst13 | intronic | . | . | . | 2qG3 | . | 0 | 1 |
| chr2 | 148828071 | A | T | 129 | Cst13 | intronic | . | . | . | 2qG3 | rs27277117 | 0 | 1 |
| chr2 | 148828078 | G | T | 153 | Cst13 | intronic | . | . | . | 2qG3 | rs47801791 | 0 | 1 |
| chr2 | 148828276 | G | A | 179 | Cst13 | intronic | . | . | . | 2qG3 | rs27277116 | 0 | 1 |
| chr2 | 148828289 | T | C | 153 | Cst13 | intronic | . | . | . | 2qG3 | rs27277115 | 0 | 1 |
| chr2 | 148828330 | A | G | 79 | Cst13 | intronic | . | . | . | 2qG3 | rs27277113 | 0 | 1 |
| chr2 | 148830423 | A | G | 93 | . | downstream | . | . | . | 2qG3 | rs27277104 | 0 | 1 |
| chr2 | 148835366 | C | T | 221 | Cst9 | exonic | synonymous SNV | Cst9:NM_009979:exon1:c.C156T:p.F52F | . | 2qG3 | rs29774563 | 0 | 1 |
| chr2 | 148835491 | G | A | 222 | Cst9 | intronic | . | . | . | 2qG3 | rs33437021 | 0 | 1 |
| chr2 | 148836583 | G | A | 86 | Cst9 | intronic | . | . | . | 2qG3 | rs27277081 | 0 | 1 |
| chr2 | 148836610 | C | T | 127 | Cst9 | intronic | . | . | . | 2qG3 | rs27277080 | 0 | 1 |
| chr2 | 148836702 | G | A | 182 | Cst9 | exonic | missense SNV | Cst9:NM_009979:exon2:c.G251A:p.R84K | . | 2qG3 | rs33623376 | 0 | 1 |
| chr2 | 148836751 | T | C | 221 | Cst9 | exonic | synonymous SNV | Cst9:NM_009979:exon2:c.T300C:p.D100D | . | 2qG3 | rs27277078 | 0 | 1 |
| chr2 | 148836806 | C | T | 199 | Cst9 | intronic | . | . | . | 2qG3 | rs32963555 | 0 | 1 |
| chr2 | 148838296 | C | T | 66 | Cst9 | intronic | . | . | . | 2qG3 | rs50643035 | 0 | 1 |
| chr2 | 148838397 | C | T | 108 | Cst9 | intronic | . | . | . | 2qG3 | rs48406246 | 0 | 1 |
| chr2 | 148838445 | T | C | 182 | Cst9 | intronic | . | . | . | 2qG3 | rs52002924 | 0 | 1 |
| chr2 | 148838586 | G | A | 222 | Cst9 | UTR3 | . | . | . | 2qG3 | rs50726048 | 0 | 1 |
| chr2 | 148846770 | A | G | 102 | 9230104L09Rik | UTR3 | . | . | . | 2qG3 | . | 0 | 1 |
| chr2 | 148846806 | T | C | 153 | 9230104L09Rik | UTR3 | . | . | . | 2qG3 | rs32998132 | 0 | 1 |
| chr2 | 148846810 | C | G | 153 | 9230104L09Rik | UTR3 | . | . | . | 2qG3 | rs33412379 | 0 | 1 |
| chr2 | 148846845 | A | T | 207 | 9230104L09Rik | UTR3 | . | . | . | 2qG3 | rs27262040 | 0 | 1 |
| chr2 | 148847085 | A | C | 176 | 9230104L09Rik | intronic | . | . | . | 2qG3 | rs27262039 | 0 | 1 |
| chr2 | 148847163 | G | T | 106 | 9230104L09Rik | intronic | . | . | . | 2qG3 | rs27262038 | 0 | 1 |
| chr2 | 148847959 | A | G | 222 | 9230104L09Rik | exonic | synonymous SNV | 9230104L09Rik:NM_029960:exon2:c.T273C:p.F91F | . | 2qG3 | rs27262035 | 0 | 1 |
| chr2 | 148848062 | C | G | 222 | 9230104L09Rik | intronic | . | . | . | 2qG3 | rs51646102 | 0 | 1 |
| chr2 | 148850734 | G | A | 222 | 9230104L09Rik | exonic | synonymous SNV | 9230104L09Rik:NM_029960:exon1:c.C147T:p.H49H | . | 2qG3 | rs27262018 | 0 | 1 |
| chr2 | 148871760 | G | A | 122 | Cst3 | UTR3 | . | . | . | 2qG3 | rs29557246 | 0 | 1 |
| chr2 | 148871761 | C | T | 122 | Cst3 | UTR3 | . | . | . | 2qG3 | rs33128608 | 0 | 1 |
| chr2 | 148871932 | G | A | 222 | Cst3 | UTR3 | . | . | . | 2qG3 | rs8256617 | 0 | 1 |
| chr2 | 148872051 | A | C | 201 | Cst3 | intronic | . | . | . | 2qG3 | rs27261932 | 0 | 1 |
| chr2 | 148872707 | T | C | 56 | Cst3 | intronic | . | . | . | 2qG3 | rs32957054 | 0 | 1 |
| chr2 | 148872710 | T | C | 58 | Cst3 | intronic | . | . | . | 2qG3 | rs29730428 | 0 | 1 |
| chr2 | 148999102 | T | C | 104 | . | intergenic | . | . | . | 2qG3 | rs27246097 | 0 | 1 |
| chr2 | 148999451 | T | C | 222 | . | intergenic | . | . | . | 2qG3 | rs27246090 | 0 | 1 |
| chr2 | 149015307 | T | C | 222 | . | intergenic | . | . | . | 2qG3 | rs27246008 | 0 | 1 |
| chr2 | 149015419 | C | G | 222 | . | intergenic | . | . | . | 2qG3 | rs27246006 | 0 | 1 |
| chr2 | 149015661 | G | A | 222 | . | intergenic | . | . | . | 2qG3 | rs27246005 | 0 | 1 |
| chr2 | 149016283 | C | G | 221 | . | intergenic | . | . | . | 2qG3 | rs33089466 | 0 | 1 |
| chr2 | 149333195 | T | G | 196 | . | intergenic | . | . | . | 2qG3 | . | 0 | 1 |
| chr2 | 149333286 | G | T | 204 | . | intergenic | . | . | . | 2qG3 | rs33560884 | 0 | 1 |
| chr2 | 149333926 | C | T | 137 | . | intergenic | . | . | . | 2qG3 | rs27262171 | 0 | 1 |
| chr2 | 149356796 | G | A | 37.6322 | . | intergenic | . | . | . | 2qG3 | rs27247931 | 0 | 1 |
| chr2 | 149357020 | C | T | 222 | . | intergenic | . | . | . | 2qG3 | rs27247923 | 0 | 1 |
| chr2 | 149357130 | T | C | 221 | . | intergenic | . | . | . | 2qG3 | . | 0 | 1 |
| chr2 | 149743099 | C | A | 222 | . | intergenic | . | . | . | 2qG3 | . | 0 | 1 |
| chr2 | 150834189 | C | T | 218 | Abhd12 | intronic | . | . | . | 2qG3 | . | 0 | 1 |
| chr2 | 151375256 | C | A | 217 | . | intergenic | . | . | . | 2qG3 | rs46819296 | 0 | 1 |
| chr2 | 151657561 | G | A | 59 | Rad21l | intronic | . | . | . | 2qG3 | . | 0 | 1 |
| chr2 | 151939183 | C | A | 135 | Angpt4 | intronic | . | . | . | 2qG3 | . | 0 | 1 |
| chr2 | 152439695 | A | T | 142 | 6820408C15Rik | exonic | missense SNV | 6820408C15Rik:NM_001289738:exon7:c.A527T:p.E176V,6820408C15Rik:NM_177656:exon7:c.A524T:p.E175 | . | 2qG3 | . | 0 | 1 |
| chr2 | 152574583 | C | G | 100 | Defb21 | intronic | . | . | . | 2qH1 | . | 0 | 1 |
| chr2 | 152622985 | T | C | 104 | Defb25 | exonic | missense SNV | Defb25:NM_001039122:exon1:c.A43G:p.S15G | . | 2qH1 | rs46594747 | 0 | 1 |
| chr2 | 152623066 | G | A | 131 | . | upstream | . | . | . | 2qH1 | rs48360580 | 0 | 1 |
| chr2 | 152623127 | A | G | 189 | . | upstream | . | . | . | 2qH1 | rs51559534 | 0 | 1 |
| chr2 | 152628003 | A | G | 125 | Rem1 | UTR5 | . | . | . | 2qH1 | rs29570146 | 0 | 1 |
| chr2 | 152628023 | A | G | 131 | Rem1 | UTR5 | . | . | . | 2qH1 | rs33127763 | 0 | 1 |
| chr2 | 152628110 | C | G | 202 | Rem1 | exonic | synonymous SNV | Rem1:NM_009047:exon2:c.C15G:p.T5T | . | 2qH1 | rs46297387 | 0 | 1 |
| chr2 | 152628422 | C | T | 154 | Rem1 | exonic | synonymous SNV | Rem1:NM_009047:exon2:c.C327T:p.H109H | . | 2qH1 | rs49394533 | 0 | 1 |
| chr2 | 152628481 | A | T | 58 | Rem1 | intronic | . | . | . | 2qH1 | rs47955382 | 0 | 1 |
| chr2 | 152628483 | G | A | 48.2252 | Rem1 | intronic | . | . | . | 2qH1 | rs49645263 | 0 | 1 |
| chr2 | 152629052 | G | A | 68 | Rem1 | intronic | . | . | . | 2qH1 | rs46497327 | 0 | 1 |
| chr2 | 152629132 | A | G | 186 | Rem1 | intronic | . | . | . | 2qH1 | rs51573506 | 0 | 1 |
| chr2 | 152629199 | A | G | 221 | Rem1 | exonic | synonymous SNV | Rem1:NM_009047:exon3:c.A363G:p.S121S | . | 2qH1 | rs49471158 | 0 | 1 |
| chr2 | 152629365 | T | C | 98 | Rem1 | intronic | . | . | . | 2qH1 | rs50421563 | 0 | 1 |
| chr2 | 152634440 | T | C | 76 | Rem1 | intronic | . | . | . | 2qH1 | rs33186838 | 0 | 1 |
| chr2 | 152634450 | T | C | 90 | Rem1 | exonic | synonymous SNV | Rem1:NM_009047:exon5:c.T627C:p.G209G | . | 2qH1 | rs27387237 | 0 | 1 |
| chr2 | 152634630 | A | C | 151 | Rem1 | exonic | synonymous SNV | Rem1:NM_009047:exon5:c.A807C:p.R269R | 233 | 2qH1 | rs27387236 | 0 | 1 |
| chr2 | 152677660 | T | A | 208 | H13 | intronic | . | . | . | 2qH1 | rs48310726 | 0 | 1 |
| chr2 | 152680927 | T | C | 71 | H13 | intronic | . | . | . | 2qH1 | rs33270428 | 0 | 1 |
| chr2 | 152686220 | T | C | 222 | H13 | exonic | missense SNV | H13:NM_001159552:exon4:c.T488C:p.F163S | . | 2qH1 | rs258157820 | 0 | 1 |
| chr2 | 152686261 | C | G | 216 | H13 | exonic | missense SNV | H13:NM_001159552:exon4:c.C529G:p.R177G | . | 2qH1 | rs27372095 | 0 | 1 |
| chr2 | 152686302 | A | G | 87 | H13 | exonic | synonymous SNV | H13:NM_001159552:exon4:c.A570G:p.S190S | . | 2qH1 | rs27372094 | 0 | 1 |
| chr2 | 152687856 | C | T | 221 | Mcts2,H13 | UTR3 | . | . | . | 2qH1 | rs47434767 | 0 | 1 |
| chr2 | 152687953 | C | T | 88 | H13 | intronic | . | . | . | 2qH1 | rs50547635 | 0 | 1 |
| chr2 | 152688551 | A | T | 129 | H13 | intronic | . | . | . | 2qH1 | rs32912611 | 0 | 1 |
| chr2 | 152688645 | T | C | 222 | H13 | intronic | . | . | . | 2qH1 | rs27372077 | 0 | 1 |
| chr2 | 152745827 | C | T | 20.9152 | . | intergenic | . | . | . | 2qH1 | rs36245958 | 0 | 1 |
| chr2 | 152745885 | G | T | 136 | . | intergenic | . | . | . | 2qH1 | rs214234497 | 0 | 1 |
| chr2 | 152745888 | A | G | 138 | . | intergenic | . | . | . | 2qH1 | rs587341295 | 0 | 1 |
| chr2 | 152746009 | C | T | 222 | . | intergenic | . | . | . | 2qH1 | rs582644255 | 0 | 1 |
| chr2 | 152755072 | C | T | 113 | Cox4i2 | intronic | . | . | . | 2qH1 | rs27371960 | 0 | 1 |
| chr2 | 152755221 | C | T | 221 | Cox4i2 | exonic | missense SNV | Cox4i2:NM_053091:exon2:c.C67T:p.H23Y | . | 2qH1 | rs27371959 | 0 | 1 |
| chr2 | 152755288 | G | A | 119 | Cox4i2 | intronic | . | . | . | 2qH1 | rs29871609 | 0 | 1 |
| chr2 | 152755290 | G | A | 118 | Cox4i2 | intronic | . | . | . | 2qH1 | rs33536360 | 0 | 1 |
| chr2 | 152760762 | A | C | 157 | Cox4i2,Bcl2l1 | exonic | synonymous SNV | Cox4i2:NM_053091:exon4:c.A375C:p.R125R | . | 2qH1 | rs50060724 | 0 | 1 |
| chr2 | 152760779 | C | T | 123 | Cox4i2,Bcl2l1 | intronic | . | . | . | 2qH1 | rs27371941 | 0 | 1 |
| chr2 | 152760809 | C | T | 98 | Bcl2l1,Cox4i2 | intronic | . | . | . | 2qH1 | rs27371940 | 0 | 1 |
| chr2 | 152764715 | G | A | 117 | Cox4i2,Bcl2l1 | intronic | . | . | . | 2qH1 | rs27371931 | 0 | 1 |
| chr2 | 152764890 | C | A | 129 | Bcl2l1,Cox4i2 | UTR3 | . | . | . | 2qH1 | rs27371929 | 0 | 1 |
| chr2 | 152802517 | C | T | 74 | Bcl2l1 | intronic | . | . | . | 2qH1 | rs27349888 | 0 | 1 |
| chr2 | 152802773 | A | G | 222 | Bcl2l1 | intronic | . | . | . | 2qH1 | rs27349886 | 0 | 1 |
| chr2 | 152829096 | A | C | 121 | Bcl2l1 | intronic | . | . | . | 2qH1 | rs27349861 | 0 | 1 |
| chr2 | 152829674 | C | T | 222 | Bcl2l1 | exonic | missense SNV | Bcl2l1:NM_001289716:exon2:c.G317A:p.S106N,Bcl2l1:NM_001289717:exon2:c.G317A:p.S106N,Bcl2l1:NM | . | 2qH1 | rs27349860 | 0 | 1 |
| chr2 | 152867365 | G | A | 222 | Tpx2 | intronic | . | . | . | 2qH1 | rs27349793 | 0 | 1 |
| chr2 | 152869496 | G | T | 106 | Tpx2 | intronic | . | . | . | 2qH1 | . | 0 | 1 |
| chr2 | 152869509 | C | T | 124 | Tpx2 | intronic | . | . | . | 2qH1 | rs33751804 | 0 | 1 |
| chr2 | 152869630 | T | C | 219 | Tpx2 | intronic | . | . | . | 2qH1 | rs27349786 | 0 | 1 |
| chr2 | 152875496 | A | G | 202 | Tpx2 | intronic | . | . | . | 2qH1 | rs27349766 | 0 | 1 |
| chr2 | 152876366 | G | A | 40.8655 | Tpx2 | intronic | . | . | . | 2qH1 | rs27349761 | 0 | 1 |
| chr2 | 152876400 | C | T | 73 | Tpx2 | intronic | . | . | . | 2qH1 | rs27349760 | 0 | 1 |
| chr2 | 152876853 | A | G | 107 | Tpx2 | intronic | . | . | . | 2qH1 | rs27349758 | 0 | 1 |
| chr2 | 152880032 | T | C | 157 | Tpx2 | intronic | . | . | . | 2qH1 | rs27349741 | 0 | 1 |
| chr2 | 152880232 | A | G | 221 | Tpx2 | intronic | . | . | . | 2qH1 | rs6304937 | 0 | 1 |
| chr2 | 152880237 | C | T | 219 | Tpx2 | intronic | . | . | . | 2qH1 | rs6304940 | 0 | 1 |
| chr2 | 152880942 | A | G | 206 | Tpx2 | intronic | . | . | . | 2qH1 | rs27349736 | 0 | 1 |
| chr2 | 152880985 | T | C | 222 | Tpx2 | intronic | . | . | . | 2qH1 | rs27349735 | 0 | 1 |
| chr2 | 152881234 | T | C | 203 | Tpx2 | intronic | . | . | . | 2qH1 | rs27349732 | 0 | 1 |
| chr2 | 152882148 | C | T | 176 | Tpx2 | intronic | . | . | . | 2qH1 | rs27349726 | 0 | 1 |
| chr2 | 152882180 | T | G | 221 | Tpx2 | intronic | . | . | . | 2qH1 | rs27349725 | 0 | 1 |
| chr2 | 152882220 | C | T | 222 | Tpx2 | exonic | synonymous SNV | Tpx2:NM_001141975:exon10:c.C915T:p.I305I,Tpx2:NM_001141976:exon10:c.C915T:p.I305I,Tpx2:NM_001 | . | 2qH1 | rs27349724 | 0 | 1 |
| chr2 | 152882394 | C | G | 222 | Tpx2 | intronic | . | . | . | 2qH1 | rs27349723 | 0 | 1 |
| chr2 | 152882440 | C | T | 209 | Tpx2 | intronic | . | . | . | 2qH1 | rs27349722 | 0 | 1 |
| chr2 | 152884150 | A | G | 222 | Tpx2 | intronic | . | . | . | 2qH1 | rs27349710 | 0 | 1 |
| chr2 | 152885071 | C | T | 221 | Tpx2 | exonic | synonymous SNV | Tpx2:NM_001141975:exon12:c.C1257T:p.P419P,Tpx2:NM_001141976:exon12:c.C1257T:p.P419P,Tpx2:NM_( | . | 2qH1 | rs27349704 | 0 | 1 |
| chr2 | 152885171 | G | A | 217 | Tpx2 | exonic | missense SNV | Tpx2:NM_001141975:exon12:c.G1357A:p.E453K,Tpx2:NM_001141976:exon12:c.G1357A:p.E453K,Tpx2:NM_( | . | 2qH1 | rs50152624 | 0 | 1 |
| chr2 | 152885258 | A | G | 112 | Tpx2 | intronic | . | . | . | 2qH1 | rs27349703 | 0 | 1 |
| chr2 | 152885279 | A | G | 52 | Tpx2 | intronic | . | . | . | 2qH1 | rs27349702 | 0 | 1 |
| chr2 | 152888048 | G | A | 215 | Tpx2 | intronic | . | . | . | 2qH1 | rs27349684 | 0 | 1 |
| chr2 | 152888333 | G | A | 112 | Tpx2 | intronic | . | . | . | 2qH1 | rs27349682 | 0 | 1 |
| chr2 | 152889061 | G | A | 40.3145 | Tpx2 | intronic | . | . | . | 2qH1 | rs27349679 | 0 | 1 |
| chr2 | 152889092 | G | A | 48.2957 | Tpx2 | intronic | . | . | . | 2qH1 | rs27349678 | 0 | 1 |
| chr2 | 152889109 | G | A | 59 | Tpx2 | intronic | . | . | . | 2qH1 | rs27349677 | 0 | 1 |
| chr2 | 152889164 | G | T | 130 | Tpx2 | exonic | missense SNV | Tpx2:NM_001141975:exon14:c.G1525T:p.V509L,Tpx2:NM_001141976:exon14:c.G1525T:p.V509L,Tpx2:NM_( | . | 2qH1 | rs27349676 | 0 | 1 |
| chr2 | 152889247 | G | A | 221 | Tpx2 | exonic | synonymous SNV | Tpx2:NM_001141975:exon14:c.G1608A:p.P536P,Tpx2:NM_001141976:exon14:c.G1608A:p.P536P,Tpx2:NM_( | . | 2qH1 | rs27349675 | 0 | 1 |
| chr2 | 152890371 | G | A | 42.1232 | Tpx2 | intronic | . | . | . | 2qH1 | rs27349670 | 0 | 1 |
| chr2 | 152890707 | A | T | 222 | Tpx2 | intronic | . | . | . | 2qH1 | rs27349669 | 0 | 1 |
| chr2 | 152891294 | C | T | 42.5918 | Tpx2 | intronic | . | . | . | 2qH1 | rs27402857 | 0 | 1 |
| chr2 | 152891686 | G | A | 35.4799 | Tpx2 | intronic | . | . | . | 2qH1 | rs27402854 | 0 | 1 |
| chr2 | 152892051 | G | A | 132 | Tpx2 | intronic | . | . | . | 2qH1 | rs27402849 | 0 | 1 |
| chr2 | 152892359 | C | T | 141 | Tpx2 | intronic | . | . | . | 2qH1 | rs27402847 | 0 | 1 |
| chr2 | 152893508 | C | T | 175 | Tpx2 | intronic | . | . | . | 2qH1 | rs49042238 | 0 | 1 |
| chr2 | 152893634 | T | C | 222 | Tpx2 | exonic | synonymous SNV | Tpx2:NM_001141975:exon18:c.T2181C:p.S727S,Tpx2:NM_001141976:exon18:c.T2181C:p.S727S,Tpx2:NM_( | . | 2qH1 | rs27402838 | 0 | 1 |
| chr2 | 152893682 | G | A | 221 | Tpx2 | exonic | synonymous SNV | Tpx2:NM_001141975:exon18:c.G2229A:p.R743R,Tpx2:NM_001141976:exon18:c.G2229A:p.R743R,Tpx2:NM_( | . | 2qH1 | rs27402836 | 0 | 1 |
| chr2 | 152893732 | G | T | 84 | Tpx2 | UTR3 | . | . | . | 2qH1 | rs27402835 | 0 | 1 |
| chr2 | 152911438 | A | T | 79 | Mylk2 | UTR5 | . | . | . | 2qH1 | rs249659613 | 0 | 1 |
| chr2 | 152911607 | G | A | 222 | Mylk2 | intronic | . | . | . | 2qH1 | rs47677079 | 0 | 1 |
| chr2 | 152911671 | G | A | 222 | Mylk2 | intronic | . | . | . | 2qH1 | rs51418684 | 0 | 1 |
| chr2 | 152912219 | C | G | 129 | Mylk2 | exonic | missense SNV | Mylk2:NM_001081044:exon2:c.C232G:p.P78A | . | 2qH1 | rs51613845 | 0 | 1 |
| chr2 | 152913494 | G | A | 222 | Mylk2 | exonic | missense SNV | Mylk2:NM_001081044:exon3:c.G577A:p.V193I | . | 2qH1 | rs48492936 | 0 | 1 |
| chr2 | 152913627 | T | C | 222 | Mylk2 | exonic | missense SNV | Mylk2:NM_001081044:exon3:c.T710C:p.V237A | . | 2qH1 | rs27387736 | 0 | 1 |
| chr2 | 152913798 | C | T | 150 | Mylk2 | intronic | . | . | . | 2qH1 | rs48154085 | 0 | 1 |
| chr2 | 152915068 | G | A | 164 | Mylk2 | intronic | . | . | . | 2qH1 | rs29544826 | 0 | 1 |
| chr2 | 152915602 | G | C | 163 | Mylk2 | intronic | . | . | . | 2qH1 | rs260099538 | 0 | 1 |
| chr2 | 152915611 | G | C | 178 | Mylk2 | intronic | . | . | . | 2qH1 | rs49776946 | 0 | 1 |
| chr2 | 152917184 | T | A | 74 | Mylk2 | intronic | . | . | . | 2qH1 | rs27387720 | 0 | 1 |
| chr2 | 152917233 | A | G | 76 | Mylk2 | intronic | . | . | . | 2qH1 | rs224318039 | 0 | 1 |
| chr2 | 152917267 | G | T | 117 | Mylk2 | intronic | . | . | . | 2qH1 | rs47154946 | 0 | 1 |
| chr2 | 152917690 | T | C | 214 | Mylk2 | intronic | . | . | . | 2qH1 | rs27387719 | 0 | 1 |
| chr2 | 152917760 | C | T | 60 | Mylk2 | intronic | . | . | . | 2qH1 | rs27387718 | 0 | 1 |
| chr2 | 152919277 | A | T | 32.7551 | Mylk2 | intronic | . | . | . | 2qH1 | rs214318125 | 0 | 1 |
| chr2 | 152919281 | T | C | 69 | Mylk2 | intronic | . | . | . | 2qH1 | rs238737098 | 0 | 1 |
| chr2 | 152919311 | T | C | 164 | Mylk2 | intronic | . | . | . | 2qH1 | rs27387707 | 0 | 1 |
| chr2 | 152919475 | G | A | 195 | Mylk2 | intronic | . | . | . | 2qH1 | rs32819805 | 0 | 1 |
| chr2 | 152919508 | G | T | 73 | Mylk2 | intronic | . | . | . | 2qH1 | rs51321933 | 0 | 1 |
| chr2 | 152919518 | T | C | 42.9116 | Mylk2 | intronic | . | . | . | 2qH1 | rs27387706 | 0 | 1 |
| chr2 | 152920231 | G | T | 88 | Mylk2 | intronic | . | . | . | 2qH1 | rs27387704 | 0 | 1 |
| chr2 | 152920485 | A | G | 221 | Mylk2 | intronic | . | . | . | 2qH1 | rs33590815 | 0 | 1 |
| chr2 | 152920507 | T | C | 222 | Mylk2 | intronic | . | . | . | 2qH1 | rs32880988 | 0 | 1 |
| chr2 | 152922006 | A | G | 145 | Mylk2 | intronic | . | . | . | 2qH1 | rs48762944 | 0 | 1 |
| chr2 | 152932138 | C | T | 212 | Foxs1 | UTR3 | . | . | . | 2qH1 | rs27387668 | 0 | 1 |
| chr2 | 152932499 | C | T | 202 | Foxs1 | exonic | synonymous SNV | Foxs1:NM_010226:exon1:c.G633A:p.S211S | . | 2qH1 | rs27387667 | 0 | 1 |
| chr2 | 152932746 | A | G | 201 | Foxs1 | exonic | missense SNV | Foxs1:NM_010226:exon1:c.T386C:p.I129T | . | 2qH1 | rs243130446 | 0 | 1 |
| chr2 | 152932772 | C | A | 198 | Foxs1 | exonic | synonymous SNV | Foxs1:NM_010226:exon1:c.G360T:p.A120A | 257 | 2qH1 | rs260912917 | 0 | 1 |

| | | | | | | | | | | | | | |
|---|---|---|---|---|---|---|---|---|---|---|---|---|---|
| chr2 | 152932814 | G | A | 222 | Foxs1 | exonic | synonymous SNV | Foxs1:NM_010226:exon1:c.C318T:p.S106S | 257 | 2qH1 | rs27387666 | 0 | 1 |
| chr2 | 152933125 | T | G | 221 | Foxs1 | exonic | missense SNV | Foxs1:NM_010226:exon1:c.A7C:p.K3Q | . | 2qH1 | rs27387664 | 0 | 1 |
| chr2 | 152944361 | A | G | 222 | Dusp15 | intronic | . | . | . | 2qH1 | rs27387643 | 0 | 1 |
| chr2 | 152944861 | C | T | 53 | Dusp15 | intronic | . | . | . | 2qH1 | rs29623168 | 0 | 1 |
| chr2 | 152944948 | C | T | 70 | Dusp15 | UTR3 | . | . | . | 2qH1 | rs33652642 | 0 | 1 |
| chr2 | 152945109 | G | A | 221 | Dusp15 | exonic | synonymous SNV | Dusp15:NM_145744:exon6:c.C312T:p.C104C | . | 2qH1 | rs27387641 | 0 | 1 |
| chr2 | 152945479 | C | T | 222 | Dusp15 | exonic | missense SNV | Dusp15:NM_001159376:exon5:c.G211A:p.V71I,Dusp15:NM_145744:exon5:c.G211A:p.V71I | . | 2qH1 | rs27387640 | 0 | 1 |
| chr2 | 152945607 | T | C | 118 | Dusp15 | intronic | . | . | . | 2qH1 | rs33318323 | 0 | 1 |
| chr2 | 152946493 | A | G | 106 | Dusp15 | intronic | . | . | . | 2qH1 | rs27387638 | 0 | 1 |
| chr2 | 152948911 | G | A | 120 | Dusp15 | intronic | . | . | . | 2qH1 | rs27387636 | 0 | 1 |
| chr2 | 152951402 | A | C | 132 | Dusp15 | UTR5 | . | . | 540 | 2qH1 | rs239275963 | 0 | 1 |
| chr2 | 152951405 | G | A | 142 | Dusp15 | UTR5 | . | . | 540 | 2qH1 | rs265632531 | 0 | 1 |
| chr2 | 152962498 | T | A | 222 | Ttll9 | UTR5 | . | . | . | 2qH1 | rs27387610 | 0 | 1 |
| chr2 | 152962733 | C | T | 49.5961 | Ttll9 | intronic | . | . | . | 2qH1 | rs27387609 | 0 | 1 |
| chr2 | 152971744 | G | A | 32.3896 | Ttll9 | intronic | . | . | . | 2qH1 | rs27387597 | 0 | 1 |
| chr2 | 152971755 | G | A | 20.3138 | Ttll9 | intronic | . | . | . | 2qH1 | rs27387596 | 0 | 1 |
| chr2 | 152972159 | G | A | 100 | Ttll9 | intronic | . | . | . | 2qH1 | rs47706316 | 0 | 1 |
| chr2 | 152972280 | C | T | 175 | Ttll9 | intronic | . | . | . | 2qH1 | rs27387592 | 0 | 1 |
| chr2 | 152972303 | G | A | 169 | Ttll9 | exonic | missense SNV | Ttll9:NM_001083618:exon2:c.G31A:p.G11S,Ttll9:NM_029064:exon2:c.G31A:p.G11S | . | 2qH1 | rs27387591 | 0 | 1 |
| chr2 | 152976088 | A | T | 39.0658 | Ttll9 | intronic | . | . | . | 2qH1 | rs47250934 | 0 | 1 |
| chr2 | 152976092 | A | G | 93 | Ttll9 | intronic | . | . | . | 2qH1 | rs47922131 | 0 | 1 |
| chr2 | 152976184 | C | A | 216 | Ttll9 | exonic | synonymous SNV | Ttll9:NM_001083618:exon3:c.C93A:p.T31T,Ttll9:NM_029064:exon3:c.C93A:p.T31T | . | 2qH1 | rs27387578 | 0 | 1 |
| chr2 | 152982966 | A | G | 57 | Ttll9 | intronic | . | . | . | 2qH1 | rs48266816 | 0 | 1 |
| chr2 | 152982983 | A | G | 68 | Ttll9 | intronic | . | . | . | 2qH1 | rs48798553 | 0 | 1 |
| chr2 | 152983006 | G | C | 89 | Ttll9 | intronic | . | . | . | 2qH1 | rs51431564 | 0 | 1 |
| chr2 | 152983133 | C | T | 101 | Ttll9 | exonic | synonymous SNV | Ttll9:NM_001083618:exon4:c.C231T:p.H77H,Ttll9:NM_029064:exon4:c.C231T:p.H77H | . | 2qH1 | rs48347135 | 0 | 1 |
| chr2 | 152983142 | A | C | 97 | Ttll9 | exonic | synonymous SNV | Ttll9:NM_001083618:exon4:c.A240C:p.I80I,Ttll9:NM_029064:exon4:c.A240C:p.I80I | . | 2qH1 | rs52033679 | 0 | 1 |
| chr2 | 152984049 | A | G | 88 | Ttll9 | intronic | . | . | . | 2qH1 | rs49534916 | 0 | 1 |
| chr2 | 152984386 | G | A | 221 | Ttll9 | intronic | . | . | . | 2qH1 | rs27387553 | 0 | 1 |
| chr2 | 152984403 | G | C | 160 | Ttll9 | intronic | . | . | . | 2qH1 | rs47706355 | 0 | 1 |
| chr2 | 152989936 | T | C | 38.5684 | Ttll9 | intronic | . | . | . | 2qH1 | rs49793176 | 0 | 1 |
| chr2 | 152989984 | A | G | 128 | Ttll9 | intronic | . | . | . | 2qH1 | rs27387529 | 0 | 1 |
| chr2 | 152990071 | A | T | 137 | Ttll9 | exonic | synonymous SNV | Ttll9:NM_001083618:exon7:c.A567T:p.R189R,Ttll9:NM_029064:exon7:c.A567T:p.R189R | . | 2qH1 | rs27387528 | 0 | 1 |
| chr2 | 152990078 | A | G | 140 | Ttll9 | exonic | missense SNV | Ttll9:NM_001083618:exon7:c.A574G:p.M192V,Ttll9:NM_029064:exon7:c.A574G:p.M192V | . | 2qH1 | rs27387527 | 0 | 1 |
| chr2 | 152990146 | C | T | 44.549 | Ttll9 | intronic | . | . | . | 2qH1 | rs259882940 | 0 | 1 |
| chr2 | 152991775 | G | A | 160 | Ttll9 | intronic | . | . | . | 2qH1 | rs50126537 | 0 | 1 |
| chr2 | 152991780 | C | A | 127 | Ttll9 | intronic | . | . | . | 2qH1 | rs48524021 | 0 | 1 |
| chr2 | 152993246 | G | A | 221 | Ttll9 | intronic | . | . | . | 2qH1 | rs229377657 | 0 | 1 |
| chr2 | 152993263 | C | T | 221 | Ttll9 | intronic | . | . | . | 2qH1 | rs47730352 | 0 | 1 |
| chr2 | 152993317 | C | T | 128 | Ttll9 | intronic | . | . | . | 2qH1 | rs27387504 | 0 | 1 |
| chr2 | 152994031 | C | T | 222 | Ttll9 | intronic | . | . | . | 2qH1 | rs27387503 | 0 | 1 |
| chr2 | 152994210 | A | C | 27.4009 | Ttll9 | intronic | . | . | . | 2qH1 | rs243396251 | 0 | 1 |
| chr2 | 153002735 | A | G | 126 | Ttll9 | intronic | . | . | . | 2qH1 | rs32868712 | 0 | 1 |
| chr2 | 153007088 | G | A | 83 | Ttll9 | UTR3 | . | . | . | 2qH1 | rs32854258 | 0 | 1 |
| chr2 | 153010226 | C | T | 29.7402 | Pdrg1 | intronic | . | . | . | 2qH1 | rs46399486 | 0 | 1 |
| chr2 | 153010255 | C | T | 114 | Pdrg1 | splicing | . | . | . | 2qH1 | rs262217185 | 0 | 1 |
| chr2 | 153010399 | A | G | 222 | Pdrg1 | intronic | . | . | . | 2qH1 | rs27373958 | 0 | 1 |
| chr2 | 153010495 | A | G | 111 | Pdrg1 | intronic | . | . | . | 2qH1 | rs32879557 | 0 | 1 |
| chr2 | 153010512 | G | A | 114 | Pdrg1 | intronic | . | . | . | 2qH1 | rs27373957 | 0 | 1 |
| chr2 | 153012162 | C | T | 70 | Pdrg1 | intronic | . | . | . | 2qH1 | rs27373951 | 0 | 1 |
| chr2 | 153014168 | G | A | 114 | Pdrg1 | intronic | . | . | . | 2qH1 | rs27373945 | 0 | 1 |
| chr2 | 153054451 | T | C | 222 | Xkr7 | exonic | synonymous SNV | Xkr7:NM_001011732:exon3:c.T1224C:p.F408F | . | 2qH1 | rs27373818 | 0 | 1 |
| chr2 | 153054502 | T | C | 221 | Xkr7 | exonic | synonymous SNV | Xkr7:NM_001011732:exon3:c.T1275C:p.F425F | . | 2qH1 | rs27373817 | 0 | 1 |
| chr2 | 153055149 | G | A | 53 | Xkr7 | UTR3 | . | . | . | 2qH1 | rs27373816 | 0 | 1 |
| chr2 | 153065866 | C | T | 106 | . | upstream | . | . | . | 2qH1 | rs49370345 | 0 | 1 |
| chr2 | 153065989 | A | C | 222 | Ccm2l | UTR5 | . | . | . | 2qH1 | rs236573203 | 0 | 1 |
| chr2 | 153066169 | T | C | 186 | Ccm2l | intronic | . | . | . | 2qH1 | rs33257321 | 0 | 1 |
| chr2 | 153067692 | G | T | 78 | Ccm2l | intronic | . | . | . | 2qH1 | rs51199728 | 0 | 1 |
| chr2 | 153067961 | A | G | 221 | Ccm2l | intronic | . | . | . | 2qH1 | rs50066316 | 0 | 1 |
| chr2 | 153068018 | G | A | 147 | Ccm2l | intronic | . | . | . | 2qH1 | rs51241002 | 0 | 1 |
| chr2 | 153070609 | T | A | 148 | Ccm2l | intronic | . | . | . | 2qH1 | rs49267201 | 0 | 1 |
| chr2 | 153070674 | C | T | 168 | Ccm2l | exonic | synonymous SNV | Ccm2l:NM_145536:exon3:c.C232T:p.L78L | . | 2qH1 | rs49278954 | 0 | 1 |
| chr2 | 153070718 | T | C | 71 | Ccm2l | exonic | synonymous SNV | Ccm2l:NM_145536:exon3:c.T276C:p.T92T | . | 2qH1 | rs51491284 | 0 | 1 |
| chr2 | 153070790 | T | C | 98 | Ccm2l | intronic | . | . | . | 2qH1 | rs51649414 | 0 | 1 |
| chr2 | 153070851 | G | A | 116 | Ccm2l | exonic | synonymous SNV | Ccm2l:NM_145536:exon4:c.G312A:p.T104T | . | 2qH1 | rs51949793 | 0 | 1 |
| chr2 | 153070914 | T | C | 175 | Ccm2l | exonic | synonymous SNV | Ccm2l:NM_145536:exon4:c.T375C:p.N125N | . | 2qH1 | rs49715369 | 0 | 1 |
| chr2 | 153078512 | C | T | 165 | Ccm2l | exonic | missense SNV | Ccm2l:NM_145536:exon7:c.C1088T:p.A363V | . | 2qH1 | rs48639763 | 0 | 1 |
| chr2 | 153078618 | A | G | 218 | Ccm2l | intronic | . | . | . | 2qH1 | rs51818007 | 0 | 1 |
| chr2 | 153078627 | C | T | 205 | Ccm2l | intronic | . | . | . | 2qH1 | rs50509099 | 0 | 1 |
| chr2 | 153079275 | A | G | 33.1867 | Ccm2l | intronic | . | . | . | 2qH1 | rs48984267 | 0 | 1 |
| chr2 | 153079455 | T | C | 143 | Ccm2l | exonic | missense SNV | Ccm2l:NM_145536:exon8:c.T1250C:p.V417A | . | 2qH1 | rs46005822 | 0 | 1 |
| chr2 | 153080810 | T | C | 63 | Ccm2l | intronic | . | . | . | 2qH1 | rs50710315 | 0 | 1 |
| chr2 | 153108665 | A | G | 115 | Hck | UTR5 | . | . | 485 | 2qH1 | rs29769080 | 0 | 1 |
| chr2 | 153108801 | G | T | 48.7322 | Hck | intronic | . | . | 485 | 2qH1 | rs33392280 | 0 | 1 |
| chr2 | 153124098 | C | G | 133 | Hck | intronic | . | . | . | 2qH1 | rs49163539 | 0 | 1 |
| chr2 | 153124272 | G | T | 202 | Hck | exonic | synonymous SNV | Hck:NM_001172117:exon2:c.G102T:p.T34T,Hck:NM_010407:exon2:c.G165T:p.T55T | . | 2qH1 | rs27352882 | 0 | 1 |
| chr2 | 153128118 | A | G | 96 | Hck | intronic | . | . | . | 2qH1 | rs46784472 | 0 | 1 |
| chr2 | 153128119 | C | T | 87 | Hck | intronic | . | . | . | 2qH1 | rs46676588 | 0 | 1 |
| chr2 | 153128216 | A | C | 221 | Hck | intronic | . | . | . | 2qH1 | rs27352866 | 0 | 1 |
| chr2 | 153128980 | T | C | 144 | Hck | intronic | . | . | . | 2qH1 | rs49352620 | 0 | 1 |
| chr2 | 153129033 | G | T | 219 | Hck | intronic | . | . | . | 2qH1 | rs27352863 | 0 | 1 |
| chr2 | 153129288 | C | T | 86 | Hck | intronic | . | . | . | 2qH1 | rs27352862 | 0 | 1 |
| chr2 | 153129722 | A | G | 78 | Hck | intronic | . | . | . | 2qH1 | rs6186603 | 0 | 1 |
| chr2 | 153129798 | A | G | 84 | Hck | intronic | . | . | . | 2qH1 | rs6187150 | 0 | 1 |
| chr2 | 153129813 | A | G | 101 | Hck | intronic | . | . | . | 2qH1 | rs6187174 | 0 | 1 |
| chr2 | 153129866 | C | A | 129 | Hck | exonic | synonymous SNV | Hck:NM_001172117:exon5:c.C283A:p.R95R,Hck:NM_010407:exon5:c.C346A:p.R116R | . | 2qH1 | rs6187679 | 0 | 1 |
| chr2 | 153129937 | A | G | 118 | Hck | exonic | synonymous SNV | Hck:NM_001172117:exon5:c.A354G:p.T118T,Hck:NM_010407:exon5:c.A417G:p.T139T | . | 2qH1 | rs6187791 | 0 | 1 |
| chr2 | 153129940 | A | G | 122 | Hck | exonic | synonymous SNV | Hck:NM_001172117:exon5:c.A357G:p.E119E,Hck:NM_010407:exon5:c.A420G:p.E140E | . | 2qH1 | rs6187792 | 0 | 1 |
| chr2 | 153131193 | C | T | 166 | Hck | intronic | . | . | . | 2qH1 | rs27352856 | 0 | 1 |
| chr2 | 153131372 | A | G | 86 | Hck | intronic | . | . | . | 2qH1 | rs27352855 | 0 | 1 |
| chr2 | 153131374 | C | T | 75 | Hck | intronic | . | . | . | 2qH1 | rs254212925 | 0 | 1 |
| chr2 | 153131376 | T | C | 86 | Hck | intronic | . | . | . | 2qH1 | rs218864715 | 0 | 1 |
| chr2 | 153134024 | G | T | 176 | Hck | intronic | . | . | . | 2qH1 | rs27352839 | 0 | 1 |
| chr2 | 153134173 | A | C | 204 | Hck | exonic | synonymous SNV | Hck:NM_001172117:exon7:c.A568C:p.R190R,Hck:NM_010407:exon7:c.A631C:p.R211R | . | 2qH1 | rs27352838 | 0 | 1 |
| chr2 | 153134305 | A | G | 157 | Hck | intronic | . | . | . | 2qH1 | rs27352837 | 0 | 1 |
| chr2 | 153134331 | C | T | 146 | Hck | intronic | . | . | . | 2qH1 | rs27352836 | 0 | 1 |
| chr2 | 153134551 | G | A | 82 | Hck | intronic | . | . | . | 2qH1 | rs27352834 | 0 | 1 |
| chr2 | 153134801 | T | C | 222 | Hck | intronic | . | . | . | 2qH1 | rs52051785 | 0 | 1 |
| chr2 | 153136756 | C | T | 222 | Hck | exonic | synonymous SNV | Hck:NM_001172117:exon9:c.C825T:p.S275S,Hck:NM_010407:exon9:c.C888T:p.S296S | . | 2qH1 | rs47854483 | 0 | 1 |
| chr2 | 153136861 | G | A | 161 | Hck | exonic | synonymous SNV | Hck:NM_001172117:exon9:c.G930A:p.T310T,Hck:NM_010407:exon9:c.G993A:p.T331T | . | 2qH1 | rs51622016 | 0 | 1 |
| chr2 | 153144288 | G | A | 110 | Hck | intronic | . | . | . | 2qH1 | rs6329408 | 0 | 1 |
| chr2 | 153144378 | C | T | 197 | Hck | intronic | . | . | . | 2qH1 | rs6232131 | 0 | 1 |
| chr2 | 153144427 | A | G | 222 | Hck | exonic | synonymous SNV | Hck:NM_001172117:exon11:c.A1032G:p.E344E,Hck:NM_010407:exon11:c.A1095G:p.E365E | . | 2qH1 | rs6232208 | 0 | 1 |
| chr2 | 153144538 | A | G | 222 | Hck | exonic | synonymous SNV | Hck:NM_001172117:exon11:c.A1143G:p.R381R,Hck:NM_010407:exon11:c.A1206G:p.R402R | . | 2qH1 | rs6232753 | 0 | 1 |
| chr2 | 153149158 | T | G | 161 | Hck | intronic | . | . | . | 2qH1 | rs234076956 | 0 | 1 |
| chr2 | 153151062 | C | G | 222 | Hck | exonic | synonymous SNV | Hck:NM_001172117:exon13:c.C1425G:p.P475P,Hck:NM_010407:exon13:c.C1488G:p.P496P | . | 2qH1 | rs108378420 | 0 | 1 |
| chr2 | 153151151 | A | G | 209 | Hck | UTR3 | . | . | . | 2qH1 | rs33257672 | 0 | 1 |
| chr2 | 153151156 | T | G | 217 | Hck | UTR3 | . | . | . | 2qH1 | rs27352779 | 0 | 1 |
| chr2 | 153179044 | C | G | 212 | Tm9sf4 | intronic | . | . | . | 2qH1 | rs27352682 | 0 | 1 |
| chr2 | 153179078 | C | T | 190 | Tm9sf4 | intronic | . | . | . | 2qH1 | rs27352681 | 0 | 1 |
| chr2 | 153182377 | C | T | 133 | Tm9sf4 | intronic | . | . | . | 2qH1 | rs29762974 | 0 | 1 |
| chr2 | 153182379 | G | A | 135 | Tm9sf4 | intronic | . | . | . | 2qH1 | rs29951585 | 0 | 1 |
| chr2 | 153182556 | G | A | 153 | Tm9sf4 | intronic | . | . | . | 2qH1 | rs27352675 | 0 | 1 |
| chr2 | 153187150 | A | C | 111 | Tm9sf4 | intronic | . | . | . | 2qH1 | rs28300432 | 0 | 1 |
| chr2 | 153187712 | T | C | 222 | Tm9sf4 | intronic | . | . | . | 2qH1 | rs28300431 | 0 | 1 |
| chr2 | 153188817 | G | A | 56 | Tm9sf4 | intronic | . | . | . | 2qH1 | rs28300424 | 0 | 1 |
| chr2 | 153190962 | A | G | 222 | Tm9sf4 | intronic | . | . | . | 2qH1 | rs28300419 | 0 | 1 |
| chr2 | 153191708 | A | G | 43.9762 | Tm9sf4 | intronic | . | . | . | 2qH1 | rs6371121 | 0 | 1 |
| chr2 | 153191897 | A | G | 222 | Tm9sf4 | intronic | . | . | . | 2qH1 | rs33851902 | 0 | 1 |
| chr2 | 153194345 | C | T | 222 | Tm9sf4 | exonic | synonymous SNV | Tm9sf4:NM_133847:exon10:c.C1026T:p.P342P | . | 2qH1 | rs28300416 | 0 | 1 |
| chr2 | 153194540 | T | C | 192 | Tm9sf4 | intronic | . | . | . | 2qH1 | rs28300415 | 0 | 1 |
| chr2 | 153196771 | C | T | 199 | Tm9sf4 | intronic | . | . | . | 2qH1 | rs46998374 | 0 | 1 |
| chr2 | 153196795 | G | A | 198 | Tm9sf4 | intronic | . | . | . | 2qH1 | rs49161474 | 0 | 1 |
| chr2 | 153196916 | G | A | 185 | Tm9sf4 | intronic | . | . | . | 2qH1 | rs28300405 | 0 | 1 |
| chr2 | 153196939 | T | C | 221 | Tm9sf4 | intronic | . | . | . | 2qH1 | rs28300404 | 0 | 1 |
| chr2 | 153197048 | T | C | 205 | Tm9sf4 | intronic | . | . | . | 2qH1 | rs48075652 | 0 | 1 |
| chr2 | 153198574 | T | G | 45.8132 | Tm9sf4 | intronic | . | . | . | 2qH1 | rs28300395 | 0 | 1 |
| chr2 | 153200552 | G | A | 60 | Tm9sf4 | intronic | . | . | . | 2qH1 | rs28300390 | 0 | 1 |
| chr2 | 153202294 | G | A | 177 | Tm9sf4 | intronic | . | . | . | 2qH1 | rs28300388 | 0 | 1 |
| chr2 | 153203724 | G | A | 40.7595 | Tm9sf4 | intronic | . | . | . | 2qH1 | rs29872550 | 0 | 1 |
| chr2 | 153203880 | G | A | 37.9865 | Tm9sf4 | exonic | synonymous SNV | Tm9sf4:NM_133847:exon16:c.G1671A:p.V557V | . | 2qH1 | rs13476847 | 0 | 1 |
| chr2 | 153204494 | G | C | 217 | Tm9sf4 | intronic | . | . | . | 2qH1 | rs28300383 | 0 | 1 |
| chr2 | 153204661 | C | A | 221 | Tm9sf4 | intronic | . | . | . | 2qH1 | rs45984026 | 0 | 1 |
| chr2 | 153204666 | T | G | 221 | Tm9sf4 | intronic | . | . | . | 2qH1 | rs50257501 | 0 | 1 |
| chr2 | 153204749 | C | A | 75 | Tm9sf4 | intronic | . | . | . | 2qH1 | rs28300382 | 0 | 1 |
| chr2 | 153208532 | C | T | 37.4759 | Tm9sf4 | intronic | . | . | . | 2qH1 | rs28283265 | 0 | 1 |
| chr2 | 153208645 | T | C | 153 | Tm9sf4 | exonic | synonymous SNV | Tm9sf4:NM_133847:exon18:c.T1821C:p.F607F | . | 2qH1 | rs28283264 | 0 | 1 |
| chr2 | 153208690 | G | A | 183 | Tm9sf4 | exonic | synonymous SNV | Tm9sf4:NM_133847:exon18:c.G1866A:p.G622G | . | 2qH1 | rs13476840 | 0 | 1 |
| chr2 | 153224232 | T | C | 222 | Tspyl3 | UTR3 | . | . | . | 2qH1 | rs29525050 | 0 | 1 |
| chr2 | 153224567 | G | T | 222 | Tspyl3 | exonic | synonymous SNV | Tspyl3:NM_198617:exon1:c.C750A:p.R250R | . | 2qH1 | rs28283237 | 0 | 1 |
| chr2 | 153225039 | A | T | 158 | Tspyl3 | exonic | missense SNV | Tspyl3:NM_198617:exon1:c.T278A:p.V93E | . | 2qH1 | rs33313544 | 0 | 1 |
| chr2 | 153225151 | C | T | 119 | Tspyl3 | exonic | missense SNV | Tspyl3:NM_198617:exon1:c.G166A:p.G56R | . | 2qH1 | rs28283235 | 0 | 1 |
| chr2 | 153231342 | C | G | 59 | Plagl2 | UTR3 | . | . | . | 2qH1 | rs28283216 | 0 | 1 |
| chr2 | 153231475 | G | A | 131 | Plagl2 | UTR3 | . | . | . | 2qH1 | rs33751626 | 0 | 1 |
| chr2 | 153232480 | C | T | 222 | Plagl2 | exonic | missense SNV | Plagl2:NM_018807:exon3:c.G500A:p.S167N | . | 2qH1 | rs28283215 | 0 | 1 |
| chr2 | 153235657 | T | C | 108 | Plagl2 | intronic | . | . | . | 2qH1 | rs28283204 | 0 | 1 |
| chr2 | 153235735 | A | G | 222 | Plagl2 | intronic | . | . | . | 2qH1 | rs28283203 | 0 | 1 |
| chr2 | 153243526 | T | C | 25.6681 | Pofut1 | intronic | . | . | . | 2qH1 | rs50953281 | 0 | 1 |
| chr2 | 153248691 | A | G | 208 | Pofut1 | intronic | . | . | . | 2qH1 | rs28283195 | 0 | 1 |
| chr2 | 153249807 | A | C | 222 | Pofut1 | intronic | . | . | . | 2qH1 | rs28283192 | 0 | 1 |
| chr2 | 153259188 | G | A | 85 | Pofut1 | intronic | . | . | . | 2qH1 | rs28283170 | 0 | 1 |
| chr2 | 153259225 | G | T | 124 | Pofut1 | intronic | . | . | . | 2qH1 | rs28283169 | 0 | 1 |
| chr2 | 153259454 | C | T | 222 | Pofut1 | intronic | . | . | . | 2qH1 | rs28283168 | 0 | 1 |
| chr2 | 153261227 | A | G | 212 | Pofut1 | exonic | synonymous SNV | Pofut1:NM_080463:exon6:c.A780G:p.G260G | . | 2qH1 | rs28283164 | 0 | 1 |
| chr2 | 153261451 | C | T | 222 | Pofut1 | intronic | . | . | . | 2qH1 | rs28283162 | 0 | 1 |
| chr2 | 153316269 | T | C | 222 | Kif3b | UTR5 | . | . | . | 2qH1 | rs28283064 | 0 | 1 |
| chr2 | 153316790 | A | G | 221 | Kif3b | exonic | synonymous SNV | Kif3b:NM_008444:exon2:c.A510G:p.P170P | . | 2qH1 | rs28283062 | 0 | 1 |
| chr2 | 153316952 | A | G | 222 | Kif3b | exonic | synonymous SNV | Kif3b:NM_008444:exon2:c.A672G:p.E224E | . | 2qH1 | rs28283061 | 0 | 1 |
| chr2 | 153317006 | T | C | 222 | Kif3b | exonic | synonymous SNV | Kif3b:NM_008444:exon2:c.T726C:p.N242N | . | 2qH1 | rs28283060 | 0 | 1 |
| chr2 | 153320553 | C | T | 221 | Kif3b | intronic | . | . | . | 2qH1 | rs28283047 | 0 | 1 |
| chr2 | 153320765 | C | T | 222 | Kif3b | intronic | . | . | . | 2qH1 | rs28283046 | 0 | 1 |
| chr2 | 153320925 | A | G | 176 | Kif3b | intronic | . | . | . | 2qH1 | rs28283045 | 0 | 1 |
| chr2 | 153320986 | A | G | 129 | Kif3b | intronic | . | . | . | 2qH1 | rs47380881 | 0 | 1 |
| chr2 | 153322805 | T | C | 157 | Kif3b | intronic | . | . | . | 2qH1 | rs28283035 | 0 | 1 |
| chr2 | 153323056 | A | G | 115 | Kif3b | intronic | . | . | . | 2qH1 | rs28283034 | 0 | 1 |
| chr2 | 153323363 | C | T | 26.97 | Kif3b | intronic | . | . | . | 2qH1 | rs48282194 | 0 | 1 |
| chr2 | 153323407 | T | C | 63 | Kif3b | intronic | . | . | . | 2qH1 | rs28283031 | 0 | 1 |
| chr2 | 153323480 | T | G | 89 | Kif3b | intronic | . | . | . | 2qH1 | rs49130482 | 0 | 1 |
| chr2 | 153323503 | C | T | 137 | Kif3b | intronic | . | . | . | 2qH1 | rs28283030 | 0 | 1 |
| chr2 | 153329732 | G | A | 199 | Kif3b | exonic | missense SNV | Kif3b:NM_008444:exon8:c.G2129A:p.S710N | . | 2qH1 | rs28283018 | 0 | 1 |
| chr2 | 153329800 | G | A | 132 | Kif3b | intronic | . | . | . | 2qH1 | rs28283017 | 0 | 1 |
| chr2 | 153329825 | C | T | 96 | Kif3b | intronic | . | . | . | 2qH1 | rs28283016 | 0 | 1 |
| chr2 | 153342711 | T | C | 191 | 2500004C02Rik | ncRNA_exonic | . | . | . | 2qH1 | rs47179965 | 0 | 1 |
| chr2 | 153342810 | C | A | 221 | 2500004C02Rik | ncRNA_exonic | . | . | . | 2qH1 | rs50485849 | 0 | 1 |
| chr2 | 153342901 | T | A | 211 | 2500004C02Rik | ncRNA_intronic | . | . | . | 2qH1 | rs50788419 | 0 | 1 |
| chr2 | 153352206 | A | G | 221 | Asxl1 | exonic | synonymous SNV | Asxl1:NM_001039939:exon2:c.A75G:p.S25S | . | 2qH1 | rs28267961 | 0 | 1 |
| chr2 | 153354809 | A | G | 96 | Asxl1 | intronic | . | . | . | 2qH1 | rs28267956 | 0 | 1 |
| chr2 | 153357254 | A | G | 168 | Asxl1 | intronic | . | . | . | 2qH1 | rs51389538 | 0 | 1 |
| chr2 | 153357273 | C | T | 95 | Asxl1 | intronic | . | . | . | 2qH1 | rs266070770 | 0 | 1 |
| chr2 | 153357279 | C | T | 94 | Asxl1 | intronic | . | . | . | 2qH1 | rs232926684 | 0 | 1 |
| chr2 | 153391953 | T | A | 108 | Asxl1 | intronic | . | . | . | 2qH1 | rs51369538 | 0 | 1 |
| chr2 | 153392750 | C | G | 108 | Asxl1 | intronic | . | . | . | 2qH1 | rs28267873 | 0 | 1 |
| chr2 | 153393316 | G | A | 151 | Asxl1 | intronic | . | . | . | 2qH1 | rs28267872 | 0 | 1 |
| chr2 | 153393620 | G | A | 137 | Asxl1 | splicing | . | . | . | 2qH1 | rs28267871 | 0 | 1 |
| chr2 | 153397857 | T | C | 222 | Asxl1 | exonic | missense SNV | Asxl1:NM_001039939:exon11:c.T1567C:p.S523P | . | 2qH1 | rs28267860 | 0 | 1 |

| | | | | | | | | | | | | | |
|---|---|---|---|---|---|---|---|---|---|---|---|---|---|
| chr2 | 153397881 | T | C | 222 | Asxl1 | exonic | missense SNV | Asxl1:NM_001039939:exon11:c.T1591C:p.S531P | . | 2qH1 | rs33147334 | 0 | 1 |
| chr2 | 153397898 | A | G | 202 | Asxl1 | exonic | synonymous SNV | Asxl1:NM_001039939:exon11:c.A1608G:p.K536K | . | 2qH1 | rs28267859 | 0 | 1 |
| chr2 | 153398026 | T | C | 221 | Asxl1 | intronic | . | . | . | 2qH1 | rs28267858 | 0 | 1 |
| chr2 | 153398098 | G | A | 118 | Asxl1 | intronic | . | . | . | 2qH1 | rs28267857 | 0 | 1 |
| chr2 | 153400007 | G | A | 221 | Asxl1 | exonic | missense SNV | Asxl1:NM_001039939:exon12:c.G2479A:p.V827I | . | 2qH1 | rs28267854 | 0 | 1 |
| chr2 | 153400071 | A | C | 221 | Asxl1 | exonic | missense SNV | Asxl1:NM_001039939:exon12:c.A2543C:p.D848A | . | 2qH1 | rs28267853 | 0 | 1 |
| chr2 | 153400883 | G | C | 221 | Asxl1 | exonic | missense SNV | Asxl1:NM_001039939:exon12:c.G3355C:p.D1119H | . | 2qH1 | rs28267852 | 0 | 1 |
| chr2 | 153401052 | C | T | 221 | Asxl1 | exonic | missense SNV | Asxl1:NM_001039939:exon12:c.C3524T:p.A1175V | . | 2qH1 | rs28267851 | 0 | 1 |
| chr2 | 153401088 | A | T | 221 | Asxl1 | exonic | missense SNV | Asxl1:NM_001039939:exon12:c.A3560T:p.E1187V | . | 2qH1 | rs28267850 | 0 | 1 |
| chr2 | 153401378 | A | G | 222 | Asxl1 | exonic | missense SNV | Asxl1:NM_001039939:exon12:c.A3850G:p.T1284A | . | 2qH1 | rs13476848 | 0 | 1 |
| chr2 | 153401541 | A | G | 222 | Asxl1 | exonic | missense SNV | Asxl1:NM_001039939:exon12:c.A4013G:p.D1338G | . | 2qH1 | rs28267849 | 0 | 1 |
| chr2 | 153401550 | A | G | 222 | Asxl1 | exonic | missense SNV | Asxl1:NM_001039939:exon12:c.A4022G:p.K1341R | . | 2qH1 | rs28267848 | 0 | 1 |
| chr2 | 153401764 | C | T | 221 | Asxl1 | exonic | synonymous SNV | Asxl1:NM_001039939:exon12:c.C4236T:p.A1412A | . | 2qH1 | rs28267847 | 0 | 1 |
| chr2 | 153401927 | G | A | 222 | Asxl1 | exonic | missense SNV | Asxl1:NM_001039939:exon12:c.G4399A:p.A1467T | . | 2qH1 | rs28267845 | 0 | 1 |
| chr2 | 153411541 | C | A | 127 | Nol41 | UTR3 | . | . | . | 2qH1 | rs28267803 | 0 | 1 |
| chr2 | 153413781 | C | A | 196 | Nol41 | intronic | . | . | . | 2qH1 | rs33372254 | 0 | 1 |
| chr2 | 153413883 | A | G | 222 | Nol41 | intronic | . | . | . | 2qH1 | rs28267794 | 0 | 1 |
| chr2 | 153414120 | A | G | 222 | Nol41 | intronic | . | . | . | 2qH1 | rs47630063 | 0 | 1 |
| chr2 | 153414129 | G | A | 222 | Nol41 | intronic | . | . | . | 2qH1 | rs48799533 | 0 | 1 |
| chr2 | 153414417 | G | A | 125 | Nol41 | intronic | . | . | . | 2qH1 | rs28267792 | 0 | 1 |
| chr2 | 153416635 | A | G | 182 | Nol41 | intronic | . | . | . | 2qH1 | rs32884432 | 0 | 1 |
| chr2 | 153416816 | G | A | 222 | Nol41 | exonic | synonymous SNV | Nol41:NM_001001986:exon7:c.C918T:p.H306H,Nol41:NM_001134300:exon10:c.C1650T:p.H550H | . | 2qH1 | rs28267779 | 0 | 1 |
| chr2 | 153417170 | C | T | 61 | Nol41 | intronic | . | . | . | 2qH1 | rs29731445 | 0 | 1 |
| chr2 | 153417197 | C | T | 96 | Nol41 | intronic | . | . | . | 2qH1 | rs33387804 | 0 | 1 |
| chr2 | 153417296 | G | C | 188 | Nol41 | intronic | . | . | . | 2qH1 | rs29504879 | 0 | 1 |
| chr2 | 153417465 | A | G | 222 | Nol41 | exonic | synonymous SNV | Nol41:NM_001001986:exon6:c.T786C:p.H262H,Nol41:NM_001134300:exon9:c.T1518C:p.H506H | . | 2qH1 | rs33488972 | 0 | 1 |
| chr2 | 153417870 | G | T | 217 | Nol41 | exonic | synonymous SNV | Nol41:NM_001001986:exon5:c.C583A:p.R195R,Nol41:NM_001134300:exon8:c.C1315A:p.R439R | . | 2qH1 | rs33514103 | 0 | 1 |
| chr2 | 153417929 | C | T | 220 | Nol41 | intronic | . | . | . | 2qH1 | rs32827790 | 0 | 1 |
| chr2 | 153418210 | A | G | 222 | Nol41 | intronic | . | . | . | 2qH1 | rs48283632 | 0 | 1 |
| chr2 | 153420443 | C | T | 62 | Nol41 | intronic | . | . | . | 2qH1 | rs33721440 | 0 | 1 |
| chr2 | 153420676 | G | A | 172 | Nol41 | exonic | synonymous SNV | Nol41:NM_001001986:exon3:c.C243T:p.T81T,Nol41:NM_001134300:exon6:c.C975T:p.T325T | . | 2qH1 | rs28267775 | 0 | 1 |
| chr2 | 153470596 | A | G | 94 | Nol41 | intronic | . | . | . | 2qH1 | rs4223571 | 0 | 1 |
| chr2 | 153470615 | G | C | 107 | Nol41 | intronic | . | . | . | 2qH1 | rs4223572 | 0 | 1 |
| chr2 | 153478039 | C | T | 115 | Nol41 | intronic | . | . | . | 2qH1 | rs33574720 | 0 | 1 |
| chr2 | 153483879 | T | C | 222 | Nol41 | intronic | . | . | . | 2qH1 | rs28267691 | 0 | 1 |
| chr2 | 153483965 | A | G | 156 | Nol41 | intronic | . | . | . | 2qH1 | rs33301220 | 0 | 1 |
| chr2 | 153619325 | G | A | 104 | Commd7 | UTR3 | . | . | . | 2qH1 | . | 0 | 1 |
| chr2 | 153619396 | A | C | 208 | Commd7 | UTR3 | . | . | . | 2qH1 | . | 0 | 1 |
| chr2 | 153619426 | G | A | 221 | Commd7 | UTR3 | . | . | . | 2qH1 | rs4223573 | 0 | 1 |
| chr2 | 153620408 | C | G | 148 | Commd7 | intronic | . | . | . | 2qH1 | rs28249406 | 0 | 1 |
| chr2 | 153620447 | C | T | 141 | Commd7 | exonic | missense SNV | Commd7:NM_133850:exon8:c.G525A:p.M175I,Commd7:NM_001195390:exon9:c.G312A:p.M104I | . | 2qH1 | rs28249405 | 0 | 1 |
| chr2 | 153621849 | T | C | 222 | Commd7 | intronic | . | . | . | 2qH1 | rs28249403 | 0 | 1 |
| chr2 | 153621921 | G | A | 222 | Commd7 | intronic | . | . | . | 2qH1 | rs28249402 | 0 | 1 |
| chr2 | 153622194 | C | T | 195 | Commd7 | intronic | . | . | . | 2qH1 | rs28249399 | 0 | 1 |
| chr2 | 153622243 | G | A | 81 | Commd7 | intronic | . | . | . | 2qH1 | rs28249398 | 0 | 1 |
| chr2 | 153622259 | T | C | 87 | Commd7 | intronic | . | . | . | 2qH1 | rs28249397 | 0 | 1 |
| chr2 | 153622608 | G | A | 222 | Commd7 | intronic | . | . | . | 2qH1 | rs33261839 | 0 | 1 |
| chr2 | 153622815 | T | C | 222 | Commd7 | exonic | synonymous SNV | Commd7:NM_133850:exon4:c.A255G:p.K85K,Commd7:NM_001195390:exon5:c.A42G:p.K14K | . | 2qH1 | rs28249395 | 0 | 1 |
| chr2 | 153622888 | G | T | 188 | Commd7 | intronic | . | . | . | 2qH1 | rs28249393 | 0 | 1 |
| chr2 | 153622964 | A | G | 40.901 | Commd7 | intronic | . | . | . | 2qH1 | rs28249392 | 0 | 1 |
| chr2 | 153628395 | G | A | 99 | Commd7 | intronic | . | . | . | 2qH1 | rs50273735 | 0 | 1 |
| chr2 | 153628400 | T | C | 103 | Commd7 | intronic | . | . | . | 2qH1 | rs50862969 | 0 | 1 |
| chr2 | 153628478 | T | A | 221 | Commd7 | exonic | synonymous SNV | Commd7:NM_133850:exon3:c.A228T:p.L76L,Commd7:NM_001195390:exon4:c.A15T:p.L5L | . | 2qH1 | rs28249368 | 0 | 1 |
| chr2 | 153629238 | G | C | 221 | Commd7 | exonic | synonymous SNV | Commd7:NM_133850:exon2:c.C93G:p.S31S | . | 2qH1 | rs27400153 | 0 | 1 |
| chr2 | 153629267 | T | G | 190 | Commd7 | intronic | . | . | . | 2qH1 | rs231648832 | 0 | 1 |
| chr2 | 153649027 | T | C | 222 | . | upstream | . | . | . | 2qH1 | rs29666562 | 0 | 1 |
| chr2 | 153649076 | G | T | 222 | . | upstream | . | . | . | 2qH1 | rs33319989 | 0 | 1 |
| chr2 | 153661256 | G | A | 81 | Dnmt3b | intronic | . | . | . | 2qH1 | rs27400096 | 0 | 1 |
| chr2 | 153662654 | G | A | 139 | Dnmt3b | intronic | . | . | . | 2qH1 | rs27400093 | 0 | 1 |
| chr2 | 153662735 | A | G | 111 | Dnmt3b | exonic | missense SNV | Dnmt3b:NM_001122997:exon4:c.A256G:p.R86G,Dnmt3b:NM_001271744:exon4:c.A256G:p.R86G,Dnmt3b:NM_( | . | 2qH1 | rs27400092 | 0 | 1 |
| chr2 | 153662749 | A | G | 116 | Dnmt3b | exonic | synonymous SNV | Dnmt3b:NM_001122997:exon4:c.A270G:p.V90V,Dnmt3b:NM_001271744:exon4:c.A270G:p.V90V,Dnmt3b:NM_( | . | 2qH1 | rs27400091 | 0 | 1 |
| chr2 | 153662794 | T | G | 153 | Dnmt3b | exonic | synonymous SNV | Dnmt3b:NM_001122997:exon4:c.T315G:p.R105R,Dnmt3b:NM_001271744:exon4:c.T315G:p.R105R,Dnmt3b:NM | . | 2qH1 | rs32863302 | 0 | 1 |
| chr2 | 153662842 | T | C | 198 | Dnmt3b | intronic | . | . | . | 2qH1 | rs27400090 | 0 | 1 |
| chr2 | 153665174 | A | G | 109 | Dnmt3b | intronic | . | . | . | 2qH1 | rs27400078 | 0 | 1 |
| chr2 | 153665218 | C | G | 154 | Dnmt3b | intronic | . | . | . | 2qH1 | rs27400077 | 0 | 1 |
| chr2 | 153665225 | C | A | 154 | Dnmt3b | intronic | . | . | . | 2qH1 | rs29962344 | 0 | 1 |
| chr2 | 153665759 | A | G | 66 | Dnmt3b | intronic | . | . | . | 2qH1 | rs27400074 | 0 | 1 |
| chr2 | 153665825 | C | T | 148 | Dnmt3b | intronic | . | . | . | 2qH1 | rs27400073 | 0 | 1 |
| chr2 | 153665927 | A | G | 221 | Dnmt3b | exonic | synonymous SNV | Dnmt3b:NM_001122997:exon6:c.A501G:p.S167S,Dnmt3b:NM_001271744:exon6:c.A501G:p.S167S,Dnmt3b:NM | . | 2qH1 | rs3722497 | 0 | 1 |
| chr2 | 153666044 | T | C | 162 | Dnmt3b | exonic | synonymous SNV | Dnmt3b:NM_001122997:exon6:c.T618C:p.G206G,Dnmt3b:NM_001271744:exon6:c.T618C:p.G206G,Dnmt3b:NM | . | 2qH1 | rs27400072 | 0 | 1 |
| chr2 | 153670034 | T | C | 40.2133 | Dnmt3b | intronic | . | . | . | 2qH1 | rs27385363 | 0 | 1 |
| chr2 | 153670754 | C | G | 189 | Dnmt3b | intronic | . | . | . | 2qH1 | rs27385360 | 0 | 1 |
| chr2 | 153670947 | A | G | 222 | Dnmt3b | intronic | . | . | . | 2qH1 | rs33030306 | 0 | 1 |
| chr2 | 153670956 | T | C | 221 | Dnmt3b | intronic | . | . | . | 2qH1 | rs49605614 | 0 | 1 |
| chr2 | 153672094 | T | G | 33.6938 | Dnmt3b | intronic | . | . | . | 2qH1 | rs27385354 | 0 | 1 |
| chr2 | 153672127 | A | T | 69 | Dnmt3b | intronic | . | . | . | 2qH1 | rs50105271 | 0 | 1 |
| chr2 | 153672199 | A | G | 204 | Dnmt3b | intronic | . | . | . | 2qH1 | rs27385190 | 0 | 1 |
| chr2 | 153672312 | G | T | 196 | Dnmt3b | exonic | missense SNV | Dnmt3b:NM_001271745:exon10:c.G1184T:p.R395L,Dnmt3b:NM_001271747:exon10:c.G1184T:p.R395L,Dnmt3 | . | 2qH1 | . | 0 | 1 |
| chr2 | 153672453 | A | T | 221 | Dnmt3b | intronic | . | . | . | 2qH1 | rs48204040 | 0 | 1 |
| chr2 | 153672543 | A | G | 221 | Dnmt3b | splicing | . | . | . | 2qH1 | rs27385188 | 0 | 1 |
| chr2 | 153672579 | A | G | 173 | Dnmt3b | intronic | . | . | . | 2qH1 | rs47940951 | 0 | 1 |
| chr2 | 153672597 | T | C | 188 | Dnmt3b | intronic | . | . | . | 2qH1 | rs50887153 | 0 | 1 |
| chr2 | 153673832 | T | C | 103 | Dnmt3b | intronic | . | . | . | 2qH1 | rs27385346 | 0 | 1 |
| chr2 | 153674175 | C | T | 87 | Dnmt3b | intronic | . | . | . | 2qH1 | rs46560625 | 0 | 1 |
| chr2 | 153674381 | T | C | 91 | Dnmt3b | exonic | synonymous SNV | Dnmt3b:NM_001271745:exon13:c.T1365C:p.D455D,Dnmt3b:NM_001271747:exon13:c.T1365C:p.D455D,Dnmt3 | . | 2qH1 | rs46525401 | 0 | 1 |
| chr2 | 153674444 | T | C | 195 | Dnmt3b | exonic | synonymous SNV | Dnmt3b:NM_001271745:exon13:c.T1428C:p.S476S,Dnmt3b:NM_001271747:exon13:c.T1428C:p.S476S,Dnmt3 | . | 2qH1 | rs50030111 | 0 | 1 |
| chr2 | 153674816 | T | C | 103 | Dnmt3b | intronic | . | . | . | 2qH1 | rs27385337 | 0 | 1 |
| chr2 | 153674848 | C | A | 121 | Dnmt3b | intronic | . | . | . | 2qH1 | rs581402809 | 0 | 1 |
| chr2 | 153676637 | G | A | 166 | Dnmt3b | intronic | . | . | . | 2qH1 | rs32966598 | 0 | 1 |
| chr2 | 153676781 | T | C | 221 | Dnmt3b | exonic | synonymous SNV | Dnmt3b:NM_001271745:exon16:c.T1803C:p.T601T,Dnmt3b:NM_001271747:exon16:c.T1803C:p.T601T,Dnmt3 | . | 2qH1 | rs27385321 | 0 | 1 |
| chr2 | 153676863 | C | T | 222 | Dnmt3b | intronic | . | . | . | 2qH1 | rs27385319 | 0 | 1 |
| chr2 | 153677056 | T | A | 139 | Dnmt3b | intronic | . | . | . | 2qH1 | rs27385318 | 0 | 1 |
| chr2 | 153677502 | C | T | 188 | Dnmt3b | intronic | . | . | . | 2qH1 | rs27385316 | 0 | 1 |
| chr2 | 153677771 | C | T | 207 | Dnmt3b | intronic | . | . | . | 2qH1 | rs27385315 | 0 | 1 |
| chr2 | 153678509 | T | A | 221 | Dnmt3b | exonic | synonymous SNV | Dnmt3b:NM_001271745:exon19:c.T2142A:p.A714A,Dnmt3b:NM_001271747:exon19:c.T2142A:p.A714A,Dnmt3 | . | 2qH1 | rs215845983 | 0 | 1 |
| chr2 | 153683540 | T | G | 108 | Dnmt3b | intronic | . | . | . | 2qH1 | rs27385285 | 0 | 1 |
| chr2 | 153684234 | G | C | 183 | Dnmt3b | intronic | . | . | . | 2qH1 | rs29927698 | 0 | 1 |
| chr2 | 153684677 | T | A | 106 | Dnmt3b | intronic | . | . | . | 2qH1 | rs27385282 | 0 | 1 |
| chr2 | 153686306 | C | T | 222 | Dnmt3b | exonic | synonymous SNV | Dnmt3b:NM_001271747:exon20:c.C2238T:p.R746R,Dnmt3b:NM_001271746:exon21:c.C2298T:p.R766R,Dnmt3 | . | 2qH1 | rs33138775 | 0 | 1 |
| chr2 | 153686345 | A | G | 221 | Dnmt3b | exonic | synonymous SNV | Dnmt3b:NM_001271747:exon20:c.A2277G:p.V759V,Dnmt3b:NM_001271746:exon21:c.A2337G:p.V779V,Dnmt3 | . | 2qH1 | rs27385271 | 0 | 1 |
| chr2 | 153686390 | C | T | 177 | Dnmt3b | exonic | synonymous SNV | Dnmt3b:NM_001271747:exon20:c.C2322T:p.A774A,Dnmt3b:NM_001271746:exon21:c.C2382T:p.A794A,Dnmt3 | . | 2qH1 | rs27385270 | 0 | 1 |
| chr2 | 153714951 | T | G | 175 | . | intergenic | . | . | . | 2qH1 | rs107974189 | 0 | 1 |
| chr2 | 153714987 | C | T | 47.7158 | . | intergenic | . | . | . | 2qH1 | rs108506682 | 0 | 1 |
| chr2 | 153715005 | T | C | 44.0217 | . | intergenic | . | . | . | 2qH1 | rs108284820 | 0 | 1 |
| chr2 | 153715184 | C | T | 121 | . | intergenic | . | . | . | 2qH1 | rs579640846 | 0 | 1 |
| chr2 | 153716679 | A | T | 84 | . | intergenic | . | . | . | 2qH1 | rs49306454 | 0 | 1 |
| chr2 | 153716755 | A | G | 141 | . | intergenic | . | . | . | 2qH1 | rs50042460 | 0 | 1 |
| chr2 | 153716798 | T | C | 94 | . | intergenic | . | . | . | 2qH1 | . | 0 | 1 |
| chr2 | 153716848 | C | T | 117 | . | intergenic | . | . | . | 2qH1 | rs27385182 | 0 | 1 |
| chr2 | 153716884 | C | T | 153 | . | intergenic | . | . | . | 2qH1 | rs27385180 | 0 | 1 |
| chr2 | 153716993 | T | C | 195 | . | intergenic | . | . | . | 2qH1 | rs50650561 | 0 | 1 |
| chr2 | 153717003 | T | C | 144 | . | intergenic | . | . | . | 2qH1 | rs27385177 | 0 | 1 |
| chr2 | 153717277 | C | T | 92 | . | intergenic | . | . | . | 2qH1 | rs108522179 | 0 | 1 |
| chr2 | 153717300 | C | T | 121 | . | intergenic | . | . | . | 2qH1 | rs107821224 | 0 | 1 |
| chr2 | 153717385 | T | C | 120 | . | intergenic | . | . | . | 2qH1 | rs27385168 | 0 | 1 |
| chr2 | 153717419 | C | G | 221 | . | intergenic | . | . | . | 2qH1 | . | 0 | 1 |
| chr2 | 153717576 | T | C | 222 | . | intergenic | . | . | . | 2qH1 | rs33316162 | 0 | 1 |
| chr2 | 153718043 | T | C | 57 | . | intergenic | . | . | . | 2qH1 | rs107729195 | 0 | 1 |
| chr2 | 153718227 | G | A | 222 | . | intergenic | . | . | . | 2qH1 | rs27385162 | 0 | 1 |
| chr2 | 153718392 | C | A | 144 | . | intergenic | . | . | . | 2qH1 | rs27385158 | 0 | 1 |
| chr2 | 153719151 | C | T | 165 | . | intergenic | . | . | . | 2qH1 | rs33437885 | 0 | 1 |
| chr2 | 153719158 | A | C | 222 | . | intergenic | . | . | . | 2qH1 | rs33494965 | 0 | 1 |
| chr2 | 153723532 | C | T | 221 | . | intergenic | . | . | . | 2qH1 | rs107790877 | 0 | 1 |
| chr2 | 153723593 | A | C | 143 | . | intergenic | . | . | . | 2qH1 | rs108908875 | 0 | 1 |
| chr2 | 153726364 | T | C | 140 | . | intergenic | . | . | . | 2qH1 | rs27385126 | 0 | 1 |
| chr2 | 153727183 | C | T | 165 | . | intergenic | . | . | . | 2qH1 | rs27385121 | 0 | 1 |
| chr2 | 153756125 | T | A | 222 | Mapre1 | intronic | . | . | . | 2qH1 | rs52355968 | 0 | 1 |
| chr2 | 153756217 | A | G | 133 | Mapre1 | intronic | . | . | . | 2qH1 | rs52493500 | 0 | 1 |
| chr2 | 153757903 | T | A | 92 | Mapre1 | intronic | . | . | . | 2qH1 | rs27385071 | 0 | 1 |
| chr2 | 153761511 | C | T | 158 | Mapre1 | intronic | . | . | . | 2qH1 | rs27369763 | 0 | 1 |
| chr2 | 153761837 | T | C | 108 | Mapre1 | intronic | . | . | . | 2qH1 | rs27369762 | 0 | 1 |
| chr2 | 153765159 | G | A | 221 | Mapre1 | intronic | . | . | . | 2qH1 | rs33583277 | 0 | 1 |
| chr2 | 153766838 | G | A | 222 | Mapre1 | intronic | . | . | . | 2qH1 | rs27369732 | 0 | 1 |
| chr2 | 153780867 | T | C | 49.613 | . | upstream | . | . | . | 2qH1 | rs27369662 | 0 | 1 |
| chr2 | 153780878 | G | A | 137 | . | upstream | . | . | . | 2qH1 | rs48815523 | 0 | 1 |
| chr2 | 153780960 | T | G | 201 | Efcab8 | ncRNA_exonic | . | . | . | 2qH1 | rs50980194 | 0 | 1 |
| chr2 | 153781974 | C | T | 90 | Efcab8 | ncRNA_intronic | . | . | . | 2qH1 | rs27369660 | 0 | 1 |
| chr2 | 153783748 | C | T | 222 | Efcab8 | ncRNA_exonic | . | . | . | 2qH1 | rs27369651 | 0 | 1 |
| chr2 | 153783876 | C | T | 221 | Efcab8 | ncRNA_intronic | . | . | . | 2qH1 | rs27369649 | 0 | 1 |
| chr2 | 153787701 | C | T | 222 | Efcab8 | ncRNA_exonic | . | . | . | 2qH1 | rs33521862 | 0 | 1 |
| chr2 | 153788952 | A | G | 38.1689 | Efcab8 | ncRNA_intronic | . | . | . | 2qH1 | rs252753840 | 0 | 1 |
| chr2 | 153789064 | G | A | 93 | Efcab8 | ncRNA_intronic | . | . | . | 2qH1 | rs216051809 | 0 | 1 |
| chr2 | 153789095 | G | A | 107 | Efcab8 | ncRNA_intronic | . | . | . | 2qH1 | rs27369648 | 0 | 1 |
| chr2 | 153789113 | C | A | 205 | Efcab8 | ncRNA_intronic | . | . | . | 2qH1 | rs51847640 | 0 | 1 |
| chr2 | 153789339 | T | G | 104 | Efcab8 | ncRNA_exonic | . | . | . | 2qH1 | rs583057837 | 0 | 1 |
| chr2 | 153789395 | A | C | 98 | Efcab8 | ncRNA_exonic | . | . | . | 2qH1 | rs50180888 | 0 | 1 |
| chr2 | 153856027 | A | G | 92 | . | downstream | . | . | . | 2qH1 | rs27343545 | 0 | 1 |
| chr2 | 153856163 | G | A | 221 | . | downstream | . | . | . | 2qH1 | rs27343544 | 0 | 1 |
| chr2 | 153858899 | A | G | 83 | Sun5 | intronic | . | . | . | 2qH1 | rs219994530 | 0 | 1 |
| chr2 | 153858904 | A | G | 135 | Sun5 | intronic | . | . | . | 2qH1 | rs237577368 | 0 | 1 |
| chr2 | 153858911 | T | C | 155 | Sun5 | intronic | . | . | . | 2qH1 | rs263491666 | 0 | 1 |
| chr2 | 153859080 | G | A | 222 | Sun5 | exonic | synonymous SNV | Sun5:NM_029599:exon10:c.C693T:p.R231R,Sun5:NM_001305048:exon11:c.C768T:p.R256R | . | 2qH1 | rs27343535 | 0 | 1 |
| chr2 | 153859238 | G | A | 114 | Sun5 | intronic | . | . | . | 2qH1 | rs49288255 | 0 | 1 |
| chr2 | 153859246 | G | A | 110 | Sun5 | intronic | . | . | . | 2qH1 | rs49456593 | 0 | 1 |
| chr2 | 153860761 | A | T | 222 | Sun5 | intronic | . | . | . | 2qH1 | rs27343532 | 0 | 1 |
| chr2 | 153865273 | A | G | 149 | Sun5 | intronic | . | . | . | 2qH1 | rs27343516 | 0 | 1 |
| chr2 | 153865373 | G | T | 221 | Sun5 | exonic | synonymous SNV | Sun5:NM_029599:exon7:c.C384A:p.I128I,Sun5:NM_001305048:exon8:c.C459A:p.I153I | . | 2qH1 | rs27343515 | 0 | 1 |
| chr2 | 153865519 | T | C | 121 | Sun5 | intronic | . | . | . | 2qH1 | rs27343514 | 0 | 1 |
| chr2 | 153865587 | T | C | 53 | Sun5 | intronic | . | . | . | 2qH1 | rs50704747 | 0 | 1 |
| chr2 | 153866155 | A | C | 75 | Sun5 | intronic | . | . | . | 2qH1 | rs27343512 | 0 | 1 |
| chr2 | 153866874 | C | T | 221 | Sun5 | intronic | . | . | . | 2qH1 | rs27343509 | 0 | 1 |
| chr2 | 153868175 | G | A | 184 | Sun5 | intronic | . | . | . | 2qH1 | rs33747700 | 0 | 1 |
| chr2 | 153868191 | C | T | 184 | Sun5 | intronic | . | . | . | 2qH1 | rs32961311 | 0 | 1 |
| chr2 | 153868319 | C | T | 222 | Sun5 | intronic | . | . | . | 2qH1 | rs27343496 | 0 | 1 |
| chr2 | 153869391 | C | T | 132 | Sun5 | intronic | . | . | . | 2qH1 | rs27343490 | 0 | 1 |
| chr2 | 153871034 | T | A | 195 | Sun5 | UTR5 | . | . | . | 2qH1 | rs27343476 | 0 | 1 |
| chr2 | 153871076 | C | G | 111 | Sun5 | UTR5 | . | . | . | 2qH1 | rs27343475 | 0 | 1 |
| chr2 | 153875783 | C | G | 81 | Bpifb2 | intronic | . | . | . | 2qH1 | rs27343440 | 0 | 1 |
| chr2 | 153875809 | C | T | 126 | Bpifb2 | intronic | . | . | . | 2qH1 | rs27343438 | 0 | 1 |
| chr2 | 153878310 | A | G | 59 | Bpifb2 | intronic | . | . | . | 2qH1 | rs27343404 | 0 | 1 |
| chr2 | 153878378 | G | A | 108 | Bpifb2 | intronic | . | . | . | 2qH1 | rs46753073 | 0 | 1 |
| chr2 | 153878604 | A | G | 116 | Bpifb2 | intronic | . | . | . | 2qH1 | rs27343402 | 0 | 1 |
| chr2 | 153879983 | G | A | 89 | Bpifb2 | intronic | . | . | . | 2qH1 | rs45846574 | 0 | 1 |
| chr2 | 153880008 | T | C | 151 | Bpifb2 | intronic | . | . | . | 2qH1 | rs45799871 | 0 | 1 |
| chr2 | 153880041 | T | C | 146 | Bpifb2 | exonic | synonymous SNV | Bpifb2:NM_025631:exon4:c.T216C:p.V72V | . | 2qH1 | rs255932313 | 0 | 1 |
| chr2 | 153882433 | C | G | 87 | Bpifb2 | intronic | . | . | . | 2qH1 | rs29868030 | 0 | 1 |
| chr2 | 153882448 | C | T | 86 | Bpifb2 | intronic | . | . | . | 2qH1 | . | 0 | 1 |
| chr2 | 153883520 | A | C | 222 | Bpifb2 | intronic | . | . | . | 2qH1 | rs27401324 | 0 | 1 |
| chr2 | 153883660 | A | T | 222 | Bpifb2 | intronic | . | . | . | 2qH1 | rs29518883 | 0 | 1 |
| chr2 | 153883711 | T | C | 214 | Bpifb2 | intronic | . | . | . | 2qH1 | rs27401322 | 0 | 1 |
| chr2 | 153888979 | A | G | 67 | Bpifb2 | intronic | . | . | . | 2qH1 | rs27401295 | 0 | 1 |
| chr2 | 153889107 | T | C | 211 | Bpifb2 | exonic | synonymous SNV | Bpifb2:NM_025631:exon8:c.T597C:p.P199P | . | 2qH1 | rs27401294 | 0 | 1 |
| chr2 | 153889340 | C | A | 64 | Bpifb2 | intronic | . | . | . | 2qH1 | rs27401292 | 0 | 1 |
| chr2 | 153889582 | G | A | 204 | Bpifb2 | intronic | . | . | . | 2qH1 | rs27401287 | 0 | 1 |
| chr2 | 153889707 | T | C | 222 | Bpifb2 | intronic | . | . | . | 2qH1 | rs33759427 | 0 | 1 |

| | | | | | | | | | | | | | |
|---|---|---|---|---|---|---|---|---|---|---|---|---|---|
| chr2 | 153889708 | G | A | 222 | Bpifb2 | intronic | . | . | . | 2qH1 | rs33363051 | 0 | 1 |
| chr2 | 153889869 | T | C | 222 | Bpifb2 | intronic | . | . | . | 2qH1 | rs27401286 | 0 | 1 |
| chr2 | 153890430 | A | G | 58 | Bpifb2 | exonic | synonymous SNV | Bpifb2:NM_025631:exon11:c.A948G:p.T316T | . | 2qH1 | rs27401285 | 0 | 1 |
| chr2 | 153890556 | C | G | 207 | Bpifb2 | exonic | missense SNV | Bpifb2:NM_025631:exon11:c.C1074G:p.F358L | . | 2qH1 | rs27401283 | 0 | 1 |
| chr2 | 153890984 | A | G | 186 | Bpifb2 | intronic | . | . | . | 2qH1 | rs27401279 | 0 | 1 |
| chr2 | 153891086 | T | C | 222 | Bpifb2 | intronic | . | . | . | 2qH1 | rs27401277 | 0 | 1 |
| chr2 | 153891366 | C | T | 222 | Bpifb2 | intronic | . | . | . | 2qH1 | rs27401274 | 0 | 1 |
| chr2 | 153891392 | C | T | 178 | Bpifb2 | intronic | . | . | . | 2qH1 | rs50174371 | 0 | 1 |
| chr2 | 153891875 | C | T | 32.5235 | Bpifb2 | intronic | . | . | . | 2qH1 | rs27386267 | 0 | 1 |
| chr2 | 153892172 | C | A | 30.4611 | Bpifb2 | intronic | . | . | . | 2qH1 | rs51217910 | 0 | 1 |
| chr2 | 153892182 | T | C | 70 | Bpifb2 | intronic | . | . | . | 2qH1 | rs27386265 | 0 | 1 |
| chr2 | 153892191 | T | C | 63 | Bpifb2 | intronic | . | . | . | 2qH1 | rs51151834 | 0 | 1 |
| chr2 | 153892380 | A | C | 221 | Bpifb2 | exonic | synonymous SNV | Bpifb2:NM_025631:exon15:c.A1314C:p.L438L | . | 2qH1 | rs27386264 | 0 | 1 |
| chr2 | 153892409 | G | A | 171 | Bpifb2 | exonic | missense SNV | Bpifb2:NM_025631:exon15:c.G1343A:p.R448H | . | 2qH1 | rs27386263 | 0 | 1 |
| chr2 | 153892421 | A | C | 152 | Bpifb2 | intronic | . | . | . | 2qH1 | rs27386262 | 0 | 1 |
| chr2 | 153894872 | C | T | 102 | Bpifb2 | intronic | . | . | . | 2qH1 | rs27386233 | 0 | 1 |
| chr2 | 153894883 | T | A | 126 | Bpifb2 | intronic | . | . | . | 2qH1 | rs27386231 | 0 | 1 |
| chr2 | 153895120 | A | G | 211 | Bpifb2 | UTR3 | . | . | . | 2qH1 | rs27386229 | 0 | 1 |
| chr2 | 153902625 | T | C | 67 | Bpifb6 | intronic | . | . | . | 2qH1 | rs48272393 | 0 | 1 |
| chr2 | 153902626 | C | T | 67 | Bpifb6 | intronic | . | . | . | 2qH1 | rs46047159 | 0 | 1 |
| chr2 | 153902677 | C | T | 93 | Bpifb6 | exonic | synonymous SNV | Bpifb6:NM_199303:exon3:c.C37T:p.L13L | . | 2qH1 | rs27386169 | 0 | 1 |
| chr2 | 153902705 | G | T | 145 | Bpifb6 | exonic | missense SNV | Bpifb6:NM_199303:exon3:c.G65T:p.G22V | . | 2qH1 | rs27386168 | 0 | 1 |
| chr2 | 153902877 | T | A | 218 | Bpifb6 | intronic | . | . | . | 2qH1 | rs27386166 | 0 | 1 |
| chr2 | 153902886 | C | T | 202 | Bpifb6 | intronic | . | . | . | 2qH1 | rs29673599 | 0 | 1 |
| chr2 | 153903119 | T | C | 135 | Bpifb6 | intronic | . | . | . | 2qH1 | rs27386165 | 0 | 1 |
| chr2 | 153903881 | G | A | 56 | Bpifb6 | intronic | . | . | . | 2qH1 | rs33000523 | 0 | 1 |
| chr2 | 153903934 | T | C | 221 | Bpifb6 | intronic | . | . | . | 2qH1 | rs33375945 | 0 | 1 |
| chr2 | 153904096 | A | C | 182 | Bpifb6 | intronic | . | . | . | 2qH1 | rs29727725 | 0 | 1 |
| chr2 | 153904407 | G | T | 216 | Bpifb6 | intronic | . | . | . | 2qH1 | rs27386158 | 0 | 1 |
| chr2 | 153904446 | G | A | 222 | Bpifb6 | intronic | . | . | . | 2qH1 | rs27386157 | 0 | 1 |
| chr2 | 153904580 | T | C | 222 | Bpifb6 | exonic | synonymous SNV | Bpifb6:NM_199303:exon6:c.T393C:p.P131P | . | 2qH1 | rs27386156 | 0 | 1 |
| chr2 | 153904650 | G | T | 208 | Bpifb6 | intronic | . | . | . | 2qH1 | rs27386155 | 0 | 1 |
| chr2 | 153904907 | A | G | 221 | Bpifb6 | intronic | . | . | . | 2qH1 | rs27386154 | 0 | 1 |
| chr2 | 153904989 | T | C | 77 | Bpifb6 | intronic | . | . | . | 2qH1 | rs27386153 | 0 | 1 |
| chr2 | 153905079 | T | C | 93 | Bpifb6 | intronic | . | . | . | 2qH1 | rs46451756 | 0 | 1 |
| chr2 | 153905160 | C | G | 129 | Bpifb6 | intronic | . | . | . | 2qH1 | rs51773575 | 0 | 1 |
| chr2 | 153905235 | A | G | 222 | Bpifb6 | intronic | . | . | . | 2qH1 | rs27386149 | 0 | 1 |
| chr2 | 153905408 | T | C | 222 | Bpifb6 | intronic | . | . | . | 2qH1 | rs49889295 | 0 | 1 |
| chr2 | 153905418 | T | C | 216 | Bpifb6 | intronic | . | . | . | 2qH1 | rs27386148 | 0 | 1 |
| chr2 | 153905436 | G | A | 159 | Bpifb6 | intronic | . | . | . | 2qH1 | rs27386147 | 0 | 1 |
| chr2 | 153906052 | C | T | 46.3452 | Bpifb6 | exonic | synonymous SNV | Bpifb6:NM_199303:exon9:c.C630T:p.A210A | . | 2qH1 | rs223325990 | 0 | 1 |
| chr2 | 153906934 | T | C | 33.6128 | Bpifb6 | intronic | . | . | . | 2qH1 | rs49320154 | 0 | 1 |
| chr2 | 153906959 | A | G | 40.1762 | Bpifb6 | intronic | . | . | . | 2qH1 | rs50189116 | 0 | 1 |
| chr2 | 153907889 | T | C | 70 | Bpifb6 | intronic | . | . | . | 2qH1 | rs27386121 | 0 | 1 |
| chr2 | 153908042 | A | G | 139 | Bpifb6 | intronic | . | . | . | 2qH1 | rs27386119 | 0 | 1 |
| chr2 | 153908111 | A | G | 208 | Bpifb6 | exonic | missense SNV | Bpifb6:NM_199303:exon11:c.A862G:p.R288G | . | 2qH1 | rs27386118 | 0 | 1 |
| chr2 | 153908134 | G | A | 204 | Bpifb6 | exonic | synonymous SNV | Bpifb6:NM_199303:exon11:c.G885A:p.P295P | . | 2qH1 | rs27386117 | 0 | 1 |
| chr2 | 153909177 | C | T | 222 | Bpifb6 | exonic | synonymous SNV | Bpifb6:NM_199303:exon13:c.C1080T:p.I360I | . | 2qH1 | rs27386109 | 0 | 1 |
| chr2 | 153909359 | A | C | 84 | Bpifb6 | intronic | . | . | . | 2qH1 | rs27386108 | 0 | 1 |
| chr2 | 153910635 | C | G | 126 | Bpifb6 | intronic | . | . | . | 2qH1 | rs27386094 | 0 | 1 |
| chr2 | 153910653 | A | G | 146 | Bpifb6 | intronic | . | . | . | 2qH1 | rs46448154 | 0 | 1 |
| chr2 | 153911347 | A | C | 129 | Bpifb6 | intronic | . | . | . | 2qH1 | rs27386088 | 0 | 1 |
| chr2 | 153911431 | A | G | 163 | Bpifb6 | intronic | . | . | . | 2qH1 | rs27386087 | 0 | 1 |
| chr2 | 153911548 | A | G | 189 | Bpifb6 | intronic | . | . | . | 2qH1 | rs27386084 | 0 | 1 |
| chr2 | 153911555 | A | T | 193 | Bpifb6 | intronic | . | . | . | 2qH1 | rs225324185 | 0 | 1 |
| chr2 | 153911819 | G | A | 101 | Bpifb6 | intronic | . | . | . | 2qH1 | rs27386083 | 0 | 1 |
| chr2 | 153911970 | G | A | 221 | Bpifb6 | exonic | missense SNV | Bpifb6:NM_199303:exon16:c.G1306A:p.D436N | . | 2qH1 | rs27386082 | 0 | 1 |
| chr2 | 153912126 | A | G | 87 | Bpifb6 | intronic | . | . | . | 2qH1 | rs27386081 | 0 | 1 |
| chr2 | 153912499 | T | C | 136 | Bpifb6 | intronic | . | . | . | 2qH1 | rs27386076 | 0 | 1 |
| chr2 | 153912616 | A | G | 221 | Bpifb6 | exonic | synonymous SNV | Bpifb6:NM_199303:exon17:c.A1344G:p.T448T | . | 2qH1 | rs27386071 | 0 | 1 |
| chr2 | 153919654 | C | T | 179 | Bpifb3 | exonic | synonymous SNV | Bpifb3:NM_194357:exon2:c.C22T:p.L8L | . | 2qH1 | rs27370664 | 0 | 1 |
| chr2 | 153920337 | C | T | 70 | Bpifb3 | intronic | . | . | . | 2qH1 | rs50876425 | 0 | 1 |
| chr2 | 153922260 | C | T | 222 | Bpifb3 | intronic | . | . | . | 2qH1 | rs27370647 | 0 | 1 |
| chr2 | 153922432 | G | A | 76 | Bpifb3 | intronic | . | . | . | 2qH1 | rs238001252 | 0 | 1 |
| chr2 | 153923811 | T | C | 168 | Bpifb3 | intronic | . | . | . | 2qH1 | rs50774765 | 0 | 1 |
| chr2 | 153923974 | A | G | 164 | Bpifb3 | intronic | . | . | . | 2qH1 | rs578416637 | 0 | 1 |
| chr2 | 153923986 | G | C | 155 | Bpifb3 | intronic | . | . | . | 2qH1 | rs27370617 | 0 | 1 |
| chr2 | 153924860 | C | T | 222 | Bpifb3 | intronic | . | . | . | 2qH1 | rs27370610 | 0 | 1 |
| chr2 | 153927200 | G | A | 86 | Bpifb3 | intronic | . | . | . | 2qH1 | rs47135306 | 0 | 1 |
| chr2 | 153927203 | A | G | 66 | Bpifb3 | intronic | . | . | . | 2qH1 | rs51305983 | 0 | 1 |
| chr2 | 153927383 | T | A | 209 | Bpifb3 | intronic | . | . | . | 2qH1 | rs27370582 | 0 | 1 |
| chr2 | 153927494 | G | T | 83 | Bpifb3 | intronic | . | . | . | 2qH1 | rs27370580 | 0 | 1 |
| chr2 | 153929140 | C | T | 115 | Bpifb3 | intronic | . | . | . | 2qH1 | rs27370563 | 0 | 1 |
| chr2 | 153929239 | T | C | 179 | Bpifb3 | intronic | . | . | . | 2qH1 | rs226002820 | 0 | 1 |
| chr2 | 153929410 | C | A | 135 | Bpifb3 | intronic | . | . | . | 2qH1 | rs50251085 | 0 | 1 |
| chr2 | 153929421 | T | A | 85 | Bpifb3 | intronic | . | . | . | 2qH1 | rs45845899 | 0 | 1 |
| chr2 | 153930628 | A | G | 175 | Bpifb3 | intronic | . | . | . | 2qH1 | rs27370546 | 0 | 1 |
| chr2 | 153931250 | A | C | 23.5475 | Bpifb3 | intronic | . | . | . | 2qH1 | rs27370538 | 0 | 1 |
| chr2 | 153931497 | A | G | 202 | Bpifb3 | intronic | . | . | . | 2qH1 | rs27370534 | 0 | 1 |
| chr2 | 153931638 | T | G | 26.2432 | Bpifb3 | intronic | . | . | . | 2qH1 | rs27370530 | 0 | 1 |
| chr2 | 153931728 | T | C | 51 | Bpifb3 | intronic | . | . | . | 2qH1 | rs27370529 | 0 | 1 |
| chr2 | 153931827 | G | A | 196 | Bpifb3 | intronic | . | . | . | 2qH1 | rs27370528 | 0 | 1 |
| chr2 | 153932002 | G | A | 221 | Bpifb3 | intronic | . | . | . | 2qH1 | rs27370527 | 0 | 1 |
| chr2 | 153932738 | G | A | 35.4278 | Bpifb3 | UTR3 | . | . | . | 2qH1 | rs27370521 | 0 | 1 |
| chr2 | 153932825 | G | A | 53 | Bpifb3 | UTR3 | . | . | . | 2qH1 | rs27370520 | 0 | 1 |
| chr2 | 153940849 | A | C | 77 | . | upstream | . | . | . | 2qH1 | rs27370448 | 0 | 1 |
| chr2 | 153940866 | G | T | 103 | Bpifb4 | UTR5 | . | . | . | 2qH1 | rs27370447 | 0 | 1 |
| chr2 | 153940909 | A | T | 175 | Bpifb4 | exonic | synonymous SNV | Bpifb4:NM_001034875:exon1:c.A33T:p.S11S | . | 2qH1 | rs27370445 | 0 | 1 |
| chr2 | 153940957 | G | A | 167 | Bpifb4 | exonic | synonymous SNV | Bpifb4:NM_001034875:exon1:c.G81A:p.R27R | . | 2qH1 | rs27370444 | 0 | 1 |
| chr2 | 153941085 | A | G | 99 | Bpifb4 | intronic | . | . | . | 2qH1 | rs50604670 | 0 | 1 |
| chr2 | 153941110 | C | T | 57 | Bpifb4 | intronic | . | . | . | 2qH1 | rs27370442 | 0 | 1 |
| chr2 | 153941132 | T | C | 29.5712 | Bpifb4 | intronic | . | . | . | 2qH1 | rs27370441 | 0 | 1 |
| chr2 | 153941884 | C | T | 49.9668 | Bpifb4 | exonic | synonymous SNV | Bpifb4:NM_001034875:exon2:c.C147T:p.S49S | . | 2qH1 | rs27370429 | 0 | 1 |
| chr2 | 153942644 | A | G | 221 | Bpifb4 | exonic | missense SNV | Bpifb4:NM_001034875:exon3:c.A271G:p.K91E | . | 2qH1 | rs27370427 | 0 | 1 |
| chr2 | 153942658 | T | C | 221 | Bpifb4 | exonic | synonymous SNV | Bpifb4:NM_001034875:exon3:c.T285C:p.N95N | . | 2qH1 | rs27370426 | 0 | 1 |
| chr2 | 153942955 | T | A | 208 | Bpifb4 | exonic | synonymous SNV | Bpifb4:NM_001034875:exon3:c.T582A:p.G194G | . | 2qH1 | rs33417885 | 0 | 1 |
| chr2 | 153942988 | T | C | 221 | Bpifb4 | exonic | synonymous SNV | Bpifb4:NM_001034875:exon3:c.T615C:p.L205L | . | 2qH1 | rs27370425 | 0 | 1 |
| chr2 | 153943119 | C | T | 222 | Bpifb4 | intronic | . | . | . | 2qH1 | rs27370424 | 0 | 1 |
| chr2 | 153943859 | A | G | 53 | Bpifb4 | intronic | . | . | . | 2qH1 | rs27370419 | 0 | 1 |
| chr2 | 153944113 | A | G | 222 | Bpifb4 | intronic | . | . | . | 2qH1 | rs27370416 | 0 | 1 |
| chr2 | 153944147 | A | G | 221 | Bpifb4 | intronic | . | . | . | 2qH1 | rs27370415 | 0 | 1 |
| chr2 | 153944662 | G | C | 25.0376 | Bpifb4 | intronic | . | . | . | 2qH1 | rs27370404 | 0 | 1 |
| chr2 | 153944742 | A | G | 87 | Bpifb4 | exonic | synonymous SNV | Bpifb4:NM_001034875:exon5:c.A813G:p.A271A | . | 2qH1 | rs27370403 | 0 | 1 |
| chr2 | 153944869 | T | C | 169 | Bpifb4 | intronic | . | . | . | 2qH1 | rs46083645 | 0 | 1 |
| chr2 | 153947126 | T | A | 163 | Bpifb4 | intronic | . | . | . | 2qH1 | rs27370376 | 0 | 1 |
| chr2 | 153947255 | T | C | 221 | Bpifb4 | exonic | synonymous SNV | Bpifb4:NM_001034875:exon6:c.T981C:p.N327N | . | 2qH1 | rs27370375 | 0 | 1 |
| chr2 | 153947312 | G | A | 221 | Bpifb4 | intronic | . | . | . | 2qH1 | rs48865795 | 0 | 1 |
| chr2 | 153947585 | G | A | 93 | Bpifb4 | intronic | . | . | . | 2qH1 | rs27370368 | 0 | 1 |
| chr2 | 153947598 | G | A | 91 | Bpifb4 | intronic | . | . | . | 2qH1 | rs52031591 | 0 | 1 |
| chr2 | 153947692 | C | T | 138 | Bpifb4 | intronic | . | . | . | 2qH1 | rs27348166 | 0 | 1 |
| chr2 | 153947744 | A | C | 140 | Bpifb4 | intronic | . | . | . | 2qH1 | rs49878600 | 0 | 1 |
| chr2 | 153948550 | C | T | 176 | Bpifb4 | intronic | . | . | . | 2qH1 | rs49370026 | 0 | 1 |
| chr2 | 153948577 | T | C | 221 | Bpifb4 | intronic | . | . | . | 2qH1 | rs27348154 | 0 | 1 |
| chr2 | 153948697 | T | C | 222 | Bpifb4 | exonic | synonymous SNV | Bpifb4:NM_001034875:exon8:c.T1149C:p.N383N | . | 2qH1 | rs27348153 | 0 | 1 |
| chr2 | 153948854 | A | T | 43.9904 | Bpifb4 | intronic | . | . | . | 2qH1 | rs27348152 | 0 | 1 |
| chr2 | 153950126 | T | C | 74 | Bpifb4 | intronic | . | . | . | 2qH1 | rs27348136 | 0 | 1 |
| chr2 | 153950139 | T | C | 51 | Bpifb4 | intronic | . | . | . | 2qH1 | rs50164444 | 0 | 1 |
| chr2 | 153950315 | A | G | 222 | Bpifb4 | exonic | synonymous SNV | Bpifb4:NM_001034875:exon9:c.A1278G:p.S426S | . | 2qH1 | rs27348134 | 0 | 1 |
| chr2 | 153950405 | C | T | 222 | Bpifb4 | intronic | . | . | . | 2qH1 | rs27348133 | 0 | 1 |
| chr2 | 153952016 | A | G | 91 | Bpifb4 | intronic | . | . | . | 2qH1 | rs27348114 | 0 | 1 |
| chr2 | 153952066 | T | C | 186 | Bpifb4 | intronic | . | . | . | 2qH1 | rs27348113 | 0 | 1 |
| chr2 | 153952188 | G | A | 222 | Bpifb4 | intronic | . | . | . | 2qH1 | rs27348112 | 0 | 1 |
| chr2 | 153952255 | T | C | 156 | Bpifb4 | intronic | . | . | . | 2qH1 | rs27348111 | 0 | 1 |
| chr2 | 153954451 | G | A | 63 | Bpifb4 | intronic | . | . | . | 2qH1 | rs51554674 | 0 | 1 |
| chr2 | 153957063 | A | G | 35.4108 | Bpifb4 | intronic | . | . | . | 2qH1 | rs27348077 | 0 | 1 |
| chr2 | 153957746 | C | T | 170 | Bpifb4 | exonic | synonymous SNV | Bpifb4:NM_001034875:exon13:c.C1674T:p.N558N | . | 2qH1 | rs27348076 | 0 | 1 |
| chr2 | 153959494 | C | T | 51 | Bpifb4 | intronic | . | . | . | 2qH1 | rs29628061 | 0 | 1 |
| chr2 | 153959540 | C | T | 91 | Bpifb4 | intronic | . | . | . | 2qH1 | rs27348062 | 0 | 1 |
| chr2 | 153959565 | T | C | 115 | Bpifb4 | intronic | . | . | . | 2qH1 | rs27348061 | 0 | 1 |
| chr2 | 153959720 | T | C | 222 | Bpifb4 | intronic | . | . | . | 2qH1 | rs27348060 | 0 | 1 |
| chr2 | 153959771 | G | T | 222 | Bpifb4 | intronic | . | . | . | 2qH1 | rs27348059 | 0 | 1 |
| chr2 | 153961502 | C | T | 221 | Bpifb4 | intronic | . | . | . | 2qH1 | rs47278361 | 0 | 1 |
| chr2 | 153961574 | A | G | 216 | Bpifb4 | exonic | synonymous SNV | Bpifb4:NM_001034875:exon15:c.A1812G:p.A604A | . | 2qH1 | rs27348048 | 0 | 1 |
| chr2 | 153961612 | A | G | 221 | Bpifb4 | intronic | . | . | . | 2qH1 | rs27348047 | 0 | 1 |
| chr2 | 153963486 | C | T | 202 | Bpifb4 | intronic | . | . | . | 2qH1 | rs47441125 | 0 | 1 |
| chr2 | 153963504 | C | T | 211 | Bpifb4 | intronic | . | . | . | 2qH1 | rs47509052 | 0 | 1 |
| chr2 | 153963562 | G | T | 222 | Bpifb4 | intronic | . | . | . | 2qH1 | rs27348036 | 0 | 1 |
| chr2 | 153963670 | G | A | 185 | Bpifb4 | UTR3 | . | . | . | 2qH1 | rs27348035 | 0 | 1 |
| chr2 | 153963690 | C | A | 135 | Bpifb4 | UTR3 | . | . | . | 2qH1 | rs27348034 | 0 | 1 |
| chr2 | 153963728 | C | T | 62 | Bpifb4 | UTR3 | . | . | . | 2qH1 | rs49650473 | 0 | 1 |
| chr2 | 153983007 | G | A | 120 | Bpifa6 | intronic | . | . | . | 2qH1 | rs46380249 | 0 | 1 |
| chr2 | 153987087 | T | C | 222 | Bpifa6 | intronic | . | . | . | 2qH1 | rs48433657 | 0 | 1 |
| chr2 | 153989159 | C | T | 65 | Bpifa6 | intronic | . | . | . | 2qH1 | rs211728333 | 0 | 1 |
| chr2 | 153989196 | C | T | 167 | Bpifa6 | intronic | . | . | . | 2qH1 | rs235002184 | 0 | 1 |
| chr2 | 153989214 | A | G | 170 | Bpifa6 | exonic | synonymous SNV | Bpifa6:NM_001080811:exon6:c.A699G:p.S233S | . | 2qH1 | rs252452208 | 0 | 1 |
| chr2 | 153989327 | G | A | 221 | Bpifa6 | exonic | missense SNV | Bpifa6:NM_001080811:exon6:c.G812A:p.G271E | . | 2qH1 | rs222187722 | 0 | 1 |
| chr2 | 153989363 | A | G | 221 | Bpifa6 | intronic | . | . | . | 2qH1 | rs50942189 | 0 | 1 |
| chr2 | 153990451 | A | G | 138 | Bpifa6 | exonic | missense SNV | Bpifa6:NM_001080811:exon7:c.A877G:p.I293V | . | 2qH1 | rs50860100 | 0 | 1 |
| chr2 | 153990472 | A | G | 78 | Bpifa6 | splicing | . | . | . | 2qH1 | rs48950058 | 0 | 1 |
| chr2 | 153990974 | G | A | 221 | Bpifa6 | intronic | . | . | . | 2qH1 | rs30625688 | 0 | 1 |
| chr2 | 153991057 | G | A | 72 | Bpifa6 | intronic | . | . | . | 2qH1 | rs30826602 | 0 | 1 |
| chr2 | 153992204 | T | C | 155 | Bpifa6 | intronic | . | . | . | 2qH1 | rs48706447 | 0 | 1 |
| chr2 | 153992249 | C | T | 150 | Bpifa6 | exonic | synonymous SNV | Bpifa6:NM_001080811:exon9:c.C960T:p.I320I | . | 2qH1 | rs48396293 | 0 | 1 |
| chr2 | 153992287 | A | G | 92 | Bpifa6 | exonic | missense SNV | Bpifa6:NM_001080811:exon9:c.A998G:p.D333G | . | 2qH1 | rs48245999 | 0 | 1 |
| chr2 | 153992304 | C | T | 93 | Bpifa6 | exonic | synonymous SNV | Bpifa6:NM_001080811:exon9:c.C1015T:p.L339L | . | 2qH1 | rs46539585 | 0 | 1 |
| chr2 | 153992376 | G | C | 71 | Bpifa6 | intronic | . | . | . | 2qH1 | rs49216760 | 0 | 1 |
| chr2 | 153992377 | T | G | 73 | Bpifa6 | intronic | . | . | . | 2qH1 | rs27347567 | 0 | 1 |
| chr2 | 153993266 | C | T | 88 | Bpifa6 | intronic | . | . | . | 2qH1 | rs30528998 | 0 | 1 |
| chr2 | 153993272 | T | C | 84 | Bpifa6 | intronic | . | . | . | 2qH1 | rs30930364 | 0 | 1 |
| chr2 | 153999928 | T | C | 64 | Bpifa6 | intronic | . | . | . | 2qH1 | rs47164509 | 0 | 1 |
| chr2 | 153999945 | T | A | 95 | Bpifa6 | intronic | . | . | . | 2qH1 | rs48558962 | 0 | 1 |
| chr2 | 153999949 | T | C | 115 | Bpifa6 | intronic | . | . | . | 2qH1 | rs49475765 | 0 | 1 |
| chr2 | 154000100 | C | T | 222 | Bpifa6 | UTR3 | . | . | . | 2qH1 | rs27347554 | 0 | 1 |
| chr2 | 154000310 | C | T | 37.7106 | Bpifa6 | UTR3 | . | . | . | 2qH1 | rs27347552 | 0 | 1 |
| chr2 | 154009041 | C | T | 114 | Bpifa2 | intronic | . | . | . | 2qH1 | rs51839823 | 0 | 1 |
| chr2 | 154009045 | C | T | 118 | Bpifa2 | intronic | . | . | . | 2qH1 | rs49198158 | 0 | 1 |
| chr2 | 154009258 | T | C | 222 | Bpifa2 | intronic | . | . | . | 2qH1 | rs27347478 | 0 | 1 |
| chr2 | 154009546 | G | T | 44.3783 | Bpifa2 | intronic | . | . | . | 2qH1 | rs27347477 | 0 | 1 |
| chr2 | 154009735 | A | G | 222 | Bpifa2 | intronic | . | . | . | 2qH1 | rs27347476 | 0 | 1 |
| chr2 | 154009967 | T | C | 222 | Bpifa2 | intronic | . | . | . | 2qH1 | rs27347475 | 0 | 1 |
| chr2 | 154010538 | T | G | 222 | Bpifa2 | exonic | missense SNV | Bpifa2:NM_008953:exon4:c.T333G:p.D111E | . | 2qH1 | rs13476849 | 0 | 1 |
| chr2 | 154010759 | T | C | 129 | Bpifa2 | intronic | . | . | . | 2qH1 | rs33003613 | 0 | 1 |
| chr2 | 154011188 | A | G | 35.9628 | Bpifa2 | intronic | . | . | . | 2qH1 | rs32990183 | 0 | 1 |
| chr2 | 154011367 | C | T | 121 | Bpifa2 | exonic | missense SNV | Bpifa2:NM_008953:exon5:c.C389T:p.T130M | . | 2qH1 | rs27347468 | 0 | 1 |
| chr2 | 154011375 | A | G | 124 | Bpifa2 | exonic | missense SNV | Bpifa2:NM_008953:exon5:c.A397G:p.I133V | . | 2qH1 | rs27347466 | 0 | 1 |
| chr2 | 154011410 | C | T | 97 | Bpifa2 | exonic | synonymous SNV | Bpifa2:NM_008953:exon5:c.C432T:p.S144S | . | 2qH1 | rs27347465 | 0 | 1 |
| chr2 | 154014317 | C | T | 73 | Bpifa2 | intronic | . | . | . | 2qH1 | rs51144964 | 0 | 1 |
| chr2 | 154014331 | A | G | 76 | Bpifa2 | intronic | . | . | . | 2qH1 | rs27347442 | 0 | 1 |
| chr2 | 154014444 | A | G | 175 | Bpifa2 | exonic | missense SNV | Bpifa2:NM_008953:exon7:c.A634G:p.S212G | . | 2qH1 | rs27347441 | 0 | 1 |
| chr2 | 154014460 | G | A | 217 | Bpifa2 | exonic | missense SNV | Bpifa2:NM_008953:exon7:c.G650A:p.S217N | . | 2qH1 | rs27347440 | 0 | 1 |
| chr2 | 154014598 | C | T | 222 | Bpifa2 | intronic | . | . | . | 2qH1 | rs27347439 | 0 | 1 |
| chr2 | 154014706 | C | A | 155 | Bpifa2 | intronic | . | . | . | 2qH1 | rs27347437 | 0 | 1 |
| chr2 | 154014745 | C | T | 119 | Bpifa2 | intronic | . | . | . | 2qH1 | rs27347436 | 0 | 1 |
| chr2 | 154014856 | G | T | 198 | Bpifa2 | intronic | . | . | . | 2qH1 | rs46666504 | 0 | 1 |
| chr2 | 154086297 | A | G | 43.7295 | . | intergenic | . | . | . | 2qH1 | rs46550681 | 0 | 1 |

| | | | | | | | | | | | | | |
|---|---|---|---|---|---|---|---|---|---|---|---|---|---|
| chr2 | 154086347 | A | G | 117 | . | intergenic | . | . | . | 2qH1 | rs50544389 | 0 | 1 |
| chr2 | 154086490 | A | G | 221 | . | intergenic | . | . | . | 2qH1 | rs27331050 | 0 | 1 |
| chr2 | 154086519 | T | C | 149 | . | intergenic | . | . | . | 2qH1 | rs45772696 | 0 | 1 |
| chr2 | 154086835 | A | G | 101 | . | intergenic | . | . | . | 2qH1 | rs49936781 | 0 | 1 |
| chr2 | 154086866 | G | A | 105 | . | intergenic | . | . | . | 2qH1 | rs48723936 | 0 | 1 |
| chr2 | 154086935 | T | C | 189 | . | intergenic | . | . | . | 2qH1 | rs49383216 | 0 | 1 |
| chr2 | 154086978 | A | G | 147 | . | intergenic | . | . | . | 2qH1 | rs47652876 | 0 | 1 |
| chr2 | 154087073 | C | T | 108 | . | intergenic | . | . | . | 2qH1 | rs108202860 | 0 | 1 |
| chr2 | 154087177 | A | G | 110 | . | intergenic | . | . | . | 2qH1 | rs49410878 | 0 | 1 |
| chr2 | 154087300 | G | C | 59 | . | intergenic | . | . | . | 2qH1 | rs47700635 | 0 | 1 |
| chr2 | 154087312 | G | A | 25.6137 | . | intergenic | . | . | . | 2qH1 | rs50461438 | 0 | 1 |
| chr2 | 154087609 | C | G | 53 | . | intergenic | . | . | . | 2qH1 | rs27331045 | 0 | 1 |
| chr2 | 154087678 | C | A | 76 | . | intergenic | . | . | . | 2qH1 | rs27331042 | 0 | 1 |
| chr2 | 154130562 | T | C | 125 | Bpifa3 | exonic | synonymous SNV | Bpifa3:NM_001291079:exon1:c.T27C:p.V9V,Bpifa3:NM_028528:exon1:c.T27C:p.V9V | . | 2qH1 | rs27330925 | 0 | 1 |
| chr2 | 154130691 | A | G | 167 | Bpifa3 | intronic | . | . | . | 2qH1 | rs49880745 | 0 | 1 |
| chr2 | 154137490 | G | A | 164 | Bpifa3 | intronic | . | . | . | 2qH1 | rs32987618 | 0 | 1 |
| chr2 | 154137803 | T | C | 104 | Bpifa3 | intronic | . | . | . | 2qH1 | rs27330881 | 0 | 1 |
| chr2 | 154144470 | T | C | 73 | Bpifa1 | intronic | . | . | . | 2qH1 | rs3673766 | 0 | 1 |
| chr2 | 154145484 | T | C | 63 | Bpifa1 | intronic | . | . | . | 2qH1 | rs27330828 | 0 | 1 |
| chr2 | 154145541 | G | A | 57 | Bpifa1 | intronic | . | . | . | 2qH1 | rs27330827 | 0 | 1 |
| chr2 | 154145688 | C | A | 221 | Bpifa1 | exonic | missense SNV | Bpifa1:NM_011126:exon4:c.C457A:p.R153S | . | 2qH1 | . | 0 | 1 |
| chr2 | 154146087 | T | C | 221 | Bpifa1 | exonic | synonymous SNV | Bpifa1:NM_011126:exon5:c.T549C:p.I183I | . | 2qH1 | rs27330822 | 0 | 1 |
| chr2 | 154146328 | A | G | 28.9041 | Bpifa1 | intronic | . | . | . | 2qH1 | rs27330819 | 0 | 1 |
| chr2 | 154147502 | T | C | 222 | Bpifa1 | intronic | . | . | . | 2qH1 | rs27330809 | 0 | 1 |
| chr2 | 154148093 | A | G | 221 | Bpifa1 | UTR3 | . | . | . | 2qH1 | rs27330808 | 0 | 1 |
| chr2 | 154163459 | C | T | 94 | Bpifa5 | intronic | . | . | . | 2qH1 | rs33230884 | 0 | 1 |
| chr2 | 154163741 | A | G | 144 | Bpifa5 | intronic | . | . | . | 2qH1 | rs50022833 | 0 | 1 |
| chr2 | 154163754 | T | C | 106 | Bpifa5 | intronic | . | . | . | 2qH1 | rs51263672 | 0 | 1 |
| chr2 | 154164289 | T | C | 221 | Bpifa5 | intronic | . | . | . | 2qH1 | rs33556316 | 0 | 1 |
| chr2 | 154164398 | T | C | 21.4091 | Bpifa5 | intronic | . | . | . | 2qH1 | rs29959167 | 0 | 1 |
| chr2 | 154165573 | G | A | 221 | Bpifa5 | exonic | missense SNV | Bpifa5:NM_025990:exon4:c.G434A:p.R145H | . | 2qH1 | rs49604819 | 0 | 1 |
| chr2 | 154165706 | C | A | 222 | Bpifa5 | intronic | . | . | . | 2qH1 | . | 0 | 1 |
| chr2 | 154165707 | T | A | 222 | Bpifa5 | intronic | . | . | . | 2qH1 | . | 0 | 1 |
| chr2 | 154166151 | T | C | 202 | Bpifa5 | intronic | . | . | . | 2qH1 | rs52223281 | 0 | 1 |
| chr2 | 154166738 | A | G | 107 | Bpifa5 | intronic | . | . | . | 2qH1 | rs51113479 | 0 | 1 |
| chr2 | 154166765 | G | A | 122 | Bpifa5 | intronic | . | . | . | 2qH1 | rs46300747 | 0 | 1 |
| chr2 | 154167110 | A | C | 219 | Bpifa5 | intronic | . | . | . | 2qH1 | rs49619222 | 0 | 1 |
| chr2 | 154167366 | A | G | 209 | Bpifa5 | intronic | . | . | . | 2qH1 | rs49924818 | 0 | 1 |
| chr2 | 154202569 | A | G | 21.0962 | Bpifb1 | splicing | . | . | . | 2qH1 | rs27315650 | 0 | 1 |
| chr2 | 154202680 | C | T | 50 | Bpifb1 | exonic | synonymous SNV | Bpifb1:NM_001012392:exon2:c.C66T:p.N22N,Bpifb1:NM_153418:exon2:c.C66T:p.N22N | . | 2qH1 | rs50032035 | 0 | 1 |
| chr2 | 154202682 | T | G | 36.8653 | Bpifb1 | exonic | missense SNV | Bpifb1:NM_001012392:exon2:c.T68G:p.V23G,Bpifb1:NM_153418:exon2:c.T68G:p.V23G | . | 2qH1 | rs48926494 | 0 | 1 |
| chr2 | 154204662 | A | C | 94 | Bpifb1 | intronic | . | . | . | 2qH1 | rs234162671 | 0 | 1 |
| chr2 | 154204803 | G | A | 222 | Bpifb1 | exonic | missense SNV | Bpifb1:NM_001012392:exon3:c.G242A:p.R81K,Bpifb1:NM_153418:exon3:c.G242A:p.R81K | . | 2qH1 | rs47357495 | 0 | 1 |
| chr2 | 154204895 | T | C | 103 | Bpifb1 | intronic | . | . | . | 2qH1 | rs47080039 | 0 | 1 |
| chr2 | 154205406 | G | C | 74 | Bpifb1 | intronic | . | . | . | 2qH1 | rs51214342 | 0 | 1 |
| chr2 | 154205474 | G | A | 20.2128 | Bpifb1 | exonic | missense SNV | Bpifb1:NM_001012392:exon4:c.G310A:p.D104N,Bpifb1:NM_153418:exon4:c.G310A:p.D104N | . | 2qH1 | rs50108791 | 0 | 1 |
| chr2 | 154206366 | C | A | 174 | Bpifb1 | intronic | . | . | . | 2qH1 | rs48331467 | 0 | 1 |
| chr2 | 154206403 | C | T | 222 | Bpifb1 | intronic | . | . | . | 2qH1 | rs51797828 | 0 | 1 |
| chr2 | 154206990 | A | C | 67 | Bpifb1 | intronic | . | . | . | 2qH1 | rs33640825 | 0 | 1 |
| chr2 | 154207280 | G | A | 88 | Bpifb1 | intronic | . | . | . | 2qH1 | rs33475301 | 0 | 1 |
| chr2 | 154209873 | G | A | 192 | Bpifb1 | exonic | synonymous SNV | Bpifb1:NM_001012392:exon7:c.G597A:p.L199L,Bpifb1:NM_153418:exon7:c.G597A:p.L199L | . | 2qH1 | rs47267579 | 0 | 1 |
| chr2 | 154209880 | G | A | 199 | Bpifb1 | exonic | missense SNV | Bpifb1:NM_001012392:exon7:c.G604A:p.V202M,Bpifb1:NM_153418:exon7:c.G604A:p.V202M | . | 2qH1 | rs49812513 | 0 | 1 |
| chr2 | 154211456 | C | T | 98 | Bpifb1 | intronic | . | . | . | 2qH1 | rs47353952 | 0 | 1 |
| chr2 | 154211514 | T | C | 125 | Bpifb1 | exonic | synonymous SNV | Bpifb1:NM_001012392:exon8:c.T675C:p.S225S,Bpifb1:NM_153418:exon8:c.T675C:p.S225S | . | 2qH1 | rs48230176 | 0 | 1 |
| chr2 | 154211524 | C | T | 104 | Bpifb1 | exonic | synonymous SNV | Bpifb1:NM_001012392:exon8:c.C685T:p.L229L,Bpifb1:NM_153418:exon8:c.C685T:p.L229L | . | 2qH1 | rs45721171 | 0 | 1 |
| chr2 | 154211577 | C | T | 28.0836 | Bpifb1 | exonic | synonymous SNV | Bpifb1:NM_001012392:exon8:c.C738T:p.N246N,Bpifb1:NM_153418:exon8:c.C738T:p.N246N | . | 2qH1 | rs48751701 | 0 | 1 |
| chr2 | 154213015 | A | G | 194 | Bpifb1 | exonic | missense SNV | Bpifb1:NM_001012392:exon9:c.A826G:p.R276G,Bpifb1:NM_153418:exon9:c.A826G:p.R276G | . | 2qH1 | rs33521708 | 0 | 1 |
| chr2 | 154213025 | T | G | 140 | Bpifb1 | exonic | missense SNV | Bpifb1:NM_001012392:exon9:c.T836G:p.F279C,Bpifb1:NM_153418:exon9:c.T836G:p.F279C | . | 2qH1 | . | 0 | 1 |
| chr2 | 154213163 | G | A | 30.2394 | Bpifb1 | intronic | . | . | . | 2qH1 | rs32914730 | 0 | 1 |
| chr2 | 154213273 | C | T | 78 | Bpifb1 | intronic | . | . | . | 2qH1 | rs32828479 | 0 | 1 |
| chr2 | 154213322 | C | T | 106 | Bpifb1 | intronic | . | . | . | 2qH1 | rs33213248 | 0 | 1 |
| chr2 | 154213724 | G | C | 50 | Bpifb1 | intronic | . | . | . | 2qH1 | rs29523273 | 0 | 1 |
| chr2 | 154213972 | T | G | 126 | Bpifb1 | intronic | . | . | . | 2qH1 | rs33646174 | 0 | 1 |
| chr2 | 154214065 | C | T | 219 | Bpifb1 | exonic | synonymous SNV | Bpifb1:NM_001012392:exon11:c.C1050T:p.N350N,Bpifb1:NM_153418:exon11:c.C1050T:p.N350N | . | 2qH1 | rs33605191 | 0 | 1 |
| chr2 | 154214095 | C | T | 221 | Bpifb1 | exonic | synonymous SNV | Bpifb1:NM_001012392:exon11:c.C1080T:p.V360V,Bpifb1:NM_153418:exon11:c.C1080T:p.V360V | . | 2qH1 | rs33084263 | 0 | 1 |
| chr2 | 154214272 | A | G | 178 | Bpifb1 | intronic | . | . | . | 2qH1 | rs27315639 | 0 | 1 |
| chr2 | 154214273 | A | G | 179 | Bpifb1 | intronic | . | . | . | 2qH1 | rs262757059 | 0 | 1 |
| chr2 | 154215222 | C | T | 222 | Bpifb1 | intronic | . | . | . | 2qH1 | rs27315624 | 0 | 1 |
| chr2 | 154215323 | C | T | 222 | Bpifb1 | exonic | synonymous SNV | Bpifb1:NM_001012392:exon12:c.C1200T:p.N400N,Bpifb1:NM_153418:exon12:c.C1200T:p.N400N | . | 2qH1 | rs27315623 | 0 | 1 |
| chr2 | 154215424 | C | G | 222 | Bpifb1 | intronic | . | . | . | 2qH1 | rs27315622 | 0 | 1 |
| chr2 | 154215519 | G | A | 96 | Bpifb1 | intronic | . | . | . | 2qH1 | rs27315621 | 0 | 1 |
| chr2 | 154216334 | A | G | 150 | Bpifb1 | intronic | . | . | . | 2qH1 | rs27315614 | 0 | 1 |
| chr2 | 154216373 | A | G | 127 | Bpifb1 | intronic | . | . | . | 2qH1 | rs33621572 | 0 | 1 |
| chr2 | 154218221 | G | A | 221 | Bpifb1 | exonic | missense SNV | Bpifb1:NM_001012392:exon15:c.G1333A:p.V445I,Bpifb1:NM_153418:exon15:c.G1333A:p.V445I | . | 2qH1 | rs27315607 | 0 | 1 |
| chr2 | 154218287 | G | A | 221 | Bpifb1 | intronic | . | . | . | 2qH1 | rs45654209 | 0 | 1 |
| chr2 | 154220299 | A | G | 124 | Bpifb1 | UTR3 | . | . | . | 2qH1 | rs27315598 | 0 | 1 |
| chr2 | 154223749 | C | T | 87 | Bpifb5 | UTR5 | . | . | . | 2qH1 | rs52044586 | 0 | 1 |
| chr2 | 154223758 | A | G | 84 | Bpifb5 | UTR5 | . | . | . | 2qH1 | rs51271497 | 0 | 1 |
| chr2 | 154223811 | A | T | 74 | Bpifb5 | exonic | missense SNV | Bpifb5:NM_144890:exon1:c.A27T:p.L9F | . | 2qH1 | rs27315572 | 0 | 1 |
| chr2 | 154225059 | C | T | 213 | Bpifb5 | exonic | synonymous SNV | Bpifb5:NM_144890:exon2:c.C231T:p.L77L | . | 2qH1 | rs27315564 | 0 | 1 |
| chr2 | 154225074 | T | C | 221 | Bpifb5 | exonic | synonymous SNV | Bpifb5:NM_144890:exon2:c.T246C:p.S82S | . | 2qH1 | rs27315563 | 0 | 1 |
| chr2 | 154227029 | G | A | 138 | Bpifb5 | intronic | . | . | . | 2qH1 | rs46997770 | 0 | 1 |
| chr2 | 154227240 | A | C | 122 | Bpifb5 | intronic | . | . | . | 2qH1 | rs48726753 | 0 | 1 |
| chr2 | 154228181 | A | G | 221 | Bpifb5 | exonic | missense SNV | Bpifb5:NM_144890:exon4:c.A550G:p.K184E | . | 2qH1 | rs48377483 | 0 | 1 |
| chr2 | 154228235 | C | A | 165 | Bpifb5 | intronic | . | . | . | 2qH1 | rs48589570 | 0 | 1 |
| chr2 | 154228255 | G | A | 98 | Bpifb5 | intronic | . | . | . | 2qH1 | rs48489616 | 0 | 1 |
| chr2 | 154228847 | C | T | 132 | Bpifb5 | intronic | . | . | . | 2qH1 | rs51966787 | 0 | 1 |
| chr2 | 154228859 | A | C | 160 | Bpifb5 | exonic | missense SNV | Bpifb5:NM_144890:exon5:c.A569C:p.D190A | . | 2qH1 | rs224781992 | 0 | 1 |
| chr2 | 154229240 | G | A | 116 | Bpifb5 | intronic | . | . | . | 2qH1 | rs48179486 | 0 | 1 |
| chr2 | 154229424 | C | T | 201 | Bpifb5 | intronic | . | . | . | 2qH1 | rs51720349 | 0 | 1 |
| chr2 | 154229551 | C | A | 221 | Bpifb5 | exonic | missense SNV | Bpifb5:NM_144890:exon7:c.C797A:p.A266E | . | 2qH1 | rs49705238 | 0 | 1 |
| chr2 | 154229690 | G | A | 87 | Bpifb5 | intronic | . | . | . | 2qH1 | rs52250229 | 0 | 1 |
| chr2 | 154229780 | C | A | 32.9694 | Bpifb5 | intronic | . | . | . | 2qH1 | rs50389402 | 0 | 1 |
| chr2 | 154232725 | A | T | 117 | Bpifb5 | intronic | . | . | . | 2qH1 | rs51733828 | 0 | 1 |
| chr2 | 154232803 | A | G | 143 | Bpifb5 | intronic | . | . | . | 2qH1 | rs46231243 | 0 | 1 |
| chr2 | 154232817 | C | T | 128 | Bpifb5 | intronic | . | . | . | 2qH1 | rs49380831 | 0 | 1 |
| chr2 | 154232881 | A | G | 133 | Bpifb5 | exonic | synonymous SNV | Bpifb5:NM_144890:exon9:c.A1029G:p.Q343Q | . | 2qH1 | rs49619370 | 0 | 1 |
| chr2 | 154232994 | C | T | 29.8266 | Bpifb5 | intronic | . | . | . | 2qH1 | rs45965104 | 0 | 1 |
| chr2 | 154233096 | A | G | 45.8071 | Bpifb5 | intronic | . | . | . | 2qH1 | rs46106448 | 0 | 1 |
| chr2 | 154233100 | G | A | 52 | Bpifb5 | intronic | . | . | . | 2qH1 | rs50047733 | 0 | 1 |
| chr2 | 154233275 | G | A | 182 | Bpifb5 | exonic | synonymous SNV | Bpifb5:NM_144890:exon10:c.G1122A:p.T374T | . | 2qH1 | rs50359352 | 0 | 1 |
| chr2 | 154233353 | A | C | 75 | Bpifb5 | intronic | . | . | . | 2qH1 | rs46204970 | 0 | 1 |
| chr2 | 154234358 | A | G | 123 | Bpifb5 | intronic | . | . | . | 2qH1 | rs251962992 | 0 | 1 |
| chr2 | 154234378 | C | T | 141 | Bpifb5 | intronic | . | . | . | 2qH1 | rs214707538 | 0 | 1 |
| chr2 | 154236791 | C | T | 104 | Bpifb5 | exonic | synonymous SNV | Bpifb5:NM_144890:exon13:c.C1333T:p.L445L | . | 2qH1 | rs49660546 | 0 | 1 |
| chr2 | 154236952 | T | C | 50 | Bpifb5 | intronic | . | . | . | 2qH1 | rs229001038 | 0 | 1 |
| chr2 | 154238708 | G | A | 105 | Bpifb5 | intronic | . | . | . | 2qH1 | rs51058165 | 0 | 1 |
| chr2 | 154238943 | G | T | 143 | Bpifb5 | intronic | . | . | . | 2qH1 | rs27315541 | 0 | 1 |
| chr2 | 154240661 | C | T | 124 | Bpifb5 | intronic | . | . | . | 2qH1 | rs27315527 | 0 | 1 |
| chr2 | 154240742 | C | T | 74 | Bpifb5 | intronic | . | . | . | 2qH1 | rs49506537 | 0 | 1 |
| chr2 | 154240744 | A | G | 71 | Bpifb5 | intronic | . | . | . | 2qH1 | rs27315526 | 0 | 1 |
| chr2 | 154285064 | T | G | 132 | . | intergenic | . | . | . | 2qH1 | rs27381683 | 0 | 1 |
| chr2 | 154285174 | G | C | 194 | . | intergenic | . | . | . | 2qH1 | rs27381682 | 0 | 1 |
| chr2 | 154285200 | T | C | 192 | . | intergenic | . | . | . | 2qH1 | rs27381681 | 0 | 1 |
| chr2 | 154285224 | G | A | 150 | . | intergenic | . | . | . | 2qH1 | rs27381680 | 0 | 1 |
| chr2 | 154285241 | A | G | 148 | . | intergenic | . | . | . | 2qH1 | rs27381679 | 0 | 1 |
| chr2 | 154335299 | C | A | 40.901 | . | downstream | . | . | . | 2qH1 | rs33254803 | 0 | 1 |
| chr2 | 154335460 | G | A | 222 | Cdk5rap1 | UTR3 | . | . | . | 2qH1 | rs33407072 | 0 | 1 |
| chr2 | 154352141 | T | C | 222 | Cdk5rap1 | intronic | . | . | . | 2qH1 | rs27366111 | 0 | 1 |
| chr2 | 154353110 | C | T | 66 | Cdk5rap1 | intronic | . | . | . | 2qH1 | rs27366107 | 0 | 1 |
| chr2 | 154353131 | C | T | 52 | Cdk5rap1 | intronic | . | . | . | 2qH1 | rs27366106 | 0 | 1 |
| chr2 | 154353179 | C | T | 107 | Cdk5rap1 | intronic | . | . | . | 2qH1 | rs27366105 | 0 | 1 |
| chr2 | 154353247 | G | A | 222 | Cdk5rap1 | exonic | synonymous SNV | Cdk5rap1:NM_025876:exon8:c.C1047T:p.I349I | . | 2qH1 | rs27366104 | 0 | 1 |
| chr2 | 154353509 | C | T | 110 | Cdk5rap1 | intronic | . | . | . | 2qH1 | rs27366103 | 0 | 1 |
| chr2 | 154353994 | C | G | 222 | Cdk5rap1 | intronic | . | . | . | 2qH1 | rs27366102 | 0 | 1 |
| chr2 | 154365999 | C | T | 221 | Cdk5rap1 | exonic | synonymous SNV | Cdk5rap1:NM_025876:exon5:c.G504A:p.R168R | . | 2qH1 | rs13475282 | 0 | 1 |
| chr2 | 154366133 | G | A | 143 | Cdk5rap1 | intronic | . | . | . | 2qH1 | rs27366094 | 0 | 1 |
| chr2 | 154368287 | G | A | 206 | Cdk5rap1 | intronic | . | . | . | 2qH1 | rs27366090 | 0 | 1 |
| chr2 | 154368619 | G | C | 26.8798 | Cdk5rap1 | intronic | . | . | . | 2qH1 | rs27366089 | 0 | 1 |
| chr2 | 154368715 | T | G | 181 | Cdk5rap1 | intronic | . | . | . | 2qH1 | rs27366086 | 0 | 1 |
| chr2 | 154370823 | A | G | 222 | Cdk5rap1 | exonic | synonymous SNV | Cdk5rap1:NM_025876:exon2:c.T114C:p.P38P | . | 2qH1 | rs27366082 | 0 | 1 |
| chr2 | 154376706 | T | G | 104 | Snta1 | UTR3 | . | . | . | 2qH1 | rs33024637 | 0 | 1 |
| chr2 | 154376992 | C | T | 222 | Snta1 | intronic | . | . | . | 2qH1 | rs29585156 | 0 | 1 |
| chr2 | 154377151 | G | A | 222 | Snta1 | exonic | synonymous SNV | Snta1:NM_009228:exon7:c.C1263T:p.I421I | . | 2qH1 | rs27366065 | 0 | 1 |
| chr2 | 154377965 | G | A | 24.8604 | Snta1 | intronic | . | . | . | 2qH1 | rs27366063 | 0 | 1 |
| chr2 | 154378026 | T | C | 127 | Snta1 | exonic | missense SNV | Snta1:NM_009228:exon6:c.A1201G:p.I401V | . | 2qH1 | rs13461839 | 0 | 1 |
| chr2 | 154383719 | A | T | 114 | Snta1 | intronic | . | . | . | 2qH1 | rs27366044 | 0 | 1 |
| chr2 | 154383744 | A | G | 158 | Snta1 | intronic | . | . | . | 2qH1 | rs27366043 | 0 | 1 |
| chr2 | 154436709 | T | C | 141 | Cbfa2t2 | intronic | . | . | 866 | 2qH1 | rs33459074 | 0 | 1 |
| chr2 | 154475927 | C | G | 134 | Cbfa2t2 | intronic | . | . | . | 2qH1 | rs29526002 | 0 | 1 |
| chr2 | 154476020 | A | G | 222 | Cbfa2t2 | intronic | . | . | . | 2qH1 | rs27365937 | 0 | 1 |
| chr2 | 154476074 | T | C | 222 | Cbfa2t2 | intronic | . | . | . | 2qH1 | rs27365936 | 0 | 1 |
| chr2 | 154500215 | A | G | 70 | Cbfa2t2 | intronic | . | . | . | 2qH1 | rs29538573 | 0 | 1 |
| chr2 | 154500222 | T | C | 100 | Cbfa2t2 | intronic | . | . | . | 2qH1 | rs27341263 | 0 | 1 |
| chr2 | 154500306 | T | G | 157 | Cbfa2t2 | intronic | . | . | . | 2qH1 | rs27341262 | 0 | 1 |
| chr2 | 154504779 | C | T | 222 | Cbfa2t2 | exonic | synonymous SNV | Cbfa2t2:NM_009823:exon3:c.C405T:p.L135L,Cbfa2t2:NM_172860:exon3:c.C405T:p.L135L | . | 2qH1 | rs27341248 | 0 | 1 |
| chr2 | 154510441 | T | A | 182 | Cbfa2t2 | intronic | . | . | . | 2qH1 | rs29627313 | 0 | 1 |
| chr2 | 154510453 | T | A | 222 | Cbfa2t2 | intronic | . | . | . | 2qH1 | rs27341220 | 0 | 1 |
| chr2 | 154510684 | A | G | 141 | Cbfa2t2 | intronic | . | . | . | 2qH1 | rs27341217 | 0 | 1 |
| chr2 | 154515752 | G | A | 47.4248 | Cbfa2t2 | intronic | . | . | . | 2qH1 | rs27341202 | 0 | 1 |
| chr2 | 154516173 | G | A | 91 | Cbfa2t2 | intronic | . | . | . | 2qH1 | rs27341199 | 0 | 1 |
| chr2 | 154520959 | A | G | 32.4458 | Cbfa2t2 | intronic | . | . | . | 2qH1 | rs27341178 | 0 | 1 |
| chr2 | 154521120 | G | T | 222 | Cbfa2t2 | intronic | . | . | . | 2qH1 | rs27341177 | 0 | 1 |
| chr2 | 154523889 | A | G | 182 | Cbfa2t2 | intronic | . | . | . | 2qH1 | rs27341171 | 0 | 1 |
| chr2 | 154524151 | T | C | 222 | Cbfa2t2 | intronic | . | . | . | 2qH1 | rs27341168 | 0 | 1 |
| chr2 | 154524673 | C | T | 221 | Cbfa2t2 | intronic | . | . | . | 2qH1 | rs27341164 | 0 | 1 |
| chr2 | 154528688 | T | C | 93 | Cbfa2t2 | intronic | . | . | . | 2qH1 | rs33200712 | 0 | 1 |
| chr2 | 154528853 | T | C | 221 | Cbfa2t2 | exonic | synonymous SNV | Cbfa2t2:NM_001285446:exon9:c.T594C:p.F198F,Cbfa2t2:NM_009823:exon9:c.T1245C:p.F415F,Cbfa2t2:N | . | 2qH1 | rs27341150 | 0 | 1 |
| chr2 | 154528912 | G | T | 202 | Cbfa2t2 | intronic | . | . | . | 2qH1 | rs47098587 | 0 | 1 |
| chr2 | 154531483 | A | G | 222 | Cbfa2t2 | intronic | . | . | . | 2qH1 | rs27341135 | 0 | 1 |
| chr2 | 154534823 | A | G | 162 | Cbfa2t2 | intronic | . | . | . | 2qH1 | rs27341109 | 0 | 1 |
| chr2 | 154535130 | C | A | 183 | Cbfa2t2 | exonic | synonymous SNV | Cbfa2t2:NM_001285446:exon11:c.C1084A:p.R362R,Cbfa2t2:NM_009823:exon11:c.C1732A:p.R578R,Cbfa2t | . | 2qH1 | rs27341108 | 0 | 1 |
| chr2 | 154546369 | A | G | 150 | Necab3 | intronic | . | . | . | 2qH1 | rs6291774 | 0 | 1 |
| chr2 | 154546509 | C | T | 137 | Necab3 | intronic | . | . | . | 2qH1 | rs6292380 | 0 | 1 |
| chr2 | 154547263 | T | C | 154 | Necab3 | intronic | . | . | . | 2qH1 | rs33048672 | 0 | 1 |
| chr2 | 154547295 | C | T | 48.6571 | Necab3 | intronic | . | . | . | 2qH1 | rs33283606 | 0 | 1 |
| chr2 | 154547372 | C | T | 89 | Necab3 | intronic | . | . | . | 2qH1 | rs27341038 | 0 | 1 |
| chr2 | 154547395 | T | C | 162 | Necab3 | intronic | . | . | . | 2qH1 | rs29574796 | 0 | 1 |
| chr2 | 154549169 | T | C | 193 | Necab3,1700003F1 | intronic | . | . | . | 2qH1 | rs27341030 | 0 | 1 |
| chr2 | 154549508 | A | G | 100 | Necab3,1700003F1 | intronic | . | . | 508 | 2qH1 | rs32986636 | 0 | 1 |
| chr2 | 154549596 | G | A | 127 | 1700003F12Rik,Nec | exonic | synonymous SNV | 1700003F12Rik:NM_029305:exon3:c.G243A:p.A81A | 508 | 2qH1 | rs49521261 | 0 | 1 |
| chr2 | 154549692 | C | T | 47.0621 | Necab3,1700003F1 | exonic | synonymous SNV | 1700003F12Rik:NM_029305:exon3:c.C339T:p.G113G | 508 | 2qH1 | rs27341027 | 0 | 1 |
| chr2 | 154552027 | G | C | 74 | Necab3,Act110 | UTR5 | . | . | . | 2qH1 | rs27341010 | 0 | 1 |
| chr2 | 154552072 | C | T | 98 | Act110,Necab3 | UTR5 | . | . | . | 2qH1 | rs27341009 | 0 | 1 |
| chr2 | 154552171 | C | T | 222 | Act110,Necab3 | exonic | synonymous SNV | Act110:NM_001171640:exon1:c.C42T:p.P14P | . | 2qH1 | rs27341008 | 0 | 1 |
| chr2 | 154552607 | T | C | 178 | Act110,Necab3 | exonic | synonymous SNV | Act110:NM_001171640:exon1:c.T478C:p.L160L | 301 | 2qH1 | rs29871464 | 0 | 1 |
| chr2 | 154552612 | A | G | 173 | Necab3,Act110 | exonic | synonymous SNV | Act110:NM_001171640:exon1:c.A483G:p.L161L | 301 | 2qH1 | rs27341007 | 0 | 1 |
| chr2 | 154554614 | T | C | 102 | Necab3 | intronic | . | . | . | 2qH1 | rs33278746 | 0 | 1 |
| chr2 | 154554640 | T | C | 110 | Necab3 | intronic | . | . | . | 2qH1 | rs29557963 | 0 | 1 |
| chr2 | 154554773 | A | G | 152 | Necab3 | intronic | . | . | . | 2qH1 | rs27341002 | 0 | 1 |
| chr2 | 154555709 | G | A | 221 | Necab3 | intronic | . | . | . | 2qH1 | rs27341001 | 0 | 1 |
| chr2 | 154555763 | T | C | 118 | Necab3 | intronic | . | . | . | 2qH1 | rs29559833 | 0 | 1 |
| chr2 | 154556925 | G | T | 38.8298 | Necab3 | intronic | . | . | . | 2qH1 | rs47628336 | 0 | 1 |
| chr2 | 154557249 | T | G | 133 | Necab3 | intronic | . | . | . | 2qH1 | rs27340998 | 0 | 1 |
| chr2 | 154557297 | A | G | 129 | Necab3 | intronic | . | . | . | 2qH1 | rs27340997 | 0 | 1 |

| | | | | | | | | | | | | | |
|---|---|---|---|---|---|---|---|---|---|---|---|---|---|
| chr2 | 154560680 | G | C | 201 | E2f1 | UTR3 | . | . | . | 2qH1 | rs234989959 | 0 | 1 |
| chr2 | 154561127 | G | T | 222 | E2f1 | exonic | synonymous SNV | E2f1:NM_001291105:exon6:c.C814A:p.R272R,E2f1:NM_007891:exon6:c.C949A:p.R317R | . | 2qH1 | rs33649742 | 0 | 1 |
| chr2 | 154562903 | C | T | 172 | E2f1 | intronic | . | . | . | 2qH1 | rs50941277 | 0 | 1 |
| chr2 | 154563781 | G | A | 98 | E2f1 | intronic | . | . | . | 2qH1 | rs29773849 | 0 | 1 |
| chr2 | 154564467 | G | C | 201 | E2f1 | exonic | synonymous SNV | E2f1:NM_001291105:exon2:c.C153G:p.L51L,E2f1:NM_007891:exon2:c.C288G:p.L96L | . | 2qH1 | rs33326359 | 0 | 1 |
| chr2 | 154564588 | C | G | 35.8064 | E2f1 | intronic | . | . | . | 2qH1 | rs33192574 | 0 | 1 |
| chr2 | 154568900 | A | G | 71 | E2f1 | intronic | . | . | . | 2qH1 | rs32865851 | 0 | 1 |
| chr2 | 154569018 | T | C | 175 | E2f1 | intronic | . | . | . | 2qH1 | rs50859161 | 0 | 1 |
| chr2 | 154569036 | G | A | 208 | E2f1 | intronic | . | . | . | 2qH1 | rs47731971 | 0 | 1 |
| chr2 | 154592151 | C | T | 124 | Pxmp4 | intronic | . | . | . | 2qH1 | . | 0 | 1 |
| chr2 | 154592348 | A | C | 222 | Pxmp4 | exonic | synonymous SNV | Pxmp4:NM_021534:exon3:c.T192G:p.L64L | . | 2qH1 | . | 0 | 1 |
| chr2 | 154596104 | G | A | 33.4543 | Pxmp4 | intronic | . | . | . | 2qH1 | rs29555465 | 0 | 1 |
| chr2 | 154596133 | A | C | 145 | Pxmp4 | intronic | . | . | . | 2qH1 | rs27371030 | 0 | 1 |
| chr2 | 154596294 | A | C | 205 | Pxmp4 | intronic | . | . | . | 2qH1 | rs27371029 | 0 | 1 |
| chr2 | 154603485 | T | C | 157 | Pxmp4 | intronic | . | . | . | 2qH1 | rs27371020 | 0 | 1 |
| chr2 | 154603669 | A | T | 64 | Pxmp4 | UTR5 | . | . | . | 2qH1 | rs33623664 | 0 | 1 |
| chr2 | 154621617 | C | T | 49.904 | Zfp341 | intronic | . | . | . | 2qH1 | rs225417593 | 0 | 1 |
| chr2 | 154621621 | T | C | 49.9101 | Zfp341 | intronic | . | . | . | 2qH1 | rs27371007 | 0 | 1 |
| chr2 | 154621659 | T | C | 109 | Zfp341 | intronic | . | . | . | 2qH1 | rs27371006 | 0 | 1 |
| chr2 | 154621944 | G | A | 147 | Zfp341 | intronic | . | . | . | 2qH1 | rs29627429 | 0 | 1 |
| chr2 | 154621951 | T | A | 132 | Zfp341 | intronic | . | . | . | 2qH1 | rs33561495 | 0 | 1 |
| chr2 | 154621988 | C | T | 30.874 | Zfp341 | intronic | . | . | . | 2qH1 | rs33269435 | 0 | 1 |
| chr2 | 154621990 | T | C | 37.1292 | Zfp341 | intronic | . | . | . | 2qH1 | rs32828483 | 0 | 1 |
| chr2 | 154624801 | A | G | 138 | Zfp341 | intronic | . | . | . | 2qH1 | rs29627198 | 0 | 1 |
| chr2 | 154624889 | A | G | 221 | Zfp341 | exonic | synonymous SNV | Zfp341:NM_199304:exon3:c.A177G:p.K59K | . | 2qH1 | rs27370998 | 0 | 1 |
| chr2 | 154628167 | A | G | 186 | Zfp341 | intronic | . | . | . | 2qH1 | rs50757963 | 0 | 1 |
| chr2 | 154628844 | A | G | 128 | Zfp341 | exonic | synonymous SNV | Zfp341:NM_199304:exon6:c.A780G:p.P260P | . | 2qH1 | rs51903717 | 0 | 1 |
| chr2 | 154629029 | C | T | 116 | Zfp341 | intronic | . | . | . | 2qH1 | rs47109846 | 0 | 1 |
| chr2 | 154630345 | C | T | 174 | Zfp341 | intronic | . | . | . | 2qH1 | rs51198061 | 0 | 1 |
| chr2 | 154630534 | C | T | 210 | Zfp341 | intronic | . | . | . | 2qH1 | rs33729548 | 0 | 1 |
| chr2 | 154630592 | G | A | 124 | Zfp341 | intronic | . | . | . | 2qH1 | rs260696974 | 0 | 1 |
| chr2 | 154630608 | A | T | 80 | Zfp341 | intronic | . | . | . | 2qH1 | rs50726494 | 0 | 1 |
| chr2 | 154632273 | G | C | 222 | Zfp341 | intronic | . | . | . | 2qH1 | rs27370983 | 0 | 1 |
| chr2 | 154632459 | A | G | 222 | Zfp341 | exonic | synonymous SNV | Zfp341:NM_199304:exon8:c.A1203G:p.E401E | . | 2qH1 | rs29778925 | 0 | 1 |
| chr2 | 154634054 | C | A | 188 | Zfp341 | intronic | . | . | . | 2qH1 | rs27370981 | 0 | 1 |
| chr2 | 154634189 | A | G | 202 | Zfp341 | exonic | missense SNV | Zfp341:NM_199304:exon9:c.A1316G:p.D439G | . | 2qH1 | rs13476853 | 0 | 1 |
| chr2 | 154637921 | C | A | 20.6431 | Zfp341 | intronic | . | . | . | 2qH1 | rs27370978 | 0 | 1 |
| chr2 | 154637949 | C | T | 30.5635 | Zfp341 | intronic | . | . | . | 2qH1 | rs27370977 | 0 | 1 |
| chr2 | 154638187 | G | A | 139 | Zfp341 | exonic | synonymous SNV | Zfp341:NM_199304:exon10:c.G1578A:p.P526P | . | 2qH1 | rs49368382 | 0 | 1 |
| chr2 | 154638214 | C | T | 127 | Zfp341 | exonic | synonymous SNV | Zfp341:NM_199304:exon10:c.C1605T:p.T535T | . | 2qH1 | rs27370976 | 0 | 1 |
| chr2 | 154641286 | G | A | 139 | Zfp341 | intronic | . | . | . | 2qH1 | rs27349365 | 0 | 1 |
| chr2 | 154641427 | G | A | 222 | Zfp341 | intronic | . | . | . | 2qH1 | rs27349363 | 0 | 1 |
| chr2 | 154641913 | T | C | 53 | Zfp341 | intronic | . | . | . | 2qH1 | rs27349360 | 0 | 1 |
| chr2 | 154643518 | G | T | 221 | Zfp341 | exonic | stopgain | Zfp341:NM_199304:exon13:c.G1873T:p.E625X | . | 2qH1 | . | 0 | 1 |
| chr2 | 154643737 | G | A | 222 | Zfp341 | intronic | . | . | . | 2qH1 | rs27349354 | 0 | 1 |
| chr2 | 154643763 | T | G | 221 | Zfp341 | intronic | . | . | . | 2qH1 | rs27349352 | 0 | 1 |
| chr2 | 154643952 | G | T | 118 | Zfp341 | intronic | . | . | . | 2qH1 | . | 0 | 1 |
| chr2 | 154645599 | C | T | 139 | Zfp341 | intronic | . | . | . | 2qH1 | rs27349342 | 0 | 1 |
| chr2 | 154645670 | T | C | 183 | Zfp341 | exonic | synonymous SNV | Zfp341:NM_199304:exon15:c.T2073C:p.R691R | . | 2qH1 | rs27349341 | 0 | 1 |
| chr2 | 154645757 | C | T | 195 | Zfp341 | exonic | synonymous SNV | Zfp341:NM_199304:exon15:c.C2160T:p.H720H | . | 2qH1 | rs27349340 | 0 | 1 |
| chr2 | 154645791 | C | T | 214 | Zfp341 | exonic | missense SNV | Zfp341:NM_199304:exon15:c.C2194T:p.P732S | . | 2qH1 | rs47569799 | 0 | 1 |
| chr2 | 154646146 | A | G | 170 | Zfp341 | UTR3 | . | . | . | 2qH1 | rs27349339 | 0 | 1 |
| chr2 | 154646189 | T | C | 69 | Zfp341 | UTR3 | . | . | . | 2qH1 | rs29522174 | 0 | 1 |
| chr2 | 154657367 | A | G | 76 | Chmp4b | exonic | synonymous SNV | Chmp4b:NM_029362:exon1:c.A186G:p.K62K | 347 | 2qH1 | rs254024930 | 0 | 1 |
| chr2 | 154657382 | T | C | 59 | Chmp4b | intronic | . | . | 347 | 2qH1 | rs216432429 | 0 | 1 |
| chr2 | 154689426 | C | T | 92 | Chmp4b | intronic | . | . | . | 2qH1 | rs47681509 | 0 | 1 |
| chr2 | 154689464 | C | T | 117 | Chmp4b | intronic | . | . | . | 2qH1 | rs32820355 | 0 | 1 |
| chr2 | 154689824 | C | T | 37.7106 | Chmp4b | intronic | . | . | . | 2qH1 | rs33160622 | 0 | 1 |
| chr2 | 154692337 | A | T | 56 | Chmp4b | intronic | . | . | . | 2qH1 | rs49192554 | 0 | 1 |
| chr2 | 154692527 | C | T | 222 | Chmp4b | intronic | . | . | . | 2qH1 | rs46977678 | 0 | 1 |
| chr2 | 154692703 | T | C | 146 | Chmp4b | intronic | . | . | . | 2qH1 | rs47646173 | 0 | 1 |
| chr2 | 154692708 | T | C | 145 | Chmp4b | intronic | . | . | . | 2qH1 | rs217497964 | 0 | 1 |
| chr2 | 154692709 | G | A | 159 | Chmp4b | intronic | . | . | . | 2qH1 | rs47644590 | 0 | 1 |
| chr2 | 154692806 | A | T | 33.9094 | Chmp4b | intronic | . | . | . | 2qH1 | rs45797548 | 0 | 1 |
| chr2 | 154693748 | G | C | 63 | Chmp4b | intronic | . | . | . | 2qH1 | rs217020682 | 0 | 1 |
| chr2 | 154694142 | A | G | 36.4618 | Chmp4b | UTR3 | . | . | . | 2qH1 | rs51339654 | 0 | 1 |
| chr2 | 154703781 | C | G | 189 | . | intergenic | . | . | . | 2qH1 | rs29721685 | 0 | 1 |
| chr2 | 154704257 | C | T | 222 | . | intergenic | . | . | . | 2qH1 | . | 0 | 1 |
| chr2 | 155047767 | T | C | 221 | a | intronic | . | . | . | 2qH1 | rs238505938 | 0 | 1 |
| chr2 | 155722466 | G | T | 221 | Edem2 | exonic | missense SNV | Edem2:NM_145537:exon4:c.C330A:p.D110E | . | 2qH1 | . | 0 | 1 |
| chr2 | 156031250 | G | A | 220 | Fer1l4 | exonic | synonymous SNV | Fer1l4:NM_001136556:exon31:c.C3909T:p.I1303I | . | 2qH1 | . | 0 | 1 |
| chr2 | 156056780 | G | C | 94 | . | intergenic | . | . | . | 2qH1 | . | 0 | 1 |
| chr2 | 156566717 | T | C | 91 | Aar2 | intronic | . | . | . | 2qH1 | . | 0 | 1 |
| chr2 | 156632859 | T | G | 81 | Dlgap4 | intronic | . | . | . | 2qH1 | rs27339380 | 0 | 1 |
| chr2 | 156700855 | A | T | 139 | Dlgap4 | exonic | synonymous SNV | Dlgap4:NM_001277186:exon3:c.A177T:p.P59P,Dlgap4:NM_001277187:exon3:c.A177T:p.P59P,Dlgap4:NM_ | . | 2qH1 | rs45960087 | 0 | 1 |
| chr2 | 156700951 | C | T | 72 | Dlgap4 | exonic | synonymous SNV | Dlgap4:NM_001277186:exon3:c.C273T:p.H91H,Dlgap4:NM_001277187:exon3:c.C273T:p.H91H,Dlgap4:NM_ | . | 2qH1 | rs29922025 | 0 | 1 |
| chr2 | 156701239 | G | A | 163 | Dlgap4 | exonic | synonymous SNV | Dlgap4:NM_001277186:exon3:c.G561A:p.R187R,Dlgap4:NM_001277187:exon3:c.G561A:p.R187R,Dlgap4:NM | . | 2qH1 | rs33491222 | 0 | 1 |
| chr2 | 156701344 | T | C | 67 | Dlgap4 | exonic | synonymous SNV | Dlgap4:NM_001277186:exon3:c.T666C:p.P222P,Dlgap4:NM_001277187:exon3:c.T666C:p.P222P,Dlgap4:NM | . | 2qH1 | rs33268943 | 0 | 1 |
| chr2 | 156701575 | T | C | 23.2861 | Dlgap4 | exonic | synonymous SNV | Dlgap4:NM_001277186:exon3:c.T897C:p.S299S,Dlgap4:NM_001277187:exon3:c.T897C:p.S299S,Dlgap4:NM | . | 2qH1 | rs220055392 | 0 | 1 |
| chr2 | 156704398 | C | T | 74 | Dlgap4 | intronic | . | . | . | 2qH1 | rs47434704 | 0 | 1 |
| chr2 | 156704653 | C | A | 210 | Dlgap4 | exonic | synonymous SNV | Dlgap4:NM_001277186:exon4:c.C1240A:p.R414R,Dlgap4:NM_001277187:exon4:c.C1240A:p.R414R,Dlgap4: | . | 2qH1 | rs260519787 | 0 | 1 |
| chr2 | 156705994 | A | G | 222 | Dlgap4 | intronic | . | . | . | 2qH1 | rs237804650 | 0 | 1 |
| chr2 | 156706934 | C | A | 76 | Dlgap4 | intronic | . | . | . | 2qH1 | rs52044971 | 0 | 1 |
| chr2 | 156706958 | G | A | 102 | Dlgap4 | intronic | . | . | . | 2qH1 | rs245499280 | 0 | 1 |
| chr2 | 156707010 | A | G | 141 | Dlgap4 | intronic | . | . | . | 2qH1 | rs33195300 | 0 | 1 |
| chr2 | 156710844 | A | G | 61 | Dlgap4 | intronic | . | . | . | 2qH1 | rs27324353 | 0 | 1 |
| chr2 | 156711032 | A | G | 222 | Dlgap4 | exonic | synonymous SNV | Dlgap4:NM_001277187:exon6:c.A1368G:p.A456A,Dlgap4:NM_001277186:exon7:c.A1419G:p.A473A,Dlgap4: | . | 2qH1 | rs13476862 | 0 | 1 |
| chr2 | 156711128 | C | T | 171 | Dlgap4 | exonic | synonymous SNV | Dlgap4:NM_001277187:exon6:c.C1464T:p.Y488Y,Dlgap4:NM_001277186:exon7:c.C1515T:p.Y505Y,Dlgap4: | . | 2qH1 | rs27324351 | 0 | 1 |
| chr2 | 156711352 | A | G | 222 | Dlgap4 | intronic | . | . | . | 2qH1 | rs213661205 | 0 | 1 |
| chr2 | 156711450 | A | G | 94 | Dlgap4 | intronic | . | . | . | 2qH1 | rs230479004 | 0 | 1 |
| chr2 | 156745752 | G | A | 161 | Dlgap4 | intronic | . | . | . | 2qH1 | . | 0 | 1 |
| chr2 | 156745902 | G | A | 222 | Dlgap4 | exonic | synonymous SNV | Dlgap4:NM_001042487:exon2:c.G99A:p.P33P,Dlgap4:NM_001277187:exon7:c.G1665A:p.P555P,Dlgap4:NM | . | 2qH1 | rs27324252 | 0 | 1 |
| chr2 | 156748398 | A | G | 221 | Dlgap4 | exonic | missense SNV | Dlgap4:NM_001042487:exon3:c.A460G:p.I154V,Dlgap4:NM_001277187:exon8:c.A2026G:p.I676V,Dlgap4:N | . | 2qH1 | rs27324237 | 0 | 1 |
| chr2 | 156761027 | A | G | 116 | Dlgap4 | intronic | . | . | . | 2qH1 | rs27324186 | 0 | 1 |
| chr2 | 156761047 | G | A | 108 | Dlgap4 | intronic | . | . | . | 2qH1 | rs27324185 | 0 | 1 |
| chr2 | 156761717 | A | G | 63 | Dlgap4 | intronic | . | . | . | 2qH1 | rs27324181 | 0 | 1 |
| chr2 | 156778505 | G | A | 135 | My19 | intronic | . | . | . | 2qH1 | rs244267299 | 0 | 1 |
| chr2 | 156778679 | A | C | 194 | My19 | exonic | synonymous SNV | My19:NM_172118:exon2:c.A135C:p.R45R | . | 2qH1 | rs27309443 | 0 | 1 |
| chr2 | 156778716 | C | T | 140 | My19 | exonic | synonymous SNV | My19:NM_172118:exon2:c.C172T:p.L58L | . | 2qH1 | rs27309442 | 0 | 1 |
| chr2 | 156778739 | C | T | 111 | My19 | intronic | . | . | . | 2qH1 | rs235850806 | 0 | 1 |
| chr2 | 156780379 | A | G | 60 | My19 | intronic | . | . | . | 2qH1 | rs27309434 | 0 | 1 |
| chr2 | 156780445 | A | G | 109 | My19 | intronic | . | . | . | 2qH1 | rs27309433 | 0 | 1 |
| chr2 | 156780504 | T | C | 168 | My19 | exonic | synonymous SNV | My19:NM_172118:exon3:c.T207C:p.Y69Y | . | 2qH1 | rs27309432 | 0 | 1 |
| chr2 | 156780718 | T | C | 30.3616 | My19 | intronic | . | . | . | 2qH1 | rs27309430 | 0 | 1 |
| chr2 | 156780878 | G | A | 60 | My19 | intronic | . | . | . | 2qH1 | rs46437002 | 0 | 1 |
| chr2 | 156780975 | C | T | 117 | My19 | intronic | . | . | . | 2qH1 | rs27309427 | 0 | 1 |
| chr2 | 156781238 | C | T | 199 | My19 | UTR3 | . | . | . | 2qH1 | rs27309426 | 0 | 1 |
| chr2 | 156789780 | C | G | 222 | . | intergenic | . | . | . | 2qH1 | rs27309406 | 0 | 1 |
| chr2 | 156789878 | A | C | 222 | . | intergenic | . | . | . | 2qH1 | rs47982507 | 0 | 1 |
| chr2 | 156790226 | G | A | 222 | . | intergenic | . | . | . | 2qH1 | rs27309403 | 0 | 1 |
| chr2 | 156790359 | C | A | 199 | . | intergenic | . | . | . | 2qH1 | rs27309400 | 0 | 1 |
| chr2 | 156844055 | A | G | 98 | Tgif2 | intronic | . | . | . | 2qH1 | rs27309299 | 0 | 1 |
| chr2 | 156844303 | T | C | 215 | Tgif2 | exonic | synonymous SNV | Tgif2:NM_001291124:exon2:c.T114C:p.Y38Y,Tgif2:NM_173396:exon2:c.T114C:p.Y38Y | . | 2qH1 | rs27309298 | 0 | 1 |
| chr2 | 156844397 | G | T | 131 | Tgif2 | intronic | . | . | . | 2qH1 | rs582190756 | 0 | 1 |
| chr2 | 156852984 | A | G | 66 | Tgif2.5430405H02R | ncRNA_intronic | . | . | . | 2qH1 | rs226644521 | 0 | 1 |
| chr2 | 156853155 | T | G | 222 | Tgif2.5430405H02R | exonic | synonymous SNV | Tgif2:NM_001291124:exon3:c.T219G:p.R73R,Tgif2:NM_173396:exon3:c.T219G:p.R73R | . | 2qH1 | rs27309239 | 0 | 1 |
| chr2 | 156853239 | T | A | 169 | Tgif2.5430405H02R | exonic | synonymous SNV | Tgif2:NM_173396:exon3:c.T303A:p.S101S | . | 2qH1 | rs252167827 | 0 | 1 |
| chr2 | 156865202 | T | G | 222 | 1110008F13Rik | intronic | . | . | . | 2qH1 | rs29866914 | 0 | 1 |
| chr2 | 156865326 | G | A | 222 | 1110008F13Rik | exonic | missense SNV | 1110008F13Rik:NM_026124:exon2:c.G184A:p.V62I | . | 2qH1 | rs13473072 | 0 | 1 |
| chr2 | 156865422 | G | C | 222 | 1110008F13Rik | intronic | . | . | . | 2qH1 | rs33317193 | 0 | 1 |
| chr2 | 156872271 | T | G | 207 | 1110008F13Rik | intronic | . | . | . | 2qH1 | rs33158210 | 0 | 1 |
| chr2 | 156872321 | G | T | 222 | 1110008F13Rik | intronic | . | . | . | 2qH1 | rs33685582 | 0 | 1 |
| chr2 | 156872476 | G | A | 221 | 1110008F13Rik | intronic | . | . | . | 2qH1 | rs33542966 | 0 | 1 |
| chr2 | 156872618 | A | G | 87 | 1110008F13Rik | intronic | . | . | . | 2qH1 | rs33335090 | 0 | 1 |
| chr2 | 156872635 | A | G | 53 | 1110008F13Rik | intronic | . | . | . | 2qH1 | rs29819096 | 0 | 1 |
| chr2 | 156873054 | C | T | 221 | 1110008F13Rik.Sla | exonic | synonymous SNV | 1110008F13Rik:NM_026124:exon4:c.C360T:p.I120I | . | 2qH1 | rs32967084 | 0 | 1 |
| chr2 | 156873151 | A | T | 128 | Sla2.1110008F13Ri | UTR3 | . | . | . | 2qH1 | rs33057524 | 0 | 1 |
| chr2 | 156873211 | A | G | 38.1602 | Sla2.1110008F13Ri | UTR3 | . | . | . | 2qH1 | rs33246076 | 0 | 1 |
| chr2 | 156874620 | A | G | 181 | Sla2 | intronic | . | . | . | 2qH1 | rs27309206 | 0 | 1 |
| chr2 | 156874677 | G | T | 55 | Sla2 | intronic | . | . | . | 2qH1 | rs27309205 | 0 | 1 |
| chr2 | 156875156 | A | G | 124 | Sla2 | intronic | . | . | . | 2qH1 | rs33378655 | 0 | 1 |
| chr2 | 156875161 | A | G | 122 | Sla2 | intronic | . | . | . | 2qH1 | rs32889392 | 0 | 1 |
| chr2 | 156875840 | A | G | 113 | Sla2 | exonic | synonymous SNV | Sla2:NM_029983:exon6:c.T511C:p.L171L | . | 2qH1 | rs50808961 | 0 | 1 |
| chr2 | 156876011 | T | C | 150 | Sla2 | intronic | . | . | . | 2qH1 | rs29505910 | 0 | 1 |
| chr2 | 156879390 | T | C | 194 | Sla2 | intronic | . | . | . | 2qH1 | rs229527620 | 0 | 1 |
| chr2 | 156879507 | G | A | 151 | Sla2 | exonic | synonymous SNV | Sla2:NM_029983:exon4:c.C210T:p.V70V | . | 2qH1 | rs27309182 | 0 | 1 |
| chr2 | 156879532 | G | T | 30.5488 | Sla2 | splicing | . | . | . | 2qH1 | rs211744027 | 0 | 1 |
| chr2 | 156879542 | A | G | 35.6398 | Sla2 | intronic | . | . | . | 2qH1 | rs228964956 | 0 | 1 |
| chr2 | 156879737 | G | A | 84 | Sla2 | intronic | . | . | . | 2qH1 | rs27309179 | 0 | 1 |
| chr2 | 156879827 | T | C | 200 | Sla2 | intronic | . | . | . | 2qH1 | rs27309178 | 0 | 1 |
| chr2 | 156879982 | T | C | 168 | Sla2 | intronic | . | . | . | 2qH1 | rs33618700 | 0 | 1 |
| chr2 | 156880022 | C | T | 60 | Sla2 | intronic | . | . | . | 2qH1 | rs218207533 | 0 | 1 |
| chr2 | 156883502 | T | C | 160 | Sla2 | intronic | . | . | . | 2qH1 | rs27356648 | 0 | 1 |
| chr2 | 156883719 | A | G | 222 | Sla2 | intronic | . | . | . | 2qH1 | rs27356647 | 0 | 1 |
| chr2 | 156883762 | T | C | 112 | Sla2 | intronic | . | . | . | 2qH1 | rs27356646 | 0 | 1 |
| chr2 | 156889924 | T | C | 146 | . | intergenic | . | . | . | 2qH1 | rs30574216 | 0 | 1 |
| chr2 | 156896670 | C | T | 61 | . | intergenic | . | . | . | 2qH1 | rs27356608 | 0 | 1 |
| chr2 | 156896856 | T | C | 222 | . | intergenic | . | . | . | 2qH1 | rs49996947 | 0 | 1 |
| chr2 | 156896963 | G | A | 222 | . | intergenic | . | . | . | 2qH1 | rs27337176 | 0 | 1 |
| chr2 | 156897014 | T | G | 222 | . | intergenic | . | . | . | 2qH1 | rs27337174 | 0 | 1 |
| chr2 | 156897057 | G | A | 222 | . | intergenic | . | . | . | 2qH1 | rs27337173 | 0 | 1 |
| chr2 | 156897149 | T | C | 112 | . | intergenic | . | . | . | 2qH1 | rs27356604 | 0 | 1 |
| chr2 | 156906197 | T | A | 88 | . | intergenic | . | . | . | 2qH1 | rs252665901 | 0 | 1 |
| chr2 | 156906338 | T | G | 153 | . | intergenic | . | . | . | 2qH1 | rs27337344 | 0 | 1 |
| chr2 | 156906383 | A | C | 221 | . | intergenic | . | . | . | 2qH1 | rs27337343 | 0 | 1 |
| chr2 | 156912218 | A | T | 195 | . | intergenic | . | . | . | 2qH1 | rs260875346 | 0 | 1 |
| chr2 | 156912374 | A | G | 222 | . | intergenic | . | . | . | 2qH1 | rs246081524 | 0 | 1 |
| chr2 | 156912393 | A | G | 222 | . | intergenic | . | . | . | 2qH1 | rs27337316 | 0 | 1 |
| chr2 | 156930064 | T | C | 188 | Ndrg3 | intronic | . | . | . | 2qH1 | rs51738419 | 0 | 1 |
| chr2 | 156930072 | A | G | 155 | Ndrg3 | intronic | . | . | . | 2qH1 | rs27337220 | 0 | 1 |
| chr2 | 156930079 | G | A | 108 | Ndrg3 | intronic | . | . | . | 2qH1 | rs224969339 | 0 | 1 |
| chr2 | 156931303 | A | G | 222 | Ndrg3 | intronic | . | . | . | 2qH1 | rs27337213 | 0 | 1 |
| chr2 | 156931599 | T | C | 222 | Ndrg3 | intronic | . | . | . | 2qH1 | rs27337211 | 0 | 1 |
| chr2 | 156934842 | T | C | 130 | Ndrg3 | intronic | . | . | . | 2qH1 | rs27337200 | 0 | 1 |
| chr2 | 156935024 | T | G | 221 | Ndrg3 | intronic | . | . | . | 2qH1 | rs27337199 | 0 | 1 |
| chr2 | 156935057 | C | G | 195 | Ndrg3 | intronic | . | . | . | 2qH1 | rs27337198 | 0 | 1 |
| chr2 | 156937448 | G | A | 221 | Ndrg3 | intronic | . | . | . | 2qH1 | rs27337175 | 0 | 1 |
| chr2 | 156938343 | A | T | 44.1795 | Ndrg3 | intronic | . | . | . | 2qH1 | rs27337163 | 0 | 1 |
| chr2 | 156938589 | G | A | 222 | Ndrg3 | exonic | synonymous SNV | Ndrg3:NM_013865:exon10:c.C654T:p.N218N,Ndrg3:NM_180956:exon10:c.C654T:p.N218N | . | 2qH1 | rs27337162 | 0 | 1 |
| chr2 | 156938684 | G | A | 221 | Ndrg3 | intronic | . | . | . | 2qH1 | rs27337161 | 0 | 1 |
| chr2 | 156940197 | C | T | 56 | Ndrg3 | intronic | . | . | . | 2qH1 | rs27337155 | 0 | 1 |
| chr2 | 156940288 | G | A | 79 | Ndrg3 | intronic | . | . | . | 2qH1 | rs51184629 | 0 | 1 |
| chr2 | 156940299 | T | G | 78 | Ndrg3 | intronic | . | . | . | 2qH1 | rs50878376 | 0 | 1 |
| chr2 | 156940344 | A | C | 152 | Ndrg3 | intronic | . | . | . | 2qH1 | rs27337154 | 0 | 1 |
| chr2 | 156948417 | C | G | 134 | Ndrg3 | intronic | . | . | . | 2qH1 | rs232331556 | 0 | 1 |
| chr2 | 156948418 | T | A | 141 | Ndrg3 | intronic | . | . | . | 2qH1 | rs250999738 | 0 | 1 |
| chr2 | 156948449 | T | C | 28.8814 | Ndrg3 | intronic | . | . | . | 2qH1 | rs27337089 | 0 | 1 |
| chr2 | 156996867 | G | A | 222 | Dsn1 | intronic | . | . | . | 2qH1 | . | 0 | 1 |
| chr2 | 157253092 | C | A | 222 | Mroh8 | intronic | . | . | . | 2qH1 | rs27338463 | 0 | 1 |
| chr2 | 157256485 | A | G | 222 | Mroh8 | intronic | . | . | . | 2qH1 | rs27338447 | 0 | 1 |
| chr2 | 157323359 | A | G | 166 | Rpn2 | intronic | . | . | . | 2qH1 | rs51354921 | 0 | 1 |
| chr2 | 157325665 | G | A | 216 | Rpn2 | intronic | . | . | . | 2qH1 | . | 0 | 1 |
| chr2 | 157737507 | C | T | 222 | Ctnnbl1 | UTR5 | . | . | 350 | 2qH1 | rs27307573 | 0 | 1 |
| chr2 | 157901494 | T | C | 188 | . | intergenic | . | . | . | 2qH1 | rs29536742 | 1 | 0 |
| chr2 | 157992770 | C | T | 52 | Tti1 | intronic | . | . | . | 2qH1 | . | 0 | 1 |

| | | | | | | | | | | | | | |
|---|---|---|---|---|---|---|---|---|---|---|---|---|---|
| chr2 | 157995224 | A | G | 101 | Tti1 | intronic | . | . | . | 2qH1 | rs3691210 | 0 | 1 |
| chr2 | 158008671 | G | A | 221 | Tti1 | exonic | missense SNV | Tti1:NM_029282:exon1:c.C647T:p.A216V | . | 2qH1 | rs27309098 | 0 | 1 |
| chr2 | 158138637 | A | G | 183 | Tgm2 | intronic | . | . | . | 2qH1 | rs8273753 | 0 | 1 |
| chr2 | 158139031 | A | G | 213 | Tgm2 | intronic | . | . | . | 2qH1 | rs3659215 | 0 | 1 |
| chr2 | 158139694 | T | A | 134 | Tgm2 | intronic | . | . | . | 2qH1 | rs29816515 | 0 | 1 |
| chr2 | 158186400 | A | G | 120 | D630003M21Rik | intronic | . | . | . | 2qH1 | rs45923805 | 0 | 1 |
| chr2 | 158194019 | C | T | 74 | D630003M21Rik,17 | ncRNA_intronic | . | . | . | 2qH1 | rs33029220 | 0 | 1 |
| chr2 | 158195336 | T | C | 22.7382 | D630003M21Rik,17 | ncRNA_intronic | . | . | . | 2qH1 | rs27320427 | 0 | 1 |
| chr2 | 158195464 | T | C | 58 | 1700060C20Rik,D63 | ncRNA_exonic | . | . | . | 2qH1 | rs27320424 | 0 | 1 |
| chr2 | 158195818 | C | A | 101 | D630003M21Rik | UTR3 | . | . | . | 2qH1 | rs27320419 | 0 | 1 |
| chr2 | 158196473 | G | A | 151 | D630003M21Rik | exonic | missense SNV | D630003M21Rik:NM_001131021:exon14:c.C3052T:p.R1018W,D630003M21Rik:NM_177657:exon14:c.C3052T:p | . | 2qH1 | rs29538638 | 0 | 1 |
| chr2 | 158200290 | A | G | 222 | D630003M21Rik | exonic | missense SNV | D630003M21Rik:NM_001131021:exon13:c.T2735C:p.M912T,D630003M21Rik:NM_177657:exon13:c.T2735C:p. | . | 2qH1 | rs6410806 | 0 | 1 |
| chr2 | 158200993 | C | T | 193 | D630003M21Rik | exonic | missense SNV | D630003M21Rik:NM_001131021:exon12:c.G2626A:p.A876T,D630003M21Rik:NM_177657:exon12:c.G2626A:p. | . | 2qH1 | rs27320369 | 0 | 1 |
| chr2 | 158202953 | C | T | 55 | D630003M21Rik | intronic | . | . | . | 2qH1 | rs47741110 | 0 | 1 |
| chr2 | 158202956 | T | G | 55 | D630003M21Rik | intronic | . | . | . | 2qH1 | rs46703513 | 0 | 1 |
| chr2 | 158203004 | T | G | 127 | D630003M21Rik | intronic | . | . | . | 2qH1 | rs27320359 | 0 | 1 |
| chr2 | 158204608 | C | T | 221 | D630003M21Rik | intronic | . | . | . | 2qH1 | rs51490348 | 0 | 1 |
| chr2 | 158205788 | T | C | 222 | D630003M21Rik | exonic | synonymous SNV | D630003M21Rik:NM_001131021:exon9:c.A2160G:p.P720P,D630003M21Rik:NM_177657:exon9:c.A2160G:p.P7 | . | 2qH1 | rs27320339 | 0 | 1 |
| chr2 | 158210060 | G | C | 66 | D630003M21Rik | intronic | . | . | . | 2qH1 | rs27320318 | 0 | 1 |
| chr2 | 158210248 | A | C | 192 | D630003M21Rik | intronic | . | . | . | 2qH1 | rs51240962 | 0 | 1 |
| chr2 | 158210257 | T | C | 159 | D630003M21Rik | intronic | . | . | . | 2qH1 | rs46017087 | 0 | 1 |
| chr2 | 158210282 | A | G | 153 | D630003M21Rik | intronic | . | . | . | 2qH1 | rs50266817 | 0 | 1 |
| chr2 | 158210380 | T | C | 38.4206 | D630003M21Rik | intronic | . | . | . | 2qH1 | rs27320316 | 0 | 1 |
| chr2 | 158211495 | G | T | 103 | D630003M21Rik | intronic | . | . | . | 2qH1 | rs27320306 | 0 | 1 |
| chr2 | 158211569 | A | C | 148 | D630003M21Rik | intronic | . | . | . | 2qH1 | rs32834007 | 0 | 1 |
| chr2 | 158211683 | C | T | 100 | D630003M21Rik | exonic | synonymous SNV | D630003M21Rik:NM_001131021:exon6:c.G1884A:p.V628V,D630003M21Rik:NM_177657:exon6:c.G1884A:p.V6 | . | 2qH1 | rs27320305 | 0 | 1 |
| chr2 | 158213476 | G | A | 199 | D630003M21Rik | exonic | synonymous SNV | D630003M21Rik:NM_001131021:exon5:c.C1740T:p.D580D,D630003M21Rik:NM_177657:exon5:c.C1740T:p.D5 | . | 2qH1 | rs47561154 | 0 | 1 |
| chr2 | 158213531 | T | C | 130 | D630003M21Rik | intronic | . | . | . | 2qH1 | rs50856052 | 0 | 1 |
| chr2 | 158220587 | A | G | 80 | D630003M21Rik | exonic | synonymous SNV | D630003M21Rik:NM_001131021:exon2:c.T12C:p.S4S,D630003M21Rik:NM_177657:exon2:c.T12C:p.S4S | . | 2qH1 | rs27320270 | 0 | 1 |
| chr2 | 158220608 | G | A | 35.7319 | D630003M21Rik | intronic | . | . | . | 2qH1 | rs49643708 | 0 | 1 |
| chr2 | 158261291 | T | C | 221 | Bpi | intronic | . | . | . | 2qH1 | rs33260448 | 0 | 1 |
| chr2 | 158262473 | T | C | 36.6984 | Bpi | exonic | synonymous SNV | Bpi:NM_177850:exon4:c.T495C:p.I165I | . | 2qH1 | rs46865386 | 0 | 1 |
| chr2 | 158267693 | T | C | 222 | Bpi | intronic | . | . | . | 2qH1 | rs27303934 | 0 | 1 |
| chr2 | 158268686 | G | A | 113 | Bpi | intronic | . | . | . | 2qH1 | rs48317920 | 0 | 1 |
| chr2 | 158268886 | C | G | 221 | Bpi | exonic | missense SNV | Bpi:NM_177850:exon7:c.C706G:p.L236V | . | 2qH1 | rs45863699 | 0 | 1 |
| chr2 | 158268955 | G | A | 71 | Bpi | intronic | . | . | . | 2qH1 | rs46097364 | 0 | 1 |
| chr2 | 158268962 | C | T | 40.5371 | Bpi | intronic | . | . | . | 2qH1 | rs50141190 | 0 | 1 |
| chr2 | 158271870 | T | C | 222 | Bpi | intronic | . | . | . | 2qH1 | rs220824610 | 0 | 1 |
| chr2 | 158272068 | T | A | 132 | Bpi | exonic | synonymous SNV | Bpi:NM_177850:exon8:c.T885A:p.A295A | . | 2qH1 | rs33487853 | 0 | 1 |
| chr2 | 158272080 | T | C | 118 | Bpi | exonic | synonymous SNV | Bpi:NM_177850:exon8:c.T897C:p.S299S | . | 2qH1 | rs32901168 | 0 | 1 |
| chr2 | 158273346 | C | T | 140 | Bpi | intronic | . | . | . | 2qH1 | rs49801150 | 0 | 1 |
| chr2 | 158284256 | C | T | 207 | Bpi | exonic | missense SNV | Bpi:NM_177850:exon15:c.C1450T:p.H484Y | . | 2qH1 | rs240601214 | 0 | 1 |
| chr2 | 158314362 | T | G | 41.9238 | Lbp | intronic | . | . | . | 2qH1 | rs29717031 | 0 | 1 |
| chr2 | 158324619 | C | T | 221 | Lbp | exonic | synonymous SNV | Lbp:NM_008489:exon10:c.C1092T:p.I364I | . | 2qH1 | rs27303797 | 0 | 1 |
| chr2 | 158325507 | A | T | 75 | Lbp | intronic | . | . | . | 2qH1 | rs27303785 | 0 | 1 |
| chr2 | 158325812 | A | G | 222 | Lbp | intronic | . | . | . | 2qH1 | rs33438101 | 0 | 1 |
| chr2 | 158328218 | A | G | 111 | Lbp | intronic | . | . | . | 2qH1 | rs27353952 | 0 | 1 |
| chr2 | 158361357 | G | A | 154 | Snhg17 | ncRNA_intronic | . | . | 276 | 2qH1 | rs27335221 | 0 | 1 |
| chr2 | 158361429 | G | A | 219 | Snhg17 | ncRNA_exonic | . | . | 276 | 2qH1 | rs33451565 | 0 | 1 |
| chr2 | 158361445 | G | A | 221 | Snhg17 | ncRNA_exonic | . | . | 276 | 2qH1 | rs33673341 | 0 | 1 |
| chr2 | 158361676 | C | G | 113 | . | upstream | . | . | . | 2qH1 | rs33280349 | 0 | 1 |
| chr2 | 158376181 | C | T | 87 | Snhg11 | UTR5 | . | . | . | 2qH1 | rs47664285 | 0 | 1 |
| chr2 | 158376410 | A | G | 152 | Snhg11 | intronic | . | . | . | 2qH1 | rs27335198 | 0 | 1 |
| chr2 | 158376827 | A | G | 145 | Snhg11 | intronic | . | . | . | 2qH1 | rs27335193 | 0 | 1 |
| chr2 | 158376830 | G | T | 123 | Snhg11 | intronic | . | . | . | 2qH1 | rs49707971 | 0 | 1 |
| chr2 | 158377039 | A | T | 222 | Snhg11 | exonic | missense SNV | Snhg11:NM_175692:exon3:c.A173T:p.Q58L | . | 2qH1 | rs27335191 | 0 | 1 |
| chr2 | 158377111 | G | A | 222 | Snhg11 | intronic | . | . | . | 2qH1 | rs231835056 | 0 | 1 |
| chr2 | 158380773 | A | G | 36.2822 | Snhg11 | exonic | missense SNV | Snhg11:NM_175692:exon4:c.A209G:p.N70S | . | 2qH1 | rs27335163 | 0 | 1 |
| chr2 | 158380817 | A | G | 54 | Snhg11 | exonic | missense SNV | Snhg11:NM_175692:exon4:c.A253G:p.I85V | . | 2qH1 | rs27335162 | 0 | 1 |
| chr2 | 158380830 | C | T | 43.1661 | Snhg11 | UTR3 | . | . | . | 2qH1 | rs27335161 | 0 | 1 |
| chr2 | 158380870 | G | T | 30.92 | Snhg11 | intronic | . | . | . | 2qH1 | rs27335160 | 0 | 1 |
| chr2 | 158380953 | A | G | 172 | Snhg11 | UTR3 | . | . | . | 2qH1 | rs27335159 | 0 | 1 |
| chr2 | 158381000 | G | A | 174 | Snhg11 | UTR3 | . | . | . | 2qH1 | rs27335158 | 0 | 1 |
| chr2 | 158420884 | G | A | 222 | Ralgapb | exonic | missense SNV | Ralgapb:NM_001291137:exon2:c.G41A:p.S14N,Ralgapb:NM_001291138:exon2:c.G41A:p.S14N | . | 2qH1 | rs33629994 | 0 | 1 |
| chr2 | 158436451 | T | C | 110 | Ralgapb | intronic | . | . | . | 2qH1 | rs33561455 | 0 | 1 |
| chr2 | 158443563 | T | C | 122 | Ralgapb | intronic | . | . | . | 2qH1 | rs27335014 | 0 | 1 |
| chr2 | 158444545 | A | T | 122 | Ralgapb | intronic | . | . | . | 2qH1 | rs33672811 | 0 | 1 |
| chr2 | 158444623 | A | G | 110 | Ralgapb | exonic | synonymous SNV | Ralgapb:NM_001291138:exon13:c.A1953G:p.K651K,Ralgapb:NM_001291137:exon14:c.A1989G:p.K663K | . | 2qH1 | rs33659533 | 0 | 1 |
| chr2 | 158446079 | G | A | 109 | Ralgapb | intronic | . | . | . | 2qH1 | rs27335009 | 0 | 1 |
| chr2 | 158450054 | C | G | 135 | Ralgapb | intronic | . | . | . | 2qH1 | rs32877082 | 0 | 1 |
| chr2 | 158454096 | G | A | 222 | Ralgapb | intronic | . | . | . | 2qH1 | rs27334994 | 0 | 1 |
| chr2 | 158461964 | C | T | 100 | Ralgapb | intronic | . | . | . | 2qH1 | rs248932253 | 0 | 1 |
| chr2 | 158462363 | G | A | 133 | Ralgapb | intronic | . | . | . | 2qH1 | rs27319656 | 0 | 1 |
| chr2 | 158463993 | G | A | 140 | Ralgapb | intronic | . | . | . | 2qH1 | rs27319651 | 0 | 1 |
| chr2 | 158466099 | A | G | 74 | Ralgapb | intronic | . | . | . | 2qH1 | rs29542482 | 0 | 1 |
| chr2 | 158466927 | A | G | 213 | Ralgapb | exonic | synonymous SNV | Ralgapb:NM_001291138:exon22:c.A3354G:p.L1118L,Ralgapb:NM_001291137:exon23:c.A3390G:p.L1130L | . | 2qH1 | rs51446454 | 0 | 1 |
| chr2 | 158473068 | G | A | 222 | Ralgapb | intronic | . | . | . | 2qH1 | rs27319629 | 0 | 1 |
| chr2 | 158473449 | A | G | 102 | Ralgapb | intronic | . | . | . | 2qH1 | rs27319626 | 0 | 1 |
| chr2 | 158477906 | G | A | 84 | Ralgapb | intronic | . | . | . | 2qH1 | rs50224071 | 0 | 1 |
| chr2 | 158477938 | G | T | 61 | Ralgapb | intronic | . | . | . | 2qH1 | rs27319597 | 0 | 1 |
| chr2 | 158482208 | T | C | 108 | Ralgapb | intronic | . | . | . | 2qH1 | rs27319584 | 0 | 1 |
| chr2 | 158492511 | G | A | 199 | Ralgapb | intronic | . | . | . | 2qH1 | rs27319562 | 0 | 1 |
| chr2 | 158494799 | G | T | 221 | Ralgapb | intronic | . | . | . | 2qH1 | rs27319556 | 0 | 1 |
| chr2 | 158495425 | C | T | 222 | Ralgapb | intronic | . | . | . | 2qH1 | rs27319555 | 0 | 1 |
| chr2 | 158505901 | C | T | 222 | Adig | exonic | synonymous SNV | Adig:NM_145635:exon2:c.C237T:p.H79H | . | 2qH1 | rs27319495 | 0 | 1 |
| chr2 | 158512687 | T | C | 26.9802 | . | upstream | . | . | . | 2qH1 | rs30626249 | 0 | 1 |
| chr2 | 158527282 | T | C | 204 | Arhgap40 | exonic | missense SNV | Arhgap40:NM_001145015:exon2:c.T323C:p.L108P | . | 2qH1 | rs33699443 | 0 | 1 |
| chr2 | 158527300 | G | C | 175 | Arhgap40 | intronic | . | . | . | 2qH1 | rs27304644 | 0 | 1 |
| chr2 | 158531321 | A | G | 222 | Arhgap40 | exonic | synonymous SNV | Arhgap40:NM_001145015:exon3:c.A498G:p.Q166Q | . | 2qH1 | rs13476869 | 0 | 1 |
| chr2 | 158539623 | C | T | 67 | Arhgap40 | intronic | . | . | . | 2qH1 | rs51675182 | 0 | 1 |
| chr2 | 158549761 | G | A | 186 | Arhgap40 | intronic | . | . | . | 2qH1 | rs33561233 | 0 | 1 |
| chr2 | 158550476 | A | G | 162 | Arhgap40 | intronic | . | . | . | 2qH1 | rs27304573 | 0 | 1 |
| chr2 | 158550594 | A | G | 221 | Arhgap40 | exonic | missense SNV | Arhgap40:NM_001145015:exon15:c.A1985G:p.H662R | . | 2qH1 | rs27304572 | 0 | 1 |
| chr2 | 158613870 | G | A | 102 | Slc32a1 | exonic | synonymous SNV | Slc32a1:NM_009508:exon2:c.G444A:p.G148G | 1151 | 2qH1 | rs32922079 | 0 | 1 |
| chr2 | 158614236 | T | C | 222 | Slc32a1 | exonic | synonymous SNV | Slc32a1:NM_009508:exon2:c.T810C:p.C270C | 1151 | 2qH1 | rs33535438 | 0 | 1 |
| chr2 | 158614272 | C | T | 222 | Slc32a1 | exonic | synonymous SNV | Slc32a1:NM_009508:exon2:c.C846T:p.I282I | 1151 | 2qH1 | rs27334958 | 0 | 1 |
| chr2 | 158614289 | G | T | 185 | Slc32a1 | exonic | missense SNV | Slc32a1:NM_009508:exon2:c.G863T:p.R288L | 1151 | 2qH1 | . | 0 | 1 |
| chr2 | 158614338 | G | A | 222 | Slc32a1 | exonic | synonymous SNV | Slc32a1:NM_009508:exon2:c.G912A:p.K304K | 1151 | 2qH1 | rs27334957 | 0 | 1 |
| chr2 | 158615327 | T | A | 103 | Slc32a1 | UTR3 | . | . | . | 2qH1 | rs50870034 | 0 | 1 |
| chr2 | 158615615 | C | T | 221 | Slc32a1 | UTR3 | . | . | . | 2qH1 | rs27334956 | 0 | 1 |
| chr2 | 158626210 | A | C | 82 | Actr5 | intronic | . | . | . | 2qH1 | rs47060458 | 0 | 1 |
| chr2 | 158626335 | T | C | 222 | Actr5 | exonic | synonymous SNV | Actr5:NM_175419:exon2:c.T384C:p.V128V | . | 2qH1 | rs52041887 | 0 | 1 |
| chr2 | 158626386 | T | C | 222 | Actr5 | exonic | synonymous SNV | Actr5:NM_175419:exon2:c.T435C:p.R145R | . | 2qH1 | rs48907902 | 0 | 1 |
| chr2 | 158626554 | G | A | 221 | Actr5 | exonic | synonymous SNV | Actr5:NM_175419:exon2:c.G603A:p.G201G | . | 2qH1 | rs51332345 | 0 | 1 |
| chr2 | 158626578 | T | C | 149 | Actr5 | intronic | . | . | . | 2qH1 | rs49357096 | 0 | 1 |
| chr2 | 158628005 | G | A | 59 | Actr5 | intronic | . | . | . | 2qH1 | rs51678856 | 0 | 1 |
| chr2 | 158628020 | T | C | 83 | Actr5 | intronic | . | . | . | 2qH1 | rs50561917 | 0 | 1 |
| chr2 | 158628144 | A | G | 183 | Actr5 | exonic | synonymous SNV | Actr5:NM_175419:exon3:c.A693G:p.P231P | . | 2qH1 | rs264033512 | 0 | 1 |
| chr2 | 158628165 | A | G | 187 | Actr5 | exonic | synonymous SNV | Actr5:NM_175419:exon3:c.A714G:p.T238T | . | 2qH1 | rs50526497 | 0 | 1 |
| chr2 | 158631624 | A | C | 159 | Actr5 | exonic | synonymous SNV | Actr5:NM_175419:exon4:c.A937C:p.R313R | . | 2qH1 | rs29561919 | 0 | 1 |
| chr2 | 158632144 | A | G | 111 | Actr5 | intronic | . | . | . | 2qH1 | rs33337680 | 0 | 1 |
| chr2 | 158632214 | T | C | 205 | Actr5 | intronic | . | . | . | 2qH1 | rs33098831 | 0 | 1 |
| chr2 | 158635330 | A | G | 32.9687 | Actr5 | exonic | synonymous SNV | Actr5:NM_175419:exon6:c.A1200G:p.E400E | . | 2qH1 | rs6303294 | 0 | 1 |
| chr2 | 158635420 | A | G | 86 | Actr5 | exonic | synonymous SNV | Actr5:NM_175419:exon6:c.A1290G:p.T430T | . | 2qH1 | rs6303829 | 0 | 1 |
| chr2 | 158635917 | A | G | 166 | Actr5 | intronic | . | . | . | 2qH1 | rs27334941 | 0 | 1 |
| chr2 | 158635960 | A | G | 222 | Actr5 | intronic | . | . | . | 2qH1 | rs27334939 | 0 | 1 |
| chr2 | 158638671 | C | T | 222 | Actr5 | exonic | synonymous SNV | Actr5:NM_175419:exon9:c.C1608T:p.Y536Y | . | 2qH1 | rs27334930 | 0 | 1 |
| chr2 | 158638855 | A | G | 222 | Actr5 | exonic | missense SNV | Actr5:NM_175419:exon9:c.A1792G:p.S598G | . | 2qH1 | rs13467411 | 0 | 1 |
| chr2 | 158740779 | T | C | 222 | Ppp1r16b | exonic | synonymous SNV | Ppp1r16b:NM_001159662:exon3:c.T294C:p.N98N,Ppp1r16b:NM_153089:exon3:c.T294C:p.N98N | . | 2qH1 | rs13476870 | 0 | 1 |
| chr2 | 158746507 | T | G | 38.4178 | Ppp1r16b | intronic | . | . | . | 2qH1 | rs51984145 | 0 | 1 |
| chr2 | 158746592 | C | T | 61 | Ppp1r16b | exonic | synonymous SNV | Ppp1r16b:NM_001159662:exon4:c.C396T:p.N132N,Ppp1r16b:NM_153089:exon4:c.C396T:p.N132N | . | 2qH1 | rs27320089 | 0 | 1 |
| chr2 | 158749131 | T | C | 61 | Ppp1r16b | intronic | . | . | . | 2qH1 | rs48601573 | 0 | 1 |
| chr2 | 158749138 | C | T | 118 | Ppp1r16b | intronic | . | . | . | 2qH1 | rs48888015 | 0 | 1 |
| chr2 | 158749263 | A | G | 123 | Ppp1r16b | exonic | synonymous SNV | Ppp1r16b:NM_001159662:exon5:c.A564G:p.A188A,Ppp1r16b:NM_153089:exon5:c.A564G:p.A188A | . | 2qH1 | rs27320070 | 0 | 1 |
| chr2 | 158756760 | T | C | 116 | Ppp1r16b | intronic | . | . | . | 2qH1 | rs27319994 | 0 | 1 |
| chr2 | 158756807 | T | C | 122 | Ppp1r16b | intronic | . | . | . | 2qH1 | rs27319992 | 0 | 1 |
| chr2 | 158761869 | T | G | 174 | Ppp1r16b | UTR3 | . | . | . | 2qH1 | rs27305565 | 0 | 1 |
| chr2 | 158767978 | A | G | 118 | . | upstream | . | . | . | 2qH1 | rs27305509 | 0 | 1 |
| chr2 | 158768087 | A | T | 222 | . | upstream | . | . | . | 2qH1 | rs27305508 | 0 | 1 |
| chr2 | 158768230 | G | T | 222 | Fam83d | exonic | synonymous SNV | Fam83d:NM_027975:exon1:c.G117T:p.L39L | 486 | 2qH1 | rs27305507 | 0 | 1 |
| chr2 | 158768242 | T | C | 210 | Fam83d | exonic | synonymous SNV | Fam83d:NM_027975:exon1:c.T129C:p.G43G | 486 | 2qH1 | rs27305506 | 0 | 1 |
| chr2 | 158768458 | T | C | 30.7116 | Fam83d | exonic | synonymous SNV | Fam83d:NM_027975:exon1:c.T345C:p.P115P | 486 | 2qH1 | rs27305502 | 0 | 1 |
| chr2 | 158779801 | C | T | 126 | Fam83d | intronic | . | . | . | 2qH1 | rs27305379 | 0 | 1 |
| chr2 | 158779825 | T | C | 148 | Fam83d | intronic | . | . | . | 2qH1 | rs27305378 | 0 | 1 |
| chr2 | 158779841 | A | G | 207 | Fam83d | splicing | . | . | . | 2qH1 | rs27305377 | 0 | 1 |
| chr2 | 158779950 | A | G | 221 | Fam83d | exonic | missense SNV | Fam83d:NM_027975:exon2:c.A580G:p.T194A | . | 2qH1 | rs27305376 | 0 | 1 |
| chr2 | 158780027 | A | G | 178 | Fam83d | intronic | . | . | . | 2qH1 | rs50886434 | 0 | 1 |
| chr2 | 158780047 | G | A | 139 | Fam83d | intronic | . | . | . | 2qH1 | rs27305374 | 0 | 1 |
| chr2 | 158783150 | A | C | 222 | Fam83d | exonic | synonymous SNV | Fam83d:NM_027975:exon3:c.A660C:p.T220T | . | 2qH1 | rs33044538 | 0 | 1 |
| chr2 | 158785001 | G | C | 65 | Fam83d | intronic | . | . | . | 2qH1 | rs27305327 | 0 | 1 |
| chr2 | 158785435 | C | T | 206 | Fam83d | exonic | missense SNV | Fam83d:NM_027975:exon4:c.C1043T:p.P348L | . | 2qH1 | rs27305323 | 0 | 1 |
| chr2 | 158785481 | T | C | 134 | Fam83d | exonic | synonymous SNV | Fam83d:NM_027975:exon4:c.T1089C:p.S363S | . | 2qH1 | rs27305322 | 0 | 1 |
| chr2 | 158785568 | C | T | 151 | Fam83d | exonic | synonymous SNV | Fam83d:NM_027975:exon4:c.C1176T:p.P392P | . | 2qH1 | rs33144460 | 0 | 1 |
| chr2 | 158785569 | A | G | 158 | Fam83d | exonic | missense SNV | Fam83d:NM_027975:exon4:c.A1177G:p.R393G | . | 2qH1 | rs27305321 | 0 | 1 |
| chr2 | 158785784 | A | G | 222 | Fam83d | exonic | synonymous SNV | Fam83d:NM_027975:exon4:c.A1392G:p.S464S | . | 2qH1 | rs27305319 | 0 | 1 |
| chr2 | 158806777 | G | T | 155 | Dhx35 | intronic | . | . | . | 2qH1 | rs28299796 | 0 | 1 |
| chr2 | 158817407 | G | A | 221 | Dhx35 | exonic | synonymous SNV | Dhx35:NM_001291144:exon6:c.G30A:p.P10P,Dhx35:NM_145742:exon6:c.G495A:p.P165P | . | 2qH1 | rs48759129 | 0 | 1 |
| chr2 | 158817467 | A | G | 139 | Dhx35 | intronic | . | . | . | 2qH1 | rs28299772 | 0 | 1 |
| chr2 | 158817472 | G | C | 124 | Dhx35 | intronic | . | . | . | 2qH1 | rs50647833 | 0 | 1 |
| chr2 | 158818206 | T | C | 151 | Dhx35 | intronic | . | . | . | 2qH1 | rs252278665 | 0 | 1 |
| chr2 | 158818220 | A | G | 221 | Dhx35 | intronic | . | . | . | 2qH1 | rs218804617 | 0 | 1 |
| chr2 | 158818231 | C | T | 221 | Dhx35 | intronic | . | . | . | 2qH1 | rs234001177 | 0 | 1 |
| chr2 | 158818417 | T | C | 176 | Dhx35 | intronic | . | . | . | 2qH1 | rs28282657 | 0 | 1 |
| chr2 | 158818467 | C | T | 120 | Dhx35 | intronic | . | . | . | 2qH1 | rs28282656 | 0 | 1 |
| chr2 | 158820531 | G | A | 185 | Dhx35 | intronic | . | . | . | 2qH1 | rs28282644 | 0 | 1 |
| chr2 | 158820570 | T | C | 23.5693 | Dhx35 | intronic | . | . | . | 2qH1 | rs28282643 | 0 | 1 |
| chr2 | 158826507 | A | G | 103 | Dhx35 | intronic | . | . | . | 2qH1 | rs47986706 | 0 | 1 |
| chr2 | 158826510 | A | T | 80 | Dhx35 | intronic | . | . | . | 2qH1 | rs48201850 | 0 | 1 |
| chr2 | 158828091 | T | C | 107 | Dhx35 | intronic | . | . | . | 2qH1 | rs32958964 | 0 | 1 |
| chr2 | 158829335 | T | C | 92 | Dhx35 | intronic | . | . | . | 2qH1 | rs28282544 | 0 | 1 |
| chr2 | 158831927 | G | A | 191 | Dhx35 | intronic | . | . | . | 2qH1 | rs33268019 | 0 | 1 |
| chr2 | 158834726 | C | T | 86 | Dhx35 | intronic | . | . | . | 2qH1 | rs33746332 | 0 | 1 |
| chr2 | 158834949 | A | G | 222 | Dhx35 | exonic | synonymous SNV | Dhx35:NM_001291144:exon13:c.A837G:p.L279L,Dhx35:NM_145742:exon13:c.A1302G:p.L434L | . | 2qH1 | rs28282477 | 0 | 1 |
| chr2 | 158836605 | C | G | 222 | Dhx35 | intronic | . | . | . | 2qH1 | rs28282453 | 0 | 1 |
| chr2 | 158836719 | A | C | 222 | Dhx35 | intronic | . | . | . | 2qH1 | rs28282452 | 0 | 1 |
| chr2 | 158840354 | C | T | 51 | Dhx35 | intronic | . | . | . | 2qH1 | rs28282434 | 0 | 1 |
| chr2 | 158840761 | T | C | 51 | Dhx35 | intronic | . | . | . | 2qH1 | rs49015318 | 0 | 1 |
| chr2 | 158842157 | A | G | 70 | Dhx35 | intronic | . | . | . | 2qH1 | rs28282420 | 0 | 1 |
| chr2 | 158842164 | C | A | 86 | Dhx35 | intronic | . | . | . | 2qH1 | rs49556565 | 0 | 1 |
| chr2 | 158842236 | A | G | 117 | Dhx35 | intronic | . | . | . | 2qH1 | rs46856683 | 0 | 1 |
| chr2 | 158842243 | A | G | 114 | Dhx35 | intronic | . | . | . | 2qH1 | rs51759936 | 0 | 1 |
| chr2 | 158842249 | C | T | 189 | Dhx35 | intronic | . | . | . | 2qH1 | rs50879002 | 0 | 1 |
| chr2 | 158842409 | G | A | 222 | Dhx35 | intronic | . | . | . | 2qH1 | rs48472720 | 0 | 1 |
| chr2 | 158842596 | T | G | 26.0626 | Dhx35 | intronic | . | . | . | 2qH1 | rs28282416 | 0 | 1 |
| chr2 | 158846826 | A | G | 135 | Dhx35 | intronic | . | . | . | 2qH1 | rs28282385 | 0 | 1 |
| chr2 | 158846873 | G | A | 221 | Dhx35 | intronic | . | . | . | 2qH1 | rs33134754 | 0 | 1 |
| chr2 | 158851591 | G | T | 93 | Dhx35 | intronic | . | . | . | 2qH1 | rs29910095 | 0 | 1 |
| chr2 | 159778067 | C | A | 27.6678 | . | intergenic | . | . | . | 2qH2 | rs27335940 | 0 | 1 |
| chr2 | 159778074 | C | T | 53 | . | intergenic | . | . | . | 2qH2 | rs27335939 | 0 | 1 |
| chr2 | 160619886 | T | C | 222 | . | intergenic | . | . | . | 2qH2 | rs29535722 | 0 | 1 |
| chr2 | 160705337 | G | T | 221 | Top1 | intronic | . | . | . | 2qH2 | . | 0 | 1 |
| chr2 | 160751644 | G | A | 189 | Plcg1 | exonic | missense SNV | Plcg1:NM_021280:exon9:c.G728A:p.R243H | . | 2qH2 | rs27303584 | 0 | 1 |
| chr2 | 160893945 | T | C | 222 | Lpin3 | intronic | . | . | . | 2qH2 | rs27334515 | 0 | 1 |
| chr2 | 160899485 | C | A | 160 | Lpin3 | intronic | . | . | . | 2qH2 | . | 0 | 1 |
| chr2 | 160904065 | T | C | 222 | Lpin3 | intronic | . | . | . | 2qH2 | rs27334450 | 0 | 1 |

| | | | | | | | | | | | | | |
|---|---|---|---|---|---|---|---|---|---|---|---|---|---|
| chr2 | 160904718 | T | C | 208 | Lpin3 | intronic | . | . | . | 2qH2 | rs27334446 | 0 | 1 |
| chr2 | 160909625 | C | T | 178 | Emilin3 | exonic | missense SNV | Emilin3:NM_001291145:exon3:c.G493A:p.E165K,Emilin3:NM_182840:exon3:c.G493A:p.E165K | . | 2qH2 | rs51242343 | 0 | 1 |
| chr2 | 160961736 | G | A | 145 | Chd6 | intronic | . | . | . | 2qH2 | rs27319950 | 0 | 1 |
| chr2 | 160983925 | G | T | 222 | Chd6 | intronic | . | . | . | 2qH2 | rs27319877 | 0 | 1 |
| chr2 | 160988168 | A | G | 108 | Chd6 | intronic | . | . | . | 2qH2 | rs32949333 | 0 | 1 |
| chr2 | 161039435 | C | G | 127 | Chd6 | intronic | . | . | . | 2qH2 | rs27319799 | 0 | 1 |
| chr2 | 161587576 | T | C | 222 | Ptprt | exonic | missense SNV | Ptprt:NM_001291149:exon17:c.A2723G:p.N908S,Ptprt:NM_021464:exon17:c.A2723G:p.N908S,Ptprt:NM_( | . | 2qH2 | . | 0 | 1 |
| chr2 | 161656060 | T | C | 168 | Ptprt | intronic | . | . | . | 2qH2 | . | 0 | 1 |
| chr2 | 161686951 | A | G | 192 | Ptprt | intronic | . | . | . | 2qH2 | . | 0 | 1 |
| chr2 | 161723515 | C | T | 59 | Ptprt | intronic | . | . | . | 2qH2 | rs27333627 | 0 | 1 |
| chr2 | 161723543 | C | G | 121 | Ptprt | intronic | . | . | . | 2qH2 | rs27333626 | 0 | 1 |
| chr2 | 161723579 | A | C | 115 | Ptprt | intronic | . | . | . | 2qH2 | rs51278233 | 0 | 1 |
| chr2 | 161723755 | C | T | 222 | Ptprt | intronic | . | . | . | 2qH2 | rs27333625 | 0 | 1 |
| chr2 | 161723830 | T | A | 191 | Ptprt | intronic | . | . | . | 2qH2 | rs46717736 | 0 | 1 |
| chr2 | 161732824 | T | C | 131 | Ptprt | intronic | . | . | . | 2qH2 | rs48540658 | 0 | 1 |
| chr2 | 161732838 | A | T | 166 | Ptprt | intronic | . | . | . | 2qH2 | rs50321159 | 0 | 1 |
| chr2 | 161732928 | G | A | 175 | Ptprt | intronic | . | . | . | 2qH2 | rs48949120 | 0 | 1 |
| chr2 | 161733069 | T | C | 96 | Ptprt | intronic | . | . | . | 2qH2 | rs49069564 | 0 | 1 |
| chr2 | 161766064 | T | C | 92 | Ptprt | intronic | . | . | . | 2qH2 | rs33733886 | 0 | 1 |
| chr2 | 161766180 | T | A | 206 | Ptprt | intronic | . | . | . | 2qH2 | rs27319054 | 0 | 1 |
| chr2 | 161810323 | T | C | 69 | Ptprt | intronic | . | . | . | 2qH2 | rs27318902 | 0 | 1 |
| chr2 | 161810426 | A | T | 101 | Ptprt | intronic | . | . | . | 2qH2 | rs51657452 | 0 | 1 |
| chr2 | 161810680 | G | A | 221 | Ptprt | intronic | . | . | . | 2qH2 | rs48023903 | 0 | 1 |
| chr2 | 161810736 | C | T | 168 | Ptprt | intronic | . | . | . | 2qH2 | rs49759864 | 0 | 1 |
| chr2 | 161811834 | G | A | 99 | Ptprt | intronic | . | . | . | 2qH2 | rs27318890 | 0 | 1 |
| chr2 | 161901246 | C | T | 26.941 | Ptprt | intronic | . | . | . | 2qH2 | rs27332849 | 0 | 1 |
| chr2 | 161901249 | T | C | 26.9476 | Ptprt | intronic | . | . | . | 2qH2 | rs27332848 | 0 | 1 |
| chr2 | 161901381 | G | T | 125 | Ptprt | intronic | . | . | . | 2qH2 | rs27332846 | 0 | 1 |
| chr2 | 161901406 | A | C | 174 | Ptprt | intronic | . | . | . | 2qH2 | rs27332845 | 0 | 1 |
| chr2 | 161901587 | A | G | 120 | Ptprt | intronic | . | . | . | 2qH2 | rs46214802 | 0 | 1 |
| chr2 | 161901599 | G | A | 60 | Ptprt | intronic | . | . | . | 2qH2 | rs48572395 | 0 | 1 |
| chr2 | 161927468 | C | T | 63 | Ptprt | intronic | . | . | . | 2qH2 | rs33341102 | 0 | 1 |
| chr2 | 161927492 | T | A | 98 | Ptprt | exonic | synonymous SNV | Ptprt:NM_001291149:exon8:c.A1452T:p.T484T,Ptprt:NM_001291150:exon8:c.A1452T:p.T484T,Ptprt:NM | . | 2qH2 | rs45921850 | 0 | 1 |
| chr2 | 161927495 | C | T | 103 | Ptprt | exonic | synonymous SNV | Ptprt:NM_001291149:exon8:c.G1449A:p.Q483Q,Ptprt:NM_001291150:exon8:c.G1449A:p.Q483Q,Ptprt:NM | . | 2qH2 | rs48721677 | 0 | 1 |
| chr2 | 161927717 | C | T | 221 | Ptprt | exonic | synonymous SNV | Ptprt:NM_001291149:exon8:c.G1227A:p.L409L,Ptprt:NM_001291150:exon8:c.G1227A:p.L409L,Ptprt:NM | . | 2qH2 | rs46435606 | 0 | 1 |
| chr2 | 161927762 | C | T | 161 | Ptprt | exonic | synonymous SNV | Ptprt:NM_001291149:exon8:c.G1182A:p.P394P,Ptprt:NM_001291150:exon8:c.G1182A:p.P394P,Ptprt:NM | . | 2qH2 | rs49622103 | 0 | 1 |
| chr2 | 161927795 | C | T | 84 | Ptprt | intronic | . | . | . | 2qH2 | rs33668875 | 0 | 1 |
| chr2 | 162135119 | T | A | 74 | Ptprt | intronic | . | . | . | 2qH2 | rs33196397 | 0 | 1 |
| chr2 | 162135264 | C | T | 209 | Ptprt | exonic | synonymous SNV | Ptprt:NM_001291149:exon7:c.G1113A:p.E371E,Ptprt:NM_001291150:exon7:c.G1113A:p.E371E,Ptprt:NM | . | 2qH2 | rs29554145 | 0 | 1 |
| chr2 | 162135401 | G | A | 221 | Ptprt | exonic | synonymous SNV | Ptprt:NM_001291149:exon7:c.C976T:p.L326L,Ptprt:NM_001291150:exon7:c.C976T:p.L326L,Ptprt:NM_0( | . | 2qH2 | rs27333820 | 0 | 1 |
| chr2 | 162135608 | C | A | 68 | Ptprt | intronic | . | . | . | 2qH2 | rs52284271 | 0 | 1 |
| chr2 | 162135609 | A | G | 70 | Ptprt | intronic | . | . | . | 2qH2 | rs52116617 | 0 | 1 |
| chr2 | 162665840 | G | A | 221 | 9430021M05Rik | ncRNA_intronic | . | . | . | 2qH2 | rs29626173 | 0 | 1 |
| chr2 | 162665842 | A | G | 221 | 9430021M05Rik | ncRNA_intronic | . | . | . | 2qH2 | rs33471374 | 0 | 1 |
| chr2 | 162948169 | T | C | 222 | L3mbtl1 | exonic | synonymous SNV | L3mbtl1:NM_001081338:exon5:c.T363C:p.N121N | . | 2qH2 | rs27347044 | 0 | 1 |
| chr2 | 162949413 | C | A | 150 | L3mbtl1 | intronic | . | . | . | 2qH2 | rs27347036 | 0 | 1 |
| chr2 | 162959406 | A | G | 80 | L3mbtl1 | intronic | . | . | . | 2qH2 | rs27346996 | 0 | 1 |
| chr2 | 162960802 | C | A | 67 | L3mbtl1 | intronic | . | . | . | 2qH2 | rs51856500 | 0 | 1 |
| chr2 | 162961051 | G | A | 80 | L3mbtl1 | exonic | synonymous SNV | L3mbtl1:NM_001081338:exon11:c.G1113A:p.E371E | . | 2qH2 | rs27346995 | 0 | 1 |
| chr2 | 163247894 | G | C | 222 | Tox2 | intronic | . | . | . | 2qH2 | rs28279292 | 0 | 1 |
| chr2 | 163415651 | T | A | 221 | Oser1 | UTR5 | . | . | . | 2qH3 | . | 0 | 1 |
| chr2 | 163607687 | G | A | 141 | Ttpal | intronic | . | . | . | 2qH3 | rs32827461 | 0 | 1 |
| chr2 | 163625452 | C | A | 221 | Serinc3 | exonic | missense SNV | Serinc3:NM_012032:exon10:c.G1408T:p.D470Y | . | 2qH3 | . | 0 | 1 |
| chr2 | 163732455 | C | T | 222 | Ada | intronic | . | . | . | 2qH3 | rs28246715 | 0 | 1 |
| chr2 | 163828793 | T | C | 128 | Wisp2 | intronic | . | . | . | 2qH3 | rs27315873 | 0 | 1 |
| chr2 | 163828883 | T | C | 173 | Wisp2 | exonic | synonymous SNV | Wisp2:NM_016873:exon4:c.T309C:p.N103N | . | 2qH3 | rs27315872 | 0 | 1 |
| chr2 | 163829068 | A | G | 207 | Wisp2 | exonic | missense SNV | Wisp2:NM_016873:exon4:c.A494G:p.Q165R | . | 2qH3 | rs27315871 | 0 | 1 |
| chr2 | 163832284 | C | T | 118 | Wisp2 | exonic | synonymous SNV | Wisp2:NM_016873:exon5:c.C603T:p.A201A | . | 2qH3 | rs29515253 | 0 | 1 |
| chr2 | 163832446 | C | T | 221 | Wisp2 | UTR3 | . | . | . | 2qH3 | rs27315857 | 0 | 1 |
| chr2 | 163853872 | T | C | 64 | Kcnk15 | exonic | synonymous SNV | Kcnk15:NM_001030292:exon1:c.T123C:p.G41G | 432 | 2qH3 | rs45897734 | 0 | 1 |
| chr2 | 163853878 | T | G | 45.8582 | Kcnk15 | exonic | synonymous SNV | Kcnk15:NM_001030292:exon1:c.T129G:p.A43A | 432 | 2qH3 | rs212611772 | 0 | 1 |
| chr2 | 163853881 | T | C | 39.2094 | Kcnk15 | exonic | synonymous SNV | Kcnk15:NM_001030292:exon1:c.T132C:p.V44V | 432 | 2qH3 | rs237721663 | 0 | 1 |
| chr2 | 163879012 | A | G | 74 | Rims4 | intronic | . | . | . | 2qH3 | rs32833643 | 0 | 1 |
| chr2 | 163879241 | C | G | 23.2875 | Rims4 | intronic | . | . | . | 2qH3 | rs33742903 | 0 | 1 |
| chr2 | 164014231 | A | G | 222 | Ywhab | intronic | . | . | . | 2qH3 | rs27317491 | 0 | 1 |
| chr2 | 164016261 | G | A | 222 | Ywhab | intronic | . | . | . | 2qH3 | rs33687274 | 0 | 1 |
| chr2 | 164016575 | A | G | 168 | Ywhab | intronic | . | . | . | 2qH3 | rs27317476 | 0 | 1 |
| chr2 | 164031125 | G | A | 111 | Pabpc1l | intronic | . | . | . | 2qH3 | rs32964676 | 0 | 1 |
| chr2 | 164031340 | G | A | 222 | Pabpc1l | intronic | . | . | . | 2qH3 | rs29860111 | 0 | 1 |
| chr2 | 164032600 | T | C | 222 | Pabpc1l | intronic | . | . | . | 2qH3 | rs51318649 | 0 | 1 |
| chr2 | 164037080 | A | C | 222 | Pabpc1l | intronic | . | . | . | 2qH3 | rs27317434 | 0 | 1 |
| chr2 | 164043618 | A | G | 177 | Pabpc1l | exonic | synonymous SNV | Pabpc1l:NM_001114079:exon9:c.A1290G:p.T430T | . | 2qH3 | rs27317408 | 0 | 1 |
| chr2 | 164044334 | G | C | 180 | Pabpc1l | exonic | missense SNV | Pabpc1l:NM_001114079:exon10:c.G1383C:p.R461S | . | 2qH3 | rs47358980 | 0 | 1 |
| chr2 | 164058842 | A | C | 221 | Tomm34 | intronic | . | . | . | 2qH3 | rs27317383 | 0 | 1 |
| chr2 | 164083717 | G | T | 222 | Stk4 | exonic | missense SNV | Stk4:NM_021420:exon3:c.G204T:p.Q68H | . | 2qH3 | . | 0 | 1 |
| chr2 | 164096725 | C | T | 221 | Stk4 | intronic | . | . | . | 2qH3 | . | 0 | 1 |
| chr2 | 164099882 | A | G | 222 | Stk4 | intronic | . | . | . | 2qH3 | rs27300422 | 0 | 1 |
| chr2 | 164100691 | C | A | 87 | Stk4 | intronic | . | . | . | 2qH3 | rs27300410 | 0 | 1 |
| chr2 | 164237750 | A | G | 221 | Svs2 | exonic | missense SNV | Svs2:NM_017390:exon2:c.T236C:p.M79T | . | 2qH3 | . | 0 | 1 |
| chr2 | 164356052 | T | G | 117 | Slpi | intronic | . | . | . | 2qH3 | . | 0 | 1 |
| chr2 | 164393445 | C | T | 113 | Matn4 | exonic | synonymous SNV | Matn4:NM_001252563:exon8:c.G1206A:p.Q402Q,Matn4:NM_013592:exon8:c.G1206A:p.Q402Q | 290 | 2qH3 | . | 0 | 1 |
| chr2 | 164405772 | C | A | 219 | Rbpjl | exonic | synonymous SNV | Rbpjl:NM_009036:exon3:c.C144A:p.A48A | . | 2qH3 | . | 0 | 1 |
| chr2 | 164501250 | C | G | 222 | Pigt | intronic | . | . | . | 2qH3 | . | 0 | 1 |
| chr2 | 164747388 | G | T | 79 | Dnttip1 | intronic | . | . | . | 2qH3 | . | 0 | 1 |
| chr2 | 165382853 | C | A | 221 | Zfp334 | intronic | . | . | . | 2qH3 | . | 0 | 1 |
| chr2 | 165445826 | A | G | 170 | Slc13a3 | intronic | . | . | . | 2qH3 | rs27345176 | 1 | 0 |
| chr2 | 166867227 | A | T | 222 | Arfgef2 | intronic | . | . | . | 2qH3 | . | 0 | 1 |
| chr2 | 166894619 | C | A | 160 | Arfgef2 | exonic | missense SNV | Arfgef2:NM_001085495:exon39:c.C5210A:p.A1737E | . | 2qH3 | . | 0 | 1 |
| chr2 | 166952594 | A | T | 151 | Stau1 | intronic | . | . | . | 2qH3 | rs261036619 | 0 | 1 |
| chr2 | 167020111 | G | C | 136 | Ddx27 | intronic | . | . | . | 2qH3 | . | 0 | 1 |
| chr2 | 167307516 | C | T | 127 | B4galt5 | intronic | . | . | . | 2qH3 | . | 0 | 1 |
| chr2 | 168675054 | G | A | 214 | Atp9a | intronic | . | . | . | 2qH3 | . | 0 | 1 |
| chr2 | 168705182 | C | G | 221 | Atp9a | exonic | missense SNV | Atp9a:NM_001289445:exon4:c.G329C:p.R110P,Atp9a:NM_001289446:exon4:c.G323C:p.R108P,Atp9a:NM_0] | . | 2qH3 | . | 0 | 1 |
| chr2 | 168900005 | A | T | 221 | . | intergenic | . | . | . | 2qH3 | . | 0 | 1 |
| chr2 | 169884615 | A | G | 222 | Tshz2 | exonic | missense SNV | Tshz2:NM_080455:exon2:c.A1130G:p.K377R | . | 2qH3 | . | 0 | 1 |
| chr2 | 172879550 | A | T | 222 | Bmp7 | intronic | . | . | . | 2qH3 | . | 0 | 1 |
| chr2 | 173112314 | C | A | 222 | Ctcfl | exonic | missense SNV | Ctcfl:NM_001081387:exon5:c.G989T:p.R330L | . | 2qH3 | . | 0 | 1 |
| chr2 | 174644132 | G | A | 222 | Zfp831 | exonic | missense SNV | Zfp831:NM_001099328:exon1:c.G599A:p.R200H | . | 2qH4 | . | 0 | 1 |
| chr2 | 174645743 | G | C | 222 | Zfp831 | exonic | missense SNV | Zfp831:NM_001099328:exon1:c.G2210C:p.G737A | . | 2qH4 | . | 0 | 1 |
| chr2 | 174705252 | A | C | 221 | Zfp831 | exonic | synonymous SNV | Zfp831:NM_001099328:exon5:c.A4227C:p.P1409P | . | 2qH4 | . | 0 | 1 |
| chr2 | 178269181 | T | C | 77 | Phactr3 | intronic | . | . | . | 2qH4 | rs250646823 | 0 | 1 |
| chr2 | 178283038 | C | A | 104 | Phactr3 | exonic | missense SNV | Phactr3:NM_001007154:exon6:c.C657A:p.S219R,Phactr3:NM_001177789:exon6:c.C765A:p.S255R,Phactr3 | . | 2qH4 | . | 0 | 1 |
| chr2 | 178351673 | C | G | 221 | Sycp2 | splicing | . | . | . | 2qH4 | . | 0 | 1 |
| chr2 | 178393762 | A | G | 40.9052 | Sycp2 | intronic | . | . | . | 2qH4 | . | 0 | 1 |
| chr2 | 178449901 | A | T | 197 | Cdh26 | exonic | missense SNV | Cdh26:NM_001291189:exon3:c.A252T:p.E84D,Cdh26:NM_198656:exon3:c.A252T:p.E84D | . | 2qH4 | . | 0 | 1 |
| chr2 | 178460853 | A | G | 222 | Cdh26 | intronic | . | . | . | 2qH4 | . | 0 | 1 |
| chr2 | 178466514 | C | A | 129 | Cdh26 | intronic | . | . | . | 2qH4 | . | 0 | 1 |
| chr2 | 178472192 | T | C | 49.9427 | Cdh26 | intronic | . | . | . | 2qH4 | . | 0 | 1 |
| chr2 | 179886050 | C | G | 110 | Cdh4 | exonic | missense SNV | Cdh4:NM_009867:exon10:c.C1562G:p.T521R | . | 2qH4 | . | 0 | 1 |
| chr2 | 179923990 | A | C | 179 | Taf4a | exonic | missense SNV | Taf4a:NM_001081092:exon11:c.T2647G:p.F883V | . | 2qH4 | . | 0 | 1 |
| chr2 | 179926111 | C | T | 102 | Taf4a | intronic | . | . | . | 2qH4 | . | 0 | 1 |
| chr2 | 180057470 | G | T | 60 | Ss18l1 | intronic | . | . | . | 2qH4 | . | 0 | 1 |
| chr2 | 180185896 | C | A | 187 | Lama5 | exonic | missense SNV | Lama5:NM_001081171:exon49:c.G6572T:p.R2191L | . | 2qH4 | . | 0 | 1 |
| chr2 | 180201550 | C | A | 221 | Lama5 | intronic | . | . | . | 2qH4 | . | 0 | 1 |
| chr2 | 180474128 | G | A | 39.7005 | Slco4a1 | exonic | missense SNV | Slco4a1:NM_148933:exon12:c.G2056A:p.A686T | . | 2qH4 | rs226716326 | 0 | 1 |
| chr2 | 180622637 | A | T | 222 | Tcf15 | exonic | synonymous SNV | Tcf15:NM_178254:exon6:c.T1425A:p.P475P | . | 2qH4 | . | 0 | 1 |
| chr2 | 181037203 | C | A | 50 | Chrna4 | intronic | . | . | . | 2qH4 | . | 0 | 1 |
| chr2 | 181150969 | G | T | 222 | Eef1a2 | exonic | missense SNV | Eef1a2:NM_007906:exon5:c.C722A:p.P241H | . | 2qH4 | . | 0 | 1 |
| chr2 | 181350754 | C | A | 221 | Rtel1 | intronic | . | . | . | 2qH4 | . | 0 | 1 |
| chr2 | 181587152 | C | A | 210 | Zfp512b | exonic | synonymous SNV | Zfp512b:NM_001164597:exon13:c.G1989T:p.S663S | . | 2qH4 | . | 0 | 1 |
| chr2 | 181697049 | G | T | 221 | Lkaaear1 | exonic | missense SNV | Lkaaear1:NM_199023:exon3:c.C493A:p.Q165K | . | 2qH4 | . | 0 | 1 |
| chr2 | 181802744 | G | T | 20.338 | Myt1 | intronic | . | . | . | 2qH4 | . | 0 | 1 |
| chr2 | 181825825 | G | T | 128 | Myt1 | intronic | . | . | . | 2qH4 | . | 0 | 1 |
| chr3 | 4891255 | A | G | 196 | . | intergenic | . | . | . | 3qA1 | . | 0 | 1 |
| chr3 | 5242544 | T | C | 199 | Zfhx4 | exonic | synonymous SNV | Zfhx4:NM_030708:exon2:c.T829C:p.L277L | . | 3qA1 | . | 0 | 1 |
| chr3 | 5403731 | A | G | 222 | Zfhx4 | exonic | missense SNV | Zfhx4:NM_030708:exon10:c.A8948G:p.Y2983C | . | 3qA1 | . | 0 | 1 |
| chr3 | 5411600 | G | A | 87 | Zfhx4 | intronic | . | . | . | 3qA1 | . | 0 | 1 |
| chr3 | 5411676 | T | A | 101 | Zfhx4 | intronic | . | . | . | 3qA1 | . | 0 | 1 |
| chr3 | 5412019 | T | C | 221 | Zfhx4 | exonic | synonymous SNV | Zfhx4:NM_030708:exon11:c.T9618C:p.T3206T | . | 3qA1 | . | 0 | 1 |
| chr3 | 6378495 | T | G | 91 | . | intergenic | . | . | . | 3qA1 | . | 0 | 1 |
| chr3 | 7518414 | T | G | 195 | Zc2hc1a | exonic | synonymous SNV | Zc2hc1a:NM_173181:exon3:c.T144G:p.T48T | . | 3qA1 | . | 0 | 1 |
| chr3 | 7538967 | A | C | 134 | Zc2hc1a | intronic | . | . | . | 3qA1 | . | 0 | 1 |
| chr3 | 8615812 | C | T | 98 | . | intergenic | . | . | . | 3qA1 | . | 0 | 1 |
| chr3 | 8931132 | C | T | 222 | Tpd52 | exonic | missense SNV | Tpd52:NM_001025262:exon6:c.G595A:p.E199K,Tpd52:NM_009412:exon6:c.G478A:p.E160K,Tpd52:NM_0010 | . | 3qA1 | . | 0 | 1 |
| chr3 | 10088675 | C | T | 104 | Gm9833 | ncRNA_exonic | . | . | . | 3qA1 | . | 0 | 1 |
| chr3 | 10246057 | G | A | 221 | Fabp12 | intronic | . | . | . | 3qA1 | rs235520439 | 0 | 1 |
| chr3 | 13776893 | C | G | 170 | Ralyl | exonic | missense SNV | Ralyl:NM_001163328:exon2:c.C46G:p.P16A,Ralyl:NM_178631:exon3:c.C46G:p.P16A | . | 3qA1 | . | 0 | 1 |
| chr3 | 13777058 | G | A | 104 | Ralyl | exonic | missense SNV | Ralyl:NM_001163328:exon2:c.G211A:p.A71T,Ralyl:NM_178631:exon3:c.G211A:p.A71T | . | 3qA1 | . | 0 | 1 |
| chr3 | 14558523 | A | C | 35.8713 | Lrrcc1 | intronic | . | . | . | 3qA1 | . | 0 | 1 |
| chr3 | 14603683 | T | G | 66 | E2f5 | exonic | missense SNV | E2f5:NM_007892:exon6:c.T631G:p.S211A | . | 3qA1 | . | 0 | 1 |
| chr3 | 14767625 | A | T | 104 | Car1 | intronic | . | . | . | 3qA1 | . | 0 | 1 |
| chr3 | 14973741 | A | G | 222 | A930001A20Rik | ncRNA_intronic | . | . | . | 3qA1 | . | 0 | 1 |
| chr3 | 15332064 | C | T | 51 | Gm9733 | intronic | . | . | . | 3qA1 | rs107637603 | 0 | 1 |
| chr3 | 15332139 | A | G | 222 | Gm9733 | intronic | . | . | . | 3qA1 | rs48399961 | 0 | 1 |
| chr3 | 15332145 | C | T | 222 | Gm9733 | intronic | . | . | . | 3qA1 | rs108902508 | 0 | 1 |
| chr3 | 15332332 | A | G | 222 | . | upstream | . | . | . | 3qA1 | rs50041124 | 0 | 1 |
| chr3 | 15332381 | A | G | 155 | . | upstream | . | . | . | 3qA1 | rs48482535 | 0 | 1 |
| chr3 | 15514515 | C | T | 199 | Sirpb1b | intronic | . | . | . | 3qA1 | . | 0 | 1 |
| chr3 | 18097829 | C | A | 221 | Cyp7b1 | intronic | . | . | . | 3qA1 | . | 0 | 1 |
| chr3 | 18103812 | T | G | 143 | Cyp7b1 | exonic | missense SNV | Cyp7b1:NM_007825:exon2:c.A142C:p.K48Q | . | 3qA1 | . | 0 | 1 |
| chr3 | 19768791 | C | T | 55 | . | intergenic | . | . | . | 3qA2 | . | 0 | 1 |
| chr3 | 19895724 | C | T | 27.1806 | 4632415L05Rik | ncRNA_exonic | . | . | . | 3qA2 | . | 0 | 1 |
| chr3 | 22192255 | T | A | 220 | Tbl1xr1 | exonic | synonymous SNV | Tbl1xr1:NM_030732:exon9:c.T852A:p.A284A | . | 3qA3 | . | 0 | 1 |
| chr3 | 22525324 | G | T | 222 | . | intergenic | . | . | . | 3qA3 | . | 0 | 1 |
| chr3 | 23805025 | T | C | 221 | . | intergenic | . | . | . | 3qA3 | . | 0 | 1 |
| chr3 | 23869476 | G | C | 78 | . | intergenic | . | . | . | 3qA3 | . | 0 | 1 |
| chr3 | 25433966 | T | C | 163 | Nlgn1 | exonic | missense SNV | Nlgn1:NM_001163387:exon6:c.A2117G:p.Q706R,Nlgn1:NM_138666:exon8:c.A2204G:p.Q735R | . | 3qA3 | . | 0 | 1 |
| chr3 | 25435722 | C | T | 123 | Nlgn1 | intronic | . | . | . | 3qA3 | . | 0 | 1 |
| chr3 | 25436545 | T | G | 82 | Nlgn1 | exonic | missense SNV | Nlgn1:NM_001163387:exon5:c.A930C:p.K310N,Nlgn1:NM_138666:exon7:c.A1017C:p.K339N | . | 3qA3 | . | 0 | 1 |
| chr3 | 25439985 | A | C | 74 | Nlgn1 | exonic | missense SNV | Nlgn1:NM_001163387:exon4:c.T739G:p.F247V,Nlgn1:NM_138666:exon6:c.T799G:p.F267V | . | 3qA3 | . | 0 | 1 |
| chr3 | 25920370 | G | T | 102 | Nlgn1 | intronic | . | . | . | 3qA3 | . | 0 | 1 |
| chr3 | 27374536 | T | C | 25.8967 | Ghsr | intronic | . | . | . | 3qA3 | . | 0 | 1 |
| chr3 | 27542177 | A | G | 222 | Fndc3b | splicing | . | . | . | 3qA3 | . | 0 | 1 |
| chr3 | 27620670 | A | G | 221 | Fndc3b | exonic | missense SNV | Fndc3b:NM_173182:exon3:c.T122C:p.V41A | . | 3qA3 | . | 0 | 1 |
| chr3 | 28076613 | G | A | 197 | Pld1 | intronic | . | . | . | 3qA3 | . | 0 | 1 |
| chr3 | 28088681 | C | T | 222 | Pld1 | exonic | synonymous SNV | Pld1:NM_008875:exon18:c.C2037T:p.P679P,Pld1:NM_001164056:exon19:c.C2037T:p.P679P | . | 3qA3 | . | 0 | 1 |
| chr3 | 28571062 | T | C | 65 | Tnik | intronic | . | . | . | 3qA3 | . | 0 | 1 |
| chr3 | 30679227 | G | A | 221 | Lrrc31 | exonic | missense SNV | Lrrc31:NM_001309233:exon9:c.C1337T:p.S446F | . | 3qA3 | . | 0 | 1 |
| chr3 | 30762237 | A | C | 221 | Samd7 | exonic | missense SNV | Samd7:NM_029489:exon7:c.A1131C:p.K377N | . | 3qA3 | . | 0 | 1 |
| chr3 | 30974890 | T | G | 172 | . | intergenic | . | . | . | 3qA3 | . | 0 | 1 |
| chr3 | 31097179 | A | G | 83 | Skil | UTR5 | . | . | . | 3qA3 | . | 0 | 1 |
| chr3 | 31150010 | C | A | 76 | Cldn11 | UTR5 | . | . | 516 | 3qA3 | rs580925156 | 0 | 1 |
| chr3 | 31257506 | G | C | 161 | Slc7a14 | intronic | . | . | . | 3qA3 | . | 0 | 1 |
| chr3 | 32341499 | C | A | 153 | Zmat3 | exonic | synonymous SNV | Zmat3:NM_009517:exon6:c.G858T:p.L286L | . | 3qA3 | . | 0 | 1 |
| chr3 | 32438048 | A | T | 133 | Pik3ca | exonic | missense SNV | Pik3ca:NM_008839:exon3:c.A794T:p.Y265F | . | 3qA3 | . | 0 | 1 |
| chr3 | 32712408 | T | G | 30.2895 | Actl6a | intronic | . | . | . | 3qA3 | . | 0 | 1 |
| chr3 | 32956853 | C | T | 128 | Pex5l | intronic | . | . | . | 3qA3 | . | 0 | 1 |
| chr3 | 33082620 | T | C | 62 | Pex5l,4930419G24I | ncRNA_intronic | . | . | . | 3qA3 | . | 0 | 1 |
| chr3 | 33816245 | G | A | 115 | Ccdc39 | intronic | . | . | . | 3qA3 | rs30301535 | 1 | 0 |
| chr3 | 33825204 | A | C | 82 | Ccdc39 | intronic | . | . | . | 3qA3 | . | 0 | 1 |
| chr3 | 34650888 | G | T | 220 | Sox2ot,Sox2 | exonic | missense SNV | Sox2:NM_011443:exon1:c.G473T:p.R158L | 2537 | 3qA3 | . | 0 | 1 |
| chr3 | 35546154 | T | C | 72 | . | intergenic | . | . | . | 3qA3 | . | 0 | 1 |
| chr3 | 36908194 | G | C | 121 | 4932438A13Rik | exonic | missense SNV | 4932438A13Rik:NM_172679:exon15:c.G1443C:p.L481F | . | 3qB | . | 0 | 1 |
| chr3 | 36988168 | A | G | 221 | 4932438A13Rik | exonic | missense SNV | 4932438A13Rik:NM_172679:exon48:c.A8299G:p.T2767A | . | 3qB | . | 0 | 1 |
| chr3 | 37734732 | G | T | 106 | . | intergenic | . | . | . | 3qB | . | 0 | 1 |

| | | | | | | | | | | | | | |
|---|---|---|---|---|---|---|---|---|---|---|---|---|---|
| chr3 | 38454182 | G | A | 83 | Ankrd50 | intronic | . | . | . | 3qB | . | 0 | 1 |
| chr3 | 38889217 | G | T | 128 | Fat4 | exonic | missense SNV | Fat4:NM_183221:exon1:c.G2258T:p.R753I | . | 3qB | . | 0 | 1 |
| chr3 | 38942747 | A | T | 134 | Fat4 | intronic | . | . | . | 3qB | . | 0 | 1 |
| chr3 | 39009730 | G | A | 198 | Fat4 | exonic | missense SNV | Fat4:NM_183221:exon17:c.G13834A:p.A4612T | . | 3qB | . | 0 | 1 |
| chr3 | 40672540 | C | A | 228 | Intu | intronic | . | . | . | 3qB | rs51733737 | 1 | 0 |
| chr3 | 40930156 | C | G | 190 | Abhd18 | exonic | missense SNV | Abhd18:NM_026622:exon9:c.C629G:p.S210C | . | 3qB | . | 0 | 1 |
| chr3 | 41734313 | T | C | 85 | Sclt1 | intronic | . | . | . | 3qB | rs214464402 | 0 | 1 |
| chr3 | 43250356 | A | T | 148 | . | intergenic | . | . | . | 3qB | . | 0 | 1 |
| chr3 | 43250406 | A | C | 104 | . | intergenic | . | . | . | 3qB | . | 0 | 1 |
| chr3 | 45392697 | C | T | 184 | Pcdh10 | exonic | missense SNV | Pcdh10:NM_001098170:exon4:c.C2926T:p.P976S,Pcdh10:NM_001098171:exon4:c.C2926T:p.P976S,Pcdh10: | . | 3qB | . | 0 | 1 |
| chr3 | 48608874 | G | T | 193 | 1700018B24Rik | ncRNA_exonic | . | . | . | 3qC | . | 0 | 1 |
| chr3 | 49382215 | C | G | 222 | . | intergenic | . | . | . | 3qC | . | 0 | 1 |
| chr3 | 49756342 | A | G | 95 | Pcdh18 | exonic | missense SNV | Pcdh18:NM_130448:exon1:c.T523C:p.S175P | . | 3qC | . | 0 | 1 |
| chr3 | 53477721 | G | T | 221 | Proser1 | exonic | synonymous SNV | Proser1:NM_173382:exon11:c.G1023T:p.T341T | . | 3qC | . | 0 | 1 |
| chr3 | 53537073 | T | A | 129 | Frem2 | intronic | . | . | . | 3qC | . | 0 | 1 |
| chr3 | 54279698 | A | C | 98 | Trpc4 | intronic | . | . | . | 3qC | . | 0 | 1 |
| chr3 | 54385555 | T | C | 97 | Postn | intronic | . | . | . | 3qC | . | 0 | 1 |
| chr3 | 54728839 | G | C | 221 | Exosc8 | exonic | missense SNV | Exosc8:NM_001163570:exon11:c.C782G:p.T261R,Exosc8:NM_027148:exon11:c.C770G:p.T257R | . | 3qC | . | 0 | 1 |
| chr3 | 55048658 | C | A | 205 | Ccna1 | intronic | . | . | . | 3qC | . | 0 | 1 |
| chr3 | 55204362 | T | C | 221 | Sohlh2 | exonic | missense SNV | Sohlh2:NM_028937:exon9:c.T904C:p.S302P | . | 3qC | . | 0 | 1 |
| chr3 | 55630391 | A | C | 221 | Nbea | intronic | . | . | . | 3qC | . | 0 | 1 |
| chr3 | 55819192 | G | C | 58 | Nbea | intronic | . | . | . | 3qC | . | 0 | 1 |
| chr3 | 56037074 | C | G | 38.371 | Nbea | intronic | . | . | . | 3qC | . | 0 | 1 |
| chr3 | 58865282 | T | G | 92 | Clrn1 | intronic | . | . | . | 3qD | . | 0 | 1 |
| chr3 | 59042233 | T | A | 141 | Med12l | intronic | . | . | . | 3qD | . | 0 | 1 |
| chr3 | 59232425 | C | T | 62 | P2ry12,Med12l | exonic | missense SNV | Med12l:NM_177855:exon17:c.C2420T:p.A807V | . | 3qD | . | 0 | 1 |
| chr3 | 59247922 | C | A | 218 | P2ry12,Med12l | exonic | synonymous SNV | Med12l:NM_177855:exon24:c.C3522A:p.P1174P | . | 3qD | . | 0 | 1 |
| chr3 | 59255744 | C | T | 66 | P2ry12,Med12l | intronic | . | . | . | 3qD | . | 0 | 1 |
| chr3 | 59301330 | G | C | 83 | Med12l | exonic | missense SNV | Med12l:NM_177855:exon42:c.G6378C:p.Q2126H | . | 3qD | . | 0 | 1 |
| chr3 | 60040152 | G | A | 49.4201 | Aadac | UTR3 | . | . | . | 3qD | . | 0 | 1 |
| chr3 | 60622144 | A | G | 162 | Mbnl1 | splicing | . | . | . | 3qD | . | 0 | 1 |
| chr3 | 60860631 | G | C | 222 | . | intergenic | . | . | . | 3qD | . | 0 | 1 |
| chr3 | 63343481 | C | A | 99 | Mme | intronic | . | . | . | 3qE1 | . | 0 | 1 |
| chr3 | 63343482 | C | A | 95 | Mme | intronic | . | . | . | 3qE1 | . | 0 | 1 |
| chr3 | 63347102 | T | G | 221 | Mme | intronic | . | . | . | 3qE1 | . | 0 | 1 |
| chr3 | 63697453 | A | C | 222 | Plch1 | exonic | missense SNV | Plch1:NM_183191:exon23:c.T5029G:p.Y1677D | . | 3qE1 | . | 0 | 1 |
| chr3 | 63698128 | G | T | 107 | Plch1 | exonic | missense SNV | Plch1:NM_183191:exon23:c.C4354A:p.Q1452K | . | 3qE1 | . | 0 | 1 |
| chr3 | 63699026 | G | A | 96 | Plch1 | exonic | synonymous SNV | Plch1:NM_183191:exon23:c.C3456T:p.D1152D | . | 3qE1 | . | 0 | 1 |
| chr3 | 63699872 | C | A | 221 | Plch1 | intronic | . | . | . | 3qE1 | . | 0 | 1 |
| chr3 | 63709262 | C | A | 51 | Plch1 | intronic | . | . | . | 3qE1 | . | 0 | 1 |
| chr3 | 63722592 | C | T | 77 | Plch1 | intronic | . | . | . | 3qE1 | rs220676969 | 0 | 1 |
| chr3 | 63731581 | G | T | 222 | Plch1 | intronic | . | . | . | 3qE1 | . | 0 | 1 |
| chr3 | 63740903 | A | C | 55 | Plch1 | intronic | . | . | . | 3qE1 | . | 0 | 1 |
| chr3 | 63740995 | C | T | 37.7165 | Plch1 | intronic | . | . | . | 3qE1 | . | 0 | 1 |
| chr3 | 63781375 | T | A | 99 | Plch1 | exonic | missense SNV | Plch1:NM_001177732:exon3:c.A371T:p.K124M,Plch1:NM_001177733:exon3:c.A371T:p.K124M,Plch1:NM_18 | . | 3qE1 | . | 0 | 1 |
| chr3 | 63920418 | C | G | 169 | E130311K13Rik | intronic | . | . | . | 3qE1 | . | 0 | 1 |
| chr3 | 64014392 | C | T | 164 | Gmps | intronic | . | . | . | 3qE1 | . | 0 | 1 |
| chr3 | 64278804 | T | A | 221 | Vmn2r3 | exonic | synonymous SNV | Vmn2r3:NM_001104614:exon2:c.A375T:p.G125G | . | 3qE1 | . | 0 | 1 |
| chr3 | 64278805 | C | A | 221 | Vmn2r3 | exonic | missense SNV | Vmn2r3:NM_001104614:exon2:c.G374T:p.G125V | . | 3qE1 | . | 0 | 1 |
| chr3 | 67180752 | G | A | 133 | Rsrc1 | intronic | . | . | . | 3qE1 | . | 0 | 1 |
| chr3 | 68625675 | G | C | 127 | Schip1,Gm21949 | intronic | . | . | . | 3qE1 | . | 0 | 1 |
| chr3 | 68846038 | G | A | 61 | . | intergenic | . | . | . | 3qE1 | rs222534283 | 0 | 1 |
| chr3 | 72906966 | C | A | 116 | Sis | intronic | . | . | . | 3qE3 | . | 0 | 1 |
| chr3 | 72924983 | G | A | 89 | Sis | exonic | synonymous SNV | Sis:NM_001081137:exon26:c.C3192T:p.G1064G | . | 3qE3 | . | 0 | 1 |
| chr3 | 72951617 | T | G | 163 | Sis | exonic | missense SNV | Sis:NM_001081137:exon9:c.A1080C:p.E360D | . | 3qE3 | . | 0 | 1 |
| chr3 | 72953036 | A | T | 108 | Sis | intronic | . | . | . | 3qE3 | . | 0 | 1 |
| chr3 | 73049374 | G | T | 222 | Slitrk3 | exonic | synonymous SNV | Slitrk3:NM_198864:exon2:c.C2064A:p.P688P | . | 3qE3 | . | 0 | 1 |
| chr3 | 73701116 | C | T | 174 | Bche | exonic | missense SNV | Bche:NM_009738:exon2:c.G976A:p.D326N | . | 3qE3 | . | 0 | 1 |
| chr3 | 74242756 | A | C | 30.3309 | . | intergenic | . | . | . | 3qE3 | . | 0 | 1 |
| chr3 | 74815218 | A | C | 222 | . | intergenic | . | . | . | 3qE3 | . | 0 | 1 |
| chr3 | 75038050 | T | A | 222 | Zbbx | exonic | synonymous SNV | Zbbx:NM_001310529:exon17:c.A2049T:p.S683S,Zbbx:NM_172515:exon17:c.A2064T:p.S688S | . | 3qE3 | . | 0 | 1 |
| chr3 | 75061391 | T | G | 99 | Zbbx | intronic | . | . | . | 3qE3 | . | 0 | 1 |
| chr3 | 75242601 | A | C | 141 | Serpini2 | intronic | . | . | . | 3qE3 | . | 0 | 1 |
| chr3 | 76648152 | T | A | 117 | Fstl5 | intronic | . | . | . | 3qE3 | . | 0 | 1 |
| chr3 | 81942059 | A | T | 118 | Ctso | intronic | . | . | . | 3qE3 | . | 1 | 0 |
| chr3 | 82034714 | G | A | 159 | Gucy1b3 | intronic | . | . | . | 3qE3 | . | 0 | 1 |
| chr3 | 82097786 | G | C | 221 | Gucy1a3 | intronic | . | . | . | 3qE3 | . | 0 | 1 |
| chr3 | 82540614 | G | T | 221 | Npy2r | exonic | missense SNV | Npy2r:NM_008731:exon2:c.C853A:p.H285N,Npy2r:NM_001205099:exon3:c.C514A:p.H172N | . | 3qE3 | . | 0 | 1 |
| chr3 | 83032735 | T | A | 221 | Fga | exonic | synonymous SNV | Fga:NM_001111048:exon6:c.T1695A:p.G565G | . | 3qE3 | . | 0 | 1 |
| chr3 | 83127941 | A | G | 198 | . | intergenic | . | . | 1994 | 3qE3 | . | 0 | 1 |
| chr3 | 85619000 | G | A | 175 | Gatb | exonic | missense SNV | Gatb:NM_144896:exon9:c.G1184A:p.S395N | . | 3qF1 | . | 0 | 1 |
| chr3 | 86799508 | G | A | 180 | Dclk2 | exonic | synonymous SNV | Dclk2:NM_001195497:exon11:c.C1647T:p.V549V,Dclk2:NM_001195500:exon11:c.C1647T:p.V549V,Dclk2:N | . | 3qF1 | . | 0 | 1 |
| chr3 | 87381987 | A | C | 51 | Fcrl1 | intronic | . | . | . | 3qF1 | . | 0 | 1 |
| chr3 | 88172120 | G | A | 57 | 1700113A16Rik | ncRNA_exonic | . | . | . | 3qF1 | . | 0 | 1 |
| chr3 | 88247421 | G | A | 88 | Rhbg | intronic | . | . | . | 3qF1 | . | 0 | 1 |
| chr3 | 88366163 | G | A | 175 | Paqr6 | exonic | missense SNV | Paqr6:NM_198410:exon5:c.G386A:p.C129Y | . | 3qF1 | . | 0 | 1 |
| chr3 | 88438526 | C | G | 141 | Sema4a | exonic | missense SNV | Sema4a:NM_001163489:exon12:c.G1351C:p.D451H,Sema4a:NM_001163490:exon12:c.G1351C:p.D451H,Sema4 | . | 3qF1 | . | 0 | 1 |
| chr3 | 88563283 | A | G | 107 | Ubqln4 | exonic | missense SNV | Ubqln4:NM_033526:exon6:c.A1061G:p.H354R | . | 3qF1 | . | 0 | 1 |
| chr3 | 88629742 | C | G | 132 | Arhgef2 | intronic | . | . | . | 3qF1 | . | 0 | 1 |
| chr3 | 88646262 | T | A | 222 | Arhgef2 | exonic | missense SNV | Arhgef2:NM_001198912:exon21:c.T2705A:p.L902H,Arhgef2:NM_001198913:exon21:c.T2699A:p.L900H,Arh | . | 3qF1 | . | 0 | 1 |
| chr3 | 89213926 | C | A | 216 | Mtx1 | exonic | stopgain | Mtx1:NM_001161824:exon1:c.G400T:p.E134X,Mtx1:NM_013604:exon1:c.G400T:p.E134X | 878 | 3qF1 | . | 0 | 1 |
| chr3 | 89772362 | C | T | 156 | 4632404H12Rik | ncRNA_intronic | . | . | . | 3qF1 | . | 0 | 1 |
| chr3 | 89946336 | G | A | 127 | Atp8b2 | intronic | . | . | . | 3qF1 | . | 0 | 1 |
| chr3 | 90015011 | C | A | 138 | Ubap2l | intronic | . | . | . | 3qF1 | . | 0 | 1 |
| chr3 | 90348545 | A | G | 221 | Gatad2b | intronic | . | . | . | 3qF1 | . | 0 | 1 |
| chr3 | 90387710 | C | T | 178 | Slc27a3 | exonic | missense SNV | Slc27a3:NM_001316688:exon4:c.G652A:p.D218N,Slc27a3:NM_011988:exon4:c.G1039A:p.D347N | . | 3qF1 | . | 0 | 1 |
| chr3 | 92028039 | C | T | 101 | Pglyrp3 | exonic | missense SNV | Pglyrp3:NM_207247:exon5:c.C595T:p.H199Y | . | 3qF1 | . | 0 | 1 |
| chr3 | 92646933 | T | C | 73 | Lce1a1 | exonic | missense SNV | Lce1a1:NM_025984:exon2:c.A233G:p.H78R | . | 3qF1 | . | 0 | 1 |
| chr3 | 92967977 | G | C | 72 | Lce3e | exonic | missense SNV | Lce3e:NM_001254725:exon2:c.G239C:p.G80A | . | 3qF1 | . | 0 | 1 |
| chr3 | 93201304 | G | C | 210 | Flg2 | exonic | missense SNV | Flg2:NM_001013804:exon3:c.G638C:p.R213T | . | 3qF2.1 | . | 0 | 1 |
| chr3 | 93320669 | A | C | 195 | Hrnr | exonic | missense SNV | Hrnr:NM_133698:exon2:c.A93C:p.E31D | . | 3qF2.1 | . | 0 | 1 |
| chr3 | 93447288 | A | T | 221 | Tchh | exonic | missense SNV | Tchh:NM_001163098:exon2:c.A4034T:p.E1345V | 4338 | 3qF2.1 | . | 0 | 1 |
| chr3 | 94391164 | A | G | 92 | Rorc | exonic | missense SNV | Rorc:NM_001293734:exon6:c.A955G:p.M319V,Rorc:NM_011281:exon7:c.A1018G:p.M340V | . | 3qF2.1 | . | 0 | 1 |
| chr3 | 94391226 | C | A | 221 | Rorc | intronic | . | . | . | 3qF2.1 | . | 0 | 1 |
| chr3 | 94423740 | T | A | 88 | Tdrkh | exonic | missense SNV | Tdrkh:NM_028307:exon3:c.T170A:p.M57K | . | 3qF2.1 | . | 0 | 1 |
| chr3 | 95591705 | C | T | 76 | Golph3l | intronic | . | . | . | 3qF2.1 | . | 0 | 1 |
| chr3 | 95734876 | T | C | 228 | Ecm1 | exonic | missense SNV | Ecm1:NM_001252653:exon10:c.A1393G:p.I465V,Ecm1:NM_007899:exon10:c.A1396G:p.I466V | . | 3qF2.1 | rs13474735 | 1 | 0 |
| chr3 | 95737980 | C | G | 228 | Ecm1 | intronic | . | . | . | 3qF2.1 | rs47116829 | 1 | 0 |
| chr3 | 95737983 | G | T | 228 | Ecm1 | intronic | . | . | . | 3qF2.1 | rs51121506 | 1 | 0 |
| chr3 | 95884817 | A | T | 115 | C920021L13Rik | ncRNA_intronic | . | . | . | 3qF2.1 | . | 0 | 1 |
| chr3 | 95989405 | G | A | 221 | Plekho1 | exonic | missense SNV | Plekho1:NM_023320:exon6:c.C751T:p.P251S | . | 3qF2.1 | rs30406266 | 0 | 1 |
| chr3 | 96047130 | T | G | 154 | Vps45 | exonic | synonymous SNV | Vps45:NM_013841:exon6:c.A444C:p.R148R | . | 3qF2.1 | . | 0 | 1 |
| chr3 | 96270124 | T | A | 181 | Hist2h2bb | exonic | missense SNV | Hist2h2bb:NM_175666:exon1:c.T373A:p.S125T | . | 3qF2.1 | . | 0 | 1 |
| chr3 | 96286873 | C | T | 221 | Fcgr1 | intronic | . | . | . | 3qF2.1 | rs30605008 | 0 | 1 |
| chr3 | 96805857 | A | G | 221 | Cd160 | exonic | synonymous SNV | Cd160:NM_001163496:exon3:c.T90C:p.I30I,Cd160:NM_001163497:exon3:c.T90C:p.I30I,Cd160:NM_01876 | . | 3qF2.1 | . | 0 | 1 |
| chr3 | 96854557 | A | G | 228 | Pdzk1 | exonic | missense SNV | Pdzk1:NM_001146001:exon4:c.A484G:p.N162D,Pdzk1:NM_021517:exon4:c.A484G:p.N162D | . | 3qF2.1 | rs13470433 | 1 | 0 |
| chr3 | 97165963 | A | G | 222 | Acp6 | exonic | missense SNV | Acp6:NM_019800:exon2:c.A319G:p.T107A | . | 3qF2.1 | . | 0 | 1 |
| chr3 | 97209422 | C | A | 221 | Bcl9 | exonic | missense SNV | Bcl9:NM_029933:exon8:c.G1955T:p.R652L | . | 3qF2.1 | . | 0 | 1 |
| chr3 | 97692763 | A | T | 220 | Pde4dip | intronic | . | . | . | 3qF2.2 | . | 0 | 1 |
| chr3 | 98884924 | C | A | 222 | Hao2 | intronic | . | . | . | 3qF2.2 | . | 0 | 1 |
| chr3 | 99235214 | C | G | 90 | . | intergenic | . | . | . | 3qF2.2 | rs49739188 | 0 | 1 |
| chr3 | 99235423 | G | A | 221 | . | intergenic | . | . | . | 3qF2.2 | rs33234243 | 0 | 1 |
| chr3 | 99316197 | T | C | 221 | Tbx15 | exonic | synonymous SNV | Tbx15:NM_009323:exon5:c.T700C:p.L234L | . | 3qF2.2 | rs31498304 | 0 | 1 |
| chr3 | 99340362 | C | T | 130 | Tbx15 | intronic | . | . | . | 3qF2.2 | rs33245101 | 0 | 1 |
| chr3 | 99340463 | C | T | 220 | Tbx15 | intronic | . | . | . | 3qF2.2 | rs30200969 | 0 | 1 |
| chr3 | 99340722 | G | A | 193 | Tbx15 | intronic | . | . | . | 3qF2.2 | rs266119570 | 0 | 1 |
| chr3 | 99340726 | C | G | 198 | Tbx15 | intronic | . | . | . | 3qF2.2 | rs232828155 | 0 | 1 |
| chr3 | 99340799 | T | C | 118 | Tbx15 | intronic | . | . | . | 3qF2.2 | rs33245103 | 0 | 1 |
| chr3 | 99501076 | T | C | 221 | . | intergenic | . | . | . | 3qF2.2 | rs33256337 | 0 | 1 |
| chr3 | 99501293 | C | T | 220 | . | intergenic | . | . | . | 3qF2.2 | rs33257129 | 0 | 1 |
| chr3 | 99501414 | A | C | 220 | . | intergenic | . | . | . | 3qF2.2 | rs46068141 | 0 | 1 |
| chr3 | 99501614 | G | A | 107 | . | intergenic | . | . | . | 3qF2.2 | rs33257905 | 1 | 0 |
| chr3 | 99939113 | C | T | 102 | Spag17 | intronic | . | . | . | 3qF2.2 | rs30054954 | 0 | 1 |
| chr3 | 99939140 | T | C | 130 | Spag17 | intronic | . | . | . | 3qF2.2 | rs31044276 | 0 | 1 |
| chr3 | 99939143 | G | A | 128 | Spag17 | intronic | . | . | . | 3qF2.2 | rs31439437 | 0 | 1 |
| chr3 | 99939272 | C | T | 222 | Spag17 | exonic | synonymous SNV | Spag17:NM_028892:exon2:c.C111T:p.F37F | . | 3qF2.2 | rs30123147 | 0 | 1 |
| chr3 | 99939329 | T | C | 221 | Spag17 | exonic | synonymous SNV | Spag17:NM_028892:exon2:c.T168C:p.V56V | . | 3qF2.2 | rs13477290 | 0 | 1 |
| chr3 | 99939442 | T | A | 222 | Spag17 | intronic | . | . | . | 3qF2.2 | rs30263070 | 0 | 1 |
| chr3 | 99939453 | T | C | 222 | Spag17 | intronic | . | . | . | 3qF2.2 | rs30993651 | 0 | 1 |
| chr3 | 99939469 | G | A | 222 | Spag17 | intronic | . | . | . | 3qF2.2 | rs31387639 | 0 | 1 |
| chr3 | 99939917 | G | A | 221 | Spag17 | intronic | . | . | . | 3qF2.2 | rs31003174 | 0 | 1 |
| chr3 | 99939966 | C | T | 221 | Spag17 | intronic | . | . | . | 3qF2.2 | rs48251180 | 0 | 1 |
| chr3 | 99940121 | T | G | 221 | Spag17 | intronic | . | . | . | 3qF2.2 | rs31647167 | 0 | 1 |
| chr3 | 99940142 | G | A | 193 | Spag17 | intronic | . | . | . | 3qF2.2 | rs31045553 | 0 | 1 |
| chr3 | 99940146 | A | T | 170 | Spag17 | intronic | . | . | . | 3qF2.2 | rs31340806 | 0 | 1 |
| chr3 | 99967300 | C | T | 219 | Spag17 | intronic | . | . | . | 3qF2.2 | rs229708931 | 0 | 1 |
| chr3 | 99967363 | T | G | 89 | Spag17 | intronic | . | . | . | 3qF2.2 | . | 0 | 1 |
| chr3 | 99984883 | C | T | 101 | Spag17 | intronic | . | . | . | 3qF2.2 | rs30213294 | 1 | 0 |
| chr3 | 100005026 | G | T | 113 | Spag17 | intronic | . | . | . | 3qF2.2 | rs33307067 | 0 | 1 |
| chr3 | 100008345 | T | C | 201 | Spag17 | intronic | . | . | . | 3qF2.2 | rs3163125 | 0 | 1 |
| chr3 | 100008530 | C | T | 209 | Spag17 | intronic | . | . | . | 3qF2.2 | rs3163124 | 0 | 1 |
| chr3 | 100008563 | C | T | 153 | Spag17 | intronic | . | . | . | 3qF2.2 | rs3159214 | 0 | 1 |
| chr3 | 100010488 | T | C | 118 | Spag17 | intronic | . | . | . | 3qF2.2 | rs3163120 | 0 | 1 |
| chr3 | 100010670 | A | G | 222 | Spag17 | intronic | . | . | . | 3qF2.2 | rs3159213 | 0 | 1 |
| chr3 | 100010893 | G | A | 221 | Spag17 | intronic | . | . | . | 3qF2.2 | rs30995062 | 0 | 1 |
| chr3 | 100013244 | C | T | 221 | Spag17 | exonic | synonymous SNV | Spag17:NM_028892:exon10:c.C1294T:p.L432L | . | 3qF2.2 | . | 0 | 1 |
| chr3 | 100027081 | T | C | 150 | Spag17 | intronic | . | . | . | 3qF2.2 | rs30946857 | 0 | 1 |
| chr3 | 100027787 | A | G | 193 | Spag17 | intronic | . | . | . | 3qF2.2 | rs30798541 | 0 | 1 |
| chr3 | 100038862 | T | C | 107 | Spag17 | intronic | . | . | . | 3qF2.2 | rs3159183 | 0 | 1 |
| chr3 | 100049081 | G | A | 221 | Spag17 | intronic | . | . | . | 3qF2.2 | rs33309618 | 0 | 1 |
| chr3 | 100049277 | C | A | 184 | Spag17 | exonic | missense SNV | Spag17:NM_028892:exon19:c.C2688A:p.S896R | . | 3qF2.2 | . | 0 | 1 |
| chr3 | 100050863 | A | C | 221 | Spag17 | intronic | . | . | . | 3qF2.2 | rs3163082 | 0 | 1 |
| chr3 | 100050973 | T | C | 115 | Spag17 | intronic | . | . | . | 3qF2.2 | rs3163081 | 0 | 1 |
| chr3 | 100053434 | C | T | 129 | Spag17 | intronic | . | . | . | 3qF2.2 | rs30253622 | 1 | 0 |
| chr3 | 100053497 | G | T | 128 | Spag17 | intronic | . | . | . | 3qF2.2 | rs3159172 | 0 | 1 |
| chr3 | 100053561 | G | A | 105 | Spag17 | intronic | . | . | . | 3qF2.2 | rs30654524 | 0 | 1 |
| chr3 | 100055185 | A | G | 122 | Spag17 | intronic | . | . | . | 3qF2.2 | rs3159166 | 0 | 1 |
| chr3 | 100056029 | C | T | 112 | Spag17 | intronic | . | . | . | 3qF2.2 | rs33315148 | 0 | 1 |
| chr3 | 100058577 | C | T | 99 | Spag17 | intronic | . | . | . | 3qF2.2 | rs3163066 | 0 | 1 |
| chr3 | 100058586 | T | C | 120 | Spag17 | intronic | . | . | . | 3qF2.2 | rs3163065 | 0 | 1 |
| chr3 | 100060610 | G | A | 102 | Spag17 | intronic | . | . | . | 3qF2.2 | rs3159160 | 0 | 1 |
| chr3 | 100063338 | C | T | 221 | Spag17 | exonic | synonymous SNV | Spag17:NM_028892:exon27:c.C3837T:p.I1279I | . | 3qF2.2 | rs3159158 | 0 | 1 |
| chr3 | 100071845 | G | C | 125 | Spag17 | intronic | . | . | . | 3qF2.2 | rs3159156 | 0 | 1 |
| chr3 | 100071901 | A | G | 158 | Spag17 | intronic | . | . | . | 3qF2.2 | rs3159155 | 0 | 1 |
| chr3 | 100072215 | T | C | 221 | Spag17 | intronic | . | . | . | 3qF2.2 | rs3163060 | 0 | 1 |
| chr3 | 100075480 | C | A | 72 | Spag17 | intronic | . | . | . | 3qF2.2 | rs52002217 | 0 | 1 |
| chr3 | 100075536 | C | A | 82 | Spag17 | intronic | . | . | . | 3qF2.2 | rs48902983 | 0 | 1 |
| chr3 | 100075545 | A | G | 100 | Spag17 | intronic | . | . | . | 3qF2.2 | rs51405865 | 0 | 1 |
| chr3 | 100075921 | A | C | 222 | Spag17 | intronic | . | . | . | 3qF2.2 | rs33313516 | 0 | 1 |
| chr3 | 100075934 | C | T | 222 | Spag17 | intronic | . | . | . | 3qF2.2 | rs46999900 | 0 | 1 |
| chr3 | 100075935 | A | G | 215 | Spag17 | intronic | . | . | . | 3qF2.2 | rs33313519 | 0 | 1 |
| chr3 | 100076895 | T | C | 71 | Spag17 | intronic | . | . | . | 3qF2.2 | rs49837664 | 0 | 1 |
| chr3 | 100076901 | C | T | 71 | Spag17 | intronic | . | . | . | 3qF2.2 | rs51460244 | 0 | 1 |
| chr3 | 100077012 | C | T | 86 | Spag17 | intronic | . | . | . | 3qF2.2 | rs3163054 | 0 | 1 |
| chr3 | 100077037 | T | C | 114 | Spag17 | intronic | . | . | . | 3qF2.2 | rs3163053 | 0 | 1 |
| chr3 | 100077081 | G | A | 169 | Spag17 | intronic | . | . | . | 3qF2.2 | rs3159147 | 0 | 1 |
| chr3 | 100077113 | G | A | 221 | Spag17 | intronic | . | . | . | 3qF2.2 | rs3159146 | 0 | 1 |
| chr3 | 100077391 | C | T | 220 | Spag17 | intronic | . | . | . | 3qF2.2 | rs3163052 | 0 | 1 |
| chr3 | 100085109 | T | A | 113 | Spag17 | intronic | . | . | . | 3qF2.2 | rs48973052 | 0 | 1 |
| chr3 | 100085547 | T | G | 115 | Spag17 | intronic | . | . | . | 3qF2.2 | rs31240140 | 0 | 1 |
| chr3 | 100095652 | A | G | 221 | Spag17 | intronic | . | . | . | 3qF2.2 | rs31374206 | 0 | 1 |
| chr3 | 100095982 | C | T | 100 | Spag17 | intronic | . | . | . | 3qF2.2 | rs31001366 | 0 | 1 |
| chr3 | 100100326 | G | A | 218 | Spag17 | exonic | synonymous SNV | Spag17:NM_028892:exon41:c.G5811A:p.S1937S | . | 3qF2.2 | rs30949744 | 0 | 1 |
| chr3 | 100102998 | A | G | 121 | Spag17 | intronic | . | . | . | 3qF2.2 | rs30174712 | 0 | 1 |
| chr3 | 100103125 | C | A | 222 | Spag17 | exonic | synonymous SNV | Spag17:NM_028892:exon43:c.C5934A:p.T1978T | . | 3qF2.2 | rs31600292 | 0 | 1 |

| | | | | | | | | | | | | | |
|---|---|---|---|---|---|---|---|---|---|---|---|---|---|
| chr3 | 100103259 | A | G | 136 | Spag17 | exonic | missense SNV | Spag17:NM_028892:exon43:c.A6068G:p.N2023S | . | 3qF2.2 | . | 0 | 1 |
| chr3 | 100106770 | C | A | 221 | Spag17 | exonic | missense SNV | Spag17:NM_028892:exon44:c.C6358A:p.Q2120K | . | 3qF2.2 | rs51393238 | 0 | 1 |
| chr3 | 100124199 | C | T | 221 | Spag17 | exonic | synonymous SNV | Spag17:NM_028892:exon47:c.C6777T:p.I2259I | . | 3qF2.2 | rs33318859 | 0 | 1 |
| chr3 | 100125111 | A | G | 63 | Spag17 | intronic | . | . | . | 3qF2.2 | rs51807875 | 0 | 1 |
| chr3 | 100138582 | C | T | 136 | Spag17,Wdr3 | UTR3 | . | . | . | 3qF2.2 | rs31576346 | 0 | 1 |
| chr3 | 100138730 | T | C | 221 | Spag17,Wdr3 | exonic | missense SNV | Wdr3:NM_175552:exon27:c.A2786G:p.K929R | . | 3qF2.2 | rs30152485 | 0 | 1 |
| chr3 | 100142147 | C | T | 220 | Wdr3,Spag17 | intronic | . | . | . | 3qF2.2 | rs30312689 | 0 | 1 |
| chr3 | 100142150 | C | T | 220 | Wdr3,Spag17 | intronic | . | . | . | 3qF2.2 | rs30702386 | 0 | 1 |
| chr3 | 100142747 | A | G | 217 | Wdr3,Spag17 | UTR3 | . | . | . | 3qF2.2 | rs30757937 | 0 | 1 |
| chr3 | 100142749 | T | A | 220 | Spag17,Wdr3 | UTR3 | . | . | . | 3qF2.2 | rs30856978 | 0 | 1 |
| chr3 | 100143308 | C | T | 141 | Wdr3,Spag17 | UTR3 | . | . | . | 3qF2.2 | rs31275649 | 0 | 1 |
| chr3 | 100150992 | T | C | 221 | Wdr3 | exonic | synonymous SNV | Wdr3:NM_175552:exon11:c.A1296G:p.A432A | . | 3qF2.2 | rs31544778 | 0 | 1 |
| chr3 | 100151144 | A | G | 221 | Wdr3 | exonic | synonymous SNV | Wdr3:NM_175552:exon11:c.T1144C:p.L382L | . | 3qF2.2 | rs31431965 | 0 | 1 |
| chr3 | 100153578 | C | T | 220 | Wdr3 | exonic | missense SNV | Wdr3:NM_175552:exon8:c.G859A:p.V287M | . | 3qF2.2 | rs30512224 | 0 | 1 |
| chr3 | 100157544 | A | G | 219 | Wdr3 | intronic | . | . | . | 3qF2.2 | rs31287637 | 0 | 1 |
| chr3 | 100161192 | C | T | 222 | Wdr3 | UTR5 | . | . | . | 3qF2.2 | rs49020823 | 0 | 1 |
| chr3 | 100161207 | A | G | 222 | Wdr3 | UTR5 | . | . | . | 3qF2.2 | rs46636576 | 0 | 1 |
| chr3 | 100170801 | T | C | 93 | Gdap2 | intronic | . | . | . | 3qF2.2 | rs30401828 | 0 | 1 |
| chr3 | 100170892 | T | C | 222 | Gdap2 | intronic | . | . | . | 3qF2.2 | rs33330973 | 0 | 1 |
| chr3 | 100170907 | T | C | 221 | Gdap2 | exonic | synonymous SNV | Gdap2:NM_010269:exon3:c.T186C:p.D62D | . | 3qF2.2 | rs30892985 | 0 | 1 |
| chr3 | 100186416 | A | G | 128 | Gdap2 | intronic | . | . | . | 3qF2.2 | rs30054174 | 0 | 1 |
| chr3 | 100186517 | T | C | 221 | Gdap2 | intronic | . | . | . | 3qF2.2 | rs30309944 | 0 | 1 |
| chr3 | 100186783 | G | A | 220 | Gdap2 | intronic | . | . | . | 3qF2.2 | rs30606921 | 0 | 1 |
| chr3 | 100187673 | T | C | 121 | Gdap2 | intronic | . | . | . | 3qF2.2 | rs33331342 | 0 | 1 |
| chr3 | 100187911 | G | A | 221 | Gdap2 | exonic | synonymous SNV | Gdap2:NM_010269:exon8:c.G834A:p.V278V | . | 3qF2.2 | rs13477291 | 0 | 1 |
| chr3 | 100190790 | G | A | 126 | Gdap2 | intronic | . | . | . | 3qF2.2 | rs108887458 | 0 | 1 |
| chr3 | 100190793 | A | G | 125 | Gdap2 | intronic | . | . | . | 3qF2.2 | rs108796873 | 0 | 1 |
| chr3 | 100191831 | A | G | 156 | Gdap2 | intronic | . | . | . | 3qF2.2 | rs31134570 | 0 | 1 |
| chr3 | 100191833 | G | A | 155 | Gdap2 | intronic | . | . | . | 3qF2.2 | rs31436462 | 0 | 1 |
| chr3 | 100194277 | C | T | 87 | Gdap2 | intronic | . | . | . | 3qF2.2 | rs31709830 | 1 | 0 |
| chr3 | 100194371 | C | T | 132 | Gdap2 | intronic | . | . | . | 3qF2.2 | rs30550447 | 0 | 1 |
| chr3 | 100194487 | C | T | 222 | Gdap2 | splicing | . | . | . | 3qF2.2 | rs31197600 | 0 | 1 |
| chr3 | 100194638 | A | T | 221 | Gdap2 | intronic | . | . | . | 3qF2.2 | rs33331413 | 0 | 1 |
| chr3 | 100196495 | T | A | 221 | Gdap2 | intronic | . | . | . | 3qF2.2 | rs30214157 | 0 | 1 |
| chr3 | 100196643 | A | C | 221 | Gdap2 | intronic | . | . | . | 3qF2.2 | rs30052802 | 0 | 1 |
| chr3 | 100196644 | C | A | 221 | Gdap2 | intronic | . | . | . | 3qF2.2 | rs30052802 | 0 | 1 |
| chr3 | 100202244 | A | G | 92 | Gdap2 | intronic | . | . | . | 3qF2.2 | rs30557469 | 0 | 1 |
| chr3 | 100205861 | A | C | 221 | Gdap2 | UTR3 | . | . | . | 3qF2.2 | rs13464343 | 0 | 1 |
| chr3 | 100413387 | C | A | 108 | . | intergenic | . | . | . | 3qF2.2 | rs3159537 | 0 | 1 |
| chr3 | 100413610 | C | G | 222 | . | intergenic | . | . | . | 3qF2.2 | rs212825508 | 0 | 1 |
| chr3 | 100413661 | T | C | 185 | . | intergenic | . | . | . | 3qF2.2 | rs33347693 | 0 | 1 |
| chr3 | 100413666 | C | T | 169 | . | intergenic | . | . | . | 3qF2.2 | rs225343277 | 0 | 1 |
| chr3 | 100414002 | A | G | 220 | . | intergenic | . | . | . | 3qF2.2 | rs33348850 | 0 | 1 |
| chr3 | 100414033 | T | C | 82 | . | intergenic | . | . | . | 3qF2.2 | rs239152383 | 0 | 1 |
| chr3 | 100414058 | T | C | 221 | . | intergenic | . | . | . | 3qF2.2 | rs45812020 | 0 | 1 |
| chr3 | 100414216 | T | G | 221 | . | intergenic | . | . | . | 3qF2.2 | rs3163359 | 0 | 1 |
| chr3 | 100414341 | C | T | 221 | . | intergenic | . | . | . | 3qF2.2 | rs3163324 | 0 | 1 |
| chr3 | 100414431 | C | G | 222 | . | intergenic | . | . | . | 3qF2.2 | rs3163325 | 0 | 1 |
| chr3 | 100414658 | T | C | 221 | . | intergenic | . | . | . | 3qF2.2 | rs3159536 | 0 | 1 |
| chr3 | 100414835 | T | C | 121 | . | intergenic | . | . | . | 3qF2.2 | rs3159535 | 0 | 1 |
| chr3 | 100473190 | G | A | 221 | Fam46c | exonic | synonymous SNV | Fam46c:NM_001142952:exon2:c.C249T:p.D83D | . | 3qF2.2 | rs3159381 | 0 | 1 |
| chr3 | 100473501 | G | A | 221 | Fam46c | intronic | . | . | . | 3qF2.2 | rs3159382 | 0 | 1 |
| chr3 | 100489895 | C | T | 110 | . | upstream | . | . | . | 3qF2.2 | rs3159398 | 0 | 1 |
| chr3 | 100522753 | T | C | 221 | . | intergenic | . | . | . | 3qF2.2 | rs3159596 | 0 | 1 |
| chr3 | 100685028 | G | A | 115 | Man1a2 | UTR5 | . | . | 1288 | 3qF2.2 | rs3159595 | 0 | 1 |
| chr3 | 100922316 | T | G | 210 | . | upstream | . | . | 561 | 3qF2.2 | . | 0 | 1 |
| chr3 | 100929973 | T | C | 221 | Trim45 | intronic | . | . | . | 3qF2.2 | rs3163472 | 0 | 1 |
| chr3 | 100943807 | T | C | 221 | Ttf2 | intronic | . | . | . | 3qF2.2 | rs245499339 | 0 | 1 |
| chr3 | 100954032 | T | A | 59 | Ttf2 | intronic | . | . | . | 3qF2.2 | rs217111736 | 0 | 1 |
| chr3 | 100966981 | G | T | 221 | Ttf2 | intronic | . | . | . | 3qF2.2 | rs216414627 | 0 | 1 |
| chr3 | 101012097 | G | A | 221 | Cd101 | exonic | missense SNV | Cd101:NM_001167906:exon6:c.C1700T:p.S567L | . | 3qF2.2 | . | 0 | 1 |
| chr3 | 101056910 | T | C | 89 | Ptgfrn | intronic | . | . | . | 3qF2.2 | rs3163456 | 1 | 0 |
| chr3 | 101061010 | G | A | 221 | Ptgfrn | exonic | synonymous SNV | Ptgfrn:NM_011197:exon5:c.C1266T:p.D422D | . | 3qF2.2 | . | 0 | 1 |
| chr3 | 101073272 | C | T | 221 | Ptgfrn | intronic | . | . | . | 3qF2.2 | rs236889830 | 0 | 1 |
| chr3 | 101076935 | T | C | 129 | Ptgfrn | intronic | . | . | . | 3qF2.2 | rs246026440 | 0 | 1 |
| chr3 | 101077021 | G | A | 221 | Ptgfrn | intronic | . | . | . | 3qF2.2 | rs33418888 | 0 | 1 |
| chr3 | 101077140 | A | G | 221 | Ptgfrn | exonic | synonymous SNV | Ptgfrn:NM_011197:exon3:c.T735C:p.Y245Y | . | 3qF2.2 | rs33418891 | 0 | 1 |
| chr3 | 101077194 | G | A | 221 | Ptgfrn | exonic | synonymous SNV | Ptgfrn:NM_011197:exon3:c.C681T:p.S227S | 219 | 3qF2.2 | rs239770231 | 0 | 1 |
| chr3 | 101080189 | A | G | 221 | Ptgfrn | exonic | synonymous SNV | Ptgfrn:NM_011197:exon2:c.T357C:p.C119C | . | 3qF2.2 | rs33416356 | 0 | 1 |
| chr3 | 101287758 | A | T | 220 | Cd2 | intronic | . | . | . | 3qF2.2 | . | 0 | 1 |
| chr3 | 101450960 | A | T | 222 | Igsf3 | exonic | synonymous SNV | Igsf3:NM_207205:exon8:c.A2106T:p.S702S | . | 3qF2.2 | . | 0 | 1 |
| chr3 | 101815167 | T | G | 221 | Mab21l3 | exonic | missense SNV | Mab21l3:NM_172295:exon7:c.A1142C:p.K381T | . | 3qF2.2 | . | 0 | 1 |
| chr3 | 102735309 | G | T | 161 | Tspan2os,Tspan2 | exonic | missense SNV | Tspan2:NM_001243132:exon1:c.G51T:p.W17C | 813 | 3qF2.2 | . | 0 | 1 |
| chr3 | 103060272 | A | T | 221 | Nras | exonic | missense SNV | Nras:NM_010937:exon3:c.A183T:p.Q61H | . | 3qF2.2 | . | 0 | 1 |
| chr3 | 103341716 | T | A | 164 | Trim33 | exonic | stopgain | Trim33:NM_001079830:exon14:c.T2454A:p.C818X,Trim33:NM_053170:exon14:c.T2454A:p.C818X | . | 3qF2.2 | . | 0 | 1 |
| chr3 | 103803185 | T | C | 171 | Gm15471,Dclre1b | exonic | missense SNV | Dclre1b:NM_001025312:exon3:c.A1031G:p.Q344R,Dclre1b:NM_133865:exon4:c.A1409G:p.Q470R | . | 3qF2.2 | . | 0 | 1 |
| chr3 | 103959872 | A | C | 106 | Rsbn1 | intronic | . | . | . | 3qF2.2 | . | 0 | 1 |
| chr3 | 104480266 | T | A | 66 | Lrig2 | intronic | . | . | . | 3qF2.2 | . | 0 | 1 |
| chr3 | 104752815 | G | C | 221 | . | intergenic | . | . | . | 3qF2.2 | . | 0 | 1 |
| chr3 | 104752817 | A | T | 221 | . | intergenic | . | . | . | 3qF2.2 | . | 0 | 1 |
| chr3 | 105680341 | T | A | 175 | Ddx20 | splicing | . | . | . | 3qF2.2 | . | 0 | 1 |
| chr3 | 105926312 | T | A | 131 | I830077J02Rik | UTR3 | . | . | . | 3qF2.2 | . | 0 | 1 |
| chr3 | 106043586 | G | T | 167 | . | intergenic | . | . | . | 3qF2.2 | rs31095074 | 1 | 0 |
| chr3 | 106129067 | T | G | 107 | Chia1 | intronic | . | . | . | 3qF2.2 | . | 0 | 1 |
| chr3 | 106202448 | A | G | 222 | Chil4 | intronic | . | . | . | 3qF2.2 | rs264143718 | 0 | 1 |
| chr3 | 106205727 | G | T | 98 | Chil4 | intronic | . | . | . | 3qF2.2 | . | 0 | 1 |
| chr3 | 106206326 | G | A | 51 | Chil4 | intronic | . | . | . | 3qF2.2 | rs47514235 | 0 | 1 |
| chr3 | 106404859 | C | A | 151 | Chil6 | intronic | . | . | . | 3qF2.3 | . | 0 | 1 |
| chr3 | 107036616 | G | A | 106 | Kcna3 | exonic | missense SNV | Kcna3:NM_008418:exon1:c.G194A:p.G65E | 1285 | 3qF2.3 | . | 0 | 1 |
| chr3 | 107195441 | T | G | 139 | Kcna10 | exonic | missense SNV | Kcna10:NM_001081140:exon2:c.T1387G:p.F463V | . | 3qF2.3 | . | 0 | 1 |
| chr3 | 107235718 | C | A | 220 | Prok1 | intronic | . | . | . | 3qF2.3 | . | 0 | 1 |
| chr3 | 107439187 | G | A | 119 | Kcnc4 | UTR3 | . | . | . | 3qF2.3 | . | 0 | 1 |
| chr3 | 107627247 | C | T | 81 | Strip1 | intronic | . | . | . | 3qF2.3 | . | 0 | 1 |
| chr3 | 107627421 | G | A | 42.4336 | Strip1 | exonic | stopgain | Strip1:NM_153563:exon3:c.C292T:p.R98X | . | 3qF2.3 | . | 0 | 1 |
| chr3 | 108387350 | G | A | 221 | Psrc1 | exonic | missense SNV | Psrc1:NM_001190161:exon7:c.G941A:p.G314D,Psrc1:NM_019976:exon7:c.G941A:p.G314D | . | 3qF3 | . | 0 | 1 |
| chr3 | 108469677 | G | C | 221 | 5330417C22Rik | intronic | . | . | . | 3qF3 | . | 0 | 1 |
| chr3 | 108781828 | C | T | 74 | Aknad1 | intronic | . | . | . | 3qF3 | . | 0 | 1 |
| chr3 | 109496740 | G | A | 130 | Vav3 | intronic | . | . | . | 3qF3 | . | 0 | 1 |
| chr3 | 109782841 | C | T | 62 | Ntng1 | exonic | synonymous SNV | Ntng1:NM_001163349:exon6:c.G1158A:p.A386A,Ntng1:NM_001163348:exon7:c.G1293A:p.A431A,Ntng1:NM | . | 3qF3 | . | 0 | 1 |
| chr3 | 109841412 | C | T | 44.8355 | Ntng1 | intronic | . | . | . | 3qF3 | . | 0 | 1 |
| chr3 | 112303094 | A | T | 221 | . | intergenic | . | . | . | 3qF3 | . | 0 | 1 |
| chr3 | 112591755 | A | G | 53 | . | intergenic | . | . | . | 3qF3 | . | 0 | 1 |
| chr3 | 113191315 | A | T | 220 | . | intergenic | . | . | . | 3qF3 | . | 0 | 1 |
| chr3 | 114111976 | A | T | 77 | Col11a1 | intronic | . | . | . | 3qF3 | . | 0 | 1 |
| chr3 | 114113513 | T | G | 221 | Col11a1 | intronic | . | . | . | 3qF3 | . | 0 | 1 |
| chr3 | 114121501 | A | T | 130 | Col11a1 | intronic | . | . | . | 3qF3 | . | 0 | 1 |
| chr3 | 114181266 | T | C | 113 | Col11a1 | intronic | . | . | . | 3qF3 | . | 0 | 1 |
| chr3 | 115712465 | G | T | 207 | S1pr1 | exonic | missense SNV | S1pr1:NM_007901:exon2:c.C479A:p.S160Y | . | 3qG1 | . | 0 | 1 |
| chr3 | 115942537 | G | A | 121 | Slc30a7 | UTR3 | . | . | . | 3qG1 | . | 0 | 1 |
| chr3 | 118433911 | A | C | 215 | Mir137 | ncRNA exonic | . | . | . | 3qG1 | . | 0 | 1 |
| chr3 | 119142067 | T | C | 90 | Dpyd | intronic | . | . | . | 3qG1 | . | 0 | 1 |
| chr3 | 119195189 | G | A | 221 | Dpyd | splicing | . | . | . | 3qG1 | . | 0 | 1 |
| chr3 | 119431653 | G | A | 221 | Dpyd | exonic | missense SNV | Dpyd:NM_170778:exon23:c.G3074A:p.C1025Y | . | 3qG1 | . | 0 | 1 |
| chr3 | 121773879 | C | G | 97 | Abcd3 | intronic | . | . | . | 3qG1 | . | 0 | 1 |
| chr3 | 121991124 | T | C | 184 | Arhgap29 | splicing | . | . | . | 3qG1 | . | 0 | 1 |
| chr3 | 122841043 | G | T | 166 | Pde5a | exonic | missense SNV | Pde5a:NM_153422:exon17:c.G2232T:p.L744F | . | 3qG1 | . | 0 | 1 |
| chr3 | 123761572 | A | C | 64 | . | intergenic | . | . | . | 3qG1 | . | 0 | 1 |
| chr3 | 123761755 | G | T | 221 | . | intergenic | . | . | . | 3qG1 | . | 0 | 1 |
| chr3 | 124322628 | C | T | 166 | Tram1l1 | UTR3 | . | . | . | 3qG1 | . | 0 | 1 |
| chr3 | 124414465 | G | A | 97 | 1700006A11Rik | exonic | synonymous SNV | 1700006A11Rik:NM_027939:exon6:c.C564T:p.L188L | . | 3qG1 | . | 0 | 1 |
| chr3 | 125697730 | T | G | 53 | Ndst4 | intronic | . | . | . | 3qG1 | . | 0 | 1 |
| chr3 | 126770856 | A | G | 157 | Camk2d | exonic | missense SNV | Camk2d:NM_001025438:exon6:c.A413G:p.K138R,Camk2d:NM_001293663:exon6:c.A413G:p.K138R,Camk2d:NM | . | 3qG1 | . | 0 | 1 |
| chr3 | 126784038 | G | A | 160 | Camk2d | exonic | missense SNV | Camk2d:NM_001025438:exon8:c.G601A:p.G201S,Camk2d:NM_001025439:exon8:c.G601A:p.G201S,Camk2d:NM | . | 3qG1 | . | 0 | 1 |
| chr3 | 126959729 | C | G | 124 | Ank2 | exonic | missense SNV | Ank2:NM_178655:exon9:c.G902C:p.S301T | . | 3qG2 | . | 0 | 1 |
| chr3 | 127546179 | C | A | 88 | Larp7 | exonic | missense SNV | Larp7:NM_138593:exon7:c.G853T:p.A285S | . | 3qG2 | . | 0 | 1 |
| chr3 | 127562273 | C | T | 222 | Zgrf1 | exonic | missense SNV | Zgrf1:NM_197997:exon6:c.C1147T:p.P383S | . | 3qG2 | . | 0 | 1 |
| chr3 | 127673080 | T | C | 23.0402 | Alpk1 | intronic | . | . | . | 3qG2 | . | 0 | 1 |
| chr3 | 127677787 | T | G | 222 | Alpk1 | exonic | missense SNV | Alpk1:NM_027808:exon11:c.A3045C:p.E1015D | . | 3qG2 | . | 0 | 1 |
| chr3 | 127724249 | C | T | 49.8704 | Alpk1 | intronic | . | . | . | 3qG2 | . | 0 | 1 |
| chr3 | 129214577 | A | T | 96 | Pitx2 | exonic | missense SNV | Pitx2:NM_001042502:exon1:c.A1T:p.M1L | 1204 | 3qG3 | . | 0 | 1 |
| chr3 | 129218489 | G | T | 59 | Pitx2 | exonic | missense SNV | Pitx2:NM_001042502:exon3:c.G567T:p.K189N,Pitx2:NM_001287048:exon3:c.G465T:p.K155N,Pitx2:NM_00 | . | 3qG3 | . | 0 | 1 |
| chr3 | 133206807 | G | T | 25.3738 | Arhgef38 | intronic | . | . | . | 3qG3 | . | 0 | 1 |
| chr3 | 133320795 | C | T | 52 | Ppa2 | intronic | . | . | . | 3qG3 | . | 0 | 1 |
| chr3 | 133487280 | T | C | 96 | Tet2 | exonic | synonymous SNV | Tet2:NM_001040400:exon3:c.A1392G:p.S464S | . | 3qG3 | . | 0 | 1 |
| chr3 | 135662911 | T | G | 99 | Nfkb1 | splicing | . | . | . | 3qG3 | . | 0 | 1 |
| chr3 | 135884117 | C | A | 107 | Slc39a8 | intronic | . | . | . | 3qG3 | . | 0 | 1 |
| chr3 | 138114257 | C | T | 220 | Mttp | intronic | . | . | . | 3qG3 | . | 0 | 1 |
| chr3 | 138147350 | A | C | 112 | Trmt10a | intronic | . | . | . | 3qG3 | . | 0 | 1 |
| chr3 | 138323265 | C | A | 164 | Adh6a | intronic | . | . | . | 3qG3 | rs46322761 | 1 | 0 |
| chr3 | 138323287 | C | A | 139 | Adh6a | intronic | . | . | . | 3qG3 | rs48089153 | 1 | 0 |
| chr3 | 138480716 | G | C | 221 | Metap1 | exonic | missense SNV | Metap1:NM_175224:exon3:c.C251G:p.T84S | . | 3qG3 | . | 0 | 1 |
| chr3 | 138489144 | A | C | 159 | Metap1 | intronic | . | . | 893 | 3qG3 | rs264346731 | 1 | 0 |
| chr3 | 138503686 | T | G | 39.159 | . | intergenic | . | . | . | 3qG3 | rs31343048 | 0 | 1 |
| chr3 | 141770868 | T | C | 76 | Unc5c | intronic | . | . | . | 3qH1 | . | 0 | 1 |
| chr3 | 142542747 | T | G | 102 | Gbp7 | intronic | . | . | . | 3qH1 | . | 0 | 1 |
| chr3 | 142567533 | G | A | 107 | Gbp3 | intronic | . | . | . | 3qH1 | . | 0 | 1 |
| chr3 | 142607866 | T | G | 64 | Gbp2b | intronic | . | . | . | 3qH1 | . | 0 | 1 |
| chr3 | 142664948 | C | T | 25.534 | . | intergenic | . | . | . | 3qH1 | . | 0 | 1 |
| chr3 | 144697357 | G | T | 191 | Sh3glb1 | intronic | . | . | . | 3qH2 | rs46149797 | 1 | 0 |
| chr3 | 144866746 | T | A | 221 | . | intergenic | . | . | . | 3qH2 | . | 0 | 1 |
| chr3 | 145008720 | G | T | 132 | Clca1 | intronic | . | . | . | 3qH2 | . | 0 | 1 |
| chr3 | 145314278 | G | A | 107 | Col24a1 | exonic | missense SNV | Col24a1:NM_027770:exon3:c.G409A:p.V137I | . | 3qH2 | . | 0 | 1 |
| chr3 | 145441662 | A | T | 221 | Col24a1 | splicing | . | . | . | 3qH2 | . | 0 | 1 |
| chr3 | 145647806 | G | A | 156 | Cyr61 | exonic | stopgain | Cyr61:NM_010516:exon5:c.C1000T:p.R334X | . | 3qH2 | . | 0 | 1 |
| chr3 | 146081573 | T | A | 43.9904 | Wdr63 | intronic | . | . | . | 3qH2 | . | 0 | 1 |
| chr3 | 146512749 | A | T | 186 | Rpf1 | intronic | . | . | . | 3qH2 | . | 0 | 1 |
| chr3 | 146596442 | T | C | 221 | Dnase2b | UTR5 | . | . | . | 3qH2 | . | 0 | 1 |
| chr3 | 150515213 | C | T | 25.1439 | . | intergenic | . | . | . | 3qH3 | . | 0 | 1 |
| chr3 | 150515246 | C | G | 76 | . | intergenic | . | . | . | 3qH3 | rs30520903 | 0 | 1 |
| chr3 | 150515251 | A | G | 86 | . | intergenic | . | . | . | 3qH3 | . | 0 | 1 |
| chr3 | 150515329 | T | C | 76 | . | intergenic | . | . | . | 3qH3 | . | 0 | 1 |
| chr3 | 150515385 | C | G | 70 | . | intergenic | . | . | . | 3qH3 | rs224472686 | 0 | 1 |
| chr3 | 150515388 | C | T | 75 | . | intergenic | . | . | . | 3qH3 | . | 0 | 1 |
| chr3 | 150515991 | G | A | 114 | . | intergenic | . | . | . | 3qH3 | . | 0 | 1 |
| chr3 | 151745818 | C | A | 221 | Ifi44 | exonic | missense SNV | Ifi44:NM_133871:exon4:c.G650T:p.C217F | . | 3qH3 | . | 0 | 1 |
| chr3 | 152219576 | A | C | 222 | Fubp1 | intronic | . | . | . | 3qH3 | rs31594267 | 1 | 0 |
| chr3 | 152290587 | C | A | 219 | Fam73a | intronic | . | . | . | 3qH3 | . | 0 | 1 |
| chr3 | 152358781 | G | A | 100 | Usp33 | intronic | . | . | . | 3qH3 | . | 0 | 1 |
| chr3 | 154171654 | A | G | 107 | Slc44a5 | intronic | . | . | . | 3qH3 | . | 0 | 1 |
| chr3 | 154856600 | T | A | 209 | Tnni3k | exonic | missense SNV | Tnni3k:NM_177066:exon21:c.A2039T:p.H680L | . | 3qH4 | . | 0 | 1 |
| chr3 | 154938834 | T | G | 221 | Tnni3k | exonic | missense SNV | Tnni3k:NM_177066:exon17:c.A1686C:p.K562N | . | 3qH4 | . | 0 | 1 |
| chr3 | 156816459 | A | T | 184 | Negr1 | intronic | . | . | . | 3qH4 | . | 0 | 1 |
| chr3 | 157160734 | A | G | 222 | Negr1 | exonic | missense SNV | Negr1:NM_001039094:exon6:c.A919G:p.N307D,Negr1:NM_177274:exon6:c.A919G:p.N307D | . | 3qH4 | . | 0 | 1 |
| chr3 | 157198324 | A | C | 49.2078 | Negr1 | intronic | . | . | . | 3qH4 | . | 0 | 1 |
| chr3 | 157897224 | G | A | 119 | Cth | exonic | missense SNV | Cth:NM_145953:exon11:c.C1156T:p.L386F | . | 3qH4 | . | 0 | 1 |
| chr3 | 158175127 | A | T | 118 | Lrrc7 | exonic | synonymous SNV | Lrrc7:NM_001081358:exon18:c.T1671A:p.T557T,Lrrc7:NM_001291452:exon18:c.T1671A:p.T557T,Lrrc7:N | . | 3qH4 | . | 0 | 1 |
| chr3 | 158961459 | C | A | 221 | . | intergenic | . | . | . | 3qH4 | . | 0 | 1 |
| chr3 | 159600385 | C | A | 221 | Rpe65 | exonic | missense SNV | Rpe65:NM_029987:exon2:c.C80A:p.T27K | . | 3qH4 | . | 0 | 1 |
| chr4 | 4058805 | G | A | 221 | Sdr16c6 | exonic | synonymous SNV | Sdr16c6:NM_001080710:exon6:c.C780T:p.Y260Y | . | 4qA1 | . | 0 | 1 |
| chr4 | 6823186 | T | G | 128 | Tox | intronic | . | . | . | 4qA1 | . | 0 | 1 |
| chr4 | 8561107 | T | C | 198 | Rab2a | intronic | . | . | . | 4qA1 | . | 0 | 1 |
| chr4 | 8840612 | T | C | 228 | Chd7 | intronic | . | . | . | 4qA1 | . | 1 | 0 |
| chr4 | 8850931 | G | C | 228 | Chd7 | intronic | . | . | . | 4qA1 | . | 1 | 0 |
| chr4 | 9430026 | T | A | 221 | Clvs1 | intronic | . | . | . | 4qA1 | . | 0 | 1 |
| chr4 | 11344087 | G | A | 150 | Esrp1 | intronic | . | . | . | 4qA1 | . | 0 | 1 |

| | | | | | | | | | | | | | |
|---|---|---|---|---|---|---|---|---|---|---|---|---|---|
| chr4 | 11400337 | G | A | 228 | . | intergenic | . | . | . | 4qA1 | . | 1 | 0 |
| chr4 | 11570060 | C | G | 135 | Rad54b | intronic | . | . | . | 4qA1 | . | 0 | 1 |
| chr4 | 11783503 | T | C | 221 | Cdh17 | exonic | missense SNV | Cdh17:NM_019753:exon7:c.T748C:p.S250P | . | 4qA1 | . | 0 | 1 |
| chr4 | 12131433 | T | A | 204 | . | intergenic | . | . | . | 4qA1 | . | 1 | 0 |
| chr4 | 13838058 | G | T | 228 | Runx1t1 | intronic | . | . | . | 4qA1 | . | 1 | 0 |
| chr4 | 14170628 | T | A | 221 | . | intergenic | . | . | . | 4qA1 | . | 0 | 1 |
| chr4 | 14548621 | A | C | 80 | Slc26a7 | intronic | . | . | . | 4qA1 | . | 0 | 1 |
| chr4 | 18011740 | C | T | 189 | Mmp16 | exonic | synonymous SNV | Mmp16:NM_019724:exon4:c.C654T:p.G218G | . | 4qA3 | . | 0 | 1 |
| chr4 | 18051954 | A | T | 222 | Mmp16 | intronic | . | . | . | 4qA3 | . | 0 | 1 |
| chr4 | 18112049 | A | C | 221 | Mmp16 | exonic | missense SNV | Mmp16:NM_019724:exon9:c.A1424C:p.K475T | . | 4qA3 | . | 0 | 1 |
| chr4 | 18845864 | G | C | 167 | . | intergenic | . | . | . | 4qA3 | . | 1 | 0 |
| chr4 | 19122183 | T | A | 96 | . | intergenic | . | . | . | 4qA3 | . | 0 | 1 |
| chr4 | 19601272 | A | C | 69 | Rmdn1 | intronic | . | . | . | 4qA3 | . | 0 | 1 |
| chr4 | 19643527 | A | C | 132 | Wwp1 | intronic | . | . | . | 4qA3 | . | 0 | 1 |
| chr4 | 19672349 | G | A | 117 | Wwp1 | intronic | . | . | . | 4qA3 | . | 0 | 1 |
| chr4 | 19672582 | C | A | 188 | Wwp1 | intronic | . | . | . | 4qA3 | . | 0 | 1 |
| chr4 | 19910545 | G | C | 194 | Atp6v0d2 | intronic | . | . | . | 4qA3 | . | 0 | 1 |
| chr4 | 20021208 | A | C | 168 | Ttpa | exonic | missense SNV | Ttpa:NM_001317723:exon3:c.A168C:p.K56N,Ttpa:NM_015767:exon3:c.A375C:p.K125N | . | 4qA3 | . | 0 | 1 |
| chr4 | 21678768 | T | G | 228 | Prdm13 | exonic | missense SNV | Prdm13:NM_001080771:exon4:c.A1721C:p.Q574P | 1657 | 4qA3 | . | 1 | 0 |
| chr4 | 21830235 | A | T | 140 | Usp45 | intronic | . | . | . | 4qA3 | . | 1 | 0 |
| chr4 | 21873684 | C | G | 228 | Pnisr | exonic | missense SNV | Pnisr:NM_025669:exon11:c.C1426G:p.R476G | . | 4qA3 | rs13460207 | 1 | 0 |
| chr4 | 24588404 | T | C | 150 | Mms221 | intronic | . | . | . | 4qA3 | . | 0 | 1 |
| chr4 | 24901724 | G | A | 87 | Ndufaf4 | exonic | missense SNV | Ndufaf4:NM_026742:exon3:c.G271A:p.E91K | . | 4qA3 | . | 0 | 1 |
| chr4 | 26336669 | A | G | 158 | Manea | exonic | synonymous SNV | Manea:NM_172865:exon3:c.T606C:p.D202D | . | 4qA3 | . | 0 | 1 |
| chr4 | 27867743 | T | G | 152 | . | intergenic | . | . | . | 4qA3 | . | 0 | 1 |
| chr4 | 28149885 | A | G | 74 | . | intergenic | . | . | . | 4qA3 | . | 0 | 1 |
| chr4 | 28947341 | C | T | 63 | Epha7 | UTR3 | . | . | . | 4qA4 | . | 0 | 1 |
| chr4 | 32801358 | T | A | 109 | Lyrm2 | UTR3 | . | . | . | 4qA5 | . | 0 | 1 |
| chr4 | 32817184 | C | A | 228 | Ankrd6 | intronic | . | . | . | 4qA5 | . | 1 | 0 |
| chr4 | 33331105 | T | C | 113 | Rngtt | intronic | . | . | . | 4qA5 | . | 0 | 1 |
| chr4 | 33946670 | G | T | 228 | Cnr1 | UTR3 | . | . | . | 4qA5 | . | 1 | 0 |
| chr4 | 34614966 | G | A | 126 | Rars2 | UTR5 | . | . | 388 | 4qA5 | . | 0 | 1 |
| chr4 | 34807455 | T | C | 221 | Zfp292 | exonic | missense SNV | Zfp292:NM_013889:exon8:c.A5603G:p.H1868R | . | 4qA5 | . | 0 | 1 |
| chr4 | 34819667 | A | T | 228 | Zfp292 | intronic | . | . | . | 4qA5 | . | 1 | 0 |
| chr4 | 41021795 | A | C | 205 | Nfx1 | exonic | missense SNV | Nfx1:NM_023739:exon20:c.A2902C:p.N968H | . | 4qA5 | . | 0 | 1 |
| chr4 | 41115961 | G | T | 221 | Nol6 | exonic | missense SNV | Nol6:NM_139236:exon24:c.C3130A:p.L1044I | . | 4qA5 | . | 0 | 1 |
| chr4 | 41625095 | C | T | 112 | Dnaic1 | intronic | . | . | . | 4qA5 | . | 0 | 1 |
| chr4 | 41725999 | T | C | 57 | Arid3c | exonic | synonymous SNV | Arid3c:NM_001017362:exon5:c.A603G:p.T201T,Arid3c:NM_001252622:exon5:c.A603G:p.T201T | 519 | 4qA5 | . | 0 | 1 |
| chr4 | 43245708 | C | A | 198 | Unc13b | intronic | . | . | . | 4qA5 | . | 0 | 1 |
| chr4 | 43510175 | T | C | 134 | Car9 | intronic | . | . | . | 4qA5 | . | 0 | 1 |
| chr4 | 43650206 | C | T | 204 | Npr2 | exonic | stopgain | Npr2:NM_173788:exon19:c.C2770T:p.Q924X | . | 4qA5 | . | 0 | 1 |
| chr4 | 43651953 | C | T | 82 | Spag8 | exonic | missense SNV | Spag8:NM_001007463:exon5:c.G1130A:p.R377Q,Spag8:NM_001290462:exon5:c.G1130A:p.R377Q | . | 4qA5 | . | 0 | 1 |
| chr4 | 43690223 | C | G | 81 | Tmem8b | exonic | missense SNV | Tmem8b:NM_001085508:exon13:c.C1280G:p.P427R | . | 4qB1 | . | 0 | 1 |
| chr4 | 43799009 | C | A | 228 | . | intergenic | . | . | . | 4qB1 | . | 1 | 0 |
| chr4 | 43918450 | T | C | 144 | Reck | exonic | synonymous SNV | Reck:NM_016678:exon10:c.T1042C:p.L348L | . | 4qB1 | . | 0 | 1 |
| chr4 | 43942571 | T | G | 204 | Reck | intronic | . | . | . | 4qB1 | . | 1 | 0 |
| chr4 | 43942573 | G | A | 204 | Reck | intronic | . | . | . | 4qB1 | . | 1 | 0 |
| chr4 | 45023219 | C | T | 221 | Polr1e | exonic | missense SNV | Polr1e:NM_001285800:exon4:c.C329T:p.T110I,Polr1e:NM_022811:exon4:c.C329T:p.T110I | . | 4qB1 | . | 0 | 1 |
| chr4 | 45372690 | G | T | 228 | Dcaf10 | exonic | missense SNV | Dcaf10:NM_153167:exon5:c.G1102T:p.G368C | . | 4qB1 | . | 1 | 0 |
| chr4 | 45864960 | C | A | 221 | Stra6l | exonic | missense SNV | Stra6l:NM_028788:exon4:c.C285A:p.F95L,Stra6l:NM_001305421:exon5:c.C12A:p.F4L | . | 4qB1 | . | 0 | 1 |
| chr4 | 47035834 | T | C | 90 | Anks6 | intronic | . | . | . | 4qB1 | . | 0 | 1 |
| chr4 | 47293203 | C | G | 124 | Col15a1 | exonic | synonymous SNV | Col15a1:NM_009928:exon27:c.C2772G:p.P924P | . | 4qB1 | . | 0 | 1 |
| chr4 | 48056362 | C | G | 182 | Nr4a3 | intronic | . | . | . | 4qB1 | . | 1 | 0 |
| chr4 | 48617475 | C | G | 144 | Tmeff1 | intronic | . | . | . | 4qB1 | . | 0 | 1 |
| chr4 | 49541128 | C | T | 132 | Aldob | splicing | . | . | . | 4qB1 | . | 0 | 1 |
| chr4 | 49670000 | G | A | 116 | Grin3a | intronic | . | . | . | 4qB1 | . | 0 | 1 |
| chr4 | 49746941 | T | C | 120 | Grin3a | intronic | . | . | . | 4qB1 | . | 0 | 1 |
| chr4 | 51253824 | A | T | 193 | . | intergenic | . | . | . | 4qB1 | . | 0 | 1 |
| chr4 | 52457566 | C | A | 114 | Smc2 | intronic | . | . | . | 4qB2 | . | 0 | 1 |
| chr4 | 52611244 | A | C | 221 | Topors1 | exonic | missense SNV | Topors1:NM_026652:exon2:c.A1136C:p.K379T | . | 4qB2 | . | 0 | 1 |
| chr4 | 53090400 | A | G | 221 | Abca1 | intronic | . | . | . | 4qB2 | . | 0 | 1 |
| chr4 | 55012037 | G | T | 222 | Zfp462 | exonic | missense SNV | Zfp462:NM_172867:exon3:c.G4002T:p.M1334I | . | 4qB2 | . | 0 | 1 |
| chr4 | 56784447 | T | C | 86 | Ikbkap | intronic | . | . | . | 4qB3 | . | 0 | 1 |
| chr4 | 56925997 | C | T | 135 | Tmem245 | intronic | . | . | . | 4qB3 | . | 1 | 0 |
| chr4 | 57076857 | T | C | 125 | Epb4114b | intronic | . | . | . | 4qB3 | . | 0 | 1 |
| chr4 | 58146769 | T | C | 105 | Svep1 | intronic | . | . | . | 4qB3 | . | 0 | 1 |
| chr4 | 58812855 | G | A | 76 | AI314180 | intronic | . | . | . | 4qB3 | . | 0 | 1 |
| chr4 | 58878999 | A | T | 172 | AI314180 | intronic | . | . | . | 4qB3 | . | 0 | 1 |
| chr4 | 59090966 | A | T | 228 | . | intergenic | . | . | . | 4qB3 | . | 1 | 0 |
| chr4 | 59720430 | A | T | 222 | E130308A19Rik | exonic | missense SNV | E130308A19Rik:NM_153158:exon3:c.A1961T:p.Y654F | . | 4qB3 | . | 0 | 1 |
| chr4 | 59892385 | G | A | 117 | Snx30 | intronic | . | . | . | 4qB3 | . | 0 | 1 |
| chr4 | 61832214 | C | T | 206 | Mup5 | intronic | . | . | . | 4qB3 | rs45698496 | 0 | 1 |
| chr4 | 61832443 | G | A | 222 | Mup5 | intronic | . | . | . | 4qB3 | . | 0 | 1 |
| chr4 | 61832868 | T | A | 172 | Mup5 | intronic | . | . | . | 4qB3 | rs263342962 | 0 | 1 |
| chr4 | 61857473 | C | T | 222 | . | intergenic | . | . | . | 4qB3 | rs47993883 | 0 | 1 |
| chr4 | 61857529 | T | G | 222 | . | intergenic | . | . | . | 4qB3 | rs32162460 | 0 | 1 |
| chr4 | 62455748 | T | C | 228 | Wdr31 | intronic | . | . | . | 4qB3 | rs31833269 | 1 | 0 |
| chr4 | 63224191 | T | C | 51 | Col27a1 | intronic | . | . | . | 4qB3 | . | 0 | 1 |
| chr4 | 63277577 | A | C | 77 | Col27a1 | intronic | . | . | . | 4qB3 | . | 0 | 1 |
| chr4 | 63346587 | G | C | 153 | Orm1 | intronic | . | . | . | 4qB3 | . | 1 | 0 |
| chr4 | 63356317 | A | G | 221 | Orm3 | splicing | . | . | . | 4qB3 | . | 0 | 1 |
| chr4 | 63362672 | C | T | 228 | Orm2 | intronic | . | . | . | 4qB3 | . | 1 | 0 |
| chr4 | 63374571 | C | T | 222 | Akna | intronic | . | . | . | 4qC1 | . | 0 | 1 |
| chr4 | 63378767 | C | A | 172 | Akna | intronic | . | . | . | 4qC1 | . | 1 | 0 |
| chr4 | 63378768 | C | A | 171 | Akna | intronic | . | . | . | 4qC1 | . | 1 | 0 |
| chr4 | 63415593 | A | C | 228 | Whrn | exonic | missense SNV | Whrn:NM_001008792:exon12:c.T2627G:p.L876R,Whrn:NM_001008793:exon12:c.T2612G:p.L871R,Whrn:NM_ | . | 4qC1 | . | 1 | 0 |
| chr4 | 63834707 | G | C | 104 | Tnfsf8 | intronic | . | . | . | 4qC1 | rs32528882 | 1 | 0 |
| chr4 | 63960499 | C | T | 222 | Tnc | exonic | synonymous SNV | Tnc:NM_011607:exon26:c.G5994A:p.Q1998Q | . | 4qC1 | . | 0 | 1 |
| chr4 | 65307744 | A | G | 221 | Pappa | exonic | missense SNV | Pappa:NM_021362:exon15:c.A3833G:p.E1278G | . | 4qC1 | . | 0 | 1 |
| chr4 | 65381771 | A | G | 70 | Astn2 | intronic | . | . | . | 4qC1 | . | 0 | 1 |
| chr4 | 65435170 | T | C | 228 | Astn2 | exonic | missense SNV | Astn2:NM_019514:exon19:c.A3362G:p.E1121G,Astn2:NM_207109:exon20:c.A3518G:p.E1173G | . | 4qC1 | . | 1 | 0 |
| chr4 | 65542681 | A | G | 222 | Astn2 | exonic | missense SNV | Astn2:NM_019514:exon17:c.T3059C:p.L1020P,Astn2:NM_207109:exon18:c.T3215C:p.L1072P | . | 4qC1 | . | 0 | 1 |
| chr4 | 65911472 | A | G | 122 | Astn2 | intronic | . | . | . | 4qC1 | . | 1 | 0 |
| chr4 | 65992319 | A | G | 216 | Astn2 | exonic | synonymous SNV | Astn2:NM_019514:exon5:c.T1275C:p.T425T,Astn2:NM_207109:exon6:c.T1431C:p.T477T | . | 4qC1 | . | 0 | 1 |
| chr4 | 66185444 | C | T | 222 | Astn2 | exonic | missense SNV | Astn2:NM_019514:exon3:c.G731A:p.R244H,Astn2:NM_207109:exon3:c.G731A:p.R244H | . | 4qC1 | . | 0 | 1 |
| chr4 | 69853880 | T | C | 38.7414 | . | intergenic | . | . | . | 4qC2 | . | 0 | 1 |
| chr4 | 69854143 | A | C | 210 | . | intergenic | . | . | . | 4qC2 | . | 0 | 1 |
| chr4 | 70337278 | G | A | 94 | Cdk5rap2 | intronic | . | . | . | 4qC2 | . | 0 | 1 |
| chr4 | 70349027 | A | G | 93 | Cdk5rap2 | intronic | . | . | . | 4qC2 | . | 0 | 1 |
| chr4 | 71679615 | A | G | 50 | . | intergenic | . | . | . | 4qC2 | . | 0 | 1 |
| chr4 | 71757621 | C | A | 65 | . | intergenic | . | . | . | 4qC2 | rs48009195 | 1 | 0 |
| chr4 | 72145424 | C | T | 221 | Tle1 | splicing | . | . | . | 4qC3 | . | 0 | 1 |
| chr4 | 72158288 | A | C | 124 | Tle1 | exonic | missense SNV | Tle1:NM_001285529:exon7:c.T499G:p.S167A,Tle1:NM_001285531:exon7:c.T502G:p.S168A,Tle1:NM_0012 | . | 4qC3 | . | 0 | 1 |
| chr4 | 73740549 | G | T | 199 | Rasef | exonic | synonymous SNV | Rasef:NM_001017427:exon12:c.C1176A:p.G392G | . | 4qC3 | . | 0 | 1 |
| chr4 | 73745156 | A | T | 228 | Rasef | intronic | . | . | . | 4qC3 | . | 1 | 0 |
| chr4 | 73748736 | G | T | 76 | Rasef | intronic | . | . | . | 4qC3 | . | 0 | 1 |
| chr4 | 73942934 | A | G | 221 | 2310002L09Rik | exonic | missense SNV | 2310002L09Rik:NM_027104:exon2:c.T428C:p.V143A,2310002L09Rik:NM_027981:exon2:c.T428C:p.V143A | . | 4qC3 | . | 0 | 1 |
| chr4 | 74144970 | C | A | 191 | Frmd3 | intronic | . | . | . | 4qC3 | . | 0 | 1 |
| chr4 | 75148653 | A | G | 88 | . | intergenic | . | . | . | 4qC3 | . | 0 | 1 |
| chr4 | 75956219 | A | G | 190 | Ptprd | intronic | . | . | . | 4qC3 | . | 0 | 1 |
| chr4 | 75957520 | T | C | 76 | Ptprd | intronic | . | . | . | 4qC3 | . | 0 | 1 |
| chr4 | 76050347 | C | T | 221 | Ptprd | exonic | missense SNV | Ptprd:NM_011211:exon28:c.G2756A:p.R919H | . | 4qC3 | . | 0 | 1 |
| chr4 | 76091284 | T | C | 110 | Ptprd | intronic | . | . | . | 4qC3 | . | 0 | 1 |
| chr4 | 79328292 | A | C | 222 | . | intergenic | . | . | . | 4qC3 | . | 0 | 1 |
| chr4 | 80846384 | C | A | 43.9904 | Tyrp1 | intronic | . | . | . | 4qC3 | . | 0 | 1 |
| chr4 | 81419170 | A | G | 35.5678 | Mpdz | intronic | . | . | . | 4qC3 | . | 0 | 1 |
| chr4 | 82744444 | A | G | 126 | . | intergenic | . | . | . | 4qC3 | . | 0 | 1 |
| chr4 | 82972391 | C | T | 209 | Frem1 | intronic | . | . | . | 4qC3 | rs32468466 | 1 | 0 |
| chr4 | 84916314 | A | C | 222 | Cntln | intronic | . | . | . | 4qC4 | . | 0 | 1 |
| chr4 | 85355344 | C | A | 228 | Sh3gl2 | exonic | missense SNV | Sh3gl2:NM_019535:exon3:c.C132A:p.S44R | . | 4qC4 | . | 1 | 0 |
| chr4 | 85377557 | G | A | 190 | Sh3gl2 | intronic | . | . | . | 4qC4 | . | 0 | 1 |
| chr4 | 85921197 | T | A | 73 | . | intergenic | . | . | . | 4qC4 | . | 0 | 1 |
| chr4 | 86199098 | A | G | 153 | Adamtsl1 | intronic | . | . | . | 4qC4 | rs28032714 | 1 | 0 |
| chr4 | 86199111 | C | T | 153 | Adamtsl1 | intronic | . | . | . | 4qC4 | rs247810108 | 1 | 0 |
| chr4 | 86199170 | C | T | 119 | Adamtsl1 | intronic | . | . | . | 4qC4 | rs244456368 | 1 | 0 |
| chr4 | 86199239 | G | C | 228 | Adamtsl1 | intronic | . | . | . | 4qC4 | rs28032712 | 1 | 0 |
| chr4 | 86199426 | G | A | 228 | Adamtsl1 | intronic | . | . | . | 4qC4 | rs28032711 | 1 | 0 |
| chr4 | 86212528 | C | A | 164 | Adamtsl1 | intronic | . | . | . | 4qC4 | rs32010485 | 1 | 0 |
| chr4 | 86212530 | G | A | 171 | Adamtsl1 | intronic | . | . | . | 4qC4 | rs31785316 | 1 | 0 |
| chr4 | 86212606 | T | C | 228 | Adamtsl1 | intronic | . | . | . | 4qC4 | rs28032632 | 1 | 0 |
| chr4 | 86212716 | T | C | 228 | Adamtsl1 | intronic | . | . | . | 4qC4 | rs28032631 | 1 | 0 |
| chr4 | 86212728 | A | G | 228 | Adamtsl1 | intronic | . | . | . | 4qC4 | rs28032630 | 1 | 0 |
| chr4 | 86212791 | C | G | 228 | Adamtsl1 | intronic | . | . | . | 4qC4 | rs28032629 | 1 | 0 |
| chr4 | 86216789 | T | C | 164 | Adamtsl1 | intronic | . | . | . | 4qC4 | rs32136680 | 1 | 0 |
| chr4 | 86216828 | A | G | 228 | Adamtsl1 | intronic | . | . | . | 4qC4 | rs28032603 | 1 | 0 |
| chr4 | 86216976 | T | C | 228 | Adamtsl1 | exonic | missense SNV | Adamtsl1:NM_029967:exon7:c.T737C:p.I246T | . | 4qC4 | rs28032602 | 1 | 0 |
| chr4 | 86227892 | A | T | 228 | Adamtsl1 | intronic | . | . | . | 4qC4 | rs108746400 | 1 | 0 |
| chr4 | 86232686 | A | G | 228 | Adamtsl1 | exonic | synonymous SNV | Adamtsl1:NM_029967:exon9:c.A1080G:p.P360P | . | 4qC4 | rs28032541 | 1 | 0 |
| chr4 | 86243779 | G | A | 228 | Adamtsl1 | intronic | . | . | . | 4qC4 | rs28032483 | 1 | 0 |
| chr4 | 86243914 | T | C | 172 | Adamtsl1 | intronic | . | . | . | 4qC4 | rs28032481 | 1 | 0 |
| chr4 | 86243976 | G | A | 143 | Adamtsl1 | intronic | . | . | . | 4qC4 | rs28032479 | 1 | 0 |
| chr4 | 86248294 | A | C | 160 | Adamtsl1 | intronic | . | . | . | 4qC4 | rs28112013 | 1 | 0 |
| chr4 | 86249931 | C | A | 228 | Adamtsl1 | intronic | . | . | . | 4qC4 | rs28112002 | 1 | 0 |
| chr4 | 86252697 | A | G | 202 | Adamtsl1 | intronic | . | . | . | 4qC4 | rs28111986 | 1 | 0 |
| chr4 | 86277117 | G | A | 228 | Adamtsl1 | exonic | synonymous SNV | Adamtsl1:NM_029967:exon13:c.G1770A:p.E590E | . | 4qC4 | rs28095352 | 1 | 0 |
| chr4 | 86277244 | G | A | 228 | Adamtsl1 | intronic | . | . | . | 4qC4 | rs32043278 | 1 | 0 |
| chr4 | 86277310 | C | T | 186 | Adamtsl1 | intronic | . | . | . | 4qC4 | rs28095351 | 1 | 0 |
| chr4 | 86289765 | A | G | 228 | Adamtsl1 | exonic | synonymous SNV | Adamtsl1:NM_029967:exon14:c.A1872G:p.E624E | . | 4qC4 | rs3691415 | 1 | 0 |
| chr4 | 86481578 | T | C | 228 | Saxo1 | intronic | . | . | . | 4qC4 | rs46113068 | 1 | 0 |
| chr4 | 86487892 | C | T | 228 | Saxo1 | exonic | synonymous SNV | Saxo1:NM_001081096:exon2:c.G69A:p.T23T | . | 4qC4 | rs28083052 | 1 | 0 |
| chr4 | 86558091 | G | A | 228 | Saxo1 | UTR5 | . | . | . | 4qC4 | rs28061796 | 1 | 0 |
| chr4 | 86576236 | C | T | 228 | Rraga | exonic | synonymous SNV | Rraga:NM_178376:exon1:c.C318T:p.Y106Y | . | 4qC4 | rs13469030 | 1 | 0 |
| chr4 | 86582966 | T | C | 228 | Haus6 | exonic | synonymous SNV | Haus6:NM_173400:exon16:c.A2667G:p.V889V | . | 4qC4 | rs28061697 | 1 | 0 |
| chr4 | 86583085 | G | A | 228 | Haus6 | exonic | missense SNV | Haus6:NM_173400:exon16:c.C2548T:p.P850S | . | 4qC4 | rs28061696 | 1 | 0 |
| chr4 | 86583251 | A | T | 228 | Haus6 | exonic | synonymous SNV | Haus6:NM_173400:exon16:c.T2382A:p.A794A | . | 4qC4 | rs28061695 | 1 | 0 |
| chr4 | 86600812 | A | T | 228 | Haus6 | intronic | . | . | . | 4qC4 | . | 1 | 0 |
| chr4 | 86601117 | A | G | 168 | Haus6 | intronic | . | . | . | 4qC4 | rs28061624 | 1 | 0 |
| chr4 | 86605749 | C | T | 228 | Haus6 | intronic | . | . | . | 4qC4 | rs3688480 | 1 | 0 |
| chr4 | 86627250 | G | A | 228 | . | intergenic | . | . | . | 4qC4 | rs28111131 | 1 | 0 |
| chr4 | 86629549 | C | G | 228 | . | intergenic | . | . | . | 4qC4 | rs32174726 | 1 | 0 |
| chr4 | 86631738 | A | T | 101 | . | intergenic | . | . | . | 4qC4 | rs28111121 | 1 | 0 |
| chr4 | 86656809 | G | C | 168 | Plin2 | UTR3 | . | . | . | 4qC4 | rs13473283 | 1 | 0 |
| chr4 | 86661976 | G | T | 228 | Plin2 | exonic | missense SNV | Plin2:NM_007408:exon5:c.C547A:p.L183M | . | 4qC4 | . | 1 | 0 |
| chr4 | 86774485 | T | C | 228 | Dennd4c | exonic | synonymous SNV | Dennd4c:NM_184088:exon2:c.T231C:p.Y77Y | . | 4qC4 | rs28094952 | 1 | 0 |
| chr4 | 86781513 | A | T | 228 | Dennd4c | intronic | . | . | . | 4qC4 | rs261669790 | 1 | 0 |
| chr4 | 86789723 | A | G | 167 | Dennd4c | intronic | . | . | . | 4qC4 | rs28094896 | 1 | 0 |
| chr4 | 86791444 | A | G | 228 | Dennd4c | exonic | synonymous SNV | Dennd4c:NM_184088:exon9:c.A1281G:p.L427L | . | 4qC4 | rs28094889 | 1 | 0 |
| chr4 | 86798154 | T | A | 228 | Dennd4c | intronic | . | . | . | 4qC4 | rs32750223 | 1 | 0 |
| chr4 | 86799277 | G | T | 210 | Dennd4c | intronic | . | . | . | 4qC4 | rs28079855 | 1 | 0 |
| chr4 | 86813694 | T | C | 181 | Dennd4c | intronic | . | . | . | 4qC4 | . | 0 | 1 |
| chr4 | 86819804 | T | G | 228 | Dennd4c | intronic | . | . | . | 4qC4 | rs31964354 | 1 | 0 |
| chr4 | 86829420 | T | A | 228 | Dennd4c | intronic | . | . | . | 4qC4 | rs247287799 | 1 | 0 |
| chr4 | 86837409 | G | A | 228 | Dennd4c | exonic | missense SNV | Dennd4c:NM_184088:exon27:c.G4837A:p.V1613I | . | 4qC4 | rs243866988 | 1 | 0 |
| chr4 | 86837590 | G | C | 228 | Dennd4c | intronic | . | . | . | 4qC4 | rs28079641 | 1 | 0 |
| chr4 | 86837688 | G | T | 172 | Dennd4c | intronic | . | . | . | 4qC4 | rs251803581 | 1 | 0 |
| chr4 | 86839490 | T | G | 175 | Dennd4c | intronic | . | . | . | 4qC4 | rs28079630 | 1 | 0 |
| chr4 | 86840820 | C | T | 164 | Dennd4c | intronic | . | . | . | 4qC4 | rs227984981 | 1 | 0 |
| chr4 | 86855020 | T | G | 228 | Rps6 | intronic | . | . | . | 4qC4 | rs28079591 | 1 | 0 |
| chr4 | 86855785 | A | T | 228 | Rps6 | intronic | . | . | . | 4qC4 | rs217031120 | 1 | 0 |
| chr4 | 86855888 | T | G | 228 | Rps6 | exonic | synonymous SNV | Rps6:NM_009096:exon4:c.A475C:p.R159R | . | 4qC4 | rs213471525 | 1 | 0 |
| chr4 | 86856058 | G | A | 228 | Rps6 | intronic | . | . | . | 4qC4 | rs32805713 | 1 | 0 |
| chr4 | 86856417 | T | C | 228 | Rps6 | intronic | . | . | . | 4qC4 | rs257652427 | 1 | 0 |
| chr4 | 86857136 | A | G | 115 | Rps6 | intronic | . | . | 1000 | 4qC4 | rs46745799 | 1 | 0 |
| chr4 | 86857176 | G | C | 84 | Rps6 | intronic | . | . | 1000 | 4qC4 | rs247427868 | 1 | 0 |
| chr4 | 86857281 | G | A | 168 | Rps6 | intronic | . | . | 1000 | 4qC4 | rs225279977 | 1 | 0 |

| | | | | | | | | | | | | | |
|---|---|---|---|---|---|---|---|---|---|---|---|---|---|
| chr4 | 86874319 | A | T | 117 | . | upstream | . | . | 687 | 4qC4 | rs47601528 | 1 | 0 |
| chr4 | 86874320 | G | C | 118 | . | upstream | . | . | 687 | 4qC4 | rs48047871 | 1 | 0 |
| chr4 | 86886056 | T | C | 228 | Acer2 | intronic | . | . | . | 4qC4 | rs32947150 | 1 | 0 |
| chr4 | 86886068 | T | C | 228 | Acer2 | intronic | . | . | . | 4qC4 | rs32052415 | 1 | 0 |
| chr4 | 86887225 | C | T | 228 | Acer2 | intronic | . | . | . | 4qC4 | rs28057025 | 1 | 0 |
| chr4 | 86887268 | G | T | 228 | Acer2 | intronic | . | . | . | 4qC4 | rs28057023 | 1 | 0 |
| chr4 | 86887302 | G | T | 201 | Acer2 | intronic | . | . | . | 4qC4 | rs33863687 | 1 | 0 |
| chr4 | 86900790 | T | C | 166 | Acer2 | intronic | . | . | . | 4qC4 | rs242127316 | 1 | 0 |
| chr4 | 86917727 | T | C | 228 | Acer2 | UTR3 | . | . | . | 4qC4 | rs248629581 | 1 | 0 |
| chr4 | 86917742 | T | C | 228 | Acer2 | UTR3 | . | . | . | 4qC4 | rs579642833 | 1 | 0 |
| chr4 | 86991225 | G | A | 227 | Slc24a2 | UTR3 | . | . | . | 4qC4 | rs32077076 | 1 | 0 |
| chr4 | 86991362 | C | A | 228 | Slc24a2 | exonic | synonymous SNV | Slc24a2:NM_001110240:exon10:c.G2118T:p.V706V,Slc24a2:NM_172426:exon11:c.G1983T:p.V661V | . | 4qC4 | rs32338686 | 1 | 0 |
| chr4 | 86996731 | T | C | 228 | Slc24a2 | intronic | . | . | . | 4qC4 | rs32305868 | 1 | 0 |
| chr4 | 86996767 | C | A | 228 | Slc24a2 | intronic | . | . | . | 4qC4 | rs31847401 | 1 | 0 |
| chr4 | 87011591 | C | A | 228 | Slc24a2 | intronic | . | . | . | 4qC4 | rs28110504 | 1 | 0 |
| chr4 | 87032153 | C | T | 228 | Slc24a2 | intronic | . | . | . | 4qC4 | rs49220489 | 1 | 0 |
| chr4 | 87073141 | C | T | 228 | Slc24a2 | intronic | . | . | . | 4qC4 | rs228606108 | 1 | 0 |
| chr4 | 87073173 | A | C | 228 | Slc24a2 | intronic | . | . | . | 4qC4 | rs252309515 | 1 | 0 |
| chr4 | 87075906 | A | T | 122 | Slc24a2 | intronic | . | . | . | 4qC4 | rs28095478 | 1 | 0 |
| chr4 | 87075920 | T | C | 122 | Slc24a2 | intronic | . | . | . | 4qC4 | rs31889866 | 1 | 0 |
| chr4 | 87226697 | C | A | 135 | Slc24a2 | intronic | . | . | . | 4qC4 | rs33002360 | 1 | 0 |
| chr4 | 87789073 | C | T | 110 | Mllt3 | intronic | . | . | . | 4qC4 | rs229278902 | 1 | 0 |
| chr4 | 87789089 | G | T | 127 | Mllt3 | intronic | . | . | . | 4qC4 | rs28047175 | 1 | 0 |
| chr4 | 87840755 | A | G | 228 | Mllt3 | exonic | missense SNV | Mllt3:NM_027326:exon5:c.T1055C:p.M352T | . | 4qC4 | rs28097387 | 1 | 0 |
| chr4 | 88128999 | T | C | 228 | Focad | exonic | synonymous SNV | Focad:NM_001081184:exon4:c.T234C:p.H78H | . | 4qC4 | rs28097593 | 1 | 0 |
| chr4 | 88174680 | C | T | 228 | Focad | exonic | synonymous SNV | Focad:NM_001081184:exon7:c.C562T:p.L188L | . | 4qC4 | rs28082198 | 1 | 0 |
| chr4 | 88174754 | G | A | 228 | Focad | exonic | synonymous SNV | Focad:NM_001081184:exon7:c.G636A:p.A212A | . | 4qC4 | rs28082196 | 1 | 0 |
| chr4 | 88182661 | A | G | 228 | Focad | exonic | synonymous SNV | Focad:NM_001081184:exon9:c.A984G:p.L328L | . | 4qC4 | rs28082152 | 1 | 0 |
| chr4 | 88196852 | A | G | 228 | Focad | exonic | missense SNV | Focad:NM_001081184:exon11:c.A1291G:p.T431A | . | 4qC4 | rs13477828 | 1 | 0 |
| chr4 | 88196952 | T | G | 228 | Focad | exonic | missense SNV | Focad:NM_001081184:exon11:c.T1391G:p.V464G | . | 4qC4 | rs32080423 | 1 | 0 |
| chr4 | 88229691 | C | T | 224 | Focad | intronic | . | . | . | 4qC4 | . | 1 | 0 |
| chr4 | 88230012 | C | T | 228 | Focad | intronic | . | . | . | 4qC4 | . | 1 | 0 |
| chr4 | 88230529 | G | T | 228 | Focad | exonic | missense SNV | Focad:NM_001081184:exon14:c.G1740T:p.Q580H | . | 4qC4 | . | 1 | 0 |
| chr4 | 88232512 | G | A | 172 | Focad | intronic | . | . | . | 4qC4 | rs254301461 | 1 | 0 |
| chr4 | 88232800 | A | G | 228 | Focad | intronic | . | . | . | 4qC4 | rs254172952 | 1 | 0 |
| chr4 | 88232971 | G | A | 115 | Focad | intronic | . | . | . | 4qC4 | . | 1 | 0 |
| chr4 | 88274356 | G | A | 228 | Focad | intronic | . | . | . | 4qC4 | rs32361277 | 1 | 0 |
| chr4 | 88278035 | T | C | 101 | Focad | intronic | . | . | . | 4qC4 | rs49007468 | 1 | 0 |
| chr4 | 88278046 | A | G | 127 | Focad | intronic | . | . | . | 4qC4 | rs31822678 | 1 | 0 |
| chr4 | 88297060 | T | A | 228 | Focad | exonic | missense SNV | Focad:NM_001081184:exon19:c.T2267A:p.F756Y | . | 4qC4 | rs28059373 | 1 | 0 |
| chr4 | 88297091 | G | A | 228 | Focad | exonic | synonymous SNV | Focad:NM_001081184:exon19:c.G2298A:p.S766S | . | 4qC4 | rs28059372 | 1 | 0 |
| chr4 | 88303386 | T | C | 228 | Focad | intronic | . | . | . | 4qC4 | rs28059343 | 1 | 0 |
| chr4 | 88310097 | G | A | 135 | Focad | intronic | . | . | . | 4qC4 | . | 1 | 0 |
| chr4 | 88326136 | C | T | 228 | Focad | intronic | . | . | . | 4qC4 | . | 1 | 0 |
| chr4 | 88336388 | A | G | 165 | Focad | intronic | . | . | . | 4qC4 | rs28059261 | 1 | 0 |
| chr4 | 88344602 | A | G | 228 | Focad | exonic | synonymous SNV | Focad:NM_001081184:exon27:c.A3093G:p.T1031T | . | 4qC4 | rs226039395 | 1 | 0 |
| chr4 | 88355428 | G | A | 228 | Focad | exonic | synonymous SNV | Focad:NM_001081184:exon29:c.G3459A:p.T1153T | . | 4qC4 | . | 1 | 0 |
| chr4 | 88357344 | C | G | 168 | Focad | intronic | . | . | . | 4qC4 | . | 1 | 0 |
| chr4 | 88357611 | G | A | 228 | Focad | intronic | . | . | . | 4qC4 | . | 1 | 0 |
| chr4 | 88360451 | A | G | 228 | Focad | intronic | . | . | . | 4qC4 | rs50743203 | 1 | 0 |
| chr4 | 88368580 | C | T | 228 | Focad | intronic | . | . | . | 4qC4 | . | 1 | 0 |
| chr4 | 88391678 | A | G | 115 | Focad | intronic | . | . | . | 4qC4 | rs28114982 | 1 | 0 |
| chr4 | 88393699 | C | T | 228 | Focad | intronic | . | . | . | 4qC4 | rs28114970 | 1 | 0 |
| chr4 | 88397026 | G | A | 228 | Focad | exonic | missense SNV | Focad:NM_001081184:exon38:c.G4420A:p.V1474M | . | 4qC4 | rs247660708 | 1 | 0 |
| chr4 | 88398177 | A | T | 228 | Focad | intronic | . | . | . | 4qC4 | rs47554633 | 1 | 0 |
| chr4 | 88398198 | A | G | 228 | Focad | intronic | . | . | . | 4qC4 | rs49289577 | 1 | 0 |
| chr4 | 88400978 | G | A | 228 | Focad | intronic | . | . | . | 4qC4 | rs240908978 | 1 | 0 |
| chr4 | 88401085 | C | T | 228 | Focad | exonic | synonymous SNV | Focad:NM_001081184:exon40:c.C4788T:p.S1596S | . | 4qC4 | rs251970305 | 1 | 0 |
| chr4 | 88403205 | T | A | 228 | Focad | intronic | . | . | . | 4qC4 | rs229133301 | 1 | 0 |
| chr4 | 88403392 | A | T | 228 | Focad | exonic | missense SNV | Focad:NM_001081184:exon41:c.A4979T:p.N1660I | . | 4qC4 | . | 1 | 0 |
| chr4 | 88403427 | A | G | 228 | Focad | intronic | . | . | . | 4qC4 | rs33001146 | 1 | 0 |
| chr4 | 88408761 | T | A | 171 | Focad | intronic | . | . | . | 4qC4 | rs28100256 | 1 | 0 |
| chr4 | 88414119 | G | T | 139 | Hacd4 | UTR3 | . | . | . | 4qC4 | rs32776497 | 1 | 0 |
| chr4 | 88414188 | G | A | 228 | Hacd4 | UTR3 | . | . | . | 4qC4 | . | 1 | 0 |
| chr4 | 88414265 | C | T | 228 | Hacd4 | exonic | synonymous SNV | Hacd4:NM_025760:exon7:c.G698A:p.X233X | . | 4qC4 | rs28100224 | 1 | 0 |
| chr4 | 88414285 | A | G | 228 | Hacd4 | exonic | synonymous SNV | Hacd4:NM_025760:exon7:c.T678C:p.S226S | . | 4qC4 | . | 1 | 0 |
| chr4 | 88414422 | T | C | 169 | Hacd4 | intronic | . | . | . | 4qC4 | . | 1 | 0 |
| chr4 | 88420015 | A | C | 110 | Hacd4 | intronic | . | . | . | 4qC4 | rs28100211 | 1 | 0 |
| chr4 | 88423272 | A | G | 200 | Hacd4 | intronic | . | . | . | 4qC4 | rs28100195 | 1 | 0 |
| chr4 | 88434996 | T | C | 228 | Hacd4 | exonic | synonymous SNV | Hacd4:NM_025760:exon3:c.A213G:p.E71E | . | 4qC4 | rs6189955 | 1 | 0 |
| chr4 | 91308873 | A | C | 222 | Elavl2 | intronic | . | . | . | 4qC5 | . | 0 | 1 |
| chr4 | 91365557 | C | G | 34.749 | Elavl2 | intronic | . | . | . | 4qC5 | . | 0 | 1 |
| chr4 | 91806462 | T | A | 221 | . | intergenic | . | . | . | 4qC5 | . | 0 | 1 |
| chr4 | 93109553 | G | A | 228 | . | intergenic | . | . | . | 4qC5 | rs28161336 | 1 | 0 |
| chr4 | 93265662 | C | A | 222 | . | intergenic | . | . | . | 4qC5 | rs28125340 | 1 | 0 |
| chr4 | 93265824 | A | G | 228 | . | intergenic | . | . | . | 4qC5 | rs243601478 | 1 | 0 |
| chr4 | 93265851 | G | A | 228 | . | intergenic | . | . | . | 4qC5 | rs28125335 | 1 | 0 |
| chr4 | 93266186 | G | A | 75 | . | intergenic | . | . | . | 4qC5 | rs28125331 | 1 | 0 |
| chr4 | 93745682 | C | G | 142 | . | intergenic | . | . | . | 4qC5 | rs28138440 | 1 | 0 |
| chr4 | 93802372 | G | T | 228 | . | intergenic | . | . | . | 4qC5 | rs261322579 | 1 | 0 |
| chr4 | 93802522 | A | C | 228 | . | intergenic | . | . | . | 4qC5 | rs31788412 | 1 | 0 |
| chr4 | 93802523 | G | A | 228 | . | intergenic | . | . | . | 4qC5 | rs237718843 | 1 | 0 |
| chr4 | 94521079 | A | C | 228 | Caap1 | exonic | missense SNV | Caap1:NM_026368:exon5:c.T662G:p.V221G | . | 4qC5 | rs28157476 | 1 | 0 |
| chr4 | 94556862 | T | C | 228 | . | upstream | . | . | . | 4qC5 | rs32821877 | 1 | 0 |
| chr4 | 94556937 | C | G | 228 | . | upstream | . | . | . | 4qC5 | rs32811266 | 1 | 0 |
| chr4 | 94582773 | G | T | 191 | Plaa | intronic | . | . | . | 4qC5 | rs32203925 | 1 | 0 |
| chr4 | 94582904 | G | A | 131 | Plaa | intronic | . | . | . | 4qC5 | rs28157380 | 1 | 0 |
| chr4 | 94586104 | T | C | 133 | Plaa | intronic | . | . | . | 4qC5 | rs31768758 | 1 | 0 |
| chr4 | 94586571 | T | A | 228 | Plaa | intronic | . | . | . | 4qC5 | rs32423243 | 1 | 0 |
| chr4 | 94587475 | C | T | 228 | Plaa | intronic | . | . | . | 4qC5 | rs28157369 | 1 | 0 |
| chr4 | 94600149 | A | G | 115 | Gm12657,Plaa | UTR3 | . | . | . | 4qC5 | rs33011495 | 1 | 0 |
| chr4 | 94873868 | C | A | 197 | Tek | intronic | . | . | . | 4qC5 | . | 0 | 1 |
| chr4 | 96227650 | G | T | 228 | . | intergenic | . | . | . | 4qC5 | . | 1 | 0 |
| chr4 | 96356347 | T | G | 87 | . | intergenic | . | . | . | 4qC5 | . | 0 | 1 |
| chr4 | 96444734 | A | G | 206 | Cyp2j8 | exonic | synonymous SNV | Cyp2j8:NM_001104927:exon9:c.T1374C:p.S458S | . | 4qC5 | . | 0 | 1 |
| chr4 | 96470871 | A | T | 43.9762 | Cyp2j8 | intronic | . | . | . | 4qC5 | . | 0 | 1 |
| chr4 | 96478969 | G | A | 158 | Cyp2j8 | intronic | . | . | . | 4qC5 | . | 0 | 1 |
| chr4 | 96503883 | C | A | 228 | Cyp2j8 | exonic | missense SNV | Cyp2j8:NM_001104927:exon2:c.G333T:p.L111F | . | 4qC5 | . | 1 | 0 |
| chr4 | 96536047 | G | A | 222 | Cyp2j6 | exonic | missense SNV | Cyp2j6:NM_010008:exon3:c.C472T:p.R158C | . | 4qC5 | . | 0 | 1 |
| chr4 | 97690590 | G | A | 228 | Nfia | intronic | . | . | . | 4qC6 | rs28081019 | 1 | 0 |
| chr4 | 97744028 | T | C | 121 | Nfia | intronic | . | . | . | 4qC6 | rs215821987 | 1 | 0 |
| chr4 | 97744351 | G | T | 221 | Nfia | intronic | . | . | . | 4qC6 | . | 0 | 1 |
| chr4 | 97782804 | A | T | 228 | Nfia | intronic | . | . | . | 4qC6 | rs32042449 | 1 | 0 |
| chr4 | 97783500 | G | A | 221 | Nfia | exonic | missense SNV | Nfia:NM_001122952:exon2:c.G515A:p.R172H,Nfia:NM_001122953:exon2:c.G446A:p.R149H,Nfia:NM_0109( | . | 4qC6 | . | 0 | 1 |
| chr4 | 97956552 | G | A | 228 | Nfia | intronic | . | . | . | 4qC6 | rs28130300 | 1 | 0 |
| chr4 | 98000508 | A | T | 228 | Nfia | intronic | . | . | . | 4qC6 | . | 1 | 0 |
| chr4 | 98014411 | T | C | 228 | Nfia | intronic | . | . | . | 4qC6 | rs28094222 | 1 | 0 |
| chr4 | 98021053 | T | C | 228 | Nfia | intronic | . | . | . | 4qC6 | rs28094156 | 1 | 0 |
| chr4 | 98041696 | G | T | 228 | Nfia | intronic | . | . | . | 4qC6 | rs28094022 | 1 | 0 |
| chr4 | 98268140 | C | T | 161 | . | intergenic | . | . | . | 4qC6 | rs28094435 | 1 | 0 |
| chr4 | 98268169 | G | A | 105 | . | intergenic | . | . | . | 4qC6 | rs28094433 | 1 | 0 |
| chr4 | 98268585 | A | G | 228 | . | intergenic | . | . | . | 4qC6 | rs28094428 | 1 | 0 |
| chr4 | 98322396 | G | A | 213 | . | intergenic | . | . | . | 4qC6 | . | 0 | 1 |
| chr4 | 98357765 | C | T | 117 | Tm2d1 | intronic | . | . | . | 4qC6 | rs28078326 | 1 | 0 |
| chr4 | 98357769 | T | A | 117 | Tm2d1 | intronic | . | . | . | 4qC6 | rs32118674 | 1 | 0 |
| chr4 | 98370364 | C | T | 203 | Tm2d1 | intronic | . | . | . | 4qC6 | rs28078242 | 1 | 0 |
| chr4 | 98432227 | C | T | 202 | Inadl | intronic | . | . | . | 4qC6 | rs252880047 | 1 | 0 |
| chr4 | 98469541 | A | C | 221 | Inadl | exonic | missense SNV | Inadl:NM_001005784:exon17:c.A2055C:p.E685D,Inadl:NM_172696:exon17:c.A2055C:p.E685D | . | 4qC6 | . | 0 | 1 |
| chr4 | 98469591 | T | A | 222 | Inadl | exonic | missense SNV | Inadl:NM_001005784:exon17:c.T2105A:p.I702N,Inadl:NM_172696:exon17:c.T2105A:p.I702N | . | 4qC6 | . | 0 | 1 |
| chr4 | 98505503 | C | G | 115 | Inadl | intronic | . | . | . | 4qC6 | rs28096820 | 1 | 0 |
| chr4 | 98505560 | T | A | 158 | Inadl | intronic | . | . | . | 4qC6 | rs28096819 | 1 | 0 |
| chr4 | 98505712 | G | C | 228 | Inadl | exonic | missense SNV | Inadl:NM_172696:exon21:c.G2881C:p.A961P | . | 4qC6 | rs222131769 | 1 | 0 |
| chr4 | 98505787 | A | C | 221 | Inadl | exonic | missense SNV | Inadl:NM_172696:exon21:c.A2956C:p.S986R | . | 4qC6 | . | 0 | 1 |
| chr4 | 98505899 | C | T | 228 | Inadl | intronic | . | . | . | 4qC6 | . | 1 | 0 |
| chr4 | 98513464 | A | G | 172 | Inadl | intronic | . | . | . | 4qC6 | rs28096760 | 1 | 0 |
| chr4 | 98518876 | A | C | 228 | Inadl | intronic | . | . | . | 4qC6 | rs28096702 | 1 | 0 |
| chr4 | 98520688 | T | G | 228 | Inadl | intronic | . | . | . | 4qC6 | rs28096692 | 1 | 0 |
| chr4 | 98520821 | G | A | 228 | Inadl | exonic | synonymous SNV | Inadl:NM_172696:exon24:c.G3300A:p.K1100K | . | 4qC6 | rs28096691 | 1 | 0 |
| chr4 | 98520833 | A | G | 228 | Inadl | exonic | synonymous SNV | Inadl:NM_172696:exon24:c.A3312G:p.E1104E | . | 4qC6 | rs28096690 | 1 | 0 |
| chr4 | 98568718 | G | A | 135 | Inadl | intronic | . | . | . | 4qC6 | rs226974821 | 1 | 0 |
| chr4 | 98568740 | A | G | 40.2188 | Inadl | intronic | . | . | . | 4qC6 | . | 0 | 1 |
| chr4 | 98667618 | G | A | 228 | Inadl | exonic | missense SNV | Inadl:NM_001005787:exon9:c.G859A:p.A287T,Inadl:NM_007704:exon9:c.G859A:p.A287T,Inadl:NM_1726( | . | 4qC6 | rs244911511 | 1 | 0 |
| chr4 | 98930721 | T | C | 132 | Usp1 | intronic | . | . | . | 4qC6 | rs234209630 | 1 | 0 |
| chr4 | 98934829 | T | G | 221 | Usp1 | UTR3 | . | . | . | 4qC6 | . | 0 | 1 |
| chr4 | 99218299 | A | G | 128 | Atg4c | intronic | . | . | . | 4qC6 | rs28075462 | 1 | 0 |
| chr4 | 99224299 | T | C | 50 | Atg4c | intronic | . | . | . | 4qC6 | . | 0 | 1 |
| chr4 | 99296042 | T | C | 228 | . | intergenic | . | . | . | 4qC6 | rs31793607 | 1 | 0 |
| chr4 | 99296060 | C | T | 228 | . | intergenic | . | . | . | 4qC6 | rs31915781 | 1 | 0 |
| chr4 | 99296141 | A | C | 228 | . | intergenic | . | . | . | 4qC6 | rs31774248 | 1 | 0 |
| chr4 | 99296469 | C | G | 228 | . | intergenic | . | . | . | 4qC6 | rs47503742 | 1 | 0 |
| chr4 | 99296480 | A | G | 228 | . | intergenic | . | . | . | 4qC6 | rs47404657 | 1 | 0 |
| chr4 | 99658352 | G | A | 228 | Foxd3 | UTR3 | . | . | . | 4qC6 | rs263252833 | 1 | 0 |
| chr4 | 99719355 | G | A | 170 | Alg6 | intronic | . | . | . | 4qC6 | rs32108220 | 1 | 0 |
| chr4 | 99738224 | C | T | 228 | Alg6 | intronic | . | . | . | 4qC6 | rs28107570 | 1 | 0 |
| chr4 | 99738326 | T | C | 180 | Alg6 | intronic | . | . | . | 4qC6 | rs28107569 | 1 | 0 |
| chr4 | 99738360 | A | C | 170 | Alg6 | intronic | . | . | . | 4qC6 | rs28107568 | 1 | 0 |
| chr4 | 99738942 | C | T | 115 | Alg6 | intronic | . | . | . | 4qC6 | rs28107558 | 1 | 0 |
| chr4 | 99738992 | T | A | 158 | Alg6 | intronic | . | . | . | 4qC6 | rs28107557 | 1 | 0 |
| chr4 | 99741390 | G | A | 103 | Alg6 | intronic | . | . | . | 4qC6 | rs52153192 | 1 | 0 |
| chr4 | 99741443 | A | C | 228 | Alg6 | intronic | . | . | . | 4qC6 | rs216069605 | 1 | 0 |
| chr4 | 99741502 | G | A | 228 | Alg6 | intronic | . | . | . | 4qC6 | rs28107548 | 1 | 0 |
| chr4 | 99741687 | T | G | 228 | Alg6 | intronic | . | . | . | 4qC6 | rs28107546 | 1 | 0 |
| chr4 | 99743658 | C | G | 182 | Alg6 | intronic | . | . | . | 4qC6 | rs262791156 | 1 | 0 |
| chr4 | 99743747 | G | A | 228 | Alg6 | intronic | . | . | . | 4qC6 | rs223114981 | 1 | 0 |
| chr4 | 99743782 | G | A | 228 | Alg6 | exonic | synonymous SNV | Alg6:NM_001081264:exon8:c.G522A:p.A174A | . | 4qC6 | rs246330269 | 1 | 0 |
| chr4 | 99743836 | A | C | 228 | Alg6 | exonic | synonymous SNV | Alg6:NM_001081264:exon8:c.A576C:p.A192A | . | 4qC6 | rs28107537 | 1 | 0 |
| chr4 | 99744085 | A | T | 170 | Alg6 | intronic | . | . | . | 4qC6 | rs28107535 | 1 | 0 |
| chr4 | 99744088 | T | C | 169 | Alg6 | intronic | . | . | . | 4qC6 | rs28107534 | 1 | 0 |
| chr4 | 99744305 | G | A | 172 | Alg6 | intronic | . | . | . | 4qC6 | rs28107533 | 1 | 0 |
| chr4 | 99744501 | T | C | 228 | Alg6 | exonic | missense SNV | Alg6:NM_001081264:exon9:c.T692C:p.F231S | . | 4qC6 | . | 1 | 0 |
| chr4 | 99744589 | T | A | 228 | Alg6 | exonic | synonymous SNV | Alg6:NM_001081264:exon9:c.T780A:p.V260V | . | 4qC6 | rs28107529 | 1 | 0 |
| chr4 | 99746142 | G | A | 135 | Alg6 | intronic | . | . | . | 4qC6 | rs28107516 | 1 | 0 |
| chr4 | 99746279 | T | C | 228 | Alg6 | intronic | . | . | . | 4qC6 | rs51672559 | 1 | 0 |
| chr4 | 99746318 | T | C | 228 | Alg6 | intronic | . | . | . | 4qC6 | rs28107514 | 1 | 0 |
| chr4 | 99746492 | C | G | 206 | Alg6 | intronic | . | . | . | 4qC6 | rs28107512 | 1 | 0 |
| chr4 | 99748679 | C | T | 228 | Alg6 | intronic | . | . | . | 4qC6 | rs28107505 | 1 | 0 |
| chr4 | 99748834 | T | C | 228 | Alg6 | intronic | . | . | . | 4qC6 | rs28107504 | 1 | 0 |
| chr4 | 99769372 | G | A | 163 | Itgb3bp | intronic | . | . | . | 4qC6 | rs31921690 | 1 | 0 |
| chr4 | 99769375 | T | G | 162 | Itgb3bp | intronic | . | . | . | 4qC6 | rs31987771 | 1 | 0 |
| chr4 | 99772318 | G | A | 228 | Itgb3bp | intronic | . | . | . | 4qC6 | . | 1 | 0 |
| chr4 | 99772915 | T | C | 157 | Itgb3bp | intronic | . | . | . | 4qC6 | rs28093324 | 1 | 0 |
| chr4 | 99773203 | C | T | 169 | Itgb3bp | intronic | . | . | . | 4qC6 | rs28093322 | 1 | 0 |
| chr4 | 99781962 | C | T | 228 | Itgb3bp | exonic | synonymous SNV | Itgb3bp:NM_026348:exon6:c.G339A:p.E113E | . | 4qC6 | rs28093292 | 1 | 0 |
| chr4 | 99789806 | A | G | 228 | Itgb3bp | intronic | . | . | . | 4qC6 | rs28093289 | 1 | 0 |
| chr4 | 99790027 | C | T | 228 | Itgb3bp | intronic | . | . | . | 4qC6 | rs28093288 | 1 | 0 |
| chr4 | 99798701 | C | A | 228 | Itgb3bp | exonic | missense SNV | Itgb3bp:NM_026348:exon4:c.G246T:p.R82S | . | 4qC6 | rs28093272 | 1 | 0 |
| chr4 | 99828889 | A | G | 172 | Itgb3bp | intronic | . | . | . | 4qC6 | rs28093154 | 1 | 0 |
| chr4 | 99829062 | G | A | 228 | Itgb3bp | intronic | . | . | . | 4qC6 | rs31894246 | 1 | 0 |
| chr4 | 99829092 | G | C | 228 | Itgb3bp | UTR5 | . | . | 434 | 4qC6 | rs28093153 | 1 | 0 |
| chr4 | 99831374 | A | T | 228 | Efcab7 | intronic | . | . | . | 4qC6 | rs254226025 | 1 | 0 |
| chr4 | 99831375 | T | A | 228 | Efcab7 | intronic | . | . | . | 4qC6 | rs217471297 | 1 | 0 |
| chr4 | 99876313 | A | T | 228 | Efcab7 | intronic | . | . | . | 4qC6 | rs249374076 | 1 | 0 |
| chr4 | 99878410 | T | C | 228 | Efcab7 | intronic | . | . | . | 4qC6 | rs28337589 | 1 | 0 |
| chr4 | 99878681 | T | C | 228 | Efcab7 | intronic | . | . | . | 4qC6 | rs28337587 | 1 | 0 |
| chr4 | 99888756 | C | A | 228 | Efcab7 | intronic | . | . | . | 4qC6 | rs28337558 | 1 | 0 |

| | | | | | | | | | | | | | |
|---|---|---|---|---|---|---|---|---|---|---|---|---|---|
| chr4 | 99892491 | A | C | 228 | Efcab7 | intronic | . | . | . | 4qC6 | rs250548013 | 1 | 0 |
| chr4 | 99897316 | C | G | 228 | Efcab7 | intronic | . | . | . | 4qC6 | rs33891757 | 1 | 0 |
| chr4 | 99900466 | C | T | 228 | Efcab7 | intronic | . | . | . | 4qC6 | rs28338134 | 1 | 0 |
| chr4 | 99929880 | G | A | 228 | Pgm2 | intronic | . | . | 783 | 4qC6 | rs261872185 | 1 | 0 |
| chr4 | 99955856 | C | G | 169 | Pgm2 | intronic | . | . | . | 4qC6 | rs250161154 | 1 | 0 |
| chr4 | 99955873 | A | G | 171 | Pgm2 | intronic | . | . | . | 4qC6 | rs28149292 | 1 | 0 |
| chr4 | 99955980 | A | G | 228 | Pgm2 | intronic | . | . | . | 4qC6 | rs28149291 | 1 | 0 |
| chr4 | 99956085 | A | C | 228 | Pgm2 | intronic | . | . | . | 4qC6 | rs28149290 | 1 | 0 |
| chr4 | 99956290 | T | C | 228 | Pgm2 | intronic | . | . | . | 4qC6 | rs216015955 | 1 | 0 |
| chr4 | 99956462 | G | T | 95 | Pgm2 | intronic | . | . | . | 4qC6 | rs28149287 | 1 | 0 |
| chr4 | 99961352 | G | A | 193 | Pgm2 | intronic | . | . | . | 4qC6 | rs32418684 | 1 | 0 |
| chr4 | 99961488 | A | C | 228 | Pgm2 | exonic | synonymous SNV | Pgm2:NM_028132:exon2:c.A279C:p.I93I | . | 4qC6 | rs13465535 | 1 | 0 |
| chr4 | 99961509 | T | C | 228 | Pgm2 | exonic | synonymous SNV | Pgm2:NM_028132:exon2:c.T300C:p.S100S | . | 4qC6 | rs32325247 | 1 | 0 |
| chr4 | 99961533 | T | C | 228 | Pgm2 | exonic | synonymous SNV | Pgm2:NM_028132:exon2:c.T324C:p.A108A | . | 4qC6 | rs13465536 | 1 | 0 |
| chr4 | 99961756 | G | A | 228 | Pgm2 | intronic | . | . | . | 4qC6 | . | 1 | 0 |
| chr4 | 99962206 | T | C | 228 | Pgm2 | intronic | . | . | . | 4qC6 | rs247871210 | 1 | 0 |
| chr4 | 99963351 | G | A | 168 | Pgm2 | intronic | . | . | . | 4qC6 | rs222278377 | 1 | 0 |
| chr4 | 99963413 | A | G | 199 | Pgm2 | intronic | . | . | . | 4qC6 | rs28149250 | 1 | 0 |
| chr4 | 99963747 | T | C | 228 | Pgm2 | intronic | . | . | . | 4qC6 | rs28149244 | 1 | 0 |
| chr4 | 99964990 | T | C | 211 | Pgm2 | intronic | . | . | . | 4qC6 | rs228875242 | 1 | 0 |
| chr4 | 99969855 | T | C | 169 | Pgm2 | intronic | . | . | . | 4qC6 | rs3718597 | 1 | 0 |
| chr4 | 99969859 | G | A | 169 | Pgm2 | intronic | . | . | . | 4qC6 | rs3719080 | 1 | 0 |
| chr4 | 99970215 | G | A | 228 | Pgm2 | intronic | . | . | . | 4qC6 | rs28149189 | 1 | 0 |
| chr4 | 99970305 | G | A | 127 | Pgm2 | intronic | . | . | . | 4qC6 | rs28149186 | 1 | 0 |
| chr4 | 99978761 | C | T | 82 | Pgm2 | intronic | . | . | . | 4qC6 | rs213614042 | 1 | 0 |
| chr4 | 99978762 | G | A | 81 | Pgm2 | intronic | . | . | . | 4qC6 | rs28130823 | 1 | 0 |
| chr4 | 99979025 | G | C | 228 | Pgm2 | intronic | . | . | . | 4qC6 | rs223682175 | 1 | 0 |
| chr4 | 99979103 | G | T | 228 | Pgm2 | intronic | . | . | . | 4qC6 | rs246189702 | 1 | 0 |
| chr4 | 99982241 | G | A | 110 | Pgm2 | intronic | . | . | . | 4qC6 | . | 1 | 0 |
| chr4 | 99984077 | G | A | 228 | Pgm2 | exonic | missense SNV | Pgm2:NM_028132:exon10:c.G1573A:p.V525I | . | 4qC6 | rs28130772 | 1 | 0 |
| chr4 | 99986476 | C | T | 125 | Pgm2 | intronic | . | . | . | 4qC6 | rs28130750 | 1 | 0 |
| chr4 | 99986964 | T | C | 101 | Pgm2 | UTR3 | . | . | . | 4qC6 | rs28130748 | 1 | 0 |
| chr4 | 100334662 | C | T | 164 | Ror1 | UTR3 | . | . | . | 4qC6 | rs28214980 | 1 | 0 |
| chr4 | 100408001 | A | T | 221 | Ror1 | intronic | . | . | . | 4qC6 | . | 0 | 1 |
| chr4 | 100412368 | C | T | 143 | Ror1 | intronic | . | . | . | 4qC6 | rs28199309 | 1 | 0 |
| chr4 | 100425767 | G | A | 146 | Ror1 | intronic | . | . | . | 4qC6 | rs28176411 | 1 | 0 |
| chr4 | 100426165 | A | G | 228 | Ror1 | intronic | . | . | . | 4qC6 | rs28176409 | 1 | 0 |
| chr4 | 100478973 | A | T | 115 | Ube2u | UTR5 | . | . | . | 4qC6 | rs47016549 | 1 | 0 |
| chr4 | 100481456 | T | G | 171 | Ube2u | intronic | . | . | . | 4qC6 | rs28176181 | 1 | 0 |
| chr4 | 100481492 | T | C | 176 | Ube2u | intronic | . | . | . | 4qC6 | rs28176180 | 1 | 0 |
| chr4 | 100486663 | T | C | 228 | Ube2u | exonic | synonymous SNV | Ube2u:NM_001033773:exon4:c.T258C:p.G86G | . | 4qC6 | rs3658306 | 1 | 0 |
| chr4 | 100486704 | A | G | 228 | Ube2u | exonic | missense SNV | Ube2u:NM_001033773:exon4:c.A299G:p.N100S | . | 4qC6 | rs3658380 | 1 | 0 |
| chr4 | 100490019 | C | T | 228 | Ube2u | intronic | . | . | . | 4qC6 | rs28232625 | 1 | 0 |
| chr4 | 100490366 | T | C | 170 | Ube2u | intronic | . | . | . | 4qC6 | rs28232624 | 1 | 0 |
| chr4 | 100567542 | T | C | 228 | . | intergenic | . | . | . | 4qC6 | rs4231149 | 1 | 0 |
| chr4 | 100567547 | A | G | 228 | . | intergenic | . | . | . | 4qC6 | rs4231150 | 1 | 0 |
| chr4 | 100567685 | A | G | 228 | . | intergenic | . | . | . | 4qC6 | rs4231154 | 1 | 0 |
| chr4 | 100567823 | G | A | 228 | . | intergenic | . | . | . | 4qC6 | rs4231159 | 1 | 0 |
| chr4 | 100567941 | C | T | 228 | . | intergenic | . | . | . | 4qC6 | rs28218035 | 1 | 0 |
| chr4 | 100568033 | T | A | 228 | . | intergenic | . | . | . | 4qC6 | rs28218033 | 1 | 0 |
| chr4 | 100568045 | C | T | 228 | . | intergenic | . | . | . | 4qC6 | rs28218032 | 1 | 0 |
| chr4 | 100568101 | A | G | 228 | . | intergenic | . | . | . | 4qC6 | rs32059488 | 1 | 0 |
| chr4 | 100568199 | G | A | 85 | . | intergenic | . | . | . | 4qC6 | rs28218030 | 1 | 0 |
| chr4 | 100581426 | T | C | 228 | . | intergenic | . | . | . | 4qC6 | rs31966128 | 1 | 0 |
| chr4 | 100581716 | C | A | 228 | . | intergenic | . | . | . | 4qC6 | rs28218002 | 1 | 0 |
| chr4 | 100952072 | C | T | 228 | Cachd1 | exonic | synonymous SNV | Cachd1:NM_198037:exon6:c.C753T:p.A251A | . | 4qC6 | rs32474345 | 1 | 0 |
| chr4 | 100952075 | A | G | 228 | Cachd1 | exonic | synonymous SNV | Cachd1:NM_198037:exon6:c.A756G:p.S252S | . | 4qC6 | rs32506916 | 1 | 0 |
| chr4 | 100952264 | T | A | 228 | Cachd1 | intronic | . | . | . | 4qC6 | rs28177903 | 1 | 0 |
| chr4 | 100952311 | C | T | 179 | Cachd1 | intronic | . | . | . | 4qC6 | rs28177902 | 1 | 0 |
| chr4 | 100959427 | T | C | 228 | Cachd1 | intronic | . | . | . | 4qC6 | rs28177848 | 1 | 0 |
| chr4 | 100964616 | T | C | 228 | Cachd1 | exonic | synonymous SNV | Cachd1:NM_198037:exon9:c.T1389C:p.D463D | . | 4qC6 | rs28177804 | 1 | 0 |
| chr4 | 100965961 | A | G | 135 | Cachd1 | intronic | . | . | . | 4qC6 | rs32952616 | 1 | 0 |
| chr4 | 100966089 | G | C | 210 | Cachd1 | intronic | . | . | . | 4qC6 | rs28177794 | 1 | 0 |
| chr4 | 100966137 | T | C | 228 | Cachd1 | intronic | . | . | . | 4qC6 | rs28177793 | 1 | 0 |
| chr4 | 100966401 | A | G | 228 | Cachd1 | intronic | . | . | . | 4qC6 | rs32979208 | 1 | 0 |
| chr4 | 100966548 | T | C | 134 | Cachd1 | intronic | . | . | . | 4qC6 | rs28177791 | 1 | 0 |
| chr4 | 100967997 | A | T | 228 | Cachd1 | intronic | . | . | . | 4qC6 | rs28177783 | 1 | 0 |
| chr4 | 100968338 | C | T | 173 | Cachd1 | intronic | . | . | . | 4qC6 | rs32202619 | 1 | 0 |
| chr4 | 100975145 | A | G | 161 | Cachd1 | intronic | . | . | . | 4qC6 | rs28177754 | 1 | 0 |
| chr4 | 100980961 | C | T | 228 | Cachd1 | intronic | . | . | . | 4qC6 | rs28177723 | 1 | 0 |
| chr4 | 100981118 | C | T | 228 | Cachd1 | intronic | . | . | . | 4qC6 | rs28177720 | 1 | 0 |
| chr4 | 100987879 | C | G | 168 | Cachd1 | intronic | . | . | . | 4qC6 | rs28177701 | 1 | 0 |
| chr4 | 100992710 | A | G | 228 | Cachd1 | intronic | . | . | . | 4qC6 | rs28177669 | 1 | 0 |
| chr4 | 100993944 | C | T | 72 | Cachd1 | intronic | . | . | . | 4qC6 | . | 0 | 1 |
| chr4 | 100993959 | T | G | 228 | Cachd1 | intronic | . | . | . | 4qC6 | rs28234432 | 1 | 0 |
| chr4 | 100994849 | G | C | 228 | Cachd1 | intronic | . | . | . | 4qC6 | rs28234425 | 1 | 0 |
| chr4 | 101002794 | A | G | 228 | Cachd1 | intronic | . | . | . | 4qC6 | rs46312737 | 1 | 0 |
| chr4 | 101002805 | G | A | 228 | Cachd1 | intronic | . | . | . | 4qC6 | rs28218458 | 1 | 0 |
| chr4 | 101002858 | C | G | 228 | Cachd1 | splicing | . | . | . | 4qC6 | rs28218456 | 1 | 0 |
| chr4 | 101096174 | A | G | 228 | Raver2 | intronic | . | . | . | 4qC6 | rs243850105 | 1 | 0 |
| chr4 | 101096358 | G | A | 228 | Raver2 | intronic | . | . | . | 4qC6 | rs247293961 | 1 | 0 |
| chr4 | 101102434 | T | C | 158 | Raver2 | intronic | . | . | . | 4qC6 | rs257221208 | 1 | 0 |
| chr4 | 101102926 | C | T | 228 | Raver2 | exonic | missense SNV | Raver2:NM_183024:exon3:c.C602T:p.A201V | . | 4qC6 | rs28202395 | 1 | 0 |
| chr4 | 101103026 | G | A | 228 | Raver2 | exonic | synonymous SNV | Raver2:NM_183024:exon3:c.G702A:p.S234S | . | 4qC6 | rs28202394 | 1 | 0 |
| chr4 | 101120616 | A | G | 228 | Raver2 | intronic | . | . | . | 4qC6 | rs28202356 | 1 | 0 |
| chr4 | 101131307 | A | C | 228 | Raver2 | exonic | synonymous SNV | Raver2:NM_183024:exon6:c.A1116C:p.S372S | . | 4qC6 | rs249294504 | 1 | 0 |
| chr4 | 101131355 | G | A | 228 | Raver2 | intronic | . | . | . | 4qC6 | rs28202326 | 1 | 0 |
| chr4 | 101133754 | T | A | 228 | Raver2 | intronic | . | . | . | 4qC6 | rs261999067 | 1 | 0 |
| chr4 | 101133763 | T | C | 228 | Raver2 | intronic | . | . | . | 4qC6 | rs28202311 | 1 | 0 |
| chr4 | 101133869 | G | C | 228 | Raver2 | exonic | synonymous SNV | Raver2:NM_183024:exon8:c.G1275C:p.L425L | . | 4qC6 | rs28202310 | 1 | 0 |
| chr4 | 101135934 | C | T | 228 | Raver2 | intronic | . | . | . | 4qC6 | rs28202292 | 1 | 0 |
| chr4 | 101175042 | C | G | 30.9199 | Jak1 | intronic | . | . | . | 4qC6 | . | 0 | 1 |
| chr4 | 101419469 | A | C | 228 | Ak4 | UTR5 | . | . | 1388 | 4qC6 | rs219865485 | 1 | 0 |
| chr4 | 101460282 | A | G | 168 | Ak4 | intronic | . | . | . | 4qC6 | rs28307147 | 1 | 0 |
| chr4 | 101462943 | A | T | 228 | Ak4 | intronic | . | . | . | 4qC6 | rs46124418 | 1 | 0 |
| chr4 | 101463701 | G | A | 228 | Ak4 | UTR3 | . | . | . | 4qC6 | rs28307133 | 1 | 0 |
| chr4 | 101600712 | T | C | 228 | Dnajc6 | intronic | . | . | . | 4qC6 | rs28207994 | 1 | 0 |
| chr4 | 101611409 | C | T | 228 | Dnajc6 | intronic | . | . | . | 4qC6 | rs28189938 | 1 | 0 |
| chr4 | 101614494 | C | T | 204 | Dnajc6 | intronic | . | . | . | 4qC6 | . | 1 | 0 |
| chr4 | 101615722 | A | T | 228 | Dnajc6 | intronic | . | . | . | 4qC6 | . | 1 | 0 |
| chr4 | 101616481 | A | G | 228 | Dnajc6 | intronic | . | . | . | 4qC6 | rs28189919 | 1 | 0 |
| chr4 | 101616875 | C | T | 228 | Dnajc6 | intronic | . | . | . | 4qC6 | rs28189915 | 1 | 0 |
| chr4 | 101618776 | G | A | 190 | Dnajc6 | exonic | synonymous SNV | Dnajc6:NM_001164583:exon12:c.G1845A:p.P615P,Dnajc6:NM_001164584:exon12:c.G1755A:p.P585P,Dnaj | . | 4qC6 | . | 0 | 1 |
| chr4 | 101647618 | G | A | 128 | . | upstream | . | . | 423 | 4qC6 | rs31772674 | 1 | 0 |
| chr4 | 101652347 | C | T | 228 | Leprot | intronic | . | . | . | 4qC6 | rs32456338 | 1 | 0 |
| chr4 | 101655969 | A | G | 157 | Leprot | intronic | . | . | . | 4qC6 | rs47192684 | 1 | 0 |
| chr4 | 101656084 | T | C | 228 | Leprot | intronic | . | . | . | 4qC6 | rs49866521 | 1 | 0 |
| chr4 | 101656103 | A | C | 228 | Leprot | intronic | . | . | . | 4qC6 | rs51271118 | 1 | 0 |
| chr4 | 101656291 | A | G | 228 | Leprot | exonic | missense SNV | Leprot:NM_175036:exon3:c.A275G:p.D92G | . | 4qC6 | rs45833115 | 1 | 0 |
| chr4 | 101656348 | T | C | 228 | Leprot | intronic | . | . | . | 4qC6 | rs45787623 | 1 | 0 |
| chr4 | 101657711 | C | G | 171 | Leprot | intronic | . | . | . | 4qC6 | rs32890700 | 1 | 0 |
| chr4 | 101657734 | A | G | 228 | Leprot | intronic | . | . | . | 4qC6 | rs32142637 | 1 | 0 |
| chr4 | 101657967 | A | G | 228 | Leprot | UTR3 | . | . | . | 4qC6 | rs264953196 | 1 | 0 |
| chr4 | 101733140 | C | T | 169 | Lepr | intronic | . | . | . | 4qC6 | rs28168512 | 1 | 0 |
| chr4 | 101735291 | G | A | 140 | Lepr | intronic | . | . | . | 4qC6 | rs28168509 | 1 | 0 |
| chr4 | 101735351 | A | G | 171 | Lepr | intronic | . | . | . | 4qC6 | rs32039690 | 1 | 0 |
| chr4 | 101735709 | T | C | 228 | Lepr | intronic | . | . | . | 4qC6 | rs28168508 | 1 | 0 |
| chr4 | 101745610 | G | A | 228 | Lepr | exonic | synonymous SNV | Lepr:NM_001122899:exon5:c.G594A:p.P198P,Lepr:NM_010704:exon5:c.G594A:p.P198P,Lepr:NM_146146:e | . | 4qC6 | rs28168475 | 1 | 0 |
| chr4 | 101764794 | C | T | 228 | Lepr | intronic | . | . | . | 4qC6 | rs28168377 | 1 | 0 |
| chr4 | 101764913 | T | C | 228 | Lepr | exonic | synonymous SNV | Lepr:NM_001122899:exon8:c.T1041C:p.S347S,Lepr:NM_010704:exon8:c.T1041C:p.S347S,Lepr:NM_14614( | . | 4qC6 | rs227961406 | 1 | 0 |
| chr4 | 101764947 | A | G | 228 | Lepr | exonic | missense SNV | Lepr:NM_001122899:exon8:c.A1075G:p.I359V,Lepr:NM_010704:exon8:c.A1075G:p.I359V,Lepr:NM_14614( | . | 4qC6 | rs254610605 | 1 | 0 |
| chr4 | 101765194 | T | C | 228 | Lepr | intronic | . | . | . | 4qC6 | rs265853143 | 1 | 0 |
| chr4 | 101765487 | G | A | 228 | Lepr | intronic | . | . | . | 4qC6 | rs31921422 | 1 | 0 |
| chr4 | 101768464 | C | A | 115 | Lepr | intronic | . | . | . | 4qC6 | rs48279793 | 1 | 0 |
| chr4 | 101768465 | A | G | 115 | Lepr | intronic | . | . | . | 4qC6 | rs49383140 | 1 | 0 |
| chr4 | 101771204 | C | G | 168 | Lepr | intronic | . | . | . | 4qC6 | rs249580660 | 1 | 0 |
| chr4 | 101772841 | T | C | 228 | Lepr | exonic | synonymous SNV | Lepr:NM_001122899:exon12:c.T1752C:p.H584H,Lepr:NM_010704:exon12:c.T1752C:p.H584H,Lepr:NM_146] | . | 4qC6 | rs28224802 | 1 | 0 |
| chr4 | 101779772 | T | C | 178 | Lepr | intronic | . | . | . | 4qC6 | rs248299381 | 1 | 0 |
| chr4 | 101780209 | T | C | 47.3072 | Lepr | intronic | . | . | . | 4qC6 | . | 0 | 1 |
| chr4 | 101789213 | G | A | 191 | Lepr | intronic | . | . | . | 4qC6 | rs28224784 | 1 | 0 |
| chr4 | 101789389 | A | T | 228 | Lepr | intronic | . | . | . | 4qC6 | rs261064905 | 1 | 0 |
| chr4 | 101791881 | G | A | 79 | Lepr | intronic | . | . | . | 4qC6 | . | 0 | 1 |
| chr4 | 101814353 | G | A | 177 | Lepr | intronic | . | . | . | 4qC6 | rs28210604 | 1 | 0 |
| chr4 | 101836637 | T | C | 135 | B020004J07Rik | intronic | . | . | . | 4qC6 | rs239124915 | 1 | 0 |
| chr4 | 101836680 | G | A | 165 | B020004J07Rik | intronic | . | . | . | 4qC6 | rs264877847 | 1 | 0 |
| chr4 | 101837556 | C | T | 228 | B020004J07Rik | intronic | . | . | . | 4qC6 | rs227789205 | 1 | 0 |
| chr4 | 101880713 | T | G | 104 | . | intergenic | . | . | . | 4qC6 | . | 0 | 1 |
| chr4 | 101890588 | T | C | 221 | . | intergenic | . | . | . | 4qC6 | . | 0 | 1 |
| chr4 | 101896315 | C | T | 228 | . | intergenic | . | . | . | 4qC6 | rs262974610 | 1 | 0 |
| chr4 | 101968055 | T | C | 228 | . | intergenic | . | . | . | 4qC6 | rs28193142 | 1 | 0 |
| chr4 | 101968090 | A | C | 228 | . | intergenic | . | . | . | 4qC6 | rs580379544 | 1 | 0 |
| chr4 | 101968092 | T | C | 228 | . | intergenic | . | . | . | 4qC6 | rs584158719 | 1 | 0 |
| chr4 | 101968236 | G | C | 228 | . | intergenic | . | . | . | 4qC6 | rs249620070 | 1 | 0 |
| chr4 | 101988317 | A | G | 228 | Gm12789 | exonic | missense SNV | Gm12789:NM_001085520:exon2:c.A202G:p.T68A | . | 4qC6 | rs586388368 | 1 | 0 |
| chr4 | 101988591 | T | C | 174 | Gm12789 | intronic | . | . | . | 4qC6 | rs220426817 | 1 | 0 |
| chr4 | 101988593 | C | T | 184 | Gm12789 | intronic | . | . | . | 4qC6 | rs245982205 | 1 | 0 |
| chr4 | 101988598 | G | T | 206 | Gm12789 | intronic | . | . | . | 4qC6 | rs264998819 | 1 | 0 |
| chr4 | 101989124 | T | C | 171 | Gm12789 | intronic | . | . | . | 4qC6 | . | 1 | 0 |
| chr4 | 102188657 | G | T | 228 | . | intergenic | . | . | . | 4qC6 | rs254475516 | 1 | 0 |
| chr4 | 102188675 | C | T | 228 | . | intergenic | . | . | . | 4qC6 | rs228240954 | 1 | 0 |
| chr4 | 102188881 | A | C | 199 | . | intergenic | . | . | . | 4qC6 | rs250611169 | 1 | 0 |
| chr4 | 102589982 | C | G | 175 | Pde4b | UTR5 | . | . | . | 4qC6 | rs6239243 | 1 | 0 |
| chr4 | 102589983 | A | G | 176 | Pde4b | UTR5 | . | . | . | 4qC6 | rs6239245 | 1 | 0 |
| chr4 | 102590092 | G | A | 228 | Pde4b | UTR5 | . | . | . | 4qC6 | rs218013854 | 1 | 0 |
| chr4 | 102590631 | A | T | 163 | Pde4b | intronic | . | . | . | 4qC6 | . | 1 | 0 |
| chr4 | 102590716 | A | G | 184 | Pde4b | intronic | . | . | . | 4qC6 | rs32854595 | 1 | 0 |
| chr4 | 102597330 | C | A | 198 | Pde4b | intronic | . | . | . | 4qC6 | rs219882373 | 1 | 0 |
| chr4 | 102597424 | G | A | 194 | Pde4b | intronic | . | . | . | 4qC6 | . | 1 | 0 |
| chr4 | 102597569 | G | A | 228 | Pde4b | intronic | . | . | . | 4qC6 | . | 1 | 0 |
| chr4 | 102597926 | T | C | 228 | Pde4b | intronic | . | . | . | 4qC6 | rs28177537 | 1 | 0 |
| chr4 | 102605381 | G | A | 228 | Pde4b | UTR3 | . | . | . | 4qC6 | . | 1 | 0 |
| chr4 | 102852823 | C | G | 228 | Sgip1 | intronic | . | . | . | 4qC6 | rs28181723 | 1 | 0 |
| chr4 | 102860536 | G | A | 153 | Sgip1 | intronic | . | . | . | 4qC6 | rs28181654 | 1 | 0 |
| chr4 | 102863773 | A | G | 228 | Sgip1 | intronic | . | . | . | 4qC6 | rs28181631 | 1 | 0 |
| chr4 | 102863901 | G | A | 115 | Sgip1 | intronic | . | . | . | 4qC6 | rs229992571 | 1 | 0 |
| chr4 | 102869618 | A | G | 177 | Sgip1 | intronic | . | . | . | 4qC6 | rs49640552 | 1 | 0 |
| chr4 | 102869825 | A | G | 228 | Sgip1 | intronic | . | . | . | 4qC6 | rs237415223 | 1 | 0 |
| chr4 | 102870574 | C | T | 228 | Sgip1 | exonic | synonymous SNV | Sgip1:NM_001285862:exon6:c.C237T:p.S79S,Sgip1:NM_001285859:exon7:c.C306T:p.S102S,Sgip1:NM_001 | . | 4qC6 | rs47932733 | 1 | 0 |
| chr4 | 102870732 | A | G | 228 | Sgip1 | intronic | . | . | . | 4qC6 | rs47410190 | 1 | 0 |
| chr4 | 102870785 | T | C | 228 | Sgip1 | intronic | . | . | . | 4qC6 | rs222303641 | 1 | 0 |
| chr4 | 102870892 | G | A | 156 | Sgip1 | intronic | . | . | . | 4qC6 | rs49167756 | 1 | 0 |
| chr4 | 102905424 | C | T | 228 | Sgip1 | intronic | . | . | . | 4qC6 | rs32601107 | 1 | 0 |
| chr4 | 102909560 | A | T | 170 | Sgip1 | intronic | . | . | . | 4qC6 | rs28163985 | 1 | 0 |
| chr4 | 102914969 | G | A | 176 | Sgip1 | intronic | . | . | . | 4qC6 | . | 1 | 0 |
| chr4 | 102920879 | G | A | 161 | Sgip1 | intronic | . | . | . | 4qC6 | rs32044147 | 1 | 0 |
| chr4 | 102921270 | A | C | 228 | Sgip1 | exonic | missense SNV | Sgip1:NM_144906:exon15:c.A1063C:p.T355P,Sgip1:NM_001285852:exon16:c.A1147C:p.T383P | . | 4qC6 | rs32662586 | 1 | 0 |
| chr4 | 102927398 | G | A | 107 | Sgip1 | intronic | . | . | . | 4qC6 | rs249466900 | 1 | 0 |
| chr4 | 102927480 | A | G | 143 | Sgip1 | exonic | synonymous SNV | Sgip1:NM_001285862:exon12:c.A738G:p.R246R,Sgip1:NM_001285859:exon15:c.A831G:p.R277R,Sgip1:NM | . | 4qC6 | rs32449216 | 1 | 0 |
| chr4 | 102934502 | T | G | 228 | Sgip1 | intronic | . | . | . | 4qC6 | . | 1 | 0 |
| chr4 | 102992353 | G | A | 172 | Tctex1d1 | intronic | . | . | . | 4qC6 | rs226048108 | 1 | 0 |
| chr4 | 103002287 | T | C | 171 | Tctex1d1 | intronic | . | . | . | 4qC6 | rs233570221 | 1 | 0 |
| chr4 | 103003986 | C | T | 153 | Tctex1d1 | intronic | . | . | . | 4qC6 | rs249058459 | 1 | 0 |
| chr4 | 103004372 | C | T | 228 | BB031773,Tctex1d1 | ncRNA_exonic | . | . | . | 4qC6 | rs212309826 | 1 | 0 |
| chr4 | 103011749 | C | T | 177 | BB031773 | ncRNA_intronic | . | . | . | 4qC6 | rs217230901 | 1 | 0 |
| chr4 | 103011886 | C | T | 228 | BB031773 | ncRNA_exonic | . | . | . | 4qC6 | rs28194244 | 1 | 0 |

| | | | | | | | | | | | | | |
|---|---|---|---|---|---|---|---|---|---|---|---|---|---|
| chr4 | 103012033 | A | G | 228 | . | upstream | . | . | . | 4qC6 | rs252790395 | 1 | 0 |
| chr4 | 103012052 | C | T | 228 | . | upstream | . | . | . | 4qC6 | rs220298427 | 1 | 0 |
| chr4 | 103017962 | A | G | 153 | Ins15 | UTR3 | . | . | . | 4qC6 | rs28194214 | 1 | 0 |
| chr4 | 103018024 | G | C | 169 | Ins15 | UTR3 | . | . | . | 4qC6 | . | 1 | 0 |
| chr4 | 103026456 | G | T | 228 | Ins15 | intronic | . | . | . | 4qC6 | rs216993168 | 1 | 0 |
| chr4 | 103026527 | A | C | 228 | Ins15 | exonic | missense SNV | Ins15:NM_011831:exon1:c.T166G:p.S56A,Ins15:NM_001290648:exon2:c.T196G:p.S66A | . | 4qC6 | rs234001624 | 1 | 0 |
| chr4 | 103026549 | T | C | 228 | Ins15 | exonic | synonymous SNV | Ins15:NM_011831:exon1:c.A144G:p.R48R,Ins15:NM_001290648:exon2:c.A174G:p.R58R | . | 4qC6 | rs28194182 | 1 | 0 |
| chr4 | 103026606 | A | G | 228 | Ins15 | exonic | synonymous SNV | Ins15:NM_011831:exon1:c.T87C:p.C29C,Ins15:NM_001290648:exon2:c.T117C:p.C39C | . | 4qC6 | rs28194179 | 1 | 0 |
| chr4 | 103039073 | A | G | 178 | Wdr78 | UTR3 | . | . | . | 4qC6 | rs108910300 | 1 | 0 |
| chr4 | 103059703 | C | A | 228 | Wdr78 | exonic | missense SNV | Wdr78:NM_146254:exon13:c.G1805T:p.G602V | . | 4qC6 | . | 1 | 0 |
| chr4 | 103129358 | C | A | 228 | Mier1 | exonic | synonymous SNV | Mier1:NM_001286221:exon4:c.C114A:p.G38G | . | 4qC6 | rs28155141 | 1 | 0 |
| chr4 | 103139976 | C | T | 166 | Mier1 | intronic | . | . | . | 4qC6 | rs28155112 | 1 | 0 |
| chr4 | 103140010 | C | T | 173 | Mier1 | intronic | . | . | . | 4qC6 | rs28155111 | 1 | 0 |
| chr4 | 103162427 | C | G | 228 | Mier1 | exonic | synonymous SNV | Mier1:NM_001039081:exon13:c.C1212G:p.V404V,Mier1:NM_001286223:exon13:c.C1209G:p.V403V,Mier1:N | . | 4qC6 | rs28155056 | 1 | 0 |
| chr4 | 103173436 | A | G | 101 | Slc35d1 | UTR3 | . | . | . | 4qC6 | rs28155025 | 1 | 0 |
| chr4 | 103231220 | C | T | 228 | 4921539E11Rik | exonic | missense SNV | 4921539E11Rik:NM_001163494:exon8:c.G788A:p.R263H,4921539E11Rik:NM_027612:exon9:c.G1091A:p.R36 | . | 4qC6 | rs28139792 | 1 | 0 |
| chr4 | 103231253 | C | T | 228 | 4921539E11Rik | exonic | missense SNV | 4921539E11Rik:NM_001163494:exon8:c.G755A:p.R252H,4921539E11Rik:NM_027612:exon9:c.G1058A:p.R35 | . | 4qC6 | rs32548772 | 1 | 0 |
| chr4 | 103231373 | A | G | 228 | 4921539E11Rik | exonic | missense SNV | 4921539E11Rik:NM_001163494:exon8:c.T635C:p.V212A,4921539E11Rik:NM_027612:exon9:c.T938C:p.V31 | . | 4qC6 | rs32567926 | 1 | 0 |
| chr4 | 103235736 | T | C | 228 | 4921539E11Rik | exonic | synonymous SNV | 4921539E11Rik:NM_001163494:exon7:c.A387G:p.P129P,4921539E11Rik:NM_027612:exon8:c.A690G:p.P23 | . | 4qC6 | rs28139784 | 1 | 0 |
| chr4 | 103242655 | C | A | 227 | 4921539E11Rik | intronic | . | . | . | 4qC6 | rs28139765 | 1 | 0 |
| chr4 | 103242840 | C | T | 228 | 4921539E11Rik | exonic | synonymous SNV | 4921539E11Rik:NM_001163494:exon5:c.G162A:p.A54A,4921539E11Rik:NM_027612:exon6:c.G465A:p.A155 | . | 4qC6 | rs28139764 | 1 | 0 |
| chr4 | 103242922 | G | A | 228 | 4921539E11Rik | intronic | . | . | . | 4qC6 | rs28139763 | 1 | 0 |
| chr4 | 103255574 | G | A | 228 | 4921539E11Rik | exonic | synonymous SNV | 4921539E11Rik:NM_001163494:exon4:c.C33T:p.D11D,4921539E11Rik:NM_027612:exon5:c.C336T:p.D112D | . | 4qC6 | rs256016193 | 1 | 0 |
| chr4 | 103266653 | A | G | 115 | 4921539E11Rik | intronic | . | . | . | 4qC6 | rs32551286 | 1 | 0 |
| chr4 | 103284168 | T | C | 105 | 4921539E11Rik | intronic | . | . | . | 4qC6 | rs28139677 | 1 | 0 |
| chr4 | 103284194 | T | C | 132 | 4921539E11Rik | intronic | . | . | . | 4qC6 | rs28139676 | 1 | 0 |
| chr4 | 103284605 | G | A | 169 | 4921539E11Rik | intronic | . | . | . | 4qC6 | rs28139674 | 1 | 0 |
| chr4 | 103291515 | T | C | 144 | 4930456L15Rik | ncRNA_exonic | . | . | . | 4qC6 | rs32617046 | 1 | 0 |
| chr4 | 103291829 | T | C | 153 | 4930456L15Rik | ncRNA_intronic | . | . | . | 4qC6 | rs28139617 | 1 | 0 |
| chr4 | 103297468 | T | G | 195 | 4930456L15Rik | ncRNA_intronic | . | . | . | 4qC6 | rs28139591 | 1 | 0 |
| chr4 | 103297657 | C | T | 228 | 4930456L15Rik | ncRNA_exonic | . | . | . | 4qC6 | rs28139589 | 1 | 0 |
| chr4 | 103309291 | A | T | 126 | 4930456L15Rik | ncRNA_intronic | . | . | . | 4qC6 | rs47489852 | 1 | 0 |
| chr4 | 103309299 | T | C | 121 | 4930456L15Rik | ncRNA_intronic | . | . | . | 4qC6 | rs45678846 | 1 | 0 |
| chr4 | 103309395 | G | A | 164 | 4930456L15Rik | ncRNA_intronic | . | . | . | 4qC6 | rs28228422 | 1 | 0 |
| chr4 | 103309497 | G | A | 228 | 4930456L15Rik | ncRNA_intronic | . | . | . | 4qC6 | rs28228421 | 1 | 0 |
| chr4 | 103309525 | C | T | 228 | 4930456L15Rik | ncRNA_exonic | . | . | . | 4qC6 | rs28228420 | 1 | 0 |
| chr4 | 103309643 | A | C | 228 | 4930456L15Rik | ncRNA_intronic | . | . | . | 4qC6 | rs48520629 | 1 | 0 |
| chr4 | 103309656 | T | G | 228 | 4930456L15Rik | ncRNA_intronic | . | . | . | 4qC6 | rs28228418 | 1 | 0 |
| chr4 | 103939403 | G | T | 228 | . | intergenic | . | . | . | 4qC6 | rs51995390 | 1 | 0 |
| chr4 | 103939433 | G | T | 228 | . | intergenic | . | . | . | 4qC6 | rs49462194 | 1 | 0 |
| chr4 | 104604999 | T | G | 76 | Dab1 | intronic | . | . | . | 4qC6 | . | 0 | 1 |
| chr4 | 104791662 | T | A | 136 | C8b | intronic | . | . | . | 4qC6 | . | 0 | 1 |
| chr4 | 104800168 | G | T | 156 | C8b | intronic | . | . | . | 4qC6 | . | 1 | 0 |
| chr4 | 104803798 | T | A | 72 | C8b | intronic | . | . | . | 4qC6 | . | 0 | 1 |
| chr4 | 104945787 | G | T | 228 | 1700024P16Rik | exonic | missense SNV | 1700024P16Rik:NM_001162980:exon2:c.G885T:p.E295D,1700024P16Rik:NM_001285952:exon2:c.G885T:p.E | . | 4qC6 | . | 1 | 0 |
| chr4 | 104950678 | A | G | 77 | 1700024P16Rik | intronic | . | . | . | 4qC6 | . | 0 | 1 |
| chr4 | 105036272 | C | T | 222 | Prkaa2 | exonic | synonymous SNV | Prkaa2:NM_178143:exon9:c.G1479A:p.A493A | . | 4qC6 | . | 0 | 1 |
| chr4 | 106702641 | G | T | 228 | Mroh7 | splicing | . | . | . | 4qC7 | . | 1 | 0 |
| chr4 | 106709989 | G | A | 221 | Mroh7 | exonic | missense SNV | Mroh7:NM_001126487:exon6:c.C1303T:p.R435W | . | 4qC7 | . | 0 | 1 |
| chr4 | 106714038 | T | A | 187 | Mroh7 | intronic | . | . | . | 4qC7 | rs52391406 | 1 | 0 |
| chr4 | 107047341 | T | A | 222 | Ssbp3 | intronic | . | . | . | 4qC7 | . | 0 | 1 |
| chr4 | 107102417 | G | A | 80 | Cdcp2 | intronic | . | . | . | 4qC7 | . | 0 | 1 |
| chr4 | 108022631 | C | A | 228 | Podn | exonic | synonymous SNV | Podn:NM_001285956:exon8:c.G591T:p.V197V,Podn:NM_001285958:exon8:c.G591T:p.V197V,Podn:NM_17287 | . | 4qC7 | . | 1 | 0 |
| chr4 | 108332145 | C | T | 108 | Coa7 | intronic | . | . | . | 4qC7 | . | 0 | 1 |
| chr4 | 108338546 | A | C | 221 | Coa7 | exonic | missense SNV | Coa7:NM_027250:exon3:c.A671C:p.N224T | . | 4qC7 | . | 0 | 1 |
| chr4 | 108627271 | C | T | 221 | Cc2d1b | intronic | . | . | . | 4qC7 | . | 0 | 1 |
| chr4 | 108933618 | A | T | 228 | Rab3b | intronic | . | . | . | 4qC7 | rs28132368 | 1 | 0 |
| chr4 | 109235990 | A | G | 228 | Calr4 | intronic | . | . | . | 4qC7 | rs28183373 | 1 | 0 |
| chr4 | 109238766 | T | C | 228 | Calr4 | intronic | . | . | . | 4qC7 | rs28165959 | 1 | 0 |
| chr4 | 109242097 | C | T | 228 | Calr4 | intronic | . | . | . | 4qC7 | rs50047250 | 1 | 0 |
| chr4 | 109242098 | C | T | 228 | Calr4 | intronic | . | . | . | 4qC7 | rs47837262 | 1 | 0 |
| chr4 | 109244451 | G | A | 116 | Calr4 | intronic | . | . | . | 4qC7 | rs28165921 | 1 | 0 |
| chr4 | 109244651 | T | C | 228 | Calr4 | intronic | . | . | . | 4qC7 | rs28165920 | 1 | 0 |
| chr4 | 109244811 | T | C | 228 | Calr4 | intronic | . | . | . | 4qC7 | rs28165917 | 1 | 0 |
| chr4 | 109323957 | T | C | 190 | Eps15 | intronic | . | . | . | 4qC7 | rs28165718 | 1 | 0 |
| chr4 | 109329523 | T | C | 143 | Eps15 | intronic | . | . | . | 4qC7 | rs32810733 | 1 | 0 |
| chr4 | 109329525 | T | A | 143 | Eps15 | intronic | . | . | . | 4qC7 | rs32050266 | 1 | 0 |
| chr4 | 109343584 | T | G | 85 | Eps15 | intronic | . | . | . | 4qC7 | rs51677314 | 1 | 0 |
| chr4 | 109361030 | C | T | 168 | Eps15 | intronic | . | . | . | 4qC7 | rs28151255 | 1 | 0 |
| chr4 | 109361434 | A | G | 179 | Eps15 | intronic | . | . | . | 4qC7 | rs28151252 | 1 | 0 |
| chr4 | 109361544 | G | A | 162 | Eps15 | intronic | . | . | . | 4qC7 | rs28151249 | 1 | 0 |
| chr4 | 109366192 | T | C | 228 | Eps15 | exonic | synonymous SNV | Eps15:NM_001159964:exon5:c.T801C:p.G267G,Eps15:NM_007943:exon17:c.T1743C:p.G581G | . | 4qC7 | rs28151239 | 1 | 0 |
| chr4 | 109366447 | A | G | 228 | Eps15 | exonic | synonymous SNV | Eps15:NM_001159964:exon6:c.A879G:p.S293S,Eps15:NM_007943:exon18:c.A1821G:p.S607S | . | 4qC7 | rs28151238 | 1 | 0 |
| chr4 | 109366632 | G | A | 164 | Eps15 | intronic | . | . | . | 4qC7 | rs28151237 | 1 | 0 |
| chr4 | 109368996 | C | T | 228 | Eps15 | intronic | . | . | . | 4qC7 | rs28151231 | 1 | 0 |
| chr4 | 109369013 | G | A | 228 | Eps15 | intronic | . | . | . | 4qC7 | rs28151230 | 1 | 0 |
| chr4 | 109370321 | A | G | 85 | Eps15 | intronic | . | . | . | 4qC7 | rs28151220 | 1 | 0 |
| chr4 | 109370352 | G | T | 146 | Eps15 | intronic | . | . | . | 4qC7 | rs28151219 | 1 | 0 |
| chr4 | 109372968 | T | C | 101 | Eps15 | intronic | . | . | . | 4qC7 | rs28151211 | 1 | 0 |
| chr4 | 109372999 | G | C | 157 | Eps15 | intronic | . | . | . | 4qC7 | rs28151210 | 1 | 0 |
| chr4 | 109373246 | C | T | 228 | Eps15 | exonic | synonymous SNV | Eps15:NM_001159964:exon9:c.C1137T:p.D379D,Eps15:NM_007943:exon21:c.C2079T:p.D693D | . | 4qC7 | rs28151209 | 1 | 0 |
| chr4 | 109373778 | T | C | 228 | Eps15 | intronic | . | . | . | 4qC7 | rs28151205 | 1 | 0 |
| chr4 | 109373816 | T | C | 228 | Eps15 | intronic | . | . | . | 4qC7 | rs28151204 | 1 | 0 |
| chr4 | 109374136 | G | C | 228 | Eps15 | intronic | . | . | . | 4qC7 | rs28151200 | 1 | 0 |
| chr4 | 109374395 | C | T | 228 | Eps15 | intronic | . | . | . | 4qC7 | rs28151193 | 1 | 0 |
| chr4 | 109374421 | T | C | 228 | Eps15 | intronic | . | . | . | 4qC7 | rs28151192 | 1 | 0 |
| chr4 | 109379756 | G | A | 167 | Eps15 | intronic | . | . | . | 4qC7 | rs6187322 | 1 | 0 |
| chr4 | 109383040 | T | A | 222 | Eps15 | intronic | . | . | . | 4qC7 | . | 0 | 1 |
| chr4 | 109426222 | A | T | 228 | Ttc39a | intronic | . | . | . | 4qC7 | . | 1 | 0 |
| chr4 | 109430888 | A | G | 228 | Ttc39a | exonic | synonymous SNV | Ttc39a:NM_001145948:exon9:c.A690G:p.A230A,Ttc39a:NM_153392:exon9:c.A684G:p.A228A | . | 4qC7 | rs28132057 | 1 | 0 |
| chr4 | 109431024 | A | G | 228 | Ttc39a | intronic | . | . | . | 4qC7 | rs28132054 | 1 | 0 |
| chr4 | 109431166 | G | A | 222 | Ttc39a | intronic | . | . | . | 4qC7 | . | 1 | 0 |
| chr4 | 109431279 | A | G | 228 | Ttc39a | intronic | . | . | . | 4qC7 | rs28132050 | 1 | 0 |
| chr4 | 109440671 | A | T | 228 | Ttc39a | intronic | . | . | . | 4qC7 | rs28131998 | 1 | 0 |
| chr4 | 109476415 | A | G | 160 | Rnf11 | UTR5 | . | . | 687 | 4qC7 | rs233107496 | 1 | 0 |
| chr4 | 109720893 | G | A | 228 | Faf1 | intronic | . | . | . | 4qC7 | rs47146596 | 1 | 0 |
| chr4 | 109727359 | A | C | 228 | Faf1 | intronic | . | . | . | 4qC7 | rs28164920 | 1 | 0 |
| chr4 | 109743040 | A | C | 228 | Faf1 | intronic | . | . | . | 4qC7 | rs28164899 | 1 | 0 |
| chr4 | 109757574 | C | T | 228 | Faf1 | intronic | . | . | . | 4qC7 | rs28164867 | 1 | 0 |
| chr4 | 109830048 | C | T | 106 | Faf1 | intronic | . | . | . | 4qC7 | . | 0 | 1 |
| chr4 | 110211363 | C | T | 136 | Elavl4 | intronic | . | . | . | 4qC7 | rs237746553 | 1 | 0 |
| chr4 | 110213031 | C | T | 228 | Elavl4 | intronic | . | . | . | 4qC7 | . | 1 | 0 |
| chr4 | 110213264 | T | C | 228 | Elavl4 | exonic | synonymous SNV | Elavl4:NM_001038698:exon4:c.A372G:p.P124P,Elavl4:NM_001163397:exon4:c.A381G:p.P127P,Elavl4:NM | . | 4qC7 | rs27515260 | 1 | 0 |
| chr4 | 110213447 | T | C | 171 | Elavl4 | intronic | . | . | . | 4qC7 | rs27515259 | 1 | 0 |
| chr4 | 110251126 | A | G | 206 | Elavl4 | intronic | . | . | . | 4qC7 | rs27515042 | 1 | 0 |
| chr4 | 110290302 | C | T | 228 | Elavl4 | intronic | . | . | . | 4qC7 | . | 1 | 0 |
| chr4 | 110290497 | A | G | 167 | Elavl4 | intronic | . | . | . | 4qC7 | . | 1 | 0 |
| chr4 | 110351838 | T | G | 111 | Elavl4 | UTR5 | . | . | 666 | 4qC7 | rs231898638 | 1 | 0 |
| chr4 | 110351839 | A | C | 111 | Elavl4 | UTR5 | . | . | 666 | 4qC7 | rs249493244 | 1 | 0 |
| chr4 | 110513413 | T | C | 228 | Agbl4 | intronic | . | . | . | 4qC7 | rs239178468 | 1 | 0 |
| chr4 | 110513798 | C | G | 126 | Agbl4 | intronic | . | . | . | 4qC7 | . | 1 | 0 |
| chr4 | 111118626 | G | A | 109 | Agbl4 | intronic | . | . | . | 4qD1 | rs28271714 | 1 | 0 |
| chr4 | 111290156 | G | T | 228 | Agbl4 | intronic | . | . | . | 4qD1 | rs214867820 | 1 | 0 |
| chr4 | 111290850 | A | G | 228 | Agbl4 | intronic | . | . | . | 4qD1 | rs3656329 | 1 | 0 |
| chr4 | 111290858 | A | C | 228 | Agbl4 | intronic | . | . | . | 4qD1 | rs3656343 | 1 | 0 |
| chr4 | 111291011 | A | G | 228 | Agbl4 | intronic | . | . | . | 4qD1 | rs28253625 | 1 | 0 |
| chr4 | 111291112 | C | T | 228 | Agbl4 | intronic | . | . | . | 4qD1 | rs28253623 | 1 | 0 |
| chr4 | 111415249 | T | C | 228 | Bend5,Agbl4 | exonic | synonymous SNV | Bend5:NM_026279:exon1:c.T75C:p.S25S | 754 | 4qD1 | rs32405432 | 1 | 0 |
| chr4 | 111415361 | G | A | 193 | Agbl4,Bend5 | exonic | missense SNV | Bend5:NM_026279:exon1:c.G187A:p.A63T | 754 | 4qD1 | . | 1 | 0 |
| chr4 | 111448510 | C | G | 228 | Bend5,Agbl4 | exonic | synonymous SNV | Bend5:NM_026279:exon4:c.C750G:p.P250P | . | 4qD1 | . | 1 | 0 |
| chr4 | 111454016 | G | A | 228 | Agbl4,Bend5 | intronic | . | . | . | 4qD1 | rs28270411 | 1 | 0 |
| chr4 | 111459735 | T | C | 228 | Bend5,Agbl4 | intronic | . | . | . | 4qD1 | rs28270378 | 1 | 0 |
| chr4 | 111526406 | G | A | 150 | Agbl4 | intronic | . | . | . | 4qD1 | rs256994686 | 1 | 0 |
| chr4 | 111526534 | T | C | 228 | Agbl4 | intronic | . | . | . | 4qD1 | rs27536048 | 1 | 0 |
| chr4 | 111526651 | G | A | 228 | Agbl4 | exonic | synonymous SNV | Agbl4:NM_001048189:exon7:c.G702A:p.T234T,Agbl4:NM_001284190:exon7:c.G702A:p.T234T,Agbl4:NM_0 | . | 4qD1 | rs13477912 | 1 | 0 |
| chr4 | 111526801 | G | A | 170 | Agbl4 | intronic | . | . | . | 4qD1 | rs27536047 | 1 | 0 |
| chr4 | 111546993 | T | C | 109 | Agbl4 | intronic | . | . | . | 4qD1 | rs46519192 | 1 | 0 |
| chr4 | 111547135 | T | C | 228 | Agbl4 | intronic | . | . | . | 4qD1 | rs52084787 | 1 | 0 |
| chr4 | 111547241 | A | G | 228 | Agbl4 | exonic | synonymous SNV | Agbl4:NM_001048189:exon8:c.A783G:p.L261L,Agbl4:NM_001284190:exon8:c.A783G:p.L261L,Agbl4:NM_0 | . | 4qD1 | rs27535996 | 1 | 0 |
| chr4 | 111547432 | A | G | 162 | Agbl4 | intronic | . | . | . | 4qD1 | rs49743323 | 1 | 0 |
| chr4 | 111566705 | T | C | 228 | Agbl4 | exonic | synonymous SNV | Agbl4:NM_001048189:exon9:c.T927C:p.L309L,Agbl4:NM_001284190:exon9:c.T927C:p.L309L,Agbl4:NM_0 | . | 4qD1 | rs32264304 | 1 | 0 |
| chr4 | 111566708 | T | C | 228 | Agbl4 | exonic | synonymous SNV | Agbl4:NM_001048189:exon9:c.T930C:p.I310I,Agbl4:NM_001284190:exon9:c.T930C:p.I310I,Agbl4:NM_0 | . | 4qD1 | rs31785688 | 1 | 0 |
| chr4 | 111614824 | G | T | 165 | Agbl4 | intronic | . | . | . | 4qD1 | rs232282410 | 1 | 0 |
| chr4 | 111617296 | A | G | 228 | Agbl4 | intronic | . | . | . | 4qD1 | rs27516509 | 1 | 0 |
| chr4 | 111747657 | C | A | 144 | Spata6 | intronic | . | . | . | 4qD1 | . | 1 | 0 |
| chr4 | 111747670 | G | A | 170 | Spata6 | intronic | . | . | . | 4qD1 | rs211855072 | 1 | 0 |
| chr4 | 111747909 | A | G | 128 | Spata6 | intronic | . | . | . | 4qD1 | rs32413964 | 1 | 0 |
| chr4 | 111767712 | A | G | 228 | Spata6 | intronic | . | . | . | 4qD1 | rs27485338 | 1 | 0 |
| chr4 | 111778862 | C | G | 95 | Spata6 | intronic | . | . | . | 4qD1 | rs27485288 | 1 | 0 |
| chr4 | 111779036 | T | C | 228 | Spata6 | exonic | missense SNV | Spata6:NM_026470:exon7:c.T523C:p.S175P | . | 4qD1 | rs27485285 | 1 | 0 |
| chr4 | 111780725 | A | C | 228 | Spata6 | intronic | . | . | . | 4qD1 | rs27485274 | 1 | 0 |
| chr4 | 111780920 | A | T | 228 | Spata6 | intronic | . | . | . | 4qD1 | rs27485271 | 1 | 0 |
| chr4 | 111784880 | C | A | 228 | Spata6 | intronic | . | . | . | 4qD1 | rs27535425 | 1 | 0 |
| chr4 | 111790884 | T | A | 228 | Spata6 | intronic | . | . | . | 4qD1 | rs46222328 | 1 | 0 |
| chr4 | 111790932 | C | G | 228 | Spata6 | intronic | . | . | . | 4qD1 | rs27535405 | 1 | 0 |
| chr4 | 111791111 | T | C | 140 | Spata6 | intronic | . | . | . | 4qD1 | rs27535400 | 1 | 0 |
| chr4 | 111791112 | G | C | 131 | Spata6 | intronic | . | . | . | 4qD1 | rs27535399 | 1 | 0 |
| chr4 | 111791189 | A | G | 210 | Spata6 | intronic | . | . | . | 4qD1 | rs32656330 | 1 | 0 |
| chr4 | 111791519 | A | C | 228 | Spata6 | intronic | . | . | . | 4qD1 | rs27535396 | 1 | 0 |
| chr4 | 111791612 | A | C | 228 | Spata6 | intronic | . | . | . | 4qD1 | rs27535393 | 1 | 0 |
| chr4 | 111806079 | C | T | 228 | Spata6 | intronic | . | . | . | 4qD1 | rs32114051 | 1 | 0 |
| chr4 | 111806083 | G | A | 228 | Spata6 | intronic | . | . | . | 4qD1 | rs32216460 | 1 | 0 |
| chr4 | 111806113 | G | T | 228 | Spata6 | intronic | . | . | . | 4qD1 | rs27515823 | 1 | 0 |
| chr4 | 111808100 | C | A | 228 | Spata6 | intronic | . | . | . | 4qD1 | . | 1 | 0 |
| chr4 | 111808373 | A | C | 228 | Spata6 | intronic | . | . | . | 4qD1 | rs32424110 | 1 | 0 |
| chr4 | 111808502 | G | A | 228 | Spata6 | intronic | . | . | . | 4qD1 | . | 1 | 0 |
| chr4 | 111877543 | C | A | 228 | Slc5a9 | exonic | missense SNV | Slc5a9:NM_145551:exon15:c.G2053T:p.A685S | . | 4qD1 | rs27500817 | 1 | 0 |
| chr4 | 111928803 | A | T | 228 | Skint8 | intronic | . | . | . | 4qD1 | rs50609634 | 1 | 0 |
| chr4 | 111928957 | T | A | 126 | Skint8 | intronic | . | . | . | 4qD1 | rs52395684 | 1 | 0 |
| chr4 | 111936702 | C | G | 175 | Skint8 | intronic | . | . | . | 4qD1 | rs46440119 | 1 | 0 |
| chr4 | 111936873 | C | T | 228 | Skint8 | exonic | synonymous SNV | Skint8:NM_001100466:exon4:c.C459T:p.A153A | . | 4qD1 | rs46805119 | 1 | 0 |
| chr4 | 111937026 | C | T | 228 | Skint8 | exonic | synonymous SNV | Skint8:NM_001100466:exon4:c.C612T:p.T204T | . | 4qD1 | rs49294192 | 1 | 0 |
| chr4 | 111937138 | T | G | 228 | Skint8 | intronic | . | . | . | 4qD1 | rs50013326 | 1 | 0 |
| chr4 | 111937165 | G | C | 228 | Skint8 | intronic | . | . | . | 4qD1 | rs46640808 | 1 | 0 |
| chr4 | 111939360 | A | T | 140 | Skint8 | intronic | . | . | . | 4qD1 | rs27500591 | 1 | 0 |
| chr4 | 111977554 | T | C | 228 | Skint7 | intronic | . | . | . | 4qD1 | rs46199074 | 1 | 0 |
| chr4 | 111977571 | G | T | 205 | Skint7 | intronic | . | . | . | 4qD1 | rs49723835 | 1 | 0 |
| chr4 | 111980416 | C | T | 228 | Skint7 | exonic | synonymous SNV | Skint7:NM_001142775:exon3:c.C390T:p.F130F,Skint7:NM_177818:exon3:c.C390T:p.F130F | . | 4qD1 | rs27484820 | 1 | 0 |
| chr4 | 111980424 | C | T | 228 | Skint7 | exonic | missense SNV | Skint7:NM_001142775:exon3:c.C398T:p.A133V,Skint7:NM_177818:exon3:c.C398T:p.A133V | . | 4qD1 | rs32943071 | 1 | 0 |
| chr4 | 111980522 | C | T | 203 | Skint7 | intronic | . | . | . | 4qD1 | rs27484819 | 1 | 0 |
| chr4 | 111981921 | C | T | 228 | Skint7 | intronic | . | . | . | 4qD1 | . | 1 | 0 |
| chr4 | 111982146 | T | C | 228 | Skint7 | exonic | synonymous SNV | Skint7:NM_001142775:exon4:c.T636C:p.H212H,Skint7:NM_177818:exon4:c.T636C:p.H212H | . | 4qD1 | rs27484815 | 1 | 0 |
| chr4 | 111983869 | G | A | 94 | Skint7 | intronic | . | . | . | 4qD1 | rs27484806 | 1 | 0 |
| chr4 | 111983950 | T | C | 153 | Skint7 | intronic | . | . | . | 4qD1 | rs27484805 | 1 | 0 |
| chr4 | 111987985 | G | A | 228 | Skint7 | exonic | synonymous SNV | Skint7:NM_001142775:exon7:c.G993A:p.L331L | . | 4qD1 | . | 1 | 0 |
| chr4 | 111987986 | T | A | 228 | Skint7 | exonic | missense SNV | Skint7:NM_001142775:exon7:c.T994A:p.F332I | . | 4qD1 | . | 1 | 0 |
| chr4 | 111988047 | T | C | 228 | Skint7 | exonic | missense SNV | Skint7:NM_001142775:exon7:c.T1055C:p.F352S | . | 4qD1 | . | 1 | 0 |
| chr4 | 112019103 | C | A | 228 | Skint1 | exonic | missense SNV | Skint1:NM_001102662:exon3:c.C220A:p.H74N | . | 4qD1 | . | 1 | 0 |
| chr4 | 112019372 | A | C | 228 | Skint1 | intronic | . | . | . | 4qD1 | . | 1 | 0 |
| chr4 | 112021294 | C | T | 228 | Skint1 | splicing | . | . | . | 4qD1 | rs27484693 | 1 | 0 |
| chr4 | 112028688 | T | C | 141 | Skint1 | intronic | . | . | . | 4qD1 | . | 0 | 1 |
| chr4 | 112028809 | T | A | 228 | Skint1 | exonic | missense SNV | Skint1:NM_001102662:exon7:c.T932A:p.V311D | . | 4qD1 | rs238376700 | 1 | 0 |
| chr4 | 112028962 | A | G | 228 | Skint1 | intronic | . | . | . | 4qD1 | rs46922944 | 1 | 0 |
| chr4 | 112028963 | C | A | 228 | Skint1 | intronic | . | . | . | 4qD1 | rs46574416 | 1 | 0 |
| chr4 | 112029116 | A | T | 228 | Skint1 | intronic | . | . | . | 4qD1 | rs32910266 | 1 | 0 |

| | | | | | | | | | | | | | |
|---|---|---|---|---|---|---|---|---|---|---|---|---|---|
| chr4 | 112029157 | G | C | 200 | Skint1 | intronic | . | . | . | 4qD1 | rs32089114 | 1 | 0 |
| chr4 | 112081477 | G | T | 165 | Skint4 | UTR5 | . | . | . | 4qD1 | rs27515432 | 1 | 0 |
| chr4 | 112081679 | T | C | 228 | Skint4 | UTR5 | . | . | . | 4qD1 | rs27515431 | 1 | 0 |
| chr4 | 112081778 | G | A | 228 | Skint4 | intronic | . | . | . | 4qD1 | rs27515430 | 1 | 0 |
| chr4 | 112081826 | C | G | 228 | Skint4 | intronic | . | . | . | 4qD1 | . | 1 | 0 |
| chr4 | 112081840 | T | G | 93 | Skint4 | intronic | . | . | . | 4qD1 | . | 0 | 1 |
| chr4 | 112086921 | T | G | 228 | Skint4 | intronic | . | . | . | 4qD1 | . | 1 | 0 |
| chr4 | 112086969 | G | A | 228 | Skint4 | intronic | . | . | . | 4qD1 | . | 1 | 0 |
| chr4 | 112087119 | G | A | 228 | Skint4 | intronic | . | . | . | 4qD1 | rs27515425 | 1 | 0 |
| chr4 | 112087181 | C | A | 173 | Skint4 | intronic | . | . | . | 4qD1 | rs27515424 | 1 | 0 |
| chr4 | 112117837 | G | A | 187 | Skint4 | intronic | . | . | . | 4qD1 | rs27515414 | 1 | 0 |
| chr4 | 112117904 | C | T | 228 | Skint4 | intronic | . | . | . | 4qD1 | rs27515413 | 1 | 0 |
| chr4 | 112118007 | A | G | 228 | Skint4 | exonic | missense SNV | Skint4:NM_178786:exon3:c.A163G:p.N55D | . | 4qD1 | . | 1 | 0 |
| chr4 | 112118195 | T | C | 228 | Skint4 | exonic | synonymous SNV | Skint4:NM_178786:exon3:c.T351C:p.I117I | . | 4qD1 | rs27515412 | 1 | 0 |
| chr4 | 112118327 | T | A | 228 | Skint4 | intronic | . | . | . | 4qD1 | rs27515411 | 1 | 0 |
| chr4 | 112118355 | A | G | 228 | Skint4 | intronic | . | . | . | 4qD1 | . | 1 | 0 |
| chr4 | 112120066 | T | C | 228 | Skint4 | exonic | missense SNV | Skint4:NM_178786:exon4:c.T676C:p.F226L | . | 4qD1 | rs27515405 | 1 | 0 |
| chr4 | 112120186 | T | C | 201 | Skint4 | intronic | . | . | . | 4qD1 | rs27515404 | 1 | 0 |
| chr4 | 112124613 | T | C | 228 | Skint4 | exonic | missense SNV | Skint4:NM_178786:exon5:c.T778C:p.S260P | . | 4qD1 | rs27515381 | 1 | 0 |
| chr4 | 112124704 | G | A | 228 | Skint4 | intronic | . | . | . | 4qD1 | . | 1 | 0 |
| chr4 | 112125338 | T | C | 148 | Skint4 | intronic | . | . | . | 4qD1 | . | 1 | 0 |
| chr4 | 112125350 | G | C | 129 | Skint4 | intronic | . | . | . | 4qD1 | rs27515377 | 1 | 0 |
| chr4 | 112135845 | G | T | 116 | Skint4 | intronic | . | . | . | 4qD1 | . | 1 | 0 |
| chr4 | 112135855 | G | A | 107 | Skint4 | intronic | . | . | . | 4qD1 | . | 1 | 0 |
| chr4 | 112136018 | C | T | 228 | Skint4 | exonic | missense SNV | Skint4:NM_178786:exon7:c.C937T:p.P313S | . | 4qD1 | rs27515353 | 1 | 0 |
| chr4 | 112136069 | G | A | 208 | Skint4 | exonic | missense SNV | Skint4:NM_178786:exon7:c.G988A:p.E330K | . | 4qD1 | . | 1 | 0 |
| chr4 | 112235557 | C | T | 119 | Skint3 | intronic | . | . | . | 4qD1 | . | 1 | 0 |
| chr4 | 112253907 | C | T | 228 | Skint3 | exonic | synonymous SNV | Skint3:NM_001102474:exon3:c.C228T:p.Y76Y,Skint3:NM_177578:exon3:c.C228T:p.Y76Y | . | 4qD1 | . | 1 | 0 |
| chr4 | 112254064 | A | G | 228 | Skint3 | exonic | missense SNV | Skint3:NM_001102474:exon3:c.A385G:p.I129V,Skint3:NM_177578:exon3:c.A385G:p.I129V | . | 4qD1 | rs27515324 | 1 | 0 |
| chr4 | 112255425 | T | C | 93 | Skint3 | intronic | . | . | . | 4qD1 | . | 1 | 0 |
| chr4 | 112255571 | G | C | 224 | Skint3 | intronic | . | . | . | 4qD1 | rs27515319 | 1 | 0 |
| chr4 | 112255599 | T | G | 228 | Skint3 | intronic | . | . | . | 4qD1 | . | 1 | 0 |
| chr4 | 112255813 | G | A | 228 | Skint3 | exonic | missense SNV | Skint3:NM_001102474:exon4:c.G619A:p.A207T,Skint3:NM_177578:exon4:c.G619A:p.A207T | . | 4qD1 | rs27515318 | 1 | 0 |
| chr4 | 112255947 | G | T | 228 | Skint3 | intronic | . | . | . | 4qD1 | rs27515317 | 1 | 0 |
| chr4 | 112255995 | G | T | 228 | Skint3 | intronic | . | . | . | 4qD1 | . | 1 | 0 |
| chr4 | 112258852 | A | G | 203 | Skint3 | intronic | . | . | . | 4qD1 | . | 1 | 0 |
| chr4 | 112258950 | A | C | 228 | Skint3 | exonic | missense SNV | Skint3:NM_001102474:exon5:c.A776C:p.Y259S | . | 4qD1 | rs27515300 | 1 | 0 |
| chr4 | 112269937 | A | T | 228 | Skint3 | intronic | . | . | . | 4qD1 | rs27515287 | 1 | 0 |
| chr4 | 112277447 | G | A | 178 | Skint3 | intronic | . | . | . | 4qD1 | rs27515270 | 1 | 0 |
| chr4 | 112277591 | A | G | 228 | Skint3 | exonic | synonymous SNV | Skint3:NM_177578:exon5:c.A744G:p.L248L,Skint3:NM_001102474:exon7:c.A960G:p.L320L | . | 4qD1 | rs27501767 | 1 | 0 |
| chr4 | 112277665 | A | T | 228 | Skint3 | exonic | missense SNV | Skint3:NM_177578:exon5:c.A818T:p.Y273F,Skint3:NM_001102474:exon7:c.A1034T:p.Y345F | . | 4qD1 | rs27501765 | 1 | 0 |
| chr4 | 112290214 | G | A | 203 | Skint3 | intronic | . | . | . | 4qD1 | . | 1 | 0 |
| chr4 | 112290365 | A | G | 209 | Skint3 | exonic | synonymous SNV | Skint3:NM_177578:exon7:c.A1068G:p.L356L,Skint3:NM_001102474:exon9:c.A1284G:p.L428L | . | 4qD1 | . | 1 | 0 |
| chr4 | 112290405 | G | A | 157 | Skint3 | intronic | . | . | . | 4qD1 | . | 1 | 0 |
| chr4 | 112386281 | T | G | 228 | Skint9 | UTR3 | . | . | . | 4qD1 | . | 1 | 0 |
| chr4 | 112386373 | T | G | 228 | Skint9 | exonic | missense SNV | Skint9:NM_177864:exon7:c.A896C:p.K299T | . | 4qD1 | . | 1 | 0 |
| chr4 | 112388992 | G | T | 139 | Skint9 | intronic | . | . | . | 4qD1 | . | 1 | 0 |
| chr4 | 112389099 | T | A | 228 | Skint9 | intronic | . | . | . | 4qD1 | . | 1 | 0 |
| chr4 | 112389115 | A | T | 228 | Skint9 | intronic | . | . | . | 4qD1 | . | 1 | 0 |
| chr4 | 112389157 | A | T | 228 | Skint9 | exonic | missense SNV | Skint9:NM_177864:exon6:c.T757A:p.C253S | . | 4qD1 | . | 1 | 0 |
| chr4 | 112389389 | G | T | 228 | Skint9 | intronic | . | . | . | 4qD1 | . | 1 | 0 |
| chr4 | 112389437 | A | C | 171 | Skint9 | intronic | . | . | . | 4qD1 | . | 1 | 0 |
| chr4 | 112390818 | C | T | 208 | Skint9 | intronic | . | . | . | 4qD1 | . | 1 | 0 |
| chr4 | 112390963 | A | T | 228 | Skint9 | exonic | missense SNV | Skint9:NM_177864:exon5:c.T583A:p.L195I | . | 4qD1 | . | 1 | 0 |
| chr4 | 112390987 | A | T | 228 | Skint9 | exonic | missense SNV | Skint9:NM_177864:exon5:c.T559A:p.L187M | . | 4qD1 | . | 1 | 0 |
| chr4 | 112391814 | G | A | 228 | Skint9 | exonic | missense SNV | Skint9:NM_177864:exon4:c.C416T:p.S139L | . | 4qD1 | . | 1 | 0 |
| chr4 | 112391897 | C | T | 228 | Skint9 | intronic | . | . | . | 4qD1 | . | 1 | 0 |
| chr4 | 112392019 | A | G | 162 | Skint9 | intronic | . | . | . | 4qD1 | . | 1 | 0 |
| chr4 | 112392089 | T | G | 118 | Skint9 | intronic | . | . | . | 4qD1 | . | 1 | 0 |
| chr4 | 112402604 | C | A | 104 | Skint9 | intronic | . | . | . | 4qD1 | . | 1 | 0 |
| chr4 | 112402617 | A | G | 92 | Skint9 | intronic | . | . | . | 4qD1 | . | 1 | 0 |
| chr4 | 112413691 | C | A | 157 | Skint9 | intronic | . | . | . | 4qD1 | rs27501702 | 1 | 0 |
| chr4 | 112414162 | T | C | 228 | Skint9 | intronic | . | . | . | 4qD1 | . | 1 | 0 |
| chr4 | 112414191 | A | T | 228 | Skint9 | intronic | . | . | . | 4qD1 | . | 1 | 0 |
| chr4 | 112414227 | A | G | 228 | Skint9 | intronic | . | . | . | 4qD1 | rs27501699 | 1 | 0 |
| chr4 | 112414343 | G | A | 101 | Skint9 | intronic | . | . | . | 4qD1 | . | 1 | 0 |
| chr4 | 112419038 | A | G | 228 | Skint9 | intronic | . | . | . | 4qD1 | . | 1 | 0 |
| chr4 | 112419131 | T | G | 228 | Skint9 | exonic | synonymous SNV | Skint9:NM_177864:exon2:c.A42C:p.S14S | . | 4qD1 | . | 1 | 0 |
| chr4 | 112419226 | A | C | 228 | Skint9 | intronic | . | . | . | 4qD1 | . | 1 | 0 |
| chr4 | 112419274 | A | C | 170 | Skint9 | intronic | . | . | . | 4qD1 | . | 1 | 0 |
| chr4 | 112433741 | A | T | 148 | Skint9 | intronic | . | . | . | 4qD1 | . | 1 | 0 |
| chr4 | 112434078 | A | T | 228 | . | upstream | . | . | . | 4qD1 | . | 1 | 0 |
| chr4 | 112434134 | A | G | 143 | . | upstream | . | . | . | 4qD1 | . | 1 | 0 |
| chr4 | 112537271 | T | C | 228 | . | intergenic | . | . | . | 4qD1 | rs27501610 | 1 | 0 |
| chr4 | 112537424 | C | T | 228 | . | intergenic | . | . | . | 4qD1 | rs27501609 | 1 | 0 |
| chr4 | 112537450 | T | C | 228 | . | intergenic | . | . | . | 4qD1 | rs27501608 | 1 | 0 |
| chr4 | 112537804 | T | G | 208 | . | intergenic | . | . | . | 4qD1 | . | 1 | 0 |
| chr4 | 112559697 | G | A | 165 | . | intergenic | . | . | . | 4qD1 | rs27501589 | 1 | 0 |
| chr4 | 112559773 | G | A | 228 | . | intergenic | . | . | . | 4qD1 | rs27501587 | 1 | 0 |
| chr4 | 112566864 | G | A | 153 | . | intergenic | . | . | . | 4qD1 | rs27501561 | 1 | 0 |
| chr4 | 112575614 | T | G | 131 | . | intergenic | . | . | . | 4qD1 | . | 1 | 0 |
| chr4 | 112575681 | A | G | 168 | . | intergenic | . | . | . | 4qD1 | . | 1 | 0 |
| chr4 | 112616383 | T | A | 173 | Skint2 | intronic | . | . | . | 4qD1 | rs27501472 | 1 | 0 |
| chr4 | 112616473 | A | T | 228 | Skint2 | intronic | . | . | . | 4qD1 | rs27501468 | 1 | 0 |
| chr4 | 112616570 | G | C | 228 | Skint2 | exonic | missense SNV | Skint2:NM_001285963:exon2:c.G48C:p.Q16H,Skint2:NM_001285965:exon2:c.G48C:p.Q16H | . | 4qD1 | rs27484067 | 1 | 0 |
| chr4 | 112616673 | T | A | 172 | Skint2 | intronic | . | . | . | 4qD1 | . | 1 | 0 |
| chr4 | 112616741 | A | G | 34.6811 | Skint2 | intronic | . | . | . | 4qD1 | rs253935622 | 1 | 0 |
| chr4 | 112623978 | G | C | 228 | Skint2 | intronic | . | . | . | 4qD1 | . | 1 | 0 |
| chr4 | 112624223 | G | A | 228 | Skint2 | exonic | synonymous SNV | Skint2:NM_001285963:exon3:c.G282A:p.L94L,Skint2:NM_001285965:exon3:c.G282A:p.L94L | . | 4qD1 | . | 1 | 0 |
| chr4 | 112624373 | T | C | 228 | Skint2 | intronic | . | . | . | 4qD1 | rs27484040 | 1 | 0 |
| chr4 | 112624447 | A | G | 189 | Skint2 | intronic | . | . | . | 4qD1 | . | 1 | 0 |
| chr4 | 112625783 | G | C | 228 | Skint2 | intronic | . | . | . | 4qD1 | rs259506203 | 1 | 0 |
| chr4 | 112625802 | G | T | 228 | Skint2 | intronic | . | . | . | 4qD1 | . | 1 | 0 |
| chr4 | 112625847 | C | T | 228 | Skint2 | exonic | missense SNV | Skint2:NM_001285963:exon4:c.C448T:p.P150S,Skint2:NM_001285965:exon4:c.C448T:p.P150S | . | 4qD1 | . | 1 | 0 |
| chr4 | 112625945 | A | T | 228 | Skint2 | exonic | synonymous SNV | Skint2:NM_001285963:exon4:c.A546T:p.P182P,Skint2:NM_001285965:exon4:c.A546T:p.P182P | . | 4qD1 | . | 1 | 0 |
| chr4 | 112626033 | A | G | 228 | Skint2 | exonic | missense SNV | Skint2:NM_001285963:exon4:c.A634G:p.I212V,Skint2:NM_001285965:exon4:c.A634G:p.I212V | . | 4qD1 | rs27484030 | 1 | 0 |
| chr4 | 112639715 | G | T | 228 | Skint2 | intronic | . | . | . | 4qD1 | rs49307412 | 1 | 0 |
| chr4 | 112639781 | A | C | 228 | Skint2 | intronic | . | . | . | 4qD1 | rs27484010 | 1 | 0 |
| chr4 | 112711632 | A | G | 228 | Skint10 | exonic | missense SNV | Skint10:NM_177668:exon7:c.T887C:p.F296S | . | 4qD1 | rs27483862 | 1 | 0 |
| chr4 | 112711728 | C | A | 228 | Skint10 | intronic | . | . | . | 4qD1 | rs51686802 | 1 | 0 |
| chr4 | 112715570 | C | G | 154 | Skint10 | intronic | . | . | . | 4qD1 | rs27483828 | 1 | 0 |
| chr4 | 112715608 | A | T | 165 | Skint10 | intronic | . | . | . | 4qD1 | rs27483826 | 1 | 0 |
| chr4 | 112715734 | A | G | 228 | Skint10 | intronic | . | . | . | 4qD1 | rs48784373 | 1 | 0 |
| chr4 | 112715821 | C | T | 228 | Skint10 | exonic | synonymous SNV | Skint10:NM_177668:exon6:c.G777A:p.V259V | . | 4qD1 | rs27483824 | 1 | 0 |
| chr4 | 112715924 | A | T | 228 | Skint10 | intronic | . | . | . | 4qD1 | rs27483823 | 1 | 0 |
| chr4 | 112715944 | A | T | 203 | Skint10 | intronic | . | . | . | 4qD1 | rs49227783 | 1 | 0 |
| chr4 | 112727746 | A | C | 175 | Skint10 | intronic | . | . | . | 4qD1 | rs27483787 | 1 | 0 |
| chr4 | 112728767 | T | C | 123 | Skint10 | intronic | . | . | . | 4qD1 | rs27483779 | 1 | 0 |
| chr4 | 112728841 | C | T | 174 | Skint10 | exonic | missense SNV | Skint10:NM_177668:exon4:c.G577A:p.E193K | . | 4qD1 | rs47606609 | 1 | 0 |
| chr4 | 112728844 | T | G | 166 | Skint10 | exonic | synonymous SNV | Skint10:NM_177668:exon4:c.A574C:p.R192R | . | 4qD1 | rs27483778 | 1 | 0 |
| chr4 | 112728926 | T | C | 228 | Skint10 | exonic | synonymous SNV | Skint10:NM_177668:exon4:c.A492G:p.T164T | . | 4qD1 | rs27483776 | 1 | 0 |
| chr4 | 112729023 | T | C | 228 | Skint10 | intronic | . | . | . | 4qD1 | rs27483772 | 1 | 0 |
| chr4 | 112746435 | A | T | 160 | Skint10 | intronic | . | . | . | 4qD1 | rs28300684 | 1 | 0 |
| chr4 | 112746472 | A | C | 176 | Skint10 | intronic | . | . | . | 4qD1 | rs32831874 | 1 | 0 |
| chr4 | 112746571 | C | T | 228 | Skint10 | exonic | missense SNV | Skint10:NM_177668:exon3:c.G418A:p.V140I | . | 4qD1 | rs13477916 | 1 | 0 |
| chr4 | 112746846 | G | A | 228 | Skint10 | intronic | . | . | . | 4qD1 | rs47922952 | 1 | 0 |
| chr4 | 112764922 | T | C | 162 | Skint10 | intronic | . | . | . | 4qD1 | rs32257176 | 1 | 0 |
| chr4 | 112764967 | C | T | 228 | Skint10 | intronic | . | . | . | 4qD1 | rs32879102 | 1 | 0 |
| chr4 | 112765080 | G | A | 228 | Skint10 | exonic | synonymous SNV | Skint10:NM_177668:exon2:c.C105T:p.F35F | . | 4qD1 | rs227747972 | 1 | 0 |
| chr4 | 112765106 | A | G | 228 | Skint10 | exonic | missense SNV | Skint10:NM_177668:exon2:c.T79C:p.S27P | . | 4qD1 | rs31946136 | 1 | 0 |
| chr4 | 112765193 | A | G | 194 | Skint10 | intronic | . | . | . | 4qD1 | rs32002325 | 1 | 0 |
| chr4 | 112774708 | C | G | 228 | Skint10 | intronic | . | . | . | 4qD1 | rs28283830 | 1 | 0 |
| chr4 | 112774783 | G | T | 228 | Skint10 | exonic | missense SNV | Skint10:NM_177668:exon1:c.C39A:p.D13E | . | 4qD1 | rs28283828 | 1 | 0 |
| chr4 | 112774958 | A | T | 156 | . | upstream | . | . | . | 4qD1 | rs28283826 | 1 | 0 |
| chr4 | 113940692 | G | A | 30.4073 | Skint5 | exonic | synonymous SNV | Skint5:NM_001167876:exon4:c.C651T:p.C217C,Skint5:NM_001167878:exon4:c.C651T:p.C217C | . | 4qD1 | rs50398711 | 0 | 1 |
| chr4 | 113940710 | A | G | 228 | Skint5 | exonic | synonymous SNV | Skint5:NM_001167876:exon4:c.T633C:p.S211S,Skint5:NM_001167878:exon4:c.T633C:p.S211S | . | 4qD1 | rs50678814 | 1 | 0 |
| chr4 | 114821821 | T | C | 130 | Gm12830 | ncRNA_exonic | . | . | 381 | 4qD1 | . | 0 | 1 |
| chr4 | 114841923 | A | G | 196 | Gm12830 | ncRNA_intronic | . | . | . | 4qD1 | rs51939005 | 1 | 0 |
| chr4 | 114841947 | T | C | 190 | Gm12830 | ncRNA_intronic | . | . | . | 4qD1 | rs48209171 | 1 | 0 |
| chr4 | 114907315 | G | A | 151 | Foxd2 | UTR3 | . | . | 2442 | 4qD1 | rs246109394 | 1 | 0 |
| chr4 | 114907433 | G | A | 226 | Foxd2 | exonic | synonymous SNV | Foxd2:NM_008593:exon1:c.C1389T:p.H463H | 2442 | 4qD1 | rs258840764 | 1 | 0 |
| chr4 | 114925516 | G | T | 105 | Foxe3 | exonic | synonymous SNV | Foxe3:NM_015758:exon1:c.C498A:p.P166P | 1069 | 4qD1 | rs229915209 | 1 | 0 |
| chr4 | 114925771 | G | C | 228 | Foxe3 | exonic | synonymous SNV | Foxe3:NM_015758:exon1:c.C243G:p.P81P | 1069 | 4qD1 | rs31952972 | 1 | 0 |
| chr4 | 114926027 | G | A | 228 | . | upstream | . | . | 1069 | 4qD1 | rs262380188 | 1 | 0 |
| chr4 | 114963035 | T | C | 228 | Cmpk1 | intronic | . | . | . | 4qD1 | rs27513548 | 1 | 0 |
| chr4 | 114963514 | A | C | 152 | Cmpk1 | intronic | . | . | . | 4qD1 | rs52407933 | 1 | 0 |
| chr4 | 114965012 | G | A | 228 | Cmpk1 | exonic | synonymous SNV | Cmpk1:NM_025647:exon3:c.C339T:p.A113A | . | 4qD1 | rs13475512 | 1 | 0 |
| chr4 | 114965059 | T | C | 228 | Cmpk1 | intronic | . | . | . | 4qD1 | rs27513546 | 1 | 0 |
| chr4 | 115006882 | A | G | 228 | Stil | intronic | . | . | . | 4qD1 | rs27499044 | 1 | 0 |
| chr4 | 115006989 | G | T | 228 | Stil | intronic | . | . | . | 4qD1 | rs27499043 | 1 | 0 |
| chr4 | 115007014 | G | A | 228 | Stil | intronic | . | . | . | 4qD1 | rs27499042 | 1 | 0 |
| chr4 | 115009360 | A | G | 228 | Stil | exonic | synonymous SNV | Stil:NM_001304555:exon6:c.A510G:p.L170L,Stil:NM_001304559:exon6:c.A510G:p.L170L,Stil:NM_0091 | . | 4qD1 | rs4224709 | 1 | 0 |
| chr4 | 115009542 | A | G | 228 | Stil | intronic | . | . | . | 4qD1 | rs27499033 | 1 | 0 |
| chr4 | 115011941 | G | C | 228 | Stil | intronic | . | . | . | 4qD1 | rs27499019 | 1 | 0 |
| chr4 | 115012014 | A | G | 173 | Stil | intronic | . | . | . | 4qD1 | rs32906242 | 1 | 0 |
| chr4 | 115015959 | A | T | 135 | Stil | intronic | . | . | . | 4qD1 | rs27499012 | 1 | 0 |
| chr4 | 115021488 | T | G | 228 | Stil | exonic | missense SNV | Stil:NM_001304551:exon10:c.T442G:p.L148V,Stil:NM_001304555:exon11:c.T1120G:p.L374V,Stil:NM_0 | . | 4qD1 | rs27498988 | 1 | 0 |
| chr4 | 115023595 | A | G | 228 | Stil | exonic | synonymous SNV | Stil:NM_001304551:exon11:c.A657G:p.L219L,Stil:NM_001304555:exon12:c.A1335G:p.L445L,Stil:NM_0 | . | 4qD1 | rs27498978 | 1 | 0 |
| chr4 | 115024317 | T | C | 228 | Stil | exonic | missense SNV | Stil:NM_001304551:exon11:c.T1379C:p.M460T,Stil:NM_001304555:exon12:c.T2057C:p.M686T,Stil:NM_ | . | 4qD1 | rs32334937 | 1 | 0 |
| chr4 | 115029929 | G | A | 228 | Stil | exonic | missense SNV | Stil:NM_001304551:exon12:c.G1540A:p.V514M,Stil:NM_001304559:exon12:c.G1282A:p.V428M,Stil:NM_ | . | 4qD1 | rs27498958 | 1 | 0 |
| chr4 | 115029954 | A | G | 228 | Stil | exonic | missense SNV | Stil:NM_001304551:exon12:c.A1565G:p.K522R,Stil:NM_001304559:exon12:c.A1307G:p.K436R,Stil:NM_ | . | 4qD1 | rs27498957 | 1 | 0 |
| chr4 | 115038319 | C | T | 228 | Stil | intronic | . | . | . | 4qD1 | rs255584381 | 1 | 0 |
| chr4 | 115038374 | T | A | 228 | Stil | exonic | synonymous SNV | Stil:NM_001304551:exon14:c.T1914A:p.P638P,Stil:NM_001304559:exon14:c.T1656A:p.P552P,Stil:NM_ | . | 4qD1 | rs27498881 | 1 | 0 |
| chr4 | 115038967 | G | A | 202 | Stil | intronic | . | . | . | 4qD1 | rs27498879 | 1 | 0 |
| chr4 | 115038978 | C | T | 205 | Stil | intronic | . | . | . | 4qD1 | rs252604828 | 1 | 0 |
| chr4 | 115039144 | G | A | 228 | Stil | exonic | missense SNV | Stil:NM_001304551:exon15:c.G2147A:p.R716H,Stil:NM_001304559:exon15:c.G1889A:p.R630H,Stil:NM_ | . | 4qD1 | rs27498876 | 1 | 0 |
| chr4 | 115041096 | T | C | 228 | Stil | intronic | . | . | . | 4qD1 | rs27498863 | 1 | 0 |
| chr4 | 115111639 | A | G | 60 | Cyp4x1 | exonic | synonymous SNV | Cyp4x1:NM_001003947:exon10:c.T1245C:p.P415P | . | 4qD1 | . | 0 | 1 |
| chr4 | 115456397 | T | A | 63 | Cyp4a30b | intronic | . | . | . | 4qD1 | . | 0 | 1 |
| chr4 | 116571012 | A | G | 221 | Gpbp1l1 | intronic | . | . | . | 4qD1 | . | 0 | 1 |
| chr4 | 116637660 | C | A | 228 | Akr1a1 | intronic | . | . | . | 4qD1 | . | 1 | 0 |
| chr4 | 116714057 | G | C | 221 | Ccdc163 | intronic | . | . | . | 4qD1 | rs27514019 | 0 | 1 |
| chr4 | 117111317 | G | A | 51 | Ptch2 | exonic | missense SNV | Ptch2:NM_001312903:exon18:c.G1922A:p.C641Y,Ptch2:NM_008958:exon18:c.G2888A:p.C963Y | . | 4qD1 | . | 0 | 1 |
| chr4 | 117490146 | G | T | 228 | Rnf220 | exonic | missense SNV | Rnf220:NM_025739:exon2:c.C68A:p.P23Q | . | 4qD1 | . | 1 | 0 |
| chr4 | 117890341 | C | T | 194 | Dph2 | exonic | missense SNV | Dph2:NM_026344:exon4:c.G845A:p.R282H | . | 4qD2.1 | . | 0 | 1 |
| chr4 | 118148680 | C | A | 228 | Kdm4a | intronic | . | . | . | 4qD2.1 | . | 1 | 0 |
| chr4 | 118235887 | C | A | 161 | Ptprf | intronic | . | . | . | 4qD2.1 | . | 0 | 1 |
| chr4 | 118486745 | C | A | 220 | Tie1 | intronic | . | . | . | 4qD2.1 | . | 1 | 0 |
| chr4 | 118734450 | T | G | 154 | . | upstream | . | . | . | 4qD2.1 | . | 0 | 1 |
| chr4 | 119178222 | G | A | 221 | Ermap | exonic | missense SNV | Ermap:NM_013848:exon13:c.C1568T:p.T523M | . | 4qD2.1 | . | 0 | 1 |
| chr4 | 119258933 | G | C | 228 | Cldn19 | exonic | missense SNV | Cldn19:NM_001038590:exon4:c.G599C:p.R200P,Cldn19:NM_153105:exon4:c.G599C:p.R200P | . | 4qD2.1 | . | 1 | 0 |
| chr4 | 119373594 | C | A | 194 | Ccdc30 | intronic | . | . | . | 4qD2.1 | . | 0 | 1 |
| chr4 | 119393601 | G | A | 204 | Ccdc30 | exonic | synonymous SNV | Ccdc30:NM_001270435:exon5:c.C445T:p.L149L,Ccdc30:NM_029286:exon5:c.C445T:p.L149L | . | 4qD2.1 | . | 0 | 1 |
| chr4 | 120462139 | C | G | 221 | Scmh1 | exonic | missense SNV | Scmh1:NM_001159630:exon5:c.C60G:p.I20M,Scmh1:NM_013883:exon5:c.C60G:p.I20M | . | 4qD2.1 | . | 0 | 1 |
| chr4 | 120549619 | A | G | 115 | Ctps | intronic | . | . | . | 4qD2.2 | . | 0 | 1 |
| chr4 | 120553038 | A | T | 182 | Ctps | intronic | . | . | . | 4qD2.2 | . | 0 | 1 |
| chr4 | 120667510 | C | T | 218 | Cited4 | UTR3 | . | . | . | 4qD2.2 | . | 0 | 1 |
| chr4 | 121053897 | C | A | 65 | Col9a2 | exonic | synonymous SNV | Col9a2:NM_007741:exon30:c.C1728A:p.G576G | . | 4qD2.2 | . | 0 | 1 |
| chr4 | 121095470 | G | C | 164 | Zmpste24 | intronic | . | . | . | 4qD2.2 | . | 0 | 1 |
| chr4 | 121159333 | C | T | 228 | Rlf | intronic | . | . | . | 4qD2.2 | . | 1 | 0 |
| chr4 | 122008218 | T | C | 175 | . | intergenic | . | . | . | 4qD2.2 | rs49617527 | 0 | 1 |
| chr4 | 123465550 | C | G | 222 | Macf1 | intronic | . | . | . | 4qD2.2 | . | 0 | 1 |
| chr4 | 125056754 | G | A | 209 | Dnali1 | intronic | . | . | . | 4qD2.2 | . | 1 | 0 |
| chr4 | 126076595 | A | G | 181 | Oscp1 | intronic | . | . | . | 4qD2.2 | rs32393297 | 1 | 0 |
| chr4 | 126083137 | C | T | 162 | Oscp1 | intronic | . | . | . | 4qD2.2 | rs27550233 | 1 | 0 |

| | | | | | | | | | | | | | |
|---|---|---|---|---|---|---|---|---|---|---|---|---|---|
| chr4 | 126088192 | T | C | 160 | Oscp1 | intronic | . | . | . | 4qD2.2 | rs27550197 | 1 | 0 |
| chr4 | 126128778 | G | A | 228 | Stk40 | intronic | . | . | . | 4qD2.2 | rs27528763 | 1 | 0 |
| chr4 | 126129439 | C | T | 104 | Stk40 | intronic | . | . | . | 4qD2.2 | rs32600373 | 1 | 0 |
| chr4 | 126129446 | T | C | 111 | Stk40 | intronic | . | . | . | 4qD2.2 | rs32664731 | 1 | 0 |
| chr4 | 126129724 | A | G | 228 | Stk40 | exonic | synonymous SNV | Stk40:NM_028800:exon6:c.A597G:p.K199K,Stk40:NM_001145827:exon8:c.A639G:p.K213K | . | 4qD2.2 | rs27528755 | 1 | 0 |
| chr4 | 126137032 | T | C | 228 | Stk40 | intronic | . | . | . | 4qD2.2 | rs31826684 | 1 | 0 |
| chr4 | 126137603 | A | G | 153 | Stk40 | intronic | . | . | . | 4qD2.2 | rs32753595 | 1 | 0 |
| chr4 | 126137958 | C | A | 206 | Stk40 | intronic | . | . | . | 4qD2.2 | rs245338529 | 1 | 0 |
| chr4 | 126137999 | G | T | 172 | Stk40 | intronic | . | . | . | 4qD2.2 | rs32322117 | 1 | 0 |
| chr4 | 126138714 | T | C | 228 | Stk40 | intronic | . | . | . | 4qD2.2 | rs50372697 | 1 | 0 |
| chr4 | 126139099 | C | T | 228 | Stk40 | UTR3 | . | . | . | 4qD2.2 | rs233739763 | 1 | 0 |
| chr4 | 126139206 | G | A | 132 | Stk40 | UTR3 | . | . | . | 4qD2.2 | rs51764952 | 1 | 0 |
| chr4 | 126149498 | T | C | 228 | Eva1b | exonic | synonymous SNV | Eva1b:NM_172145:exon3:c.T330C:p.S110S | 1183 | 4qD2.2 | rs236734518 | 1 | 0 |
| chr4 | 126152461 | C | T | 228 | Sh3d21 | intronic | . | . | . | 4qD2.2 | rs27528743 | 1 | 0 |
| chr4 | 126161613 | A | G | 228 | Sh3d21 | exonic | synonymous SNV | Sh3d21:NM_025856:exon4:c.T201C:p.P67P,Sh3d21:NM_001162533:exon8:c.T549C:p.P183P | . | 4qD2.2 | rs262151303 | 1 | 0 |
| chr4 | 126161813 | A | G | 228 | Sh3d21 | intronic | . | . | . | 4qD2.2 | rs257352400 | 1 | 0 |
| chr4 | 126162004 | T | C | 228 | Sh3d21 | exonic | missense SNV | Sh3d21:NM_025856:exon2:c.A108G:p.I36M,Sh3d21:NM_001162533:exon6:c.A456G:p.I152M | . | 4qD2.2 | rs27528730 | 1 | 0 |
| chr4 | 126166867 | C | G | 228 | Thrap3 | intronic | . | . | . | 4qD2.2 | . | 1 | 0 |
| chr4 | 126167260 | T | C | 228 | Thrap3 | intronic | . | . | . | 4qD2.2 | rs27528702 | 1 | 0 |
| chr4 | 126167346 | T | A | 228 | Thrap3 | intronic | . | . | . | 4qD2.2 | rs27528701 | 1 | 0 |
| chr4 | 126171676 | A | G | 228 | Thrap3 | intronic | . | . | . | 4qD2.2 | rs27528687 | 1 | 0 |
| chr4 | 126175237 | A | G | 228 | Thrap3 | intronic | . | . | . | 4qD2.2 | rs27528681 | 1 | 0 |
| chr4 | 126176126 | T | C | 228 | Thrap3 | intronic | . | . | . | 4qD2.2 | rs27528675 | 1 | 0 |
| chr4 | 126178083 | A | G | 150 | Thrap3 | exonic | synonymous SNV | Thrap3:NM_146153:exon4:c.T1515C:p.S505S | . | 4qD2.2 | rs27528668 | 1 | 0 |
| chr4 | 126233142 | T | C | 228 | Map7d1 | intronic | . | . | . | 4qD2.2 | rs32989852 | 1 | 0 |
| chr4 | 126234196 | G | A | 96 | Map7d1 | exonic | missense SNV | Map7d1:NM_001145970:exon11:c.C1729T:p.R577W,Map7d1:NM_144941:exon11:c.C1945T:p.R649W | 547 | 4qD2.2 | . | 1 | 0 |
| chr4 | 126234521 | A | G | 228 | Map7d1 | splicing | . | . | . | 4qD2.2 | rs32055887 | 1 | 0 |
| chr4 | 126237311 | A | G | 228 | Map7d1 | intronic | . | . | . | 4qD2.2 | rs32856041 | 1 | 0 |
| chr4 | 126309551 | G | A | 179 | Col8a2 | intronic | . | . | . | 4qD2.2 | rs27578648 | 1 | 0 |
| chr4 | 126309583 | T | C | 207 | Col8a2 | intronic | . | . | . | 4qD2.2 | rs27578647 | 1 | 0 |
| chr4 | 126309823 | T | G | 228 | Col8a2 | intronic | . | . | . | 4qD2.2 | rs251527973 | 1 | 0 |
| chr4 | 126309843 | A | C | 228 | Col8a2 | intronic | . | . | . | 4qD2.2 | rs265491713 | 1 | 0 |
| chr4 | 126310609 | G | A | 228 | Col8a2 | exonic | synonymous SNV | Col8a2:NM_199473:exon4:c.G411A:p.G137G | . | 4qD2.2 | rs254765630 | 1 | 0 |
| chr4 | 126311554 | T | C | 154 | Col8a2 | exonic | synonymous SNV | Col8a2:NM_199473:exon4:c.T1356C:p.P452P | . | 4qD2.2 | rs233079723 | 1 | 0 |
| chr4 | 126311950 | A | G | 228 | Col8a2 | exonic | synonymous SNV | Col8a2:NM_199473:exon4:c.A1752G:p.S584S | . | 4qD2.2 | rs253849328 | 1 | 0 |
| chr4 | 126312226 | T | C | 228 | Col8a2 | exonic | synonymous SNV | Col8a2:NM_199473:exon4:c.T2028C:p.D676D | . | 4qD2.2 | rs27578646 | 1 | 0 |
| chr4 | 126316464 | C | T | 170 | Adprhl2 | UTR3 | . | . | . | 4qD2.2 | rs228577923 | 1 | 0 |
| chr4 | 126316554 | C | G | 206 | Adprhl2 | UTR3 | . | . | . | 4qD2.2 | rs27578632 | 1 | 0 |
| chr4 | 126316986 | A | C | 211 | Adprhl2 | intronic | . | . | . | 4qD2.2 | rs27578630 | 1 | 0 |
| chr4 | 126317212 | A | C | 208 | Adprhl2 | intronic | . | . | . | 4qD2.2 | rs245856845 | 1 | 0 |
| chr4 | 126317229 | T | C | 217 | Adprhl2 | intronic | . | . | . | 4qD2.2 | rs27578628 | 1 | 0 |
| chr4 | 126317466 | T | C | 103 | Adprhl2 | intronic | . | . | . | 4qD2.2 | rs242851201 | 1 | 0 |
| chr4 | 126317473 | G | A | 103 | Adprhl2 | intronic | . | . | . | 4qD2.2 | rs256064693 | 1 | 0 |
| chr4 | 126317890 | C | T | 228 | Adprhl2 | exonic | synonymous SNV | Adprhl2:NM_133883:exon4:c.G549A:p.S183S | . | 4qD2.2 | rs27578622 | 1 | 0 |
| chr4 | 126318277 | A | G | 228 | Adprhl2 | intronic | . | . | . | 4qD2.2 | rs229312251 | 1 | 0 |
| chr4 | 126318319 | G | A | 228 | Adprhl2 | intronic | . | . | . | 4qD2.2 | rs27578621 | 1 | 0 |
| chr4 | 126318449 | C | T | 228 | Adprhl2 | exonic | synonymous SNV | Adprhl2:NM_133883:exon2:c.G276A:p.V92V | . | 4qD2.2 | rs231788597 | 1 | 0 |
| chr4 | 126322314 | G | T | 228 | Tekt2 | exonic | synonymous SNV | Tekt2:NM_011902:exon10:c.C1185A:p.R395R | . | 4qD2.2 | rs27578609 | 1 | 0 |
| chr4 | 126322616 | T | C | 228 | Tekt2 | exonic | synonymous SNV | Tekt2:NM_011902:exon9:c.A1035G:p.L345L | . | 4qD2.2 | rs27578607 | 1 | 0 |
| chr4 | 126322757 | A | C | 228 | Tekt2 | intronic | . | . | . | 4qD2.2 | rs27578606 | 1 | 0 |
| chr4 | 126323020 | A | G | 228 | Tekt2 | intronic | . | . | . | 4qD2.2 | rs32637606 | 1 | 0 |
| chr4 | 126323656 | G | A | 228 | Tekt2 | exonic | synonymous SNV | Tekt2:NM_011902:exon5:c.C615T:p.P205P | . | 4qD2.2 | rs235444556 | 1 | 0 |
| chr4 | 126324624 | A | G | 228 | Tekt2 | exonic | synonymous SNV | Tekt2:NM_011902:exon3:c.T252C:p.D84D | . | 4qD2.2 | rs27578603 | 1 | 0 |
| chr4 | 126325056 | A | G | 228 | Tekt2 | intronic | . | . | . | 4qD2.2 | rs32991180 | 1 | 0 |
| chr4 | 126345320 | G | A | 173 | Ago3 | UTR3 | . | . | . | 4qD2.2 | rs27564111 | 1 | 0 |
| chr4 | 126346835 | G | A | 228 | Ago3 | intronic | . | . | . | 4qD2.2 | rs27564110 | 1 | 0 |
| chr4 | 126347625 | C | T | 184 | Ago3 | intronic | . | . | . | 4qD2.2 | rs237367759 | 1 | 0 |
| chr4 | 126349997 | A | G | 126 | Ago3 | intronic | . | . | . | 4qD2.2 | rs27564105 | 1 | 0 |
| chr4 | 126350195 | C | T | 228 | Ago3 | exonic | synonymous SNV | Ago3:NM_153402:exon16:c.G2163A:p.R721R | . | 4qD2.2 | rs216000016 | 1 | 0 |
| chr4 | 126350216 | T | C | 228 | Ago3 | exonic | synonymous SNV | Ago3:NM_153402:exon16:c.A2142G:p.T714T | . | 4qD2.2 | rs27564104 | 1 | 0 |
| chr4 | 126350407 | T | C | 228 | Ago3 | intronic | . | . | . | 4qD2.2 | rs27564102 | 1 | 0 |
| chr4 | 126353525 | T | C | 188 | Ago3 | intronic | . | . | . | 4qD2.2 | rs27564099 | 1 | 0 |
| chr4 | 126353899 | T | C | 177 | Ago3 | intronic | . | . | . | 4qD2.2 | rs27564097 | 1 | 0 |
| chr4 | 126363479 | T | C | 162 | Ago3 | intronic | . | . | . | 4qD2.2 | rs27564089 | 1 | 0 |
| chr4 | 126363653 | T | C | 228 | Ago3 | exonic | synonymous SNV | Ago3:NM_153402:exon12:c.A1584G:p.P528P | . | 4qD2.2 | rs244163322 | 1 | 0 |
| chr4 | 126363656 | T | C | 228 | Ago3 | exonic | synonymous SNV | Ago3:NM_153402:exon12:c.A1581G:p.T527T | . | 4qD2.2 | rs261971275 | 1 | 0 |
| chr4 | 126367749 | T | C | 228 | Ago3 | exonic | synonymous SNV | Ago3:NM_153402:exon11:c.A1305G:p.V435V | . | 4qD2.2 | rs27564084 | 1 | 0 |
| chr4 | 126368508 | T | A | 228 | Ago3 | exonic | synonymous SNV | Ago3:NM_153402:exon10:c.A1248T:p.A416A | . | 4qD2.2 | rs27564083 | 1 | 0 |
| chr4 | 126368737 | C | T | 169 | Ago3 | intronic | . | . | . | 4qD2.2 | rs214324872 | 1 | 0 |
| chr4 | 126368794 | A | C | 127 | Ago3 | intronic | . | . | . | 4qD2.2 | rs27564082 | 1 | 0 |
| chr4 | 126370252 | T | C | 228 | Ago3 | intronic | . | . | . | 4qD2.2 | rs27564080 | 1 | 0 |
| chr4 | 126370396 | A | T | 228 | Ago3 | intronic | . | . | . | 4qD2.2 | rs216326207 | 1 | 0 |
| chr4 | 126370579 | C | T | 140 | Ago3 | intronic | . | . | . | 4qD2.2 | rs27564079 | 1 | 0 |
| chr4 | 126371409 | T | C | 228 | Ago3 | intronic | . | . | . | 4qD2.2 | rs27564076 | 1 | 0 |
| chr4 | 126371552 | T | C | 228 | Ago3 | exonic | synonymous SNV | Ago3:NM_153402:exon8:c.A945G:p.E315E | . | 4qD2.2 | rs27564074 | 1 | 0 |
| chr4 | 126371710 | C | T | 228 | Ago3 | intronic | . | . | . | 4qD2.2 | rs27564073 | 1 | 0 |
| chr4 | 126377090 | C | T | 170 | Ago3 | intronic | . | . | . | 4qD2.2 | rs245083794 | 1 | 0 |
| chr4 | 126403991 | A | C | 160 | Ago3 | intronic | . | . | . | 4qD2.2 | . | 1 | 0 |
| chr4 | 126404146 | A | G | 228 | Ago3 | intronic | . | . | . | 4qD2.2 | rs229189997 | 1 | 0 |
| chr4 | 126404456 | G | T | 228 | Ago3 | intronic | . | . | . | 4qD2.2 | rs227110486 | 1 | 0 |
| chr4 | 126404481 | G | A | 228 | Ago3 | intronic | . | . | . | 4qD2.2 | rs246212843 | 1 | 0 |
| chr4 | 126407680 | G | T | 228 | Ago3 | intronic | . | . | . | 4qD2.2 | rs225984943 | 1 | 0 |
| chr4 | 126407901 | T | C | 228 | Ago3 | intronic | . | . | . | 4qD2.2 | rs27563971 | 1 | 0 |
| chr4 | 126417153 | T | C | 228 | Ago3 | intronic | . | . | . | 4qD2.2 | rs239769553 | 1 | 0 |
| chr4 | 126417493 | T | C | 228 | Ago3 | intronic | . | . | . | 4qD2.2 | rs27563902 | 1 | 0 |
| chr4 | 126429260 | C | T | 228 | Ago3 | intronic | . | . | 598 | 4qD2.2 | rs27548249 | 1 | 0 |
| chr4 | 126439288 | T | G | 150 | Ago1 | UTR3 | . | . | . | 4qD2.2 | rs247391171 | 1 | 0 |
| chr4 | 126439500 | C | T | 228 | Ago1 | exonic | unknown | UNKNOWN | . | 4qD2.2 | rs27548206 | 1 | 0 |
| chr4 | 126439719 | G | A | 228 | Ago1 | intronic | . | . | . | 4qD2.2 | rs249010270 | 1 | 0 |
| chr4 | 126439829 | A | C | 228 | Ago1 | intronic | . | . | . | 4qD2.2 | rs27548204 | 1 | 0 |
| chr4 | 126439976 | G | A | 228 | Ago1 | exonic | synonymous SNV | Ago1:NM_001317174:exon18:c.C1878T:p.L626L,Ago1:NM_153403:exon18:c.C2331T:p.L777L | . | 4qD2.2 | rs27548203 | 1 | 0 |
| chr4 | 126440340 | A | C | 228 | Ago1 | intronic | . | . | . | 4qD2.2 | rs258437018 | 1 | 0 |
| chr4 | 126441041 | G | A | 196 | Ago1 | intronic | . | . | . | 4qD2.2 | rs27548202 | 1 | 0 |
| chr4 | 126441240 | G | T | 228 | Ago1 | exonic | synonymous SNV | Ago1:NM_001317174:exon16:c.C1647A:p.I549I,Ago1:NM_153403:exon16:c.C2100A:p.I700I | . | 4qD2.2 | rs27548201 | 1 | 0 |
| chr4 | 126441457 | C | T | 228 | Ago1 | intronic | . | . | . | 4qD2.2 | rs27548200 | 1 | 0 |
| chr4 | 126441847 | C | G | 228 | Ago1 | intronic | . | . | . | 4qD2.2 | rs27548197 | 1 | 0 |
| chr4 | 126441907 | G | A | 228 | Ago1 | exonic | synonymous SNV | Ago1:NM_001317174:exon15:c.C1557T:p.P519P,Ago1:NM_153403:exon15:c.C2010T:p.P670P | . | 4qD2.2 | rs27548196 | 1 | 0 |
| chr4 | 126442103 | C | T | 228 | Ago1 | intronic | . | . | . | 4qD2.2 | rs244302410 | 1 | 0 |
| chr4 | 126442572 | T | C | 101 | Ago1 | intronic | . | . | . | 4qD2.2 | rs245994261 | 1 | 0 |
| chr4 | 126442720 | A | T | 228 | Ago1 | intronic | . | . | . | 4qD2.2 | rs27548190 | 1 | 0 |
| chr4 | 126442802 | C | T | 228 | Ago1 | intronic | . | . | . | 4qD2.2 | rs27548189 | 1 | 0 |
| chr4 | 126443055 | G | T | 228 | Ago1 | intronic | . | . | . | 4qD2.2 | rs27548187 | 1 | 0 |
| chr4 | 126443366 | T | C | 210 | Ago1 | intronic | . | . | . | 4qD2.2 | rs246227771 | 1 | 0 |
| chr4 | 126448974 | G | A | 228 | Ago1 | intronic | . | . | . | 4qD2.2 | . | 1 | 0 |
| chr4 | 126449101 | C | G | 170 | Ago1 | intronic | . | . | . | 4qD2.2 | rs249408192 | 1 | 0 |
| chr4 | 126449111 | T | C | 165 | Ago1 | intronic | . | . | . | 4qD2.2 | rs264114469 | 1 | 0 |
| chr4 | 126453626 | C | T | 228 | Ago1 | exonic | synonymous SNV | Ago1:NM_001317174:exon11:c.G876A:p.G292G,Ago1:NM_153403:exon11:c.G1329A:p.G443G | . | 4qD2.2 | rs27548145 | 1 | 0 |
| chr4 | 126453737 | T | G | 228 | Ago1 | intronic | . | . | . | 4qD2.2 | rs27548144 | 1 | 0 |
| chr4 | 126453769 | A | G | 228 | Ago1 | intronic | . | . | . | 4qD2.2 | rs239054137 | 1 | 0 |
| chr4 | 126453868 | T | C | 228 | Ago1 | exonic | synonymous SNV | Ago1:NM_001317174:exon10:c.A768G:p.T256T,Ago1:NM_153403:exon10:c.A1221G:p.T407T | . | 4qD2.2 | rs27548143 | 1 | 0 |
| chr4 | 126454101 | G | A | 228 | Ago1 | intronic | . | . | . | 4qD2.2 | rs264019064 | 1 | 0 |
| chr4 | 126454262 | T | G | 228 | Ago1 | intronic | . | . | . | 4qD2.2 | rs27548141 | 1 | 0 |
| chr4 | 126454284 | T | C | 228 | Ago1 | intronic | . | . | . | 4qD2.2 | rs27548140 | 1 | 0 |
| chr4 | 126454462 | C | T | 228 | Ago1 | intronic | . | . | . | 4qD2.2 | rs27548139 | 1 | 0 |
| chr4 | 126454485 | T | C | 228 | Ago1 | intronic | . | . | . | 4qD2.2 | rs27548138 | 1 | 0 |
| chr4 | 126459085 | T | C | 228 | Ago1 | intronic | . | . | . | 4qD2.2 | rs27548118 | 1 | 0 |
| chr4 | 126459355 | C | A | 228 | Ago1 | intronic | . | . | . | 4qD2.2 | rs221786805 | 1 | 0 |
| chr4 | 126459356 | C | T | 228 | Ago1 | intronic | . | . | . | 4qD2.2 | rs241101904 | 1 | 0 |
| chr4 | 126459584 | A | G | 149 | Ago1 | intronic | . | . | . | 4qD2.2 | rs27548114 | 1 | 0 |
| chr4 | 126459636 | A | G | 169 | Ago1 | intronic | . | . | . | 4qD2.2 | rs27548113 | 1 | 0 |
| chr4 | 126459731 | G | C | 228 | Ago1 | intronic | . | . | . | 4qD2.2 | rs27548111 | 1 | 0 |
| chr4 | 126459924 | T | C | 228 | Ago1 | intronic | . | . | . | 4qD2.2 | rs27548110 | 1 | 0 |
| chr4 | 126460150 | T | G | 228 | Ago1 | intronic | . | . | . | 4qD2.2 | rs242931156 | 1 | 0 |
| chr4 | 126461146 | G | T | 228 | Ago1 | intronic | . | . | . | 4qD2.2 | rs27548107 | 1 | 0 |
| chr4 | 126461579 | C | T | 102 | Ago1 | intronic | . | . | . | 4qD2.2 | rs27548103 | 1 | 0 |
| chr4 | 126461659 | A | T | 205 | Ago1 | intronic | . | . | . | 4qD2.2 | rs212615654 | 1 | 0 |
| chr4 | 126461669 | C | T | 228 | Ago1 | intronic | . | . | . | 4qD2.2 | rs27548102 | 1 | 0 |
| chr4 | 126463608 | G | A | 228 | Ago1 | intronic | . | . | . | 4qD2.2 | rs215255777 | 1 | 0 |
| chr4 | 126463871 | C | T | 228 | Ago1 | intronic | . | . | . | 4qD2.2 | rs217408140 | 1 | 0 |
| chr4 | 126463876 | C | G | 228 | Ago1 | intronic | . | . | . | 4qD2.2 | rs31949146 | 1 | 0 |
| chr4 | 126463972 | G | A | 157 | Ago1 | intronic | . | . | . | 4qD2.2 | rs256112531 | 1 | 0 |
| chr4 | 126493163 | G | A | 169 | Ago4 | UTR3 | . | . | . | 4qD2.2 | rs27548013 | 1 | 0 |
| chr4 | 126493183 | G | A | 228 | Ago4 | UTR3 | . | . | . | 4qD2.2 | rs27548012 | 1 | 0 |
| chr4 | 126493487 | G | C | 146 | Ago4 | intronic | . | . | . | 4qD2.2 | rs27548005 | 1 | 0 |
| chr4 | 126493499 | A | T | 127 | Ago4 | intronic | . | . | . | 4qD2.2 | rs27548004 | 1 | 0 |
| chr4 | 126496610 | G | A | 166 | Ago4 | intronic | . | . | . | 4qD2.2 | rs234730028 | 1 | 0 |
| chr4 | 126496652 | C | T | 173 | Ago4 | intronic | . | . | . | 4qD2.2 | rs261679991 | 1 | 0 |
| chr4 | 126496952 | G | A | 228 | Ago4 | splicing | . | . | . | 4qD2.2 | . | 1 | 0 |
| chr4 | 126497334 | T | A | 228 | Ago4 | intronic | . | . | . | 4qD2.2 | rs221648518 | 1 | 0 |
| chr4 | 126497378 | A | G | 228 | Ago4 | intronic | . | . | . | 4qD2.2 | rs241992620 | 1 | 0 |
| chr4 | 126502218 | A | G | 228 | Ago4 | intronic | . | . | . | 4qD2.2 | rs259872588 | 1 | 0 |
| chr4 | 126502241 | A | C | 228 | Ago4 | intronic | . | . | . | 4qD2.2 | rs226641862 | 1 | 0 |
| chr4 | 126502249 | A | C | 228 | Ago4 | intronic | . | . | . | 4qD2.2 | rs248700853 | 1 | 0 |
| chr4 | 126502383 | C | T | 228 | Ago4 | intronic | . | . | . | 4qD2.2 | rs264674048 | 1 | 0 |
| chr4 | 126502399 | G | A | 228 | Ago4 | intronic | . | . | . | 4qD2.2 | rs578566940 | 1 | 0 |
| chr4 | 126504456 | C | T | 170 | Ago4 | intronic | . | . | . | 4qD2.2 | rs220841847 | 1 | 0 |
| chr4 | 126504461 | A | G | 171 | Ago4 | intronic | . | . | . | 4qD2.2 | rs238730297 | 1 | 0 |
| chr4 | 126504662 | C | T | 228 | Ago4 | intronic | . | . | . | 4qD2.2 | rs226909740 | 1 | 0 |
| chr4 | 126504763 | G | A | 228 | Ago4 | intronic | . | . | . | 4qD2.2 | rs27547986 | 1 | 0 |
| chr4 | 126504809 | G | A | 228 | Ago4 | intronic | . | . | . | 4qD2.2 | rs239710961 | 1 | 0 |
| chr4 | 126506518 | G | A | 98 | Ago4 | intronic | . | . | . | 4qD2.2 | rs238929544 | 1 | 0 |
| chr4 | 126506627 | C | T | 228 | Ago4 | intronic | . | . | . | 4qD2.2 | rs254311007 | 1 | 0 |
| chr4 | 126506733 | C | T | 228 | Ago4 | intronic | . | . | . | 4qD2.2 | rs244352693 | 1 | 0 |
| chr4 | 126506804 | A | G | 228 | Ago4 | exonic | synonymous SNV | Ago4:NM_153177:exon15:c.T2115C:p.T705T | . | 4qD2.2 | rs27547982 | 1 | 0 |
| chr4 | 126507131 | G | A | 228 | Ago4 | exonic | synonymous SNV | Ago4:NM_153177:exon14:c.C1920T:p.V640V | . | 4qD2.2 | rs27547981 | 1 | 0 |
| chr4 | 126507160 | T | C | 228 | Ago4 | exonic | missense SNV | Ago4:NM_153177:exon14:c.A1891G:p.T631A | . | 4qD2.2 | rs27547980 | 1 | 0 |
| chr4 | 126508392 | G | A | 228 | Ago4 | exonic | synonymous SNV | Ago4:NM_153177:exon13:c.C1680T:p.N560N | . | 4qD2.2 | rs27547977 | 1 | 0 |
| chr4 | 126508476 | T | C | 228 | Ago4 | exonic | synonymous SNV | Ago4:NM_153177:exon13:c.A1596G:p.L532L | . | 4qD2.2 | rs249633206 | 1 | 0 |
| chr4 | 126510155 | G | A | 228 | Ago4 | intronic | . | . | . | 4qD2.2 | rs27547968 | 1 | 0 |
| chr4 | 126511133 | C | G | 148 | Ago4 | intronic | . | . | . | 4qD2.2 | rs27523055 | 1 | 0 |
| chr4 | 126511319 | T | C | 228 | Ago4 | intronic | . | . | . | 4qD2.2 | rs27523054 | 1 | 0 |
| chr4 | 126511383 | A | G | 228 | Ago4 | intronic | . | . | . | 4qD2.2 | rs27523053 | 1 | 0 |
| chr4 | 126512489 | G | A | 228 | Ago4 | intronic | . | . | . | 4qD2.2 | rs27523051 | 1 | 0 |
| chr4 | 126512684 | T | C | 180 | Ago4 | intronic | . | . | . | 4qD2.2 | rs27523048 | 1 | 0 |
| chr4 | 126517629 | T | C | 228 | Ago4 | intronic | . | . | . | 4qD2.2 | rs257427337 | 1 | 0 |
| chr4 | 126517688 | T | C | 158 | Ago4 | intronic | . | . | . | 4qD2.2 | rs258341853 | 1 | 0 |
| chr4 | 126520073 | T | G | 195 | Ago4 | intronic | . | . | . | 4qD2.2 | rs27523025 | 1 | 0 |
| chr4 | 126520115 | A | C | 228 | Ago4 | intronic | . | . | . | 4qD2.2 | rs27523024 | 1 | 0 |
| chr4 | 126520266 | A | G | 228 | Ago4 | exonic | synonymous SNV | Ago4:NM_153177:exon3:c.T231C:p.F77F | . | 4qD2.2 | rs27523021 | 1 | 0 |
| chr4 | 126525869 | A | G | 142 | Ago4 | intronic | . | . | . | 4qD2.2 | rs240311544 | 1 | 0 |
| chr4 | 126525870 | T | A | 146 | Ago4 | intronic | . | . | . | 4qD2.2 | rs264137944 | 1 | 0 |
| chr4 | 126525938 | A | G | 195 | Ago4 | intronic | . | . | . | 4qD2.2 | rs241625928 | 1 | 0 |
| chr4 | 126526171 | G | A | 228 | Ago4 | intronic | . | . | . | 4qD2.2 | . | 1 | 0 |
| chr4 | 126541476 | C | T | 228 | . | intergenic | . | . | . | 4qD2.2 | rs256701202 | 1 | 0 |
| chr4 | 126556972 | C | A | 205 | . | upstream | . | . | 723 | 4qD2.2 | rs27522933 | 1 | 0 |
| chr4 | 126557173 | T | C | 228 | Clspn | intronic | . | . | 723 | 4qD2.2 | rs48777846 | 1 | 0 |
| chr4 | 126557199 | A | G | 228 | Clspn | intronic | . | . | 723 | 4qD2.2 | rs27522932 | 1 | 0 |
| chr4 | 126559063 | T | C | 168 | Clspn | intronic | . | . | . | 4qD2.2 | rs27522923 | 1 | 0 |
| chr4 | 126559396 | T | G | 183 | Clspn | intronic | . | . | . | 4qD2.2 | rs27522922 | 1 | 0 |
| chr4 | 126559770 | A | G | 160 | Clspn | intronic | . | . | . | 4qD2.2 | rs27522921 | 1 | 0 |
| chr4 | 126559848 | T | G | 210 | Clspn | intronic | . | . | . | 4qD2.2 | rs27522920 | 1 | 0 |
| chr4 | 126560328 | A | G | 228 | Clspn | exonic | synonymous SNV | Clspn:NM_175554:exon3:c.A543G:p.L181L | . | 4qD2.2 | rs27522916 | 1 | 0 |
| chr4 | 126561683 | A | G | 39.83 | Clspn | intronic | . | . | . | 4qD2.2 | rs27522913 | 0 | 1 |
| chr4 | 126561710 | A | G | 228 | Clspn | intronic | . | . | . | 4qD2.2 | rs27522912 | 1 | 0 |
| chr4 | 126561859 | G | A | 228 | Clspn | exonic | synonymous SNV | Clspn:NM_175554:exon4:c.G669A:p.E223E | . | 4qD2.2 | rs27522911 | 1 | 0 |

| | | | | | | | | | | | | | |
|---|---|---|---|---|---|---|---|---|---|---|---|---|---|
| chr4 | 126564783 | G | A | 171 | Clspn | intronic | . | . | . | 4qD2.2 | rs27522909 | 1 | 0 |
| chr4 | 126565112 | A | C | 228 | Clspn | intronic | . | . | . | 4qD2.2 | rs27522907 | 1 | 0 |
| chr4 | 126565966 | T | C | 228 | Clspn | intronic | . | . | . | 4qD2.2 | rs27522903 | 1 | 0 |
| chr4 | 126566263 | T | C | 228 | Clspn | exonic | missense SNV | Clspn:NM_175554:exon8:c.T1282C:p.S428P | . | 4qD2.2 | rs230375845 | 1 | 0 |
| chr4 | 126566666 | G | A | 228 | Clspn | intronic | . | . | . | 4qD2.2 | rs27522902 | 1 | 0 |
| chr4 | 126566731 | G | A | 202 | Clspn | intronic | . | . | . | 4qD2.2 | rs253419262 | 1 | 0 |
| chr4 | 126574585 | A | C | 228 | Clspn | intronic | . | . | . | 4qD2.2 | rs27522875 | 1 | 0 |
| chr4 | 126575132 | T | C | 228 | Clspn | exonic | missense SNV | Clspn:NM_175554:exon12:c.T2179C:p.Y727H | . | 4qD2.2 | rs27522874 | 1 | 0 |
| chr4 | 126576285 | G | C | 228 | Clspn | intronic | . | . | . | 4qD2.2 | rs27522863 | 1 | 0 |
| chr4 | 126577564 | T | C | 228 | Clspn | intronic | . | . | . | 4qD2.2 | rs27522861 | 1 | 0 |
| chr4 | 126577608 | C | T | 228 | Clspn | intronic | . | . | . | 4qD2.2 | rs27522860 | 1 | 0 |
| chr4 | 126581162 | C | T | 228 | Clspn | intronic | . | . | . | 4qD2.2 | rs27522854 | 1 | 0 |
| chr4 | 126581653 | G | A | 218 | Clspn | intronic | . | . | . | 4qD2.2 | rs27522850 | 1 | 0 |
| chr4 | 126584482 | A | T | 212 | Clspn | intronic | . | . | . | 4qD2.2 | rs27522837 | 1 | 0 |
| chr4 | 126584524 | C | T | 228 | Clspn | intronic | . | . | . | 4qD2.2 | rs260554608 | 1 | 0 |
| chr4 | 126584545 | G | A | 228 | Clspn | intronic | . | . | . | 4qD2.2 | rs27522835 | 1 | 0 |
| chr4 | 126584829 | G | A | 228 | Clspn | intronic | . | . | . | 4qD2.2 | rs27522830 | 1 | 0 |
| chr4 | 126584864 | T | A | 228 | Clspn | intronic | . | . | . | 4qD2.2 | rs27522829 | 1 | 0 |
| chr4 | 126584937 | A | G | 203 | Clspn | intronic | . | . | . | 4qD2.2 | rs27522828 | 1 | 0 |
| chr4 | 126585757 | G | T | 228 | Clspn | intronic | . | . | . | 4qD2.2 | rs215082256 | 1 | 0 |
| chr4 | 126585862 | T | C | 228 | Clspn | exonic | synonymous SNV | Clspn:NM_175554:exon19:c.T3099C:p.D1033D | . | 4qD2.2 | rs27522822 | 1 | 0 |
| chr4 | 126586017 | G | A | 228 | Clspn | intronic | . | . | . | 4qD2.2 | rs27522820 | 1 | 0 |
| chr4 | 126586238 | C | T | 228 | Clspn | intronic | . | . | . | 4qD2.2 | rs27522819 | 1 | 0 |
| chr4 | 126587427 | G | A | 180 | Clspn | intronic | . | . | . | 4qD2.2 | rs27522817 | 1 | 0 |
| chr4 | 126587465 | G | A | 228 | Clspn | intronic | . | . | . | 4qD2.2 | rs27522816 | 1 | 0 |
| chr4 | 126587538 | G | T | 208 | Clspn | intronic | . | . | . | 4qD2.2 | rs213297616 | 1 | 0 |
| chr4 | 126587580 | A | G | 228 | Clspn | exonic | synonymous SNV | Clspn:NM_175554:exon21:c.A3384G:p.L1128L | . | 4qD2.2 | rs27522815 | 1 | 0 |
| chr4 | 126587625 | C | A | 228 | Clspn | exonic | missense SNV | Clspn:NM_175554:exon21:c.C3429A:p.D1143E | . | 4qD2.2 | rs27522814 | 1 | 0 |
| chr4 | 126590662 | A | T | 228 | Clspn | intronic | . | . | . | 4qD2.2 | rs27522810 | 1 | 0 |
| chr4 | 126590677 | G | A | 228 | Clspn | intronic | . | . | . | 4qD2.2 | rs27522809 | 1 | 0 |
| chr4 | 126590974 | T | C | 127 | Clspn | intronic | . | . | . | 4qD2.2 | rs27522806 | 1 | 0 |
| chr4 | 126591192 | A | G | 228 | Clspn | exonic | missense SNV | Clspn:NM_175554:exon23:c.A3601G:p.T1201A | . | 4qD2.2 | rs27522805 | 1 | 0 |
| chr4 | 126591258 | G | A | 228 | Clspn | exonic | missense SNV | Clspn:NM_175554:exon23:c.G3667A:p.A1223T | . | 4qD2.2 | rs27522804 | 1 | 0 |
| chr4 | 126591333 | C | T | 228 | Clspn | intronic | . | . | . | 4qD2.2 | rs254355436 | 1 | 0 |
| chr4 | 126591385 | A | C | 73 | Clspn | intronic | . | . | . | 4qD2.2 | rs578404540 | 1 | 0 |
| chr4 | 126591506 | C | T | 156 | Clspn | intronic | . | . | . | 4qD2.2 | rs230537498 | 1 | 0 |
| chr4 | 126591735 | A | G | 228 | Clspn | exonic | synonymous SNV | Clspn:NM_175554:exon24:c.A3816G:p.A1272A | . | 4qD2.2 | rs265688164 | 1 | 0 |
| chr4 | 126592514 | A | G | 174 | Clspn | intronic | . | . | . | 4qD2.2 | rs27522803 | 1 | 0 |
| chr4 | 126592539 | C | A | 178 | Clspn | intronic | . | . | . | 4qD2.2 | rs27522802 | 1 | 0 |
| chr4 | 126592668 | T | C | 228 | Clspn | exonic | missense SNV | Clspn:NM_175554:exon25:c.T3854C:p.L1285P | . | 4qD2.2 | rs27522801 | 1 | 0 |
| chr4 | 126611575 | A | C | 139 | 5730409E04Rik | intronic | . | . | . | 4qD2.2 | rs221538370 | 1 | 0 |
| chr4 | 126612253 | G | A | 228 | 5730409E04Rik | exonic | synonymous SNV | 5730409E04Rik:NM_001013755:exon2:c.G573A:p.Q191Q,5730409E04Rik:NM_001145950:exon2:c.G573A:p. | . | 4qD2.2 | . | 1 | 0 |
| chr4 | 126612341 | G | A | 228 | 5730409E04Rik | exonic | missense SNV | 5730409E04Rik:NM_001013755:exon2:c.G661A:p.E221K,5730409E04Rik:NM_001145950:exon2:c.G661A:p. | . | 4qD2.2 | . | 1 | 0 |
| chr4 | 126665336 | T | C | 195 | . | intergenic | . | . | . | 4qD2.2 | rs27564678 | 1 | 0 |
| chr4 | 126665351 | C | T | 194 | . | intergenic | . | . | . | 4qD2.2 | rs218350195 | 1 | 0 |
| chr4 | 126665361 | G | C | 57 | . | intergenic | . | . | . | 4qD2.2 | rs235584229 | 1 | 0 |
| chr4 | 126665481 | A | G | 228 | . | intergenic | . | . | . | 4qD2.2 | rs27564674 | 1 | 0 |
| chr4 | 126665556 | G | A | 228 | . | intergenic | . | . | . | 4qD2.2 | rs581198173 | 1 | 0 |
| chr4 | 126665622 | T | A | 228 | . | intergenic | . | . | . | 4qD2.2 | rs27564671 | 1 | 0 |
| chr4 | 126665739 | T | C | 228 | . | intergenic | . | . | . | 4qD2.2 | rs584306368 | 1 | 0 |
| chr4 | 126665756 | G | A | 228 | . | intergenic | . | . | . | 4qD2.2 | rs27564670 | 1 | 0 |
| chr4 | 126665909 | C | T | 228 | . | intergenic | . | . | . | 4qD2.2 | . | 1 | 0 |
| chr4 | 126666180 | C | A | 228 | . | intergenic | . | . | . | 4qD2.2 | rs583831372 | 1 | 0 |
| chr4 | 126686978 | G | C | 228 | Psmb2 | intronic | . | . | . | 4qD2.2 | rs234504463 | 1 | 0 |
| chr4 | 126687116 | C | T | 228 | Psmb2 | intronic | . | . | . | 4qD2.2 | rs253359919 | 1 | 0 |
| chr4 | 126687117 | A | G | 228 | Psmb2 | intronic | . | . | . | 4qD2.2 | rs27564596 | 1 | 0 |
| chr4 | 126687203 | T | C | 228 | Psmb2 | intronic | . | . | . | 4qD2.2 | rs27564595 | 1 | 0 |
| chr4 | 126687277 | A | G | 126 | Psmb2 | intronic | . | . | . | 4qD2.2 | rs27564594 | 1 | 0 |
| chr4 | 126707645 | A | G | 228 | Psmb2 | intronic | . | . | . | 4qD2.2 | rs263419040 | 1 | 0 |
| chr4 | 126707679 | T | G | 228 | Psmb2 | intronic | . | . | . | 4qD2.2 | . | 1 | 0 |
| chr4 | 126707714 | T | C | 159 | Psmb2 | intronic | . | . | . | 4qD2.2 | rs254267255 | 1 | 0 |
| chr4 | 126709278 | A | T | 155 | Psmb2 | intronic | . | . | . | 4qD2.2 | rs239850168 | 1 | 0 |
| chr4 | 126709380 | G | A | 228 | Psmb2 | intronic | . | . | . | 4qD2.2 | . | 1 | 0 |
| chr4 | 126709508 | C | G | 228 | Psmb2 | exonic | synonymous SNV | Psmb2:NM_011970:exon6:c.C528G:p.P176P | . | 4qD2.2 | rs231149963 | 1 | 0 |
| chr4 | 126709724 | G | T | 130 | . | downstream | . | . | . | 4qD2.2 | rs234303042 | 1 | 0 |
| chr4 | 126716370 | T | G | 143 | Tfap2e | UTR3 | . | . | . | 4qD2.2 | . | 1 | 0 |
| chr4 | 126716371 | T | C | 148 | Tfap2e | UTR3 | . | . | . | 4qD2.2 | . | 1 | 0 |
| chr4 | 126716457 | G | C | 228 | Tfap2e | UTR3 | . | . | . | 4qD2.2 | . | 1 | 0 |
| chr4 | 126716711 | A | G | 228 | Tfap2e | exonic | synonymous SNV | Tfap2e:NM_198960:exon7:c.T1131C:p.P377P | . | 4qD2.2 | rs32386196 | 1 | 0 |
| chr4 | 126719282 | T | A | 90 | Tfap2e | intronic | . | . | . | 4qD2.2 | rs220802364 | 1 | 0 |
| chr4 | 126719284 | T | C | 90 | Tfap2e | intronic | . | . | . | 4qD2.2 | rs27564471 | 1 | 0 |
| chr4 | 126719459 | G | A | 228 | Tfap2e | exonic | synonymous SNV | Tfap2e:NM_198960:exon6:c.C999T:p.A333A | . | 4qD2.2 | rs261893316 | 1 | 0 |
| chr4 | 126719608 | G | A | 228 | Tfap2e | intronic | . | . | . | 4qD2.2 | rs27564468 | 1 | 0 |
| chr4 | 126722214 | A | G | 188 | Tfap2e | intronic | . | . | . | 4qD2.2 | . | 1 | 0 |
| chr4 | 126734681 | T | C | 228 | Tfap2e | intronic | . | . | . | 4qD2.2 | rs31862691 | 1 | 0 |
| chr4 | 126734811 | G | A | 165 | Tfap2e | intronic | . | . | . | 4qD2.2 | . | 1 | 0 |
| chr4 | 126735368 | A | G | 103 | Tfap2e | exonic | synonymous SNV | Tfap2e:NM_198960:exon2:c.T312C:p.R104R | 1120 | 4qD2.2 | rs214001956 | 1 | 0 |
| chr4 | 126735791 | G | A | 199 | Tfap2e | intronic | . | . | 1120 | 4qD2.2 | . | 1 | 0 |
| chr4 | 126735840 | C | T | 228 | Tfap2e | intronic | . | . | 1120 | 4qD2.2 | rs263495144 | 1 | 0 |
| chr4 | 126736160 | A | C | 207 | Tfap2e | UTR5 | . | . | 1120 | 4qD2.2 | rs215913160 | 1 | 0 |
| chr4 | 126745072 | C | T | 228 | Ncdn | exonic | synonymous SNV | Ncdn:NM_011986:exon7:c.G1953A:p.L651L | . | 4qD2.2 | rs261692262 | 1 | 0 |
| chr4 | 126745311 | T | C | 228 | Ncdn | intronic | . | . | . | 4qD2.2 | rs27549411 | 1 | 0 |
| chr4 | 126746423 | A | G | 182 | Ncdn | intronic | . | . | . | 4qD2.2 | rs264721477 | 1 | 0 |
| chr4 | 126746524 | A | G | 228 | Ncdn | intronic | . | . | . | 4qD2.2 | rs27549409 | 1 | 0 |
| chr4 | 126746688 | T | C | 228 | Ncdn | exonic | synonymous SNV | Ncdn:NM_011986:exon6:c.A1611G:p.K537K | . | 4qD2.2 | rs27549408 | 1 | 0 |
| chr4 | 126746840 | T | C | 217 | Ncdn | intronic | . | . | . | 4qD2.2 | rs27549405 | 1 | 0 |
| chr4 | 126746891 | C | T | 228 | Ncdn | intronic | . | . | . | 4qD2.2 | rs27549404 | 1 | 0 |
| chr4 | 126746943 | A | C | 170 | Ncdn | intronic | . | . | . | 4qD2.2 | rs27549403 | 1 | 0 |
| chr4 | 126747041 | G | A | 228 | Ncdn | intronic | . | . | . | 4qD2.2 | rs223203400 | 1 | 0 |
| chr4 | 126747182 | C | G | 228 | Ncdn | exonic | synonymous SNV | Ncdn:NM_011986:exon5:c.G1524C:p.S508S | . | 4qD2.2 | rs27549401 | 1 | 0 |
| chr4 | 126747462 | G | T | 141 | Ncdn | intronic | . | . | . | 4qD2.2 | rs226974598 | 1 | 0 |
| chr4 | 126748498 | T | A | 225 | Ncdn | exonic | synonymous SNV | Ncdn:NM_011986:exon4:c.A1365T:p.S455S | . | 4qD2.2 | rs240162690 | 1 | 0 |
| chr4 | 126748651 | C | T | 228 | Ncdn | exonic | synonymous SNV | Ncdn:NM_011986:exon4:c.G1212A:p.A404A | . | 4qD2.2 | rs27549398 | 1 | 0 |
| chr4 | 126749867 | G | A | 228 | Ncdn | intronic | . | . | . | 4qD2.2 | rs27549391 | 1 | 0 |
| chr4 | 126750074 | T | C | 228 | Ncdn | exonic | synonymous SNV | Ncdn:NM_011986:exon3:c.A954G:p.V318V | . | 4qD2.2 | rs27549390 | 1 | 0 |
| chr4 | 126750089 | T | C | 228 | Ncdn | exonic | synonymous SNV | Ncdn:NM_011986:exon3:c.A939G:p.L313L | . | 4qD2.2 | rs27549389 | 1 | 0 |
| chr4 | 126750235 | G | A | 228 | Ncdn | exonic | synonymous SNV | Ncdn:NM_011986:exon3:c.C793T:p.L265L | . | 4qD2.2 | rs27549388 | 1 | 0 |
| chr4 | 126750458 | G | A | 228 | Ncdn | exonic | synonymous SNV | Ncdn:NM_011986:exon3:c.C570T:p.Y190Y | . | 4qD2.2 | rs27549387 | 1 | 0 |
| chr4 | 126750515 | G | A | 228 | Ncdn | exonic | synonymous SNV | Ncdn:NM_011986:exon3:c.C513T:p.H171H | . | 4qD2.2 | . | 1 | 0 |
| chr4 | 126753168 | T | C | 126 | Ncdn | UTR5 | . | . | 1152 | 4qD2.2 | rs27549383 | 1 | 0 |
| chr4 | 126757129 | T | C | 228 | AU040320 | intronic | . | . | . | 4qD2.2 | rs47125202 | 1 | 0 |
| chr4 | 126791733 | C | T | 228 | AU040320 | intronic | . | . | . | 4qD2.2 | rs27549256 | 1 | 0 |
| chr4 | 126791965 | T | C | 228 | AU040320 | exonic | synonymous SNV | AU040320:NM_001035525:exon3:c.T333C:p.S111S,AU040320:NM_001035526:exon3:c.T333C:p.S111S,AU04 | . | 4qD2.2 | rs27549255 | 1 | 0 |
| chr4 | 126792069 | A | C | 221 | AU040320 | exonic | missense SNV | AU040320:NM_001035525:exon3:c.A437C:p.D146A,AU040320:NM_001035526:exon3:c.A437C:p.D146A,AU04 | . | 4qD2.2 | . | 0 | 1 |
| chr4 | 126792259 | G | C | 228 | AU040320 | exonic | synonymous SNV | AU040320:NM_001035525:exon3:c.G627C:p.S209S,AU040320:NM_001035526:exon3:c.G627C:p.S209S,AU04 | . | 4qD2.2 | rs27549253 | 1 | 0 |
| chr4 | 126792333 | A | G | 228 | AU040320 | intronic | . | . | . | 4qD2.2 | rs27549252 | 1 | 0 |
| chr4 | 126792340 | C | A | 228 | AU040320 | intronic | . | . | . | 4qD2.2 | rs251088266 | 1 | 0 |
| chr4 | 126795619 | A | G | 228 | AU040320 | intronic | . | . | . | 4qD2.2 | rs50453366 | 1 | 0 |
| chr4 | 126795625 | A | G | 228 | AU040320 | intronic | . | . | . | 4qD2.2 | rs27549242 | 1 | 0 |
| chr4 | 126796473 | A | G | 228 | AU040320 | intronic | . | . | . | 4qD2.2 | rs27549241 | 1 | 0 |
| chr4 | 126814628 | T | C | 149 | AU040320 | intronic | . | . | . | 4qD2.2 | rs242830374 | 1 | 0 |
| chr4 | 126816245 | T | C | 110 | AU040320 | intronic | . | . | . | 4qD2.2 | rs217776229 | 1 | 0 |
| chr4 | 126816288 | G | A | 166 | AU040320 | intronic | . | . | . | 4qD2.2 | rs27549192 | 1 | 0 |
| chr4 | 126816446 | T | C | 228 | AU040320 | exonic | synonymous SNV | AU040320:NM_001035525:exon5:c.T984C:p.N328N,AU040320:NM_001035526:exon5:c.T984C:p.N328N,AU04 | . | 4qD2.2 | rs27549189 | 1 | 0 |
| chr4 | 126816555 | G | A | 228 | AU040320 | intronic | . | . | . | 4qD2.2 | rs27549187 | 1 | 0 |
| chr4 | 126816576 | G | A | 205 | AU040320 | intronic | . | . | . | 4qD2.2 | rs27549186 | 1 | 0 |
| chr4 | 126823512 | G | A | 177 | AU040320 | intronic | . | . | . | 4qD2.2 | rs48532158 | 1 | 0 |
| chr4 | 126828585 | G | T | 139 | AU040320 | intronic | . | . | . | 4qD2.2 | rs27525458 | 1 | 0 |
| chr4 | 126828644 | G | C | 160 | AU040320 | intronic | . | . | . | 4qD2.2 | rs27525457 | 1 | 0 |
| chr4 | 126828737 | C | T | 228 | AU040320 | intronic | . | . | . | 4qD2.2 | rs27525456 | 1 | 0 |
| chr4 | 126828768 | C | T | 228 | AU040320 | intronic | . | . | . | 4qD2.2 | rs27525455 | 1 | 0 |
| chr4 | 126828851 | C | A | 228 | AU040320 | exonic | synonymous SNV | AU040320:NM_001035525:exon7:c.C1161A:p.A387A,AU040320:NM_001035526:exon7:c.C1161A:p.A387A,AU | . | 4qD2.2 | rs27525454 | 1 | 0 |
| chr4 | 126830306 | T | G | 228 | AU040320 | intronic | . | . | . | 4qD2.2 | rs227756408 | 1 | 0 |
| chr4 | 126830445 | G | T | 228 | AU040320 | intronic | . | . | . | 4qD2.2 | rs27525439 | 1 | 0 |
| chr4 | 126830481 | G | A | 228 | AU040320 | intronic | . | . | . | 4qD2.2 | rs27525438 | 1 | 0 |
| chr4 | 126832002 | C | G | 187 | AU040320 | intronic | . | . | . | 4qD2.2 | rs226276970 | 1 | 0 |
| chr4 | 126832014 | T | C | 228 | AU040320 | intronic | . | . | . | 4qD2.2 | rs259497687 | 1 | 0 |
| chr4 | 126836484 | G | C | 170 | AU040320 | intronic | . | . | . | 4qD2.2 | rs27525410 | 1 | 0 |
| chr4 | 126836557 | T | C | 228 | AU040320 | intronic | . | . | . | 4qD2.2 | rs27525409 | 1 | 0 |
| chr4 | 126836823 | A | G | 228 | AU040320 | intronic | . | . | . | 4qD2.2 | rs6221068 | 1 | 0 |
| chr4 | 126836912 | T | C | 152 | AU040320 | intronic | . | . | . | 4qD2.2 | rs27525408 | 1 | 0 |
| chr4 | 126837130 | T | C | 85 | AU040320 | intronic | . | . | . | 4qD2.2 | rs27525405 | 1 | 0 |
| chr4 | 126839524 | T | C | 228 | AU040320 | intronic | . | . | . | 4qD2.2 | rs213121635 | 1 | 0 |
| chr4 | 126839533 | T | C | 228 | AU040320 | intronic | . | . | . | 4qD2.2 | rs27525397 | 1 | 0 |
| chr4 | 126839765 | C | A | 228 | AU040320 | intronic | . | . | . | 4qD2.2 | rs27525396 | 1 | 0 |
| chr4 | 126839921 | T | C | 162 | AU040320 | intronic | . | . | . | 4qD2.2 | rs579994091 | 1 | 0 |
| chr4 | 126840710 | A | G | 228 | AU040320 | exonic | synonymous SNV | AU040320:NM_001035525:exon14:c.A2124G:p.T708T,AU040320:NM_001035526:exon14:c.A2124G:p.T708T, | . | 4qD2.2 | rs241343776 | 1 | 0 |
| chr4 | 126840713 | T | C | 228 | AU040320 | exonic | synonymous SNV | AU040320:NM_001035525:exon14:c.T2127C:p.A709A,AU040320:NM_001035526:exon14:c.T2127C:p.A709A, | . | 4qD2.2 | rs254108324 | 1 | 0 |
| chr4 | 126841338 | A | C | 228 | AU040320 | intronic | . | . | . | 4qD2.2 | rs265569244 | 1 | 0 |
| chr4 | 126841342 | G | T | 228 | AU040320 | intronic | . | . | . | 4qD2.2 | rs232643671 | 1 | 0 |
| chr4 | 126842319 | A | G | 157 | AU040320 | intronic | . | . | . | 4qD2.2 | rs241921945 | 1 | 0 |
| chr4 | 126842412 | C | T | 228 | AU040320 | intronic | . | . | . | 4qD2.2 | rs27525382 | 1 | 0 |
| chr4 | 126842495 | A | G | 228 | AU040320 | exonic | synonymous SNV | AU040320:NM_001035525:exon16:c.A2370G:p.L790L,AU040320:NM_001035526:exon16:c.A2370G:p.L790L, | . | 4qD2.2 | rs27525381 | 1 | 0 |
| chr4 | 126842640 | T | C | 228 | AU040320 | intronic | . | . | . | 4qD2.2 | rs27525379 | 1 | 0 |
| chr4 | 126842789 | A | C | 214 | AU040320 | intronic | . | . | . | 4qD2.2 | rs27525378 | 1 | 0 |
| chr4 | 126842814 | G | A | 216 | AU040320 | intronic | . | . | . | 4qD2.2 | rs260117485 | 1 | 0 |
| chr4 | 126846523 | A | G | 228 | AU040320 | intronic | . | . | . | 4qD2.2 | rs27525363 | 1 | 0 |
| chr4 | 126846691 | C | G | 132 | AU040320 | intronic | . | . | . | 4qD2.2 | rs27525361 | 1 | 0 |
| chr4 | 126846998 | A | G | 159 | AU040320 | intronic | . | . | . | 4qD2.2 | rs27525356 | 1 | 0 |
| chr4 | 126847050 | T | C | 172 | AU040320 | intronic | . | . | . | 4qD2.2 | rs27525355 | 1 | 0 |
| chr4 | 126847357 | C | T | 228 | AU040320 | intronic | . | . | . | 4qD2.2 | . | 1 | 0 |
| chr4 | 126847443 | C | T | 160 | AU040320 | intronic | . | . | . | 4qD2.2 | rs27525354 | 1 | 0 |
| chr4 | 126847774 | C | T | 228 | AU040320 | splicing | . | . | . | 4qD2.2 | rs230395410 | 1 | 0 |
| chr4 | 126848645 | T | C | 167 | AU040320 | intronic | . | . | . | 4qD2.2 | rs241741930 | 1 | 0 |
| chr4 | 126848681 | T | C | 173 | AU040320 | intronic | . | . | . | 4qD2.2 | rs260921856 | 1 | 0 |
| chr4 | 126848766 | C | T | 228 | AU040320 | intronic | . | . | . | 4qD2.2 | rs27525351 | 1 | 0 |
| chr4 | 126848987 | A | G | 228 | AU040320 | intronic | . | . | . | 4qD2.2 | rs27525350 | 1 | 0 |
| chr4 | 126849022 | G | T | 170 | AU040320 | intronic | . | . | . | 4qD2.2 | rs27525349 | 1 | 0 |
| chr4 | 126853047 | C | T | 228 | AU040320 | intronic | . | . | . | 4qD2.2 | rs242690240 | 1 | 0 |
| chr4 | 126853146 | G | C | 228 | AU040320 | intronic | . | . | . | 4qD2.2 | rs27525329 | 1 | 0 |
| chr4 | 126853293 | C | A | 228 | AU040320 | exonic | synonymous SNV | AU040320:NM_133886:exon21:c.C3087A:p.G1029G | . | 4qD2.2 | rs27525328 | 1 | 0 |
| chr4 | 126854370 | C | A | 228 | AU040320 | intronic | . | . | . | 4qD2.2 | rs27525323 | 1 | 0 |
| chr4 | 126863952 | C | T | 58 | AU040320,Zmym4 | UTR3 | . | . | . | 4qD2.2 | rs256084788 | 1 | 0 |
| chr4 | 126863970 | T | C | 135 | Zmym4,AU040320 | UTR3 | . | . | . | 4qD2.2 | rs27525310 | 1 | 0 |
| chr4 | 126864370 | C | T | 228 | Zmym4,AU040320 | intronic | . | . | . | 4qD2.2 | rs212531764 | 1 | 0 |
| chr4 | 126868999 | G | T | 228 | Zmym4,AU040320 | intronic | . | . | . | 4qD2.2 | rs234379298 | 1 | 0 |
| chr4 | 126869134 | T | C | 219 | AU040320,Zmym4 | intronic | . | . | . | 4qD2.2 | rs260452950 | 1 | 0 |
| chr4 | 126869364 | G | A | 228 | AU040320,Zmym4 | exonic | synonymous SNV | AU040320:NM_001035525:exon24:c.G3231A:p.T1077T,AU040320:NM_001035526:exon24:c.G3231A:p.T1077 | . | 4qD2.2 | rs27525300 | 1 | 0 |
| chr4 | 126869587 | C | A | 166 | Zmym4,AU040320 | UTR3 | . | . | . | 4qD2.2 | rs27525299 | 1 | 0 |
| chr4 | 126870378 | A | G | 170 | Zmym4 | intronic | . | . | . | 4qD2.2 | rs27525297 | 1 | 0 |
| chr4 | 126877482 | G | A | 174 | Zmym4 | intronic | . | . | . | 4qD2.2 | rs27525283 | 1 | 0 |
| chr4 | 126877829 | T | C | 228 | Zmym4 | intronic | . | . | . | 4qD2.2 | rs6252195 | 1 | 0 |
| chr4 | 126877861 | C | T | 187 | Zmym4 | intronic | . | . | . | 4qD2.2 | rs586891434 | 1 | 0 |
| chr4 | 126877864 | T | C | 201 | Zmym4 | intronic | . | . | . | 4qD2.2 | rs580773775 | 1 | 0 |
| chr4 | 126882199 | T | C | 228 | Zmym4 | intronic | . | . | . | 4qD2.2 | rs27525273 | 1 | 0 |
| chr4 | 126882365 | T | A | 228 | Zmym4 | intronic | . | . | . | 4qD2.2 | rs244046381 | 1 | 0 |
| chr4 | 126882684 | A | C | 228 | Zmym4 | intronic | . | . | . | 4qD2.2 | rs230703804 | 1 | 0 |
| chr4 | 126889493 | G | C | 228 | Zmym4 | intronic | . | . | . | 4qD2.2 | rs221471946 | 1 | 0 |
| chr4 | 126889508 | C | T | 228 | Zmym4 | intronic | . | . | . | 4qD2.2 | rs27525262 | 1 | 0 |
| chr4 | 126889578 | C | T | 228 | Zmym4 | exonic | synonymous SNV | Zmym4:NM_001114399:exon23:c.G3468A:p.G1156G | . | 4qD2.2 | rs27525261 | 1 | 0 |
| chr4 | 126889721 | G | A | 188 | Zmym4 | intronic | . | . | . | 4qD2.2 | rs249163627 | 1 | 0 |
| chr4 | 126889758 | T | C | 206 | Zmym4 | intronic | . | . | . | 4qD2.2 | rs27525259 | 1 | 0 |
| chr4 | 126889804 | G | T | 174 | Zmym4 | intronic | . | . | . | 4qD2.2 | rs242325154 | 1 | 0 |
| chr4 | 126889958 | C | A | 111 | Zmym4 | intronic | . | . | . | 4qD2.2 | rs586694947 | 1 | 0 |
| chr4 | 126893473 | A | G | 228 | Zmym4 | intronic | . | . | . | 4qD2.2 | rs222684503 | 1 | 0 |

| | | | | | | | | | | | | | |
|---|---|---|---|---|---|---|---|---|---|---|---|---|---|
| chr4 | 126893483 | C | G | 203 | Zmym4 | intronic | . | . | . | 4qD2.2 | rs246835872 | 1 | 0 |
| chr4 | 126898046 | T | C | 148 | Zmym4 | intronic | . | . | . | 4qD2.2 | rs27525253 | 1 | 0 |
| chr4 | 126898292 | G | A | 228 | Zmym4 | splicing | . | . | . | 4qD2.2 | rs245515666 | 1 | 0 |
| chr4 | 126899804 | T | C | 164 | Zmym4 | intronic | . | . | . | 4qD2.2 | rs240764202 | 1 | 0 |
| chr4 | 126899897 | A | G | 228 | Zmym4 | intronic | . | . | . | 4qD2.2 | rs260198623 | 1 | 0 |
| chr4 | 126905493 | A | G | 219 | Zmym4 | intronic | . | . | . | 4qD2.2 | rs27525250 | 1 | 0 |
| chr4 | 126906265 | C | T | 228 | Zmym4 | intronic | . | . | . | 4qD2.2 | rs27525248 | 1 | 0 |
| chr4 | 126906386 | G | A | 228 | Zmym4 | intronic | . | . | . | 4qD2.2 | rs225709388 | 1 | 0 |
| chr4 | 126906715 | T | C | 228 | Zmym4 | intronic | . | . | . | 4qD2.2 | rs233727651 | 1 | 0 |
| chr4 | 126910796 | G | A | 228 | Zmym4 | exonic | synonymous SNV | Zmym4:NM_001114399:exon9:c.C1563T:p.Y521Y | . | 4qD2.2 | rs6250665 | 1 | 0 |
| chr4 | 126915755 | G | A | 212 | Zmym4 | intronic | . | . | . | 4qD2.2 | rs233775191 | 1 | 0 |
| chr4 | 126923301 | A | C | 223 | Zmym4 | intronic | . | . | . | 4qD2.2 | rs219683614 | 1 | 0 |
| chr4 | 126923738 | T | C | 140 | Zmym4 | intronic | . | . | . | 4qD2.2 | rs233486788 | 1 | 0 |
| chr4 | 126926073 | G | T | 160 | Zmym4 | intronic | . | . | . | 4qD2.2 | . | 1 | 0 |
| chr4 | 126948614 | T | A | 153 | Zmym4 | intronic | . | . | . | 4qD2.2 | rs245039883 | 1 | 0 |
| chr4 | 127022585 | G | A | 96 | Sfpq | intronic | . | . | . | 4qD2.2 | rs246546756 | 1 | 0 |
| chr4 | 127022593 | G | A | 129 | Sfpq | intronic | . | . | . | 4qD2.2 | rs217251103 | 1 | 0 |
| chr4 | 127022623 | G | A | 154 | Sfpq | intronic | . | . | . | 4qD2.2 | rs580491553 | 1 | 0 |
| chr4 | 127022731 | C | A | 228 | Sfpq | intronic | . | . | . | 4qD2.2 | rs248718902 | 1 | 0 |
| chr4 | 127023092 | A | G | 228 | Sfpq | intronic | . | . | . | 4qD2.2 | rs224608985 | 1 | 0 |
| chr4 | 127023125 | A | G | 228 | Sfpq | intronic | . | . | . | 4qD2.2 | rs48369476 | 1 | 0 |
| chr4 | 127023837 | A | T | 228 | Sfpq | intronic | . | . | . | 4qD2.2 | rs264919845 | 1 | 0 |
| chr4 | 127025729 | T | C | 180 | Sfpq | intronic | . | . | . | 4qD2.2 | rs244358028 | 1 | 0 |
| chr4 | 127025773 | A | C | 221 | Sfpq | intronic | . | . | . | 4qD2.2 | rs227667465 | 1 | 0 |
| chr4 | 127026090 | A | G | 228 | Sfpq | intronic | . | . | . | 4qD2.2 | rs46626755 | 1 | 0 |
| chr4 | 127026682 | A | T | 228 | Sfpq | intronic | . | . | . | 4qD2.2 | rs230220630 | 1 | 0 |
| chr4 | 127026990 | G | A | 228 | Sfpq | intronic | . | . | . | 4qD2.2 | rs258860091 | 1 | 0 |
| chr4 | 127047666 | G | A | 228 | Zmym1 | exonic | synonymous SNV | Zmym1:NM_026670:exon8:c.C2928T:p.F976F | . | 4qD2.2 | rs27581317 | 1 | 0 |
| chr4 | 127047898 | G | C | 228 | Zmym1 | exonic | missense SNV | Zmym1:NM_026670:exon8:c.C2696G:p.A899G | . | 4qD2.2 | rs27581316 | 1 | 0 |
| chr4 | 127047947 | C | T | 228 | Zmym1 | exonic | missense SNV | Zmym1:NM_026670:exon8:c.G2647A:p.D883N | . | 4qD2.2 | rs27581315 | 1 | 0 |
| chr4 | 127048230 | A | G | 228 | Zmym1 | exonic | synonymous SNV | Zmym1:NM_026670:exon8:c.T2364C:p.P788P | . | 4qD2.2 | rs250159364 | 1 | 0 |
| chr4 | 127048250 | C | T | 225 | Zmym1 | exonic | missense SNV | Zmym1:NM_026670:exon8:c.G2344A:p.V782I | . | 4qD2.2 | rs220481434 | 1 | 0 |
| chr4 | 127048304 | T | G | 153 | Zmym1 | exonic | missense SNV | Zmym1:NM_026670:exon8:c.A2290C:p.I764L | . | 4qD2.2 | rs239597854 | 1 | 0 |
| chr4 | 127048539 | G | A | 228 | Zmym1 | exonic | synonymous SNV | Zmym1:NM_026670:exon8:c.C2055T:p.A685A | . | 4qD2.2 | rs247793628 | 1 | 0 |
| chr4 | 127048566 | G | T | 228 | Zmym1 | exonic | synonymous SNV | Zmym1:NM_026670:exon8:c.C2028A:p.R676R | . | 4qD2.2 | rs251686288 | 1 | 0 |
| chr4 | 127048590 | A | G | 228 | Zmym1 | exonic | synonymous SNV | Zmym1:NM_026670:exon8:c.T2004C:p.V668V | . | 4qD2.2 | rs27581313 | 1 | 0 |
| chr4 | 127048903 | G | C | 228 | Zmym1 | exonic | missense SNV | Zmym1:NM_026670:exon8:c.C1691G:p.T564R | . | 4qD2.2 | rs258386259 | 1 | 0 |
| chr4 | 127048968 | C | T | 228 | Zmym1 | exonic | synonymous SNV | Zmym1:NM_026670:exon8:c.G1626A:p.L542L | . | 4qD2.2 | rs27581312 | 1 | 0 |
| chr4 | 127049673 | A | C | 228 | Zmym1 | exonic | missense SNV | Zmym1:NM_026670:exon8:c.T921G:p.H307Q | . | 4qD2.2 | rs214871576 | 1 | 0 |
| chr4 | 127049683 | G | C | 228 | Zmym1 | exonic | missense SNV | Zmym1:NM_026670:exon8:c.C911G:p.A304G | . | 4qD2.2 | rs259944744 | 1 | 0 |
| chr4 | 127051095 | T | C | 222 | Zmym1 | intronic | . | . | . | 4qD2.2 | rs233395456 | 1 | 0 |
| chr4 | 127051452 | G | A | 228 | Zmym1 | intronic | . | . | . | 4qD2.2 | rs215083838 | 1 | 0 |
| chr4 | 127051650 | C | T | 115 | Zmym1 | intronic | . | . | . | 4qD2.2 | rs218690038 | 1 | 0 |
| chr4 | 127056581 | C | T | 228 | Zmym1 | intronic | . | . | . | 4qD2.2 | rs239769117 | 1 | 0 |
| chr4 | 127056754 | A | G | 228 | Zmym1 | intronic | . | . | . | 4qD2.2 | rs259329566 | 1 | 0 |
| chr4 | 127078379 | A | G | 228 | Zmym6 | UTR5 | . | . | . | 4qD2.2 | rs249911609 | 1 | 0 |
| chr4 | 127078610 | C | T | 228 | Zmym6 | intronic | . | . | . | 4qD2.2 | rs238347173 | 1 | 0 |
| chr4 | 127086901 | C | T | 228 | Zmym6 | intronic | . | . | . | 4qD2.2 | rs226618404 | 1 | 0 |
| chr4 | 127086939 | A | G | 228 | Zmym6 | intronic | . | . | . | 4qD2.2 | rs212376526 | 1 | 0 |
| chr4 | 127087126 | G | C | 228 | Zmym6 | intronic | . | . | . | 4qD2.2 | rs234262155 | 1 | 0 |
| chr4 | 127087198 | C | T | 166 | Zmym6 | intronic | . | . | . | 4qD2.2 | rs222872910 | 1 | 0 |
| chr4 | 127088272 | G | A | 228 | Zmym6 | intronic | . | . | . | 4qD2.2 | rs255676968 | 1 | 0 |
| chr4 | 127088458 | T | C | 228 | Zmym6 | exonic | missense SNV | Zmym6:NM_001285885:exon3:c.T287C:p.F96S,Zmym6:NM_001285887:exon4:c.T287C:p.F96S,Zmym6:NM_1774 | . | 4qD2.2 | rs214771877 | 1 | 0 |
| chr4 | 127092789 | T | C | 170 | Zmym6 | intronic | . | . | . | 4qD2.2 | rs228124759 | 1 | 0 |
| chr4 | 127092854 | C | T | 208 | Zmym6 | intronic | . | . | . | 4qD2.2 | rs27581290 | 1 | 0 |
| chr4 | 127093002 | T | A | 228 | Zmym6 | exonic | synonymous SNV | Zmym6:NM_001285885:exon4:c.T474A:p.S158S,Zmym6:NM_001285887:exon5:c.T474A:p.S158S,Zmym6:NM_0( | . | 4qD2.2 | rs27581288 | 1 | 0 |
| chr4 | 127093133 | A | G | 228 | Zmym6 | intronic | . | . | . | 4qD2.2 | rs27581287 | 1 | 0 |
| chr4 | 127102012 | A | G | 228 | Zmym6 | intronic | . | . | . | 4qD2.2 | rs238500554 | 1 | 0 |
| chr4 | 127105566 | T | G | 176 | Zmym6 | intronic | . | . | . | 4qD2.2 | rs230926486 | 1 | 0 |
| chr4 | 127106662 | G | A | 228 | Zmym6 | exonic | synonymous SNV | Zmym6:NM_001285885:exon10:c.G1509A:p.L503L,Zmym6:NM_001285887:exon10:c.G1233A:p.L411L,Zmym6:N | . | 4qD2.2 | rs27565956 | 1 | 0 |
| chr4 | 127108699 | G | T | 228 | Zmym6 | exonic | synonymous SNV | Zmym6:NM_001285885:exon11:c.G1722T:p.T574T,Zmym6:NM_001285887:exon11:c.G1446T:p.T482T,Zmym6:N | . | 4qD2.2 | rs6377633 | 1 | 0 |
| chr4 | 127109892 | A | G | 228 | Zmym6 | intronic | . | . | . | 4qD2.2 | rs27565942 | 1 | 0 |
| chr4 | 127120541 | G | A | 228 | Zmym6 | exonic | synonymous SNV | Zmym6:NM_001285885:exon13:c.G1890A:p.R630R,Zmym6:NM_001285887:exon13:c.G1614A:p.R538R,Zmym6:N | . | 4qD2.2 | rs27565891 | 1 | 0 |
| chr4 | 127123949 | A | G | 228 | Zmym6 | exonic | synonymous SNV | Zmym6:NM_001285885:exon14:c.A3522G:p.S1174S,Zmym6:NM_001285887:exon14:c.A3246G:p.S1082S,Zmym6 | . | 4qD2.2 | rs27565884 | 1 | 0 |
| chr4 | 127123961 | A | G | 228 | Zmym6 | exonic | synonymous SNV | Zmym6:NM_001285885:exon14:c.A3534G:p.T1178T,Zmym6:NM_001285887:exon14:c.A3258G:p.T1086T,Zmym6 | . | 4qD2.2 | rs27565883 | 1 | 0 |
| chr4 | 127127763 | T | G | 228 | Gm12942 | intronic | . | . | . | 4qD2.2 | rs219584043 | 1 | 0 |
| chr4 | 127127784 | A | G | 228 | Gm12942 | intronic | . | . | . | 4qD2.2 | rs27565859 | 1 | 0 |
| chr4 | 127128047 | G | T | 228 | Gm12942 | intronic | . | . | . | 4qD2.2 | rs228013308 | 1 | 0 |
| chr4 | 127129029 | A | G | 228 | Gm12942 | UTR3 | . | . | . | 4qD2.2 | rs27565847 | 1 | 0 |
| chr4 | 127215050 | G | A | 228 | Dlgap3 | intronic | . | . | . | 4qD2.2 | rs27550940 | 1 | 0 |
| chr4 | 127215411 | C | T | 228 | Dlgap3 | intronic | . | . | . | 4qD2.2 | rs27550937 | 1 | 0 |
| chr4 | 127233431 | C | T | 127 | Dlgap3 | intronic | . | . | . | 4qD2.2 | rs27550862 | 1 | 0 |
| chr4 | 127233513 | T | C | 228 | Dlgap3 | intronic | . | . | . | 4qD2.2 | rs234848145 | 1 | 0 |
| chr4 | 127233516 | T | C | 228 | Dlgap3 | intronic | . | . | . | 4qD2.2 | rs27550861 | 1 | 0 |
| chr4 | 127233607 | C | T | 228 | Dlgap3 | exonic | synonymous SNV | Dlgap3:NM_001302081:exon9:c.C2067T:p.A689A,Dlgap3:NM_198618:exon9:c.C2067T:p.A689A | . | 4qD2.2 | rs216856826 | 1 | 0 |
| chr4 | 127233733 | A | G | 228 | Dlgap3 | exonic | synonymous SNV | Dlgap3:NM_001302081:exon9:c.A2193G:p.T731T,Dlgap3:NM_198618:exon9:c.A2193G:p.T731T | 272 | 4qD2.2 | rs27550859 | 1 | 0 |
| chr4 | 127233823 | C | T | 179 | Dlgap3 | exonic | synonymous SNV | Dlgap3:NM_001302081:exon9:c.C2283T:p.A761A,Dlgap3:NM_198618:exon9:c.C2283T:p.A761A | 272 | 4qD2.2 | rs265646613 | 1 | 0 |
| chr4 | 127234031 | G | A | 228 | Dlgap3 | intronic | . | . | . | 4qD2.2 | rs27550858 | 1 | 0 |
| chr4 | 127234820 | A | C | 200 | Dlgap3 | intronic | . | . | . | 4qD2.2 | rs27550849 | 1 | 0 |
| chr4 | 127234833 | T | G | 201 | Dlgap3 | intronic | . | . | . | 4qD2.2 | rs221299419 | 1 | 0 |
| chr4 | 127235076 | A | G | 228 | Dlgap3 | intronic | . | . | . | 4qD2.2 | rs238976423 | 1 | 0 |
| chr4 | 127235109 | C | T | 228 | Dlgap3 | intronic | . | . | . | 4qD2.2 | rs27550848 | 1 | 0 |
| chr4 | 127235336 | A | G | 175 | Dlgap3 | intronic | . | . | . | 4qD2.2 | rs27550844 | 1 | 0 |
| chr4 | 127246865 | A | G | 190 | Smim12 | intronic | . | . | . | 4qD2.2 | rs27550821 | 1 | 0 |
| chr4 | 127247483 | G | C | 228 | Smim12 | UTR3 | . | . | . | 4qD2.2 | rs45704816 | 1 | 0 |
| chr4 | 127247541 | G | A | 228 | Smim12 | UTR3 | . | . | . | 4qD2.2 | rs49934705 | 1 | 0 |
| chr4 | 127247873 | A | G | 228 | . | downstream | . | . | . | 4qD2.2 | rs27550813 | 1 | 0 |
| chr4 | 127311839 | C | T | 171 | Gja4 | UTR3 | . | . | . | 4qD2.2 | rs27528973 | 1 | 0 |
| chr4 | 127312231 | G | C | 228 | Gja4 | exonic | missense SNV | Gja4:NM_008120:exon2:c.C738G:p.H246Q | . | 4qD2.2 | rs387599551 | 1 | 0 |
| chr4 | 127312399 | C | T | 228 | Gja4 | exonic | synonymous SNV | Gja4:NM_008120:exon2:c.G570A:p.A190A | . | 4qD2.2 | rs231013816 | 1 | 0 |
| chr4 | 127325855 | A | G | 166 | Gjb3 | UTR3 | . | . | . | 4qD2.2 | rs220971333 | 1 | 0 |
| chr4 | 127325861 | T | C | 183 | Gjb3 | UTR3 | . | . | . | 4qD2.2 | rs262328576 | 1 | 0 |
| chr4 | 127325908 | C | T | 228 | Gjb3 | UTR3 | . | . | . | 4qD2.2 | rs27528932 | 1 | 0 |
| chr4 | 127326192 | C | A | 228 | Gjb3 | exonic | synonymous SNV | Gjb3:NM_001160012:exon2:c.G546T:p.T182T,Gjb3:NM_008126:exon2:c.G546T:p.T182T | 298 | 4qD2.2 | rs27528931 | 1 | 0 |
| chr4 | 127326291 | G | T | 228 | Gjb3 | exonic | synonymous SNV | Gjb3:NM_001160012:exon2:c.C447A:p.L149L,Gjb3:NM_008126:exon2:c.C447A:p.L149L | 298 | 4qD2.2 | rs27528930 | 1 | 0 |
| chr4 | 127326408 | C | T | 228 | Gjb3 | exonic | synonymous SNV | Gjb3:NM_001160012:exon2:c.G330A:p.G110G,Gjb3:NM_008126:exon2:c.G330A:p.G110G | 298 | 4qD2.2 | . | 1 | 0 |
| chr4 | 127326630 | A | G | 228 | Gjb3 | exonic | synonymous SNV | Gjb3:NM_001160012:exon2:c.T108C:p.Y36Y,Gjb3:NM_008126:exon2:c.T108C:p.Y36Y | . | 4qD2.2 | rs265078695 | 1 | 0 |
| chr4 | 127351298 | T | A | 228 | Gjb4 | UTR3 | . | . | . | 4qD2.2 | rs248437856 | 1 | 0 |
| chr4 | 127351335 | A | C | 228 | Gjb4 | UTR3 | . | . | . | 4qD2.2 | rs27528829 | 1 | 0 |
| chr4 | 127351592 | G | A | 228 | Gjb4 | exonic | synonymous SNV | Gjb4:NM_008127:exon2:c.C555T:p.V185V | . | 4qD2.2 | rs243195204 | 1 | 0 |
| chr4 | 127351706 | G | A | 228 | Gjb4 | exonic | synonymous SNV | Gjb4:NM_008127:exon2:c.C441T:p.Y147Y | . | 4qD2.2 | rs27528826 | 1 | 0 |
| chr4 | 127356020 | A | G | 228 | Gjb5 | exonic | synonymous SNV | Gjb5:NM_010291:exon2:c.T330C:p.G110G | . | 4qD2.2 | rs27528810 | 1 | 0 |
| chr4 | 127356025 | T | C | 228 | Gjb5 | exonic | missense SNV | Gjb5:NM_010291:exon2:c.A325G:p.I109V | . | 4qD2.2 | rs213303945 | 1 | 0 |
| chr4 | 127356134 | A | G | 228 | Gjb5 | exonic | synonymous SNV | Gjb5:NM_010291:exon2:c.T216C:p.S72S | . | 4qD2.2 | rs230307904 | 1 | 0 |
| chr4 | 127356317 | G | A | 228 | Gjb5 | exonic | synonymous SNV | Gjb5:NM_010291:exon2:c.C33T:p.S11S | . | 4qD2.2 | rs27528809 | 1 | 0 |
| chr4 | 128113787 | C | T | 228 | Csmd2 | intronic | . | . | . | 4qD2.2 | rs28274331 | 1 | 0 |
| chr4 | 128113805 | C | T | 228 | Csmd2 | intronic | . | . | . | 4qD2.2 | rs28274330 | 1 | 0 |
| chr4 | 128209593 | T | C | 205 | Csmd2 | intronic | . | . | . | 4qD2.2 | rs27529527 | 1 | 0 |
| chr4 | 128209661 | A | G | 228 | Csmd2 | intronic | . | . | . | 4qD2.2 | rs27529526 | 1 | 0 |
| chr4 | 128209943 | G | C | 228 | Csmd2 | intronic | . | . | . | 4qD2.2 | rs50821347 | 1 | 0 |
| chr4 | 128210102 | T | A | 95 | Csmd2 | intronic | . | . | . | 4qD2.2 | rs27529524 | 1 | 0 |
| chr4 | 128297070 | G | A | 228 | Csmd2 | intronic | . | . | . | 4qD2.2 | rs27496038 | 1 | 0 |
| chr4 | 128306433 | T | C | 228 | Csmd2 | exonic | synonymous SNV | Csmd2:NM_001281955:exon10:c.T1410C:p.Y470Y | . | 4qD2.2 | rs46298059 | 1 | 0 |
| chr4 | 128306520 | C | T | 228 | Csmd2 | intronic | . | . | . | 4qD2.2 | rs27495966 | 1 | 0 |
| chr4 | 128306606 | A | G | 228 | Csmd2 | intronic | . | . | . | 4qD2.2 | rs6402892 | 1 | 0 |
| chr4 | 128306659 | G | A | 165 | Csmd2 | intronic | . | . | . | 4qD2.2 | rs6403395 | 1 | 0 |
| chr4 | 128321134 | A | G | 170 | Csmd2 | intronic | . | . | . | 4qD2.2 | rs32698638 | 1 | 0 |
| chr4 | 128321174 | T | G | 217 | Csmd2 | intronic | . | . | . | 4qD2.2 | rs32303453 | 1 | 0 |
| chr4 | 128321397 | G | A | 228 | Csmd2 | intronic | . | . | . | 4qD2.2 | rs27495816 | 1 | 0 |
| chr4 | 128321834 | T | G | 115 | Csmd2 | intronic | . | . | . | 4qD2.2 | rs49120866 | 1 | 0 |
| chr4 | 128321933 | C | T | 228 | Csmd2 | intronic | . | . | . | 4qD2.2 | rs27495809 | 1 | 0 |
| chr4 | 128322058 | A | G | 228 | Csmd2 | exonic | synonymous SNV | Csmd2:NM_001281955:exon12:c.A1605G:p.T535T | . | 4qD2.2 | rs27495806 | 1 | 0 |
| chr4 | 128338341 | G | A | 166 | Csmd2 | intronic | . | . | . | 4qD2.2 | rs27573770 | 1 | 0 |
| chr4 | 128338344 | C | T | 167 | Csmd2 | intronic | . | . | . | 4qD2.2 | rs32607972 | 1 | 0 |
| chr4 | 128338797 | T | C | 228 | Csmd2 | intronic | . | . | . | 4qD2.2 | rs27573769 | 1 | 0 |
| chr4 | 128338842 | A | G | 188 | Csmd2 | intronic | . | . | . | 4qD2.2 | rs27573768 | 1 | 0 |
| chr4 | 128372798 | T | C | 127 | Csmd2 | intronic | . | . | . | 4qD2.2 | rs27558953 | 1 | 0 |
| chr4 | 128382778 | G | A | 173 | Csmd2 | intronic | . | . | . | 4qD2.2 | rs27558890 | 1 | 0 |
| chr4 | 128383971 | A | G | 228 | Csmd2 | exonic | missense SNV | Csmd2:NM_001281955:exon18:c.A2879G:p.H960R | . | 4qD2.2 | rs32631491 | 1 | 0 |
| chr4 | 128384085 | T | A | 228 | Csmd2 | intronic | . | . | . | 4qD2.2 | rs32557294 | 1 | 0 |
| chr4 | 128384405 | G | A | 228 | Csmd2 | intronic | . | . | . | 4qD2.2 | rs32142360 | 1 | 0 |
| chr4 | 128393698 | C | A | 222 | Csmd2 | exonic | synonymous SNV | Csmd2:NM_001281955:exon20:c.C3225A:p.T1075T | . | 4qD2.2 | . | 0 | 1 |
| chr4 | 128394796 | C | A | 115 | Csmd2 | intronic | . | . | . | 4qD2.2 | rs27558810 | 1 | 0 |
| chr4 | 128412477 | A | G | 164 | Csmd2 | intronic | . | . | . | 4qD2.2 | rs27543658 | 1 | 0 |
| chr4 | 128414326 | T | C | 228 | Csmd2 | exonic | synonymous SNV | Csmd2:NM_001281955:exon24:c.T3876C:p.S1292S | . | 4qD2.2 | rs27543636 | 1 | 0 |
| chr4 | 128442884 | T | C | 228 | Csmd2 | exonic | synonymous SNV | Csmd2:NM_001281955:exon26:c.T4254C:p.F1418F | . | 4qD2.2 | rs50003509 | 1 | 0 |
| chr4 | 128449212 | G | A | 228 | Csmd2 | exonic | missense SNV | Csmd2:NM_001281955:exon27:c.G4396A:p.A1466T | . | 4qD2.2 | rs247940438 | 1 | 0 |
| chr4 | 128449303 | C | T | 228 | Csmd2 | intronic | . | . | . | 4qD2.2 | rs45813482 | 1 | 0 |
| chr4 | 128452705 | A | G | 209 | Csmd2 | intronic | . | . | . | 4qD2.2 | rs32015853 | 1 | 0 |
| chr4 | 128462630 | C | T | 85 | Csmd2 | intronic | . | . | . | 4qD2.2 | rs217481399 | 1 | 0 |
| chr4 | 128463554 | T | C | 180 | Csmd2 | intronic | . | . | . | 4qD2.2 | rs27519641 | 1 | 0 |
| chr4 | 128475964 | G | C | 80 | Csmd2 | intronic | . | . | . | 4qD2.2 | . | 0 | 1 |
| chr4 | 128477362 | A | G | 228 | Csmd2 | exonic | synonymous SNV | Csmd2:NM_001281955:exon37:c.A5742G:p.T1914T | . | 4qD2.2 | rs27519484 | 1 | 0 |
| chr4 | 128480043 | A | G | 228 | Csmd2 | intronic | . | . | . | 4qD2.2 | rs27575635 | 1 | 0 |
| chr4 | 128480183 | C | T | 228 | Csmd2 | exonic | synonymous SNV | Csmd2:NM_001281955:exon38:c.C5919T:p.N1973N | . | 4qD2.2 | rs27575633 | 1 | 0 |
| chr4 | 128480256 | G | A | 228 | Csmd2 | intronic | . | . | . | 4qD2.2 | rs27575632 | 1 | 0 |
| chr4 | 128480262 | G | A | 228 | Csmd2 | intronic | . | . | . | 4qD2.2 | rs27575631 | 1 | 0 |
| chr4 | 128480608 | C | T | 138 | Csmd2 | intronic | . | . | . | 4qD2.2 | rs27575625 | 1 | 0 |
| chr4 | 128480646 | T | C | 160 | Csmd2 | intronic | . | . | . | 4qD2.2 | rs27575624 | 1 | 0 |
| chr4 | 128480763 | T | C | 228 | Csmd2 | intronic | . | . | . | 4qD2.2 | rs27575622 | 1 | 0 |
| chr4 | 128480790 | C | T | 228 | Csmd2 | exonic | synonymous SNV | Csmd2:NM_001281955:exon39:c.C5982T:p.C1994C | . | 4qD2.2 | rs27575621 | 1 | 0 |
| chr4 | 128483595 | G | A | 227 | Csmd2 | intronic | . | . | . | 4qD2.2 | rs27575601 | 1 | 0 |
| chr4 | 128483630 | T | C | 152 | Csmd2 | intronic | . | . | . | 4qD2.2 | rs32156245 | 1 | 0 |
| chr4 | 128487049 | T | C | 228 | Csmd2 | intronic | . | . | . | 4qD2.2 | rs32418747 | 1 | 0 |
| chr4 | 128487065 | T | C | 228 | Csmd2 | intronic | . | . | . | 4qD2.2 | rs32049534 | 1 | 0 |
| chr4 | 128493526 | G | T | 228 | Csmd2 | intronic | . | . | . | 4qD2.2 | rs27561126 | 1 | 0 |
| chr4 | 128493542 | C | T | 228 | Csmd2 | intronic | . | . | . | 4qD2.2 | rs47232776 | 1 | 0 |
| chr4 | 128493544 | A | C | 228 | Csmd2 | intronic | . | . | . | 4qD2.2 | rs49815195 | 1 | 0 |
| chr4 | 128508554 | T | C | 228 | Csmd2 | intronic | . | . | . | 4qD2.2 | rs50021886 | 1 | 0 |
| chr4 | 128508655 | T | C | 228 | Csmd2 | intronic | . | . | . | 4qD2.2 | rs46999536 | 1 | 0 |
| chr4 | 128508964 | T | G | 206 | Csmd2 | intronic | . | . | . | 4qD2.2 | rs51964521 | 1 | 0 |
| chr4 | 128510772 | A | G | 169 | Csmd2 | intronic | . | . | . | 4qD2.2 | rs27561054 | 1 | 0 |
| chr4 | 128510905 | T | C | 228 | Csmd2 | exonic | synonymous SNV | Csmd2:NM_001281955:exon47:c.T7083C:p.N2361N | . | 4qD2.2 | rs27561053 | 1 | 0 |
| chr4 | 128513518 | G | C | 228 | Csmd2 | exonic | synonymous SNV | Csmd2:NM_001281955:exon48:c.G7395C:p.R2465R | . | 4qD2.2 | rs27561022 | 1 | 0 |
| chr4 | 128513555 | A | G | 228 | Csmd2 | intronic | . | . | . | 4qD2.2 | rs27561021 | 1 | 0 |
| chr4 | 128513623 | C | A | 228 | Csmd2 | intronic | . | . | . | 4qD2.2 | rs50507431 | 1 | 0 |
| chr4 | 128517681 | A | C | 228 | Csmd2 | exonic | synonymous SNV | Csmd2:NM_001281955:exon49:c.A7491C:p.G2497G | . | 4qD2.2 | rs27560996 | 1 | 0 |
| chr4 | 128517738 | G | A | 228 | Csmd2 | exonic | synonymous SNV | Csmd2:NM_001281955:exon49:c.G7548A:p.P2516P | . | 4qD2.2 | rs27560995 | 1 | 0 |
| chr4 | 128528468 | C | T | 126 | Csmd2 | intronic | . | . | . | 4qD2.2 | rs27560904 | 1 | 0 |
| chr4 | 128530731 | A | G | 228 | Csmd2 | exonic | missense SNV | Csmd2:NM_001281955:exon55:c.A8548G:p.T2850A | . | 4qD2.2 | rs27560887 | 1 | 0 |
| chr4 | 128534136 | T | C | 228 | Csmd2 | intronic | . | . | . | 4qD2.2 | rs27560870 | 1 | 0 |
| chr4 | 128539109 | C | T | 228 | Csmd2 | exonic | synonymous SNV | Csmd2:NM_001281955:exon57:c.C8958T:p.D2986D | . | 4qD2.2 | rs27546415 | 1 | 0 |
| chr4 | 128539118 | C | T | 228 | Csmd2 | exonic | synonymous SNV | Csmd2:NM_001281955:exon57:c.C8967T:p.H2989H | . | 4qD2.2 | rs266082692 | 1 | 0 |
| chr4 | 128544812 | T | C | 228 | Csmd2 | exonic | synonymous SNV | Csmd2:NM_001281955:exon58:c.T9183C:p.N3061N | . | 4qD2.2 | rs27546375 | 1 | 0 |
| chr4 | 128548927 | T | A | 228 | Csmd2 | intronic | . | . | . | 4qD2.2 | rs262151795 | 1 | 0 |
| chr4 | 128549273 | T | C | 228 | Csmd2 | exonic | synonymous SNV | Csmd2:NM_001281955:exon62:c.T9816C:p.Y3272Y | . | 4qD2.2 | rs27546340 | 1 | 0 |
| chr4 | 128549353 | G | A | 126 | Csmd2 | intronic | . | . | . | 4qD2.2 | rs27546337 | 1 | 0 |
| chr4 | 128551918 | G | A | 228 | Csmd2 | intronic | . | . | . | 4qD2.2 | rs27546302 | 1 | 0 |
| chr4 | 128552108 | A | G | 228 | Csmd2 | intronic | . | . | . | 4qD2.2 | rs32771868 | 1 | 0 |
| chr4 | 128554902 | A | C | 228 | Csmd2 | exonic | missense SNV | Csmd2:NM_001281955:exon65:c.A10156C:p.M3386L | . | 4qD2.2 | rs27546280 | 1 | 0 |
| chr4 | 128554969 | T | C | 228 | Csmd2 | intronic | . | . | . | 4qD2.2 | rs27546279 | 1 | 0 |
| chr4 | 128557714 | G | A | 228 | Csmd2 | exonic | missense SNV | Csmd2:NM_001281955:exon66:c.G10264A:p.G3422S | . | 4qD2.2 | rs27546266 | 1 | 0 |
| chr4 | 128563102 | C | T | 174 | Csmd2 | intronic | . | . | . | 4qD2.2 | rs233653641 | 1 | 0 |
| chr4 | 128585774 | T | C | 228 | Zscan20 | exonic | missense SNV | Zscan20:NM_177758:exon8:c.A2923G:p.K975E | 430 | 4qD2.2 | rs27546176 | 1 | 0 |
| chr4 | 128586064 | G | A | 228 | Zscan20 | exonic | missense SNV | Zscan20:NM_177758:exon8:c.C2633T:p.T878M | . | 4qD2.2 | rs27546169 | 1 | 0 |

| | | | | | | | | | | | | | |
|---|---|---|---|---|---|---|---|---|---|---|---|---|---|
| chr4 | 128589742 | T | C | 101 | Zscan20 | exonic | missense SNV | Zscan20:NM_177758:exon6:c.A1133G:p.N378S | . | 4qD2.2 | rs27520948 | 1 | 0 |
| chr4 | 128591311 | A | T | 156 | Zscan20 | intronic | . | . | . | 4qD2.2 | rs27520932 | 1 | 0 |
| chr4 | 128591388 | A | G | 228 | Zscan20 | intronic | . | . | . | 4qD2.2 | rs27520931 | 1 | 0 |
| chr4 | 128592470 | C | T | 219 | Zscan20 | exonic | missense SNV | Zscan20:NM_177758:exon3:c.G436A:p.E146K | . | 4qD2.2 | . | 0 | 1 |
| chr4 | 128604157 | T | C | 228 | Zscan20 | intronic | . | . | . | 4qD2.2 | rs27520900 | 1 | 0 |
| chr4 | 128604448 | T | C | 228 | Zscan20 | exonic | synonymous SNV | Zscan20:NM_177758:exon2:c.A156G:p.R52R | . | 4qD2.2 | rs27520897 | 1 | 0 |
| chr4 | 128616067 | G | C | 228 | Tlr12 | exonic | missense SNV | Tlr12:NM_205823:exon1:c.C2389G:p.L797V | . | 4qD2.2 | rs27520825 | 1 | 0 |
| chr4 | 128616629 | A | G | 228 | Tlr12 | exonic | synonymous SNV | Tlr12:NM_205823:exon1:c.T1827C:p.A609A | . | 4qD2.2 | rs27520820 | 1 | 0 |
| chr4 | 128616854 | A | G | 228 | Tlr12 | exonic | synonymous SNV | Tlr12:NM_205823:exon1:c.T1602C:p.L534L | . | 4qD2.2 | rs27520819 | 1 | 0 |
| chr4 | 128616899 | A | G | 228 | Tlr12 | exonic | synonymous SNV | Tlr12:NM_205823:exon1:c.T1557C:p.L519L | . | 4qD2.2 | rs27520818 | 1 | 0 |
| chr4 | 128617358 | G | T | 228 | Tlr12 | exonic | synonymous SNV | Tlr12:NM_205823:exon1:c.C1098A:p.P366P | . | 4qD2.2 | rs224224557 | 1 | 0 |
| chr4 | 128617490 | C | G | 228 | Tlr12 | exonic | missense SNV | Tlr12:NM_205823:exon1:c.G966C:p.E322D | . | 4qD2.2 | rs27520813 | 1 | 0 |
| chr4 | 128617743 | A | G | 228 | Tlr12 | exonic | missense SNV | Tlr12:NM_205823:exon1:c.T713C:p.V238A | . | 4qD2.2 | rs27520812 | 1 | 0 |
| chr4 | 128617828 | A | G | 228 | Tlr12 | exonic | missense SNV | Tlr12:NM_205823:exon1:c.T628C:p.W210R | . | 4qD2.2 | rs27520810 | 1 | 0 |
| chr4 | 128617921 | C | T | 228 | Tlr12 | exonic | missense SNV | Tlr12:NM_205823:exon1:c.G535A:p.V179M | . | 4qD2.2 | rs27520809 | 1 | 0 |
| chr4 | 128617979 | G | A | 228 | Tlr12 | exonic | synonymous SNV | Tlr12:NM_205823:exon1:c.C477T:p.G159G | . | 4qD2.2 | rs216467641 | 1 | 0 |
| chr4 | 128618070 | C | T | 228 | Tlr12 | exonic | missense SNV | Tlr12:NM_205823:exon1:c.G386A:p.R129K | . | 4qD2.2 | rs262169527 | 1 | 0 |
| chr4 | 128618201 | T | C | 228 | Tlr12 | exonic | synonymous SNV | Tlr12:NM_205823:exon1:c.A255G:p.P85P | . | 4qD2.2 | rs27520808 | 1 | 0 |
| chr4 | 128618321 | A | G | 228 | Tlr12 | exonic | synonymous SNV | Tlr12:NM_205823:exon1:c.T135C:p.H45H | . | 4qD2.2 | rs27520807 | 1 | 0 |
| chr4 | 128618582 | C | T | 228 | Tlr12 | UTR5 | . | . | . | 4qD2.2 | rs27520802 | 1 | 0 |
| chr4 | 128618717 | T | C | 181 | . | upstream | . | . | . | 4qD2.2 | rs216346854 | 1 | 0 |
| chr4 | 128704985 | T | C | 228 | Phc2 | intronic | . | . | . | 4qD2.2 | rs248683471 | 1 | 0 |
| chr4 | 128707852 | A | G | 91 | Phc2 | intronic | . | . | . | 4qD2.2 | rs225275853 | 1 | 0 |
| chr4 | 128707882 | G | A | 159 | Phc2 | intronic | . | . | . | 4qD2.2 | rs242729633 | 1 | 0 |
| chr4 | 128707978 | G | A | 228 | Phc2 | exonic | synonymous SNV | Phc2:NM_001195130:exon3:c.G225A:p.A75A,Phc2:NM_018774:exon3:c.G225A:p.A75A | . | 4qD2.2 | rs211767098 | 1 | 0 |
| chr4 | 128708105 | G | A | 228 | Phc2 | intronic | . | . | . | 4qD2.2 | rs233586373 | 1 | 0 |
| chr4 | 128709573 | G | A | 228 | Phc2 | exonic | synonymous SNV | Phc2:NM_001195130:exon5:c.G495A:p.S165S,Phc2:NM_018774:exon5:c.G495A:p.S165S | . | 4qD2.2 | rs236646350 | 1 | 0 |
| chr4 | 128709676 | G | A | 228 | Phc2 | intronic | . | . | . | 4qD2.2 | rs234585096 | 1 | 0 |
| chr4 | 128709743 | A | G | 179 | Phc2 | intronic | . | . | . | 4qD2.2 | rs253674864 | 1 | 0 |
| chr4 | 128709814 | A | C | 124 | Phc2 | intronic | . | . | . | 4qD2.2 | rs216848497 | 1 | 0 |
| chr4 | 128712408 | C | A | 228 | Phc2 | intronic | . | . | . | 4qD2.2 | rs260335957 | 1 | 0 |
| chr4 | 128712423 | G | C | 228 | Phc2 | intronic | . | . | . | 4qD2.2 | rs225384815 | 1 | 0 |
| chr4 | 128723756 | G | A | 228 | Phc2 | intronic | . | . | . | 4qD2.2 | rs220970954 | 1 | 0 |
| chr4 | 128723760 | C | T | 228 | Phc2 | intronic | . | . | . | 4qD2.2 | rs27558273 | 1 | 0 |
| chr4 | 128745571 | G | A | 124 | Phc2 | intronic | . | . | . | 4qD2.2 | rs230557994 | 1 | 0 |
| chr4 | 128745589 | C | T | 132 | Phc2 | intronic | . | . | . | 4qD2.2 | rs27558237 | 1 | 0 |
| chr4 | 128745990 | G | C | 172 | Phc2 | intronic | . | . | . | 4qD2.2 | rs27558235 | 1 | 0 |
| chr4 | 128748452 | A | C | 163 | Phc2 | intronic | . | . | . | 4qD2.2 | rs32554898 | 1 | 0 |
| chr4 | 128751286 | G | A | 157 | Phc2 | intronic | . | . | . | 4qD2.2 | rs27558220 | 1 | 0 |
| chr4 | 128751857 | G | A | 130 | Phc2 | UTR3 | . | . | . | 4qD2.2 | rs261725742 | 1 | 0 |
| chr4 | 128755601 | T | C | 228 | . | intergenic | . | . | . | 4qD2.2 | rs237718542 | 1 | 0 |
| chr4 | 128755606 | T | C | 228 | . | intergenic | . | . | . | 4qD2.2 | rs27558207 | 1 | 0 |
| chr4 | 128760104 | T | C | 228 | A3galt2 | intronic | . | . | . | 4qD2.2 | rs27558183 | 1 | 0 |
| chr4 | 128761351 | A | G | 228 | A3galt2 | intronic | . | . | . | 4qD2.2 | rs27558177 | 1 | 0 |
| chr4 | 128761707 | C | T | 228 | A3galt2 | exonic | missense SNV | A3galt2:NM_001009819:exon3:c.C254T:p.P85L | . | 4qD2.2 | rs27558175 | 1 | 0 |
| chr4 | 128774706 | A | G | 228 | Zfp362 | intronic | . | . | . | 4qD2.2 | rs27542526 | 1 | 0 |
| chr4 | 128776921 | T | G | 115 | Zfp362 | intronic | . | . | . | 4qD2.2 | rs27542518 | 1 | 0 |
| chr4 | 128777316 | A | G | 140 | Zfp362 | intronic | . | . | . | 4qD2.2 | rs217002352 | 1 | 0 |
| chr4 | 128777326 | T | G | 101 | Zfp362 | intronic | . | . | . | 4qD2.2 | rs235199097 | 1 | 0 |
| chr4 | 128777425 | T | G | 144 | Zfp362 | exonic | synonymous SNV | Zfp362:NM_001081098:exon7:c.A924C:p.P308P | . | 4qD2.2 | rs27542517 | 1 | 0 |
| chr4 | 128785978 | T | C | 228 | Zfp362 | exonic | synonymous SNV | Zfp362:NM_001081098:exon6:c.A894G:p.Q298Q | . | 4qD2.2 | rs27542447 | 1 | 0 |
| chr4 | 128786197 | A | G | 228 | Zfp362 | splicing | . | . | . | 4qD2.2 | rs259846449 | 1 | 0 |
| chr4 | 128786210 | C | T | 228 | Zfp362 | intronic | . | . | . | 4qD2.2 | rs27542446 | 1 | 0 |
| chr4 | 128786218 | A | C | 228 | Zfp362 | intronic | . | . | . | 4qD2.2 | rs244265405 | 1 | 0 |
| chr4 | 128787019 | C | G | 228 | Zfp362 | exonic | missense SNV | Zfp362:NM_001081098:exon5:c.G551C:p.G184A | . | 4qD2.2 | rs228481380 | 1 | 0 |
| chr4 | 128787159 | T | G | 228 | Zfp362 | exonic | synonymous SNV | Zfp362:NM_001081098:exon5:c.A411C:p.G137G | . | 4qD2.2 | rs27542442 | 1 | 0 |
| chr4 | 128787201 | T | C | 228 | Zfp362 | exonic | synonymous SNV | Zfp362:NM_001081098:exon5:c.A369G:p.R123R | . | 4qD2.2 | rs27542441 | 1 | 0 |
| chr4 | 128794589 | G | A | 107 | Zfp362 | intronic | . | . | . | 4qD2.2 | rs223183864 | 1 | 0 |
| chr4 | 128794710 | A | G | 137 | Zfp362 | intronic | . | . | . | 4qD2.2 | rs264553020 | 1 | 0 |
| chr4 | 128794733 | A | G | 198 | Zfp362 | intronic | . | . | . | 4qD2.2 | rs258699143 | 1 | 0 |
| chr4 | 128794738 | T | C | 222 | Zfp362 | intronic | . | . | . | 4qD2.2 | rs232150170 | 1 | 0 |
| chr4 | 128869470 | G | A | 228 | . | intergenic | . | . | . | 4qD2.2 | rs27518804 | 1 | 0 |
| chr4 | 128896725 | T | C | 204 | Trim62 | intronic | . | . | . | 4qD2.2 | rs259478507 | 1 | 0 |
| chr4 | 128896767 | G | T | 228 | Trim62 | intronic | . | . | . | 4qD2.2 | rs27518698 | 1 | 0 |
| chr4 | 128896901 | T | C | 228 | Trim62 | exonic | synonymous SNV | Trim62:NM_178110:exon2:c.T490C:p.L164L | . | 4qD2.2 | rs27518697 | 1 | 0 |
| chr4 | 128896949 | A | G | 228 | Trim62 | intronic | . | . | . | 4qD2.2 | rs249390941 | 1 | 0 |
| chr4 | 128900421 | C | T | 129 | Trim62 | intronic | . | . | . | 4qD2.2 | rs225501179 | 1 | 0 |
| chr4 | 128900517 | A | G | 202 | Trim62 | intronic | . | . | . | 4qD2.2 | rs45910311 | 1 | 0 |
| chr4 | 128902406 | T | C | 228 | Trim62 | intronic | . | . | . | 4qD2.2 | rs32668035 | 1 | 0 |
| chr4 | 128902431 | C | T | 228 | Trim62 | intronic | . | . | . | 4qD2.2 | rs31791046 | 1 | 0 |
| chr4 | 128902584 | T | C | 228 | Trim62 | exonic | synonymous SNV | Trim62:NM_178110:exon4:c.T873C:p.H291H | . | 4qD2.2 | rs32418693 | 1 | 0 |
| chr4 | 128908962 | C | T | 85 | Trim62 | intronic | . | . | . | 4qD2.2 | rs27518645 | 1 | 0 |
| chr4 | 128909040 | A | G | 227 | Trim62 | exonic | synonymous SNV | Trim62:NM_178110:exon5:c.A882G:p.P294P | . | 4qD2.2 | rs27518643 | 1 | 0 |
| chr4 | 128909232 | G | A | 228 | Trim62 | exonic | synonymous SNV | Trim62:NM_178110:exon5:c.G1074A:p.A358A | . | 4qD2.2 | rs27518642 | 1 | 0 |
| chr4 | 128909322 | C | T | 228 | Trim62 | exonic | synonymous SNV | Trim62:NM_178110:exon5:c.C1164T:p.C388C | . | 4qD2.2 | rs27518641 | 1 | 0 |
| chr4 | 128909373 | T | G | 228 | Trim62 | exonic | synonymous SNV | Trim62:NM_178110:exon5:c.T1215G:p.T405T | . | 4qD2.2 | rs27518640 | 1 | 0 |
| chr4 | 128930803 | G | A | 228 | Azin2 | UTR3 | . | . | . | 4qD2.2 | rs27548613 | 1 | 0 |
| chr4 | 128930858 | C | A | 228 | Azin2 | UTR3 | . | . | . | 4qD2.2 | rs27548612 | 1 | 0 |
| chr4 | 128932424 | G | A | 228 | Azin2 | exonic | synonymous SNV | Azin2:NM_172875:exon11:c.C1309T:p.L437L | . | 4qD2.2 | rs27548600 | 1 | 0 |
| chr4 | 128934528 | G | A | 228 | Azin2 | exonic | synonymous SNV | Azin2:NM_001301841:exon8:c.C885T:p.T295T,Azin2:NM_172875:exon10:c.C1170T:p.T390T | . | 4qD2.2 | rs27548585 | 1 | 0 |
| chr4 | 128934531 | G | A | 95 | Azin2 | exonic | synonymous SNV | Azin2:NM_001301841:exon8:c.C882T:p.Y294Y,Azin2:NM_172875:exon10:c.C1167T:p.Y389Y | . | 4qD2.2 | . | 0 | 1 |
| chr4 | 128934642 | G | A | 228 | Azin2 | exonic | synonymous SNV | Azin2:NM_001301841:exon8:c.C771T:p.S257S,Azin2:NM_172875:exon10:c.C1056T:p.S352S | . | 4qD2.2 | rs27548584 | 1 | 0 |
| chr4 | 128946021 | G | A | 189 | Azin2 | intronic | . | . | . | 4qD2.2 | rs27524798 | 1 | 0 |
| chr4 | 128947765 | T | C | 228 | Azin2 | intronic | . | . | . | 4qD2.2 | rs27524778 | 1 | 0 |
| chr4 | 128947784 | A | C | 172 | Azin2 | intronic | . | . | . | 4qD2.2 | rs254856081 | 1 | 0 |
| chr4 | 128948668 | A | G | 116 | Azin2 | intronic | . | . | . | 4qD2.2 | rs27524765 | 1 | 0 |
| chr4 | 128948750 | T | C | 228 | Azin2 | intronic | . | . | . | 4qD2.2 | rs239980593 | 1 | 0 |
| chr4 | 128948775 | G | A | 228 | Azin2 | intronic | . | . | . | 4qD2.2 | rs27524764 | 1 | 0 |
| chr4 | 128948975 | G | A | 228 | Azin2 | intronic | . | . | . | 4qD2.2 | rs27524761 | 1 | 0 |
| chr4 | 128949076 | A | G | 168 | Azin2 | intronic | . | . | . | 4qD2.2 | rs27524760 | 1 | 0 |
| chr4 | 128949100 | C | T | 157 | Azin2 | intronic | . | . | . | 4qD2.2 | rs27524759 | 1 | 0 |
| chr4 | 128949132 | G | A | 130 | Azin2 | intronic | . | . | . | 4qD2.2 | rs27524758 | 1 | 0 |
| chr4 | 128949424 | A | G | 153 | Azin2 | intronic | . | . | . | 4qD2.2 | rs27524752 | 1 | 0 |
| chr4 | 128949468 | A | G | 185 | Azin2 | intronic | . | . | . | 4qD2.2 | rs248435399 | 1 | 0 |
| chr4 | 128949709 | A | T | 228 | Azin2 | exonic | synonymous SNV | Azin2:NM_001301841:exon4:c.T183A:p.I61I,Azin2:NM_172875:exon6:c.T468A:p.I156I | . | 4qD2.2 | rs27524750 | 1 | 0 |
| chr4 | 128949735 | C | T | 228 | Azin2 | intronic | . | . | . | 4qD2.2 | rs27524749 | 1 | 0 |
| chr4 | 128950550 | T | C | 116 | Azin2 | intronic | . | . | . | 4qD2.2 | rs32321940 | 1 | 0 |
| chr4 | 128950556 | C | T | 123 | Azin2 | intronic | . | . | . | 4qD2.2 | rs234126406 | 1 | 0 |
| chr4 | 128950593 | T | A | 140 | Azin2 | intronic | . | . | . | 4qD2.2 | rs27524740 | 1 | 0 |
| chr4 | 128960007 | C | A | 228 | Azin2 | intronic | . | . | . | 4qD2.2 | rs31951563 | 1 | 0 |
| chr4 | 128961214 | G | A | 204 | Azin2 | intronic | . | . | . | 4qD2.2 | rs220256597 | 1 | 0 |
| chr4 | 128961250 | A | T | 228 | Azin2 | intronic | . | . | . | 4qD2.2 | rs32849470 | 1 | 0 |
| chr4 | 128962087 | G | A | 91 | Azin2 | UTR5 | . | . | 356 | 4qD2.2 | rs250792771 | 1 | 0 |
| chr4 | 128993164 | T | G | 168 | . | upstream | . | . | 537 | 4qD2.2 | rs234570285 | 1 | 0 |
| chr4 | 128993180 | G | A | 170 | . | upstream | . | . | 537 | 4qD2.2 | rs252113259 | 1 | 0 |
| chr4 | 128993397 | C | T | 228 | Ak2 | exonic | synonymous SNV | Ak2:NM_001033966:exon1:c.C54T:p.A18A,Ak2:NM_016895:exon1:c.C54T:p.A18A | 537 | 4qD2.2 | rs13472978 | 1 | 0 |
| chr4 | 128993455 | T | C | 228 | Ak2 | intronic | . | . | 537 | 4qD2.2 | rs27524584 | 1 | 0 |
| chr4 | 128999214 | A | G | 228 | Ak2 | exonic | synonymous SNV | Ak2:NM_001033966:exon2:c.A174G:p.S58S,Ak2:NM_016895:exon2:c.A174G:p.S58S | . | 4qD2.2 | rs13472976 | 1 | 0 |
| chr4 | 128999361 | C | A | 228 | Ak2 | intronic | . | . | . | 4qD2.2 | rs27508048 | 1 | 0 |
| chr4 | 128999421 | G | C | 164 | Ak2 | intronic | . | . | . | 4qD2.2 | rs27508047 | 1 | 0 |
| chr4 | 129001917 | A | C | 145 | Ak2 | intronic | . | . | . | 4qD2.2 | rs27508024 | 1 | 0 |
| chr4 | 129001929 | C | T | 162 | Ak2 | intronic | . | . | . | 4qD2.2 | rs223584835 | 1 | 0 |
| chr4 | 129002278 | T | C | 228 | Ak2 | intronic | . | . | . | 4qD2.2 | rs27508022 | 1 | 0 |
| chr4 | 129002500 | T | C | 228 | Ak2 | intronic | . | . | . | 4qD2.2 | rs27508021 | 1 | 0 |
| chr4 | 129002508 | T | G | 228 | Ak2 | intronic | . | . | . | 4qD2.2 | rs50403964 | 1 | 0 |
| chr4 | 129007591 | G | A | 225 | Ak2 | intronic | . | . | . | 4qD2.2 | rs27507988 | 1 | 0 |
| chr4 | 129007982 | A | G | 228 | Ak2 | intronic | . | . | . | 4qD2.2 | rs27507986 | 1 | 0 |
| chr4 | 129008072 | C | T | 228 | Ak2 | exonic | synonymous SNV | Ak2:NM_001033966:exon6:c.C537T:p.N179N,Ak2:NM_016895:exon6:c.C537T:p.N179N | . | 4qD2.2 | rs13472979 | 1 | 0 |
| chr4 | 129058928 | C | T | 186 | Rnf19b | intronic | . | . | 983 | 4qD2.2 | rs238494327 | 1 | 0 |
| chr4 | 129071767 | T | C | 228 | Rnf19b | exonic | synonymous SNV | Rnf19b:NM_029219:exon2:c.T657C:p.S219S | . | 4qD2.2 | rs243051479 | 1 | 0 |
| chr4 | 129071993 | C | T | 228 | Rnf19b | intronic | . | . | . | 4qD2.2 | rs260924846 | 1 | 0 |
| chr4 | 129073426 | A | G | 228 | Rnf19b | intronic | . | . | . | 4qD2.2 | rs260689273 | 1 | 0 |
| chr4 | 129073772 | G | A | 228 | Rnf19b | intronic | . | . | . | 4qD2.2 | rs241806923 | 1 | 0 |
| chr4 | 129075427 | C | A | 170 | Rnf19b | intronic | . | . | . | 4qD2.2 | rs223163653 | 1 | 0 |
| chr4 | 129078757 | G | A | 177 | Rnf19b | intronic | . | . | . | 4qD2.2 | rs27507777 | 1 | 0 |
| chr4 | 129078962 | T | C | 228 | Rnf19b | exonic | synonymous SNV | Rnf19b:NM_029219:exon6:c.T1308C:p.L436L | . | 4qD2.2 | rs13472389 | 1 | 0 |
| chr4 | 129080571 | A | C | 228 | Rnf19b | intronic | . | . | . | 4qD2.2 | rs27507769 | 1 | 0 |
| chr4 | 129080572 | G | A | 228 | Rnf19b | intronic | . | . | . | 4qD2.2 | rs27507768 | 1 | 0 |
| chr4 | 129082513 | G | A | 115 | Rnf19b | intronic | . | . | . | 4qD2.2 | rs27493653 | 1 | 0 |
| chr4 | 129082683 | T | G | 228 | Rnf19b | intronic | . | . | . | 4qD2.2 | rs27493651 | 1 | 0 |
| chr4 | 129082762 | T | C | 228 | Rnf19b | exonic | synonymous SNV | Rnf19b:NM_029219:exon8:c.T1617C:p.A539A | . | 4qD2.2 | rs13472388 | 1 | 0 |
| chr4 | 129082920 | A | T | 228 | Rnf19b | intronic | . | . | . | 4qD2.2 | rs27493649 | 1 | 0 |
| chr4 | 129082984 | G | A | 228 | Rnf19b | intronic | . | . | . | 4qD2.2 | rs27493647 | 1 | 0 |
| chr4 | 129083024 | A | T | 158 | Rnf19b | intronic | . | . | . | 4qD2.2 | rs27493646 | 1 | 0 |
| chr4 | 129083671 | G | A | 228 | Rnf19b | intronic | . | . | . | 4qD2.2 | rs27493643 | 1 | 0 |
| chr4 | 129084167 | T | C | 219 | Rnf19b | exonic | missense SNV | Rnf19b:NM_029219:exon9:c.T2173C:p.S725P | . | 4qD2.2 | rs27493637 | 1 | 0 |
| chr4 | 129110776 | T | C | 228 | Tmem54 | intronic | . | . | . | 4qD2.2 | rs48619001 | 1 | 0 |
| chr4 | 129111185 | T | C | 228 | Tmem54 | intronic | . | . | . | 4qD2.2 | rs46543081 | 1 | 0 |
| chr4 | 129111443 | T | C | 228 | Tmem54 | UTR3 | . | . | . | 4qD2.2 | rs233233997 | 1 | 0 |
| chr4 | 129112312 | T | C | 124 | Hpca | UTR3 | . | . | . | 4qD2.2 | rs256568259 | 1 | 0 |
| chr4 | 129112802 | G | A | 228 | Hpca | intronic | . | . | . | 4qD2.2 | rs242913730 | 1 | 0 |
| chr4 | 129112803 | G | A | 228 | Hpca | intronic | . | . | . | 4qD2.2 | rs211965219 | 1 | 0 |
| chr4 | 129112855 | G | A | 228 | Hpca | intronic | . | . | . | 4qD2.2 | rs233808225 | 1 | 0 |
| chr4 | 129138632 | G | A | 189 | Fndc5 | intronic | . | . | . | 4qD2.2 | rs256571681 | 1 | 0 |
| chr4 | 129138698 | C | T | 163 | Fndc5 | exonic | synonymous SNV | Fndc5:NM_027402:exon2:c.C93T:p.P31P | . | 4qD2.2 | rs220488675 | 1 | 0 |
| chr4 | 129138701 | A | G | 163 | Fndc5 | exonic | synonymous SNV | Fndc5:NM_027402:exon2:c.A96G:p.S32S | . | 4qD2.2 | rs240727711 | 1 | 0 |
| chr4 | 129139230 | T | C | 133 | Fndc5 | intronic | . | . | . | 4qD2.2 | rs244823727 | 1 | 0 |
| chr4 | 129139255 | A | G | 153 | Fndc5 | intronic | . | . | . | 4qD2.2 | rs261423505 | 1 | 0 |
| chr4 | 129139549 | C | T | 228 | Fndc5 | intronic | . | . | . | 4qD2.2 | rs234920505 | 1 | 0 |
| chr4 | 129139764 | C | T | 228 | Fndc5 | intronic | . | . | . | 4qD2.2 | rs212307593 | 1 | 0 |
| chr4 | 129139951 | T | C | 228 | Fndc5 | intronic | . | . | . | 4qD2.2 | rs243485125 | 1 | 0 |
| chr4 | 129139971 | G | T | 228 | Fndc5 | intronic | . | . | . | 4qD2.2 | rs252903491 | 1 | 0 |
| chr4 | 129142141 | C | T | 228 | Fndc5 | exonic | synonymous SNV | Fndc5:NM_027402:exon5:c.C570T:p.S190S | . | 4qD2.2 | rs234172029 | 1 | 0 |
| chr4 | 129142228 | T | C | 228 | Fndc5 | intronic | . | . | . | 4qD2.2 | rs246960851 | 1 | 0 |
| chr4 | 129142277 | A | G | 228 | Fndc5 | intronic | . | . | . | 4qD2.2 | rs216236914 | 1 | 0 |
| chr4 | 129142358 | T | C | 228 | Fndc5 | intronic | . | . | . | 4qD2.2 | rs233210022 | 1 | 0 |
| chr4 | 129142372 | G | A | 228 | Fndc5 | intronic | . | . | . | 4qD2.2 | rs250521781 | 1 | 0 |
| chr4 | 129150930 | G | A | 228 | S100pbp | UTR3 | . | . | . | 4qD2.2 | rs27493465 | 1 | 0 |
| chr4 | 129155463 | C | T | 228 | S100pbp | exonic | synonymous SNV | S100pbp:NM_029036:exon6:c.G1011A:p.Q337Q | . | 4qD2.2 | rs27493457 | 1 | 0 |
| chr4 | 129155673 | A | G | 228 | S100pbp | intronic | . | . | . | 4qD2.2 | rs262504932 | 1 | 0 |
| chr4 | 129177929 | G | A | 167 | S100pbp | intronic | . | . | . | 4qD2.2 | rs27493405 | 1 | 0 |
| chr4 | 129178190 | G | A | 228 | S100pbp | intronic | . | . | . | 4qD2.2 | rs27493404 | 1 | 0 |
| chr4 | 129180150 | G | A | 48.8885 | S100pbp | intronic | . | . | . | 4qD2.2 | rs583923614 | 1 | 0 |
| chr4 | 129180197 | A | G | 228 | S100pbp | exonic | synonymous SNV | S100pbp:NM_029036:exon4:c.T864C:p.T288T | . | 4qD2.2 | rs237751767 | 1 | 0 |
| chr4 | 129181903 | C | T | 228 | S100pbp | exonic | missense SNV | S100pbp:NM_029036:exon3:c.G628A:p.E210K | . | 4qD2.2 | rs27493397 | 1 | 0 |
| chr4 | 129182003 | T | A | 228 | S100pbp | exonic | missense SNV | S100pbp:NM_029036:exon3:c.A528T:p.Q176H | . | 4qD2.2 | rs27493396 | 1 | 0 |
| chr4 | 129182283 | A | G | 228 | S100pbp | exonic | missense SNV | S100pbp:NM_029036:exon3:c.T248C:p.I83T | . | 4qD2.2 | rs31850329 | 1 | 0 |
| chr4 | 129182600 | G | A | 228 | S100pbp | intronic | . | . | . | 4qD2.2 | rs27493392 | 1 | 0 |
| chr4 | 129189818 | A | T | 212 | Yars | UTR5 | . | . | 1323 | 4qD2.2 | rs27493378 | 1 | 0 |
| chr4 | 129189860 | G | A | 228 | Yars | UTR5 | . | . | 1323 | 4qD2.2 | rs263434422 | 1 | 0 |
| chr4 | 129189928 | C | T | 228 | Yars | exonic | synonymous SNV | Yars:NM_134151:exon1:c.C21T:p.S7S | 1323 | 4qD2.2 | rs27493377 | 1 | 0 |
| chr4 | 129190014 | C | A | 228 | Yars | exonic | missense SNV | Yars:NM_134151:exon1:c.C107A:p.A36D | 1323 | 4qD2.2 | rs27493376 | 1 | 0 |
| chr4 | 129196838 | A | G | 228 | Yars | exonic | synonymous SNV | Yars:NM_134151:exon2:c.A174G:p.L58L | . | 4qD2.2 | rs47162775 | 1 | 0 |
| chr4 | 129196958 | T | C | 228 | Yars | exonic | synonymous SNV | Yars:NM_134151:exon2:c.T294C:p.F98F | . | 4qD2.2 | rs27540206 | 1 | 0 |
| chr4 | 129196985 | C | T | 228 | Yars | intronic | . | . | . | 4qD2.2 | rs27540204 | 1 | 0 |
| chr4 | 129197130 | A | G | 228 | Yars | exonic | synonymous SNV | Yars:NM_134151:exon3:c.A330G:p.A110A | . | 4qD2.2 | rs3167103 | 1 | 0 |
| chr4 | 129200611 | A | G | 226 | Yars | intronic | . | . | . | 4qD2.2 | rs27540195 | 1 | 0 |
| chr4 | 129200874 | C | T | 205 | Yars | intronic | . | . | . | 4qD2.2 | rs240713966 | 1 | 0 |
| chr4 | 129205865 | T | A | 126 | Yars | intronic | . | . | . | 4qD2.2 | rs27540180 | 1 | 0 |
| chr4 | 129205993 | C | A | 228 | Yars | intronic | . | . | . | 4qD2.2 | rs27540179 | 1 | 0 |
| chr4 | 129206037 | C | T | 228 | Yars | intronic | . | . | . | 4qD2.2 | rs27540178 | 1 | 0 |
| chr4 | 129206116 | T | C | 228 | Yars | intronic | . | . | . | 4qD2.2 | rs27540175 | 1 | 0 |
| chr4 | 129206255 | A | G | 228 | Yars | intronic | . | . | . | 4qD2.2 | rs27540173 | 1 | 0 |
| chr4 | 129206385 | C | T | 162 | Yars | intronic | . | . | . | 4qD2.2 | rs254957982 | 1 | 0 |
| chr4 | 129206386 | C | G | 162 | Yars | intronic | . | . | . | 4qD2.2 | rs27540172 | 1 | 0 |

| | | | | | | | | | | | | | |
|---|---|---|---|---|---|---|---|---|---|---|---|---|---|
| chr4 | 129207870 | T | C | 228 | Yars | intronic | . | . | . | 4qD2.2 | rs264155727 | 1 | 0 |
| chr4 | 129207888 | C | A | 228 | Yars | intronic | . | . | . | 4qD2.2 | rs27517065 | 1 | 0 |
| chr4 | 129207952 | A | T | 228 | Yars | exonic | synonymous SNV | Yars:NM_134151:exon6:c.A711T:p.A237A | . | 4qD2.2 | rs27517064 | 1 | 0 |
| chr4 | 129208063 | T | A | 228 | Yars | intronic | . | . | . | 4qD2.2 | rs27517063 | 1 | 0 |
| chr4 | 129209939 | C | T | 91 | Yars | intronic | . | . | . | 4qD2.2 | rs27517057 | 1 | 0 |
| chr4 | 129210447 | G | A | 228 | Yars | intronic | . | . | . | 4qD2.2 | rs27517052 | 1 | 0 |
| chr4 | 129210710 | A | T | 157 | Yars | intronic | . | . | . | 4qD2.2 | rs235559911 | 1 | 0 |
| chr4 | 129214288 | T | A | 93 | Yars | intronic | . | . | . | 4qD2.2 | rs3167124 | 1 | 0 |
| chr4 | 129214635 | C | T | 228 | Yars | intronic | . | . | . | 4qD2.2 | rs27517037 | 1 | 0 |
| chr4 | 129215526 | C | T | 228 | Yars | intronic | . | . | . | 4qD2.2 | rs27517033 | 1 | 0 |
| chr4 | 129215564 | T | C | 216 | Yars | intronic | . | . | . | 4qD2.2 | rs27517032 | 1 | 0 |
| chr4 | 129216515 | G | A | 210 | Yars | intronic | . | . | . | 4qD2.2 | rs27517031 | 1 | 0 |
| chr4 | 129216826 | C | T | 191 | Yars | intronic | . | . | . | 4qD2.2 | rs27517028 | 1 | 0 |
| chr4 | 129218405 | A | C | 228 | Yars | intronic | . | . | . | 4qD2.2 | rs3165254 | 1 | 0 |
| chr4 | 129218425 | A | G | 228 | Yars | intronic | . | . | . | 4qD2.2 | rs227216447 | 1 | 0 |
| chr4 | 129218590 | A | G | 228 | Yars | UTR3 | . | . | . | 4qD2.2 | rs3167132 | 1 | 0 |
| chr4 | 129221123 | G | A | 118 | C77080 | UTR3 | . | . | . | 4qD2.2 | rs251105942 | 1 | 0 |
| chr4 | 129222314 | G | A | 228 | C77080 | exonic | synonymous SNV | C77080:NM_001033189:exon6:c.C2691T:p.R897R,C77080:NM_001285865:exon6:c.C2556T:p.R852R,C77080: | . | 4qD2.2 | rs27517004 | 1 | 0 |
| chr4 | 129222475 | G | T | 228 | C77080 | exonic | missense SNV | C77080:NM_001033189:exon6:c.C2530A:p.H844N,C77080:NM_001285865:exon6:c.C2395A:p.H799N,C77080: | . | 4qD2.2 | rs237420218 | 1 | 0 |
| chr4 | 129222526 | A | C | 228 | C77080 | exonic | missense SNV | C77080:NM_001033189:exon6:c.T2479G:p.S827A,C77080:NM_001285865:exon6:c.T2344G:p.S782A,C77080: | . | 4qD2.2 | rs27517003 | 1 | 0 |
| chr4 | 129222922 | T | C | 228 | C77080 | exonic | missense SNV | C77080:NM_001033189:exon6:c.A2083G:p.T695A,C77080:NM_001285865:exon6:c.A1948G:p.T650A,C77080: | . | 4qD2.2 | rs3165259 | 1 | 0 |
| chr4 | 129223553 | A | G | 147 | C77080 | exonic | synonymous SNV | C77080:NM_001033189:exon6:c.T1452C:p.S484S,C77080:NM_001285865:exon6:c.T1317C:p.S439S,C77080: | 263 | 4qD2.2 | . | 0 | 1 |
| chr4 | 129223949 | C | T | 228 | C77080 | exonic | synonymous SNV | C77080:NM_001033189:exon6:c.G1056A:p.P352P,C77080:NM_001285865:exon6:c.G921A:p.P307P,C77080:N | 675 | 4qD2.2 | rs27517002 | 1 | 0 |
| chr4 | 129224048 | G | A | 228 | C77080 | exonic | synonymous SNV | C77080:NM_001033189:exon6:c.C957T:p.L319L,C77080:NM_001285865:exon6:c.C822T:p.L274L,C77080:NM | 675 | 4qD2.2 | rs27517000 | 1 | 0 |
| chr4 | 129224185 | G | A | 228 | C77080 | exonic | synonymous SNV | C77080:NM_001033189:exon6:c.C820T:p.L274L,C77080:NM_001285865:exon6:c.C685T:p.L229L,C77080:NM | 675 | 4qD2.2 | rs27516999 | 1 | 0 |
| chr4 | 129224361 | G | C | 228 | C77080 | intronic | . | . | 675 | 4qD2.2 | rs246289727 | 1 | 0 |
| chr4 | 129225374 | G | A | 228 | C77080 | exonic | synonymous SNV | C77080:NM_001033189:exon4:c.C552T:p.R184R,C77080:NM_001285865:exon4:c.C417T:p.R139R,C77080:NM | . | 4qD2.2 | rs6234035 | 1 | 0 |
| chr4 | 129225974 | C | T | 228 | C77080 | exonic | missense SNV | C77080:NM_001033189:exon2:c.G266A:p.G89E,C77080:NM_001285865:exon2:c.G131A:p.G44E,C77080:NM_( | . | 4qD2.2 | rs27516992 | 1 | 0 |
| chr4 | 129248085 | A | T | 228 | C77080 | intronic | . | . | . | 4qD2.2 | rs234762941 | 1 | 0 |
| chr4 | 129248086 | T | C | 228 | C77080 | intronic | . | . | . | 4qD2.2 | rs253304777 | 1 | 0 |
| chr4 | 129293187 | A | G | 228 | Sync | intronic | . | . | . | 4qD2.2 | rs232239506 | 1 | 0 |
| chr4 | 129293380 | T | C | 228 | Sync | exonic | synonymous SNV | Sync:NM_023485:exon2:c.T204C:p.S68S | . | 4qD2.2 | rs27516918 | 1 | 0 |
| chr4 | 129293412 | T | A | 228 | Sync | exonic | missense SNV | Sync:NM_023485:exon2:c.T236A:p.L79Q | . | 4qD2.2 | rs27516917 | 1 | 0 |
| chr4 | 129293638 | T | G | 228 | Sync | exonic | missense SNV | Sync:NM_023485:exon2:c.T462G:p.D154E | . | 4qD2.2 | rs27516915 | 1 | 0 |
| chr4 | 129293815 | C | A | 228 | Sync | exonic | synonymous SNV | Sync:NM_023485:exon2:c.C639A:p.L213L | . | 4qD2.2 | rs27516914 | 1 | 0 |
| chr4 | 129293878 | G | A | 228 | Sync | exonic | synonymous SNV | Sync:NM_023485:exon2:c.G702A:p.K234K | . | 4qD2.2 | rs27516913 | 1 | 0 |
| chr4 | 129293941 | T | C | 228 | Sync | exonic | synonymous SNV | Sync:NM_023485:exon2:c.T765C:p.D255D | . | 4qD2.2 | rs233800045 | 1 | 0 |
| chr4 | 129294073 | G | T | 228 | Sync | exonic | synonymous SNV | Sync:NM_023485:exon2:c.G897T:p.P299P | . | 4qD2.2 | rs237917620 | 1 | 0 |
| chr4 | 129294346 | A | G | 228 | Sync | exonic | synonymous SNV | Sync:NM_023485:exon2:c.A1170G:p.Q390Q | . | 4qD2.2 | rs27516910 | 1 | 0 |
| chr4 | 129306266 | C | T | 228 | Sync | intronic | . | . | . | 4qD2.2 | rs225394300 | 1 | 0 |
| chr4 | 129306336 | G | A | 228 | Sync | intronic | . | . | . | 4qD2.2 | rs27516868 | 1 | 0 |
| chr4 | 129306436 | T | C | 228 | Sync | exonic | missense SNV | Sync:NM_023485:exon3:c.T1295C:p.M432T | . | 4qD2.2 | rs27516867 | 1 | 0 |
| chr4 | 129306602 | T | C | 228 | Sync | intronic | . | . | . | 4qD2.2 | rs27516866 | 1 | 0 |
| chr4 | 129306746 | T | A | 141 | Sync | intronic | . | . | . | 4qD2.2 | . | 0 | 1 |
| chr4 | 129308098 | T | C | 228 | Sync,Rbbp4 | UTR3 | . | . | . | 4qD2.2 | rs27516858 | 1 | 0 |
| chr4 | 129309971 | T | G | 228 | Rbbp4 | UTR3 | . | . | . | 4qD2.2 | rs243308597 | 1 | 0 |
| chr4 | 129318748 | C | T | 161 | Rbbp4 | intronic | . | . | . | 4qD2.2 | rs27516811 | 1 | 0 |
| chr4 | 129320397 | G | T | 228 | Rbbp4 | intronic | . | . | . | 4qD2.2 | rs27516801 | 1 | 0 |
| chr4 | 129322073 | A | G | 228 | Rbbp4 | intronic | . | . | . | 4qD2.2 | rs27516798 | 1 | 0 |
| chr4 | 129322502 | T | C | 228 | Rbbp4 | intronic | . | . | . | 4qD2.2 | rs27516795 | 1 | 0 |
| chr4 | 129328873 | G | A | 201 | Rbbp4 | intronic | . | . | . | 4qD2.2 | rs27516771 | 1 | 0 |
| chr4 | 129334316 | C | A | 152 | Rbbp4 | intronic | . | . | . | 4qD2.2 | rs221989480 | 1 | 0 |
| chr4 | 129334317 | A | C | 152 | Rbbp4 | intronic | . | . | . | 4qD2.2 | rs240138616 | 1 | 0 |
| chr4 | 129334471 | T | C | 228 | Rbbp4 | intronic | . | . | . | 4qD2.2 | rs32410664 | 1 | 0 |
| chr4 | 129334682 | G | A | 228 | Rbbp4 | intronic | . | . | 1390 | 4qD2.2 | rs32158345 | 1 | 0 |
| chr4 | 129334689 | G | A | 228 | Rbbp4 | intronic | . | . | 1390 | 4qD2.2 | rs32899125 | 1 | 0 |
| chr4 | 129334782 | A | G | 165 | Rbbp4 | intronic | . | . | 1390 | 4qD2.2 | rs264818184 | 1 | 0 |
| chr4 | 129334831 | C | T | 164 | Rbbp4 | intronic | . | . | 1390 | 4qD2.2 | rs224113832 | 1 | 0 |
| chr4 | 129335193 | T | C | 228 | Rbbp4 | intronic | . | . | 1390 | 4qD2.2 | rs260643599 | 1 | 0 |
| chr4 | 129335823 | A | G | 118 | . | upstream | . | . | 1390 | 4qD2.2 | rs237731595 | 1 | 0 |
| chr4 | 129335965 | G | A | 228 | . | upstream | . | . | 1390 | 4qD2.2 | rs230272326 | 1 | 0 |
| chr4 | 129336025 | A | G | 228 | . | upstream | . | . | . | 4qD2.2 | rs253503782 | 1 | 0 |
| chr4 | 129336212 | A | C | 228 | Zbtb8os | intronic | . | . | . | 4qD2.2 | rs32514925 | 1 | 0 |
| chr4 | 129336298 | G | A | 203 | Zbtb8os | intronic | . | . | . | 4qD2.2 | rs32629173 | 1 | 0 |
| chr4 | 129340545 | A | G | 172 | Zbtb8os | intronic | . | . | . | 4qD2.2 | rs253703728 | 1 | 0 |
| chr4 | 129341422 | A | G | 228 | Zbtb8os | intronic | . | . | . | 4qD2.2 | rs257699369 | 1 | 0 |
| chr4 | 129341642 | G | A | 228 | Zbtb8os | intronic | . | . | . | 4qD2.2 | rs215229965 | 1 | 0 |
| chr4 | 129341646 | T | C | 228 | Zbtb8os | intronic | . | . | . | 4qD2.2 | rs238160887 | 1 | 0 |
| chr4 | 129341671 | G | C | 228 | Zbtb8os | intronic | . | . | . | 4qD2.2 | rs250465170 | 1 | 0 |
| chr4 | 129341973 | T | C | 172 | Zbtb8os | intronic | . | . | . | 4qD2.2 | rs245005200 | 1 | 0 |
| chr4 | 129343082 | C | T | 94 | Zbtb8os | intronic | . | . | . | 4qD2.2 | rs246618469 | 1 | 0 |
| chr4 | 129343206 | G | A | 185 | Zbtb8os | intronic | . | . | . | 4qD2.2 | rs244598252 | 1 | 0 |
| chr4 | 129344030 | T | C | 159 | Zbtb8os | intronic | . | . | . | 4qD2.2 | rs27503255 | 1 | 0 |
| chr4 | 129344093 | A | G | 168 | Zbtb8os | intronic | . | . | . | 4qD2.2 | rs48193523 | 1 | 0 |
| chr4 | 129344173 | T | C | 228 | Zbtb8os | intronic | . | . | . | 4qD2.2 | rs27503254 | 1 | 0 |
| chr4 | 129344242 | C | T | 228 | Zbtb8os | exonic | synonymous SNV | Zbtb8os:NM_025970:exon6:c.C411T:p.H137H | . | 4qD2.2 | rs263595458 | 1 | 0 |
| chr4 | 129346654 | A | G | 200 | Zbtb8os | intronic | . | . | . | 4qD2.2 | rs27503238 | 1 | 0 |
| chr4 | 129346764 | C | T | 228 | Zbtb8os | intronic | . | . | . | 4qD2.2 | rs253128760 | 1 | 0 |
| chr4 | 129346921 | G | A | 228 | Zbtb8os | UTR3 | . | . | . | 4qD2.2 | rs27503237 | 1 | 0 |
| chr4 | 129346939 | G | T | 201 | Zbtb8os | UTR3 | . | . | . | 4qD2.2 | rs27503236 | 1 | 0 |
| chr4 | 129354007 | G | A | 86 | Zbtb8a | UTR3 | . | . | . | 4qD2.2 | rs27503210 | 1 | 0 |
| chr4 | 129354214 | G | A | 228 | Zbtb8a | exonic | synonymous SNV | Zbtb8a:NM_028603:exon5:c.C1263T:p.S421S | . | 4qD2.2 | rs236300280 | 1 | 0 |
| chr4 | 129354274 | G | T | 228 | Zbtb8a | exonic | synonymous SNV | Zbtb8a:NM_028603:exon5:c.C1203A:p.S401S | . | 4qD2.2 | rs263731988 | 1 | 0 |
| chr4 | 129354460 | G | A | 228 | Zbtb8a | exonic | synonymous SNV | Zbtb8a:NM_028603:exon5:c.C1017T:p.H339H | . | 4qD2.2 | rs27503206 | 1 | 0 |
| chr4 | 129357670 | C | T | 228 | Zbtb8a | intronic | . | . | . | 4qD2.2 | rs27503191 | 1 | 0 |
| chr4 | 129359968 | G | A | 228 | Zbtb8a | exonic | synonymous SNV | Zbtb8a:NM_028603:exon3:c.C732T:p.A244A | . | 4qD2.2 | rs27503179 | 1 | 0 |
| chr4 | 129359976 | C | A | 228 | Zbtb8a | exonic | missense SNV | Zbtb8a:NM_028603:exon3:c.G724T:p.V242L | . | 4qD2.2 | rs27503178 | 1 | 0 |
| chr4 | 129360004 | A | G | 228 | Zbtb8a | exonic | synonymous SNV | Zbtb8a:NM_028603:exon3:c.T696C:p.H232H | . | 4qD2.2 | rs27503177 | 1 | 0 |
| chr4 | 129360212 | T | C | 228 | Zbtb8a | exonic | missense SNV | Zbtb8a:NM_028603:exon3:c.A488G:p.H163R | . | 4qD2.2 | rs256074302 | 1 | 0 |
| chr4 | 129360394 | C | T | 228 | Zbtb8a | exonic | synonymous SNV | Zbtb8a:NM_028603:exon3:c.G306A:p.A102A | . | 4qD2.2 | rs235077177 | 1 | 0 |
| chr4 | 129360397 | G | A | 228 | Zbtb8a | exonic | synonymous SNV | Zbtb8a:NM_028603:exon3:c.C303T:p.A101A | . | 4qD2.2 | rs27503176 | 1 | 0 |
| chr4 | 129360568 | T | C | 228 | Zbtb8a | exonic | synonymous SNV | Zbtb8a:NM_028603:exon3:c.A132G:p.A44A | . | 4qD2.2 | rs27503174 | 1 | 0 |
| chr4 | 129396027 | C | G | 145 | . | intergenic | . | . | . | 4qD2.2 | rs27503025 | 1 | 0 |
| chr4 | 129396066 | A | G | 157 | . | intergenic | . | . | . | 4qD2.2 | rs585795596 | 1 | 0 |
| chr4 | 129396235 | T | C | 228 | . | intergenic | . | . | . | 4qD2.2 | rs27503023 | 1 | 0 |
| chr4 | 129427696 | C | A | 228 | Zbtb8b | exonic | missense SNV | Zbtb8b:NM_153541:exon4:c.G1272T:p.E424D | . | 4qD2.2 | rs27486670 | 1 | 0 |
| chr4 | 129427833 | A | G | 228 | Zbtb8b | intronic | . | . | . | 4qD2.2 | rs218871280 | 1 | 0 |
| chr4 | 129427859 | A | C | 228 | Zbtb8b | intronic | . | . | . | 4qD2.2 | rs27486669 | 1 | 0 |
| chr4 | 129428624 | C | T | 140 | Zbtb8b | intronic | . | . | . | 4qD2.2 | rs27486661 | 1 | 0 |
| chr4 | 129432227 | T | A | 228 | Zbtb8b | intronic | . | . | . | 4qD2.2 | rs27486629 | 1 | 0 |
| chr4 | 129432484 | T | C | 228 | Zbtb8b | exonic | missense SNV | Zbtb8b:NM_153541:exon2:c.A788G:p.N263S | . | 4qD2.2 | rs27486628 | 1 | 0 |
| chr4 | 129432954 | C | T | 228 | Zbtb8b | exonic | synonymous SNV | Zbtb8b:NM_153541:exon2:c.G318A:p.Q106Q | . | 4qD2.2 | rs27486626 | 1 | 0 |
| chr4 | 129433278 | G | A | 228 | Zbtb8b | UTR5 | . | . | . | 4qD2.2 | rs27486624 | 1 | 0 |
| chr4 | 129433342 | A | G | 228 | Zbtb8b | intronic | . | . | . | 4qD2.2 | rs27486623 | 1 | 0 |
| chr4 | 129465114 | C | T | 210 | Bsdc1 | intronic | . | . | . | 4qD2.2 | rs27486506 | 1 | 0 |
| chr4 | 129465489 | C | T | 195 | Bsdc1 | intronic | . | . | . | 4qD2.2 | rs259563549 | 1 | 0 |
| chr4 | 129465497 | C | A | 188 | Bsdc1 | intronic | . | . | . | 4qD2.2 | rs227144240 | 1 | 0 |
| chr4 | 129466620 | C | T | 127 | Bsdc1 | intronic | . | . | . | 4qD2.2 | rs214013942 | 1 | 0 |
| chr4 | 129466760 | C | T | 228 | Bsdc1 | intronic | . | . | . | 4qD2.2 | rs227156549 | 1 | 0 |
| chr4 | 129467054 | A | T | 228 | Bsdc1 | intronic | . | . | . | 4qD2.2 | rs27486501 | 1 | 0 |
| chr4 | 129468803 | G | A | 162 | Bsdc1 | intronic | . | . | . | 4qD2.2 | rs27486495 | 1 | 0 |
| chr4 | 129468980 | T | C | 228 | Bsdc1 | intronic | . | . | . | 4qD2.2 | rs27486494 | 1 | 0 |
| chr4 | 129471728 | C | T | 153 | Bsdc1 | intronic | . | . | . | 4qD2.2 | rs262389914 | 1 | 0 |
| chr4 | 129472234 | C | T | 228 | Bsdc1 | intronic | . | . | . | 4qD2.2 | rs229458256 | 1 | 0 |
| chr4 | 129472273 | A | G | 172 | Bsdc1 | intronic | . | . | . | 4qD2.2 | rs27486491 | 1 | 0 |
| chr4 | 129472501 | G | A | 228 | Bsdc1 | intronic | . | . | . | 4qD2.2 | rs27486490 | 1 | 0 |
| chr4 | 129472647 | A | G | 228 | Bsdc1 | intronic | . | . | . | 4qD2.2 | rs27486489 | 1 | 0 |
| chr4 | 129474025 | G | C | 228 | Bsdc1 | exonic | synonymous SNV | Bsdc1:NM_133889:exon9:c.G927C:p.A309A | . | 4qD2.2 | rs27486482 | 1 | 0 |
| chr4 | 129474102 | C | G | 228 | Bsdc1 | exonic | missense SNV | Bsdc1:NM_133889:exon9:c.C1004G:p.T335S | . | 4qD2.2 | rs221807492 | 1 | 0 |
| chr4 | 129474270 | A | G | 228 | Bsdc1 | intronic | . | . | . | 4qD2.2 | rs27486480 | 1 | 0 |
| chr4 | 129474346 | G | T | 94 | Bsdc1 | intronic | . | . | . | 4qD2.2 | rs27486479 | 1 | 0 |
| chr4 | 129485077 | C | T | 228 | Bsdc1 | exonic | synonymous SNV | Bsdc1:NM_133889:exon10:c.C1188T:p.F396F | . | 4qD2.2 | rs13460392 | 1 | 0 |
| chr4 | 129485154 | A | G | 228 | Bsdc1 | intronic | . | . | . | 4qD2.2 | rs27541994 | 1 | 0 |
| chr4 | 129485297 | G | C | 164 | Bsdc1 | intronic | . | . | . | 4qD2.2 | rs27541993 | 1 | 0 |
| chr4 | 129487077 | A | G | 228 | Bsdc1 | UTR3 | . | . | . | 4qD2.2 | rs27541985 | 1 | 0 |
| chr4 | 129487129 | G | A | 228 | Bsdc1 | UTR3 | . | . | . | 4qD2.2 | rs27541984 | 1 | 0 |
| chr4 | 129489010 | C | A | 228 | Tssk3 | UTR3 | . | . | . | 4qD2.2 | rs252539168 | 1 | 0 |
| chr4 | 129490351 | G | A | 140 | Tssk3 | intronic | . | . | . | 4qD2.2 | rs27541977 | 1 | 0 |
| chr4 | 129490400 | T | C | 220 | Tssk3 | intronic | . | . | . | 4qD2.2 | rs27541976 | 1 | 0 |
| chr4 | 129490557 | C | T | 228 | Tssk3 | exonic | missense SNV | Tssk3:NM_080442:exon1:c.G50A:p.G17E | . | 4qD2.2 | . | 1 | 0 |
| chr4 | 129491020 | C | T | 115 | . | upstream | . | . | . | 4qD2.2 | rs27541974 | 1 | 0 |
| chr4 | 129491574 | T | C | 199 | Fam229a | intronic | . | . | 703 | 4qD2.2 | rs246897578 | 1 | 0 |
| chr4 | 129491628 | C | T | 221 | Fam229a | intronic | . | . | 703 | 4qD2.2 | rs216076940 | 1 | 0 |
| chr4 | 130068745 | G | T | 228 | Col16a1 | exonic | missense SNV | Col16a1:NM_028266:exon38:c.G2557T:p.G853W | . | 4qD2.2 | . | 1 | 0 |
| chr4 | 130092990 | T | C | 228 | Col16a1 | intronic | . | . | . | 4qD2.2 | rs32305736 | 1 | 0 |
| chr4 | 130093154 | T | C | 228 | Col16a1 | exonic | synonymous SNV | Col16a1:NM_028266:exon63:c.T3939C:p.P1313P | . | 4qD2.2 | rs31965138 | 1 | 0 |
| chr4 | 130093202 | G | C | 179 | Col16a1 | intronic | . | . | . | 4qD2.2 | rs27491891 | 1 | 0 |
| chr4 | 130094665 | T | C | 209 | Col16a1 | intronic | . | . | . | 4qD2.2 | rs27491879 | 1 | 0 |
| chr4 | 130095524 | A | G | 228 | Col16a1 | intronic | . | . | . | 4qD2.2 | rs27491873 | 1 | 0 |
| chr4 | 130096293 | C | T | 228 | Col16a1 | intronic | . | . | . | 4qD2.2 | rs27540479 | 1 | 0 |
| chr4 | 130096408 | A | G | 228 | Col16a1 | exonic | synonymous SNV | Col16a1:NM_028266:exon66:c.A4092G:p.P1364P | . | 4qD2.2 | rs47055495 | 1 | 0 |
| chr4 | 130097752 | T | G | 228 | Col16a1 | intronic | . | . | . | 4qD2.2 | rs27540477 | 1 | 0 |
| chr4 | 130098123 | T | A | 228 | Col16a1 | intronic | . | . | . | 4qD2.2 | rs223009116 | 1 | 0 |
| chr4 | 130098859 | G | A | 228 | Col16a1 | intronic | . | . | . | 4qD2.2 | . | 1 | 0 |
| chr4 | 130098975 | G | A | 228 | Col16a1 | exonic | synonymous SNV | Col16a1:NM_028266:exon70:c.G4626A:p.P1542P | . | 4qD2.2 | . | 1 | 0 |
| chr4 | 130125024 | T | C | 228 | Pef1 | intronic | . | . | . | 4qD2.2 | rs27517821 | 1 | 0 |
| chr4 | 130125537 | T | G | 126 | Pef1 | intronic | . | . | . | 4qD2.2 | rs32528434 | 1 | 0 |
| chr4 | 130125916 | T | G | 208 | Pef1 | intronic | . | . | . | 4qD2.2 | rs225575106 | 1 | 0 |
| chr4 | 130126013 | C | T | 85 | Pef1 | intronic | . | . | . | 4qD2.2 | rs244026989 | 1 | 0 |
| chr4 | 130127201 | C | T | 221 | Pef1 | intronic | . | . | . | 4qD2.2 | rs230100656 | 1 | 0 |
| chr4 | 130127485 | C | T | 228 | Pef1 | UTR3 | . | . | . | 4qD2.2 | rs255090540 | 1 | 0 |
| chr4 | 130132333 | G | A | 78 | Hcrtr1 | intronic | . | . | . | 4qD2.2 | rs27517787 | 1 | 0 |
| chr4 | 130133563 | G | A | 169 | Hcrtr1 | intronic | . | . | . | 4qD2.2 | rs27517786 | 1 | 0 |
| chr4 | 130133671 | T | C | 228 | Hcrtr1 | intronic | . | . | . | 4qD2.2 | rs32323079 | 1 | 0 |
| chr4 | 130133844 | G | A | 228 | Hcrtr1 | exonic | synonymous SNV | Hcrtr1:NM_001163027:exon7:c.C816T:p.L272L,Hcrtr1:NM_198959:exon7:c.C816T:p.L272L,Hcrtr1:NM_0( | . | 4qD2.2 | rs32671351 | 1 | 0 |
| chr4 | 130135938 | A | G | 228 | Hcrtr1 | intronic | . | . | . | 4qD2.2 | rs32485362 | 1 | 0 |
| chr4 | 130135948 | A | G | 206 | Hcrtr1 | intronic | . | . | . | 4qD2.2 | rs32700856 | 1 | 0 |
| chr4 | 130165983 | A | G | 136 | Tinagl1 | UTR3 | . | . | . | 4qD2.2 | rs27517674 | 1 | 0 |
| chr4 | 130166170 | T | C | 228 | Tinagl1 | exonic | synonymous SNV | Tinagl1:NM_001168333:exon12:c.A1296G:p.E432E,Tinagl1:NM_023476:exon12:c.A1296G:p.E432E | . | 4qD2.2 | rs8274319 | 1 | 0 |
| chr4 | 130166381 | G | T | 228 | Tinagl1 | intronic | . | . | . | 4qD2.2 | rs33009115 | 1 | 0 |
| chr4 | 130166834 | A | T | 228 | Tinagl1 | intronic | . | . | . | 4qD2.2 | rs16794680 | 1 | 0 |
| chr4 | 130167784 | T | C | 228 | Tinagl1 | exonic | synonymous SNV | Tinagl1:NM_001168333:exon7:c.A768G:p.Q256Q,Tinagl1:NM_023476:exon7:c.A768G:p.Q256Q | . | 4qD2.2 | rs8274322 | 1 | 0 |
| chr4 | 130168926 | C | T | 228 | Tinagl1 | intronic | . | . | . | 4qD2.2 | rs27502966 | 1 | 0 |
| chr4 | 130169157 | A | G | 228 | Tinagl1 | intronic | . | . | . | 4qD2.2 | rs27502965 | 1 | 0 |
| chr4 | 130169372 | C | T | 228 | Tinagl1 | exonic | missense SNV | Tinagl1:NM_001168333:exon4:c.G403A:p.D135N,Tinagl1:NM_023476:exon4:c.G403A:p.D135N | . | 4qD2.2 | rs16784312 | 1 | 0 |
| chr4 | 130209760 | G | A | 228 | Gm853 | UTR3 | . | . | . | 4qD2.2 | rs256786070 | 1 | 0 |
| chr4 | 130211371 | G | A | 228 | Gm853 | exonic | synonymous SNV | Gm853:NM_001034872:exon8:c.C1005T:p.D335D | . | 4qD2.2 | rs230655664 | 1 | 0 |
| chr4 | 130211542 | G | A | 143 | Gm853 | intronic | . | . | . | 4qD2.2 | rs27502870 | 1 | 0 |
| chr4 | 130221545 | A | G | 228 | Gm853 | intronic | . | . | . | 4qD2.2 | rs255222202 | 1 | 0 |
| chr4 | 130255211 | A | G | 171 | Serinc2 | intronic | . | . | . | 4qD2.2 | rs240022482 | 1 | 0 |
| chr4 | 130258193 | C | G | 53 | Serinc2 | intronic | . | . | . | 4qD2.2 | rs223171046 | 1 | 0 |
| chr4 | 130258720 | A | G | 174 | Serinc2 | exonic | synonymous SNV | Serinc2:NM_001253386:exon7:c.T687C:p.S229S,Serinc2:NM_172702:exon7:c.T852C:p.S284S | . | 4qD2.2 | rs231628352 | 1 | 0 |
| chr4 | 130258774 | C | A | 228 | Serinc2 | exonic | synonymous SNV | Serinc2:NM_001253386:exon7:c.G633T:p.S211S,Serinc2:NM_172702:exon7:c.G798T:p.S266S | . | 4qD2.2 | rs251039793 | 1 | 0 |
| chr4 | 130258874 | G | A | 228 | Serinc2 | intronic | . | . | . | 4qD2.2 | rs234778795 | 1 | 0 |
| chr4 | 130258901 | A | G | 156 | Serinc2 | intronic | . | . | . | 4qD2.2 | rs253238061 | 1 | 0 |
| chr4 | 130260610 | A | G | 174 | Serinc2 | intronic | . | . | . | 4qD2.2 | rs220445609 | 1 | 0 |
| chr4 | 130260614 | G | C | 175 | Serinc2 | intronic | . | . | . | 4qD2.2 | rs236313610 | 1 | 0 |
| chr4 | 130275531 | T | G | 186 | Serinc2 | UTR5 | . | . | 755 | 4qD2.2 | rs46600786 | 1 | 0 |
| chr4 | 130275547 | G | A | 154 | Serinc2 | UTR5 | . | . | 755 | 4qD2.2 | rs51798506 | 1 | 0 |
| chr4 | 130531908 | T | C | 228 | Nkain1 | exonic | synonymous SNV | Nkain1:NM_025998:exon7:c.A621G:p.G207G | . | 4qD2.2 | rs28338515 | 1 | 0 |
| chr4 | 130532767 | A | T | 163 | Nkain1 | intronic | . | . | . | 4qD2.2 | rs28338519 | 1 | 0 |
| chr4 | 130533823 | G | A | 217 | Nkain1 | intronic | . | . | . | 4qD2.2 | rs235866575 | 1 | 0 |
| chr4 | 130537225 | A | G | 228 | Nkain1 | intronic | . | . | . | 4qD2.2 | rs28338530 | 1 | 0 |
| chr4 | 130537426 | C | G | 228 | Nkain1 | intronic | . | . | . | 4qD2.2 | rs229377946 | 1 | 0 |
| chr4 | 130537444 | C | T | 228 | Nkain1 | intronic | . | . | . | 4qD2.2 | rs262557288 | 1 | 0 |

| | | | | | | | | | | | | | |
|---|---|---|---|---|---|---|---|---|---|---|---|---|---|
| chr4 | 130539269 | C | T | 228 | Nkain1 | intronic | . | . | . | 4qD2.2 | rs28338547 | 1 | 0 |
| chr4 | 130719285 | C | A | 228 | Pum1 | exonic | synonymous SNV | Pum1:NM_001159603:exon5:c.C654A:p.G218G,Pum1:NM_001159604:exon5:c.C654A:p.G218G,Pum1:NM_03072 | . | 4qD2.2 | rs255055278 | 1 | 0 |
| chr4 | 130741375 | G | A | 228 | Pum1 | intronic | . | . | . | 4qD2.2 | . | 1 | 0 |
| chr4 | 130816745 | A | G | 167 | Sdc3 | intronic | . | . | . | 4qD2.2 | rs27590043 | 1 | 0 |
| chr4 | 130816767 | G | A | 177 | Sdc3 | intronic | . | . | . | 4qD2.2 | rs27590042 | 1 | 0 |
| chr4 | 130818700 | A | G | 169 | Sdc3 | exonic | synonymous SNV | Sdc3:NM_011520:exon3:c.A369G:p.P123P | . | 4qD2.2 | rs27590032 | 1 | 0 |
| chr4 | 130819191 | C | T | 228 | Sdc3 | exonic | missense SNV | Sdc3:NM_011520:exon3:c.C860T:p.T287M | . | 4qD2.2 | rs254369871 | 1 | 0 |
| chr4 | 130821595 | G | T | 228 | Sdc3 | exonic | synonymous SNV | Sdc3:NM_011520:exon4:c.G1101T:p.S367S | . | 4qD2.2 | rs222205323 | 1 | 0 |
| chr4 | 130822650 | C | T | 167 | Sdc3 | exonic | synonymous SNV | Sdc3:NM_011520:exon5:c.C1170T:p.I390I | . | 4qD2.2 | . | 1 | 0 |
| chr4 | 130822677 | C | T | 198 | Sdc3 | exonic | synonymous SNV | Sdc3:NM_011520:exon5:c.C1197T:p.F399F | . | 4qD2.2 | rs242391312 | 1 | 0 |
| chr4 | 130926677 | C | T | 173 | Laptm5 | intronic | . | . | . | 4qD2.2 | rs27557497 | 1 | 0 |
| chr4 | 130931858 | G | A | 171 | Laptm5 | intronic | . | . | . | 4qD2.2 | rs27557460 | 1 | 0 |
| chr4 | 130944583 | C | T | 228 | Matn1 | intronic | . | . | . | 4qD2.2 | rs245654896 | 1 | 0 |
| chr4 | 130950176 | C | T | 228 | Matn1 | intronic | . | . | 226 | 4qD2.2 | rs32420300 | 1 | 0 |
| chr4 | 130951320 | T | C | 228 | Matn1 | exonic | synonymous SNV | Matn1:NM_010769:exon4:c.T708C:p.H236H | . | 4qD2.2 | rs27557371 | 1 | 0 |
| chr4 | 130953150 | C | G | 202 | Matn1 | intronic | . | . | . | 4qD2.2 | rs27557360 | 1 | 0 |
| chr4 | 130953175 | T | C | 197 | Matn1 | intronic | . | . | . | 4qD2.2 | rs32190563 | 1 | 0 |
| chr4 | 131489071 | A | G | 228 | . | intergenic | . | . | . | 4qD2.3 | rs27555108 | 1 | 0 |
| chr4 | 131489090 | C | T | 228 | . | intergenic | . | . | . | 4qD2.3 | rs27555107 | 1 | 0 |
| chr4 | 131489185 | C | A | 129 | . | intergenic | . | . | . | 4qD2.3 | rs27555106 | 1 | 0 |
| chr4 | 131802825 | T | G | 32.3158 | Ptpru | intronic | . | . | . | 4qD2.3 | . | 0 | 1 |
| chr4 | 131819261 | T | C | 40.9052 | Ptpru | intronic | . | . | . | 4qD2.3 | . | 0 | 1 |
| chr4 | 131991086 | G | A | 169 | Epb41 | intronic | . | . | . | 4qD2.3 | . | 1 | 0 |
| chr4 | 132074898 | T | A | 228 | Epb41 | intronic | . | . | 797 | 4qD2.3 | rs31883581 | 1 | 0 |
| chr4 | 132117376 | G | A | 228 | Oprd1 | exonic | missense SNV | Oprd1:NM_013622:exon2:c.C320T:p.A107V | . | 4qD2.3 | . | 1 | 0 |
| chr4 | 132232058 | G | C | 228 | Gmeb1 | exonic | synonymous SNV | Gmeb1:NM_020273:exon7:c.C684G:p.T228T,Gmeb1:NM_001122992:exon8:c.C684G:p.T228T | . | 4qD2.3 | . | 1 | 0 |
| chr4 | 132236896 | G | C | 143 | Gmeb1 | intronic | . | . | . | 4qD2.3 | rs27556605 | 1 | 0 |
| chr4 | 132237386 | A | G | 101 | Gmeb1 | intronic | . | . | . | 4qD2.3 | rs27556604 | 1 | 0 |
| chr4 | 132242316 | A | G | 228 | Gmeb1 | intronic | . | . | . | 4qD2.3 | rs27556582 | 1 | 0 |
| chr4 | 132242564 | T | G | 155 | Gmeb1 | intronic | . | . | . | 4qD2.3 | rs225833470 | 1 | 0 |
| chr4 | 132270004 | G | A | 174 | . | downstream | . | . | . | 4qD2.3 | rs32629361 | 1 | 0 |
| chr4 | 132270030 | G | T | 186 | . | downstream | . | . | . | 4qD2.3 | . | 1 | 0 |
| chr4 | 132270117 | A | G | 228 | Rnu11 | ncRNA_exonic | . | . | . | 4qD2.3 | . | 1 | 0 |
| chr4 | 132302298 | A | G | 228 | Rab42 | exonic | synonymous SNV | Rab42:NM_001081651:exon2:c.T612C:p.S204S | . | 4qD2.3 | rs27556445 | 1 | 0 |
| chr4 | 132310189 | A | G | 175 | Snhg12 | ncRNA_exonic | . | . | . | 4qD2.3 | rs27556394 | 1 | 0 |
| chr4 | 132310495 | C | T | 228 | Snhg12 | ncRNA_exonic | . | . | . | 4qD2.3 | rs32926047 | 1 | 0 |
| chr4 | 132310565 | A | C | 228 | Snhg12 | ncRNA_intronic | . | . | . | 4qD2.3 | rs32646935 | 1 | 0 |
| chr4 | 132310846 | A | T | 228 | Snhg12 | ncRNA_exonic | . | . | . | 4qD2.3 | . | 1 | 0 |
| chr4 | 132312105 | C | T | 228 | Trnau1ap | intronic | . | . | . | 4qD2.3 | . | 1 | 0 |
| chr4 | 132314128 | C | A | 228 | Trnau1ap | intronic | . | . | . | 4qD2.3 | rs32607896 | 1 | 0 |
| chr4 | 132319359 | A | G | 112 | Trnau1ap | intronic | . | . | . | 4qD2.3 | rs242998144 | 1 | 0 |
| chr4 | 132319624 | A | G | 228 | Trnau1ap | intronic | . | . | . | 4qD2.3 | rs237864571 | 1 | 0 |
| chr4 | 132319626 | C | A | 228 | Trnau1ap | intronic | . | . | . | 4qD2.3 | rs245609124 | 1 | 0 |
| chr4 | 132321628 | G | A | 228 | Trnau1ap | intronic | . | . | . | 4qD2.3 | rs32051564 | 1 | 0 |
| chr4 | 132328392 | A | G | 228 | Trnau1ap | intronic | . | . | . | 4qD2.3 | rs27557720 | 1 | 0 |
| chr4 | 132328408 | A | G | 228 | Trnau1ap | intronic | . | . | . | 4qD2.3 | rs233132534 | 1 | 0 |
| chr4 | 132328676 | A | C | 219 | Trnau1ap | intronic | . | . | . | 4qD2.3 | . | 1 | 0 |
| chr4 | 132358056 | A | T | 142 | Phactr4 | UTR3 | . | . | . | 4qD2.3 | rs27556370 | 1 | 0 |
| chr4 | 132358437 | C | T | 228 | Phactr4 | intronic | . | . | . | 4qD2.3 | rs27556368 | 1 | 0 |
| chr4 | 132360392 | C | G | 228 | Phactr4 | intronic | . | . | . | 4qD2.3 | rs27539858 | 1 | 0 |
| chr4 | 132361726 | C | T | 228 | Phactr4 | intronic | . | . | . | 4qD2.3 | rs27539855 | 1 | 0 |
| chr4 | 132361757 | A | C | 228 | Phactr4 | intronic | . | . | . | 4qD2.3 | rs27557767 | 1 | 0 |
| chr4 | 132361765 | A | C | 228 | Phactr4 | intronic | . | . | . | 4qD2.3 | rs27539854 | 1 | 0 |
| chr4 | 132363813 | C | G | 228 | Phactr4 | intronic | . | . | . | 4qD2.3 | rs27539838 | 1 | 0 |
| chr4 | 132363907 | C | T | 169 | Phactr4 | intronic | . | . | . | 4qD2.3 | rs27539837 | 1 | 0 |
| chr4 | 132365775 | A | G | 169 | Phactr4 | intronic | . | . | . | 4qD2.3 | rs27539814 | 1 | 0 |
| chr4 | 132368065 | C | G | 228 | Phactr4 | intronic | . | . | . | 4qD2.3 | rs27539803 | 1 | 0 |
| chr4 | 132368143 | G | A | 228 | Phactr4 | intronic | . | . | . | 4qD2.3 | rs27539802 | 1 | 0 |
| chr4 | 132369600 | C | T | 163 | Phactr4 | intronic | . | . | . | 4qD2.3 | rs27539796 | 1 | 0 |
| chr4 | 132369601 | T | C | 163 | Phactr4 | intronic | . | . | . | 4qD2.3 | rs27539795 | 1 | 0 |
| chr4 | 132369785 | T | C | 228 | Phactr4 | exonic | missense SNV | Phactr4:NM_001161797:exon7:c.A1450G:p.I484V,Phactr4:NM_175306:exon8:c.A1531G:p.I511V | . | 4qD2.3 | rs27539794 | 1 | 0 |
| chr4 | 132369828 | C | A | 228 | Phactr4 | exonic | synonymous SNV | Phactr4:NM_001161797:exon7:c.G1407T:p.L469L,Phactr4:NM_175306:exon8:c.G1488T:p.L496L | . | 4qD2.3 | rs27539793 | 1 | 0 |
| chr4 | 132369927 | T | C | 228 | Phactr4 | intronic | . | . | . | 4qD2.3 | rs32639204 | 1 | 0 |
| chr4 | 132370337 | C | T | 88 | Phactr4 | intronic | . | . | . | 4qD2.3 | rs27539789 | 1 | 0 |
| chr4 | 132370439 | C | T | 176 | Phactr4 | intronic | . | . | . | 4qD2.3 | rs27539788 | 1 | 0 |
| chr4 | 132370489 | G | C | 228 | Phactr4 | intronic | . | . | . | 4qD2.3 | rs27539787 | 1 | 0 |
| chr4 | 132371088 | C | G | 228 | Phactr4 | exonic | missense SNV | Phactr4:NM_001161797:exon6:c.G757C:p.V253L,Phactr4:NM_175306:exon7:c.G838C:p.V280L | . | 4qD2.3 | rs108651229 | 1 | 0 |
| chr4 | 132371155 | C | T | 214 | Phactr4 | intronic | . | . | . | 4qD2.3 | rs578555117 | 1 | 0 |
| chr4 | 132371211 | G | T | 228 | Phactr4 | intronic | . | . | . | 4qD2.3 | rs27539784 | 1 | 0 |
| chr4 | 132377246 | C | T | 228 | Phactr4 | exonic | missense SNV | Phactr4:NM_001161797:exon5:c.G658A:p.A220T,Phactr4:NM_175306:exon6:c.G739A:p.A247T | . | 4qD2.3 | rs251826878 | 1 | 0 |
| chr4 | 132378218 | C | T | 169 | Phactr4 | intronic | . | . | . | 4qD2.3 | rs32126835 | 1 | 0 |
| chr4 | 132378331 | A | T | 228 | Phactr4 | intronic | . | . | . | 4qD2.3 | rs27539753 | 1 | 0 |
| chr4 | 132413101 | T | C | 228 | Phactr4 | UTR5 | . | . | . | 4qD2.3 | rs27539651 | 1 | 0 |
| chr4 | 132459502 | T | G | 228 | Med18 | UTR3 | . | . | . | 4qD2.3 | rs27557811 | 1 | 0 |
| chr4 | 132459707 | G | A | 228 | Med18 | exonic | synonymous SNV | Med18:NM_026039:exon3:c.C480T:p.T160T | . | 4qD2.3 | . | 1 | 0 |
| chr4 | 132459932 | C | G | 228 | Med18 | exonic | synonymous SNV | Med18:NM_026039:exon3:c.G255C:p.L85L | . | 4qD2.3 | rs216467361 | 1 | 0 |
| chr4 | 132460160 | G | A | 228 | Med18 | intronic | . | . | . | 4qD2.3 | rs262102232 | 1 | 0 |
| chr4 | 132460295 | C | A | 106 | Med18 | intronic | . | . | . | 4qD2.3 | rs27557814 | 1 | 0 |
| chr4 | 132498870 | T | C | 140 | Sesn2 | intronic | . | . | . | 4qD2.3 | rs27591213 | 1 | 0 |
| chr4 | 132579262 | C | T | 228 | Ptafr | UTR5 | . | . | . | 4qD2.3 | rs51997790 | 1 | 0 |
| chr4 | 132579411 | A | G | 228 | Ptafr | exonic | synonymous SNV | Ptafr:NM_001081211:exon2:c.A111G:p.L37L | . | 4qD2.3 | rs32345808 | 1 | 0 |
| chr4 | 132579537 | C | T | 228 | Ptafr | exonic | synonymous SNV | Ptafr:NM_001081211:exon2:c.C237T:p.N79N | . | 4qD2.3 | rs51839631 | 1 | 0 |
| chr4 | 132580374 | C | T | 228 | Ptafr | UTR3 | . | . | . | 4qD2.3 | rs50370607 | 1 | 0 |
| chr4 | 132580445 | G | A | 228 | Ptafr | UTR3 | . | . | . | 4qD2.3 | rs46911083 | 1 | 0 |
| chr4 | 132580610 | T | G | 228 | Ptafr | UTR3 | . | . | . | 4qD2.3 | rs51851079 | 1 | 0 |
| chr4 | 132580698 | T | C | 228 | Ptafr | UTR3 | . | . | . | 4qD2.3 | rs48803110 | 1 | 0 |
| chr4 | 132580832 | A | G | 228 | Ptafr | UTR3 | . | . | . | 4qD2.3 | rs48995826 | 1 | 0 |
| chr4 | 132580975 | C | T | 169 | Ptafr | UTR3 | . | . | . | 4qD2.3 | rs51513717 | 1 | 0 |
| chr4 | 132661759 | G | A | 228 | Eya3 | intronic | . | . | . | 4qD2.3 | rs27539888 | 1 | 0 |
| chr4 | 132661816 | A | G | 228 | Eya3 | intronic | . | . | . | 4qD2.3 | rs32194766 | 1 | 0 |
| chr4 | 132667371 | T | C | 214 | Eya3 | intronic | . | . | . | 4qD2.3 | rs32989077 | 1 | 0 |
| chr4 | 132667468 | C | A | 176 | Eya3 | intronic | . | . | . | 4qD2.3 | . | 1 | 0 |
| chr4 | 132672942 | C | T | 228 | Eya3 | exonic | missense SNV | Eya3:NM_210071:exon6:c.C271T:p.P91S,Eya3:NM_010166:exon7:c.C223T:p.P75S | . | 4qD2.3 | rs27593913 | 1 | 0 |
| chr4 | 132741742 | G | C | 135 | Smpd13b | intronic | . | . | . | 4qD2.3 | . | 1 | 0 |
| chr4 | 132786175 | G | A | 177 | Themis2 | exonic | missense SNV | Themis2:NM_001033308:exon4:c.C739T:p.R247W | . | 4qD2.3 | . | 0 | 1 |
| chr4 | 132920700 | A | C | 228 | Fam76a | intronic | . | . | . | 4qD2.3 | . | 1 | 0 |
| chr4 | 132985990 | A | G | 121 | Fgr | intronic | . | . | . | 4qD2.3 | rs27572455 | 1 | 0 |
| chr4 | 132986260 | T | A | 228 | Fgr | exonic | missense SNV | Fgr:NM_010208:exon3:c.T98A:p.F33Y | . | 4qD2.3 | rs32143534 | 1 | 0 |
| chr4 | 132987025 | T | C | 168 | Fgr | intronic | . | . | . | 4qD2.3 | rs27572447 | 1 | 0 |
| chr4 | 132987038 | A | G | 169 | Fgr | intronic | . | . | . | 4qD2.3 | rs27572446 | 1 | 0 |
| chr4 | 132987459 | A | G | 195 | Fgr | intronic | . | . | . | 4qD2.3 | rs27572443 | 1 | 0 |
| chr4 | 132995165 | A | G | 228 | Fgr | intronic | . | . | . | 4qD2.3 | rs27572390 | 1 | 0 |
| chr4 | 132995211 | C | T | 173 | Fgr | intronic | . | . | . | 4qD2.3 | rs27572389 | 1 | 0 |
| chr4 | 132997608 | A | G | 228 | Fgr | intronic | . | . | . | 4qD2.3 | rs52316039 | 1 | 0 |
| chr4 | 132997653 | G | A | 228 | Fgr | intronic | . | . | . | 4qD2.3 | rs52351107 | 1 | 0 |
| chr4 | 132997753 | C | T | 136 | Fgr | intronic | . | . | . | 4qD2.3 | rs27572375 | 1 | 0 |
| chr4 | 132997865 | G | C | 228 | Fgr | intronic | . | . | . | 4qD2.3 | rs45869403 | 1 | 0 |
| chr4 | 132997866 | G | A | 228 | Fgr | intronic | . | . | . | 4qD2.3 | rs45692418 | 1 | 0 |
| chr4 | 132997984 | C | T | 228 | Fgr | exonic | synonymous SNV | Fgr:NM_010208:exon10:c.C999T:p.F333F | . | 4qD2.3 | rs27572370 | 1 | 0 |
| chr4 | 132998265 | A | C | 186 | Fgr | intronic | . | . | . | 4qD2.3 | rs49249472 | 1 | 0 |
| chr4 | 132998304 | A | G | 228 | Fgr | intronic | . | . | . | 4qD2.3 | rs27572369 | 1 | 0 |
| chr4 | 132998620 | A | G | 197 | Fgr | intronic | . | . | . | 4qD2.3 | rs27572367 | 1 | 0 |
| chr4 | 132999886 | T | C | 228 | Fgr | intronic | . | . | . | 4qD2.3 | rs4224803 | 1 | 0 |
| chr4 | 132999897 | A | G | 228 | Fgr | intronic | . | . | . | 4qD2.3 | rs4224804 | 1 | 0 |
| chr4 | 133000202 | T | C | 228 | Fgr | exonic | synonymous SNV | Fgr:NM_010208:exon13:c.T1413C:p.C471C | . | 4qD2.3 | rs4224807 | 1 | 0 |
| chr4 | 133000235 | G | C | 228 | Fgr | exonic | synonymous SNV | Fgr:NM_010208:exon13:c.G1446C:p.A482A | . | 4qD2.3 | rs4224808 | 1 | 0 |
| chr4 | 133061800 | G | A | 228 | Ahdc1 | exonic | synonymous SNV | Ahdc1:NM_146155:exon6:c.G351A:p.L117L | 362 | 4qD2.3 | rs27557220 | 1 | 0 |
| chr4 | 133061851 | G | C | 228 | Ahdc1 | exonic | synonymous SNV | Ahdc1:NM_146155:exon6:c.G402C:p.S134S | 362 | 4qD2.3 | rs27557219 | 1 | 0 |
| chr4 | 133061920 | G | A | 228 | Ahdc1 | exonic | synonymous SNV | Ahdc1:NM_146155:exon6:c.G471A:p.P157P | 362 | 4qD2.3 | rs45636162 | 1 | 0 |
| chr4 | 133062100 | C | T | 228 | Ahdc1 | exonic | synonymous SNV | Ahdc1:NM_146155:exon6:c.C651T:p.A217A | . | 4qD2.3 | rs27557216 | 1 | 0 |
| chr4 | 133062271 | T | C | 228 | Ahdc1 | exonic | synonymous SNV | Ahdc1:NM_146155:exon6:c.T822C:p.L274L | . | 4qD2.3 | rs49595544 | 1 | 0 |
| chr4 | 133062652 | C | G | 228 | Ahdc1 | exonic | synonymous SNV | Ahdc1:NM_146155:exon6:c.C1203G:p.G401G | . | 4qD2.3 | rs48443871 | 1 | 0 |
| chr4 | 133062741 | T | C | 228 | Ahdc1 | exonic | missense SNV | Ahdc1:NM_146155:exon6:c.T1292C:p.L431P | . | 4qD2.3 | rs32180187 | 1 | 0 |
| chr4 | 133062745 | A | G | 228 | Ahdc1 | exonic | synonymous SNV | Ahdc1:NM_146155:exon6:c.A1296G:p.P432P | . | 4qD2.3 | rs32053305 | 1 | 0 |
| chr4 | 133062952 | G | A | 228 | Ahdc1 | exonic | synonymous SNV | Ahdc1:NM_146155:exon6:c.G1503A:p.V501V | . | 4qD2.3 | rs27557213 | 1 | 0 |
| chr4 | 133063147 | T | C | 228 | Ahdc1 | exonic | synonymous SNV | Ahdc1:NM_146155:exon6:c.T1698C:p.A566A | . | 4qD2.3 | rs32477300 | 1 | 0 |
| chr4 | 133063168 | G | T | 228 | Ahdc1 | exonic | synonymous SNV | Ahdc1:NM_146155:exon6:c.G1719T:p.A573A | . | 4qD2.3 | rs31888099 | 1 | 0 |
| chr4 | 133063186 | T | C | 228 | Ahdc1 | exonic | synonymous SNV | Ahdc1:NM_146155:exon6:c.T1737C:p.P579P | . | 4qD2.3 | rs27557211 | 1 | 0 |
| chr4 | 133064326 | C | T | 228 | Ahdc1 | exonic | synonymous SNV | Ahdc1:NM_146155:exon6:c.C2877T:p.P959P | . | 4qD2.3 | rs31972171 | 1 | 0 |
| chr4 | 133064479 | T | C | 228 | Ahdc1 | exonic | synonymous SNV | Ahdc1:NM_146155:exon6:c.T3030C:p.S1010S | . | 4qD2.3 | rs27557208 | 1 | 0 |
| chr4 | 133065121 | C | T | 228 | Ahdc1 | exonic | synonymous SNV | Ahdc1:NM_146155:exon6:c.C3672T:p.P1224P | . | 4qD2.3 | rs27557207 | 1 | 0 |
| chr4 | 133065364 | T | C | 228 | Ahdc1 | exonic | synonymous SNV | Ahdc1:NM_146155:exon6:c.T3915C:p.L1305L | . | 4qD2.3 | rs13466112 | 1 | 0 |
| chr4 | 133066135 | G | T | 228 | Ahdc1 | exonic | synonymous SNV | Ahdc1:NM_146155:exon6:c.G4686T:p.P1562P | . | 4qD2.3 | rs27557206 | 1 | 0 |
| chr4 | 133087938 | A | C | 157 | . | intergenic | . | . | . | 4qD2.3 | rs51057847 | 1 | 0 |
| chr4 | 133088095 | G | A | 228 | . | intergenic | . | . | . | 4qD2.3 | rs50034566 | 1 | 0 |
| chr4 | 133088169 | T | C | 228 | . | intergenic | . | . | . | 4qD2.3 | rs50763250 | 1 | 0 |
| chr4 | 133088260 | C | T | 228 | . | intergenic | . | . | . | 4qD2.3 | rs48063255 | 1 | 0 |
| chr4 | 133176379 | G | A | 228 | Wasf2 | intronic | . | . | . | 4qD2.3 | rs27541584 | 1 | 0 |
| chr4 | 133176623 | G | C | 228 | Wasf2 | intronic | . | . | . | 4qD2.3 | rs27541583 | 1 | 0 |
| chr4 | 133184819 | A | C | 164 | Wasf2 | intronic | . | . | . | 4qD2.3 | rs27541535 | 1 | 0 |
| chr4 | 133185141 | G | C | 228 | Wasf2 | intronic | . | . | . | 4qD2.3 | rs32782636 | 1 | 0 |
| chr4 | 133185162 | T | C | 228 | Wasf2 | intronic | . | . | . | 4qD2.3 | rs32771696 | 1 | 0 |
| chr4 | 133188872 | T | C | 148 | Wasf2 | intronic | . | . | . | 4qD2.3 | rs27541521 | 1 | 0 |
| chr4 | 133189017 | T | G | 228 | Wasf2 | intronic | . | . | . | 4qD2.3 | rs27541518 | 1 | 0 |
| chr4 | 133189302 | G | A | 170 | Wasf2 | intronic | . | . | . | 4qD2.3 | rs27541515 | 1 | 0 |
| chr4 | 133189382 | C | T | 124 | Wasf2 | intronic | . | . | . | 4qD2.3 | rs217572237 | 1 | 0 |
| chr4 | 133190441 | C | T | 163 | Wasf2 | intronic | . | . | . | 4qD2.3 | rs31815926 | 1 | 0 |
| chr4 | 133192177 | A | G | 228 | Wasf2 | exonic | synonymous SNV | Wasf2:NM_153423:exon7:c.A741G:p.P247P | . | 4qD2.3 | rs13463835 | 1 | 0 |
| chr4 | 133194900 | T | C | 228 | Wasf2 | exonic | synonymous SNV | Wasf2:NM_153423:exon8:c.T1329C:p.I443I | . | 4qD2.3 | rs27541492 | 1 | 0 |
| chr4 | 133194924 | A | G | 228 | Wasf2 | intronic | . | . | . | 4qD2.3 | rs27541491 | 1 | 0 |
| chr4 | 133196019 | C | A | 155 | Wasf2 | UTR3 | . | . | . | 4qD2.3 | rs27541488 | 1 | 0 |
| chr4 | 133211435 | G | C | 116 | Gpr3 | intronic | . | . | . | 4qD2.3 | rs32159769 | 1 | 0 |
| chr4 | 133221418 | C | T | 211 | Cd16412 | intronic | . | . | . | 4qD2.3 | rs587033143 | 1 | 0 |
| chr4 | 133221490 | G | T | 228 | Cd16412 | exonic | missense SNV | Cd16412:NM_027152:exon2:c.G136T:p.V46F | . | 4qD2.3 | rs252544774 | 1 | 0 |
| chr4 | 133221509 | G | A | 228 | Cd16412 | exonic | missense SNV | Cd16412:NM_027152:exon2:c.G155A:p.G52E | . | 4qD2.3 | rs27541401 | 1 | 0 |
| chr4 | 133221647 | T | G | 228 | Cd16412 | intronic | . | . | . | 4qD2.3 | rs260946770 | 1 | 0 |
| chr4 | 133221842 | G | A | 228 | Cd16412 | intronic | . | . | . | 4qD2.3 | rs27541399 | 1 | 0 |
| chr4 | 133222350 | G | A | 160 | Cd16412 | intronic | . | . | . | 4qD2.3 | rs222398103 | 1 | 0 |
| chr4 | 133223471 | G | C | 133 | Cd16412 | intronic | . | . | . | 4qD2.3 | rs27541385 | 1 | 0 |
| chr4 | 133223523 | C | A | 228 | Cd16412 | intronic | . | . | . | 4qD2.3 | rs250586340 | 1 | 0 |
| chr4 | 133223712 | C | A | 228 | Cd16412 | exonic | synonymous SNV | Cd16412:NM_027152:exon5:c.C477A:p.R159R | . | 4qD2.3 | rs27541383 | 1 | 0 |
| chr4 | 133223817 | A | G | 228 | Cd16412 | intronic | . | . | . | 4qD2.3 | rs27541382 | 1 | 0 |
| chr4 | 133224185 | T | C | 228 | Cd16412 | intronic | . | . | . | 4qD2.3 | rs27541381 | 1 | 0 |
| chr4 | 133224203 | G | A | 228 | Cd16412 | intronic | . | . | . | 4qD2.3 | rs47495023 | 1 | 0 |
| chr4 | 133224309 | T | C | 228 | Cd16412 | UTR3 | . | . | . | 4qD2.3 | rs32627831 | 1 | 0 |
| chr4 | 133224507 | T | C | 228 | Cd16412 | UTR3 | . | . | . | 4qD2.3 | rs27541378 | 1 | 0 |
| chr4 | 133224806 | T | C | 170 | . | downstream | . | . | . | 4qD2.3 | rs32657038 | 1 | 0 |
| chr4 | 133242879 | C | T | 228 | Map3k6 | exonic | synonymous SNV | Map3k6:NM_016693:exon2:c.C405T:p.G135G | . | 4qD2.3 | rs27593613 | 1 | 0 |
| chr4 | 133243139 | C | T | 228 | Map3k6 | intronic | . | . | . | 4qD2.3 | rs27593612 | 1 | 0 |
| chr4 | 133243529 | C | T | 228 | Map3k6 | intronic | . | . | . | 4qD2.3 | rs32475675 | 1 | 0 |
| chr4 | 133243661 | A | G | 186 | Map3k6 | intronic | . | . | . | 4qD2.3 | rs52204470 | 1 | 0 |
| chr4 | 133243668 | A | G | 193 | Map3k6 | intronic | . | . | . | 4qD2.3 | rs52207884 | 1 | 0 |
| chr4 | 133244938 | T | C | 228 | Map3k6 | intronic | . | . | . | 4qD2.3 | rs27593606 | 1 | 0 |
| chr4 | 133245096 | G | A | 228 | Map3k6 | intronic | . | . | . | 4qD2.3 | rs27593605 | 1 | 0 |
| chr4 | 133245143 | G | A | 228 | Map3k6 | intronic | . | . | . | 4qD2.3 | rs27593604 | 1 | 0 |
| chr4 | 133245488 | C | T | 228 | Map3k6 | intronic | . | . | . | 4qD2.3 | rs27593602 | 1 | 0 |
| chr4 | 133245653 | C | G | 228 | Map3k6 | exonic | synonymous SNV | Map3k6:NM_016693:exon7:c.C1122G:p.G374G | . | 4qD2.3 | rs27593601 | 1 | 0 |
| chr4 | 133245850 | T | C | 228 | Map3k6 | exonic | synonymous SNV | Map3k6:NM_016693:exon8:c.T1218C:p.A406A | . | 4qD2.3 | rs27593599 | 1 | 0 |
| chr4 | 133245953 | G | A | 172 | Map3k6 | intronic | . | . | . | 4qD2.3 | rs46632304 | 1 | 0 |
| chr4 | 133245989 | T | G | 157 | Map3k6 | intronic | . | . | . | 4qD2.3 | rs27593595 | 1 | 0 |
| chr4 | 133246237 | A | G | 228 | Map3k6 | intronic | . | . | . | 4qD2.3 | rs46014799 | 1 | 0 |
| chr4 | 133246439 | C | A | 228 | Map3k6 | intronic | . | . | . | 4qD2.3 | rs27593591 | 1 | 0 |
| chr4 | 133246822 | A | G | 228 | Map3k6 | intronic | . | . | . | 4qD2.3 | rs50638896 | 1 | 0 |
| chr4 | 133247188 | C | T | 228 | Map3k6 | intronic | . | . | . | 4qD2.3 | rs27593582 | 1 | 0 |
| chr4 | 133247223 | C | A | 199 | Map3k6 | exonic | synonymous SNV | Map3k6:NM_016693:exon13:c.C1761A:p.R587R | . | 4qD2.3 | rs27593581 | 1 | 0 |

| | | | | | | | | | | | | | |
|---|---|---|---|---|---|---|---|---|---|---|---|---|---|
| chr4 | 133247324 | C | A | 228 | Map3k6 | intronic | . | . | . | 4qD2.3 | rs27593579 | 1 | 0 |
| chr4 | 133247492 | G | A | 228 | Map3k6 | exonic | synonymous SNV | Map3k6:NM_016693:exon14:c.G1914A:p.E638E | . | 4qD2.3 | rs27593577 | 1 | 0 |
| chr4 | 133247535 | T | C | 228 | Map3k6 | intronic | . | . | . | 4qD2.3 | rs48580933 | 1 | 0 |
| chr4 | 133247604 | T | C | 228 | Map3k6 | exonic | synonymous SNV | Map3k6:NM_016693:exon15:c.T1947C:p.T649T | . | 4qD2.3 | rs27593576 | 1 | 0 |
| chr4 | 133247620 | T | C | 228 | Map3k6 | exonic | synonymous SNV | Map3k6:NM_016693:exon15:c.T1963C:p.L655L | . | 4qD2.3 | rs27593575 | 1 | 0 |
| chr4 | 133247824 | A | G | 228 | Map3k6 | intronic | . | . | . | 4qD2.3 | rs27593574 | 1 | 0 |
| chr4 | 133247845 | G | A | 228 | Map3k6 | intronic | . | . | . | 4qD2.3 | . | 1 | 0 |
| chr4 | 133248437 | C | T | 228 | Map3k6 | exonic | synonymous SNV | Map3k6:NM_016693:exon17:c.C2235T:p.N745N | . | 4qD2.3 | rs27593572 | 1 | 0 |
| chr4 | 133248503 | T | C | 228 | Map3k6 | exonic | synonymous SNV | Map3k6:NM_016693:exon17:c.T2301C:p.R767R | . | 4qD2.3 | rs27593571 | 1 | 0 |
| chr4 | 133248628 | C | T | 228 | Map3k6 | intronic | . | . | . | 4qD2.3 | rs51619987 | 1 | 0 |
| chr4 | 133248659 | A | G | 228 | Map3k6 | intronic | . | . | . | 4qD2.3 | rs27593570 | 1 | 0 |
| chr4 | 133248678 | T | C | 228 | Map3k6 | intronic | . | . | . | 4qD2.3 | rs27593569 | 1 | 0 |
| chr4 | 133248851 | C | A | 228 | Map3k6 | exonic | synonymous SNV | Map3k6:NM_016693:exon18:c.C2382A:p.T794T | . | 4qD2.3 | rs27573467 | 1 | 0 |
| chr4 | 133248925 | A | G | 162 | Map3k6 | intronic | . | . | . | 4qD2.3 | rs27573466 | 1 | 0 |
| chr4 | 133249450 | T | G | 88 | Map3k6 | intronic | . | . | . | 4qD2.3 | rs27573463 | 1 | 0 |
| chr4 | 133249541 | A | C | 169 | Map3k6 | intronic | . | . | . | 4qD2.3 | rs27573462 | 1 | 0 |
| chr4 | 133249617 | A | G | 228 | Map3k6 | intronic | . | . | . | 4qD2.3 | rs27573461 | 1 | 0 |
| chr4 | 133249639 | C | T | 228 | Map3k6 | intronic | . | . | . | 4qD2.3 | rs27573460 | 1 | 0 |
| chr4 | 133249837 | A | G | 228 | Map3k6 | intronic | . | . | . | 4qD2.3 | rs47921790 | 1 | 0 |
| chr4 | 133249845 | T | C | 228 | Map3k6 | intronic | . | . | . | 4qD2.3 | rs51804094 | 1 | 0 |
| chr4 | 133250073 | A | G | 58 | Map3k6 | exonic | synonymous SNV | Map3k6:NM_016693:exon20:c.A2730G:p.G910G | . | 4qD2.3 | rs27573458 | 0 | 1 |
| chr4 | 133250168 | T | C | 166 | Map3k6 | intronic | . | . | . | 4qD2.3 | rs47204657 | 1 | 0 |
| chr4 | 133250422 | G | A | 171 | Map3k6 | intronic | . | . | . | 4qD2.3 | rs27573457 | 1 | 0 |
| chr4 | 133250445 | T | C | 115 | Map3k6 | intronic | . | . | . | 4qD2.3 | rs27573456 | 1 | 0 |
| chr4 | 133251429 | A | C | 228 | Map3k6 | exonic | missense SNV | Map3k6:NM_016693:exon24:c.A3340C:p.M1114L | 221 | 4qD2.3 | rs27573452 | 1 | 0 |
| chr4 | 133251531 | G | A | 104 | Map3k6 | intronic | . | . | 221 | 4qD2.3 | . | 0 | 1 |
| chr4 | 133251534 | T | C | 228 | Map3k6 | intronic | . | . | 221 | 4qD2.3 | rs32661337 | 1 | 0 |
| chr4 | 133251802 | G | C | 228 | Map3k6 | intronic | . | . | . | 4qD2.3 | rs27573450 | 1 | 0 |
| chr4 | 133251814 | C | T | 228 | Map3k6 | intronic | . | . | . | 4qD2.3 | rs27573449 | 1 | 0 |
| chr4 | 133251961 | A | C | 228 | Map3k6 | exonic | synonymous SNV | Map3k6:NM_016693:exon26:c.A3624C:p.S1208S | . | 4qD2.3 | rs32281313 | 1 | 0 |
| chr4 | 133252016 | A | G | 228 | Map3k6 | intronic | . | . | . | 4qD2.3 | rs27573447 | 1 | 0 |
| chr4 | 133252462 | G | C | 228 | Map3k6 | exonic | synonymous SNV | Map3k6:NM_016693:exon28:c.G3771C:p.V1257V | . | 4qD2.3 | rs27573445 | 1 | 0 |
| chr4 | 133252903 | A | G | 95 | Map3k6 | UTR3 | . | . | . | 4qD2.3 | rs27573441 | 1 | 0 |
| chr4 | 133253399 | G | T | 228 | Sytl1 | intronic | . | . | . | 4qD2.3 | rs27573439 | 1 | 0 |
| chr4 | 133253537 | C | T | 228 | Sytl1 | exonic | synonymous SNV | Sytl1:NM_031393:exon14:c.G1485A:p.Q495Q | . | 4qD2.3 | rs46463840 | 1 | 0 |
| chr4 | 133255485 | G | A | 228 | Sytl1 | intronic | . | . | . | 4qD2.3 | rs27573433 | 1 | 0 |
| chr4 | 133255521 | T | G | 228 | Sytl1 | intronic | . | . | . | 4qD2.3 | rs27573432 | 1 | 0 |
| chr4 | 133255692 | C | A | 228 | Sytl1 | intronic | . | . | . | 4qD2.3 | rs221295309 | 1 | 0 |
| chr4 | 133255693 | G | T | 228 | Sytl1 | intronic | . | . | . | 4qD2.3 | rs46921141 | 1 | 0 |
| chr4 | 133255835 | A | G | 228 | Sytl1 | intronic | . | . | . | 4qD2.3 | rs27573430 | 1 | 0 |
| chr4 | 133255963 | A | G | 228 | Sytl1 | intronic | . | . | . | 4qD2.3 | rs27573428 | 1 | 0 |
| chr4 | 133256117 | G | A | 228 | Sytl1 | exonic | synonymous SNV | Sytl1:NM_031393:exon11:c.C1107T:p.F369F | . | 4qD2.3 | rs27573424 | 1 | 0 |
| chr4 | 133256239 | G | A | 228 | Sytl1 | intronic | . | . | . | 4qD2.3 | rs27573422 | 1 | 0 |
| chr4 | 133256453 | C | T | 228 | Sytl1 | intronic | . | . | . | 4qD2.3 | rs27573420 | 1 | 0 |
| chr4 | 133256648 | A | G | 228 | Sytl1 | intronic | . | . | . | 4qD2.3 | rs27573416 | 1 | 0 |
| chr4 | 133256655 | A | C | 228 | Sytl1 | intronic | . | . | . | 4qD2.3 | rs49854212 | 1 | 0 |
| chr4 | 133257002 | C | T | 86 | Sytl1 | exonic | synonymous SNV | Sytl1:NM_031393:exon9:c.G789A:p.G263G | . | 4qD2.3 | rs240129549 | 1 | 0 |
| chr4 | 133257295 | T | C | 228 | Sytl1 | intronic | . | . | . | 4qD2.3 | rs32258715 | 1 | 0 |
| chr4 | 133257317 | G | A | 190 | Sytl1 | intronic | . | . | . | 4qD2.3 | rs32017210 | 1 | 0 |
| chr4 | 133257343 | T | C | 223 | Sytl1 | intronic | . | . | . | 4qD2.3 | rs32149735 | 1 | 0 |
| chr4 | 133257808 | A | G | 228 | Sytl1 | exonic | missense SNV | Sytl1:NM_031393:exon6:c.T476C:p.F159S | . | 4qD2.3 | rs27573405 | 1 | 0 |
| chr4 | 133258802 | A | G | 228 | Sytl1 | intronic | . | . | . | 4qD2.3 | rs27573399 | 1 | 0 |
| chr4 | 133258874 | T | C | 228 | Sytl1 | exonic | missense SNV | Sytl1:NM_031393:exon5:c.A449G:p.N150S | . | 4qD2.3 | rs27573398 | 1 | 0 |
| chr4 | 133259010 | T | C | 228 | Sytl1 | exonic | missense SNV | Sytl1:NM_031393:exon4:c.A388G:p.N130D | . | 4qD2.3 | rs27573397 | 1 | 0 |
| chr4 | 133259088 | A | G | 228 | Sytl1 | intronic | . | . | . | 4qD2.3 | rs27573396 | 1 | 0 |
| chr4 | 133259125 | C | T | 225 | Sytl1 | intronic | . | . | . | 4qD2.3 | rs27573395 | 1 | 0 |
| chr4 | 133259208 | G | C | 228 | Sytl1 | intronic | . | . | . | 4qD2.3 | rs48367717 | 1 | 0 |
| chr4 | 133259325 | G | A | 228 | Sytl1 | exonic | synonymous SNV | Sytl1:NM_031393:exon3:c.C303T:p.L101L | . | 4qD2.3 | rs13472962 | 1 | 0 |
| chr4 | 133259610 | A | G | 185 | Sytl1 | intronic | . | . | . | 4qD2.3 | rs27573394 | 1 | 0 |
| chr4 | 133260780 | T | C | 123 | Sytl1 | intronic | . | . | . | 4qD2.3 | rs27573382 | 1 | 0 |
| chr4 | 133260785 | T | C | 119 | Sytl1 | intronic | . | . | . | 4qD2.3 | rs31788964 | 1 | 0 |
| chr4 | 133260961 | G | A | 228 | Sytl1 | exonic | missense SNV | Sytl1:NM_031393:exon2:c.C83T:p.A28V | . | 4qD2.3 | rs27573381 | 1 | 0 |
| chr4 | 133267011 | G | A | 228 | Tmem222 | intronic | . | . | . | 4qD2.3 | rs27573350 | 1 | 0 |
| chr4 | 133267115 | C | G | 146 | Tmem222 | intronic | . | . | . | 4qD2.3 | rs27573349 | 1 | 0 |
| chr4 | 133267992 | T | C | 164 | Tmem222 | intronic | . | . | . | 4qD2.3 | rs27573347 | 1 | 0 |
| chr4 | 133268154 | G | A | 228 | Tmem222 | intronic | . | . | . | 4qD2.3 | rs27573346 | 1 | 0 |
| chr4 | 133268526 | G | T | 228 | Tmem222 | intronic | . | . | . | 4qD2.3 | rs31993259 | 1 | 0 |
| chr4 | 133268582 | T | C | 142 | Tmem222 | intronic | . | . | . | 4qD2.3 | rs27573345 | 1 | 0 |
| chr4 | 133268583 | G | A | 142 | Tmem222 | intronic | . | . | . | 4qD2.3 | rs32468680 | 1 | 0 |
| chr4 | 133269827 | C | T | 228 | Tmem222 | intronic | . | . | . | 4qD2.3 | rs45971635 | 1 | 0 |
| chr4 | 133269829 | G | T | 228 | Tmem222 | intronic | . | . | . | 4qD2.3 | rs46996123 | 1 | 0 |
| chr4 | 133269865 | T | A | 228 | Tmem222 | intronic | . | . | . | 4qD2.3 | rs27573337 | 1 | 0 |
| chr4 | 133277779 | A | G | 101 | Tmem222 | UTR5 | . | . | 488 | 4qD2.3 | rs27573299 | 1 | 0 |
| chr4 | 133294185 | G | C | 121 | Wdtc1 | UTR3 | . | . | . | 4qD2.3 | rs27573197 | 1 | 0 |
| chr4 | 133296355 | A | G | 218 | Wdtc1 | intronic | . | . | . | 4qD2.3 | rs27573179 | 1 | 0 |
| chr4 | 133296433 | C | T | 228 | Wdtc1 | exonic | synonymous SNV | Wdtc1:NM_199306:exon14:c.G1626A:p.E542E | . | 4qD2.3 | rs27573178 | 1 | 0 |
| chr4 | 133297313 | T | G | 174 | Wdtc1 | intronic | . | . | . | 4qD2.3 | rs27573169 | 1 | 0 |
| chr4 | 133297364 | G | A | 228 | Wdtc1 | intronic | . | . | . | 4qD2.3 | rs27573168 | 1 | 0 |
| chr4 | 133297690 | A | G | 228 | Wdtc1 | intronic | . | . | . | 4qD2.3 | rs27558167 | 1 | 0 |
| chr4 | 133300587 | T | C | 228 | Wdtc1 | exonic | synonymous SNV | Wdtc1:NM_199306:exon12:c.A1203G:p.R401R | . | 4qD2.3 | rs13468737 | 1 | 0 |
| chr4 | 133300796 | C | T | 204 | Wdtc1 | intronic | . | . | . | 4qD2.3 | rs27558157 | 1 | 0 |
| chr4 | 133300873 | T | C | 95 | Wdtc1 | intronic | . | . | . | 4qD2.3 | rs27558156 | 1 | 0 |
| chr4 | 133301857 | T | C | 228 | Wdtc1 | intronic | . | . | . | 4qD2.3 | rs27558151 | 1 | 0 |
| chr4 | 133302328 | A | G | 228 | Wdtc1 | intronic | . | . | . | 4qD2.3 | rs27558150 | 1 | 0 |
| chr4 | 133302369 | A | G | 228 | Wdtc1 | intronic | . | . | . | 4qD2.3 | rs51083156 | 1 | 0 |
| chr4 | 133302458 | A | G | 228 | Wdtc1 | splicing | . | . | . | 4qD2.3 | rs49052716 | 1 | 0 |
| chr4 | 133302471 | G | T | 228 | Wdtc1 | intronic | . | . | . | 4qD2.3 | rs48294795 | 1 | 0 |
| chr4 | 133302581 | C | T | 193 | Wdtc1 | intronic | . | . | . | 4qD2.3 | rs27558149 | 1 | 0 |
| chr4 | 133302607 | C | T | 155 | Wdtc1 | intronic | . | . | . | 4qD2.3 | rs46919534 | 1 | 0 |
| chr4 | 133303434 | G | A | 214 | Wdtc1 | intronic | . | . | . | 4qD2.3 | rs27558148 | 1 | 0 |
| chr4 | 133303451 | C | G | 228 | Wdtc1 | intronic | . | . | . | 4qD2.3 | rs48127795 | 1 | 0 |
| chr4 | 133303656 | C | T | 228 | Wdtc1 | exonic | synonymous SNV | Wdtc1:NM_199306:exon9:c.G768A:p.P256P | . | 4qD2.3 | rs49881708 | 1 | 0 |
| chr4 | 133303729 | C | T | 228 | Wdtc1 | intronic | . | . | . | 4qD2.3 | rs27558147 | 1 | 0 |
| chr4 | 133303841 | C | T | 172 | Wdtc1 | intronic | . | . | . | 4qD2.3 | rs46541584 | 1 | 0 |
| chr4 | 133304097 | C | T | 228 | Wdtc1 | intronic | . | . | . | 4qD2.3 | rs230875658 | 1 | 0 |
| chr4 | 133304311 | C | A | 161 | Wdtc1 | intronic | . | . | . | 4qD2.3 | rs27558142 | 1 | 0 |
| chr4 | 133305811 | T | C | 228 | Wdtc1 | intronic | . | . | . | 4qD2.3 | rs48746413 | 1 | 0 |
| chr4 | 133306022 | G | A | 228 | Wdtc1 | intronic | . | . | . | 4qD2.3 | rs27558139 | 1 | 0 |
| chr4 | 133308820 | C | T | 228 | Wdtc1 | exonic | synonymous SNV | Wdtc1:NM_199306:exon6:c.G408A:p.R136R | . | 4qD2.3 | rs27558122 | 1 | 0 |
| chr4 | 133308871 | G | A | 228 | Wdtc1 | exonic | synonymous SNV | Wdtc1:NM_199306:exon6:c.C357T:p.H119H | . | 4qD2.3 | rs27558121 | 1 | 0 |
| chr4 | 133308975 | G | T | 228 | Wdtc1 | intronic | . | . | . | 4qD2.3 | rs27558120 | 1 | 0 |
| chr4 | 133308997 | A | C | 228 | Wdtc1 | intronic | . | . | . | 4qD2.3 | rs27558119 | 1 | 0 |
| chr4 | 133313283 | T | C | 228 | Wdtc1 | intronic | . | . | . | 4qD2.3 | rs46958560 | 1 | 0 |
| chr4 | 133313288 | C | T | 228 | Wdtc1 | intronic | . | . | . | 4qD2.3 | rs49910218 | 1 | 0 |
| chr4 | 133313729 | C | A | 228 | Wdtc1 | intronic | . | . | . | 4qD2.3 | rs47020315 | 1 | 0 |
| chr4 | 133313838 | A | G | 228 | Wdtc1 | intronic | . | . | . | 4qD2.3 | rs27558101 | 1 | 0 |
| chr4 | 133323533 | G | A | 112 | Wdtc1 | intronic | . | . | . | 4qD2.3 | rs50468598 | 1 | 0 |
| chr4 | 133324008 | A | G | 166 | Wdtc1 | intronic | . | . | . | 4qD2.3 | rs27558058 | 1 | 0 |
| chr4 | 133324019 | A | G | 154 | Wdtc1 | intronic | . | . | . | 4qD2.3 | rs47743244 | 1 | 0 |
| chr4 | 133370806 | C | T | 147 | Slc9a1 | exonic | missense SNV | Slc9a1:NM_016981:exon1:c.C263T:p.P88L | . | 4qD2.3 | . | 0 | 1 |
| chr4 | 133411928 | C | T | 228 | Slc9a1 | exonic | synonymous SNV | Slc9a1:NM_016981:exon2:c.C456T:p.G152G | . | 4qD2.3 | rs49473772 | 1 | 0 |
| chr4 | 133412189 | T | C | 228 | Slc9a1 | exonic | synonymous SNV | Slc9a1:NM_016981:exon2:c.T717C:p.S239S | . | 4qD2.3 | rs27541303 | 1 | 0 |
| chr4 | 133412201 | T | C | 228 | Slc9a1 | exonic | synonymous SNV | Slc9a1:NM_016981:exon2:c.T729C:p.P243P | . | 4qD2.3 | rs27541302 | 1 | 0 |
| chr4 | 133412313 | T | C | 228 | Slc9a1 | intronic | . | . | . | 4qD2.3 | rs32082812 | 1 | 0 |
| chr4 | 133415225 | T | C | 160 | Slc9a1 | intronic | . | . | . | 4qD2.3 | rs27541288 | 1 | 0 |
| chr4 | 133415236 | A | G | 160 | Slc9a1 | intronic | . | . | . | 4qD2.3 | rs27541287 | 1 | 0 |
| chr4 | 133415429 | A | C | 228 | Slc9a1 | exonic | synonymous SNV | Slc9a1:NM_016981:exon3:c.A954C:p.V318V | . | 4qD2.3 | rs27541286 | 1 | 0 |
| chr4 | 133415459 | T | C | 228 | Slc9a1 | exonic | synonymous SNV | Slc9a1:NM_016981:exon3:c.T984C:p.S328S | . | 4qD2.3 | rs27541284 | 1 | 0 |
| chr4 | 133415601 | G | A | 228 | Slc9a1 | intronic | . | . | . | 4qD2.3 | rs27541283 | 1 | 0 |
| chr4 | 133415610 | G | A | 228 | Slc9a1 | intronic | . | . | . | 4qD2.3 | rs45764512 | 1 | 0 |
| chr4 | 133415656 | G | A | 228 | Slc9a1 | intronic | . | . | . | 4qD2.3 | rs27541282 | 1 | 0 |
| chr4 | 133416126 | C | T | 101 | Slc9a1 | intronic | . | . | . | 4qD2.3 | rs27541276 | 1 | 0 |
| chr4 | 133416237 | A | G | 225 | Slc9a1 | intronic | . | . | . | 4qD2.3 | rs27541275 | 1 | 0 |
| chr4 | 133419367 | T | C | 228 | Slc9a1 | intronic | . | . | . | 4qD2.3 | rs32979117 | 1 | 0 |
| chr4 | 133419372 | G | A | 228 | Slc9a1 | intronic | . | . | . | 4qD2.3 | rs32938061 | 1 | 0 |
| chr4 | 133419781 | G | A | 214 | Slc9a1 | intronic | . | . | . | 4qD2.3 | rs27541254 | 1 | 0 |
| chr4 | 133419859 | A | G | 228 | Slc9a1 | intronic | . | . | . | 4qD2.3 | rs32185248 | 1 | 0 |
| chr4 | 133420021 | G | A | 228 | Slc9a1 | intronic | . | . | . | 4qD2.3 | rs47050421 | 1 | 0 |
| chr4 | 133420035 | T | G | 228 | Slc9a1 | intronic | . | . | . | 4qD2.3 | rs32917554 | 1 | 0 |
| chr4 | 133420057 | C | T | 228 | Slc9a1 | intronic | . | . | . | 4qD2.3 | rs32024706 | 1 | 0 |
| chr4 | 133420369 | A | G | 228 | Slc9a1 | intronic | . | . | . | 4qD2.3 | rs27541252 | 1 | 0 |
| chr4 | 133420631 | T | C | 228 | Slc9a1 | intronic | . | . | . | 4qD2.3 | rs27541251 | 1 | 0 |
| chr4 | 133420768 | T | C | 155 | Slc9a1 | intronic | . | . | . | 4qD2.3 | rs27541250 | 1 | 0 |
| chr4 | 133421654 | A | G | 203 | Slc9a1 | intronic | . | . | . | 4qD2.3 | rs27541243 | 1 | 0 |
| chr4 | 133421676 | G | A | 200 | Slc9a1 | intronic | . | . | . | 4qD2.3 | rs27541242 | 1 | 0 |
| chr4 | 133422781 | A | G | 146 | Slc9a1 | UTR3 | . | . | . | 4qD2.3 | rs27541241 | 1 | 0 |
| chr4 | 133422800 | C | T | 200 | Slc9a1 | UTR3 | . | . | . | 4qD2.3 | rs27541240 | 1 | 0 |
| chr4 | 133480424 | C | T | 179 | Fam46b | exonic | missense SNV | Fam46b:NM_175307:exon1:c.C125T:p.S42L | 453 | 4qD2.3 | . | 1 | 0 |
| chr4 | 133486055 | G | A | 227 | Fam46b | intronic | . | . | . | 4qD2.3 | rs32320807 | 1 | 0 |
| chr4 | 133486086 | G | A | 228 | Fam46b | exonic | synonymous SNV | Fam46b:NM_175307:exon2:c.G267A:p.V89V | . | 4qD2.3 | rs27598696 | 1 | 0 |
| chr4 | 133486232 | T | C | 228 | Fam46b | exonic | missense SNV | Fam46b:NM_175307:exon2:c.T413C:p.V138A | . | 4qD2.3 | rs27598695 | 1 | 0 |
| chr4 | 133486528 | A | T | 228 | Fam46b | exonic | missense SNV | Fam46b:NM_175307:exon2:c.A709T:p.M237L | 547 | 4qD2.3 | rs27598694 | 1 | 0 |
| chr4 | 133486563 | G | A | 228 | Fam46b | exonic | synonymous SNV | Fam46b:NM_175307:exon2:c.G744A:p.E248E | 547 | 4qD2.3 | rs27598693 | 1 | 0 |
| chr4 | 133486786 | C | T | 228 | Fam46b | exonic | synonymous SNV | Fam46b:NM_175307:exon2:c.C967T:p.L323L | 547 | 4qD2.3 | rs27598692 | 1 | 0 |
| chr4 | 133527888 | T | G | 218 | Kdf1 | intronic | . | . | . | 4qD2.3 | rs13474463 | 1 | 0 |
| chr4 | 133528252 | A | C | 170 | Kdf1 | exonic | synonymous SNV | Kdf1:NM_001083916:exon2:c.A279C:p.R93R,Kdf1:NM_133707:exon2:c.A279C:p.R93R | . | 4qD2.3 | rs46924675 | 1 | 0 |
| chr4 | 133528312 | G | A | 226 | Kdf1 | exonic | synonymous SNV | Kdf1:NM_001083916:exon2:c.G339A:p.L113L,Kdf1:NM_133707:exon2:c.G339A:p.L113L | . | 4qD2.3 | rs27575516 | 1 | 0 |
| chr4 | 133528387 | A | G | 228 | Kdf1 | exonic | synonymous SNV | Kdf1:NM_001083916:exon2:c.A414G:p.P138P,Kdf1:NM_133707:exon2:c.A414G:p.P138P | . | 4qD2.3 | rs51918174 | 1 | 0 |
| chr4 | 133528432 | A | C | 228 | Kdf1 | exonic | synonymous SNV | Kdf1:NM_001083916:exon2:c.A459C:p.T153T,Kdf1:NM_133707:exon2:c.A459C:p.T153T | . | 4qD2.3 | rs27575515 | 1 | 0 |
| chr4 | 133528567 | G | A | 228 | Kdf1 | exonic | synonymous SNV | Kdf1:NM_001083916:exon2:c.G594A:p.T198T,Kdf1:NM_133707:exon2:c.G594A:p.T198T | . | 4qD2.3 | rs27575513 | 1 | 0 |
| chr4 | 133529949 | C | T | 228 | Kdf1 | intronic | . | . | . | 4qD2.3 | rs27575507 | 1 | 0 |
| chr4 | 133530181 | A | G | 147 | Kdf1 | intronic | . | . | . | 4qD2.3 | rs27575504 | 1 | 0 |
| chr4 | 133532477 | G | A | 146 | . | downstream | . | . | . | 4qD2.3 | rs27575489 | 1 | 0 |
| chr4 | 133532717 | C | T | 228 | Nudc | UTR3 | . | . | . | 4qD2.3 | rs27575488 | 1 | 0 |
| chr4 | 133532861 | T | C | 228 | Nudc | intronic | . | . | . | 4qD2.3 | rs47596575 | 1 | 0 |
| chr4 | 133532881 | G | A | 228 | Nudc | intronic | . | . | . | 4qD2.3 | rs27575487 | 1 | 0 |
| chr4 | 133533564 | T | C | 228 | Nudc | intronic | . | . | . | 4qD2.3 | rs47495788 | 1 | 0 |
| chr4 | 133533711 | A | G | 116 | Nudc | intronic | . | . | . | 4qD2.3 | rs51565379 | 1 | 0 |
| chr4 | 133533723 | T | A | 70 | Nudc | intronic | . | . | . | 4qD2.3 | rs27575483 | 1 | 0 |
| chr4 | 133534486 | G | A | 166 | Nudc | exonic | synonymous SNV | Nudc:NM_010948:exon5:c.C486T:p.P162P | . | 4qD2.3 | rs13463906 | 1 | 0 |
| chr4 | 133535413 | G | A | 206 | Nudc | intronic | . | . | . | 4qD2.3 | rs32810997 | 1 | 0 |
| chr4 | 133535450 | T | G | 228 | Nudc | intronic | . | . | . | 4qD2.3 | rs32552840 | 1 | 0 |
| chr4 | 133535890 | C | T | 228 | Nudc | intronic | . | . | . | 4qD2.3 | rs27575477 | 1 | 0 |
| chr4 | 133535913 | A | C | 228 | Nudc | intronic | . | . | . | 4qD2.3 | rs27575475 | 1 | 0 |
| chr4 | 133536025 | A | C | 116 | Nudc | intronic | . | . | . | 4qD2.3 | rs49817697 | 1 | 0 |
| chr4 | 133541121 | G | A | 228 | Nudc | intronic | . | . | . | 4qD2.3 | rs31794257 | 1 | 0 |
| chr4 | 133541130 | C | G | 228 | Nudc | intronic | . | . | . | 4qD2.3 | rs31910435 | 1 | 0 |
| chr4 | 133541311 | A | G | 228 | Nudc | intronic | . | . | . | 4qD2.3 | rs27575440 | 1 | 0 |
| chr4 | 133553727 | G | T | 228 | Nr0b2 | exonic | synonymous SNV | Nr0b2:NM_011850:exon1:c.G303T:p.L101L | . | 4qD2.3 | rs13470145 | 1 | 0 |
| chr4 | 133556113 | C | T | 228 | Nr0b2 | exonic | synonymous SNV | Nr0b2:NM_011850:exon2:c.C660T:p.I220I | . | 4qD2.3 | rs27575423 | 1 | 0 |
| chr4 | 133556174 | C | T | 228 | Nr0b2 | exonic | missense SNV | Nr0b2:NM_011850:exon2:c.C721T:p.R241C | . | 4qD2.3 | rs16808235 | 1 | 0 |
| chr4 | 133574701 | C | T | 121 | . | upstream | . | . | . | 4qD2.3 | rs236703149 | 1 | 0 |
| chr4 | 133574736 | C | T | 204 | . | upstream | . | . | 536 | 4qD2.3 | rs46457677 | 1 | 0 |
| chr4 | 133575019 | G | C | 157 | Gpatch3 | exonic | missense SNV | Gpatch3:NM_172876:exon1:c.G260C:p.R87P | 536 | 4qD2.3 | rs49536004 | 1 | 0 |
| chr4 | 133575047 | A | G | 121 | Gpatch3 | exonic | synonymous SNV | Gpatch3:NM_172876:exon1:c.A288G:p.A96A | 536 | 4qD2.3 | rs51539381 | 1 | 0 |
| chr4 | 133575205 | T | C | 161 | Gpatch3 | exonic | missense SNV | Gpatch3:NM_172876:exon1:c.T446C:p.V149A | 536 | 4qD2.3 | rs48815118 | 1 | 0 |
| chr4 | 133577959 | G | C | 228 | Gpatch3 | exonic | synonymous SNV | Gpatch3:NM_172876:exon2:c.G504C:p.A168A | . | 4qD2.3 | rs30776626 | 1 | 0 |
| chr4 | 133578030 | A | G | 228 | Gpatch3 | exonic | missense SNV | Gpatch3:NM_172876:exon2:c.A575G:p.N192S | . | 4qD2.3 | rs51086162 | 1 | 0 |
| chr4 | 133578376 | C | G | 126 | Gpatch3 | intronic | . | . | . | 4qD2.3 | rs50475708 | 1 | 0 |
| chr4 | 133579639 | G | A | 133 | Gpatch3 | intronic | . | . | . | 4qD2.3 | rs47637899 | 1 | 0 |
| chr4 | 133579757 | C | G | 228 | Gpatch3 | intronic | . | . | . | 4qD2.3 | rs46994281 | 1 | 0 |
| chr4 | 133579796 | G | A | 228 | Gpatch3 | exonic | missense SNV | Gpatch3:NM_172876:exon3:c.G890A:p.R297Q | . | 4qD2.3 | rs50114802 | 1 | 0 |
| chr4 | 133579855 | A | G | 228 | Gpatch3 | exonic | missense SNV | Gpatch3:NM_172876:exon3:c.A949G:p.T317A | . | 4qD2.3 | rs49464520 | 1 | 0 |
| chr4 | 133579935 | T | C | 228 | Gpatch3 | exonic | synonymous SNV | Gpatch3:NM_172876:exon3:c.T1029C:p.A343A | . | 4qD2.3 | rs48249290 | 1 | 0 |
| chr4 | 133580694 | C | T | 228 | Gpatch3 | exonic | synonymous SNV | Gpatch3:NM_172876:exon4:c.C1098T:p.S366S | . | 4qD2.3 | rs49048141 | 1 | 0 |

| | | | | | | | | | | | | | |
|---|---|---|---|---|---|---|---|---|---|---|---|---|---|
| chr4 | 133580832 | A | C | 228 | Gpatch3 | intronic | . | . | . | 4qD2.3 | rs47891391 | 1 | 0 |
| chr4 | 133582467 | T | C | 228 | Gpatch3 | intronic | . | . | . | 4qD2.3 | rs27575365 | 1 | 0 |
| chr4 | 133582561 | G | C | 228 | Gpatch3 | exonic | missense SNV | Gpatch3:NM_172876:exon5:c.G1204C:p.G402R | . | 4qD2.3 | rs27575364 | 1 | 0 |
| chr4 | 133582590 | C | T | 228 | Gpatch3 | exonic | synonymous SNV | Gpatch3:NM_172876:exon5:c.C1233T:p.T411T | . | 4qD2.3 | rs27575363 | 1 | 0 |
| chr4 | 133583086 | T | C | 228 | Gpatch3 | exonic | synonymous SNV | Gpatch3:NM_172876:exon6:c.T1341C:p.H447H | . | 4qD2.3 | rs27575359 | 1 | 0 |
| chr4 | 133583186 | A | G | 228 | Gpatch3 | intronic | . | . | . | 4qD2.3 | rs32890938 | 1 | 0 |
| chr4 | 133583654 | T | C | 228 | Gpatch3 | exonic | missense SNV | Gpatch3:NM_172876:exon7:c.T1400C:p.L467P | . | 4qD2.3 | rs27575351 | 1 | 0 |
| chr4 | 133583775 | A | G | 228 | Gpatch3 | exonic | synonymous SNV | Gpatch3:NM_172876:exon7:c.A1521G:p.T507T | . | 4qD2.3 | rs27575349 | 1 | 0 |
| chr4 | 133583905 | G | C | 225 | Gpatch3 | UTR3 | . | . | . | 4qD2.3 | rs27575347 | 1 | 0 |
| chr4 | 133584891 | A | C | 101 | Gpn2 | intronic | . | . | . | 4qD2.3 | rs27575342 | 1 | 0 |
| chr4 | 133585924 | C | T | 228 | Gpn2 | exonic | synonymous SNV | Gpn2:NM_001290742:exon2:c.C357T:p.H119H,Gpn2:NM_133884:exon2:c.C426T:p.H142H | . | 4qD2.3 | rs13472419 | 1 | 0 |
| chr4 | 133586018 | A | G | 228 | Gpn2 | exonic | missense SNV | Gpn2:NM_001290742:exon2:c.A451G:p.I151V,Gpn2:NM_133884:exon2:c.A520G:p.I174V | . | 4qD2.3 | rs27575336 | 1 | 0 |
| chr4 | 133588440 | T | C | 228 | Gpn2 | intronic | . | . | . | 4qD2.3 | rs32217960 | 1 | 0 |
| chr4 | 133588461 | C | A | 228 | Gpn2 | intronic | . | . | . | 4qD2.3 | rs27575312 | 1 | 0 |
| chr4 | 133588599 | A | G | 228 | Gpn2 | exonic | missense SNV | Gpn2:NM_001290742:exon3:c.A599G:p.E200G,Gpn2:NM_133884:exon3:c.A668G:p.E223G | . | 4qD2.3 | rs27575310 | 1 | 0 |
| chr4 | 133588606 | G | A | 228 | Gpn2 | exonic | synonymous SNV | Gpn2:NM_001290742:exon3:c.G606A:p.L202L,Gpn2:NM_133884:exon3:c.G675A:p.L225L | . | 4qD2.3 | rs13472423 | 1 | 0 |
| chr4 | 133588636 | C | G | 228 | Gpn2 | exonic | synonymous SNV | Gpn2:NM_001290742:exon3:c.C636G:p.V212V,Gpn2:NM_133884:exon3:c.C705G:p.V235V | . | 4qD2.3 | rs13472422 | 1 | 0 |
| chr4 | 133589637 | A | G | 201 | Gpn2 | intronic | . | . | . | 4qD2.3 | rs46125090 | 1 | 0 |
| chr4 | 133589693 | C | G | 228 | Gpn2 | intronic | . | . | . | 4qD2.3 | rs27575296 | 1 | 0 |
| chr4 | 133589811 | T | C | 228 | Gpn2 | exonic | synonymous SNV | Gpn2:NM_001290742:exon4:c.T705C:p.A235A,Gpn2:NM_133884:exon4:c.T774C:p.A258A | . | 4qD2.3 | rs27575294 | 1 | 0 |
| chr4 | 133589914 | T | C | 228 | Gpn2 | intronic | . | . | . | 4qD2.3 | rs27575292 | 1 | 0 |
| chr4 | 133589943 | C | T | 228 | Gpn2 | intronic | . | . | . | 4qD2.3 | rs27575291 | 1 | 0 |
| chr4 | 133601473 | T | C | 228 | Sfn | exonic | synonymous SNV | Sfn:NM_018754:exon1:c.A297G:p.V99V | 360 | 4qD2.3 | rs32935330 | 1 | 0 |
| chr4 | 133601476 | G | A | 228 | Sfn | exonic | synonymous SNV | Sfn:NM_018754:exon1:c.C294T:p.T98T | 360 | 4qD2.3 | rs32465812 | 1 | 0 |
| chr4 | 133601629 | A | G | 228 | Sfn | exonic | synonymous SNV | Sfn:NM_018754:exon1:c.T141C:p.A47A | 360 | 4qD2.3 | rs31992884 | 1 | 0 |
| chr4 | 133601683 | G | A | 228 | Sfn | exonic | synonymous SNV | Sfn:NM_018754:exon1:c.C87T:p.A29A | 360 | 4qD2.3 | rs13463296 | 1 | 0 |
| chr4 | 133602279 | C | T | 178 | . | upstream | . | . | . | 4qD2.3 | rs31931337 | 1 | 0 |
| chr4 | 133608638 | G | A | 228 | Zdhhc18 | UTR3 | . | . | . | 4qD2.3 | rs27559936 | 1 | 0 |
| chr4 | 133610214 | G | A | 167 | Zdhhc18 | intronic | . | . | . | 4qD2.3 | rs27559929 | 1 | 0 |
| chr4 | 133610543 | A | G | 228 | Zdhhc18 | intronic | . | . | . | 4qD2.3 | rs32349935 | 1 | 0 |
| chr4 | 133613402 | A | G | 111 | Zdhhc18 | intronic | . | . | . | 4qD2.3 | rs27559905 | 1 | 0 |
| chr4 | 133613518 | C | T | 228 | Zdhhc18 | intronic | . | . | . | 4qD2.3 | rs27559904 | 1 | 0 |
| chr4 | 133613627 | C | G | 228 | Zdhhc18 | intronic | . | . | . | 4qD2.3 | rs27559903 | 1 | 0 |
| chr4 | 133613817 | G | A | 228 | Zdhhc18 | exonic | synonymous SNV | Zdhhc18:NM_001017968:exon4:c.C744T:p.V248V | . | 4qD2.3 | rs27559902 | 1 | 0 |
| chr4 | 133614028 | C | T | 228 | Zdhhc18 | intronic | . | . | . | 4qD2.3 | rs3721799 | 1 | 0 |
| chr4 | 133615262 | T | C | 228 | Zdhhc18 | exonic | synonymous SNV | Zdhhc18:NM_001017968:exon3:c.A540G:p.T180T | . | 4qD2.3 | rs27559893 | 1 | 0 |
| chr4 | 133615455 | G | A | 211 | Zdhhc18 | intronic | . | . | . | 4qD2.3 | rs50223957 | 1 | 0 |
| chr4 | 133615520 | T | A | 110 | Zdhhc18 | intronic | . | . | . | 4qD2.3 | rs46463227 | 1 | 0 |
| chr4 | 133615542 | A | G | 101 | Zdhhc18 | intronic | . | . | . | 4qD2.3 | rs27559892 | 1 | 0 |
| chr4 | 133627172 | C | T | 228 | Zdhhc18 | intronic | . | . | . | 4qD2.3 | rs27559841 | 1 | 0 |
| chr4 | 133627207 | C | T | 228 | Zdhhc18 | exonic | synonymous SNV | Zdhhc18:NM_001017968:exon2:c.G465A:p.K155K | . | 4qD2.3 | rs27559840 | 1 | 0 |
| chr4 | 133627387 | T | C | 228 | Zdhhc18 | intronic | . | . | . | 4qD2.3 | rs49201244 | 1 | 0 |
| chr4 | 133633033 | G | A | 123 | Zdhhc18 | intronic | . | . | 259 | 4qD2.3 | rs234960762 | 1 | 0 |
| chr4 | 133638554 | G | A | 228 | . | intergenic | . | . | . | 4qD2.3 | rs46285712 | 1 | 0 |
| chr4 | 133638709 | A | G | 228 | . | intergenic | . | . | . | 4qD2.3 | rs45645898 | 1 | 0 |
| chr4 | 133662493 | T | A | 228 | Pigv | UTR3 | . | . | . | 4qD2.3 | rs50626422 | 1 | 0 |
| chr4 | 133662494 | T | C | 228 | Pigv | UTR3 | . | . | . | 4qD2.3 | rs27559751 | 1 | 0 |
| chr4 | 133662814 | A | G | 228 | Pigv | intronic | . | . | . | 4qD2.3 | rs27559748 | 1 | 0 |
| chr4 | 133664611 | A | T | 228 | Pigv | intronic | . | . | . | 4qD2.3 | rs46845894 | 1 | 0 |
| chr4 | 133665126 | T | C | 228 | Pigv | exonic | synonymous SNV | Pigv:NM_178698:exon3:c.A732G:p.S244S | . | 4qD2.3 | rs32370790 | 1 | 0 |
| chr4 | 133665401 | A | G | 228 | Pigv | exonic | synonymous SNV | Pigv:NM_178698:exon3:c.T457C:p.L153L | . | 4qD2.3 | rs3694632 | 1 | 0 |
| chr4 | 133669640 | A | G | 228 | Pigv | intronic | . | . | . | 4qD2.3 | rs32192540 | 1 | 0 |
| chr4 | 133669706 | T | C | 228 | Pigv | intronic | . | . | . | 4qD2.3 | rs32339849 | 1 | 0 |
| chr4 | 133669895 | C | T | 228 | Pigv | intronic | . | . | . | 4qD2.3 | rs32758792 | 1 | 0 |
| chr4 | 133669929 | T | C | 202 | Pigv | intronic | . | . | . | 4qD2.3 | rs32144750 | 1 | 0 |
| chr4 | 133681919 | T | G | 228 | Arid1a | exonic | missense SNV | Arid1a:NM_001080819:exon20:c.A5264C:p.Y1755S | . | 4qD2.3 | rs27559728 | 1 | 0 |
| chr4 | 133684198 | T | G | 160 | Arid1a | intronic | . | . | . | 4qD2.3 | rs27559720 | 1 | 0 |
| chr4 | 133684211 | G | A | 164 | Arid1a | intronic | . | . | . | 4qD2.3 | rs27559719 | 1 | 0 |
| chr4 | 133686344 | G | T | 228 | Arid1a | intronic | . | . | . | 4qD2.3 | rs27559717 | 1 | 0 |
| chr4 | 133686980 | A | G | 150 | Arid1a | intronic | . | . | . | 4qD2.3 | rs49380318 | 1 | 0 |
| chr4 | 133688889 | C | A | 164 | Arid1a | intronic | . | . | . | 4qD2.3 | rs27559705 | 1 | 0 |
| chr4 | 133688907 | A | C | 166 | Arid1a | intronic | . | . | . | 4qD2.3 | rs27559704 | 1 | 0 |
| chr4 | 133690988 | C | A | 218 | Arid1a | intronic | . | . | . | 4qD2.3 | rs27559699 | 1 | 0 |
| chr4 | 133693611 | C | T | 169 | Arid1a | intronic | . | . | . | 4qD2.3 | rs27559692 | 1 | 0 |
| chr4 | 133694079 | A | G | 228 | Arid1a | intronic | . | . | . | 4qD2.3 | rs32159794 | 1 | 0 |
| chr4 | 133694616 | C | T | 228 | Arid1a | intronic | . | . | . | 4qD2.3 | rs27559688 | 1 | 0 |
| chr4 | 133694943 | T | C | 178 | Arid1a | intronic | . | . | . | 4qD2.3 | rs27559687 | 1 | 0 |
| chr4 | 133695416 | C | G | 121 | Arid1a | intronic | . | . | . | 4qD2.3 | rs27559686 | 1 | 0 |
| chr4 | 133695809 | T | C | 228 | Arid1a | intronic | . | . | . | 4qD2.3 | rs27559685 | 1 | 0 |
| chr4 | 133695876 | G | A | 122 | Arid1a | intronic | . | . | . | 4qD2.3 | rs27559684 | 1 | 0 |
| chr4 | 133719287 | A | C | 179 | Arid1a | intronic | . | . | . | 4qD2.3 | rs27542802 | 1 | 0 |
| chr4 | 133720405 | C | T | 139 | Arid1a | intronic | . | . | . | 4qD2.3 | rs27542801 | 1 | 0 |
| chr4 | 133720457 | T | G | 163 | Arid1a | intronic | . | . | . | 4qD2.3 | rs27542800 | 1 | 0 |
| chr4 | 133720542 | T | C | 228 | Arid1a | intronic | . | . | . | 4qD2.3 | rs27542798 | 1 | 0 |
| chr4 | 133720621 | C | A | 228 | Arid1a | exonic | missense SNV | Arid1a:NM_001080819:exon3:c.G1735T:p.A579S | . | 4qD2.3 | rs50925529 | 1 | 0 |
| chr4 | 133720622 | A | G | 228 | Arid1a | exonic | synonymous SNV | Arid1a:NM_001080819:exon3:c.T1734C:p.A578A | . | 4qD2.3 | rs27542797 | 1 | 0 |
| chr4 | 133723133 | T | C | 228 | Arid1a | intronic | . | . | . | 4qD2.3 | rs27542794 | 1 | 0 |
| chr4 | 133723163 | A | T | 147 | Arid1a | intronic | . | . | . | 4qD2.3 | rs108670366 | 0 | 1 |
| chr4 | 133848163 | C | T | 228 | Rps6ka1 | exonic | synonymous SNV | Rps6ka1:NM_001285506:exon21:c.G2049A:p.A683A,Rps6ka1:NM_001285505:exon22:c.G2064A:p.A688A,Rps | . | 4qD2.3 | rs13459958 | 1 | 0 |
| chr4 | 133848408 | A | T | 228 | Rps6ka1 | intronic | . | . | . | 4qD2.3 | rs27546602 | 1 | 0 |
| chr4 | 133848686 | A | C | 228 | Rps6ka1 | intronic | . | . | . | 4qD2.3 | rs50174166 | 1 | 0 |
| chr4 | 133850546 | G | A | 168 | Rps6ka1 | intronic | . | . | . | 4qD2.3 | rs27546587 | 1 | 0 |
| chr4 | 133851297 | G | A | 228 | Rps6ka1 | exonic | synonymous SNV | Rps6ka1:NM_001285506:exon17:c.C1572T:p.N524N,Rps6ka1:NM_001285505:exon18:c.C1587T:p.N529N,Rps | . | 4qD2.3 | rs13459952 | 1 | 0 |
| chr4 | 133851382 | A | C | 228 | Rps6ka1 | intronic | . | . | . | 4qD2.3 | rs27546584 | 1 | 0 |
| chr4 | 133851447 | T | A | 228 | Rps6ka1 | intronic | . | . | . | 4qD2.3 | rs46407149 | 1 | 0 |
| chr4 | 133859969 | C | G | 228 | Rps6ka1 | intronic | . | . | . | 4qD2.3 | rs27546531 | 1 | 0 |
| chr4 | 133860128 | T | C | 228 | Rps6ka1 | exonic | synonymous SNV | Rps6ka1:NM_001285506:exon16:c.A1467G:p.K489K,Rps6ka1:NM_001285505:exon17:c.A1482G:p.K494K,Rps | . | 4qD2.3 | rs13459949 | 1 | 0 |
| chr4 | 133860354 | G | T | 228 | Rps6ka1 | intronic | . | . | . | 4qD2.3 | rs47664181 | 1 | 0 |
| chr4 | 133860419 | C | T | 228 | Rps6ka1 | intronic | . | . | . | 4qD2.3 | rs27546525 | 1 | 0 |
| chr4 | 133860627 | C | T | 228 | Rps6ka1 | intronic | . | . | . | 4qD2.3 | rs51832107 | 1 | 0 |
| chr4 | 133860794 | A | C | 228 | Rps6ka1 | intronic | . | . | . | 4qD2.3 | rs27546520 | 1 | 0 |
| chr4 | 133861135 | A | G | 180 | Rps6ka1 | intronic | . | . | . | 4qD2.3 | rs27546518 | 1 | 0 |
| chr4 | 133861500 | A | C | 228 | Rps6ka1 | exonic | synonymous SNV | Rps6ka1:NM_001285506:exon13:c.T1083G:p.R361R,Rps6ka1:NM_001285505:exon14:c.T1098G:p.R366R,Rps | . | 4qD2.3 | rs13459955 | 1 | 0 |
| chr4 | 133861530 | A | G | 228 | Rps6ka1 | exonic | synonymous SNV | Rps6ka1:NM_001285506:exon13:c.T1053C:p.P351P,Rps6ka1:NM_001285505:exon14:c.T1068C:p.P356P,Rps | . | 4qD2.3 | rs13459959 | 1 | 0 |
| chr4 | 133863620 | A | T | 228 | Rps6ka1 | intronic | . | . | . | 4qD2.3 | rs27546492 | 1 | 0 |
| chr4 | 133863631 | T | C | 228 | Rps6ka1 | intronic | . | . | . | 4qD2.3 | rs27546491 | 1 | 0 |
| chr4 | 133863754 | T | C | 228 | Rps6ka1 | splicing | . | . | . | 4qD2.3 | rs46331355 | 1 | 0 |
| chr4 | 133863765 | A | G | 228 | Rps6ka1 | intronic | . | . | . | 4qD2.3 | rs48251309 | 1 | 0 |
| chr4 | 133864943 | T | C | 179 | Rps6ka1 | intronic | . | . | . | 4qD2.3 | rs27546478 | 1 | 0 |
| chr4 | 133865238 | A | G | 228 | Rps6ka1 | intronic | . | . | . | 4qD2.3 | rs27546477 | 1 | 0 |
| chr4 | 133865544 | T | G | 228 | Rps6ka1 | intronic | . | . | . | 4qD2.3 | rs27546476 | 1 | 0 |
| chr4 | 133866232 | C | G | 174 | Rps6ka1 | intronic | . | . | . | 4qD2.3 | rs27546471 | 1 | 0 |
| chr4 | 133866519 | T | C | 162 | Rps6ka1 | intronic | . | . | . | 4qD2.3 | rs27546469 | 1 | 0 |
| chr4 | 133869381 | A | G | 228 | Rps6ka1 | intronic | . | . | . | 4qD2.3 | rs27594538 | 1 | 0 |
| chr4 | 133869506 | T | C | 228 | Rps6ka1 | exonic | synonymous SNV | Rps6ka1:NM_001285506:exon4:c.A333G:p.L111L,Rps6ka1:NM_001285505:exon5:c.A381G:p.L127L,Rps6ka1 | . | 4qD2.3 | rs27594537 | 1 | 0 |
| chr4 | 133871278 | T | C | 101 | Rps6ka1 | intronic | . | . | . | 4qD2.3 | rs27594525 | 1 | 0 |
| chr4 | 133871660 | G | A | 228 | Rps6ka1 | intronic | . | . | . | 4qD2.3 | rs48251932 | 1 | 0 |
| chr4 | 133872083 | G | A | 228 | Rps6ka1 | intronic | . | . | . | 4qD2.3 | rs27594524 | 1 | 0 |
| chr4 | 133872143 | C | T | 228 | Rps6ka1 | intronic | . | . | . | 4qD2.3 | rs27594523 | 1 | 0 |
| chr4 | 133872213 | A | G | 92 | Rps6ka1 | intronic | . | . | . | 4qD2.3 | rs27594522 | 1 | 0 |
| chr4 | 133880854 | G | C | 228 | Rps6ka1 | intronic | . | . | . | 4qD2.3 | rs32322286 | 1 | 0 |
| chr4 | 133966224 | T | C | 228 | Hmgn2 | intronic | . | . | . | 4qD3 | rs32624994 | 1 | 0 |
| chr4 | 133966360 | G | T | 228 | Hmgn2 | intronic | . | . | . | 4qD3 | rs32036128 | 1 | 0 |
| chr4 | 133966495 | T | G | 62 | Hmgn2 | intronic | . | . | . | 4qD3 | . | 1 | 0 |
| chr4 | 133966501 | A | G | 63 | Hmgn2 | intronic | . | . | . | 4qD3 | rs32234601 | 1 | 0 |
| chr4 | 133966583 | A | G | 228 | Hmgn2 | intronic | . | . | . | 4qD3 | rs13459995 | 1 | 0 |
| chr4 | 133966648 | A | T | 228 | Hmgn2 | intronic | . | . | . | 4qD3 | rs32615334 | 1 | 0 |
| chr4 | 133966778 | C | T | 228 | Hmgn2 | exonic | synonymous SNV | Hmgn2:NM_016957:exon3:c.G78A:p.A26A | . | 4qD3 | rs33012668 | 1 | 0 |
| chr4 | 133967158 | A | G | 228 | Hmgn2 | intronic | . | . | 1272 | 4qD3 | rs51020329 | 1 | 0 |
| chr4 | 133967695 | G | C | 87 | Hmgn2 | intronic | . | . | 1272 | 4qD3 | rs248648687 | 1 | 0 |
| chr4 | 133971402 | A | G | 168 | Dhdds | intronic | . | . | . | 4qD3 | rs46563781 | 1 | 0 |
| chr4 | 133980241 | G | A | 228 | Dhdds | intronic | . | . | . | 4qD3 | rs27574239 | 1 | 0 |
| chr4 | 133982678 | G | A | 155 | Dhdds | intronic | . | . | . | 4qD3 | rs27574230 | 1 | 0 |
| chr4 | 133982703 | G | A | 168 | Dhdds | intronic | . | . | . | 4qD3 | rs27574229 | 1 | 0 |
| chr4 | 133982784 | T | C | 228 | Dhdds | intronic | . | . | . | 4qD3 | rs27574228 | 1 | 0 |
| chr4 | 133982893 | G | A | 228 | Dhdds | exonic | synonymous SNV | Dhdds:NM_026144:exon7:c.C564T:p.L188L | . | 4qD3 | rs27574227 | 1 | 0 |
| chr4 | 133991419 | A | C | 228 | Dhdds | intronic | . | . | . | 4qD3 | rs27574191 | 1 | 0 |
| chr4 | 133992556 | C | T | 228 | Dhdds | exonic | synonymous SNV | Dhdds:NM_026144:exon5:c.G348A:p.V116V | . | 4qD3 | rs13469621 | 1 | 0 |
| chr4 | 133994027 | C | A | 170 | Dhdds | intronic | . | . | . | 4qD3 | rs27574183 | 1 | 0 |
| chr4 | 133994131 | C | T | 228 | Dhdds | intronic | . | . | . | 4qD3 | rs27574182 | 1 | 0 |
| chr4 | 133994154 | C | A | 228 | Dhdds | intronic | . | . | . | 4qD3 | rs27574181 | 1 | 0 |
| chr4 | 133994369 | C | A | 228 | Dhdds | intronic | . | . | . | 4qD3 | rs27574179 | 1 | 0 |
| chr4 | 133996813 | C | T | 228 | Dhdds | intronic | . | . | . | 4qD3 | rs27574169 | 1 | 0 |
| chr4 | 134000375 | C | G | 95 | Dhdds | intronic | . | . | . | 4qD3 | rs32767104 | 1 | 0 |
| chr4 | 134004179 | A | G | 228 | Lin28a | UTR3 | . | . | . | 4qD3 | rs27574148 | 1 | 0 |
| chr4 | 134004628 | C | T | 228 | Lin28a | UTR3 | . | . | . | 4qD3 | rs27574147 | 1 | 0 |
| chr4 | 134004681 | A | T | 228 | Lin28a | UTR3 | . | . | . | 4qD3 | rs27574146 | 1 | 0 |
| chr4 | 134006357 | A | C | 228 | Lin28a | intronic | . | . | . | 4qD3 | rs48499600 | 1 | 0 |
| chr4 | 134006395 | C | T | 228 | Lin28a | intronic | . | . | . | 4qD3 | rs27574140 | 1 | 0 |
| chr4 | 134007949 | T | C | 228 | Lin28a | exonic | synonymous SNV | Lin28a:NM_145833:exon3:c.A375G:p.K125K | . | 4qD3 | rs31849723 | 1 | 0 |
| chr4 | 134007967 | C | T | 228 | Lin28a | exonic | synonymous SNV | Lin28a:NM_145833:exon3:c.G357A:p.G119G | . | 4qD3 | rs27574133 | 1 | 0 |
| chr4 | 134008158 | C | A | 170 | Lin28a | intronic | . | . | . | 4qD3 | rs32111662 | 1 | 0 |
| chr4 | 134008220 | T | C | 146 | Lin28a | intronic | . | . | . | 4qD3 | rs27574132 | 1 | 0 |
| chr4 | 134018095 | A | G | 228 | Lin28a | exonic | synonymous SNV | Lin28a:NM_145833:exon2:c.T186C:p.A62A | . | 4qD3 | rs31968322 | 1 | 0 |
| chr4 | 134018307 | A | G | 121 | Lin28a | intronic | . | . | . | 4qD3 | rs32638095 | 1 | 0 |
| chr4 | 134018487 | A | C | 131 | Lin28a | intronic | . | . | . | 4qD3 | rs32683895 | 1 | 0 |
| chr4 | 134018588 | A | G | 219 | Lin28a | intronic | . | . | . | 4qD3 | rs32556995 | 1 | 0 |
| chr4 | 134018659 | A | C | 228 | Lin28a | intronic | . | . | . | 4qD3 | rs32422652 | 1 | 0 |
| chr4 | 134018876 | C | A | 135 | . | upstream | . | . | . | 4qD3 | . | 1 | 0 |
| chr4 | 134054030 | G | T | 228 | . | intergenic | . | . | . | 4qD3 | rs32306768 | 1 | 0 |
| chr4 | 134054105 | G | A | 146 | . | intergenic | . | . | . | 4qD3 | rs31810997 | 1 | 0 |
| chr4 | 134054418 | T | C | 178 | . | intergenic | . | . | . | 4qD3 | rs27560226 | 1 | 0 |
| chr4 | 134055598 | T | C | 228 | . | intergenic | . | . | . | 4qD3 | rs27560223 | 1 | 0 |
| chr4 | 134055624 | A | C | 174 | . | intergenic | . | . | . | 4qD3 | rs50113238 | 1 | 0 |
| chr4 | 134055990 | A | G | 228 | . | intergenic | . | . | . | 4qD3 | rs46019765 | 1 | 0 |
| chr4 | 134058616 | C | A | 228 | . | intergenic | . | . | . | 4qD3 | rs27560211 | 1 | 0 |
| chr4 | 134058740 | T | C | 228 | . | intergenic | . | . | . | 4qD3 | rs27560210 | 1 | 0 |
| chr4 | 134058765 | A | C | 228 | . | intergenic | . | . | . | 4qD3 | rs27560209 | 1 | 0 |
| chr4 | 134058792 | A | T | 228 | . | intergenic | . | . | . | 4qD3 | rs27560208 | 1 | 0 |
| chr4 | 134059081 | A | G | 192 | . | intergenic | . | . | . | 4qD3 | rs27560207 | 1 | 0 |
| chr4 | 134072354 | G | T | 112 | Aim1l | intronic | . | . | . | 4qD3 | rs27560145 | 1 | 0 |
| chr4 | 134072590 | G | A | 228 | Aim1l | exonic | missense SNV | Aim1l:NM_001162970:exon2:c.G133A:p.A45T | . | 4qD3 | rs27560144 | 1 | 0 |
| chr4 | 134073028 | C | A | 228 | Aim1l | exonic | synonymous SNV | Aim1l:NM_001162970:exon2:c.C571A:p.R191R | 212 | 4qD3 | rs27560141 | 1 | 0 |
| chr4 | 134073045 | T | C | 228 | Aim1l | exonic | synonymous SNV | Aim1l:NM_001162970:exon2:c.T588C:p.S196S | 212 | 4qD3 | rs27560140 | 1 | 0 |
| chr4 | 134074850 | A | G | 228 | Aim1l | exonic | missense SNV | Aim1l:NM_001162970:exon2:c.A2393G:p.K798R | . | 4qD3 | rs13477992 | 1 | 0 |
| chr4 | 134074999 | A | G | 228 | Aim1l | intronic | . | . | . | 4qD3 | rs32164415 | 1 | 0 |
| chr4 | 134075021 | A | G | 228 | Aim1l | intronic | . | . | . | 4qD3 | rs32965045 | 1 | 0 |
| chr4 | 134075198 | G | T | 227 | Aim1l | intronic | . | . | . | 4qD3 | rs3709931 | 1 | 0 |
| chr4 | 134075389 | G | A | 228 | Aim1l | intronic | . | . | . | 4qD3 | rs3711125 | 1 | 0 |
| chr4 | 134075866 | G | A | 228 | Aim1l | intronic | . | . | . | 4qD3 | rs47705360 | 1 | 0 |
| chr4 | 134075872 | A | G | 212 | Aim1l | intronic | . | . | . | 4qD3 | rs51854251 | 1 | 0 |
| chr4 | 134078665 | T | C | 171 | Aim1l | intronic | . | . | . | 4qD3 | rs27560105 | 1 | 0 |
| chr4 | 134078826 | T | C | 74 | Aim1l | intronic | . | . | . | 4qD3 | rs47306533 | 1 | 0 |
| chr4 | 134079000 | T | C | 177 | Aim1l | intronic | . | . | . | 4qD3 | rs27560102 | 1 | 0 |
| chr4 | 134080654 | A | G | 228 | Aim1l | intronic | . | . | . | 4qD3 | rs48382756 | 1 | 0 |
| chr4 | 134080890 | C | T | 228 | Aim1l | intronic | . | . | . | 4qD3 | rs27560094 | 1 | 0 |
| chr4 | 134080937 | T | G | 228 | Aim1l | intronic | . | . | . | 4qD3 | rs32904038 | 1 | 0 |
| chr4 | 134080938 | T | C | 228 | Aim1l | intronic | . | . | . | 4qD3 | rs27560093 | 1 | 0 |
| chr4 | 134081140 | A | G | 228 | Aim1l | exonic | missense SNV | Aim1l:NM_001162970:exon9:c.A3124G:p.R1042G | . | 4qD3 | rs27560092 | 1 | 0 |
| chr4 | 134081268 | T | G | 228 | Aim1l | intronic | . | . | . | 4qD3 | rs27560091 | 1 | 0 |
| chr4 | 134081429 | A | C | 228 | Aim1l | exonic | missense SNV | Aim1l:NM_001162970:exon10:c.A3286C:p.N1096H | . | 4qD3 | rs27560090 | 1 | 0 |
| chr4 | 134081582 | C | T | 228 | Aim1l | intronic | . | . | . | 4qD3 | rs32408938 | 1 | 0 |
| chr4 | 134081620 | C | G | 228 | Aim1l | intronic | . | . | . | 4qD3 | rs32384935 | 1 | 0 |
| chr4 | 134081670 | C | T | 228 | Aim1l | intronic | . | . | . | 4qD3 | rs27560089 | 1 | 0 |
| chr4 | 134081919 | C | T | 228 | Aim1l | intronic | . | . | 314 | 4qD3 | rs235142573 | 1 | 0 |

| | | | | | | | | | | | | | |
|---|---|---|---|---|---|---|---|---|---|---|---|---|---|
| chr4 | 134081942 | C | T | 228 | Aim1l | intronic | . | . | 314 | 4qD3 | rs264193696 | 1 | 0 |
| chr4 | 134081997 | T | A | 228 | Aim1l | exonic | missense SNV | Aim1l:NM_001162970:exon12:c.T3518A:p.F1173Y | 314 | 4qD3 | rs27560087 | 1 | 0 |
| chr4 | 134082130 | G | A | 228 | Aim1l | intronic | . | . | 314 | 4qD3 | rs220930082 | 1 | 0 |
| chr4 | 134082142 | C | T | 228 | Aim1l | intronic | . | . | 314 | 4qD3 | rs32570613 | 1 | 0 |
| chr4 | 134082197 | A | T | 228 | Aim1l | intronic | . | . | . | 4qD3 | rs27560086 | 1 | 0 |
| chr4 | 134082264 | T | G | 189 | Aim1l | intronic | . | . | . | 4qD3 | rs32983158 | 1 | 0 |
| chr4 | 134082338 | C | T | 228 | Aim1l | intronic | . | . | . | 4qD3 | rs27560085 | 1 | 0 |
| chr4 | 134082383 | C | T | 228 | Aim1l | intronic | . | . | . | 4qD3 | rs27560084 | 1 | 0 |
| chr4 | 134082554 | C | G | 228 | Aim1l | intronic | . | . | . | 4qD3 | rs27560083 | 1 | 0 |
| chr4 | 134082641 | C | T | 228 | Aim1l | exonic | synonymous SNV | Aim1l:NM_001162970:exon14:c.C3703T:p.L1235L | . | 4qD3 | rs27560081 | 1 | 0 |
| chr4 | 134082731 | A | G | 228 | Aim1l | intronic | . | . | . | 4qD3 | rs230363353 | 1 | 0 |
| chr4 | 134088770 | C | T | 228 | Aim1l | intronic | . | . | . | 4qD3 | rs27560059 | 1 | 0 |
| chr4 | 134088906 | G | A | 228 | Aim1l | exonic | synonymous SNV | Aim1l:NM_001162970:exon16:c.G3993A:p.V1331V | . | 4qD3 | rs27560058 | 1 | 0 |
| chr4 | 134089108 | G | A | 115 | Aim1l | intronic | . | . | . | 4qD3 | rs27560056 | 1 | 0 |
| chr4 | 134089261 | G | T | 228 | Aim1l | intronic | . | . | . | 4qD3 | rs27560055 | 1 | 0 |
| chr4 | 134090942 | A | G | 172 | Aim1l | intronic | . | . | . | 4qD3 | rs27560042 | 1 | 0 |
| chr4 | 134091010 | C | T | 228 | Aim1l | intronic | . | . | . | 4qD3 | rs27560041 | 1 | 0 |
| chr4 | 134091184 | A | G | 228 | Aim1l | exonic | missense SNV | Aim1l:NM_001162970:exon18:c.A4250G:p.Q1417R | . | 4qD3 | rs27560038 | 1 | 0 |
| chr4 | 134091257 | T | C | 228 | Aim1l | intronic | . | . | . | 4qD3 | rs27560036 | 1 | 0 |
| chr4 | 134091341 | C | T | 225 | Aim1l | intronic | . | . | . | 4qD3 | rs27560035 | 1 | 0 |
| chr4 | 134091368 | A | C | 151 | Aim1l | intronic | . | . | . | 4qD3 | rs46566760 | 1 | 0 |
| chr4 | 134091965 | A | G | 176 | Aim1l | intronic | . | . | . | 4qD3 | rs50264444 | 1 | 0 |
| chr4 | 134092516 | C | G | 198 | . | downstream | . | . | . | 4qD3 | rs27560031 | 1 | 0 |
| chr4 | 134095007 | T | G | 228 | Cd52 | UTR5 | . | . | . | 4qD3 | rs27560022 | 1 | 0 |
| chr4 | 134108053 | A | G | 99 | Ubxn11 | intronic | . | . | . | 4qD3 | rs47915679 | 1 | 0 |
| chr4 | 134108136 | C | T | 144 | Ubxn11 | intronic | . | . | . | 4qD3 | rs32475829 | 1 | 0 |
| chr4 | 134108158 | T | C | 161 | Ubxn11 | intronic | . | . | . | 4qD3 | rs32590296 | 1 | 0 |
| chr4 | 134108223 | C | T | 228 | Ubxn11 | intronic | . | . | . | 4qD3 | rs50583984 | 1 | 0 |
| chr4 | 134108404 | A | C | 228 | Ubxn11 | intronic | . | . | . | 4qD3 | rs32525786 | 1 | 0 |
| chr4 | 134109173 | C | T | 228 | Ubxn11 | intronic | . | . | . | 4qD3 | rs31788408 | 1 | 0 |
| chr4 | 134112528 | A | G | 228 | Ubxn11 | exonic | synonymous SNV | Ubxn11:NM_026257:exon5:c.A213G:p.S71S | . | 4qD3 | rs50219297 | 1 | 0 |
| chr4 | 134112633 | T | C | 228 | Ubxn11 | intronic | . | . | . | 4qD3 | rs32404237 | 1 | 0 |
| chr4 | 134112882 | T | C | 228 | Ubxn11 | intronic | . | . | . | 4qD3 | rs31841978 | 1 | 0 |
| chr4 | 134112889 | T | A | 228 | Ubxn11 | intronic | . | . | . | 4qD3 | rs31967694 | 1 | 0 |
| chr4 | 134112919 | G | A | 228 | Ubxn11 | exonic | missense SNV | Ubxn11:NM_026257:exon6:c.G299A:p.R100Q | . | 4qD3 | rs32633611 | 1 | 0 |
| chr4 | 134113300 | A | G | 228 | Ubxn11 | intronic | . | . | . | 4qD3 | rs48628596 | 1 | 0 |
| chr4 | 134113460 | G | A | 228 | Ubxn11 | intronic | . | . | . | 4qD3 | rs32217634 | 1 | 0 |
| chr4 | 134116223 | C | G | 228 | Ubxn11 | intronic | . | . | . | 4qD3 | rs27559977 | 1 | 0 |
| chr4 | 134116273 | C | T | 162 | Ubxn11 | intronic | . | . | . | 4qD3 | rs27559976 | 1 | 0 |
| chr4 | 134123627 | G | A | 228 | Ubxn11 | intronic | . | . | . | 4qD3 | rs27544035 | 1 | 0 |
| chr4 | 134124757 | G | A | 228 | Ubxn11 | intronic | . | . | . | 4qD3 | rs32738392 | 1 | 0 |
| chr4 | 134126784 | T | C | 143 | . | downstream | . | . | . | 4qD3 | rs27544025 | 1 | 0 |
| chr4 | 134131174 | A | C | 130 | Cep85 | UTR3 | . | . | . | 4qD3 | rs27544013 | 1 | 0 |
| chr4 | 134131176 | A | C | 136 | Cep85 | UTR3 | . | . | . | 4qD3 | rs48638674 | 1 | 0 |
| chr4 | 134131209 | T | G | 160 | Cep85 | UTR3 | . | . | . | 4qD3 | rs27544012 | 1 | 0 |
| chr4 | 134131423 | C | T | 228 | Cep85 | exonic | synonymous SNV | Cep85:NM_144527:exon14:c.G2142A:p.G714G | . | 4qD3 | rs27544011 | 1 | 0 |
| chr4 | 134137385 | T | C | 117 | Cep85 | intronic | . | . | . | 4qD3 | rs27543995 | 1 | 0 |
| chr4 | 134137416 | T | C | 145 | Cep85 | intronic | . | . | . | 4qD3 | rs27543994 | 1 | 0 |
| chr4 | 134137443 | A | G | 159 | Cep85 | intronic | . | . | . | 4qD3 | rs27543993 | 1 | 0 |
| chr4 | 134138707 | C | A | 171 | Cep85 | intronic | . | . | . | 4qD3 | rs27543988 | 1 | 0 |
| chr4 | 134138809 | T | G | 228 | Cep85 | intronic | . | . | . | 4qD3 | rs31862976 | 1 | 0 |
| chr4 | 134147962 | G | A | 228 | Cep85 | intronic | . | . | . | 4qD3 | rs6348836 | 1 | 0 |
| chr4 | 134147963 | A | G | 228 | Cep85 | intronic | . | . | . | 4qD3 | rs6348837 | 1 | 0 |
| chr4 | 134148561 | T | C | 228 | Cep85 | intronic | . | . | . | 4qD3 | rs32529456 | 1 | 0 |
| chr4 | 134153726 | A | G | 228 | Cep85 | intronic | . | . | . | 4qD3 | rs27543959 | 1 | 0 |
| chr4 | 134153988 | T | C | 228 | Cep85 | intronic | . | . | . | 4qD3 | rs27543958 | 1 | 0 |
| chr4 | 134154121 | C | T | 228 | Cep85 | intronic | . | . | . | 4qD3 | rs27543957 | 1 | 0 |
| chr4 | 134154431 | G | A | 228 | Cep85 | intronic | . | . | . | 4qD3 | rs27543953 | 1 | 0 |
| chr4 | 134156487 | A | G | 170 | Cep85 | intronic | . | . | . | 4qD3 | rs51107569 | 1 | 0 |
| chr4 | 134164828 | T | G | 228 | Cep85 | intronic | . | . | . | 4qD3 | rs27543944 | 1 | 0 |
| chr4 | 134167149 | T | C | 102 | Cep85 | intronic | . | . | . | 4qD3 | rs251437970 | 1 | 0 |
| chr4 | 134167274 | G | A | 228 | Cep85 | intronic | . | . | . | 4qD3 | rs27543941 | 1 | 0 |
| chr4 | 134172792 | T | G | 228 | Cep85 | intronic | . | . | . | 4qD3 | rs27543921 | 1 | 0 |
| chr4 | 134172917 | A | T | 228 | Cep85 | intronic | . | . | . | 4qD3 | rs27543920 | 1 | 0 |
| chr4 | 134190960 | C | T | 228 | Gm7534 | UTR3 | . | . | . | 4qD3 | rs27543878 | 1 | 0 |
| chr4 | 134191950 | T | C | 130 | Gm7534 | intronic | . | . | . | 4qD3 | rs27543877 | 1 | 0 |
| chr4 | 134192140 | T | C | 228 | Gm7534 | intronic | . | . | . | 4qD3 | rs27543873 | 1 | 0 |
| chr4 | 134192438 | A | C | 132 | Gm7534 | intronic | . | . | . | 4qD3 | rs27543872 | 1 | 0 |
| chr4 | 134192717 | G | T | 228 | Gm7534 | exonic | missense SNV | Gm7534:NM_001080712:exon6:c.C1914A:p.S638R | . | 4qD3 | rs27543871 | 1 | 0 |
| chr4 | 134195547 | C | T | 228 | Gm7534 | exonic | missense SNV | Gm7534:NM_001080712:exon4:c.G1474A:p.V492I | . | 4qD3 | rs27543865 | 1 | 0 |
| chr4 | 134201990 | T | C | 228 | Gm7534 | exonic | missense SNV | Gm7534:NM_001080712:exon1:c.A1003G:p.T335A | . | 4qD3 | rs27543852 | 1 | 0 |
| chr4 | 134201993 | C | T | 228 | Gm7534 | exonic | missense SNV | Gm7534:NM_001080712:exon1:c.G1000A:p.G334S | . | 4qD3 | rs49800012 | 1 | 0 |
| chr4 | 134202430 | A | G | 228 | Gm7534 | exonic | missense SNV | Gm7534:NM_001080712:exon1:c.T563C:p.I188T | . | 4qD3 | rs31921943 | 1 | 0 |
| chr4 | 134202523 | T | C | 228 | Gm7534 | exonic | missense SNV | Gm7534:NM_001080712:exon1:c.A470G:p.D157G | . | 4qD3 | rs32707331 | 1 | 0 |
| chr4 | 134202624 | C | T | 228 | Gm7534 | exonic | synonymous SNV | Gm7534:NM_001080712:exon1:c.G369A:p.T123T | . | 4qD3 | rs51684466 | 1 | 0 |
| chr4 | 134202670 | C | T | 228 | Gm7534 | exonic | missense SNV | Gm7534:NM_001080712:exon1:c.G323A:p.G108D | . | 4qD3 | rs27543849 | 1 | 0 |
| chr4 | 134202900 | C | T | 228 | Gm7534 | exonic | synonymous SNV | Gm7534:NM_001080712:exon1:c.G93A:p.V31V | . | 4qD3 | rs32343052 | 1 | 0 |
| chr4 | 134212178 | T | C | 183 | Catsper4 | UTR3 | . | . | . | 4qD3 | rs32460990 | 1 | 0 |
| chr4 | 134212244 | G | A | 228 | Catsper4 | UTR3 | . | . | . | 4qD3 | rs32281181 | 1 | 0 |
| chr4 | 134213157 | A | G | 228 | Catsper4 | intronic | . | . | . | 4qD3 | rs32071426 | 1 | 0 |
| chr4 | 134213511 | A | G | 228 | Catsper4 | intronic | . | . | . | 4qD3 | rs46560075 | 1 | 0 |
| chr4 | 134215350 | T | C | 228 | Catsper4 | intronic | . | . | . | 4qD3 | rs27543832 | 1 | 0 |
| chr4 | 134217655 | G | C | 170 | Catsper4 | intronic | . | . | . | 4qD3 | rs27543823 | 1 | 0 |
| chr4 | 134218027 | T | C | 228 | Catsper4 | intronic | . | . | . | 4qD3 | rs27543822 | 1 | 0 |
| chr4 | 134218040 | A | G | 228 | Catsper4 | intronic | . | . | . | 4qD3 | rs27543821 | 1 | 0 |
| chr4 | 134218174 | A | G | 228 | Catsper4 | exonic | synonymous SNV | Catsper4:NM_177866:exon6:c.T573C:p.A191A | . | 4qD3 | rs27543820 | 1 | 0 |
| chr4 | 134218527 | C | T | 228 | Catsper4 | intronic | . | . | . | 4qD3 | rs27543818 | 1 | 0 |
| chr4 | 134218538 | C | T | 228 | Catsper4 | intronic | . | . | . | 4qD3 | rs27543817 | 1 | 0 |
| chr4 | 134221502 | A | G | 228 | Catsper4 | intronic | . | . | . | 4qD3 | rs27543808 | 1 | 0 |
| chr4 | 134221616 | A | G | 228 | Catsper4 | exonic | synonymous SNV | Catsper4:NM_001130030:exon4:c.T291C:p.H97H,Catsper4:NM_177866:exon4:c.T291C:p.H97H | . | 4qD3 | rs27543807 | 1 | 0 |
| chr4 | 134221648 | T | C | 228 | Catsper4 | intronic | . | . | . | 4qD3 | rs27543806 | 1 | 0 |
| chr4 | 134226601 | A | G | 228 | Catsper4 | exonic | synonymous SNV | Catsper4:NM_001130030:exon3:c.T246C:p.A82A,Catsper4:NM_177866:exon3:c.T246C:p.A82A | . | 4qD3 | rs47504276 | 1 | 0 |
| chr4 | 134226799 | A | G | 228 | Catsper4 | intronic | . | . | . | 4qD3 | rs50212136 | 1 | 0 |
| chr4 | 134227256 | C | G | 228 | Catsper4 | intronic | . | . | . | 4qD3 | rs27543794 | 1 | 0 |
| chr4 | 134227373 | G | A | 228 | Catsper4 | UTR5 | . | . | . | 4qD3 | rs27543793 | 1 | 0 |
| chr4 | 134228097 | A | C | 149 | Cnksr1 | UTR3 | . | . | . | 4qD3 | rs27543788 | 1 | 0 |
| chr4 | 134228335 | G | A | 228 | Cnksr1 | exonic | missense SNV | Cnksr1:NM_001081047:exon21:c.C2083T:p.R695C | . | 4qD3 | rs27543787 | 1 | 0 |
| chr4 | 134228381 | G | A | 228 | Cnksr1 | exonic | synonymous SNV | Cnksr1:NM_001081047:exon21:c.C2037T:p.D679D | . | 4qD3 | rs32813851 | 1 | 0 |
| chr4 | 134229298 | C | G | 228 | Cnksr1 | intronic | . | . | . | 4qD3 | rs27543783 | 1 | 0 |
| chr4 | 134229401 | G | A | 228 | Cnksr1 | exonic | missense SNV | Cnksr1:NM_001081047:exon18:c.C1547T:p.A516V | . | 4qD3 | rs27543782 | 1 | 0 |
| chr4 | 134229767 | G | T | 228 | Cnksr1 | intronic | . | . | . | 4qD3 | rs27543777 | 1 | 0 |
| chr4 | 134230128 | T | C | 228 | Cnksr1 | intronic | . | . | . | 4qD3 | . | 1 | 0 |
| chr4 | 134230287 | G | A | 228 | Cnksr1 | intronic | . | . | . | 4qD3 | rs48481512 | 1 | 0 |
| chr4 | 134230379 | T | C | 166 | Cnksr1 | intronic | . | . | . | 4qD3 | . | 1 | 0 |
| chr4 | 134230380 | T | C | 205 | Cnksr1 | intronic | . | . | . | 4qD3 | rs32448376 | 1 | 0 |
| chr4 | 134230561 | C | T | 228 | Cnksr1 | intronic | . | . | . | 4qD3 | rs27543773 | 1 | 0 |
| chr4 | 134232018 | A | G | 228 | Cnksr1 | intronic | . | . | . | 4qD3 | rs27543770 | 1 | 0 |
| chr4 | 134232527 | A | C | 174 | Cnksr1 | intronic | . | . | . | 4qD3 | rs27543768 | 1 | 0 |
| chr4 | 134232560 | A | G | 228 | Cnksr1 | intronic | . | . | . | 4qD3 | rs27599939 | 1 | 0 |
| chr4 | 134232842 | A | G | 228 | Cnksr1 | exonic | missense SNV | Cnksr1:NM_001081047:exon12:c.T982C:p.F328L | . | 4qD3 | rs27599937 | 1 | 0 |
| chr4 | 134232877 | G | A | 228 | Cnksr1 | intronic | . | . | . | 4qD3 | rs48976607 | 1 | 0 |
| chr4 | 134232961 | G | A | 228 | Cnksr1 | intronic | . | . | . | 4qD3 | rs27599936 | 1 | 0 |
| chr4 | 134233046 | C | T | 228 | Cnksr1 | exonic | synonymous SNV | Cnksr1:NM_001081047:exon11:c.G918A:p.P306P | . | 4qD3 | rs27599935 | 1 | 0 |
| chr4 | 134233257 | A | G | 228 | Cnksr1 | intronic | . | . | . | 4qD3 | rs27599933 | 1 | 0 |
| chr4 | 134233357 | C | T | 228 | Cnksr1 | exonic | missense SNV | Cnksr1:NM_001081047:exon9:c.G778A:p.E260K | . | 4qD3 | rs27599932 | 1 | 0 |
| chr4 | 134233474 | T | C | 228 | Cnksr1 | intronic | . | . | . | 4qD3 | rs27599931 | 1 | 0 |
| chr4 | 134233778 | C | T | 228 | Cnksr1 | intronic | . | . | . | 4qD3 | rs32833073 | 1 | 0 |
| chr4 | 134233858 | A | G | 228 | Cnksr1 | intronic | . | . | . | 4qD3 | rs32077525 | 1 | 0 |
| chr4 | 134234590 | T | C | 228 | Cnksr1 | exonic | missense SNV | Cnksr1:NM_001081047:exon6:c.A523G:p.S175G | . | 4qD3 | rs27599927 | 1 | 0 |
| chr4 | 134234676 | T | C | 228 | Cnksr1 | exonic | synonymous SNV | Cnksr1:NM_001081047:exon5:c.A513G:p.L171L | . | 4qD3 | rs50078770 | 1 | 0 |
| chr4 | 134235830 | G | A | 228 | Cnksr1 | intronic | . | . | . | 4qD3 | rs27599925 | 1 | 0 |
| chr4 | 134236172 | C | T | 228 | Cnksr1 | exonic | synonymous SNV | Cnksr1:NM_001081047:exon2:c.G198A:p.Q66Q | . | 4qD3 | rs27599923 | 1 | 0 |
| chr4 | 134244738 | T | C | 201 | E130218I03Rik,Zfr | ncRNA_intronic | . | . | . | 4qD3 | rs32623168 | 1 | 0 |
| chr4 | 134244976 | C | T | 228 | E130218I03Rik,Zfr | ncRNA_exonic | . | . | . | 4qD3 | rs52009984 | 1 | 0 |
| chr4 | 134245051 | A | G | 228 | Zfp593,E130218I03 | exonic | synonymous SNV | Zfp593:NM_024215:exon2:c.T237C:p.H79H | . | 4qD3 | rs50475716 | 1 | 0 |
| chr4 | 134251337 | T | C | 101 | Grrp1 | UTR3 | . | . | 834 | 4qD3 | rs32991992 | 1 | 0 |
| chr4 | 134251680 | T | C | 228 | Grrp1 | exonic | synonymous SNV | Grrp1:NM_001099296:exon2:c.A486G:p.K162K | 834 | 4qD3 | rs32277122 | 1 | 0 |
| chr4 | 134251737 | T | C | 228 | Grrp1 | exonic | synonymous SNV | Grrp1:NM_001099296:exon2:c.A429G:p.P143P | 834 | 4qD3 | rs32234828 | 1 | 0 |
| chr4 | 134252180 | G | A | 228 | Grrp1 | UTR5 | . | . | . | 4qD3 | rs32683779 | 1 | 0 |
| chr4 | 134278300 | C | T | 228 | Pdik1l | UTR3 | . | . | . | 4qD3 | rs32877105 | 1 | 0 |
| chr4 | 134278302 | T | C | 228 | Pdik1l | UTR3 | . | . | . | 4qD3 | rs27578207 | 1 | 0 |
| chr4 | 134284232 | G | C | 228 | Pdik1l | intronic | . | . | . | 4qD3 | rs27578193 | 1 | 0 |
| chr4 | 134286619 | T | C | 216 | Pdik1l | intronic | . | . | . | 4qD3 | rs50318130 | 1 | 0 |
| chr4 | 134287148 | G | A | 228 | Pdik1l | intronic | . | . | 1331 | 4qD3 | rs48583129 | 1 | 0 |
| chr4 | 134287355 | C | A | 156 | Pdik1l | intronic | . | . | 1331 | 4qD3 | . | 1 | 0 |
| chr4 | 134316322 | A | G | 101 | Trim63 | intronic | . | . | . | 4qD3 | rs27578112 | 1 | 0 |
| chr4 | 134316610 | C | T | 228 | Trim63 | intronic | . | . | . | 4qD3 | rs32649482 | 1 | 0 |
| chr4 | 134319686 | A | G | 228 | Trim63 | exonic | synonymous SNV | Trim63:NM_001039048:exon3:c.A387G:p.K129K | . | 4qD3 | rs27578088 | 1 | 0 |
| chr4 | 134319925 | C | A | 228 | Trim63 | intronic | . | . | . | 4qD3 | rs46847987 | 1 | 0 |
| chr4 | 134319928 | T | C | 227 | Trim63 | intronic | . | . | . | 4qD3 | rs47270445 | 1 | 0 |
| chr4 | 134319958 | A | G | 144 | Trim63 | intronic | . | . | . | 4qD3 | rs48943972 | 1 | 0 |
| chr4 | 134321221 | C | G | 228 | Trim63 | exonic | synonymous SNV | Trim63:NM_001039048:exon4:c.C540G:p.G180G | . | 4qD3 | rs27578080 | 1 | 0 |
| chr4 | 134321263 | C | G | 228 | Trim63 | exonic | synonymous SNV | Trim63:NM_001039048:exon4:c.C582G:p.S194S | . | 4qD3 | rs27578079 | 1 | 0 |
| chr4 | 134321354 | T | A | 169 | Trim63 | intronic | . | . | . | 4qD3 | rs27578078 | 1 | 0 |
| chr4 | 134322879 | A | G | 158 | Trim63 | intronic | . | . | . | 4qD3 | rs31796446 | 1 | 0 |
| chr4 | 134323180 | A | G | 228 | Trim63 | exonic | synonymous SNV | Trim63:NM_001039048:exon5:c.A774G:p.K258K | . | 4qD3 | rs27578070 | 1 | 0 |
| chr4 | 134323280 | T | G | 217 | Trim63 | intronic | . | . | . | 4qD3 | rs27578069 | 1 | 0 |
| chr4 | 134323329 | T | C | 194 | Trim63 | intronic | . | . | . | 4qD3 | rs31879911 | 1 | 0 |
| chr4 | 134324769 | G | T | 101 | Trim63 | intronic | . | . | . | 4qD3 | rs27578067 | 1 | 0 |
| chr4 | 134324937 | A | G | 228 | Trim63 | intronic | . | . | . | 4qD3 | rs27578063 | 1 | 0 |
| chr4 | 134325035 | C | T | 228 | Trim63 | intronic | . | . | . | 4qD3 | rs27578062 | 1 | 0 |
| chr4 | 134325059 | C | A | 228 | Trim63 | exonic | synonymous SNV | Trim63:NM_001039048:exon6:c.C846A:p.L282L | . | 4qD3 | rs27578061 | 1 | 0 |
| chr4 | 134325708 | G | A | 221 | Trim63 | exonic | missense SNV | Trim63:NM_001039048:exon7:c.G979A:p.D327N | . | 4qD3 | . | 0 | 1 |
| chr4 | 134327619 | C | T | 213 | Trim63 | intronic | . | . | . | 4qD3 | rs27578054 | 1 | 0 |
| chr4 | 134327664 | G | A | 228 | Trim63 | intronic | . | . | . | 4qD3 | rs27578053 | 1 | 0 |
| chr4 | 134327884 | G | A | 142 | Trim63 | intronic | . | . | . | 4qD3 | rs49576176 | 1 | 0 |
| chr4 | 134327910 | G | A | 144 | Trim63 | intronic | . | . | . | 4qD3 | rs49222592 | 1 | 0 |
| chr4 | 134328758 | A | G | 140 | Trim63 | intronic | . | . | . | 4qD3 | rs27578049 | 1 | 0 |
| chr4 | 134329007 | T | C | 228 | Trim63 | UTR3 | . | . | . | 4qD3 | rs52358077 | 1 | 0 |
| chr4 | 134343146 | T | C | 109 | Slc30a2 | UTR5 | . | . | 480 | 4qD3 | . | 0 | 1 |
| chr4 | 134343276 | A | G | 228 | Slc30a2 | UTR5 | . | . | 480 | 4qD3 | rs45897335 | 1 | 0 |
| chr4 | 134345823 | T | C | 228 | Slc30a2 | intronic | . | . | . | 4qD3 | rs27577996 | 1 | 0 |
| chr4 | 134347201 | G | A | 133 | Slc30a2 | intronic | . | . | . | 4qD3 | rs27577992 | 1 | 0 |
| chr4 | 134347207 | C | A | 133 | Slc30a2 | intronic | . | . | . | 4qD3 | rs49723491 | 1 | 0 |
| chr4 | 134347404 | G | A | 228 | Slc30a2 | exonic | synonymous SNV | Slc30a2:NM_001039677:exon4:c.G471A:p.L157L | . | 4qD3 | rs27577989 | 1 | 0 |
| chr4 | 134348530 | G | A | 228 | Slc30a2 | exonic | missense SNV | Slc30a2:NM_001039677:exon5:c.G614A:p.R205H | . | 4qD3 | rs27577980 | 1 | 0 |
| chr4 | 134348673 | G | A | 228 | Slc30a2 | intronic | . | . | . | 4qD3 | rs50847762 | 1 | 0 |
| chr4 | 134348675 | A | G | 228 | Slc30a2 | intronic | . | . | . | 4qD3 | rs49079255 | 1 | 0 |
| chr4 | 134349102 | C | T | 168 | Slc30a2 | intronic | . | . | . | 4qD3 | rs27577977 | 1 | 0 |
| chr4 | 134349235 | G | A | 228 | Slc30a2 | intronic | . | . | . | 4qD3 | rs32487450 | 1 | 0 |
| chr4 | 134351916 | G | C | 106 | Slc30a2 | intronic | . | . | . | 4qD3 | rs250786485 | 0 | 1 |
| chr4 | 134351947 | T | C | 228 | Slc30a2 | splicing | . | . | . | 4qD3 | rs51167280 | 1 | 0 |
| chr4 | 134352270 | A | G | 228 | Slc30a2 | intronic | . | . | . | 4qD3 | rs48332304 | 1 | 0 |
| chr4 | 134352329 | C | T | 222 | Slc30a2 | intronic | . | . | . | 4qD3 | rs51567336 | 1 | 0 |
| chr4 | 134352680 | G | A | 122 | Slc30a2 | UTR3 | . | . | . | 4qD3 | rs46899838 | 1 | 0 |
| chr4 | 134357531 | G | A | 228 | Ext1l | UTR3 | . | . | . | 4qD3 | rs51498041 | 1 | 0 |
| chr4 | 134357583 | G | A | 228 | Ext1l | UTR3 | . | . | . | 4qD3 | rs50397869 | 1 | 0 |
| chr4 | 134357874 | A | C | 228 | Ext1l | intronic | . | . | . | 4qD3 | rs27563261 | 1 | 0 |
| chr4 | 134357885 | C | A | 228 | Ext1l | intronic | . | . | . | 4qD3 | rs221812307 | 1 | 0 |
| chr4 | 134358954 | T | C | 93 | Ext1l | intronic | . | . | . | 4qD3 | rs48374194 | 1 | 0 |
| chr4 | 134359076 | A | G | 228 | Ext1l | intronic | . | . | . | 4qD3 | rs50702468 | 1 | 0 |
| chr4 | 134359269 | A | G | 228 | Ext1l | intronic | . | . | . | 4qD3 | rs49361750 | 1 | 0 |
| chr4 | 134359655 | A | G | 228 | Ext1l | intronic | . | . | . | 4qD3 | rs48269444 | 1 | 0 |
| chr4 | 134359721 | C | T | 228 | Ext1l | intronic | . | . | . | 4qD3 | rs46559868 | 1 | 0 |
| chr4 | 134360593 | A | G | 228 | Ext1l | exonic | missense SNV | Ext1l:NM_019578:exon7:c.T1349C:p.L450P | . | 4qD3 | rs51260336 | 1 | 0 |

| | | | | | | | | | | | | | |
|---|---|---|---|---|---|---|---|---|---|---|---|---|---|
| chr4 | 134362229 | T | C | 88 | Extl1 | intronic | . | . | . | 4qD3 | rs27563258 | 1 | 0 |
| chr4 | 134362274 | A | T | 136 | Extl1 | intronic | . | . | . | 4qD3 | rs32960141 | 1 | 0 |
| chr4 | 134362459 | C | T | 222 | Extl1 | intronic | . | . | . | 4qD3 | rs3709127 | 1 | 0 |
| chr4 | 134362715 | T | C | 228 | Extl1 | exonic | missense SNV | Extl1:NM_019578:exon5:c.A1081G:p.I361V | . | 4qD3 | rs27563256 | 1 | 0 |
| chr4 | 134362738 | A | G | 228 | Extl1 | intronic | . | . | . | 4qD3 | rs27563255 | 1 | 0 |
| chr4 | 134362871 | T | C | 228 | Extl1 | intronic | . | . | . | 4qD3 | rs27563252 | 1 | 0 |
| chr4 | 134362932 | C | A | 228 | Extl1 | intronic | . | . | . | 4qD3 | rs27563251 | 1 | 0 |
| chr4 | 134363019 | A | G | 228 | Extl1 | intronic | . | . | . | 4qD3 | rs27563250 | 1 | 0 |
| chr4 | 134363071 | C | T | 228 | Extl1 | intronic | . | . | . | 4qD3 | rs32533901 | 1 | 0 |
| chr4 | 134363214 | G | C | 228 | Extl1 | intronic | . | . | . | 4qD3 | rs32159797 | 1 | 0 |
| chr4 | 134364496 | A | G | 161 | Extl1 | intronic | . | . | . | 4qD3 | rs27563242 | 1 | 0 |
| chr4 | 134364828 | G | T | 228 | Extl1 | intronic | . | . | . | 4qD3 | rs27563240 | 1 | 0 |
| chr4 | 134364881 | T | C | 228 | Extl1 | intronic | . | . | . | 4qD3 | rs50202306 | 1 | 0 |
| chr4 | 134365054 | C | T | 228 | Extl1 | intronic | . | . | . | 4qD3 | rs48082971 | 1 | 0 |
| chr4 | 134365076 | C | T | 165 | Extl1 | intronic | . | . | . | 4qD3 | rs27563239 | 1 | 0 |
| chr4 | 134371095 | C | T | 228 | Extl1 | exonic | synonymous SNV | Extl1:NM_019578:exon1:c.G624A:p.K208K | . | 4qD3 | rs27563209 | 1 | 0 |
| chr4 | 134371151 | A | T | 228 | Extl1 | exonic | missense SNV | Extl1:NM_019578:exon1:c.T568A:p.L190I | . | 4qD3 | rs27563207 | 1 | 0 |
| chr4 | 134371624 | A | G | 228 | Extl1 | exonic | missense SNV | Extl1:NM_019578:exon1:c.T95C:p.L32S | . | 4qD3 | rs27563205 | 1 | 0 |
| chr4 | 134397403 | C | T | 228 | Pafah2 | intronic | . | . | . | 4qD3 | rs50391752 | 1 | 0 |
| chr4 | 134397482 | T | C | 228 | Pafah2 | UTR5 | . | . | . | 4qD3 | rs50866822 | 1 | 0 |
| chr4 | 134397680 | G | C | 228 | Pafah2 | intronic | . | . | . | 4qD3 | rs48641561 | 1 | 0 |
| chr4 | 134397741 | G | A | 173 | Pafah2 | intronic | . | . | . | 4qD3 | rs50671819 | 1 | 0 |
| chr4 | 134402788 | G | C | 135 | Pafah2 | intronic | . | . | . | 4qD3 | rs47992844 | 1 | 0 |
| chr4 | 134402860 | C | G | 215 | Pafah2 | intronic | . | . | . | 4qD3 | rs51740254 | 1 | 0 |
| chr4 | 134403190 | A | C | 221 | Pafah2 | intronic | . | . | . | 4qD3 | rs27563161 | 1 | 0 |
| chr4 | 134405088 | T | C | 228 | Pafah2 | exonic | synonymous SNV | Pafah2:NM_001285874:exon3:c.T324C:p.G108G,Pafah2:NM_001285872:exon4:c.T399C:p.G133G,Pafah2:NM | . | 4qD3 | rs27563153 | 1 | 0 |
| chr4 | 134411026 | G | C | 228 | Pafah2 | intronic | . | . | . | 4qD3 | rs52393192 | 1 | 0 |
| chr4 | 134411701 | G | A | 228 | Pafah2 | intronic | . | . | . | 4qD3 | rs32904181 | 1 | 0 |
| chr4 | 134411754 | G | A | 228 | Pafah2 | intronic | . | . | . | 4qD3 | rs27563128 | 1 | 0 |
| chr4 | 134411803 | G | A | 228 | Pafah2 | exonic | synonymous SNV | Pafah2:NM_001285877:exon3:c.G84A:p.P28P,Pafah2:NM_001285874:exon5:c.G444A:p.P148P,Pafah2:NM_( | . | 4qD3 | rs27563127 | 1 | 0 |
| chr4 | 134411922 | T | C | 228 | Pafah2 | intronic | . | . | . | 4qD3 | rs27563125 | 1 | 0 |
| chr4 | 134411948 | G | A | 228 | Pafah2 | intronic | . | . | . | 4qD3 | rs27563124 | 1 | 0 |
| chr4 | 134413209 | G | A | 143 | Pafah2 | intronic | . | . | . | 4qD3 | rs27563115 | 1 | 0 |
| chr4 | 134413513 | C | T | 153 | Pafah2 | intronic | . | . | . | 4qD3 | rs27563114 | 1 | 0 |
| chr4 | 134419855 | T | C | 228 | Pafah2 | intronic | . | . | . | 4qD3 | rs27563083 | 1 | 0 |
| chr4 | 134422164 | C | G | 144 | Pafah2 | intronic | . | . | . | 4qD3 | rs46440653 | 1 | 0 |
| chr4 | 134422280 | A | G | 228 | Pafah2 | intronic | . | . | . | 4qD3 | rs27563070 | 1 | 0 |
| chr4 | 134422543 | A | T | 228 | Pafah2 | intronic | . | . | . | 4qD3 | rs51939453 | 1 | 0 |
| chr4 | 134422565 | G | A | 228 | Pafah2 | intronic | . | . | . | 4qD3 | rs32651412 | 1 | 0 |
| chr4 | 134425305 | C | T | 173 | Pafah2 | intronic | . | . | . | 4qD3 | rs256942310 | 1 | 0 |
| chr4 | 134425412 | A | G | 228 | Pafah2 | intronic | . | . | . | 4qD3 | rs27563061 | 1 | 0 |
| chr4 | 134425603 | C | A | 228 | Pafah2 | UTR3 | . | . | . | 4qD3 | rs32879008 | 1 | 0 |
| chr4 | 134469876 | C | T | 76 | Stmn1 | intronic | . | . | . | 4qD3 | rs46241086 | 1 | 0 |
| chr4 | 134469885 | A | T | 115 | Stmn1 | intronic | . | . | . | 4qD3 | rs31868967 | 1 | 0 |
| chr4 | 134470282 | A | T | 228 | Stmn1 | intronic | . | . | . | 4qD3 | rs27547260 | 1 | 0 |
| chr4 | 134470527 | C | T | 201 | Stmn1 | intronic | . | . | . | 4qD3 | rs27547259 | 1 | 0 |
| chr4 | 134470856 | T | A | 228 | Stmn1 | intronic | . | . | . | 4qD3 | rs27547257 | 1 | 0 |
| chr4 | 134472620 | C | T | 164 | Stmn1 | intronic | . | . | . | 4qD3 | rs27547247 | 1 | 0 |
| chr4 | 134472666 | T | C | 175 | Stmn1 | intronic | . | . | . | 4qD3 | rs27547245 | 1 | 0 |
| chr4 | 134473067 | T | C | 228 | Stmn1 | intronic | . | . | . | 4qD3 | rs27547243 | 1 | 0 |
| chr4 | 134473284 | A | G | 228 | Stmn1 | intronic | . | . | . | 4qD3 | rs27547239 | 1 | 0 |
| chr4 | 134507364 | C | T | 228 | Paqr7 | exonic | synonymous SNV | Paqr7:NM_027995:exon2:c.C531T:p.A177A,Paqr7:NM_001285845:exon3:c.C531T:p.A177A,Paqr7:NM_00128 | . | 4qD3 | rs28310931 | 1 | 0 |
| chr4 | 134507571 | C | T | 228 | Paqr7 | exonic | synonymous SNV | Paqr7:NM_027995:exon2:c.C738T:p.F246F,Paqr7:NM_001285845:exon3:c.C738T:p.F246F,Paqr7:NM_00128 | . | 4qD3 | rs28310928 | 1 | 0 |
| chr4 | 134507817 | T | C | 228 | Paqr7 | exonic | synonymous SNV | Paqr7:NM_027995:exon2:c.T984C:p.T328T,Paqr7:NM_001285845:exon3:c.T984C:p.T328T,Paqr7:NM_00128 | . | 4qD3 | rs32237721 | 1 | 0 |
| chr4 | 134507965 | G | A | 228 | Paqr7 | UTR3 | . | . | . | 4qD3 | rs28310927 | 1 | 0 |
| chr4 | 134508099 | A | G | 228 | Paqr7 | UTR3 | . | . | . | 4qD3 | rs108127854 | 1 | 0 |
| chr4 | 134508301 | C | A | 228 | Paqr7 | UTR3 | . | . | . | 4qD3 | rs28310926 | 1 | 0 |
| chr4 | 134508349 | C | T | 228 | Paqr7 | UTR3 | . | . | . | 4qD3 | rs243076694 | 1 | 0 |
| chr4 | 134508509 | A | T | 228 | Paqr7 | UTR3 | . | . | . | 4qD3 | rs28310924 | 1 | 0 |
| chr4 | 134508907 | A | G | 112 | Paqr7 | UTR3 | . | . | . | 4qD3 | rs49717371 | 1 | 0 |
| chr4 | 134511254 | G | T | 162 | Aunip | intronic | . | . | 360 | 4qD3 | rs50219113 | 1 | 0 |
| chr4 | 134521392 | C | T | 228 | Aunip | intronic | . | . | . | 4qD3 | rs45972791 | 1 | 0 |
| chr4 | 134521667 | C | G | 228 | Aunip | intronic | . | . | . | 4qD3 | rs46606823 | 1 | 0 |
| chr4 | 134521687 | A | G | 228 | Aunip | intronic | . | . | . | 4qD3 | rs49741757 | 1 | 0 |
| chr4 | 134523456 | A | G | 228 | Aunip | exonic | synonymous SNV | Aunip:NM_001081099:exon3:c.A711G:p.G237G | . | 4qD3 | rs226919000 | 1 | 0 |
| chr4 | 134523576 | A | C | 228 | Aunip | exonic | synonymous SNV | Aunip:NM_001081099:exon3:c.A831C:p.S277S | . | 4qD3 | rs28310870 | 1 | 0 |
| chr4 | 134523701 | A | G | 228 | Aunip | exonic | missense SNV | Aunip:NM_001081099:exon3:c.A956G:p.Q319R | . | 4qD3 | rs213823669 | 1 | 0 |
| chr4 | 134523717 | A | T | 228 | Aunip | exonic | synonymous SNV | Aunip:NM_001081099:exon3:c.A972T:p.S324S | . | 4qD3 | rs230871278 | 1 | 0 |
| chr4 | 134523744 | G | A | 228 | Aunip | exonic | synonymous SNV | Aunip:NM_001081099:exon3:c.G999A:p.E333E | . | 4qD3 | rs28310869 | 1 | 0 |
| chr4 | 134523877 | G | A | 165 | Aunip | UTR3 | . | . | . | 4qD3 | . | 1 | 0 |
| chr4 | 134523911 | C | T | 109 | Aunip | UTR3 | . | . | . | 4qD3 | rs28310868 | 1 | 0 |
| chr4 | 134525741 | C | T | 228 | Mtfr1l | UTR3 | . | . | . | 4qD3 | rs13462549 | 1 | 0 |
| chr4 | 134526009 | T | C | 89 | Mtfr1l | UTR3 | . | . | . | 4qD3 | rs13462551 | 1 | 0 |
| chr4 | 134526283 | A | G | 125 | Mtfr1l | UTR3 | . | . | . | 4qD3 | rs28291963 | 1 | 0 |
| chr4 | 134526295 | C | T | 128 | Mtfr1l | UTR3 | . | . | . | 4qD3 | rs28291962 | 1 | 0 |
| chr4 | 134526399 | C | G | 228 | Mtfr1l | UTR3 | . | . | . | 4qD3 | rs32090197 | 1 | 0 |
| chr4 | 134526401 | G | A | 228 | Mtfr1l | UTR3 | . | . | . | 4qD3 | rs28291960 | 1 | 0 |
| chr4 | 134529190 | T | C | 228 | Mtfr1l | exonic | synonymous SNV | Mtfr1l:NM_001256112:exon6:c.A663G:p.S221S,Mtfr1l:NM_029759:exon6:c.A663G:p.S221S | . | 4qD3 | rs28291949 | 1 | 0 |
| chr4 | 134529226 | C | T | 228 | Mtfr1l | exonic | synonymous SNV | Mtfr1l:NM_001256112:exon6:c.G627A:p.E209E,Mtfr1l:NM_029759:exon6:c.G627A:p.E209E | . | 4qD3 | rs28291948 | 1 | 0 |
| chr4 | 134529355 | C | A | 228 | Mtfr1l | exonic | synonymous SNV | Mtfr1l:NM_001256112:exon6:c.G498T:p.S166S,Mtfr1l:NM_029759:exon6:c.G498T:p.S166S | . | 4qD3 | rs28291947 | 1 | 0 |
| chr4 | 134530208 | T | C | 162 | Mtfr1l | intronic | . | . | . | 4qD3 | rs28291946 | 1 | 0 |
| chr4 | 134530300 | G | A | 196 | Mtfr1l | exonic | synonymous SNV | Mtfr1l:NM_001256112:exon5:c.C417T:p.I139I,Mtfr1l:NM_029759:exon5:c.C417T:p.I139I | . | 4qD3 | rs28291945 | 1 | 0 |
| chr4 | 134530405 | A | C | 228 | Mtfr1l | exonic | synonymous SNV | Mtfr1l:NM_001256112:exon5:c.T312G:p.L104L,Mtfr1l:NM_029759:exon5:c.T312G:p.L104L | . | 4qD3 | rs28291944 | 1 | 0 |
| chr4 | 134530498 | G | A | 228 | Mtfr1l | intronic | . | . | . | 4qD3 | rs32115698 | 1 | 0 |
| chr4 | 134530503 | G | A | 228 | Mtfr1l | intronic | . | . | . | 4qD3 | rs32560706 | 1 | 0 |
| chr4 | 134530655 | C | T | 228 | Mtfr1l | intronic | . | . | . | 4qD3 | rs28291942 | 1 | 0 |
| chr4 | 134530873 | A | G | 228 | Mtfr1l | intronic | . | . | . | 4qD3 | rs31925971 | 1 | 0 |
| chr4 | 134530878 | C | T | 228 | Mtfr1l | intronic | . | . | . | 4qD3 | rs32696626 | 1 | 0 |
| chr4 | 134531985 | G | A | 168 | Mtfr1l | intronic | . | . | . | 4qD3 | rs28291933 | 1 | 0 |
| chr4 | 134532207 | T | G | 228 | Mtfr1l | exonic | synonymous SNV | Mtfr1l:NM_001256112:exon3:c.A57C:p.R19R,Mtfr1l:NM_029759:exon3:c.A57C:p.R19R | . | 4qD3 | rs28291932 | 1 | 0 |
| chr4 | 134532443 | T | A | 228 | Mtfr1l | intronic | . | . | . | 4qD3 | rs28291930 | 1 | 0 |
| chr4 | 134539451 | C | T | 228 | Sepn1 | UTR3 | . | . | . | 4qD3 | rs13468444 | 1 | 0 |
| chr4 | 134539570 | G | A | 228 | Sepn1 | UTR3 | . | . | . | 4qD3 | rs13468445 | 1 | 0 |
| chr4 | 134539952 | A | T | 228 | Sepn1 | intronic | . | . | . | 4qD3 | rs32933553 | 1 | 0 |
| chr4 | 134540704 | A | T | 228 | Sepn1 | exonic | unknown | UNKNOWN | . | 4qD3 | rs13468448 | 1 | 0 |
| chr4 | 134541920 | C | T | 148 | Sepn1 | intronic | . | . | . | 4qD3 | rs28291903 | 1 | 0 |
| chr4 | 134542487 | T | C | 228 | Sepn1 | intronic | . | . | . | 4qD3 | rs28291899 | 1 | 0 |
| chr4 | 134542678 | A | T | 103 | Sepn1 | intronic | . | . | . | 4qD3 | rs48913332 | 1 | 0 |
| chr4 | 134542857 | T | C | 228 | Sepn1 | exonic | unknown | UNKNOWN | . | 4qD3 | rs28291896 | 1 | 0 |
| chr4 | 134543156 | C | T | 228 | Sepn1 | intronic | . | . | . | 4qD3 | rs28291893 | 1 | 0 |
| chr4 | 134543167 | A | T | 228 | Sepn1 | intronic | . | . | . | 4qD3 | rs32450257 | 1 | 0 |
| chr4 | 134544481 | C | T | 137 | Sepn1 | intronic | . | . | . | 4qD3 | rs32784665 | 1 | 0 |
| chr4 | 134551193 | C | T | 146 | Sepn1 | intronic | . | . | . | 4qD3 | rs46668743 | 1 | 0 |
| chr4 | 134551310 | T | C | 172 | Sepn1 | intronic | . | . | . | 4qD3 | rs46889794 | 1 | 0 |
| chr4 | 134551551 | A | G | 228 | Sepn1 | intronic | . | . | . | 4qD3 | rs50891971 | 1 | 0 |
| chr4 | 134563731 | A | T | 228 | Man1c1 | exonic | missense SNV | Man1c1:NM_207237:exon12:c.T1853A:p.F618Y | . | 4qD3 | rs256016720 | 1 | 0 |
| chr4 | 134564471 | G | A | 228 | Man1c1 | intronic | . | . | . | 4qD3 | rs28291845 | 1 | 0 |
| chr4 | 134564475 | A | T | 228 | Man1c1 | intronic | . | . | . | 4qD3 | rs28291844 | 1 | 0 |
| chr4 | 134564673 | G | A | 205 | Man1c1 | intronic | . | . | . | 4qD3 | rs28291842 | 1 | 0 |
| chr4 | 134566034 | T | C | 228 | Man1c1 | intronic | . | . | . | 4qD3 | rs28291838 | 1 | 0 |
| chr4 | 134568933 | C | T | 228 | Man1c1 | exonic | synonymous SNV | Man1c1:NM_207237:exon9:c.G1425A:p.Q475Q | . | 4qD3 | rs32872720 | 1 | 0 |
| chr4 | 134575830 | T | G | 214 | Man1c1 | intronic | . | . | . | 4qD3 | rs32943969 | 1 | 0 |
| chr4 | 134576171 | A | G | 228 | Man1c1 | intronic | . | . | . | 4qD3 | rs47730270 | 1 | 0 |
| chr4 | 134576191 | G | A | 228 | Man1c1 | intronic | . | . | . | 4qD3 | rs48249755 | 1 | 0 |
| chr4 | 134578218 | A | T | 228 | Man1c1 | intronic | . | . | . | 4qD3 | rs47380123 | 1 | 0 |
| chr4 | 134578419 | T | C | 228 | Man1c1 | intronic | . | . | . | 4qD3 | rs50120419 | 1 | 0 |
| chr4 | 134578467 | T | C | 214 | Man1c1 | intronic | . | . | . | 4qD3 | rs49900749 | 1 | 0 |
| chr4 | 134580936 | C | T | 228 | Man1c1 | intronic | . | . | . | 4qD3 | rs50370344 | 1 | 0 |
| chr4 | 134640508 | T | C | 157 | Man1c1 | intronic | . | . | . | 4qD3 | rs28276740 | 1 | 0 |
| chr4 | 134703418 | G | T | 228 | Man1c1 | exonic | synonymous SNV | Man1c1:NM_207237:exon1:c.C52A:p.R18R | 1702 | 4qD3 | rs32749789 | 1 | 0 |
| chr4 | 134747295 | T | C | 228 | Ldlrap1 | intronic | . | . | . | 4qD3 | rs258065443 | 1 | 0 |
| chr4 | 134749146 | G | A | 140 | Ldlrap1 | intronic | . | . | . | 4qD3 | rs27591478 | 1 | 0 |
| chr4 | 134749403 | G | A | 228 | Ldlrap1 | exonic | synonymous SNV | Ldlrap1:NM_145554:exon7:c.C678T:p.S226S | . | 4qD3 | . | 1 | 0 |
| chr4 | 134749529 | A | C | 228 | Ldlrap1 | intronic | . | . | . | 4qD3 | rs27591476 | 1 | 0 |
| chr4 | 134750106 | C | A | 228 | Ldlrap1 | intronic | . | . | . | 4qD3 | rs27591471 | 1 | 0 |
| chr4 | 134755110 | G | A | 182 | Ldlrap1 | intronic | . | . | . | 4qD3 | rs27572838 | 1 | 0 |
| chr4 | 134755344 | G | A | 228 | Ldlrap1 | intronic | . | . | . | 4qD3 | rs27572836 | 1 | 0 |
| chr4 | 134755499 | A | G | 134 | Ldlrap1 | intronic | . | . | . | 4qD3 | rs27572834 | 1 | 0 |
| chr4 | 134757242 | T | G | 202 | Ldlrap1 | intronic | . | . | . | 4qD3 | rs27572818 | 1 | 0 |
| chr4 | 134757263 | T | A | 228 | Ldlrap1 | intronic | . | . | . | 4qD3 | rs27572817 | 1 | 0 |
| chr4 | 134757493 | A | G | 228 | Ldlrap1 | intronic | . | . | . | 4qD3 | rs246791355 | 1 | 0 |
| chr4 | 134758994 | G | A | 228 | Ldlrap1 | exonic | synonymous SNV | Ldlrap1:NM_145554:exon2:c.C153T:p.Y51Y | . | 4qD3 | rs13469283 | 1 | 0 |
| chr4 | 134804518 | T | C | 228 | Tmem57 | exonic | synonymous SNV | Tmem57:NM_025382:exon11:c.A1839G:p.K613K | . | 4qD3 | rs27572718 | 1 | 0 |
| chr4 | 134804557 | A | G | 228 | Tmem57 | exonic | synonymous SNV | Tmem57:NM_025382:exon11:c.T1800C:p.I600I | . | 4qD3 | rs27572717 | 1 | 0 |
| chr4 | 134804618 | A | G | 228 | Tmem57 | intronic | . | . | . | 4qD3 | rs27572716 | 1 | 0 |
| chr4 | 134804690 | T | A | 108 | Tmem57 | intronic | . | . | . | 4qD3 | rs27572715 | 1 | 0 |
| chr4 | 134806456 | A | G | 177 | Tmem57 | intronic | . | . | . | 4qD3 | rs27572704 | 1 | 0 |
| chr4 | 134806694 | G | A | 228 | Tmem57 | splicing | . | . | . | 4qD3 | rs27572703 | 1 | 0 |
| chr4 | 134806780 | A | T | 228 | Tmem57 | intronic | . | . | . | 4qD3 | rs27572701 | 1 | 0 |
| chr4 | 134806856 | C | T | 190 | Tmem57 | intronic | . | . | . | 4qD3 | rs27572700 | 1 | 0 |
| chr4 | 134806872 | C | G | 178 | Tmem57 | intronic | . | . | . | 4qD3 | rs27572699 | 1 | 0 |
| chr4 | 134812865 | G | A | 164 | Tmem57 | intronic | . | . | . | 4qD3 | rs27572671 | 1 | 0 |
| chr4 | 134827871 | A | G | 169 | Tmem57 | intronic | . | . | . | 4qD3 | rs27572589 | 1 | 0 |
| chr4 | 134827907 | C | G | 228 | Tmem57 | intronic | . | . | . | 4qD3 | rs27572588 | 1 | 0 |
| chr4 | 134830556 | A | G | 228 | Tmem57 | exonic | synonymous SNV | Tmem57:NM_025382:exon5:c.T645C:p.A215A | . | 4qD3 | rs27572580 | 1 | 0 |
| chr4 | 134833361 | T | C | 228 | Tmem57 | intronic | . | . | . | 4qD3 | rs27572569 | 1 | 0 |
| chr4 | 134833434 | A | G | 215 | Tmem57 | intronic | . | . | . | 4qD3 | rs27572568 | 1 | 0 |
| chr4 | 134838041 | G | A | 228 | Tmem57 | intronic | . | . | . | 4qD3 | rs246181084 | 1 | 0 |
| chr4 | 134864848 | A | G | 207 | Rhd | intronic | . | . | . | 4qD3 | rs27558654 | 1 | 0 |
| chr4 | 134876305 | T | A | 146 | Rhd | intronic | . | . | . | 4qD3 | rs266016525 | 1 | 0 |
| chr4 | 134876436 | A | G | 228 | Rhd | exonic | synonymous SNV | Rhd:NM_011270:exon2:c.A267G:p.G89G | . | 4qD3 | rs31923577 | 1 | 0 |
| chr4 | 134876550 | A | G | 228 | Rhd | intronic | . | . | . | 4qD3 | rs226099718 | 1 | 0 |
| chr4 | 134876590 | A | C | 228 | Rhd | intronic | . | . | . | 4qD3 | rs244405413 | 1 | 0 |
| chr4 | 134879868 | G | C | 178 | Rhd | intronic | . | . | . | 4qD3 | rs32302078 | 1 | 0 |
| chr4 | 134879875 | A | C | 188 | Rhd | intronic | . | . | . | 4qD3 | rs33025331 | 1 | 0 |
| chr4 | 134879975 | G | A | 228 | Rhd | exonic | missense SNV | Rhd:NM_011270:exon3:c.G418A:p.V140M | . | 4qD3 | rs27558574 | 1 | 0 |
| chr4 | 134880107 | C | T | 228 | Rhd | intronic | . | . | . | 4qD3 | rs264249076 | 1 | 0 |
| chr4 | 134880148 | G | A | 228 | Rhd | intronic | . | . | . | 4qD3 | rs27558572 | 1 | 0 |
| chr4 | 134883942 | G | A | 228 | Rhd | intronic | . | . | . | 4qD3 | rs246646840 | 1 | 0 |
| chr4 | 134884299 | T | C | 224 | Rhd | intronic | . | . | . | 4qD3 | rs222557371 | 1 | 0 |
| chr4 | 134884300 | G | A | 219 | Rhd | intronic | . | . | . | 4qD3 | rs242331322 | 1 | 0 |
| chr4 | 134884747 | C | A | 228 | Rhd | intronic | . | . | . | 4qD3 | rs27558553 | 1 | 0 |
| chr4 | 134885103 | C | T | 115 | Rhd | intronic | . | . | . | 4qD3 | rs27558548 | 1 | 0 |
| chr4 | 134885241 | C | G | 228 | Rhd | intronic | . | . | . | 4qD3 | rs27558546 | 1 | 0 |
| chr4 | 134885264 | T | C | 228 | Rhd | exonic | synonymous SNV | Rhd:NM_011270:exon6:c.T825C:p.N275N | . | 4qD3 | rs27558545 | 1 | 0 |
| chr4 | 134885318 | A | T | 228 | Rhd | exonic | synonymous SNV | Rhd:NM_011270:exon6:c.A879T:p.S293S | . | 4qD3 | rs27558544 | 1 | 0 |
| chr4 | 134890147 | G | A | 141 | Rhd | intronic | . | . | . | 4qD3 | rs27558534 | 1 | 0 |
| chr4 | 134890272 | G | T | 228 | Rhd | intronic | . | . | . | 4qD3 | rs27558532 | 1 | 0 |
| chr4 | 134890499 | G | T | 228 | Rhd | intronic | . | . | . | 4qD3 | rs212234358 | 1 | 0 |
| chr4 | 134892081 | T | C | 186 | Rhd | intronic | . | . | . | 4qD3 | rs27558519 | 1 | 0 |
| chr4 | 134892139 | G | A | 228 | Rhd | intronic | . | . | . | 4qD3 | rs27558518 | 1 | 0 |
| chr4 | 134892327 | T | C | 228 | Rhd | intronic | . | . | . | 4qD3 | rs27558516 | 1 | 0 |
| chr4 | 134892373 | C | T | 228 | Rhd | intronic | . | . | . | 4qD3 | rs27558515 | 1 | 0 |
| chr4 | 134894370 | G | A | 228 | Rhd | exonic | missense SNV | Rhd:NM_011270:exon9:c.G1193A:p.R398H | . | 4qD3 | rs27558502 | 1 | 0 |
| chr4 | 134896001 | A | G | 228 | Rhd | UTR3 | . | . | . | 4qD3 | rs27558501 | 1 | 0 |
| chr4 | 134896119 | G | C | 228 | Rhd | UTR3 | . | . | . | 4qD3 | rs27558500 | 1 | 0 |
| chr4 | 134896136 | G | A | 228 | Rhd | UTR3 | . | . | . | 4qD3 | rs27558499 | 1 | 0 |
| chr4 | 134898243 | A | T | 170 | Tmem50a | UTR3 | . | . | . | 4qD3 | rs27558483 | 1 | 0 |
| chr4 | 134898413 | G | T | 228 | Tmem50a | UTR3 | . | . | . | 4qD3 | rs27558482 | 1 | 0 |
| chr4 | 134898533 | A | G | 228 | Tmem50a | intronic | . | . | . | 4qD3 | rs27558480 | 1 | 0 |
| chr4 | 134900669 | A | G | 228 | Tmem50a | intronic | . | . | . | 4qD3 | rs27543467 | 1 | 0 |
| chr4 | 134900775 | T | G | 228 | Tmem50a | intronic | . | . | . | 4qD3 | rs27543466 | 1 | 0 |
| chr4 | 134905183 | A | G | 160 | Tmem50a | intronic | . | . | . | 4qD3 | rs27543443 | 1 | 0 |
| chr4 | 134909657 | A | G | 228 | Tmem50a | exonic | synonymous SNV | Tmem50a:NM_027935:exon3:c.T162C:p.H54H | . | 4qD3 | rs27543409 | 1 | 0 |
| chr4 | 134909775 | G | A | 228 | Tmem50a | intronic | . | . | . | 4qD3 | rs233651720 | 1 | 0 |

| | | | | | | | | | | | | | |
|---|---|---|---|---|---|---|---|---|---|---|---|---|---|
| chr4 | 134909816 | G | A | 228 | Tmem50a | intronic | . | . | . | 4qD3 | rs241825841 | 1 | 0 |
| chr4 | 134913009 | A | C | 110 | Tmem50a | intronic | . | . | . | 4qD3 | rs583234629 | 1 | 0 |
| chr4 | 134913055 | A | C | 228 | Tmem50a | intronic | . | . | . | 4qD3 | rs265108844 | 1 | 0 |
| chr4 | 134913283 | A | G | 176 | Tmem50a | intronic | . | . | . | 4qD3 | rs215039433 | 1 | 0 |
| chr4 | 134924331 | G | A | 228 | Rsrp1 | exonic | synonymous SNV | Rsrp1:NM_023665:exon2:c.G405A:p.Q135Q | 992 | 4qD3 | rs27543375 | 1 | 0 |
| chr4 | 134924463 | A | T | 228 | Rsrp1 | exonic | synonymous SNV | Rsrp1:NM_023665:exon2:c.A537T:p.P179P | . | 4qD3 | rs27543373 | 1 | 0 |
| chr4 | 134924576 | C | G | 146 | Rsrp1 | intronic | . | . | . | 4qD3 | rs223274972 | 1 | 0 |
| chr4 | 134924605 | G | T | 103 | Rsrp1 | intronic | . | . | . | 4qD3 | rs27543372 | 1 | 0 |
| chr4 | 134925224 | T | G | 228 | Rsrp1 | intronic | . | . | . | 4qD3 | rs27543369 | 1 | 0 |
| chr4 | 134925271 | A | C | 228 | Rsrp1 | intronic | . | . | . | 4qD3 | rs27543368 | 1 | 0 |
| chr4 | 134926759 | G | A | 228 | Rsrp1 | exonic | missense SNV | Rsrp1:NM_023665:exon4:c.G721A:p.A241T | . | 4qD3 | rs13470100 | 1 | 0 |
| chr4 | 134927253 | C | T | 169 | Rsrp1 | UTR3 | . | . | . | 4qD3 | rs27543365 | 1 | 0 |
| chr4 | 134930895 | G | A | 228 | . | upstream | . | . | 634 | 4qD3 | rs27543355 | 1 | 0 |
| chr4 | 134931002 | C | A | 228 | Syf2 | exonic | missense SNV | Syf2:NM_026780:exon1:c.C8A:p.A3D | 634 | 4qD3 | rs27543354 | 1 | 0 |
| chr4 | 134931070 | A | G | 228 | Syf2 | intronic | . | . | 634 | 4qD3 | rs251965865 | 1 | 0 |
| chr4 | 134931087 | C | G | 228 | Syf2 | intronic | . | . | 634 | 4qD3 | rs259446358 | 1 | 0 |
| chr4 | 134931317 | T | C | 228 | Syf2 | intronic | . | . | 634 | 4qD3 | rs51651053 | 1 | 0 |
| chr4 | 134931331 | C | G | 228 | Syf2 | intronic | . | . | 634 | 4qD3 | rs241110278 | 1 | 0 |
| chr4 | 134935786 | G | A | 120 | Syf2 | intronic | . | . | . | 4qD3 | rs27543348 | 1 | 0 |
| chr4 | 134936096 | G | A | 228 | Syf2 | intronic | . | . | . | 4qD3 | rs27543346 | 1 | 0 |
| chr4 | 134936234 | G | C | 161 | Syf2 | intronic | . | . | . | 4qD3 | rs27543345 | 1 | 0 |
| chr4 | 134936650 | T | A | 132 | Syf2 | intronic | . | . | . | 4qD3 | rs27543344 | 1 | 0 |
| chr4 | 134937060 | C | T | 228 | Syf2 | UTR3 | . | . | . | 4qD3 | rs243560249 | 1 | 0 |
| chr4 | 135121078 | C | T | 228 | Runx3 | UTR5 | . | . | . | 4qD3 | rs217942781 | 1 | 0 |
| chr4 | 135121201 | G | A | 228 | Runx3 | intronic | . | . | . | 4qD3 | rs231097802 | 1 | 0 |
| chr4 | 135121230 | T | G | 228 | Runx3 | intronic | . | . | . | 4qD3 | rs28335097 | 1 | 0 |
| chr4 | 135121321 | T | C | 190 | Runx3 | intronic | . | . | . | 4qD3 | rs28335096 | 1 | 0 |
| chr4 | 135153141 | G | A | 228 | Runx3 | intronic | . | . | . | 4qD3 | rs6190274 | 1 | 0 |
| chr4 | 135155198 | A | C | 104 | Runx3 | intronic | . | . | . | 4qD3 | rs28335277 | 1 | 0 |
| chr4 | 135155332 | C | G | 228 | Runx3 | exonic | synonymous SNV | Runx3:NM_019732:exon3:c.C378G:p.A126A | . | 4qD3 | rs28335276 | 1 | 0 |
| chr4 | 135155368 | C | T | 228 | Runx3 | exonic | synonymous SNV | Runx3:NM_019732:exon3:c.C414T:p.N138N | . | 4qD3 | rs28335275 | 1 | 0 |
| chr4 | 135163552 | G | A | 110 | Runx3 | intronic | . | . | . | 4qD3 | rs28335390 | 1 | 0 |
| chr4 | 135175250 | T | A | 57 | Runx3 | intronic | . | . | . | 4qD3 | rs240406245 | 1 | 0 |
| chr4 | 135175661 | C | T | 132 | Runx3 | exonic | synonymous SNV | Runx3:NM_019732:exon6:c.C1101T:p.P367P | 498 | 4qD3 | rs244110417 | 1 | 0 |
| chr4 | 135218700 | T | C | 83 | Clic4 | intronic | . | . | 326 | 4qD3 | rs6396271 | 1 | 0 |
| chr4 | 135218740 | T | C | 228 | Clic4 | intronic | . | . | 326 | 4qD3 | rs6396722 | 1 | 0 |
| chr4 | 135223584 | G | A | 228 | Clic4 | intronic | . | . | . | 4qD3 | rs27608442 | 1 | 0 |
| chr4 | 135226128 | G | A | 228 | Clic4 | exonic | synonymous SNV | Clic4:NM_013885:exon3:c.C204T:p.N68N | . | 4qD3 | rs27608427 | 1 | 0 |
| chr4 | 135226185 | A | G | 228 | Clic4 | intronic | . | . | . | 4qD3 | rs27608426 | 1 | 0 |
| chr4 | 135226237 | T | C | 228 | Clic4 | intronic | . | . | . | 4qD3 | rs27608425 | 1 | 0 |
| chr4 | 135239088 | G | A | 223 | Clic4 | intronic | . | . | . | 4qD3 | rs50733245 | 1 | 0 |
| chr4 | 135239178 | G | A | 115 | Clic4 | intronic | . | . | . | 4qD3 | rs27608368 | 1 | 0 |
| chr4 | 135272578 | C | A | 227 | Clic4 | exonic | synonymous SNV | Clic4:NM_013885:exon1:c.G12T:p.S4S | 1045 | 4qD3 | rs32753601 | 1 | 0 |
| chr4 | 135272610 | G | A | 228 | Clic4 | UTR5 | . | . | 1045 | 4qD3 | rs32403163 | 1 | 0 |
| chr4 | 135290306 | G | A | 162 | . | intergenic | . | . | . | 4qD3 | rs222368672 | 1 | 0 |
| chr4 | 135290491 | C | G | 228 | . | intergenic | . | . | . | 4qD3 | rs215852611 | 1 | 0 |
| chr4 | 135321472 | C | A | 228 | Srrm1 | intronic | . | . | . | 4qD3 | rs27583858 | 1 | 0 |
| chr4 | 135321506 | G | A | 228 | Srrm1 | intronic | . | . | . | 4qD3 | rs27583857 | 1 | 0 |
| chr4 | 135322067 | A | G | 119 | Srrm1 | intronic | . | . | . | 4qD3 | rs27583855 | 1 | 0 |
| chr4 | 135322259 | C | T | 228 | Srrm1 | intronic | . | . | . | 4qD3 | rs27583853 | 1 | 0 |
| chr4 | 135322385 | T | C | 228 | Srrm1 | exonic | missense SNV | Srrm1:NM_001130477:exon17:c.A2554G:p.I852V,Srrm1:NM_016799:exon17:c.A2569G:p.I857V | . | 4qD3 | rs257602473 | 1 | 0 |
| chr4 | 135322388 | T | A | 228 | Srrm1 | exonic | missense SNV | Srrm1:NM_001130477:exon17:c.A2551T:p.T851S,Srrm1:NM_016799:exon17:c.A2566T:p.T856S | . | 4qD3 | rs215022487 | 1 | 0 |
| chr4 | 135323923 | G | T | 228 | Srrm1 | exonic | synonymous SNV | Srrm1:NM_001130477:exon16:c.C2430A:p.S810S,Srrm1:NM_016799:exon16:c.C2445A:p.S815S | . | 4qD3 | rs33034331 | 1 | 0 |
| chr4 | 135323927 | A | G | 228 | Srrm1 | exonic | missense SNV | Srrm1:NM_001130477:exon16:c.T2426C:p.L809P,Srrm1:NM_016799:exon16:c.T2441C:p.L814P | . | 4qD3 | rs32298545 | 1 | 0 |
| chr4 | 135324187 | G | C | 228 | Srrm1 | intronic | . | . | . | 4qD3 | rs27583838 | 1 | 0 |
| chr4 | 135325058 | G | A | 228 | Srrm1 | exonic | synonymous SNV | Srrm1:NM_001130477:exon15:c.C2004T:p.P668P,Srrm1:NM_016799:exon15:c.C2019T:p.P673P | . | 4qD3 | rs27583836 | 1 | 0 |
| chr4 | 135325081 | T | G | 228 | Srrm1 | exonic | missense SNV | Srrm1:NM_001130477:exon15:c.A1981C:p.T661P,Srrm1:NM_016799:exon15:c.A1996C:p.T666P | . | 4qD3 | rs27583835 | 1 | 0 |
| chr4 | 135327065 | C | T | 116 | Srrm1 | intronic | . | . | . | 4qD3 | rs27583823 | 1 | 0 |
| chr4 | 135327177 | T | G | 228 | Srrm1 | exonic | synonymous SNV | Srrm1:NM_001130477:exon14:c.A1696C:p.R566R,Srrm1:NM_016799:exon14:c.A1711C:p.R571R | . | 4qD3 | rs244864967 | 1 | 0 |
| chr4 | 135327273 | C | T | 151 | Srrm1 | intronic | . | . | . | 4qD3 | rs27583822 | 1 | 0 |
| chr4 | 135328906 | A | C | 201 | Srrm1 | intronic | . | . | . | 4qD3 | rs27583814 | 1 | 0 |
| chr4 | 135328945 | C | T | 228 | Srrm1 | intronic | . | . | . | 4qD3 | rs27583813 | 1 | 0 |
| chr4 | 135331695 | G | A | 228 | Srrm1 | intronic | . | . | . | 4qD3 | rs27583802 | 1 | 0 |
| chr4 | 135332011 | C | G | 178 | Srrm1 | intronic | . | . | . | 4qD3 | rs27583799 | 1 | 0 |
| chr4 | 135332039 | A | C | 75 | Srrm1 | intronic | . | . | . | 4qD3 | rs225987030 | 1 | 0 |
| chr4 | 135337723 | C | T | 228 | Srrm1 | intronic | . | . | . | 4qD3 | rs27583771 | 1 | 0 |
| chr4 | 135340293 | A | G | 146 | Srrm1 | intronic | . | . | . | 4qD3 | rs27583760 | 1 | 0 |
| chr4 | 135340924 | T | A | 228 | Srrm1 | exonic | synonymous SNV | Srrm1:NM_001130477:exon7:c.A837T:p.R279R,Srrm1:NM_016799:exon7:c.A837T:p.R279R | . | 4qD3 | rs27583755 | 1 | 0 |
| chr4 | 135344036 | C | G | 228 | Srrm1 | intronic | . | . | . | 4qD3 | rs27583743 | 1 | 0 |
| chr4 | 135344136 | C | T | 202 | Srrm1 | intronic | . | . | . | 4qD3 | rs32430684 | 1 | 0 |
| chr4 | 135344200 | C | G | 112 | Srrm1 | intronic | . | . | . | 4qD3 | rs27583742 | 1 | 0 |
| chr4 | 135346594 | C | T | 135 | Srrm1 | intronic | . | . | . | 4qD3 | rs32219040 | 1 | 0 |
| chr4 | 135346941 | T | G | 228 | Srrm1 | intronic | . | . | . | 4qD3 | rs49030661 | 1 | 0 |
| chr4 | 135370454 | T | A | 228 | Ncmap | UTR3 | . | . | . | 4qD3 | rs234499404 | 1 | 0 |
| chr4 | 135370717 | C | T | 228 | Ncmap | intronic | . | . | . | 4qD3 | rs27583674 | 1 | 0 |
| chr4 | 135371980 | G | C | 228 | Ncmap | intronic | . | . | . | 4qD3 | rs3679825 | 1 | 0 |
| chr4 | 135372031 | T | C | 228 | Ncmap | intronic | . | . | . | 4qD3 | rs27634149 | 1 | 0 |
| chr4 | 135377071 | C | A | 228 | Ncmap | exonic | missense SNV | Ncmap:NM_001243306:exon4:c.G50T:p.G17V | . | 4qD3 | rs27634114 | 1 | 0 |
| chr4 | 135380103 | G | C | 228 | Ncmap | intronic | . | . | . | 4qD3 | rs27634083 | 1 | 0 |
| chr4 | 135380107 | C | A | 228 | Ncmap | intronic | . | . | . | 4qD3 | rs259455974 | 1 | 0 |
| chr4 | 135380234 | A | G | 228 | Ncmap | UTR5 | . | . | . | 4qD3 | rs27634081 | 1 | 0 |
| chr4 | 135403258 | C | T | 228 | . | intergenic | . | . | . | 4qD3 | rs258241022 | 1 | 0 |
| chr4 | 135403403 | G | A | 228 | . | intergenic | . | . | . | 4qD3 | rs214742162 | 1 | 0 |
| chr4 | 135416349 | C | T | 228 | Rcan3 | UTR3 | . | . | . | 4qD3 | rs27619484 | 1 | 0 |
| chr4 | 135418331 | C | G | 228 | Rcan3 | intronic | . | . | . | 4qD3 | rs27619469 | 1 | 0 |
| chr4 | 135418353 | T | G | 228 | Rcan3 | intronic | . | . | . | 4qD3 | rs27619468 | 1 | 0 |
| chr4 | 135418368 | G | A | 228 | Rcan3 | intronic | . | . | . | 4qD3 | rs27619467 | 1 | 0 |
| chr4 | 135420019 | C | T | 64 | Rcan3 | intronic | . | . | . | 4qD3 | rs226200188 | 1 | 0 |
| chr4 | 135420124 | T | C | 195 | Rcan3 | intronic | . | . | . | 4qD3 | rs27619461 | 1 | 0 |
| chr4 | 135420306 | C | A | 228 | Rcan3 | exonic | synonymous SNV | Rcan3:NM_022980:exon3:c.G309T:p.A103A | . | 4qD3 | rs253286291 | 1 | 0 |
| chr4 | 135425095 | T | C | 153 | Rcan3 | intronic | . | . | . | 4qD3 | rs255398075 | 1 | 0 |
| chr4 | 135425124 | T | C | 168 | Rcan3 | intronic | . | . | . | 4qD3 | rs27619444 | 1 | 0 |
| chr4 | 135425277 | G | A | 228 | Rcan3 | exonic | synonymous SNV | Rcan3:NM_022980:exon2:c.C132T:p.D44D | . | 4qD3 | rs226260955 | 1 | 0 |
| chr4 | 135425445 | T | G | 228 | Rcan3 | UTR5 | . | . | . | 4qD3 | rs27619443 | 1 | 0 |
| chr4 | 135425512 | C | T | 207 | Rcan3 | intronic | . | . | . | 4qD3 | rs27619442 | 1 | 0 |
| chr4 | 135447397 | C | A | 228 | . | intergenic | . | . | . | 4qD3 | rs27619415 | 1 | 0 |
| chr4 | 135452313 | G | A | 206 | Nipal3 | UTR3 | . | . | . | 4qD3 | rs27619388 | 1 | 0 |
| chr4 | 135452544 | T | C | 228 | Nipal3 | exonic | synonymous SNV | Nipal3:NM_028995:exon12:c.A1062G:p.T354T | . | 4qD3 | rs27619385 | 1 | 0 |
| chr4 | 135463324 | T | C | 135 | Nipal3 | intronic | . | . | . | 4qD3 | rs27604953 | 1 | 0 |
| chr4 | 135463749 | G | T | 228 | Nipal3 | intronic | . | . | . | 4qD3 | rs246828296 | 1 | 0 |
| chr4 | 135463756 | C | T | 228 | Nipal3 | intronic | . | . | . | 4qD3 | rs216029826 | 1 | 0 |
| chr4 | 135463904 | G | A | 228 | Nipal3 | intronic | . | . | . | 4qD3 | rs244581194 | 1 | 0 |
| chr4 | 135465566 | G | A | 162 | Nipal3 | intronic | . | . | . | 4qD3 | rs247192745 | 1 | 0 |
| chr4 | 135465634 | T | G | 228 | Nipal3 | intronic | . | . | . | 4qD3 | rs226415960 | 1 | 0 |
| chr4 | 135466700 | T | C | 159 | Nipal3 | intronic | . | . | . | 4qD3 | rs27604927 | 1 | 0 |
| chr4 | 135468487 | C | T | 228 | Nipal3 | intronic | . | . | . | 4qD3 | rs243492688 | 1 | 0 |
| chr4 | 135468496 | C | T | 228 | Nipal3 | intronic | . | . | . | 4qD3 | rs212894267 | 1 | 0 |
| chr4 | 135468871 | G | A | 206 | Nipal3 | intronic | . | . | . | 4qD3 | rs27604911 | 1 | 0 |
| chr4 | 135471764 | A | G | 163 | Nipal3 | intronic | . | . | . | 4qD3 | rs27604897 | 1 | 0 |
| chr4 | 135471840 | A | G | 228 | Nipal3 | intronic | . | . | . | 4qD3 | rs256474169 | 1 | 0 |
| chr4 | 135474193 | G | C | 191 | Nipal3 | intronic | . | . | . | 4qD3 | rs27604893 | 1 | 0 |
| chr4 | 135474315 | G | A | 228 | Nipal3 | intronic | . | . | . | 4qD3 | rs237629775 | 1 | 0 |
| chr4 | 135474475 | C | G | 228 | Nipal3 | intronic | . | . | . | 4qD3 | rs32362343 | 1 | 0 |
| chr4 | 135477323 | C | T | 220 | Nipal3 | intronic | . | . | . | 4qD3 | rs584825935 | 1 | 0 |
| chr4 | 135477331 | T | C | 195 | Nipal3 | intronic | . | . | . | 4qD3 | rs224852096 | 1 | 0 |
| chr4 | 135477488 | G | A | 228 | Nipal3 | exonic | synonymous SNV | Nipal3:NM_028995:exon5:c.C351T:p.S117S | . | 4qD3 | rs246062645 | 1 | 0 |
| chr4 | 135477587 | A | G | 228 | Nipal3 | intronic | . | . | . | 4qD3 | rs212450326 | 1 | 0 |
| chr4 | 135477671 | T | C | 170 | Nipal3 | intronic | . | . | . | 4qD3 | rs223880788 | 1 | 0 |
| chr4 | 135479511 | A | G | 228 | Nipal3 | intronic | . | . | . | 4qD3 | rs231373113 | 1 | 0 |
| chr4 | 135479582 | A | G | 228 | Nipal3 | exonic | synonymous SNV | Nipal3:NM_028995:exon4:c.T300C:p.F100F | . | 4qD3 | rs212042037 | 1 | 0 |
| chr4 | 135479823 | G | A | 168 | Nipal3 | intronic | . | . | . | 4qD3 | rs224717211 | 1 | 0 |
| chr4 | 135482590 | G | A | 228 | Nipal3 | intronic | . | . | . | 4qD3 | rs255650878 | 1 | 0 |
| chr4 | 135482645 | C | T | 198 | Nipal3 | intronic | . | . | . | 4qD3 | rs227325268 | 1 | 0 |
| chr4 | 135490862 | T | G | 169 | Nipal3 | intronic | . | . | . | 4qD3 | rs261974401 | 1 | 0 |
| chr4 | 135491048 | C | T | 228 | Nipal3 | UTR5 | . | . | . | 4qD3 | rs235939955 | 1 | 0 |
| chr4 | 135491097 | G | A | 155 | Nipal3 | UTR5 | . | . | . | 4qD3 | rs261311217 | 1 | 0 |
| chr4 | 135506294 | T | C | 63 | Stpg1 | intronic | . | . | . | 4qD3 | rs47787445 | 1 | 0 |
| chr4 | 135506644 | A | G | 134 | Stpg1 | intronic | . | . | . | 4qD3 | rs243165510 | 1 | 0 |
| chr4 | 135507872 | A | G | 228 | Stpg1 | intronic | . | . | . | 4qD3 | rs213657200 | 1 | 0 |
| chr4 | 135515074 | C | T | 170 | Stpg1 | intronic | . | . | . | 4qD3 | rs27604819 | 1 | 0 |
| chr4 | 135525398 | G | A | 195 | Stpg1 | intronic | . | . | . | 4qD3 | rs27604757 | 1 | 0 |
| chr4 | 135525408 | C | T | 197 | Stpg1 | intronic | . | . | . | 4qD3 | rs27604756 | 1 | 0 |
| chr4 | 135525607 | G | A | 194 | Stpg1 | intronic | . | . | . | 4qD3 | rs229982189 | 1 | 0 |
| chr4 | 135525613 | C | T | 148 | Stpg1 | intronic | . | . | . | 4qD3 | rs226074330 | 1 | 0 |
| chr4 | 135529223 | T | G | 59 | Stpg1 | intronic | . | . | . | 4qD3 | rs27604736 | 1 | 0 |
| chr4 | 135529230 | T | C | 98 | Stpg1 | intronic | . | . | . | 4qD3 | rs244332006 | 1 | 0 |
| chr4 | 135529448 | A | T | 228 | Stpg1 | intronic | . | . | . | 4qD3 | rs27604732 | 1 | 0 |
| chr4 | 135529733 | C | T | 228 | Stpg1 | intronic | . | . | . | 4qD3 | rs247372200 | 1 | 0 |
| chr4 | 135533654 | G | C | 228 | Stpg1 | exonic | synonymous SNV | Stpg1:NM_030189:exon8:c.G774C:p.L258L | . | 4qD3 | rs266236522 | 1 | 0 |
| chr4 | 135533687 | G | A | 228 | Stpg1 | exonic | synonymous SNV | Stpg1:NM_030189:exon8:c.G807A:p.P269P | . | 4qD3 | rs226111737 | 1 | 0 |
| chr4 | 135533852 | T | C | 228 | Stpg1 | intronic | . | . | . | 4qD3 | rs27604717 | 1 | 0 |
| chr4 | 135533893 | A | G | 228 | Stpg1 | intronic | . | . | . | 4qD3 | rs27604716 | 1 | 0 |
| chr4 | 135536435 | G | A | 170 | Stpg1 | intronic | . | . | . | 4qD3 | rs27604710 | 1 | 0 |
| chr4 | 135536562 | C | T | 228 | Stpg1 | intronic | . | . | . | 4qD3 | rs27604709 | 1 | 0 |
| chr4 | 135536692 | G | C | 228 | Stpg1 | UTR3 | . | . | . | 4qD3 | rs226633431 | 1 | 0 |
| chr4 | 135542642 | C | T | 228 | Grhl3 | UTR3 | . | . | . | 4qD3 | rs27580959 | 1 | 0 |
| chr4 | 135542818 | A | G | 228 | Grhl3 | intronic | . | . | . | 4qD3 | rs27580957 | 1 | 0 |
| chr4 | 135542896 | C | T | 202 | Grhl3 | intronic | . | . | . | 4qD3 | rs27580956 | 1 | 0 |
| chr4 | 135546068 | C | T | 165 | Grhl3 | intronic | . | . | . | 4qD3 | rs27580929 | 1 | 0 |
| chr4 | 135549094 | T | C | 185 | Grhl3 | intronic | . | . | . | 4qD3 | rs27580922 | 1 | 0 |
| chr4 | 135549229 | C | G | 228 | Grhl3 | intronic | . | . | . | 4qD3 | rs27580921 | 1 | 0 |
| chr4 | 135549385 | T | G | 228 | Grhl3 | intronic | . | . | . | 4qD3 | rs222486621 | 1 | 0 |
| chr4 | 135550261 | A | G | 228 | Grhl3 | intronic | . | . | . | 4qD3 | rs27580919 | 1 | 0 |
| chr4 | 135550432 | C | T | 228 | Grhl3 | intronic | . | . | . | 4qD3 | rs27580918 | 1 | 0 |
| chr4 | 135550451 | T | G | 202 | Grhl3 | intronic | . | . | . | 4qD3 | rs222442783 | 1 | 0 |
| chr4 | 135551786 | A | T | 228 | Grhl3 | exonic | missense SNV | Grhl3:NM_001013756:exon11:c.T1382A:p.I461N | . | 4qD3 | rs27580905 | 1 | 0 |
| chr4 | 135552058 | G | C | 228 | Grhl3 | exonic | synonymous SNV | Grhl3:NM_001013756:exon10:c.C1227G:p.R409R | . | 4qD3 | rs27580904 | 1 | 0 |
| chr4 | 135552073 | A | G | 228 | Grhl3 | exonic | synonymous SNV | Grhl3:NM_001013756:exon10:c.T1212C:p.A404A | . | 4qD3 | rs27580903 | 1 | 0 |
| chr4 | 135552208 | C | T | 166 | Grhl3 | intronic | . | . | . | 4qD3 | rs27580902 | 1 | 0 |
| chr4 | 135552689 | A | G | 228 | Grhl3 | exonic | synonymous SNV | Grhl3:NM_001013756:exon9:c.T1053C:p.F351F | . | 4qD3 | rs27580898 | 1 | 0 |
| chr4 | 135552716 | A | G | 228 | Grhl3 | intronic | . | . | . | 4qD3 | rs27580897 | 1 | 0 |
| chr4 | 135554285 | C | T | 137 | Grhl3 | intronic | . | . | . | 4qD3 | rs27580884 | 1 | 0 |
| chr4 | 135554446 | A | G | 228 | Grhl3 | intronic | . | . | . | 4qD3 | rs27580883 | 1 | 0 |
| chr4 | 135554652 | G | T | 228 | Grhl3 | intronic | . | . | . | 4qD3 | rs263085859 | 1 | 0 |
| chr4 | 135554731 | C | G | 204 | Grhl3 | intronic | . | . | . | 4qD3 | rs27580881 | 1 | 0 |
| chr4 | 135556319 | G | A | 228 | Grhl3 | intronic | . | . | . | 4qD3 | rs265910958 | 1 | 0 |
| chr4 | 135556333 | G | A | 228 | Grhl3 | intronic | . | . | . | 4qD3 | rs226696211 | 1 | 0 |
| chr4 | 135556383 | G | C | 137 | Grhl3 | intronic | . | . | . | 4qD3 | rs27580871 | 1 | 0 |
| chr4 | 135556662 | C | T | 164 | Grhl3 | intronic | . | . | . | 4qD3 | rs27580869 | 1 | 0 |
| chr4 | 135556823 | T | C | 228 | Grhl3 | intronic | . | . | . | 4qD3 | rs27580868 | 1 | 0 |
| chr4 | 135556824 | G | A | 228 | Grhl3 | intronic | . | . | . | 4qD3 | rs245198953 | 1 | 0 |
| chr4 | 135556975 | G | C | 228 | Grhl3 | intronic | . | . | . | 4qD3 | rs256162847 | 1 | 0 |
| chr4 | 135557161 | C | T | 228 | Grhl3 | intronic | . | . | . | 4qD3 | rs235274778 | 1 | 0 |
| chr4 | 135557297 | G | T | 228 | Grhl3 | exonic | missense SNV | Grhl3:NM_001013756:exon4:c.C483A:p.D161E | . | 4qD3 | rs27580863 | 1 | 0 |
| chr4 | 135557358 | G | T | 228 | Grhl3 | exonic | missense SNV | Grhl3:NM_001013756:exon4:c.C422A:p.T141N | . | 4qD3 | rs259420198 | 1 | 0 |
| chr4 | 135557393 | T | C | 228 | Grhl3 | exonic | synonymous SNV | Grhl3:NM_001013756:exon4:c.A387G:p.K129K | . | 4qD3 | rs27580862 | 1 | 0 |
| chr4 | 135557586 | G | A | 228 | Grhl3 | intronic | . | . | . | 4qD3 | rs27580861 | 1 | 0 |
| chr4 | 135557604 | T | C | 228 | Grhl3 | intronic | . | . | . | 4qD3 | rs27580860 | 1 | 0 |
| chr4 | 135557717 | T | C | 88 | Grhl3 | intronic | . | . | . | 4qD3 | rs231887210 | 1 | 0 |
| chr4 | 135558888 | G | A | 185 | Grhl3 | intronic | . | . | . | 4qD3 | rs27580852 | 1 | 0 |
| chr4 | 135559148 | C | T | 228 | Grhl3 | exonic | missense SNV | Grhl3:NM_001013756:exon3:c.G221A:p.R74Q | . | 4qD3 | rs27580850 | 1 | 0 |
| chr4 | 135561109 | C | T | 228 | Grhl3 | intronic | . | . | . | 4qD3 | rs27580832 | 1 | 0 |
| chr4 | 135698701 | A | G | 221 | Ifnlr1 | intronic | . | . | . | 4qD3 | rs27601967 | 1 | 0 |
| chr4 | 135703525 | C | G | 70 | Ifnlr1 | intronic | . | . | . | 4qD3 | rs216835772 | 1 | 0 |
| chr4 | 135703929 | G | A | 228 | Ifnlr1 | intronic | . | . | . | 4qD3 | rs27601959 | 1 | 0 |

| | | | | | | | | | | | | | |
|---|---|---|---|---|---|---|---|---|---|---|---|---|---|
| chr4 | 135704141 | A | T | 228 | Ifnlr1 | intronic | . | . | . | 4qD3 | rs27601957 | 1 | 0 |
| chr4 | 135704948 | G | C | 153 | Ifnlr1 | intronic | . | . | . | 4qD3 | rs27601951 | 1 | 0 |
| chr4 | 135705256 | C | T | 228 | Ifnlr1 | exonic | synonymous SNV | Ifnlr1:NM_174851:exon7:c.C1002T:p.D334D | . | 4qD3 | rs27601949 | 1 | 0 |
| chr4 | 135705327 | C | T | 228 | Ifnlr1 | exonic | missense SNV | Ifnlr1:NM_174851:exon7:c.C1073T:p.S358L | . | 4qD3 | rs256904584 | 1 | 0 |
| chr4 | 135705370 | A | T | 228 | Ifnlr1 | exonic | synonymous SNV | Ifnlr1:NM_174851:exon7:c.A1116T:p.T372T | . | 4qD3 | rs254994601 | 1 | 0 |
| chr4 | 135705502 | G | A | 228 | Ifnlr1 | exonic | synonymous SNV | Ifnlr1:NM_174851:exon7:c.G1248A:p.A416A | . | 4qD3 | rs27601947 | 1 | 0 |
| chr4 | 135705604 | G | A | 228 | Ifnlr1 | exonic | synonymous SNV | Ifnlr1:NM_174851:exon7:c.G1350A:p.R450R | . | 4qD3 | rs27601945 | 1 | 0 |
| chr4 | 135715859 | G | A | 228 | . | intergenic | . | . | . | 4qD3 | rs27601884 | 1 | 0 |
| chr4 | 135716021 | T | C | 228 | . | intergenic | . | . | . | 4qD3 | rs27601883 | 1 | 0 |
| chr4 | 135716063 | A | G | 228 | . | intergenic | . | . | . | 4qD3 | rs27601882 | 1 | 0 |
| chr4 | 135716158 | C | T | 228 | . | intergenic | . | . | . | 4qD3 | rs27601881 | 1 | 0 |
| chr4 | 135716269 | C | T | 228 | . | intergenic | . | . | . | 4qD3 | rs27601880 | 1 | 0 |
| chr4 | 135716356 | T | C | 228 | . | intergenic | . | . | . | 4qD3 | rs27601879 | 1 | 0 |
| chr4 | 135716393 | A | G | 228 | . | intergenic | . | . | . | 4qD3 | rs27601878 | 1 | 0 |
| chr4 | 135728190 | C | A | 228 | Il22ra1 | UTR5 | . | . | . | 4qD3 | rs247567466 | 1 | 0 |
| chr4 | 135732884 | G | A | 135 | Il22ra1 | intronic | . | . | . | 4qD3 | rs587256739 | 1 | 0 |
| chr4 | 135732991 | G | C | 228 | Il22ra1 | exonic | synonymous SNV | Il22ra1:NM_178257:exon2:c.G57C:p.V19V | . | 4qD3 | rs27601801 | 1 | 0 |
| chr4 | 135733118 | C | T | 228 | Il22ra1 | intronic | . | . | . | 4qD3 | rs232228284 | 1 | 0 |
| chr4 | 135733150 | T | C | 228 | Il22ra1 | intronic | . | . | . | 4qD3 | rs229990541 | 1 | 0 |
| chr4 | 135734302 | A | C | 228 | Il22ra1 | intronic | . | . | . | 4qD3 | rs27601785 | 1 | 0 |
| chr4 | 135734455 | A | G | 116 | Il22ra1 | intronic | . | . | . | 4qD3 | rs239658392 | 1 | 0 |
| chr4 | 135737353 | G | A | 228 | Il22ra1 | exonic | synonymous SNV | Il22ra1:NM_178257:exon4:c.G369A:p.P123P | . | 4qD3 | rs27601761 | 1 | 0 |
| chr4 | 135737544 | C | T | 228 | Il22ra1 | intronic | . | . | . | 4qD3 | rs233727061 | 1 | 0 |
| chr4 | 135737594 | A | G | 220 | Il22ra1 | intronic | . | . | . | 4qD3 | rs27601759 | 1 | 0 |
| chr4 | 135748024 | A | G | 130 | Il22ra1 | intronic | . | . | . | 4qD3 | rs27601703 | 1 | 0 |
| chr4 | 135748116 | G | A | 222 | Il22ra1 | exonic | synonymous SNV | Il22ra1:NM_178257:exon6:c.G696A:p.S232S | . | 4qD3 | rs27601702 | 1 | 0 |
| chr4 | 135748337 | G | A | 145 | Il22ra1 | intronic | . | . | . | 4qD3 | rs27601701 | 1 | 0 |
| chr4 | 135748343 | C | T | 143 | Il22ra1 | intronic | . | . | . | 4qD3 | rs251416400 | 1 | 0 |
| chr4 | 135750381 | T | C | 194 | Il22ra1 | intronic | . | . | . | 4qD3 | rs27601694 | 1 | 0 |
| chr4 | 135750420 | A | G | 228 | Il22ra1 | exonic | synonymous SNV | Il22ra1:NM_178257:exon7:c.A801G:p.Q267Q | . | 4qD3 | rs27601693 | 1 | 0 |
| chr4 | 135750609 | A | G | 228 | Il22ra1 | exonic | synonymous SNV | Il22ra1:NM_178257:exon7:c.A990G:p.S330S | . | 4qD3 | rs234333320 | 1 | 0 |
| chr4 | 135750768 | G | A | 228 | Il22ra1 | exonic | missense SNV | Il22ra1:NM_178257:exon7:c.G1149A:p.M383I | . | 4qD3 | rs27601691 | 1 | 0 |
| chr4 | 135750825 | T | C | 228 | Il22ra1 | exonic | synonymous SNV | Il22ra1:NM_178257:exon7:c.T1206C:p.S402S | . | 4qD3 | rs27601690 | 1 | 0 |
| chr4 | 135756295 | A | G | 228 | . | downstream | . | . | . | 4qD3 | rs218343795 | 1 | 0 |
| chr4 | 135756309 | T | G | 228 | . | downstream | . | . | . | 4qD3 | rs240529024 | 1 | 0 |
| chr4 | 135756493 | A | G | 228 | . | intergenic | . | . | . | 4qD3 | rs216673159 | 1 | 0 |
| chr4 | 135756617 | C | T | 205 | . | intergenic | . | . | . | 4qD3 | rs27579464 | 1 | 0 |
| chr4 | 135762572 | C | G | 228 | Myom3 | exonic | missense SNV | Myom3:NM_001085509:exon2:c.C53G:p.T18S | . | 4qD3 | rs239179494 | 1 | 0 |
| chr4 | 135763588 | T | A | 228 | Myom3 | intronic | . | . | . | 4qD3 | rs220152435 | 1 | 0 |
| chr4 | 135763601 | G | A | 228 | Myom3 | intronic | . | . | . | 4qD3 | rs581754761 | 1 | 0 |
| chr4 | 135763607 | G | C | 106 | Myom3 | intronic | . | . | . | 4qD3 | rs580124151 | 1 | 0 |
| chr4 | 135763612 | A | T | 115 | Myom3 | intronic | . | . | . | 4qD3 | rs580595682 | 1 | 0 |
| chr4 | 135763784 | A | G | 228 | Myom3 | intronic | . | . | . | 4qD3 | rs229825871 | 1 | 0 |
| chr4 | 135764713 | T | A | 169 | Myom3 | intronic | . | . | . | 4qD3 | rs245782163 | 1 | 0 |
| chr4 | 135764956 | A | G | 228 | Myom3 | intronic | . | . | . | 4qD3 | rs223967691 | 1 | 0 |
| chr4 | 135764973 | T | C | 228 | Myom3 | intronic | . | . | . | 4qD3 | rs253915232 | 1 | 0 |
| chr4 | 135765425 | T | C | 176 | Myom3 | intronic | . | . | . | 4qD3 | rs27579404 | 1 | 0 |
| chr4 | 135765583 | T | C | 228 | Myom3 | exonic | synonymous SNV | Myom3:NM_001085509:exon5:c.T442C:p.L148L | . | 4qD3 | rs27579403 | 1 | 0 |
| chr4 | 135770569 | T | C | 228 | Myom3 | intronic | . | . | . | 4qD3 | rs234642876 | 1 | 0 |
| chr4 | 135770804 | C | T | 228 | Myom3 | intronic | . | . | . | 4qD3 | rs237759999 | 1 | 0 |
| chr4 | 135771583 | C | T | 228 | Myom3 | exonic | synonymous SNV | Myom3:NM_001085509:exon7:c.C708T:p.Y236Y | . | 4qD3 | rs27579360 | 1 | 0 |
| chr4 | 135773757 | G | C | 228 | Myom3 | intronic | . | . | . | 4qD3 | rs27579345 | 1 | 0 |
| chr4 | 135774315 | G | T | 228 | Myom3 | exonic | synonymous SNV | Myom3:NM_001085509:exon9:c.G825T:p.S275S | . | 4qD3 | rs27579342 | 1 | 0 |
| chr4 | 135774363 | C | T | 228 | Myom3 | exonic | synonymous SNV | Myom3:NM_001085509:exon9:c.C873T:p.T291T | . | 4qD3 | rs249325461 | 1 | 0 |
| chr4 | 135774593 | T | C | 167 | Myom3 | intronic | . | . | . | 4qD3 | rs50864621 | 1 | 0 |
| chr4 | 135775804 | T | C | 228 | Myom3 | intronic | . | . | . | 4qD3 | rs27579333 | 1 | 0 |
| chr4 | 135776257 | A | G | 228 | Myom3 | intronic | . | . | . | 4qD3 | rs235354826 | 1 | 0 |
| chr4 | 135777856 | A | G | 112 | Myom3 | intronic | . | . | . | 4qD3 | rs27579319 | 1 | 0 |
| chr4 | 135778124 | A | G | 228 | Myom3 | exonic | synonymous SNV | Myom3:NM_001085509:exon12:c.A1323G:p.G441G | . | 4qD3 | rs242910891 | 1 | 0 |
| chr4 | 135778135 | A | G | 228 | Myom3 | exonic | missense SNV | Myom3:NM_001085509:exon12:c.A1334G:p.Q445R | . | 4qD3 | rs265248528 | 1 | 0 |
| chr4 | 135779109 | C | T | 115 | Myom3 | intronic | . | . | . | 4qD3 | rs230191910 | 1 | 0 |
| chr4 | 135779274 | T | C | 228 | Myom3 | intronic | . | . | . | 4qD3 | rs234233053 | 1 | 0 |
| chr4 | 135779396 | C | T | 228 | Myom3 | exonic | synonymous SNV | Myom3:NM_001085509:exon13:c.C1479T:p.N493N | . | 4qD3 | rs214217302 | 1 | 0 |
| chr4 | 135779521 | G | A | 228 | Myom3 | intronic | . | . | . | 4qD3 | rs217714014 | 1 | 0 |
| chr4 | 135779532 | A | G | 211 | Myom3 | intronic | . | . | . | 4qD3 | rs252481849 | 1 | 0 |
| chr4 | 135779837 | C | T | 228 | Myom3 | exonic | synonymous SNV | Myom3:NM_001085509:exon14:c.C1527T:p.H509H | . | 4qD3 | rs47023357 | 1 | 0 |
| chr4 | 135779843 | C | T | 228 | Myom3 | exonic | synonymous SNV | Myom3:NM_001085509:exon14:c.C1533T:p.S511S | . | 4qD3 | rs50527014 | 1 | 0 |
| chr4 | 135779906 | G | A | 228 | Myom3 | exonic | synonymous SNV | Myom3:NM_001085509:exon14:c.G1596A:p.P532P | . | 4qD3 | rs225075093 | 1 | 0 |
| chr4 | 135782889 | T | A | 194 | Myom3 | intronic | . | . | . | 4qD3 | rs32684209 | 1 | 0 |
| chr4 | 135782909 | C | A | 202 | Myom3 | intronic | . | . | . | 4qD3 | rs32147779 | 1 | 0 |
| chr4 | 135785790 | T | C | 196 | Myom3 | intronic | . | . | . | 4qD3 | rs32612023 | 1 | 0 |
| chr4 | 135785876 | G | A | 228 | Myom3 | exonic | missense SNV | Myom3:NM_001085509:exon16:c.G1864A:p.A622T | . | 4qD3 | rs224830733 | 1 | 0 |
| chr4 | 135787307 | C | T | 228 | Myom3 | intronic | . | . | . | 4qD3 | rs245173687 | 1 | 0 |
| chr4 | 135787598 | A | G | 228 | Myom3 | intronic | . | . | . | 4qD3 | rs256144984 | 1 | 0 |
| chr4 | 135787611 | C | T | 228 | Myom3 | intronic | . | . | . | 4qD3 | rs225135947 | 1 | 0 |
| chr4 | 135787820 | T | G | 145 | Myom3 | intronic | . | . | . | 4qD3 | rs253437602 | 1 | 0 |
| chr4 | 135787876 | A | C | 228 | Myom3 | intronic | . | . | . | 4qD3 | rs226442703 | 1 | 0 |
| chr4 | 135787929 | A | G | 228 | Myom3 | exonic | synonymous SNV | Myom3:NM_001085509:exon18:c.A2133G:p.L711L | . | 4qD3 | rs244269825 | 1 | 0 |
| chr4 | 135788008 | A | C | 228 | Myom3 | exonic | missense SNV | Myom3:NM_001085509:exon18:c.A2212C:p.M738L | . | 4qD3 | rs211782430 | 1 | 0 |
| chr4 | 135788256 | T | A | 228 | Myom3 | intronic | . | . | . | 4qD3 | rs249672774 | 1 | 0 |
| chr4 | 135795036 | C | A | 228 | Myom3 | intronic | . | . | . | 4qD3 | rs242211339 | 1 | 0 |
| chr4 | 135795042 | A | G | 228 | Myom3 | intronic | . | . | . | 4qD3 | rs260997631 | 1 | 0 |
| chr4 | 135795227 | T | C | 228 | Myom3 | intronic | . | . | . | 4qD3 | rs240263236 | 1 | 0 |
| chr4 | 135795236 | C | T | 228 | Myom3 | intronic | . | . | . | 4qD3 | rs220359428 | 1 | 0 |
| chr4 | 135796147 | A | G | 151 | Myom3 | intronic | . | . | . | 4qD3 | rs52336282 | 1 | 0 |
| chr4 | 135796388 | C | A | 228 | Myom3 | exonic | missense SNV | Myom3:NM_001085509:exon22:c.C2815A:p.Q939K | . | 4qD3 | rs252387874 | 1 | 0 |
| chr4 | 135797156 | T | C | 228 | Myom3 | intronic | . | . | . | 4qD3 | rs243903794 | 1 | 0 |
| chr4 | 135797261 | G | C | 228 | Myom3 | intronic | . | . | . | 4qD3 | rs250146887 | 1 | 0 |
| chr4 | 135797324 | A | T | 228 | Myom3 | intronic | . | . | . | 4qD3 | rs236689232 | 1 | 0 |
| chr4 | 135797373 | G | A | 228 | Myom3 | intronic | . | . | . | 4qD3 | rs253190873 | 1 | 0 |
| chr4 | 135798540 | G | T | 133 | Myom3 | intronic | . | . | . | 4qD3 | rs226128184 | 1 | 0 |
| chr4 | 135803213 | A | G | 228 | Myom3 | exonic | synonymous SNV | Myom3:NM_001085509:exon29:c.A3435G:p.T1145T | . | 4qD3 | rs27579305 | 1 | 0 |
| chr4 | 135803419 | C | T | 175 | Myom3 | intronic | . | . | . | 4qD3 | rs27579303 | 1 | 0 |
| chr4 | 135806204 | T | C | 228 | Myom3 | intronic | . | . | . | 4qD3 | rs27579286 | 1 | 0 |
| chr4 | 135806725 | T | C | 144 | Myom3 | intronic | . | . | . | 4qD3 | rs586189433 | 1 | 0 |
| chr4 | 135806936 | G | C | 228 | Myom3 | intronic | . | . | . | 4qD3 | rs230709663 | 1 | 0 |
| chr4 | 135806957 | A | G | 228 | Myom3 | intronic | . | . | . | 4qD3 | rs27579282 | 1 | 0 |
| chr4 | 135807050 | A | G | 228 | Myom3 | intronic | . | . | . | 4qD3 | rs263472477 | 1 | 0 |
| chr4 | 135807397 | G | A | 101 | Myom3 | intronic | . | . | . | 4qD3 | rs27579280 | 1 | 0 |
| chr4 | 135808687 | T | C | 228 | Myom3 | intronic | . | . | . | 4qD3 | rs27579270 | 1 | 0 |
| chr4 | 135808720 | C | T | 228 | Myom3 | intronic | . | . | . | 4qD3 | rs27579269 | 1 | 0 |
| chr4 | 135808817 | C | T | 228 | Myom3 | exonic | synonymous SNV | Myom3:NM_001085509:exon33:c.C3798T:p.G1266G | . | 4qD3 | rs27579268 | 1 | 0 |
| chr4 | 135808889 | A | G | 228 | Myom3 | exonic | synonymous SNV | Myom3:NM_001085509:exon33:c.A3870G:p.K1290K | . | 4qD3 | rs27579267 | 1 | 0 |
| chr4 | 135808955 | A | G | 228 | Myom3 | exonic | synonymous SNV | Myom3:NM_001085509:exon33:c.A3936G:p.G1312G | . | 4qD3 | rs27579266 | 1 | 0 |
| chr4 | 135810771 | G | A | 228 | Myom3 | exonic | synonymous SNV | Myom3:NM_001085509:exon34:c.G3963A:p.E1321E | . | 4qD3 | rs258568025 | 1 | 0 |
| chr4 | 135810780 | A | G | 228 | Myom3 | exonic | synonymous SNV | Myom3:NM_001085509:exon34:c.A3972G:p.R1324R | . | 4qD3 | rs27579262 | 1 | 0 |
| chr4 | 135811118 | A | C | 158 | Myom3 | intronic | . | . | . | 4qD3 | rs27579260 | 1 | 0 |
| chr4 | 135811139 | G | T | 143 | Myom3 | intronic | . | . | . | 4qD3 | rs27579259 | 1 | 0 |
| chr4 | 135812288 | G | A | 184 | Myom3 | intronic | . | . | . | 4qD3 | rs27579244 | 1 | 0 |
| chr4 | 135814427 | A | G | 228 | Myom3 | UTR3 | . | . | . | 4qD3 | rs216225168 | 1 | 0 |
| chr4 | 135856159 | C | T | 201 | Srsf10 | UTR5 | . | . | 1017 | 4qD3 | rs261978705 | 1 | 0 |
| chr4 | 135856224 | A | G | 228 | Srsf10 | UTR5 | . | . | 1017 | 4qD3 | rs6349342 | 1 | 0 |
| chr4 | 135861691 | G | A | 228 | Srsf10 | intronic | . | . | . | 4qD3 | rs32241517 | 1 | 0 |
| chr4 | 135861692 | C | T | 228 | Srsf10 | intronic | . | . | . | 4qD3 | rs27635620 | 1 | 0 |
| chr4 | 135864235 | G | A | 140 | Srsf10 | UTR3 | . | . | . | 4qD3 | rs13466535 | 1 | 0 |
| chr4 | 135871983 | C | T | 228 | Pnrc2 | UTR3 | . | . | . | 4qD3 | rs32405035 | 1 | 0 |
| chr4 | 135872315 | T | A | 228 | Pnrc2 | exonic | missense SNV | Pnrc2:NM_026383:exon3:c.A122T:p.H41L | . | 4qD3 | rs13478000 | 1 | 0 |
| chr4 | 135872481 | A | C | 171 | Pnrc2 | intronic | . | . | . | 4qD3 | . | 0 | 1 |
| chr4 | 135872604 | T | C | 167 | Pnrc2 | intronic | . | . | . | 4qD3 | rs27635590 | 1 | 0 |
| chr4 | 135872606 | G | A | 167 | Pnrc2 | intronic | . | . | . | 4qD3 | rs32453492 | 1 | 0 |
| chr4 | 135891280 | G | A | 228 | . | intergenic | . | . | . | 4qD3 | rs32966580 | 1 | 0 |
| chr4 | 135891305 | A | G | 228 | . | intergenic | . | . | . | 4qD3 | rs32613010 | 1 | 0 |
| chr4 | 135916699 | C | T | 228 | Cnr2 | exonic | synonymous SNV | Cnr2:NM_001305278:exon2:c.C87T:p.S29S,Cnr2:NM_009924:exon2:c.C87T:p.S29S | . | 4qD3 | rs27620184 | 1 | 0 |
| chr4 | 135917362 | G | T | 228 | Cnr2 | exonic | synonymous SNV | Cnr2:NM_001305278:exon2:c.G750T:p.V250V,Cnr2:NM_009924:exon2:c.G750T:p.V250V | . | 4qD3 | rs27620183 | 1 | 0 |
| chr4 | 135917427 | C | T | 228 | Cnr2 | exonic | missense SNV | Cnr2:NM_001305278:exon2:c.C815T:p.T272M,Cnr2:NM_009924:exon2:c.C815T:p.T272M | . | 4qD3 | rs32448315 | 1 | 0 |
| chr4 | 135917530 | T | C | 228 | Cnr2 | exonic | synonymous SNV | Cnr2:NM_001305278:exon2:c.T918C:p.I306I,Cnr2:NM_009924:exon2:c.T918C:p.I306I | . | 4qD3 | rs27620182 | 1 | 0 |
| chr4 | 135917607 | G | C | 228 | Cnr2 | exonic | missense SNV | Cnr2:NM_001305278:exon2:c.G995C:p.G332A,Cnr2:NM_009924:exon2:c.G995C:p.G332A | . | 4qD3 | rs27620181 | 1 | 0 |
| chr4 | 135917882 | A | G | 228 | Cnr2 | UTR3 | . | . | . | 4qD3 | rs27620180 | 1 | 0 |
| chr4 | 135917924 | T | C | 228 | Cnr2 | UTR3 | . | . | . | 4qD3 | rs27620179 | 1 | 0 |
| chr4 | 135917939 | G | C | 228 | Cnr2 | UTR3 | . | . | . | 4qD3 | rs27620178 | 1 | 0 |
| chr4 | 135918328 | C | T | 228 | Cnr2 | UTR3 | . | . | . | 4qD3 | rs27620177 | 1 | 0 |
| chr4 | 135918821 | G | A | 228 | Cnr2 | UTR3 | . | . | . | 4qD3 | rs27620176 | 1 | 0 |
| chr4 | 135918911 | A | C | 228 | Cnr2 | UTR3 | . | . | . | 4qD3 | rs27620175 | 1 | 0 |
| chr4 | 135919434 | C | T | 168 | Cnr2 | UTR3 | . | . | . | 4qD3 | rs27620172 | 1 | 0 |
| chr4 | 135919547 | T | A | 228 | Cnr2 | UTR3 | . | . | . | 4qD3 | rs27620171 | 1 | 0 |
| chr4 | 135921192 | A | G | 228 | Fuca1 | intronic | . | . | 576 | 4qD3 | rs32094834 | 1 | 0 |
| chr4 | 135922749 | G | A | 112 | Fuca1 | intronic | . | . | . | 4qD3 | rs50247104 | 1 | 0 |
| chr4 | 135922817 | C | T | 173 | Fuca1 | intronic | . | . | . | 4qD3 | rs27620156 | 1 | 0 |
| chr4 | 135925702 | G | A | 228 | Fuca1 | intronic | . | . | . | 4qD3 | rs32721376 | 1 | 0 |
| chr4 | 135925759 | A | T | 228 | Fuca1 | intronic | . | . | . | 4qD3 | rs27620140 | 1 | 0 |
| chr4 | 135925890 | G | A | 94 | Fuca1 | intronic | . | . | . | 4qD3 | rs31966004 | 1 | 0 |
| chr4 | 135925916 | C | T | 108 | Fuca1 | intronic | . | . | . | 4qD3 | rs27620139 | 1 | 0 |
| chr4 | 135930072 | A | G | 228 | Fuca1 | intronic | . | . | . | 4qD3 | rs32055622 | 1 | 0 |
| chr4 | 135933025 | A | G | 228 | Fuca1 | exonic | missense SNV | Fuca1:NM_024243:exon5:c.A853G:p.M285V | . | 4qD3 | rs27620132 | 1 | 0 |
| chr4 | 135933036 | A | G | 228 | Fuca1 | exonic | synonymous SNV | Fuca1:NM_024243:exon5:c.A864G:p.A288A | . | 4qD3 | rs32189717 | 1 | 0 |
| chr4 | 135934609 | C | T | 176 | Fuca1 | intronic | . | . | . | 4qD3 | rs51132188 | 1 | 0 |
| chr4 | 135934658 | A | G | 228 | Fuca1 | intronic | . | . | . | 4qD3 | rs27620121 | 1 | 0 |
| chr4 | 135934755 | T | C | 228 | Fuca1 | exonic | synonymous SNV | Fuca1:NM_024243:exon6:c.T993C:p.G331G | . | 4qD3 | rs27620120 | 1 | 0 |
| chr4 | 135934864 | A | G | 228 | Fuca1 | exonic | missense SNV | Fuca1:NM_024243:exon6:c.A1102G:p.N368D | . | 4qD3 | rs13464433 | 1 | 0 |
| chr4 | 135934947 | T | C | 228 | Fuca1 | intronic | . | . | . | 4qD3 | rs27620116 | 1 | 0 |
| chr4 | 135936684 | A | G | 86 | Fuca1 | intronic | . | . | . | 4qD3 | rs27620108 | 1 | 0 |
| chr4 | 135936930 | A | T | 228 | Fuca1 | exonic | missense SNV | Fuca1:NM_024243:exon7:c.A1126T:p.T376S | . | 4qD3 | rs27620106 | 1 | 0 |
| chr4 | 135938967 | A | G | 187 | Fuca1 | intronic | . | . | . | 4qD3 | rs27620102 | 1 | 0 |
| chr4 | 135939241 | T | C | 228 | Fuca1 | UTR3 | . | . | . | 4qD3 | rs13464432 | 1 | 0 |
| chr4 | 135950488 | A | C | 228 | Hmgcl | intronic | . | . | . | 4qD3 | rs27620076 | 1 | 0 |
| chr4 | 135950615 | T | C | 228 | Hmgcl | exonic | synonymous SNV | Hmgcl:NM_008254:exon2:c.T126C:p.D42D | . | 4qD3 | rs240224503 | 1 | 0 |
| chr4 | 135952296 | C | T | 228 | Hmgcl | intronic | . | . | . | 4qD3 | rs246412662 | 1 | 0 |
| chr4 | 135952475 | A | G | 228 | Hmgcl | exonic | synonymous SNV | Hmgcl:NM_008254:exon3:c.A249G:p.P83P | . | 4qD3 | rs13468076 | 1 | 0 |
| chr4 | 135952505 | C | G | 228 | Hmgcl | intronic | . | . | . | 4qD3 | rs50251235 | 1 | 0 |
| chr4 | 135953821 | T | C | 228 | Hmgcl | intronic | . | . | . | 4qD3 | rs27620067 | 1 | 0 |
| chr4 | 135953901 | C | T | 228 | Hmgcl | exonic | synonymous SNV | Hmgcl:NM_008254:exon4:c.C297T:p.P99P | . | 4qD3 | rs27620066 | 1 | 0 |
| chr4 | 135953975 | C | T | 228 | Hmgcl | intronic | . | . | . | 4qD3 | rs27620065 | 1 | 0 |
| chr4 | 135954033 | A | G | 212 | Hmgcl | intronic | . | . | . | 4qD3 | rs27620064 | 1 | 0 |
| chr4 | 135955758 | A | G | 228 | Hmgcl | intronic | . | . | . | 4qD3 | rs27620055 | 1 | 0 |
| chr4 | 135958741 | C | G | 228 | Hmgcl | exonic | synonymous SNV | Hmgcl:NM_008254:exon6:c.C516G:p.L172L | . | 4qD3 | rs248693426 | 1 | 0 |
| chr4 | 135959912 | A | T | 228 | Hmgcl | intronic | . | . | . | 4qD3 | rs3703182 | 1 | 0 |
| chr4 | 135960036 | C | T | 228 | Hmgcl | exonic | synonymous SNV | Hmgcl:NM_008254:exon7:c.C621T:p.G207G | . | 4qD3 | rs233247926 | 1 | 0 |
| chr4 | 135960042 | T | C | 228 | Hmgcl | exonic | synonymous SNV | Hmgcl:NM_008254:exon7:c.T627C:p.G209G | . | 4qD3 | rs13468077 | 1 | 0 |
| chr4 | 135960181 | C | T | 228 | Hmgcl | intronic | . | . | . | 4qD3 | rs27620043 | 1 | 0 |
| chr4 | 135960252 | A | G | 228 | Hmgcl | intronic | . | . | . | 4qD3 | rs236744352 | 1 | 0 |
| chr4 | 135960985 | C | G | 228 | Hmgcl | intronic | . | . | . | 4qD3 | rs27620041 | 1 | 0 |
| chr4 | 135961002 | A | G | 228 | Hmgcl | intronic | . | . | . | 4qD3 | rs27620040 | 1 | 0 |
| chr4 | 135962051 | C | T | 228 | Hmgcl | intronic | . | . | . | 4qD3 | rs260739236 | 1 | 0 |
| chr4 | 135962072 | G | A | 228 | Hmgcl | intronic | . | . | . | 4qD3 | rs234047812 | 1 | 0 |
| chr4 | 135962216 | T | A | 228 | Hmgcl | UTR3 | . | . | . | 4qD3 | rs257561788 | 1 | 0 |
| chr4 | 135962217 | C | T | 228 | Hmgcl | UTR3 | . | . | . | 4qD3 | rs217140081 | 1 | 0 |
| chr4 | 135962349 | T | C | 157 | Hmgcl | UTR3 | . | . | . | 4qD3 | rs31784303 | 1 | 0 |
| chr4 | 135962416 | A | G | 93 | Hmgcl | UTR3 | . | . | . | 4qD3 | rs32646421 | 1 | 0 |
| chr4 | 135965419 | A | G | 228 | Gale | exonic | synonymous SNV | Gale:NM_178389:exon1:c.A81G:p.S27S | . | 4qD3 | rs47255479 | 1 | 0 |
| chr4 | 135965494 | A | G | 228 | Gale | intronic | . | . | . | 4qD3 | rs45880359 | 1 | 0 |
| chr4 | 135965606 | G | A | 228 | Gale | exonic | synonymous SNV | Gale:NM_178389:exon2:c.G159A:p.E53E | . | 4qD3 | rs251928459 | 1 | 0 |
| chr4 | 135966395 | A | G | 228 | Gale | exonic | missense SNV | Gale:NM_178389:exon4:c.A374G:p.N125S | . | 4qD3 | rs32486047 | 1 | 0 |
| chr4 | 135966763 | G | A | 228 | Gale | intronic | . | . | . | 4qD3 | rs32984153 | 1 | 0 |
| chr4 | 135967129 | C | T | 73 | Gale | intronic | . | . | . | 4qD3 | rs45829653 | 1 | 0 |
| chr4 | 135967164 | C | T | 228 | Gale | intronic | . | . | . | 4qD3 | rs47351133 | 1 | 0 |

| | | | | | | | | | | | | | |
|---|---|---|---|---|---|---|---|---|---|---|---|---|---|
| chr4 | 135967279 | G | T | 228 | Gale | intronic | . | . | . | 4qD3 | rs231607449 | 1 | 0 |
| chr4 | 135967797 | T | A | 228 | Gale | splicing | . | . | . | 4qD3 | rs32230916 | 1 | 0 |
| chr4 | 135968005 | A | G | 169 | Gale | UTR3 | . | . | . | 4qD3 | rs255742725 | 1 | 0 |
| chr4 | 135969237 | T | C | 228 | Lypla2 | exonic | synonymous SNV | Lypla2:NM_011942:exon9:c.A552G:p.G184G | . | 4qD3 | rs13459078 | 1 | 0 |
| chr4 | 135969555 | G | A | 228 | Lypla2 | intronic | . | . | . | 4qD3 | rs251480402 | 1 | 0 |
| chr4 | 135969569 | C | A | 228 | Lypla2 | intronic | . | . | . | 4qD3 | rs32485795 | 1 | 0 |
| chr4 | 135969760 | T | G | 228 | Lypla2 | intronic | . | . | . | 4qD3 | rs31772528 | 1 | 0 |
| chr4 | 135970092 | A | G | 228 | Lypla2 | intronic | . | . | . | 4qD3 | rs254686715 | 1 | 0 |
| chr4 | 135970093 | A | C | 228 | Lypla2 | intronic | . | . | . | 4qD3 | rs234221357 | 1 | 0 |
| chr4 | 135970321 | T | C | 190 | Lypla2 | intronic | . | . | . | 4qD3 | rs48556354 | 1 | 0 |
| chr4 | 135970529 | G | A | 228 | Lypla2 | intronic | . | . | . | 4qD3 | rs50987966 | 1 | 0 |
| chr4 | 135970540 | C | A | 228 | Lypla2 | intronic | . | . | . | 4qD3 | rs47826782 | 1 | 0 |
| chr4 | 135970889 | C | G | 228 | Lypla2 | exonic | missense SNV | Lypla2:NM_011942:exon2:c.G49C:p.V17L | . | 4qD3 | rs49708194 | 1 | 0 |
| chr4 | 135970986 | G | A | 203 | Lypla2 | intronic | . | . | . | 4qD3 | rs48049199 | 1 | 0 |
| chr4 | 135970992 | T | C | 174 | Lypla2 | intronic | . | . | . | 4qD3 | rs48609525 | 1 | 0 |
| chr4 | 135976591 | T | C | 228 | Pithd1 | intronic | . | . | . | 4qD3 | rs259614595 | 1 | 0 |
| chr4 | 135976674 | C | T | 160 | Pithd1 | intronic | . | . | . | 4qD3 | rs219347570 | 1 | 0 |
| chr4 | 135977103 | T | C | 228 | Pithd1 | intronic | . | . | . | 4qD3 | rs32857576 | 1 | 0 |
| chr4 | 135977110 | G | A | 228 | Pithd1 | intronic | . | . | . | 4qD3 | rs32217471 | 1 | 0 |
| chr4 | 135978751 | C | T | 228 | Pithd1 | intronic | . | . | . | 4qD3 | rs51321691 | 1 | 0 |
| chr4 | 135985900 | T | C | 228 | Pithd1 | intronic | . | . | . | 4qD3 | rs48422745 | 1 | 0 |
| chr4 | 135986297 | T | A | 228 | Pithd1 | intronic | . | . | . | 4qD3 | rs50022892 | 1 | 0 |
| chr4 | 135986333 | C | T | 228 | Pithd1 | intronic | . | . | . | 4qD3 | rs50724933 | 1 | 0 |
| chr4 | 135986374 | A | C | 228 | Pithd1 | intronic | . | . | . | 4qD3 | rs31959910 | 1 | 0 |
| chr4 | 135986599 | T | C | 210 | Pithd1 | intronic | . | . | . | 4qD3 | rs47478499 | 1 | 0 |
| chr4 | 136006133 | T | C | 228 | Tceb3 | intronic | . | . | . | 4qD3 | rs27619975 | 1 | 0 |
| chr4 | 136006419 | T | C | 228 | Tceb3 | intronic | . | . | . | 4qD3 | rs27619974 | 1 | 0 |
| chr4 | 136008844 | C | T | 160 | Tceb3 | intronic | . | . | . | 4qD3 | rs3700836 | 1 | 0 |
| chr4 | 136008918 | G | A | 184 | Tceb3 | intronic | . | . | . | 4qD3 | rs3701412 | 1 | 0 |
| chr4 | 136009020 | G | A | 228 | Tceb3 | intronic | . | . | . | 4qD3 | rs3702002 | 1 | 0 |
| chr4 | 136009224 | G | C | 228 | Tceb3 | intronic | . | . | . | 4qD3 | rs27619968 | 1 | 0 |
| chr4 | 136010178 | C | A | 227 | Tceb3 | intronic | . | . | . | 4qD3 | rs230997753 | 1 | 0 |
| chr4 | 136010179 | A | G | 222 | Tceb3 | intronic | . | . | . | 4qD3 | rs27604664 | 1 | 0 |
| chr4 | 136010898 | T | C | 228 | Tceb3 | exonic | synonymous SNV | Tceb3:NM_013736:exon4:c.A750G:p.A250A | . | 4qD3 | rs27604660 | 1 | 0 |
| chr4 | 136010967 | A | C | 228 | Tceb3 | exonic | synonymous SNV | Tceb3:NM_013736:exon4:c.T681G:p.G227G | . | 4qD3 | rs236567934 | 1 | 0 |
| chr4 | 136012857 | G | A | 228 | Tceb3 | intronic | . | . | . | 4qD3 | rs27604657 | 1 | 0 |
| chr4 | 136013058 | T | G | 198 | Tceb3 | intronic | . | . | . | 4qD3 | rs27604656 | 1 | 0 |
| chr4 | 136014331 | C | T | 101 | Tceb3 | intronic | . | . | . | 4qD3 | rs27604652 | 1 | 0 |
| chr4 | 136014354 | G | A | 161 | Tceb3 | intronic | . | . | . | 4qD3 | rs27604650 | 1 | 0 |
| chr4 | 136014429 | T | C | 198 | Tceb3 | exonic | missense SNV | Tceb3:NM_013736:exon2:c.A103G:p.I35V | . | 4qD3 | rs27604649 | 1 | 0 |
| chr4 | 136014530 | G | A | 101 | Tceb3 | intronic | . | . | . | 4qD3 | rs27604648 | 1 | 0 |
| chr4 | 136049840 | C | A | 165 | . | downstream | . | . | . | 4qD3 | rs27604539 | 1 | 0 |
| chr4 | 136049970 | A | G | 228 | Rpl11 | UTR3 | . | . | . | 4qD3 | rs32006736 | 1 | 0 |
| chr4 | 136050014 | G | A | 228 | Rpl11 | exonic | synonymous SNV | Rpl11:NM_025919:exon6:c.C510T:p.Y170Y | . | 4qD3 | rs32144911 | 1 | 0 |
| chr4 | 136050271 | C | T | 228 | Rpl11 | intronic | . | . | . | 4qD3 | rs231162279 | 1 | 0 |
| chr4 | 136050577 | T | C | 114 | Rpl11 | intronic | . | . | . | 4qD3 | rs32815327 | 1 | 0 |
| chr4 | 136051105 | T | C | 228 | Rpl11 | intronic | . | . | . | 4qD3 | rs27604538 | 1 | 0 |
| chr4 | 136051116 | C | T | 228 | Rpl11 | intronic | . | . | . | 4qD3 | rs32564648 | 1 | 0 |
| chr4 | 136051128 | C | A | 228 | Rpl11 | intronic | . | . | . | 4qD3 | rs31864946 | 1 | 0 |
| chr4 | 136051267 | C | T | 228 | Rpl11 | exonic | synonymous SNV | Rpl11:NM_025919:exon4:c.G300A:p.S100S | . | 4qD3 | rs32415095 | 1 | 0 |
| chr4 | 136051354 | C | T | 228 | Rpl11 | intronic | . | . | . | 4qD3 | rs32876929 | 1 | 0 |
| chr4 | 136051398 | A | T | 228 | Rpl11 | intronic | . | . | . | 4qD3 | rs47028952 | 1 | 0 |
| chr4 | 136051570 | G | A | 228 | Rpl11 | intronic | . | . | . | 4qD3 | rs32871627 | 1 | 0 |
| chr4 | 136051751 | T | C | 228 | Rpl11 | intronic | . | . | . | 4qD3 | rs31997508 | 1 | 0 |
| chr4 | 136051828 | T | C | 228 | Rpl11 | intronic | . | . | . | 4qD3 | rs32134899 | 1 | 0 |
| chr4 | 136052484 | T | G | 202 | Rpl11 | intronic | . | . | . | 4qD3 | rs32283533 | 1 | 0 |
| chr4 | 136052548 | T | C | 228 | Rpl11 | intronic | . | . | . | 4qD3 | rs31918617 | 1 | 0 |
| chr4 | 136053201 | A | G | 228 | Rpl11 | intronic | . | . | 730 | 4qD3 | rs31883402 | 1 | 0 |
| chr4 | 136053209 | C | A | 228 | Rpl11 | intronic | . | . | 730 | 4qD3 | rs32962669 | 1 | 0 |
| chr4 | 136053519 | T | C | 196 | . | upstream | . | . | . | 4qD3 | rs27604537 | 1 | 0 |
| chr4 | 136143841 | G | T | 228 | Id3 | UTR5 | . | . | 1313 | 4qD3 | rs32138579 | 1 | 0 |
| chr4 | 136144512 | A | G | 143 | Id3 | intronic | . | . | 1313 | 4qD3 | rs211766504 | 1 | 0 |
| chr4 | 136173019 | C | T | 228 | E2f2 | exonic | missense SNV | E2f2:NM_177733:exon1:c.C239T:p.A80V | 952 | 4qD3 | rs47906384 | 1 | 0 |
| chr4 | 136178553 | T | C | 228 | E2f2 | intronic | . | . | . | 4qD3 | rs27580014 | 1 | 0 |
| chr4 | 136178608 | C | T | 228 | E2f2 | intronic | . | . | . | 4qD3 | rs3705385 | 1 | 0 |
| chr4 | 136180548 | C | G | 228 | E2f2 | exonic | synonymous SNV | E2f2:NM_177733:exon3:c.C555G:p.R185R | . | 4qD3 | rs27580000 | 1 | 0 |
| chr4 | 136180716 | G | T | 130 | E2f2 | intronic | . | . | . | 4qD3 | rs48853546 | 1 | 0 |
| chr4 | 136180719 | G | T | 130 | E2f2 | intronic | . | . | . | 4qD3 | rs47619532 | 1 | 0 |
| chr4 | 136181198 | T | G | 228 | E2f2 | intronic | . | . | . | 4qD3 | rs27579997 | 1 | 0 |
| chr4 | 136181367 | C | T | 228 | E2f2 | exonic | synonymous SNV | E2f2:NM_001305399:exon2:c.C9T:p.A3A,E2f2:NM_177733:exon4:c.C666T:p.A222A | . | 4qD3 | rs27579996 | 1 | 0 |
| chr4 | 136184489 | G | A | 228 | E2f2 | exonic | synonymous SNV | E2f2:NM_001305399:exon3:c.G153A:p.A51A,E2f2:NM_177733:exon5:c.G810A:p.A270A | . | 4qD3 | rs27579987 | 1 | 0 |
| chr4 | 136184638 | A | G | 212 | E2f2 | intronic | . | . | . | 4qD3 | rs27579986 | 1 | 0 |
| chr4 | 136186927 | T | C | 228 | E2f2 | exonic | synonymous SNV | E2f2:NM_001305399:exon4:c.T330C:p.I110I,E2f2:NM_177733:exon6:c.T987C:p.I329I | . | 4qD3 | rs263071042 | 1 | 0 |
| chr4 | 136186945 | G | C | 228 | E2f2 | exonic | synonymous SNV | E2f2:NM_001305399:exon4:c.G348C:p.P116P,E2f2:NM_177733:exon6:c.G1005C:p.P335P | . | 4qD3 | rs232776415 | 1 | 0 |
| chr4 | 136187112 | G | T | 125 | E2f2 | intronic | . | . | . | 4qD3 | rs264507423 | 1 | 0 |
| chr4 | 136192671 | A | G | 104 | E2f2 | intronic | . | . | . | 4qD3 | rs48727188 | 1 | 0 |
| chr4 | 136193126 | G | A | 203 | E2f2 | UTR3 | . | . | . | 4qD3 | rs214518304 | 1 | 0 |
| chr4 | 136227452 | T | C | 228 | Asap3 | exonic | synonymous SNV | Asap3:NM_001008232:exon2:c.T174C:p.A58A | . | 4qD3 | rs27579830 | 1 | 0 |
| chr4 | 136227521 | A | G | 228 | Asap3 | intronic | . | . | . | 4qD3 | rs51154743 | 1 | 0 |
| chr4 | 136227535 | C | T | 228 | Asap3 | intronic | . | . | . | 4qD3 | rs261966125 | 1 | 0 |
| chr4 | 136227725 | G | A | 228 | Asap3 | intronic | . | . | . | 4qD3 | rs27579829 | 1 | 0 |
| chr4 | 136228367 | T | C | 228 | Asap3 | intronic | . | . | . | 4qD3 | rs49000087 | 1 | 0 |
| chr4 | 136228368 | C | T | 228 | Asap3 | intronic | . | . | . | 4qD3 | rs48901823 | 1 | 0 |
| chr4 | 136228513 | T | C | 228 | Asap3 | intronic | . | . | . | 4qD3 | rs215003547 | 1 | 0 |
| chr4 | 136229243 | A | G | 169 | Asap3 | intronic | . | . | . | 4qD3 | rs47086668 | 1 | 0 |
| chr4 | 136229340 | T | A | 228 | Asap3 | intronic | . | . | . | 4qD3 | rs27579812 | 1 | 0 |
| chr4 | 136229465 | C | T | 228 | Asap3 | intronic | . | . | . | 4qD3 | rs27579809 | 1 | 0 |
| chr4 | 136229571 | G | A | 228 | Asap3 | intronic | . | . | . | 4qD3 | rs216686002 | 1 | 0 |
| chr4 | 136229604 | A | C | 196 | Asap3 | intronic | . | . | . | 4qD3 | rs27579808 | 1 | 0 |
| chr4 | 136229691 | G | T | 106 | Asap3 | intronic | . | . | . | 4qD3 | rs47644749 | 1 | 0 |
| chr4 | 136233426 | A | G | 228 | Asap3 | intronic | . | . | . | 4qD3 | rs32947620 | 1 | 0 |
| chr4 | 136233474 | T | C | 228 | Asap3 | intronic | . | . | . | 4qD3 | rs27579792 | 1 | 0 |
| chr4 | 136233696 | G | A | 228 | Asap3 | intronic | . | . | . | 4qD3 | rs32368142 | 1 | 0 |
| chr4 | 136234219 | C | G | 209 | Asap3 | intronic | . | . | . | 4qD3 | rs27579788 | 1 | 0 |
| chr4 | 136234220 | A | G | 30.9185 | Asap3 | intronic | . | . | . | 4qD3 | rs27579787 | 0 | 1 |
| chr4 | 136234243 | C | G | 228 | Asap3 | intronic | . | . | . | 4qD3 | rs27579786 | 1 | 0 |
| chr4 | 136234421 | T | C | 228 | Asap3 | intronic | . | . | . | 4qD3 | rs27579785 | 1 | 0 |
| chr4 | 136234798 | C | T | 157 | Asap3 | intronic | . | . | . | 4qD3 | rs46629275 | 1 | 0 |
| chr4 | 136234818 | G | A | 135 | Asap3 | intronic | . | . | . | 4qD3 | rs223829069 | 1 | 0 |
| chr4 | 136236196 | C | T | 169 | Asap3 | intronic | . | . | . | 4qD3 | rs27579772 | 1 | 0 |
| chr4 | 136236343 | G | A | 228 | Asap3 | intronic | . | . | . | 4qD3 | rs27579771 | 1 | 0 |
| chr4 | 136236402 | G | A | 228 | Asap3 | exonic | synonymous SNV | Asap3:NM_001008232:exon11:c.G972A:p.K324K | . | 4qD3 | rs27579770 | 1 | 0 |
| chr4 | 136236469 | A | C | 228 | Asap3 | intronic | . | . | . | 4qD3 | rs32279781 | 1 | 0 |
| chr4 | 136236653 | C | T | 228 | Asap3 | intronic | . | . | . | 4qD3 | rs107698953 | 1 | 0 |
| chr4 | 136236665 | G | A | 228 | Asap3 | intronic | . | . | . | 4qD3 | rs47276409 | 1 | 0 |
| chr4 | 136236833 | G | A | 228 | Asap3 | intronic | . | . | . | 4qD3 | rs48137678 | 1 | 0 |
| chr4 | 136236842 | G | A | 228 | Asap3 | intronic | . | . | . | 4qD3 | rs49749876 | 1 | 0 |
| chr4 | 136238048 | T | C | 161 | Asap3 | intronic | . | . | . | 4qD3 | rs47779738 | 1 | 0 |
| chr4 | 136238138 | C | A | 228 | Asap3 | intronic | . | . | . | 4qD3 | rs27638041 | 1 | 0 |
| chr4 | 136238171 | G | A | 228 | Asap3 | intronic | . | . | . | 4qD3 | rs27638039 | 1 | 0 |
| chr4 | 136238235 | G | A | 228 | Asap3 | intronic | . | . | . | 4qD3 | rs27638038 | 1 | 0 |
| chr4 | 136238496 | G | A | 228 | Asap3 | exonic | missense SNV | Asap3:NM_001008232:exon14:c.G1316A:p.R439H | . | 4qD3 | rs27638036 | 1 | 0 |
| chr4 | 136238672 | C | T | 228 | Asap3 | exonic | synonymous SNV | Asap3:NM_001008232:exon15:c.C1413T:p.G471G | . | 4qD3 | rs27638035 | 1 | 0 |
| chr4 | 136238824 | A | T | 228 | Asap3 | intronic | . | . | . | 4qD3 | rs27638033 | 1 | 0 |
| chr4 | 136238867 | C | T | 228 | Asap3 | intronic | . | . | . | 4qD3 | rs27638032 | 1 | 0 |
| chr4 | 136239006 | T | G | 228 | Asap3 | intronic | . | . | . | 4qD3 | rs50966870 | 1 | 0 |
| chr4 | 136239007 | T | C | 228 | Asap3 | intronic | . | . | . | 4qD3 | rs27638031 | 1 | 0 |
| chr4 | 136239070 | G | A | 228 | Asap3 | intronic | . | . | . | 4qD3 | rs27638030 | 1 | 0 |
| chr4 | 136239161 | A | C | 228 | Asap3 | intronic | . | . | . | 4qD3 | rs27638029 | 1 | 0 |
| chr4 | 136240285 | C | A | 225 | Asap3 | intronic | . | . | . | 4qD3 | rs52297045 | 1 | 0 |
| chr4 | 136240500 | G | A | 100 | Asap3 | intronic | . | . | . | 4qD3 | rs27638018 | 1 | 0 |
| chr4 | 136241049 | A | G | 228 | Asap3 | exonic | synonymous SNV | Asap3:NM_001008232:exon20:c.A1992G:p.R664R | . | 4qD3 | rs27638014 | 1 | 0 |
| chr4 | 136241416 | T | G | 228 | Asap3 | exonic | missense SNV | Asap3:NM_001008232:exon22:c.T2171G:p.V724G | . | 4qD3 | rs32650437 | 1 | 0 |
| chr4 | 136241462 | G | A | 228 | Asap3 | exonic | synonymous SNV | Asap3:NM_001008232:exon22:c.G2217A:p.P739P | . | 4qD3 | rs32135376 | 1 | 0 |
| chr4 | 136241475 | A | G | 228 | Asap3 | exonic | missense SNV | Asap3:NM_001008232:exon22:c.A2230G:p.T744A | . | 4qD3 | rs27638012 | 1 | 0 |
| chr4 | 136241581 | T | G | 228 | Asap3 | intronic | . | . | . | 4qD3 | rs27638011 | 1 | 0 |
| chr4 | 136242687 | A | G | 202 | Asap3 | exonic | synonymous SNV | Asap3:NM_001008232:exon23:c.A2355G:p.E785E | . | 4qD3 | rs27637998 | 1 | 0 |
| chr4 | 136243496 | C | T | 228 | Asap3 | exonic | synonymous SNV | Asap3:NM_001008232:exon24:c.C2598T:p.D866D | . | 4qD3 | rs27637988 | 1 | 0 |
| chr4 | 136243619 | G | A | 228 | Asap3 | intronic | . | . | . | 4qD3 | rs252547425 | 1 | 0 |
| chr4 | 136243763 | T | C | 228 | Asap3 | intronic | . | . | . | 4qD3 | rs27637987 | 1 | 0 |
| chr4 | 136243774 | T | C | 228 | Asap3 | intronic | . | . | . | 4qD3 | rs27637986 | 1 | 0 |
| chr4 | 136248261 | G | T | 65 | Tcea3 | intronic | . | . | 785 | 4qD3 | rs51068217 | 1 | 0 |
| chr4 | 136253580 | C | T | 172 | Tcea3 | intronic | . | . | . | 4qD3 | rs32142636 | 1 | 0 |
| chr4 | 136253881 | C | T | 228 | Tcea3 | intronic | . | . | . | 4qD3 | rs32472588 | 1 | 0 |
| chr4 | 136254001 | G | C | 101 | Tcea3 | intronic | . | . | . | 4qD3 | rs32278082 | 1 | 0 |
| chr4 | 136254247 | C | T | 126 | Tcea3 | intronic | . | . | . | 4qD3 | rs32797786 | 1 | 0 |
| chr4 | 136254396 | A | G | 228 | Tcea3 | intronic | . | . | . | 4qD3 | rs27623248 | 1 | 0 |
| chr4 | 136257892 | G | A | 228 | Tcea3 | intronic | . | . | . | 4qD3 | rs31954564 | 1 | 0 |
| chr4 | 136264390 | G | C | 139 | Tcea3 | intronic | . | . | . | 4qD3 | rs250469526 | 1 | 0 |
| chr4 | 136267901 | G | C | 171 | Tcea3 | intronic | . | . | . | 4qD3 | rs45670946 | 1 | 0 |
| chr4 | 136267978 | G | A | 228 | Tcea3 | intronic | . | . | . | 4qD3 | rs237782330 | 1 | 0 |
| chr4 | 136273612 | A | G | 228 | Tcea3 | intronic | . | . | . | 4qD3 | rs51782360 | 1 | 0 |
| chr4 | 136273661 | T | C | 228 | Tcea3 | exonic | synonymous SNV | Tcea3:NM_011542:exon10:c.T981C:p.A327A | . | 4qD3 | rs27623217 | 1 | 0 |
| chr4 | 136273795 | A | G | 228 | Tcea3 | intronic | . | . | . | 4qD3 | rs49498288 | 1 | 0 |
| chr4 | 136273817 | A | G | 198 | Tcea3 | intronic | . | . | . | 4qD3 | rs51135414 | 1 | 0 |
| chr4 | 136274802 | A | C | 228 | Tcea3 | UTR3 | . | . | . | 4qD3 | rs50085716 | 1 | 0 |
| chr4 | 136275004 | A | G | 156 | . | downstream | . | . | . | 4qD3 | rs46806065 | 1 | 0 |
| chr4 | 136275007 | G | A | 156 | . | downstream | . | . | . | 4qD3 | rs49068150 | 1 | 0 |
| chr4 | 136287001 | C | G | 134 | Zfp46 | intronic | . | . | . | 4qD3 | rs27623134 | 1 | 0 |
| chr4 | 136287322 | G | T | 228 | Zfp46 | intronic | . | . | . | 4qD3 | rs27623133 | 1 | 0 |
| chr4 | 136287421 | C | T | 175 | Zfp46 | intronic | . | . | . | 4qD3 | rs27623130 | 1 | 0 |
| chr4 | 136289801 | C | G | 59 | Zfp46 | intronic | . | . | . | 4qD3 | . | 1 | 0 |
| chr4 | 136289892 | C | G | 228 | Zfp46 | intronic | . | . | . | 4qD3 | rs27623121 | 1 | 0 |
| chr4 | 136290171 | T | C | 228 | Zfp46 | exonic | synonymous SNV | Zfp46:NM_009557:exon3:c.T315C:p.T105T | . | 4qD3 | rs33031808 | 1 | 0 |
| chr4 | 136290678 | C | T | 105 | Zfp46 | exonic | synonymous SNV | Zfp46:NM_009557:exon3:c.C822T:p.D274D | 799 | 4qD3 | rs3718632 | 1 | 0 |
| chr4 | 136311078 | A | C | 152 | Hnrnpr | UTR5 | . | . | 1268 | 4qD3 | rs13474532 | 1 | 0 |
| chr4 | 136313938 | G | A | 157 | Hnrnpr | intronic | . | . | . | 4qD3 | rs6374866 | 1 | 0 |
| chr4 | 136313985 | T | G | 200 | Hnrnpr | intronic | . | . | . | 4qD3 | rs6375297 | 1 | 0 |
| chr4 | 136314415 | G | A | 127 | Hnrnpr | intronic | . | . | . | 4qD3 | rs27623076 | 1 | 0 |
| chr4 | 136316163 | G | A | 110 | Hnrnpr | intronic | . | . | . | 4qD3 | rs31833763 | 1 | 0 |
| chr4 | 136316331 | G | A | 228 | Hnrnpr | intronic | . | . | . | 4qD3 | rs263812934 | 1 | 0 |
| chr4 | 136316695 | T | A | 180 | Hnrnpr | intronic | . | . | . | 4qD3 | rs27623068 | 1 | 0 |
| chr4 | 136316982 | C | T | 171 | Hnrnpr | intronic | . | . | . | 4qD3 | rs27623065 | 1 | 0 |
| chr4 | 136319265 | T | C | 183 | Hnrnpr | intronic | . | . | . | 4qD3 | rs27623060 | 1 | 0 |
| chr4 | 136319435 | G | A | 228 | Hnrnpr | exonic | synonymous SNV | Hnrnpr:NM_001277122:exon4:c.G123A:p.T41T,Hnrnpr:NM_001277123:exon4:c.G123A:p.T41T,Hnrnpr:NM_( | . | 4qD3 | rs27623059 | 1 | 0 |
| chr4 | 136319517 | T | C | 228 | Hnrnpr | intronic | . | . | . | 4qD3 | rs27623058 | 1 | 0 |
| chr4 | 136319675 | T | A | 167 | Hnrnpr | intronic | . | . | . | 4qD3 | rs27623057 | 1 | 0 |
| chr4 | 136327487 | G | A | 228 | Hnrnpr | intronic | . | . | . | 4qD3 | rs27623039 | 1 | 0 |
| chr4 | 136327510 | A | G | 228 | Hnrnpr | intronic | . | . | . | 4qD3 | rs27623038 | 1 | 0 |
| chr4 | 136329674 | A | C | 167 | Hnrnpr | intronic | . | . | . | 4qD3 | rs27623030 | 1 | 0 |
| chr4 | 136332693 | T | C | 167 | Hnrnpr | intronic | . | . | . | 4qD3 | rs27623019 | 1 | 0 |
| chr4 | 136336107 | C | T | 158 | Hnrnpr | intronic | . | . | . | 4qD3 | rs27623000 | 1 | 0 |
| chr4 | 136463003 | G | T | 228 | . | intergenic | . | . | . | 4qD3 | . | 1 | 0 |
| chr4 | 136631153 | C | A | 228 | Lactbl1 | intronic | . | . | . | 4qD3 | . | 1 | 0 |
| chr4 | 136673088 | C | A | 162 | Ephb2 | intronic | . | . | . | 4qD3 | . | 1 | 0 |
| chr4 | 136999496 | A | G | 159 | Zbtb40 | exonic | synonymous SNV | Zbtb40:NM_198248:exon8:c.T1528C:p.L510L | . | 4qD3 | . | 0 | 1 |
| chr4 | 137455020 | C | T | 197 | 1700013G24Rik | exonic | missense SNV | 1700013G24Rik:NM_027063:exon2:c.C485T:p.A162V | . | 4qD3 | . | 0 | 1 |
| chr4 | 137548336 | C | T | 117 | Hspg2 | intronic | . | . | . | 4qD3 | . | 0 | 1 |
| chr4 | 137569264 | G | T | 228 | Hspg2 | exonic | missense SNV | Hspg2:NM_008305:exon96:c.G12879T:p.E4293D | . | 4qD3 | . | 1 | 0 |
| chr4 | 137610631 | C | T | 178 | Usp48 | intronic | . | . | . | 4qD3 | . | 0 | 1 |
| chr4 | 137913708 | C | T | 228 | Ece1 | intronic | . | . | 489 | 4qD3 | . | 1 | 0 |
| chr4 | 138240689 | A | G | 221 | Hp1bp3 | exonic | missense SNV | Hp1bp3:NM_001122896:exon11:c.A1208G:p.D403G,Hp1bp3:NM_001285480:exon11:c.A1169G:p.D390G,Hp1bı | . | 4qD3 | . | 0 | 1 |
| chr4 | 138581597 | G | A | 222 | Vwa5b1 | exonic | stopgain | Vwa5b1:NM_029401:exon15:c.C2281T:p.Q761X | . | 4qD3 | . | 0 | 1 |
| chr4 | 138612858 | C | A | 228 | Vwa5b1 | exonic | synonymous SNV | Vwa5b1:NM_029401:exon2:c.G87T:p.L29L | . | 4qD3 | . | 1 | 0 |
| chr4 | 138967803 | T | G | 228 | Rnf186 | exonic | missense SNV | Rnf186:NM_025786:exon1:c.T653G:p.V218G | . | 4qD3 | . | 1 | 0 |

| | | | | | | | | | | | | | |
|---|---|---|---|---|---|---|---|---|---|---|---|---|---|
| chr4 | 139058625 | T | C | 95 | Tmco4 | UTR3 | . | . | . | 4qD3 | rs46813052 | 1 | 0 |
| chr4 | 139359178 | C | T | 88 | Emc1 | intronic | . | . | . | 4qD3 | . | 0 | 1 |
| chr4 | 139365313 | G | A | 228 | Emc1 | exonic | synonymous SNV | Emc1:NM_001039200:exon14:c.G1554A:p.K518K,Emc1:NM_146157:exon15:c.G1563A:p.K521K | . | 4qD3 | rs13478016 | 1 | 0 |
| chr4 | 139365375 | C | T | 228 | Emc1 | exonic | missense SNV | Emc1:NM_001039200:exon14:c.C1616T:p.T539M,Emc1:NM_146157:exon15:c.C1625T:p.T542M | . | 4qD3 | rs27567931 | 1 | 0 |
| chr4 | 139365575 | G | A | 228 | Emc1 | exonic | missense SNV | Emc1:NM_001039200:exon15:c.G1693A:p.V565I,Emc1:NM_146157:exon16:c.G1702A:p.V568I | . | 4qD3 | . | 1 | 0 |
| chr4 | 139460296 | G | A | 92 | Ubr4 | exonic | missense SNV | Ubr4:NM_001160319:exon78:c.G11746A:p.A3916T | . | 4qD3 | . | 0 | 1 |
| chr4 | 139740570 | A | G | 110 | Pax7 | UTR3 | . | . | . | 4qD3 | rs245436644 | 1 | 0 |
| chr4 | 139740686 | T | C | 228 | Pax7 | UTR3 | . | . | . | 4qD3 | rs32719319 | 1 | 0 |
| chr4 | 139740708 | A | G | 228 | Pax7 | UTR3 | . | . | . | 4qD3 | rs48456913 | 1 | 0 |
| chr4 | 139748874 | C | T | 228 | Pax7 | intronic | . | . | . | 4qD3 | rs27642522 | 1 | 0 |
| chr4 | 139749219 | T | C | 228 | Pax7 | intronic | . | . | . | 4qD3 | rs27642521 | 1 | 0 |
| chr4 | 139779693 | C | T | 228 | Pax7 | exonic | synonymous SNV | Pax7:NM_011039:exon7:c.G1032A:p.A344A | . | 4qD3 | rs32812351 | 1 | 0 |
| chr4 | 139779774 | T | C | 228 | Pax7 | exonic | synonymous SNV | Pax7:NM_011039:exon7:c.A951G:p.G317G | . | 4qD3 | rs32281799 | 1 | 0 |
| chr4 | 139780721 | T | C | 228 | Pax7 | intronic | . | . | . | 4qD3 | rs27626222 | 1 | 0 |
| chr4 | 139780725 | G | A | 228 | Pax7 | intronic | . | . | . | 4qD3 | rs50004401 | 1 | 0 |
| chr4 | 139780894 | A | G | 228 | Pax7 | exonic | synonymous SNV | Pax7:NM_011039:exon6:c.T813C:p.R271R | . | 4qD3 | rs27626220 | 1 | 0 |
| chr4 | 139780980 | G | T | 192 | Pax7 | intronic | . | . | . | 4qD3 | rs48949224 | 1 | 0 |
| chr4 | 139784561 | G | A | 228 | Pax7 | exonic | synonymous SNV | Pax7:NM_011039:exon5:c.C702T:p.F234F | . | 4qD3 | rs27626205 | 1 | 0 |
| chr4 | 139784657 | A | G | 228 | Pax7 | exonic | synonymous SNV | Pax7:NM_011039:exon5:c.T606C:p.D202D | . | 4qD3 | . | 1 | 0 |
| chr4 | 139784717 | A | G | 228 | Pax7 | intronic | . | . | . | 4qD3 | rs108762220 | 1 | 0 |
| chr4 | 139828428 | A | T | 132 | Pax7 | intronic | . | . | . | 4qD3 | rs49295337 | 1 | 0 |
| chr4 | 139828790 | T | C | 228 | Pax7 | intronic | . | . | . | 4qD3 | rs48999014 | 1 | 0 |
| chr4 | 139828847 | T | C | 208 | Pax7 | intronic | . | . | . | 4qD3 | rs47960732 | 1 | 0 |
| chr4 | 139829642 | T | C | 228 | Pax7 | intronic | . | . | . | 4qD3 | rs229702859 | 1 | 0 |
| chr4 | 139829727 | T | C | 203 | Pax7 | intronic | . | . | . | 4qD3 | rs248630201 | 1 | 0 |
| chr4 | 139830081 | T | C | 228 | Pax7 | intronic | . | . | . | 4qD3 | rs27626103 | 1 | 0 |
| chr4 | 139830222 | G | A | 228 | Pax7 | exonic | synonymous SNV | Pax7:NM_011039:exon2:c.C237T:p.S79S | . | 4qD3 | rs27626102 | 1 | 0 |
| chr4 | 139830318 | G | A | 228 | Pax7 | exonic | synonymous SNV | Pax7:NM_011039:exon2:c.C141T:p.N47N | . | 4qD3 | rs13478018 | 1 | 0 |
| chr4 | 139830491 | T | C | 228 | Pax7 | intronic | . | . | . | 4qD3 | rs32434554 | 1 | 0 |
| chr4 | 139965184 | C | T | 137 | Klhdc7a | UTR3 | . | . | . | 4qD3 | rs46823880 | 1 | 0 |
| chr4 | 139965201 | C | G | 156 | Klhdc7a | UTR3 | . | . | . | 4qD3 | rs51265189 | 1 | 0 |
| chr4 | 139965526 | T | C | 228 | Klhdc7a | exonic | synonymous SNV | Klhdc7a:NM_173427:exon1:c.A2109G:p.T703T | . | 4qD3 | rs13469345 | 1 | 0 |
| chr4 | 139965973 | G | A | 228 | Klhdc7a | exonic | synonymous SNV | Klhdc7a:NM_173427:exon1:c.C1662T:p.R554R | . | 4qD3 | rs27592011 | 1 | 0 |
| chr4 | 139966582 | T | C | 228 | Klhdc7a | exonic | synonymous SNV | Klhdc7a:NM_173427:exon1:c.A1053G:p.P351P | . | 4qD3 | rs27592009 | 1 | 0 |
| chr4 | 139967030 | A | G | 228 | Klhdc7a | exonic | missense SNV | Klhdc7a:NM_173427:exon1:c.T605C:p.L202P | . | 4qD3 | rs51358303 | 1 | 0 |
| chr4 | 139967152 | T | C | 228 | Klhdc7a | exonic | synonymous SNV | Klhdc7a:NM_173427:exon1:c.A483G:p.S161S | . | 4qD3 | rs32140991 | 1 | 0 |
| chr4 | 139967441 | G | C | 218 | Klhdc7a | exonic | missense SNV | Klhdc7a:NM_173427:exon1:c.C194G:p.A65G | . | 4qD3 | rs27592007 | 1 | 0 |
| chr4 | 140547194 | C | A | 191 | Arhgef10l | intronic | . | . | . | 4qD3 | . | 1 | 0 |
| chr4 | 140803436 | A | G | 144 | Padi3 | intronic | . | . | . | 4qD3 | . | 0 | 1 |
| chr4 | 141005515 | T | A | 170 | Atp13a2 | intronic | . | . | . | 4qD3 | . | 1 | 0 |
| chr4 | 141308435 | A | G | 151 | Epha2 | exonic | missense SNV | Epha2:NM_010139:exon3:c.A181G:p.M61V | . | 4qD3 | . | 0 | 1 |
| chr4 | 141463550 | G | T | 228 | Zbtb17 | intronic | . | . | . | 4qD3 | rs27604118 | 1 | 0 |
| chr4 | 141464310 | G | A | 228 | Zbtb17 | exonic | synonymous SNV | Zbtb17:NM_009541:exon7:c.G732A:p.K244K | . | 4qD3 | rs6212221 | 1 | 0 |
| chr4 | 141464722 | T | C | 228 | Zbtb17 | intronic | . | . | . | 4qD3 | rs27604115 | 1 | 0 |
| chr4 | 141464978 | C | T | 228 | Zbtb17 | exonic | synonymous SNV | Zbtb17:NM_009541:exon9:c.C1233T:p.C411C | . | 4qD3 | rs32080005 | 1 | 0 |
| chr4 | 141465290 | T | C | 228 | Zbtb17 | intronic | . | . | . | 4qD3 | rs32137119 | 1 | 0 |
| chr4 | 141465858 | C | T | 228 | Zbtb17 | intronic | . | . | . | 4qD3 | rs49035414 | 1 | 0 |
| chr4 | 141466118 | G | A | 228 | Zbtb17 | intronic | . | . | . | 4qD3 | rs46598200 | 1 | 0 |
| chr4 | 141466209 | T | C | 228 | Zbtb17 | intronic | . | . | . | 4qD3 | rs27604113 | 1 | 0 |
| chr4 | 141466373 | A | G | 228 | Zbtb17 | exonic | synonymous SNV | Zbtb17:NM_009541:exon14:c.A1827G:p.K609K | . | 4qD3 | rs27604112 | 1 | 0 |
| chr4 | 141466553 | A | C | 228 | Zbtb17 | exonic | synonymous SNV | Zbtb17:NM_009541:exon14:c.A2007C:p.L669L | . | 4qD3 | rs13461716 | 1 | 0 |
| chr4 | 141469570 | T | G | 228 | Spen | intronic | . | . | . | 4qD3 | rs27604096 | 1 | 0 |
| chr4 | 141469770 | G | C | 167 | Spen | intronic | . | . | . | 4qD3 | rs31911865 | 1 | 0 |
| chr4 | 141471910 | A | G | 228 | Spen | exonic | synonymous SNV | Spen:NM_019763:exon12:c.T9405C:p.H3135H | . | 4qD3 | rs27604084 | 1 | 0 |
| chr4 | 141472289 | A | G | 228 | Spen | exonic | missense SNV | Spen:NM_019763:exon12:c.T9026C:p.L3009P | . | 4qD3 | rs27604082 | 1 | 0 |
| chr4 | 141473572 | G | A | 228 | Spen | exonic | synonymous SNV | Spen:NM_019763:exon12:c.C7743T:p.G2581G | . | 4qD3 | rs27604081 | 1 | 0 |
| chr4 | 141473658 | A | T | 228 | Spen | exonic | missense SNV | Spen:NM_019763:exon12:c.T7657A:p.S2553T | . | 4qD3 | rs27604080 | 1 | 0 |
| chr4 | 141473782 | T | C | 228 | Spen | exonic | synonymous SNV | Spen:NM_019763:exon12:c.A7533G:p.P2511P | . | 4qD3 | rs32455050 | 1 | 0 |
| chr4 | 141474164 | T | G | 228 | Spen | exonic | missense SNV | Spen:NM_019763:exon12:c.A7151C:p.Q2384P | . | 4qD3 | rs32080916 | 1 | 0 |
| chr4 | 141474328 | G | A | 228 | Spen | exonic | synonymous SNV | Spen:NM_019763:exon12:c.C6987T:p.D2329D | . | 4qD3 | rs27604078 | 1 | 0 |
| chr4 | 141474353 | A | G | 228 | Spen | exonic | missense SNV | Spen:NM_019763:exon12:c.T6962C:p.V2321A | . | 4qD3 | rs27604077 | 1 | 0 |
| chr4 | 141474511 | C | T | 228 | Spen | exonic | synonymous SNV | Spen:NM_019763:exon12:c.G6804A:p.G2268G | . | 4qD3 | rs32247690 | 1 | 0 |
| chr4 | 141475022 | G | C | 170 | Spen | exonic | missense SNV | Spen:NM_019763:exon12:c.C6293G:p.A2098G | . | 4qD3 | rs27604073 | 1 | 0 |
| chr4 | 141475118 | T | C | 228 | Spen | exonic | missense SNV | Spen:NM_019763:exon12:c.A6197G:p.N2066S | . | 4qD3 | rs27604072 | 1 | 0 |
| chr4 | 141476198 | G | A | 228 | Spen | exonic | missense SNV | Spen:NM_019763:exon12:c.C5117T:p.A1706V | . | 4qD3 | rs27604069 | 1 | 0 |
| chr4 | 141476263 | T | C | 228 | Spen | exonic | synonymous SNV | Spen:NM_019763:exon12:c.A5052G:p.P1684P | . | 4qD3 | rs27604068 | 1 | 0 |
| chr4 | 141476337 | C | T | 228 | Spen | exonic | missense SNV | Spen:NM_019763:exon12:c.G4978A:p.V1660I | . | 4qD3 | rs27580365 | 1 | 0 |
| chr4 | 141476671 | G | A | 158 | Spen | exonic | synonymous SNV | Spen:NM_019763:exon12:c.C4644T:p.H1548H | . | 4qD3 | rs27580360 | 1 | 0 |
| chr4 | 141476741 | G | T | 222 | Spen | exonic | missense SNV | Spen:NM_019763:exon12:c.C4574A:p.A1525D | . | 4qD3 | rs27580357 | 1 | 0 |
| chr4 | 141476860 | C | G | 228 | Spen | exonic | synonymous SNV | Spen:NM_019763:exon12:c.G4455C:p.S1485S | . | 4qD3 | rs27580356 | 1 | 0 |
| chr4 | 141477331 | G | A | 228 | Spen | exonic | synonymous SNV | Spen:NM_019763:exon12:c.C3984T:p.Y1328Y | . | 4qD3 | rs27580353 | 1 | 0 |
| chr4 | 141477814 | G | C | 228 | Spen | exonic | missense SNV | Spen:NM_019763:exon12:c.C3501G:p.H1167Q | . | 4qD3 | rs27580352 | 1 | 0 |
| chr4 | 141477980 | A | G | 228 | Spen | exonic | missense SNV | Spen:NM_019763:exon12:c.T3335C:p.V1112A | . | 4qD3 | rs27580351 | 1 | 0 |
| chr4 | 141478339 | A | C | 228 | Spen | exonic | synonymous SNV | Spen:NM_019763:exon12:c.T2976G:p.A992A | . | 4qD3 | rs27580349 | 1 | 0 |
| chr4 | 141478488 | C | A | 228 | Spen | exonic | missense SNV | Spen:NM_019763:exon12:c.G2827T:p.A943S | 802 | 4qD3 | rs32427655 | 1 | 0 |
| chr4 | 141478524 | T | C | 228 | Spen | exonic | missense SNV | Spen:NM_019763:exon12:c.A2791G:p.T931A | 802 | 4qD3 | rs27580348 | 1 | 0 |
| chr4 | 141478555 | T | C | 228 | Spen | exonic | synonymous SNV | Spen:NM_019763:exon12:c.A2760G:p.E920E | 802 | 4qD3 | rs32465167 | 1 | 0 |
| chr4 | 141478683 | T | C | 228 | Spen | exonic | missense SNV | Spen:NM_019763:exon12:c.A2632G:p.T878A | 802 | 4qD3 | rs27580347 | 1 | 0 |
| chr4 | 141480835 | C | T | 228 | Spen | intronic | . | . | . | 4qD3 | rs32424703 | 1 | 0 |
| chr4 | 141483906 | G | A | 210 | Spen | intronic | . | . | . | 4qD3 | rs46222604 | 1 | 0 |
| chr4 | 141485456 | T | C | 178 | Spen | intronic | . | . | . | 4qD3 | rs27580313 | 1 | 0 |
| chr4 | 141485777 | G | A | 130 | Spen | intronic | . | . | . | 4qD3 | rs27580310 | 1 | 0 |
| chr4 | 141487520 | C | T | 228 | Spen | intronic | . | . | . | 4qD3 | rs27580301 | 1 | 0 |
| chr4 | 141487793 | G | A | 228 | Spen | intronic | . | . | . | 4qD3 | rs32341758 | 1 | 0 |
| chr4 | 141487859 | C | T | 228 | Spen | intronic | . | . | . | 4qD3 | rs27580299 | 1 | 0 |
| chr4 | 141487973 | G | A | 228 | Spen | intronic | . | . | . | 4qD3 | rs27580297 | 1 | 0 |
| chr4 | 141487986 | T | C | 228 | Spen | intronic | . | . | . | 4qD3 | rs27580296 | 1 | 0 |
| chr4 | 141488127 | G | A | 228 | Spen | intronic | . | . | . | 4qD3 | rs27580295 | 1 | 0 |
| chr4 | 141488239 | A | G | 216 | Spen | intronic | . | . | . | 4qD3 | rs108574666 | 1 | 0 |
| chr4 | 141488302 | A | G | 160 | Spen | intronic | . | . | . | 4qD3 | rs27580294 | 1 | 0 |
| chr4 | 141489660 | G | A | 144 | Spen | intronic | . | . | . | 4qD3 | rs27580290 | 1 | 0 |
| chr4 | 141489690 | C | G | 161 | Spen | intronic | . | . | . | 4qD3 | rs27580289 | 1 | 0 |
| chr4 | 141489760 | A | T | 211 | Spen | intronic | . | . | . | 4qD3 | rs27580287 | 1 | 0 |
| chr4 | 141489819 | C | T | 228 | Spen | intronic | . | . | . | 4qD3 | rs27580286 | 1 | 0 |
| chr4 | 141490028 | G | A | 228 | Spen | intronic | . | . | . | 4qD3 | rs27580285 | 1 | 0 |
| chr4 | 141490054 | T | C | 228 | Spen | intronic | . | . | . | 4qD3 | rs46876757 | 1 | 0 |
| chr4 | 141490126 | G | A | 228 | Spen | intronic | . | . | . | 4qD3 | rs27580283 | 1 | 0 |
| chr4 | 141494165 | A | G | 168 | Spen | intronic | . | . | . | 4qD3 | rs107854742 | 1 | 0 |
| chr4 | 141494179 | C | T | 210 | Spen | intronic | . | . | . | 4qD3 | rs212261807 | 1 | 0 |
| chr4 | 141516777 | C | G | 132 | Spen | intronic | . | . | . | 4qD3 | rs27580187 | 1 | 0 |
| chr4 | 141517493 | G | T | 135 | Spen | intronic | . | . | . | 4qD3 | rs27580186 | 1 | 0 |
| chr4 | 141522433 | T | C | 228 | Spen | intronic | . | . | . | 4qD3 | rs27580168 | 1 | 0 |
| chr4 | 141522505 | A | G | 153 | Spen | intronic | . | . | . | 4qD3 | rs27580167 | 1 | 0 |
| chr4 | 141577647 | A | C | 170 | Fblim1 | UTR3 | . | . | . | 4qD3 | rs224786573 | 1 | 0 |
| chr4 | 141577857 | C | T | 228 | Fblim1 | exonic | synonymous SNV | Fblim1:NM_001163256:exon9:c.G1101A:p.L367L,Fblim1:NM_133754:exon9:c.G1101A:p.L367L | . | 4qD3 | rs27580073 | 1 | 0 |
| chr4 | 141577914 | A | G | 228 | Fblim1 | exonic | synonymous SNV | Fblim1:NM_001163256:exon9:c.T1044C:p.P348P,Fblim1:NM_133754:exon9:c.T1044C:p.P348P | . | 4qD3 | rs27580072 | 1 | 0 |
| chr4 | 141577986 | C | T | 228 | Fblim1 | intronic | . | . | . | 4qD3 | rs27580071 | 1 | 0 |
| chr4 | 141583031 | C | G | 132 | Fblim1 | intronic | . | . | . | 4qD3 | rs582695654 | 1 | 0 |
| chr4 | 141583284 | G | A | 228 | Fblim1 | intronic | . | . | . | 4qD3 | rs224799437 | 1 | 0 |
| chr4 | 141584765 | G | A | 228 | Fblim1 | exonic | synonymous SNV | Fblim1:NM_001163256:exon6:c.C648T:p.C216C,Fblim1:NM_133754:exon6:c.C648T:p.C216C | . | 4qD3 | rs241648920 | 1 | 0 |
| chr4 | 141584831 | A | G | 228 | Fblim1 | exonic | synonymous SNV | Fblim1:NM_001163256:exon6:c.T582C:p.P194P,Fblim1:NM_133754:exon6:c.T582C:p.P194P | . | 4qD3 | rs257081995 | 1 | 0 |
| chr4 | 141584837 | C | A | 228 | Fblim1 | exonic | synonymous SNV | Fblim1:NM_001163256:exon6:c.G576T:p.V192V,Fblim1:NM_133754:exon6:c.G576T:p.V192V | . | 4qD3 | rs228714273 | 1 | 0 |
| chr4 | 141590004 | C | T | 220 | Fblim1 | exonic | synonymous SNV | Fblim1:NM_001163256:exon4:c.G369A:p.S123S,Fblim1:NM_133754:exon4:c.G369A:p.S123S | . | 4qD3 | rs27565049 | 1 | 0 |
| chr4 | 141590195 | T | A | 95 | Fblim1 | intronic | . | . | . | 4qD3 | rs27565048 | 1 | 0 |
| chr4 | 141595276 | A | G | 228 | Fblim1 | exonic | synonymous SNV | Fblim1:NM_001163256:exon3:c.T195C:p.S65S,Fblim1:NM_133754:exon3:c.T195C:p.S65S | . | 4qD3 | rs218200904 | 1 | 0 |
| chr4 | 141595476 | C | G | 228 | Fblim1 | UTR5 | . | . | . | 4qD3 | rs235692882 | 1 | 0 |
| chr4 | 141614909 | G | A | 228 | Tmem82,AI507597 | ncRNA intronic | . | . | . | 4qD3 | rs32793501 | 1 | 0 |
| chr4 | 141615127 | A | G | 228 | AI507597,Tmem82 | exonic | missense SNV | Tmem82:NM_145987:exon5:c.T763C:p.W255R | . | 4qD3 | rs13478025 | 1 | 0 |
| chr4 | 141615223 | G | C | 126 | AI507597,Tmem82 | ncRNA exonic | . | . | . | 4qD3 | rs32735357 | 1 | 0 |
| chr4 | 141616113 | G | A | 187 | Tmem82 | intronic | . | . | . | 4qD3 | rs32968816 | 1 | 0 |
| chr4 | 141617240 | T | C | 195 | Tmem82 | intronic | . | . | . | 4qD3 | rs251262830 | 1 | 0 |
| chr4 | 141617357 | C | A | 228 | Tmem82 | exonic | missense SNV | Tmem82:NM_145987:exon3:c.G274T:p.A92S | . | 4qD3 | rs263116929 | 1 | 0 |
| chr4 | 141617491 | C | G | 228 | Tmem82 | intronic | . | . | . | 4qD3 | rs32865454 | 1 | 0 |
| chr4 | 141617672 | G | A | 228 | Tmem82 | exonic | synonymous SNV | Tmem82:NM_145987:exon2:c.C129T:p.L43L | . | 4qD3 | rs245186898 | 1 | 0 |
| chr4 | 141617957 | C | A | 228 | Tmem82 | exonic | missense SNV | Tmem82:NM_145987:exon1:c.G30T:p.W10C | . | 4qD3 | rs258528851 | 1 | 0 |
| chr4 | 141618129 | T | C | 127 | Tmem82 | UTR5 | . | . | . | 4qD3 | rs31896249 | 1 | 0 |
| chr4 | 141620360 | A | G | 228 | Slc25a34 | UTR3 | . | . | . | 4qD3 | rs32433574 | 1 | 0 |
| chr4 | 141620656 | A | G | 228 | Slc25a34 | intronic | . | . | . | 4qD3 | rs225719549 | 1 | 0 |
| chr4 | 141620691 | G | A | 221 | Slc25a34 | intronic | . | . | . | 4qD3 | rs248323413 | 1 | 0 |
| chr4 | 141621348 | A | G | 173 | Slc25a34 | intronic | . | . | . | 4qD3 | rs258207547 | 1 | 0 |
| chr4 | 141621540 | T | C | 228 | Slc25a34 | intronic | . | . | . | 4qD3 | rs224422931 | 1 | 0 |
| chr4 | 141622096 | A | G | 154 | Slc25a34 | intronic | . | . | . | 4qD3 | rs231649217 | 1 | 0 |
| chr4 | 141622179 | T | A | 228 | Slc25a34 | intronic | . | . | . | 4qD3 | rs264062766 | 1 | 0 |
| chr4 | 141622393 | A | G | 228 | Slc25a34 | intronic | . | . | . | 4qD3 | rs32561168 | 1 | 0 |
| chr4 | 141622600 | C | T | 110 | Slc25a34 | intronic | . | . | . | 4qD3 | rs252724325 | 1 | 0 |
| chr4 | 141623397 | G | T | 228 | Slc25a34 | exonic | synonymous SNV | Slc25a34:NM_001013780:exon1:c.C300A:p.V100V | . | 4qD3 | rs231741338 | 1 | 0 |
| chr4 | 141623457 | T | C | 228 | Slc25a34 | exonic | synonymous SNV | Slc25a34:NM_001013780:exon1:c.A240G:p.L80L | . | 4qD3 | rs244842387 | 1 | 0 |
| chr4 | 141623719 | C | T | 228 | Slc25a34 | UTR5 | . | . | . | 4qD3 | rs212357667 | 1 | 0 |
| chr4 | 141626449 | G | A | 177 | Plekhm2 | UTR3 | . | . | . | 4qE1 | . | 1 | 0 |
| chr4 | 141626642 | G | A | 228 | Plekhm2 | exonic | synonymous SNV | Plekhm2:NM_001033150:exon20:c.C3030T:p.S1010S | . | 4qE1 | rs27564999 | 1 | 0 |
| chr4 | 141626801 | T | C | 228 | Plekhm2 | intronic | . | . | . | 4qE1 | rs27564998 | 1 | 0 |
| chr4 | 141626860 | A | G | 155 | Plekhm2 | intronic | . | . | . | 4qE1 | rs27564997 | 1 | 0 |
| chr4 | 141628274 | A | G | 228 | Plekhm2 | intronic | . | . | . | 4qE1 | rs27564991 | 1 | 0 |
| chr4 | 141628919 | G | A | 168 | Plekhm2 | intronic | . | . | . | 4qE1 | rs27564986 | 1 | 0 |
| chr4 | 141629054 | C | A | 228 | Plekhm2 | intronic | . | . | . | 4qE1 | rs258186107 | 1 | 0 |
| chr4 | 141629215 | T | C | 228 | Plekhm2 | intronic | . | . | . | 4qE1 | rs27564985 | 1 | 0 |
| chr4 | 141629388 | A | G | 228 | Plekhm2 | intronic | . | . | . | 4qE1 | rs32853973 | 1 | 0 |
| chr4 | 141629545 | A | G | 228 | Plekhm2 | exonic | synonymous SNV | Plekhm2:NM_001033150:exon15:c.T2346C:p.A782A | . | 4qE1 | rs27564984 | 1 | 0 |
| chr4 | 141629675 | A | G | 228 | Plekhm2 | intronic | . | . | . | 4qE1 | rs229277722 | 1 | 0 |
| chr4 | 141629677 | T | C | 228 | Plekhm2 | intronic | . | . | . | 4qE1 | rs27564982 | 1 | 0 |
| chr4 | 141630449 | A | C | 166 | Plekhm2 | intronic | . | . | . | 4qE1 | rs49442473 | 1 | 0 |
| chr4 | 141630541 | G | A | 228 | Plekhm2 | intronic | . | . | . | 4qE1 | rs231230641 | 1 | 0 |
| chr4 | 141630794 | A | G | 228 | Plekhm2 | intronic | . | . | . | 4qE1 | rs251634107 | 1 | 0 |
| chr4 | 141630965 | T | C | 228 | Plekhm2 | intronic | . | . | . | 4qE1 | rs27564980 | 1 | 0 |
| chr4 | 141631049 | T | C | 228 | Plekhm2 | exonic | synonymous SNV | Plekhm2:NM_001033150:exon12:c.A1971G:p.T657T | . | 4qE1 | rs27564979 | 1 | 0 |
| chr4 | 141631074 | T | A | 228 | Plekhm2 | intronic | . | . | . | 4qE1 | rs27564978 | 1 | 0 |
| chr4 | 141631167 | G | A | 228 | Plekhm2 | intronic | . | . | . | 4qE1 | rs27564977 | 1 | 0 |
| chr4 | 141631495 | G | A | 228 | Plekhm2 | intronic | . | . | . | 4qE1 | rs46429881 | 1 | 0 |
| chr4 | 141632459 | T | C | 228 | Plekhm2 | exonic | synonymous SNV | Plekhm2:NM_001033150:exon9:c.A1056G:p.G352G | . | 4qE1 | rs27564974 | 1 | 0 |
| chr4 | 141632513 | T | C | 228 | Plekhm2 | exonic | synonymous SNV | Plekhm2:NM_001033150:exon9:c.A1002G:p.P334P | . | 4qE1 | rs253955825 | 1 | 0 |
| chr4 | 141634160 | A | G | 158 | Plekhm2 | intronic | . | . | . | 4qE1 | rs245476433 | 1 | 0 |
| chr4 | 141634240 | A | G | 228 | Plekhm2 | intronic | . | . | . | 4qE1 | rs27564969 | 1 | 0 |
| chr4 | 141637133 | G | T | 101 | Plekhm2 | intronic | . | . | . | 4qE1 | rs245487503 | 1 | 0 |
| chr4 | 141637156 | A | C | 137 | Plekhm2 | intronic | . | . | . | 4qE1 | rs264495039 | 1 | 0 |
| chr4 | 141637595 | A | G | 171 | Plekhm2 | intronic | . | . | . | 4qE1 | rs228256196 | 1 | 0 |
| chr4 | 141637631 | A | G | 155 | Plekhm2 | intronic | . | . | . | 4qE1 | rs259215576 | 1 | 0 |
| chr4 | 141639607 | C | T | 228 | Plekhm2 | intronic | . | . | . | 4qE1 | rs228944761 | 1 | 0 |
| chr4 | 141639800 | C | T | 228 | Plekhm2 | exonic | synonymous SNV | Plekhm2:NM_001033150:exon6:c.G528A:p.L176L | . | 4qE1 | rs247022117 | 1 | 0 |
| chr4 | 141639964 | T | C | 228 | Plekhm2 | intronic | . | . | . | 4qE1 | rs266049417 | 1 | 0 |
| chr4 | 141641239 | A | C | 171 | Plekhm2 | intronic | . | . | . | 4qE1 | rs221251883 | 1 | 0 |
| chr4 | 141641246 | A | T | 191 | Plekhm2 | intronic | . | . | . | 4qE1 | rs249802567 | 1 | 0 |
| chr4 | 141641555 | A | G | 228 | Plekhm2 | intronic | . | . | . | 4qE1 | rs264264581 | 1 | 0 |
| chr4 | 141641851 | G | A | 152 | Plekhm2 | intronic | . | . | . | 4qE1 | rs226495933 | 1 | 0 |
| chr4 | 141641903 | A | G | 228 | Plekhm2 | intronic | . | . | . | 4qE1 | rs240024290 | 1 | 0 |
| chr4 | 141641932 | G | T | 228 | Plekhm2 | intronic | . | . | . | 4qE1 | rs255388176 | 1 | 0 |
| chr4 | 141642269 | A | G | 109 | Plekhm2 | intronic | . | . | . | 4qE1 | . | 1 | 0 |
| chr4 | 141642326 | C | T | 225 | Plekhm2 | intronic | . | . | . | 4qE1 | rs226041914 | 1 | 0 |
| chr4 | 141642443 | A | G | 228 | Plekhm2 | exonic | synonymous SNV | Plekhm2:NM_001033150:exon3:c.T240C:p.I80I | . | 4qE1 | rs6372162 | 1 | 0 |
| chr4 | 141642703 | G | A | 228 | Plekhm2 | exonic | synonymous SNV | Plekhm2:NM_001033150:exon2:c.C114T:p.H38H | . | 4qE1 | rs213103125 | 1 | 0 |
| chr4 | 141642792 | T | G | 228 | Plekhm2 | intronic | . | . | . | 4qE1 | rs233281824 | 1 | 0 |
| chr4 | 141683825 | C | A | 228 | Ddi2,Rsc1a1 | UTR3 | . | . | . | 4qE1 | rs13464402 | 1 | 0 |
| chr4 | 141683983 | A | G | 228 | Ddi2,Rsc1a1 | exonic | synonymous SNV | Rsc1a1:NM_023544:exon1:c.T1617C:p.A539A | . | 4qE1 | rs27564778 | 1 | 0 |
| chr4 | 141684217 | T | C | 228 | Rsc1a1,Ddi2 | exonic | synonymous SNV | Rsc1a1:NM_023544:exon1:c.A1383G:p.S461S | . | 4qE1 | rs13464401 | 1 | 0 |
| chr4 | 141684281 | T | C | 228 | Ddi2,Rsc1a1 | exonic | missense SNV | Rsc1a1:NM_023544:exon1:c.A1319G:p.D440G | . | 4qE1 | rs13464403 | 1 | 0 |
| chr4 | 141684448 | C | T | 228 | Rsc1a1,Ddi2 | exonic | synonymous SNV | Rsc1a1:NM_023544:exon1:c.G1152A:p.R384R | . | 4qE1 | rs27564776 | 1 | 0 |

| | | | | | | | | | | | | | | |
|---|---|---|---|---|---|---|---|---|---|---|---|---|---|---|
| chr4 | 141684459 | A | G | 228 | Rsc1a1,Ddi2 | exonic | missense SNV | Rsc1a1:NM_023544:exon1:c.T1141C:p.S381P | . | | 4qE1 | rs27564775 | 1 | 0 |
| chr4 | 141684523 | T | C | 228 | Rsc1a1,Ddi2 | exonic | synonymous SNV | Rsc1a1:NM_023544:exon1:c.A1077G:p.S359S | . | | 4qE1 | rs27564774 | 1 | 0 |
| chr4 | 141684633 | C | T | 228 | Rsc1a1,Ddi2 | exonic | missense SNV | Rsc1a1:NM_023544:exon1:c.G967A:p.G323S | . | | 4qE1 | rs27564773 | 1 | 0 |
| chr4 | 141684999 | C | T | 228 | Rsc1a1,Ddi2 | exonic | missense SNV | Rsc1a1:NM_023544:exon1:c.G601A:p.A201T | . | | 4qE1 | rs27564771 | 1 | 0 |
| chr4 | 141685047 | A | C | 228 | Rsc1a1,Ddi2 | exonic | missense SNV | Rsc1a1:NM_023544:exon1:c.T553G:p.C185G | . | | 4qE1 | rs27564770 | 1 | 0 |
| chr4 | 141685178 | G | A | 228 | Rsc1a1,Ddi2 | exonic | missense SNV | Rsc1a1:NM_023544:exon1:c.C422T:p.A141V | . | | 4qE1 | rs27564769 | 1 | 0 |
| chr4 | 141685195 | C | A | 228 | Ddi2,Rsc1a1 | exonic | synonymous SNV | Rsc1a1:NM_023544:exon1:c.G405T:p.R135R | . | | 4qE1 | rs222329765 | 1 | 0 |
| chr4 | 141685196 | C | T | 228 | Rsc1a1,Ddi2 | exonic | missense SNV | Rsc1a1:NM_023544:exon1:c.G404A:p.R135Q | . | | 4qE1 | rs27564768 | 1 | 0 |
| chr4 | 141689118 | A | G | 169 | Ddi2 | intronic | . | . | . | | 4qE1 | rs27645184 | 1 | 0 |
| chr4 | 141689642 | T | A | 142 | Ddi2 | intronic | . | . | . | | 4qE1 | rs27645183 | 1 | 0 |
| chr4 | 141692115 | G | A | 170 | Ddi2 | intronic | . | . | . | | 4qE1 | rs27628359 | 1 | 0 |
| chr4 | 141692170 | T | C | 228 | Ddi2 | intronic | . | . | . | | 4qE1 | rs27628358 | 1 | 0 |
| chr4 | 141692519 | C | T | 149 | Ddi2 | intronic | . | . | . | | 4qE1 | rs27628357 | 1 | 0 |
| chr4 | 141695581 | A | G | 228 | Ddi2 | intronic | . | . | . | | 4qE1 | rs27628344 | 1 | 0 |
| chr4 | 141695607 | C | T | 169 | Ddi2 | intronic | . | . | . | | 4qE1 | rs27628343 | 1 | 0 |
| chr4 | 141695674 | A | G | 133 | Ddi2 | intronic | . | . | . | | 4qE1 | rs27628342 | 1 | 0 |
| chr4 | 141699027 | A | T | 169 | Ddi2 | intronic | . | . | . | | 4qE1 | rs27628321 | 1 | 0 |
| chr4 | 141702930 | T | C | 153 | Ddi2 | intronic | . | . | . | | 4qE1 | rs47924148 | 1 | 0 |
| chr4 | 141703258 | T | C | 228 | Ddi2 | intronic | . | . | . | | 4qE1 | rs27628298 | 1 | 0 |
| chr4 | 141708189 | T | C | 228 | Ddi2 | intronic | . | . | . | | 4qE1 | rs27628280 | 1 | 0 |
| chr4 | 141708205 | G | C | 228 | Ddi2 | intronic | . | . | . | | 4qE1 | rs27628279 | 1 | 0 |
| chr4 | 141713371 | G | A | 228 | Ddi2 | intronic | . | . | . | | 4qE1 | rs27628261 | 1 | 0 |
| chr4 | 141713449 | C | T | 228 | Ddi2 | exonic | synonymous SNV | Ddi2:NM_001017966:exon2:c.G216A:p.V72V | . | | 4qE1 | rs27628260 | 1 | 0 |
| chr4 | 141746664 | G | A | 101 | . | upstream | . | . | . | | 4qE1 | rs258820215 | 1 | 0 |
| chr4 | 141746816 | A | C | 228 | Agmat | exonic | synonymous SNV | Agmat:NM_001081408:exon1:c.A7C:p.R3R | . | | 4qE1 | rs13461329 | 1 | 0 |
| chr4 | 141746992 | T | C | 226 | Agmat | exonic | synonymous SNV | Agmat:NM_001081408:exon1:c.T183C:p.G61G | . | | 4qE1 | rs13461327 | 1 | 0 |
| chr4 | 141747011 | C | T | 228 | Agmat | exonic | synonymous SNV | Agmat:NM_001081408:exon1:c.C202T:p.L68L | . | | 4qE1 | rs13461331 | 1 | 0 |
| chr4 | 141749718 | T | G | 228 | Agmat | exonic | missense SNV | Agmat:NM_001081408:exon2:c.T434G:p.L145R | . | | 4qE1 | rs13461330 | 1 | 0 |
| chr4 | 141749859 | T | C | 228 | Agmat | intronic | . | . | . | | 4qE1 | rs27628182 | 1 | 0 |
| chr4 | 141749920 | A | C | 95 | Agmat | intronic | . | . | . | | 4qE1 | rs27628181 | 1 | 0 |
| chr4 | 141754902 | G | A | 228 | Agmat | intronic | . | . | . | | 4qE1 | rs217140719 | 1 | 0 |
| chr4 | 141756016 | T | C | 228 | Agmat | intronic | . | . | . | | 4qE1 | rs27628166 | 1 | 0 |
| chr4 | 141756026 | A | T | 228 | Agmat | intronic | . | . | . | | 4qE1 | rs583783596 | 1 | 0 |
| chr4 | 141757243 | C | A | 153 | Agmat | intronic | . | . | . | | 4qE1 | rs249279174 | 1 | 0 |
| chr4 | 141758386 | C | T | 228 | Agmat | exonic | synonymous SNV | Agmat:NM_001081408:exon6:c.C966T:p.C322C | . | | 4qE1 | rs262237336 | 1 | 0 |
| chr4 | 141758460 | T | C | 228 | Agmat | intronic | . | . | . | | 4qE1 | rs230787879 | 1 | 0 |
| chr4 | 141758538 | A | G | 59 | Agmat | intronic | . | . | . | | 4qE1 | rs248858035 | 1 | 0 |
| chr4 | 141759253 | T | C | 102 | Agmat | UTR3 | . | . | . | | 4qE1 | rs238522097 | 1 | 0 |
| chr4 | 141763537 | C | G | 228 | Dnajc16 | exonic | synonymous SNV | Dnajc16:NM_172338:exon15:c.G2301C:p.S767S | . | | 4qE1 | rs27628143 | 1 | 0 |
| chr4 | 141763919 | C | T | 228 | Dnajc16 | intronic | . | . | . | | 4qE1 | rs27628142 | 1 | 0 |
| chr4 | 141763926 | T | C | 228 | Dnajc16 | intronic | . | . | . | | 4qE1 | rs265508985 | 1 | 0 |
| chr4 | 141764484 | C | T | 107 | Dnajc16 | intronic | . | . | . | | 4qE1 | rs245327901 | 1 | 0 |
| chr4 | 141764570 | G | A | 228 | Dnajc16 | exonic | synonymous SNV | Dnajc16:NM_172338:exon14:c.C1941T:p.T647T | . | | 4qE1 | rs236538528 | 1 | 0 |
| chr4 | 141766507 | C | T | 228 | Dnajc16 | exonic | synonymous SNV | Dnajc16:NM_172338:exon13:c.G1749A:p.P583P | . | | 4qE1 | rs27628133 | 1 | 0 |
| chr4 | 141766633 | C | T | 228 | Dnajc16 | intronic | . | . | . | | 4qE1 | rs6269268 | 1 | 0 |
| chr4 | 141766763 | G | A | 228 | Dnajc16 | intronic | . | . | . | | 4qE1 | rs6172091 | 1 | 0 |
| chr4 | 141766808 | C | T | 215 | Dnajc16 | intronic | . | . | . | | 4qE1 | rs6172571 | 1 | 0 |
| chr4 | 141767659 | G | A | 228 | Dnajc16 | intronic | . | . | . | | 4qE1 | rs27628125 | 1 | 0 |
| chr4 | 141767827 | G | A | 228 | Dnajc16 | intronic | . | . | . | | 4qE1 | rs237593913 | 1 | 0 |
| chr4 | 141767829 | C | A | 228 | Dnajc16 | intronic | . | . | . | | 4qE1 | rs257840694 | 1 | 0 |
| chr4 | 141769787 | C | T | 228 | Dnajc16 | intronic | . | . | . | | 4qE1 | rs27628113 | 1 | 0 |
| chr4 | 141769824 | A | G | 228 | Dnajc16 | intronic | . | . | . | | 4qE1 | rs27628112 | 1 | 0 |
| chr4 | 141769979 | G | A | 228 | Dnajc16 | exonic | synonymous SNV | Dnajc16:NM_172338:exon9:c.C1206T:p.A402A | . | | 4qE1 | rs27628111 | 1 | 0 |
| chr4 | 141770851 | A | G | 228 | Dnajc16 | intronic | . | . | . | | 4qE1 | rs27628104 | 1 | 0 |
| chr4 | 141770933 | A | G | 228 | Dnajc16 | exonic | synonymous SNV | Dnajc16:NM_172338:exon8:c.T1122C:p.P374P | . | | 4qE1 | rs27628102 | 1 | 0 |
| chr4 | 141771026 | C | T | 228 | Dnajc16 | exonic | synonymous SNV | Dnajc16:NM_172338:exon8:c.G1029A:p.L343L | . | | 4qE1 | rs27628101 | 1 | 0 |
| chr4 | 141771091 | C | A | 228 | Dnajc16 | intronic | . | . | . | | 4qE1 | rs27628100 | 1 | 0 |
| chr4 | 141771147 | A | G | 228 | Dnajc16 | intronic | . | . | . | | 4qE1 | rs27628099 | 1 | 0 |
| chr4 | 141774423 | G | A | 228 | Dnajc16 | intronic | . | . | . | | 4qE1 | rs27628084 | 1 | 0 |
| chr4 | 141774676 | A | G | 228 | Dnajc16 | intronic | . | . | . | | 4qE1 | rs27628082 | 1 | 0 |
| chr4 | 141774823 | A | G | 171 | Dnajc16 | intronic | . | . | . | | 4qE1 | rs27628079 | 1 | 0 |
| chr4 | 141775328 | C | T | 228 | Dnajc16 | intronic | . | . | . | | 4qE1 | rs220356074 | 1 | 0 |
| chr4 | 141775331 | C | T | 228 | Dnajc16 | intronic | . | . | . | | 4qE1 | rs236186459 | 1 | 0 |
| chr4 | 141776863 | G | A | 228 | Dnajc16 | intronic | . | . | . | | 4qE1 | rs27628072 | 1 | 0 |
| chr4 | 141776979 | C | T | 228 | Dnajc16 | exonic | synonymous SNV | Dnajc16:NM_172338:exon5:c.G669A:p.K223K | . | | 4qE1 | rs27628071 | 1 | 0 |
| chr4 | 141788817 | A | G | 228 | Dnajc16 | intronic | . | . | . | | 4qE1 | rs262709373 | 1 | 0 |
| chr4 | 141793826 | A | G | 228 | Casp9 | UTR5 | . | . | 640 | | 4qE1 | rs49260707 | 1 | 0 |
| chr4 | 141794116 | T | C | 135 | Casp9 | intronic | . | . | . | | 4qE1 | rs48898539 | 1 | 0 |
| chr4 | 141796273 | A | G | 161 | Casp9 | intronic | . | . | . | | 4qE1 | rs237275950 | 1 | 0 |
| chr4 | 141796692 | C | G | 228 | Casp9 | exonic | missense SNV | Casp9:NM_001277932:exon2:c.C390G:p.D130E,Casp9:NM_015733:exon2:c.C390G:p.D130E | . | | 4qE1 | rs253858154 | 1 | 0 |
| chr4 | 141796800 | C | G | 228 | Casp9 | exonic | synonymous SNV | Casp9:NM_001277932:exon2:c.C498G:p.P166P,Casp9:NM_015733:exon2:c.C498G:p.P166P | . | | 4qE1 | rs218953378 | 1 | 0 |
| chr4 | 141796965 | A | G | 204 | Casp9 | intronic | . | . | . | | 4qE1 | rs237702295 | 1 | 0 |
| chr4 | 141796970 | T | A | 203 | Casp9 | intronic | . | . | . | | 4qE1 | rs253809371 | 1 | 0 |
| chr4 | 141803556 | G | A | 101 | Casp9 | intronic | . | . | . | | 4qE1 | rs16783018 | 1 | 0 |
| chr4 | 141803560 | G | C | 109 | Casp9 | intronic | . | . | . | | 4qE1 | rs16783020 | 1 | 0 |
| chr4 | 141805553 | C | T | 228 | Casp9 | intronic | . | . | . | | 4qE1 | rs16791604 | 1 | 0 |
| chr4 | 141805562 | A | G | 228 | Casp9 | intronic | . | . | . | | 4qE1 | rs16791603 | 1 | 0 |
| chr4 | 141805804 | A | G | 228 | Casp9 | intronic | . | . | . | | 4qE1 | rs16791595 | 1 | 0 |
| chr4 | 141805952 | G | C | 228 | Casp9 | intronic | . | . | . | | 4qE1 | rs16791587 | 1 | 0 |
| chr4 | 141805993 | G | T | 228 | Casp9 | intronic | . | . | . | | 4qE1 | rs16791586 | 1 | 0 |
| chr4 | 141806110 | G | C | 61 | Casp9 | intronic | . | . | . | | 4qE1 | rs16791582 | 1 | 0 |
| chr4 | 141806998 | A | G | 196 | Casp9 | intronic | . | . | . | | 4qE1 | rs16783047 | 1 | 0 |
| chr4 | 141811624 | C | A | 228 | Casp9 | exonic | synonymous SNV | Casp9:NM_001277932:exon7:c.C1068A:p.P356P,Casp9:NM_015733:exon7:c.C1068A:p.P356P | . | | 4qE1 | rs16783063 | 1 | 0 |
| chr4 | 141811757 | T | C | 228 | Casp9 | intronic | . | . | . | | 4qE1 | rs16792960 | 1 | 0 |
| chr4 | 141811794 | G | T | 201 | Casp9 | intronic | . | . | . | | 4qE1 | rs16792964 | 1 | 0 |
| chr4 | 141812379 | T | G | 228 | Casp9 | intronic | . | . | . | | 4qE1 | rs27609374 | 1 | 0 |
| chr4 | 141817789 | C | T | 146 | Cela2a | intronic | . | . | . | | 4qE1 | rs27609313 | 1 | 0 |
| chr4 | 141817853 | T | G | 228 | Cela2a | intronic | . | . | . | | 4qE1 | rs219869140 | 1 | 0 |
| chr4 | 141818045 | A | G | 222 | Cela2a | intronic | . | . | . | | 4qE1 | rs27609310 | 1 | 0 |
| chr4 | 141821310 | C | G | 225 | Cela2a | intronic | . | . | . | | 4qE1 | rs227179473 | 1 | 0 |
| chr4 | 141821317 | A | T | 228 | Cela2a | intronic | . | . | . | | 4qE1 | rs247459750 | 1 | 0 |
| chr4 | 141821446 | T | C | 228 | Cela2a | exonic | synonymous SNV | Cela2a:NM_007919:exon5:c.A378G:p.L126L | . | | 4qE1 | rs27609266 | 1 | 0 |
| chr4 | 141821712 | C | T | 228 | Cela2a | exonic | missense SNV | Cela2a:NM_007919:exon4:c.G352A:p.V118I | . | | 4qE1 | rs13460877 | 1 | 0 |
| chr4 | 141821812 | T | A | 228 | Cela2a | exonic | synonymous SNV | Cela2a:NM_007919:exon4:c.A252T:p.R84R | . | | 4qE1 | rs13460878 | 1 | 0 |
| chr4 | 141822333 | G | A | 228 | Cela2a | intronic | . | . | . | | 4qE1 | rs220625709 | 1 | 0 |
| chr4 | 141822376 | T | C | 228 | Cela2a | intronic | . | . | . | | 4qE1 | rs247422339 | 1 | 0 |
| chr4 | 141825421 | A | G | 161 | Cela2a | intronic | . | . | . | | 4qE1 | rs8256397 | 1 | 0 |
| chr4 | 141825481 | A | C | 228 | Cela2a | intronic | . | . | . | | 4qE1 | rs8256392 | 1 | 0 |
| chr4 | 141825645 | G | A | 228 | Cela2a | intronic | . | . | . | | 4qE1 | rs8256375 | 1 | 0 |
| chr4 | 141825689 | T | C | 134 | Cela2a | intronic | . | . | . | | 4qE1 | rs8256369 | 1 | 0 |
| chr4 | 141825727 | C | T | 228 | Cela2a | intronic | . | . | . | | 4qE1 | rs8256365 | 1 | 0 |
| chr4 | 141838683 | A | G | 228 | Ctrc | exonic | unknown | UNKNOWN | . | | 4qE1 | rs255639458 | 1 | 0 |
| chr4 | 141838836 | G | T | 228 | Ctrc | intronic | . | . | . | | 4qE1 | rs215033547 | 1 | 0 |
| chr4 | 141838838 | C | T | 228 | Ctrc | intronic | . | . | . | | 4qE1 | rs238834285 | 1 | 0 |
| chr4 | 141840140 | G | T | 156 | Ctrc | intronic | . | . | . | | 4qE1 | rs251033136 | 1 | 0 |
| chr4 | 141840156 | G | C | 163 | Ctrc | intronic | . | . | . | | 4qE1 | rs265362901 | 1 | 0 |
| chr4 | 141840441 | G | T | 228 | Ctrc | intronic | . | . | . | | 4qE1 | rs228007791 | 1 | 0 |
| chr4 | 141840479 | T | C | 228 | Ctrc | intronic | . | . | . | | 4qE1 | rs27609213 | 1 | 0 |
| chr4 | 141841459 | C | G | 228 | Ctrc | intronic | . | . | . | | 4qE1 | rs32615448 | 1 | 0 |
| chr4 | 141841479 | T | A | 228 | Ctrc | intronic | . | . | . | | 4qE1 | rs246448167 | 1 | 0 |
| chr4 | 141841611 | A | G | 228 | Ctrc | exonic | unknown | UNKNOWN | . | | 4qE1 | rs13461198 | 1 | 0 |
| chr4 | 141841630 | G | A | 228 | Ctrc | exonic | unknown | UNKNOWN | . | | 4qE1 | rs224508850 | 1 | 0 |
| chr4 | 141843520 | G | A | 133 | Ctrc | intronic | . | . | . | | 4qE1 | rs223734688 | 1 | 0 |
| chr4 | 141843799 | A | G | 228 | Ctrc | splicing | . | . | . | | 4qE1 | rs245654447 | 1 | 0 |
| chr4 | 141843863 | G | A | 228 | Ctrc | intronic | . | . | . | | 4qE1 | rs255924154 | 1 | 0 |
| chr4 | 141844916 | C | T | 148 | Ctrc,Ctrcos | ncRNA_intronic | . | . | . | | 4qE1 | rs27609200 | 1 | 0 |
| chr4 | 141845170 | A | C | 228 | Ctrcos,Ctrc | ncRNA_intronic | . | . | . | | 4qE1 | rs216973816 | 1 | 0 |
| chr4 | 141845202 | G | A | 228 | Ctrc,Ctrcos | exonic | unknown | UNKNOWN | . | | 4qE1 | rs579948436 | 1 | 0 |
| chr4 | 141845287 | A | G | 228 | Ctrcos,Ctrc | ncRNA_intronic | . | . | . | | 4qE1 | rs236380762 | 1 | 0 |
| chr4 | 141846456 | G | A | 137 | Ctrcos | ncRNA_intronic | . | . | . | | 4qE1 | rs27609186 | 1 | 0 |
| chr4 | 141859679 | A | G | 178 | Efhd2 | UTR3 | . | . | . | | 4qE1 | . | 1 | 0 |
| chr4 | 141859969 | C | A | 228 | Efhd2 | intronic | . | . | . | | 4qE1 | rs27586309 | 1 | 0 |
| chr4 | 141860563 | G | A | 228 | Efhd2 | exonic | synonymous SNV | Efhd2:NM_025994:exon3:c.C576T:p.N192N | . | | 4qE1 | rs27586305 | 1 | 0 |
| chr4 | 141860768 | G | A | 228 | Efhd2 | intronic | . | . | . | | 4qE1 | rs27586304 | 1 | 0 |
| chr4 | 141860774 | A | G | 222 | Efhd2 | intronic | . | . | . | | 4qE1 | rs225250863 | 1 | 0 |
| chr4 | 141861766 | G | A | 212 | Efhd2 | intronic | . | . | . | | 4qE1 | rs249790035 | 1 | 0 |
| chr4 | 141861779 | T | C | 228 | Efhd2 | intronic | . | . | . | | 4qE1 | rs27586288 | 1 | 0 |
| chr4 | 141861889 | C | T | 228 | Efhd2 | exonic | synonymous SNV | Efhd2:NM_025994:exon2:c.G372A:p.L124L | . | | 4qE1 | rs240505916 | 1 | 0 |
| chr4 | 141861979 | C | A | 228 | Efhd2 | intronic | . | . | . | | 4qE1 | rs257467256 | 1 | 0 |
| chr4 | 141861989 | A | G | 228 | Efhd2 | intronic | . | . | . | | 4qE1 | rs27586286 | 1 | 0 |
| chr4 | 141862081 | C | T | 176 | Efhd2 | intronic | . | . | . | | 4qE1 | rs252438733 | 1 | 0 |
| chr4 | 141862136 | G | A | 118 | Efhd2 | intronic | . | . | . | | 4qE1 | rs218500944 | 1 | 0 |
| chr4 | 141874366 | T | C | 168 | Efhd2 | intronic | . | . | . | | 4qE1 | rs32508980 | 1 | 0 |
| chr4 | 141874667 | C | T | 228 | Efhd2 | exonic | synonymous SNV | Efhd2:NM_025994:exon1:c.G201A:p.Q67Q | 400 | | 4qE1 | rs27586221 | 1 | 0 |
| chr4 | 141890652 | G | A | 80 | Fhad1 | UTR3 | . | . | . | | 4qE1 | rs235916790 | 1 | 0 |
| chr4 | 141890719 | A | G | 167 | Fhad1 | UTR3 | . | . | . | | 4qE1 | rs586650006 | 1 | 0 |
| chr4 | 141890791 | C | T | 228 | Fhad1 | UTR3 | . | . | . | | 4qE1 | rs219896775 | 1 | 0 |
| chr4 | 141890961 | C | T | 228 | Fhad1 | exonic | missense SNV | Fhad1:NM_177868:exon32:c.G4153A:p.A1385T | . | | 4qE1 | rs237308841 | 1 | 0 |
| chr4 | 141890966 | G | A | 228 | Fhad1 | intronic | . | . | . | | 4qE1 | rs260217999 | 1 | 0 |
| chr4 | 141896635 | T | A | 150 | Fhad1 | intronic | . | . | . | | 4qE1 | rs49593198 | 1 | 0 |
| chr4 | 141899334 | G | T | 164 | Fhad1 | intronic | . | . | . | | 4qE1 | rs244441549 | 1 | 0 |
| chr4 | 141903839 | T | C | 219 | Fhad1 | intronic | . | . | . | | 4qE1 | rs33023203 | 1 | 0 |
| chr4 | 141904082 | T | C | 228 | Fhad1 | intronic | . | . | . | | 4qE1 | rs214882352 | 1 | 0 |
| chr4 | 141905014 | A | G | 228 | Fhad1 | exonic | synonymous SNV | Fhad1:NM_177868:exon28:c.T3813C:p.L1271L | . | | 4qE1 | rs108616000 | 1 | 0 |
| chr4 | 141905241 | T | C | 228 | Fhad1 | intronic | . | . | . | | 4qE1 | rs27586188 | 1 | 0 |
| chr4 | 141905271 | A | G | 228 | Fhad1 | intronic | . | . | . | | 4qE1 | rs27586187 | 1 | 0 |
| chr4 | 141905491 | T | C | 228 | Fhad1 | intronic | . | . | . | | 4qE1 | rs27586186 | 1 | 0 |
| chr4 | 141905518 | A | G | 228 | Fhad1 | intronic | . | . | . | | 4qE1 | rs253203874 | 1 | 0 |
| chr4 | 141905633 | T | C | 228 | Fhad1 | exonic | missense SNV | Fhad1:NM_177868:exon27:c.A3559G:p.S1187G | . | | 4qE1 | rs27586185 | 1 | 0 |
| chr4 | 141905689 | T | C | 228 | Fhad1 | intronic | . | . | . | | 4qE1 | rs217447528 | 1 | 0 |
| chr4 | 141905741 | G | A | 164 | Fhad1 | intronic | . | . | . | | 4qE1 | rs238462522 | 1 | 0 |
| chr4 | 141908716 | A | C | 177 | Fhad1 | intronic | . | . | . | | 4qE1 | rs27586160 | 1 | 0 |
| chr4 | 141908805 | C | T | 129 | Fhad1 | intronic | . | . | . | | 4qE1 | rs3680812 | 1 | 0 |
| chr4 | 141909093 | G | A | 228 | Fhad1 | exonic | synonymous SNV | Fhad1:NM_177868:exon26:c.C3435T:p.H1145H | . | | 4qE1 | rs3682673 | 1 | 0 |
| chr4 | 141909661 | G | A | 104 | Fhad1 | intronic | . | . | . | | 4qE1 | rs27586155 | 1 | 0 |
| chr4 | 141909749 | C | T | 228 | Fhad1 | exonic | synonymous SNV | Fhad1:NM_177868:exon25:c.G3405A:p.E1135E | . | | 4qE1 | rs27586154 | 1 | 0 |
| chr4 | 141909767 | C | A | 228 | Fhad1 | exonic | synonymous SNV | Fhad1:NM_177868:exon25:c.G3387T:p.T1129T | . | | 4qE1 | rs27586152 | 1 | 0 |
| chr4 | 141909880 | T | C | 228 | Fhad1 | intronic | . | . | . | | 4qE1 | rs27586151 | 1 | 0 |
| chr4 | 141918089 | T | G | 150 | Fhad1 | intronic | . | . | . | | 4qE1 | rs3665891 | 1 | 0 |
| chr4 | 141918098 | T | C | 163 | Fhad1 | intronic | . | . | . | | 4qE1 | rs27586111 | 1 | 0 |
| chr4 | 141918423 | A | C | 228 | Fhad1 | intronic | . | . | . | | 4qE1 | rs27586108 | 1 | 0 |
| chr4 | 141918574 | C | T | 228 | Fhad1 | intronic | . | . | . | | 4qE1 | rs27586107 | 1 | 0 |
| chr4 | 141918679 | C | T | 228 | Fhad1 | intronic | . | . | . | | 4qE1 | rs27586106 | 1 | 0 |
| chr4 | 141918815 | A | G | 228 | Fhad1 | exonic | missense SNV | Fhad1:NM_177868:exon21:c.T2830C:p.S944P | . | | 4qE1 | rs32411976 | 1 | 0 |
| chr4 | 141920715 | T | C | 228 | Fhad1 | intronic | . | . | . | | 4qE1 | rs216987183 | 1 | 0 |
| chr4 | 141920740 | T | G | 228 | Fhad1 | exonic | missense SNV | Fhad1:NM_177868:exon20:c.A2809C:p.T937P | . | | 4qE1 | rs236692753 | 1 | 0 |
| chr4 | 141920864 | T | C | 228 | Fhad1 | exonic | synonymous SNV | Fhad1:NM_177868:exon20:c.A2685G:p.S895S | . | | 4qE1 | rs27586083 | 1 | 0 |
| chr4 | 141922233 | C | G | 228 | Fhad1 | intronic | . | . | . | | 4qE1 | rs27586072 | 1 | 0 |
| chr4 | 141922367 | T | C | 228 | Fhad1 | exonic | synonymous SNV | Fhad1:NM_177868:exon19:c.A2463G:p.K821K | . | | 4qE1 | rs219838484 | 1 | 0 |
| chr4 | 141922388 | G | A | 228 | Fhad1 | exonic | synonymous SNV | Fhad1:NM_177868:exon19:c.C2442T:p.N814N | . | | 4qE1 | rs229672019 | 1 | 0 |
| chr4 | 141928347 | G | A | 228 | Fhad1 | intronic | . | . | . | | 4qE1 | rs224722985 | 1 | 0 |
| chr4 | 141928376 | C | T | 228 | Fhad1 | splicing | . | . | . | | 4qE1 | rs27608024 | 1 | 0 |
| chr4 | 141929435 | C | A | 104 | Fhad1 | intronic | . | . | . | | 4qE1 | rs6251394 | 1 | 0 |
| chr4 | 141929508 | G | A | 177 | Fhad1 | intronic | . | . | . | | 4qE1 | rs6264089 | 1 | 0 |
| chr4 | 141932788 | T | C | 228 | Fhad1 | intronic | . | . | . | | 4qE1 | rs32891998 | 1 | 0 |
| chr4 | 141939147 | G | A | 228 | Fhad1 | exonic | synonymous SNV | Fhad1:NM_177868:exon15:c.C1965T:p.N655N | . | | 4qE1 | rs27607976 | 1 | 0 |
| chr4 | 141939165 | A | G | 228 | Fhad1 | intronic | . | . | . | | 4qE1 | rs27607975 | 1 | 0 |
| chr4 | 141939201 | T | C | 208 | Fhad1 | intronic | . | . | . | | 4qE1 | rs27607974 | 1 | 0 |
| chr4 | 141939832 | T | G | 135 | Fhad1 | intronic | . | . | . | | 4qE1 | rs27607972 | 1 | 0 |
| chr4 | 141939907 | C | T | 166 | Fhad1 | intronic | . | . | . | | 4qE1 | rs27607971 | 1 | 0 |
| chr4 | 141940063 | A | T | 228 | Fhad1 | exonic | missense SNV | Fhad1:NM_177868:exon14:c.T1947A:p.D649E | . | | 4qE1 | rs27607970 | 1 | 0 |
| chr4 | 141940149 | G | A | 228 | Fhad1 | intronic | . | . | . | | 4qE1 | rs216654894 | 1 | 0 |
| chr4 | 141951474 | T | C | 228 | Fhad1 | intronic | . | . | . | | 4qE1 | rs27584508 | 1 | 0 |
| chr4 | 141951682 | A | G | 228 | Fhad1 | intronic | . | . | . | | 4qE1 | rs27584507 | 1 | 0 |
| chr4 | 141951723 | T | C | 228 | Fhad1 | intronic | . | . | . | | 4qE1 | rs219729576 | 1 | 0 |

| | | | | | | | | | | | | | |
|---|---|---|---|---|---|---|---|---|---|---|---|---|---|
| chr4 | 141955108 | T | A | 174 | Fhad1 | intronic | . | . | . | 4qE1 | rs27584492 | 1 | 0 |
| chr4 | 141955244 | G | A | 228 | Fhad1 | intronic | . | . | . | 4qE1 | rs27584491 | 1 | 0 |
| chr4 | 141957356 | G | C | 164 | Fhad1 | intronic | . | . | . | 4qE1 | rs254225660 | 1 | 0 |
| chr4 | 141957509 | T | C | 228 | Fhad1 | intronic | . | . | . | 4qE1 | rs50325125 | 1 | 0 |
| chr4 | 141957745 | T | C | 228 | Fhad1 | intronic | . | . | . | 4qE1 | rs245825761 | 1 | 0 |
| chr4 | 141963209 | T | G | 154 | Fhad1 | intronic | . | . | . | 4qE1 | rs223059315 | 1 | 0 |
| chr4 | 141963219 | G | A | 162 | Fhad1 | intronic | . | . | . | 4qE1 | rs241028776 | 1 | 0 |
| chr4 | 141963278 | C | A | 188 | Fhad1 | intronic | . | . | . | 4qE1 | rs261442428 | 1 | 0 |
| chr4 | 141964479 | T | C | 228 | Fhad1 | intronic | . | . | . | 4qE1 | rs218415010 | 1 | 0 |
| chr4 | 141964775 | C | T | 164 | Fhad1 | intronic | . | . | . | 4qE1 | rs228156492 | 1 | 0 |
| chr4 | 141972706 | C | G | 157 | Fhad1 | intronic | . | . | . | 4qE1 | rs27584443 | 1 | 0 |
| chr4 | 141972707 | A | T | 157 | Fhad1 | intronic | . | . | . | 4qE1 | rs256178547 | 1 | 0 |
| chr4 | 141972919 | C | T | 228 | Fhad1 | exonic | missense SNV | Fhad1:NM_177868:exon7:c.G1019A:p.G340E | . | 4qE1 | rs27584442 | 1 | 0 |
| chr4 | 141972991 | C | T | 228 | Fhad1 | intronic | . | . | . | 4qE1 | rs27584441 | 1 | 0 |
| chr4 | 141973081 | T | C | 154 | Fhad1 | intronic | . | . | . | 4qE1 | rs27584440 | 1 | 0 |
| chr4 | 141973105 | G | A | 131 | Fhad1 | intronic | . | . | . | 4qE1 | rs27584439 | 1 | 0 |
| chr4 | 141973147 | T | C | 97 | Fhad1 | intronic | . | . | . | 4qE1 | rs27584437 | 1 | 0 |
| chr4 | 141974426 | G | C | 228 | Fhad1 | intronic | . | . | . | 4qE1 | rs27584426 | 1 | 0 |
| chr4 | 141974454 | T | C | 228 | Fhad1 | intronic | . | . | . | 4qE1 | rs27584425 | 1 | 0 |
| chr4 | 141982113 | T | C | 185 | Fhad1 | intronic | . | . | . | 4qE1 | rs229494769 | 1 | 0 |
| chr4 | 141982118 | C | A | 185 | Fhad1 | intronic | . | . | . | 4qE1 | rs27584390 | 1 | 0 |
| chr4 | 141982240 | C | A | 228 | Fhad1 | exonic | missense SNV | Fhad1:NM_177868:exon5:c.G802T:p.A268S | . | 4qE1 | rs27584389 | 1 | 0 |
| chr4 | 141982411 | G | A | 228 | Fhad1 | intronic | . | . | . | 4qE1 | rs232925124 | 1 | 0 |
| chr4 | 141982424 | T | C | 228 | Fhad1 | intronic | . | . | . | 4qE1 | rs246017141 | 1 | 0 |
| chr4 | 141982527 | A | T | 163 | Fhad1 | intronic | . | . | . | 4qE1 | rs27584388 | 1 | 0 |
| chr4 | 142011361 | C | T | 170 | Fhad1 | intronic | . | . | . | 4qE1 | rs27584308 | 1 | 0 |
| chr4 | 142011474 | C | T | 228 | Fhad1 | intronic | . | . | . | 4qE1 | rs249347832 | 1 | 0 |
| chr4 | 142011475 | A | G | 228 | Fhad1 | intronic | . | . | . | 4qE1 | rs262445934 | 1 | 0 |
| chr4 | 142011560 | T | C | 228 | Fhad1 | exonic | synonymous SNV | Fhad1:NM_177868:exon1:c.A78G:p.S26S | . | 4qE1 | rs27584306 | 1 | 0 |
| chr4 | 142011611 | A | G | 228 | Fhad1 | exonic | synonymous SNV | Fhad1:NM_177868:exon1:c.T27C:p.D9D | . | 4qE1 | rs27584305 | 1 | 0 |
| chr4 | 142011747 | G | A | 167 | . | upstream | . | . | . | 4qE1 | rs27584304 | 1 | 0 |
| chr4 | 142032079 | C | T | 228 | Tmem51 | exonic | synonymous SNV | Tmem51:NM_145402:exon3:c.G357A:p.E119E | . | 4qE1 | rs241380340 | 1 | 0 |
| chr4 | 142032179 | T | C | 228 | Tmem51 | intronic | . | . | . | 4qE1 | rs27567679 | 1 | 0 |
| chr4 | 142032207 | C | T | 195 | Tmem51 | intronic | . | . | . | 4qE1 | rs27567678 | 1 | 0 |
| chr4 | 142037295 | G | A | 103 | Tmem51 | intronic | . | . | . | 4qE1 | rs27567637 | 1 | 0 |
| chr4 | 142037375 | G | A | 217 | Tmem51 | intronic | . | . | . | 4qE1 | rs27567633 | 1 | 0 |
| chr4 | 142086336 | A | G | 228 | Tmem51os1 | ncRNA_intronic | . | . | . | 4qE1 | rs27552329 | 1 | 0 |
| chr4 | 142086453 | A | G | 228 | Tmem51os1 | ncRNA_exonic | . | . | . | 4qE1 | rs27552326 | 1 | 0 |
| chr4 | 142086934 | C | T | 228 | Tmem51os1 | ncRNA_exonic | . | . | . | 4qE1 | rs27552325 | 1 | 0 |
| chr4 | 142087026 | T | C | 228 | Tmem51os1 | ncRNA_exonic | . | . | . | 4qE1 | rs27552324 | 1 | 0 |
| chr4 | 142108461 | A | G | 196 | Kazn | intronic | . | . | . | 4qE1 | rs27552212 | 1 | 0 |
| chr4 | 142108641 | G | A | 228 | Kazn | exonic | unknown | UNKNOWN | . | 4qE1 | rs27552211 | 1 | 0 |
| chr4 | 142108650 | T | C | 228 | Kazn | exonic | unknown | UNKNOWN | . | 4qE1 | rs27552210 | 1 | 0 |
| chr4 | 142108686 | G | A | 228 | Kazn | exonic | unknown | UNKNOWN | . | 4qE1 | rs27552208 | 1 | 0 |
| chr4 | 142109199 | C | A | 151 | Kazn | intronic | . | . | . | 4qE1 | rs257261148 | 1 | 0 |
| chr4 | 142109212 | G | T | 219 | Kazn | intronic | . | . | . | 4qE1 | rs227921790 | 1 | 0 |
| chr4 | 142109226 | C | T | 228 | Kazn | intronic | . | . | . | 4qE1 | rs27552207 | 1 | 0 |
| chr4 | 142112055 | T | G | 186 | Kazn | intronic | . | . | . | 4qE1 | rs27552199 | 1 | 0 |
| chr4 | 142112093 | C | T | 228 | Kazn | intronic | . | . | . | 4qE1 | rs237987782 | 1 | 0 |
| chr4 | 142112237 | A | G | 228 | Kazn | exonic | unknown | UNKNOWN | . | 4qE1 | rs27552198 | 1 | 0 |
| chr4 | 142117071 | T | C | 228 | Kazn | intronic | . | . | . | 4qE1 | rs27552171 | 1 | 0 |
| chr4 | 142117231 | C | G | 228 | Kazn | intronic | . | . | . | 4qE1 | rs27552170 | 1 | 0 |
| chr4 | 142117309 | G | C | 102 | Kazn | intronic | . | . | . | 4qE1 | rs27552168 | 1 | 0 |
| chr4 | 142118171 | C | T | 228 | Kazn | exonic | unknown | UNKNOWN | . | 4qE1 | rs27637451 | 1 | 0 |
| chr4 | 142141900 | G | C | 228 | Kazn | intronic | . | . | . | 4qE1 | rs32563708 | 1 | 0 |
| chr4 | 142141901 | G | A | 228 | Kazn | intronic | . | . | . | 4qE1 | rs227760498 | 1 | 0 |
| chr4 | 142141966 | C | T | 142 | Kazn | intronic | . | . | . | 4qE1 | rs27622898 | 1 | 0 |
| chr4 | 142143190 | C | T | 228 | Kazn | intronic | . | . | . | 4qE1 | rs27622891 | 1 | 0 |
| chr4 | 142143253 | G | C | 228 | Kazn | intronic | . | . | . | 4qE1 | rs259429406 | 1 | 0 |
| chr4 | 142146901 | C | T | 176 | Kazn | intronic | . | . | . | 4qE1 | rs27622868 | 1 | 0 |
| chr4 | 142147263 | C | T | 228 | Kazn | intronic | . | . | . | 4qE1 | rs27622867 | 1 | 0 |
| chr4 | 142150874 | T | G | 170 | Kazn | exonic | synonymous SNV | Kazn:NM_001109685:exon5:c.A870C:p.S290S | . | 4qE1 | rs47959795 | 1 | 0 |
| chr4 | 142151048 | C | T | 228 | Kazn | intronic | . | . | . | 4qE1 | rs27622839 | 1 | 0 |
| chr4 | 142151198 | G | T | 128 | Kazn | intronic | . | . | . | 4qE1 | rs27622837 | 1 | 0 |
| chr4 | 142159059 | A | G | 198 | Kazn | exonic | synonymous SNV | Kazn:NM_001109685:exon3:c.T522C:p.Y174Y | . | 4qE1 | rs27622794 | 1 | 0 |
| chr4 | 142159209 | C | T | 228 | Kazn | intronic | . | . | . | 4qE1 | rs256292960 | 1 | 0 |
| chr4 | 142159225 | A | G | 228 | Kazn | intronic | . | . | . | 4qE1 | rs27622793 | 1 | 0 |
| chr4 | 142159340 | T | A | 151 | Kazn | intronic | . | . | . | 4qE1 | rs27622791 | 1 | 0 |
| chr4 | 142189101 | A | G | 95 | Kazn | intronic | . | . | . | 4qE1 | rs27607892 | 1 | 0 |
| chr4 | 142189270 | C | T | 228 | Kazn | intronic | . | . | . | 4qE1 | rs27607891 | 1 | 0 |
| chr4 | 142189290 | T | C | 228 | Kazn | intronic | . | . | . | 4qE1 | rs215306862 | 1 | 0 |
| chr4 | 142210009 | A | G | 159 | Kazn | intronic | . | . | . | 4qE1 | rs27607806 | 1 | 0 |
| chr4 | 142210154 | C | T | 228 | Kazn | exonic | synonymous SNV | Kazn:NM_001109685:exon2:c.G261A:p.R87R | . | 4qE1 | rs27607805 | 1 | 0 |
| chr4 | 143389990 | G | A | 221 | . | intergenic | . | . | . | 4qE1 | . | 0 | 1 |
| chr4 | 143499536 | A | G | 228 | . | intergenic | . | . | . | 4qE1 | . | 1 | 0 |
| chr4 | 144247856 | T | C | 117 | . | intergenic | . | . | . | 4qE1 | . | 0 | 1 |
| chr4 | 144394579 | A | C | 123 | Pramef12 | exonic | missense SNV | Pramef12:NM_029948:exon3:c.T874G:p.F292V | . | 4qE1 | . | 0 | 1 |
| chr4 | 144456354 | A | G | 221 | Aadacl3 | exonic | synonymous SNV | Aadacl3:NM_001085503:exon4:c.T543C:p.Y181Y | . | 4qE1 | . | 0 | 1 |
| chr4 | 144522475 | A | C | 52 | 9430007A20Rik | intronic | . | . | . | 4qE1 | . | 0 | 1 |
| chr4 | 145065717 | T | A | 228 | Vps13d | intronic | . | . | . | 4qE1 | . | 1 | 0 |
| chr4 | 145070269 | C | G | 37.7223 | Vps13d | intronic | . | . | . | 4qE1 | . | 0 | 1 |
| chr4 | 145155974 | A | C | 221 | Vps13d | exonic | missense SNV | Vps13d:NM_001276502:exon18:c.T3048G:p.D1016E,Vps13d:NM_001276465:exon19:c.T3048G:p.D1016E | . | 4qE1 | . | 0 | 1 |
| chr4 | 145160839 | C | T | 100 | Vps13d | intronic | . | . | . | 4qE1 | . | 0 | 1 |
| chr4 | 145222830 | T | C | 70 | Tnfrsf1b | intronic | . | . | . | 4qE1 | . | 0 | 1 |
| chr4 | 147510773 | T | C | 222 | Gm13152 | exonic | synonymous SNV | Gm13152:NM_001039209:exon2:c.T51C:p.D17D | . | 4qE1 | rs27633869 | 0 | 1 |
| chr4 | 147512322 | T | C | 222 | Gm13152 | intronic | . | . | . | 4qE1 | rs48534157 | 0 | 1 |
| chr4 | 147527776 | C | T | 222 | . | intergenic | . | . | . | 4qE1 | . | 0 | 1 |
| chr4 | 147527929 | G | C | 110 | . | intergenic | . | . | . | 4qE1 | rs27633825 | 0 | 1 |
| chr4 | 147528000 | C | G | 222 | . | intergenic | . | . | . | 4qE1 | rs49994546 | 0 | 1 |
| chr4 | 147528003 | G | A | 156 | . | intergenic | . | . | . | 4qE1 | rs49414817 | 0 | 1 |
| chr4 | 147528062 | T | C | 222 | . | intergenic | . | . | . | 4qE1 | . | 0 | 1 |
| chr4 | 147528193 | G | A | 187 | . | intergenic | . | . | . | 4qE1 | rs51554430 | 0 | 1 |
| chr4 | 147528198 | C | T | 222 | . | intergenic | . | . | . | 4qE1 | rs27633823 | 0 | 1 |
| chr4 | 147528268 | T | G | 181 | . | intergenic | . | . | . | 4qE1 | rs46492652 | 0 | 1 |
| chr4 | 147528328 | T | C | 41.4017 | . | intergenic | . | . | . | 4qE1 | rs48725714 | 0 | 1 |
| chr4 | 147528418 | A | G | 31.2422 | . | intergenic | . | . | . | 4qE1 | rs50127575 | 0 | 1 |
| chr4 | 147821719 | A | G | 74 | . | downstream | . | . | . | 4qE1 | rs236414896 | 0 | 1 |
| chr4 | 148052043 | G | T | 208 | Mthfr | intronic | . | . | . | 4qE2 | . | 0 | 1 |
| chr4 | 148083761 | C | A | 158 | Agtrap | intronic | . | . | . | 4qE2 | . | 1 | 0 |
| chr4 | 148108230 | T | A | 23.0118 | Draxin | intronic | . | . | . | 4qE2 | . | 0 | 1 |
| chr4 | 148153429 | C | A | 228 | Fbxo44 | UTR3 | . | . | . | 4qE2 | . | 1 | 0 |
| chr4 | 148243215 | C | T | 126 | Ptchd2 | intronic | . | . | . | 4qE2 | . | 1 | 0 |
| chr4 | 148569401 | C | A | 228 | Exosc10 | intronic | . | . | . | 4qE2 | . | 1 | 0 |
| chr4 | 148928385 | C | A | 228 | Casz1 | intronic | . | . | . | 4qE2 | . | 1 | 0 |
| chr4 | 148944359 | T | C | 228 | Casz1 | exonic | missense SNV | Casz1:NM_001159344:exon16:c.T3260C:p.L1087P,Casz1:NM_027195:exon16:c.T3260C:p.L1087P | . | 4qE2 | rs49392164 | 1 | 0 |
| chr4 | 149127263 | G | A | 171 | . | upstream | . | . | . | 4qE2 | . | 0 | 1 |
| chr4 | 149386944 | A | T | 126 | Ube4b | intronic | . | . | . | 4qE2 | . | 0 | 1 |
| chr4 | 149486906 | C | T | 222 | Lzic | intronic | . | . | . | 4qE2 | . | 0 | 1 |
| chr4 | 150615791 | A | G | 67 | Rere | exonic | missense SNV | Rere:NM_001085492:exon18:c.A3331G:p.T1111A | . | 4qE2 | . | 0 | 1 |
| chr4 | 151050821 | T | C | 228 | Vamp3 | intronic | . | . | . | 4qE2 | . | 1 | 0 |
| chr4 | 151586342 | C | A | 228 | Camta1 | intronic | . | . | . | 4qE2 | . | 1 | 0 |
| chr4 | 153432590 | C | A | 134 | Ajap1 | exonic | missense SNV | Ajap1:NM_001099299:exon2:c.G293T:p.R98L | 257 | 4qE2 | . | 0 | 1 |
| chr4 | 153985324 | C | T | 228 | Cep104 | exonic | missense SNV | Cep104:NM_177673:exon8:c.C754T:p.R252C | . | 4qE2 | . | 1 | 0 |
| chr4 | 154006874 | G | T | 228 | Cep104 | UTR3 | . | . | . | 4qE2 | . | 1 | 0 |
| chr4 | 154262670 | C | A | 228 | Megf6 | intronic | . | . | . | 4qE2 | . | 1 | 0 |
| chr4 | 154890773 | A | G | 228 | Mmel1 | intronic | . | . | . | 4qE2 | . | 1 | 0 |
| chr4 | 155272925 | C | G | 228 | Prkcz | intronic | . | . | . | 4qE2 | . | 1 | 0 |
| chr4 | 155533915 | G | A | 104 | Gnb1 | intronic | . | . | . | 4qE2 | . | 0 | 1 |
| chr4 | 155839836 | G | T | 84 | Mxra8 | UTR5 | . | . | . | 4qE2 | . | 0 | 1 |
| chr4 | 155855159 | G | T | 228 | Dvl1 | exonic | missense SNV | Dvl1:NM_010091:exon9:c.G965T:p.R322L,Dvl1:NM_001302342:exon10:c.G965T:p.R322L | . | 4qE2 | . | 1 | 0 |
| chr4 | 155904897 | G | A | 221 | Acap3 | exonic | unknown | UNKNOWN | . | 4qE2 | . | 0 | 1 |
| chr4 | 155957234 | G | T | 228 | Gm5801,Ube2j2 | exonic | missense SNV | Ube2j2:NM_001284314:exon6:c.G568T:p.A190S,Ube2j2:NM_001039158:exon7:c.G649T:p.A217S,Ube2j2:NM | . | 4qE2 | . | 1 | 0 |
| chr4 | 156214512 | C | T | 180 | . | intergenic | . | . | 277 | 4qE2 | . | 0 | 1 |
| chr4 | 156342100 | G | T | 45.7003 | . | intergenic | . | . | . | 4qE2 | . | 0 | 1 |
| chr4 | 156342942 | G | T | 23.0118 | . | intergenic | . | . | . | 4qE2 | rs584761221 | 0 | 1 |
| chr5 | 3629763 | A | C | 73 | Pex1 | intronic | . | . | . | 5qA1 | . | 0 | 1 |
| chr5 | 3956187 | C | T | 222 | Akap9 | exonic | stopgain | Akap9:NM_194462:exon5:c.C511T:p.R171X | . | 5qA1 | . | 0 | 1 |
| chr5 | 5455858 | A | T | 123 | 1700015F17Rik | intronic | . | . | . | 5qA1 | . | 0 | 1 |
| chr5 | 5782334 | T | C | 221 | . | intergenic | . | . | . | 5qA1 | rs107944843 | 0 | 1 |
| chr5 | 5782755 | G | A | 222 | . | intergenic | . | . | . | 5qA1 | . | 0 | 1 |
| chr5 | 5783565 | C | T | 221 | . | intergenic | . | . | . | 5qA1 | rs578527625 | 0 | 1 |
| chr5 | 6771287 | T | G | 221 | Zfp804b | exonic | missense SNV | Zfp804b:NM_001163223:exon4:c.A1775C:p.N592T | . | 5qA1 | . | 0 | 1 |
| chr5 | 6772384 | T | C | 221 | Zfp804b | exonic | synonymous SNV | Zfp804b:NM_001163223:exon4:c.A678G:p.K226K | . | 5qA1 | . | 0 | 1 |
| chr5 | 6772389 | T | C | 221 | Zfp804b | exonic | missense SNV | Zfp804b:NM_001163223:exon4:c.A673G:p.K225E | . | 5qA1 | . | 0 | 1 |
| chr5 | 6876357 | G | T | 222 | Zfp804b | intronic | . | . | . | 5qA1 | . | 0 | 1 |
| chr5 | 7177750 | G | C | 24.566 | Zfp804b | intronic | . | . | . | 5qA1 | . | 0 | 1 |
| chr5 | 7304759 | T | A | 79 | 4921511H03Rik,Zfp | UTR5 | . | . | . | 5qA1 | . | 0 | 1 |
| chr5 | 8132679 | A | C | 37.7106 | Adam22 | intronic | . | . | . | 5qA1 | . | 0 | 1 |
| chr5 | 8422468 | C | A | 222 | Dbf4 | exonic | synonymous SNV | Dbf4:NM_001190717:exon1:c.G45T:p.P15P,Dbf4:NM_013726:exon1:c.G45T:p.P15P | 1027 | 5qA1 | . | 0 | 1 |
| chr5 | 8821429 | G | T | 222 | Abcb1b | exonic | stopgain | Abcb1b:NM_011075:exon9:c.G982T:p.G328X | . | 5qA1 | . | 0 | 1 |
| chr5 | 8835000 | T | A | 210 | Abcb1b | intronic | . | . | . | 5qA1 | . | 0 | 1 |
| chr5 | 8914005 | G | C | 221 | Abcb4 | intronic | . | . | . | 5qA1 | . | 0 | 1 |
| chr5 | 9399115 | A | T | 81 | 9330182L06Rik | intronic | . | . | . | 5qA1 | . | 0 | 1 |
| chr5 | 9410590 | T | G | 210 | 9330182L06Rik | exonic | synonymous SNV | 9330182L06Rik:NM_172706:exon4:c.T510G:p.S170S | . | 5qA1 | . | 0 | 1 |
| chr5 | 13570208 | A | G | 228 | Sema3a | exonic | missense SNV | Sema3a:NM_009152:exon12:c.A1423G:p.I475V,Sema3a:NM_001243072:exon13:c.A1423G:p.I475V,Sema3a:N | . | 5qA1 | rs46532600 | 1 | 0 |
| chr5 | 14540696 | A | C | 222 | Pclo | exonic | missense SNV | Pclo:NM_001110796:exon3:c.A3009C:p.E1003D,Pclo:NM_011995:exon3:c.A3009C:p.E1003D | . | 5qA1 | . | 0 | 1 |
| chr5 | 14679503 | G | C | 221 | Pclo | exonic | missense SNV | Pclo:NM_001110796:exon5:c.G8374C:p.V2792L,Pclo:NM_011995:exon5:c.G8374C:p.V2792L | . | 5qA1 | . | 0 | 1 |
| chr5 | 14681543 | A | G | 221 | Pclo | exonic | missense SNV | Pclo:NM_001110796:exon6:c.A10058G:p.D3353G,Pclo:NM_011995:exon6:c.A10058G:p.D3353G | . | 5qA1 | . | 0 | 1 |
| chr5 | 14681875 | A | G | 221 | Pclo | exonic | missense SNV | Pclo:NM_001110796:exon6:c.A10390G:p.R3464G,Pclo:NM_011995:exon6:c.A10390G:p.R3464G | . | 5qA1 | . | 0 | 1 |
| chr5 | 16163258 | A | G | 23.5223 | Cacna2d1 | intronic | . | . | . | 5qA2 | . | 0 | 1 |
| chr5 | 16730563 | A | G | 172 | . | intergenic | . | . | . | 5qA3 | . | 0 | 1 |
| chr5 | 17346026 | A | T | 26.8684 | . | intergenic | . | . | . | 5qA3 | . | 0 | 1 |
| chr5 | 17711195 | C | G | 48.7322 | Sema3c | intronic | . | . | . | 5qA3 | . | 0 | 1 |
| chr5 | 17724136 | C | T | 222 | Sema3c | exonic | synonymous SNV | Sema3c:NM_013657:exon17:c.C1767T:p.F589F | . | 5qA3 | . | 0 | 1 |
| chr5 | 17727243 | T | C | 106 | Sema3c | intronic | . | . | . | 5qA3 | . | 0 | 1 |
| chr5 | 20998681 | C | T | 222 | Ptpn12 | exonic | synonymous SNV | Ptpn12:NM_011203:exon13:c.G1098A:p.T366T | . | 5qA3 | . | 0 | 1 |
| chr5 | 21847213 | T | A | 222 | Slc26a5 | exonic | synonymous SNV | Slc26a5:NM_001289787:exon2:c.A117T:p.T39T,Slc26a5:NM_030727:exon3:c.A117T:p.T39T | . | 5qA3 | . | 0 | 1 |
| chr5 | 22048529 | C | T | 65 | Reln | intronic | . | . | . | 5qA3 | . | 0 | 1 |
| chr5 | 22154899 | G | A | 94 | Reln | intronic | . | . | . | 5qA3 | . | 0 | 1 |
| chr5 | 22533660 | A | G | 127 | Orc5 | intronic | . | . | . | 5qA3 | . | 0 | 1 |
| chr5 | 22746946 | G | A | 116 | Lhfpl3 | intronic | . | . | . | 5qA3 | . | 0 | 1 |
| chr5 | 24325455 | C | T | 116 | Kcnh2 | intronic | . | . | . | 5qA3 | . | 0 | 1 |
| chr5 | 24543785 | T | G | 75 | 4931409K22Rik | intronic | . | . | . | 5qA3 | . | 0 | 1 |
| chr5 | 24546927 | G | A | 176 | 4931409K22Rik | exonic | missense SNV | 4931409K22Rik:NM_177676:exon13:c.C1472T:p.S491F | . | 5qA3 | . | 0 | 1 |
| chr5 | 25022095 | T | C | 140 | Prkag2 | intronic | . | . | . | 5qA3 | . | 0 | 1 |
| chr5 | 25217427 | G | T | 122 | Galntl5 | intronic | . | . | . | 5qA3 | rs223589603 | 0 | 1 |
| chr5 | 27557609 | G | T | 222 | Dpp6 | exonic | synonymous SNV | Dpp6:NM_001136060:exon7:c.G552T:p.G184G,Dpp6:NM_001198886:exon7:c.G546T:p.G182G,Dpp6:NM_01007 | . | 5qB1 | . | 0 | 1 |
| chr5 | 27860961 | A | T | 171 | . | intergenic | . | . | . | 5qB1 | . | 0 | 1 |
| chr5 | 28413168 | G | C | 95 | Rbm33 | intronic | . | . | . | 5qB1 | . | 0 | 1 |
| chr5 | 30926814 | C | T | 222 | Khk | exonic | synonymous SNV | Khk:NM_001310524:exon3:c.C336T:p.H112H | . | 5qB1 | . | 0 | 1 |
| chr5 | 31040019 | T | C | 43.9904 | Slc5a6 | intronic | . | . | . | 5qB1 | . | 0 | 1 |
| chr5 | 31061237 | G | T | 222 | Cad | exonic | missense SNV | Cad:NM_001289522:exon11:c.G1507T:p.G503C,Cad:NM_001289523:exon11:c.G1507T:p.G503C,Cad:NM_023 | . | 5qB1 | . | 0 | 1 |
| chr5 | 31074097 | G | T | 222 | Cad | exonic | missense SNV | Cad:NM_001289522:exon32:c.G5162T:p.G1721V,Cad:NM_001289523:exon32:c.G5162T:p.G1721V,Cad:NM_02 | . | 5qB1 | . | 0 | 1 |
| chr5 | 31300095 | C | A | 221 | Gckr | intronic | . | . | . | 5qB1 | . | 0 | 1 |
| chr5 | 31614505 | T | A | 104 | Mrpl33 | intronic | . | . | . | 5qB1 | . | 0 | 1 |
| chr5 | 32659303 | G | T | 89 | Yes1 | intronic | . | . | . | 5qB1 | . | 0 | 1 |
| chr5 | 33391873 | G | T | 222 | Uvssa | intronic | . | . | . | 5qB1 | . | 0 | 1 |
| chr5 | 34118702 | C | G | 116 | Poln | intronic | . | . | . | 5qB2 | . | 0 | 1 |
| chr5 | 34133152 | C | A | 222 | 4930557I02Rik,Pol | exonic | missense SNV | Poln:NM_001289803:exon3:c.G178T:p.V60L,Poln:NM_181857:exon3:c.G178T:p.V60L,Poln:NM_001289804: | . | 5qB2 | . | 0 | 1 |
| chr5 | 34168354 | A | G | 222 | Haus3,Poln | UTR5 | . | . | . | 5qB2 | . | 0 | 1 |
| chr5 | 34195835 | G | T | 186 | Zfyve28 | UTR3 | . | . | . | 5qB2 | . | 0 | 1 |
| chr5 | 34854888 | G | A | 180 | Htt | intronic | . | . | . | 5qB2 | . | 0 | 1 |
| chr5 | 36031151 | C | G | 107 | Sorcs2 | splicing | . | . | . | 5qB3 | . | 0 | 1 |
| chr5 | 36813002 | C | T | 185 | Man2b2 | exonic | synonymous SNV | Man2b2:NM_008550:exon15:c.G2559A:p.Q853Q | . | 5qB3 | . | 0 | 1 |
| chr5 | 36967737 | C | G | 222 | Wfs1 | exonic | synonymous SNV | Wfs1:NM_011716:exon8:c.G1809C:p.T603T | . | 5qB3 | . | 0 | 1 |

| | | | | | | | | | | | | | |
|---|---|---|---|---|---|---|---|---|---|---|---|---|---|
| chr5 | 37125090 | T | A | 96 | Jakmip1 | UTR3 | . | . | . | 5qB3 | . | 0 | 1 |
| chr5 | 37320549 | C | G | 128 | Evc | intronic | . | . | . | 5qB3 | . | 0 | 1 |
| chr5 | 37735624 | C | G | 222 | Cytl1 | exonic | missense SNV | Cytl1:NM_001081106:exon1:c.C77G:p.A26G | . | 5qB3 | . | 0 | 1 |
| chr5 | 38260635 | C | A | 85 | Tmem128 | intronic | . | . | . | 5qB3 | rs48873236 | 1 | 0 |
| chr5 | 38300085 | G | C | 228 | Otop1 | exonic | missense SNV | Otop1:NM_172709:exon5:c.G1187C:p.G396A | . | 5qB3 | rs49325632 | 1 | 0 |
| chr5 | 38768007 | G | A | 130 | Clnk | intronic | . | . | . | 5qB3 | . | 0 | 1 |
| chr5 | 38769716 | A | G | 195 | Clnk | intronic | . | . | . | 5qB3 | . | 0 | 1 |
| chr5 | 43668634 | A | G | 228 | Cc2d2a | intronic | . | . | . | 5qB3 | rs46835784 | 1 | 0 |
| chr5 | 44045488 | A | T | 222 | Prom1 | intronic | . | . | . | 5qB3 | . | 0 | 1 |
| chr5 | 44479158 | T | C | 222 | Ldb2 | exonic | missense SNV | Ldb2:NM_001077398:exon7:c.A826G:p.T276A,Ldb2:NM_001286348:exon7:c.A826G:p.T276A,Ldb2:NM_0106 | . | 5qB3 | . | 0 | 1 |
| chr5 | 45507026 | C | T | 100 | Lap3 | intronic | . | . | . | 5qB3 | . | 0 | 1 |
| chr5 | 45584054 | C | T | 221 | Fam184b | exonic | synonymous SNV | Fam184b:NM_021416:exon2:c.G834A:p.G278G | . | 5qB3 | . | 0 | 1 |
| chr5 | 48249968 | T | C | 111 | Slit2 | intronic | . | . | . | 5qB3 | . | 0 | 1 |
| chr5 | 52368807 | G | C | 157 | Sod3 | UTR3 | . | . | 619 | 5qC1 | . | 0 | 1 |
| chr5 | 53889309 | A | G | 228 | Tbc1d19 | intronic | . | . | . | 5qC1 | rs29502300 | 1 | 0 |
| chr5 | 54358501 | T | C | 158 | Gm10440 | ncRNA_exonic | . | . | . | 5qC1 | . | 0 | 1 |
| chr5 | 57719746 | G | A | 56 | Pcdh7 | exonic | synonymous SNV | Pcdh7:NM_001122758:exon1:c.G642A:p.A214A,Pcdh7:NM_001310606:exon1:c.G642A:p.A214A,Pcdh7:NM_0( | 3261 | 5qC1 | . | 0 | 1 |
| chr5 | 61810446 | T | A | 221 | G6pd2 | UTR3 | . | . | . | 5qC3.1 | . | 0 | 1 |
| chr5 | 62004632 | T | C | 182 | . | intergenic | . | . | . | 5qC3.1 | . | 0 | 1 |
| chr5 | 64345331 | C | T | 133 | Tbc1d1 | exonic | missense SNV | Tbc1d1:NM_001289514:exon18:c.C2807T:p.S936F,Tbc1d1:NM_019636:exon19:c.C3194T:p.S1065F,Tbc1d1: | . | 5qC3.1 | . | 0 | 1 |
| chr5 | 65231359 | G | C | 222 | Wdr19 | exonic | missense SNV | Wdr19:NM_153391:exon19:c.G2224C:p.A742P | . | 5qC3.1 | . | 0 | 1 |
| chr5 | 66284193 | A | G | 192 | Nsun7 | exonic | missense SNV | Nsun7:NM_027602:exon8:c.A1204G:p.S402G,Nsun7:NM_001302833:exon9:c.A1204G:p.S402G | . | 5qC3.1 | . | 0 | 1 |
| chr5 | 66953738 | A | G | 222 | Limch1 | exonic | missense SNV | Limch1:NM_001001980:exon4:c.A307G:p.S103G | . | 5qC3.1 | . | 0 | 1 |
| chr5 | 67027492 | A | G | 200 | Limch1 | exonic | missense SNV | Limch1:NM_001256122:exon13:c.A1823G:p.K608R,Limch1:NM_001001980:exon18:c.A2291G:p.K764R | . | 5qC3.1 | . | 0 | 1 |
| chr5 | 67264248 | C | T | 221 | Tmem33 | intronic | . | . | . | 5qC3.1 | . | 0 | 1 |
| chr5 | 67467654 | A | C | 120 | C330024D21Rik | ncRNA_intronic | . | . | . | 5qC3.1 | . | 0 | 1 |
| chr5 | 67611300 | C | G | 221 | Shisa3 | exonic | missense SNV | Shisa3:NM_001033415:exon2:c.C542G:p.A181G | . | 5qC3.1 | . | 0 | 1 |
| chr5 | 67769017 | T | G | 157 | Atp8a1 | intronic | . | . | . | 5qC3.1 | . | 0 | 1 |
| chr5 | 68032170 | G | A | 221 | Grxcr1 | exonic | missense SNV | Grxcr1:NM_001018019:exon1:c.G283A:p.E95K | . | 5qC3.1 | . | 0 | 1 |
| chr5 | 68110497 | G | T | 222 | Grxcr1 | exonic | missense SNV | Grxcr1:NM_001018019:exon2:c.G588T:p.R196S | . | 5qC3.1 | . | 0 | 1 |
| chr5 | 69517090 | G | A | 222 | Yipf7 | exonic | synonymous SNV | Yipf7:NM_023784:exon6:c.C711T:p.V237V | . | 5qC3.1 | . | 0 | 1 |
| chr5 | 69568536 | G | A | 222 | Guf1 | intronic | . | . | . | 5qC3.1 | . | 0 | 1 |
| chr5 | 69575743 | G | A | 118 | Gnpda2 | UTR3 | . | . | . | 5qC3.1 | . | 0 | 1 |
| chr5 | 70754084 | A | T | 212 | Gabrg1 | exonic | missense SNV | Gabrg1:NM_010252:exon9:c.T1336A:p.F446I | . | 5qC3.1 | . | 0 | 1 |
| chr5 | 71633524 | C | A | 110 | Gabra4 | intronic | . | . | . | 5qC3.2 | . | 0 | 1 |
| chr5 | 72239234 | G | A | 221 | Atp10d | exonic | unknown | UNKNOWN | . | 5qC3.2 | . | 0 | 1 |
| chr5 | 72435313 | C | T | 221 | Corin | exonic | missense SNV | Corin:NM_001122756:exon4:c.G542A:p.R181H,Corin:NM_016869:exon4:c.G740A:p.R247H | . | 5qC3.2 | . | 0 | 1 |
| chr5 | 72616702 | T | C | 221 | Cnga1 | intronic | . | . | . | 5qC3.2 | . | 0 | 1 |
| chr5 | 72667494 | T | C | 68 | Nipal1 | intronic | . | . | . | 5qC3.2 | rs31540608 | 0 | 1 |
| chr5 | 72696701 | A | G | 222 | Txk | intronic | . | . | . | 5qC3.2 | . | 0 | 1 |
| chr5 | 73059398 | A | G | 221 | Fryl | exonic | missense SNV | Fryl:NM_028194:exon48:c.T6548C:p.L2183P | . | 5qC3.2 | . | 0 | 1 |
| chr5 | 73095412 | T | A | 222 | Fryl | splicing | . | . | . | 5qC3.2 | . | 0 | 1 |
| chr5 | 73122124 | A | C | 208 | Fryl | intronic | . | . | . | 5qC3.2 | . | 0 | 1 |
| chr5 | 73635469 | C | G | 222 | Sgcb | UTR3 | . | . | . | 5qC3.3 | . | 0 | 1 |
| chr5 | 75011327 | T | C | 222 | Chic2 | intronic | . | . | . | 5qC3.3 | rs29543566 | 0 | 1 |
| chr5 | 75609448 | C | A | 169 | Kit | exonic | synonymous SNV | Kit:NM_001122733:exon3:c.C519A:p.I173I,Kit:NM_021099:exon3:c.C519A:p.I173I | . | 5qC3.3 | . | 0 | 1 |
| chr5 | 75609578 | G | T | 168 | Kit | intronic | . | . | . | 5qC3.3 | . | 0 | 1 |
| chr5 | 75648502 | C | T | 145 | Kit | exonic | synonymous SNV | Kit:NM_001122733:exon16:c.C2355T:p.L785L,Kit:NM_021099:exon16:c.C2343T:p.L781L | . | 5qC3.3 | . | 0 | 1 |
| chr5 | 76545199 | C | A | 95 | Exoc1 | intronic | . | . | . | 5qC3.3 | . | 0 | 1 |
| chr5 | 76595534 | G | C | 221 | Cep135 | exonic | missense SNV | Cep135:NM_199032:exon4:c.G614C:p.R205T | . | 5qC3.3 | . | 0 | 1 |
| chr5 | 76657956 | G | A | 59 | . | intergenic | . | . | 866 | 5qC3.3 | . | 0 | 1 |
| chr5 | 77047249 | T | A | 221 | Thegl | exonic | missense SNV | Thegl:NM_027970:exon4:c.T689A:p.L230Q | . | 5qC3.3 | . | 0 | 1 |
| chr5 | 80900703 | T | G | 196 | . | intergenic | . | . | . | 5qD | . | 0 | 1 |
| chr5 | 80901367 | G | T | 221 | . | intergenic | . | . | . | 5qD | . | 0 | 1 |
| chr5 | 81646526 | C | T | 221 | Adgrl3 | exonic | missense SNV | Adgrl3:NM_198702:exon11:c.C1597T:p.R533C | . | 5qD | . | 0 | 1 |
| chr5 | 81688732 | T | G | 155 | Adgrl3 | intronic | . | . | . | 5qE1 | . | 0 | 1 |
| chr5 | 83729091 | C | A | 79 | . | intergenic | . | . | . | 5qE1 | . | 0 | 1 |
| chr5 | 84081056 | T | C | 222 | Epha5 | intronic | . | . | . | 5qE1 | . | 0 | 1 |
| chr5 | 84123502 | G | A | 121 | Epha5 | intronic | . | . | . | 5qE1 | . | 0 | 1 |
| chr5 | 84156450 | C | T | 222 | Epha5 | exonic | missense SNV | Epha5:NM_007937:exon4:c.G1003A:p.G335R | . | 5qE1 | . | 0 | 1 |
| chr5 | 86034226 | A | T | 222 | Cenpc1 | intronic | . | . | . | 5qE1 | . | 0 | 1 |
| chr5 | 86185365 | G | T | 222 | Gnrhr | exonic | missense SNV | Gnrhr:NM_001310653:exon2:c.C645A:p.F215L,Gnrhr:NM_010323:exon2:c.C645A:p.F215L | . | 5qE1 | . | 0 | 1 |
| chr5 | 86235572 | C | G | 47.6416 | Tmprss11c | exonic | missense SNV | Tmprss11c:NM_001030297:exon9:c.G994C:p.D332H | . | 5qE1 | . | 0 | 1 |
| chr5 | 86306551 | A | T | 45.5975 | Tmprss11d | intronic | . | . | . | 5qE1 | . | 0 | 1 |
| chr5 | 86326776 | A | G | 65 | Tmprss11d | intronic | . | . | . | 5qE1 | . | 0 | 1 |
| chr5 | 86715376 | G | A | 87 | Tmprss11e | intronic | . | . | . | 5qE1 | . | 0 | 1 |
| chr5 | 86892936 | T | G | 221 | Ugt2b34 | exonic | missense SNV | Ugt2b34:NM_153598:exon5:c.A1126C:p.T376P | . | 5qE1 | . | 0 | 1 |
| chr5 | 86893742 | C | A | 221 | Ugt2b34 | exonic | missense SNV | Ugt2b34:NM_153598:exon4:c.G1090T:p.D364Y | . | 5qE1 | . | 0 | 1 |
| chr5 | 87066160 | T | G | 133 | Ugt2b36 | UTR3 | . | . | . | 5qE1 | . | 0 | 1 |
| chr5 | 87250364 | A | G | 35.563 | Ugt2b37 | intronic | . | . | . | 5qE1 | . | 0 | 1 |
| chr5 | 87369909 | T | G | 222 | . | intergenic | . | . | . | 5qE1 | . | 0 | 1 |
| chr5 | 87601312 | A | C | 222 | . | intergenic | . | . | . | 5qE1 | . | 0 | 1 |
| chr5 | 87669543 | C | A | 132 | Csn1s1 | exonic | missense SNV | Csn1s1:NM_001286015:exon5:c.C141A:p.N47K,Csn1s1:NM_001286016:exon5:c.C141A:p.N47K,Csn1s1:NM_( | . | 5qE1 | . | 0 | 1 |
| chr5 | 87670911 | T | G | 132 | Csn1s1 | exonic | missense SNV | Csn1s1:NM_001286015:exon6:c.T167G:p.M56R,Csn1s1:NM_001286016:exon6:c.T167G:p.M56R,Csn1s1:NM_( | . | 5qE1 | . | 0 | 1 |
| chr5 | 87787508 | G | A | 43.9904 | Csn1s2a | intronic | . | . | . | 5qE1 | . | 0 | 1 |
| chr5 | 87929659 | G | A | 119 | Csn3,1700066N21Ri | ncRNA_intronic | . | . | . | 5qE1 | . | 0 | 1 |
| chr5 | 87972835 | A | G | 182 | 1700066N21Rik | ncRNA_intronic | . | . | . | 5qE1 | . | 0 | 1 |
| chr5 | 88503439 | A | G | 203 | Enam | exonic | missense SNV | Enam:NM_017468:exon10:c.A2581G:p.N861D | . | 5qE1 | . | 0 | 1 |
| chr5 | 89422010 | C | T | 222 | Gc | intronic | . | . | . | 5qE1 | rs234625831 | 0 | 1 |
| chr5 | 89698299 | G | A | 222 | Adamts3 | exonic | missense SNV | Adamts3:NM_001081401:exon15:c.C2084T:p.S695F,Adamts3:NM_177872:exon15:c.C2084T:p.S695F | . | 5qE1 | . | 0 | 1 |
| chr5 | 89775351 | C | T | 222 | Adamts3 | exonic | missense SNV | Adamts3:NM_001081401:exon4:c.G595A:p.V199I,Adamts3:NM_177872:exon4:c.G595A:p.V199I | . | 5qE1 | rs262569844 | 0 | 1 |
| chr5 | 91593876 | T | G | 170 | Parm1 | exonic | missense SNV | Parm1:NM_145562:exon2:c.T102G:p.S34R | . | 5qE2 | . | 0 | 1 |
| chr5 | 92650566 | A | T | 71 | Ccdc158 | intronic | . | . | . | 5qE2 | . | 0 | 1 |
| chr5 | 92940459 | C | T | 222 | Shroom3 | exonic | missense SNV | Shroom3:NM_001077595:exon4:c.C542T:p.T181M,Shroom3:NM_001077596:exon4:c.C542T:p.T181M,Shroom3 | . | 5qE2 | . | 0 | 1 |
| chr5 | 96262358 | A | C | 43.9771 | Mrpl1 | intronic | . | . | . | 5qE3 | . | 0 | 1 |
| chr5 | 96733772 | G | A | 222 | Fras1 | intronic | . | . | . | 5qE3 | . | 0 | 1 |
| chr5 | 96758142 | T | C | 228 | Fras1 | exonic | missense SNV | Fras1:NM_175473:exon62:c.T9404C:p.L3135P | . | 5qE3 | rs46608930 | 1 | 0 |
| chr5 | 99233967 | G | C | 222 | Rasgef1b | exonic | missense SNV | Rasgef1b:NM_145839:exon6:c.C677G:p.P226R,Rasgef1b:NM_181318:exon6:c.C674G:p.P225R | . | 5qE3 | . | 0 | 1 |
| chr5 | 100475530 | A | T | 126 | Lin54 | intronic | . | . | . | 5qE4 | . | 0 | 1 |
| chr5 | 101926413 | A | C | 222 | Wdfy3 | intronic | . | . | . | 5qE5 | . | 0 | 1 |
| chr5 | 101941495 | C | T | 222 | Wdfy3 | exonic | missense SNV | Wdfy3:NM_172882:exon13:c.G1832A:p.G611E | . | 5qE5 | . | 0 | 1 |
| chr5 | 103990473 | C | T | 222 | Hsd17b11 | UTR3 | . | . | . | 5qE5 | . | 0 | 1 |
| chr5 | 104211773 | G | A | 222 | Dmp1 | exonic | missense SNV | Dmp1:NM_016779:exon6:c.G314A:p.S105N | . | 5qE5 | . | 0 | 1 |
| chr5 | 106618122 | T | C | 125 | Zfp644 | UTR3 | . | . | . | 5qE5 | . | 0 | 1 |
| chr5 | 107357016 | G | T | 131 | Brdt | intronic | . | . | . | 5qE5 | . | 0 | 1 |
| chr5 | 107560802 | C | T | 185 | Glmn | intronic | . | . | . | 5qE5 | . | 0 | 1 |
| chr5 | 108092153 | A | C | 222 | Mtf2 | exonic | missense SNV | Mtf2:NM_001253880:exon5:c.A419C:p.D140A,Mtf2:NM_001253879:exon7:c.A419C:p.D140A,Mtf2:NM_0138 | . | 5qF | . | 0 | 1 |
| chr5 | 108638335 | G | A | 36.704 | Tmem175 | UTR5 | . | . | . | 5qF | . | 0 | 1 |
| chr5 | 109156173 | G | T | 222 | Vmn2r13 | exonic | synonymous SNV | Vmn2r13:NM_001104624:exon6:c.C2391A:p.T797T | . | 5qF | . | 0 | 1 |
| chr5 | 109757467 | C | T | 131 | . | intergenic | . | . | . | 5qF | . | 0 | 1 |
| chr5 | 110202515 | A | C | 172 | Golga3 | intronic | . | . | . | 5qF | . | 0 | 1 |
| chr5 | 110336894 | T | G | 222 | Pole | intronic | . | . | . | 5qF | . | 0 | 1 |
| chr5 | 110808975 | C | A | 222 | Ulk1 | intronic | . | . | . | 5qF | . | 0 | 1 |
| chr5 | 112790055 | T | A | 98 | Myo18b | exonic | missense SNV | Myo18b:NM_028901:exon31:c.A5203T:p.T1735S | . | 5qF | . | 0 | 1 |
| chr5 | 112969344 | C | T | 78 | Adrbk2 | intronic | . | . | . | 5qF | . | 0 | 1 |
| chr5 | 113062130 | C | T | 204 | Crybb2 | exonic | missense SNV | Crybb2:NM_007773:exon6:c.G366A:p.M122I | . | 5qF | . | 0 | 1 |
| chr5 | 113062134 | T | G | 149 | Crybb2 | exonic | missense SNV | Crybb2:NM_007773:exon6:c.A362C:p.K121T | . | 5qF | . | 0 | 1 |
| chr5 | 113561021 | G | T | 222 | Wscd2 | exonic | synonymous SNV | Wscd2:NM_177292:exon3:c.G636T:p.V212V | . | 5qF | . | 0 | 1 |
| chr5 | 113614128 | A | T | 222 | Cmklr1 | exonic | missense SNV | Cmklr1:NM_008153:exon3:c.T811A:p.W271R | . | 5qF | . | 0 | 1 |
| chr5 | 113751118 | G | A | 123 | Sart3 | intronic | . | . | . | 5qF | . | 0 | 1 |
| chr5 | 113817463 | A | G | 86 | . | downstream | . | . | . | 5qF | . | 0 | 1 |
| chr5 | 114184328 | G | T | 60 | Acacb | intronic | . | . | . | 5qF | . | 0 | 1 |
| chr5 | 114345832 | A | T | 211 | Myo1h | intronic | . | . | . | 5qF | . | 0 | 1 |
| chr5 | 115247076 | G | C | 222 | Rnf10 | intronic | . | . | . | 5qF | . | 0 | 1 |
| chr5 | 115571563 | G | A | 222 | Gcn1l1 | intronic | . | . | . | 5qF | . | 0 | 1 |
| chr5 | 115592356 | G | C | 222 | Gcn1l1 | exonic | missense SNV | Gcn1l1:NM_172719:exon24:c.G2598C:p.M866I | . | 5qF | . | 0 | 1 |
| chr5 | 115637607 | G | A | 222 | Rab35 | intronic | . | . | . | 5qF | . | 0 | 1 |
| chr5 | 116051494 | C | G | 221 | Tmem233 | intronic | . | . | . | 5qF | . | 0 | 1 |
| chr5 | 117263841 | A | T | 133 | Taok3 | intronic | . | . | . | 5qF | . | 0 | 1 |
| chr5 | 117334896 | G | C | 139 | Vsig10 | exonic | missense SNV | Vsig10:NM_001033311:exon4:c.G553C:p.E185Q | . | 5qF | . | 0 | 1 |
| chr5 | 117949780 | G | T | 219 | Nos1 | exonic | missense SNV | Nos1:NM_008712:exon26:c.G4107T:p.Q1369H | . | 5qF | . | 0 | 1 |
| chr5 | 118077514 | A | C | 208 | Fbxw8 | intronic | . | . | . | 5qF | . | 0 | 1 |
| chr5 | 118077591 | G | T | 222 | Fbxw8 | exonic | synonymous SNV | Fbxw8:NM_172721:exon8:c.C1353A:p.G451G | . | 5qF | . | 0 | 1 |
| chr5 | 119838675 | A | T | 222 | Tbx5 | intronic | . | . | . | 5qF | rs48642259 | 0 | 1 |
| chr5 | 120761343 | G | C | 166 | Oas3 | intronic | . | . | . | 5qF | . | 0 | 1 |
| chr5 | 120814360 | G | T | 98 | Oas1b | intronic | . | . | . | 5qF | . | 0 | 1 |
| chr5 | 121487869 | G | C | 222 | Tmem116 | intronic | . | . | . | 5qF | . | 0 | 1 |
| chr5 | 122171726 | G | A | 195 | Ppp1cc | intronic | . | . | . | 5qF | . | 0 | 1 |
| chr5 | 122458097 | G | C | 222 | Atp2a2 | exonic | missense SNV | Atp2a2:NM_001110140:exon18:c.C2732G:p.A911G,Atp2a2:NM_009722:exon18:c.C2732G:p.A911G | . | 5qF | . | 0 | 1 |
| chr5 | 122673300 | G | T | 174 | P2rx7 | intronic | . | . | . | 5qF | . | 0 | 1 |
| chr5 | 122697217 | C | A | 111 | . | intergenic | . | . | . | 5qF | . | 0 | 1 |
| chr5 | 122799170 | G | A | 80 | Anapc5 | intronic | . | . | . | 5qF | . | 0 | 1 |
| chr5 | 123570925 | C | A | 222 | Vps33a | exonic | missense SNV | Vps33a:NM_029929:exon3:c.G226T:p.V76F | . | 5qF | . | 0 | 1 |
| chr5 | 124388718 | A | G | 108 | Sbno1 | intronic | . | . | . | 5qF | . | 0 | 1 |
| chr5 | 124514652 | T | G | 150 | Rilpl1 | exonic | missense SNV | Rilpl1:NM_021430:exon4:c.A671C:p.E224A | . | 5qF | . | 0 | 1 |
| chr5 | 124720005 | C | T | 222 | Atp6v0a2 | exonic | synonymous SNV | Atp6v0a2:NM_011596:exon19:c.C2448T:p.H816H | . | 5qF | . | 0 | 1 |
| chr5 | 126026315 | G | C | 168 | . | intergenic | . | . | . | 5qG1.1 | . | 0 | 1 |
| chr5 | 127462891 | C | G | 212 | Tmem132c | intronic | . | . | . | 5qG1.2 | . | 0 | 1 |
| chr5 | 128269155 | G | T | 222 | Tmem132d | exonic | stopgain | Tmem132d:NM_172885:exon2:c.C302A:p.S101X | . | 5qG1.3 | . | 0 | 1 |
| chr5 | 128585499 | G | A | 186 | 5930412G12Rik | ncRNA_intronic | . | . | . | 5qG1.3 | . | 0 | 1 |
| chr5 | 128752392 | G | A | 85 | Piwil1 | intronic | . | . | . | 5qG1.3 | . | 0 | 1 |
| chr5 | 128797628 | C | A | 222 | Rimbp2 | exonic | missense SNV | Rimbp2:NM_001310733:exon10:c.G898T:p.D300Y,Rimbp2:NM_001081388:exon11:c.G919T:p.D307Y | . | 5qG1.3 | . | 0 | 1 |
| chr5 | 128797629 | C | T | 222 | Rimbp2 | exonic | synonymous SNV | Rimbp2:NM_001310733:exon10:c.G897A:p.L299L,Rimbp2:NM_001081388:exon11:c.G918A:p.L306L | . | 5qG1.3 | . | 0 | 1 |
| chr5 | 129132648 | G | A | 222 | Adgrd1 | intronic | . | . | . | 5qG1.3 | . | 0 | 1 |
| chr5 | 129136816 | A | T | 142 | Adgrd1 | intronic | . | . | . | 5qG1.3 | . | 0 | 1 |
| chr5 | 129504699 | A | G | 130 | Sfswap | intronic | . | . | . | 5qG1.3 | . | 0 | 1 |
| chr5 | 129683528 | A | T | 222 | 14-9月 | exonic | missense SNV | Sept14:NM_028826:exon11:c.T1252A:p.C418S | . | 5qG1.3 | . | 0 | 1 |
| chr5 | 130839279 | C | A | 222 | Caln1 | splicing | . | . | . | 5qG2 | . | 0 | 1 |
| chr5 | 136105701 | G | T | 172 | Rasa4 | exonic | missense SNV | Rasa4:NM_001039103:exon17:c.G1925T:p.G642V,Rasa4:NM_133914:exon18:c.G2063T:p.G688V | . | 5qG2 | . | 0 | 1 |
| chr5 | 136116724 | G | T | 188 | Polr2j | UTR5 | . | . | 524 | 5qG2 | . | 0 | 1 |
| chr5 | 136232067 | A | G | 201 | Sh2b2 | exonic | synonymous SNV | Sh2b2:NM_001302938:exon2:c.T294C:p.T98T,Sh2b2:NM_001302939:exon2:c.T294C:p.T98T,Sh2b2:NM_018 | 691 | 5qG2 | . | 0 | 1 |
| chr5 | 136993117 | G | C | 163 | Plod3 | intronic | . | . | . | 5qG2 | . | 0 | 1 |
| chr5 | 137138070 | G | C | 222 | . | intergenic | . | . | . | 5qG2 | . | 0 | 1 |
| chr5 | 137426246 | G | A | 220 | Zan | exonic | synonymous SNV | Zan:NM_011741:exon39:c.C7779T:p.V2593V | . | 5qG2 | . | 0 | 1 |
| chr5 | 137564748 | A | G | 222 | Actl6b | exonic | missense SNV | Actl6b:NM_031404:exon7:c.A658G:p.I220V | . | 5qG2 | . | 0 | 1 |
| chr5 | 138133441 | A | T | 222 | Zscan21 | exonic | missense SNV | Zscan21:NM_001044703:exon4:c.A1226T:p.H409L,Zscan21:NM_001044705:exon4:c.A1226T:p.H409L,Zscan | . | 5qG2 | . | 0 | 1 |
| chr5 | 138311841 | G | A | 222 | Stag3 | intronic | . | . | . | 5qG2 | . | 0 | 1 |
| chr5 | 138364113 | T | C | 166 | . | intergenic | . | . | . | 5qG2 | rs49109595 | 1 | 0 |
| chr5 | 138996168 | G | A | 220 | . | intergenic | . | . | 3301 | 5qG2 | . | 0 | 1 |
| chr5 | 139252742 | G | C | 102 | Get4 | intronic | . | . | . | 5qG2 | . | 0 | 1 |
| chr5 | 139354203 | G | A | 82 | Cyp2w1 | intronic | . | . | . | 5qG2 | . | 0 | 1 |
| chr5 | 139754096 | G | A | 222 | Ints1 | exonic | stopgain | Ints1:NM_026748:exon41:c.C6031T:p.Q2011X | . | 5qG2 | . | 0 | 1 |
| chr5 | 140614024 | G | A | 117 | Lfng | intronic | . | . | . | 5qG2 | . | 0 | 1 |
| chr5 | 142046197 | T | C | 222 | Sdk1 | exonic | missense SNV | Sdk1:NM_177879:exon21:c.T3026C:p.M1009T | . | 5qG2 | rs29564987 | 0 | 1 |
| chr5 | 142146269 | A | C | 215 | Sdk1 | exonic | missense SNV | Sdk1:NM_177879:exon35:c.A4952C:p.K1651T | . | 5qG2 | . | 0 | 1 |
| chr5 | 142161696 | C | T | 86 | Sdk1 | intronic | . | . | . | 5qG2 | rs232483151 | 0 | 1 |
| chr5 | 142659108 | G | A | 221 | Wipi2 | exonic | synonymous SNV | Wipi2:NM_178398:exon5:c.G468A:p.A156A | . | 5qG2 | . | 0 | 1 |
| chr5 | 143221240 | A | C | 143 | Spdye4a | intronic | . | . | . | 5qG2 | . | 0 | 1 |
| chr5 | 143308042 | C | G | 222 | E130309D02Rik | exonic | missense SNV | E130309D02Rik:NM_172726:exon4:c.G679C:p.A227P | . | 5qG2 | . | 0 | 1 |
| chr5 | 143320633 | G | A | 110 | Zdhhc4 | intronic | . | . | . | 5qG2 | rs255254032 | 0 | 1 |
| chr5 | 143728053 | T | C | 221 | Usp42 | intronic | . | . | . | 5qG2 | . | 0 | 1 |
| chr5 | 144183452 | G | C | 45.2675 | Lmtk2 | intronic | . | . | . | 5qG2 | . | 0 | 1 |
| chr5 | 144806038 | T | C | 222 | Trrap | intronic | . | . | . | 5qG2 | . | 0 | 1 |
| chr5 | 144807168 | T | A | 130 | Trrap | intronic | . | . | . | 5qG2 | . | 0 | 1 |
| chr5 | 144857528 | G | C | 222 | Trrap | exonic | synonymous SNV | Trrap:NM_001081362:exon70:c.G11208C:p.L3736L | . | 5qG2 | . | 0 | 1 |
| chr5 | 144893094 | T | A | 222 | Smurf1 | intronic | . | . | . | 5qG2 | . | 0 | 1 |
| chr5 | 144985587 | G | A | 168 | Kpna7 | intronic | . | . | . | 5qG2 | . | 0 | 1 |
| chr5 | 145368830 | G | T | 67 | Cyp3a57 | intronic | . | . | . | 5qG2 | . | 0 | 1 |
| chr5 | 147453122 | C | T | 107 | Pan3 | intronic | . | . | . | 5qG3 | . | 0 | 1 |
| chr5 | 149272513 | G | C | 222 | Alox5ap | intronic | . | . | . | 5qG3 | . | 0 | 1 |
| chr5 | 149611713 | G | T | 221 | Wdr95 | exonic | missense SNV | Wdr95:NM_029440:exon23:c.G1909T:p.G637C | . | 5qG3 | . | 0 | 1 |
| chr5 | 150056784 | T | G | 217 | Rxfp2 | intronic | . | . | . | 5qG3 | . | 0 | 1 |
| chr5 | 150395658 | G | A | 59 | Fry | intronic | . | . | . | 5qG3 | . | 0 | 1 |
| chr5 | 150395933 | A | C | 221 | Fry | intronic | . | . | . | 5qG3 | . | 0 | 1 |
| chr5 | 150403751 | A | C | 105 | Fry | intronic | . | . | . | 5qG3 | . | 0 | 1 |

| | | | | | | | | | | | | | |
|---|---|---|---|---|---|---|---|---|---|---|---|---|---|
| chr5 | 150560661 | G | T | 221 | Brca2 | intronic | . | . | . | 5qG3 | . | 0 | 1 |
| chr5 | 150953055 | G | A | 163 | Kl | exonic | synonymous SNV | Kl:NM_013823:exon1:c.G339A:p.S113S | 927 | 5qG3 | . | 0 | 1 |
| chr5 | 150991744 | C | A | 221 | Kl | exonic | missense SNV | Kl:NM_013823:exon5:c.C2935A:p.Q979K | . | 5qG3 | . | 0 | 1 |
| chr6 | 3517774 | A | C | 154 | Vps50 | intronic | . | . | . | 6qA1 | . | 0 | 1 |
| chr6 | 4509747 | A | C | 222 | Col1a2 | intronic | . | . | . | 6qA1 | . | 0 | 1 |
| chr6 | 4512499 | T | C | 212 | Col1a2 | intronic | . | . | . | 6qA1 | . | 0 | 1 |
| chr6 | 4756435 | A | C | 149 | Peg10 | exonic | unknown | UNKNOWN | . | 6qA1 | rs52233015 | 1 | 0 |
| chr6 | 5171711 | G | T | 131 | Pon1 | intronic | . | . | . | 6qA1 | . | 0 | 1 |
| chr6 | 5429621 | T | C | 88 | Asb4 | intronic | . | . | . | 6qA1 | . | 0 | 1 |
| chr6 | 5961813 | G | T | 221 | Dync1i1 | intronic | . | . | . | 6qA1 | . | 0 | 1 |
| chr6 | 5969258 | T | C | 176 | 1700019G24Rik,Dyn | ncRNA_exonic | . | . | . | 6qA1 | . | 0 | 1 |
| chr6 | 6329980 | C | T | 67 | . | intergenic | . | . | . | 6qA1 | . | 0 | 1 |
| chr6 | 6735566 | C | A | 174 | . | intergenic | . | . | . | 6qA1 | . | 0 | 1 |
| chr6 | 7562401 | C | T | 61 | Tac1 | exonic | missense SNV | Tac1:NM_001311060:exon6:c.C317T:p.A106V,Tac1:NM_009311:exon7:c.C362T:p.A121V | . | 6qA1 | . | 0 | 1 |
| chr6 | 7866674 | C | T | 161 | C1galt1 | exonic | synonymous SNV | C1galt1:NM_052993:exon3:c.C519T:p.Y173Y | . | 6qA1 | . | 0 | 1 |
| chr6 | 8256511 | T | G | 90 | Rpa3 | intronic | . | . | . | 6qA1 | . | 0 | 1 |
| chr6 | 8537988 | T | G | 221 | Glcci1 | intronic | . | . | . | 6qA1 | rs37592724 | 0 | 1 |
| chr6 | 8538023 | A | G | 222 | Glcci1 | intronic | . | . | . | 6qA1 | rs49293910 | 0 | 1 |
| chr6 | 8538039 | A | G | 149 | Glcci1 | intronic | . | . | . | 6qA1 | rs45721151 | 0 | 1 |
| chr6 | 8538060 | C | A | 42.1614 | Glcci1 | intronic | . | . | . | 6qA1 | rs46603526 | 0 | 1 |
| chr6 | 8538062 | T | G | 52 | Glcci1 | intronic | . | . | . | 6qA1 | rs50331376 | 0 | 1 |
| chr6 | 8582919 | A | G | 123 | Glcci1 | intronic | . | . | . | 6qA1 | rs31003969 | 0 | 1 |
| chr6 | 8591708 | C | G | 220 | Glcci1 | intronic | . | . | . | 6qA1 | rs214337173 | 0 | 1 |
| chr6 | 8592712 | C | T | 64 | Glcci1 | intronic | . | . | . | 6qA1 | rs30074521 | 0 | 1 |
| chr6 | 8593177 | A | T | 221 | Glcci1 | exonic | synonymous SNV | Glcci1:NM_001286728:exon8:c.A981T:p.P327P,Glcci1:NM_001286729:exon8:c.A978T:p.P326P,Glcci1:NM | . | 6qA1 | rs36281196 | 0 | 1 |
| chr6 | 8596272 | G | A | 221 | Glcci1 | UTR3 | . | . | . | 6qA1 | rs253394221 | 0 | 1 |
| chr6 | 8596781 | T | C | 220 | Glcci1 | UTR3 | . | . | . | 6qA1 | rs37608077 | 0 | 1 |
| chr6 | 8596841 | A | G | 220 | Glcci1 | UTR3 | . | . | . | 6qA1 | rs245515878 | 0 | 1 |
| chr6 | 8643735 | C | A | 120 | Ica1 | intronic | . | . | . | 6qA1 | rs234712955 | 0 | 1 |
| chr6 | 8644098 | T | C | 219 | Ica1 | exonic | missense SNV | Ica1:NM_001252266:exon11:c.A1127G:p.Q376R,Ica1:NM_010492:exon13:c.A1190G:p.Q397R | . | 6qA1 | rs6218746 | 0 | 1 |
| chr6 | 8653581 | T | C | 221 | Ica1 | intronic | . | . | . | 6qA1 | rs37861549 | 0 | 1 |
| chr6 | 8653758 | T | C | 221 | Ica1 | intronic | . | . | . | 6qA1 | rs38132695 | 0 | 1 |
| chr6 | 8653865 | G | A | 86 | Ica1 | intronic | . | . | . | 6qA1 | rs36424368 | 1 | 0 |
| chr6 | 8656371 | C | T | 221 | Ica1 | exonic | missense SNV | Ica1:NM_001252266:exon9:c.G934A:p.A312T,Ica1:NM_010492:exon10:c.G934A:p.A312T | . | 6qA1 | rs51311141 | 0 | 1 |
| chr6 | 8658187 | A | G | 222 | Ica1 | intronic | . | . | . | 6qA1 | rs225252316 | 0 | 1 |
| chr6 | 8658279 | T | C | 222 | Ica1 | exonic | synonymous SNV | Ica1:NM_001252266:exon8:c.A837G:p.K279K,Ica1:NM_010492:exon9:c.A837G:p.K279K | . | 6qA1 | rs46916259 | 0 | 1 |
| chr6 | 8667092 | C | T | 29.6081 | Ica1 | intronic | . | . | . | 6qA1 | rs51464239 | 0 | 1 |
| chr6 | 8667138 | A | G | 76 | Ica1 | intronic | . | . | . | 6qA1 | rs47910215 | 0 | 1 |
| chr6 | 8667207 | T | C | 211 | Ica1 | intronic | . | . | . | 6qA1 | rs39204098 | 0 | 1 |
| chr6 | 8667239 | G | A | 222 | Ica1 | intronic | . | . | . | 6qA1 | rs37144831 | 0 | 1 |
| chr6 | 8667448 | A | C | 202 | Ica1 | intronic | . | . | . | 6qA1 | rs36365562 | 0 | 1 |
| chr6 | 8670486 | C | A | 106 | Ica1 | intronic | . | . | . | 6qA1 | rs231334510 | 0 | 1 |
| chr6 | 8670491 | G | A | 104 | Ica1 | intronic | . | . | . | 6qA1 | rs36661463 | 0 | 1 |
| chr6 | 8670896 | C | T | 109 | Ica1 | intronic | . | . | . | 6qA1 | rs217630964 | 0 | 1 |
| chr6 | 8672429 | G | A | 222 | Ica1 | intronic | . | . | . | 6qA1 | rs234867783 | 0 | 1 |
| chr6 | 8742285 | C | G | 190 | Ica1 | exonic | missense SNV | Ica1:NM_001252266:exon4:c.G347C:p.G116A,Ica1:NM_010492:exon5:c.G347C:p.G116A | . | 6qA1 | . | 0 | 1 |
| chr6 | 8749919 | C | T | 67 | Ica1 | intronic | . | . | . | 6qA1 | . | 0 | 1 |
| chr6 | 8754472 | A | G | 152 | Ica1 | intronic | . | . | . | 6qA1 | rs252446959 | 0 | 1 |
| chr6 | 8754622 | A | T | 221 | Ica1 | exonic | synonymous SNV | Ica1:NM_001252266:exon2:c.T147A:p.V49V,Ica1:NM_010492:exon3:c.T147A:p.V49V | . | 6qA1 | rs36353906 | 0 | 1 |
| chr6 | 8758355 | A | C | 222 | Ica1 | intronic | . | . | . | 6qA1 | rs242151579 | 0 | 1 |
| chr6 | 8950325 | C | T | 220 | Nxph1 | intronic | . | . | . | 6qA1 | rs30454007 | 0 | 1 |
| chr6 | 8951437 | G | A | 127 | Nxph1 | intronic | . | . | 462 | 6qA1 | rs13478620 | 0 | 1 |
| chr6 | 10172039 | G | A | 40.452 | . | intergenic | . | . | . | 6qA1 | rs48536047 | 0 | 1 |
| chr6 | 10172043 | A | G | 28.085 | . | intergenic | . | . | . | 6qA1 | rs49176621 | 0 | 1 |
| chr6 | 10172118 | C | G | 56 | . | intergenic | . | . | . | 6qA1 | rs46293109 | 0 | 1 |
| chr6 | 10172222 | C | A | 222 | . | intergenic | . | . | . | 6qA1 | rs49061374 | 0 | 1 |
| chr6 | 10172281 | T | G | 222 | . | intergenic | . | . | . | 6qA1 | rs50980752 | 0 | 1 |
| chr6 | 10334484 | G | T | 48.7612 | . | intergenic | . | . | . | 6qA1 | rs30563004 | 0 | 1 |
| chr6 | 10334571 | A | G | 66 | . | intergenic | . | . | . | 6qA1 | rs29826391 | 0 | 1 |
| chr6 | 10334696 | A | G | 145 | . | intergenic | . | . | . | 6qA1 | rs36501254 | 0 | 1 |
| chr6 | 10441700 | T | C | 228 | . | intergenic | . | . | . | 6qA1 | rs30605551 | 1 | 0 |
| chr6 | 10882464 | A | C | 131 | . | intergenic | . | . | . | 6qA1 | rs214869844 | 1 | 0 |
| chr6 | 10884560 | T | C | 167 | . | intergenic | . | . | . | 6qA1 | rs30226761 | 0 | 1 |
| chr6 | 10884659 | A | T | 148 | . | intergenic | . | . | . | 6qA1 | rs252765773 | 0 | 1 |
| chr6 | 10888390 | G | A | 154 | . | intergenic | . | . | . | 6qA1 | rs229009787 | 0 | 1 |
| chr6 | 10888593 | A | G | 222 | . | intergenic | . | . | . | 6qA1 | rs237119321 | 0 | 1 |
| chr6 | 10888692 | G | A | 79 | . | intergenic | . | . | . | 6qA1 | rs256654291 | 0 | 1 |
| chr6 | 11900544 | T | A | 222 | Ndufa4 | exonic | synonymous SNV | Ndufa4:NM_010886:exon4:c.A240T:p.P80P | . | 6qA1 | . | 0 | 1 |
| chr6 | 11926561 | T | C | 160 | Phf14 | intronic | . | . | . | 6qA1 | rs222506414 | 0 | 1 |
| chr6 | 11926660 | C | T | 221 | Phf14 | intronic | . | . | . | 6qA1 | rs242770409 | 0 | 1 |
| chr6 | 11933797 | G | A | 222 | Phf14 | exonic | synonymous SNV | Phf14:NM_001168382:exon3:c.G657A:p.A219A,Phf14:NM_029404:exon3:c.G657A:p.A219A | . | 6qA1 | rs52075887 | 0 | 1 |
| chr6 | 11933922 | G | T | 221 | Phf14 | exonic | missense SNV | Phf14:NM_001168382:exon3:c.G782T:p.G261V,Phf14:NM_029404:exon3:c.G782T:p.G261V | . | 6qA1 | rs229602402 | 0 | 1 |
| chr6 | 11941721 | G | A | 112 | Phf14 | intronic | . | . | . | 6qA1 | rs240243837 | 0 | 1 |
| chr6 | 11953672 | C | A | 123 | Phf14 | intronic | . | . | . | 6qA1 | rs33376648 | 1 | 0 |
| chr6 | 11962812 | G | C | 222 | Phf14 | intronic | . | . | . | 6qA1 | rs33379170 | 0 | 1 |
| chr6 | 11962824 | G | C | 222 | Phf14 | intronic | . | . | . | 6qA1 | rs33379171 | 0 | 1 |
| chr6 | 11988552 | G | A | 131 | Phf14 | intronic | . | . | . | 6qA1 | rs33375368 | 0 | 1 |
| chr6 | 11988964 | G | A | 163 | Phf14 | intronic | . | . | . | 6qA1 | rs33376154 | 0 | 1 |
| chr6 | 11989966 | G | A | 158 | Phf14 | intronic | . | . | . | 6qA1 | rs235820913 | 0 | 1 |
| chr6 | 11996872 | G | A | 123 | Phf14 | intronic | . | . | . | 6qA1 | rs33379470 | 0 | 1 |
| chr6 | 12327720 | C | G | 138 | Thsd7a | intronic | . | . | . | 6qA1 | . | 0 | 1 |
| chr6 | 12337091 | A | G | 58 | Thsd7a | intronic | . | . | . | 6qA1 | . | 0 | 1 |
| chr6 | 12468984 | G | A | 144 | Thsd7a | exonic | stopgain | Thsd7a:NM_001164805:exon6:c.C1594T:p.R532X | . | 6qA1 | . | 0 | 1 |
| chr6 | 12857382 | T | G | 219 | . | intergenic | . | . | . | 6qA1 | rs29909768 | 0 | 1 |
| chr6 | 12857628 | A | T | 222 | . | intergenic | . | . | . | 6qA1 | rs250415555 | 0 | 1 |
| chr6 | 12858177 | T | G | 222 | . | intergenic | . | . | . | 6qA1 | rs37466150 | 0 | 1 |
| chr6 | 13055484 | T | C | 62 | . | intergenic | . | . | . | 6qA1 | . | 0 | 1 |
| chr6 | 13071705 | A | C | 222 | Tmem106b | intronic | . | . | . | 6qA1 | rs237113430 | 0 | 1 |
| chr6 | 13075000 | T | C | 222 | Tmem106b | intronic | . | . | . | 6qA1 | rs255566138 | 0 | 1 |
| chr6 | 13075005 | G | A | 222 | Tmem106b | intronic | . | . | . | 6qA1 | rs226388136 | 0 | 1 |
| chr6 | 13075299 | T | C | 94 | Tmem106b | intronic | . | . | . | 6qA1 | rs257585379 | 0 | 1 |
| chr6 | 13077937 | A | G | 130 | Tmem106b | intronic | . | . | . | 6qA1 | rs257003637 | 0 | 1 |
| chr6 | 13078218 | C | T | 222 | Tmem106b | exonic | synonymous SNV | Tmem106b:NM_027992:exon5:c.C411T:p.D137D | . | 6qA1 | rs216389822 | 0 | 1 |
| chr6 | 13081685 | G | A | 222 | Tmem106b | intronic | . | . | . | 6qA1 | rs263938690 | 0 | 1 |
| chr6 | 13081703 | G | T | 222 | Tmem106b | intronic | . | . | . | 6qA1 | rs579860207 | 0 | 1 |
| chr6 | 13081775 | T | C | 142 | Tmem106b | intronic | . | . | . | 6qA1 | rs37767682 | 0 | 1 |
| chr6 | 13081812 | T | C | 85 | Tmem106b | intronic | . | . | . | 6qA1 | rs241356387 | 0 | 1 |
| chr6 | 13082457 | C | T | 51 | Tmem106b | exonic | synonymous SNV | Tmem106b:NM_027992:exon7:c.C630T:p.Y210Y | . | 6qA1 | rs229353091 | 0 | 1 |
| chr6 | 13082470 | T | C | 76 | Tmem106b | intronic | . | . | . | 6qA1 | rs243696472 | 0 | 1 |
| chr6 | 13083325 | A | G | 131 | Tmem106b | intronic | . | . | . | 6qA1 | rs234907087 | 0 | 1 |
| chr6 | 13083355 | A | C | 222 | Tmem106b | intronic | . | . | . | 6qA1 | rs247962751 | 0 | 1 |
| chr6 | 13083399 | G | A | 222 | Tmem106b | exonic | synonymous SNV | Tmem106b:NM_027992:exon8:c.G666A:p.V222V | . | 6qA1 | rs218495845 | 0 | 1 |
| chr6 | 13083580 | G | A | 114 | Tmem106b | intronic | . | . | . | 6qA1 | rs38806149 | 0 | 1 |
| chr6 | 13084018 | T | C | 125 | Tmem106b | intronic | . | . | . | 6qA1 | rs251563530 | 0 | 1 |
| chr6 | 13084112 | A | G | 175 | Tmem106b | intronic | . | . | . | 6qA1 | rs216278868 | 0 | 1 |
| chr6 | 13084447 | T | C | 176 | Tmem106b | UTR3 | . | . | . | 6qA1 | rs39349632 | 0 | 1 |
| chr6 | 13084491 | C | G | 103 | Tmem106b | UTR3 | . | . | . | 6qA1 | rs36959190 | 0 | 1 |
| chr6 | 13166139 | C | T | 48.2753 | . | intergenic | . | . | . | 6qA1 | rs222113440 | 0 | 1 |
| chr6 | 13193399 | T | A | 125 | Vwde | intronic | . | . | . | 6qA1 | rs6317203 | 0 | 1 |
| chr6 | 14006224 | G | C | 65 | . | intergenic | . | . | . | 6qA1 | rs231185245 | 0 | 1 |
| chr6 | 14717533 | C | T | 137 | Ppp1r3a | UTR3 | . | . | . | 6qA1 | rs213558161 | 0 | 1 |
| chr6 | 14754854 | C | T | 111 | Ppp1r3a | exonic | synonymous SNV | Ppp1r3a:NM_080464:exon1:c.G393A:p.V131V | . | 6qA1 | . | 0 | 1 |
| chr6 | 15376947 | C | A | 135 | Foxp2 | intronic | . | . | . | 6qA1 | rs30057134 | 0 | 1 |
| chr6 | 15394556 | C | T | 150 | Foxp2 | intronic | . | . | . | 6qA1 | rs30116547 | 0 | 1 |
| chr6 | 15409997 | A | G | 222 | Foxp2 | intronic | . | . | . | 6qA1 | rs46662800 | 0 | 1 |
| chr6 | 15415680 | T | C | 37.7788 | Foxp2 | intronic | . | . | . | 6qA1 | rs51558245 | 0 | 1 |
| chr6 | 15770274 | C | T | 222 | Mdfic | exonic | missense SNV | Mdfic:NM_175088:exon4:c.C278T:p.T93I | . | 6qA1 | rs232168810 | 0 | 1 |
| chr6 | 16237712 | A | C | 221 | . | intergenic | . | . | . | 6qA1 | rs255040545 | 0 | 1 |
| chr6 | 16237804 | T | G | 221 | . | intergenic | . | . | . | 6qA1 | rs29921420 | 0 | 1 |
| chr6 | 17065200 | T | G | 221 | Tes | UTR5 | . | . | 543 | 6qA2 | rs33517304 | 0 | 1 |
| chr6 | 17065516 | C | T | 43.9904 | Tes | intronic | . | . | 543 | 6qA2 | . | 0 | 1 |
| chr6 | 17065517 | C | T | 43.9904 | Tes | intronic | . | . | 543 | 6qA2 | . | 0 | 1 |
| chr6 | 17085904 | A | C | 117 | Tes | intronic | . | . | . | 6qA2 | rs33515789 | 0 | 1 |
| chr6 | 17086050 | G | A | 222 | Tes | intronic | . | . | . | 6qA2 | rs212393384 | 0 | 1 |
| chr6 | 17096107 | T | C | 222 | Tes | intronic | . | . | . | 6qA2 | rs233268591 | 0 | 1 |
| chr6 | 17099584 | A | G | 111 | Tes | intronic | . | . | . | 6qA2 | rs33519650 | 0 | 1 |
| chr6 | 17099965 | C | G | 221 | Tes | intronic | . | . | . | 6qA2 | rs247822497 | 0 | 1 |
| chr6 | 17100029 | C | G | 208 | Tes | intronic | . | . | . | 6qA2 | rs33520514 | 0 | 1 |
| chr6 | 17100252 | G | A | 222 | Tes | intronic | . | . | . | 6qA2 | rs222569448 | 0 | 1 |
| chr6 | 17100493 | G | A | 221 | Tes | intronic | . | . | . | 6qA2 | rs242666674 | 0 | 1 |
| chr6 | 17100679 | C | T | 79 | Tes | intronic | . | . | . | 6qA2 | rs256160536 | 0 | 1 |
| chr6 | 17281435 | T | C | 221 | Cav2 | exonic | synonymous SNV | Cav2:NM_001277756:exon1:c.T75C:p.V25V,Cav2:NM_016900:exon1:c.T75C:p.V25V | . | 6qA2 | rs49453071 | 0 | 1 |
| chr6 | 17513119 | G | A | 58 | Met | intronic | . | . | . | 6qA2 | rs33560866 | 0 | 1 |
| chr6 | 17513207 | T | C | 124 | Met | intronic | . | . | . | 6qA2 | rs50167520 | 0 | 1 |
| chr6 | 17513264 | C | A | 155 | Met | intronic | . | . | . | 6qA2 | rs33560868 | 0 | 1 |
| chr6 | 17513370 | C | G | 222 | Met | exonic | synonymous SNV | Met:NM_008591:exon4:c.C1218G:p.S406S | . | 6qA2 | rs33554494 | 0 | 1 |
| chr6 | 17513618 | A | G | 222 | Met | intronic | . | . | . | 6qA2 | rs33554500 | 0 | 1 |
| chr6 | 17526164 | T | C | 102 | Met | intronic | . | . | . | 6qA2 | rs33559963 | 0 | 1 |
| chr6 | 17526204 | G | A | 145 | Met | intronic | . | . | . | 6qA2 | rs33560964 | 0 | 1 |
| chr6 | 17527237 | G | A | 222 | Met | exonic | synonymous SNV | Met:NM_008591:exon6:c.G1686A:p.P562P | . | 6qA2 | rs33563956 | 0 | 1 |
| chr6 | 17527272 | A | T | 222 | Met | intronic | . | . | . | 6qA2 | rs33563958 | 0 | 1 |
| chr6 | 17527307 | G | A | 167 | Met | intronic | . | . | . | 6qA2 | rs33563962 | 0 | 1 |
| chr6 | 17531172 | C | T | 73 | Met | intronic | . | . | . | 6qA2 | rs29832299 | 0 | 1 |
| chr6 | 17531381 | A | T | 222 | Met | intronic | . | . | . | 6qA2 | rs47734295 | 0 | 1 |
| chr6 | 17531593 | A | G | 222 | Met | intronic | . | . | . | 6qA2 | rs30451135 | 0 | 1 |
| chr6 | 17531597 | T | C | 222 | Met | intronic | . | . | . | 6qA2 | rs30996895 | 0 | 1 |
| chr6 | 17531749 | T | G | 126 | Met | intronic | . | . | . | 6qA2 | rs30031492 | 0 | 1 |
| chr6 | 17531755 | T | C | 125 | Met | intronic | . | . | . | 6qA2 | rs29921278 | 0 | 1 |
| chr6 | 17533666 | G | A | 22.0144 | Met | intronic | . | . | . | 6qA2 | rs33559836 | 0 | 1 |
| chr6 | 17533779 | C | A | 131 | Met | intronic | . | . | . | 6qA2 | rs50252147 | 0 | 1 |
| chr6 | 17533783 | G | A | 131 | Met | intronic | . | . | . | 6qA2 | rs50882693 | 0 | 1 |
| chr6 | 17533986 | G | A | 222 | Met | exonic | synonymous SNV | Met:NM_008591:exon8:c.G1935A:p.T645T | . | 6qA2 | rs249129433 | 0 | 1 |
| chr6 | 17533989 | G | A | 222 | Met | exonic | synonymous SNV | Met:NM_008591:exon8:c.G1938A:p.T646T | . | 6qA2 | rs33560494 | 0 | 1 |
| chr6 | 17534047 | G | A | 222 | Met | intronic | . | . | . | 6qA2 | rs33560495 | 0 | 1 |
| chr6 | 17534841 | C | G | 135 | Met | intronic | . | . | . | 6qA2 | rs30988991 | 0 | 1 |
| chr6 | 17534863 | A | G | 200 | Met | intronic | . | . | . | 6qA2 | rs30219684 | 0 | 1 |
| chr6 | 17535112 | A | G | 222 | Met | intronic | . | . | . | 6qA2 | rs29919957 | 0 | 1 |
| chr6 | 17535140 | A | G | 191 | Met | intronic | . | . | . | 6qA2 | rs256709933 | 0 | 1 |
| chr6 | 17535142 | G | C | 192 | Met | intronic | . | . | . | 6qA2 | rs222441971 | 0 | 1 |
| chr6 | 17535245 | T | C | 90 | Met | intronic | . | . | . | 6qA2 | rs30906091 | 0 | 1 |
| chr6 | 17535922 | T | C | 222 | Met | exonic | synonymous SNV | Met:NM_008591:exon11:c.T2346C:p.G782G | . | 6qA2 | rs46103250 | 0 | 1 |
| chr6 | 17535934 | A | G | 222 | Met | exonic | synonymous SNV | Met:NM_008591:exon11:c.A2358G:p.T786T | . | 6qA2 | rs49401199 | 0 | 1 |
| chr6 | 17535956 | A | C | 222 | Met | intronic | . | . | . | 6qA2 | rs49666166 | 0 | 1 |
| chr6 | 17536047 | G | A | 91 | Met | intronic | . | . | . | 6qA2 | rs30814339 | 0 | 1 |
| chr6 | 17536079 | A | G | 102 | Met | intronic | . | . | . | 6qA2 | rs52056299 | 0 | 1 |
| chr6 | 17540273 | C | A | 124 | Met | intronic | . | . | . | 6qA2 | rs33557873 | 0 | 1 |
| chr6 | 17540280 | T | A | 129 | Met | intronic | . | . | . | 6qA2 | rs52001375 | 0 | 1 |
| chr6 | 17540629 | A | G | 222 | Met | exonic | missense SNV | Met:NM_008591:exon12:c.A2553G:p.I851M | . | 6qA2 | rs33558729 | 0 | 1 |
| chr6 | 17540696 | T | C | 222 | Met | intronic | . | . | . | 6qA2 | rs33558731 | 0 | 1 |
| chr6 | 17540722 | A | G | 157 | Met | intronic | . | . | . | 6qA2 | rs33558733 | 0 | 1 |
| chr6 | 17546856 | T | C | 191 | Met | intronic | . | . | . | 6qA2 | rs33565847 | 0 | 1 |
| chr6 | 17548465 | G | A | 106 | Met | intronic | . | . | . | 6qA2 | rs213334064 | 0 | 1 |
| chr6 | 17548563 | C | T | 135 | Met | intronic | . | . | . | 6qA2 | rs33567531 | 0 | 1 |
| chr6 | 17548850 | T | C | 222 | Met | intronic | . | . | . | 6qA2 | rs227084773 | 0 | 1 |
| chr6 | 17548855 | A | G | 222 | Met | intronic | . | . | . | 6qA2 | rs250932544 | 0 | 1 |
| chr6 | 17553096 | T | C | 89 | Met | intronic | . | . | . | 6qA2 | rs33565077 | 0 | 1 |
| chr6 | 17553185 | G | A | 173 | Met | intronic | . | . | . | 6qA2 | rs50593757 | 0 | 1 |
| chr6 | 17553288 | C | T | 222 | Met | exonic | synonymous SNV | Met:NM_008591:exon16:c.C3072T:p.Y1024Y | . | 6qA2 | rs33565079 | 0 | 1 |
| chr6 | 17553318 | A | G | 222 | Met | exonic | synonymous SNV | Met:NM_008591:exon16:c.A3102G:p.T1034T | . | 6qA2 | rs33565081 | 0 | 1 |
| chr6 | 17553417 | A | T | 222 | Met | exonic | synonymous SNV | Met:NM_008591:exon16:c.A3201T:p.V1067V | . | 6qA2 | rs33565083 | 0 | 1 |
| chr6 | 17555306 | G | T | 99 | Met | intronic | . | . | . | 6qA2 | rs33568451 | 0 | 1 |
| chr6 | 17555508 | A | G | 221 | Met | intronic | . | . | . | 6qA2 | rs33568453 | 0 | 1 |
| chr6 | 17555623 | A | G | 222 | Met | exonic | synonymous SNV | Met:NM_008591:exon17:c.A3330G:p.L1110L | . | 6qA2 | rs33569285 | 0 | 1 |
| chr6 | 17558544 | A | G | 48.9516 | Met | intronic | . | . | . | 6qA2 | rs46934650 | 0 | 1 |
| chr6 | 17558562 | C | A | 72 | Met | intronic | . | . | . | 6qA2 | rs50900132 | 0 | 1 |

| | | | | | | | | | | | | | |
|---|---|---|---|---|---|---|---|---|---|---|---|---|---|
| chr6 | 17558573 | G | A | 83 | Met | intronic | . | . | . | 6qA2 | rs33560035 | 0 | 1 |
| chr6 | 17558927 | A | G | 222 | Met | intronic | . | . | . | 6qA2 | rs33560039 | 0 | 1 |
| chr6 | 17562121 | C | T | 222 | Met | intronic | . | . | . | 6qA2 | rs47266923 | 0 | 1 |
| chr6 | 17562143 | C | T | 222 | Met | intronic | . | . | . | 6qA2 | rs33562556 | 0 | 1 |
| chr6 | 17562305 | G | T | 222 | Met | intronic | . | . | . | 6qA2 | rs33562558 | 0 | 1 |
| chr6 | 17562345 | C | A | 183 | Met | intronic | . | . | . | 6qA2 | rs33562560 | 0 | 1 |
| chr6 | 17563371 | A | T | 122 | Met | intronic | . | . | . | 6qA2 | rs30999066 | 0 | 1 |
| chr6 | 17563662 | A | G | 222 | Met | exonic | synonymous SNV | Met:NM_008591:exon20:c.A3735G:p.V1245V | . | 6qA2 | rs30609098 | 0 | 1 |
| chr6 | 17563754 | A | G | 222 | Met | intronic | . | . | . | 6qA2 | rs30517920 | 0 | 1 |
| chr6 | 17563767 | G | A | 209 | Met | intronic | . | . | . | 6qA2 | rs30174777 | 0 | 1 |
| chr6 | 17563796 | G | A | 162 | Met | intronic | . | . | . | 6qA2 | rs30796494 | 0 | 1 |
| chr6 | 17563835 | C | T | 79 | Met | intronic | . | . | . | 6qA2 | rs30703047 | 0 | 1 |
| chr6 | 17571253 | C | A | 67 | Met | intronic | . | . | . | 6qA2 | rs48979393 | 0 | 1 |
| chr6 | 17571256 | C | T | 77 | Met | intronic | . | . | . | 6qA2 | rs50643361 | 0 | 1 |
| chr6 | 17571303 | A | G | 149 | Met | intronic | . | . | . | 6qA2 | rs33558134 | 0 | 1 |
| chr6 | 17571521 | T | C | 222 | Met | intronic | . | . | . | 6qA2 | rs33558136 | 0 | 1 |
| chr6 | 17571835 | T | C | 222 | Met | UTR3 | . | . | . | 6qA2 | rs33558140 | 0 | 1 |
| chr6 | 17643479 | C | T | 222 | Capza2 | intronic | . | . | . | 6qA2 | rs29930976 | 0 | 1 |
| chr6 | 17650113 | C | T | 222 | Capza2 | intronic | . | . | . | 6qA2 | rs45637665 | 0 | 1 |
| chr6 | 17654246 | G | A | 222 | Capza2 | intronic | . | . | . | 6qA2 | rs48310198 | 0 | 1 |
| chr6 | 17656607 | C | T | 154 | Capza2 | intronic | . | . | . | 6qA2 | rs216967836 | 0 | 1 |
| chr6 | 17657268 | A | G | 221 | Capza2 | intronic | . | . | . | 6qA2 | rs30023447 | 0 | 1 |
| chr6 | 17657278 | T | C | 221 | Capza2 | intronic | . | . | . | 6qA2 | rs264063493 | 0 | 1 |
| chr6 | 17665135 | G | C | 222 | Capza2 | intronic | . | . | . | 6qA2 | rs33573754 | 0 | 1 |
| chr6 | 17933972 | A | G | 128 | St7 | intronic | . | . | . | 6qA2 | rs29832951 | 0 | 1 |
| chr6 | 17933983 | A | C | 159 | St7 | intronic | . | . | . | 6qA2 | rs30212657 | 0 | 1 |
| chr6 | 17934255 | A | G | 222 | St7 | exonic | synonymous SNV | St7:NM_001083315:exon14:c.A1437G:p.K479K,St7:NM_001289626:exon14:c.A1566G:p.K522K,St7:NM_001 | . | 6qA2 | rs33590830 | 0 | 1 |
| chr6 | 17934312 | G | A | 222 | St7 | intronic | . | . | . | 6qA2 | rs213045151 | 0 | 1 |
| chr6 | 17942620 | T | C | 222 | St7 | intronic | . | . | . | 6qA2 | rs30016920 | 0 | 1 |
| chr6 | 18008431 | T | A | 120 | Wnt2 | intronic | . | . | . | 6qA2 | rs30710338 | 0 | 1 |
| chr6 | 18022900 | C | T | 101 | Wnt2 | intronic | . | . | . | 6qA2 | rs48028814 | 0 | 1 |
| chr6 | 18022931 | T | C | 149 | Wnt2 | intronic | . | . | . | 6qA2 | rs3157375 | 0 | 1 |
| chr6 | 18022953 | A | T | 174 | Wnt2 | intronic | . | . | . | 6qA2 | rs33595046 | 0 | 1 |
| chr6 | 18023331 | C | T | 222 | Wnt2 | exonic | synonymous SNV | Wnt2:NM_023653:exon3:c.G318A:p.R106R | . | 6qA2 | rs13462721 | 0 | 1 |
| chr6 | 18073786 | G | C | 222 | Asz1 | exonic | synonymous SNV | Asz1:NM_023729:exon9:c.C921G:p.T307T | . | 6qA2 | rs30019946 | 0 | 1 |
| chr6 | 18104849 | A | G | 221 | Asz1 | exonic | synonymous SNV | Asz1:NM_023729:exon3:c.T255C:p.Y85Y | . | 6qA2 | rs30364889 | 0 | 1 |
| chr6 | 18108144 | T | A | 63 | Asz1 | intronic | . | . | . | 6qA2 | rs30129555 | 0 | 1 |
| chr6 | 18144637 | A | G | 112 | . | intergenic | . | . | . | 6qA2 | rs3161259 | 0 | 1 |
| chr6 | 18144644 | T | A | 111 | . | intergenic | . | . | . | 6qA2 | rs47054320 | 0 | 1 |
| chr6 | 18144683 | C | G | 173 | . | intergenic | . | . | . | 6qA2 | rs3157291 | 0 | 1 |
| chr6 | 18144882 | A | G | 222 | . | intergenic | . | . | . | 6qA2 | rs47867777 | 0 | 1 |
| chr6 | 18144904 | T | C | 221 | . | intergenic | . | . | . | 6qA2 | rs48713858 | 0 | 1 |
| chr6 | 18144929 | A | C | 30.9316 | . | intergenic | . | . | . | 6qA2 | rs50349190 | 0 | 1 |
| chr6 | 18144971 | T | C | 70 | . | intergenic | . | . | . | 6qA2 | rs48107225 | 0 | 1 |
| chr6 | 18145004 | G | C | 222 | . | intergenic | . | . | . | 6qA2 | rs252707366 | 0 | 1 |
| chr6 | 18145058 | G | A | 222 | . | intergenic | . | . | . | 6qA2 | rs47050947 | 0 | 1 |
| chr6 | 18145132 | A | G | 222 | . | intergenic | . | . | . | 6qA2 | rs50011920 | 0 | 1 |
| chr6 | 18145191 | C | G | 222 | . | intergenic | . | . | . | 6qA2 | . | 0 | 1 |
| chr6 | 18145285 | G | T | 222 | . | intergenic | . | . | . | 6qA2 | rs50110544 | 0 | 1 |
| chr6 | 18145337 | G | A | 107 | . | intergenic | . | . | . | 6qA2 | rs50301132 | 0 | 1 |
| chr6 | 18145399 | C | T | 222 | . | intergenic | . | . | . | 6qA2 | rs51434696 | 0 | 1 |
| chr6 | 18145536 | A | G | 221 | . | intergenic | . | . | . | 6qA2 | rs50039551 | 0 | 1 |
| chr6 | 18145549 | C | G | 221 | . | intergenic | . | . | . | 6qA2 | rs47315674 | 0 | 1 |
| chr6 | 18145564 | T | C | 222 | . | intergenic | . | . | . | 6qA2 | rs51003355 | 0 | 1 |
| chr6 | 18145720 | C | T | 222 | . | intergenic | . | . | . | 6qA2 | rs3157292 | 0 | 1 |
| chr6 | 18145721 | T | C | 222 | . | intergenic | . | . | . | 6qA2 | rs3157293 | 0 | 1 |
| chr6 | 18145872 | T | C | 222 | . | intergenic | . | . | . | 6qA2 | rs3157294 | 0 | 1 |
| chr6 | 18156436 | G | A | 222 | . | intergenic | . | . | . | 6qA2 | rs50854642 | 0 | 1 |
| chr6 | 18156613 | C | T | 138 | . | intergenic | . | . | . | 6qA2 | rs49646490 | 0 | 1 |
| chr6 | 18156622 | T | G | 126 | . | intergenic | . | . | . | 6qA2 | rs47280431 | 0 | 1 |
| chr6 | 18156868 | T | C | 146 | . | intergenic | . | . | . | 6qA2 | rs48690243 | 0 | 1 |
| chr6 | 18170785 | T | C | 222 | Cftr | UTR5 | . | . | . | 6qA2 | rs3161301 | 0 | 1 |
| chr6 | 18198272 | G | A | 221 | Cftr | intronic | . | . | . | 6qA2 | rs46552716 | 0 | 1 |
| chr6 | 18198328 | G | A | 114 | Cftr | intronic | . | . | . | 6qA2 | rs47881088 | 0 | 1 |
| chr6 | 18198348 | C | T | 109 | Cftr | intronic | . | . | . | 6qA2 | rs48719213 | 0 | 1 |
| chr6 | 18214481 | G | A | 153 | Cftr | intronic | . | . | . | 6qA2 | rs47087998 | 1 | 0 |
| chr6 | 18226222 | G | T | 221 | Cftr | intronic | . | . | . | 6qA2 | rs30612377 | 0 | 1 |
| chr6 | 18227710 | T | C | 60 | Cftr | intronic | . | . | . | 6qA2 | rs30018515 | 0 | 1 |
| chr6 | 18228037 | G | T | 222 | Cftr | intronic | . | . | . | 6qA2 | rs30863920 | 0 | 1 |
| chr6 | 18228151 | G | A | 131 | Cftr | intronic | . | . | . | 6qA2 | rs30067129 | 0 | 1 |
| chr6 | 18234961 | A | G | 125 | Cftr | intronic | . | . | . | 6qA2 | rs47155600 | 0 | 1 |
| chr6 | 18274811 | G | A | 222 | Cftr | intronic | . | . | . | 6qA2 | rs50955376 | 0 | 1 |
| chr6 | 18277945 | A | G | 221 | Cftr | intronic | . | . | . | 6qA2 | rs49679982 | 0 | 1 |
| chr6 | 18282286 | A | G | 168 | Cftr | intronic | . | . | . | 6qA2 | rs257597097 | 0 | 1 |
| chr6 | 18282326 | C | G | 166 | Cftr | intronic | . | . | . | 6qA2 | rs226672412 | 0 | 1 |
| chr6 | 18282680 | G | A | 222 | Cftr | intronic | . | . | . | 6qA2 | rs252699232 | 0 | 1 |
| chr6 | 18294439 | A | G | 169 | Cftr | intronic | . | . | . | 6qA2 | rs30071203 | 0 | 1 |
| chr6 | 18306041 | A | G | 221 | Cftr | intronic | . | . | . | 6qA2 | rs30747314 | 0 | 1 |
| chr6 | 18319178 | A | G | 222 | Cftr | intronic | . | . | . | 6qA2 | rs30808209 | 0 | 1 |
| chr6 | 18375849 | A | C | 221 | Cttnbp2 | intronic | . | . | . | 6qA2 | rs36504198 | 0 | 1 |
| chr6 | 18376076 | C | A | 222 | Cttnbp2 | intronic | . | . | . | 6qA2 | rs36564370 | 0 | 1 |
| chr6 | 18376126 | T | C | 222 | Cttnbp2 | intronic | . | . | . | 6qA2 | rs36553735 | 0 | 1 |
| chr6 | 18378098 | G | A | 51 | Cttnbp2 | intronic | . | . | . | 6qA2 | rs36911184 | 0 | 1 |
| chr6 | 18378132 | T | C | 118 | Cttnbp2 | intronic | . | . | . | 6qA2 | rs36582363 | 0 | 1 |
| chr6 | 18378179 | G | A | 62 | Cttnbp2 | intronic | . | . | . | 6qA2 | rs586849689 | 0 | 1 |
| chr6 | 18378256 | C | A | 158 | Cttnbp2 | intronic | . | . | . | 6qA2 | rs226201258 | 0 | 1 |
| chr6 | 18378279 | A | G | 222 | Cttnbp2 | intronic | . | . | . | 6qA2 | rs37305317 | 0 | 1 |
| chr6 | 18378335 | G | T | 222 | Cttnbp2 | intronic | . | . | . | 6qA2 | rs37214919 | 0 | 1 |
| chr6 | 18378537 | C | A | 221 | Cttnbp2 | intronic | . | . | . | 6qA2 | rs254571599 | 0 | 1 |
| chr6 | 18378600 | G | T | 109 | Cttnbp2 | intronic | . | . | . | 6qA2 | rs36866726 | 0 | 1 |
| chr6 | 18378621 | A | C | 66 | Cttnbp2 | intronic | . | . | . | 6qA2 | rs36646805 | 0 | 1 |
| chr6 | 18380449 | T | C | 169 | Cttnbp2 | intronic | . | . | . | 6qA2 | rs243479820 | 0 | 1 |
| chr6 | 18382784 | C | A | 222 | Cttnbp2 | exonic | synonymous SNV | Cttnbp2:NM_080285:exon17:c.G3984T:p.S1328S | . | 6qA2 | rs37952997 | 0 | 1 |
| chr6 | 19784672 | C | G | 90 | . | intergenic | . | . | . | 6qA2 | . | 0 | 1 |
| chr6 | 21217106 | A | G | 147 | Kcnd2 | exonic | missense SNV | Kcnd2:NM_019697:exon1:c.A808G:p.I270V | . | 6qA2 | . | 0 | 1 |
| chr6 | 22059990 | A | G | 181 | Cped1 | intronic | . | . | . | 6qA3.1 | . | 0 | 1 |
| chr6 | 22329392 | G | A | 53 | Fam3c | intronic | . | . | . | 6qA3.1 | . | 0 | 1 |
| chr6 | 22987341 | T | C | 72 | Ptprz1 | exonic | missense SNV | Ptprz1:NM_001081306:exon10:c.T1124C:p.L375P,Ptprz1:NM_001311064:exon10:c.T1124C:p.L375P | . | 6qA3.1 | . | 0 | 1 |
| chr6 | 22987456 | C | T | 221 | Ptprz1 | exonic | synonymous SNV | Ptprz1:NM_001081306:exon10:c.C1239T:p.A413A,Ptprz1:NM_001311064:exon10:c.C1239T:p.A413A | . | 6qA3.1 | rs33745356 | 0 | 1 |
| chr6 | 22994756 | A | C | 40.901 | Ptprz1 | intronic | . | . | . | 6qA3.1 | rs33742064 | 0 | 1 |
| chr6 | 23000007 | G | A | 221 | Ptprz1 | exonic | missense SNV | Ptprz1:NM_001081306:exon12:c.G2095A:p.E699K,Ptprz1:NM_001311064:exon12:c.G2095A:p.E699K | . | 6qA3.1 | rs33747298 | 0 | 1 |
| chr6 | 23001555 | A | G | 221 | Ptprz1 | exonic | missense SNV | Ptprz1:NM_001081306:exon12:c.A3643G:p.I1215V | . | 6qA3.1 | rs33748864 | 0 | 1 |
| chr6 | 23002166 | C | T | 220 | Ptprz1 | exonic | synonymous SNV | Ptprz1:NM_001081306:exon12:c.C4254T:p.A1418A | . | 6qA3.1 | rs251352506 | 0 | 1 |
| chr6 | 23015928 | G | T | 65 | Ptprz1 | intronic | . | . | . | 6qA3.1 | rs229362019 | 0 | 1 |
| chr6 | 23016250 | G | A | 221 | Ptprz1 | intronic | . | . | . | 6qA3.1 | rs231792954 | 0 | 1 |
| chr6 | 23016350 | A | T | 99 | Ptprz1 | intronic | . | . | . | 6qA3.1 | rs262649570 | 0 | 1 |
| chr6 | 23016362 | G | A | 80 | Ptprz1 | intronic | . | . | . | 6qA3.1 | rs216094544 | 0 | 1 |
| chr6 | 23016386 | T | A | 92 | Ptprz1 | intronic | . | . | . | 6qA3.1 | rs33746100 | 0 | 1 |
| chr6 | 23022161 | C | T | 221 | Ptprz1 | intronic | . | . | . | 6qA3.1 | rs257553833 | 0 | 1 |
| chr6 | 23022315 | A | T | 221 | Ptprz1 | intronic | . | . | . | 6qA3.1 | rs215433860 | 0 | 1 |
| chr6 | 23022346 | T | C | 222 | Ptprz1 | intronic | . | . | . | 6qA3.1 | rs227317467 | 0 | 1 |
| chr6 | 23024896 | A | G | 222 | Ptprz1 | intronic | . | . | . | 6qA3.1 | rs33742460 | 0 | 1 |
| chr6 | 23024933 | C | T | 209 | Ptprz1 | intronic | . | . | . | 6qA3.1 | rs33742462 | 0 | 1 |
| chr6 | 23024940 | T | C | 222 | Ptprz1 | intronic | . | . | . | 6qA3.1 | rs215819138 | 0 | 1 |
| chr6 | 23025049 | C | T | 221 | Ptprz1 | intronic | . | . | . | 6qA3.1 | rs33743226 | 0 | 1 |
| chr6 | 23035386 | G | A | 85 | Ptprz1 | intronic | . | . | . | 6qA3.1 | rs215396020 | 0 | 1 |
| chr6 | 23043047 | C | T | 115 | Ptprz1 | intronic | . | . | . | 6qA3.1 | rs33748368 | 1 | 0 |
| chr6 | 23044204 | A | T | 79 | Ptprz1 | intronic | . | . | . | 6qA3.1 | rs33750858 | 0 | 1 |
| chr6 | 23044212 | G | T | 128 | Ptprz1 | intronic | . | . | . | 6qA3.1 | rs47383859 | 0 | 1 |
| chr6 | 23044249 | C | T | 222 | Ptprz1 | exonic | synonymous SNV | Ptprz1:NM_001081306:exon26:c.C6246T:p.G2082G,Ptprz1:NM_001311064:exon26:c.C3699T:p.G1233G | . | 6qA3.1 | rs33750860 | 0 | 1 |
| chr6 | 23044521 | T | G | 112 | Ptprz1 | intronic | . | . | . | 6qA3.1 | rs33750862 | 0 | 1 |
| chr6 | 23044549 | A | T | 73 | Ptprz1 | intronic | . | . | . | 6qA3.1 | rs33751604 | 0 | 1 |
| chr6 | 23045777 | A | T | 222 | Ptprz1 | intronic | . | . | . | 6qA3.1 | rs261975458 | 0 | 1 |
| chr6 | 23045788 | T | C | 222 | Ptprz1 | intronic | . | . | . | 6qA3.1 | rs230721305 | 0 | 1 |
| chr6 | 23045791 | A | G | 222 | Ptprz1 | intronic | . | . | . | 6qA3.1 | rs33753058 | 0 | 1 |
| chr6 | 23046027 | G | C | 85 | Ptprz1 | intronic | . | . | . | 6qA3.1 | rs248243852 | 0 | 1 |
| chr6 | 23049457 | T | G | 201 | Ptprz1 | intronic | . | . | . | 6qA3.1 | rs33746653 | 0 | 1 |
| chr6 | 23051998 | A | G | 221 | Ptprz1 | exonic | synonymous SNV | Ptprz1:NM_001081306:exon30:c.A6825G:p.V2275V,Ptprz1:NM_001311064:exon30:c.A4278G:p.V1426V | . | 6qA3.1 | rs33747443 | 0 | 1 |
| chr6 | 23073059 | G | T | 222 | Aass | exonic | synonymous SNV | Aass:NM_013930:exon24:c.C2694A:p.P898P | . | 6qA3.1 | rs13474630 | 0 | 1 |
| chr6 | 23073211 | G | T | 82 | Aass | intronic | . | . | . | 6qA3.1 | rs261573977 | 0 | 1 |
| chr6 | 23074595 | G | A | 221 | Aass | exonic | synonymous SNV | Aass:NM_013930:exon23:c.C2595T:p.N865N | . | 6qA3.1 | rs13474631 | 0 | 1 |
| chr6 | 23074757 | C | T | 221 | Aass | intronic | . | . | . | 6qA3.1 | rs245461601 | 0 | 1 |
| chr6 | 23074759 | C | T | 221 | Aass | intronic | . | . | . | 6qA3.1 | rs262526566 | 0 | 1 |
| chr6 | 23074782 | T | C | 184 | Aass | intronic | . | . | . | 6qA3.1 | rs220259671 | 0 | 1 |
| chr6 | 23075062 | C | G | 172 | Aass | intronic | . | . | . | 6qA3.1 | rs33747207 | 0 | 1 |
| chr6 | 23075071 | C | T | 222 | Aass | intronic | . | . | . | 6qA3.1 | rs213657826 | 0 | 1 |
| chr6 | 23075103 | T | C | 222 | Aass | intronic | . | . | . | 6qA3.1 | rs232655951 | 0 | 1 |
| chr6 | 23075214 | C | G | 222 | Aass | exonic | synonymous SNV | Aass:NM_013930:exon22:c.G2433C:p.A811A | . | 6qA3.1 | rs13474632 | 0 | 1 |
| chr6 | 23075268 | C | G | 222 | Aass | intronic | . | . | . | 6qA3.1 | rs33747209 | 0 | 1 |
| chr6 | 23075328 | G | C | 222 | Aass | intronic | . | . | . | 6qA3.1 | rs107806532 | 0 | 1 |
| chr6 | 23075391 | T | G | 85 | Aass | intronic | . | . | . | 6qA3.1 | rs107867304 | 0 | 1 |
| chr6 | 23075500 | T | A | 75 | Aass | intronic | . | . | . | 6qA3.1 | rs33748075 | 0 | 1 |
| chr6 | 23075541 | C | G | 134 | Aass | intronic | . | . | . | 6qA3.1 | rs33748077 | 0 | 1 |
| chr6 | 23075623 | T | A | 190 | Aass | intronic | . | . | . | 6qA3.1 | rs33748079 | 0 | 1 |
| chr6 | 23075890 | C | T | 222 | Aass | intronic | . | . | . | 6qA3.1 | rs221307038 | 0 | 1 |
| chr6 | 23075974 | T | A | 95 | Aass | intronic | . | . | . | 6qA3.1 | rs249857009 | 0 | 1 |
| chr6 | 23076849 | A | C | 117 | Aass | intronic | . | . | . | 6qA3.1 | rs33748969 | 0 | 1 |
| chr6 | 23076896 | C | T | 123 | Aass | intronic | . | . | . | 6qA3.1 | rs228711911 | 0 | 1 |
| chr6 | 23077029 | T | C | 221 | Aass | intronic | . | . | . | 6qA3.1 | rs260363647 | 0 | 1 |
| chr6 | 23077243 | G | C | 181 | Aass | intronic | . | . | . | 6qA3.1 | rs579715987 | 0 | 1 |
| chr6 | 23078915 | G | C | 221 | Aass | exonic | synonymous SNV | Aass:NM_013930:exon19:c.C2097G:p.G699G | . | 6qA3.1 | rs233215513 | 0 | 1 |
| chr6 | 23091394 | T | C | 176 | Aass | intronic | . | . | . | 6qA3.1 | rs227314634 | 0 | 1 |
| chr6 | 23092034 | A | C | 79 | Aass | intronic | . | . | . | 6qA3.1 | rs33748736 | 0 | 1 |
| chr6 | 23092121 | T | C | 97 | Aass | intronic | . | . | . | 6qA3.1 | rs33748738 | 0 | 1 |
| chr6 | 23092183 | C | T | 92 | Aass | intronic | . | . | . | 6qA3.1 | rs50110973 | 0 | 1 |
| chr6 | 23092189 | T | G | 84 | Aass | intronic | . | . | . | 6qA3.1 | rs33748740 | 0 | 1 |
| chr6 | 23092246 | T | C | 221 | Aass | intronic | . | . | . | 6qA3.1 | rs45737125 | 0 | 1 |
| chr6 | 23092319 | A | G | 220 | Aass | exonic | synonymous SNV | Aass:NM_013930:exon17:c.T1821C:p.L607L | . | 6qA3.1 | rs33748742 | 0 | 1 |
| chr6 | 23092496 | A | G | 221 | Aass | exonic | synonymous SNV | Aass:NM_013930:exon16:c.T1734C:p.Y578Y | . | 6qA3.1 | rs33749566 | 0 | 1 |
| chr6 | 23092544 | C | T | 220 | Aass | exonic | synonymous SNV | Aass:NM_013930:exon16:c.G1686A:p.V562V | . | 6qA3.1 | rs33749568 | 0 | 1 |
| chr6 | 23106928 | A | G | 113 | Aass | intronic | . | . | . | 6qA3.1 | rs33751486 | 0 | 1 |
| chr6 | 23107356 | A | G | 208 | Aass | intronic | . | . | . | 6qA3.1 | rs220416290 | 0 | 1 |
| chr6 | 23107405 | C | T | 219 | Aass | intronic | . | . | . | 6qA3.1 | rs241792662 | 0 | 1 |
| chr6 | 23107434 | T | C | 143 | Aass | intronic | . | . | . | 6qA3.1 | rs242452398 | 0 | 1 |
| chr6 | 23107547 | C | G | 221 | Aass | intronic | . | . | . | 6qA3.1 | rs254434247 | 0 | 1 |
| chr6 | 23107773 | A | G | 222 | Aass | intronic | . | . | . | 6qA3.1 | rs225004492 | 0 | 1 |
| chr6 | 23107784 | G | T | 222 | Aass | intronic | . | . | . | 6qA3.1 | rs244307013 | 0 | 1 |
| chr6 | 23113391 | T | C | 220 | Aass | intronic | . | . | . | 6qA3.1 | rs233046674 | 0 | 1 |
| chr6 | 23114713 | G | A | 133 | Aass | intronic | . | . | . | 6qA3.1 | rs220845940 | 0 | 1 |
| chr6 | 23114952 | G | A | 221 | Aass | exonic | synonymous SNV | Aass:NM_013930:exon6:c.C576T:p.S192S | . | 6qA3.1 | rs33749048 | 0 | 1 |
| chr6 | 23115188 | T | G | 221 | Aass | intronic | . | . | . | 6qA3.1 | rs33749052 | 0 | 1 |
| chr6 | 23115358 | G | A | 102 | Aass | intronic | . | . | . | 6qA3.1 | rs219209978 | 0 | 1 |
| chr6 | 23118444 | T | C | 105 | Aass | intronic | . | . | . | 6qA3.1 | rs212775404 | 0 | 1 |
| chr6 | 23118480 | A | G | 98 | Aass | intronic | . | . | . | 6qA3.1 | rs231029286 | 0 | 1 |
| chr6 | 23118571 | T | C | 113 | Aass | intronic | . | . | . | 6qA3.1 | rs250029150 | 0 | 1 |
| chr6 | 23118573 | C | T | 113 | Aass | intronic | . | . | . | 6qA3.1 | rs220270757 | 0 | 1 |
| chr6 | 23118768 | A | G | 221 | Aass | intronic | . | . | . | 6qA3.1 | rs33751321 | 0 | 1 |
| chr6 | 23120767 | C | A | 132 | Aass | intronic | . | . | . | 6qA3.1 | rs33752921 | 0 | 1 |
| chr6 | 23120949 | C | T | 221 | Aass | exonic | synonymous SNV | Aass:NM_013930:exon3:c.G315A:p.T105T | . | 6qA3.1 | rs33753615 | 0 | 1 |
| chr6 | 23246073 | T | C | 220 | Fezf1 | exonic | synonymous SNV | Fezf1:NM_028462:exon4:c.A1092G:p.L364L | . | 6qA3.1 | rs33753916 | 0 | 1 |
| chr6 | 23246612 | T | C | 221 | Fezf1 | exonic | synonymous SNV | Fezf1:NM_028462:exon3:c.A972G:p.A324A | . | 6qA3.1 | rs3669430 | 0 | 1 |
| chr6 | 23246691 | A | G | 221 | Fezf1 | intronic | . | . | . | 6qA3.1 | rs3684277 | 0 | 1 |
| chr6 | 23247844 | G | A | 221 | Fezf1 | exonic | synonymous SNV | Fezf1:NM_028462:exon1:c.C231T:p.F77F | 2271 | 6qA3.1 | rs33756275 | 0 | 1 |
| chr6 | 23248000 | T | C | 222 | Fezf1 | exonic | synonymous SNV | Fezf1:NM_028462:exon1:c.A75G:p.T25T | 2271 | 6qA3.1 | rs33756279 | 0 | 1 |
| chr6 | 23248181 | G | A | 125 | Fezf1 | UTR5 | . | . | 2271 | 6qA3.1 | . | 0 | 1 |

| | | | | | | | | | | | | | |
|---|---|---|---|---|---|---|---|---|---|---|---|---|---|
| chr6 | 23263771 | T | C | 221 | Cadps2 | intronic | . | . | . | 6qA3.1 | rs33760486 | 0 | 1 |
| chr6 | 23321586 | T | C | 117 | Cadps2 | intronic | . | . | . | 6qA3.1 | rs30068465 | 0 | 1 |
| chr6 | 23323315 | T | C | 221 | Cadps2 | exonic | synonymous SNV | Cadps2:NM_001252110:exon20:c.A3003G:p.L1001L,Cadps2:NM_001252106:exon21:c.A2832G:p.L944L,Cadr | . | 6qA3.1 | rs30999238 | 0 | 1 |
| chr6 | 23323475 | C | T | 221 | Cadps2 | intronic | . | . | . | 6qA3.1 | rs29917628 | 0 | 1 |
| chr6 | 23354137 | T | A | 217 | Cadps2 | intronic | . | . | . | 6qA3.1 | rs51577972 | 0 | 1 |
| chr6 | 23354294 | G | A | 221 | Cadps2 | intronic | . | . | . | 6qA3.1 | rs30902772 | 0 | 1 |
| chr6 | 23354438 | C | T | 45.8132 | Cadps2 | intronic | . | . | . | 6qA3.1 | rs30699018 | 0 | 1 |
| chr6 | 23356048 | T | C | 168 | Cadps2 | intronic | . | . | . | 6qA3.1 | rs30957165 | 0 | 1 |
| chr6 | 23385568 | T | C | 221 | Cadps2 | intronic | . | . | . | 6qA3.1 | rs48374104 | 0 | 1 |
| chr6 | 23385612 | G | A | 221 | Cadps2 | intronic | . | . | . | 6qA3.1 | rs45834056 | 0 | 1 |
| chr6 | 23412926 | A | G | 221 | Cadps2 | intronic | . | . | . | 6qA3.1 | rs30707610 | 0 | 1 |
| chr6 | 23427462 | C | T | 219 | Cadps2 | intronic | . | . | . | 6qA3.1 | rs30257384 | 0 | 1 |
| chr6 | 23496951 | T | G | 221 | Cadps2 | intronic | . | . | . | 6qA3.1 | rs30111053 | 0 | 1 |
| chr6 | 23583259 | A | G | 49.1386 | Cadps2 | intronic | . | . | . | 6qA3.1 | . | 0 | 1 |
| chr6 | 23599681 | C | T | 107 | Cadps2 | intronic | . | . | . | 6qA3.1 | rs36412263 | 0 | 1 |
| chr6 | 23654177 | A | G | 221 | Rnf148,Cadps2 | exonic | synonymous SNV | Rnf148:NM_027754:exon1:c.T819C:p.F273F | . | 6qA3.1 | rs31000439 | 0 | 1 |
| chr6 | 23688667 | T | G | 66 | Cadps2 | intronic | . | . | . | 6qA3.1 | . | 0 | 1 |
| chr6 | 23838730 | G | A | 124 | Cadps2 | intronic | . | . | 935 | 6qA3.1 | rs211973591 | 0 | 1 |
| chr6 | 23838745 | A | G | 134 | Cadps2 | intronic | . | . | 935 | 6qA3.1 | rs30758424 | 0 | 1 |
| chr6 | 23838761 | G | A | 173 | Cadps2 | intronic | . | . | 935 | 6qA3.1 | rs217203797 | 0 | 1 |
| chr6 | 23839055 | A | C | 43.9904 | Cadps2 | exonic | missense SNV | Cadps2:NM_001252105:exon1:c.T83G:p.V28G,Cadps2:NM_001252106:exon1:c.T83G:p.V28G,Cadps2:NM_001 | 935 | 6qA3.1 | rs249879236 | 0 | 1 |
| chr6 | 23969070 | G | A | 207 | . | downstream | . | . | . | 6qA3.1 | rs30169384 | 0 | 1 |
| chr6 | 23969136 | T | C | 221 | . | downstream | . | . | . | 6qA3.1 | rs30869991 | 0 | 1 |
| chr6 | 23970141 | T | C | 221 | . | upstream | . | . | . | 6qA3.1 | rs30022853 | 0 | 1 |
| chr6 | 23970174 | T | C | 106 | . | upstream | . | . | . | 6qA3.1 | rs30172465 | 0 | 1 |
| chr6 | 23970192 | A | C | 94 | . | upstream | . | . | . | 6qA3.1 | rs29828241 | 0 | 1 |
| chr6 | 24088760 | G | A | 164 | Slc13a1 | UTR3 | . | . | . | 6qA3.1 | rs37157094 | 1 | 0 |
| chr6 | 24092084 | C | T | 124 | Slc13a1 | intronic | . | . | . | 6qA3.1 | rs30609931 | 0 | 1 |
| chr6 | 24097388 | C | T | 72 | Slc13a1 | intronic | . | . | . | 6qA3.1 | rs30667719 | 0 | 1 |
| chr6 | 24103239 | C | T | 126 | Slc13a1 | intronic | . | . | . | 6qA3.1 | rs30942654 | 0 | 1 |
| chr6 | 24103271 | A | G | 124 | Slc13a1 | intronic | . | . | . | 6qA3.1 | rs30362304 | 0 | 1 |
| chr6 | 24103390 | A | C | 221 | Slc13a1 | intronic | . | . | . | 6qA3.1 | rs38759439 | 0 | 1 |
| chr6 | 24104054 | T | C | 222 | Slc13a1 | exonic | missense SNV | Slc13a1:NM_019481:exon9:c.A988G:p.K330E | . | 6qA3.1 | rs38009804 | 0 | 1 |
| chr6 | 24104055 | T | G | 222 | Slc13a1 | exonic | synonymous SNV | Slc13a1:NM_019481:exon9:c.A987C:p.A329A | . | 6qA3.1 | rs233200675 | 0 | 1 |
| chr6 | 24104197 | T | G | 90 | Slc13a1 | intronic | . | . | . | 6qA3.1 | rs30421254 | 0 | 1 |
| chr6 | 24108037 | G | A | 154 | Slc13a1 | intronic | . | . | . | 6qA3.1 | rs30305830 | 0 | 1 |
| chr6 | 24108071 | C | A | 179 | Slc13a1 | intronic | . | . | . | 6qA3.1 | rs30605866 | 0 | 1 |
| chr6 | 24108446 | C | T | 124 | Slc13a1 | intronic | . | . | . | 6qA3.1 | rs29918103 | 0 | 1 |
| chr6 | 24133592 | C | A | 220 | Slc13a1 | intronic | . | . | . | 6qA3.1 | rs30320842 | 0 | 1 |
| chr6 | 24134134 | T | A | 99 | Slc13a1 | intronic | . | . | . | 6qA3.1 | rs30564676 | 0 | 1 |
| chr6 | 24134563 | T | C | 221 | Slc13a1 | intronic | . | . | . | 6qA3.1 | rs30664086 | 0 | 1 |
| chr6 | 24134617 | A | G | 222 | Slc13a1 | intronic | . | . | . | 6qA3.1 | rs36550827 | 0 | 1 |
| chr6 | 24136878 | A | C | 52 | Slc13a1 | intronic | . | . | . | 6qA3.1 | rs30073224 | 0 | 1 |
| chr6 | 24136908 | C | T | 85 | Slc13a1 | intronic | . | . | . | 6qA3.1 | rs30461493 | 0 | 1 |
| chr6 | 24136943 | A | G | 135 | Slc13a1 | intronic | . | . | . | 6qA3.1 | rs30521898 | 0 | 1 |
| chr6 | 24136957 | C | A | 162 | Slc13a1 | intronic | . | . | . | 6qA3.1 | rs37137258 | 0 | 1 |
| chr6 | 24137411 | A | T | 96 | Slc13a1 | intronic | . | . | . | 6qA3.1 | rs30014221 | 0 | 1 |
| chr6 | 24139539 | G | A | 33.8563 | Slc13a1 | intronic | . | . | . | 6qA3.1 | rs48724548 | 0 | 1 |
| chr6 | 24139605 | G | A | 135 | Slc13a1 | intronic | . | . | . | 6qA3.1 | rs50411844 | 0 | 1 |
| chr6 | 24139831 | T | C | 71 | Slc13a1 | intronic | . | . | . | 6qA3.1 | rs50361725 | 0 | 1 |
| chr6 | 24446161 | C | G | 221 | Iqub | exonic | missense SNV | Iqub:NM_172535:exon13:c.G2263C:p.A755P | . | 6qA3.1 | . | 0 | 1 |
| chr6 | 24454045 | G | A | 41.2181 | Iqub | intronic | . | . | . | 6qA3.1 | rs46703466 | 0 | 1 |
| chr6 | 24522886 | C | A | 21.8574 | Ndufa5 | intronic | . | . | . | 6qA3.1 | . | 0 | 1 |
| chr6 | 24562656 | A | G | 193 | Asb15 | exonic | missense SNV | Asb15:NM_080847:exon6:c.A616G:p.R206G | . | 6qA3.1 | . | 0 | 1 |
| chr6 | 24876058 | T | C | 71 | Hyal5 | UTR5 | . | . | . | 6qA3.1 | . | 0 | 1 |
| chr6 | 24954788 | G | A | 129 | Tmem229a | exonic | synonymous SNV | Tmem229a:NM_177013:exon1:c.C966T:p.C322C | . | 6qA3.1 | . | 0 | 1 |
| chr6 | 25047126 | A | G | 221 | . | intergenic | . | . | . | 6qA3.1 | . | 0 | 1 |
| chr6 | 25666898 | A | T | 211 | . | intergenic | . | . | . | 6qA3.1 | rs30790978 | 0 | 1 |
| chr6 | 25666899 | A | C | 204 | . | intergenic | . | . | . | 6qA3.1 | rs30559990 | 0 | 1 |
| chr6 | 25744559 | C | T | 222 | Pot1a | UTR3 | . | . | . | 6qA3.1 | rs217280876 | 0 | 1 |
| chr6 | 25748135 | T | G | 128 | Pot1a | intronic | . | . | . | 6qA3.1 | rs51181967 | 0 | 1 |
| chr6 | 25749960 | G | C | 221 | Pot1a | intronic | . | . | . | 6qA3.1 | rs265731009 | 0 | 1 |
| chr6 | 25753438 | A | T | 190 | Pot1a | intronic | . | . | . | 6qA3.1 | rs244168427 | 0 | 1 |
| chr6 | 25771756 | G | A | 221 | Pot1a | intronic | . | . | . | 6qA3.1 | rs47911814 | 0 | 1 |
| chr6 | 25775509 | C | A | 221 | Pot1a | intronic | . | . | . | 6qA3.1 | rs49930140 | 0 | 1 |
| chr6 | 25778616 | C | T | 96 | Pot1a | intronic | . | . | . | 6qA3.1 | rs3667234 | 0 | 1 |
| chr6 | 25778624 | T | G | 111 | Pot1a | intronic | . | . | . | 6qA3.1 | rs3667240 | 0 | 1 |
| chr6 | 25795077 | C | T | 222 | Pot1a | intronic | . | . | . | 6qA3.1 | rs48934776 | 0 | 1 |
| chr6 | 25795098 | C | G | 222 | Pot1a | intronic | . | . | . | 6qA3.1 | rs49901585 | 0 | 1 |
| chr6 | 27936635 | T | C | 221 | Grm8 | intronic | . | . | . | 6qA3.2 | rs33810050 | 0 | 1 |
| chr6 | 28244227 | A | G | 222 | Zfp800 | exonic | synonymous SNV | Zfp800:NM_001081678:exon5:c.T738C:p.V246V | . | 6qA3.2 | rs4225722 | 0 | 1 |
| chr6 | 28361781 | A | G | 47.7041 | . | intergenic | . | . | . | 6qA3.2 | rs33820927 | 0 | 1 |
| chr6 | 28361859 | G | A | 58 | . | intergenic | . | . | . | 6qA3.2 | . | 0 | 1 |
| chr6 | 28361933 | A | G | 221 | . | intergenic | . | . | . | 6qA3.2 | rs33820929 | 0 | 1 |
| chr6 | 28361973 | C | G | 221 | . | intergenic | . | . | . | 6qA3.2 | rs229492818 | 0 | 1 |
| chr6 | 28361977 | A | G | 221 | . | intergenic | . | . | . | 6qA3.2 | rs242728781 | 0 | 1 |
| chr6 | 28362579 | C | T | 159 | . | intergenic | . | . | . | 6qA3.2 | rs213730818 | 0 | 1 |
| chr6 | 28362637 | G | A | 213 | . | intergenic | . | . | . | 6qA3.2 | rs33821731 | 0 | 1 |
| chr6 | 28362745 | A | G | 220 | . | intergenic | . | . | . | 6qA3.2 | rs33821733 | 0 | 1 |
| chr6 | 28362870 | T | A | 221 | . | intergenic | . | . | . | 6qA3.2 | . | 0 | 1 |
| chr6 | 28362933 | A | G | 221 | . | intergenic | . | . | . | 6qA3.2 | rs33822675 | 0 | 1 |
| chr6 | 28362963 | A | G | 192 | . | intergenic | . | . | . | 6qA3.2 | rs237124243 | 0 | 1 |
| chr6 | 28363060 | T | A | 164 | . | intergenic | . | . | . | 6qA3.2 | rs33822677 | 0 | 1 |
| chr6 | 28417956 | C | T | 195 | Gcc1 | UTR3 | . | . | . | 6qA3.3 | . | 0 | 1 |
| chr6 | 28418133 | G | A | 221 | Gcc1 | exonic | synonymous SNV | Gcc1:NM_028900:exon2:c.C2200T:p.L734L | . | 6qA3.3 | rs33825236 | 0 | 1 |
| chr6 | 28424384 | A | C | 221 | Arf5 | intronic | . | . | . | 6qA3.3 | rs248296025 | 0 | 1 |
| chr6 | 28424891 | C | G | 221 | Arf5 | intronic | . | . | . | 6qA3.3 | rs219708876 | 0 | 1 |
| chr6 | 28426064 | C | T | 101 | Arf5 | intronic | . | . | . | 6qA3.3 | rs214285529 | 0 | 1 |
| chr6 | 28435401 | A | C | 164 | Fscn3 | intronic | . | . | . | 6qA3.3 | rs30213535 | 0 | 1 |
| chr6 | 28435738 | T | C | 220 | Fscn3 | intronic | . | . | . | 6qA3.3 | rs254617657 | 0 | 1 |
| chr6 | 28442363 | T | C | 221 | Pax4 | UTR3 | . | . | . | 6qA3.3 | rs33823771 | 0 | 1 |
| chr6 | 28442486 | G | T | 221 | Pax4 | exonic | synonymous SNV | Pax4:NM_001159925:exon11:c.C903A:p.S301S,Pax4:NM_001159926:exon11:c.C900A:p.S300S,Pax4:NM_011 | . | 6qA3.3 | . | 0 | 1 |
| chr6 | 28513080 | C | T | 120 | Snd1 | intronic | . | . | . | 6qA3.3 | rs219177682 | 0 | 1 |
| chr6 | 28520030 | C | T | 175 | Snd1 | intronic | . | . | . | 6qA3.3 | rs30936662 | 0 | 1 |
| chr6 | 28523602 | T | A | 130 | Snd1 | intronic | . | . | . | 6qA3.3 | rs33824756 | 0 | 1 |
| chr6 | 28526847 | G | A | 217 | Snd1 | intronic | . | . | . | 6qA3.3 | . | 0 | 1 |
| chr6 | 28527013 | A | G | 222 | Snd1 | intronic | . | . | . | 6qA3.3 | . | 0 | 1 |
| chr6 | 28527093 | T | C | 131 | Snd1 | intronic | . | . | . | 6qA3.3 | rs33828632 | 0 | 1 |
| chr6 | 28527751 | C | A | 222 | Snd1 | intronic | . | . | . | 6qA3.3 | rs30217570 | 0 | 1 |
| chr6 | 28527873 | A | T | 56 | Snd1 | intronic | . | . | . | 6qA3.3 | . | 0 | 1 |
| chr6 | 28531610 | C | T | 116 | Snd1 | intronic | . | . | . | 6qA3.3 | . | 0 | 1 |
| chr6 | 28545468 | C | T | 222 | Snd1 | intronic | . | . | . | 6qA3.3 | rs29875107 | 0 | 1 |
| chr6 | 28625983 | T | C | 123 | Snd1 | intronic | . | . | . | 6qA3.3 | rs33837766 | 0 | 1 |
| chr6 | 28706913 | C | T | 109 | Snd1 | intronic | . | . | . | 6qA3.3 | rs258078101 | 0 | 1 |
| chr6 | 28706970 | C | T | 140 | Snd1 | intronic | . | . | . | 6qA3.3 | rs33832335 | 0 | 1 |
| chr6 | 28707012 | C | T | 221 | Snd1 | intronic | . | . | . | 6qA3.3 | rs33832338 | 0 | 1 |
| chr6 | 28707169 | A | C | 221 | Snd1 | intronic | . | . | . | 6qA3.3 | rs33833210 | 0 | 1 |
| chr6 | 28707268 | T | C | 169 | Snd1 | intronic | . | . | . | 6qA3.3 | rs33833212 | 0 | 1 |
| chr6 | 28745006 | C | A | 99 | Snd1 | intronic | . | . | . | 6qA3.3 | . | 0 | 1 |
| chr6 | 28745060 | A | G | 126 | Snd1 | intronic | . | . | . | 6qA3.3 | rs33840623 | 0 | 1 |
| chr6 | 28745128 | G | A | 222 | Snd1 | intronic | . | . | . | 6qA3.3 | rs33841477 | 0 | 1 |
| chr6 | 28745476 | T | C | 125 | Snd1 | intronic | . | . | . | 6qA3.3 | rs33834344 | 0 | 1 |
| chr6 | 28795696 | G | T | 132 | Snd1 | intronic | . | . | . | 6qA3.3 | rs30902483 | 0 | 1 |
| chr6 | 28883384 | A | G | 221 | Snd1 | splicing | . | . | . | 6qA3.3 | rs33841581 | 0 | 1 |
| chr6 | 29069300 | A | T | 115 | Lep | intronic | . | . | . | 6qA3.3 | rs13478682 | 0 | 1 |
| chr6 | 29070749 | G | T | 220 | Lep | intronic | . | . | . | 6qA3.3 | rs29605219 | 0 | 1 |
| chr6 | 29177041 | A | G | 222 | Prrt4 | intronic | . | . | . | 6qA3.3 | rs30024731 | 0 | 1 |
| chr6 | 29177869 | G | A | 79 | Prrt4 | intronic | . | . | . | 6qA3.3 | . | 0 | 1 |
| chr6 | 29202281 | G | A | 110 | Impdh1 | intronic | . | . | . | 6qA3.3 | . | 0 | 1 |
| chr6 | 29202809 | A | G | 221 | Impdh1 | exonic | synonymous SNV | Impdh1:NM_011829:exon12:c.T1296C:p.S432S,Impdh1:NM_001302933:exon13:c.T1470C:p.S490S,Impdh1:N | . | 6qA3.3 | rs211934420 | 0 | 1 |
| chr6 | 29203209 | C | A | 222 | Impdh1 | exonic | synonymous SNV | Impdh1:NM_011829:exon11:c.G1188T:p.A396A,Impdh1:NM_001302933:exon12:c.G1362T:p.A454A,Impdh1:N | . | 6qA3.3 | . | 0 | 1 |
| chr6 | 29203346 | C | T | 221 | Impdh1 | splicing | . | . | . | 6qA3.3 | . | 0 | 1 |
| chr6 | 29203446 | G | A | 222 | Impdh1 | exonic | synonymous SNV | Impdh1:NM_011829:exon10:c.C1053T:p.A351A,Impdh1:NM_001302933:exon11:c.C1227T:p.A409A,Impdh1:N | . | 6qA3.3 | rs224877249 | 0 | 1 |
| chr6 | 29204275 | C | T | 221 | Impdh1 | splicing | . | . | . | 6qA3.3 | . | 0 | 1 |
| chr6 | 29204585 | T | C | 222 | Impdh1 | exonic | synonymous SNV | Impdh1:NM_011829:exon8:c.A894G:p.Q298Q,Impdh1:NM_001302933:exon9:c.A1068G:p.Q356Q,Impdh1:NM_( | . | 6qA3.3 | rs244326407 | 0 | 1 |
| chr6 | 29204665 | A | G | 222 | Impdh1 | intronic | . | . | . | 6qA3.3 | . | 0 | 1 |
| chr6 | 29205016 | G | A | 222 | Impdh1 | intronic | . | . | . | 6qA3.3 | . | 0 | 1 |
| chr6 | 29205941 | A | G | 114 | Impdh1 | intronic | . | . | . | 6qA3.3 | rs6390569 | 0 | 1 |
| chr6 | 29209502 | C | A | 56 | Impdh1 | intronic | . | . | . | 6qA3.3 | . | 0 | 1 |
| chr6 | 29282963 | A | C | 114 | Fam71f2 | intronic | . | . | . | 6qA3.3 | rs231179280 | 0 | 1 |
| chr6 | 29282983 | T | C | 127 | Fam71f2 | intronic | . | . | . | 6qA3.3 | rs219674935 | 0 | 1 |
| chr6 | 29290375 | T | A | 221 | Fam71f2 | exonic | missense SNV | Fam71f2:NM_001101486:exon5:c.T751A:p.S251T | . | 6qA3.3 | rs249288555 | 0 | 1 |
| chr6 | 29290419 | T | C | 222 | Fam71f2 | exonic | synonymous SNV | Fam71f2:NM_001101486:exon5:c.T795C:p.A265A | . | 6qA3.3 | rs29605116 | 0 | 1 |
| chr6 | 29290533 | C | G | 222 | Fam71f2 | UTR3 | . | . | . | 6qA3.3 | rs235628567 | 0 | 1 |
| chr6 | 29290544 | C | T | 222 | Fam71f2 | UTR3 | . | . | . | 6qA3.3 | . | 0 | 1 |
| chr6 | 29318972 | T | C | 79 | . | upstream | . | . | . | 6qA3.3 | rs29609022 | 0 | 1 |
| chr6 | 29319255 | A | C | 221 | Fam71f1 | exonic | missense SNV | Fam71f1:NM_001289663:exon1:c.A57C:p.K19N,Fam71f1:NM_001289664:exon1:c.A57C:p.K19N,Fam71f1:NM_ | . | 6qA3.3 | rs29609726 | 0 | 1 |
| chr6 | 29319458 | A | G | 222 | Fam71f1 | intronic | . | . | . | 6qA3.3 | rs29609728 | 0 | 1 |
| chr6 | 29319561 | G | A | 222 | Fam71f1 | intronic | . | . | . | 6qA3.3 | rs29609732 | 0 | 1 |
| chr6 | 29320729 | C | T | 221 | Fam71f1 | exonic | synonymous SNV | Fam71f1:NM_001289663:exon2:c.C351T:p.L117L,Fam71f1:NM_001289664:exon2:c.C351T:p.L117L,Fam71f1 | . | 6qA3.3 | . | 0 | 1 |
| chr6 | 29323631 | T | C | 220 | Fam71f1 | intronic | . | . | . | 6qA3.3 | rs264633308 | 0 | 1 |
| chr6 | 29323671 | C | G | 222 | Fam71f1 | intronic | . | . | . | 6qA3.3 | rs6180734 | 0 | 1 |
| chr6 | 29323736 | G | A | 222 | Fam71f1 | exonic | synonymous SNV | Fam71f1:NM_001289663:exon3:c.G459A:p.E153E,Fam71f1:NM_001289664:exon3:c.G459A:p.E153E,Fam71f1 | . | 6qA3.3 | . | 0 | 1 |
| chr6 | 29356453 | T | C | 222 | Calu | intronic | . | . | . | 6qA3.3 | rs29608773 | 0 | 1 |
| chr6 | 29356528 | T | C | 222 | Calu | exonic | synonymous SNV | Calu:NM_007594:exon2:c.T52C:p.L18L,Calu:NM_184053:exon2:c.T52C:p.L18L | . | 6qA3.3 | . | 0 | 1 |
| chr6 | 29361262 | T | C | 221 | Calu | intronic | . | . | . | 6qA3.3 | rs259896799 | 0 | 1 |
| chr6 | 29365824 | C | T | 65 | Calu | intronic | . | . | . | 6qA3.3 | . | 0 | 1 |
| chr6 | 29366737 | G | A | 128 | Calu | intronic | . | . | . | 6qA3.3 | . | 0 | 1 |
| chr6 | 29366879 | A | G | 221 | Calu | intronic | . | . | . | 6qA3.3 | rs29609573 | 0 | 1 |
| chr6 | 29374286 | C | T | 214 | Calu | intronic | . | . | . | 6qA3.3 | . | 0 | 1 |
| chr6 | 29378056 | C | G | 222 | Opn1sw | exonic | synonymous SNV | Opn1sw:NM_007538:exon5:c.G927C:p.R309R | . | 6qA3.3 | rs29607543 | 0 | 1 |
| chr6 | 29378090 | C | T | 222 | Opn1sw | intronic | . | . | . | 6qA3.3 | rs29608755 | 0 | 1 |
| chr6 | 29379058 | G | A | 221 | Opn1sw | intronic | . | . | . | 6qA3.3 | . | 0 | 1 |
| chr6 | 29379110 | C | T | 157 | Opn1sw | intronic | . | . | . | 6qA3.3 | . | 0 | 1 |
| chr6 | 29379259 | T | C | 171 | Opn1sw | intronic | . | . | . | 6qA3.3 | rs238790534 | 0 | 1 |
| chr6 | 29379570 | C | G | 221 | Opn1sw | intronic | . | . | . | 6qA3.3 | . | 0 | 1 |
| chr6 | 29379733 | A | G | 222 | Opn1sw | exonic | synonymous SNV | Opn1sw:NM_007538:exon2:c.T501C:p.F167F | . | 6qA3.3 | rs214897940 | 0 | 1 |
| chr6 | 29380362 | G | T | 221 | Opn1sw | exonic | synonymous SNV | Opn1sw:NM_007538:exon1:c.C54A:p.P18P | . | 6qA3.3 | . | 0 | 1 |
| chr6 | 29399435 | C | T | 200 | Ccdc136 | exonic | missense SNV | Ccdc136:NM_001201378:exon2:c.C125T:p.S42F,Ccdc136:NM_145574:exon2:c.C125T:p.S42F | . | 6qA3.3 | . | 0 | 1 |
| chr6 | 29409475 | T | C | 127 | Ccdc136 | intronic | . | . | . | 6qA3.3 | rs29608536 | 0 | 1 |
| chr6 | 29410284 | A | G | 221 | Ccdc136 | intronic | . | . | . | 6qA3.3 | . | 0 | 1 |
| chr6 | 29410335 | T | A | 221 | Ccdc136 | intronic | . | . | . | 6qA3.3 | . | 0 | 1 |
| chr6 | 29412338 | C | T | 221 | Ccdc136 | intronic | . | . | . | 6qA3.3 | rs29609499 | 0 | 1 |
| chr6 | 29413213 | T | C | 131 | Ccdc136 | intronic | . | . | . | 6qA3.3 | . | 0 | 1 |
| chr6 | 29414752 | T | C | 222 | Ccdc136 | intronic | . | . | . | 6qA3.3 | rs214138794 | 0 | 1 |
| chr6 | 29417024 | A | C | 193 | Ccdc136 | intronic | . | . | . | 6qA3.3 | . | 0 | 1 |
| chr6 | 29419419 | C | T | 221 | Ccdc136 | intronic | . | . | . | 6qA3.3 | rs29612709 | 0 | 1 |
| chr6 | 29419477 | C | T | 221 | Ccdc136 | intronic | . | . | . | 6qA3.3 | . | 0 | 1 |
| chr6 | 29419559 | A | G | 89 | Ccdc136 | intronic | . | . | . | 6qA3.3 | rs29612711 | 0 | 1 |
| chr6 | 29433856 | A | G | 221 | Flnc | intronic | . | . | . | 6qA3.3 | rs219020856 | 0 | 1 |
| chr6 | 29438642 | C | T | 221 | Flnc | exonic | synonymous SNV | Flnc:NM_001081185:exon2:c.C534T:p.P178P,Flnc:NM_001311074:exon2:c.C534T:p.P178P | . | 6qA3.3 | rs29610584 | 0 | 1 |
| chr6 | 29440108 | G | A | 178 | Flnc | intronic | . | . | . | 6qA3.3 | . | 0 | 1 |
| chr6 | 29440116 | A | G | 182 | Flnc | intronic | . | . | . | 6qA3.3 | . | 0 | 1 |
| chr6 | 29440405 | T | C | 222 | Flnc | exonic | synonymous SNV | Flnc:NM_001081185:exon4:c.T711C:p.A237A,Flnc:NM_001311074:exon4:c.T711C:p.A237A | . | 6qA3.3 | rs247424295 | 0 | 1 |
| chr6 | 29440495 | C | T | 222 | Flnc | exonic | synonymous SNV | Flnc:NM_001081185:exon4:c.C801T:p.A267A,Flnc:NM_001311074:exon4:c.C801T:p.A267A | . | 6qA3.3 | . | 0 | 1 |
| chr6 | 29440534 | T | C | 222 | Flnc | exonic | synonymous SNV | Flnc:NM_001081185:exon4:c.T840C:p.I280I,Flnc:NM_001311074:exon4:c.T840C:p.I280I | . | 6qA3.3 | rs265720113 | 0 | 1 |
| chr6 | 29440546 | T | C | 205 | Flnc | exonic | synonymous SNV | Flnc:NM_001081185:exon4:c.T852C:p.P284P,Flnc:NM_001311074:exon4:c.T852C:p.P284P | . | 6qA3.3 | rs246776082 | 0 | 1 |
| chr6 | 29440610 | A | G | 106 | Flnc | intronic | . | . | . | 6qA3.3 | rs29612094 | 0 | 1 |
| chr6 | 29440624 | G | A | 160 | Flnc | intronic | . | . | . | 6qA3.3 | . | 0 | 1 |
| chr6 | 29440708 | A | G | 222 | Flnc | exonic | synonymous SNV | Flnc:NM_001081185:exon5:c.A924G:p.V308V,Flnc:NM_001311074:exon5:c.A924G:p.V308V | . | 6qA3.3 | rs212151191 | 0 | 1 |
| chr6 | 29440750 | G | T | 222 | Flnc | exonic | synonymous SNV | Flnc:NM_001081185:exon5:c.G966T:p.T322T,Flnc:NM_001311074:exon5:c.G966T:p.T322T | . | 6qA3.3 | rs253976261 | 0 | 1 |
| chr6 | 29441016 | G | A | 222 | Flnc | intronic | . | . | . | 6qA3.3 | . | 0 | 1 |
| chr6 | 29441035 | T | C | 195 | Flnc | intronic | . | . | . | 6qA3.3 | rs230403876 | 0 | 1 |
| chr6 | 29441064 | G | A | 180 | Flnc | intronic | . | . | . | 6qA3.3 | rs249335571 | 0 | 1 |
| chr6 | 29441128 | C | T | 222 | Flnc | exonic | synonymous SNV | Flnc:NM_001081185:exon7:c.C1080T:p.I360I,Flnc:NM_001311074:exon7:c.C1080T:p.I360I | . | 6qA3.3 | . | 0 | 1 |

| | | | | | | | | | | | | | |
|---|---|---|---|---|---|---|---|---|---|---|---|---|---|
| chr6 | 29443051 | T | G | 222 | Flnc | intronic | . | . | . | 6qA3.3 | rs29614034 | 0 | 1 |
| chr6 | 29443535 | T | C | 221 | Flnc | intronic | . | . | . | 6qA3.3 | rs218673507 | 0 | 1 |
| chr6 | 29444159 | C | T | 222 | Flnc | exonic | synonymous SNV | Flnc:NM_001081185:exon12:c.C1971T:p.A657A,Flnc:NM_001311074:exon12:c.C1971T:p.A657A | . | 6qA3.3 | . | 0 | 1 |
| chr6 | 29445167 | T | C | 222 | Flnc | intronic | . | . | . | 6qA3.3 | rs29607994 | 0 | 1 |
| chr6 | 29445363 | A | C | 221 | Flnc | intronic | . | . | . | 6qA3.3 | rs246780745 | 0 | 1 |
| chr6 | 29445916 | G | A | 222 | Flnc | intronic | . | . | . | 6qA3.3 | . | 0 | 1 |
| chr6 | 29446098 | C | T | 221 | Flnc | exonic | synonymous SNV | Flnc:NM_001081185:exon17:c.C2601T:p.D867D,Flnc:NM_001311074:exon17:c.C2601T:p.D867D | . | 6qA3.3 | rs29607996 | 0 | 1 |
| chr6 | 29446369 | A | G | 221 | Flnc | exonic | synonymous SNV | Flnc:NM_001081185:exon18:c.A2784G:p.S928S,Flnc:NM_001311074:exon18:c.A2784G:p.S928S | . | 6qA3.3 | rs29608002 | 0 | 1 |
| chr6 | 29446432 | C | A | 222 | Flnc | intronic | . | . | . | 6qA3.3 | . | 0 | 1 |
| chr6 | 29446531 | A | G | 221 | Flnc | intronic | . | . | . | 6qA3.3 | rs29608936 | 0 | 1 |
| chr6 | 29446730 | A | T | 221 | Flnc | intronic | . | . | . | 6qA3.3 | rs29608938 | 0 | 1 |
| chr6 | 29447046 | T | C | 221 | Flnc | exonic | synonymous SNV | Flnc:NM_001081185:exon20:c.T3171C:p.G1057G,Flnc:NM_001311074:exon20:c.T3171C:p.G1057G | . | 6qA3.3 | rs249581700 | 0 | 1 |
| chr6 | 29447152 | A | C | 222 | Flnc | intronic | . | . | . | 6qA3.3 | . | 0 | 1 |
| chr6 | 29447244 | A | G | 221 | Flnc | intronic | . | . | . | 6qA3.3 | . | 0 | 1 |
| chr6 | 29447296 | G | A | 221 | Flnc | intronic | . | . | . | 6qA3.3 | rs254470207 | 0 | 1 |
| chr6 | 29451946 | C | T | 222 | Flnc | intronic | . | . | . | 6qA3.3 | . | 0 | 1 |
| chr6 | 29452278 | T | C | 221 | Flnc | exonic | synonymous SNV | Flnc:NM_001081185:exon30:c.T5100C:p.D1700D,Flnc:NM_001311074:exon30:c.T5100C:p.D1700D | . | 6qA3.3 | rs260640055 | 0 | 1 |
| chr6 | 29452308 | C | T | 221 | Flnc | exonic | synonymous SNV | Flnc:NM_001081185:exon30:c.C5130T:p.P1710P,Flnc:NM_001311074:exon30:c.C5130T:p.P1710P | . | 6qA3.3 | . | 0 | 1 |
| chr6 | 29453799 | A | G | 222 | Flnc | intronic | . | . | . | 6qA3.3 | rs29612254 | 0 | 1 |
| chr6 | 29454456 | C | T | 222 | Flnc | intronic | . | . | . | 6qA3.3 | rs218900476 | 0 | 1 |
| chr6 | 29454514 | C | T | 157 | Flnc | intronic | . | . | . | 6qA3.3 | . | 0 | 1 |
| chr6 | 29454930 | T | C | 74 | Flnc | intronic | . | . | . | 6qA3.3 | rs249709034 | 0 | 1 |
| chr6 | 29454931 | A | G | 74 | Flnc | intronic | . | . | . | 6qA3.3 | rs212864439 | 0 | 1 |
| chr6 | 29455088 | G | A | 219 | Flnc | intronic | . | . | . | 6qA3.3 | . | 0 | 1 |
| chr6 | 29455099 | C | T | 222 | Flnc | intronic | . | . | . | 6qA3.3 | . | 0 | 1 |
| chr6 | 29455325 | G | C | 222 | Flnc | intronic | . | . | . | 6qA3.3 | rs221050185 | 0 | 1 |
| chr6 | 29455386 | C | T | 222 | Flnc | exonic | synonymous SNV | Flnc:NM_001311074:exon36:c.C5934T:p.V1978V,Flnc:NM_001081185:exon37:c.C6033T:p.V2011V | . | 6qA3.3 | . | 0 | 1 |
| chr6 | 29455464 | G | A | 222 | Flnc | exonic | synonymous SNV | Flnc:NM_001311074:exon36:c.G6012A:p.S2004S,Flnc:NM_001081185:exon37:c.G6111A:p.S2037S | . | 6qA3.3 | . | 0 | 1 |
| chr6 | 29455560 | T | C | 222 | Flnc | exonic | synonymous SNV | Flnc:NM_001311074:exon36:c.T6108C:p.N2036N,Flnc:NM_001081185:exon37:c.T6207C:p.N2069N | . | 6qA3.3 | rs29612259 | 0 | 1 |
| chr6 | 29455975 | C | T | 222 | Flnc | intronic | . | . | . | 6qA3.3 | rs253805532 | 0 | 1 |
| chr6 | 29456192 | G | A | 222 | Flnc | intronic | . | . | . | 6qA3.3 | . | 0 | 1 |
| chr6 | 29456590 | G | A | 222 | Flnc | intronic | . | . | . | 6qA3.3 | . | 0 | 1 |
| chr6 | 29459598 | G | T | 222 | Flnc | intronic | . | . | . | 6qA3.3 | . | 0 | 1 |
| chr6 | 29459608 | G | A | 222 | Flnc | intronic | . | . | . | 6qA3.3 | . | 0 | 1 |
| chr6 | 29460676 | T | C | 143 | Flnc | intronic | . | . | . | 6qA3.3 | rs29609880 | 0 | 1 |
| chr6 | 29468059 | C | G | 222 | Atp6v1f | intronic | . | . | . | 6qA3.3 | rs240710518 | 0 | 1 |
| chr6 | 29487032 | T | C | 139 | Kcp | intronic | . | . | . | 6qA3.3 | rs259936804 | 0 | 1 |
| chr6 | 29489198 | G | A | 183 | Kcp | intronic | . | . | . | 6qA3.3 | rs258032400 | 0 | 1 |
| chr6 | 29498972 | A | G | 221 | Kcp | exonic | synonymous SNV | Kcp:NM_001029985:exon10:c.T1002C:p.S334S | . | 6qA3.3 | rs218770819 | 0 | 1 |
| chr6 | 29501121 | C | G | 190 | Kcp | intronic | . | . | . | 6qA3.3 | . | 0 | 1 |
| chr6 | 29536548 | T | C | 222 | Irf5 | exonic | synonymous SNV | Irf5:NM_001252382:exon9:c.T1284C:p.P428P,Irf5:NM_001311083:exon9:c.T1257C:p.P419P,Irf5:NM_01 | . | 6qA3.3 | rs29612678 | 0 | 1 |
| chr6 | 29543026 | C | T | 79 | Tnpo3 | intronic | . | . | . | 6qA3.3 | . | 0 | 1 |
| chr6 | 29559057 | T | C | 111 | Tnpo3 | intronic | . | . | . | 6qA3.3 | rs29614822 | 0 | 1 |
| chr6 | 29568626 | A | C | 89 | Tnpo3 | intronic | . | . | . | 6qA3.3 | . | 0 | 1 |
| chr6 | 29575246 | A | G | 221 | Tnpo3 | intronic | . | . | . | 6qA3.3 | . | 0 | 1 |
| chr6 | 29575281 | C | A | 92 | Tnpo3 | intronic | . | . | . | 6qA3.3 | . | 0 | 1 |
| chr6 | 29575322 | T | A | 36.9876 | Tnpo3 | intronic | . | . | . | 6qA3.3 | rs265183480 | 0 | 1 |
| chr6 | 29582370 | G | A | 221 | Tnpo3 | intronic | . | . | . | 6qA3.3 | . | 0 | 1 |
| chr6 | 29694402 | T | C | 60 | Tspan33 | UTR5 | . | . | 385 | 6qA3.3 | rs248376594 | 0 | 1 |
| chr6 | 29694407 | G | A | 60 | Tspan33 | UTR5 | . | . | 385 | 6qA3.3 | rs222501131 | 0 | 1 |
| chr6 | 29694481 | C | G | 128 | Tspan33 | exonic | missense SNV | Tspan33:NM_001301407:exon1:c.C39G:p.D13E,Tspan33:NM_146173:exon1:c.C39G:p.D13E | 385 | 6qA3.3 | rs259126941 | 0 | 1 |
| chr6 | 29716862 | A | C | 221 | Tspan33 | intronic | . | . | . | 6qA3.3 | rs29611940 | 0 | 1 |
| chr6 | 29717028 | A | G | 222 | Tspan33 | intronic | . | . | . | 6qA3.3 | rs233910192 | 0 | 1 |
| chr6 | 29717168 | G | A | 221 | Tspan33 | intronic | . | . | . | 6qA3.3 | . | 0 | 1 |
| chr6 | 29717421 | G | A | 221 | Tspan33 | intronic | . | . | . | 6qA3.3 | rs51190524 | 0 | 1 |
| chr6 | 29717554 | A | G | 221 | Tspan33 | exonic | synonymous SNV | Tspan33:NM_001301407:exon8:c.A765G:p.L255L,Tspan33:NM_146173:exon8:c.A768G:p.L256L | . | 6qA3.3 | rs29613024 | 0 | 1 |
| chr6 | 29736170 | G | A | 145 | Smo | exonic | missense SNV | Smo:NM_176996:exon1:c.G160A:p.D54N | 1125 | 6qA3.3 | rs30765548 | 0 | 1 |
| chr6 | 29756717 | T | C | 176 | Smo | intronic | . | . | . | 6qA3.3 | . | 0 | 1 |
| chr6 | 30089998 | G | A | 221 | Nrf1 | exonic | synonymous SNV | Nrf1:NM_001164226:exon3:c.G75A:p.Q25Q,Nrf1:NM_001164227:exon3:c.G75A:p.Q25Q,Nrf1:NM_00116422 | . | 6qA3.3 | rs13478688 | 0 | 1 |
| chr6 | 30090157 | T | C | 221 | Nrf1 | intronic | . | . | . | 6qA3.3 | rs29654090 | 0 | 1 |
| chr6 | 30098350 | G | C | 216 | Nrf1 | intronic | . | . | . | 6qA3.3 | rs29653760 | 0 | 1 |
| chr6 | 30102431 | G | A | 221 | Nrf1 | intronic | . | . | . | 6qA3.3 | rs29657334 | 0 | 1 |
| chr6 | 30102504 | C | A | 122 | Nrf1 | intronic | . | . | . | 6qA3.3 | rs29657338 | 0 | 1 |
| chr6 | 30117189 | G | A | 221 | Nrf1 | intronic | . | . | . | 6qA3.3 | rs29653974 | 0 | 1 |
| chr6 | 30118773 | G | C | 125 | Nrf1 | intronic | . | . | . | 6qA3.3 | rs29654866 | 0 | 1 |
| chr6 | 30118960 | C | T | 222 | Nrf1 | exonic | synonymous SNV | Nrf1:NM_001164226:exon10:c.C1077T:p.N359N,Nrf1:NM_001164227:exon10:c.C1077T:p.N359N,Nrf1:NM_( | . | 6qA3.3 | rs29654868 | 0 | 1 |
| chr6 | 30119074 | G | A | 222 | Nrf1 | exonic | synonymous SNV | Nrf1:NM_001164226:exon10:c.G1191A:p.Q397Q,Nrf1:NM_001164227:exon10:c.G1191A:p.Q397Q,Nrf1:NM_( | . | 6qA3.3 | rs29654872 | 0 | 1 |
| chr6 | 30119169 | G | T | 222 | Nrf1 | intronic | . | . | . | 6qA3.3 | rs29655816 | 0 | 1 |
| chr6 | 30119214 | C | T | 108 | Nrf1 | intronic | . | . | . | 6qA3.3 | rs29655818 | 0 | 1 |
| chr6 | 30140074 | G | A | 143 | Nrf1 | intronic | . | . | . | 6qA3.3 | rs29654742 | 0 | 1 |
| chr6 | 30140300 | G | A | 222 | Nrf1 | intronic | . | . | . | 6qA3.3 | rs29655668 | 0 | 1 |
| chr6 | 30140370 | T | C | 222 | Nrf1 | intronic | . | . | . | 6qA3.3 | rs29655670 | 0 | 1 |
| chr6 | 30144813 | A | G | 221 | Nrf1 | exonic | synonymous SNV | Nrf1:NM_001164227:exon12:c.A1581G:p.S527S | . | 6qA3.3 | rs29653694 | 0 | 1 |
| chr6 | 30144825 | G | A | 221 | Nrf1 | exonic | synonymous SNV | Nrf1:NM_001164227:exon12:c.G1593A:p.L531L | . | 6qA3.3 | rs29653696 | 0 | 1 |
| chr6 | 30151387 | G | A | 148 | Nrf1 | intronic | . | . | . | 6qA3.3 | rs29651940 | 0 | 1 |
| chr6 | 30260751 | A | T | 104 | Ube2h | intronic | . | . | . | 6qA3.3 | rs30105365 | 0 | 1 |
| chr6 | 30370486 | A | T | 221 | Zc3hc1 | intronic | . | . | . | 6qA3.3 | . | 0 | 1 |
| chr6 | 30374851 | A | G | 221 | Zc3hc1 | exonic | synonymous SNV | Zc3hc1:NM_001311086:exon6:c.T690C:p.D230D,Zc3hc1:NM_172735:exon6:c.T690C:p.D230D | . | 6qA3.3 | rs36506464 | 0 | 1 |
| chr6 | 30482713 | C | T | 222 | Tmem209 | UTR3 | . | . | . | 6qA3.3 | . | 0 | 1 |
| chr6 | 30487613 | G | T | 80 | Tmem209 | intronic | . | . | . | 6qA3.3 | . | 0 | 1 |
| chr6 | 30551813 | C | T | 127 | Cpa2 | intronic | . | . | . | 6qA3.3 | . | 0 | 1 |
| chr6 | 30641252 | T | C | 146 | Cpa1 | intronic | . | . | . | 6qA3.3 | rs37078175 | 0 | 1 |
| chr6 | 30679441 | C | A | 221 | Cep41 | intronic | . | . | . | 6qA3.3 | rs252022031 | 0 | 1 |
| chr6 | 30812772 | C | A | 221 | Copg2 | exonic | missense SNV | Copg2:NM_017478:exon16:c.G1620T:p.M540I | . | 6qA3.3 | . | 0 | 1 |
| chr6 | 30825600 | T | C | 113 | Copg2 | intronic | . | . | . | 6qA3.3 | . | 0 | 1 |
| chr6 | 30900159 | T | G | 160 | Tsga13 | intronic | . | . | . | 6qA3.3 | . | 0 | 1 |
| chr6 | 31426693 | T | C | 228 | Mkln1 | intronic | . | . | . | 6qA3.3 | rs30718075 | 1 | 0 |
| chr6 | 32192054 | T | C | 119 | Plxna4 | intronic | . | . | . | 6qA3.3 | . | 0 | 1 |
| chr6 | 32192195 | G | A | 221 | Plxna4 | intronic | . | . | . | 6qA3.3 | . | 0 | 1 |
| chr6 | 32192337 | T | C | 221 | Plxna4 | exonic | synonymous SNV | Plxna4:NM_175750:exon21:c.A3924G:p.G1308G | . | 6qA3.3 | rs242356762 | 0 | 1 |
| chr6 | 32192412 | T | G | 170 | Plxna4 | intronic | . | . | . | 6qA3.3 | rs39325488 | 0 | 1 |
| chr6 | 32196140 | C | G | 90 | Plxna4 | exonic | synonymous SNV | Plxna4:NM_175750:exon20:c.G3855C:p.A1285A | . | 6qA3.3 | rs37196952 | 0 | 1 |
| chr6 | 32196269 | C | T | 74 | Plxna4 | exonic | synonymous SNV | Plxna4:NM_175750:exon20:c.G3726A:p.A1242A | . | 6qA3.3 | . | 0 | 1 |
| chr6 | 32196287 | G | A | 146 | Plxna4 | exonic | synonymous SNV | Plxna4:NM_175750:exon20:c.C3708T:p.P1236P | . | 6qA3.3 | . | 0 | 1 |
| chr6 | 32196395 | A | T | 129 | Plxna4 | intronic | . | . | . | 6qA3.3 | . | 0 | 1 |
| chr6 | 32196413 | G | A | 101 | Plxna4 | intronic | . | . | . | 6qA3.3 | rs251765961 | 0 | 1 |
| chr6 | 32197004 | A | G | 93 | Plxna4 | intronic | . | . | . | 6qA3.3 | . | 0 | 1 |
| chr6 | 32197017 | T | C | 101 | Plxna4 | intronic | . | . | . | 6qA3.3 | . | 0 | 1 |
| chr6 | 32197880 | T | G | 222 | Plxna4 | exonic | synonymous SNV | Plxna4:NM_175750:exon18:c.A3273C:p.T1091T | . | 6qA3.3 | rs38427998 | 0 | 1 |
| chr6 | 32197908 | A | G | 222 | Plxna4 | intronic | . | . | . | 6qA3.3 | rs36345406 | 0 | 1 |
| chr6 | 32198002 | T | A | 222 | Plxna4 | intronic | . | . | . | 6qA3.3 | rs37648493 | 0 | 1 |
| chr6 | 32198052 | A | G | 176 | Plxna4 | intronic | . | . | . | 6qA3.3 | rs37916606 | 0 | 1 |
| chr6 | 32198086 | A | C | 146 | Plxna4 | intronic | . | . | . | 6qA3.3 | rs39144061 | 0 | 1 |
| chr6 | 32198122 | A | G | 97 | Plxna4 | intronic | . | . | . | 6qA3.3 | rs255500137 | 0 | 1 |
| chr6 | 32198213 | C | T | 222 | Plxna4 | intronic | . | . | . | 6qA3.3 | rs220143490 | 0 | 1 |
| chr6 | 32198421 | T | A | 222 | Plxna4 | intronic | . | . | . | 6qA3.3 | . | 0 | 1 |
| chr6 | 32198423 | G | T | 222 | Plxna4 | intronic | . | . | . | 6qA3.3 | rs265467146 | 0 | 1 |
| chr6 | 32198432 | T | C | 192 | Plxna4 | intronic | . | . | . | 6qA3.3 | . | 0 | 1 |
| chr6 | 32198495 | A | G | 33.2381 | Plxna4 | intronic | . | . | . | 6qA3.3 | rs259202605 | 0 | 1 |
| chr6 | 32200557 | A | G | 37.7458 | Plxna4 | intronic | . | . | . | 6qA3.3 | rs247249414 | 0 | 1 |
| chr6 | 32201084 | C | T | 199 | Plxna4 | intronic | . | . | . | 6qA3.3 | . | 0 | 1 |
| chr6 | 32202230 | G | A | 221 | Plxna4 | intronic | . | . | . | 6qA3.3 | . | 0 | 1 |
| chr6 | 32205887 | C | T | 70 | Plxna4 | intronic | . | . | . | 6qA3.3 | . | 0 | 1 |
| chr6 | 32205900 | G | A | 93 | Plxna4 | intronic | . | . | . | 6qA3.3 | . | 0 | 1 |
| chr6 | 32206045 | G | A | 222 | Plxna4 | intronic | . | . | . | 6qA3.3 | rs222630764 | 0 | 1 |
| chr6 | 32206075 | G | A | 221 | Plxna4 | intronic | . | . | . | 6qA3.3 | rs253877049 | 0 | 1 |
| chr6 | 32206189 | T | C | 221 | Plxna4 | exonic | synonymous SNV | Plxna4:NM_175750:exon14:c.A2778G:p.A926A | . | 6qA3.3 | rs36310687 | 0 | 1 |
| chr6 | 32206192 | A | G | 222 | Plxna4 | exonic | synonymous SNV | Plxna4:NM_175750:exon14:c.T2775C:p.H925H | . | 6qA3.3 | rs240749714 | 0 | 1 |
| chr6 | 32206309 | A | G | 177 | Plxna4 | intronic | . | . | . | 6qA3.3 | . | 0 | 1 |
| chr6 | 32206321 | T | G | 116 | Plxna4 | intronic | . | . | . | 6qA3.3 | rs39680107 | 0 | 1 |
| chr6 | 32206322 | C | T | 123 | Plxna4 | intronic | . | . | . | 6qA3.3 | . | 0 | 1 |
| chr6 | 32210985 | A | G | 45.9048 | Plxna4 | exonic | synonymous SNV | Plxna4:NM_175750:exon13:c.T2715C:p.D905D | . | 6qA3.3 | rs37999290 | 0 | 1 |
| chr6 | 32214987 | C | G | 89 | Plxna4 | intronic | . | . | . | 6qA3.3 | . | 0 | 1 |
| chr6 | 32215048 | T | C | 222 | Plxna4 | intronic | . | . | . | 6qA3.3 | rs258030007 | 0 | 1 |
| chr6 | 32215128 | G | A | 221 | Plxna4 | exonic | synonymous SNV | Plxna4:NM_175750:exon12:c.C2526T:p.S842S | . | 6qA3.3 | . | 0 | 1 |
| chr6 | 32215206 | G | A | 222 | Plxna4 | exonic | synonymous SNV | Plxna4:NM_175750:exon12:c.C2448T:p.A816A | . | 6qA3.3 | rs248176163 | 0 | 1 |
| chr6 | 32215535 | A | T | 161 | Plxna4 | intronic | . | . | . | 6qA3.3 | rs223855490 | 0 | 1 |
| chr6 | 32215549 | T | C | 171 | Plxna4 | intronic | . | . | . | 6qA3.3 | rs263915822 | 0 | 1 |
| chr6 | 32215815 | G | A | 173 | Plxna4 | intronic | . | . | . | 6qA3.3 | . | 0 | 1 |
| chr6 | 32215834 | G | A | 140 | Plxna4 | intronic | . | . | . | 6qA3.3 | rs257108129 | 0 | 1 |
| chr6 | 32223779 | T | C | 74 | Plxna4 | intronic | . | . | . | 6qA3.3 | rs247493154 | 0 | 1 |
| chr6 | 32223788 | G | A | 91 | Plxna4 | intronic | . | . | . | 6qA3.3 | rs259958505 | 0 | 1 |
| chr6 | 32223933 | A | G | 222 | Plxna4 | intronic | . | . | . | 6qA3.3 | rs38102592 | 0 | 1 |
| chr6 | 32224172 | G | A | 222 | Plxna4 | intronic | . | . | . | 6qA3.3 | rs254497947 | 0 | 1 |
| chr6 | 32224235 | C | T | 131 | Plxna4 | intronic | . | . | . | 6qA3.3 | rs36995433 | 0 | 1 |
| chr6 | 32234599 | C | T | 221 | Plxna4 | exonic | synonymous SNV | Plxna4:NM_175750:exon9:c.G2004A:p.P668P | . | 6qA3.3 | rs36284906 | 0 | 1 |
| chr6 | 32236157 | A | T | 133 | Plxna4 | intronic | . | . | . | 6qA3.3 | . | 0 | 1 |
| chr6 | 32236208 | G | A | 210 | Plxna4 | intronic | . | . | . | 6qA3.3 | rs253255915 | 0 | 1 |
| chr6 | 32236254 | T | G | 222 | Plxna4 | intronic | . | . | . | 6qA3.3 | rs37310367 | 0 | 1 |
| chr6 | 32237484 | C | A | 41.9425 | Plxna4 | intronic | . | . | . | 6qA3.3 | . | 0 | 1 |
| chr6 | 32237516 | C | T | 96 | Plxna4 | intronic | . | . | . | 6qA3.3 | rs251653500 | 0 | 1 |
| chr6 | 32237561 | C | T | 127 | Plxna4 | intronic | . | . | . | 6qA3.3 | . | 0 | 1 |
| chr6 | 32237784 | T | A | 219 | Plxna4 | exonic | synonymous SNV | Plxna4:NM_175750:exon7:c.A1761T:p.S587S | . | 6qA3.3 | . | 0 | 1 |
| chr6 | 32239714 | T | C | 123 | Plxna4 | intronic | . | . | . | 6qA3.3 | rs241322176 | 0 | 1 |
| chr6 | 32516463 | C | T | 222 | Plxna4 | intronic | . | . | . | 6qA3.3 | rs37585129 | 0 | 1 |
| chr6 | 32516813 | A | G | 221 | Plxna4 | exonic | synonymous SNV | Plxna4:NM_175750:exon2:c.T867C:p.F289F | . | 6qA3.3 | rs36330231 | 0 | 1 |
| chr6 | 32517026 | A | G | 221 | Plxna4 | exonic | synonymous SNV | Plxna4:NM_175750:exon2:c.T654C:p.H218H | . | 6qA3.3 | rs36332557 | 0 | 1 |
| chr6 | 32517140 | A | G | 222 | Plxna4 | exonic | synonymous SNV | Plxna4:NM_175750:exon2:c.T540C:p.D180D | . | 6qA3.3 | rs36641064 | 0 | 1 |
| chr6 | 32517197 | C | T | 222 | Plxna4 | exonic | synonymous SNV | Plxna4:NM_175750:exon2:c.G483A:p.G161G | . | 6qA3.3 | rs256996493 | 0 | 1 |
| chr6 | 32517446 | T | C | 221 | Plxna4 | exonic | synonymous SNV | Plxna4:NM_175750:exon2:c.A234G:p.K78K | . | 6qA3.3 | rs263746881 | 0 | 1 |
| chr6 | 32517693 | C | T | 164 | Plxna4 | UTR5 | . | . | . | 6qA3.3 | rs36306994 | 0 | 1 |
| chr6 | 33059934 | C | T | 198 | Chchd3 | intronic | . | . | 421 | 6qA3.3 | . | 0 | 1 |
| chr6 | 33060182 | A | G | 106 | . | upstream | . | . | 421 | 6qA3.3 | rs213236903 | 0 | 1 |
| chr6 | 33971925 | A | T | 222 | Exoc4 | exonic | missense SNV | Exoc4:NM_009148:exon18:c.A2702T:p.E901V | . | 6qA3.3 | . | 0 | 1 |
| chr6 | 33971926 | G | A | 222 | Exoc4 | exonic | synonymous SNV | Exoc4:NM_009148:exon18:c.G2703A:p.E901E | . | 6qA3.3 | . | 0 | 1 |
| chr6 | 34160620 | G | A | 156 | Slc35b4 | intronic | . | . | . | 6qA3.3 | . | 0 | 1 |
| chr6 | 34420121 | G | A | 116 | Akr1b7 | intronic | . | . | . | 6qB1 | . | 0 | 1 |
| chr6 | 34896148 | C | T | 72 | Wdr91 | intronic | . | . | . | 6qB1 | . | 0 | 1 |
| chr6 | 35313956 | G | T | 35.757 | Fam180a | intronic | . | . | . | 6qB1 | rs52575645 | 0 | 1 |
| chr6 | 36862700 | T | A | 43.9904 | Dgki | intronic | . | . | . | 6qB1 | . | 0 | 1 |
| chr6 | 36916381 | G | A | 114 | Dgki | intronic | . | . | . | 6qB1 | . | 0 | 1 |
| chr6 | 37021296 | G | C | 220 | Dgki | intronic | . | . | . | 6qB1 | . | 0 | 1 |
| chr6 | 37567235 | C | A | 176 | Akr1d1 | UTR3 | . | . | . | 6qB1 | . | 0 | 1 |
| chr6 | 38078735 | C | A | 222 | Atp6v0a4 | intronic | . | . | . | 6qB1 | . | 0 | 1 |
| chr6 | 39111596 | A | C | 159 | Parp12 | exonic | synonymous SNV | Parp12:NM_172893:exon3:c.T705G:p.T235T | . | 6qB1 | . | 0 | 1 |
| chr6 | 39400456 | T | A | 228 | Mkrn1 | exonic | missense SNV | Mkrn1:NM_001311109:exon6:c.A844T:p.N282Y,Mkrn1:NM_018810:exon6:c.A1036T:p.N346Y | . | 6qB1 | rs30899669 | 1 | 0 |
| chr6 | 40364246 | A | G | 206 | Agk | intronic | . | . | . | 6qB1 | . | 0 | 1 |
| chr6 | 40880111 | A | T | 129 | Moxd2,Mir1950 | ncRNA_intronic | . | . | . | 6qB1 | . | 0 | 1 |
| chr6 | 40884972 | T | G | 85 | Mir1950,Moxd2 | exonic | missense SNV | Moxd2:NM_139296:exon4:c.A665C:p.K222T | . | 6qB1 | . | 0 | 1 |
| chr6 | 40891579 | G | A | 56 | Mir1950 | ncRNA_intronic | . | . | . | 6qB1 | . | 0 | 1 |
| chr6 | 40900039 | A | C | 123 | Mir1950,Prss58 | ncRNA_intronic | . | . | . | 6qB1 | . | 0 | 1 |
| chr6 | 40929544 | G | A | 130 | 1700074P13Rik | UTR5 | . | . | . | 6qB1 | . | 0 | 1 |
| chr6 | 41162828 | A | T | 89 | . | intergenic | . | . | . | 6qB1 | . | 0 | 1 |
| chr6 | 41302159 | T | G | 142 | . | upstream | . | . | . | 6qB1 | . | 0 | 1 |
| chr6 | 41543103 | A | G | 222 | . | intergenic | . | . | . | 6qB1 | . | 0 | 1 |
| chr6 | 41614375 | A | G | 33.4999 | Ephb6 | exonic | synonymous SNV | Ephb6:NM_001146351:exon5:c.A465G:p.K155K,Ephb6:NM_007680:exon5:c.A465G:p.K155K | . | 6qB2.1 | . | 0 | 1 |
| chr6 | 41660211 | G | C | 85 | Trpv5 | intronic | . | . | . | 6qB2.1 | . | 0 | 1 |
| chr6 | 41674530 | T | G | 206 | Trpv5 | exonic | missense SNV | Trpv5:NM_001007572:exon6:c.A713C:p.K238T | . | 6qB2.1 | . | 0 | 1 |
| chr6 | 41698974 | A | G | 221 | Kel | exonic | missense SNV | Kel:NM_032540:exon8:c.T781C:p.S261P | . | 6qB2.1 | . | 0 | 1 |
| chr6 | 41771370 | G | T | 222 | Olfr459 | exonic | missense SNV | Olfr459:NM_146576:exon1:c.C928A:p.L310M | . | 6qB2.1 | . | 0 | 1 |
| chr6 | 41852093 | A | G | 64 | . | downstream | . | . | . | 6qB2.1 | . | 0 | 1 |
| chr6 | 42247325 | G | A | 125 | Gstk1 | intronic | . | . | . | 6qB2.1 | . | 0 | 1 |
| chr6 | 42435444 | G | A | 210 | Tas2r126 | exonic | missense SNV | Tas2r126:NM_207028:exon1:c.G910A:p.G304R | . | 6qB2.1 | . | 0 | 1 |
| chr6 | 42519540 | T | G | 136 | . | intergenic | . | . | . | 6qB2.1 | . | 0 | 1 |
| chr6 | 42911999 | C | A | 210 | Olfr447 | exonic | missense SNV | Olfr447:NM_146988:exon1:c.C475A:p.L159I | . | 6qB2.1 | . | 0 | 1 |

| | | | | | | | | | | | | | |
|---|---|---|---|---|---|---|---|---|---|---|---|---|---|
| chr6 | 43273710 | C | G | 134 | Arhgef5 | exonic | missense SNV | Arhgef5:NM_133674:exon2:c.C1394G:p.P465R | . | 6qB2.1 | . | 0 | 1 |
| chr6 | 43285272 | G | T | 221 | Arhgef5 | intronic | . | . | . | 6qB2.1 | . | 0 | 1 |
| chr6 | 46001087 | C | G | 65 | Cntnap2 | intronic | . | . | . | 6qB2.3 | . | 0 | 1 |
| chr6 | 46001097 | G | A | 68 | Cntnap2 | intronic | . | . | . | 6qB2.3 | . | 0 | 1 |
| chr6 | 47021414 | T | A | 116 | Cntnap2 | intronic | . | . | . | 6qB2.3 | rs251180894 | 0 | 1 |
| chr6 | 47021640 | A | T | 221 | Cntnap2 | exonic | synonymous SNV | Cntnap2:NM_001004357:exon17:c.A2625T:p.S875S | . | 6qB2.3 | rs252780151 | 0 | 1 |
| chr6 | 47021887 | T | C | 125 | Cntnap2 | intronic | . | . | . | 6qB2.3 | rs51114068 | 0 | 1 |
| chr6 | 47021889 | C | A | 124 | Cntnap2 | intronic | . | . | . | 6qB2.3 | . | 0 | 1 |
| chr6 | 47049039 | A | G | 93 | Cntnap2 | intronic | . | . | . | 6qB2.3 | . | 0 | 1 |
| chr6 | 47049277 | G | A | 221 | Cntnap2 | exonic | synonymous SNV | Cntnap2:NM_001004357:exon18:c.G2859A:p.A953A | . | 6qB2.3 | rs244297678 | 0 | 1 |
| chr6 | 47049537 | A | G | 221 | Cntnap2 | intronic | . | . | . | 6qB2.3 | rs260243289 | 0 | 1 |
| chr6 | 47049548 | C | T | 171 | Cntnap2 | intronic | . | . | . | 6qB2.3 | . | 0 | 1 |
| chr6 | 47432161 | T | G | 165 | . | intergenic | . | . | . | 6qB2.3 | . | 0 | 1 |
| chr6 | 47432495 | G | A | 95 | . | intergenic | . | . | . | 6qB2.3 | . | 0 | 1 |
| chr6 | 47432551 | T | G | 221 | . | intergenic | . | . | . | 6qB2.3 | rs38022963 | 0 | 1 |
| chr6 | 47432565 | A | G | 221 | . | intergenic | . | . | . | 6qB2.3 | rs584155559 | 0 | 1 |
| chr6 | 47432681 | C | G | 221 | . | intergenic | . | . | . | 6qB2.3 | . | 0 | 1 |
| chr6 | 47433053 | G | A | 221 | . | intergenic | . | . | . | 6qB2.3 | . | 0 | 1 |
| chr6 | 47516430 | T | C | 221 | Cul1 | intronic | . | . | . | 6qB2.3 | rs29962396 | 0 | 1 |
| chr6 | 47518449 | G | A | 170 | Cul1 | intronic | . | . | . | 6qB2.3 | rs6212026 | 0 | 1 |
| chr6 | 47519624 | G | A | 221 | Cul1 | intronic | . | . | . | 6qB2.3 | . | 0 | 1 |
| chr6 | 47523864 | A | T | 221 | Cul1 | intronic | . | . | . | 6qB2.3 | rs33863525 | 0 | 1 |
| chr6 | 47524472 | A | G | 221 | Cul1 | intronic | . | . | . | 6qB2.3 | . | 0 | 1 |
| chr6 | 47524812 | A | G | 79 | Cul1 | intronic | . | . | . | 6qB2.3 | . | 0 | 1 |
| chr6 | 47788244 | T | C | 221 | . | intergenic | . | . | . | 6qB2.3 | . | 0 | 1 |
| chr6 | 47796423 | A | G | 216 | Pdia4 | UTR3 | . | . | . | 6qB2.3 | rs30509424 | 0 | 1 |
| chr6 | 47806885 | G | A | 98 | Pdia4 | intronic | . | . | . | 6qB2.3 | . | 0 | 1 |
| chr6 | 47808362 | T | C | 221 | Pdia4 | intronic | . | . | . | 6qB2.3 | rs30719579 | 0 | 1 |
| chr6 | 47813113 | A | C | 84 | Pdia4 | intronic | . | . | . | 6qB2.3 | rs30754243 | 1 | 0 |
| chr6 | 47813177 | A | G | 119 | Pdia4 | intronic | . | . | . | 6qB2.3 | rs30549111 | 0 | 1 |
| chr6 | 47813221 | G | C | 191 | Pdia4 | exonic | missense SNV | Pdia4:NM_009787:exon1:c.C84G:p.H28Q | 476 | 6qB2.3 | rs13466818 | 0 | 1 |
| chr6 | 47815455 | G | A | 221 | . | intergenic | . | . | . | 6qB2.3 | rs107766942 | 0 | 1 |
| chr6 | 47815517 | T | G | 221 | . | intergenic | . | . | . | 6qB2.3 | rs108730277 | 0 | 1 |
| chr6 | 47815585 | T | G | 222 | . | intergenic | . | . | . | 6qB2.3 | rs48681455 | 0 | 1 |
| chr6 | 47815634 | T | C | 222 | . | intergenic | . | . | . | 6qB2.3 | rs48750288 | 0 | 1 |
| chr6 | 47819804 | T | C | 221 | Zfp786 | exonic | synonymous SNV | Zfp786:NM_177882:exon4:c.A2199G:p.G733G | . | 6qB2.3 | rs29968394 | 0 | 1 |
| chr6 | 47819831 | C | A | 221 | Zfp786 | exonic | missense SNV | Zfp786:NM_177882:exon4:c.G2172T:p.E724D | . | 6qB2.3 | rs30755359 | 0 | 1 |
| chr6 | 47820762 | A | T | 221 | Zfp786 | exonic | missense SNV | Zfp786:NM_177882:exon4:c.T1241A:p.L414H | . | 6qB2.3 | rs30268803 | 0 | 1 |
| chr6 | 47820897 | T | C | 221 | Zfp786 | exonic | missense SNV | Zfp786:NM_177882:exon4:c.A1106G:p.Y369C | . | 6qB2.3 | rs30948859 | 0 | 1 |
| chr6 | 47859321 | C | T | 167 | Zfp398 | intronic | . | . | . | 6qB2.3 | rs30908262 | 0 | 1 |
| chr6 | 47866674 | T | C | 221 | Zfp398 | exonic | synonymous SNV | Zfp398:NM_173034:exon5:c.T1263C:p.H421H,Zfp398:NM_027477:exon6:c.T1659C:p.H553H | 245 | 6qB2.3 | rs30600311 | 0 | 1 |
| chr6 | 47880179 | A | G | 221 | Zfp282 | exonic | synonymous SNV | Zfp282:NM_146175:exon2:c.A345G:p.Q115Q | . | 6qB2.3 | rs50934816 | 0 | 1 |
| chr6 | 47880212 | A | G | 221 | Zfp282 | exonic | synonymous SNV | Zfp282:NM_146175:exon2:c.A378G:p.T126T | . | 6qB2.3 | rs47784325 | 0 | 1 |
| chr6 | 47880496 | G | A | 216 | Zfp282 | intronic | . | . | . | 6qB2.3 | rs47103730 | 0 | 1 |
| chr6 | 47880554 | C | T | 129 | Zfp282 | intronic | . | . | . | 6qB2.3 | rs49175745 | 1 | 0 |
| chr6 | 47880572 | T | A | 61 | Zfp282 | intronic | . | . | . | 6qB2.3 | rs49565858 | 1 | 0 |
| chr6 | 47892699 | C | T | 121 | Zfp282 | intronic | . | . | . | 6qB2.3 | . | 0 | 1 |
| chr6 | 47897757 | C | T | 221 | Zfp282 | intronic | . | . | . | 6qB2.3 | . | 0 | 1 |
| chr6 | 47904492 | G | T | 49.2901 | Zfp282 | intronic | . | . | . | 6qB2.3 | . | 0 | 1 |
| chr6 | 47920870 | T | C | 221 | Zfp212 | intronic | . | . | 329 | 6qB2.3 | rs30022802 | 0 | 1 |
| chr6 | 47928838 | A | G | 125 | Zfp212 | intronic | . | . | . | 6qB2.3 | rs30023513 | 0 | 1 |
| chr6 | 47931273 | C | T | 221 | Zfp212 | exonic | synonymous SNV | Zfp212:NM_001145881:exon5:c.C1182T:p.T394T,Zfp212:NM_145576:exon5:c.C1185T:p.T395T | . | 6qB2.3 | . | 0 | 1 |
| chr6 | 47931706 | T | A | 86 | Zfp212 | UTR3 | . | . | . | 6qB2.3 | rs30907406 | 0 | 1 |
| chr6 | 47957529 | T | C | 221 | Zfp956 | exonic | synonymous SNV | Zfp956:NM_178898:exon3:c.T432C:p.G144G | . | 6qB2.3 | rs30215745 | 0 | 1 |
| chr6 | 47957582 | G | A | 221 | Zfp956 | exonic | missense SNV | Zfp956:NM_178898:exon3:c.G485A:p.R162H | . | 6qB2.3 | rs13478742 | 0 | 1 |
| chr6 | 47964153 | G | A | 222 | Zfp956 | exonic | missense SNV | Zfp956:NM_178898:exon5:c.G1445A:p.R482H | . | 6qB2.3 | . | 0 | 1 |
| chr6 | 47964756 | T | C | 181 | Zfp956 | UTR3 | . | . | . | 6qB2.3 | rs33893887 | 0 | 1 |
| chr6 | 47977242 | A | G | 221 | . | intergenic | . | . | . | 6qB2.3 | rs48394729 | 0 | 1 |
| chr6 | 47977573 | T | G | 221 | . | intergenic | . | . | . | 6qB2.3 | . | 0 | 1 |
| chr6 | 47977753 | G | A | 221 | . | intergenic | . | . | . | 6qB2.3 | . | 0 | 1 |
| chr6 | 48024477 | G | A | 105 | Zfp777 | UTR3 | . | . | . | 6qB2.3 | . | 1 | 0 |
| chr6 | 48025088 | C | T | 222 | Zfp777 | exonic | synonymous SNV | Zfp777:NM_001081382:exon6:c.G2199A:p.L733L | 795 | 6qB2.3 | . | 0 | 1 |
| chr6 | 48025275 | G | A | 222 | Zfp777 | exonic | missense SNV | Zfp777:NM_001081382:exon6:c.C2012T:p.S671L | 795 | 6qB2.3 | . | 0 | 1 |
| chr6 | 48025350 | A | T | 222 | Zfp777 | exonic | missense SNV | Zfp777:NM_001081382:exon6:c.T1937A:p.L646Q | 795 | 6qB2.3 | . | 0 | 1 |
| chr6 | 48025571 | G | A | 28.8489 | Zfp777 | exonic | synonymous SNV | Zfp777:NM_001081382:exon6:c.C1716T:p.H572H | . | 6qB2.3 | . | 0 | 1 |
| chr6 | 48037389 | A | C | 107 | Zfp777 | intronic | . | . | . | 6qB2.3 | rs233748062 | 0 | 1 |
| chr6 | 48037443 | A | G | 157 | Zfp777 | intronic | . | . | . | 6qB2.3 | rs49434322 | 0 | 1 |
| chr6 | 48037604 | T | C | 221 | Zfp777 | exonic | synonymous SNV | Zfp777:NM_001081382:exon4:c.A1167G:p.Q389Q | . | 6qB2.3 | rs51353507 | 0 | 1 |
| chr6 | 48063797 | C | T | 138 | Zfp746 | UTR3 | . | . | . | 6qB2.3 | rs51824345 | 0 | 1 |
| chr6 | 48063979 | G | A | 220 | Zfp746 | exonic | synonymous SNV | Zfp746:NM_001163475:exon7:c.C1812T:p.H604H | 203 | 6qB2.3 | rs51861736 | 0 | 1 |
| chr6 | 48064323 | T | C | 110 | Zfp746 | exonic | missense SNV | Zfp746:NM_001163475:exon7:c.A1468G:p.T490A | . | 6qB2.3 | rs50848307 | 1 | 0 |
| chr6 | 48066745 | A | G | 155 | Zfp746 | intronic | . | . | . | 6qB2.3 | rs48078564 | 0 | 1 |
| chr6 | 48082056 | T | C | 103 | Zfp746 | intronic | . | . | . | 6qB2.3 | . | 0 | 1 |
| chr6 | 48406207 | A | C | 169 | Krba1 | intronic | . | . | . | 6qB2.3 | . | 0 | 1 |
| chr6 | 48409415 | C | T | 220 | Krba1 | intronic | . | . | . | 6qB2.3 | . | 0 | 1 |
| chr6 | 48409716 | A | G | 107 | Krba1 | splicing | . | . | . | 6qB2.3 | . | 0 | 1 |
| chr6 | 48414018 | G | C | 208 | Krba1 | intronic | . | . | . | 6qB2.3 | rs38457451 | 0 | 1 |
| chr6 | 48416109 | A | T | 187 | Krba1 | exonic | missense SNV | Krba1:NM_133922:exon18:c.A2851T:p.I951F | . | 6qB2.3 | . | 0 | 1 |
| chr6 | 48434248 | A | G | 222 | Zfp467 | intronic | . | . | . | 6qB2.3 | rs29920448 | 0 | 1 |
| chr6 | 48434286 | T | A | 222 | Zfp467 | intronic | . | . | . | 6qB2.3 | rs38876057 | 0 | 1 |
| chr6 | 48434315 | G | C | 222 | Zfp467 | intronic | . | . | . | 6qB2.3 | rs30836616 | 0 | 1 |
| chr6 | 48437917 | G | A | 221 | Zfp467 | UTR3 | . | . | . | 6qB2.3 | . | 0 | 1 |
| chr6 | 48438167 | G | A | 210 | Zfp467 | exonic | missense SNV | Zfp467:NM_001085415:exon5:c.C1550T:p.A517V,Zfp467:NM_020589:exon5:c.C1550T:p.A517V | 1261 | 6qB2.3 | . | 0 | 1 |
| chr6 | 48438214 | C | G | 221 | Zfp467 | exonic | synonymous SNV | Zfp467:NM_001085415:exon5:c.G1503C:p.L501L,Zfp467:NM_020589:exon5:c.G1503C:p.L501L | 1261 | 6qB2.3 | rs29870476 | 0 | 1 |
| chr6 | 48438234 | A | C | 186 | Zfp467 | exonic | missense SNV | Zfp467:NM_001085415:exon5:c.T1483G:p.F495V,Zfp467:NM_020589:exon5:c.T1483G:p.F495V | 1261 | 6qB2.3 | . | 0 | 1 |
| chr6 | 48438520 | G | A | 204 | Zfp467 | exonic | synonymous SNV | Zfp467:NM_001085415:exon5:c.C1197T:p.A399A,Zfp467:NM_020589:exon5:c.C1197T:p.A399A | 1261 | 6qB2.3 | . | 0 | 1 |
| chr6 | 48438556 | A | C | 221 | Zfp467 | exonic | missense SNV | Zfp467:NM_001085415:exon5:c.T1161G:p.D387E,Zfp467:NM_020589:exon5:c.T1161G:p.D387E | 1261 | 6qB2.3 | rs37632802 | 0 | 1 |
| chr6 | 48439072 | G | C | 221 | Zfp467 | exonic | synonymous SNV | Zfp467:NM_001085415:exon5:c.C645G:p.P215P,Zfp467:NM_020589:exon5:c.C645G:p.P215P | 1261 | 6qB2.3 | . | 0 | 1 |
| chr6 | 48448953 | G | A | 128 | Sspo | intronic | . | . | . | 6qB2.3 | rs38975944 | 0 | 1 |
| chr6 | 48449520 | A | G | 89 | Sspo | exonic | missense SNV | Sspo:NM_173428:exon4:c.A472G:p.S158G | . | 6qB2.3 | . | 0 | 1 |
| chr6 | 48449626 | A | G | 215 | Sspo | intronic | . | . | . | 6qB2.3 | rs40145082 | 0 | 1 |
| chr6 | 48449723 | C | T | 219 | Sspo | intronic | . | . | . | 6qB2.3 | rs37790858 | 0 | 1 |
| chr6 | 48450967 | C | G | 202 | Sspo | intronic | . | . | . | 6qB2.3 | rs36570600 | 0 | 1 |
| chr6 | 48451298 | C | T | 200 | Sspo | intronic | . | . | . | 6qB2.3 | . | 0 | 1 |
| chr6 | 48451575 | T | C | 213 | Sspo | intronic | . | . | . | 6qB2.3 | rs30759223 | 0 | 1 |
| chr6 | 48451862 | C | T | 221 | Sspo | exonic | missense SNV | Sspo:NM_173428:exon9:c.C1210T:p.P404S | . | 6qB2.3 | . | 0 | 1 |
| chr6 | 48453944 | C | T | 221 | Sspo | intronic | . | . | . | 6qB2.3 | . | 0 | 1 |
| chr6 | 48454566 | C | T | 152 | Sspo | intronic | . | . | . | 6qB2.3 | . | 1 | 0 |
| chr6 | 48454992 | A | C | 221 | Sspo | exonic | synonymous SNV | Sspo:NM_173428:exon15:c.A2139C:p.T713T | . | 6qB2.3 | . | 0 | 1 |
| chr6 | 48455030 | C | T | 221 | Sspo | exonic | missense SNV | Sspo:NM_173428:exon15:c.C2177T:p.A726V | . | 6qB2.3 | . | 0 | 1 |
| chr6 | 48455120 | T | C | 222 | Sspo | exonic | missense SNV | Sspo:NM_173428:exon15:c.T2267C:p.F756S | . | 6qB2.3 | rs30515135 | 0 | 1 |
| chr6 | 48455122 | A | G | 222 | Sspo | exonic | missense SNV | Sspo:NM_173428:exon15:c.A2269G:p.T757A | . | 6qB2.3 | rs30058771 | 0 | 1 |
| chr6 | 48457544 | A | G | 222 | Sspo | intronic | . | . | . | 6qB2.3 | rs252502753 | 0 | 1 |
| chr6 | 48457861 | A | T | 222 | Sspo | intronic | . | . | . | 6qB2.3 | rs30849987 | 0 | 1 |
| chr6 | 48458855 | A | G | 121 | Sspo | intronic | . | . | . | 6qB2.3 | rs228083347 | 0 | 1 |
| chr6 | 48459453 | G | A | 221 | Sspo | intronic | . | . | . | 6qB2.3 | . | 0 | 1 |
| chr6 | 48460194 | A | T | 221 | Sspo | intronic | . | . | . | 6qB2.3 | rs30018336 | 0 | 1 |
| chr6 | 48460195 | A | C | 221 | Sspo | intronic | . | . | . | 6qB2.3 | rs30058956 | 0 | 1 |
| chr6 | 48460211 | A | G | 221 | Sspo | exonic | missense SNV | Sspo:NM_173428:exon26:c.A3985G:p.M1329V | . | 6qB2.3 | rs30313187 | 0 | 1 |
| chr6 | 48460417 | G | A | 221 | Sspo | exonic | missense SNV | Sspo:NM_173428:exon27:c.G4108A:p.A1370T | . | 6qB2.3 | . | 0 | 1 |
| chr6 | 48460435 | G | A | 221 | Sspo | exonic | missense SNV | Sspo:NM_173428:exon27:c.G4126A:p.E1376K | . | 6qB2.3 | . | 0 | 1 |
| chr6 | 48465289 | C | T | 75 | Sspo | intronic | . | . | . | 6qB2.3 | rs260328679 | 0 | 1 |
| chr6 | 48465839 | A | G | 220 | Sspo | intronic | . | . | . | 6qB2.3 | rs37882780 | 0 | 1 |
| chr6 | 48465856 | G | A | 220 | Sspo | intronic | . | . | . | 6qB2.3 | . | 0 | 1 |
| chr6 | 48475811 | G | T | 157 | Sspo | exonic | missense SNV | Sspo:NM_173428:exon56:c.G8452T:p.A2818S | . | 6qB2.3 | rs30070770 | 0 | 1 |
| chr6 | 48477382 | G | A | 129 | Sspo | intronic | . | . | . | 6qB2.3 | rs33893901 | 0 | 1 |
| chr6 | 48477861 | A | C | 221 | Sspo | exonic | missense SNV | Sspo:NM_173428:exon59:c.A8981C:p.E2994A | . | 6qB2.3 | rs30161604 | 0 | 1 |
| chr6 | 48478004 | A | G | 220 | Sspo | intronic | . | . | . | 6qB2.3 | rs30223084 | 0 | 1 |
| chr6 | 48478847 | C | T | 221 | Sspo | intronic | . | . | . | 6qB2.3 | rs36812878 | 0 | 1 |
| chr6 | 48479469 | G | C | 181 | Sspo | exonic | missense SNV | Sspo:NM_173428:exon62:c.G9350C:p.C3117S | . | 6qB2.3 | . | 0 | 1 |
| chr6 | 48483124 | A | G | 96 | Sspo | intronic | . | . | . | 6qB2.3 | rs30869090 | 0 | 1 |
| chr6 | 48483433 | T | C | 221 | Sspo | intronic | . | . | . | 6qB2.3 | rs29963232 | 0 | 1 |
| chr6 | 48483468 | A | G | 221 | Sspo | exonic | missense SNV | Sspo:NM_173428:exon69:c.A10189G:p.K3397E | . | 6qB2.3 | rs30315607 | 0 | 1 |
| chr6 | 48485626 | A | C | 117 | Sspo | intronic | . | . | . | 6qB2.3 | rs258580934 | 0 | 1 |
| chr6 | 48486415 | T | A | 222 | Sspo | exonic | missense SNV | Sspo:NM_173428:exon75:c.T11300A:p.V3767D | . | 6qB2.3 | rs246571252 | 0 | 1 |
| chr6 | 48486999 | T | C | 220 | Sspo | intronic | . | . | . | 6qB2.3 | . | 0 | 1 |
| chr6 | 48490567 | C | T | 221 | Sspo | intronic | . | . | . | 6qB2.3 | . | 0 | 1 |
| chr6 | 48496654 | C | T | 221 | Sspo | exonic | missense SNV | Sspo:NM_173428:exon97:c.C14744T:p.T4915M | . | 6qB2.3 | rs264478208 | 0 | 1 |
| chr6 | 48499011 | A | G | 98 | Sspo | intronic | . | . | . | 6qB2.3 | rs31003130 | 0 | 1 |
| chr6 | 48525056 | T | C | 187 | Zfp862-ps | ncRNA_exonic | . | . | . | 6qB2.3 | . | 0 | 1 |
| chr6 | 48534840 | C | T | 222 | . | downstream | . | . | . | 6qB2.3 | . | 0 | 1 |
| chr6 | 48539945 | C | A | 126 | Atp6v0e2 | intronic | . | . | . | 6qB2.3 | . | 0 | 1 |
| chr6 | 48568696 | A | G | 221 | Lrrc61 | exonic | missense SNV | Lrrc61:NM_001110160:exon2:c.A452G:p.Q151R,Lrrc61:NM_177736:exon3:c.A452G:p.Q151R | . | 6qB2.3 | rs30619919 | 0 | 1 |
| chr6 | 48569464 | C | T | 221 | Lrrc61 | UTR3 | . | . | . | 6qB2.3 | rs38433910 | 0 | 1 |
| chr6 | 48589394 | T | A | 70 | . | upstream | . | . | . | 6qB2.3 | rs50966299 | 0 | 1 |
| chr6 | 48589518 | C | T | 127 | Gm5111 | UTR5 | . | . | . | 6qB2.3 | rs51772299 | 0 | 1 |
| chr6 | 48589573 | G | A | 221 | Gm5111 | UTR5 | . | . | . | 6qB2.3 | . | 0 | 1 |
| chr6 | 48589653 | A | C | 221 | Gm5111 | UTR5 | . | . | . | 6qB2.3 | rs46299419 | 0 | 1 |
| chr6 | 48590182 | T | C | 73 | Gm5111 | exonic | synonymous SNV | Gm5111:NM_183309:exon2:c.T330C:p.H110H | . | 6qB2.3 | rs51281590 | 0 | 1 |
| chr6 | 48590229 | C | T | 104 | Gm5111 | exonic | missense SNV | Gm5111:NM_183309:exon2:c.C377T:p.T126I | . | 6qB2.3 | rs45735914 | 0 | 1 |
| chr6 | 48590275 | C | T | 222 | Gm5111 | exonic | synonymous SNV | Gm5111:NM_183309:exon2:c.C423T:p.L141L | . | 6qB2.3 | rs48372926 | 0 | 1 |
| chr6 | 48590308 | C | T | 221 | Gm5111 | exonic | synonymous SNV | Gm5111:NM_183309:exon2:c.C456T:p.D152D | . | 6qB2.3 | rs48859730 | 0 | 1 |
| chr6 | 48590417 | A | G | 221 | Gm5111 | exonic | missense SNV | Gm5111:NM_183309:exon2:c.A565G:p.N189D | . | 6qB2.3 | rs46609166 | 0 | 1 |
| chr6 | 48590558 | A | G | 128 | Gm5111 | UTR3 | . | . | . | 6qB2.3 | . | 0 | 1 |
| chr6 | 48590584 | C | T | 145 | Gm5111 | UTR3 | . | . | . | 6qB2.3 | rs48535016 | 1 | 0 |
| chr6 | 48597314 | G | T | 220 | Repin1 | exonic | synonymous SNV | Repin1:NM_001079905:exon2:c.G1008T:p.G336G,Repin1:NM_175099:exon2:c.G1008T:p.G336G,Repin1:NM | 872 | 6qB2.3 | . | 0 | 1 |
| chr6 | 48620352 | A | G | 221 | Zfp775 | exonic | missense SNV | Zfp775:NM_173429:exon2:c.A1159G:p.T387A | 765 | 6qB2.3 | rs30917939 | 0 | 1 |
| chr6 | 48658695 | A | G | 221 | Gimap8 | intronic | . | . | . | 6qB2.3 | rs30805642 | 0 | 1 |
| chr6 | 48659305 | A | G | 221 | Gimap8 | exonic | missense SNV | Gimap8:NM_212486:exon5:c.A2003G:p.H668R,Gimap8:NM_001077410:exon6:c.A2003G:p.H668R | . | 6qB2.3 | rs37474540 | 0 | 1 |
| chr6 | 48677397 | T | C | 221 | Gimap9 | intronic | . | . | . | 6qB2.3 | rs30263504 | 0 | 1 |
| chr6 | 48742767 | T | C | 222 | Gimap1 | exonic | synonymous SNV | Gimap1:NM_008376:exon3:c.T312C:p.F104F,Gimap1:NM_175860:exon3:c.T312C:p.F104F | 450 | 6qB2.3 | rs29874491 | 0 | 1 |
| chr6 | 48742853 | C | A | 221 | Gimap1 | exonic | missense SNV | Gimap1:NM_008376:exon3:c.C398A:p.A133E,Gimap1:NM_175860:exon3:c.C398A:p.A133E | 450 | 6qB2.3 | . | 0 | 1 |
| chr6 | 48743032 | G | A | 221 | Gimap1 | exonic | missense SNV | Gimap1:NM_008376:exon3:c.G577A:p.G193S,Gimap1:NM_175860:exon3:c.G577A:p.G193S | 450 | 6qB2.3 | . | 0 | 1 |
| chr6 | 48743308 | A | C | 221 | Gimap1 | exonic | missense SNV | Gimap1:NM_008376:exon3:c.A853C:p.I285L,Gimap1:NM_175860:exon3:c.A853C:p.I285L | . | 6qB2.3 | . | 0 | 1 |
| chr6 | 48752654 | C | G | 221 | Gimap5 | exonic | missense SNV | Gimap5:NM_175035:exon3:c.C157G:p.Q53E | . | 6qB2.3 | rs37352392 | 0 | 1 |
| chr6 | 48752824 | A | C | 221 | Gimap5 | exonic | synonymous SNV | Gimap5:NM_175035:exon3:c.A327C:p.G109G | . | 6qB2.3 | rs46198457 | 0 | 1 |
| chr6 | 48753023 | A | G | 222 | Gimap5 | exonic | missense SNV | Gimap5:NM_175035:exon3:c.A526G:p.T176A | . | 6qB2.3 | rs46153374 | 0 | 1 |
| chr6 | 48753024 | C | T | 222 | Gimap5 | exonic | missense SNV | Gimap5:NM_175035:exon3:c.C527T:p.T176I | . | 6qB2.3 | rs49310329 | 0 | 1 |
| chr6 | 48753117 | G | A | 183 | Gimap5 | exonic | missense SNV | Gimap5:NM_175035:exon3:c.G620A:p.R207K | . | 6qB2.3 | rs48757751 | 0 | 1 |
| chr6 | 48753121 | G | T | 189 | Gimap5 | exonic | missense SNV | Gimap5:NM_175035:exon3:c.G624T:p.R208S | . | 6qB2.3 | rs47624644 | 0 | 1 |
| chr6 | 48753175 | T | C | 222 | Gimap5 | exonic | synonymous SNV | Gimap5:NM_175035:exon3:c.T678C:p.A226A | . | 6qB2.3 | rs49210291 | 0 | 1 |
| chr6 | 48753266 | C | T | 222 | Gimap5 | exonic | missense SNV | Gimap5:NM_175035:exon3:c.C769T:p.R257W | . | 6qB2.3 | rs48795944 | 0 | 1 |
| chr6 | 48753389 | A | G | 222 | Gimap5 | exonic | missense SNV | Gimap5:NM_175035:exon3:c.A892G:p.T298A | . | 6qB2.3 | rs36970291 | 0 | 1 |
| chr6 | 48765982 | G | T | 220 | Gimap3 | intronic | . | . | . | 6qB2.3 | . | 0 | 1 |
| chr6 | 48833899 | A | G | 130 | Tmem176b | UTR3 | . | . | . | 6qB2.3 | rs36773899 | 0 | 1 |
| chr6 | 48834024 | A | C | 222 | Tmem176b | UTR3 | . | . | . | 6qB2.3 | rs258607176 | 0 | 1 |
| chr6 | 48834044 | G | T | 222 | Tmem176b | UTR3 | . | . | . | 6qB2.3 | rs580350470 | 0 | 1 |
| chr6 | 48834103 | G | C | 222 | Tmem176b | exonic | missense SNV | Tmem176b:NM_001164207:exon7:c.C742G:p.H248D,Tmem176b:NM_001164208:exon7:c.C742G:p.H248D,Tmem1 | . | 6qB2.3 | rs36418269 | 0 | 1 |
| chr6 | 48834476 | T | C | 171 | Tmem176b | intronic | . | . | . | 6qB2.3 | rs38134601 | 0 | 1 |
| chr6 | 48835315 | T | C | 104 | Tmem176b | intronic | . | . | . | 6qB2.3 | rs37061586 | 0 | 1 |
| chr6 | 48835349 | G | A | 80 | Tmem176b | intronic | . | . | . | 6qB2.3 | . | 0 | 1 |
| chr6 | 48835432 | G | A | 195 | Tmem176b | intronic | . | . | . | 6qB2.3 | . | 0 | 1 |
| chr6 | 48835526 | G | A | 222 | Tmem176b | exonic | synonymous SNV | Tmem176b:NM_001164207:exon5:c.C504T:p.D168D,Tmem176b:NM_001164208:exon5:c.C504T:p.D168D,Tmem1 | . | 6qB2.3 | . | 0 | 1 |
| chr6 | 48835930 | A | G | 220 | Tmem176b | exonic | synonymous SNV | Tmem176b:NM_001164207:exon4:c.T330C:p.H110H,Tmem176b:NM_001164208:exon4:c.T330C:p.H110H,Tmem1 | . | 6qB2.3 | rs37876194 | 0 | 1 |
| chr6 | 48838109 | A | G | 221 | Tmem176b | intronic | . | . | . | 6qB2.3 | rs37672711 | 0 | 1 |
| chr6 | 48838507 | C | T | 114 | Tmem176b | intronic | . | . | . | 6qB2.3 | rs265033517 | 0 | 1 |
| chr6 | 48838525 | A | G | 93 | Tmem176b | intronic | . | . | . | 6qB2.3 | rs232290077 | 0 | 1 |
| chr6 | 48842049 | T | A | 156 | Tmem176a | intronic | . | . | . | 6qB2.3 | . | 0 | 1 |
| chr6 | 48842206 | G | A | 222 | Tmem176a | UTR5 | . | . | . | 6qB2.3 | rs241380789 | 0 | 1 |
| chr6 | 48842234 | G | A | 222 | Tmem176a | exonic | missense SNV | Tmem176a:NM_001098271:exon2:c.G28A:p.V10I,Tmem176a:NM_025326:exon2:c.G28A:p.V10I | . | 6qB2.3 | rs218403256 | 0 | 1 |
| chr6 | 48842680 | C | G | 200 | Tmem176a | exonic | missense SNV | Tmem176a:NM_001098271:exon3:c.C287G:p.P96R,Tmem176a:NM_025326:exon3:c.C287G:p.P96R | . | 6qB2.3 | . | 0 | 1 |
| chr6 | 48842730 | G | C | 220 | Tmem176a | intronic | . | . | . | 6qB2.3 | . | 0 | 1 |
| chr6 | 48842735 | A | G | 220 | Tmem176a | intronic | . | . | . | 6qB2.3 | . | 0 | 1 |
| chr6 | 48842792 | C | T | 102 | Tmem176a | intronic | . | . | . | 6qB2.3 | rs236199496 | 0 | 1 |
| chr6 | 48844053 | T | C | 221 | Tmem176a | exonic | synonymous SNV | Tmem176a:NM_001098271:exon5:c.T456C:p.Y152Y,Tmem176a:NM_025326:exon5:c.T456C:p.Y152Y | . | 6qB2.3 | rs212926452 | 0 | 1 |

| | | | | | | | | | | | | | |
|---|---|---|---|---|---|---|---|---|---|---|---|---|---|
| chr6 | 48844137 | T | C | 221 | Tmem176a | exonic | synonymous SNV | Tmem176a:NM_001098271:exon5:c.T540C:p.A180A,Tmem176a:NM_025326:exon5:c.T540C:p.A180A | . | 6qB2.3 | . | 0 | 1 |
| chr6 | 48844363 | G | A | 117 | Tmem176a | intronic | . | . | . | 6qB2.3 | rs246366595 | 0 | 1 |
| chr6 | 48844394 | G | C | 115 | Tmem176a | intronic | . | . | . | 6qB2.3 | rs253917490 | 0 | 1 |
| chr6 | 48845165 | C | T | 221 | Tmem176a | UTR3 | . | . | . | 6qB2.3 | . | 0 | 1 |
| chr6 | 48860257 | G | T | 221 | . | intergenic | . | . | . | 6qB2.3 | . | 0 | 1 |
| chr6 | 48860362 | G | A | 219 | . | intergenic | . | . | . | 6qB2.3 | rs242686506 | 0 | 1 |
| chr6 | 48860363 | C | T | 219 | . | intergenic | . | . | . | 6qB2.3 | rs261156797 | 0 | 1 |
| chr6 | 48860592 | C | T | 202 | . | intergenic | . | . | . | 6qB2.3 | . | 0 | 1 |
| chr6 | 48860599 | A | G | 204 | . | intergenic | . | . | . | 6qB2.3 | rs36648701 | 0 | 1 |
| chr6 | 48860713 | T | C | 135 | . | intergenic | . | . | . | 6qB2.3 | rs252881212 | 0 | 1 |
| chr6 | 48864098 | C | T | 128 | . | intergenic | . | . | . | 6qB2.3 | rs38237605 | 0 | 1 |
| chr6 | 48864201 | C | T | 222 | . | intergenic | . | . | . | 6qB2.3 | . | 0 | 1 |
| chr6 | 48864283 | G | A | 222 | . | intergenic | . | . | . | 6qB2.3 | rs250288096 | 0 | 1 |
| chr6 | 48864286 | G | T | 222 | . | intergenic | . | . | . | 6qB2.3 | rs218008927 | 0 | 1 |
| chr6 | 48906789 | A | G | 220 | Aoc1 | intronic | . | . | . | 6qB2.3 | . | 0 | 1 |
| chr6 | 48929887 | G | A | 114 | . | upstream | . | . | . | 6qB2.3 | rs47458710 | 0 | 1 |
| chr6 | 48929888 | A | C | 110 | . | upstream | . | . | . | 6qB2.3 | rs51329236 | 0 | 1 |
| chr6 | 48930581 | T | C | 220 | 1600015I10Rik | exonic | missense SNV | 1600015I10Rik:NM_001081273:exon1:c.T514C:p.S172P | . | 6qB2.3 | rs30425782 | 0 | 1 |
| chr6 | 48930886 | A | C | 221 | 1600015I10Rik | exonic | synonymous SNV | 1600015I10Rik:NM_001081273:exon1:c.A819C:p.V273V | . | 6qB2.3 | rs30510019 | 0 | 1 |
| chr6 | 48931033 | G | A | 221 | 1600015I10Rik | exonic | synonymous SNV | 1600015I10Rik:NM_001081273:exon1:c.G966A:p.T322T | . | 6qB2.3 | rs30905792 | 0 | 1 |
| chr6 | 48931108 | T | C | 221 | 1600015I10Rik | exonic | synonymous SNV | 1600015I10Rik:NM_001081273:exon1:c.T1041C:p.F347F | . | 6qB2.3 | rs36976457 | 0 | 1 |
| chr6 | 48931519 | A | G | 221 | 1600015I10Rik | exonic | synonymous SNV | 1600015I10Rik:NM_001081273:exon1:c.A1452G:p.K484K | . | 6qB2.3 | rs30122788 | 0 | 1 |
| chr6 | 48931706 | G | A | 221 | 1600015I10Rik | intronic | . | . | . | 6qB2.3 | rs30947204 | 0 | 1 |
| chr6 | 48931757 | A | G | 160 | 1600015I10Rik | intronic | . | . | . | 6qB2.3 | rs30709789 | 0 | 1 |
| chr6 | 48931759 | C | G | 146 | 1600015I10Rik | intronic | . | . | . | 6qB2.3 | rs30466445 | 0 | 1 |
| chr6 | 48932261 | G | C | 94 | 1600015I10Rik | intronic | . | . | . | 6qB2.3 | rs30692589 | 0 | 1 |
| chr6 | 48932281 | A | C | 116 | 1600015I10Rik | intronic | . | . | . | 6qB2.3 | rs29873639 | 0 | 1 |
| chr6 | 48932323 | C | T | 179 | 1600015I10Rik | intronic | . | . | . | 6qB2.3 | rs30811147 | 0 | 1 |
| chr6 | 48932764 | C | T | 220 | 1600015I10Rik | intronic | . | . | . | 6qB2.3 | rs29826259 | 0 | 1 |
| chr6 | 48933708 | G | A | 55 | . | downstream | . | . | . | 6qB2.3 | rs47192866 | 0 | 1 |
| chr6 | 48939563 | G | A | 56 | . | intergenic | . | . | . | 6qB2.3 | rs38151027 | 0 | 1 |
| chr6 | 48939686 | C | T | 171 | . | intergenic | . | . | . | 6qB2.3 | rs37754374 | 0 | 1 |
| chr6 | 48939997 | A | G | 221 | . | intergenic | . | . | . | 6qB2.3 | rs29924623 | 0 | 1 |
| chr6 | 48956064 | A | G | 88 | . | intergenic | . | . | . | 6qB2.3 | rs37130581 | 1 | 0 |
| chr6 | 48956465 | G | A | 222 | . | intergenic | . | . | . | 6qB2.3 | . | 0 | 1 |
| chr6 | 48956671 | T | C | 52 | . | intergenic | . | . | . | 6qB2.3 | rs224363246 | 0 | 1 |
| chr6 | 48975184 | C | T | 221 | Dox12 | exonic | synonymous SNV | Dox12:NM_001029987:exon1:c.C42T:p.A14A | . | 6qB2.3 | . | 0 | 1 |
| chr6 | 48975313 | G | A | 221 | Dox12 | exonic | synonymous SNV | Dox12:NM_001029987:exon1:c.G171A:p.T57T | . | 6qB2.3 | rs233613590 | 0 | 1 |
| chr6 | 48975646 | G | T | 221 | Dox12 | exonic | missense SNV | Dox12:NM_001029987:exon1:c.G504T:p.K168N | . | 6qB2.3 | . | 0 | 1 |
| chr6 | 48976134 | G | T | 221 | Dox12 | exonic | missense SNV | Dox12:NM_001029987:exon1:c.G992T:p.G331V | . | 6qB2.3 | . | 0 | 1 |
| chr6 | 48976333 | C | T | 221 | Dox12 | exonic | synonymous SNV | Dox12:NM_001029987:exon1:c.C1191T:p.D397D | . | 6qB2.3 | . | 0 | 1 |
| chr6 | 48976754 | A | G | 221 | Dox12 | intronic | . | . | . | 6qB2.3 | . | 0 | 1 |
| chr6 | 48976783 | A | C | 213 | Dox12 | intronic | . | . | . | 6qB2.3 | rs220113130 | 0 | 1 |
| chr6 | 48978356 | G | A | 222 | Dox12 | intronic | . | . | . | 6qB2.3 | rs255825118 | 0 | 1 |
| chr6 | 48978591 | A | G | 221 | Dox12 | exonic | missense SNV | Dox12:NM_001029987:exon4:c.A2134G:p.I712V | . | 6qB2.3 | rs249959172 | 0 | 1 |
| chr6 | 48981078 | T | C | 221 | . | intergenic | . | . | . | 6qB2.3 | rs30366403 | 0 | 1 |
| chr6 | 48981204 | G | A | 174 | . | intergenic | . | . | . | 6qB2.3 | . | 0 | 1 |
| chr6 | 48981212 | G | T | 133 | . | intergenic | . | . | . | 6qB2.3 | . | 0 | 1 |
| chr6 | 48981216 | A | G | 83 | . | intergenic | . | . | . | 6qB2.3 | rs37024854 | 0 | 1 |
| chr6 | 48981226 | A | G | 61 | . | intergenic | . | . | . | 6qB2.3 | rs30023478 | 0 | 1 |
| chr6 | 48981271 | C | T | 48.2975 | . | intergenic | . | . | . | 6qB2.3 | rs30411368 | 0 | 1 |
| chr6 | 48981340 | A | G | 117 | . | intergenic | . | . | . | 6qB2.3 | rs30171378 | 0 | 1 |
| chr6 | 48981717 | T | G | 221 | . | intergenic | . | . | . | 6qB2.3 | rs48801920 | 0 | 1 |
| chr6 | 48981857 | A | G | 222 | . | intergenic | . | . | . | 6qB2.3 | rs221593707 | 0 | 1 |
| chr6 | 48981861 | A | G | 222 | . | intergenic | . | . | . | 6qB2.3 | rs230079021 | 0 | 1 |
| chr6 | 48981958 | A | G | 122 | . | intergenic | . | . | . | 6qB2.3 | rs29917983 | 0 | 1 |
| chr6 | 48988280 | C | T | 221 | 4930563H07Rik,Svs | exonic | synonymous SNV | Svs1:NM_172888:exon1:c.C1221T:p.G407G | . | 6qB2.3 | . | 0 | 1 |
| chr6 | 48988654 | G | A | 221 | Svs1,4930563H07Ri | exonic | missense SNV | Svs1:NM_172888:exon1:c.G1595A:p.R532K | . | 6qB2.3 | . | 0 | 1 |
| chr6 | 48988962 | G | A | 220 | Svs1,4930563H07Ri | ncRNA_intronic | . | . | . | 6qB2.3 | . | 0 | 1 |
| chr6 | 48989095 | C | T | 147 | Svs1,4930563H07Ri | ncRNA_intronic | . | . | . | 6qB2.3 | rs30895313 | 0 | 1 |
| chr6 | 48989813 | T | G | 65 | Svs1,4930563H07Ri | ncRNA_intronic | . | . | . | 6qB2.3 | rs30562533 | 0 | 1 |
| chr6 | 48990372 | G | T | 221 | 4930563H07Rik,Svs | ncRNA_intronic | . | . | . | 6qB2.3 | rs37574703 | 0 | 1 |
| chr6 | 48990419 | C | T | 172 | 4930563H07Rik,Svs | ncRNA_intronic | . | . | . | 6qB2.3 | . | 0 | 1 |
| chr6 | 48991138 | C | A | 222 | Svs1,4930563H07Ri | ncRNA_intronic | . | . | . | 6qB2.3 | rs30368081 | 0 | 1 |
| chr6 | 48991645 | A | G | 221 | 4930563H07Rik,Svs | ncRNA_exonic | . | . | . | 6qB2.3 | rs30270241 | 0 | 1 |
| chr6 | 49042629 | A | C | 121 | Gpnmb | intronic | . | . | . | 6qB2.3 | rs36260719 | 0 | 1 |
| chr6 | 49042937 | C | T | 222 | Gpnmb | intronic | . | . | . | 6qB2.3 | . | 0 | 1 |
| chr6 | 49042959 | G | A | 211 | Gpnmb | intronic | . | . | . | 6qB2.3 | rs218377428 | 0 | 1 |
| chr6 | 49042985 | T | A | 33.0132 | Gpnmb | intronic | . | . | . | 6qB2.3 | rs220187835 | 0 | 1 |
| chr6 | 49043874 | G | A | 140 | Gpnmb | intronic | . | . | . | 6qB2.3 | rs240438734 | 0 | 1 |
| chr6 | 49044037 | T | C | 222 | Gpnmb | exonic | synonymous SNV | Gpnmb:NM_053110:exon3:c.T264C:p.A88A | . | 6qB2.3 | rs229856277 | 0 | 1 |
| chr6 | 49044200 | G | T | 221 | Gpnmb | intronic | . | . | . | 6qB2.3 | rs226868368 | 0 | 1 |
| chr6 | 49045318 | A | T | 220 | Gpnmb | exonic | synonymous SNV | Gpnmb:NM_053110:exon4:c.A381T:p.T127T | . | 6qB2.3 | rs30606480 | 0 | 1 |
| chr6 | 49045531 | G | A | 221 | Gpnmb | intronic | . | . | . | 6qB2.3 | . | 0 | 1 |
| chr6 | 49045535 | T | G | 221 | Gpnmb | intronic | . | . | . | 6qB2.3 | rs38378746 | 0 | 1 |
| chr6 | 49045628 | C | T | 115 | Gpnmb | intronic | . | . | . | 6qB2.3 | . | 0 | 1 |
| chr6 | 49045657 | C | A | 85 | Gpnmb | intronic | . | . | . | 6qB2.3 | rs249577274 | 0 | 1 |
| chr6 | 49047081 | G | A | 133 | Gpnmb | intronic | . | . | . | 6qB2.3 | rs260623962 | 0 | 1 |
| chr6 | 49055683 | C | T | 221 | Gpnmb | exonic | missense SNV | Gpnmb:NM_053110:exon10:c.C1540T:p.L514F | . | 6qB2.3 | rs38890885 | 0 | 1 |
| chr6 | 49055886 | A | G | 83 | Gpnmb | intronic | . | . | . | 6qB2.3 | rs30963924 | 0 | 1 |
| chr6 | 49056218 | G | A | 221 | Gpnmb | exonic | synonymous SNV | Gpnmb:NM_053110:exon11:c.G1629A:p.K543K | . | 6qB2.3 | rs30014223 | 0 | 1 |
| chr6 | 49056239 | T | C | 222 | Gpnmb | exonic | synonymous SNV | Gpnmb:NM_053110:exon11:c.T1650C:p.S550S | . | 6qB2.3 | rs30219354 | 0 | 1 |
| chr6 | 49071957 | A | G | 221 | . | intergenic | . | . | . | 6qB2.3 | rs30060404 | 0 | 1 |
| chr6 | 49072062 | T | C | 213 | . | intergenic | . | . | . | 6qB2.3 | . | 0 | 1 |
| chr6 | 49080736 | G | A | 222 | Malsu1 | intronic | . | . | . | 6qB2.3 | rs251444253 | 0 | 1 |
| chr6 | 49080738 | G | A | 222 | Malsu1 | intronic | . | . | . | 6qB2.3 | rs220913569 | 0 | 1 |
| chr6 | 49080796 | G | A | 222 | Malsu1 | intronic | . | . | . | 6qB2.3 | rs51929006 | 0 | 1 |
| chr6 | 49081117 | G | T | 200 | Malsu1 | intronic | . | . | . | 6qB2.3 | rs50622503 | 0 | 1 |
| chr6 | 49081118 | C | A | 206 | Malsu1 | intronic | . | . | . | 6qB2.3 | rs37536962 | 0 | 1 |
| chr6 | 49081132 | C | A | 200 | Malsu1 | intronic | . | . | . | 6qB2.3 | . | 0 | 1 |
| chr6 | 49088273 | T | C | 74 | Igf2bp3 | intronic | . | . | . | 6qB2.3 | rs232033891 | 0 | 1 |
| chr6 | 49088277 | A | G | 74 | Igf2bp3 | intronic | . | . | . | 6qB2.3 | rs261920775 | 0 | 1 |
| chr6 | 49088335 | G | A | 142 | Igf2bp3 | intronic | . | . | . | 6qB2.3 | rs37112988 | 0 | 1 |
| chr6 | 49105455 | A | G | 164 | Igf2bp3 | intronic | . | . | . | 6qB2.3 | . | 0 | 1 |
| chr6 | 49109169 | T | C | 221 | Igf2bp3 | intronic | . | . | . | 6qB2.3 | rs38502788 | 0 | 1 |
| chr6 | 49117099 | C | A | 76 | Igf2bp3 | intronic | . | . | . | 6qB2.3 | . | 0 | 1 |
| chr6 | 49117129 | A | G | 221 | Igf2bp3 | intronic | . | . | . | 6qB2.3 | rs30313814 | 0 | 1 |
| chr6 | 49168697 | T | C | 106 | Igf2bp3 | intronic | . | . | . | 6qB2.3 | rs30763962 | 0 | 1 |
| chr6 | 49214382 | C | A | 199 | Igf2bp3 | exonic | synonymous SNV | Igf2bp3:NM_023670:exon1:c.G90T:p.A30A | 1785 | 6qB2.3 | . | 0 | 1 |
| chr6 | 49245352 | C | T | 127 | Tra2a | intronic | . | . | . | 6qB2.3 | rs30267112 | 0 | 1 |
| chr6 | 49249342 | T | C | 122 | Tra2a | intronic | . | . | . | 6qB2.3 | rs30989339 | 0 | 1 |
| chr6 | 49262964 | A | G | 221 | Tra2a | intronic | . | . | . | 6qB2.3 | . | 0 | 1 |
| chr6 | 49334042 | C | G | 221 | Ccdc126 | UTR5 | . | . | . | 6qB2.3 | . | 0 | 1 |
| chr6 | 49340072 | A | C | 89 | Ccdc126 | UTR3 | . | . | . | 6qB2.3 | rs4225893 | 0 | 1 |
| chr6 | 49388836 | C | T | 222 | Fam221a | exonic | missense SNV | Fam221a:NM_172727:exon7:c.C857T:p.A286V | . | 6qB2.3 | . | 0 | 1 |
| chr6 | 49388953 | A | G | 222 | Fam221a | UTR3 | . | . | . | 6qB2.3 | rs36311132 | 0 | 1 |
| chr6 | 49389031 | G | A | 127 | Fam221a | UTR3 | . | . | . | 6qB2.3 | rs247044185 | 0 | 1 |
| chr6 | 49389092 | G | A | 31.97 | Fam221a | UTR3 | . | . | . | 6qB2.3 | rs37699610 | 0 | 1 |
| chr6 | 49395513 | A | C | 152 | . | upstream | . | . | 413 | 6qB2.3 | . | 0 | 1 |
| chr6 | 49395532 | C | T | 175 | . | upstream | . | . | 413 | 6qB2.3 | rs3658007 | 0 | 1 |
| chr6 | 49395687 | C | T | 221 | Stk31 | splicing | . | . | 413 | 6qB2.3 | rs3659178 | 0 | 1 |
| chr6 | 49395733 | T | C | 207 | Stk31 | intronic | . | . | 413 | 6qB2.3 | rs229809129 | 0 | 1 |
| chr6 | 49395746 | G | A | 188 | Stk31 | intronic | . | . | 413 | 6qB2.3 | rs38174930 | 0 | 1 |
| chr6 | 49397973 | T | G | 90 | Stk31 | intronic | . | . | . | 6qB2.3 | . | 0 | 1 |
| chr6 | 49398142 | A | G | 221 | Stk31 | intronic | . | . | . | 6qB2.3 | rs48785285 | 0 | 1 |
| chr6 | 49419345 | T | C | 221 | Stk31 | intronic | . | . | . | 6qB2.3 | rs30951346 | 0 | 1 |
| chr6 | 49424488 | A | G | 221 | Stk31 | intronic | . | . | . | 6qB2.3 | rs30611350 | 0 | 1 |
| chr6 | 49823467 | G | T | 121 | Npy | intronic | . | . | . | 6qB2.3 | . | 0 | 1 |
| chr6 | 49823478 | G | A | 119 | Npy | intronic | . | . | . | 6qB2.3 | rs36242616 | 0 | 1 |
| chr6 | 49823708 | C | T | 182 | Npy | exonic | synonymous SNV | Npy:NM_023456:exon2:c.C111T:p.G37G | . | 6qB2.3 | . | 0 | 1 |
| chr6 | 49823837 | A | G | 220 | Npy | intronic | . | . | . | 6qB2.3 | rs47087750 | 0 | 1 |
| chr6 | 49827419 | C | A | 193 | Npy | intronic | . | . | . | 6qB2.3 | rs37575453 | 0 | 1 |
| chr6 | 49827510 | T | C | 221 | Npy | exonic | synonymous SNV | Npy:NM_023456:exon3:c.T210C:p.P70P | . | 6qB2.3 | rs36514938 | 0 | 1 |
| chr6 | 49829244 | G | A | 222 | Npy | intronic | . | . | . | 6qB2.3 | rs30543896 | 0 | 1 |
| chr6 | 49829537 | C | T | 79 | . | downstream | . | . | . | 6qB2.3 | rs37049163 | 0 | 1 |
| chr6 | 50043310 | T | G | 109 | . | intergenic | . | . | . | 6qB2.3 | . | 0 | 1 |
| chr6 | 50158994 | C | T | 99 | Mpp6 | intronic | . | . | . | 6qB2.3 | rs29657476 | 0 | 1 |
| chr6 | 50162433 | C | T | 106 | Mpp6 | intronic | . | . | . | 6qB2.3 | rs30613020 | 0 | 1 |
| chr6 | 50176689 | C | T | 124 | Mpp6 | intronic | . | . | . | 6qB2.3 | rs29664570 | 0 | 1 |
| chr6 | 50176702 | C | T | 127 | Mpp6 | intronic | . | . | . | 6qB2.3 | rs47357923 | 0 | 1 |
| chr6 | 50176955 | A | G | 221 | Mpp6 | intronic | . | . | . | 6qB2.3 | rs52002773 | 0 | 1 |
| chr6 | 50180278 | G | A | 221 | Mpp6 | splicing | . | . | . | 6qB2.3 | rs29659398 | 0 | 1 |
| chr6 | 50180432 | T | A | 80 | Mpp6 | intronic | . | . | . | 6qB2.3 | . | 0 | 1 |
| chr6 | 50194744 | C | T | 222 | Mpp6 | intronic | . | . | . | 6qB2.3 | rs29660938 | 0 | 1 |
| chr6 | 50196871 | G | A | 113 | Mpp6 | intronic | . | . | . | 6qB2.3 | rs29663768 | 0 | 1 |
| chr6 | 50207784 | A | G | 104 | Dfna5 | UTR3 | . | . | . | 6qB2.3 | rs29666934 | 0 | 1 |
| chr6 | 50207826 | C | T | 135 | Dfna5 | UTR3 | . | . | . | 6qB2.3 | rs29666936 | 0 | 1 |
| chr6 | 50208123 | G | A | 221 | Dfna5 | exonic | synonymous SNV | Dfna5:NM_018769:exon10:c.C1350T:p.I450I | . | 6qB2.3 | rs29666938 | 0 | 1 |
| chr6 | 50219231 | C | T | 221 | Dfna5 | intronic | . | . | . | 6qB2.3 | rs29666483 | 0 | 1 |
| chr6 | 50219418 | A | G | 222 | Dfna5 | exonic | synonymous SNV | Dfna5:NM_018769:exon8:c.T1011C:p.G337G | . | 6qB2.3 | rs3708108 | 0 | 1 |
| chr6 | 50220950 | G | A | 222 | Dfna5 | intronic | . | . | . | 6qB2.3 | rs29660806 | 0 | 1 |
| chr6 | 50221088 | A | G | 222 | Dfna5 | exonic | synonymous SNV | Dfna5:NM_018769:exon7:c.T891C:p.H297H | . | 6qB2.3 | rs29660808 | 0 | 1 |
| chr6 | 50221206 | C | T | 221 | Dfna5 | intronic | . | . | . | 6qB2.3 | rs29660812 | 0 | 1 |
| chr6 | 50221256 | G | A | 117 | Dfna5 | intronic | . | . | . | 6qB2.3 | rs29661716 | 0 | 1 |
| chr6 | 50222792 | A | G | 221 | Dfna5 | exonic | missense SNV | Dfna5:NM_018769:exon6:c.T806C:p.L269P | . | 6qB2.3 | rs30713457 | 0 | 1 |
| chr6 | 50227149 | G | A | 74 | Dfna5 | intronic | . | . | . | 6qB2.3 | rs30347928 | 0 | 1 |
| chr6 | 50227203 | G | T | 221 | Dfna5 | intronic | . | . | . | 6qB2.3 | rs29667392 | 0 | 1 |
| chr6 | 50227230 | C | A | 222 | Dfna5 | intronic | . | . | . | 6qB2.3 | rs29686634 | 0 | 1 |
| chr6 | 50227367 | C | T | 222 | Dfna5 | exonic | synonymous SNV | Dfna5:NM_018769:exon5:c.G609A:p.T203T | . | 6qB2.3 | rs29686638 | 0 | 1 |
| chr6 | 50227376 | C | A | 222 | Dfna5 | exonic | synonymous SNV | Dfna5:NM_018769:exon5:c.G600T:p.T200T | . | 6qB2.3 | rs30459531 | 0 | 1 |
| chr6 | 50229183 | C | T | 104 | Dfna5 | intronic | . | . | . | 6qB2.3 | rs29689955 | 0 | 1 |
| chr6 | 50229196 | C | T | 125 | Dfna5 | intronic | . | . | . | 6qB2.3 | rs223203716 | 0 | 1 |
| chr6 | 50229209 | A | C | 164 | Dfna5 | intronic | . | . | . | 6qB2.3 | rs29689957 | 0 | 1 |
| chr6 | 50229322 | A | G | 221 | Dfna5 | exonic | synonymous SNV | Dfna5:NM_018769:exon4:c.T540C:p.C180C | . | 6qB2.3 | rs29689959 | 0 | 1 |
| chr6 | 50229495 | T | C | 222 | Dfna5 | intronic | . | . | . | 6qB2.3 | rs30598167 | 0 | 1 |
| chr6 | 50229497 | G | A | 222 | Dfna5 | intronic | . | . | . | 6qB2.3 | rs253054937 | 0 | 1 |
| chr6 | 50229682 | A | G | 64 | Dfna5 | intronic | . | . | . | 6qB2.3 | rs29689961 | 0 | 1 |
| chr6 | 50299286 | A | T | 190 | Osbpl3 | intronic | . | . | . | 6qB2.3 | rs51231546 | 0 | 1 |
| chr6 | 50299512 | G | A | 218 | Osbpl3 | intronic | . | . | . | 6qB2.3 | rs29698047 | 0 | 1 |
| chr6 | 50302949 | C | T | 220 | Osbpl3 | intronic | . | . | . | 6qB2.3 | rs30416015 | 0 | 1 |
| chr6 | 50302989 | A | C | 220 | Osbpl3 | intronic | . | . | . | 6qB2.3 | rs30901306 | 0 | 1 |
| chr6 | 50309090 | A | G | 123 | Osbpl3 | intronic | . | . | . | 6qB2.3 | rs29698078 | 0 | 1 |
| chr6 | 50312534 | A | G | 200 | Osbpl3 | intronic | . | . | . | 6qB2.3 | rs29699901 | 0 | 1 |
| chr6 | 50312563 | A | G | 221 | Osbpl3 | intronic | . | . | . | 6qB2.3 | rs29699903 | 0 | 1 |
| chr6 | 50312609 | A | G | 222 | Osbpl3 | intronic | . | . | . | 6qB2.3 | rs29700785 | 0 | 1 |
| chr6 | 50312794 | A | T | 221 | Osbpl3 | intronic | . | . | . | 6qB2.3 | rs29961464 | 0 | 1 |
| chr6 | 50312850 | C | T | 111 | Osbpl3 | intronic | . | . | . | 6qB2.3 | rs30618364 | 0 | 1 |
| chr6 | 50322865 | A | T | 106 | Osbpl3 | intronic | . | . | . | 6qB2.3 | rs263907961 | 0 | 1 |
| chr6 | 50322880 | T | C | 111 | Osbpl3 | intronic | . | . | . | 6qB2.3 | rs29696306 | 0 | 1 |
| chr6 | 50322999 | T | G | 222 | Osbpl3 | exonic | synonymous SNV | Osbpl3:NM_001163645:exon14:c.A1516C:p.R506R,Osbpl3:NM_027881:exon14:c.A1624C:p.R542R | . | 6qB2.3 | rs245684586 | 0 | 1 |
| chr6 | 50323219 | A | G | 221 | Osbpl3 | exonic | synonymous SNV | Osbpl3:NM_001163645:exon14:c.T1296C:p.P432P,Osbpl3:NM_027881:exon14:c.T1404C:p.P468P | . | 6qB2.3 | rs29696309 | 0 | 1 |
| chr6 | 50327544 | C | T | 113 | Osbpl3 | intronic | . | . | . | 6qB2.3 | rs29702849 | 0 | 1 |
| chr6 | 50328961 | T | C | 91 | Osbpl3 | intronic | . | . | . | 6qB2.3 | rs29696286 | 0 | 1 |
| chr6 | 50332997 | T | C | 121 | Osbpl3 | intronic | . | . | . | 6qB2.3 | rs29702750 | 0 | 1 |
| chr6 | 50336453 | T | C | 165 | Osbpl3 | intronic | . | . | . | 6qB2.3 | rs29698944 | 0 | 1 |
| chr6 | 50344920 | C | T | 221 | Osbpl3 | exonic | synonymous SNV | Osbpl3:NM_027881:exon9:c.G885A:p.S295S,Osbpl3:NM_001163645:exon10:c.G885A:p.S295S | . | 6qB2.3 | rs29700320 | 0 | 1 |
| chr6 | 50345963 | C | T | 220 | Osbpl3 | intronic | . | . | . | 6qB2.3 | rs29702872 | 0 | 1 |
| chr6 | 50346429 | T | G | 221 | Osbpl3 | exonic | missense SNV | Osbpl3:NM_027881:exon8:c.A673C:p.M225L,Osbpl3:NM_001163645:exon9:c.A673C:p.M225L | . | 6qB2.3 | rs29977063 | 0 | 1 |
| chr6 | 50347262 | T | C | 216 | Osbpl3 | intronic | . | . | . | 6qB2.3 | rs29698406 | 0 | 1 |
| chr6 | 50347554 | A | G | 221 | Osbpl3 | intronic | . | . | . | 6qB2.3 | rs30123207 | 0 | 1 |
| chr6 | 50347806 | C | T | 112 | Osbpl3 | intronic | . | . | . | 6qB2.3 | rs29699366 | 0 | 1 |
| chr6 | 50347878 | T | C | 136 | Osbpl3 | intronic | . | . | . | 6qB2.3 | rs29699368 | 0 | 1 |
| chr6 | 50348365 | T | C | 65 | Osbpl3 | intronic | . | . | . | 6qB2.3 | rs30173566 | 0 | 1 |
| chr6 | 50348368 | A | G | 65 | Osbpl3 | intronic | . | . | . | 6qB2.3 | rs30702837 | 0 | 1 |
| chr6 | 50351788 | T | C | 187 | Osbpl3 | intronic | . | . | . | 6qB2.3 | rs30812607 | 0 | 1 |

| | | | | | | | | | | | | | |
|---|---|---|---|---|---|---|---|---|---|---|---|---|---|
| chr6 | 50351913 | A | G | 221 | Osbpl3 | exonic | synonymous SNV | Osbpl3:NM_027881:exon5:c.T309C:p.S103S,Osbpl3:NM_001163645:exon6:c.T309C:p.S103S | . | 6qB2.3 | rs13460722 | 0 | 1 |
| chr6 | 50351951 | G | T | 221 | Osbpl3 | exonic | synonymous SNV | Osbpl3:NM_027881:exon5:c.C271A:p.R91R,Osbpl3:NM_001163645:exon6:c.C271A:p.R91R | . | 6qB2.3 | rs29696065 | 0 | 1 |
| chr6 | 50352015 | T | G | 102 | Osbpl3 | intronic | . | . | . | 6qB2.3 | rs30906489 | 0 | 1 |
| chr6 | 50352634 | G | A | 130 | Osbpl3 | intronic | . | . | . | 6qB2.3 | rs30464345 | 0 | 1 |
| chr6 | 50353136 | A | C | 222 | Osbpl3 | intronic | . | . | . | 6qB2.3 | rs29699023 | 0 | 1 |
| chr6 | 50565738 | T | C | 168 | Cycs | intronic | . | . | . | 6qB2.3 | rs29708962 | 0 | 1 |
| chr6 | 50566392 | T | C | 117 | Cycs | intronic | . | . | 621 | 6qB2.3 | rs30620564 | 0 | 1 |
| chr6 | 50574599 | C | T | 221 | 4921507P07Rik,543 | ncRNA_intronic | . | . | . | 6qB2.3 | rs29707014 | 0 | 1 |
| chr6 | 50577255 | A | G | 123 | 4921507P07Rik,543 | ncRNA_intronic | . | . | . | 6qB2.3 | rs29707923 | 0 | 1 |
| chr6 | 50587178 | T | C | 222 | 5430402013Rik,492 | exonic | missense SNV | 4921507P07Rik:NM_027564:exon5:c.A413G:p.K138R | . | 6qB2.3 | rs29871380 | 0 | 1 |
| chr6 | 50587390 | G | A | 128 | 4921507P07Rik,543 | ncRNA_intronic | . | . | . | 6qB2.3 | rs30766430 | 0 | 1 |
| chr6 | 50588879 | T | C | 57 | 5430402013Rik,492 | ncRNA_intronic | . | . | . | 6qB2.3 | rs6340637 | 0 | 1 |
| chr6 | 50600045 | T | C | 53 | . | intergenic | . | . | . | 6qB2.3 | rs30120954 | 0 | 1 |
| chr6 | 50650918 | C | G | 222 | Npvf | exonic | missense SNV | Npvf:NM_021892:exon3:c.G520C:p.G174R | . | 6qB2.3 | rs29712909 | 0 | 1 |
| chr6 | 50652596 | T | C | 222 | Npvf | exonic | missense SNV | Npvf:NM_021892:exon2:c.A413G:p.D138G | . | 6qB2.3 | rs29707110 | 0 | 1 |
| chr6 | 50746780 | T | C | 27.997 | . | intergenic | . | . | . | 6qB2.3 | rs231212688 | 0 | 1 |
| chr6 | 50746879 | T | C | 33.9765 | . | intergenic | . | . | . | 6qB2.3 | rs265803525 | 0 | 1 |
| chr6 | 50747025 | G | A | 116 | . | intergenic | . | . | . | 6qB2.3 | rs233484178 | 0 | 1 |
| chr6 | 50747080 | G | A | 32.9629 | . | intergenic | . | . | . | 6qB2.3 | rs583396162 | 0 | 1 |
| chr6 | 50846952 | T | C | 222 | . | intergenic | . | . | . | 6qB2.3 | rs3147726 | 0 | 1 |
| chr6 | 50846997 | C | T | 222 | . | intergenic | . | . | . | 6qB2.3 | rs3142368 | 0 | 1 |
| chr6 | 50846998 | C | T | 222 | . | intergenic | . | . | . | 6qB2.3 | rs3142367 | 0 | 1 |
| chr6 | 50847090 | T | C | 222 | . | intergenic | . | . | . | 6qB2.3 | rs3147725 | 0 | 1 |
| chr6 | 50847501 | A | G | 94 | . | intergenic | . | . | . | 6qB2.3 | rs3142366 | 0 | 1 |
| chr6 | 50847541 | G | A | 131 | . | intergenic | . | . | . | 6qB2.3 | rs3147722 | 0 | 1 |
| chr6 | 50847655 | T | C | 222 | . | intergenic | . | . | . | 6qB2.3 | rs3142365 | 0 | 1 |
| chr6 | 50847741 | G | T | 222 | . | intergenic | . | . | . | 6qB2.3 | rs29752845 | 0 | 1 |
| chr6 | 50847944 | A | G | 51 | . | intergenic | . | . | . | 6qB2.3 | rs29752849 | 0 | 1 |
| chr6 | 50848010 | G | A | 110 | . | intergenic | . | . | . | 6qB2.3 | rs51908022 | 0 | 1 |
| chr6 | 50848063 | A | G | 60 | . | intergenic | . | . | . | 6qB2.3 | rs29752851 | 0 | 1 |
| chr6 | 50967461 | T | C | 221 | . | intergenic | . | . | . | 6qB3 | rs29754987 | 0 | 1 |
| chr6 | 50967564 | A | G | 220 | . | intergenic | . | . | . | 6qB3 | rs29754990 | 0 | 1 |
| chr6 | 50967685 | A | G | 109 | . | intergenic | . | . | . | 6qB3 | rs29754992 | 0 | 1 |
| chr6 | 51270081 | A | G | 121 | . | upstream | . | . | . | 6qB3 | rs29788378 | 0 | 1 |
| chr6 | 51285279 | T | C | 126 | . | intergenic | . | . | . | 6qB3 | rs29797409 | 0 | 1 |
| chr6 | 51285519 | A | G | 64 | . | intergenic | . | . | . | 6qB3 | . | 0 | 1 |
| chr6 | 51285661 | C | T | 222 | . | intergenic | . | . | . | 6qB3 | rs228783278 | 0 | 1 |
| chr6 | 51285666 | T | G | 222 | . | intergenic | . | . | . | 6qB3 | rs249031209 | 0 | 1 |
| chr6 | 51457907 | G | A | 222 | Nfe2l3 | exonic | synonymous SNV | Nfe2l3:NM_010903:exon4:c.G1446A:p.E482E | . | 6qB3 | rs252308770 | 0 | 1 |
| chr6 | 51466015 | C | T | 138 | Hnrnpa2b1 | intronic | . | . | . | 6qB3 | rs226722250 | 0 | 1 |
| chr6 | 51466888 | T | C | 222 | Hnrnpa2b1 | intronic | . | . | . | 6qB3 | rs47522479 | 0 | 1 |
| chr6 | 51467084 | C | T | 222 | Hnrnpa2b1 | intronic | . | . | . | 6qB3 | rs51031918 | 0 | 1 |
| chr6 | 51469567 | G | A | 221 | Hnrnpa2b1 | intronic | . | . | 2398 | 6qB3 | rs239268432 | 0 | 1 |
| chr6 | 51469765 | G | A | 221 | Hnrnpa2b1 | UTR5 | . | . | 2398 | 6qB3 | rs228820180 | 0 | 1 |
| chr6 | 51478323 | G | A | 148 | Cbx3 | intronic | . | . | . | 6qB3 | rs51664102 | 0 | 1 |
| chr6 | 51481827 | G | A | 222 | Cbx3 | intronic | . | . | . | 6qB3 | rs52411308 | 0 | 1 |
| chr6 | 51576002 | C | A | 188 | Snx10 | intronic | . | . | . | 6qB3 | . | 0 | 1 |
| chr6 | 52170544 | C | T | 214 | Hoxa3 | exonic | missense SNV | Hoxa3:NM_010452:exon5:c.G728A:p.R243H | . | 6qB3 | . | 0 | 1 |
| chr6 | 52179162 | A | C | 221 | Hoxa3 | intronic | . | . | . | 6qB3 | . | 0 | 1 |
| chr6 | 52765109 | T | G | 91 | Tax1bp1 | intronic | . | . | . | 6qB3 | . | 0 | 1 |
| chr6 | 53692904 | A | G | 182 | Creb5 | exonic | synonymous SNV | Creb5:NM_172728:exon6:c.A807G:p.E269E | . | 6qB3 | . | 0 | 1 |
| chr6 | 53818435 | G | A | 207 | Tril | exonic | missense SNV | Tril:NM_025817:exon1:c.C1801T:p.R601C | 3234 | 6qB3 | . | 0 | 1 |
| chr6 | 54586895 | T | C | 35.4537 | Fkbp14 | intronic | . | . | . | 6qB3 | . | 0 | 1 |
| chr6 | 55075338 | G | T | 22.0169 | Gars | intronic | . | . | . | 6qB3 | . | 0 | 1 |
| chr6 | 55872579 | C | T | 119 | Ccdc129 | exonic | missense SNV | Ccdc129:NM_001081665:exon2:c.C95T:p.A32V | . | 6qB3 | . | 0 | 1 |
| chr6 | 55874085 | A | C | 66 | Ccdc129 | intronic | . | . | . | 6qB3 | . | 0 | 1 |
| chr6 | 55968089 | T | G | 112 | Ccdc129 | exonic | synonymous SNV | Ccdc129:NM_001081665:exon11:c.T1794G:p.P598P | . | 6qB3 | . | 0 | 1 |
| chr6 | 56159081 | G | T | 114 | Pde1c | intronic | . | . | . | 6qB3 | . | 0 | 1 |
| chr6 | 56173651 | A | G | 177 | Pde1c | exonic | synonymous SNV | Pde1c:NM_001159952:exon6:c.T675C:p.A225A,Pde1c:NM_001159956:exon6:c.T591C:p.A197A,Pde1c:NM_0 | . | 6qB3 | . | 0 | 1 |
| chr6 | 56868836 | A | G | 167 | Fkbp9 | exonic | missense SNV | Fkbp9:NM_012056:exon7:c.A1156G:p.S386G | . | 6qB3 | . | 0 | 1 |
| chr6 | 56956706 | T | G | 222 | Vmn1r4 | exonic | missense SNV | Vmn1r4:NM_134176:exon1:c.T194G:p.L65R | . | 6qB3 | . | 0 | 1 |
| chr6 | 57002911 | T | G | 151 | Vmn1r6 | exonic | missense SNV | Vmn1r6:NM_134175:exon1:c.T557G:p.F186C | . | 6qB3 | . | 0 | 1 |
| chr6 | 57389893 | C | A | 222 | Vmn1r18 | exonic | missense SNV | Vmn1r18:NM_134181:exon1:c.G675T:p.R225S | . | 6qB3 | . | 0 | 1 |
| chr6 | 57447472 | G | T | 74 | . | intergenic | . | . | . | 6qB3 | . | 0 | 1 |
| chr6 | 57774727 | T | G | 102 | Vopp1 | exonic | synonymous SNV | Vopp1:NM_146168:exon3:c.A175C:p.R59R | . | 6qB3 | . | 0 | 1 |
| chr6 | 58077991 | A | C | 157 | . | intergenic | . | . | . | 6qB3 | . | 0 | 1 |
| chr6 | 58655764 | G | T | 111 | Abcg2 | exonic | synonymous SNV | Abcg2:NM_011920:exon2:c.G108T:p.V36V | . | 6qB3 | . | 0 | 1 |
| chr6 | 58876998 | A | C | 56 | Herc3 | intronic | . | . | . | 6qB3 | . | 0 | 1 |
| chr6 | 58906944 | T | C | 78 | Nap1l5,Herc3 | exonic | synonymous SNV | Nap1l5:NM_021432:exon1:c.A24G:p.G8G | 424 | 6qB3 | . | 0 | 1 |
| chr6 | 61310940 | T | C | 71 | Ccser1 | exonic | missense SNV | Ccser1:NM_001164316:exon2:c.T86C:p.L29P,Ccser1:NM_183310:exon2:c.T86C:p.L29P | . | 6qB3 | . | 0 | 1 |
| chr6 | 61311565 | C | T | 169 | Ccser1 | exonic | synonymous SNV | Ccser1:NM_001164316:exon2:c.C711T:p.R237R,Ccser1:NM_183310:exon2:c.C711T:p.R237R | . | 6qB3 | rs245614149 | 0 | 1 |
| chr6 | 61312146 | A | C | 56 | Ccser1 | exonic | missense SNV | Ccser1:NM_001164316:exon2:c.A1292C:p.Y431S,Ccser1:NM_183310:exon2:c.A1292C:p.Y431S | . | 6qB3 | . | 0 | 1 |
| chr6 | 61422954 | C | T | 67 | Ccser1 | splicing | . | . | . | 6qB3 | . | 0 | 1 |
| chr6 | 61422955 | A | G | 76 | Ccser1 | splicing | . | . | . | 6qB3 | . | 0 | 1 |
| chr6 | 62380153 | T | C | 175 | Ccser1 | exonic | synonymous SNV | Ccser1:NM_001164316:exon11:c.T2574C:p.F858F,Ccser1:NM_183310:exon11:c.T2574C:p.F858F | . | 6qB3 | . | 0 | 1 |
| chr6 | 63098576 | C | A | 96 | . | intergenic | . | . | . | 6qC1 | . | 0 | 1 |
| chr6 | 64730932 | G | T | 87 | Atoh1 | UTR3 | . | . | . | 6qC1 | . | 0 | 1 |
| chr6 | 65108478 | T | A | 27.8856 | Smarcad1 | intronic | . | . | . | 6qC1 | . | 0 | 1 |
| chr6 | 68909462 | G | A | 59 | . | intergenic | . | . | . | 6qC1 | . | 0 | 1 |
| chr6 | 69362844 | T | A | 72 | . | intergenic | . | . | . | 6qC1 | . | 0 | 1 |
| chr6 | 70917751 | A | G | 222 | Tex37 | intronic | . | . | . | 6qC1 | . | 0 | 1 |
| chr6 | 70956728 | C | T | 91 | Foxi3 | exonic | synonymous SNV | Foxi3:NM_001101464:exon1:c.C120T:p.A40A | 1102 | 6qC1 | . | 0 | 1 |
| chr6 | 71134415 | A | C | 228 | Thnsl2 | intronic | . | . | . | 6qC1 | rs49593081 | 1 | 0 |
| chr6 | 71134423 | T | C | 228 | Thnsl2 | intronic | . | . | . | 6qC1 | rs36385312 | 1 | 0 |
| chr6 | 71134510 | C | T | 134 | Thnsl2 | intronic | . | . | . | 6qC1 | rs39439158 | 1 | 0 |
| chr6 | 71672177 | G | T | 222 | . | intergenic | . | . | . | 6qC1 | . | 0 | 1 |
| chr6 | 71951087 | A | T | 169 | Polr1a | intronic | . | . | . | 6qC1 | rs51867136 | 1 | 0 |
| chr6 | 71972489 | A | T | 102 | Polr1a | intronic | . | . | . | 6qC1 | rs30717920 | 0 | 1 |
| chr6 | 72305981 | G | C | 228 | Sftpb | intronic | . | . | . | 6qC1 | rs52137143 | 1 | 0 |
| chr6 | 72337926 | C | G | 198 | Usp39 | intronic | . | . | . | 6qC1 | . | 0 | 1 |
| chr6 | 73017542 | A | G | 168 | . | downstream | . | . | . | 6qC1 | rs51571671 | 1 | 0 |
| chr6 | 73095035 | G | A | 53 | Dnah6 | exonic | missense SNV | Dnah6:NM_001164669:exon46:c.C7463T:p.A2488V | . | 6qC1 | . | 0 | 1 |
| chr6 | 75297521 | G | A | 140 | . | intergenic | . | . | . | 6qC2 | . | 0 | 1 |
| chr6 | 77143935 | C | A | 221 | Ctnna2 | exonic | missense SNV | Ctnna2:NM_009819:exon8:c.G1065T:p.R355S,Ctnna2:NM_001109764:exon9:c.G1065T:p.R355S,Ctnna2:NM_ | . | 6qC3 | . | 0 | 1 |
| chr6 | 78216403 | T | G | 142 | . | intergenic | . | . | . | 6qC3 | . | 0 | 1 |
| chr6 | 82554803 | C | T | 120 | Tacr1 | intronic | . | . | . | 6qC3 | . | 0 | 1 |
| chr6 | 83048857 | T | C | 221 | Loxl3 | intronic | . | . | . | 6qC3 | . | 0 | 1 |
| chr6 | 83069484 | G | T | 27.0957 | Tlx2 | intronic | . | . | 2785 | 6qC3 | . | 0 | 1 |
| chr6 | 83153684 | C | G | 228 | Wdr54 | intronic | . | . | . | 6qC3 | rs51091930 | 1 | 0 |
| chr6 | 83270788 | C | A | 61 | Slc4a5 | intronic | . | . | . | 6qC3 | . | 0 | 1 |
| chr6 | 83376779 | C | A | 201 | Tet3 | exonic | missense SNV | Tet3:NM_183138:exon6:c.G2494T:p.G832C | . | 6qC3 | . | 0 | 1 |
| chr6 | 83519793 | C | G | 222 | Actg2 | exonic | missense SNV | Actg2:NM_009610:exon7:c.G780C:p.E260D | . | 6qC3 | . | 0 | 1 |
| chr6 | 85333371 | G | A | 97 | Sfxn5 | exonic | synonymous SNV | Sfxn5:NM_178639:exon1:c.C45T:p.A15A | 969 | 6qC3 | . | 0 | 1 |
| chr6 | 85621251 | C | A | 222 | Alms1 | exonic | missense SNV | Alms1:NM_145223:exon8:c.C3058A:p.L1020I | . | 6qC3 | . | 0 | 1 |
| chr6 | 87385496 | C | T | 222 | Gkn3 | exonic | missense SNV | Gkn3:NM_026860:exon4:c.G310A:p.D104N | . | 6qD1 | . | 0 | 1 |
| chr6 | 89337390 | G | C | 222 | Plxna1 | intronic | . | . | . | 6qD1 | . | 0 | 1 |
| chr6 | 90369600 | G | T | 122 | Zxdc | UTR5 | . | . | 1239 | 6qD1 | . | 0 | 1 |
| chr6 | 91725056 | G | C | 199 | Slc6a6 | intronic | . | . | . | 6qD1 | . | 0 | 1 |
| chr6 | 91782800 | C | A | 222 | Grip2 | exonic | missense SNV | Grip2:NM_001159507:exon10:c.G1151T:p.G384V | . | 6qD1 | . | 0 | 1 |
| chr6 | 92211514 | A | C | 141 | Rbsn | intronic | . | . | . | 6qD1 | . | 0 | 1 |
| chr6 | 92376807 | T | G | 222 | Prickle2 | exonic | missense SNV | Prickle2:NM_001134459:exon8:c.A1677C:p.E559D,Prickle2:NM_001134460:exon8:c.A1677C:p.E559D,Pri | . | 6qD1 | . | 0 | 1 |
| chr6 | 92880653 | C | A | 222 | Adamts9 | exonic | missense SNV | Adamts9:NM_175314:exon8:c.G910T:p.G304W | . | 6qD1 | . | 0 | 1 |
| chr6 | 92912827 | T | A | 222 | . | intergenic | . | . | . | 6qD1 | . | 0 | 1 |
| chr6 | 95117832 | G | T | 60 | . | upstream | . | . | 848 | 6qD2 | . | 0 | 1 |
| chr6 | 96032851 | T | G | 93 | . | intergenic | . | . | . | 6qD3 | . | 0 | 1 |
| chr6 | 96649300 | C | T | 121 | Fam19a1 | intronic | . | . | . | 6qD3 | . | 0 | 1 |
| chr6 | 97191592 | T | C | 222 | Uba3 | splicing | . | . | . | 6qD3 | . | 0 | 1 |
| chr6 | 100665345 | T | A | 222 | Shq1 | exonic | stopgain | Shq1:NM_181590:exon3:c.A292T:p.R98X | . | 6qD3 | . | 0 | 1 |
| chr6 | 101150767 | C | A | 148 | Pdzrn3 | exonic | synonymous SNV | Pdzrn3:NM_018884:exon10:c.G2937T:p.L979L | . | 6qD3 | . | 0 | 1 |
| chr6 | 101354300 | C | A | 61 | Pdzrn3 | intronic | . | . | . | 6qD3 | . | 0 | 1 |
| chr6 | 102278298 | G | C | 221 | Cntn3,4930587E11 | exonic | missense SNV | Cntn3:NM_008779:exon5:c.C638G:p.P213R | . | 6qD3 | . | 0 | 1 |
| chr6 | 103700762 | G | T | 70 | Chl1 | intronic | . | . | . | 6qE1 | . | 0 | 1 |
| chr6 | 104568908 | G | T | 145 | Cntn6 | intronic | . | . | . | 6qE1 | . | 0 | 1 |
| chr6 | 106674407 | T | C | 58 | Cntn4 | intronic | . | . | . | 6qE1 | . | 0 | 1 |
| chr6 | 106731730 | G | T | 190 | Il5ra | intronic | . | . | . | 6qE1 | . | 0 | 1 |
| chr6 | 106742884 | G | C | 115 | Il5ra | intronic | . | . | . | 6qE1 | . | 0 | 1 |
| chr6 | 107567433 | C | A | 222 | Lrrn1 | exonic | missense SNV | Lrrn1:NM_008516:exon2:c.C191A:p.T64N | . | 6qE1 | . | 0 | 1 |
| chr6 | 110914378 | C | T | 221 | Grm7 | exonic | missense SNV | Grm7:NM_177328:exon2:c.C571T:p.R191C | . | 6qE3 | . | 0 | 1 |
| chr6 | 111253946 | G | A | 199 | Grm7 | intronic | . | . | . | 6qE3 | . | 0 | 1 |
| chr6 | 112287928 | C | T | 222 | Lmcd1,5031434C07 | ncRNA_exonic | . | . | . | 6qE3 | rs48059424 | 0 | 1 |
| chr6 | 112310861 | C | T | 108 | Lmcd1 | intronic | . | . | . | 6qE3 | . | 0 | 1 |
| chr6 | 112489121 | T | C | 222 | Oxtr | exonic | missense SNV | Oxtr:NM_001081147:exon1:c.A677G:p.K226R | 859 | 6qE3 | . | 0 | 1 |
| chr6 | 112750567 | C | A | 125 | Srgap3 | intronic | . | . | . | 6qE3 | . | 0 | 1 |
| chr6 | 113269924 | A | G | 138 | Mtmr14 | intronic | . | . | . | 6qE3 | . | 0 | 1 |
| chr6 | 113372540 | C | G | 164 | Tada3 | exonic | missense SNV | Tada3:NM_133932:exon5:c.G631C:p.D211H | . | 6qE3 | . | 0 | 1 |
| chr6 | 113495536 | A | T | 222 | Prrt3 | exonic | synonymous SNV | Prrt3:NM_001289699:exon4:c.T1674A:p.P558P,Prrt3:NM_001289700:exon4:c.T1674A:p.P558P,Prrt3:NM_ | . | 6qE3 | . | 0 | 1 |
| chr6 | 113595789 | A | C | 172 | Fancd2 | intronic | . | . | . | 6qE3 | . | 0 | 1 |
| chr6 | 113647703 | G | T | 222 | Irak2 | exonic | synonymous SNV | Irak2:NM_001113553:exon2:c.G159T:p.G53G,Irak2:NM_172161:exon2:c.G159T:p.G53G | . | 6qE3 | . | 0 | 1 |
| chr6 | 115250043 | T | C | 104 | Timp4,Syn2 | intronic | . | . | . | 6qE3 | . | 0 | 1 |
| chr6 | 115792895 | G | A | 119 | Cand2 | exonic | missense SNV | Cand2:NM_025958:exon10:c.G2665A:p.A889T | . | 6qE3 | . | 0 | 1 |
| chr6 | 115935494 | G | C | 145 | Rho | exonic | missense SNV | Rho:NM_145383:exon4:c.G795C:p.W265C | . | 6qE3 | . | 0 | 1 |
| chr6 | 119300037 | G | A | 81 | Cacna2d4 | intronic | . | . | . | 6qF1 | . | 0 | 1 |
| chr6 | 119950433 | T | A | 222 | Wnk1 | intronic | . | . | . | 6qF1 | . | 0 | 1 |
| chr6 | 119992410 | A | G | 222 | Wnk1 | exonic | synonymous SNV | Wnk1:NM_001185020:exon3:c.T1083C:p.T361T,Wnk1:NM_001185021:exon3:c.T1083C:p.T361T,Wnk1:NM_00 | . | 6qF1 | . | 0 | 1 |
| chr6 | 121354207 | T | A | 222 | Slc6a12 | intronic | . | . | . | 6qF1 | . | 0 | 1 |
| chr6 | 121413161 | C | T | 151 | Iqsec3 | exonic | synonymous SNV | Iqsec3:NM_001289685:exon3:c.G450A:p.A150A,Iqsec3:NM_001033354:exon4:c.G1350A:p.A450A | 777 | 6qF1 | . | 0 | 1 |
| chr6 | 122695959 | A | T | 118 | . | intergenic | . | . | . | 6qF1 | . | 0 | 1 |
| chr6 | 122828604 | G | T | 133 | Foxj2 | intronic | . | . | . | 6qF2 | . | 0 | 1 |
| chr6 | 122850948 | A | G | 222 | C3ar1 | exonic | synonymous SNV | C3ar1:NM_009779:exon2:c.T309C:p.I103I | . | 6qF2 | . | 0 | 1 |
| chr6 | 123204072 | T | G | 193 | Clec4b2 | intronic | . | . | . | 6qF2 | . | 0 | 1 |
| chr6 | 123331364 | A | C | 89 | Vmn2r19 | intronic | . | . | . | 6qF2 | . | 0 | 1 |
| chr6 | 124139502 | A | G | 77 | . | intergenic | . | . | . | 6qF2 | . | 0 | 1 |
| chr6 | 124145802 | A | T | 69 | . | intergenic | . | . | . | 6qF2 | . | 0 | 1 |
| chr6 | 124311694 | T | C | 228 | Cd163 | exonic | synonymous SNV | Cd163:NM_001170395:exon5:c.T1083C:p.G361G,Cd163:NM_053094:exon5:c.T1083C:p.G361G | . | 6qF2 | rs30514715 | 1 | 0 |
| chr6 | 124740795 | C | G | 217 | Grcc10 | intronic | . | . | 516 | 6qF2 | . | 0 | 1 |
| chr6 | 124861308 | G | C | 222 | Gpr162 | exonic | synonymous SNV | Gpr162:NM_013533:exon2:c.C378G:p.R126R | 213 | 6qF2 | . | 0 | 1 |
| chr6 | 125110244 | A | G | 222 | Chd4 | intronic | . | . | . | 6qF2 | rs30031775 | 0 | 1 |
| chr6 | 125128640 | A | C | 40.901 | Chd4 | intronic | . | . | . | 6qF2 | . | 0 | 1 |
| chr6 | 125151367 | C | T | 222 | Iffo1 | intronic | . | . | . | 6qF2 | . | 0 | 1 |
| chr6 | 125710539 | T | A | 87 | Ano2 | intronic | . | . | . | 6qF3 | . | 0 | 1 |
| chr6 | 126642807 | G | C | 221 | Kcna1 | exonic | missense SNV | Kcna1:NM_010595:exon2:c.C549G:p.I183M | . | 6qF3 | . | 0 | 1 |
| chr6 | 126643048 | C | T | 62 | Kcna1 | exonic | missense SNV | Kcna1:NM_010595:exon2:c.G308A:p.R103H | 366 | 6qF3 | . | 0 | 1 |
| chr6 | 126877113 | C | G | 144 | Dyrk4 | intronic | . | . | . | 6qF3 | . | 0 | 1 |
| chr6 | 127657924 | G | A | 136 | . | intergenic | . | . | . | 6qF3 | . | 0 | 1 |
| chr6 | 127732732 | C | A | 222 | Prmt8 | exonic | missense SNV | Prmt8:NM_201371:exon2:c.G164T:p.C55F | . | 6qF3 | . | 0 | 1 |
| chr6 | 127943568 | C | A | 136 | Tspan11 | intronic | . | . | . | 6qF3 | . | 0 | 1 |
| chr6 | 128375553 | A | G | 221 | Foxm1,4933413G19 | exonic | missense SNV | 4933413G19Rik:NM_027697:exon1:c.A35G:p.K12R | . | 6qF3 | . | 0 | 1 |
| chr6 | 128432753 | G | A | 70 | Fkbp4 | intronic | . | . | . | 6qF3 | . | 0 | 1 |
| chr6 | 128780137 | A | G | 91 | Klrb1c | UTR3 | . | . | . | 6qF3 | . | 0 | 1 |
| chr6 | 128819058 | G | T | 222 | Klrb1b | exonic | missense SNV | Klrb1b:NM_030599:exon4:c.C269A:p.A90D | . | 6qF3 | . | 0 | 1 |
| chr6 | 129053232 | T | C | 222 | Klrb1f | exonic | synonymous SNV | Klrb1f:NM_153094:exon3:c.T234C:p.A78A | . | 6qF3 | . | 0 | 1 |
| chr6 | 129353333 | T | C | 190 | Clec12a | intronic | . | . | . | 6qF3 | . | 0 | 1 |
| chr6 | 129488732 | C | A | 155 | Olr1 | intronic | . | . | . | 6qF3 | . | 0 | 1 |
| chr6 | 129911557 | A | G | 102 | Klra5 | intronic | . | . | . | 6qF3 | . | 0 | 1 |
| chr6 | 130462216 | T | G | 156 | . | intergenic | . | . | . | 6qF3 | . | 0 | 1 |
| chr6 | 131677927 | C | T | 77 | . | downstream | . | . | . | 6qF3 | . | 0 | 1 |
| chr6 | 132657327 | T | C | 163 | Tas2r120 | exonic | missense SNV | Tas2r120:NM_207023:exon1:c.T371C:p.L124P | . | 6qG1 | . | 0 | 1 |
| chr6 | 132868262 | A | C | 161 | Tas2r110 | exonic | missense SNV | Tas2r110:NM_199155:exon1:c.A255C:p.E85D | . | 6qG1 | . | 0 | 1 |
| chr6 | 132893595 | G | A | 161 | Tas2r113 | exonic | synonymous SNV | Tas2r113:NM_207018:exon1:c.G585A:p.T195T | . | 6qG1 | . | 0 | 1 |
| chr6 | 135016675 | G | T | 222 | Ddx47 | intronic | . | . | . | 6qG1 | . | 0 | 1 |
| chr6 | 135107889 | G | C | 123 | Gprc5d | intronic | . | . | . | 6qG1 | . | 0 | 1 |
| chr6 | 135310110 | C | G | 222 | Pbp2 | exonic | missense SNV | Pbp2:NM_029595:exon1:c.G238C:p.V80L | . | 6qG1 | . | 0 | 1 |
| chr6 | 136401960 | C | G | 221 | E330021D16Rik | intronic | . | . | . | 6qG1 | . | 0 | 1 |
| chr6 | 136401962 | C | A | 221 | E330021D16Rik | intronic | . | . | . | 6qG1 | . | 0 | 1 |
| chr6 | 136922136 | T | C | 222 | Erp27 | UTR5 | . | . | . | 6qG1 | . | 0 | 1 |

| | | | | | | | | | | | | | |
|---|---|---|---|---|---|---|---|---|---|---|---|---|---|
| chr6 | 137386514 | G | T | 20.3806 | Ptpro | intronic | . | . | . | 6qG1 | . | 0 | 1 |
| chr6 | 138153488 | T | G | 221 | Mgst1 | intronic | . | . | . | 6qG1 | . | 0 | 1 |
| chr6 | 138416416 | T | A | 142 | Lmo3 | exonic | missense SNV | Lmo3:NM_207222:exon3:c.A205T:p.R69W | . | 6qG1 | . | 0 | 1 |
| chr6 | 139754710 | T | A | 175 | Pik3c2g | intronic | . | . | . | 6qG2 | . | 0 | 1 |
| chr6 | 140572784 | T | G | 49.3218 | Plekha5 | intronic | . | . | . | 6qG2 | . | 0 | 1 |
| chr6 | 140633939 | G | T | 228 | Aebp2 | intronic | . | . | . | 6qG2 | rs30320388 | 1 | 0 |
| chr6 | 141459668 | T | C | 176 | Pde3a | intronic | . | . | . | 6qG2 | . | 0 | 1 |
| chr6 | 141466364 | G | T | 105 | Pde3a | intronic | . | . | . | 6qG2 | . | 0 | 1 |
| chr6 | 141484031 | G | A | 81 | Pde3a | intronic | . | . | . | 6qG2 | . | 0 | 1 |
| chr6 | 141484032 | C | A | 81 | Pde3a | intronic | . | . | . | 6qG2 | . | 0 | 1 |
| chr6 | 141648804 | T | A | 33.7532 | Slco1b2 | intronic | . | . | . | 6qG2 | . | 0 | 1 |
| chr6 | 142367343 | C | A | 221 | Recql | exonic | missense SNV | Recql:NM_001204906:exon10:c.G1147T:p.V383L,Recql:NM_001204907:exon10:c.G1147T:p.V383L,Recql:N | . | 6qG2 | . | 0 | 1 |
| chr6 | 142392254 | G | A | 199 | Golt1b | intronic | . | . | . | 6qG2 | . | 0 | 1 |
| chr6 | 142414199 | A | T | 133 | Spx | intronic | . | . | . | 6qG2 | . | 0 | 1 |
| chr6 | 142472698 | C | G | 222 | Gys2 | exonic | missense SNV | Gys2:NM_145572:exon1:c.G104C:p.W35S | . | 6qG2 | . | 0 | 1 |
| chr6 | 142599105 | G | C | 222 | Abcc9 | exonic | missense SNV | Abcc9:NM_001044720:exon36:c.C4216G:p.L1406V,Abcc9:NM_001310143:exon36:c.C4216G:p.L1406V,Abcc9 | . | 6qG2 | . | 0 | 1 |
| chr6 | 142599196 | A | T | 222 | Abcc9 | intronic | . | . | . | 6qG2 | . | 0 | 1 |
| chr6 | 142876567 | A | C | 197 | St8sia1 | intronic | . | . | . | 6qG2 | rs36515889 | 0 | 1 |
| chr6 | 143012239 | G | A | 123 | C2cd5 | intronic | . | . | . | 6qG3 | rs36644491 | 0 | 1 |
| chr6 | 145160281 | A | T | 145 | Lrmp | intronic | . | . | . | 6qG3 | . | 0 | 1 |
| chr6 | 145186072 | T | C | 67 | Casc1 | intronic | . | . | . | 6qG3 | . | 0 | 1 |
| chr6 | 145246710 | A | G | 228 | Kras | exonic | synonymous SNV | Kras:NM_021284:exon2:c.T96C:p.Y32Y | . | 6qG3 | rs30119689 | 1 | 0 |
| chr6 | 145246772 | C | A | 221 | Kras | exonic | missense SNV | Kras:NM_021284:exon2:c.G34T:p.G12C | . | 6qG3 | . | 0 | 1 |
| chr6 | 146328210 | G | A | 222 | Itpr2 | intronic | . | . | . | 6qG3 | . | 0 | 1 |
| chr6 | 146373181 | C | T | 221 | Itpr2 | exonic | synonymous SNV | Itpr2:NM_010586:exon19:c.G2403A:p.T801T,Itpr2:NM_019923:exon20:c.G2502A:p.T834T | . | 6qG3 | rs212187526 | 0 | 1 |
| chr6 | 146376117 | A | G | 222 | Itpr2 | exonic | missense SNV | Itpr2:NM_010586:exon16:c.T1853C:p.L618P,Itpr2:NM_019923:exon17:c.T1952C:p.L651P | . | 6qG3 | . | 0 | 1 |
| chr6 | 148138518 | T | A | 220 | Far2 | intronic | . | . | . | 6qG3 | . | 0 | 1 |
| chr6 | 148336027 | G | T | 93 | Tmtc1 | intronic | . | . | . | 6qG3 | . | 0 | 1 |
| chr6 | 148357986 | C | T | 93 | Tmtc1 | intronic | . | . | . | 6qG3 | rs248500017 | 1 | 0 |
| chr6 | 148894910 | C | A | 86 | Caprin2 | intronic | . | . | . | 6qG3 | . | 0 | 1 |
| chr6 | 148926219 | C | G | 219 | Fam60a | intronic | . | . | . | 6qG3 | . | 0 | 1 |
| chr7 | 3222537 | T | C | 228 | Nlrp12 | exonic | missense SNV | Nlrp12:NM_001033431:exon10:c.A3101G:p.K1034R | . | 7qA1 | rs33895134 | 1 | 0 |
| chr7 | 3489253 | C | A | 222 | Tarm1 | intronic | . | . | . | 7qA1 | . | 0 | 1 |
| chr7 | 3655922 | G | C | 222 | Cnot3 | exonic | missense SNV | Cnot3:NM_146176:exon11:c.G1246C:p.G416R | . | 7qA1 | . | 0 | 1 |
| chr7 | 4242292 | A | G | 198 | Lilra5 | UTR3 | . | . | . | 7qA1 | . | 0 | 1 |
| chr7 | 4632995 | C | A | 34.7585 | Ppp6r1 | intronic | . | . | . | 7qA1 | . | 0 | 1 |
| chr7 | 6202862 | C | A | 176 | Galp | exonic | missense SNV | Galp:NM_178028:exon2:c.C62A:p.P21Q | . | 7qA1 | . | 0 | 1 |
| chr7 | 6202863 | G | T | 160 | Galp | exonic | synonymous SNV | Galp:NM_178028:exon2:c.G63T:p.P21P | . | 7qA1 | . | 0 | 1 |
| chr7 | 6208526 | T | G | 63 | Galp | intronic | . | . | . | 7qA1 | . | 0 | 1 |
| chr7 | 6440851 | G | C | 196 | Olfr1344 | exonic | missense SNV | Olfr1344:NM_177061:exon1:c.G950C:p.R317P | . | 7qA1 | . | 0 | 1 |
| chr7 | 6708502 | A | G | 222 | Peg3 | exonic | synonymous SNV | Peg3:NM_008817:exon9:c.T3720C:p.S1240S | . | 7qA1 | . | 0 | 1 |
| chr7 | 7061956 | T | C | 202 | . | intergenic | . | . | . | 7qA1 | rs31275568 | 1 | 0 |
| chr7 | 7097408 | A | T | 124 | . | intergenic | . | . | . | 7qA1 | . | 0 | 1 |
| chr7 | 12086410 | G | T | 221 | . | intergenic | . | . | . | 7qA1 | . | 0 | 1 |
| chr7 | 12192989 | G | T | 120 | Vmn1r80 | exonic | stopgain | Vmn1r80:NM_134204:exon1:c.G25T:p.G9X | . | 7qA1 | . | 0 | 1 |
| chr7 | 12512768 | A | C | 222 | 2900092C05Rik | intronic | . | . | . | 7qA1 | . | 0 | 1 |
| chr7 | 13178853 | T | C | 222 | . | downstream | . | . | . | 7qA1 | . | 0 | 1 |
| chr7 | 13307591 | G | T | 222 | Lig1 | exonic | missense SNV | Lig1:NM_001083188:exon25:c.G2347T:p.G783W,Lig1:NM_001199310:exon25:c.G2347T:p.G783W,Lig1:NM_( | . | 7qA1 | . | 0 | 1 |
| chr7 | 13383105 | A | T | 222 | . | intergenic | . | . | . | 7qA1 | . | 0 | 1 |
| chr7 | 13561240 | T | G | 209 | Bsph2 | intronic | . | . | . | 7qA1 | . | 0 | 1 |
| chr7 | 14518082 | A | G | 119 | . | intergenic | . | . | . | 7qA1 | rs32468292 | 0 | 1 |
| chr7 | 16647097 | A | G | 79 | . | intergenic | . | . | . | 7qA2 | . | 0 | 1 |
| chr7 | 16693801 | T | G | 171 | . | intergenic | . | . | . | 7qA2 | . | 0 | 1 |
| chr7 | 16747223 | C | T | 148 | Ap2s1 | intronic | . | . | . | 7qA2 | . | 0 | 1 |
| chr7 | 16961659 | C | A | 101 | Pnmal1 | intronic | . | . | . | 7qA2 | . | 0 | 1 |
| chr7 | 17154802 | T | C | 228 | Ceacam3 | exonic | synonymous SNV | Ceacam3:NM_054059:exon3:c.T495C:p.S165S | . | 7qA2 | rs47063870 | 1 | 0 |
| chr7 | 18013467 | A | C | 222 | Ceacam13 | UTR3 | . | . | . | 7qA2 | . | 0 | 1 |
| chr7 | 18069662 | A | T | 89 | Ceacam12 | UTR3 | . | . | . | 7qA2 | . | 0 | 1 |
| chr7 | 18245686 | T | C | 222 | Mill1 | exonic | missense SNV | Mill1:NM_153749:exon1:c.T77C:p.L26P | . | 7qA3 | . | 0 | 1 |
| chr7 | 18245687 | G | T | 222 | Mill1 | exonic | synonymous SNV | Mill1:NM_153749:exon1:c.G78T:p.L26L | . | 7qA3 | . | 0 | 1 |
| chr7 | 18893797 | A | T | 109 | Ccdc61 | exonic | missense SNV | Ccdc61:NM_001033314:exon7:c.T809A:p.L270Q | . | 7qA3 | . | 0 | 1 |
| chr7 | 19053319 | G | C | 208 | Sympk | exonic | missense SNV | Sympk:NM_026605:exon24:c.G3131C:p.R1044P | . | 7qA3 | . | 0 | 1 |
| chr7 | 19451880 | A | G | 84 | Mark4 | intronic | . | . | . | 7qA3 | . | 0 | 1 |
| chr7 | 19886229 | T | G | 221 | Ceacam19 | intronic | . | . | . | 7qA3 | . | 0 | 1 |
| chr7 | 23358496 | C | A | 177 | Nlrp4e | intronic | . | . | . | 7qA3 | . | 0 | 1 |
| chr7 | 23965476 | G | A | 222 | . | intergenic | . | . | . | 7qA3 | rs218192852 | 0 | 1 |
| chr7 | 24104416 | G | A | 91 | Zfp180 | exonic | missense SNV | Zfp180:NM_001289637:exon4:c.G187A:p.V63I,Zfp180:NM_001045486:exon5:c.G259A:p.V87I,Zfp180:NM_( | . | 7qA3 | . | 0 | 1 |
| chr7 | 24104775 | T | C | 222 | Zfp180 | exonic | synonymous SNV | Zfp180:NM_172483:exon3:c.T93C:p.S31S,Zfp180:NM_001289637:exon4:c.T546C:p.S182S,Zfp180:NM_001( | . | 7qA3 | rs36681335 | 0 | 1 |
| chr7 | 24104830 | C | T | 222 | Zfp180 | exonic | missense SNV | Zfp180:NM_172483:exon3:c.C148T:p.R50C,Zfp180:NM_001289637:exon4:c.C601T:p.R201C,Zfp180:NM_001 | . | 7qA3 | rs221623714 | 0 | 1 |
| chr7 | 24104972 | G | A | 222 | Zfp180 | exonic | missense SNV | Zfp180:NM_172483:exon3:c.G290A:p.R97H,Zfp180:NM_001289637:exon4:c.G743A:p.R248H,Zfp180:NM_001 | . | 7qA3 | rs36944654 | 0 | 1 |
| chr7 | 24104995 | G | A | 222 | Zfp180 | exonic | missense SNV | Zfp180:NM_172483:exon3:c.G313A:p.V105M,Zfp180:NM_001289637:exon4:c.G766A:p.V256M,Zfp180:NM_0( | . | 7qA3 | rs108061405 | 0 | 1 |
| chr7 | 24994791 | G | T | 210 | Atp1a3 | intronic | . | . | . | 7qA3 | . | 0 | 1 |
| chr7 | 25628830 | C | G | 222 | B3gnt8 | exonic | missense SNV | B3gnt8:NM_146184:exon1:c.C684G:p.C228W,B3gnt8:NM_001036740:exon2:c.C684G:p.C228W | . | 7qA3 | . | 0 | 1 |
| chr7 | 25764085 | A | C | 192 | Axl | intronic | . | . | . | 7qA3 | . | 0 | 1 |
| chr7 | 25764252 | C | A | 98 | Axl | intronic | . | . | . | 7qA3 | . | 0 | 1 |
| chr7 | 26558588 | T | C | 222 | Nlrp9a | exonic | missense SNV | Nlrp9a:NM_001302792:exon2:c.T1363C:p.C455R,Nlrp9a:NM_001048219:exon3:c.T1630C:p.C544R,Nlrp9a: | . | 7qA3 | . | 0 | 1 |
| chr7 | 26560877 | G | T | 222 | Nlrp9a | intronic | . | . | . | 7qA3 | . | 0 | 1 |
| chr7 | 27180654 | C | G | 139 | Mia | intronic | . | . | . | 7qA3 | . | 0 | 1 |
| chr7 | 27366804 | A | T | 56 | Sptbn4 | intronic | . | . | . | 7qA3 | . | 0 | 1 |
| chr7 | 27423784 | C | A | 222 | Sptbn4 | exonic | missense SNV | Sptbn4:NM_032610:exon7:c.G712T:p.A238S | . | 7qA3 | . | 0 | 1 |
| chr7 | 27428187 | G | C | 114 | Sptbn4 | exonic | missense SNV | Sptbn4:NM_032610:exon5:c.C587G:p.A196G | . | 7qA3 | . | 0 | 1 |
| chr7 | 27749524 | G | A | 222 | Zfp60 | exonic | missense SNV | Zfp60:NM_009560:exon5:c.G1616A:p.C539Y,Zfp60:NM_029531:exon5:c.G1616A:p.C539Y | . | 7qA3 | . | 0 | 1 |
| chr7 | 28089857 | G | T | 222 | Fcgbp | exonic | missense SNV | Fcgbp:NM_001122603:exon6:c.G1847T:p.W616L | . | 7qA3 | . | 0 | 1 |
| chr7 | 29143154 | G | A | 125 | Rasgrp4 | intronic | . | . | . | 7qB1 | rs232575540 | 0 | 1 |
| chr7 | 30360697 | G | C | 140 | Lrfn3 | exonic | missense SNV | Lrfn3:NM_175478:exon2:c.C102G:p.C34W | 405 | 7qB1 | . | 0 | 1 |
| chr7 | 30452756 | A | T | 140 | Kirrel2 | exonic | missense SNV | Kirrel2:NM_172898:exon11:c.T1426A:p.F476I | . | 7qB1 | . | 0 | 1 |
| chr7 | 30634603 | A | T | 203 | Etv2 | intronic | . | . | . | 7qB1 | . | 0 | 1 |
| chr7 | 30659231 | C | A | 203 | Haus5 | intronic | . | . | . | 7qB1 | . | 0 | 1 |
| chr7 | 30664930 | G | C | 221 | Haus5 | UTR5 | . | . | 267 | 7qB1 | . | 0 | 1 |
| chr7 | 30854785 | C | T | 72 | Ffar3 | UTR3 | . | . | . | 7qB1 | . | 0 | 1 |
| chr7 | 31052070 | G | T | 222 | Fxyd1 | intronic | . | . | . | 7qB1 | . | 0 | 1 |
| chr7 | 35363547 | T | G | 222 | Rhpn2 | intronic | . | . | . | 7qB2 | rs49121363 | 0 | 1 |
| chr7 | 35385333 | G | T | 222 | Rhpn2 | exonic | missense SNV | Rhpn2:NM_027897:exon14:c.G1651T:p.G551W | . | 7qB2 | . | 0 | 1 |
| chr7 | 36235048 | A | G | 222 | . | intergenic | . | . | . | 7qB2 | . | 0 | 1 |
| chr7 | 40191028 | A | C | 222 | . | intergenic | . | . | . | 7qB3 | rs579814763 | 0 | 1 |
| chr7 | 40191315 | A | T | 86 | . | intergenic | . | . | . | 7qB3 | rs583640163 | 0 | 1 |
| chr7 | 41428351 | G | T | 222 | Vmn2r57 | intronic | . | . | . | 7qB3 | . | 0 | 1 |
| chr7 | 41719647 | T | C | 222 | Vmn2r-ps54 | ncRNA_intronic | . | . | . | 7qB3 | . | 0 | 1 |
| chr7 | 42299895 | A | C | 222 | Vmn2r61 | exonic | missense SNV | Vmn2r61:NM_001105058:exon6:c.A1738C:p.S580R | . | 7qB3 | . | 0 | 1 |
| chr7 | 43356107 | A | C | 72 | Siglecf | intronic | . | . | . | 7qB3 | . | 0 | 1 |
| chr7 | 43443022 | C | T | 158 | Cldnd2 | intronic | . | . | . | 7qB3 | . | 0 | 1 |
| chr7 | 43498231 | C | T | 222 | 4931406B18Rik | exonic | missense SNV | 4931406B18Rik:NM_028737:exon5:c.G715A:p.G239R | . | 7qB3 | . | 0 | 1 |
| chr7 | 43803786 | T | G | 203 | Klk8 | UTR3 | . | . | . | 7qB3 | rs31326467 | 0 | 1 |
| chr7 | 43953936 | C | A | 222 | Klk1b8 | exonic | missense SNV | Klk1b8:NM_008457:exon3:c.C292A:p.H98N | . | 7qB3 | . | 0 | 1 |
| chr7 | 44250843 | T | C | 83 | 2410002F23Rik | exonic | synonymous SNV | 2410002F23Rik:NM_025880:exon6:c.T171C:p.S57S | . | 7qB3 | rs49857703 | 0 | 1 |
| chr7 | 44250858 | G | T | 87 | 2410002F23Rik | exonic | synonymous SNV | 2410002F23Rik:NM_025880:exon6:c.G186T:p.L62L | . | 7qB3 | rs49491671 | 0 | 1 |
| chr7 | 44250959 | G | A | 99 | 2410002F23Rik | exonic | missense SNV | 2410002F23Rik:NM_025880:exon6:c.G287A:p.R96H | . | 7qB3 | rs45798994 | 0 | 1 |
| chr7 | 44250996 | C | T | 47.7781 | 2410002F23Rik | exonic | synonymous SNV | 2410002F23Rik:NM_025880:exon6:c.C324T:p.T108T | . | 7qB3 | rs47247608 | 0 | 1 |
| chr7 | 44251008 | C | G | 64 | 2410002F23Rik | exonic | missense SNV | 2410002F23Rik:NM_025880:exon6:c.C336G:p.F112L | . | 7qB3 | rs27331048 | 0 | 1 |
| chr7 | 44251127 | C | T | 162 | 2410002F23Rik | exonic | missense SNV | 2410002F23Rik:NM_025880:exon6:c.C455T:p.T152I | . | 7qB3 | rs51876501 | 0 | 1 |
| chr7 | 44251128 | T | G | 159 | 2410002F23Rik | exonic | synonymous SNV | 2410002F23Rik:NM_025880:exon6:c.T456G:p.T152T | . | 7qB3 | rs52023267 | 0 | 1 |
| chr7 | 44251230 | G | A | 118 | 2410002F23Rik | exonic | missense SNV | 2410002F23Rik:NM_025880:exon6:c.G558A:p.M186I | . | 7qB3 | rs46939607 | 0 | 1 |
| chr7 | 44251231 | G | A | 105 | 2410002F23Rik | exonic | missense SNV | 2410002F23Rik:NM_025880:exon6:c.G559A:p.E187K | . | 7qB3 | rs47886978 | 0 | 1 |
| chr7 | 44252002 | G | A | 130 | 2410002F23Rik | UTR3 | . | . | . | 7qB3 | rs27243944 | 0 | 1 |
| chr7 | 44513762 | A | C | 89 | Mybpc2 | exonic | missense SNV | Mybpc2:NM_146189:exon13:c.T1363G:p.C455G | . | 7qB3 | . | 0 | 1 |
| chr7 | 44516544 | C | A | 222 | Mybpc2 | intronic | . | . | . | 7qB3 | . | 0 | 1 |
| chr7 | 45049014 | C | G | 212 | Prr12 | exonic | missense SNV | Prr12:NM_175022:exon4:c.G1476C:p.Q492H | . | 7qB3 | . | 0 | 1 |
| chr7 | 45185275 | T | A | 222 | Ccdc155 | splicing | . | . | . | 7qB3 | . | 0 | 1 |
| chr7 | 45287221 | G | T | 94 | 1700039E15Rik | exonic | missense SNV | 1700039E15Rik:NM_001033176:exon4:c.G498T:p.L166F | . | 7qB3 | . | 0 | 1 |
| chr7 | 45615170 | C | A | 222 | Fgf21 | exonic | missense SNV | Fgf21:NM_020013:exon1:c.G137T:p.R46L | . | 7qB3 | . | 0 | 1 |
| chr7 | 45793821 | G | T | 105 | Lmtk3 | exonic | missense SNV | Lmtk3:NM_001290990:exon11:c.G1849T:p.A617S,Lmtk3:NM_001005511:exon12:c.G1849T:p.A617S | . | 7qB3 | . | 0 | 1 |
| chr7 | 45999013 | A | C | 222 | Abcc6 | exonic | missense SNV | Abcc6:NM_018795:exon16:c.T2054G:p.V685G | . | 7qB3 | . | 0 | 1 |
| chr7 | 46305295 | C | T | 222 | Otog | exonic | synonymous SNV | Otog:NM_013624:exon50:c.C7998T:p.Y2666Y | . | 7qB3 | . | 0 | 1 |
| chr7 | 46815468 | A | C | 222 | Gtf2h1 | intronic | . | . | . | 7qB3 | . | 0 | 1 |
| chr7 | 46998153 | G | T | 222 | Spty2d1 | exonic | missense SNV | Spty2d1:NM_175318:exon3:c.C1027A:p.P343T | . | 7qB3 | . | 0 | 1 |
| chr7 | 47390724 | C | T | 135 | . | intergenic | . | . | . | 7qB3 | . | 0 | 1 |
| chr7 | 48642832 | A | T | 207 | . | downstream | . | . | . | 7qB4 | . | 0 | 1 |
| chr7 | 48724304 | A | C | 121 | . | intergenic | . | . | . | 7qB4 | . | 0 | 1 |
| chr7 | 49912097 | G | A | 168 | Slc6a5 | exonic | missense SNV | Slc6a5:NM_148931:exon1:c.G376A:p.A126T,Slc6a5:NM_001146013:exon2:c.G400A:p.A134T | 493 | 7qB4 | . | 0 | 1 |
| chr7 | 56131292 | G | A | 228 | Herc2 | exonic | missense SNV | Herc2:NM_010418:exon24:c.G3704A:p.G1235D | . | 7qB5 | rs50155421 | 1 | 0 |
| chr7 | 57488843 | C | T | 222 | Gabra5 | exonic | synonymous SNV | Gabra5:NM_176942:exon5:c.G456A:p.L152L | . | 7qB5 | . | 0 | 1 |
| chr7 | 57490791 | A | G | 222 | Gabra5 | exonic | synonymous SNV | Gabra5:NM_176942:exon3:c.T157C:p.L53L | . | 7qB5 | . | 0 | 1 |
| chr7 | 57824699 | C | T | 222 | Gabrb3 | exonic | synonymous SNV | Gabrb3:NM_001038701:exon9:c.C1362T:p.I454I,Gabrb3:NM_008071:exon9:c.C1362T:p.I454I | . | 7qB5 | . | 0 | 1 |
| chr7 | 59806733 | T | C | 107 | . | intergenic | . | . | . | 7qB5 | . | 0 | 1 |
| chr7 | 61549343 | G | T | 222 | B230209E15Rik | ncRNA_intronic | . | . | . | 7qC | . | 0 | 1 |
| chr7 | 62463503 | T | G | 195 | Peg12 | exonic | missense SNV | Peg12:NM_013788:exon1:c.A845C:p.N282T | . | 7qC | . | 0 | 1 |
| chr7 | 63606307 | A | G | 222 | Otud7a | intronic | . | . | . | 7qC | . | 0 | 1 |
| chr7 | 63757804 | C | A | 200 | Otud7a | exonic | missense SNV | Otud7a:NM_130880:exon14:c.C1854A:p.S618R | 1179 | 7qC | . | 0 | 1 |
| chr7 | 65342893 | C | A | 78 | Tjp1 | intronic | . | . | . | 7qC | . | 0 | 1 |
| chr7 | 67266202 | C | T | 196 | Mef2a | intronic | . | . | . | 7qC | . | 0 | 1 |
| chr7 | 67618012 | C | G | 222 | Lrrc28 | exonic | missense SNV | Lrrc28:NM_027413:exon5:c.G366C:p.Q122H,Lrrc28:NM_175124:exon5:c.G366C:p.Q122H | . | 7qC | . | 0 | 1 |
| chr7 | 68218580 | G | T | 206 | Igf1r | intronic | . | . | . | 7qC | . | 0 | 1 |
| chr7 | 72090365 | G | T | 146 | Mctp2 | intronic | . | . | . | 7qD1 | . | 0 | 1 |
| chr7 | 78192646 | T | A | 222 | Ntrk3 | exonic | synonymous SNV | Ntrk3:NM_008746:exon17:c.A2391T:p.P797P | . | 7qD2 | . | 0 | 1 |
| chr7 | 78516616 | A | G | 155 | Ntrk3 | intronic | . | . | . | 7qD2 | . | 0 | 1 |
| chr7 | 78843111 | C | A | 222 | Det1 | intronic | . | . | . | 7qD2 | . | 0 | 1 |
| chr7 | 79111322 | T | C | 228 | Acan | exonic | synonymous SNV | Acan:NM_007424:exon13:c.T5781C:p.T1927T | . | 7qD2 | rs31327362 | 1 | 0 |
| chr7 | 79111354 | A | C | 228 | Acan | exonic | missense SNV | Acan:NM_007424:exon13:c.A5813C:p.H1938P | . | 7qD2 | rs32310727 | 1 | 0 |
| chr7 | 80367698 | C | A | 195 | Man2a2 | exonic | synonymous SNV | Man2a2:NM_172903:exon7:c.G834T:p.L278L | . | 7qD2 | . | 0 | 1 |
| chr7 | 80898160 | T | G | 116 | Wdr73 | intronic | . | . | . | 7qD3 | . | 0 | 1 |
| chr7 | 81068076 | G | A | 174 | Alpk3 | intronic | . | . | . | 7qD3 | . | 0 | 1 |
| chr7 | 81076758 | C | A | 143 | Alpk3 | intronic | . | . | . | 7qD3 | . | 0 | 1 |
| chr7 | 81222356 | C | A | 222 | Pde8a | intronic | . | . | . | 7qD3 | . | 0 | 1 |
| chr7 | 81332885 | C | T | 218 | Pde8a | exonic | stopgain | Pde8a:NM_008803:exon21:c.C2251T:p.Q751X | . | 7qD3 | . | 0 | 1 |
| chr7 | 82603563 | G | A | 121 | Adamts13 | intronic | . | . | . | 7qD3 | . | 0 | 1 |
| chr7 | 82645821 | C | T | 222 | Saxo2 | intronic | . | . | . | 7qD3 | . | 0 | 1 |
| chr7 | 85561749 | T | C | 115 | Vmn2r70 | intronic | . | . | . | 7qD3 | . | 0 | 1 |
| chr7 | 85565790 | A | T | 177 | Vmn2r70 | intronic | . | . | . | 7qD3 | . | 0 | 1 |
| chr7 | 86466390 | A | G | 185 | Olfr299 | exonic | synonymous SNV | Olfr299:NM_001011767:exon1:c.A978G:p.R326R | . | 7qD3 | . | 0 | 1 |
| chr7 | 86803144 | T | C | 214 | Vmn2r77 | intronic | . | . | . | 7qD3 | . | 0 | 1 |
| chr7 | 89158974 | G | C | 222 | Tmem135 | exonic | stopgain | Tmem135:NM_028343:exon8:c.C593G:p.S198X | . | 7qD3 | . | 0 | 1 |
| chr7 | 89846352 | C | A | 222 | Me3 | intronic | . | . | . | 7qD3 | . | 0 | 1 |
| chr7 | 89866552 | A | T | 222 | Ccdc81 | exonic | missense SNV | Ccdc81:NM_001162979:exon15:c.T1918A:p.L640M | . | 7qD3 | . | 0 | 1 |
| chr7 | 90195587 | C | A | 152 | Picalm | intronic | . | . | . | 7qD3 | . | 0 | 1 |
| chr7 | 90451475 | T | C | 222 | Tmem126a | intronic | . | . | . | 7qE1 | . | 0 | 1 |
| chr7 | 91872723 | T | A | 45.8132 | Dlg2 | intronic | . | . | . | 7qE1 | . | 0 | 1 |
| chr7 | 96435802 | C | T | 179 | Tenm4 | intronic | . | . | . | 7qE1 | . | 0 | 1 |
| chr7 | 96892775 | G | C | 118 | Tenm4 | intronic | . | . | . | 7qE1 | . | 0 | 1 |
| chr7 | 97417057 | A | G | 228 | Thrsp | exonic | synonymous SNV | Thrsp:NM_009381:exon1:c.T447C:p.V149V | . | 7qE1 | rs13471530 | 1 | 0 |
| chr7 | 97526691 | C | T | 222 | Ints4 | exonic | missense SNV | Ints4:NM_027256:exon16:c.C1988T:p.T663I | . | 7qE1 | . | 0 | 1 |
| chr7 | 98594767 | T | C | 222 | Emsy | exonic | synonymous SNV | Emsy:NM_172280:exon18:c.A3105G:p.K1035K | . | 7qE1 | . | 0 | 1 |
| chr7 | 99667261 | A | G | 222 | Slco2b1 | intronic | . | . | . | 7qE1 | . | 0 | 1 |
| chr7 | 99685811 | C | A | 168 | Slco2b1 | intronic | . | . | . | 7qE1 | . | 0 | 1 |
| chr7 | 99688716 | C | T | 96 | Slco2b1 | intronic | . | . | . | 7qE1 | . | 0 | 1 |
| chr7 | 100427106 | G | A | 186 | C2cd3 | intronic | . | . | . | 7qE2 | . | 0 | 1 |
| chr7 | 100469445 | C | T | 207 | C2cd3 | exonic | missense SNV | C2cd3:NM_001017985:exon33:c.C6889T:p.P2297S | . | 7qE2 | . | 0 | 1 |
| chr7 | 100546591 | C | A | 124 | . | intergenic | . | . | . | 7qE2 | . | 0 | 1 |
| chr7 | 100582957 | C | T | 222 | Mrpl48 | intronic | . | . | 385 | 7qE2 | . | 0 | 1 |
| chr7 | 100653602 | C | T | 222 | Plekhb1 | intronic | . | . | . | 7qE2 | . | 0 | 1 |
| chr7 | 101300367 | G | A | 93 | Atg16l2 | intronic | . | . | . | 7qE2 | . | 0 | 1 |
| chr7 | 101997984 | C | A | 171 | Numa1 | intronic | . | . | . | 7qE2 | . | 0 | 1 |
| chr7 | 102087663 | C | T | 222 | Xntrpc,Trpc2 | exonic | synonymous SNV | Trpc2:NM_001109897:exon4:c.C1096T:p.L366L,Xntrpc:NM_011644:exon13:c.C2218T:p.L740L | . | 7qE2 | . | 0 | 1 |
| chr7 | 102698567 | A | G | 222 | Olfr557 | exonic | missense SNV | Olfr557:NM_146361:exon1:c.A328G:p.T110A | . | 7qE3 | . | 0 | 1 |
| chr7 | 102754003 | T | G | 147 | Olfr78 | intronic | . | . | . | 7qE3 | . | 0 | 1 |
| chr7 | 103041472 | A | T | 222 | . | upstream | . | . | . | 7qE3 | . | 0 | 1 |

| | | | | | | | | | | | | | |
|---|---|---|---|---|---|---|---|---|---|---|---|---|---|
| chr7 | 103187173 | T | C | 222 | Olfr592 | exonic | missense SNV | Olfr592:NM_207556:exon1:c.T571C:p.S191P | . | 7qE3 | . | 0 | 1 |
| chr7 | 103470673 | C | T | 222 | Olfr608 | exonic | synonymous SNV | Olfr608:NM_146756:exon1:c.C633T:p.D211D | . | 7qE3 | . | 0 | 1 |
| chr7 | 103604046 | A | C | 222 | Olfr619 | exonic | missense SNV | Olfr619:NM_147076:exon1:c.A391C:p.I131L | . | 7qE3 | . | 0 | 1 |
| chr7 | 103639478 | T | G | 222 | Olfr622 | exonic | missense SNV | Olfr622:NM_147083:exon1:c.A661C:p.K221Q | . | 7qE3 | . | 0 | 1 |
| chr7 | 103700022 | T | A | 106 | . | intergenic | . | . | . | 7qE3 | . | 0 | 1 |
| chr7 | 103777476 | G | A | 222 | Olfr68 | exonic | synonymous SNV | Olfr68:NM_013620:exon1:c.C868T:p.L290L | . | 7qE3 | . | 0 | 1 |
| chr7 | 103788073 | A | C | 222 | Olfr67 | exonic | missense SNV | Olfr67:NM_013619:exon2:c.T203G:p.F68C | . | 7qE3 | . | 0 | 1 |
| chr7 | 103989510 | G | C | 222 | . | intergenic | . | . | . | 7qE3 | . | 0 | 1 |
| chr7 | 104154265 | G | A | 222 | Olfm5 | exonic | synonymous SNV | Olfm5:NM_172905:exon6:c.C990T:p.Y330Y | . | 7qE3 | . | 0 | 1 |
| chr7 | 104484265 | T | C | 222 | Trim30d | exonic | synonymous SNV | Trim30d:NM_199146:exon3:c.A429G:p.L143L | . | 7qE3 | . | 0 | 1 |
| chr7 | 104688779 | T | A | 222 | Olfr661 | exonic | missense SNV | Olfr661:NM_146748:exon1:c.T763A:p.F255I | . | 7qE3 | . | 0 | 1 |
| chr7 | 104734515 | A | C | 70 | . | intergenic | . | . | . | 7qE3 | . | 0 | 1 |
| chr7 | 104821738 | G | C | 178 | . | intergenic | . | . | . | 7qE3 | . | 0 | 1 |
| chr7 | 104893424 | A | C | 222 | Olfr666 | exonic | missense SNV | Olfr666:NM_147096:exon1:c.T203G:p.L68R | . | 7qE3 | . | 0 | 1 |
| chr7 | 105384236 | C | A | 222 | Fam160a2 | exonic | missense SNV | Fam160a2:NM_001242364:exon9:c.G1814T:p.R605L,Fam160a2:NM_199009:exon9:c.G1772T:p.R591L,Fam160 | . | 7qE3 | . | 0 | 1 |
| chr7 | 105741457 | C | T | 221 | Ilk | exonic | synonymous SNV | Ilk:NM_001161724:exon8:c.C702T:p.D234D,Ilk:NM_010562:exon8:c.C702T:p.D234D | . | 7qE3 | . | 0 | 1 |
| chr7 | 105772795 | C | A | 186 | Dchs1 | exonic | missense SNV | Dchs1:NM_001162943:exon2:c.G417T:p.Q139H | . | 7qE3 | . | 0 | 1 |
| chr7 | 106418537 | A | G | 71 | . | intergenic | . | . | . | 7qE3 | . | 0 | 1 |
| chr7 | 106741655 | G | T | 222 | Olfr697 | exonic | missense SNV | Olfr697:NM_146599:exon1:c.C278A:p.S93Y | . | 7qE3 | . | 0 | 1 |
| chr7 | 106824116 | T | A | 222 | Olfr702 | exonic | missense SNV | Olfr702:NM_146597:exon2:c.A409T:p.T137S | . | 7qE3 | . | 0 | 1 |
| chr7 | 106971610 | G | A | 222 | Olfr711 | exonic | missense SNV | Olfr711:NM_147035:exon3:c.C733T:p.L245F | . | 7qE3 | . | 0 | 1 |
| chr7 | 107721174 | A | C | 128 | Ppfibp2 | intronic | . | . | . | 7qE3 | . | 0 | 1 |
| chr7 | 107721176 | G | C | 128 | Ppfibp2 | intronic | . | . | . | 7qE3 | . | 0 | 1 |
| chr7 | 107754622 | G | A | 228 | Cyb5r2 | intronic | . | . | . | 7qE3 | rs31235099 | 1 | 0 |
| chr7 | 108255755 | C | T | 222 | Olfr488 | exonic | missense SNV | Olfr488:NM_146732:exon1:c.G382A:p.A128T | . | 7qE3 | . | 0 | 1 |
| chr7 | 108459211 | A | G | 56 | . | intergenic | . | . | . | 7qE3 | . | 0 | 1 |
| chr7 | 108523402 | C | G | 222 | Olfr502 | exonic | missense SNV | Olfr502:NM_146739:exon1:c.G547C:p.D183H | . | 7qE3 | . | 0 | 1 |
| chr7 | 111077362 | C | A | 222 | Eif4g2 | intronic | . | . | . | 7qE3 | . | 0 | 1 |
| chr7 | 111858534 | G | C | 222 | . | intergenic | . | . | . | 7qF1 | rs213807270 | 0 | 1 |
| chr7 | 112056504 | G | C | 222 | Usp47 | exonic | synonymous SNV | Usp47:NM_133758:exon4:c.G387C:p.V129V,Usp47:NM_177249:exon5:c.G447C:p.V149V | . | 7qF1 | . | 0 | 1 |
| chr7 | 112856812 | G | T | 222 | Tead1 | exonic | missense SNV | Tead1:NM_001166585:exon4:c.G300T:p.K100N,Tead1:NM_009346:exon6:c.G312T:p.K104N,Tead1:NM_0011 | . | 7qF1 | . | 0 | 1 |
| chr7 | 114705330 | A | G | 39.8428 | . | intergenic | . | . | . | 7qF1 | . | 0 | 1 |
| chr7 | 114705422 | G | A | 119 | . | intergenic | . | . | . | 7qF1 | . | 0 | 1 |
| chr7 | 114705561 | A | G | 221 | . | intergenic | . | . | . | 7qF1 | . | 0 | 1 |
| chr7 | 114705629 | G | A | 101 | . | intergenic | . | . | . | 7qF1 | rs27349224 | 0 | 1 |
| chr7 | 116521658 | T | G | 75 | Nucb2 | intronic | . | . | . | 7qF1 | . | 0 | 1 |
| chr7 | 118141817 | C | T | 222 | Smg1 | exonic | missense SNV | Smg1:NM_001031814:exon56:c.G9788A:p.G3263D | . | 7qF1 | . | 0 | 1 |
| chr7 | 118166469 | T | G | 222 | Smg1 | exonic | missense SNV | Smg1:NM_001031814:exon37:c.A5730C:p.K1910N | . | 7qF1 | . | 0 | 1 |
| chr7 | 118564101 | C | A | 93 | Tmc7 | intronic | . | . | . | 7qF1 | . | 0 | 1 |
| chr7 | 119500591 | T | A | 222 | Pdilt | intronic | . | . | . | 7qF2 | . | 0 | 1 |
| chr7 | 119542799 | A | G | 222 | Acsm5 | UTR3 | . | . | . | 7qF2 | . | 0 | 1 |
| chr7 | 119596044 | C | T | 222 | Acsm2 | UTR3 | . | . | . | 7qF2 | . | 0 | 1 |
| chr7 | 119818292 | A | T | 114 | 2610020H08Rik | intronic | . | . | . | 7qF2 | . | 0 | 1 |
| chr7 | 119818401 | A | C | 222 | 2610020H08Rik | exonic | missense SNV | 2610020H08Rik:NM_028129:exon3:c.A47C:p.N16T,2610020H08Rik:NM_001004187:exon6:c.A623C:p.N208T | . | 7qF2 | . | 0 | 1 |
| chr7 | 119843851 | C | T | 219 | 2610020H08Rik | exonic | missense SNV | 2610020H08Rik:NM_028129:exon13:c.C1099T:p.R367C | . | 7qF2 | . | 0 | 1 |
| chr7 | 120022807 | C | A | 55 | . | intergenic | . | . | . | 7qF2 | . | 1 | 0 |
| chr7 | 120135179 | G | A | 222 | Zp2 | exonic | missense SNV | Zp2:NM_011775:exon14:c.C1646T:p.A549V | . | 7qF2 | rs235606231 | 0 | 1 |
| chr7 | 120212838 | T | C | 222 | Abca14 | intronic | . | . | . | 7qF2 | . | 0 | 1 |
| chr7 | 120289660 | A | C | 222 | Abca14 | intronic | . | . | . | 7qF2 | . | 0 | 1 |
| chr7 | 120312403 | T | A | 222 | Abca14 | intronic | . | . | . | 7qF2 | . | 0 | 1 |
| chr7 | 120319354 | A | C | 222 | Abca14 | exonic | missense SNV | Abca14:NM_026458:exon29:c.A4700C:p.K1567T | . | 7qF2 | . | 0 | 1 |
| chr7 | 120387942 | C | A | 117 | Abca15 | intronic | . | . | . | 7qF2 | . | 0 | 1 |
| chr7 | 120762274 | T | G | 141 | Vwa3a | intronic | . | . | . | 7qF2 | . | 0 | 1 |
| chr7 | 120946391 | A | G | 94 | Polr3e | UTR3 | . | . | . | 7qF2 | rs31991213 | 1 | 0 |
| chr7 | 121763405 | C | A | 222 | Scnn1g | intronic | . | . | . | 7qF2 | . | 0 | 1 |
| chr7 | 122010628 | C | T | 222 | Gga2 | intronic | . | . | . | 7qF2 | . | 0 | 1 |
| chr7 | 123191717 | G | T | 97 | Tnrc6a | intronic | . | . | . | 7qF3 | . | 0 | 1 |
| chr7 | 126104620 | C | A | 84 | Xpo6 | intronic | . | . | . | 7qF3 | . | 0 | 1 |
| chr7 | 126105056 | A | C | 222 | Xpo6 | intronic | . | . | . | 7qF3 | . | 0 | 1 |
| chr7 | 126174089 | G | T | 222 | Xpo6 | intronic | . | . | . | 7qF3 | . | 0 | 1 |
| chr7 | 126364125 | C | A | 159 | Lat | intronic | . | . | . | 7qF3 | . | 0 | 1 |
| chr7 | 126976844 | G | T | 222 | Cdipt | intronic | . | . | . | 7qF3 | . | 0 | 1 |
| chr7 | 127328115 | C | A | 169 | Itgal | intronic | . | . | . | 7qF3 | . | 0 | 1 |
| chr7 | 127934577 | C | A | 222 | Prss36 | exonic | synonymous SNV | Prss36:NM_001081374:exon11:c.G1590T:p.L530L | . | 7qF3 | . | 0 | 1 |
| chr7 | 128017048 | G | T | 222 | . | intergenic | . | . | . | 7qF3 | . | 0 | 1 |
| chr7 | 128108017 | G | T | 222 | Itgam | intronic | . | . | . | 7qF3 | . | 0 | 1 |
| chr7 | 128132918 | G | T | 214 | Itgax | intronic | . | . | . | 7qF3 | . | 0 | 1 |
| chr7 | 128175840 | T | C | 107 | Itgad | intronic | . | . | . | 7qF3 | . | 0 | 1 |
| chr7 | 128206273 | A | C | 72 | Cox6a2 | exonic | missense SNV | Cox6a2:NM_009943:exon1:c.T23G:p.L8R | . | 7qF3 | . | 0 | 1 |
| chr7 | 128389117 | G | A | 228 | Rgs10 | intronic | . | . | . | 7qF3 | rs49277200 | 1 | 0 |
| chr7 | 129634778 | C | G | 153 | Wdr11 | intronic | . | . | . | 7qF3 | . | 0 | 1 |
| chr7 | 130164173 | A | C | 222 | Fgfr2 | intronic | . | . | . | 7qF3 | . | 0 | 1 |
| chr7 | 130797113 | G | T | 122 | Btbd16 | intronic | . | . | . | 7qF3 | . | 0 | 1 |
| chr7 | 131049814 | T | C | 141 | Dmbt1 | intronic | . | . | . | 7qF3 | . | 0 | 1 |
| chr7 | 131063179 | T | C | 128 | Dmbt1 | intronic | . | . | . | 7qF3 | . | 0 | 1 |
| chr7 | 131065072 | C | A | 228 | Dmbt1 | exonic | missense SNV | Dmbt1:NM_007769:exon15:c.C1342A:p.P448T | . | 7qF3 | rs47873094 | 1 | 0 |
| chr7 | 131109526 | C | A | 222 | Dmbt1 | exonic | missense SNV | Dmbt1:NM_007769:exon42:c.C4510A:p.Q1504K | . | 7qF3 | . | 0 | 1 |
| chr7 | 132316726 | G | A | 43.9762 | Gm10584,Chst15 | ncRNA exonic | . | . | 1722 | 7qF3 | . | 0 | 1 |
| chr7 | 132991727 | G | A | 222 | Ctbp2 | exonic | synonymous SNV | Ctbp2:NM_001170744:exon5:c.C2244T:p.S748S,Ctbp2:NM_009980:exon7:c.C615T:p.S205S | . | 7qF3 | . | 0 | 1 |
| chr7 | 134266568 | C | G | 153 | D7Ertd443e | UTR3 | . | . | . | 7qF3 | rs33358138 | 0 | 1 |
| chr7 | 134266588 | C | A | 222 | D7Ertd443e | UTR3 | . | . | . | 7qF3 | rs33358140 | 0 | 1 |
| chr7 | 134266764 | G | T | 222 | D7Ertd443e | exonic | synonymous SNV | D7Ertd443e:NM_001081331:exon7:c.C1870A:p.R624R,D7Ertd443e:NM_001199941:exon8:c.C2011A:p.R671 | . | 7qF3 | rs33358141 | 0 | 1 |
| chr7 | 134293002 | T | C | 72 | D7Ertd443e | intronic | . | . | . | 7qF3 | rs33358688 | 0 | 1 |
| chr7 | 134293028 | T | C | 66 | D7Ertd443e | intronic | . | . | . | 7qF3 | rs33358691 | 0 | 1 |
| chr7 | 134293490 | T | G | 222 | D7Ertd443e | intronic | . | . | . | 7qF3 | rs33358693 | 0 | 1 |
| chr7 | 134293545 | T | C | 85 | D7Ertd443e | intronic | . | . | . | 7qF3 | rs254985473 | 0 | 1 |
| chr7 | 134298449 | T | C | 222 | D7Ertd443e | intronic | . | . | . | 7qF3 | rs33361140 | 0 | 1 |
| chr7 | 134348517 | T | A | 45.9771 | D7Ertd443e | intronic | . | . | . | 7qF3 | rs248197119 | 0 | 1 |
| chr7 | 134348562 | C | G | 86 | D7Ertd443e | intronic | . | . | . | 7qF3 | rs265901593 | 0 | 1 |
| chr7 | 134348580 | A | G | 90 | D7Ertd443e | intronic | . | . | . | 7qF3 | rs228443396 | 0 | 1 |
| chr7 | 134349405 | C | T | 222 | D7Ertd443e | exonic | missense SNV | D7Ertd443e:NM_001081331:exon2:c.G397A:p.G133S,D7Ertd443e:NM_001199941:exon3:c.G538A:p.G180S | . | 7qF3 | rs243733853 | 0 | 1 |
| chr7 | 134349544 | G | A | 222 | D7Ertd443e | exonic | synonymous SNV | D7Ertd443e:NM_001081331:exon2:c.C258T:p.S86S,D7Ertd443e:NM_001199941:exon3:c.C399T:p.S133S | . | 7qF3 | rs257980437 | 0 | 1 |
| chr7 | 134660371 | C | T | 37.6384 | . | intergenic | . | . | . | 7qF3 | rs215124535 | 0 | 1 |
| chr7 | 134660427 | C | T | 116 | . | intergenic | . | . | . | 7qF3 | rs230421136 | 0 | 1 |
| chr7 | 134660804 | A | T | 222 | . | intergenic | . | . | . | 7qF3 | rs215243583 | 0 | 1 |
| chr7 | 134660826 | T | C | 214 | . | intergenic | . | . | . | 7qF3 | rs258196934 | 0 | 1 |
| chr7 | 134661288 | G | A | 56 | . | intergenic | . | . | . | 7qF3 | rs33396927 | 0 | 1 |
| chr7 | 134661337 | G | A | 48.7212 | . | intergenic | . | . | . | 7qF3 | rs4228415 | 0 | 1 |
| chr7 | 134661647 | G | A | 41.7702 | . | intergenic | . | . | . | 7qF3 | rs33389818 | 0 | 1 |
| chr7 | 134661767 | C | T | 24.2823 | . | intergenic | . | . | . | 7qF3 | rs241660923 | 0 | 1 |
| chr7 | 134724151 | C | T | 77 | Dock1 | intronic | . | . | . | 7qF3 | rs259765490 | 0 | 1 |
| chr7 | 134724303 | T | C | 88 | Dock1 | intronic | . | . | . | 7qF3 | rs33406364 | 0 | 1 |
| chr7 | 134724315 | T | C | 203 | Dock1 | intronic | . | . | . | 7qF3 | rs33406367 | 0 | 1 |
| chr7 | 134724317 | A | T | 206 | Dock1 | intronic | . | . | . | 7qF3 | rs265276145 | 0 | 1 |
| chr7 | 134729934 | G | T | 170 | Dock1 | intronic | . | . | . | 7qF3 | rs243821477 | 0 | 1 |
| chr7 | 134729977 | A | G | 222 | Dock1 | intronic | . | . | . | 7qF3 | rs265012689 | 0 | 1 |
| chr7 | 134730095 | T | C | 175 | Dock1 | intronic | . | . | . | 7qF3 | rs232045752 | 0 | 1 |
| chr7 | 134733856 | C | T | 96 | Dock1 | intronic | . | . | . | 7qF3 | rs220893703 | 0 | 1 |
| chr7 | 134733895 | A | G | 126 | Dock1 | intronic | . | . | . | 7qF3 | rs247270692 | 0 | 1 |
| chr7 | 134734174 | A | G | 222 | Dock1 | intronic | . | . | . | 7qF3 | rs13479542 | 0 | 1 |
| chr7 | 134734209 | T | G | 192 | Dock1 | intronic | . | . | . | 7qF3 | rs228228738 | 0 | 1 |
| chr7 | 134737587 | T | C | 222 | Dock1 | intronic | . | . | . | 7qF3 | rs33401225 | 0 | 1 |
| chr7 | 134737754 | A | C | 222 | Dock1 | intronic | . | . | . | 7qF3 | rs33401227 | 0 | 1 |
| chr7 | 134737806 | T | C | 125 | Dock1 | intronic | . | . | . | 7qF3 | rs226465984 | 0 | 1 |
| chr7 | 134740812 | G | A | 70 | Dock1 | intronic | . | . | . | 7qF3 | rs33398024 | 0 | 1 |
| chr7 | 134740826 | C | T | 86 | Dock1 | intronic | . | . | . | 7qF3 | rs33398026 | 0 | 1 |
| chr7 | 134741120 | T | C | 222 | Dock1 | exonic | synonymous SNV | Dock1:NM_001033420:exon6:c.T459C:p.I153I | . | 7qF3 | rs33399024 | 0 | 1 |
| chr7 | 134741204 | G | A | 175 | Dock1 | intronic | . | . | . | 7qF3 | rs33399026 | 0 | 1 |
| chr7 | 134741231 | A | G | 134 | Dock1 | intronic | . | . | . | 7qF3 | rs33399029 | 0 | 1 |
| chr7 | 134741266 | A | G | 91 | Dock1 | intronic | . | . | . | 7qF3 | rs33399032 | 0 | 1 |
| chr7 | 134744161 | T | C | 182 | Dock1 | intronic | . | . | . | 7qF3 | rs219853190 | 0 | 1 |
| chr7 | 134744853 | A | G | 192 | Dock1 | intronic | . | . | . | 7qF3 | rs33402600 | 0 | 1 |
| chr7 | 134744944 | G | T | 222 | Dock1 | intronic | . | . | . | 7qF3 | rs33402603 | 0 | 1 |
| chr7 | 134744988 | G | A | 222 | Dock1 | exonic | synonymous SNV | Dock1:NM_001033420:exon8:c.G648A:p.K216K | . | 7qF3 | rs33403516 | 0 | 1 |
| chr7 | 134746468 | A | G | 222 | Dock1 | exonic | synonymous SNV | Dock1:NM_001033420:exon9:c.A807G:p.P269P | . | 7qF3 | rs252758177 | 0 | 1 |
| chr7 | 134746474 | T | C | 222 | Dock1 | exonic | synonymous SNV | Dock1:NM_001033420:exon9:c.T813C:p.D271D | . | 7qF3 | rs33405123 | 0 | 1 |
| chr7 | 134746537 | T | C | 204 | Dock1 | intronic | . | . | . | 7qF3 | rs33401165 | 0 | 1 |
| chr7 | 134746594 | A | G | 93 | Dock1 | intronic | . | . | . | 7qF3 | rs261475005 | 0 | 1 |
| chr7 | 134746801 | C | T | 36.4035 | Dock1 | intronic | . | . | . | 7qF3 | rs33401171 | 0 | 1 |
| chr7 | 134746827 | T | C | 80 | Dock1 | intronic | . | . | . | 7qF3 | rs249474534 | 0 | 1 |
| chr7 | 134746897 | C | A | 192 | Dock1 | intronic | . | . | . | 7qF3 | rs33401904 | 0 | 1 |
| chr7 | 134746967 | T | A | 222 | Dock1 | exonic | synonymous SNV | Dock1:NM_001033420:exon10:c.T909A:p.R303R | . | 7qF3 | rs33401907 | 0 | 1 |
| chr7 | 134747001 | A | C | 222 | Dock1 | exonic | synonymous SNV | Dock1:NM_001033420:exon10:c.A943C:p.R315R | . | 7qF3 | rs33401910 | 0 | 1 |
| chr7 | 134747104 | T | C | 203 | Dock1 | intronic | . | . | . | 7qF3 | rs33402656 | 0 | 1 |
| chr7 | 134747128 | T | C | 85 | Dock1 | intronic | . | . | . | 7qF3 | rs247790517 | 0 | 1 |
| chr7 | 134747153 | G | A | 60 | Dock1 | intronic | . | . | . | 7qF3 | rs264924618 | 0 | 1 |
| chr7 | 134753314 | C | T | 185 | Dock1 | intronic | . | . | . | 7qF3 | rs232464199 | 0 | 1 |
| chr7 | 134753324 | T | C | 193 | Dock1 | intronic | . | . | . | 7qF3 | rs33404777 | 0 | 1 |
| chr7 | 134753338 | T | C | 201 | Dock1 | intronic | . | . | . | 7qF3 | rs33404780 | 0 | 1 |
| chr7 | 134753461 | A | G | 222 | Dock1 | intronic | . | . | . | 7qF3 | rs33404783 | 0 | 1 |
| chr7 | 134753514 | A | G | 129 | Dock1 | intronic | . | . | . | 7qF3 | rs33405516 | 0 | 1 |
| chr7 | 134756972 | A | G | 220 | Dock1 | intronic | . | . | . | 7qF3 | rs218352097 | 0 | 1 |
| chr7 | 134760669 | A | C | 21.0457 | Dock1 | intronic | . | . | . | 7qF3 | rs33406063 | 0 | 1 |
| chr7 | 134760737 | T | G | 108 | Dock1 | intronic | . | . | . | 7qF3 | rs578858189 | 0 | 1 |
| chr7 | 134760753 | G | A | 127 | Dock1 | intronic | . | . | . | 7qF3 | rs33406956 | 0 | 1 |
| chr7 | 134760786 | T | C | 182 | Dock1 | intronic | . | . | . | 7qF3 | rs33406959 | 0 | 1 |
| chr7 | 134760921 | T | C | 222 | Dock1 | exonic | synonymous SNV | Dock1:NM_001033420:exon13:c.T1248C:p.P416P | . | 7qF3 | rs33407625 | 0 | 1 |
| chr7 | 134760998 | C | T | 222 | Dock1 | intronic | . | . | . | 7qF3 | rs219422459 | 0 | 1 |
| chr7 | 134761016 | A | G | 217 | Dock1 | intronic | . | . | . | 7qF3 | rs33407631 | 0 | 1 |
| chr7 | 134764106 | C | A | 55 | Dock1 | intronic | . | . | . | 7qF3 | rs33405150 | 0 | 1 |
| chr7 | 134764130 | G | T | 48.5299 | Dock1 | intronic | . | . | . | 7qF3 | rs33405153 | 0 | 1 |
| chr7 | 134765282 | T | C | 222 | Dock1 | exonic | synonymous SNV | Dock1:NM_001033420:exon15:c.T1452C:p.Y484Y | . | 7qF3 | rs33408976 | 0 | 1 |
| chr7 | 134765378 | C | T | 222 | Dock1 | intronic | . | . | . | 7qF3 | rs51365695 | 0 | 1 |
| chr7 | 134765428 | T | C | 127 | Dock1 | intronic | . | . | . | 7qF3 | rs33408979 | 0 | 1 |
| chr7 | 134766168 | A | G | 222 | Dock1 | intronic | . | . | . | 7qF3 | rs33407019 | 0 | 1 |
| chr7 | 134766316 | A | G | 222 | Dock1 | intronic | . | . | . | 7qF3 | rs266127670 | 0 | 1 |
| chr7 | 134766477 | C | T | 58 | Dock1 | intronic | . | . | . | 7qF3 | rs33407695 | 0 | 1 |
| chr7 | 134771375 | G | C | 148 | Dock1 | intronic | . | . | . | 7qF3 | rs243332363 | 0 | 1 |
| chr7 | 134771427 | C | T | 216 | Dock1 | intronic | . | . | . | 7qF3 | rs33406310 | 0 | 1 |
| chr7 | 134771586 | C | T | 222 | Dock1 | intronic | . | . | . | 7qF3 | rs33407196 | 0 | 1 |
| chr7 | 134771623 | A | C | 222 | Dock1 | intronic | . | . | . | 7qF3 | rs33407199 | 0 | 1 |
| chr7 | 134772162 | G | A | 102 | Dock1 | intronic | . | . | . | 7qF3 | rs33407980 | 0 | 1 |
| chr7 | 134772262 | G | T | 123 | Dock1 | intronic | . | . | . | 7qF3 | rs33407981 | 0 | 1 |
| chr7 | 134777308 | T | C | 161 | Dock1 | intronic | . | . | . | 7qF3 | rs33410402 | 0 | 1 |
| chr7 | 134777356 | G | T | 203 | Dock1 | intronic | . | . | . | 7qF3 | rs33411095 | 0 | 1 |
| chr7 | 134777538 | C | G | 222 | Dock1 | intronic | . | . | . | 7qF3 | rs33411097 | 0 | 1 |
| chr7 | 134777541 | C | A | 222 | Dock1 | intronic | . | . | . | 7qF3 | rs214934134 | 0 | 1 |
| chr7 | 134777592 | T | C | 69 | Dock1 | intronic | . | . | . | 7qF3 | rs33411100 | 0 | 1 |
| chr7 | 134782272 | G | A | 186 | Dock1 | intronic | . | . | . | 7qF3 | rs256982456 | 0 | 1 |
| chr7 | 134782312 | T | C | 222 | Dock1 | intronic | . | . | . | 7qF3 | rs228247256 | 0 | 1 |
| chr7 | 134782399 | A | C | 222 | Dock1 | exonic | synonymous SNV | Dock1:NM_001033420:exon20:c.A1998C:p.S666S | . | 7qF3 | rs33408757 | 0 | 1 |
| chr7 | 134782558 | G | A | 135 | Dock1 | intronic | . | . | . | 7qF3 | rs33408759 | 0 | 1 |
| chr7 | 134782583 | G | A | 148 | Dock1 | intronic | . | . | . | 7qF3 | rs33408760 | 0 | 1 |
| chr7 | 134782668 | G | A | 173 | Dock1 | intronic | . | . | . | 7qF3 | rs33408763 | 0 | 1 |
| chr7 | 134782707 | C | T | 171 | Dock1 | intronic | . | . | . | 7qF3 | rs33409956 | 0 | 1 |
| chr7 | 134782861 | C | T | 222 | Dock1 | intronic | . | . | . | 7qF3 | rs33409961 | 0 | 1 |
| chr7 | 134782889 | C | T | 204 | Dock1 | intronic | . | . | . | 7qF3 | rs33409963 | 0 | 1 |
| chr7 | 134782958 | G | C | 100 | Dock1 | intronic | . | . | . | 7qF3 | rs3698858 | 0 | 1 |
| chr7 | 134789059 | A | G | 55 | Dock1 | intronic | . | . | . | 7qF3 | rs33408000 | 0 | 1 |
| chr7 | 134789085 | G | A | 86 | Dock1 | intronic | . | . | . | 7qF3 | . | 0 | 1 |
| chr7 | 134789121 | T | C | 129 | Dock1 | intronic | . | . | . | 7qF3 | rs33408995 | 0 | 1 |
| chr7 | 134789154 | G | A | 163 | Dock1 | exonic | synonymous SNV | Dock1:NM_001033420:exon22:c.G2142A:p.K714K | . | 7qF3 | rs33408997 | 0 | 1 |
| chr7 | 134789199 | G | A | 213 | Dock1 | exonic | synonymous SNV | Dock1:NM_001033420:exon22:c.G2187A:p.P729P | . | 7qF3 | rs33409000 | 0 | 1 |
| chr7 | 134789303 | A | G | 109 | Dock1 | intronic | . | . | . | 7qF3 | rs33409984 | 0 | 1 |
| chr7 | 134876155 | T | C | 222 | Dock1 | intronic | . | . | . | 7qF3 | rs49134452 | 0 | 1 |

| | | | | | | | | | | | | | |
|---|---|---|---|---|---|---|---|---|---|---|---|---|---|
| chr7 | 134884321 | T | C | 119 | Dock1,Fam196a | UTR3 | . | . | . | 7qF3 | rs31935286 | 0 | 1 |
| chr7 | 134884707 | A | G | 86 | Dock1,Fam196a | intronic | . | . | . | 7qF3 | rs33425014 | 0 | 1 |
| chr7 | 134898976 | G | A | 87 | Dock1,Fam196a | intronic | . | . | . | 7qF3 | rs222674056 | 0 | 1 |
| chr7 | 134918342 | G | A | 222 | Fam196a,Dock1 | exonic | missense SNV | Fam196a:NM_001143802:exon2:c.C458T:p.A153V | . | 7qF3 | rs31429334 | 0 | 1 |
| chr7 | 136323300 | A | G | 222 | . | intergenic | . | . | . | 7qF3 | rs33569460 | 0 | 1 |
| chr7 | 136657773 | A | G | 190 | . | intergenic | . | . | . | 7qF3 | rs32015745 | 0 | 1 |
| chr7 | 136811460 | A | G | 222 | . | intergenic | . | . | . | 7qF3 | rs32386390 | 0 | 1 |
| chr7 | 136811627 | C | G | 222 | . | intergenic | . | . | . | 7qF3 | . | 0 | 1 |
| chr7 | 136811715 | T | C | 169 | . | intergenic | . | . | . | 7qF3 | rs31138635 | 0 | 1 |
| chr7 | 136812041 | T | C | 222 | . | intergenic | . | . | . | 7qF3 | rs32036367 | 0 | 1 |
| chr7 | 136812216 | A | G | 184 | . | intergenic | . | . | . | 7qF3 | rs13479551 | 0 | 1 |
| chr7 | 137127997 | C | T | 115 | Mgmt | exonic | missense SNV | Mgmt:NM_008598:exon5:c.C539T:p.P180L | . | 7qF3 | rs33673371 | 0 | 1 |
| chr7 | 137128098 | A | G | 44.4189 | Mgmt | UTR3 | . | . | . | 7qF3 | rs33674227 | 0 | 1 |
| chr7 | 137200463 | T | A | 222 | Ebf3 | exonic | missense SNV | Ebf3:NM_001113414:exon13:c.A1265T:p.H422L,Ebf3:NM_001113415:exon13:c.A1292T:p.H431L,Ebf3:NM_( | . | 7qF3 | . | 0 | 1 |
| chr7 | 137225594 | T | G | 148 | Ebf3 | intronic | . | . | . | 7qF3 | rs33691211 | 0 | 1 |
| chr7 | 137231441 | A | G | 196 | Ebf3 | intronic | . | . | . | 7qF3 | rs32013464 | 0 | 1 |
| chr7 | 137234666 | T | G | 60 | Ebf3 | intronic | . | . | . | 7qF3 | rs32107513 | 0 | 1 |
| chr7 | 137234757 | G | T | 116 | Gm10578,Ebf3 | ncRNA_exonic | . | . | . | 7qF3 | rs32350897 | 0 | 1 |
| chr7 | 137237059 | A | G | 161 | Gm10578,Ebf3 | ncRNA_intronic | . | . | . | 7qF3 | rs33695599 | 0 | 1 |
| chr7 | 137307440 | T | C | 222 | Ebf3 | intronic | . | . | . | 7qF3 | rs31645565 | 0 | 1 |
| chr7 | 137309431 | C | T | 218 | Ebf3 | intronic | . | . | 1434 | 7qF3 | rs50189300 | 0 | 1 |
| chr7 | 137313233 | C | G | 100 | Ebf3 | intronic | . | . | 2465 | 7qF3 | rs51362555 | 0 | 1 |
| chr7 | 137387382 | A | C | 222 | 9430038I01Rik | exonic | missense SNV | 9430038I01Rik:NM_029886:exon3:c.T283G:p.S95A | . | 7qF4 | rs51078189 | 0 | 1 |
| chr7 | 138062021 | A | G | 135 | . | intergenic | . | . | . | 7qF4 | rs49562962 | 0 | 1 |
| chr7 | 138062541 | T | C | 222 | . | intergenic | . | . | . | 7qF4 | . | 0 | 1 |
| chr7 | 138209059 | C | T | 121 | Tcergl1 | UTR3 | . | . | . | 7qF4 | rs6170408 | 0 | 1 |
| chr7 | 138209202 | A | G | 222 | Tcergl1 | UTR3 | . | . | . | 7qF4 | rs264961960 | 0 | 1 |
| chr7 | 138209350 | G | A | 43.9904 | Tcergl1 | UTR3 | . | . | . | 7qF4 | rs228080203 | 0 | 1 |
| chr7 | 138209637 | A | T | 52 | Tcergl1 | UTR3 | . | . | . | 7qF4 | rs213939309 | 0 | 1 |
| chr7 | 138209667 | C | T | 111 | Tcergl1 | UTR3 | . | . | . | 7qF4 | rs227798761 | 0 | 1 |
| chr7 | 138209675 | C | T | 119 | Tcergl1 | UTR3 | . | . | . | 7qF4 | rs33723657 | 0 | 1 |
| chr7 | 138209811 | A | C | 222 | Tcergl1 | exonic | synonymous SNV | Tcergl1:NM_183289:exon12:c.T1653G:p.R551R | . | 7qF4 | rs33723660 | 0 | 1 |
| chr7 | 138218141 | G | A | 222 | Tcergl1 | intronic | . | . | . | 7qF4 | rs33726479 | 0 | 1 |
| chr7 | 138248150 | C | A | 40.0031 | Tcergl1 | intronic | . | . | . | 7qF4 | rs252686389 | 0 | 1 |
| chr7 | 138248273 | T | A | 111 | Tcergl1 | intronic | . | . | . | 7qF4 | rs237573396 | 0 | 1 |
| chr7 | 138248289 | G | A | 174 | Tcergl1 | intronic | . | . | . | 7qF4 | rs33733649 | 0 | 1 |
| chr7 | 138248413 | A | G | 196 | Tcergl1 | exonic | synonymous SNV | Tcergl1:NM_183289:exon8:c.T1230C:p.D410D | . | 7qF4 | rs33734624 | 0 | 1 |
| chr7 | 138248425 | A | T | 184 | Tcergl1 | exonic | synonymous SNV | Tcergl1:NM_183289:exon8:c.T1218A:p.G406G | . | 7qF4 | rs33734626 | 0 | 1 |
| chr7 | 138248431 | A | G | 183 | Tcergl1 | exonic | synonymous SNV | Tcergl1:NM_183289:exon8:c.T1212C:p.S404S | . | 7qF4 | rs33734629 | 0 | 1 |
| chr7 | 138259784 | T | C | 25.4228 | Tcergl1 | intronic | . | . | . | 7qF4 | rs31980965 | 0 | 1 |
| chr7 | 138276546 | G | A | 193 | Tcergl1 | intronic | . | . | . | 7qF4 | rs32518830 | 0 | 1 |
| chr7 | 138276563 | C | T | 211 | Tcergl1 | intronic | . | . | . | 7qF4 | rs31585750 | 0 | 1 |
| chr7 | 138276727 | T | G | 222 | Tcergl1 | intronic | . | . | . | 7qF4 | rs32287726 | 0 | 1 |
| chr7 | 138276766 | T | C | 100 | Tcergl1 | intronic | . | . | . | 7qF4 | rs31697982 | 0 | 1 |
| chr7 | 138361579 | T | C | 146 | Tcergl1 | intronic | . | . | . | 7qF4 | rs33749587 | 0 | 1 |
| chr7 | 138874321 | G | A | 222 | Ppp2r2d | intronic | . | . | . | 7qF4 | rs244033857 | 0 | 1 |
| chr7 | 138874342 | G | A | 222 | Ppp2r2d | intronic | . | . | . | 7qF4 | rs211764222 | 0 | 1 |
| chr7 | 138876872 | T | C | 100 | Ppp2r2d | intronic | . | . | . | 7qF4 | rs33790362 | 0 | 1 |
| chr7 | 138894630 | T | C | 222 | Bnip3 | exonic | synonymous SNV | Bnip3:NM_009760:exon4:c.A348G:p.R116R | . | 7qF4 | rs13470974 | 0 | 1 |
| chr7 | 138894767 | T | C | 189 | Bnip3 | intronic | . | . | . | 7qF4 | rs33789908 | 0 | 1 |
| chr7 | 138897958 | A | G | 222 | Bnip3 | intronic | . | . | . | 7qF4 | rs33789911 | 0 | 1 |
| chr7 | 138915043 | C | T | 222 | . | intergenic | . | . | . | 7qF4 | rs252546482 | 0 | 1 |
| chr7 | 138915174 | C | T | 222 | . | intergenic | . | . | . | 7qF4 | rs31751419 | 0 | 1 |
| chr7 | 138989176 | C | A | 58 | Jakmip3 | intronic | . | . | . | 7qF4 | rs251664914 | 0 | 1 |
| chr7 | 139007456 | A | G | 222 | Jakmip3 | exonic | unknown | UNKNOWN | 219 | 7qF4 | rs32166712 | 0 | 1 |
| chr7 | 139017795 | G | T | 76 | Jakmip3 | intronic | . | . | . | 7qF4 | . | 0 | 1 |
| chr7 | 139018892 | T | C | 70 | Jakmip3 | intronic | . | . | . | 7qF4 | rs46673347 | 0 | 1 |
| chr7 | 139018965 | A | C | 198 | Jakmip3 | intronic | . | . | . | 7qF4 | rs257589270 | 0 | 1 |
| chr7 | 139018985 | C | T | 205 | Jakmip3 | intronic | . | . | . | 7qF4 | rs47551689 | 0 | 1 |
| chr7 | 139019133 | T | C | 222 | Jakmip3 | exonic | unknown | UNKNOWN | . | 7qF4 | rs33795455 | 0 | 1 |
| chr7 | 139019154 | T | C | 222 | Jakmip3 | exonic | unknown | UNKNOWN | . | 7qF4 | rs107815985 | 0 | 1 |
| chr7 | 139019157 | G | A | 222 | Jakmip3 | exonic | unknown | UNKNOWN | . | 7qF4 | rs212110223 | 0 | 1 |
| chr7 | 139019247 | T | C | 222 | Jakmip3 | intronic | . | . | . | 7qF4 | rs232909981 | 0 | 1 |
| chr7 | 139019359 | C | G | 162 | Jakmip3 | intronic | . | . | . | 7qF4 | rs107778929 | 0 | 1 |
| chr7 | 139019421 | C | T | 123 | Jakmip3 | intronic | . | . | . | 7qF4 | rs217116963 | 0 | 1 |
| chr7 | 139020419 | C | G | 103 | Jakmip3 | intronic | . | . | . | 7qF4 | rs33789574 | 0 | 1 |
| chr7 | 139022653 | A | C | 222 | Jakmip3 | intronic | . | . | . | 7qF4 | rs248349800 | 0 | 1 |
| chr7 | 139022838 | C | G | 102 | Jakmip3 | intronic | . | . | . | 7qF4 | rs33791918 | 0 | 1 |
| chr7 | 139025616 | A | G | 214 | Jakmip3 | intronic | . | . | . | 7qF4 | rs214185227 | 0 | 1 |
| chr7 | 139214705 | G | T | 121 | Lrrc27 | UTR5 | . | . | . | 7qF4 | rs13463174 | 0 | 1 |
| chr7 | 139214827 | C | T | 222 | Lrrc27 | exonic | synonymous SNV | Lrrc27:NM_027164:exon2:c.C105T:p.P35P,Lrrc27:NM_001143755:exon3:c.C105T:p.P35P | . | 7qF4 | rs13463173 | 0 | 1 |
| chr7 | 139214992 | C | T | 222 | Lrrc27 | intronic | . | . | . | 7qF4 | rs32086194 | 0 | 1 |
| chr7 | 139218004 | T | G | 86 | Lrrc27 | intronic | . | . | . | 7qF4 | rs50009007 | 0 | 1 |
| chr7 | 139218129 | G | A | 161 | Lrrc27 | intronic | . | . | . | 7qF4 | rs51389911 | 0 | 1 |
| chr7 | 139218136 | G | A | 174 | Lrrc27 | intronic | . | . | . | 7qF4 | rs47924633 | 0 | 1 |
| chr7 | 139223777 | A | T | 222 | Lrrc27 | intronic | . | . | . | 7qF4 | rs31615304 | 0 | 1 |
| chr7 | 139223852 | A | G | 106 | Lrrc27 | intronic | . | . | . | 7qF4 | rs32487426 | 0 | 1 |
| chr7 | 139226212 | A | G | 222 | Lrrc27 | exonic | synonymous SNV | Lrrc27:NM_027164:exon6:c.A792G:p.R264R,Lrrc27:NM_001143755:exon7:c.A792G:p.R264R | . | 7qF4 | rs32189661 | 0 | 1 |
| chr7 | 139226353 | C | T | 222 | Lrrc27 | exonic | synonymous SNV | Lrrc27:NM_027164:exon6:c.C933T:p.P311P,Lrrc27:NM_001143755:exon7:c.C933T:p.P311P | . | 7qF4 | rs31450359 | 0 | 1 |
| chr7 | 139226435 | A | G | 215 | Lrrc27 | intronic | . | . | . | 7qF4 | rs31294935 | 0 | 1 |
| chr7 | 139227946 | T | C | 222 | Lrrc27 | exonic | missense SNV | Lrrc27:NM_027164:exon7:c.T968C:p.I323T | . | 7qF4 | rs31605784 | 0 | 1 |
| chr7 | 139227967 | G | C | 222 | Lrrc27 | exonic | missense SNV | Lrrc27:NM_027164:exon7:c.G989C:p.G330A,Lrrc27:NM_001143755:exon8:c.G942C:p.R314S | . | 7qF4 | rs31992816 | 0 | 1 |
| chr7 | 139228113 | A | G | 219 | Lrrc27 | intronic | . | . | . | 7qF4 | rs32443395 | 0 | 1 |
| chr7 | 139230090 | A | G | 114 | Lrrc27 | intronic | . | . | . | 7qF4 | rs32287366 | 0 | 1 |
| chr7 | 139230188 | G | C | 222 | Lrrc27 | intronic | . | . | . | 7qF4 | rs31390458 | 0 | 1 |
| chr7 | 139230212 | A | G | 222 | Lrrc27 | exonic | synonymous SNV | Lrrc27:NM_027164:exon8:c.A1098G:p.R366R | . | 7qF4 | rs31178011 | 0 | 1 |
| chr7 | 139230323 | T | C | 211 | Lrrc27 | intronic | . | . | . | 7qF4 | rs13479558 | 0 | 1 |
| chr7 | 139236926 | A | G | 222 | Lrrc27 | exonic | synonymous SNV | Lrrc27:NM_027164:exon9:c.A1239G:p.V413V | . | 7qF4 | rs37240399 | 0 | 1 |
| chr7 | 139237115 | T | A | 185 | Lrrc27 | intronic | . | . | . | 7qF4 | rs51350344 | 0 | 1 |
| chr7 | 139237128 | G | T | 184 | Lrrc27 | intronic | . | . | . | 7qF4 | rs47844957 | 0 | 1 |
| chr7 | 139238487 | G | T | 80 | Lrrc27 | intronic | . | . | . | 7qF4 | rs32385450 | 0 | 1 |
| chr7 | 139238493 | C | T | 71 | Lrrc27 | intronic | . | . | . | 7qF4 | rs31623413 | 0 | 1 |
| chr7 | 139238494 | A | G | 78 | Lrrc27 | intronic | . | . | . | 7qF4 | rs31244297 | 0 | 1 |
| chr7 | 139238562 | T | C | 198 | Lrrc27 | intronic | . | . | . | 7qF4 | rs31979905 | 0 | 1 |
| chr7 | 139238763 | A | G | 222 | Lrrc27 | intronic | . | . | . | 7qF4 | rs32111024 | 0 | 1 |
| chr7 | 139242395 | T | G | 28.9574 | Lrrc27 | intronic | . | . | . | 7qF4 | rs50471483 | 0 | 1 |
| chr7 | 139242495 | T | G | 111 | Lrrc27 | intronic | . | . | . | 7qF4 | rs46955462 | 0 | 1 |
| chr7 | 139242549 | T | C | 155 | Lrrc27 | intronic | . | . | . | 7qF4 | rs50216756 | 0 | 1 |
| chr7 | 139242839 | A | G | 222 | Lrrc27 | UTR3 | . | . | . | 7qF4 | rs50640365 | 0 | 1 |
| chr7 | 139242939 | T | C | 130 | Lrrc27 | UTR3 | . | . | . | 7qF4 | rs39352668 | 0 | 1 |
| chr7 | 139255142 | A | C | 222 | Pwwp2b | exonic | synonymous SNV | Pwwp2b:NM_001033206:exon2:c.A498C:p.G166G,Pwwp2b:NM_001098636:exon2:c.A498C:p.G166G | . | 7qF4 | rs46450485 | 0 | 1 |
| chr7 | 139255220 | A | G | 222 | Pwwp2b | exonic | synonymous SNV | Pwwp2b:NM_001033206:exon2:c.A576G:p.Q192Q,Pwwp2b:NM_001098636:exon2:c.A576G:p.Q192Q | . | 7qF4 | rs31096494 | 0 | 1 |
| chr7 | 139255795 | G | C | 222 | Pwwp2b | exonic | missense SNV | Pwwp2b:NM_001033206:exon2:c.G1151C:p.S384T,Pwwp2b:NM_001098636:exon2:c.G1151C:p.S384T | . | 7qF4 | rs31736276 | 0 | 1 |
| chr7 | 139523637 | A | G | 71 | Inpp5a | intronic | . | . | . | 7qF4 | rs49629396 | 0 | 1 |
| chr7 | 139525576 | G | A | 207 | Inpp5a | intronic | . | . | . | 7qF4 | rs31763242 | 0 | 1 |
| chr7 | 139558855 | A | C | 222 | Inpp5a | intronic | . | . | . | 7qF4 | rs31051294 | 0 | 1 |
| chr7 | 139558864 | G | A | 222 | Inpp5a | intronic | . | . | . | 7qF4 | rs32478895 | 0 | 1 |
| chr7 | 139558915 | T | C | 222 | Inpp5a | exonic | synonymous SNV | Inpp5a:NM_001127363:exon10:c.T774C:p.A258A,Inpp5a:NM_183144:exon10:c.T774C:p.A258A | . | 7qF4 | rs6303477 | 0 | 1 |
| chr7 | 139559275 | A | G | 102 | Inpp5a | intronic | . | . | . | 7qF4 | rs6207380 | 0 | 1 |
| chr7 | 139567451 | T | C | 222 | Inpp5a | intronic | . | . | . | 7qF4 | rs32376166 | 0 | 1 |
| chr7 | 139575077 | C | G | 222 | Inpp5a | intronic | . | . | . | 7qF4 | rs46111664 | 0 | 1 |
| chr7 | 139575125 | G | A | 217 | Inpp5a | intronic | . | . | . | 7qF4 | rs39622005 | 0 | 1 |
| chr7 | 139575161 | A | G | 92 | Inpp5a | intronic | . | . | . | 7qF4 | rs37135107 | 0 | 1 |
| chr7 | 139577436 | G | T | 222 | Inpp5a | intronic | . | . | . | 7qF4 | rs31404344 | 0 | 1 |
| chr7 | 139578336 | C | T | 153 | Inpp5a | intronic | . | . | . | 7qF4 | rs31221542 | 0 | 1 |
| chr7 | 139578339 | T | C | 151 | Inpp5a | intronic | . | . | . | 7qF4 | rs31541932 | 0 | 1 |
| chr7 | 139578348 | C | A | 145 | Inpp5a | intronic | . | . | . | 7qF4 | rs31684732 | 0 | 1 |
| chr7 | 139581430 | G | A | 92 | Nkx6-2 | UTR3 | . | . | 2008 | 7qF4 | rs32129156 | 0 | 1 |
| chr7 | 139603223 | T | C | 222 | Cfap46 | exonic | synonymous SNV | Cfap46:NM_001302546:exon58:c.A7779G:p.K2593K | . | 7qF4 | rs31252076 | 0 | 1 |
| chr7 | 139603364 | A | G | 222 | Cfap46 | intronic | . | . | . | 7qF4 | rs31812907 | 0 | 1 |
| chr7 | 139603686 | A | G | 70 | Cfap46 | intronic | . | . | . | 7qF4 | rs32458901 | 0 | 1 |
| chr7 | 139603698 | A | G | 79 | Cfap46 | intronic | . | . | . | 7qF4 | rs31423812 | 0 | 1 |
| chr7 | 139605460 | A | G | 32.6594 | Cfap46 | intronic | . | . | . | 7qF4 | rs31876477 | 0 | 1 |
| chr7 | 139607332 | C | T | 222 | Cfap46 | intronic | . | . | . | 7qF4 | rs51668507 | 0 | 1 |
| chr7 | 139613659 | C | A | 96 | Cfap46 | intronic | . | . | . | 7qF4 | rs48781299 | 0 | 1 |
| chr7 | 139613679 | G | A | 45.9871 | Cfap46 | intronic | . | . | . | 7qF4 | rs50080972 | 0 | 1 |
| chr7 | 139614275 | A | G | 102 | Cfap46 | intronic | . | . | . | 7qF4 | rs31765983 | 0 | 1 |
| chr7 | 139614340 | T | C | 175 | Cfap46 | intronic | . | . | . | 7qF4 | rs31553142 | 0 | 1 |
| chr7 | 139614624 | G | C | 58 | Cfap46 | intronic | . | . | . | 7qF4 | rs31496557 | 0 | 1 |
| chr7 | 139614901 | T | C | 94 | Cfap46 | intronic | . | . | . | 7qF4 | rs32414369 | 0 | 1 |
| chr7 | 139614961 | A | G | 133 | Cfap46 | intronic | . | . | . | 7qF4 | rs107837103 | 0 | 1 |
| chr7 | 139614970 | T | G | 144 | Cfap46 | intronic | . | . | . | 7qF4 | rs31247916 | 0 | 1 |
| chr7 | 139615057 | G | T | 222 | Cfap46 | splicing | . | . | . | 7qF4 | rs31981016 | 0 | 1 |
| chr7 | 139615203 | T | C | 208 | Cfap46 | intronic | . | . | . | 7qF4 | rs31336895 | 0 | 1 |
| chr7 | 139615214 | G | A | 171 | Cfap46 | intronic | . | . | . | 7qF4 | rs31221184 | 0 | 1 |
| chr7 | 139615421 | G | A | 93 | Cfap46 | intronic | . | . | . | 7qF4 | rs32358190 | 0 | 1 |
| chr7 | 139639071 | A | G | 217 | Cfap46 | intronic | . | . | . | 7qF4 | rs48873699 | 0 | 1 |
| chr7 | 139639140 | G | A | 222 | Cfap46 | intronic | . | . | . | 7qF4 | rs51294350 | 0 | 1 |
| chr7 | 139639228 | C | A | 66 | Cfap46 | intronic | . | . | . | 7qF4 | rs4139554 | 0 | 1 |
| chr7 | 139639454 | A | C | 106 | Cfap46 | intronic | . | . | . | 7qF4 | rs3681993 | 0 | 1 |
| chr7 | 139639500 | G | C | 141 | Cfap46 | intronic | . | . | . | 7qF4 | rs3682064 | 0 | 1 |
| chr7 | 139640363 | C | T | 122 | Cfap46 | exonic | missense SNV | Cfap46:NM_001302546:exon28:c.G3775A:p.A1259T | . | 7qF4 | rs32479790 | 0 | 1 |
| chr7 | 139640371 | T | C | 169 | Cfap46 | exonic | missense SNV | Cfap46:NM_001302546:exon28:c.A3767G:p.N1256S | . | 7qF4 | rs31052266 | 0 | 1 |
| chr7 | 139640399 | T | C | 222 | Cfap46 | exonic | missense SNV | Cfap46:NM_001302546:exon28:c.A3739G:p.I1247V | . | 7qF4 | rs32305044 | 0 | 1 |
| chr7 | 139640594 | C | T | 211 | Cfap46 | intronic | . | . | . | 7qF4 | rs3702720 | 0 | 1 |
| chr7 | 139646118 | A | C | 60 | Cfap46 | intronic | . | . | . | 7qF4 | rs217252831 | 0 | 1 |
| chr7 | 139646484 | A | G | 167 | Cfap46 | intronic | . | . | . | 7qF4 | rs31995989 | 0 | 1 |
| chr7 | 139646522 | A | G | 120 | Cfap46 | intronic | . | . | . | 7qF4 | rs31543455 | 0 | 1 |
| chr7 | 139646536 | G | A | 55 | Cfap46 | intronic | . | . | . | 7qF4 | rs32423843 | 0 | 1 |
| chr7 | 139651489 | C | T | 222 | Cfap46 | intronic | . | . | . | 7qF4 | rs31139369 | 0 | 1 |
| chr7 | 139653301 | C | T | 185 | Cfap46 | intronic | . | . | . | 7qF4 | rs31522069 | 0 | 1 |
| chr7 | 139654558 | C | T | 145 | Cfap46 | intronic | . | . | . | 7qF4 | rs239830236 | 0 | 1 |
| chr7 | 139654604 | C | G | 187 | Cfap46 | exonic | synonymous SNV | Cfap46:NM_001302546:exon17:c.G2079C:p.V693V | . | 7qF4 | rs32241365 | 0 | 1 |
| chr7 | 139656033 | C | T | 155 | Cfap46 | intronic | . | . | . | 7qF4 | rs31207604 | 0 | 1 |
| chr7 | 139656401 | C | T | 137 | Cfap46 | intronic | . | . | . | 7qF4 | rs39565253 | 0 | 1 |
| chr7 | 139657236 | A | G | 58 | Cfap46 | intronic | . | . | . | 7qF4 | rs32513071 | 0 | 1 |
| chr7 | 139657239 | A | G | 60 | Cfap46 | intronic | . | . | . | 7qF4 | rs31935267 | 0 | 1 |
| chr7 | 139657253 | G | A | 189 | Cfap46 | intronic | . | . | . | 7qF4 | rs49725343 | 0 | 1 |
| chr7 | 139657376 | C | T | 222 | Cfap46 | exonic | synonymous SNV | Cfap46:NM_001302546:exon14:c.G1617A:p.G539G | . | 7qF4 | rs259785822 | 0 | 1 |
| chr7 | 139657458 | A | G | 80 | Cfap46 | intronic | . | . | . | 7qF4 | rs32518847 | 0 | 1 |
| chr7 | 139657467 | G | A | 82 | Cfap46 | intronic | . | . | . | 7qF4 | rs32017731 | 0 | 1 |
| chr7 | 139660151 | G | A | 55 | Cfap46 | intronic | . | . | 252 | 7qF4 | rs49704295 | 0 | 1 |
| chr7 | 139660159 | C | T | 44.2113 | Cfap46 | intronic | . | . | 252 | 7qF4 | rs31823045 | 0 | 1 |
| chr7 | 139660546 | T | G | 222 | Cfap46 | intronic | . | . | . | 7qF4 | rs31417104 | 0 | 1 |
| chr7 | 139666336 | T | C | 222 | Cfap46 | exonic | synonymous SNV | Cfap46:NM_001302546:exon11:c.A1110G:p.R370R | . | 7qF4 | rs31398468 | 0 | 1 |
| chr7 | 139667178 | A | G | 69 | Cfap46 | intronic | . | . | . | 7qF4 | rs31910275 | 0 | 1 |
| chr7 | 139675937 | G | A | 222 | Cfap46 | intronic | . | . | . | 7qF4 | rs3686087 | 0 | 1 |
| chr7 | 139676165 | A | G | 120 | Cfap46 | intronic | . | . | . | 7qF4 | rs50233177 | 0 | 1 |
| chr7 | 139678322 | C | T | 108 | Cfap46 | intronic | . | . | . | 7qF4 | rs4137072 | 0 | 1 |
| chr7 | 139678333 | C | T | 105 | Cfap46 | intronic | . | . | . | 7qF4 | rs4137092 | 0 | 1 |
| chr7 | 139679775 | C | T | 107 | Cfap46 | intronic | . | . | . | 7qF4 | rs46741280 | 0 | 1 |
| chr7 | 139680797 | C | A | 149 | Cfap46 | intronic | . | . | . | 7qF4 | rs32137922 | 0 | 1 |
| chr7 | 139682276 | C | T | 67 | Cfap46 | intronic | . | . | . | 7qF4 | rs31530697 | 0 | 1 |
| chr7 | 139719392 | C | T | 201 | . | intergenic | . | . | . | 7qF4 | rs47434405 | 0 | 1 |
| chr7 | 139719614 | G | A | 222 | . | intergenic | . | . | . | 7qF4 | rs241087208 | 0 | 1 |
| chr7 | 139719700 | G | T | 222 | . | intergenic | . | . | . | 7qF4 | rs229264054 | 0 | 1 |
| chr7 | 139719734 | T | C | 93 | . | intergenic | . | . | . | 7qF4 | rs246782855 | 0 | 1 |
| chr7 | 139774780 | C | G | 124 | . | intergenic | . | . | . | 7qF4 | rs36399503 | 0 | 1 |
| chr7 | 139774789 | G | A | 139 | . | intergenic | . | . | . | 7qF4 | rs221155036 | 0 | 1 |
| chr7 | 139775013 | A | G | 222 | . | intergenic | . | . | . | 7qF4 | rs36951586 | 0 | 1 |
| chr7 | 139845551 | G | A | 66 | Adgra1 | intronic | . | . | . | 7qF4 | rs31450664 | 0 | 1 |
| chr7 | 139845629 | C | T | 134 | Adgra1 | exonic | synonymous SNV | Adgra1:NM_177469:exon4:c.C57T:p.F19F | . | 7qF4 | rs256117190 | 0 | 1 |
| chr7 | 139875126 | A | G | 208 | Adgra1 | exonic | synonymous SNV | Adgra1:NM_177469:exon8:c.A669G:p.R223R | . | 7qF4 | rs31666023 | 0 | 1 |
| chr7 | 139901376 | A | G | 112 | Kndc1 | intronic | . | . | . | 7qF4 | rs33864057 | 0 | 1 |
| chr7 | 139901853 | G | A | 84 | Kndc1 | intronic | . | . | . | 7qF4 | rs47477990 | 0 | 1 |

| | | | | | | | | | | | | | |
|---|---|---|---|---|---|---|---|---|---|---|---|---|---|
| chr7 | 139901885 | G | A | 84 | Kndc1 | intronic | . | . | . | 7qF4 | rs49743005 | 0 | 1 |
| chr7 | 139901932 | G | A | 114 | Kndc1 | intronic | . | . | . | 7qF4 | rs50723236 | 0 | 1 |
| chr7 | 139901974 | T | C | 222 | Kndc1 | intronic | . | . | . | 7qF4 | rs48384135 | 0 | 1 |
| chr7 | 139902013 | C | T | 222 | Kndc1 | exonic | synonymous SNV | Kndc1:NM_177261:exon3:c.C318T:p.A106A | . | 7qF4 | rs47811780 | 0 | 1 |
| chr7 | 139902117 | A | G | 222 | Kndc1 | intronic | . | . | . | 7qF4 | rs235775865 | 0 | 1 |
| chr7 | 139902213 | C | A | 70 | Kndc1 | intronic | . | . | . | 7qF4 | rs256146449 | 0 | 1 |
| chr7 | 139902234 | A | G | 56 | Kndc1 | intronic | . | . | . | 7qF4 | rs225122693 | 0 | 1 |
| chr7 | 139902260 | T | C | 20.4772 | Kndc1 | intronic | . | . | . | 7qF4 | rs245782202 | 0 | 1 |
| chr7 | 139910019 | G | A | 80 | Kndc1 | intronic | . | . | . | 7qF4 | rs230021617 | 0 | 1 |
| chr7 | 139910046 | G | A | 91 | Kndc1 | intronic | . | . | . | 7qF4 | rs47045846 | 0 | 1 |
| chr7 | 139910340 | A | G | 140 | Kndc1 | exonic | missense SNV | Kndc1:NM_177261:exon6:c.A757G:p.R253G | . | 7qF4 | rs47305175 | 0 | 1 |
| chr7 | 139910341 | G | A | 140 | Kndc1 | exonic | missense SNV | Kndc1:NM_177261:exon6:c.G758A:p.R253K | . | 7qF4 | rs48410848 | 0 | 1 |
| chr7 | 139910408 | T | C | 95 | Kndc1 | exonic | synonymous SNV | Kndc1:NM_177261:exon6:c.T825C:p.P275P | . | 7qF4 | rs32437772 | 0 | 1 |
| chr7 | 139910449 | A | G | 159 | Kndc1 | exonic | missense SNV | Kndc1:NM_177261:exon6:c.A866G:p.D289G | . | 7qF4 | rs32378494 | 0 | 1 |
| chr7 | 139910636 | T | C | 222 | Kndc1 | exonic | synonymous SNV | Kndc1:NM_177261:exon6:c.T1053C:p.P351P | . | 7qF4 | rs31503732 | 0 | 1 |
| chr7 | 139910863 | C | T | 222 | Kndc1 | exonic | missense SNV | Kndc1:NM_177261:exon6:c.C1280T:p.S427L | . | 7qF4 | rs49826399 | 0 | 1 |
| chr7 | 139913421 | G | T | 58 | Kndc1 | intronic | . | . | . | 7qF4 | rs31056308 | 0 | 1 |
| chr7 | 139913429 | T | C | 58 | Kndc1 | intronic | . | . | . | 7qF4 | rs31746422 | 0 | 1 |
| chr7 | 139913450 | A | G | 54 | Kndc1 | intronic | . | . | . | 7qF4 | rs32412157 | 0 | 1 |
| chr7 | 139913886 | C | A | 158 | Kndc1 | intronic | . | . | . | 7qF4 | rs31907333 | 0 | 1 |
| chr7 | 139913893 | T | C | 166 | Kndc1 | intronic | . | . | . | 7qF4 | rs31490202 | 0 | 1 |
| chr7 | 139914079 | A | G | 222 | Kndc1 | intronic | . | . | . | 7qF4 | rs32189090 | 0 | 1 |
| chr7 | 139914171 | A | G | 222 | Kndc1 | exonic | synonymous SNV | Kndc1:NM_177261:exon9:c.A1581G:p.E527E | . | 7qF4 | rs32086351 | 0 | 1 |
| chr7 | 139914294 | C | G | 61 | Kndc1 | intronic | . | . | . | 7qF4 | rs32243439 | 0 | 1 |
| chr7 | 139918723 | C | T | 101 | Kndc1 | intronic | . | . | . | 7qF4 | rs48248800 | 1 | 0 |
| chr7 | 139919051 | C | T | 41.2537 | Kndc1 | intronic | . | . | . | 7qF4 | rs47491118 | 0 | 1 |
| chr7 | 139919305 | G | C | 110 | Kndc1 | intronic | . | . | . | 7qF4 | rs48649546 | 0 | 1 |
| chr7 | 139921115 | T | C | 130 | Kndc1 | exonic | synonymous SNV | Kndc1:NM_177261:exon14:c.T2319C:p.C773C | . | 7qF4 | rs31622575 | 0 | 1 |
| chr7 | 139921135 | C | T | 151 | Kndc1 | exonic | missense SNV | Kndc1:NM_177261:exon14:c.C2339T:p.A780V | . | 7qF4 | rs31933797 | 0 | 1 |
| chr7 | 139921231 | G | A | 172 | Kndc1 | exonic | missense SNV | Kndc1:NM_177261:exon14:c.G2435A:p.G812D | . | 7qF4 | rs13479562 | 0 | 1 |
| chr7 | 139921436 | T | G | 222 | Kndc1 | exonic | synonymous SNV | Kndc1:NM_177261:exon14:c.T2640G:p.L880L | . | 7qF4 | rs51732830 | 0 | 1 |
| chr7 | 139921751 | C | T | 222 | Kndc1 | intronic | . | . | . | 7qF4 | rs50692720 | 0 | 1 |
| chr7 | 139922598 | T | G | 69 | Kndc1 | intronic | . | . | . | 7qF4 | rs47008563 | 0 | 1 |
| chr7 | 139922599 | C | G | 66 | Kndc1 | intronic | . | . | . | 7qF4 | rs46193546 | 0 | 1 |
| chr7 | 139922630 | A | G | 89 | Kndc1 | intronic | . | . | . | 7qF4 | rs48580160 | 0 | 1 |
| chr7 | 139922718 | A | G | 173 | Kndc1 | exonic | synonymous SNV | Kndc1:NM_177261:exon16:c.A2919G:p.L973L | . | 7qF4 | rs48213749 | 0 | 1 |
| chr7 | 139922787 | A | G | 87 | Kndc1 | intronic | . | . | . | 7qF4 | rs48986918 | 0 | 1 |
| chr7 | 139923627 | T | A | 74 | Kndc1 | intronic | . | . | . | 7qF4 | rs3726791 | 0 | 1 |
| chr7 | 139923878 | G | A | 222 | Kndc1 | exonic | synonymous SNV | Kndc1:NM_177261:exon18:c.G3096A:p.Q1032Q | . | 7qF4 | rs50032753 | 0 | 1 |
| chr7 | 139924107 | A | G | 222 | Kndc1 | exonic | missense SNV | Kndc1:NM_177261:exon18:c.A3325G:p.T1109A | . | 7qF4 | rs31383756 | 0 | 1 |
| chr7 | 139927210 | A | G | 188 | Kndc1 | exonic | synonymous SNV | Kndc1:NM_177261:exon19:c.A3393G:p.R1131R | . | 7qF4 | rs47505326 | 0 | 1 |
| chr7 | 139927216 | T | C | 191 | Kndc1 | exonic | synonymous SNV | Kndc1:NM_177261:exon19:c.T3399C:p.Y1133Y | . | 7qF4 | rs46338122 | 0 | 1 |
| chr7 | 139927280 | A | G | 222 | Kndc1 | intronic | . | . | . | 7qF4 | rs36513723 | 0 | 1 |
| chr7 | 139927484 | C | T | 222 | Kndc1 | intronic | . | . | . | 7qF4 | rs51736845 | 0 | 1 |
| chr7 | 139927515 | C | G | 136 | Kndc1 | intronic | . | . | . | 7qF4 | rs47828173 | 0 | 1 |
| chr7 | 139927524 | C | T | 66 | Kndc1 | intronic | . | . | . | 7qF4 | rs49323256 | 0 | 1 |
| chr7 | 139927639 | T | C | 55 | Kndc1 | intronic | . | . | . | 7qF4 | rs37023366 | 0 | 1 |
| chr7 | 139927838 | A | G | 222 | Kndc1 | exonic | synonymous SNV | Kndc1:NM_177261:exon21:c.A3708G:p.R1236R | . | 7qF4 | rs36869899 | 0 | 1 |
| chr7 | 139927936 | T | C | 140 | Kndc1 | intronic | . | . | . | 7qF4 | rs46207330 | 0 | 1 |
| chr7 | 139930196 | A | G | 222 | Kndc1 | intronic | . | . | . | 7qF4 | rs220450563 | 0 | 1 |
| chr7 | 139931183 | A | G | 175 | Kndc1 | intronic | . | . | . | 7qF4 | rs31086177 | 0 | 1 |
| chr7 | 139931549 | T | C | 86 | Kndc1 | intronic | . | . | . | 7qF4 | rs31092776 | 1 | 0 |
| chr7 | 139931644 | C | G | 170 | Kndc1 | intronic | . | . | . | 7qF4 | rs32250293 | 0 | 1 |
| chr7 | 139931677 | G | A | 213 | Kndc1 | intronic | . | . | . | 7qF4 | rs32488450 | 0 | 1 |
| chr7 | 139931954 | G | A | 154 | Kndc1 | intronic | . | . | . | 7qF4 | rs32001620 | 0 | 1 |
| chr7 | 139932423 | C | G | 66 | Kndc1 | intronic | . | . | . | 7qF4 | rs32042972 | 0 | 1 |
| chr7 | 139932443 | C | T | 114 | Kndc1 | intronic | . | . | . | 7qF4 | rs32376540 | 0 | 1 |
| chr7 | 139932450 | A | G | 148 | Kndc1 | intronic | . | . | . | 7qF4 | rs31944445 | 0 | 1 |
| chr7 | 139932617 | C | A | 222 | Kndc1 | intronic | . | . | . | 7qF4 | rs31600602 | 0 | 1 |
| chr7 | 139932709 | A | G | 222 | Kndc1 | intronic | . | . | . | 7qF4 | rs31858342 | 0 | 1 |
| chr7 | 139933038 | C | T | 222 | Kndc1 | intronic | . | . | . | 7qF4 | rs32432901 | 0 | 1 |
| chr7 | 139933093 | A | G | 178 | Kndc1 | intronic | . | . | . | 7qF4 | rs32205824 | 0 | 1 |
| chr7 | 139936631 | C | A | 68 | Kndc1 | intronic | . | . | . | 7qF4 | rs32110471 | 0 | 1 |
| chr7 | 139936742 | T | C | 203 | Kndc1 | intronic | . | . | . | 7qF4 | rs31236879 | 0 | 1 |
| chr7 | 139936787 | T | G | 222 | Kndc1 | exonic | synonymous SNV | Kndc1:NM_177261:exon28:c.T4659G:p.L1553L | . | 7qF4 | rs31530470 | 0 | 1 |
| chr7 | 139938096 | G | A | 134 | Kndc1 | exonic | synonymous SNV | Kndc1:NM_177261:exon30:c.G4944A:p.L1648L | . | 7qF4 | rs32323470 | 0 | 1 |
| chr7 | 139938102 | A | G | 133 | Kndc1 | exonic | synonymous SNV | Kndc1:NM_177261:exon30:c.A4950G:p.T1650T | . | 7qF4 | rs31716683 | 0 | 1 |
| chr7 | 139938201 | G | A | 190 | Kndc1 | intronic | . | . | . | 7qF4 | rs32417161 | 0 | 1 |
| chr7 | 139944068 | C | T | 73 | Utf1 | exonic | synonymous SNV | Utf1:NM_009482:exon1:c.C195T:p.A65A | 1250 | 7qF4 | rs36296043 | 0 | 1 |
| chr7 | 139945025 | T | C | 58 | Utf1 | UTR3 | . | . | . | 7qF4 | rs31709990 | 0 | 1 |
| chr7 | 139945036 | C | T | 58 | Utf1 | UTR3 | . | . | . | 7qF4 | rs32333928 | 0 | 1 |
| chr7 | 139977965 | A | C | 97 | 6430531B16Rik | intronic | . | . | . | 7qF4 | . | 0 | 1 |
| chr7 | 139979517 | T | C | 95 | Adam8 | exonic | missense SNV | Adam8:NM_001291066:exon24:c.A2438G:p.E813G,Adam8:NM_007403:exon24:c.A2441G:p.E814G | . | 7qF4 | rs31575297 | 0 | 1 |
| chr7 | 139988609 | A | G | 222 | Adam8 | exonic | synonymous SNV | Adam8:NM_001291066:exon9:c.T711C:p.F237F,Adam8:NM_007403:exon9:c.T714C:p.F238F | . | 7qF4 | rs31554268 | 0 | 1 |
| chr7 | 140005056 | C | G | 222 | Tubgcp2 | intronic | . | . | . | 7qF4 | rs259294122 | 0 | 1 |
| chr7 | 140005133 | C | T | 199 | Tubgcp2 | intronic | . | . | . | 7qF4 | rs33794905 | 0 | 1 |
| chr7 | 140005170 | C | T | 186 | Tubgcp2 | intronic | . | . | . | 7qF4 | rs33794908 | 0 | 1 |
| chr7 | 140005466 | G | A | 222 | Tubgcp2 | intronic | . | . | . | 7qF4 | rs33794913 | 0 | 1 |
| chr7 | 140005548 | C | G | 104 | Tubgcp2 | intronic | . | . | . | 7qF4 | rs33795729 | 0 | 1 |
| chr7 | 140005559 | C | T | 78 | Tubgcp2 | intronic | . | . | . | 7qF4 | rs245814799 | 0 | 1 |
| chr7 | 140005565 | A | G | 81 | Tubgcp2 | intronic | . | . | . | 7qF4 | rs33795732 | 0 | 1 |
| chr7 | 140006164 | T | C | 178 | Tubgcp2 | splicing | . | . | . | 7qF4 | rs33797110 | 0 | 1 |
| chr7 | 140006215 | T | G | 124 | Tubgcp2 | intronic | . | . | . | 7qF4 | rs33791724 | 0 | 1 |
| chr7 | 140006238 | G | A | 85 | Tubgcp2 | intronic | . | . | . | 7qF4 | rs33791727 | 0 | 1 |
| chr7 | 140006718 | C | T | 32.6877 | Tubgcp2 | intronic | . | . | . | 7qF4 | rs255853793 | 0 | 1 |
| chr7 | 140006746 | A | G | 94 | Tubgcp2 | intronic | . | . | . | 7qF4 | rs33792679 | 0 | 1 |
| chr7 | 140006765 | C | G | 115 | Tubgcp2 | intronic | . | . | . | 7qF4 | rs33792682 | 0 | 1 |
| chr7 | 140006956 | G | A | 222 | Tubgcp2 | exonic | synonymous SNV | Tubgcp2:NM_001286011:exon7:c.C225T:p.S75S,Tubgcp2:NM_001286007:exon8:c.C1110T:p.S370S,Tubgcp2 | . | 7qF4 | rs33793568 | 0 | 1 |
| chr7 | 140007064 | C | T | 77 | Tubgcp2 | intronic | . | . | . | 7qF4 | rs33793571 | 0 | 1 |
| chr7 | 140007130 | G | A | 35.3878 | Tubgcp2 | intronic | . | . | . | 7qF4 | rs33794454 | 0 | 1 |
| chr7 | 140007401 | T | C | 222 | Tubgcp2 | intronic | . | . | . | 7qF4 | rs227784043 | 0 | 1 |
| chr7 | 140007423 | A | G | 222 | Tubgcp2 | splicing | . | . | . | 7qF4 | rs246896501 | 0 | 1 |
| chr7 | 140007682 | C | T | 110 | Tubgcp2 | intronic | . | . | . | 7qF4 | rs33794457 | 0 | 1 |
| chr7 | 140007698 | T | C | 67 | Tubgcp2 | intronic | . | . | . | 7qF4 | rs243950916 | 0 | 1 |
| chr7 | 140007721 | C | T | 62 | Tubgcp2 | intronic | . | . | . | 7qF4 | rs578846697 | 0 | 1 |
| chr7 | 140008084 | G | A | 222 | Tubgcp2 | exonic | synonymous SNV | Tubgcp2:NM_001286007:exon6:c.C627T:p.P209P,Tubgcp2:NM_001286009:exon6:c.C627T:p.P209P,Tubgcp2 | . | 7qF4 | rs33795189 | 0 | 1 |
| chr7 | 140029819 | C | T | 222 | Tubgcp2 | intronic | . | . | . | 7qF4 | rs261827856 | 0 | 1 |
| chr7 | 140029996 | A | G | 126 | Tubgcp2 | intronic | . | . | . | 7qF4 | rs217889597 | 0 | 1 |
| chr7 | 140030781 | C | T | 53 | Tubgcp2 | intronic | . | . | . | 7qF4 | rs216007239 | 0 | 1 |
| chr7 | 140030938 | G | A | 222 | Tubgcp2 | intronic | . | . | . | 7qF4 | rs33791719 | 0 | 1 |
| chr7 | 140031957 | T | C | 43.6627 | Tubgcp2 | intronic | . | . | . | 7qF4 | rs263848755 | 0 | 1 |
| chr7 | 140031958 | C | T | 43.6627 | Tubgcp2 | intronic | . | . | . | 7qF4 | rs218958824 | 0 | 1 |
| chr7 | 140032013 | A | G | 147 | Tubgcp2 | intronic | . | . | . | 7qF4 | rs33792665 | 0 | 1 |
| chr7 | 140032146 | C | T | 222 | Tubgcp2 | exonic | synonymous SNV | Tubgcp2:NM_001286007:exon3:c.G252A:p.L84L,Tubgcp2:NM_001286009:exon3:c.G252A:p.L84L,Tubgcp2:N | . | 7qF4 | rs33792668 | 0 | 1 |
| chr7 | 140032355 | G | C | 118 | Tubgcp2 | intronic | . | . | . | 7qF4 | rs33792671 | 0 | 1 |
| chr7 | 140033715 | C | G | 222 | Tubgcp2 | exonic | synonymous SNV | Tubgcp2:NM_001286007:exon2:c.G48C:p.L16L,Tubgcp2:NM_001286009:exon2:c.G48C:p.L16L,Tubgcp2:NM | . | 7qF4 | rs233548064 | 0 | 1 |
| chr7 | 140033817 | G | C | 195 | Tubgcp2 | intronic | . | . | . | 7qF4 | rs248417498 | 0 | 1 |
| chr7 | 140033844 | G | C | 158 | Tubgcp2 | intronic | . | . | . | 7qF4 | rs33793580 | 0 | 1 |
| chr7 | 140033882 | C | T | 42.2829 | Tubgcp2 | intronic | . | . | . | 7qF4 | rs33793583 | 0 | 1 |
| chr7 | 140036736 | A | G | 57 | Zfp511 | intronic | . | . | 797 | 7qF4 | rs224734021 | 0 | 1 |
| chr7 | 140036755 | C | A | 79 | Zfp511 | intronic | . | . | 797 | 7qF4 | rs238289842 | 0 | 1 |
| chr7 | 140036756 | G | A | 66 | Zfp511 | intronic | . | . | 797 | 7qF4 | rs253516203 | 0 | 1 |
| chr7 | 140036782 | G | C | 161 | Zfp511 | intronic | . | . | 797 | 7qF4 | rs33795532 | 0 | 1 |
| chr7 | 140037012 | A | G | 78 | Zfp511 | intronic | . | . | . | 7qF4 | rs263628723 | 0 | 1 |
| chr7 | 140037166 | G | C | 43.9818 | Zfp511 | intronic | . | . | . | 7qF4 | rs228513948 | 0 | 1 |
| chr7 | 140037186 | A | G | 71 | Zfp511 | intronic | . | . | . | 7qF4 | rs244182313 | 0 | 1 |
| chr7 | 140037205 | C | T | 52 | Zfp511 | intronic | . | . | . | 7qF4 | rs211896878 | 0 | 1 |
| chr7 | 140037435 | G | A | 222 | Zfp511 | intronic | . | . | . | 7qF4 | rs254947263 | 0 | 1 |
| chr7 | 140037443 | T | C | 222 | Zfp511 | intronic | . | . | . | 7qF4 | rs216345801 | 0 | 1 |
| chr7 | 140037764 | G | C | 57 | Zfp511 | intronic | . | . | . | 7qF4 | rs33796298 | 0 | 1 |
| chr7 | 140039273 | T | C | 72 | Zfp511 | intronic | . | . | . | 7qF4 | rs259436603 | 0 | 1 |
| chr7 | 140039277 | G | A | 65 | Zfp511 | intronic | . | . | . | 7qF4 | rs220674942 | 0 | 1 |
| chr7 | 140039368 | A | G | 85 | Zfp511 | exonic | synonymous SNV | Zfp511:NM_027201:exon5:c.A597G:p.P199P | . | 7qF4 | rs252518987 | 0 | 1 |
| chr7 | 140039465 | G | C | 70 | Zfp511 | intronic | . | . | . | 7qF4 | rs33798239 | 0 | 1 |
| chr7 | 140039501 | T | C | 38.2038 | Zfp511 | intronic | . | . | . | 7qF4 | rs249653272 | 0 | 1 |
| chr7 | 140040362 | T | C | 185 | Zfp511 | UTR3 | . | . | . | 7qF4 | rs583818313 | 0 | 1 |
| chr7 | 140040380 | T | C | 85 | Zfp511 | UTR3 | . | . | . | 7qF4 | rs245975432 | 0 | 1 |
| chr7 | 140040389 | C | T | 54 | Zfp511 | UTR3 | . | . | . | 7qF4 | rs33800014 | 0 | 1 |
| chr7 | 140047770 | G | A | 150 | Msx3 | exonic | synonymous SNV | Msx3:NM_010836:exon2:c.C585T:p.V195V | 1555 | 7qF4 | rs33797249 | 0 | 1 |
| chr7 | 140048076 | T | G | 222 | Msx3 | exonic | synonymous SNV | Msx3:NM_010836:exon2:c.A279C:p.P93P | 1555 | 7qF4 | rs33797252 | 0 | 1 |
| chr7 | 140048207 | C | A | 115 | Msx3 | intronic | . | . | 1555 | 7qF4 | rs33798285 | 0 | 1 |
| chr7 | 140070379 | C | T | 71 | Caly | exonic | missense SNV | Caly:NM_001190385:exon5:c.G649A:p.A217T,Caly:NM_026769:exon5:c.G649A:p.A217T,Caly:NM_0011903 | 392 | 7qF4 | rs33799317 | 0 | 1 |
| chr7 | 140070650 | G | A | 193 | Caly | exonic | synonymous SNV | Caly:NM_001190385:exon5:c.C378T:p.T126T,Caly:NM_026769:exon5:c.C378T:p.T126T,Caly:NM_0011903 | 392 | 7qF4 | rs33799320 | 0 | 1 |
| chr7 | 140071537 | G | A | 121 | Caly | intronic | . | . | . | 7qF4 | rs216035524 | 0 | 1 |
| chr7 | 140071540 | G | A | 128 | Caly | intronic | . | . | . | 7qF4 | rs217423815 | 0 | 1 |
| chr7 | 140071592 | G | A | 222 | Caly | exonic | synonymous SNV | Caly:NM_001190385:exon4:c.C355T:p.L119L,Caly:NM_026769:exon4:c.C355T:p.L119L,Caly:NM_0011903 | . | 7qF4 | rs33792424 | 0 | 1 |
| chr7 | 140071725 | G | A | 222 | Caly | intronic | . | . | . | 7qF4 | rs33792433 | 0 | 1 |
| chr7 | 140071850 | A | G | 31.8097 | Caly | intronic | . | . | . | 7qF4 | rs248911350 | 0 | 1 |
| chr7 | 140072464 | C | T | 23.1385 | Caly | intronic | . | . | . | 7qF4 | rs33794328 | 0 | 1 |
| chr7 | 140073926 | C | T | 204 | Caly | intronic | . | . | . | 7qF4 | rs33797607 | 0 | 1 |
| chr7 | 140073998 | G | A | 222 | Caly | exonic | synonymous SNV | Caly:NM_001190385:exon2:c.C78T:p.S26S,Caly:NM_026769:exon2:c.C78T:p.S26S,Caly:NM_001190386:e | . | 7qF4 | rs214854779 | 0 | 1 |
| chr7 | 140074085 | T | G | 122 | Caly | UTR5 | . | . | . | 7qF4 | rs234548937 | 0 | 1 |
| chr7 | 140074092 | C | T | 122 | Caly | UTR5 | . | . | . | 7qF4 | rs33797610 | 0 | 1 |
| chr7 | 140093284 | G | A | 115 | . | upstream | . | . | . | 7qF4 | rs223021459 | 0 | 1 |
| chr7 | 140093361 | G | A | 186 | . | upstream | . | . | . | 7qF4 | rs241066192 | 0 | 1 |
| chr7 | 140093522 | G | A | 215 | Prap1 | intronic | . | . | . | 7qF4 | rs228118119 | 0 | 1 |
| chr7 | 140093628 | C | T | 85 | Prap1 | intronic | . | . | . | 7qF4 | rs236286539 | 0 | 1 |
| chr7 | 140095140 | T | C | 116 | Prap1 | intronic | . | . | . | 7qF4 | rs239881453 | 0 | 1 |
| chr7 | 140096676 | T | C | 222 | Prap1 | exonic | missense SNV | Prap1:NM_009475:exon4:c.T131C:p.V44A | . | 7qF4 | rs245305284 | 0 | 1 |
| chr7 | 140096694 | T | C | 222 | Prap1 | exonic | missense SNV | Prap1:NM_009475:exon4:c.T149C:p.L50P | . | 7qF4 | rs255455411 | 0 | 1 |
| chr7 | 140096846 | G | A | 222 | Prap1 | intronic | . | . | . | 7qF4 | rs260959116 | 0 | 1 |
| chr7 | 140096878 | A | T | 222 | Prap1 | splicing | . | . | . | 7qF4 | rs584914871 | 0 | 1 |
| chr7 | 140096928 | C | A | 222 | Prap1 | exonic | synonymous SNV | Prap1:NM_009475:exon5:c.C282A:p.R94R | . | 7qF4 | rs238365832 | 0 | 1 |
| chr7 | 140099477 | C | G | 97 | Fuom | UTR3 | . | . | . | 7qF4 | rs33798992 | 0 | 1 |
| chr7 | 140099520 | G | C | 173 | Fuom | UTR3 | . | . | . | 7qF4 | rs247136470 | 0 | 1 |
| chr7 | 140099643 | C | T | 222 | Fuom | exonic | synonymous SNV | Fuom:NM_001286218:exon5:c.G441A:p.X147X,Fuom:NM_026928:exon6:c.G462A:p.X154X | . | 7qF4 | rs256820283 | 0 | 1 |
| chr7 | 140099744 | T | C | 151 | Fuom | intronic | . | . | . | 7qF4 | rs240369712 | 0 | 1 |
| chr7 | 140099755 | A | G | 88 | Fuom | intronic | . | . | . | 7qF4 | rs251484126 | 0 | 1 |
| chr7 | 140099831 | T | G | 104 | Fuom | intronic | . | . | . | 7qF4 | rs215578431 | 0 | 1 |
| chr7 | 140099844 | T | C | 176 | Fuom | intronic | . | . | . | 7qF4 | rs33799768 | 0 | 1 |
| chr7 | 140100026 | T | C | 219 | Fuom | intronic | . | . | . | 7qF4 | rs263089573 | 0 | 1 |
| chr7 | 140100175 | G | A | 204 | Fuom | intronic | . | . | . | 7qF4 | rs33800629 | 0 | 1 |
| chr7 | 140100942 | A | G | 54 | Fuom | intronic | . | . | . | 7qF4 | rs259100965 | 0 | 1 |
| chr7 | 140101018 | C | T | 103 | Fuom | intronic | . | . | . | 7qF4 | . | 0 | 1 |
| chr7 | 140101104 | C | T | 125 | Fuom | exonic | missense SNV | Fuom:NM_001286218:exon3:c.G289A:p.E97K,Fuom:NM_001286217:exon4:c.G310A:p.E104K,Fuom:NM_02692 | . | 7qF4 | rs252725963 | 0 | 1 |
| chr7 | 140101213 | C | T | 195 | Fuom | intronic | . | . | . | 7qF4 | rs239812324 | 0 | 1 |
| chr7 | 140101251 | G | A | 200 | Fuom | intronic | . | . | . | 7qF4 | . | 0 | 1 |
| chr7 | 140101350 | G | A | 190 | Fuom | exonic | synonymous SNV | Fuom:NM_001286217:exon3:c.C207T:p.D69D,Fuom:NM_026928:exon3:c.C207T:p.D69D | . | 7qF4 | rs221984632 | 0 | 1 |
| chr7 | 140101641 | G | A | 60 | Fuom | exonic | missense SNV | Fuom:NM_001286218:exon2:c.C164T:p.P55L | . | 7qF4 | rs242965992 | 0 | 1 |
| chr7 | 140101691 | A | C | 47.9317 | Fuom | intronic | . | . | . | 7qF4 | rs250242153 | 0 | 1 |
| chr7 | 140101734 | C | T | 29.154 | Fuom | intronic | . | . | . | 7qF4 | . | 0 | 1 |
| chr7 | 140102238 | A | G | 220 | Fuom | intronic | . | . | . | 7qF4 | rs33801468 | 0 | 1 |
| chr7 | 140106199 | T | C | 172 | Echs1 | UTR3 | . | . | . | 7qF4 | rs250598806 | 0 | 1 |
| chr7 | 140106209 | G | C | 175 | Echs1 | UTR3 | . | . | . | 7qF4 | rs239789188 | 0 | 1 |
| chr7 | 140106299 | T | C | 222 | Echs1 | UTR3 | . | . | . | 7qF4 | rs33796327 | 0 | 1 |
| chr7 | 140106400 | A | G | 222 | Echs1 | intronic | . | . | . | 7qF4 | rs265476347 | 0 | 1 |
| chr7 | 140107969 | C | T | 117 | Echs1 | intronic | . | . | . | 7qF4 | rs217878596 | 0 | 1 |
| chr7 | 140108070 | A | C | 222 | Echs1 | intronic | . | . | . | 7qF4 | rs261908387 | 0 | 1 |
| chr7 | 140108251 | A | G | 158 | Echs1 | intronic | . | . | . | 7qF4 | rs33797209 | 0 | 1 |
| chr7 | 140109921 | A | G | 44.5005 | Echs1 | intronic | . | . | . | 7qF4 | rs263554541 | 0 | 1 |
| chr7 | 140109944 | T | G | 61 | Echs1 | intronic | . | . | . | 7qF4 | rs224511851 | 0 | 1 |
| chr7 | 140109974 | C | G | 117 | Echs1 | intronic | . | . | . | 7qF4 | rs259286289 | 0 | 1 |
| chr7 | 140110015 | T | G | 114 | Echs1 | exonic | synonymous SNV | Echs1:NM_053119:exon6:c.A711C:p.V237V | . | 7qF4 | rs224587420 | 0 | 1 |
| chr7 | 140110127 | G | A | 178 | Echs1 | intronic | . | . | . | 7qF4 | rs50691843 | 0 | 1 |
| chr7 | 140110190 | C | T | 139 | Echs1 | intronic | . | . | . | 7qF4 | rs48973415 | 0 | 1 |

| | | | | | | | | | | | | | |
|---|---|---|---|---|---|---|---|---|---|---|---|---|---|
| chr7 | 140110483 | C | T | 62 | Echs1 | intronic | . | . | . | 7qF4 | rs51548740 | 0 | 1 |
| chr7 | 140110484 | G | A | 71 | Echs1 | intronic | . | . | . | 7qF4 | rs50305840 | 0 | 1 |
| chr7 | 140110507 | C | A | 68 | Echs1 | intronic | . | . | . | 7qF4 | rs45955581 | 0 | 1 |
| chr7 | 140110558 | A | G | 197 | Echs1 | exonic | synonymous SNV | Echs1:NM_053119:exon5:c.T600C:p.A200A | . | 7qF4 | rs51907589 | 0 | 1 |
| chr7 | 140111807 | G | A | 222 | Echs1 | intronic | . | . | . | 7qF4 | rs248706177 | 0 | 1 |
| chr7 | 140111959 | C | T | 34.9773 | Echs1 | intronic | . | . | . | 7qF4 | rs212513505 | 0 | 1 |
| chr7 | 140112240 | G | A | 78 | Echs1 | intronic | . | . | . | 7qF4 | rs244028018 | 0 | 1 |
| chr7 | 140112311 | C | T | 120 | Echs1 | intronic | . | . | . | 7qF4 | rs257818777 | 0 | 1 |
| chr7 | 140112550 | C | T | 222 | Echs1 | splicing | . | . | . | 7qF4 | rs229033866 | 0 | 1 |
| chr7 | 140112565 | G | T | 222 | Echs1 | intronic | . | . | . | 7qF4 | rs254346222 | 0 | 1 |
| chr7 | 140112695 | A | G | 84 | Echs1 | intronic | . | . | . | 7qF4 | rs246772185 | 0 | 1 |
| chr7 | 140112701 | T | G | 47.5908 | Echs1 | intronic | . | . | . | 7qF4 | rs33799686 | 0 | 1 |
| chr7 | 140112973 | A | G | 166 | Echs1 | exonic | synonymous SNV | Echs1:NM_053119:exon2:c.T249C:p.G83G | . | 7qF4 | rs240640501 | 0 | 1 |
| chr7 | 140116204 | G | A | 87 | Echs1 | intronic | . | . | . | 7qF4 | . | 0 | 1 |
| chr7 | 140116258 | A | T | 113 | Echs1 | intronic | . | . | . | 7qF4 | rs252559611 | 0 | 1 |
| chr7 | 140116468 | G | A | 81 | . | upstream | . | . | 290 | 7qF4 | rs223611500 | 0 | 1 |
| chr7 | 140126269 | T | C | 120 | Paox | intronic | . | . | . | 7qF4 | rs50885310 | 0 | 1 |
| chr7 | 140126539 | G | A | 175 | Paox | exonic | missense SNV | Paox:NM_153783:exon2:c.G371A:p.R124H | . | 7qF4 | rs46462852 | 0 | 1 |
| chr7 | 140127443 | C | G | 154 | Paox | intronic | . | . | . | 7qF4 | rs262921796 | 0 | 1 |
| chr7 | 140127761 | T | C | 194 | Paox | intronic | . | . | . | 7qF4 | rs220898336 | 0 | 1 |
| chr7 | 140129391 | A | G | 210 | Paox | intronic | . | . | . | 7qF4 | rs257459327 | 0 | 1 |
| chr7 | 140129450 | A | G | 56 | Paox | intronic | . | . | . | 7qF4 | rs249929195 | 0 | 1 |
| chr7 | 140132233 | A | G | 41.9238 | Paox | intronic | . | . | . | 7qF4 | rs33804519 | 0 | 1 |
| chr7 | 140132256 | C | G | 84 | Paox | intronic | . | . | . | 7qF4 | rs33804522 | 0 | 1 |
| chr7 | 140132569 | G | A | 222 | Paox | intronic | . | . | . | 7qF4 | . | 0 | 1 |
| chr7 | 140132571 | G | A | 213 | Paox | intronic | . | . | . | 7qF4 | rs220156468 | 0 | 1 |
| chr7 | 140133877 | G | C | 22.7104 | Paox | intronic | . | . | . | 7qF4 | rs33798664 | 0 | 1 |
| chr7 | 140134179 | T | C | 102 | Paox | UTR3 | . | . | . | 7qF4 | rs33798667 | 0 | 1 |
| chr7 | 140137678 | C | T | 104 | Mtg1 | exonic | missense SNV | Mtg1:NM_199301:exon1:c.C14T:p.P5L | 295 | 7qF4 | rs264472500 | 0 | 1 |
| chr7 | 140138275 | C | T | 222 | Mtg1 | intronic | . | . | . | 7qF4 | rs33804207 | 0 | 1 |
| chr7 | 140138381 | A | C | 222 | Mtg1 | exonic | synonymous SNV | Mtg1:NM_199301:exon2:c.A172C:p.R58R | . | 7qF4 | rs13465213 | 0 | 1 |
| chr7 | 140138420 | C | T | 222 | Mtg1 | intronic | . | . | . | 7qF4 | rs33804212 | 0 | 1 |
| chr7 | 140138487 | C | T | 118 | Mtg1 | intronic | . | . | . | 7qF4 | rs33805208 | 0 | 1 |
| chr7 | 140140189 | T | C | 222 | Mtg1 | intronic | . | . | . | 7qF4 | rs33799367 | 0 | 1 |
| chr7 | 140140246 | A | G | 222 | Mtg1 | exonic | synonymous SNV | Mtg1:NM_199301:exon3:c.A225G:p.K75K | . | 7qF4 | rs33799370 | 0 | 1 |
| chr7 | 140140336 | A | G | 222 | Mtg1 | intronic | . | . | . | 7qF4 | rs227209568 | 0 | 1 |
| chr7 | 140143782 | C | T | 221 | Mtg1 | exonic | synonymous SNV | Mtg1:NM_199301:exon4:c.C324T:p.L108L | . | 7qF4 | rs33803195 | 0 | 1 |
| chr7 | 140143846 | G | T | 177 | Mtg1 | intronic | . | . | . | 7qF4 | rs33803198 | 0 | 1 |
| chr7 | 140143857 | G | A | 179 | Mtg1 | intronic | . | . | . | 7qF4 | . | 0 | 1 |
| chr7 | 140144098 | G | T | 108 | Mtg1 | intronic | . | . | . | 7qF4 | rs33803201 | 0 | 1 |
| chr7 | 140144552 | T | C | 222 | Mtg1 | intronic | . | . | . | 7qF4 | rs33804117 | 0 | 1 |
| chr7 | 140144888 | T | C | 45.8132 | Mtg1 | intronic | . | . | . | 7qF4 | rs33804120 | 0 | 1 |
| chr7 | 140145952 | T | C | 222 | Mtg1 | intronic | . | . | . | 7qF4 | rs33796970 | 0 | 1 |
| chr7 | 140146161 | A | G | 125 | Mtg1 | intronic | . | . | . | 7qF4 | rs227936191 | 0 | 1 |
| chr7 | 140146184 | C | T | 68 | Mtg1 | intronic | . | . | . | 7qF4 | . | 0 | 1 |
| chr7 | 140146831 | A | G | 222 | Mtg1 | exonic | synonymous SNV | Mtg1:NM_199301:exon8:c.A591G:p.V197V | . | 7qF4 | rs33798016 | 0 | 1 |
| chr7 | 140147077 | T | G | 155 | Mtg1 | intronic | . | . | . | 7qF4 | rs233050825 | 0 | 1 |
| chr7 | 140147079 | C | T | 153 | Mtg1 | intronic | . | . | . | 7qF4 | rs253346750 | 0 | 1 |
| chr7 | 140147341 | G | T | 221 | Mtg1 | intronic | . | . | . | 7qF4 | rs222060009 | 0 | 1 |
| chr7 | 140147348 | T | C | 203 | Mtg1 | intronic | . | . | . | 7qF4 | rs235520786 | 0 | 1 |
| chr7 | 140147365 | T | C | 102 | Mtg1 | intronic | . | . | . | 7qF4 | rs260930176 | 0 | 1 |
| chr7 | 140147371 | T | G | 58 | Mtg1 | intronic | . | . | . | 7qF4 | rs220416783 | 0 | 1 |
| chr7 | 140149736 | A | G | 160 | Mtg1 | intronic | . | . | . | 7qF4 | rs33798858 | 0 | 1 |
| chr7 | 140149763 | T | C | 172 | Mtg1 | exonic | synonymous SNV | Mtg1:NM_199301:exon10:c.T765C:p.Y255Y | . | 7qF4 | rs33798861 | 0 | 1 |
| chr7 | 140149866 | T | C | 191 | Mtg1 | intronic | . | . | . | 7qF4 | rs231262254 | 0 | 1 |
| chr7 | 140149868 | T | A | 192 | Mtg1 | intronic | . | . | . | 7qF4 | rs251756292 | 0 | 1 |
| chr7 | 140149884 | T | C | 185 | Mtg1 | intronic | . | . | . | 7qF4 | rs234872710 | 0 | 1 |
| chr7 | 140150070 | C | T | 80 | Mtg1 | intronic | . | . | . | 7qF4 | rs241650702 | 0 | 1 |
| chr7 | 140150083 | G | T | 67 | Mtg1 | intronic | . | . | . | 7qF4 | rs265375878 | 0 | 1 |
| chr7 | 140150241 | A | C | 197 | Mtg1 | exonic | missense SNV | Mtg1:NM_199301:exon11:c.A949C:p.M317L | . | 7qF4 | rs33799696 | 0 | 1 |
| chr7 | 140150306 | A | T | 167 | Mtg1 | UTR3 | . | . | . | 7qF4 | rs229358725 | 0 | 1 |
| chr7 | 140150311 | C | G | 167 | Mtg1 | UTR3 | . | . | . | 7qF4 | rs259287871 | 0 | 1 |
| chr7 | 140153132 | C | T | 86 | Sprn | UTR3 | . | . | . | 7qF4 | rs235868648 | 0 | 1 |
| chr7 | 140153178 | G | A | 140 | Sprn | UTR3 | . | . | . | 7qF4 | rs263172051 | 0 | 1 |
| chr7 | 140153203 | T | C | 158 | Sprn | UTR3 | . | . | . | 7qF4 | rs227083798 | 0 | 1 |
| chr7 | 140161897 | G | A | 49.0528 | . | downstream | . | . | . | 7qF4 | rs223994944 | 0 | 1 |
| chr7 | 140161997 | C | T | 121 | . | downstream | . | . | . | 7qF4 | rs257220798 | 0 | 1 |
| chr7 | 140162082 | T | G | 222 | Olfr522 | exonic | synonymous SNV | Olfr522:NM_146952:exon1:c.A867C:p.I289I | . | 7qF4 | rs257741291 | 0 | 1 |
| chr7 | 140162127 | T | C | 222 | Olfr522 | exonic | synonymous SNV | Olfr522:NM_146952:exon1:c.A822G:p.L274L | . | 7qF4 | rs33804108 | 0 | 1 |
| chr7 | 140162373 | G | A | 222 | Olfr522 | exonic | synonymous SNV | Olfr522:NM_146952:exon1:c.C576T:p.D192D | . | 7qF4 | rs33804111 | 0 | 1 |
| chr7 | 140162466 | C | T | 222 | Olfr522 | exonic | synonymous SNV | Olfr522:NM_146952:exon1:c.G483A:p.T161T | . | 7qF4 | rs33804984 | 0 | 1 |
| chr7 | 140162628 | A | C | 222 | Olfr522 | exonic | synonymous SNV | Olfr522:NM_146952:exon1:c.T321G:p.S107S | . | 7qF4 | rs33804987 | 0 | 1 |
| chr7 | 140162661 | C | T | 200 | Olfr522 | exonic | synonymous SNV | Olfr522:NM_146952:exon1:c.G288A:p.S96S | . | 7qF4 | rs51172344 | 0 | 1 |
| chr7 | 140162695 | T | C | 220 | Olfr522 | exonic | missense SNV | Olfr522:NM_146952:exon1:c.A254G:p.D85G | . | 7qF4 | rs33804990 | 0 | 1 |
| chr7 | 140162772 | G | A | 222 | Olfr522 | exonic | synonymous SNV | Olfr522:NM_146952:exon1:c.C177T:p.P59P | . | 7qF4 | rs51342588 | 0 | 1 |
| chr7 | 140162939 | A | G | 222 | Olfr522 | exonic | missense SNV | Olfr522:NM_146952:exon1:c.T10C:p.C4R | . | 7qF4 | rs33797115 | 0 | 1 |
| chr7 | 140162977 | T | C | 174 | . | upstream | . | . | . | 7qF4 | rs50504647 | 0 | 1 |
| chr7 | 140162992 | A | G | 103 | . | upstream | . | . | . | 7qF4 | rs51513926 | 0 | 1 |
| chr7 | 140163011 | A | G | 31.9904 | . | upstream | . | . | . | 7qF4 | rs51006994 | 0 | 1 |
| chr7 | 140176146 | C | T | 169 | Olfr523 | exonic | synonymous SNV | Olfr523:NM_146518:exon1:c.C43T:p.L15L | . | 7qF4 | rs33804665 | 0 | 1 |
| chr7 | 140176163 | A | G | 171 | Olfr523 | exonic | synonymous SNV | Olfr523:NM_146518:exon1:c.A60G:p.P20P | . | 7qF4 | rs33804668 | 0 | 1 |
| chr7 | 140176205 | T | C | 222 | Olfr523 | exonic | synonymous SNV | Olfr523:NM_146518:exon1:c.T102C:p.I34I | . | 7qF4 | rs33804671 | 0 | 1 |
| chr7 | 140176546 | G | A | 130 | Olfr523 | exonic | missense SNV | Olfr523:NM_146518:exon1:c.G443A:p.G148E | . | 7qF4 | rs33797140 | 0 | 1 |
| chr7 | 140176600 | T | C | 126 | Olfr523 | exonic | missense SNV | Olfr523:NM_146518:exon1:c.T497C:p.I166T | . | 7qF4 | rs33797143 | 0 | 1 |
| chr7 | 140177004 | C | A | 222 | Olfr523 | exonic | missense SNV | Olfr523:NM_146518:exon1:c.C901A:p.L301M | . | 7qF4 | . | 0 | 1 |
| chr7 | 140177106 | T | G | 222 | . | downstream | . | . | . | 7qF4 | rs33797849 | 0 | 1 |
| chr7 | 140177177 | C | T | 214 | . | downstream | . | . | . | 7qF4 | rs33797852 | 0 | 1 |
| chr7 | 140201845 | C | A | 222 | Olfr524 | exonic | missense SNV | Olfr524:NM_001011814:exon1:c.G924T:p.Q308H | . | 7qF4 | rs33802465 | 0 | 1 |
| chr7 | 140201932 | G | A | 222 | Olfr524 | exonic | synonymous SNV | Olfr524:NM_001011814:exon1:c.C837T:p.V279V | . | 7qF4 | . | 0 | 1 |
| chr7 | 140201986 | A | G | 222 | Olfr524 | exonic | synonymous SNV | Olfr524:NM_001011814:exon1:c.T783C:p.Y261Y | . | 7qF4 | rs33802468 | 0 | 1 |
| chr7 | 140202352 | G | A | 222 | Olfr524 | exonic | synonymous SNV | Olfr524:NM_001011814:exon1:c.C417T:p.S139S | . | 7qF4 | rs243861426 | 0 | 1 |
| chr7 | 140202723 | C | A | 192 | Olfr524 | exonic | missense SNV | Olfr524:NM_001011814:exon1:c.G46T:p.V16L | . | 7qF4 | rs214720504 | 0 | 1 |
| chr7 | 140202754 | C | T | 167 | Olfr524 | exonic | synonymous SNV | Olfr524:NM_001011814:exon1:c.G15A:p.R5R | . | 7qF4 | rs33803400 | 0 | 1 |
| chr7 | 140218517 | C | T | 36.7997 | Cd163l1 | UTR5 | . | . | . | 7qF4 | rs33806166 | 0 | 1 |
| chr7 | 140218561 | G | A | 81 | Cd163l1 | UTR5 | . | . | . | 7qF4 | rs33806169 | 0 | 1 |
| chr7 | 140218590 | G | T | 132 | Cd163l1 | exonic | missense SNV | Cd163l1:NM_172909:exon1:c.G26T:p.S9I | . | 7qF4 | rs33798615 | 0 | 1 |
| chr7 | 140218737 | A | G | 89 | Cd163l1 | intronic | . | . | . | 7qF4 | rs217447843 | 0 | 1 |
| chr7 | 140220239 | G | C | 84 | Cd163l1 | intronic | . | . | . | 7qF4 | rs258024491 | 0 | 1 |
| chr7 | 140220262 | T | C | 102 | Cd163l1 | intronic | . | . | . | 7qF4 | rs33800303 | 0 | 1 |
| chr7 | 140220298 | A | G | 113 | Cd163l1 | intronic | . | . | . | 7qF4 | rs33801076 | 0 | 1 |
| chr7 | 140222281 | T | C | 192 | Cd163l1 | exonic | synonymous SNV | Cd163l1:NM_172909:exon3:c.T522C:p.F174F | . | 7qF4 | rs33802093 | 0 | 1 |
| chr7 | 140222294 | C | G | 194 | Cd163l1 | exonic | missense SNV | Cd163l1:NM_172909:exon3:c.C535G:p.Q179E | . | 7qF4 | rs33802816 | 0 | 1 |
| chr7 | 140223314 | C | T | 222 | Cd163l1 | exonic | synonymous SNV | Cd163l1:NM_172909:exon4:c.C790T:p.L264L | . | 7qF4 | rs33806177 | 0 | 1 |
| chr7 | 140223824 | G | T | 122 | Cd163l1 | intronic | . | . | . | 7qF4 | rs33798896 | 0 | 1 |
| chr7 | 140224170 | G | A | 222 | Cd163l1 | exonic | missense SNV | Cd163l1:NM_172909:exon5:c.G1280A:p.G427E | . | 7qF4 | rs241564095 | 0 | 1 |
| chr7 | 140224171 | G | A | 222 | Cd163l1 | exonic | synonymous SNV | Cd163l1:NM_172909:exon5:c.G1281A:p.G427G | . | 7qF4 | rs256693734 | 0 | 1 |
| chr7 | 140227842 | C | G | 222 | Cd163l1 | intronic | . | . | . | 7qF4 | rs33803440 | 0 | 1 |
| chr7 | 140228067 | A | G | 222 | Cd163l1 | exonic | missense SNV | Cd163l1:NM_172909:exon8:c.A2150G:p.N717S | . | 7qF4 | rs33803443 | 0 | 1 |
| chr7 | 140228276 | A | G | 164 | Cd163l1 | intronic | . | . | . | 7qF4 | rs33804376 | 0 | 1 |
| chr7 | 140228364 | A | C | 163 | Cd163l1 | exonic | missense SNV | Cd163l1:NM_172909:exon9:c.A2260C:p.T754P | . | 7qF4 | rs33804379 | 0 | 1 |
| chr7 | 140228802 | A | G | 197 | Cd163l1 | exonic | synonymous SNV | Cd163l1:NM_172909:exon10:c.A2625G:p.A875A | . | 7qF4 | rs33805334 | 0 | 1 |
| chr7 | 140228898 | A | G | 222 | Cd163l1 | intronic | . | . | . | 7qF4 | rs33805340 | 0 | 1 |
| chr7 | 140229017 | T | C | 221 | Cd163l1 | intronic | . | . | . | 7qF4 | rs33805343 | 0 | 1 |
| chr7 | 140229169 | G | C | 76 | Cd163l1 | intronic | . | . | . | 7qF4 | rs33806099 | 0 | 1 |
| chr7 | 140229893 | T | C | 222 | Cd163l1 | intronic | . | . | . | 7qF4 | rs216304582 | 0 | 1 |
| chr7 | 140229895 | C | T | 222 | Cd163l1 | intronic | . | . | . | 7qF4 | rs231320840 | 0 | 1 |
| chr7 | 140230020 | T | C | 57 | Cd163l1 | intronic | . | . | . | 7qF4 | rs33798935 | 0 | 1 |
| chr7 | 140230064 | C | T | 41.0405 | Cd163l1 | intronic | . | . | . | 7qF4 | rs33798938 | 0 | 1 |
| chr7 | 140230367 | A | G | 222 | Cd163l1 | exonic | synonymous SNV | Cd163l1:NM_172909:exon13:c.A2871G:p.P957P | . | 7qF4 | rs33798941 | 0 | 1 |
| chr7 | 140230450 | C | T | 222 | Cd163l1 | exonic | missense SNV | Cd163l1:NM_172909:exon13:c.C2954T:p.T985I | . | 7qF4 | rs33799834 | 0 | 1 |
| chr7 | 140230588 | C | A | 41.8642 | Cd163l1 | intronic | . | . | . | 7qF4 | rs33799836 | 0 | 1 |
| chr7 | 140247292 | C | T | 222 | . | upstream | . | . | . | 7qF4 | rs33805501 | 0 | 1 |
| chr7 | 140247394 | T | C | 222 | 5830411N06Rik | exonic | missense SNV | 5830411N06Rik:NM_001128145:exon1:c.T14C:p.F5S,5830411N06Rik:NM_001128146:exon1:c.T14C:p.F5S,5 | . | 7qF4 | rs33806374 | 0 | 1 |
| chr7 | 140247512 | T | G | 222 | 5830411N06Rik | intronic | . | . | . | 7qF4 | rs33806380 | 0 | 1 |
| chr7 | 140247925 | T | C | 186 | 5830411N06Rik | intronic | . | . | . | 7qF4 | rs235266732 | 0 | 1 |
| chr7 | 140247964 | C | T | 222 | 5830411N06Rik | exonic | synonymous SNV | 5830411N06Rik:NM_001128145:exon2:c.C84T:p.F28F,5830411N06Rik:NM_001128146:exon2:c.C84T:p.F28F | . | 7qF4 | rs33799455 | 0 | 1 |
| chr7 | 140248641 | C | T | 76 | 5830411N06Rik | intronic | . | . | . | 7qF4 | rs246821758 | 0 | 1 |
| chr7 | 140248785 | G | T | 222 | 5830411N06Rik | intronic | . | . | . | 7qF4 | rs238801535 | 0 | 1 |
| chr7 | 140248789 | G | A | 222 | 5830411N06Rik | intronic | . | . | . | 7qF4 | rs33800319 | 0 | 1 |
| chr7 | 140249080 | C | T | 222 | 5830411N06Rik | exonic | synonymous SNV | 5830411N06Rik:NM_001128145:exon3:c.C651T:p.N217N,5830411N06Rik:NM_001128146:exon3:c.C651T:p.N | . | 7qF4 | rs33801048 | 0 | 1 |
| chr7 | 140249209 | C | G | 222 | 5830411N06Rik | intronic | . | . | . | 7qF4 | rs33801967 | 0 | 1 |
| chr7 | 140262673 | C | A | 221 | 5830411N06Rik | intronic | . | . | . | 7qF4 | rs33803824 | 0 | 1 |
| chr7 | 140262701 | G | C | 222 | 5830411N06Rik | intronic | . | . | . | 7qF4 | rs222157032 | 0 | 1 |
| chr7 | 140262860 | C | T | 222 | 5830411N06Rik | intronic | . | . | . | 7qF4 | rs33803833 | 0 | 1 |
| chr7 | 140262875 | C | T | 222 | 5830411N06Rik | intronic | . | . | . | 7qF4 | rs33804726 | 0 | 1 |
| chr7 | 140273888 | C | G | 134 | 5830411N06Rik | intronic | . | . | . | 7qF4 | rs262287296 | 0 | 1 |
| chr7 | 140274114 | C | T | 221 | 5830411N06Rik | exonic | synonymous SNV | 5830411N06Rik:NM_001128146:exon6:c.C1029T:p.Y343Y | . | 7qF4 | rs252192462 | 0 | 1 |
| chr7 | 140274118 | C | T | 221 | 5830411N06Rik | exonic | stopgain | 5830411N06Rik:NM_001128146:exon6:c.C1033T:p.Q345X | . | 7qF4 | rs217288315 | 0 | 1 |
| chr7 | 140274265 | C | G | 183 | 5830411N06Rik | intronic | . | . | . | 7qF4 | rs33806503 | 0 | 1 |
| chr7 | 140294656 | A | C | 222 | 5830411N06Rik | splicing | . | . | . | 7qF4 | rs33802770 | 0 | 1 |
| chr7 | 140294858 | G | A | 222 | 5830411N06Rik | exonic | synonymous SNV | 5830411N06Rik:NM_001128145:exon4:c.G900A:p.L300L,5830411N06Rik:NM_175533:exon4:c.G900A:p.L30( | . | 7qF4 | rs33803846 | 0 | 1 |
| chr7 | 140294888 | C | T | 222 | 5830411N06Rik | exonic | synonymous SNV | 5830411N06Rik:NM_001128145:exon4:c.C930T:p.N310N,5830411N06Rik:NM_175533:exon4:c.C930T:p.N31( | . | 7qF4 | . | 0 | 1 |
| chr7 | 140295605 | C | A | 48.7322 | 5830411N06Rik | intronic | . | . | . | 7qF4 | rs231395170 | 0 | 1 |
| chr7 | 140295686 | C | T | 154 | 5830411N06Rik | intronic | . | . | . | 7qF4 | rs33804708 | 0 | 1 |
| chr7 | 140295782 | T | A | 222 | 5830411N06Rik | intronic | . | . | . | 7qF4 | rs257115228 | 0 | 1 |
| chr7 | 140296174 | A | G | 222 | 5830411N06Rik | UTR3 | . | . | . | 7qF4 | rs33805457 | 0 | 1 |
| chr7 | 140296545 | A | G | 177 | 5830411N06Rik | exonic | synonymous SNV | 5830411N06Rik:NM_001128145:exon6:c.A1581G:p.V527V,5830411N06Rik:NM_001128146:exon9:c.A1929G:r | . | 7qF4 | rs33805460 | 0 | 1 |
| chr7 | 140296566 | T | C | 173 | 5830411N06Rik | exonic | synonymous SNV | 5830411N06Rik:NM_001128145:exon6:c.T1602C:p.H534H,5830411N06Rik:NM_001128146:exon9:c.T1950C:r | . | 7qF4 | rs33805463 | 0 | 1 |
| chr7 | 140296596 | G | C | 133 | 5830411N06Rik | exonic | synonymous SNV | 5830411N06Rik:NM_001128145:exon6:c.G1632C:p.V544V,5830411N06Rik:NM_001128146:exon9:c.G1980C:r | . | 7qF4 | rs33806216 | 0 | 1 |
| chr7 | 140296718 | A | G | 222 | 5830411N06Rik | exonic | missense SNV | 5830411N06Rik:NM_001128145:exon7:c.A1673G:p.N558S,5830411N06Rik:NM_001128146:exon10:c.A2021G: | . | 7qF4 | rs33807035 | 0 | 1 |
| chr7 | 140297270 | G | C | 222 | 5830411N06Rik | exonic | missense SNV | 5830411N06Rik:NM_001128145:exon8:c.G2149C:p.V717L,5830411N06Rik:NM_001128146:exon11:c.G2497C: | . | 7qF4 | rs33807041 | 0 | 1 |
| chr7 | 140297418 | G | A | 222 | 5830411N06Rik | UTR3 | . | . | . | 7qF4 | rs33807817 | 0 | 1 |
| chr7 | 140298293 | G | A | 222 | 5830411N06Rik | intronic | . | . | . | 7qF4 | rs33809565 | 0 | 1 |
| chr7 | 140298927 | C | T | 168 | 5830411N06Rik | intronic | . | . | . | 7qF4 | . | 0 | 1 |
| chr7 | 140315755 | C | T | 186 | . | intergenic | . | . | . | 7qF4 | rs33808682 | 0 | 1 |
| chr7 | 140316217 | C | T | 93 | . | intergenic | . | . | . | 7qF4 | rs33809718 | 0 | 1 |
| chr7 | 140322602 | C | T | 125 | . | upstream | . | . | . | 7qF4 | rs225428775 | 0 | 1 |
| chr7 | 140322874 | C | T | 221 | Olfr525 | exonic | synonymous SNV | Olfr525:NM_146956:exon1:c.C174T:p.P58P | . | 7qF4 | rs585278092 | 0 | 1 |
| chr7 | 140322976 | T | C | 222 | Olfr525 | exonic | synonymous SNV | Olfr525:NM_146956:exon1:c.T276C:p.I92I | . | 7qF4 | rs220082818 | 0 | 1 |
| chr7 | 140322985 | G | A | 222 | Olfr525 | exonic | synonymous SNV | Olfr525:NM_146956:exon1:c.G285A:p.K95K | . | 7qF4 | rs238658766 | 0 | 1 |
| chr7 | 140323066 | C | A | 220 | Olfr525 | exonic | synonymous SNV | Olfr525:NM_146956:exon1:c.C366A:p.R122R | . | 7qF4 | rs259770895 | 0 | 1 |
| chr7 | 140323646 | C | T | 221 | . | downstream | . | . | . | 7qF4 | . | 0 | 1 |
| chr7 | 140335850 | G | A | 222 | . | upstream | . | . | . | 7qF4 | rs236832719 | 0 | 1 |
| chr7 | 140336124 | G | A | 222 | Olfr527 | exonic | synonymous SNV | Olfr527:NM_001011776:exon1:c.G261A:p.R87R | . | 7qF4 | rs33807710 | 0 | 1 |
| chr7 | 140336541 | G | C | 222 | Olfr527 | exonic | synonymous SNV | Olfr527:NM_001011776:exon1:c.G678C:p.R226R | . | 7qF4 | rs586139199 | 0 | 1 |
| chr7 | 140336562 | A | G | 222 | Olfr527 | exonic | synonymous SNV | Olfr527:NM_001011776:exon1:c.A699G:p.R233R | . | 7qF4 | rs580061027 | 0 | 1 |
| chr7 | 140336589 | T | C | 222 | Olfr527 | exonic | synonymous SNV | Olfr527:NM_001011776:exon1:c.T726C:p.L242L | . | 7qF4 | rs584084169 | 0 | 1 |
| chr7 | 140336637 | C | T | 222 | Olfr527 | exonic | synonymous SNV | Olfr527:NM_001011776:exon1:c.C774T:p.R258R | . | 7qF4 | rs33809608 | 0 | 1 |
| chr7 | 140336807 | G | T | 222 | . | downstream | . | . | . | 7qF4 | . | 0 | 1 |
| chr7 | 140336810 | A | C | 222 | . | downstream | . | . | . | 7qF4 | rs235161156 | 0 | 1 |
| chr7 | 140336874 | T | G | 181 | . | downstream | . | . | . | 7qF4 | . | 0 | 1 |
| chr7 | 140344959 | G | A | 34.0777 | . | downstream | . | . | . | 7qF4 | rs33805642 | 0 | 1 |
| chr7 | 140344983 | G | A | 52 | . | downstream | . | . | . | 7qF4 | rs227896897 | 0 | 1 |
| chr7 | 140344988 | T | G | 51 | . | downstream | . | . | . | 7qF4 | rs252711133 | 0 | 1 |
| chr7 | 140345021 | C | A | 117 | . | downstream | . | . | . | 7qF4 | rs33806325 | 0 | 1 |
| chr7 | 140345111 | A | G | 142 | Olfr60 | exonic | synonymous SNV | Olfr60:NM_146955:exon1:c.T877C:p.L293L | . | 7qF4 | rs221701290 | 0 | 1 |
| chr7 | 140345156 | A | G | 144 | Olfr60 | exonic | synonymous SNV | Olfr60:NM_146955:exon1:c.T832C:p.L278L | . | 7qF4 | rs240454629 | 0 | 1 |
| chr7 | 140345160 | A | T | 148 | Olfr60 | exonic | synonymous SNV | Olfr60:NM_146955:exon1:c.T828A:p.S276S | . | 7qF4 | rs260884072 | 0 | 1 |
| chr7 | 140345187 | A | G | 221 | Olfr60 | exonic | synonymous SNV | Olfr60:NM_146955:exon1:c.T801C:p.Y267Y | . | 7qF4 | rs227210928 | 0 | 1 |
| chr7 | 140345196 | T | A | 221 | Olfr60 | exonic | synonymous SNV | Olfr60:NM_146955:exon1:c.A792T:p.A264A | . | 7qF4 | rs247508701 | 0 | 1 |
| chr7 | 140345406 | G | A | 222 | Olfr60 | exonic | synonymous SNV | Olfr60:NM_146955:exon1:c.C582T:p.Y194Y | . | 7qF4 | rs223641497 | 0 | 1 |
| chr7 | 140345569 | T | G | 222 | Olfr60 | exonic | missense SNV | Olfr60:NM_146955:exon1:c.A419C:p.Q140P | . | 7qF4 | rs219732856 | 0 | 1 |
| chr7 | 140345900 | C | A | 222 | Olfr60 | exonic | missense SNV | Olfr60:NM_146955:exon1:c.G88T:p.G30C | . | 7qF4 | . | 0 | 1 |
| chr7 | 140372638 | A | C | 117 | . | downstream | . | . | . | 7qF4 | rs33811618 | 0 | 1 |
| chr7 | 140372693 | G | A | 179 | Olfr530 | exonic | missense SNV | Olfr530:NM_146519:exon1:c.C916T:p.P306S | . | 7qF4 | rs253668219 | 0 | 1 |

| | | | | | | | | | | | | | |
|---|---|---|---|---|---|---|---|---|---|---|---|---|---|
| chr7 | 140372716 | A | G | 222 | Olfr530 | exonic | missense SNV | Olfr530:NM_146519:exon1:c.T893C:p.I298T | . | 7qF4 | rs212983130 | 0 | 1 |
| chr7 | 140372910 | T | C | 222 | Olfr530 | exonic | synonymous SNV | Olfr530:NM_146519:exon1:c.A699G:p.K233K | . | 7qF4 | rs583434785 | 0 | 1 |
| chr7 | 140373027 | A | G | 222 | Olfr530 | exonic | synonymous SNV | Olfr530:NM_146519:exon1:c.T582C:p.N194N | . | 7qF4 | rs245578966 | 0 | 1 |
| chr7 | 140373213 | G | A | 222 | Olfr530 | exonic | synonymous SNV | Olfr530:NM_146519:exon1:c.C396T:p.G132G | . | 7qF4 | rs584897793 | 0 | 1 |
| chr7 | 140373306 | G | A | 222 | Olfr530 | exonic | synonymous SNV | Olfr530:NM_146519:exon1:c.C303T:p.F101F | . | 7qF4 | rs580881712 | 0 | 1 |
| chr7 | 140373324 | T | C | 222 | Olfr530 | exonic | synonymous SNV | Olfr530:NM_146519:exon1:c.A285G:p.G95G | . | 7qF4 | rs584421281 | 0 | 1 |
| chr7 | 140373420 | A | T | 222 | Olfr530 | exonic | synonymous SNV | Olfr530:NM_146519:exon1:c.T189A:p.L63L | . | 7qF4 | rs33812414 | 0 | 1 |
| chr7 | 140373461 | G | A | 222 | Olfr530 | exonic | missense SNV | Olfr530:NM_146519:exon1:c.C148T:p.H50Y | . | 7qF4 | rs221248366 | 0 | 1 |
| chr7 | 140385966 | A | G | 65 | . | intergenic | . | . | . | 7qF4 | rs33807501 | 0 | 1 |
| chr7 | 140400082 | G | A | 175 | . | downstream | . | . | . | 7qF4 | rs214662652 | 0 | 1 |
| chr7 | 140400144 | A | G | 211 | Olfr531 | exonic | missense SNV | Olfr531:NM_146953:exon1:c.T901C:p.F301L | . | 7qF4 | rs33808083 | 0 | 1 |
| chr7 | 140400175 | A | G | 186 | Olfr531 | exonic | synonymous SNV | Olfr531:NM_146953:exon1:c.T870C:p.N290N | . | 7qF4 | rs33808926 | 0 | 1 |
| chr7 | 140400184 | A | G | 187 | Olfr531 | exonic | synonymous SNV | Olfr531:NM_146953:exon1:c.T861C:p.T287T | . | 7qF4 | rs224671198 | 0 | 1 |
| chr7 | 140400520 | A | G | 222 | Olfr531 | exonic | synonymous SNV | Olfr531:NM_146953:exon1:c.T525C:p.C175C | . | 7qF4 | rs219455632 | 0 | 1 |
| chr7 | 140400628 | A | G | 222 | Olfr531 | exonic | synonymous SNV | Olfr531:NM_146953:exon1:c.T417C:p.F139F | . | 7qF4 | rs33808932 | 0 | 1 |
| chr7 | 140400727 | C | T | 222 | Olfr531 | exonic | synonymous SNV | Olfr531:NM_146953:exon1:c.G318A:p.V106V | . | 7qF4 | rs265939699 | 0 | 1 |
| chr7 | 140400728 | A | G | 222 | Olfr531 | exonic | missense SNV | Olfr531:NM_146953:exon1:c.T317C:p.V106A | . | 7qF4 | rs228922574 | 0 | 1 |
| chr7 | 140400907 | G | A | 222 | Olfr531 | exonic | synonymous SNV | Olfr531:NM_146953:exon1:c.C138T:p.I46I | . | 7qF4 | rs231625562 | 0 | 1 |
| chr7 | 140400961 | G | A | 222 | Olfr531 | exonic | synonymous SNV | Olfr531:NM_146953:exon1:c.C84T:p.F28F | . | 7qF4 | rs33809855 | 0 | 1 |
| chr7 | 140418836 | G | A | 110 | . | downstream | . | . | . | 7qF4 | rs579016734 | 0 | 1 |
| chr7 | 140418896 | T | C | 218 | Olfr532 | exonic | synonymous SNV | Olfr532:NM_147026:exon1:c.A876G:p.L292L | . | 7qF4 | rs220829204 | 0 | 1 |
| chr7 | 140418947 | G | T | 191 | Olfr532 | exonic | synonymous SNV | Olfr532:NM_147026:exon1:c.C825A:p.T275T | . | 7qF4 | rs248517518 | 0 | 1 |
| chr7 | 140418992 | T | C | 68 | Olfr532 | exonic | synonymous SNV | Olfr532:NM_147026:exon1:c.A780G:p.L260L | . | 7qF4 | rs582646327 | 0 | 1 |
| chr7 | 140419181 | A | G | 37.8057 | Olfr532 | exonic | synonymous SNV | Olfr532:NM_147026:exon1:c.T591C:p.I197I | . | 7qF4 | rs234650037 | 0 | 1 |
| chr7 | 140419280 | C | A | 207 | Olfr532 | exonic | synonymous SNV | Olfr532:NM_147026:exon1:c.G492T:p.V164V | . | 7qF4 | rs213020088 | 0 | 1 |
| chr7 | 140419340 | A | C | 128 | Olfr532 | exonic | synonymous SNV | Olfr532:NM_147026:exon1:c.T432G:p.L144L | . | 7qF4 | rs213541311 | 0 | 1 |
| chr7 | 140419348 | C | T | 84 | Olfr532 | exonic | missense SNV | Olfr532:NM_147026:exon1:c.G424A:p.V142I | . | 7qF4 | rs233512667 | 0 | 1 |
| chr7 | 140419558 | T | C | 165 | Olfr532 | exonic | missense SNV | Olfr532:NM_147026:exon1:c.A214G:p.I72V | . | 7qF4 | rs579006868 | 0 | 1 |
| chr7 | 140419664 | G | A | 125 | Olfr532 | exonic | synonymous SNV | Olfr532:NM_147026:exon1:c.C108T:p.I36I | . | 7qF4 | rs581214696 | 0 | 1 |
| chr7 | 140419673 | G | A | 87 | Olfr532 | exonic | synonymous SNV | Olfr532:NM_147026:exon1:c.C99T:p.L33L | . | 7qF4 | rs585307068 | 0 | 1 |
| chr7 | 140419679 | G | A | 76 | Olfr532 | exonic | synonymous SNV | Olfr532:NM_147026:exon1:c.C93T:p.F31F | . | 7qF4 | rs579374286 | 0 | 1 |
| chr7 | 140466074 | A | G | 119 | . | upstream | . | . | . | 7qF4 | rs249958048 | 0 | 1 |
| chr7 | 140466439 | T | C | 222 | Olfr533 | exonic | synonymous SNV | Olfr533:NM_001011815:exon1:c.T237C:p.P79P | . | 7qF4 | rs33807738 | 0 | 1 |
| chr7 | 140466879 | G | A | 222 | Olfr533 | exonic | missense SNV | Olfr533:NM_001011815:exon1:c.G677A:p.R226H | . | 7qF4 | rs584318366 | 0 | 1 |
| chr7 | 140466884 | C | T | 222 | Olfr533 | exonic | missense SNV | Olfr533:NM_001011815:exon1:c.C682T:p.R228C | . | 7qF4 | . | 0 | 1 |
| chr7 | 140467057 | C | T | 222 | Olfr533 | exonic | synonymous SNV | Olfr533:NM_001011815:exon1:c.C855T:p.N285N | . | 7qF4 | rs260047163 | 0 | 1 |
| chr7 | 140467120 | C | T | 160 | Olfr533 | exonic | synonymous SNV | Olfr533:NM_001011815:exon1:c.C918T:p.L306L | . | 7qF4 | rs33808652 | 0 | 1 |
| chr7 | 140467161 | G | A | 100 | Olfr533 | exonic | missense SNV | Olfr533:NM_001011815:exon1:c.G959A:p.G320E | . | 7qF4 | rs33809664 | 0 | 1 |
| chr7 | 140467173 | A | C | 31.3736 | . | downstream | . | . | . | 7qF4 | rs232267336 | 0 | 1 |
| chr7 | 140467177 | A | C | 34.4708 | . | downstream | . | . | . | 7qF4 | rs249845063 | 0 | 1 |
| chr7 | 140492531 | G | A | 150 | . | upstream | . | . | . | 7qF4 | rs242056406 | 0 | 1 |
| chr7 | 140492595 | T | C | 218 | . | upstream | . | . | . | 7qF4 | rs33810254 | 0 | 1 |
| chr7 | 140492687 | G | A | 222 | Olfr535 | exonic | synonymous SNV | Olfr535:NM_146954:exon1:c.G48A:p.L16L | . | 7qF4 | rs33810256 | 0 | 1 |
| chr7 | 140492708 | T | G | 222 | Olfr535 | exonic | synonymous SNV | Olfr535:NM_146954:exon1:c.T69G:p.P23P | . | 7qF4 | rs238423684 | 0 | 1 |
| chr7 | 140492760 | C | T | 222 | Olfr535 | exonic | synonymous SNV | Olfr535:NM_146954:exon1:c.C121T:p.L41L | . | 7qF4 | rs33810259 | 0 | 1 |
| chr7 | 140492816 | T | C | 185 | Olfr535 | exonic | synonymous SNV | Olfr535:NM_146954:exon1:c.T177C:p.S59S | . | 7qF4 | rs227654742 | 0 | 1 |
| chr7 | 140492828 | T | C | 188 | Olfr535 | exonic | synonymous SNV | Olfr535:NM_146954:exon1:c.T189C:p.F63F | . | 7qF4 | rs245401383 | 0 | 1 |
| chr7 | 140492885 | G | A | 222 | Olfr535 | exonic | synonymous SNV | Olfr535:NM_146954:exon1:c.G246A:p.K82K | . | 7qF4 | rs234360861 | 0 | 1 |
| chr7 | 140492905 | T | C | 222 | Olfr535 | exonic | missense SNV | Olfr535:NM_146954:exon1:c.T266C:p.F89S | . | 7qF4 | rs33811184 | 0 | 1 |
| chr7 | 140493028 | A | G | 222 | Olfr535 | exonic | missense SNV | Olfr535:NM_146954:exon1:c.A389G:p.Y130C | . | 7qF4 | rs233367079 | 0 | 1 |
| chr7 | 140493095 | T | C | 222 | Olfr535 | exonic | synonymous SNV | Olfr535:NM_146954:exon1:c.T456C:p.S152S | . | 7qF4 | rs33811190 | 0 | 1 |
| chr7 | 140493188 | C | A | 222 | Olfr535 | exonic | synonymous SNV | Olfr535:NM_146954:exon1:c.C549A:p.I183I | . | 7qF4 | rs233470567 | 0 | 1 |
| chr7 | 140493313 | C | G | 222 | Olfr535 | exonic | missense SNV | Olfr535:NM_146954:exon1:c.C674G:p.A225G | . | 7qF4 | rs33812148 | 0 | 1 |
| chr7 | 140493423 | G | A | 217 | Olfr535 | exonic | missense SNV | Olfr535:NM_146954:exon1:c.G784A:p.V262I | . | 7qF4 | rs225575317 | 0 | 1 |
| chr7 | 140493428 | C | T | 222 | Olfr535 | exonic | synonymous SNV | Olfr535:NM_146954:exon1:c.C789T:p.S263S | . | 7qF4 | rs237106831 | 0 | 1 |
| chr7 | 140493437 | T | C | 222 | Olfr535 | exonic | synonymous SNV | Olfr535:NM_146954:exon1:c.T798C:p.S266S | . | 7qF4 | rs255410877 | 0 | 1 |
| chr7 | 140493571 | A | C | 173 | Olfr535 | exonic | missense SNV | Olfr535:NM_146954:exon1:c.A932C:p.Y311S | . | 7qF4 | rs33813107 | 0 | 1 |
| chr7 | 140503361 | T | G | 38.94 | Olfr536 | UTR3 | . | . | . | 7qF4 | rs33812744 | 0 | 1 |
| chr7 | 140503406 | T | A | 80 | Olfr536 | UTR3 | . | . | . | 7qF4 | rs223183254 | 0 | 1 |
| chr7 | 140503425 | T | C | 108 | Olfr536 | UTR3 | . | . | . | 7qF4 | rs235326637 | 0 | 1 |
| chr7 | 140503539 | G | A | 222 | Olfr536 | exonic | missense SNV | Olfr536:NM_146520:exon4:c.C919T:p.R307C | . | 7qF4 | . | 0 | 1 |
| chr7 | 140503624 | T | G | 222 | Olfr536 | exonic | synonymous SNV | Olfr536:NM_146520:exon4:c.A834C:p.T278T | . | 7qF4 | rs265063898 | 0 | 1 |
| chr7 | 140503678 | T | C | 145 | Olfr536 | exonic | synonymous SNV | Olfr536:NM_146520:exon4:c.A780G:p.R260R | . | 7qF4 | rs249179314 | 0 | 1 |
| chr7 | 140503681 | G | A | 151 | Olfr536 | exonic | synonymous SNV | Olfr536:NM_146520:exon4:c.C777T:p.V259V | . | 7qF4 | rs263199259 | 0 | 1 |
| chr7 | 140503738 | G | A | 221 | Olfr536 | exonic | synonymous SNV | Olfr536:NM_146520:exon4:c.C720T:p.C240C | . | 7qF4 | rs229317997 | 0 | 1 |
| chr7 | 140503759 | C | T | 221 | Olfr536 | exonic | synonymous SNV | Olfr536:NM_146520:exon4:c.G699A:p.K233K | . | 7qF4 | rs584465830 | 0 | 1 |
| chr7 | 140503885 | G | A | 222 | Olfr536 | exonic | synonymous SNV | Olfr536:NM_146520:exon4:c.C573T:p.T191T | . | 7qF4 | rs223723110 | 0 | 1 |
| chr7 | 140503894 | G | A | 222 | Olfr536 | exonic | synonymous SNV | Olfr536:NM_146520:exon4:c.C564T:p.C188C | . | 7qF4 | rs33812749 | 0 | 1 |
| chr7 | 140503909 | T | C | 222 | Olfr536 | exonic | synonymous SNV | Olfr536:NM_146520:exon4:c.A549G:p.V183V | . | 7qF4 | rs214620341 | 0 | 1 |
| chr7 | 140504173 | T | C | 222 | Olfr536 | exonic | synonymous SNV | Olfr536:NM_146520:exon4:c.A285G:p.G95G | . | 7qF4 | rs581143296 | 0 | 1 |
| chr7 | 140504176 | T | C | 222 | Olfr536 | exonic | synonymous SNV | Olfr536:NM_146520:exon4:c.A282G:p.G94G | . | 7qF4 | rs578610703 | 0 | 1 |
| chr7 | 140504311 | A | G | 107 | Olfr536 | exonic | synonymous SNV | Olfr536:NM_146520:exon4:c.T147C:p.I49I | . | 7qF4 | rs217059224 | 0 | 1 |
| chr7 | 140504317 | G | C | 45.0322 | Olfr536 | exonic | synonymous SNV | Olfr536:NM_146520:exon4:c.C141G:p.A47A | . | 7qF4 | rs33813854 | 0 | 1 |
| chr7 | 140504352 | T | C | 35.5891 | Olfr536 | exonic | missense SNV | Olfr536:NM_146520:exon4:c.A106G:p.M36V | . | 7qF4 | rs33813857 | 0 | 1 |
| chr7 | 140504705 | C | G | 222 | Olfr536 | intronic | . | . | . | 7qF4 | rs33814746 | 0 | 1 |
| chr7 | 140504717 | G | A | 222 | Olfr536 | intronic | . | . | . | 7qF4 | rs254758489 | 0 | 1 |
| chr7 | 140637690 | C | G | 222 | . | upstream | . | . | . | 7qF4 | rs50715966 | 0 | 1 |
| chr7 | 140637843 | T | C | 222 | Olfr61 | exonic | synonymous SNV | Olfr61:NM_146964:exon1:c.T141C:p.I47I | . | 7qF4 | rs51864101 | 0 | 1 |
| chr7 | 140637906 | C | T | 222 | Olfr61 | exonic | synonymous SNV | Olfr61:NM_146964:exon1:c.C204T:p.A68A | . | 7qF4 | rs243287248 | 0 | 1 |
| chr7 | 140637969 | A | G | 222 | Olfr61 | exonic | synonymous SNV | Olfr61:NM_146964:exon1:c.A267G:p.E89E | . | 7qF4 | rs231868366 | 0 | 1 |
| chr7 | 140638035 | G | A | 222 | Olfr61 | exonic | synonymous SNV | Olfr61:NM_146964:exon1:c.G333A:p.S111S | . | 7qF4 | rs47559877 | 0 | 1 |
| chr7 | 140638179 | G | A | 222 | Olfr61 | exonic | synonymous SNV | Olfr61:NM_146964:exon1:c.G477A:p.V159V | . | 7qF4 | rs581444441 | 0 | 1 |
| chr7 | 140638188 | C | T | 221 | Olfr61 | exonic | synonymous SNV | Olfr61:NM_146964:exon1:c.C486T:p.G162G | . | 7qF4 | rs583703931 | 0 | 1 |
| chr7 | 140638215 | C | A | 169 | Olfr61 | exonic | synonymous SNV | Olfr61:NM_146964:exon1:c.C513A:p.G171G | . | 7qF4 | rs587532441 | 0 | 1 |
| chr7 | 140638248 | T | A | 104 | Olfr61 | exonic | synonymous SNV | Olfr61:NM_146964:exon1:c.T546A:p.I182I | . | 7qF4 | rs216999363 | 0 | 1 |
| chr7 | 140638266 | T | C | 111 | Olfr61 | exonic | synonymous SNV | Olfr61:NM_146964:exon1:c.T564C:p.L188L | . | 7qF4 | rs584440564 | 0 | 1 |
| chr7 | 140638326 | G | A | 197 | Olfr61 | exonic | synonymous SNV | Olfr61:NM_146964:exon1:c.G624A:p.G208G | . | 7qF4 | rs50218360 | 0 | 1 |
| chr7 | 140638377 | T | C | 212 | Olfr61 | exonic | synonymous SNV | Olfr61:NM_146964:exon1:c.T675C:p.S225S | . | 7qF4 | rs48455700 | 0 | 1 |
| chr7 | 140638380 | T | C | 210 | Olfr61 | exonic | synonymous SNV | Olfr61:NM_146964:exon1:c.T678C:p.V226V | . | 7qF4 | rs214050358 | 0 | 1 |
| chr7 | 140638461 | C | T | 222 | Olfr61 | exonic | synonymous SNV | Olfr61:NM_146964:exon1:c.C759T:p.Y253Y | . | 7qF4 | rs237656454 | 0 | 1 |
| chr7 | 140638500 | C | T | 222 | Olfr61 | exonic | synonymous SNV | Olfr61:NM_146964:exon1:c.C798T:p.S266S | . | 7qF4 | rs215918665 | 0 | 1 |
| chr7 | 140638625 | T | C | 222 | Olfr61 | exonic | missense SNV | Olfr61:NM_146964:exon1:c.T923C:p.F308S | . | 7qF4 | rs46556381 | 0 | 1 |
| chr7 | 140638638 | G | A | 222 | Olfr61 | exonic | synonymous SNV | Olfr61:NM_146964:exon1:c.G936A:p.X312X | . | 7qF4 | rs46329324 | 0 | 1 |
| chr7 | 140651826 | A | G | 63 | Olfr53 | intronic | . | . | . | 7qF4 | rs46513953 | 0 | 1 |
| chr7 | 140651863 | A | G | 115 | Olfr53 | intronic | . | . | . | 7qF4 | rs46828193 | 0 | 1 |
| chr7 | 140651909 | C | G | 215 | Olfr53 | UTR5 | . | . | . | 7qF4 | rs51445460 | 0 | 1 |
| chr7 | 140652263 | A | T | 222 | Olfr53 | exonic | missense SNV | Olfr53:NM_146960:exon2:c.A283T:p.T95S | . | 7qF4 | rs260260433 | 0 | 1 |
| chr7 | 140652281 | G | T | 222 | Olfr53 | exonic | missense SNV | Olfr53:NM_146960:exon2:c.G301T:p.A101S | . | 7qF4 | rs218164421 | 0 | 1 |
| chr7 | 140652313 | C | T | 221 | Olfr53 | exonic | synonymous SNV | Olfr53:NM_146960:exon2:c.C333T:p.S111S | . | 7qF4 | rs235535252 | 0 | 1 |
| chr7 | 140652361 | T | C | 222 | Olfr53 | exonic | synonymous SNV | Olfr53:NM_146960:exon2:c.T381C:p.A127A | . | 7qF4 | rs221271841 | 0 | 1 |
| chr7 | 140652430 | A | G | 222 | Olfr53 | exonic | synonymous SNV | Olfr53:NM_146960:exon2:c.A450G:p.V150V | . | 7qF4 | rs225091078 | 0 | 1 |
| chr7 | 140652527 | A | C | 222 | Olfr53 | exonic | missense SNV | Olfr53:NM_146960:exon2:c.A547C:p.I183L | . | 7qF4 | rs578787734 | 0 | 1 |
| chr7 | 140652662 | C | T | 222 | Olfr53 | exonic | synonymous SNV | Olfr53:NM_146960:exon2:c.C682T:p.L228L | . | 7qF4 | rs249208442 | 0 | 1 |
| chr7 | 140652730 | C | A | 222 | Olfr53 | exonic | synonymous SNV | Olfr53:NM_146960:exon2:c.C750A:p.V250V | . | 7qF4 | rs585656343 | 0 | 1 |
| chr7 | 140652733 | A | T | 222 | Olfr53 | exonic | synonymous SNV | Olfr53:NM_146960:exon2:c.A753T:p.S251S | . | 7qF4 | rs578971867 | 0 | 1 |
| chr7 | 140652827 | G | T | 222 | Olfr53 | exonic | missense SNV | Olfr53:NM_146960:exon2:c.G847T:p.V283F | . | 7qF4 | rs47259658 | 0 | 1 |
| chr7 | 140652898 | G | A | 222 | Olfr53 | exonic | missense SNV | Olfr53:NM_146960:exon2:c.G918A:p.M306I | . | 7qF4 | rs50190918 | 0 | 1 |
| chr7 | 140667261 | A | G | 222 | . | upstream | . | . | . | 7qF4 | rs47451532 | 0 | 1 |
| chr7 | 140667345 | A | G | 222 | Olfr539 | exonic | synonymous SNV | Olfr539:NM_146961:exon1:c.A57G:p.T19T | . | 7qF4 | rs225189137 | 0 | 1 |
| chr7 | 140667628 | A | G | 222 | Olfr539 | exonic | missense SNV | Olfr539:NM_146961:exon1:c.A340G:p.T114A | . | 7qF4 | rs256410403 | 0 | 1 |
| chr7 | 140668107 | T | C | 222 | Olfr539 | exonic | synonymous SNV | Olfr539:NM_146961:exon1:c.T819C:p.S273S | . | 7qF4 | rs224250656 | 0 | 1 |
| chr7 | 140690820 | A | G | 123 | . | upstream | . | . | . | 7qF4 | rs51871031 | 0 | 1 |
| chr7 | 140690844 | A | T | 190 | . | upstream | . | . | . | 7qF4 | rs50499389 | 0 | 1 |
| chr7 | 140690857 | C | T | 211 | . | upstream | . | . | . | 7qF4 | rs52018467 | 0 | 1 |
| chr7 | 140690974 | A | G | 222 | Olfr45 | exonic | missense SNV | Olfr45:NM_146963:exon1:c.A68G:p.D23G | . | 7qF4 | rs245262272 | 0 | 1 |
| chr7 | 140691017 | A | G | 222 | Olfr45 | exonic | missense SNV | Olfr45:NM_146963:exon1:c.A111G:p.I37M | . | 7qF4 | rs50048701 | 0 | 1 |
| chr7 | 140691182 | T | C | 222 | Olfr45 | exonic | synonymous SNV | Olfr45:NM_146963:exon1:c.T276C:p.T92T | . | 7qF4 | rs585340509 | 0 | 1 |
| chr7 | 140691299 | G | A | 222 | Olfr45 | exonic | synonymous SNV | Olfr45:NM_146963:exon1:c.G393A:p.L131L | . | 7qF4 | rs583905909 | 0 | 1 |
| chr7 | 140691327 | A | C | 222 | Olfr45 | exonic | missense SNV | Olfr45:NM_146963:exon1:c.A421C:p.M141L | . | 7qF4 | rs219874035 | 0 | 1 |
| chr7 | 140691588 | C | T | 222 | Olfr45 | exonic | missense SNV | Olfr45:NM_146963:exon1:c.C682T:p.R228C | . | 7qF4 | rs51929222 | 0 | 1 |
| chr7 | 140691620 | C | A | 195 | Olfr45 | exonic | synonymous SNV | Olfr45:NM_146963:exon1:c.C714A:p.A238A | . | 7qF4 | rs586498206 | 0 | 1 |
| chr7 | 140691692 | C | T | 56 | Olfr45 | exonic | synonymous SNV | Olfr45:NM_146963:exon1:c.C786T:p.S262S | . | 7qF4 | rs232553134 | 0 | 1 |
| chr7 | 140691697 | C | G | 67 | Olfr45 | exonic | missense SNV | Olfr45:NM_146963:exon1:c.C791G:p.A264G | . | 7qF4 | rs255114869 | 0 | 1 |
| chr7 | 140691710 | T | C | 153 | Olfr45 | exonic | synonymous SNV | Olfr45:NM_146963:exon1:c.T804C:p.S268S | . | 7qF4 | rs217705088 | 0 | 1 |
| chr7 | 140691935 | C | T | 222 | . | downstream | . | . | . | 7qF4 | rs214149768 | 0 | 1 |
| chr7 | 140691944 | G | A | 222 | . | downstream | . | . | . | 7qF4 | rs231236213 | 0 | 1 |
| chr7 | 140691975 | A | C | 222 | . | downstream | . | . | . | 7qF4 | rs49228937 | 0 | 1 |
| chr7 | 140704192 | C | G | 222 | . | upstream | . | . | . | 7qF4 | rs49449416 | 0 | 1 |
| chr7 | 140704200 | C | T | 222 | . | upstream | . | . | . | 7qF4 | rs239093716 | 0 | 1 |
| chr7 | 140704426 | T | C | 222 | Olfr541 | exonic | synonymous SNV | Olfr541:NM_146962:exon1:c.T174C:p.H58H | . | 7qF4 | rs246686408 | 0 | 1 |
| chr7 | 140704505 | G | A | 222 | Olfr541 | exonic | missense SNV | Olfr541:NM_146962:exon1:c.G253A:p.V85I | . | 7qF4 | rs262320952 | 0 | 1 |
| chr7 | 140704606 | G | A | 222 | Olfr541 | exonic | synonymous SNV | Olfr541:NM_146962:exon1:c.G354A:p.T118T | . | 7qF4 | rs49623152 | 0 | 1 |
| chr7 | 140704702 | G | A | 222 | Olfr541 | exonic | synonymous SNV | Olfr541:NM_146962:exon1:c.G450A:p.V150V | . | 7qF4 | rs252285067 | 0 | 1 |
| chr7 | 140704768 | A | C | 222 | Olfr541 | exonic | synonymous SNV | Olfr541:NM_146962:exon1:c.A516C:p.G172G | . | 7qF4 | rs583313105 | 0 | 1 |
| chr7 | 140704775 | A | G | 222 | Olfr541 | exonic | missense SNV | Olfr541:NM_146962:exon1:c.A523G:p.I175V | . | 7qF4 | rs46775196 | 0 | 1 |
| chr7 | 140704906 | G | A | 221 | Olfr541 | exonic | synonymous SNV | Olfr541:NM_146962:exon1:c.G654A:p.L218L | . | 7qF4 | rs45947363 | 0 | 1 |
| chr7 | 140705095 | A | G | 74 | Olfr541 | exonic | synonymous SNV | Olfr541:NM_146962:exon1:c.A843G:p.S281S | . | 7qF4 | rs234875629 | 0 | 1 |
| chr7 | 140705104 | C | T | 144 | Olfr541 | exonic | synonymous SNV | Olfr541:NM_146962:exon1:c.C852T:p.S284S | . | 7qF4 | rs256581419 | 0 | 1 |
| chr7 | 140705211 | C | T | 222 | . | downstream | . | . | . | 7qF4 | rs51825597 | 0 | 1 |
| chr7 | 140705276 | C | T | 222 | . | downstream | . | . | . | 7qF4 | rs45867565 | 0 | 1 |
| chr7 | 140764982 | A | T | 222 | Cyp2e1 | intronic | . | . | . | 7qF4 | rs264497103 | 0 | 1 |
| chr7 | 140765010 | G | A | 222 | Cyp2e1 | intronic | . | . | . | 7qF4 | rs243293876 | 0 | 1 |
| chr7 | 140765075 | A | G | 160 | Cyp2e1 | intronic | . | . | . | 7qF4 | rs212357303 | 0 | 1 |
| chr7 | 140769762 | C | T | 222 | Cyp2e1 | intronic | . | . | . | 7qF4 | rs52008665 | 0 | 1 |
| chr7 | 140769940 | C | G | 179 | Cyp2e1 | intronic | . | . | . | 7qF4 | rs50071630 | 0 | 1 |
| chr7 | 140770084 | T | C | 222 | Cyp2e1 | exonic | synonymous SNV | Cyp2e1:NM_021282:exon5:c.T681C:p.Y227Y | . | 7qF4 | rs49540258 | 0 | 1 |
| chr7 | 140770858 | G | A | 178 | Cyp2e1 | intronic | . | . | . | 7qF4 | rs50624744 | 0 | 1 |
| chr7 | 140771174 | A | G | 208 | Cyp2e1 | intronic | . | . | . | 7qF4 | rs8268063 | 0 | 1 |
| chr7 | 140771242 | T | A | 51 | Cyp2e1 | intronic | . | . | . | 7qF4 | rs8268064 | 0 | 1 |
| chr7 | 140771298 | G | T | 51 | Cyp2e1 | intronic | . | . | . | 7qF4 | rs8268065 | 0 | 1 |
| chr7 | 140772963 | G | A | 158 | Cyp2e1 | intronic | . | . | . | 7qF4 | rs8268009 | 0 | 1 |
| chr7 | 140772966 | T | C | 159 | Cyp2e1 | intronic | . | . | . | 7qF4 | rs239683247 | 0 | 1 |
| chr7 | 140773194 | T | C | 222 | Cyp2e1 | exonic | synonymous SNV | Cyp2e1:NM_021282:exon7:c.T1143C:p.Y381Y | . | 7qF4 | rs45817143 | 0 | 1 |
| chr7 | 140773257 | C | T | 169 | Cyp2e1 | intronic | . | . | . | 7qF4 | rs50944624 | 0 | 1 |
| chr7 | 140773261 | G | T | 177 | Cyp2e1 | intronic | . | . | . | 7qF4 | rs8268012 | 0 | 1 |
| chr7 | 140773620 | A | C | 202 | Cyp2e1 | intronic | . | . | . | 7qF4 | rs8268014 | 0 | 1 |
| chr7 | 140773698 | A | G | 222 | Cyp2e1 | exonic | synonymous SNV | Cyp2e1:NM_021282:exon8:c.A1224G:p.T408T | . | 7qF4 | rs46993892 | 0 | 1 |
| chr7 | 140773761 | G | A | 222 | Cyp2e1 | exonic | synonymous SNV | Cyp2e1:NM_021282:exon8:c.G1287A:p.A429A | . | 7qF4 | rs48539141 | 0 | 1 |
| chr7 | 140773877 | G | A | 168 | Cyp2e1 | intronic | . | . | . | 7qF4 | rs8268021 | 0 | 1 |
| chr7 | 140773908 | A | G | 117 | Cyp2e1 | intronic | . | . | . | 7qF4 | rs50308420 | 0 | 1 |
| chr7 | 140774452 | A | G | 54 | Cyp2e1 | intronic | . | . | . | 7qF4 | rs50928389 | 0 | 1 |
| chr7 | 140774503 | G | A | 108 | Cyp2e1 | intronic | . | . | . | 7qF4 | rs49691253 | 0 | 1 |
| chr7 | 140774583 | C | T | 187 | Cyp2e1 | exonic | synonymous SNV | Cyp2e1:NM_021282:exon9:c.C1323T:p.G441G | . | 7qF4 | rs8268023 | 0 | 1 |
| chr7 | 140777474 | T | C | 124 | Syce1 | UTR3 | . | . | . | 7qF4 | rs47101030 | 0 | 1 |
| chr7 | 140777774 | C | A | 222 | Syce1 | intronic | . | . | . | 7qF4 | rs254838704 | 0 | 1 |
| chr7 | 140777780 | T | C | 222 | Syce1 | intronic | . | . | . | 7qF4 | rs221490904 | 0 | 1 |
| chr7 | 140777948 | G | A | 222 | Syce1 | exonic | missense SNV | Syce1:NM_001143765:exon12:c.C887T:p.T296M | . | 7qF4 | rs49748317 | 0 | 1 |
| chr7 | 140778958 | T | C | 150 | Syce1 | intronic | . | . | . | 7qF4 | rs48330367 | 0 | 1 |
| chr7 | 140779107 | C | T | 222 | Syce1 | exonic | synonymous SNV | Syce1:NM_001143765:exon8:c.G492A:p.L164L | . | 7qF4 | rs47871406 | 0 | 1 |
| chr7 | 140779188 | T | C | 129 | Syce1 | intronic | . | . | . | 7qF4 | rs51255050 | 0 | 1 |
| chr7 | 140779207 | A | G | 100 | Syce1 | intronic | . | . | . | 7qF4 | rs50725158 | 0 | 1 |
| chr7 | 140779239 | G | C | 122 | Syce1 | intronic | . | . | . | 7qF4 | rs46112094 | 0 | 1 |
| chr7 | 140779310 | G | T | 222 | Syce1 | intronic | . | . | . | 7qF4 | . | 0 | 1 |
| chr7 | 140779569 | A | G | 222 | Syce1 | intronic | . | . | . | 7qF4 | rs45890343 | 0 | 1 |
| chr7 | 140779576 | G | A | 222 | Syce1 | intronic | . | . | . | 7qF4 | rs250967793 | 0 | 1 |
| chr7 | 140779899 | A | G | 222 | Syce1 | exonic | synonymous SNV | Syce1:NM_001143765:exon5:c.T309C:p.I103I | . | 7qF4 | rs50329206 | 0 | 1 |
| chr7 | 140780305 | G | A | 103 | Syce1 | intronic | . | . | . | 7qF4 | rs47210273 | 0 | 1 |
| chr7 | 140780872 | C | T | 222 | Syce1 | splicing | . | . | . | 7qF4 | rs46635279 | 0 | 1 |
| chr7 | 140781078 | A | G | 74 | Syce1 | intronic | . | . | . | 7qF4 | rs50353014 | 0 | 1 |
| chr7 | 140782130 | A | C | 222 | Syce1 | intronic | . | . | . | 7qF4 | rs47523083 | 0 | 1 |
| chr7 | 140782221 | A | G | 115 | Syce1 | intronic | . | . | . | 7qF4 | rs49368908 | 0 | 1 |
| chr7 | 140782278 | A | G | 35.8568 | Syce1 | intronic | . | . | . | 7qF4 | rs45936441 | 0 | 1 |
| chr7 | 140807987 | T | G | 222 | . | intergenic | . | . | . | 7qF4 | rs46601486 | 0 | 1 |
| chr7 | 140808183 | C | T | 222 | . | intergenic | . | . | . | 7qF4 | rs50777418 | 0 | 1 |
| chr7 | 140808204 | A | G | 222 | . | intergenic | . | . | . | 7qF4 | rs50565456 | 0 | 1 |

| | | | | | | | | | | | | | |
|---|---|---|---|---|---|---|---|---|---|---|---|---|---|
| chr7 | 140812445 | G | T | 24.3493 | Zfp941 | exonic | missense SNV | Zfp941:NM_001001180:exon3:c.C1000A:p.L334I | . | 7qF4 | rs51741670 | 0 | 1 |
| chr7 | 140812519 | A | G | 26.6023 | Zfp941 | exonic | missense SNV | Zfp941:NM_001001180:exon3:c.T926C:p.L309P | . | 7qF4 | rs47550655 | 0 | 1 |
| chr7 | 140812533 | C | A | 30.5207 | Zfp941 | exonic | missense SNV | Zfp941:NM_001001180:exon3:c.G912T:p.K304N | . | 7qF4 | rs231824710 | 0 | 1 |
| chr7 | 140812694 | A | G | 124 | Zfp941 | exonic | missense SNV | Zfp941:NM_001001180:exon3:c.T751C:p.W251R | . | 7qF4 | rs255942145 | 0 | 1 |
| chr7 | 140812698 | C | T | 130 | Zfp941 | exonic | synonymous SNV | Zfp941:NM_001001180:exon3:c.G747A:p.T249T | . | 7qF4 | rs224969666 | 0 | 1 |
| chr7 | 140812740 | T | C | 183 | Zfp941 | exonic | synonymous SNV | Zfp941:NM_001001180:exon3:c.A705G:p.K235K | . | 7qF4 | rs50913838 | 0 | 1 |
| chr7 | 140812941 | A | G | 222 | Zfp941 | exonic | synonymous SNV | Zfp941:NM_001001180:exon3:c.T504C:p.L168L | . | 7qF4 | rs46862552 | 0 | 1 |
| chr7 | 140812984 | A | G | 205 | Zfp941 | exonic | missense SNV | Zfp941:NM_001001180:exon3:c.T461C:p.F154S | . | 7qF4 | rs51256440 | 0 | 1 |
| chr7 | 140813040 | T | G | 210 | Zfp941 | exonic | missense SNV | Zfp941:NM_001001180:exon3:c.A405C:p.Q135H | . | 7qF4 | rs46712257 | 0 | 1 |
| chr7 | 140813055 | G | C | 222 | Zfp941 | exonic | synonymous SNV | Zfp941:NM_001001180:exon3:c.C390G:p.V130V | . | 7qF4 | rs47779080 | 0 | 1 |
| chr7 | 140813147 | T | C | 222 | Zfp941 | exonic | missense SNV | Zfp941:NM_001001180:exon3:c.A298G:p.R100G | . | 7qF4 | rs49654212 | 0 | 1 |
| chr7 | 140813468 | C | A | 20.3138 | Zfp941 | intronic | . | . | . | 7qF4 | rs51185139 | 0 | 1 |
| chr7 | 140818457 | A | G | 125 | Zfp941 | intronic | . | . | . | 7qF4 | rs45997346 | 0 | 1 |
| chr7 | 140818516 | G | A | 171 | Zfp941 | intronic | . | . | . | 7qF4 | rs256075655 | 0 | 1 |
| chr7 | 140818552 | C | T | 200 | Zfp941 | intronic | . | . | . | 7qF4 | rs46855932 | 0 | 1 |
| chr7 | 140818572 | G | A | 222 | Zfp941 | intronic | . | . | . | 7qF4 | rs258123309 | 0 | 1 |
| chr7 | 140897851 | A | C | 98 | Psmd13 | intronic | . | . | . | 7qF5 | . | 0 | 1 |
| chr7 | 141092105 | G | T | 168 | Sigirr | intronic | . | . | . | 7qF5 | . | 0 | 1 |
| chr7 | 141178297 | C | A | 169 | . | intergenic | . | . | . | 7qF5 | rs253436837 | 0 | 1 |
| chr7 | 141178369 | A | T | 32.9015 | . | intergenic | . | . | . | 7qF5 | rs237093406 | 0 | 1 |
| chr7 | 141213456 | G | A | 100 | Lmntd2 | intronic | . | . | . | 7qF5 | . | 0 | 1 |
| chr7 | 141216542 | G | C | 58 | Rassf7 | intronic | . | . | . | 7qF5 | . | 0 | 1 |
| chr7 | 141638476 | T | G | 222 | Muc6 | exonic | unknown | UNKNOWN | . | 7qF5 | rs51860151 | 0 | 1 |
| chr7 | 141638566 | C | T | 216 | Muc6 | exonic | unknown | UNKNOWN | . | 7qF5 | rs48692207 | 0 | 1 |
| chr7 | 141638584 | A | C | 44.0726 | Muc6 | exonic | unknown | UNKNOWN | . | 7qF5 | . | 0 | 1 |
| chr7 | 141638697 | G | T | 228 | Muc6 | exonic | unknown | UNKNOWN | . | 7qF5 | rs50243900 | 1 | 0 |
| chr7 | 141638789 | A | C | 222 | Muc6 | exonic | unknown | UNKNOWN | . | 7qF5 | rs45936581 | 0 | 1 |
| chr7 | 141638872 | G | A | 221 | Muc6 | exonic | unknown | UNKNOWN | . | 7qF5 | rs579103615 | 0 | 1 |
| chr7 | 141638890 | C | T | 221 | Muc6 | exonic | unknown | UNKNOWN | . | 7qF5 | rs585877881 | 0 | 1 |
| chr7 | 141638949 | G | A | 101 | Muc6 | exonic | unknown | UNKNOWN | . | 7qF5 | . | 0 | 1 |
| chr7 | 141639368 | C | G | 222 | Muc6 | exonic | unknown | UNKNOWN | . | 7qF5 | . | 0 | 1 |
| chr7 | 141639531 | G | A | 222 | Muc6 | exonic | unknown | UNKNOWN | . | 7qF5 | . | 0 | 1 |
| chr7 | 141701334 | A | C | 228 | Muc2 | splicing | . | . | . | 7qF5 | rs47009255 | 1 | 0 |
| chr7 | 141809744 | C | T | 179 | Muc5ac | intronic | . | . | . | 7qF5 | rs215979909 | 0 | 1 |
| chr7 | 141858445 | T | A | 222 | Muc5b | exonic | synonymous SNV | Muc5b:NM_028801:exon31:c.T5127A:p.S1709S | . | 7qF5 | rs51669259 | 0 | 1 |
| chr7 | 141858623 | T | C | 222 | Muc5b | exonic | missense SNV | Muc5b:NM_028801:exon31:c.T5305C:p.Y1769H | . | 7qF5 | rs37435548 | 0 | 1 |
| chr7 | 141858787 | C | T | 222 | Muc5b | exonic | synonymous SNV | Muc5b:NM_028801:exon31:c.C5469T:p.S1823S | . | 7qF5 | rs3142422 | 0 | 1 |
| chr7 | 141858900 | T | C | 222 | Muc5b | exonic | missense SNV | Muc5b:NM_028801:exon31:c.T5582C:p.F1861S | . | 7qF5 | rs3147780 | 0 | 1 |
| chr7 | 142540467 | G | A | 182 | Mrpl23 | intronic | . | . | . | 7qF5 | . | 0 | 1 |
| chr7 | 142850895 | G | T | 142 | . | intergenic | . | . | . | 7qF5 | rs33825402 | 0 | 1 |
| chr7 | 142851256 | G | A | 56 | . | intergenic | . | . | . | 7qF5 | rs217285973 | 0 | 1 |
| chr7 | 142893966 | T | A | 45.5375 | Th | intronic | . | . | . | 7qF5 | rs262591565 | 0 | 1 |
| chr7 | 142893972 | A | G | 50 | Th | intronic | . | . | . | 7qF5 | rs223622098 | 0 | 1 |
| chr7 | 142893980 | A | G | 50 | Th | intronic | . | . | . | 7qF5 | rs243575328 | 0 | 1 |
| chr7 | 142894196 | C | T | 87 | Th | intronic | . | . | . | 7qF5 | rs260221532 | 0 | 1 |
| chr7 | 142894197 | A | G | 96 | Th | intronic | . | . | . | 7qF5 | rs227623782 | 0 | 1 |
| chr7 | 142894530 | G | A | 221 | Th | intronic | . | . | . | 7qF5 | rs249010788 | 0 | 1 |
| chr7 | 142894551 | A | T | 220 | Th | intronic | . | . | . | 7qF5 | rs260772101 | 0 | 1 |
| chr7 | 142894653 | T | C | 215 | Th | exonic | synonymous SNV | Th:NM_009377:exon10:c.A1095G:p.E365E | . | 7qF5 | rs33822523 | 0 | 1 |
| chr7 | 142894733 | C | T | 221 | Th | intronic | . | . | . | 7qF5 | rs33823556 | 0 | 1 |
| chr7 | 142894820 | T | G | 134 | Th | intronic | . | . | . | 7qF5 | rs214804644 | 0 | 1 |
| chr7 | 142894834 | A | G | 63 | Th | intronic | . | . | . | 7qF5 | rs238070010 | 0 | 1 |
| chr7 | 142894899 | G | A | 94 | Th | intronic | . | . | . | 7qF5 | rs215102968 | 0 | 1 |
| chr7 | 142894915 | T | C | 109 | Th | intronic | . | . | . | 7qF5 | rs235063115 | 0 | 1 |
| chr7 | 142894966 | A | G | 185 | Th | intronic | . | . | . | 7qF5 | rs33823562 | 0 | 1 |
| chr7 | 142895275 | G | A | 222 | Th | intronic | . | . | . | 7qF5 | rs33824307 | 0 | 1 |
| chr7 | 142895534 | T | C | 222 | Th | exonic | missense SNV | Th:NM_009377:exon7:c.A833G:p.H278R | . | 7qF5 | rs262280270 | 0 | 1 |
| chr7 | 142895679 | C | T | 127 | Th | intronic | . | . | . | 7qF5 | rs222252477 | 0 | 1 |
| chr7 | 142896449 | G | A | 222 | Th | exonic | synonymous SNV | Th:NM_009377:exon5:c.C609T:p.R203R | . | 7qF5 | rs33824309 | 0 | 1 |
| chr7 | 142896829 | G | A | 64 | Th | intronic | . | . | . | 7qF5 | rs33824312 | 0 | 1 |
| chr7 | 142897948 | A | C | 155 | Th | intronic | . | . | . | 7qF5 | rs241387258 | 0 | 1 |
| chr7 | 142898143 | G | T | 183 | Th | exonic | synonymous SNV | Th:NM_009377:exon2:c.C138A:p.R46R | . | 7qF5 | rs265315210 | 0 | 1 |
| chr7 | 142899817 | T | C | 152 | Th | intronic | . | . | . | 7qF5 | rs257270330 | 0 | 1 |
| chr7 | 142899953 | G | A | 153 | Th | exonic | synonymous SNV | Th:NM_009377:exon1:c.C9T:p.T3T | . | 7qF5 | rs229228537 | 0 | 1 |
| chr7 | 142899999 | C | T | 68 | . | upstream | . | . | . | 7qF5 | rs246659222 | 0 | 1 |
| chr7 | 142930551 | G | C | 197 | . | intergenic | . | . | . | 7qF5 | rs6272473 | 0 | 1 |
| chr7 | 142967839 | T | C | 35.4493 | Ascl2 | intronic | . | . | 2006 | 7qF5 | rs230439511 | 0 | 1 |
| chr7 | 142967977 | C | G | 107 | Ascl2 | exonic | missense SNV | Ascl2:NM_008554:exon2:c.G733C:p.E245Q | 2006 | 7qF5 | . | 0 | 1 |
| chr7 | 142968578 | C | T | 73 | Ascl2 | exonic | synonymous SNV | Ascl2:NM_008554:exon2:c.G132A:p.P44P | 2006 | 7qF5 | rs33823696 | 0 | 1 |
| chr7 | 142989949 | G | A | 27.3874 | . | intergenic | . | . | . | 7qF5 | . | 0 | 1 |
| chr7 | 142990138 | G | A | 119 | . | intergenic | . | . | . | 7qF5 | rs223001443 | 0 | 1 |
| chr7 | 142990602 | A | G | 73 | . | intergenic | . | . | . | 7qF5 | rs33821997 | 0 | 1 |
| chr7 | 142990604 | A | G | 73 | . | intergenic | . | . | . | 7qF5 | rs241795075 | 0 | 1 |
| chr7 | 142990910 | T | C | 115 | . | intergenic | . | . | . | 7qF5 | rs33822002 | 0 | 1 |
| chr7 | 142990974 | A | G | 56 | . | intergenic | . | . | . | 7qF5 | rs33823015 | 0 | 1 |
| chr7 | 143005230 | T | G | 222 | Tspan32 | intronic | . | . | . | 7qF5 | . | 0 | 1 |
| chr7 | 143005838 | T | C | 107 | Tspan32 | UTR5 | . | . | . | 7qF5 | rs231618785 | 0 | 1 |
| chr7 | 143011094 | T | C | 63 | Tspan32 | exonic | missense SNV | Tspan32:NM_001128081:exon3:c.T259C:p.Y87H,Tspan32:NM_001128082:exon3:c.T259C:p.Y87H,Tspan32:N | . | 7qF5 | rs241217871 | 0 | 1 |
| chr7 | 143014970 | G | A | 222 | Tspan32 | exonic | synonymous SNV | Tspan32:NM_001128081:exon4:c.G285A:p.A95A,Tspan32:NM_020286:exon4:c.G285A:p.A95A,Tspan32:NM_( | . | 7qF5 | rs260194383 | 0 | 1 |
| chr7 | 143015046 | G | A | 222 | Tspan32 | exonic | missense SNV | Tspan32:NM_001128081:exon4:c.G361A:p.V121I,Tspan32:NM_020286:exon4:c.G361A:p.V121I,Tspan32:NM | . | 7qF5 | rs227124239 | 0 | 1 |
| chr7 | 143015207 | T | C | 72 | Tspan32 | intronic | . | . | . | 7qF5 | rs241201233 | 0 | 1 |
| chr7 | 143015411 | G | A | 70 | Tspan32 | intronic | . | . | . | 7qF5 | rs238519968 | 0 | 1 |
| chr7 | 143015507 | C | T | 155 | Tspan32 | intronic | . | . | . | 7qF5 | rs579392917 | 0 | 1 |
| chr7 | 143015546 | A | G | 215 | Tspan32 | intronic | . | . | . | 7qF5 | rs257436740 | 0 | 1 |
| chr7 | 143015691 | T | G | 218 | Tspan32 | intronic | . | . | . | 7qF5 | rs582549796 | 0 | 1 |
| chr7 | 143016811 | A | G | 53 | Tspan32 | intronic | . | . | . | 7qF5 | rs33828393 | 0 | 1 |
| chr7 | 143016820 | C | T | 44.1342 | Tspan32 | intronic | . | . | . | 7qF5 | rs231290634 | 0 | 1 |
| chr7 | 143017135 | A | G | 222 | Tspan32 | intronic | . | . | . | 7qF5 | rs587376611 | 0 | 1 |
| chr7 | 143017331 | T | C | 212 | Tspan32 | intronic | . | . | . | 7qF5 | rs214971449 | 0 | 1 |
| chr7 | 143017485 | T | C | 189 | Tspan32 | intronic | . | . | . | 7qF5 | rs238501413 | 0 | 1 |
| chr7 | 143017565 | T | C | 221 | Tspan32 | intronic | . | . | . | 7qF5 | rs33821045 | 0 | 1 |
| chr7 | 143017665 | A | G | 181 | Tspan32 | exonic | synonymous SNV | Tspan32:NM_001128082:exon5:c.A447G:p.T149T,Tspan32:NM_001128081:exon6:c.A549G:p.T183T,Tspan32 | . | 7qF5 | rs226601120 | 0 | 1 |
| chr7 | 143017672 | A | C | 170 | Tspan32 | exonic | synonymous SNV | Tspan32:NM_001128082:exon5:c.A454C:p.R152R,Tspan32:NM_001128081:exon6:c.A556C:p.R186R,Tspan32 | . | 7qF5 | rs236168660 | 0 | 1 |
| chr7 | 143018597 | T | G | 29.4923 | Tspan32 | intronic | . | . | . | 7qF5 | rs33821051 | 0 | 1 |
| chr7 | 143018763 | T | C | 170 | Tspan32 | UTR3 | . | . | . | 7qF5 | rs266209986 | 0 | 1 |
| chr7 | 143018812 | A | G | 122 | Tspan32 | UTR3 | . | . | . | 7qF5 | rs33822024 | 0 | 1 |
| chr7 | 143037121 | T | A | 99 | R74862 | ncRNA_intronic | . | . | . | 7qF5 | rs258503666 | 0 | 1 |
| chr7 | 143037150 | C | T | 126 | R74862 | ncRNA_intronic | . | . | . | 7qF5 | rs218377760 | 0 | 1 |
| chr7 | 143044414 | T | G | 134 | R74862 | ncRNA_intronic | . | . | . | 7qF5 | rs249465501 | 0 | 1 |
| chr7 | 143053242 | T | C | 153 | Cd81,R74862 | ncRNA_exonic | . | . | 614 | 7qF5 | rs238591442 | 0 | 1 |
| chr7 | 143065908 | T | C | 55 | Cd81 | intronic | . | . | . | 7qF5 | rs213684230 | 0 | 1 |
| chr7 | 143066025 | T | G | 222 | Cd81 | intronic | . | . | . | 7qF5 | rs237081166 | 0 | 1 |
| chr7 | 143066064 | C | G | 222 | Cd81 | intronic | . | . | . | 7qF5 | rs262970381 | 0 | 1 |
| chr7 | 143067128 | G | A | 222 | Cd81 | intronic | . | . | . | 7qF5 | rs225136778 | 0 | 1 |
| chr7 | 143070953 | G | A | 79 | Tssc4 | UTR3 | . | . | . | 7qF5 | rs239006176 | 0 | 1 |
| chr7 | 143072419 | A | G | 222 | Trpm5 | exonic | missense SNV | Trpm5:NM_020277:exon25:c.T3406C:p.C1136R | . | 7qF5 | rs248440224 | 0 | 1 |
| chr7 | 143072588 | C | T | 193 | Trpm5 | intronic | . | . | . | 7qF5 | rs263001170 | 0 | 1 |
| chr7 | 143072628 | C | T | 131 | Trpm5 | intronic | . | . | . | 7qF5 | rs224810136 | 0 | 1 |
| chr7 | 143072848 | C | G | 222 | Trpm5 | intronic | . | . | . | 7qF5 | rs240578484 | 0 | 1 |
| chr7 | 143072894 | C | T | 189 | Trpm5 | intronic | . | . | . | 7qF5 | rs259643211 | 0 | 1 |
| chr7 | 143073143 | T | C | 222 | Trpm5 | intronic | . | . | . | 7qF5 | rs223337870 | 0 | 1 |
| chr7 | 143073250 | T | C | 222 | Trpm5 | intronic | . | . | . | 7qF5 | rs242950944 | 0 | 1 |
| chr7 | 143073345 | C | T | 126 | Trpm5 | intronic | . | . | . | 7qF5 | rs255290911 | 0 | 1 |
| chr7 | 143073945 | C | T | 111 | Trpm5 | intronic | . | . | . | 7qF5 | rs216740621 | 0 | 1 |
| chr7 | 143074193 | G | A | 222 | Trpm5 | intronic | . | . | . | 7qF5 | rs242194697 | 0 | 1 |
| chr7 | 143074202 | A | G | 193 | Trpm5 | intronic | . | . | . | 7qF5 | rs248932958 | 0 | 1 |
| chr7 | 143074203 | C | T | 201 | Trpm5 | intronic | . | . | . | 7qF5 | rs212241029 | 0 | 1 |
| chr7 | 143074347 | C | T | 58 | Trpm5 | intronic | . | . | . | 7qF5 | rs230911238 | 0 | 1 |
| chr7 | 143074362 | T | C | 53 | Trpm5 | intronic | . | . | . | 7qF5 | rs251452726 | 0 | 1 |
| chr7 | 143074395 | G | T | 66 | Trpm5 | intronic | . | . | . | 7qF5 | rs223493119 | 0 | 1 |
| chr7 | 143074438 | A | G | 142 | Trpm5 | exonic | synonymous SNV | Trpm5:NM_020277:exon21:c.T3099C:p.H1033H | . | 7qF5 | rs33827624 | 0 | 1 |
| chr7 | 143074704 | G | C | 100 | Trpm5 | intronic | . | . | . | 7qF5 | rs263575590 | 0 | 1 |
| chr7 | 143075701 | A | C | 222 | Trpm5 | intronic | . | . | . | 7qF5 | rs236490436 | 0 | 1 |
| chr7 | 143078597 | T | C | 63 | Trpm5 | intronic | . | . | . | 7qF5 | rs240328988 | 0 | 1 |
| chr7 | 143080304 | G | T | 222 | Trpm5 | exonic | synonymous SNV | Trpm5:NM_020277:exon16:c.C2164A:p.R722R | . | 7qF5 | rs218566395 | 0 | 1 |
| chr7 | 143080839 | G | A | 192 | Trpm5 | intronic | . | . | . | 7qF5 | rs33829445 | 0 | 1 |
| chr7 | 143081709 | T | C | 94 | Trpm5 | intronic | . | . | . | 7qF5 | rs236422895 | 0 | 1 |
| chr7 | 143083398 | C | T | 81 | Trpm5 | intronic | . | . | . | 7qF5 | rs261892878 | 0 | 1 |
| chr7 | 143083402 | G | C | 81 | Trpm5 | intronic | . | . | . | 7qF5 | rs223195466 | 0 | 1 |
| chr7 | 143085625 | G | A | 88 | Trpm5 | intronic | . | . | . | 7qF5 | rs33821931 | 0 | 1 |
| chr7 | 143087528 | T | C | 112 | Trpm5 | intronic | . | . | . | 7qF5 | rs33823803 | 0 | 1 |
| chr7 | 143087850 | G | A | 222 | Trpm5 | exonic | missense SNV | Trpm5:NM_020277:exon4:c.C322T:p.L108F | . | 7qF5 | rs250840517 | 0 | 1 |
| chr7 | 143088963 | C | G | 122 | Trpm5 | exonic | synonymous SNV | Trpm5:NM_020277:exon3:c.G246C:p.S82S | . | 7qF5 | rs33824548 | 0 | 1 |
| chr7 | 143148642 | C | A | 53 | Kcnq1 | intronic | . | . | . | 7qF5 | rs240293385 | 0 | 1 |
| chr7 | 143148661 | G | A | 31.3927 | Kcnq1 | intronic | . | . | . | 7qF5 | rs266004773 | 0 | 1 |
| chr7 | 143148841 | C | T | 196 | Kcnq1 | intronic | . | . | . | 7qF5 | rs227116776 | 0 | 1 |
| chr7 | 143149459 | G | A | 96 | Kcnq1 | intronic | . | . | . | 7qF5 | rs253019165 | 0 | 1 |
| chr7 | 143149667 | C | T | 28.9571 | Kcnq1 | intronic | . | . | . | 7qF5 | rs217993625 | 0 | 1 |
| chr7 | 143182572 | A | G | 90 | Kcnq1 | intronic | . | . | . | 7qF5 | rs262693800 | 0 | 1 |
| chr7 | 143183041 | C | A | 222 | Kcnq1 | intronic | . | . | . | 7qF5 | rs231975766 | 0 | 1 |
| chr7 | 143184335 | T | C | 222 | Kcnq1 | intronic | . | . | . | 7qF5 | rs215974760 | 0 | 1 |
| chr7 | 143184561 | T | C | 222 | Kcnq1 | intronic | . | . | . | 7qF5 | rs241414231 | 0 | 1 |
| chr7 | 143191407 | T | C | 222 | Kcnq1 | intronic | . | . | . | 7qF5 | rs261393935 | 0 | 1 |
| chr7 | 143191553 | T | C | 222 | Kcnq1 | intronic | . | . | . | 7qF5 | rs33829138 | 0 | 1 |
| chr7 | 143191628 | T | C | 91 | Kcnq1 | intronic | . | . | . | 7qF5 | rs33829141 | 0 | 1 |
| chr7 | 143192381 | G | A | 84 | Kcnq1 | intronic | . | . | . | 7qF5 | rs33829876 | 0 | 1 |
| chr7 | 143192504 | G | A | 172 | Kcnq1 | intronic | . | . | . | 7qF5 | rs33829882 | 0 | 1 |
| chr7 | 143192532 | T | C | 222 | Kcnq1 | intronic | . | . | . | 7qF5 | rs229761629 | 0 | 1 |
| chr7 | 143192653 | T | C | 222 | Kcnq1 | exonic | synonymous SNV | Kcnq1:NM_008434:exon8:c.T1107C:p.A369A | . | 7qF5 | rs248751982 | 0 | 1 |
| chr7 | 143192695 | A | G | 222 | Kcnq1 | intronic | . | . | . | 7qF5 | rs33830884 | 0 | 1 |
| chr7 | 143192718 | A | G | 139 | Kcnq1 | intronic | . | . | . | 7qF5 | rs33830887 | 0 | 1 |
| chr7 | 143194299 | T | C | 222 | Kcnq1 | exonic | synonymous SNV | Kcnq1:NM_008434:exon9:c.T1203C:p.S401S | . | 7qF5 | rs241993005 | 0 | 1 |
| chr7 | 143194410 | G | A | 119 | Kcnq1 | intronic | . | . | . | 7qF5 | rs255308222 | 0 | 1 |
| chr7 | 143195144 | A | C | 79 | Kcnq1 | intronic | . | . | . | 7qF5 | . | 0 | 1 |
| chr7 | 143195199 | G | A | 192 | Kcnq1 | intronic | . | . | . | 7qF5 | rs212505882 | 0 | 1 |
| chr7 | 143195431 | G | A | 222 | Kcnq1 | intronic | . | . | . | 7qF5 | rs33825701 | 0 | 1 |
| chr7 | 143195513 | G | A | 157 | Kcnq1 | intronic | . | . | . | 7qF5 | rs242796305 | 0 | 1 |
| chr7 | 143195571 | G | C | 88 | Kcnq1 | intronic | . | . | . | 7qF5 | rs33826614 | 0 | 1 |
| chr7 | 143296521 | G | A | 222 | Kcnq1,Kcnq1ot1 | ncRNA_exonic | . | . | 246 | 7qF5 | rs229876214 | 0 | 1 |
| chr7 | 143362783 | T | C | 60 | Kcnq1 | intronic | . | . | . | 7qF5 | rs253543228 | 0 | 1 |
| chr7 | 143363359 | C | G | 180 | Kcnq1 | intronic | . | . | . | 7qF5 | rs243632887 | 0 | 1 |
| chr7 | 143425784 | A | C | 99 | Kcnq1 | intronic | . | . | . | 7qF5 | rs33837956 | 0 | 1 |
| chr7 | 143476224 | C | A | 74 | Slc22a18 | exonic | synonymous SNV | Slc22a18:NM_001042760:exon2:c.C27A:p.P9P,Slc22a18:NM_008767:exon2:c.C27A:p.P9P | . | 7qF5 | rs8262529 | 0 | 1 |
| chr7 | 143479178 | G | T | 125 | Slc22a18 | intronic | . | . | . | 7qF5 | rs8262538 | 0 | 1 |
| chr7 | 143479179 | T | G | 125 | Slc22a18 | intronic | . | . | . | 7qF5 | rs8262539 | 0 | 1 |
| chr7 | 143479443 | C | T | 163 | Slc22a18 | intronic | . | . | . | 7qF5 | rs8262542 | 0 | 1 |
| chr7 | 143479933 | A | G | 83 | Slc22a18 | intronic | . | . | . | 7qF5 | rs8262544 | 0 | 1 |
| chr7 | 143479979 | G | A | 25.1406 | Slc22a18 | intronic | . | . | . | 7qF5 | rs33833885 | 0 | 1 |
| chr7 | 143480606 | A | G | 222 | Slc22a18 | intronic | . | . | . | 7qF5 | rs8262545 | 0 | 1 |
| chr7 | 143491943 | T | C | 44.9053 | Slc22a18 | intronic | . | . | . | 7qF5 | rs33832989 | 0 | 1 |
| chr7 | 143492783 | A | G | 134 | Slc22a18 | intronic | . | . | . | 7qF5 | rs8262579 | 0 | 1 |
| chr7 | 143497005 | T | C | 199 | Slc22a18 | exonic | synonymous SNV | Slc22a18:NM_001042760:exon9:c.T877C:p.L293L,Slc22a18:NM_008767:exon9:c.T877C:p.L293L | . | 7qF5 | rs33837074 | 0 | 1 |
| chr7 | 143497600 | A | G | 78 | Slc22a18 | intronic | . | . | . | 7qF5 | rs8262608 | 0 | 1 |
| chr7 | 143499260 | A | C | 222 | Slc22a18 | exonic | missense SNV | Slc22a18:NM_001042760:exon11:c.A1221C:p.E407D,Slc22a18:NM_008767:exon11:c.A1221C:p.E407D | . | 7qF5 | rs8262638 | 0 | 1 |
| chr7 | 143501849 | G | A | 63 | Phlda2 | intronic | . | . | 1316 | 7qF5 | rs216341637 | 0 | 1 |
| chr7 | 143502389 | T | G | 222 | Phlda2 | exonic | synonymous SNV | Phlda2:NM_009434:exon1:c.A102C:p.V34V | 1316 | 7qF5 | rs239988740 | 0 | 1 |
| chr7 | 143502535 | G | A | 222 | . | upstream | . | . | 1316 | 7qF5 | rs224816146 | 0 | 1 |
| chr7 | 143514455 | A | G | 205 | Nap1l4 | UTR3 | . | . | . | 7qF5 | rs245435220 | 0 | 1 |
| chr7 | 143521295 | C | T | 114 | Nap1l4 | intronic | . | . | . | 7qF5 | rs253963469 | 0 | 1 |
| chr7 | 143521327 | T | C | 133 | Nap1l4 | intronic | . | . | . | 7qF5 | rs213556554 | 0 | 1 |
| chr7 | 143521383 | C | A | 222 | Nap1l4 | intronic | . | . | . | 7qF5 | rs33833522 | 0 | 1 |
| chr7 | 143524045 | G | A | 152 | Nap1l4 | intronic | . | . | . | 7qF5 | rs227046091 | 0 | 1 |
| chr7 | 143524451 | T | C | 92 | Nap1l4 | intronic | . | . | . | 7qF5 | rs33837145 | 0 | 1 |
| chr7 | 143527016 | G | T | 65 | Nap1l4 | intronic | . | . | . | 7qF5 | rs33838080 | 0 | 1 |
| chr7 | 143529410 | G | T | 222 | Nap1l4 | intronic | . | . | . | 7qF5 | rs219900033 | 0 | 1 |

| | | | | | | | | | | | | | |
|---|---|---|---|---|---|---|---|---|---|---|---|---|---|
| chr7 | 143529619 | C | T | 192 | Nap1l4 | intronic | . | . | . | 7qF5 | rs33839678 | 0 | 1 |
| chr7 | 143532283 | T | C | 222 | Nap1l4 | intronic | . | . | . | 7qF5 | rs33834120 | 0 | 1 |
| chr7 | 143532425 | G | A | 222 | Nap1l4 | intronic | . | . | . | 7qF5 | rs257920530 | 0 | 1 |
| chr7 | 143532482 | C | T | 128 | Nap1l4 | intronic | . | . | . | 7qF5 | rs225634005 | 0 | 1 |
| chr7 | 143535635 | T | C | 222 | Nap1l4 | intronic | . | . | . | 7qF5 | rs33837625 | 0 | 1 |
| chr7 | 143536071 | A | G | 186 | Nap1l4 | intronic | . | . | . | 7qF5 | rs33837628 | 0 | 1 |
| chr7 | 143540016 | T | G | 68 | Nap1l4 | intronic | . | . | . | 7qF5 | rs33839496 | 0 | 1 |
| chr7 | 143540045 | T | A | 107 | Nap1l4 | intronic | . | . | . | 7qF5 | rs33839499 | 0 | 1 |
| chr7 | 143540295 | A | C | 222 | Nap1l4 | intronic | . | . | . | 7qF5 | rs33839502 | 0 | 1 |
| chr7 | 143541572 | A | G | 44.7452 | Nap1l4 | intronic | . | . | . | 7qF5 | rs33840947 | 0 | 1 |
| chr7 | 143541688 | C | T | 188 | Nap1l4 | intronic | . | . | . | 7qF5 | rs33840949 | 0 | 1 |
| chr7 | 143541841 | C | A | 222 | Nap1l4 | intronic | . | . | . | 7qF5 | rs33840952 | 0 | 1 |
| chr7 | 143541944 | T | C | 107 | Nap1l4 | intronic | . | . | . | 7qF5 | rs252801573 | 0 | 1 |
| chr7 | 143559726 | A | G | 222 | Cars | intronic | . | . | . | 7qF5 | rs33837974 | 0 | 1 |
| chr7 | 143561818 | G | A | 156 | Cars | intronic | . | . | . | 7qF5 | rs33837983 | 0 | 1 |
| chr7 | 143561836 | G | C | 176 | Cars | intronic | . | . | . | 7qF5 | rs33838835 | 0 | 1 |
| chr7 | 143561869 | C | T | 222 | Cars | exonic | missense SNV | Cars:NM_001252593:exon19:c.G1958A:p.G653E,Cars:NM_013742:exon20:c.G2207A:p.G736E | . | 7qF5 | rs234309198 | 0 | 1 |
| chr7 | 143562899 | G | A | 213 | Cars | intronic | . | . | . | 7qF5 | rs33839644 | 0 | 1 |
| chr7 | 143563071 | A | G | 222 | Cars | intronic | . | . | . | 7qF5 | rs33839647 | 0 | 1 |
| chr7 | 143563111 | G | T | 97 | Cars | intronic | . | . | . | 7qF5 | rs264061202 | 0 | 1 |
| chr7 | 143563112 | G | A | 97 | Cars | intronic | . | . | . | 7qF5 | rs217760398 | 0 | 1 |
| chr7 | 143564869 | A | G | 156 | Cars | intronic | . | . | . | 7qF5 | rs249489210 | 0 | 1 |
| chr7 | 143564928 | T | G | 222 | Cars | intronic | . | . | . | 7qF5 | rs219900549 | 0 | 1 |
| chr7 | 143565027 | T | C | 222 | Cars | exonic | synonymous SNV | Cars:NM_001252593:exon17:c.A1776G:p.S592S,Cars:NM_013742:exon18:c.A2025G:p.S675S | . | 7qF5 | rs245029322 | 0 | 1 |
| chr7 | 143565140 | A | G | 222 | Cars | intronic | . | . | . | 7qF5 | rs263308174 | 0 | 1 |
| chr7 | 143568280 | G | A | 220 | Cars | intronic | . | . | . | 7qF5 | rs236325979 | 0 | 1 |
| chr7 | 143568424 | C | T | 222 | Cars | intronic | . | . | . | 7qF5 | rs33836359 | 0 | 1 |
| chr7 | 143568875 | T | C | 35.646 | Cars | intronic | . | . | . | 7qF5 | rs33837516 | 0 | 1 |
| chr7 | 143568921 | C | T | 86 | Cars | intronic | . | . | . | 7qF5 | rs224341112 | 0 | 1 |
| chr7 | 143571434 | A | G | 113 | Cars | intronic | . | . | . | 7qF5 | rs243507277 | 0 | 1 |
| chr7 | 143571453 | T | C | 120 | Cars | intronic | . | . | . | 7qF5 | rs214766324 | 0 | 1 |
| chr7 | 143571636 | C | T | 120 | Cars | intronic | . | . | . | 7qF5 | rs33838431 | 0 | 1 |
| chr7 | 143571647 | A | T | 85 | Cars | intronic | . | . | . | 7qF5 | rs33839194 | 0 | 1 |
| chr7 | 143574400 | T | C | 131 | Cars | intronic | . | . | . | 7qF5 | rs231668459 | 0 | 1 |
| chr7 | 143574688 | A | G | 222 | Cars | intronic | . | . | . | 7qF5 | rs262446531 | 0 | 1 |
| chr7 | 143574692 | A | G | 222 | Cars | intronic | . | . | . | 7qF5 | rs215743910 | 0 | 1 |
| chr7 | 143575764 | G | A | 100 | Cars | intronic | . | . | . | 7qF5 | rs219622266 | 0 | 1 |
| chr7 | 143575772 | G | A | 114 | Cars | intronic | . | . | . | 7qF5 | rs244549682 | 0 | 1 |
| chr7 | 143575926 | A | G | 222 | Cars | exonic | synonymous SNV | Cars:NM_001252593:exon8:c.T774C:p.N258N,Cars:NM_013742:exon9:c.T1023C:p.N341N | . | 7qF5 | rs33837138 | 0 | 1 |
| chr7 | 143575947 | G | A | 222 | Cars | exonic | synonymous SNV | Cars:NM_001252593:exon8:c.C753T:p.F251F,Cars:NM_013742:exon9:c.C1002T:p.F334F | . | 7qF5 | rs232750419 | 0 | 1 |
| chr7 | 143575986 | T | C | 222 | Cars | exonic | synonymous SNV | Cars:NM_001252593:exon8:c.A714G:p.L238L,Cars:NM_013742:exon9:c.A963G:p.L321L | . | 7qF5 | rs33837141 | 0 | 1 |
| chr7 | 143576431 | A | G | 66 | Cars | intronic | . | . | . | 7qF5 | rs243337637 | 0 | 1 |
| chr7 | 143576768 | G | T | 222 | Cars | intronic | . | . | . | 7qF5 | rs212712210 | 0 | 1 |
| chr7 | 143576789 | C | T | 213 | Cars | intronic | . | . | . | 7qF5 | rs225622779 | 0 | 1 |
| chr7 | 143585538 | C | T | 117 | Cars | intronic | . | . | . | 7qF5 | rs249028095 | 0 | 1 |
| chr7 | 143585618 | T | G | 218 | Cars | intronic | . | . | . | 7qF5 | rs212348969 | 0 | 1 |
| chr7 | 143585732 | G | A | 222 | Cars | exonic | synonymous SNV | Cars:NM_001252593:exon5:c.C369T:p.T123T,Cars:NM_013742:exon6:c.C618T:p.T206T | . | 7qF5 | rs231093560 | 0 | 1 |
| chr7 | 143585828 | A | G | 72 | Cars | intronic | . | . | . | 7qF5 | rs251573221 | 0 | 1 |
| chr7 | 143585864 | C | T | 58 | Cars | intronic | . | . | . | 7qF5 | rs215685817 | 0 | 1 |
| chr7 | 143586390 | A | C | 181 | Cars | intronic | . | . | . | 7qF5 | rs253046556 | 0 | 1 |
| chr7 | 143586404 | G | A | 201 | Cars | intronic | . | . | . | 7qF5 | rs217868441 | 0 | 1 |
| chr7 | 143586410 | C | T | 186 | Cars | intronic | . | . | . | 7qF5 | rs236605141 | 0 | 1 |
| chr7 | 143587016 | G | A | 126 | Cars | intronic | . | . | . | 7qF5 | rs245332241 | 0 | 1 |
| chr7 | 143587031 | A | T | 160 | Cars | intronic | . | . | . | 7qF5 | rs265736745 | 0 | 1 |
| chr7 | 143587149 | A | G | 222 | Cars | exonic | synonymous SNV | Cars:NM_001252593:exon3:c.T162C:p.C54C,Cars:NM_013742:exon4:c.T411C:p.C137C | . | 7qF5 | rs33836161 | 0 | 1 |
| chr7 | 143588345 | T | C | 82 | Cars | intronic | . | . | . | 7qF5 | rs33837232 | 0 | 1 |
| chr7 | 143588378 | A | G | 73 | Cars | intronic | . | . | . | 7qF5 | rs33838134 | 0 | 1 |
| chr7 | 143588419 | C | A | 85 | Cars | intronic | . | . | . | 7qF5 | rs33838136 | 0 | 1 |
| chr7 | 143588450 | T | C | 117 | Cars | intronic | . | . | . | 7qF5 | rs33838139 | 0 | 1 |
| chr7 | 143588485 | T | C | 222 | Cars | intronic | . | . | . | 7qF5 | rs33838142 | 0 | 1 |
| chr7 | 143588603 | T | C | 222 | Cars | intronic | . | . | . | 7qF5 | rs33839084 | 0 | 1 |
| chr7 | 143592312 | C | A | 69 | Cars | intronic | . | . | . | 7qF5 | rs214541170 | 0 | 1 |
| chr7 | 143592361 | T | C | 83 | Cars | intronic | . | . | . | 7qF5 | rs239791262 | 0 | 1 |
| chr7 | 143592393 | G | A | 159 | Cars | intronic | . | . | . | 7qF5 | rs253533269 | 0 | 1 |
| chr7 | 143592788 | G | A | 68 | Cars | intronic | . | . | . | 7qF5 | rs236368250 | 0 | 1 |
| chr7 | 143592841 | T | C | 35.8663 | Cars | intronic | . | . | . | 7qF5 | rs221214063 | 0 | 1 |
| chr7 | 143596605 | A | G | 36.3109 | Cars | intronic | . | . | . | 7qF5 | rs33838989 | 0 | 1 |
| chr7 | 143596764 | C | T | 40.9399 | Cars | intronic | . | . | . | 7qF5 | rs247076043 | 0 | 1 |
| chr7 | 143596765 | A | T | 40.9381 | Cars | intronic | . | . | . | 7qF5 | rs216536212 | 0 | 1 |
| chr7 | 143596922 | A | T | 172 | Cars | intronic | . | . | . | 7qF5 | rs229101720 | 0 | 1 |
| chr7 | 143597034 | T | C | 39.5854 | Cars | intronic | . | . | . | 7qF5 | rs33839778 | 0 | 1 |
| chr7 | 143597073 | C | T | 61 | Cars | intronic | . | . | . | 7qF5 | . | 0 | 1 |
| chr7 | 143609922 | C | T | 131 | Tnfrsf26 | UTR3 | . | . | . | 7qF5 | rs221296258 | 0 | 1 |
| chr7 | 143610038 | G | A | 221 | Tnfrsf26 | UTR3 | . | . | . | 7qF5 | rs33838597 | 0 | 1 |
| chr7 | 143610158 | G | A | 106 | Tnfrsf26 | intronic | . | . | . | 7qF5 | rs253107348 | 0 | 1 |
| chr7 | 143610207 | A | C | 32.3956 | Tnfrsf26 | intronic | . | . | . | 7qF5 | rs33838600 | 0 | 1 |
| chr7 | 143611568 | G | A | 51 | Tnfrsf26 | intronic | . | . | . | 7qF5 | . | 1 | 0 |
| chr7 | 143611942 | C | T | 221 | Tnfrsf26 | intronic | . | . | . | 7qF5 | rs50955996 | 0 | 1 |
| chr7 | 143611945 | T | C | 221 | Tnfrsf26 | intronic | . | . | . | 7qF5 | rs48734651 | 0 | 1 |
| chr7 | 143611965 | G | A | 211 | Tnfrsf26 | intronic | . | . | . | 7qF5 | rs47959452 | 0 | 1 |
| chr7 | 143614866 | T | G | 222 | Tnfrsf26 | exonic | missense SNV | Tnfrsf26:NM_175649:exon5:c.A434C:p.K145T | . | 7qF5 | rs33840669 | 0 | 1 |
| chr7 | 143614912 | T | C | 222 | Tnfrsf26 | exonic | missense SNV | Tnfrsf26:NM_175649:exon5:c.A388G:p.R130G | . | 7qF5 | rs227219154 | 0 | 1 |
| chr7 | 143614916 | G | A | 222 | Tnfrsf26 | exonic | synonymous SNV | Tnfrsf26:NM_175649:exon5:c.C384T:p.D128D | . | 7qF5 | rs244942454 | 0 | 1 |
| chr7 | 143615012 | G | A | 200 | Tnfrsf26 | intronic | . | . | . | 7qF5 | rs33840672 | 0 | 1 |
| chr7 | 143615089 | T | A | 122 | Tnfrsf26 | intronic | . | . | . | 7qF5 | rs228062639 | 0 | 1 |
| chr7 | 143617752 | C | T | 105 | Tnfrsf26 | intronic | . | . | . | 7qF5 | rs227134835 | 0 | 1 |
| chr7 | 143617801 | G | T | 149 | Tnfrsf26 | intronic | . | . | . | 7qF5 | rs240426146 | 0 | 1 |
| chr7 | 143617802 | C | T | 148 | Tnfrsf26 | intronic | . | . | . | 7qF5 | rs259467934 | 0 | 1 |
| chr7 | 143617968 | G | A | 222 | Tnfrsf26 | intronic | . | . | . | 7qF5 | rs33841840 | 0 | 1 |
| chr7 | 143618029 | T | C | 185 | Tnfrsf26 | intronic | . | . | . | 7qF5 | rs254438297 | 0 | 1 |
| chr7 | 143618030 | A | G | 191 | Tnfrsf26 | intronic | . | . | . | 7qF5 | rs211906390 | 0 | 1 |
| chr7 | 143618339 | T | C | 222 | Tnfrsf26 | exonic | missense SNV | Tnfrsf26:NM_175649:exon3:c.A233G:p.E78G | . | 7qF5 | rs33835050 | 0 | 1 |
| chr7 | 143619152 | G | A | 23.6493 | Tnfrsf26 | intronic | . | . | . | 7qF5 | rs6294118 | 0 | 1 |
| chr7 | 143619353 | T | C | 222 | Tnfrsf26 | exonic | synonymous SNV | Tnfrsf26:NM_175649:exon2:c.A123G:p.Q41Q | . | 7qF5 | rs6308071 | 0 | 1 |
| chr7 | 143619399 | A | T | 222 | Tnfrsf26 | exonic | missense SNV | Tnfrsf26:NM_175649:exon2:c.T77A:p.L26Q | . | 7qF5 | rs6308499 | 0 | 1 |
| chr7 | 143619522 | T | C | 121 | Tnfrsf26 | intronic | . | . | . | 7qF5 | rs6309073 | 0 | 1 |
| chr7 | 143689614 | A | G | 72 | Osbpl5 | UTR3 | . | . | . | 7qF5 | rs33836561 | 0 | 1 |
| chr7 | 143689965 | G | C | 222 | Osbpl5 | intronic | . | . | . | 7qF5 | rs226336036 | 0 | 1 |
| chr7 | 143690033 | G | C | 177 | Osbpl5 | intronic | . | . | . | 7qF5 | rs240519587 | 0 | 1 |
| chr7 | 143690041 | A | G | 132 | Osbpl5 | intronic | . | . | . | 7qF5 | rs261087966 | 0 | 1 |
| chr7 | 143691798 | T | A | 125 | Osbpl5 | intronic | . | . | . | 7qF5 | rs233754910 | 0 | 1 |
| chr7 | 143691827 | G | A | 132 | Osbpl5 | intronic | . | . | . | 7qF5 | rs254474352 | 0 | 1 |
| chr7 | 143692751 | T | C | 58 | Osbpl5 | intronic | . | . | . | 7qF5 | rs233895363 | 0 | 1 |
| chr7 | 143692758 | C | T | 72 | Osbpl5 | intronic | . | . | . | 7qF5 | rs258311339 | 0 | 1 |
| chr7 | 143693605 | G | A | 110 | Osbpl5 | intronic | . | . | . | 7qF5 | rs33839344 | 0 | 1 |
| chr7 | 143693951 | G | A | 222 | Osbpl5 | intronic | . | . | . | 7qF5 | rs33839348 | 0 | 1 |
| chr7 | 143694387 | T | C | 108 | Osbpl5 | splicing | . | . | . | 7qF5 | rs33839351 | 0 | 1 |
| chr7 | 143695154 | G | T | 222 | Osbpl5 | intronic | . | . | . | 7qF5 | rs221065984 | 0 | 1 |
| chr7 | 143695272 | C | T | 51 | Osbpl5 | intronic | . | . | . | 7qF5 | rs240424828 | 0 | 1 |
| chr7 | 143695274 | T | C | 51 | Osbpl5 | intronic | . | . | . | 7qF5 | rs33840150 | 0 | 1 |
| chr7 | 143695369 | G | T | 45.4007 | Osbpl5 | intronic | . | . | . | 7qF5 | rs33840819 | 0 | 1 |
| chr7 | 143695467 | A | T | 159 | Osbpl5 | intronic | . | . | . | 7qF5 | rs250818154 | 0 | 1 |
| chr7 | 143695714 | G | A | 118 | Osbpl5 | intronic | . | . | . | 7qF5 | rs261317716 | 0 | 1 |
| chr7 | 143700630 | T | A | 113 | Osbpl5 | intronic | . | . | . | 7qF5 | rs215438592 | 0 | 1 |
| chr7 | 143700689 | T | C | 202 | Osbpl5 | intronic | . | . | . | 7qF5 | rs241478050 | 0 | 1 |
| chr7 | 143700993 | G | T | 207 | Osbpl5 | intronic | . | . | . | 7qF5 | rs260551997 | 0 | 1 |
| chr7 | 143701409 | T | A | 41.9837 | Osbpl5 | intronic | . | . | . | 7qF5 | rs51167624 | 0 | 1 |
| chr7 | 143702620 | T | A | 49.8413 | Osbpl5 | intronic | . | . | . | 7qF5 | rs37187533 | 0 | 1 |
| chr7 | 143702683 | C | T | 81 | Osbpl5 | intronic | . | . | . | 7qF5 | rs580956709 | 0 | 1 |
| chr7 | 143702756 | A | G | 92 | Osbpl5 | exonic | synonymous SNV | Osbpl5:NM_001199227:exon11:c.T1257C:p.D419D,Osbpl5:NM_024289:exon11:c.T1329C:p.D443D | . | 7qF5 | rs36330123 | 0 | 1 |
| chr7 | 143702796 | T | C | 65 | Osbpl5 | intronic | . | . | . | 7qF5 | rs219684735 | 0 | 1 |
| chr7 | 143703427 | A | G | 43.9771 | Osbpl5 | intronic | . | . | . | 7qF5 | rs218566282 | 0 | 1 |
| chr7 | 143704912 | C | G | 81 | Osbpl5 | intronic | . | . | . | 7qF5 | rs235817386 | 0 | 1 |
| chr7 | 143705077 | G | A | 82 | Osbpl5 | exonic | missense SNV | Osbpl5:NM_001199227:exon8:c.C755T:p.T252M,Osbpl5:NM_024289:exon8:c.C827T:p.T276M | . | 7qF5 | rs254626854 | 0 | 1 |
| chr7 | 143705091 | G | C | 82 | Osbpl5 | exonic | synonymous SNV | Osbpl5:NM_001199227:exon8:c.C741G:p.L247L,Osbpl5:NM_024289:exon8:c.C813G:p.L271L | . | 7qF5 | rs225374000 | 0 | 1 |
| chr7 | 143707227 | C | T | 154 | Osbpl5 | intronic | . | . | . | 7qF5 | rs254591716 | 0 | 1 |
| chr7 | 143707386 | A | G | 222 | Osbpl5 | exonic | synonymous SNV | Osbpl5:NM_001199227:exon7:c.T651C:p.P217P,Osbpl5:NM_024289:exon7:c.T723C:p.P241P | . | 7qF5 | rs219358703 | 0 | 1 |
| chr7 | 143707446 | T | C | 222 | Osbpl5 | intronic | . | . | . | 7qF5 | rs238393472 | 0 | 1 |
| chr7 | 143707490 | T | G | 103 | Osbpl5 | intronic | . | . | . | 7qF5 | rs265528749 | 0 | 1 |
| chr7 | 143709506 | C | G | 159 | Osbpl5 | intronic | . | . | . | 7qF5 | . | 0 | 1 |
| chr7 | 143709727 | A | T | 222 | Osbpl5 | intronic | . | . | . | 7qF5 | rs238384819 | 0 | 1 |
| chr7 | 143709869 | A | G | 222 | Osbpl5 | intronic | . | . | . | 7qF5 | rs263657383 | 0 | 1 |
| chr7 | 143715654 | G | A | 222 | Osbpl5 | intronic | . | . | . | 7qF5 | rs233466846 | 0 | 1 |
| chr7 | 143741551 | G | A | 48.2181 | Osbpl5 | intronic | . | . | . | 7qF5 | . | 0 | 1 |
| chr7 | 143741587 | G | C | 63 | Osbpl5 | intronic | . | . | . | 7qF5 | rs232553642 | 0 | 1 |
| chr7 | 143741609 | T | C | 114 | Osbpl5 | intronic | . | . | . | 7qF5 | rs36578126 | 0 | 1 |
| chr7 | 143741647 | G | A | 179 | Osbpl5 | intronic | . | . | . | 7qF5 | rs261499717 | 0 | 1 |
| chr7 | 143764545 | A | C | 99 | Mrgprg | exonic | missense SNV | Mrgprg:NM_203492:exon2:c.T829G:p.S277A | . | 7qF5 | rs236108013 | 0 | 1 |
| chr7 | 143764630 | G | A | 195 | Mrgprg | exonic | synonymous SNV | Mrgprg:NM_203492:exon2:c.C744T:p.P248P | . | 7qF5 | rs37198213 | 0 | 1 |
| chr7 | 143765005 | T | C | 222 | Mrgprg | exonic | synonymous SNV | Mrgprg:NM_203492:exon2:c.A369G:p.S123S | . | 7qF5 | rs36241914 | 0 | 1 |
| chr7 | 143765433 | C | T | 222 | Mrgprg | intronic | . | . | . | 7qF5 | rs36850230 | 0 | 1 |
| chr7 | 143765586 | A | G | 45.8132 | Mrgprg | intronic | . | . | . | 7qF5 | rs257762980 | 0 | 1 |
| chr7 | 143781438 | G | A | 220 | Mrgpre | exonic | synonymous SNV | Mrgpre:NM_175534:exon2:c.C327T:p.L109L | . | 7qF5 | rs246063758 | 0 | 1 |
| chr7 | 143781480 | C | T | 221 | Mrgpre | exonic | synonymous SNV | Mrgpre:NM_175534:exon2:c.G285A:p.L95L | . | 7qF5 | rs259496643 | 0 | 1 |
| chr7 | 143781647 | C | T | 211 | Mrgpre | exonic | missense SNV | Mrgpre:NM_175534:exon2:c.G118A:p.V40M | . | 7qF5 | rs226994857 | 0 | 1 |
| chr7 | 143781792 | C | T | 188 | Mrgpre | UTR5 | . | . | . | 7qF5 | rs246982175 | 0 | 1 |
| chr7 | 143781805 | C | T | 181 | Mrgpre | UTR5 | . | . | . | 7qF5 | rs217786789 | 0 | 1 |
| chr7 | 143781841 | G | A | 45.0522 | Mrgpre | intronic | . | . | . | 7qF5 | rs240064445 | 0 | 1 |
| chr7 | 143781890 | G | A | 27.1181 | Mrgpre | intronic | . | . | . | 7qF5 | rs212700859 | 0 | 1 |
| chr7 | 143795915 | A | G | 152 | Nadsyn1 | UTR3 | . | . | . | 7qF5 | rs6304885 | 0 | 1 |
| chr7 | 143795999 | A | G | 222 | Nadsyn1 | exonic | missense SNV | Nadsyn1:NM_001308095:exon19:c.T1390C:p.F464L,Nadsyn1:NM_030221:exon21:c.T2128C:p.F710L | . | 7qF5 | rs6305405 | 0 | 1 |
| chr7 | 143797816 | T | C | 185 | Nadsyn1 | intronic | . | . | . | 7qF5 | rs36734108 | 0 | 1 |
| chr7 | 143797867 | A | G | 222 | Nadsyn1 | exonic | synonymous SNV | Nadsyn1:NM_001308095:exon18:c.T1290C:p.Y430Y,Nadsyn1:NM_030221:exon20:c.T2028C:p.Y676Y | . | 7qF5 | rs36747124 | 0 | 1 |
| chr7 | 143798095 | C | G | 157 | Nadsyn1 | intronic | . | . | . | 7qF5 | rs248114787 | 0 | 1 |
| chr7 | 143798110 | T | C | 77 | Nadsyn1 | intronic | . | . | . | 7qF5 | rs218652467 | 0 | 1 |
| chr7 | 143798328 | T | G | 97 | Nadsyn1 | intronic | . | . | . | 7qF5 | rs226979161 | 0 | 1 |
| chr7 | 143798378 | A | G | 123 | Nadsyn1 | intronic | . | . | . | 7qF5 | rs36300591 | 0 | 1 |
| chr7 | 143798693 | G | A | 159 | Nadsyn1 | intronic | . | . | . | 7qF5 | rs257110408 | 0 | 1 |
| chr7 | 143798700 | G | A | 144 | Nadsyn1 | intronic | . | . | . | 7qF5 | rs226130297 | 0 | 1 |
| chr7 | 143799180 | G | T | 173 | Nadsyn1 | intronic | . | . | . | 7qF5 | rs252864828 | 0 | 1 |
| chr7 | 143799750 | A | G | 187 | Nadsyn1 | intronic | . | . | . | 7qF5 | rs250495338 | 0 | 1 |
| chr7 | 143800041 | T | C | 156 | Nadsyn1 | intronic | . | . | . | 7qF5 | rs215816756 | 0 | 1 |
| chr7 | 143803108 | T | C | 62 | Nadsyn1 | intronic | . | . | . | 7qF5 | rs36699116 | 0 | 1 |
| chr7 | 143803338 | A | G | 179 | Nadsyn1 | intronic | . | . | . | 7qF5 | rs37132916 | 0 | 1 |
| chr7 | 143804088 | T | C | 220 | Nadsyn1 | exonic | missense SNV | Nadsyn1:NM_001308095:exon13:c.A643G:p.M215V,Nadsyn1:NM_030221:exon15:c.A1381G:p.M461V | . | 7qF5 | rs36612792 | 0 | 1 |
| chr7 | 143805971 | C | T | 86 | Nadsyn1 | exonic | synonymous SNV | Nadsyn1:NM_001308095:exon12:c.G525A:p.E175E,Nadsyn1:NM_030221:exon14:c.G1263A:p.E421E | . | 7qF5 | rs263039787 | 0 | 1 |
| chr7 | 143806731 | A | G | 26.2936 | Nadsyn1 | intronic | . | . | . | 7qF5 | rs219198779 | 0 | 1 |
| chr7 | 143806863 | A | G | 173 | Nadsyn1 | intronic | . | . | . | 7qF5 | rs243898713 | 0 | 1 |
| chr7 | 143806866 | G | A | 182 | Nadsyn1 | intronic | . | . | . | 7qF5 | rs265468672 | 0 | 1 |
| chr7 | 143806968 | A | C | 153 | Nadsyn1 | exonic | synonymous SNV | Nadsyn1:NM_001308095:exon11:c.T324G:p.L108L,Nadsyn1:NM_030221:exon13:c.T1062G:p.L354L | . | 7qF5 | rs232302428 | 0 | 1 |
| chr7 | 143806980 | G | A | 125 | Nadsyn1 | exonic | synonymous SNV | Nadsyn1:NM_001308095:exon11:c.C312T:p.A104A,Nadsyn1:NM_030221:exon13:c.C1050T:p.A350A | . | 7qF5 | rs259179244 | 0 | 1 |
| chr7 | 143807018 | T | C | 74 | Nadsyn1 | intronic | . | . | . | 7qF5 | rs261259317 | 0 | 1 |
| chr7 | 143807301 | A | T | 219 | Nadsyn1 | intronic | . | . | . | 7qF5 | rs239168329 | 0 | 1 |
| chr7 | 143807328 | C | T | 216 | Nadsyn1 | intronic | . | . | . | 7qF5 | rs252492693 | 0 | 1 |
| chr7 | 143807467 | G | A | 222 | Nadsyn1 | intronic | . | . | . | 7qF5 | rs217485244 | 0 | 1 |
| chr7 | 143808020 | C | T | 172 | Nadsyn1 | exonic | missense SNV | Nadsyn1:NM_001308095:exon9:c.G225A:p.M75I,Nadsyn1:NM_030221:exon11:c.G963A:p.M321I | . | 7qF5 | rs247321994 | 0 | 1 |
| chr7 | 143808077 | C | T | 204 | Nadsyn1 | exonic | synonymous SNV | Nadsyn1:NM_001308095:exon9:c.G168A:p.T56T,Nadsyn1:NM_030221:exon11:c.G906A:p.T302T | . | 7qF5 | rs261230622 | 0 | 1 |
| chr7 | 143808161 | C | T | 142 | Nadsyn1 | intronic | . | . | . | 7qF5 | rs224165056 | 0 | 1 |
| chr7 | 143808192 | A | G | 86 | Nadsyn1 | intronic | . | . | . | 7qF5 | rs238070923 | 0 | 1 |
| chr7 | 143808244 | T | G | 24.6719 | Nadsyn1 | intronic | . | . | . | 7qF5 | rs257604942 | 0 | 1 |
| chr7 | 143808763 | C | T | 109 | Nadsyn1 | intronic | . | . | . | 7qF5 | rs236676418 | 0 | 1 |
| chr7 | 143808768 | G | A | 129 | Nadsyn1 | intronic | . | . | . | 7qF5 | rs262754124 | 0 | 1 |
| chr7 | 143808875 | A | G | 173 | Nadsyn1 | intronic | . | . | . | 7qF5 | rs224779318 | 0 | 1 |
| chr7 | 143811069 | C | A | 22.5518 | Nadsyn1 | intronic | . | . | . | 7qF5 | rs255079384 | 0 | 1 |
| chr7 | 143811071 | C | T | 22.5151 | Nadsyn1 | intronic | . | . | . | 7qF5 | rs217998197 | 0 | 1 |

| | | | | | | | | | | | | | |
|---|---|---|---|---|---|---|---|---|---|---|---|---|---|
| chr7 | 143811137 | G | A | 125 | Nadsyn1 | intronic | . | . | . | 7qF5 | rs230491148 | 0 | 1 |
| chr7 | 143811259 | A | G | 142 | Nadsyn1 | exonic | synonymous SNV | Nadsyn1:NM_030221:exon9:c.T708C:p.D236D | . | 7qF5 | rs251167215 | 0 | 1 |
| chr7 | 143811262 | A | G | 128 | Nadsyn1 | exonic | synonymous SNV | Nadsyn1:NM_030221:exon9:c.T705C:p.C235C | . | 7qF5 | rs223404618 | 0 | 1 |
| chr7 | 143811294 | C | T | 81 | Nadsyn1 | exonic | missense SNV | Nadsyn1:NM_030221:exon9:c.G673A:p.G225S | . | 7qF5 | rs247805404 | 0 | 1 |
| chr7 | 143811326 | A | G | 44.7349 | Nadsyn1 | intronic | . | . | . | 7qF5 | rs263601536 | 0 | 1 |
| chr7 | 143811433 | A | T | 47.3552 | Nadsyn1 | intronic | . | . | . | 7qF5 | rs226328548 | 0 | 1 |
| chr7 | 143812600 | T | C | 100 | Nadsyn1 | exonic | missense SNV | Nadsyn1:NM_030221:exon8:c.A556G:p.I186V | . | 7qF5 | rs218798877 | 0 | 1 |
| chr7 | 143812813 | T | C | 79 | Nadsyn1 | exonic | synonymous SNV | Nadsyn1:NM_030221:exon7:c.A498G:p.Q166Q | . | 7qF5 | rs211765886 | 0 | 1 |
| chr7 | 143812855 | G | C | 105 | Nadsyn1 | splicing | . | . | . | 7qF5 | rs224747517 | 0 | 1 |
| chr7 | 143812917 | T | C | 30.9928 | Nadsyn1 | intronic | . | . | . | 7qF5 | rs255006747 | 0 | 1 |
| chr7 | 143815471 | A | G | 91 | Nadsyn1 | intronic | . | . | . | 7qF5 | rs257146678 | 0 | 1 |
| chr7 | 143815478 | C | T | 88 | Nadsyn1 | intronic | . | . | . | 7qF5 | rs228258729 | 0 | 1 |
| chr7 | 143815481 | T | C | 79 | Nadsyn1 | intronic | . | . | . | 7qF5 | rs245613348 | 0 | 1 |
| chr7 | 143816035 | G | A | 104 | Nadsyn1 | intronic | . | . | . | 7qF5 | rs231838458 | 0 | 1 |
| chr7 | 143816114 | C | T | 222 | Nadsyn1 | intronic | . | . | . | 7qF5 | rs252974274 | 0 | 1 |
| chr7 | 143818989 | A | T | 72 | Nadsyn1 | intronic | . | . | . | 7qF5 | rs230064786 | 0 | 1 |
| chr7 | 143819242 | A | G | 222 | Nadsyn1 | intronic | . | . | . | 7qF5 | rs250706025 | 0 | 1 |
| chr7 | 143821031 | G | A | 120 | Nadsyn1 | intronic | . | . | . | 7qF5 | rs228120778 | 0 | 1 |
| chr7 | 143821114 | T | A | 222 | Nadsyn1 | intronic | . | . | . | 7qF5 | rs248072430 | 0 | 1 |
| chr7 | 143822819 | T | C | 146 | Nadsyn1 | UTR5 | . | . | 302 | 7qF5 | rs231462237 | 0 | 1 |
| chr7 | 143822851 | C | T | 85 | Nadsyn1 | UTR5 | . | . | 302 | 7qF5 | rs252533087 | 0 | 1 |
| chr7 | 143836507 | T | A | 112 | Dhcr7 | intronic | . | . | . | 7qF5 | rs6317957 | 0 | 1 |
| chr7 | 143836691 | C | T | 222 | Dhcr7 | exonic | synonymous SNV | Dhcr7:NM_007856:exon3:c.C48T:p.N16N | . | 7qF5 | rs6318605 | 0 | 1 |
| chr7 | 143837878 | C | T | 222 | Dhcr7 | exonic | missense SNV | Dhcr7:NM_007856:exon4:c.C203T:p.A68V | . | 7qF5 | rs248337659 | 0 | 1 |
| chr7 | 143837879 | A | G | 222 | Dhcr7 | exonic | synonymous SNV | Dhcr7:NM_007856:exon4:c.A204G:p.A68A | . | 7qF5 | rs221928985 | 0 | 1 |
| chr7 | 143838058 | T | C | 144 | Dhcr7 | intronic | . | . | . | 7qF5 | rs246308320 | 0 | 1 |
| chr7 | 143838069 | G | A | 93 | Dhcr7 | intronic | . | . | . | 7qF5 | rs36670487 | 0 | 1 |
| chr7 | 143840308 | A | G | 91 | Dhcr7 | intronic | . | . | . | 7qF5 | rs221274098 | 0 | 1 |
| chr7 | 143843099 | C | T | 222 | Dhcr7 | intronic | . | . | . | 7qF5 | rs222297126 | 0 | 1 |
| chr7 | 143845482 | T | C | 222 | Dhcr7 | exonic | synonymous SNV | Dhcr7:NM_007856:exon8:c.T861C:p.Y287Y | . | 7qF5 | rs13467624 | 0 | 1 |
| chr7 | 143845618 | A | G | 139 | Dhcr7 | intronic | . | . | . | 7qF5 | rs13479567 | 0 | 1 |
| chr7 | 143845654 | T | A | 59 | Dhcr7 | intronic | . | . | . | 7qF5 | rs36837272 | 0 | 1 |
| chr7 | 143847118 | T | C | 77 | Dhcr7 | exonic | synonymous SNV | Dhcr7:NM_007856:exon9:c.T1017C:p.L339L | . | 7qF5 | rs37917874 | 0 | 1 |
| chr7 | 143847130 | T | C | 74 | Dhcr7 | exonic | synonymous SNV | Dhcr7:NM_007856:exon9:c.T1029C:p.G343G | . | 7qF5 | rs228185644 | 0 | 1 |
| chr7 | 143847223 | T | C | 152 | Dhcr7 | exonic | synonymous SNV | Dhcr7:NM_007856:exon9:c.T1122C:p.I374I | . | 7qF5 | rs36471599 | 0 | 1 |
| chr7 | 143871636 | G | C | 189 | . | intergenic | . | . | . | 7qF5 | . | 0 | 1 |
| chr7 | 143871963 | G | A | 146 | . | intergenic | . | . | . | 7qF5 | rs234774964 | 0 | 1 |
| chr7 | 143881245 | C | T | 222 | . | intergenic | . | . | . | 7qF5 | rs36429421 | 0 | 1 |
| chr7 | 143883872 | C | T | 118 | . | intergenic | . | . | . | 7qF5 | rs257179343 | 0 | 1 |
| chr7 | 143893874 | T | G | 22.5029 | . | intergenic | . | . | . | 7qF5 | rs243679746 | 0 | 1 |
| chr7 | 143893898 | G | A | 100 | . | intergenic | . | . | . | 7qF5 | rs37691157 | 0 | 1 |
| chr7 | 143894185 | T | C | 222 | . | intergenic | . | . | . | 7qF5 | rs36936216 | 0 | 1 |
| chr7 | 143896227 | A | G | 115 | . | intergenic | . | . | . | 7qF5 | rs36596793 | 0 | 1 |
| chr7 | 143896475 | C | G | 222 | . | intergenic | . | . | . | 7qF5 | rs221991190 | 0 | 1 |
| chr7 | 143896507 | C | T | 222 | . | intergenic | . | . | . | 7qF5 | rs37483627 | 0 | 1 |
| chr7 | 144031383 | C | T | 222 | Mir3470b | ncRNA_intronic | . | . | . | 7qF5 | rs251573634 | 0 | 1 |
| chr7 | 144031403 | T | C | 222 | Mir3470b | ncRNA_intronic | . | . | . | 7qF5 | rs216294993 | 0 | 1 |
| chr7 | 144031509 | C | T | 118 | Mir3470b | ncRNA_intronic | . | . | . | 7qF5 | rs238773049 | 0 | 1 |
| chr7 | 144052115 | C | T | 53 | Mir3470b | ncRNA_intronic | . | . | . | 7qF5 | rs253659916 | 0 | 1 |
| chr7 | 144052228 | T | C | 211 | Mir3470b | ncRNA_intronic | . | . | . | 7qF5 | rs37533288 | 0 | 1 |
| chr7 | 144054734 | A | G | 222 | Mir3470b | ncRNA_intronic | . | . | . | 7qF5 | rs48118059 | 0 | 1 |
| chr7 | 144054796 | A | G | 222 | Mir3470b | ncRNA_intronic | . | . | . | 7qF5 | rs48623747 | 0 | 1 |
| chr7 | 144054973 | A | G | 158 | Mir3470b | ncRNA_intronic | . | . | . | 7qF5 | rs224504161 | 0 | 1 |
| chr7 | 144055063 | G | A | 107 | Mir3470b | ncRNA_intronic | . | . | . | 7qF5 | rs233824202 | 0 | 1 |
| chr7 | 144055065 | C | A | 107 | Mir3470b | ncRNA_intronic | . | . | . | 7qF5 | rs252852779 | 0 | 1 |
| chr7 | 144057645 | C | T | 72 | Mir3470b | ncRNA_intronic | . | . | . | 7qF5 | rs583850808 | 0 | 1 |
| chr7 | 144057673 | T | C | 113 | Mir3470b | ncRNA_intronic | . | . | . | 7qF5 | rs256689115 | 0 | 1 |
| chr7 | 144057714 | G | A | 222 | Mir3470b | ncRNA_intronic | . | . | . | 7qF5 | rs46259259 | 0 | 1 |
| chr7 | 144068720 | C | T | 137 | Mir3470b | ncRNA_intronic | . | . | . | 7qF5 | rs3719308 | 0 | 1 |
| chr7 | 144068727 | G | A | 139 | Mir3470b | ncRNA_intronic | . | . | . | 7qF5 | rs3719328 | 0 | 1 |
| chr7 | 144068751 | T | C | 182 | Mir3470b | ncRNA_intronic | . | . | . | 7qF5 | rs3719771 | 0 | 1 |
| chr7 | 144068987 | A | G | 87 | Mir3470b | ncRNA_intronic | . | . | . | 7qF5 | rs49768102 | 0 | 1 |
| chr7 | 144070018 | T | C | 68 | Mir3470b | ncRNA_intronic | . | . | . | 7qF5 | rs46175413 | 0 | 1 |
| chr7 | 144070089 | C | T | 113 | Mir3470b | ncRNA_intronic | . | . | . | 7qF5 | rs219743151 | 0 | 1 |
| chr7 | 144070215 | A | G | 179 | Mir3470b | ncRNA_intronic | . | . | . | 7qF5 | rs50739201 | 0 | 1 |
| chr7 | 144070232 | C | T | 149 | Mir3470b | ncRNA_intronic | . | . | . | 7qF5 | rs49213146 | 0 | 1 |
| chr7 | 144091633 | A | G | 133 | Mir3470b | ncRNA_intronic | . | . | . | 7qF5 | rs263215935 | 0 | 1 |
| chr7 | 144091644 | T | C | 190 | Mir3470b | ncRNA_intronic | . | . | . | 7qF5 | rs225569909 | 0 | 1 |
| chr7 | 144091662 | G | A | 222 | Mir3470b | ncRNA_intronic | . | . | . | 7qF5 | rs49636278 | 0 | 1 |
| chr7 | 144091805 | C | T | 222 | Mir3470b | ncRNA_intronic | . | . | . | 7qF5 | rs49508354 | 0 | 1 |
| chr7 | 144091838 | G | A | 193 | Mir3470b | ncRNA_intronic | . | . | . | 7qF5 | rs45995888 | 0 | 1 |
| chr7 | 144091854 | C | A | 95 | Mir3470b | ncRNA_intronic | . | . | . | 7qF5 | rs238788007 | 0 | 1 |
| chr7 | 144128111 | T | C | 69 | Mir3470b | ncRNA_intronic | . | . | . | 7qF5 | rs221170713 | 0 | 1 |
| chr7 | 144128317 | T | C | 222 | Mir3470b | ncRNA_intronic | . | . | . | 7qF5 | rs51944748 | 0 | 1 |
| chr7 | 144128418 | A | G | 101 | Mir3470b | ncRNA_intronic | . | . | . | 7qF5 | rs234031802 | 0 | 1 |
| chr7 | 144128433 | C | T | 94 | Mir3470b | ncRNA_intronic | . | . | . | 7qF5 | rs253002651 | 0 | 1 |
| chr7 | 144187097 | T | C | 55 | Mir3470b,Shank2 | ncRNA_intronic | . | . | . | 7qF5 | rs39341184 | 0 | 1 |
| chr7 | 144192576 | T | C | 38.8209 | Shank2,Mir3470b | ncRNA_intronic | . | . | . | 7qF5 | rs37868487 | 0 | 1 |
| chr7 | 144259178 | T | C | 222 | Shank2,Mir3470b | ncRNA_intronic | . | . | . | 7qF5 | rs37766116 | 0 | 1 |
| chr7 | 144259209 | A | G | 164 | Shank2,Mir3470b | ncRNA_intronic | . | . | . | 7qF5 | rs214700476 | 0 | 1 |
| chr7 | 144286571 | G | A | 186 | Mir3470b,Shank2 | ncRNA_intronic | . | . | . | 7qF5 | rs212390804 | 0 | 1 |
| chr7 | 144286643 | C | T | 222 | Mir3470b,Shank2 | exonic | synonymous SNV | Shank2:NM_001081370:exon2:c.C171T:p.A57A,Shank2:NM_001113373:exon7:c.C801T:p.A267A | . | 7qF5 | rs230978936 | 0 | 1 |
| chr7 | 144395373 | C | T | 111 | Mir3470b,Shank2 | ncRNA_intronic | . | . | . | 7qF5 | rs50643648 | 0 | 1 |
| chr7 | 144395785 | G | A | 84 | Shank2,Mir3470b | ncRNA_intronic | . | . | . | 7qF5 | rs215248889 | 0 | 1 |
| chr7 | 144395794 | A | C | 82 | Shank2,Mir3470b | ncRNA_intronic | . | . | . | 7qF5 | rs238011432 | 0 | 1 |
| chr7 | 144395796 | G | A | 82 | Mir3470b,Shank2 | ncRNA_intronic | . | . | . | 7qF5 | rs253786939 | 0 | 1 |
| chr7 | 144396488 | T | A | 111 | Mir3470b,Shank2 | ncRNA_intronic | . | . | . | 7qF5 | rs222744789 | 0 | 1 |
| chr7 | 144398219 | C | T | 205 | Mir3470b,Shank2 | ncRNA_intronic | . | . | . | 7qF5 | rs228221356 | 0 | 1 |
| chr7 | 144398335 | A | G | 83 | Mir3470b,Shank2 | ncRNA_intronic | . | . | . | 7qF5 | rs247359088 | 0 | 1 |
| chr7 | 144407981 | C | T | 222 | Mir3470b,Shank2 | ncRNA_intronic | . | . | . | 7qF5 | rs217613784 | 0 | 1 |
| chr7 | 144408094 | C | T | 222 | Shank2,Mir3470b | ncRNA_intronic | . | . | . | 7qF5 | rs239977556 | 0 | 1 |
| chr7 | 144409589 | T | A | 32.9694 | Shank2,Mir3470b | exonic | synonymous SNV | Shank2:NM_001081370:exon10:c.T933A:p.P311P,Shank2:NM_001113373:exon15:c.T1563A:p.P521P | . | 7qF5 | rs241168499 | 0 | 1 |
| chr7 | 144409764 | A | C | 222 | Shank2,Mir3470b | exonic | synonymous SNV | Shank2:NM_001081370:exon10:c.A1108C:p.R370R,Shank2:NM_001113373:exon15:c.A1738C:p.R580R | . | 7qF5 | rs37957232 | 0 | 1 |
| chr7 | 144409946 | C | T | 221 | Mir3470b,Shank2 | exonic | synonymous SNV | Shank2:NM_001081370:exon10:c.C1290T:p.N430N,Shank2:NM_001113373:exon15:c.C1920T:p.N640N | . | 7qF5 | rs36423900 | 0 | 1 |
| chr7 | 144410552 | A | C | 190 | Mir3470b,Shank2 | exonic | synonymous SNV | Shank2:NM_001081370:exon10:c.A1896C:p.S632S,Shank2:NM_001113373:exon15:c.A2526C:p.S842S | . | 7qF5 | rs247034115 | 0 | 1 |
| chr7 | 144410594 | A | G | 167 | Mir3470b,Shank2 | exonic | synonymous SNV | Shank2:NM_001081370:exon10:c.A1938G:p.K646K,Shank2:NM_001113373:exon15:c.A2568G:p.K856K | . | 7qF5 | rs257464330 | 0 | 1 |
| chr7 | 144410612 | T | C | 155 | Mir3470b,Shank2 | exonic | synonymous SNV | Shank2:NM_001081370:exon10:c.T1956C:p.A652A,Shank2:NM_001113373:exon15:c.T2586C:p.A862A | . | 7qF5 | rs227800681 | 0 | 1 |
| chr7 | 144410735 | C | T | 104 | Mir3470b,Shank2 | exonic | synonymous SNV | Shank2:NM_001081370:exon10:c.C2079T:p.Y693Y,Shank2:NM_001113373:exon15:c.C2709T:p.Y903Y | . | 7qF5 | rs38184178 | 0 | 1 |
| chr7 | 144410741 | A | G | 79 | Shank2,Mir3470b | exonic | synonymous SNV | Shank2:NM_001081370:exon10:c.A2085G:p.T695T,Shank2:NM_001113373:exon15:c.A2715G:p.T905T | . | 7qF5 | rs260237012 | 0 | 1 |
| chr7 | 144410765 | G | A | 32.5182 | Mir3470b,Shank2 | exonic | synonymous SNV | Shank2:NM_001081370:exon10:c.G2109A:p.A703A,Shank2:NM_001113373:exon15:c.G2739A:p.A913A | . | 7qF5 | rs228688419 | 0 | 1 |
| chr7 | 144410768 | C | A | 34.5153 | Mir3470b,Shank2 | exonic | missense SNV | Shank2:NM_001081370:exon10:c.C2112A:p.D704E,Shank2:NM_001113373:exon15:c.C2742A:p.D914E | . | 7qF5 | rs36708307 | 0 | 1 |
| chr7 | 144410993 | C | T | 90 | Shank2,Mir3470b | exonic | synonymous SNV | Shank2:NM_001081370:exon10:c.C2337T:p.A779A,Shank2:NM_001113373:exon15:c.C2967T:p.A989A | . | 7qF5 | rs36353627 | 0 | 1 |
| chr7 | 144411225 | T | C | 134 | Mir3470b,Shank2 | exonic | missense SNV | Shank2:NM_001081370:exon10:c.T2569C:p.S857P,Shank2:NM_001113373:exon15:c.T3199C:p.S1067P | . | 7qF5 | rs229463675 | 0 | 1 |
| chr7 | 144411654 | G | A | 222 | Mir3470b,Shank2 | exonic | missense SNV | Shank2:NM_001081370:exon10:c.G2998A:p.A1000T,Shank2:NM_001113373:exon15:c.G3628A:p.A1210T | . | 7qF5 | rs245510419 | 0 | 1 |
| chr7 | 144421238 | A | G | 167 | Shank2,Mir3470b | ncRNA_intronic | . | . | . | 7qF5 | rs245260875 | 0 | 1 |
| chr7 | 144436859 | A | G | 81 | Cttn,Mir3470b | ncRNA_intronic | . | . | . | 7qF5 | rs246679163 | 0 | 1 |
| chr7 | 144436943 | C | T | 99 | Mir3470b,Cttn | ncRNA_intronic | . | . | . | 7qF5 | rs225613065 | 0 | 1 |
| chr7 | 144437139 | A | G | 221 | Cttn,Mir3470b | ncRNA_intronic | . | . | . | 7qF5 | rs31488022 | 0 | 1 |
| chr7 | 144438853 | T | C | 96 | Mir3470b,Cttn | exonic | missense SNV | Cttn:NM_001252572:exon15:c.A1234G:p.I412V,Cttn:NM_007803:exon16:c.A1345G:p.I449V | . | 7qF5 | rs38335552 | 0 | 1 |
| chr7 | 144438878 | A | G | 94 | Cttn,Mir3470b | exonic | synonymous SNV | Cttn:NM_001252572:exon15:c.T1209C:p.P403P,Cttn:NM_007803:exon16:c.T1320C:p.P440P | . | 7qF5 | rs37426976 | 0 | 1 |
| chr7 | 144439027 | T | G | 81 | Cttn,Mir3470b | ncRNA_intronic | . | . | . | 7qF5 | rs38935427 | 0 | 1 |
| chr7 | 144439056 | A | G | 80 | Mir3470b,Cttn | ncRNA_intronic | . | . | . | 7qF5 | rs221344642 | 0 | 1 |
| chr7 | 144439919 | A | G | 178 | Mir3470b,Cttn | ncRNA_intronic | . | . | . | 7qF5 | rs241378838 | 0 | 1 |
| chr7 | 144445761 | T | C | 222 | Cttn | intronic | . | . | . | 7qF5 | rs36468236 | 0 | 1 |
| chr7 | 144445971 | C | T | 124 | Cttn | intronic | . | . | . | 7qF5 | rs234588161 | 0 | 1 |
| chr7 | 144449379 | G | C | 102 | Cttn | intronic | . | . | . | 7qF5 | rs37417134 | 0 | 1 |
| chr7 | 144449526 | G | A | 222 | Cttn | intronic | . | . | . | 7qF5 | rs37629261 | 0 | 1 |
| chr7 | 144449661 | G | A | 222 | Cttn | intronic | . | . | . | 7qF5 | rs37884394 | 0 | 1 |
| chr7 | 144449720 | G | A | 166 | Cttn | intronic | . | . | . | 7qF5 | rs38990524 | 0 | 1 |
| chr7 | 144451161 | G | A | 22.9853 | Cttn | intronic | . | . | . | 7qF5 | rs36692049 | 0 | 1 |
| chr7 | 144452696 | G | C | 222 | Cttn | intronic | . | . | . | 7qF5 | rs264323253 | 0 | 1 |
| chr7 | 144454599 | A | G | 119 | Cttn | intronic | . | . | . | 7qF5 | rs36790876 | 0 | 1 |
| chr7 | 144454874 | G | A | 126 | Cttn | intronic | . | . | . | 7qF5 | rs37247397 | 0 | 1 |
| chr7 | 144456596 | T | C | 149 | Cttn | intronic | . | . | . | 7qF5 | rs38413079 | 0 | 1 |
| chr7 | 144457576 | C | A | 144 | Cttn | intronic | . | . | . | 7qF5 | rs36636279 | 0 | 1 |
| chr7 | 144457652 | T | C | 192 | Cttn | intronic | . | . | . | 7qF5 | rs245178092 | 0 | 1 |
| chr7 | 144457655 | T | C | 198 | Cttn | intronic | . | . | . | 7qF5 | rs37062294 | 0 | 1 |
| chr7 | 144457738 | G | A | 222 | Cttn | exonic | synonymous SNV | Cttn:NM_001252572:exon6:c.C333T:p.H111H,Cttn:NM_007803:exon6:c.C333T:p.H111H | . | 7qF5 | rs37969206 | 0 | 1 |
| chr7 | 144461323 | A | G | 130 | Cttn | intronic | . | . | . | 7qF5 | rs263511181 | 0 | 1 |
| chr7 | 144463153 | C | T | 96 | Cttn | intronic | . | . | . | 7qF5 | rs37137037 | 0 | 1 |
| chr7 | 144463242 | C | T | 211 | Cttn | intronic | . | . | . | 7qF5 | rs263259546 | 0 | 1 |
| chr7 | 144463842 | C | T | 169 | Cttn | intronic | . | . | . | 7qF5 | rs239012035 | 0 | 1 |
| chr7 | 144463877 | C | G | 222 | Cttn | intronic | . | . | . | 7qF5 | rs37269223 | 0 | 1 |
| chr7 | 144464093 | A | G | 61 | Cttn | intronic | . | . | . | 7qF5 | rs39577876 | 0 | 1 |
| chr7 | 144477834 | T | C | 222 | Ppfia1 | UTR3 | . | . | . | 7qF5 | rs37956400 | 0 | 1 |
| chr7 | 144477941 | A | G | 222 | Ppfia1 | UTR3 | . | . | . | 7qF5 | rs13460347 | 0 | 1 |
| chr7 | 144478129 | A | G | 143 | Ppfia1 | intronic | . | . | . | 7qF5 | rs50247438 | 0 | 1 |
| chr7 | 144481045 | T | C | 134 | Ppfia1 | intronic | . | . | . | 7qF5 | rs31378198 | 0 | 1 |
| chr7 | 144481170 | G | A | 222 | Ppfia1 | exonic | synonymous SNV | Ppfia1:NM_001033319:exon29:c.C3582T:p.G1194G,Ppfia1:NM_001195086:exon30:c.C3657T:p.G1219G | . | 7qF5 | rs36820950 | 0 | 1 |
| chr7 | 144481605 | C | T | 159 | Ppfia1 | intronic | . | . | . | 7qF5 | rs36517637 | 0 | 1 |
| chr7 | 144481975 | G | A | 109 | Ppfia1 | intronic | . | . | . | 7qF5 | rs37698301 | 0 | 1 |
| chr7 | 144484655 | G | A | 86 | Ppfia1 | intronic | . | . | . | 7qF5 | rs36519773 | 0 | 1 |
| chr7 | 144484831 | G | A | 222 | Ppfia1 | intronic | . | . | . | 7qF5 | rs36875900 | 0 | 1 |
| chr7 | 144485085 | A | G | 222 | Ppfia1 | exonic | synonymous SNV | Ppfia1:NM_001033319:exon25:c.T3106C:p.L1036L,Ppfia1:NM_001195086:exon26:c.T3181C:p.L1061L | . | 7qF5 | rs13460346 | 0 | 1 |
| chr7 | 144485292 | T | C | 201 | Ppfia1 | intronic | . | . | . | 7qF5 | rs38250405 | 0 | 1 |
| chr7 | 144488565 | G | A | 164 | Ppfia1 | intronic | . | . | . | 7qF5 | rs36616000 | 0 | 1 |
| chr7 | 144491527 | A | G | 222 | Ppfia1 | exonic | synonymous SNV | Ppfia1:NM_001033319:exon22:c.T2817C:p.H939H,Ppfia1:NM_001195086:exon23:c.T2892C:p.H964H | . | 7qF5 | rs13460345 | 0 | 1 |
| chr7 | 144491879 | A | C | 21.5601 | Ppfia1 | intronic | . | . | . | 7qF5 | rs36397402 | 0 | 1 |
| chr7 | 144500237 | A | G | 93 | Ppfia1 | intronic | . | . | . | 7qF5 | . | 0 | 1 |
| chr7 | 144500293 | T | C | 130 | Ppfia1 | exonic | synonymous SNV | Ppfia1:NM_001033319:exon18:c.A2301G:p.A767A,Ppfia1:NM_001195086:exon19:c.A2376G:p.A792A | . | 7qF5 | rs31803764 | 0 | 1 |
| chr7 | 144502769 | G | A | 173 | Ppfia1 | intronic | . | . | . | 7qF5 | rs31847678 | 0 | 1 |
| chr7 | 144502815 | A | T | 222 | Ppfia1 | intronic | . | . | . | 7qF5 | rs31275895 | 0 | 1 |
| chr7 | 144505104 | T | C | 190 | Ppfia1 | exonic | synonymous SNV | Ppfia1:NM_001033319:exon16:c.A1959G:p.E653E,Ppfia1:NM_001195086:exon17:c.A2034G:p.E678E | . | 7qF5 | rs36657434 | 0 | 1 |
| chr7 | 144508213 | T | C | 222 | Ppfia1 | exonic | synonymous SNV | Ppfia1:NM_001033319:exon14:c.A1620G:p.G540G,Ppfia1:NM_001195086:exon15:c.A1695G:p.G565G | . | 7qF5 | rs37177434 | 0 | 1 |
| chr7 | 144510096 | T | C | 84 | Ppfia1 | intronic | . | . | . | 7qF5 | rs36448245 | 0 | 1 |
| chr7 | 144510798 | T | C | 222 | Ppfia1 | intronic | . | . | . | 7qF5 | rs37804059 | 0 | 1 |
| chr7 | 144513563 | T | C | 37.7788 | Ppfia1 | intronic | . | . | . | 7qF5 | rs39007937 | 0 | 1 |
| chr7 | 144515877 | G | A | 37.7106 | Ppfia1 | intronic | . | . | . | 7qF5 | rs37309958 | 0 | 1 |
| chr7 | 144516034 | C | G | 222 | Ppfia1 | exonic | synonymous SNV | Ppfia1:NM_001033319:exon9:c.G1161C:p.L387L,Ppfia1:NM_001195086:exon9:c.G1161C:p.L387L | . | 7qF5 | rs37596154 | 0 | 1 |
| chr7 | 144517772 | C | T | 214 | Ppfia1 | intronic | . | . | . | 7qF5 | rs37946495 | 0 | 1 |
| chr7 | 144517858 | G | A | 32.941 | Ppfia1 | intronic | . | . | . | 7qF5 | rs36791215 | 0 | 1 |
| chr7 | 144518993 | C | T | 76 | Ppfia1 | intronic | . | . | . | 7qF5 | rs37546442 | 0 | 1 |
| chr7 | 144519112 | T | C | 210 | Ppfia1 | intronic | . | . | . | 7qF5 | rs36482730 | 0 | 1 |
| chr7 | 144519492 | T | C | 222 | Ppfia1 | intronic | . | . | . | 7qF5 | rs50034912 | 0 | 1 |
| chr7 | 144520584 | C | T | 160 | Ppfia1 | intronic | . | . | . | 7qF5 | rs229539639 | 0 | 1 |
| chr7 | 144520724 | T | C | 222 | Ppfia1 | exonic | synonymous SNV | Ppfia1:NM_001033319:exon4:c.A432G:p.V144V,Ppfia1:NM_001195086:exon4:c.A432G:p.V144V | . | 7qF5 | rs37085443 | 0 | 1 |
| chr7 | 144520837 | C | T | 222 | Ppfia1 | intronic | . | . | . | 7qF5 | rs36473044 | 0 | 1 |
| chr7 | 144520948 | C | T | 222 | Ppfia1 | intronic | . | . | . | 7qF5 | rs31689692 | 0 | 1 |
| chr7 | 144580439 | G | A | 136 | Fadd | UTR3 | . | . | . | 7qF5 | rs241078156 | 0 | 1 |
| chr7 | 144580661 | A | G | 222 | Fadd | exonic | synonymous SNV | Fadd:NM_010175:exon2:c.T486C:p.V162V | . | 7qF5 | rs13472364 | 0 | 1 |
| chr7 | 144580920 | C | T | 222 | Fadd | intronic | . | . | . | 7qF5 | rs6180293 | 0 | 1 |
| chr7 | 144581022 | A | C | 222 | Fadd | intronic | . | . | . | 7qF5 | rs6181272 | 0 | 1 |
| chr7 | 144581114 | G | A | 222 | Fadd | intronic | . | . | . | 7qF5 | rs6181421 | 0 | 1 |
| chr7 | 144581137 | C | T | 222 | Fadd | intronic | . | . | . | 7qF5 | rs6181885 | 0 | 1 |
| chr7 | 144581310 | T | C | 222 | Fadd | intronic | . | . | . | 7qF5 | rs6182504 | 0 | 1 |
| chr7 | 144581386 | T | A | 197 | Fadd | intronic | . | . | . | 7qF5 | rs38882827 | 0 | 1 |
| chr7 | 144589947 | A | G | 32.9694 | Ano1 | UTR3 | . | . | . | 7qF5 | rs261283211 | 0 | 1 |
| chr7 | 144590005 | C | T | 72 | Ano1 | exonic | synonymous SNV | Ano1:NM_001242349:exon25:c.G3039A:p.A1013A,Ano1:NM_178642:exon26:c.G3048A:p.A1016A | . | 7qF5 | rs213418220 | 0 | 1 |
| chr7 | 144590312 | A | G | 222 | Ano1 | intronic | . | . | . | 7qF5 | rs231945261 | 0 | 1 |
| chr7 | 144590343 | G | A | 201 | Ano1 | intronic | . | . | . | 7qF5 | rs49935278 | 0 | 1 |
| chr7 | 144595372 | A | G | 171 | Ano1 | intronic | . | . | . | 7qF5 | rs31093556 | 0 | 1 |
| chr7 | 144595703 | A | G | 209 | Ano1 | intronic | . | . | . | 7qF5 | rs31826727 | 0 | 1 |
| chr7 | 144597258 | C | T | 222 | Ano1 | intronic | . | . | . | 7qF5 | rs225905531 | 0 | 1 |
| chr7 | 144597311 | T | A | 217 | Ano1 | intronic | . | . | . | 7qF5 | rs251659647 | 0 | 1 |
| chr7 | 144597340 | G | C | 150 | Ano1 | intronic | . | . | . | 7qF5 | rs261665402 | 0 | 1 |

| | | | | | | | | | | | | | |
|---|---|---|---|---|---|---|---|---|---|---|---|---|---|
| chr7 | 144603424 | T | A | 200 | Ano1 | intronic | . | . | . | 7qF5 | rs218613439 | 0 | 1 |
| chr7 | 144603657 | A | G | 210 | Ano1 | intronic | . | . | . | 7qF5 | rs238614396 | 0 | 1 |
| chr7 | 144603707 | A | T | 81 | Ano1 | intronic | . | . | . | 7qF5 | rs258082263 | 0 | 1 |
| chr7 | 144603727 | C | T | 38.8323 | Ano1 | intronic | . | . | . | 7qF5 | rs220821435 | 0 | 1 |
| chr7 | 144607937 | C | T | 144 | Ano1 | exonic | synonymous SNV | Ano1:NM_001242349:exon20:c.G2262A:p.T754T,Ano1:NM_178642:exon21:c.G2271A:p.T757T | . | 7qF5 | rs36716543 | 0 | 1 |
| chr7 | 144608116 | C | G | 56 | Ano1 | intronic | . | . | . | 7qF5 | rs260080655 | 0 | 1 |
| chr7 | 144608978 | A | G | 182 | Ano1 | intronic | . | . | . | 7qF5 | rs232490304 | 0 | 1 |
| chr7 | 144610838 | A | G | 185 | Ano1 | intronic | . | . | . | 7qF5 | rs31666178 | 0 | 1 |
| chr7 | 144610882 | A | G | 214 | Ano1 | exonic | synonymous SNV | Ano1:NM_001242349:exon18:c.T1995C:p.G665G,Ano1:NM_178642:exon19:c.T2004C:p.G668G | . | 7qF5 | rs36493951 | 0 | 1 |
| chr7 | 144611005 | G | A | 92 | Ano1 | intronic | . | . | . | 7qF5 | rs38547267 | 0 | 1 |
| chr7 | 144611165 | T | C | 102 | Ano1 | intronic | . | . | . | 7qF5 | rs39197228 | 0 | 1 |
| chr7 | 144611283 | G | C | 222 | Ano1 | intronic | . | . | . | 7qF5 | rs37164796 | 0 | 1 |
| chr7 | 144611382 | A | G | 222 | Ano1 | exonic | synonymous SNV | Ano1:NM_001242349:exon17:c.T1929C:p.F643F,Ano1:NM_178642:exon18:c.T1938C:p.F646F | . | 7qF5 | rs36562043 | 0 | 1 |
| chr7 | 144611525 | A | G | 222 | Ano1 | intronic | . | . | . | 7qF5 | rs266163979 | 0 | 1 |
| chr7 | 144611573 | T | C | 222 | Ano1 | intronic | . | . | . | 7qF5 | rs220620045 | 0 | 1 |
| chr7 | 144611732 | C | T | 222 | Ano1 | exonic | synonymous SNV | Ano1:NM_001242349:exon16:c.G1746A:p.P582P,Ano1:NM_178642:exon17:c.G1755A:p.P585P | . | 7qF5 | . | 0 | 1 |
| chr7 | 144614673 | T | G | 26.7648 | Ano1 | intronic | . | . | . | 7qF5 | rs37595744 | 0 | 1 |
| chr7 | 144614761 | T | A | 64 | Ano1 | intronic | . | . | . | 7qF5 | rs220413543 | 0 | 1 |
| chr7 | 144614766 | C | T | 76 | Ano1 | intronic | . | . | . | 7qF5 | rs248056779 | 0 | 1 |
| chr7 | 144614810 | C | T | 173 | Ano1 | intronic | . | . | . | 7qF5 | rs259718763 | 0 | 1 |
| chr7 | 144615068 | C | G | 130 | Ano1 | intronic | . | . | . | 7qF5 | . | 0 | 1 |
| chr7 | 144618752 | T | C | 183 | Ano1 | intronic | . | . | . | 7qF5 | rs245020247 | 0 | 1 |
| chr7 | 144618757 | A | G | 183 | Ano1 | intronic | . | . | . | 7qF5 | rs214380545 | 0 | 1 |
| chr7 | 144619369 | A | G | 171 | Ano1 | intronic | . | . | . | 7qF5 | rs218284384 | 0 | 1 |
| chr7 | 144619498 | C | A | 222 | Ano1 | intronic | . | . | . | 7qF5 | rs240529306 | 0 | 1 |
| chr7 | 144633489 | C | G | 58 | Ano1 | intronic | . | . | . | 7qF5 | rs250430889 | 0 | 1 |
| chr7 | 144633537 | C | T | 155 | Ano1 | intronic | . | . | . | 7qF5 | rs36454643 | 0 | 1 |
| chr7 | 144633772 | A | G | 216 | Ano1 | intronic | . | . | . | 7qF5 | rs224436054 | 0 | 1 |
| chr7 | 144633821 | T | C | 94 | Ano1 | intronic | . | . | . | 7qF5 | rs38384960 | 0 | 1 |
| chr7 | 144636985 | A | C | 130 | Ano1 | intronic | . | . | . | 7qF5 | rs235574357 | 0 | 1 |
| chr7 | 144637011 | G | A | 166 | Ano1 | intronic | . | . | . | 7qF5 | rs36286615 | 0 | 1 |
| chr7 | 144637228 | G | A | 180 | Ano1 | intronic | . | . | . | 7qF5 | rs218299430 | 0 | 1 |
| chr7 | 144637243 | C | T | 172 | Ano1 | intronic | . | . | . | 7qF5 | rs238269579 | 0 | 1 |
| chr7 | 144638317 | G | T | 101 | Ano1 | intronic | . | . | . | 7qF5 | rs37074862 | 0 | 1 |
| chr7 | 144638318 | C | T | 101 | Ano1 | intronic | . | . | . | 7qF5 | rs212191171 | 0 | 1 |
| chr7 | 144638408 | A | G | 222 | Ano1 | intronic | . | . | . | 7qF5 | rs36807107 | 0 | 1 |
| chr7 | 144638576 | A | G | 222 | Ano1 | intronic | . | . | . | 7qF5 | rs262093707 | 0 | 1 |
| chr7 | 144638592 | G | C | 157 | Ano1 | intronic | . | . | . | 7qF5 | rs223646266 | 0 | 1 |
| chr7 | 144644600 | G | A | 58 | Ano1 | intronic | . | . | . | 7qF5 | rs243174510 | 0 | 1 |
| chr7 | 144644616 | A | G | 60 | Ano1 | intronic | . | . | . | 7qF5 | rs265280957 | 0 | 1 |
| chr7 | 144644651 | A | G | 141 | Ano1 | intronic | . | . | . | 7qF5 | rs231684912 | 0 | 1 |
| chr7 | 144644682 | C | T | 172 | Ano1 | intronic | . | . | . | 7qF5 | rs246070485 | 0 | 1 |
| chr7 | 144644763 | C | T | 222 | Ano1 | intronic | . | . | . | 7qF5 | rs216806522 | 0 | 1 |
| chr7 | 144644841 | C | T | 191 | Ano1 | intronic | . | . | . | 7qF5 | rs37254757 | 0 | 1 |
| chr7 | 144647972 | A | G | 222 | Ano1 | intronic | . | . | . | 7qF5 | rs259123217 | 0 | 1 |
| chr7 | 144654331 | T | C | 222 | Ano1 | intronic | . | . | . | 7qF5 | rs32156818 | 0 | 1 |
| chr7 | 144655655 | G | A | 222 | Ano1 | exonic | synonymous SNV | Ano1:NM_001242349:exon5:c.C810T:p.H270H,Ano1:NM_178642:exon5:c.C807T:p.H269H | . | 7qF5 | rs252052521 | 0 | 1 |
| chr7 | 144655781 | T | C | 222 | Ano1 | intronic | . | . | . | 7qF5 | rs212579368 | 0 | 1 |
| chr7 | 144657003 | G | A | 222 | Ano1 | intronic | . | . | . | 7qF5 | rs38133333 | 0 | 1 |
| chr7 | 144657153 | A | G | 222 | Ano1 | intronic | . | . | . | 7qF5 | rs222676279 | 0 | 1 |
| chr7 | 144657175 | A | C | 191 | Ano1 | intronic | . | . | . | 7qF5 | rs32290785 | 0 | 1 |
| chr7 | 144669281 | C | T | 111 | Ano1 | intronic | . | . | . | 7qF5 | rs240492526 | 0 | 1 |
| chr7 | 144669649 | G | A | 222 | Ano1 | exonic | synonymous SNV | Ano1:NM_001242349:exon3:c.C309T:p.P103P,Ano1:NM_178642:exon3:c.C306T:p.P102P | . | 7qF5 | rs36517239 | 0 | 1 |
| chr7 | 144763169 | G | A | 136 | . | intergenic | . | . | . | 7qF5 | rs224297749 | 1 | 0 |
| chr7 | 144763200 | G | T | 129 | . | intergenic | . | . | . | 7qF5 | rs36516028 | 0 | 1 |
| chr7 | 144763635 | A | G | 222 | . | intergenic | . | . | . | 7qF5 | rs37725907 | 0 | 1 |
| chr7 | 144763870 | G | A | 222 | . | intergenic | . | . | . | 7qF5 | rs36573150 | 0 | 1 |
| chr7 | 144764015 | G | A | 222 | . | intergenic | . | . | . | 7qF5 | rs257652653 | 0 | 1 |
| chr7 | 144764025 | A | T | 222 | . | intergenic | . | . | . | 7qF5 | rs217297761 | 0 | 1 |
| chr7 | 144764103 | C | G | 222 | . | intergenic | . | . | . | 7qF5 | rs38051801 | 0 | 1 |
| chr7 | 144764168 | C | T | 222 | . | intergenic | . | . | . | 7qF5 | rs37324262 | 0 | 1 |
| chr7 | 144764236 | A | C | 222 | . | intergenic | . | . | . | 7qF5 | rs222347195 | 0 | 1 |
| chr7 | 144764297 | T | C | 222 | . | intergenic | . | . | . | 7qF5 | rs37170636 | 0 | 1 |
| chr7 | 144764370 | C | T | 222 | . | intergenic | . | . | . | 7qF5 | rs256030762 | 0 | 1 |
| chr7 | 144791928 | T | C | 105 | . | intergenic | . | . | . | 7qF5 | rs36936681 | 0 | 1 |
| chr7 | 144792330 | T | C | 137 | . | intergenic | . | . | . | 7qF5 | rs47375806 | 0 | 1 |
| chr7 | 144840461 | A | T | 53 | Fgf3 | intronic | . | . | . | 7qF5 | rs214850307 | 0 | 1 |
| chr7 | 144840803 | T | G | 222 | Fgf3 | intronic | . | . | . | 7qF5 | rs238384758 | 0 | 1 |
| chr7 | 144840868 | T | G | 157 | Fgf3 | intronic | . | . | . | 7qF5 | rs258934218 | 0 | 1 |
| chr7 | 144842428 | G | A | 79 | Fgf3 | intronic | . | . | . | 7qF5 | rs50447985 | 0 | 1 |
| chr7 | 144842922 | G | A | 190 | Fgf3 | exonic | missense SNV | Fgf3:NM_008007:exon3:c.G672A:p.M224I | . | 7qF5 | rs219466677 | 0 | 1 |
| chr7 | 144916283 | G | A | 128 | Oraov1 | intronic | . | . | . | 7qF5 | . | 0 | 1 |
| chr7 | 145280238 | G | A | 222 | Tpcn2 | exonic | synonymous SNV | Tpcn2:NM_146206:exon2:c.C117T:p.D39D | . | 7qF5 | . | 0 | 1 |
| chr8 | 3387976 | A | T | 176 | A430078G23Rik | intronic | . | . | . | 8qA1.1 | . | 0 | 1 |
| chr8 | 3387977 | C | A | 177 | A430078G23Rik | intronic | . | . | . | 8qA1.1 | . | 0 | 1 |
| chr8 | 3511879 | T | A | 222 | Mcoln1 | intronic | . | . | . | 8qA1.1 | . | 0 | 1 |
| chr8 | 3511880 | G | T | 222 | Mcoln1 | intronic | . | . | . | 8qA1.1 | . | 0 | 1 |
| chr8 | 3616128 | G | T | 222 | Xab2 | exonic | missense SNV | Xab2:NM_026156:exon6:c.C688A:p.Q230K | . | 8qA1.1 | . | 0 | 1 |
| chr8 | 3877892 | T | C | 221 | Cd209d | exonic | missense SNV | Cd209d:NM_130904:exon3:c.A211G:p.I71V | . | 8qA1.1 | . | 0 | 1 |
| chr8 | 3921864 | C | T | 180 | Cd209b | intronic | . | . | . | 8qA1.1 | . | 0 | 1 |
| chr8 | 3995964 | C | A | 222 | . | intergenic | . | . | . | 8qA1.1 | . | 0 | 1 |
| chr8 | 3997678 | A | G | 108 | . | intergenic | . | . | . | 8qA1.1 | . | 0 | 1 |
| chr8 | 4235619 | C | A | 222 | Lrrc8e | exonic | missense SNV | Lrrc8e:NM_028175:exon3:c.C1843A:p.H615N | . | 8qA1.1 | . | 0 | 1 |
| chr8 | 4243242 | C | T | 28.7209 | Map2k7 | intronic | . | . | . | 8qA1.1 | . | 0 | 1 |
| chr8 | 4861349 | A | G | 221 | . | intergenic | . | . | . | 8qA1.1 | . | 0 | 1 |
| chr8 | 5640766 | G | T | 221 | . | intergenic | . | . | . | 8qA1.1 | . | 0 | 1 |
| chr8 | 9770981 | T | C | 131 | Fam155a | exonic | missense SNV | Fam155a:NM_173446:exon1:c.A38G:p.D13G | . | 8qA1.1 | . | 0 | 1 |
| chr8 | 9989941 | G | T | 221 | Abhd13 | UTR3 | . | . | . | 8qA1.1 | . | 0 | 1 |
| chr8 | 10021429 | G | T | 222 | Tnfsf13b | exonic | stopgain | Tnfsf13b:NM_033622:exon4:c.G553T:p.G185X | . | 8qA1.1 | . | 0 | 1 |
| chr8 | 10272903 | A | C | 32.4083 | Myo16 | intronic | . | . | . | 8qA1.1 | . | 0 | 1 |
| chr8 | 10373273 | A | T | 135 | Myo16 | intronic | . | . | . | 8qA1.1 | rs32667625 | 1 | 0 |
| chr8 | 12713867 | T | G | 217 | Gm15348 | ncRNA_splicing | . | . | . | 8qA1.1 | . | 0 | 1 |
| chr8 | 12827618 | G | A | 222 | Atp11a | intronic | . | . | . | 8qA1.1 | . | 0 | 1 |
| chr8 | 13003549 | A | C | 228 | Mcf2l | intronic | . | . | . | 8qA1.1 | rs33424492 | 1 | 0 |
| chr8 | 13012935 | G | T | 221 | Mcf2l | intronic | . | . | . | 8qA1.1 | . | 0 | 1 |
| chr8 | 13140248 | C | T | 221 | Cul4a | exonic | missense SNV | Cul4a:NM_146207:exon16:c.C1684T:p.H562Y | . | 8qA1.1 | . | 0 | 1 |
| chr8 | 13878231 | C | T | 222 | Champ1 | exonic | missense SNV | Champ1:NM_181854:exon2:c.C388T:p.L130F | . | 8qA1.1 | . | 0 | 1 |
| chr8 | 14179788 | T | C | 89 | Dlgap2 | intronic | . | . | . | 8qA1.1 | . | 0 | 1 |
| chr8 | 15109061 | C | T | 218 | Myom2 | intronic | . | . | . | 8qA1.1 | . | 0 | 1 |
| chr8 | 16037293 | G | A | 222 | Csmd1 | exonic | missense SNV | Csmd1:NM_053171:exon38:c.C5741T:p.A1914V | . | 8qA1.2 | . | 0 | 1 |
| chr8 | 16068785 | C | A | 72 | Csmd1 | intronic | . | . | . | 8qA1.2 | . | 0 | 1 |
| chr8 | 16185438 | A | C | 169 | Csmd1 | intronic | . | . | . | 8qA1.2 | . | 0 | 1 |
| chr8 | 16232936 | T | C | 221 | Csmd1 | exonic | missense SNV | Csmd1:NM_053171:exon14:c.A2005G:p.I669V | . | 8qA1.2 | . | 0 | 1 |
| chr8 | 16271632 | A | G | 209 | Csmd1 | exonic | missense SNV | Csmd1:NM_053171:exon12:c.T1451C:p.L484P | . | 8qA1.2 | . | 0 | 1 |
| chr8 | 19038314 | C | T | 222 | . | intergenic | . | . | . | 8qA1.3 | . | 0 | 1 |
| chr8 | 19374012 | T | C | 221 | . | intergenic | . | . | . | 8qA1.3 | . | 0 | 1 |
| chr8 | 19493337 | C | G | 40.7707 | . | intergenic | . | . | . | 8qA1.3 | rs108679312 | 0 | 1 |
| chr8 | 19493387 | G | T | 222 | . | intergenic | . | . | . | 8qA1.3 | . | 0 | 1 |
| chr8 | 21885413 | T | G | 221 | Defb9 | UTR5 | . | . | . | 8qA2 | . | 0 | 1 |
| chr8 | 22002701 | G | A | 222 | Atp7b | intronic | . | . | . | 8qA2 | . | 0 | 1 |
| chr8 | 22027669 | G | A | 221 | Atp7b | exonic | synonymous SNV | Atp7b:NM_007511:exon2:c.C1152T:p.S384S | . | 8qA2 | . | 0 | 1 |
| chr8 | 22095255 | G | T | 222 | Nek5 | exonic | missense SNV | Nek5:NM_177898:exon13:c.C1105A:p.H369N | . | 8qA2 | . | 0 | 1 |
| chr8 | 22206650 | T | A | 68 | Vps36 | intronic | . | . | . | 8qA2 | . | 0 | 1 |
| chr8 | 22327582 | T | G | 110 | Tpte | intronic | . | . | . | 8qA2 | rs32925949 | 1 | 0 |
| chr8 | 22359315 | G | T | 222 | Tpte | intronic | . | . | . | 8qA2 | . | 0 | 1 |
| chr8 | 22577601 | T | C | 125 | Vdac3 | exonic | synonymous SNV | Vdac3:NM_001198998:exon9:c.A765G:p.G255G,Vdac3:NM_011696:exon9:c.A762G:p.G254G | . | 8qA2 | . | 0 | 1 |
| chr8 | 22767217 | A | G | 222 | Plat | intronic | . | . | . | 8qA2 | rs8269535 | 0 | 1 |
| chr8 | 22767248 | C | T | 185 | Plat | intronic | . | . | . | 8qA2 | rs8269533 | 0 | 1 |
| chr8 | 22772084 | G | A | 156 | Plat | intronic | . | . | . | 8qA2 | rs32811646 | 0 | 1 |
| chr8 | 22772821 | C | T | 222 | Plat | intronic | . | . | . | 8qA2 | rs8269565 | 0 | 1 |
| chr8 | 22772835 | A | G | 222 | Plat | intronic | . | . | . | 8qA2 | rs8269563 | 0 | 1 |
| chr8 | 22790663 | T | C | 101 | Ap3m2 | intronic | . | . | . | 8qA2 | rs32628240 | 0 | 1 |
| chr8 | 22791046 | A | C | 222 | Ap3m2 | intronic | . | . | . | 8qA2 | rs50526332 | 0 | 1 |
| chr8 | 22791074 | A | G | 222 | Ap3m2 | intronic | . | . | . | 8qA2 | rs48660464 | 0 | 1 |
| chr8 | 22791187 | T | C | 222 | Ap3m2 | intronic | . | . | . | 8qA2 | rs32727888 | 0 | 1 |
| chr8 | 22792612 | C | A | 140 | Ap3m2 | intronic | . | . | . | 8qA2 | rs33517773 | 0 | 1 |
| chr8 | 22799608 | T | C | 105 | Ap3m2 | intronic | . | . | . | 8qA2 | rs234012423 | 0 | 1 |
| chr8 | 22803726 | A | G | 203 | Ap3m2 | intronic | . | . | . | 8qA2 | rs47579036 | 0 | 1 |
| chr8 | 22803778 | T | G | 222 | Ap3m2 | exonic | synonymous SNV | Ap3m2:NM_001122820:exon2:c.A255C:p.R85R,Ap3m2:NM_029505:exon2:c.A255C:p.R85R | . | 8qA2 | rs51205532 | 0 | 1 |
| chr8 | 22803859 | A | G | 199 | Ap3m2 | exonic | synonymous SNV | Ap3m2:NM_001122820:exon2:c.T174C:p.S58S,Ap3m2:NM_029505:exon2:c.T174C:p.S58S | . | 8qA2 | rs46544665 | 0 | 1 |
| chr8 | 22803877 | A | G | 136 | Ap3m2 | exonic | synonymous SNV | Ap3m2:NM_001122820:exon2:c.T156C:p.P52P,Ap3m2:NM_029505:exon2:c.T156C:p.P52P | . | 8qA2 | rs237294438 | 0 | 1 |
| chr8 | 22803913 | G | T | 135 | Ap3m2 | exonic | synonymous SNV | Ap3m2:NM_001122820:exon2:c.C120A:p.T40T,Ap3m2:NM_029505:exon2:c.C120A:p.T40T | . | 8qA2 | rs49586522 | 0 | 1 |
| chr8 | 22804044 | T | C | 186 | Ap3m2 | UTR5 | . | . | . | 8qA2 | rs46796445 | 0 | 1 |
| chr8 | 22804058 | C | T | 154 | Ap3m2 | UTR5 | . | . | . | 8qA2 | rs46322698 | 0 | 1 |
| chr8 | 22804062 | G | C | 152 | Ap3m2 | UTR5 | . | . | . | 8qA2 | rs46945039 | 0 | 1 |
| chr8 | 22804161 | A | C | 26.7143 | Ap3m2 | intronic | . | . | . | 8qA2 | rs46111224 | 0 | 1 |
| chr8 | 22804177 | C | T | 66 | Ap3m2 | intronic | . | . | . | 8qA2 | rs50795816 | 0 | 1 |
| chr8 | 22975288 | C | G | 83 | Ank1 | intronic | . | . | 1275 | 8qA2 | rs255700269 | 0 | 1 |
| chr8 | 22975395 | T | C | 59 | Ank1 | intronic | . | . | 1275 | 8qA2 | rs236261202 | 0 | 1 |
| chr8 | 23082050 | G | A | 89 | Ank1 | intronic | . | . | . | 8qA2 | . | 0 | 1 |
| chr8 | 23082062 | G | A | 129 | Ank1 | intronic | . | . | . | 8qA2 | . | 0 | 1 |
| chr8 | 23082322 | T | G | 221 | Ank1 | intronic | . | . | . | 8qA2 | rs33415164 | 0 | 1 |
| chr8 | 23082332 | G | A | 221 | Ank1 | intronic | . | . | . | 8qA2 | . | 0 | 1 |
| chr8 | 23084609 | G | A | 123 | Ank1 | intronic | . | . | . | 8qA2 | rs50662080 | 0 | 1 |
| chr8 | 23084646 | C | T | 132 | Ank1 | intronic | . | . | . | 8qA2 | rs51454358 | 0 | 1 |
| chr8 | 23084839 | A | G | 221 | Ank1 | intronic | . | . | . | 8qA2 | rs45784222 | 0 | 1 |
| chr8 | 23085503 | T | C | 221 | Ank1 | intronic | . | . | . | 8qA2 | rs221875185 | 0 | 1 |
| chr8 | 23085569 | T | G | 222 | Ank1 | intronic | . | . | . | 8qA2 | rs51507419 | 0 | 1 |
| chr8 | 23085742 | T | C | 222 | Ank1 | intronic | . | . | . | 8qA2 | rs48083901 | 0 | 1 |
| chr8 | 23086815 | A | G | 116 | Ank1 | intronic | . | . | . | 8qA2 | rs47152650 | 0 | 1 |
| chr8 | 23087833 | T | C | 91 | Ank1 | intronic | . | . | . | 8qA2 | rs51255607 | 0 | 1 |
| chr8 | 23087863 | T | C | 83 | Ank1 | intronic | . | . | . | 8qA2 | rs47383858 | 0 | 1 |
| chr8 | 23087951 | C | T | 120 | Ank1 | intronic | . | . | . | 8qA2 | rs46095791 | 0 | 1 |
| chr8 | 23087955 | G | A | 130 | Ank1 | intronic | . | . | . | 8qA2 | rs247170331 | 0 | 1 |
| chr8 | 23088513 | T | C | 196 | Ank1 | intronic | . | . | . | 8qA2 | rs50203862 | 0 | 1 |
| chr8 | 23088747 | T | G | 204 | Ank1 | intronic | . | . | . | 8qA2 | rs239375604 | 0 | 1 |
| chr8 | 23088790 | C | A | 125 | Ank1 | intronic | . | . | . | 8qA2 | rs242664465 | 0 | 1 |
| chr8 | 23088801 | C | T | 93 | Ank1 | intronic | . | . | . | 8qA2 | . | 0 | 1 |
| chr8 | 23088871 | G | A | 222 | Ank1 | intronic | . | . | . | 8qA2 | rs223497216 | 0 | 1 |
| chr8 | 23088930 | T | C | 222 | Ank1 | intronic | . | . | . | 8qA2 | . | 0 | 1 |
| chr8 | 23093621 | A | G | 82 | Ank1 | intronic | . | . | . | 8qA2 | rs255792770 | 0 | 1 |
| chr8 | 23093624 | C | T | 81 | Ank1 | intronic | . | . | . | 8qA2 | rs224776620 | 0 | 1 |
| chr8 | 23093771 | T | A | 222 | Ank1 | intronic | . | . | . | 8qA2 | rs48231348 | 0 | 1 |
| chr8 | 23093881 | A | G | 222 | Ank1 | exonic | synonymous SNV | Ank1:NM_001110783:exon10:c.A1086G:p.A362A,Ank1:NM_001277280:exon10:c.A1086G:p.A362A,Ank1:NM_( | . | 8qA2 | rs48794644 | 0 | 1 |
| chr8 | 23093923 | T | C | 222 | Ank1 | exonic | synonymous SNV | Ank1:NM_001110783:exon10:c.T1128C:p.H376H,Ank1:NM_001277280:exon10:c.T1128C:p.H376H,Ank1:NM_( | . | 8qA2 | rs49908396 | 0 | 1 |
| chr8 | 23094096 | A | C | 48.9771 | Ank1 | intronic | . | . | . | 8qA2 | rs108760644 | 0 | 1 |
| chr8 | 23094109 | T | C | 45.9823 | Ank1 | intronic | . | . | . | 8qA2 | rs580921248 | 0 | 1 |
| chr8 | 23095109 | G | T | 150 | Ank1 | intronic | . | . | . | 8qA2 | rs49470137 | 0 | 1 |
| chr8 | 23095189 | T | G | 222 | Ank1 | intronic | . | . | . | 8qA2 | rs50616498 | 0 | 1 |
| chr8 | 23095385 | T | C | 185 | Ank1 | intronic | . | . | . | 8qA2 | rs52566111 | 0 | 1 |
| chr8 | 23099142 | T | C | 123 | Ank1 | intronic | . | . | . | 8qA2 | rs48159264 | 0 | 1 |
| chr8 | 23099533 | G | C | 222 | Ank1 | intronic | . | . | . | 8qA2 | rs264271340 | 0 | 1 |
| chr8 | 23104697 | C | T | 141 | Ank1 | intronic | . | . | . | 8qA2 | rs221842149 | 0 | 1 |
| chr8 | 23104730 | G | C | 158 | Ank1 | intronic | . | . | . | 8qA2 | rs32608467 | 0 | 1 |
| chr8 | 23104903 | A | G | 150 | Ank1 | exonic | synonymous SNV | Ank1:NM_001110783:exon17:c.A2013G:p.P671P,Ank1:NM_001277280:exon17:c.A2013G:p.P671P,Ank1:NM_( | . | 8qA2 | rs227055000 | 0 | 1 |
| chr8 | 23104933 | A | G | 168 | Ank1 | exonic | synonymous SNV | Ank1:NM_001110783:exon17:c.A2043G:p.T681T,Ank1:NM_001277280:exon17:c.A2043G:p.T681T,Ank1:NM_( | . | 8qA2 | rs246246300 | 0 | 1 |
| chr8 | 23104969 | C | T | 170 | Ank1 | exonic | synonymous SNV | Ank1:NM_001110783:exon17:c.C2079T:p.N693N,Ank1:NM_001277280:exon17:c.C2079T:p.N693N,Ank1:NM_( | . | 8qA2 | rs33895797 | 0 | 1 |
| chr8 | 23108023 | C | A | 222 | Ank1 | intronic | . | . | . | 8qA2 | . | 0 | 1 |
| chr8 | 23108078 | G | A | 222 | Ank1 | intronic | . | . | . | 8qA2 | . | 0 | 1 |
| chr8 | 23109010 | T | C | 37.7106 | Ank1 | intronic | . | . | . | 8qA2 | rs32966739 | 0 | 1 |
| chr8 | 23110198 | C | T | 198 | Ank1 | intronic | . | . | . | 8qA2 | rs46131640 | 0 | 1 |
| chr8 | 23110253 | G | A | 221 | Ank1 | intronic | . | . | . | 8qA2 | rs49638456 | 0 | 1 |
| chr8 | 23110470 | C | A | 220 | Ank1 | intronic | . | . | . | 8qA2 | . | 0 | 1 |
| chr8 | 23110495 | A | G | 138 | Ank1 | intronic | . | . | . | 8qA2 | rs222688145 | 0 | 1 |
| chr8 | 23111364 | C | T | 221 | Ank1 | exonic | synonymous SNV | Ank1:NM_001277280:exon23:c.C2565T:p.D855D,Ank1:NM_001277281:exon23:c.C2454T:p.D818D,Ank1:NM_( | . | 8qA2 | . | 0 | 1 |

| | | | | | | | | | | | | | | |
|---|---|---|---|---|---|---|---|---|---|---|---|---|---|---|
| chr8 | 23111544 | T | C | 221 | Ank1 | intronic | . | . | . | 8qA2 | rs247523404 | 0 | 1 |
| chr8 | 23111577 | T | C | 222 | Ank1 | intronic | . | . | . | 8qA2 | rs259252319 | 0 | 1 |
| chr8 | 23111675 | T | C | 116 | Ank1 | intronic | . | . | . | 8qA2 | rs254203891 | 0 | 1 |
| chr8 | 23111959 | A | G | 169 | Ank1 | intronic | . | . | . | 8qA2 | rs211999200 | 0 | 1 |
| chr8 | 23113757 | T | C | 150 | Ank1 | intronic | . | . | . | 8qA2 | . | 0 | 1 |
| chr8 | 23113772 | A | G | 183 | Ank1 | exonic | synonymous SNV | Ank1:NM_001277280:exon25:c.A2745G:p.K915K,Ank1:NM_001277281:exon25:c.A2634G:p.K878K,Ank1:NM_( | . | 8qA2 | . | 0 | 1 |
| chr8 | 23115483 | T | C | 221 | Ank1 | exonic | synonymous SNV | Ank1:NM_001277280:exon28:c.T3342C:p.G1114G,Ank1:NM_001277281:exon28:c.T3231C:p.G1077G,Ank1:NM | . | 8qA2 | rs32545346 | 0 | 1 |
| chr8 | 23116607 | A | G | 222 | Ank1 | exonic | missense SNV | Ank1:NM_001277280:exon30:c.A3682G:p.I1228V,Ank1:NM_001277281:exon30:c.A3571G:p.I1191V,Ank1:NM | . | 8qA2 | rs49711901 | 0 | 1 |
| chr8 | 23118821 | C | T | 32.9726 | Ank1 | intronic | . | . | . | 8qA2 | rs46650214 | 0 | 1 |
| chr8 | 23118859 | G | T | 91 | Ank1 | intronic | . | . | . | 8qA2 | rs50931022 | 0 | 1 |
| chr8 | 23119015 | G | A | 222 | Ank1 | intronic | . | . | . | 8qA2 | rs234204455 | 0 | 1 |
| chr8 | 23119207 | G | A | 222 | Ank1 | intronic | . | . | . | 8qA2 | rs32585918 | 0 | 1 |
| chr8 | 23119211 | C | T | 222 | Ank1 | intronic | . | . | . | 8qA2 | rs33111546 | 0 | 1 |
| chr8 | 23119309 | C | T | 222 | Ank1 | intronic | . | . | . | 8qA2 | rs33156318 | 0 | 1 |
| chr8 | 23119358 | A | G | 222 | Ank1 | exonic | synonymous SNV | Ank1:NM_001277280:exon33:c.A4128G:p.L1376L,Ank1:NM_001277281:exon33:c.A4017G:p.L1339L,Ank1:NM | . | 8qA2 | rs32796095 | 0 | 1 |
| chr8 | 23120987 | G | A | 222 | Ank1 | intronic | . | . | . | 8qA2 | rs51120182 | 0 | 1 |
| chr8 | 23121013 | C | T | 222 | Ank1 | intronic | . | . | . | 8qA2 | rs227777453 | 0 | 1 |
| chr8 | 23122935 | G | A | 222 | Ank1 | intronic | . | . | . | 8qA2 | . | 0 | 1 |
| chr8 | 23132191 | G | T | 222 | Ank1 | exonic | synonymous SNV | Ank1:NM_001277281:exon38:c.G4620T:p.V1540V,Ank1:NM_001277284:exon38:c.G4620T:p.V1540V,Ank1:NM | . | 8qA2 | . | 0 | 1 |
| chr8 | 23135523 | C | T | 73 | Ank1 | intronic | . | . | . | 8qA2 | rs45928992 | 0 | 1 |
| chr8 | 23135553 | T | C | 129 | Ank1 | intronic | . | . | . | 8qA2 | rs47795082 | 0 | 1 |
| chr8 | 23135593 | T | C | 205 | Ank1 | exonic | missense SNV | Ank1:NM_001277280:exon39:c.T4745C:p.V1582A,Ank1:NM_001277281:exon39:c.T5099C:p.V1700A,Ank1:NM | . | 8qA2 | rs51953956 | 0 | 1 |
| chr8 | 23135813 | C | T | 222 | Ank1 | exonic | synonymous SNV | Ank1:NM_001277280:exon39:c.C4965T:p.S1655S,Ank1:NM_001277281:exon39:c.C5319T:p.S1773S,Ank1:NM | . | 8qA2 | rs50202222 | 0 | 1 |
| chr8 | 23135892 | C | T | 127 | Ank1 | intronic | . | . | . | 8qA2 | rs50673120 | 0 | 1 |
| chr8 | 23139264 | A | G | 122 | Ank1,Gm15816 | UTR5;UTR3 | . | . | . | 8qA2 | rs48814617 | 0 | 1 |
| chr8 | 23139268 | G | A | 122 | Ank1,Gm15816 | UTR5;UTR3 | . | . | . | 8qA2 | rs33393627 | 0 | 1 |
| chr8 | 23140001 | A | G | 55 | Gm15816,Ank1 | intronic | . | . | . | 8qA2 | rs50355457 | 0 | 1 |
| chr8 | 23140079 | T | C | 155 | Ank1,Gm15816 | intronic | . | . | . | 8qA2 | rs50327450 | 0 | 1 |
| chr8 | 23140103 | C | T | 222 | Ank1,Gm15816 | intronic | . | . | . | 8qA2 | rs46609677 | 0 | 1 |
| chr8 | 23140139 | C | T | 222 | Gm15816,Ank1 | exonic | synonymous SNV | Gm15816:NM_001282148:exon5:c.G348A:p.E116E | . | 8qA2 | rs48557945 | 0 | 1 |
| chr8 | 23140341 | G | C | 146 | Ank1,Gm15816 | intronic | . | . | . | 8qA2 | rs234541276 | 0 | 1 |
| chr8 | 23140343 | T | G | 146 | Gm15816,Ank1 | intronic | . | . | . | 8qA2 | rs580101249 | 0 | 1 |
| chr8 | 23140382 | G | A | 88 | Gm15816,Ank1 | intronic | . | . | . | 8qA2 | rs47104135 | 0 | 1 |
| chr8 | 23140960 | T | G | 75 | Ank1,Gm15816 | exonic | missense SNV | Gm15816:NM_001282148:exon4:c.A108C:p.K36N | . | 8qA2 | rs51192452 | 1 | 0 |
| chr8 | 23141004 | C | T | 126 | Gm15816,Ank1 | exonic | missense SNV | Gm15816:NM_001282148:exon4:c.G64A:p.G22S | . | 8qA2 | rs50699742 | 1 | 0 |
| chr8 | 23141071 | C | T | 106 | Gm15816,Ank1 | UTR5 | . | . | . | 8qA2 | rs107655677 | 0 | 1 |
| chr8 | 23141146 | C | T | 181 | Gm15816,Ank1 | UTR5 | . | . | . | 8qA2 | rs48045382 | 0 | 1 |
| chr8 | 23141220 | G | A | 176 | Gm15816,Ank1 | exonic | synonymous SNV | Ank1:NM_001310436:exon3:c.G360A:p.Q120Q,Ank1:NM_001310437:exon3:c.G360A:p.Q120Q,Ank1:NM_00127 | . | 8qA2 | rs49830536 | 0 | 1 |
| chr8 | 23141274 | A | G | 111 | Gm15816,Ank1 | exonic | synonymous SNV | Ank1:NM_001310436:exon3:c.A414G:p.A138A,Ank1:NM_001277284:exon41:c.A5535G:p.A1845A | . | 8qA2 | rs242085394 | 0 | 1 |
| chr8 | 23141784 | A | C | 89 | Gm15816,Ank1 | intronic | . | . | . | 8qA2 | rs52018291 | 0 | 1 |
| chr8 | 23142799 | G | A | 65 | Ank1,Gm15816 | intronic | . | . | . | 8qA2 | rs52063976 | 0 | 1 |
| chr8 | 23148236 | G | A | 221 | Gm15816,Ank1 | intronic | . | . | . | 8qA2 | . | 0 | 1 |
| chr8 | 23153436 | C | T | 60 | Nkx6-3 | UTR5 | . | . | . | 8qA2 | . | 0 | 1 |
| chr8 | 23153551 | G | A | 162 | Nkx6-3 | UTR5 | . | . | . | 8qA2 | . | 0 | 1 |
| chr8 | 23153552 | T | C | 162 | Nkx6-3 | UTR5 | . | . | . | 8qA2 | rs242019450 | 0 | 1 |
| chr8 | 23153923 | A | G | 173 | Nkx6-3 | exonic | synonymous SNV | Nkx6-3:NM_029002:exon1:c.A339G:p.A113A | . | 8qA2 | rs33044604 | 0 | 1 |
| chr8 | 23174458 | G | A | 126 | Gpat4 | UTR3 | . | . | . | 8qA2 | . | 0 | 1 |
| chr8 | 23175571 | C | T | 222 | Gpat4 | exonic | synonymous SNV | Gpat4:NM_018743:exon11:c.G1119A:p.L373L | . | 8qA2 | . | 0 | 1 |
| chr8 | 23179255 | T | C | 37.7047 | Gpat4 | intronic | . | . | . | 8qA2 | . | 0 | 1 |
| chr8 | 23179743 | A | C | 222 | Gpat4 | intronic | . | . | . | 8qA2 | rs32593575 | 0 | 1 |
| chr8 | 23181571 | A | G | 148 | Gpat4 | intronic | . | . | . | 8qA2 | rs46226541 | 1 | 0 |
| chr8 | 23226845 | G | T | 113 | Gins4 | UTR3 | . | . | . | 8qA2 | rs33119278 | 0 | 1 |
| chr8 | 23227207 | G | A | 222 | Gins4 | intronic | . | . | . | 8qA2 | rs33235181 | 0 | 1 |
| chr8 | 23227232 | G | A | 222 | Gins4 | intronic | . | . | . | 8qA2 | rs33024231 | 0 | 1 |
| chr8 | 23227358 | C | T | 222 | Gins4 | intronic | . | . | . | 8qA2 | rs47943121 | 0 | 1 |
| chr8 | 23246115 | C | T | 112 | Golga7 | intronic | . | . | . | 8qA2 | . | 0 | 1 |
| chr8 | 23256670 | A | G | 175 | Golga7 | splicing | . | . | . | 8qA2 | rs52299242 | 0 | 1 |
| chr8 | 23257790 | T | C | 222 | . | upstream | . | . | . | 8qA2 | . | 0 | 1 |
| chr8 | 23417414 | C | G | 222 | Sfrp1 | intronic | . | . | . | 8qA2 | rs33424317 | 0 | 1 |
| chr8 | 23446565 | G | A | 221 | Sfrp1 | UTR3 | . | . | . | 8qA2 | rs32603499 | 0 | 1 |
| chr8 | 23748442 | T | C | 222 | Zmat4 | exonic | synonymous SNV | Zmat4:NM_177086:exon2:c.T39C:p.D13D | . | 8qA2 | rs32854630 | 0 | 1 |
| chr8 | 23797455 | G | T | 220 | Zmat4 | intronic | . | . | . | 8qA2 | . | 0 | 1 |
| chr8 | 23929336 | C | T | 221 | Zmat4 | intronic | . | . | . | 8qA2 | rs264150087 | 0 | 1 |
| chr8 | 23929345 | T | C | 215 | Zmat4 | intronic | . | . | . | 8qA2 | rs215485043 | 0 | 1 |
| chr8 | 24015201 | A | G | 222 | Zmat4 | exonic | synonymous SNV | Zmat4:NM_177086:exon6:c.A663G:p.K221K,Zmat4:NM_001277239:exon7:c.A456G:p.K152K | . | 8qA2 | rs52633135 | 0 | 1 |
| chr8 | 24059628 | T | C | 94 | Zmat4 | intronic | . | . | . | 8qA2 | rs257329514 | 0 | 1 |
| chr8 | 24059634 | T | C | 102 | Zmat4 | intronic | . | . | . | 8qA2 | rs216845169 | 0 | 1 |
| chr8 | 24059636 | A | C | 105 | Zmat4 | intronic | . | . | . | 8qA2 | rs230396575 | 0 | 1 |
| chr8 | 24059849 | A | G | 222 | Zmat4 | UTR3 | . | . | . | 8qA2 | rs50585952 | 0 | 1 |
| chr8 | 24059881 | A | G | 222 | Zmat4 | UTR3 | . | . | . | 8qA2 | . | 0 | 1 |
| chr8 | 24098528 | C | T | 44.9215 | . | intergenic | . | . | . | 8qA2 | . | 0 | 1 |
| chr8 | 24098581 | T | A | 45.8205 | . | intergenic | . | . | . | 8qA2 | rs226171118 | 0 | 1 |
| chr8 | 24185422 | A | G | 222 | . | intergenic | . | . | . | 8qA2 | . | 0 | 1 |
| chr8 | 24963063 | C | A | 222 | Adam9 | exonic | missense SNV | Adam9:NM_001270996:exon20:c.G2227T:p.V743F,Adam9:NM_007404:exon20:c.G2227T:p.V743F | . | 8qA2 | . | 0 | 1 |
| chr8 | 25161348 | G | A | 131 | Tacc1 | intronic | . | . | . | 8qA2 | . | 0 | 1 |
| chr8 | 25675611 | G | T | 228 | Whsc1l1 | intronic | . | . | . | 8qA2 | rs107872478 | 1 | 0 |
| chr8 | 25900106 | G | C | 221 | Kcnu1 | intronic | . | . | . | 8qA2 | . | 0 | 1 |
| chr8 | 26158405 | C | A | 198 | Thap1 | UTR5 | . | . | 1021 | 8qA2 | . | 0 | 1 |
| chr8 | 26160882 | C | T | 166 | Thap1 | exonic | missense SNV | Thap1:NM_199042:exon2:c.C179T:p.P60L | . | 8qA2 | rs259480790 | 0 | 1 |
| chr8 | 26160946 | T | C | 114 | Thap1 | exonic | synonymous SNV | Thap1:NM_199042:exon2:c.T243C:p.F81F | . | 8qA2 | rs225686928 | 0 | 1 |
| chr8 | 26162757 | C | T | 184 | Thap1 | exonic | synonymous SNV | Thap1:NM_199042:exon3:c.C591T:p.I197I | . | 8qA2 | rs261023027 | 0 | 1 |
| chr8 | 26162833 | C | T | 221 | Thap1 | UTR3 | . | . | . | 8qA2 | rs227103984 | 0 | 1 |
| chr8 | 27124093 | G | T | 39.8895 | Brf2 | exonic | missense SNV | Brf2:NM_025686:exon4:c.C1064A:p.P355H | . | 8qA2 | . | 0 | 1 |
| chr8 | 28759212 | A | G | 158 | Unc5d | intronic | . | . | . | 8qA2 | . | 0 | 1 |
| chr8 | 33693646 | A | G | 111 | Gsr | intronic | . | . | . | 8qA3 | . | 0 | 1 |
| chr8 | 34108197 | C | A | 73 | Dctn6 | intronic | . | . | 428 | 8qA4 | . | 0 | 1 |
| chr8 | 34137850 | A | G | 221 | Leprotl1 | intronic | . | . | . | 8qA4 | . | 0 | 1 |
| chr8 | 34165492 | G | A | 161 | Saraf | exonic | missense SNV | Saraf:NM_026432:exon3:c.G724A:p.A242T | . | 8qA4 | . | 0 | 1 |
| chr8 | 34321884 | C | T | 222 | . | intergenic | . | . | . | 8qA4 | . | 0 | 1 |
| chr8 | 35468991 | C | A | 106 | Eri1 | UTR3 | . | . | . | 8qA4 | rs13475012 | 0 | 1 |
| chr8 | 35469074 | A | G | 228 | Eri1 | UTR3 | . | . | . | 8qA4 | rs30363272 | 1 | 0 |
| chr8 | 35469199 | A | G | 221 | Eri1 | exonic | synonymous SNV | Eri1:NM_026067:exon7:c.T967C:p.L323L | . | 8qA4 | rs13479698 | 0 | 1 |
| chr8 | 35469259 | T | C | 221 | Eri1 | exonic | missense SNV | Eri1:NM_026067:exon7:c.A907G:p.I303V | . | 8qA4 | rs30364277 | 0 | 1 |
| chr8 | 35469374 | G | T | 222 | Eri1 | splicing | . | . | . | 8qA4 | rs30364280 | 0 | 1 |
| chr8 | 35474626 | A | G | 125 | Eri1 | intronic | . | . | . | 8qA4 | rs30363228 | 0 | 1 |
| chr8 | 35474681 | A | G | 37.2729 | Eri1 | intronic | . | . | . | 8qA4 | rs32737472 | 0 | 1 |
| chr8 | 35474697 | C | A | 37.9393 | Eri1 | intronic | . | . | . | 8qA4 | rs33302057 | 0 | 1 |
| chr8 | 35476394 | G | T | 156 | Eri1 | intronic | . | . | . | 8qA4 | rs30365387 | 0 | 1 |
| chr8 | 35476650 | T | C | 222 | Eri1 | intronic | . | . | . | 8qA4 | rs30365390 | 0 | 1 |
| chr8 | 35478603 | T | G | 222 | Eri1 | exonic | missense SNV | Eri1:NM_026067:exon4:c.A527C:p.D176A | . | 8qA4 | rs13475014 | 0 | 1 |
| chr8 | 35478638 | A | G | 222 | Eri1 | exonic | synonymous SNV | Eri1:NM_026067:exon4:c.T492C:p.D164D | . | 8qA4 | rs30369126 | 0 | 1 |
| chr8 | 35478649 | A | G | 222 | Eri1 | intronic | . | . | . | 8qA4 | rs50222367 | 0 | 1 |
| chr8 | 35478748 | T | C | 145 | Eri1 | intronic | . | . | . | 8qA4 | rs30369129 | 0 | 1 |
| chr8 | 35478793 | T | C | 93 | Eri1 | intronic | . | . | . | 8qA4 | rs50057721 | 0 | 1 |
| chr8 | 35478805 | T | G | 98 | Eri1 | intronic | . | . | . | 8qA4 | rs51479431 | 0 | 1 |
| chr8 | 35482489 | G | A | 222 | Eri1 | intronic | . | . | . | 8qA4 | rs30369257 | 0 | 1 |
| chr8 | 35491062 | A | G | 76 | Eri1 | intronic | . | . | . | 8qA4 | rs32869781 | 0 | 1 |
| chr8 | 35491172 | A | G | 191 | Eri1 | intronic | . | . | . | 8qA4 | rs32613955 | 0 | 1 |
| chr8 | 35491296 | C | T | 222 | Eri1 | exonic | synonymous SNV | Eri1:NM_026067:exon2:c.G180A:p.P60P | . | 8qA4 | rs13475011 | 0 | 1 |
| chr8 | 35590067 | T | C | 222 | Mfhas1 | exonic | synonymous SNV | Mfhas1:NM_001081279:exon1:c.T1695C:p.L565L | . | 8qA4 | rs49896526 | 0 | 1 |
| chr8 | 35664530 | T | C | 98 | Mfhas1 | intronic | . | . | . | 8qA4 | rs30388841 | 0 | 1 |
| chr8 | 35664953 | T | C | 118 | Mfhas1 | intronic | . | . | . | 8qA4 | rs30389737 | 0 | 1 |
| chr8 | 35676612 | C | T | 135 | Mfhas1 | intronic | . | . | . | 8qA4 | rs30384586 | 0 | 1 |
| chr8 | 35676683 | T | A | 222 | Mfhas1 | intronic | . | . | . | 8qA4 | rs30384588 | 0 | 1 |
| chr8 | 35676772 | C | G | 164 | Mfhas1 | UTR3 | . | . | . | 8qA4 | rs48327683 | 0 | 1 |
| chr8 | 35676775 | G | A | 137 | Mfhas1 | UTR3 | . | . | . | 8qA4 | rs51489462 | 0 | 1 |
| chr8 | 35825465 | G | A | 36.1218 | Cldn23 | exonic | synonymous SNV | Cldn23:NM_027998:exon1:c.C868T:p.L290L | . | 8qA4 | rs30423132 | 0 | 1 |
| chr8 | 35825484 | A | G | 63 | Cldn23 | exonic | synonymous SNV | Cldn23:NM_027998:exon1:c.T849C:p.T283T | . | 8qA4 | rs30424015 | 0 | 1 |
| chr8 | 35825598 | A | C | 222 | Cldn23 | exonic | synonymous SNV | Cldn23:NM_027998:exon1:c.T735G:p.V245V | 1282 | 8qA4 | rs30424018 | 0 | 1 |
| chr8 | 35825835 | G | C | 221 | Cldn23 | exonic | synonymous SNV | Cldn23:NM_027998:exon1:c.C498G:p.G166G | 1282 | 8qA4 | rs30424021 | 0 | 1 |
| chr8 | 35825856 | G | A | 222 | Cldn23 | exonic | synonymous SNV | Cldn23:NM_027998:exon1:c.C477T:p.S159S | 1282 | 8qA4 | rs30424894 | 0 | 1 |
| chr8 | 35826030 | A | G | 221 | Cldn23 | exonic | synonymous SNV | Cldn23:NM_027998:exon1:c.T303C:p.G101G | 1282 | 8qA4 | rs13460203 | 0 | 1 |
| chr8 | 35826376 | T | C | 183 | Cldn23 | UTR5 | . | . | 1282 | 8qA4 | rs13460202 | 0 | 1 |
| chr8 | 36571147 | A | G | 177 | Dlc1 | intronic | . | . | . | 8qA4 | rs37566021 | 0 | 1 |
| chr8 | 39642650 | C | T | 137 | Mir669e,Msr1 | ncRNA_intronic | . | . | . | 8qA4 | . | 0 | 1 |
| chr8 | 40562558 | C | T | 179 | Mtmr7 | intronic | . | . | . | 8qA4 | rs265774603 | 0 | 1 |
| chr8 | 40913837 | A | G | 108 | Slc7a2 | intronic | . | . | . | 8qA4 | . | 0 | 1 |
| chr8 | 40999128 | G | T | 45.8132 | Mtus1 | intronic | . | . | . | 8qA4 | . | 0 | 1 |
| chr8 | 41282619 | T | C | 222 | Pcm1 | exonic | missense SNV | Pcm1:NM_023662:exon15:c.T2356C:p.Y786H | . | 8qA4 | . | 0 | 1 |
| chr8 | 44950080 | T | C | 52 | . | upstream | . | . | . | 8qB1.1 | rs36803097 | 0 | 1 |
| chr8 | 44950156 | A | G | 184 | . | upstream | . | . | . | 8qB1.1 | rs36978909 | 0 | 1 |
| chr8 | 44950229 | A | G | 221 | Fat1 | exonic | missense SNV | Fat1:NM_001081286:exon1:c.A16G:p.T6A | . | 8qB1.1 | rs36940459 | 0 | 1 |
| chr8 | 44950264 | G | T | 221 | Fat1 | exonic | missense SNV | Fat1:NM_001081286:exon1:c.G51T:p.Q17H | . | 8qB1.1 | rs36876010 | 0 | 1 |
| chr8 | 44950438 | C | T | 221 | Fat1 | exonic | synonymous SNV | Fat1:NM_001081286:exon1:c.C225T:p.I75I | . | 8qB1.1 | rs36484102 | 0 | 1 |
| chr8 | 44950603 | T | C | 222 | Fat1 | exonic | synonymous SNV | Fat1:NM_001081286:exon1:c.T390C:p.A130A | 261 | 8qB1.1 | rs36842425 | 0 | 1 |
| chr8 | 44950627 | A | G | 222 | Fat1 | exonic | synonymous SNV | Fat1:NM_001081286:exon1:c.A414G:p.V138V | 261 | 8qB1.1 | rs36720888 | 0 | 1 |
| chr8 | 44950885 | G | A | 221 | Fat1 | exonic | synonymous SNV | Fat1:NM_001081286:exon1:c.G672A:p.L224L | . | 8qB1.1 | . | 0 | 1 |
| chr8 | 44951098 | C | T | 222 | Fat1 | exonic | synonymous SNV | Fat1:NM_001081286:exon1:c.C885T:p.I295I | . | 8qB1.1 | . | 0 | 1 |
| chr8 | 44951176 | C | T | 222 | Fat1 | exonic | synonymous SNV | Fat1:NM_001081286:exon1:c.C963T:p.V321V | . | 8qB1.1 | rs37357278 | 0 | 1 |
| chr8 | 44951554 | C | T | 222 | Fat1 | exonic | synonymous SNV | Fat1:NM_001081286:exon1:c.C1341T:p.S447S | . | 8qB1.1 | . | 0 | 1 |
| chr8 | 44952286 | G | A | 222 | Fat1 | exonic | synonymous SNV | Fat1:NM_001081286:exon1:c.G2073A:p.A691A | . | 8qB1.1 | rs37640248 | 0 | 1 |
| chr8 | 44952733 | T | C | 222 | Fat1 | exonic | synonymous SNV | Fat1:NM_001081286:exon1:c.T2520C:p.S840S | . | 8qB1.1 | rs37441425 | 0 | 1 |
| chr8 | 44952833 | A | G | 222 | Fat1 | exonic | missense SNV | Fat1:NM_001081286:exon1:c.A2620G:p.M874V | . | 8qB1.1 | rs37482480 | 0 | 1 |
| chr8 | 44952881 | C | T | 222 | Fat1 | exonic | missense SNV | Fat1:NM_001081286:exon1:c.C2668T:p.P890S | . | 8qB1.1 | . | 0 | 1 |
| chr8 | 44953307 | A | G | 221 | Fat1 | exonic | missense SNV | Fat1:NM_001081286:exon1:c.A3094G:p.T1032A | . | 8qB1.1 | rs36644879 | 0 | 1 |
| chr8 | 44988700 | T | C | 43.9904 | Fat1 | intronic | . | . | . | 8qB1.1 | rs36667010 | 0 | 1 |
| chr8 | 45009590 | A | C | 38.7414 | Fat1 | intronic | . | . | . | 8qB1.1 | . | 0 | 1 |
| chr8 | 45010132 | C | A | 222 | Fat1 | intronic | . | . | 284 | 8qB1.1 | . | 0 | 1 |
| chr8 | 45010604 | C | T | 104 | Fat1 | exonic | synonymous SNV | Fat1:NM_001081286:exon5:c.C4182T:p.D1394D | 221 | 8qB1.1 | . | 0 | 1 |
| chr8 | 45044106 | T | G | 222 | Fat1 | exonic | synonymous SNV | Fat1:NM_001081286:exon24:c.T12558G:p.G4186G | . | 8qB1.1 | rs39072741 | 0 | 1 |
| chr8 | 45044709 | A | G | 43.9112 | Fat1 | intronic | . | . | . | 8qB1.1 | rs230289033 | 0 | 1 |
| chr8 | 45044711 | A | G | 43.9112 | Fat1 | intronic | . | . | . | 8qB1.1 | rs253399409 | 0 | 1 |
| chr8 | 45044926 | C | T | 156 | Fat1 | intronic | . | . | . | 8qB1.1 | rs258735387 | 0 | 1 |
| chr8 | 45044992 | T | C | 195 | Fat1 | intronic | . | . | . | 8qB1.1 | rs37398385 | 0 | 1 |
| chr8 | 45045062 | T | C | 222 | Fat1 | exonic | synonymous SNV | Fat1:NM_001081286:exon25:c.T13038C:p.S4346S | . | 8qB1.1 | rs37435844 | 0 | 1 |
| chr8 | 45045181 | T | C | 222 | Fat1 | intronic | . | . | . | 8qB1.1 | rs38744940 | 0 | 1 |
| chr8 | 45045258 | T | C | 168 | Fat1 | intronic | . | . | . | 8qB1.1 | rs38467431 | 0 | 1 |
| chr8 | 45045292 | G | A | 62 | Fat1 | intronic | . | . | . | 8qB1.1 | rs37838910 | 0 | 1 |
| chr8 | 45249468 | T | A | 221 | F11 | intronic | . | . | . | 8qB1.1 | rs261033767 | 0 | 1 |
| chr8 | 45255403 | A | G | 222 | F11 | intronic | . | . | . | 8qB1.1 | rs36795780 | 0 | 1 |
| chr8 | 45398752 | T | C | 221 | Tlr3 | exonic | synonymous SNV | Tlr3:NM_126166:exon6:c.A1107G:p.P369P | . | 8qB1.1 | rs36870083 | 0 | 1 |
| chr8 | 45400595 | T | C | 219 | Tlr3 | intronic | . | . | . | 8qB1.1 | . | 0 | 1 |
| chr8 | 45402812 | A | G | 222 | Tlr3 | exonic | synonymous SNV | Tlr3:NM_126166:exon4:c.T330C:p.N110N | . | 8qB1.1 | rs259813261 | 0 | 1 |
| chr8 | 45736095 | G | C | 222 | Sorbs2,Sorbs2os | ncRNA_intronic | . | . | . | 8qB1.1 | . | 0 | 1 |
| chr8 | 45745270 | A | G | 211 | Sorbs2os,Sorbs2 | ncRNA_intronic | . | . | . | 8qB1.1 | . | 0 | 1 |
| chr8 | 45745595 | G | A | 221 | Sorbs2,Sorbs2os | ncRNA_intronic | . | . | . | 8qB1.1 | . | 0 | 1 |
| chr8 | 45745802 | C | T | 222 | Sorbs2os,Sorbs2 | exonic | synonymous SNV | Sorbs2:NM_001310708:exon4:c.C54T:p.S18S,Sorbs2:NM_001205219:exon6:c.C216T:p.S72S,Sorbs2:NM_0( | . | 8qB1.1 | . | 0 | 1 |
| chr8 | 45745927 | G | C | 222 | Sorbs2os,Sorbs2 | ncRNA_intronic | . | . | . | 8qB1.1 | . | 0 | 1 |
| chr8 | 45745991 | A | G | 129 | Sorbs2os,Sorbs2 | ncRNA_intronic | . | . | . | 8qB1.1 | rs227482307 | 0 | 1 |
| chr8 | 45763205 | A | G | 222 | Sorbs2,Sorbs2os | ncRNA_intronic | . | . | . | 8qB1.1 | rs37208837 | 0 | 1 |
| chr8 | 45917450 | T | C | 221 | Pdlim3 | intronic | . | . | . | 8qB1.1 | . | 0 | 1 |
| chr8 | 45939298 | A | G | 222 | Ccdc110 | exonic | synonymous SNV | Ccdc110:NM_001033246:exon4:c.A219G:p.L73L | . | 8qB1.1 | rs36678833 | 0 | 1 |
| chr8 | 45943119 | C | T | 221 | Ccdc110 | exonic | synonymous SNV | Ccdc110:NM_001033246:exon6:c.C2046T:p.A682A | . | 8qB1.1 | . | 0 | 1 |
| chr8 | 45995888 | C | T | 58 | Ufsp2 | intronic | . | . | . | 8qB1.1 | rs33364840 | 0 | 1 |
| chr8 | 46034285 | C | T | 92 | Snx25 | intronic | . | . | . | 8qB1.1 | . | 0 | 1 |
| chr8 | 46049184 | C | A | 130 | Snx25 | intronic | . | . | . | 8qB1.1 | rs36788994 | 0 | 1 |
| chr8 | 46049264 | A | G | 222 | Snx25 | intronic | . | . | . | 8qB1.1 | rs232470662 | 0 | 1 |
| chr8 | 46054178 | C | T | 122 | Snx25 | intronic | . | . | . | 8qB1.1 | rs36922159 | 0 | 1 |
| chr8 | 46054211 | G | C | 134 | Snx25 | intronic | . | . | . | 8qB1.1 | . | 0 | 1 |
| chr8 | 46056320 | G | C | 79 | Snx25 | intronic | . | . | . | 8qB1.1 | rs37844640 | 0 | 1 |
| chr8 | 46058258 | T | C | 111 | Snx25 | intronic | . | . | . | 8qB1.1 | . | 0 | 1 |
| chr8 | 46080229 | T | C | 221 | Snx25 | exonic | synonymous SNV | Snx25:NM_207213:exon5:c.A663G:p.R221R | . | 8qB1.1 | rs37081183 | 0 | 1 |
| chr8 | 46116569 | G | A | 117 | Snx25 | intronic | . | . | . | 8qB1.1 | rs37992836 | 0 | 1 |
| chr8 | 46124042 | T | C | 221 | Snx25 | intronic | . | . | . | 8qB1.1 | rs37078899 | 0 | 1 |
| chr8 | 46170554 | G | A | 222 | Cfap97 | exonic | missense SNV | Cfap97:NM_025747:exon1:c.G980A:p.S327N | . | 8qB1.1 | rs36834234 | 0 | 1 |

| | | | | | | | | | | | | | |
|---|---|---|---|---|---|---|---|---|---|---|---|---|---|
| chr8 | 46181567 | C | A | 109 | Cfap97 | intronic | . | . | . | 8qB1.1 | . | 0 | 1 |
| chr8 | 46181899 | C | T | 222 | Cfap97 | exonic | synonymous SNV | Cfap97:NM_025747:exon2:c.C1260T:p.P420P | . | 8qB1.1 | . | 0 | 1 |
| chr8 | 46192462 | T | C | 146 | Cfap97 | intronic | . | . | . | 8qB1.1 | rs36816101 | 0 | 1 |
| chr8 | 46192829 | G | A | 131 | Cfap97 | UTR3 | . | . | . | 8qB1.1 | rs37061939 | 0 | 1 |
| chr8 | 46208290 | A | G | 128 | Slc25a4 | intronic | . | . | . | 8qB1.1 | rs249571840 | 0 | 1 |
| chr8 | 46208309 | C | T | 168 | Slc25a4 | intronic | . | . | . | 8qB1.1 | rs218522260 | 0 | 1 |
| chr8 | 46208338 | T | A | 207 | Slc25a4 | intronic | . | . | . | 8qB1.1 | . | 0 | 1 |
| chr8 | 46229581 | G | A | 221 | . | intergenic | . | . | . | 8qB1.1 | rs36458304 | 0 | 1 |
| chr8 | 46229895 | T | A | 221 | . | intergenic | . | . | . | 8qB1.1 | . | 0 | 1 |
| chr8 | 46254857 | T | C | 221 | . | intergenic | . | . | . | 8qB1.1 | rs36760746 | 0 | 1 |
| chr8 | 46279731 | T | C | 145 | . | intergenic | . | . | . | 8qB1.1 | rs243026669 | 0 | 1 |
| chr8 | 46292169 | T | A | 151 | Helt | UTR3 | . | . | . | 8qB1.1 | rs227871348 | 0 | 1 |
| chr8 | 46292198 | C | T | 221 | Helt | UTR3 | . | . | 217 | 8qB1.1 | . | 0 | 1 |
| chr8 | 46292670 | G | A | 221 | Helt | exonic | synonymous SNV | Helt:NM_173789:exon4:c.C289T:p.L97L | . | 8qB1.1 | . | 0 | 1 |
| chr8 | 46292686 | G | A | 221 | Helt | exonic | synonymous SNV | Helt:NM_173789:exon4:c.C273T:p.H91H | . | 8qB1.1 | . | 0 | 1 |
| chr8 | 46293057 | T | G | 128 | Helt | intronic | . | . | . | 8qB1.1 | rs248761013 | 0 | 1 |
| chr8 | 46293259 | G | A | 220 | Helt | exonic | synonymous SNV | Helt:NM_173789:exon3:c.C138T:p.S46S | . | 8qB1.1 | . | 0 | 1 |
| chr8 | 46293276 | C | A | 208 | Helt | intronic | . | . | . | 8qB1.1 | rs33222689 | 0 | 1 |
| chr8 | 46293277 | C | A | 207 | Helt | intronic | . | . | . | 8qB1.1 | rs258104620 | 0 | 1 |
| chr8 | 46293859 | G | C | 121 | Helt | intronic | . | . | . | 8qB1.1 | rs240173033 | 0 | 1 |
| chr8 | 46508521 | T | C | 131 | Acsl1 | intronic | . | . | . | 8qB1.1 | rs33233181 | 0 | 1 |
| chr8 | 46511422 | C | T | 222 | Acsl1 | exonic | synonymous SNV | Acsl1:NM_001302163:exon5:c.C441T:p.S147S,Acsl1:NM_007981:exon5:c.C441T:p.S147S | . | 8qB1.1 | rs13463500 | 0 | 1 |
| chr8 | 46511585 | G | A | 164 | Acsl1 | intronic | . | . | . | 8qB1.1 | rs32974763 | 0 | 1 |
| chr8 | 46513078 | T | G | 27.8583 | Acsl1 | intronic | . | . | . | 8qB1.1 | rs36390704 | 0 | 1 |
| chr8 | 46513161 | C | T | 131 | Acsl1 | intronic | . | . | . | 8qB1.1 | rs246899381 | 0 | 1 |
| chr8 | 46513162 | A | G | 131 | Acsl1 | intronic | . | . | . | 8qB1.1 | rs258611446 | 0 | 1 |
| chr8 | 46518278 | G | A | 222 | Acsl1 | exonic | missense SNV | Acsl1:NM_001302163:exon8:c.G770A:p.R257K,Acsl1:NM_007981:exon8:c.G770A:p.R257K | . | 8qB1.1 | rs47909860 | 0 | 1 |
| chr8 | 46518282 | G | A | 222 | Acsl1 | exonic | synonymous SNV | Acsl1:NM_001302163:exon8:c.G774A:p.V258V,Acsl1:NM_007981:exon8:c.G774A:p.V258V | . | 8qB1.1 | rs33563378 | 0 | 1 |
| chr8 | 46518397 | T | C | 222 | Acsl1 | intronic | . | . | . | 8qB1.1 | rs36326190 | 0 | 1 |
| chr8 | 46518556 | A | C | 222 | Acsl1 | intronic | . | . | . | 8qB1.1 | rs36565394 | 0 | 1 |
| chr8 | 46518718 | A | G | 215 | Acsl1 | intronic | . | . | . | 8qB1.1 | rs37593729 | 0 | 1 |
| chr8 | 46518881 | T | C | 220 | Acsl1 | intronic | . | . | . | 8qB1.1 | rs32951514 | 0 | 1 |
| chr8 | 46521379 | G | A | 222 | Acsl1 | intronic | . | . | . | 8qB1.1 | rs33459562 | 0 | 1 |
| chr8 | 46521382 | C | T | 222 | Acsl1 | intronic | . | . | . | 8qB1.1 | rs33129770 | 0 | 1 |
| chr8 | 46521600 | G | A | 181 | Acsl1 | intronic | . | . | . | 8qB1.1 | rs32559869 | 0 | 1 |
| chr8 | 46524901 | C | T | 221 | Acsl1 | intronic | . | . | . | 8qB1.1 | rs229309191 | 0 | 1 |
| chr8 | 46525194 | A | G | 131 | Acsl1 | intronic | . | . | . | 8qB1.1 | rs36544425 | 0 | 1 |
| chr8 | 46525272 | T | C | 37.9887 | Acsl1 | intronic | . | . | . | 8qB1.1 | rs46161801 | 0 | 1 |
| chr8 | 46526109 | A | G | 141 | Acsl1 | intronic | . | . | . | 8qB1.1 | rs252196694 | 0 | 1 |
| chr8 | 46526936 | C | T | 90 | Acsl1 | intronic | . | . | . | 8qB1.1 | rs33189096 | 0 | 1 |
| chr8 | 46526973 | T | C | 106 | Acsl1 | intronic | . | . | . | 8qB1.1 | rs33014218 | 0 | 1 |
| chr8 | 46527252 | G | C | 222 | Acsl1 | exonic | synonymous SNV | Acsl1:NM_001302163:exon14:c.G1329C:p.V443V,Acsl1:NM_007981:exon14:c.G1329C:p.V443V | . | 8qB1.1 | rs222184235 | 0 | 1 |
| chr8 | 46527300 | T | C | 222 | Acsl1 | intronic | . | . | . | 8qB1.1 | rs248568918 | 0 | 1 |
| chr8 | 46528524 | C | T | 222 | Acsl1 | intronic | . | . | . | 8qB1.1 | rs33540140 | 0 | 1 |
| chr8 | 46530848 | G | A | 152 | Acsl1 | intronic | . | . | . | 8qB1.1 | rs49775974 | 0 | 1 |
| chr8 | 46552044 | G | C | 222 | . | upstream | . | . | 452 | 8qB1.1 | rs36550667 | 0 | 1 |
| chr8 | 46552060 | G | A | 222 | . | upstream | . | . | 452 | 8qB1.1 | rs225084617 | 0 | 1 |
| chr8 | 46552182 | C | T | 222 | Cenpu | intronic | . | . | 452 | 8qB1.1 | rs213643240 | 0 | 1 |
| chr8 | 46576540 | C | A | 99 | Primpol,Cenpu | UTR3 | . | . | . | 8qB1.1 | . | 0 | 1 |
| chr8 | 46576591 | G | A | 92 | Cenpu,Primpol | UTR3 | . | . | . | 8qB1.1 | . | 0 | 1 |
| chr8 | 46592350 | G | T | 116 | Primpol | intronic | . | . | . | 8qB1.1 | rs217797282 | 0 | 1 |
| chr8 | 46592581 | A | G | 222 | Primpol | intronic | . | . | . | 8qB1.1 | rs239998994 | 0 | 1 |
| chr8 | 46592819 | C | T | 114 | Primpol | intronic | . | . | . | 8qB1.1 | rs252083008 | 0 | 1 |
| chr8 | 46592828 | C | A | 103 | Primpol | intronic | . | . | . | 8qB1.1 | rs212478702 | 0 | 1 |
| chr8 | 46593332 | G | A | 54 | Primpol | intronic | . | . | . | 8qB1.1 | rs260985322 | 0 | 1 |
| chr8 | 46593799 | A | G | 35.8519 | Primpol | intronic | . | . | . | 8qB1.1 | rs36733079 | 0 | 1 |
| chr8 | 46599701 | T | C | 222 | Primpol | intronic | . | . | . | 8qB1.1 | rs233702843 | 0 | 1 |
| chr8 | 46599749 | T | C | 222 | Primpol | exonic | missense SNV | Primpol:NM_001001184:exon6:c.A526G:p.N176D | . | 8qB1.1 | rs36782205 | 0 | 1 |
| chr8 | 46599928 | T | C | 222 | Primpol | intronic | . | . | . | 8qB1.1 | rs51205106 | 0 | 1 |
| chr8 | 46599939 | T | A | 222 | Primpol | intronic | . | . | . | 8qB1.1 | rs37616908 | 0 | 1 |
| chr8 | 46629587 | C | T | 112 | Casp3 | intronic | . | . | . | 8qB1.1 | rs16788160 | 0 | 1 |
| chr8 | 46637941 | A | G | 221 | Casp3 | exonic | synonymous SNV | Casp3:NM_001284409:exon7:c.A627G:p.S209S,Casp3:NM_009810:exon7:c.A627G:p.S209S | . | 8qB1.1 | rs37701887 | 0 | 1 |
| chr8 | 46807793 | T | C | 220 | Irf2 | intronic | . | . | . | 8qB1.1 | rs49853783 | 0 | 1 |
| chr8 | 46846057 | T | G | 222 | Irf2 | exonic | missense SNV | Irf2:NM_008391:exon9:c.T885G:p.D295E | . | 8qB1.1 | rs13467537 | 0 | 1 |
| chr8 | 47082658 | G | A | 222 | Enpp6 | intronic | . | . | . | 8qB1.1 | . | 0 | 1 |
| chr8 | 47413312 | C | T | 221 | . | intergenic | . | . | 1524 | 8qB1.1 | . | 0 | 1 |
| chr8 | 47413435 | C | A | 184 | . | intergenic | . | . | 1524 | 8qB1.1 | rs245052741 | 0 | 1 |
| chr8 | 47413446 | G | A | 176 | . | intergenic | . | . | 1524 | 8qB1.1 | rs214207957 | 0 | 1 |
| chr8 | 47413514 | T | G | 21.3964 | . | intergenic | . | . | 1524 | 8qB1.1 | rs240240286 | 0 | 1 |
| chr8 | 47413529 | G | A | 58 | . | intergenic | . | . | 1524 | 8qB1.1 | rs216521647 | 0 | 1 |
| chr8 | 47490641 | C | T | 52 | Trappc11 | UTR3 | . | . | . | 8qB1.1 | rs47372736 | 0 | 1 |
| chr8 | 47490770 | T | C | 153 | Trappc11 | UTR3 | . | . | . | 8qB1.1 | rs33590420 | 0 | 1 |
| chr8 | 47490773 | T | G | 147 | Trappc11 | UTR3 | . | . | . | 8qB1.1 | rs32600899 | 0 | 1 |
| chr8 | 47490832 | T | A | 222 | Trappc11 | UTR3 | . | . | . | 8qB1.1 | . | 0 | 1 |
| chr8 | 47490989 | T | C | 222 | Trappc11 | intronic | . | . | . | 8qB1.1 | . | 0 | 1 |
| chr8 | 47491024 | C | T | 210 | Trappc11 | intronic | . | . | . | 8qB1.1 | rs250674563 | 0 | 1 |
| chr8 | 47493053 | T | C | 54 | Trappc11 | intronic | . | . | . | 8qB1.1 | rs46955457 | 0 | 1 |
| chr8 | 47493083 | T | A | 84 | Trappc11 | intronic | . | . | . | 8qB1.1 | rs51220646 | 0 | 1 |
| chr8 | 47493421 | C | T | 221 | Trappc11 | intronic | . | . | . | 8qB1.1 | . | 0 | 1 |
| chr8 | 47493457 | G | A | 222 | Trappc11 | intronic | . | . | . | 8qB1.1 | rs50555775 | 0 | 1 |
| chr8 | 47493470 | A | T | 174 | Trappc11 | intronic | . | . | . | 8qB1.1 | rs50436475 | 0 | 1 |
| chr8 | 47494823 | G | A | 38.7354 | Trappc11 | intronic | . | . | . | 8qB1.1 | rs50070222 | 0 | 1 |
| chr8 | 47494825 | T | C | 38.8862 | Trappc11 | intronic | . | . | . | 8qB1.1 | rs254221889 | 0 | 1 |
| chr8 | 47495114 | G | A | 221 | Trappc11 | exonic | synonymous SNV | Trappc11:NM_177240:exon28:c.C3078T:p.V1026V | . | 8qB1.1 | . | 0 | 1 |
| chr8 | 47495212 | C | T | 222 | Trappc11 | intronic | . | . | . | 8qB1.1 | . | 0 | 1 |
| chr8 | 47495237 | T | C | 222 | Trappc11 | intronic | . | . | . | 8qB1.1 | rs48481209 | 0 | 1 |
| chr8 | 47495346 | T | C | 109 | Trappc11 | intronic | . | . | . | 8qB1.1 | rs50550085 | 0 | 1 |
| chr8 | 47496980 | G | A | 222 | Trappc11 | exonic | synonymous SNV | Trappc11:NM_177240:exon27:c.C2994T:p.V998V | . | 8qB1.1 | rs240896321 | 0 | 1 |
| chr8 | 47497117 | G | A | 164 | Trappc11 | intronic | . | . | . | 8qB1.1 | rs48541759 | 0 | 1 |
| chr8 | 47497164 | G | T | 110 | Trappc11 | intronic | . | . | . | 8qB1.1 | rs47291537 | 0 | 1 |
| chr8 | 47498522 | T | C | 92 | Trappc11 | intronic | . | . | . | 8qB1.1 | rs46536213 | 0 | 1 |
| chr8 | 47498660 | C | A | 222 | Trappc11 | exonic | missense SNV | Trappc11:NM_177240:exon26:c.G2957T:p.W986L | . | 8qB1.1 | . | 0 | 1 |
| chr8 | 47498713 | C | T | 222 | Trappc11 | exonic | synonymous SNV | Trappc11:NM_177240:exon26:c.G2904A:p.P968P | . | 8qB1.1 | . | 0 | 1 |
| chr8 | 47498778 | A | T | 222 | Trappc11 | intronic | . | . | . | 8qB1.1 | rs45778508 | 0 | 1 |
| chr8 | 47501459 | G | T | 121 | Trappc11 | intronic | . | . | . | 8qB1.1 | rs47999274 | 0 | 1 |
| chr8 | 47501472 | T | C | 187 | Trappc11 | intronic | . | . | . | 8qB1.1 | rs48420696 | 0 | 1 |
| chr8 | 47501493 | G | A | 222 | Trappc11 | intronic | . | . | . | 8qB1.1 | rs51624418 | 0 | 1 |
| chr8 | 47501781 | G | A | 222 | Trappc11 | intronic | . | . | . | 8qB1.1 | rs252264824 | 0 | 1 |
| chr8 | 47501925 | G | A | 222 | Trappc11 | intronic | . | . | . | 8qB1.1 | rs245146295 | 0 | 1 |
| chr8 | 47502020 | A | G | 127 | Trappc11 | intronic | . | . | . | 8qB1.1 | rs585224309 | 0 | 1 |
| chr8 | 47503133 | T | A | 40.8725 | Trappc11 | intronic | . | . | . | 8qB1.1 | rs585946731 | 0 | 1 |
| chr8 | 47503203 | T | C | 143 | Trappc11 | intronic | . | . | . | 8qB1.1 | rs219175685 | 0 | 1 |
| chr8 | 47503247 | G | A | 157 | Trappc11 | intronic | . | . | . | 8qB1.1 | rs47720159 | 0 | 1 |
| chr8 | 47503575 | C | T | 103 | Trappc11 | intronic | . | . | . | 8qB1.1 | . | 0 | 1 |
| chr8 | 47503588 | G | A | 87 | Trappc11 | intronic | . | . | . | 8qB1.1 | . | 0 | 1 |
| chr8 | 47503746 | T | C | 104 | Trappc11 | intronic | . | . | . | 8qB1.1 | rs47457314 | 0 | 1 |
| chr8 | 47507477 | T | C | 109 | Trappc11 | intronic | . | . | . | 8qB1.1 | rs46291344 | 0 | 1 |
| chr8 | 47507725 | T | C | 221 | Trappc11 | exonic | synonymous SNV | Trappc11:NM_177240:exon19:c.A1905G:p.Q635Q | . | 8qB1.1 | rs47460720 | 0 | 1 |
| chr8 | 47511068 | A | G | 222 | Trappc11 | intronic | . | . | . | 8qB1.1 | . | 0 | 1 |
| chr8 | 47511071 | A | G | 222 | Trappc11 | intronic | . | . | . | 8qB1.1 | . | 0 | 1 |
| chr8 | 47512514 | A | G | 222 | Trappc11 | intronic | . | . | . | 8qB1.1 | rs48864098 | 0 | 1 |
| chr8 | 47513368 | C | A | 221 | Trappc11 | exonic | synonymous SNV | Trappc11:NM_177240:exon15:c.G1482T:p.S494S | . | 8qB1.1 | rs46715794 | 0 | 1 |
| chr8 | 47514019 | G | T | 222 | Trappc11 | intronic | . | . | . | 8qB1.1 | . | 0 | 1 |
| chr8 | 47514051 | A | G | 222 | Trappc11 | exonic | synonymous SNV | Trappc11:NM_177240:exon12:c.T1287C:p.S429S | . | 8qB1.1 | rs33246330 | 0 | 1 |
| chr8 | 47514231 | C | T | 164 | Trappc11 | intronic | . | . | . | 8qB1.1 | . | 0 | 1 |
| chr8 | 47514253 | T | C | 128 | Trappc11 | intronic | . | . | . | 8qB1.1 | rs32687885 | 0 | 1 |
| chr8 | 47514898 | C | T | 222 | Trappc11 | intronic | . | . | . | 8qB1.1 | . | 0 | 1 |
| chr8 | 47516315 | C | T | 155 | Trappc11 | intronic | . | . | . | 8qB1.1 | . | 0 | 1 |
| chr8 | 47519512 | G | T | 222 | Trappc11 | intronic | . | . | . | 8qB1.1 | rs33599539 | 0 | 1 |
| chr8 | 47519715 | A | G | 222 | Trappc11 | intronic | . | . | . | 8qB1.1 | rs33205531 | 0 | 1 |
| chr8 | 47519800 | C | T | 222 | Trappc11 | intronic | . | . | . | 8qB1.1 | . | 0 | 1 |
| chr8 | 47524558 | C | T | 80 | Trappc11 | exonic | missense SNV | Trappc11:NM_177240:exon7:c.G701A:p.S234N | . | 8qB1.1 | . | 0 | 1 |
| chr8 | 47524882 | T | A | 128 | Trappc11 | intronic | . | . | . | 8qB1.1 | rs3696110 | 0 | 1 |
| chr8 | 47529313 | C | T | 221 | Trappc11 | intronic | . | . | . | 8qB1.1 | . | 0 | 1 |
| chr8 | 47529518 | C | A | 222 | Trappc11 | intronic | . | . | . | 8qB1.1 | . | 0 | 1 |
| chr8 | 47530861 | G | A | 222 | Trappc11 | UTR5 | . | . | . | 8qB1.1 | rs13479742 | 0 | 1 |
| chr8 | 47530932 | C | T | 131 | Trappc11 | intronic | . | . | . | 8qB1.1 | rs33481014 | 0 | 1 |
| chr8 | 47533796 | A | G | 169 | Rwdd4a | UTR5 | . | . | 734 | 8qB1.1 | rs13462866 | 0 | 1 |
| chr8 | 47542679 | C | T | 221 | Rwdd4a | intronic | . | . | . | 8qB1.1 | . | 0 | 1 |
| chr8 | 47543059 | G | T | 221 | Rwdd4a | intronic | . | . | . | 8qB1.1 | rs33197500 | 0 | 1 |
| chr8 | 47543066 | G | A | 221 | Rwdd4a | intronic | . | . | . | 8qB1.1 | . | 0 | 1 |
| chr8 | 47544134 | G | A | 221 | Rwdd4a | exonic | synonymous SNV | Rwdd4a:NM_203507:exon5:c.G387A:p.S129S | . | 8qB1.1 | rs226002935 | 0 | 1 |
| chr8 | 47547878 | A | G | 222 | Rwdd4a | intronic | . | . | . | 8qB1.1 | rs47262454 | 0 | 1 |
| chr8 | 47550501 | C | G | 34.9183 | Rwdd4a | intronic | . | . | . | 8qB1.1 | rs238954572 | 0 | 1 |
| chr8 | 47550600 | G | T | 128 | Rwdd4a | intronic | . | . | . | 8qB1.1 | . | 0 | 1 |
| chr8 | 47550612 | A | G | 131 | Rwdd4a | intronic | . | . | . | 8qB1.1 | rs219888415 | 0 | 1 |
| chr8 | 47550901 | T | C | 120 | Rwdd4a | UTR3 | . | . | . | 8qB1.1 | rs232881041 | 0 | 1 |
| chr8 | 47626739 | G | A | 222 | . | intergenic | . | . | . | 8qB1.1 | . | 0 | 1 |
| chr8 | 47626919 | G | A | 128 | . | intergenic | . | . | . | 8qB1.1 | . | 0 | 1 |
| chr8 | 47632199 | G | C | 100 | . | intergenic | . | . | . | 8qB1.1 | . | 0 | 1 |
| chr8 | 47632217 | G | C | 125 | . | intergenic | . | . | . | 8qB1.1 | . | 0 | 1 |
| chr8 | 47632241 | T | G | 145 | . | intergenic | . | . | . | 8qB1.1 | . | 0 | 1 |
| chr8 | 47632615 | A | G | 222 | . | intergenic | . | . | . | 8qB1.1 | . | 0 | 1 |
| chr8 | 47674605 | A | T | 169 | Ing2 | exonic | missense SNV | Ing2:NM_023503:exon1:c.T76A:p.S26T | 2292 | 8qB1.1 | rs224608791 | 0 | 1 |
| chr8 | 47676402 | A | C | 222 | . | intergenic | . | . | . | 8qB1.1 | . | 0 | 1 |
| chr8 | 47711029 | C | T | 222 | Cdkn2aip | exonic | missense SNV | Cdkn2aip:NM_172407:exon3:c.G1648A:p.V550I | . | 8qB1.1 | . | 0 | 1 |
| chr8 | 47712167 | C | T | 221 | Cdkn2aip | exonic | synonymous SNV | Cdkn2aip:NM_172407:exon3:c.G510A:p.P170P | . | 8qB1.1 | . | 0 | 1 |
| chr8 | 47712371 | T | G | 220 | Cdkn2aip | intronic | . | . | . | 8qB1.1 | rs223692754 | 0 | 1 |
| chr8 | 47713101 | T | C | 221 | Cdkn2aip | intronic | . | . | . | 8qB1.1 | . | 0 | 1 |
| chr8 | 47822259 | G | A | 222 | Cldn24 | exonic | synonymous SNV | Cldn24:NM_001111318:exon1:c.G117A:p.E39E | . | 8qB1.1 | rs235316232 | 0 | 1 |
| chr8 | 47822476 | G | A | 102 | Cldn24 | exonic | missense SNV | Cldn24:NM_001111318:exon1:c.G334A:p.E112K | . | 8qB1.1 | . | 0 | 1 |
| chr8 | 47822969 | C | T | 123 | . | downstream | . | . | . | 8qB1.1 | rs242552030 | 0 | 1 |
| chr8 | 47822986 | T | G | 115 | . | downstream | . | . | . | 8qB1.1 | rs52567876 | 0 | 1 |
| chr8 | 47824416 | C | A | 98 | . | upstream | . | . | . | 8qB1.1 | . | 0 | 1 |
| chr8 | 47825117 | A | G | 222 | Cldn22 | exonic | missense SNV | Cldn22:NM_029383:exon1:c.A589G:p.S197G | . | 8qB1.1 | rs259882812 | 0 | 1 |
| chr8 | 47825134 | G | A | 222 | Cldn22 | exonic | synonymous SNV | Cldn22:NM_029383:exon1:c.G606A:p.A202A | . | 8qB1.1 | rs239900519 | 0 | 1 |
| chr8 | 47825139 | C | T | 222 | Cldn22 | exonic | missense SNV | Cldn22:NM_029383:exon1:c.C611T:p.P204L | . | 8qB1.1 | rs46060820 | 0 | 1 |
| chr8 | 47846322 | G | A | 133 | Wwc2 | intronic | . | . | . | 8qB1.1 | . | 0 | 1 |
| chr8 | 47856067 | C | A | 146 | Wwc2 | intronic | . | . | . | 8qB1.1 | rs263741301 | 0 | 1 |
| chr8 | 47856391 | G | T | 222 | Wwc2 | intronic | . | . | . | 8qB1.1 | rs6163586 | 0 | 1 |
| chr8 | 47856444 | G | A | 145 | Wwc2 | intronic | . | . | . | 8qB1.1 | rs6176752 | 0 | 1 |
| chr8 | 47858667 | T | C | 145 | Wwc2 | intronic | . | . | . | 8qB1.1 | rs6213286 | 0 | 1 |
| chr8 | 47858672 | C | T | 146 | Wwc2 | intronic | . | . | . | 8qB1.1 | rs6213298 | 0 | 1 |
| chr8 | 47858684 | A | G | 210 | Wwc2 | intronic | . | . | . | 8qB1.1 | rs6213318 | 0 | 1 |
| chr8 | 47864188 | G | A | 65 | Wwc2 | intronic | . | . | . | 8qB1.1 | . | 0 | 1 |
| chr8 | 47864460 | T | C | 95 | Wwc2 | intronic | . | . | . | 8qB1.1 | rs6209138 | 0 | 1 |
| chr8 | 47864462 | T | C | 93 | Wwc2 | intronic | . | . | . | 8qB1.1 | rs6209140 | 0 | 1 |
| chr8 | 47868072 | G | T | 134 | Wwc2 | intronic | . | . | . | 8qB1.1 | rs51699818 | 0 | 1 |
| chr8 | 47868206 | A | G | 222 | Wwc2 | exonic | synonymous SNV | Wwc2:NM_133791:exon11:c.T1872C:p.D624D | . | 8qB1.1 | rs50367312 | 0 | 1 |
| chr8 | 47868334 | T | C | 221 | Wwc2 | exonic | missense SNV | Wwc2:NM_133791:exon11:c.A1744G:p.T582A | . | 8qB1.1 | rs33262801 | 0 | 1 |
| chr8 | 47868828 | T | A | 221 | Wwc2 | intronic | . | . | . | 8qB1.1 | . | 0 | 1 |
| chr8 | 47869759 | G | A | 222 | Wwc2 | intronic | . | . | . | 8qB1.1 | . | 0 | 1 |
| chr8 | 47869936 | A | G | 130 | Wwc2 | intronic | . | . | . | 8qB1.1 | . | 0 | 1 |
| chr8 | 47869952 | T | G | 129 | Wwc2 | intronic | . | . | . | 8qB1.1 | rs49622895 | 0 | 1 |
| chr8 | 47878667 | A | G | 133 | Wwc2 | intronic | . | . | . | 8qB1.1 | rs224851289 | 0 | 1 |
| chr8 | 47878912 | C | T | 221 | Wwc2 | intronic | . | . | . | 8qB1.1 | . | 0 | 1 |
| chr8 | 47883412 | A | G | 222 | Wwc2 | intronic | . | . | . | 8qB1.1 | rs48873211 | 0 | 1 |
| chr8 | 47887274 | G | A | 128 | Wwc2 | intronic | . | . | . | 8qB1.1 | rs47107496 | 0 | 1 |
| chr8 | 47899300 | A | G | 142 | Wwc2 | intronic | . | . | . | 8qB1.1 | rs247906988 | 0 | 1 |
| chr8 | 47899409 | C | T | 222 | Wwc2 | intronic | . | . | . | 8qB1.1 | rs51648609 | 0 | 1 |
| chr8 | 47900783 | G | A | 222 | Wwc2 | exonic | synonymous SNV | Wwc2:NM_133791:exon3:c.C315T:p.S105S | . | 8qB1.1 | . | 0 | 1 |
| chr8 | 48070673 | T | C | 37.2315 | . | intergenic | . | . | . | 8qB1.1 | rs224395434 | 0 | 1 |
| chr8 | 48070677 | C | T | 32.3675 | . | intergenic | . | . | . | 8qB1.1 | rs239879864 | 0 | 1 |
| chr8 | 48070685 | T | G | 32.3675 | . | intergenic | . | . | . | 8qB1.1 | rs47292423 | 0 | 1 |
| chr8 | 48228403 | T | C | 222 | Tenm3 | UTR3 | . | . | . | 8qB1.1 | rs255463935 | 0 | 1 |

| | | | | | | | | | | | | | |
|---|---|---|---|---|---|---|---|---|---|---|---|---|---|
| chr8 | 48228479 | C | T | 222 | Tenm3 | exonic | synonymous SNV | Tenm3:NM_001145937:exon27:c.G8067A:p.Q2689Q,Tenm3:NM_011857:exon29:c.G8115A:p.Q2705Q | . | 8qB1.1 | rs240469361 | 0 | 1 |
| chr8 | 48228533 | G | A | 222 | Tenm3 | exonic | synonymous SNV | Tenm3:NM_001145937:exon27:c.C8013T:p.Y2671Y,Tenm3:NM_011857:exon29:c.C8061T:p.Y2687Y | 626 | 8qB1.1 | rs257774542 | 0 | 1 |
| chr8 | 48228551 | C | T | 216 | Tenm3 | exonic | synonymous SNV | Tenm3:NM_001145937:exon27:c.G7995A:p.Q2665Q,Tenm3:NM_011857:exon29:c.G8043A:p.Q2681Q | 626 | 8qB1.1 | . | 0 | 1 |
| chr8 | 48228587 | A | G | 148 | Tenm3 | exonic | synonymous SNV | Tenm3:NM_001145937:exon27:c.T7959C:p.G2653G,Tenm3:NM_011857:exon29:c.T8007C:p.G2669G | 626 | 8qB1.1 | rs243843461 | 0 | 1 |
| chr8 | 48228908 | T | C | 222 | Tenm3 | exonic | synonymous SNV | Tenm3:NM_001145937:exon27:c.A7638G:p.T2546T,Tenm3:NM_011857:exon29:c.A7686G:p.T2562T | 626 | 8qB1.1 | rs258014536 | 0 | 1 |
| chr8 | 48229049 | G | A | 222 | Tenm3 | exonic | synonymous SNV | Tenm3:NM_001145937:exon27:c.C7497T:p.G2499G,Tenm3:NM_011857:exon29:c.C7545T:p.G2515G | 626 | 8qB1.1 | rs260793395 | 0 | 1 |
| chr8 | 48229100 | G | A | 222 | Tenm3 | exonic | synonymous SNV | Tenm3:NM_001145937:exon27:c.C7446T:p.A2482A,Tenm3:NM_011857:exon29:c.C7494T:p.A2498A | 626 | 8qB1.1 | . | 0 | 1 |
| chr8 | 48229224 | T | A | 222 | Tenm3 | intronic | . | . | . | 8qB1.1 | rs222608735 | 0 | 1 |
| chr8 | 48232014 | A | G | 222 | Tenm3 | exonic | synonymous SNV | Tenm3:NM_001145937:exon26:c.T7338C:p.D2446D,Tenm3:NM_011857:exon28:c.T7386C:p.D2462D | . | 8qB1.1 | rs222479048 | 0 | 1 |
| chr8 | 48232160 | A | G | 222 | Tenm3 | intronic | . | . | . | 8qB1.1 | rs224533551 | 0 | 1 |
| chr8 | 48235468 | C | T | 222 | Tenm3 | exonic | synonymous SNV | Tenm3:NM_001145937:exon25:c.G7035A:p.P2345P,Tenm3:NM_011857:exon27:c.G7083A:p.P2361P | . | 8qB1.1 | . | 0 | 1 |
| chr8 | 48235486 | G | A | 222 | Tenm3 | exonic | synonymous SNV | Tenm3:NM_001145937:exon25:c.C7017T:p.H2339H,Tenm3:NM_011857:exon27:c.C7065T:p.H2355H | . | 8qB1.1 | . | 0 | 1 |
| chr8 | 48235516 | G | A | 222 | Tenm3 | exonic | synonymous SNV | Tenm3:NM_001145937:exon25:c.C6987T:p.N2329N,Tenm3:NM_011857:exon27:c.C7035T:p.N2345N | . | 8qB1.1 | . | 0 | 1 |
| chr8 | 48236029 | G | A | 222 | Tenm3 | exonic | synonymous SNV | Tenm3:NM_001145937:exon25:c.C6474T:p.N2158N,Tenm3:NM_011857:exon27:c.C6522T:p.N2174N | . | 8qB1.1 | rs260557162 | 0 | 1 |
| chr8 | 48236116 | C | T | 222 | Tenm3 | exonic | synonymous SNV | Tenm3:NM_001145937:exon25:c.G6387A:p.A2129A,Tenm3:NM_011857:exon27:c.G6435A:p.A2145A | . | 8qB1.1 | rs245181199 | 0 | 1 |
| chr8 | 48236146 | A | G | 222 | Tenm3 | exonic | synonymous SNV | Tenm3:NM_001145937:exon25:c.T6357C:p.I2119I,Tenm3:NM_011857:exon27:c.T6405C:p.I2135I | . | 8qB1.1 | rs265287301 | 0 | 1 |
| chr8 | 48236161 | C | T | 222 | Tenm3 | exonic | synonymous SNV | Tenm3:NM_001145937:exon25:c.G6342A:p.K2114K,Tenm3:NM_011857:exon27:c.G6390A:p.K2130K | . | 8qB1.1 | rs229436852 | 0 | 1 |
| chr8 | 48236581 | C | T | 222 | Tenm3 | exonic | synonymous SNV | Tenm3:NM_001145937:exon25:c.G5922A:p.A1974A,Tenm3:NM_011857:exon27:c.G5970A:p.A1990A | . | 8qB1.1 | . | 0 | 1 |
| chr8 | 48236755 | A | G | 221 | Tenm3 | exonic | synonymous SNV | Tenm3:NM_001145937:exon25:c.T5748C:p.N1916N,Tenm3:NM_011857:exon27:c.T5796C:p.N1932N | . | 8qB1.1 | rs253665725 | 0 | 1 |
| chr8 | 48236940 | G | C | 142 | Tenm3 | intronic | . | . | . | 8qB1.1 | rs247683992 | 0 | 1 |
| chr8 | 48236941 | T | C | 81 | Tenm3 | intronic | . | . | . | 8qB1.1 | rs218448733 | 0 | 1 |
| chr8 | 48240124 | G | C | 120 | Tenm3 | intronic | . | . | . | 8qB1.1 | . | 0 | 1 |
| chr8 | 48240146 | A | G | 115 | Tenm3 | intronic | . | . | . | 8qB1.1 | rs215754944 | 0 | 1 |
| chr8 | 48240198 | T | C | 177 | Tenm3 | intronic | . | . | . | 8qB1.1 | . | 0 | 1 |
| chr8 | 48240257 | C | T | 222 | Tenm3 | exonic | synonymous SNV | Tenm3:NM_001145937:exon24:c.G5589A:p.T1863T,Tenm3:NM_011857:exon26:c.G5637A:p.T1879T | . | 8qB1.1 | . | 0 | 1 |
| chr8 | 48240371 | G | A | 222 | Tenm3 | exonic | synonymous SNV | Tenm3:NM_001145937:exon24:c.C5475T:p.S1825S,Tenm3:NM_011857:exon26:c.C5523T:p.S1841S | . | 8qB1.1 | rs250923575 | 0 | 1 |
| chr8 | 48240374 | T | G | 222 | Tenm3 | exonic | synonymous SNV | Tenm3:NM_001145937:exon24:c.A5472C:p.S1824S,Tenm3:NM_011857:exon26:c.A5520C:p.S1840S | . | 8qB1.1 | rs212616235 | 0 | 1 |
| chr8 | 48240386 | G | A | 212 | Tenm3 | exonic | synonymous SNV | Tenm3:NM_001145937:exon24:c.C5460T:p.N1820N,Tenm3:NM_011857:exon26:c.C5508T:p.N1836N | . | 8qB1.1 | rs229756390 | 0 | 1 |
| chr8 | 48240458 | G | A | 222 | Tenm3 | exonic | synonymous SNV | Tenm3:NM_001145937:exon24:c.C5388T:p.L1796L,Tenm3:NM_011857:exon26:c.C5436T:p.L1812L | . | 8qB1.1 | . | 0 | 1 |
| chr8 | 48240573 | C | T | 221 | Tenm3 | intronic | . | . | . | 8qB1.1 | . | 0 | 1 |
| chr8 | 48240588 | C | T | 220 | Tenm3 | intronic | . | . | . | 8qB1.1 | . | 0 | 1 |
| chr8 | 48240720 | C | G | 90 | Tenm3 | intronic | . | . | . | 8qB1.1 | . | 0 | 1 |
| chr8 | 48254301 | C | G | 73 | Tenm3 | intronic | . | . | . | 8qB1.1 | rs47705281 | 0 | 1 |
| chr8 | 48254322 | A | G | 58 | Tenm3 | intronic | . | . | . | 8qB1.1 | rs50393517 | 0 | 1 |
| chr8 | 48254328 | G | A | 60 | Tenm3 | intronic | . | . | . | 8qB1.1 | . | 0 | 1 |
| chr8 | 48256672 | A | T | 98 | Tenm3 | intronic | . | . | . | 8qB1.1 | rs234790361 | 0 | 1 |
| chr8 | 48256674 | T | G | 94 | Tenm3 | intronic | . | . | . | 8qB1.1 | rs248942033 | 0 | 1 |
| chr8 | 48256723 | C | T | 135 | Tenm3 | intronic | . | . | . | 8qB1.1 | rs582056171 | 0 | 1 |
| chr8 | 48256872 | C | T | 222 | Tenm3 | exonic | synonymous SNV | Tenm3:NM_001145937:exon22:c.G5028A:p.S1676S,Tenm3:NM_011857:exon24:c.G5076A:p.S1692S | . | 8qB1.1 | . | 0 | 1 |
| chr8 | 48256971 | G | A | 222 | Tenm3 | exonic | synonymous SNV | Tenm3:NM_001145937:exon22:c.C4929T:p.F1643F,Tenm3:NM_011857:exon24:c.C4977T:p.F1659F | . | 8qB1.1 | . | 0 | 1 |
| chr8 | 48257004 | A | G | 222 | Tenm3 | exonic | synonymous SNV | Tenm3:NM_001145937:exon22:c.T4896C:p.Y1632Y,Tenm3:NM_011857:exon24:c.T4944C:p.Y1648Y | . | 8qB1.1 | rs51778661 | 0 | 1 |
| chr8 | 48257157 | A | G | 115 | Tenm3 | intronic | . | . | . | 8qB1.1 | rs265570274 | 0 | 1 |
| chr8 | 48257208 | C | G | 42.5268 | Tenm3 | intronic | . | . | . | 8qB1.1 | rs231208956 | 0 | 1 |
| chr8 | 48257209 | T | C | 42.7393 | Tenm3 | intronic | . | . | . | 8qB1.1 | rs255719802 | 0 | 1 |
| chr8 | 48270319 | G | A | 30.8759 | Tenm3 | intronic | . | . | . | 8qB1.1 | rs216918174 | 0 | 1 |
| chr8 | 48270322 | A | T | 30.8759 | Tenm3 | intronic | . | . | . | 8qB1.1 | rs237684182 | 0 | 1 |
| chr8 | 48270415 | G | A | 52 | Tenm3 | intronic | . | . | . | 8qB1.1 | rs250839405 | 0 | 1 |
| chr8 | 48270421 | T | C | 47.1396 | Tenm3 | intronic | . | . | . | 8qB1.1 | rs220454641 | 0 | 1 |
| chr8 | 48270433 | C | T | 43.9523 | Tenm3 | intronic | . | . | . | 8qB1.1 | rs251473052 | 0 | 1 |
| chr8 | 48270491 | C | A | 156 | Tenm3 | intronic | . | . | . | 8qB1.1 | rs239436368 | 0 | 1 |
| chr8 | 48270503 | T | C | 203 | Tenm3 | intronic | . | . | . | 8qB1.1 | rs256153835 | 0 | 1 |
| chr8 | 48276930 | G | A | 222 | Tenm3 | intronic | . | . | . | 8qB1.1 | . | 0 | 1 |
| chr8 | 48277001 | T | G | 222 | Tenm3 | intronic | . | . | . | 8qB1.1 | rs49411243 | 0 | 1 |
| chr8 | 48277039 | T | C | 129 | Tenm3 | intronic | . | . | . | 8qB1.1 | rs50600318 | 0 | 1 |
| chr8 | 48277706 | A | T | 222 | Tenm3 | exonic | synonymous SNV | Tenm3:NM_001145937:exon20:c.T3978A:p.A1326A,Tenm3:NM_011857:exon22:c.T4026A:p.A1342A | . | 8qB1.1 | rs214739058 | 0 | 1 |
| chr8 | 48278010 | T | C | 113 | Tenm3 | intronic | . | . | . | 8qB1.1 | rs243202707 | 0 | 1 |
| chr8 | 48287602 | C | T | 129 | Tenm3 | intronic | . | . | . | 8qB1.1 | rs250694298 | 0 | 1 |
| chr8 | 48293320 | C | T | 113 | Tenm3 | intronic | . | . | . | 8qB1.2 | rs48935981 | 0 | 1 |
| chr8 | 48293390 | C | T | 160 | Tenm3 | intronic | . | . | . | 8qB1.2 | rs241423400 | 0 | 1 |
| chr8 | 48293604 | C | T | 222 | Tenm3 | exonic | synonymous SNV | Tenm3:NM_001145937:exon16:c.G3138A:p.Q1046Q,Tenm3:NM_011857:exon17:c.G3165A:p.Q1055Q | . | 8qB1.2 | rs51960462 | 0 | 1 |
| chr8 | 48298740 | T | A | 175 | Tenm3 | intronic | . | . | . | 8qB1.2 | rs218735646 | 0 | 1 |
| chr8 | 48298849 | A | C | 221 | Tenm3 | exonic | synonymous SNV | Tenm3:NM_001145937:exon15:c.T2904G:p.S968S,Tenm3:NM_011857:exon16:c.T2931G:p.S977S | . | 8qB1.2 | rs250783998 | 0 | 1 |
| chr8 | 48299141 | T | A | 83 | Tenm3 | intronic | . | . | . | 8qB1.2 | rs33115539 | 0 | 1 |
| chr8 | 48299160 | G | T | 34.9123 | Tenm3 | intronic | . | . | . | 8qB1.2 | rs33511545 | 0 | 1 |
| chr8 | 48299630 | G | A | 222 | Tenm3 | exonic | missense SNV | Tenm3:NM_001145937:exon14:c.C2669T:p.S890L,Tenm3:NM_011857:exon15:c.C2696T:p.S899L | . | 8qB1.2 | rs266042493 | 0 | 1 |
| chr8 | 48299731 | A | G | 222 | Tenm3 | intronic | . | . | . | 8qB1.2 | rs33044274 | 0 | 1 |
| chr8 | 48299822 | G | C | 149 | Tenm3 | intronic | . | . | . | 8qB1.2 | rs48570713 | 0 | 1 |
| chr8 | 48299823 | G | A | 145 | Tenm3 | intronic | . | . | . | 8qB1.2 | rs587204602 | 0 | 1 |
| chr8 | 48300572 | T | C | 43.9904 | Tenm3 | intronic | . | . | . | 8qB1.2 | rs225863506 | 0 | 1 |
| chr8 | 48300658 | C | A | 118 | Tenm3 | intronic | . | . | . | 8qB1.2 | rs33029539 | 0 | 1 |
| chr8 | 48300781 | G | T | 222 | Tenm3 | exonic | synonymous SNV | Tenm3:NM_001145937:exon13:c.C2559A:p.L853L,Tenm3:NM_011857:exon14:c.C2586A:p.L862L | . | 8qB1.2 | rs213016817 | 0 | 1 |
| chr8 | 48301059 | C | T | 147 | Tenm3 | intronic | . | . | . | 8qB1.2 | rs30945459 | 0 | 1 |
| chr8 | 48301068 | T | C | 143 | Tenm3 | intronic | . | . | . | 8qB1.2 | rs30945462 | 0 | 1 |
| chr8 | 48310529 | T | G | 110 | Tenm3 | intronic | . | . | . | 8qB1.2 | rs30953417 | 0 | 1 |
| chr8 | 48310568 | A | C | 194 | Tenm3 | intronic | . | . | . | 8qB1.2 | rs30953420 | 0 | 1 |
| chr8 | 48313006 | G | A | 106 | Tenm3 | intronic | . | . | . | 8qB1.2 | rs30955081 | 0 | 1 |
| chr8 | 48335311 | T | A | 113 | Tenm3 | intronic | . | . | . | 8qB1.2 | rs30955592 | 0 | 1 |
| chr8 | 48335467 | T | C | 222 | Tenm3 | intronic | . | . | . | 8qB1.2 | rs30956495 | 0 | 1 |
| chr8 | 48335495 | C | T | 222 | Tenm3 | intronic | . | . | . | 8qB1.2 | rs219083511 | 0 | 1 |
| chr8 | 48335695 | C | G | 222 | Tenm3 | exonic | synonymous SNV | Tenm3:NM_001145937:exon11:c.G2115C:p.P705P,Tenm3:NM_011857:exon11:c.G2115C:p.P705P | . | 8qB1.2 | rs222586963 | 0 | 1 |
| chr8 | 48335773 | T | C | 212 | Tenm3 | exonic | synonymous SNV | Tenm3:NM_001145937:exon11:c.A2037G:p.E679E,Tenm3:NM_011857:exon11:c.A2037G:p.E679E | . | 8qB1.2 | rs30956501 | 0 | 1 |
| chr8 | 48340889 | C | T | 173 | Tenm3 | intronic | . | . | . | 8qB1.2 | rs30963808 | 0 | 1 |
| chr8 | 48341174 | G | A | 222 | Tenm3 | intronic | . | . | . | 8qB1.2 | rs30964724 | 0 | 1 |
| chr8 | 48341227 | T | C | 185 | Tenm3 | intronic | . | . | . | 8qB1.2 | rs30964727 | 0 | 1 |
| chr8 | 48342184 | T | C | 181 | Tenm3 | intronic | . | . | . | 8qB1.2 | rs33275354 | 0 | 1 |
| chr8 | 48343015 | T | C | 20.7239 | Tenm3 | intronic | . | . | . | 8qB1.2 | rs33395548 | 0 | 1 |
| chr8 | 48343092 | C | T | 126 | Tenm3 | intronic | . | . | . | 8qB1.2 | rs33323711 | 0 | 1 |
| chr8 | 48343098 | A | G | 128 | Tenm3 | intronic | . | . | . | 8qB1.2 | rs33003654 | 0 | 1 |
| chr8 | 48349420 | G | A | 222 | Tenm3 | intronic | . | . | . | 8qB1.2 | . | 0 | 1 |
| chr8 | 48367167 | C | T | 126 | Tenm3 | intronic | . | . | . | 8qB1.2 | rs211806085 | 0 | 1 |
| chr8 | 48367656 | G | A | 76 | Tenm3 | intronic | . | . | . | 8qB1.2 | rs30960993 | 0 | 1 |
| chr8 | 48395330 | A | G | 67 | Tenm3 | intronic | . | . | . | 8qB1.2 | rs30960952 | 0 | 1 |
| chr8 | 48395399 | G | A | 211 | Tenm3 | intronic | . | . | . | 8qB1.2 | rs30961805 | 0 | 1 |
| chr8 | 48395470 | T | C | 222 | Tenm3 | exonic | synonymous SNV | Tenm3:NM_001145937:exon4:c.A954G:p.S318S,Tenm3:NM_011857:exon4:c.A954G:p.S318S | . | 8qB1.2 | rs30961808 | 0 | 1 |
| chr8 | 48416956 | C | T | 200 | Tenm3 | intronic | . | . | . | 8qB1.2 | rs237248396 | 0 | 1 |
| chr8 | 48416992 | G | A | 212 | Tenm3 | intronic | . | . | . | 8qB1.2 | rs30967527 | 0 | 1 |
| chr8 | 48417273 | T | C | 221 | Tenm3 | intronic | . | . | . | 8qB1.2 | rs30967530 | 0 | 1 |
| chr8 | 48417323 | C | T | 221 | Tenm3 | intronic | . | . | . | 8qB1.2 | rs30968415 | 0 | 1 |
| chr8 | 48631109 | T | C | 37.4118 | Tenm3 | intronic | . | . | . | 8qB1.2 | . | 0 | 1 |
| chr8 | 49058891 | A | T | 221 | . | intergenic | . | . | . | 8qB1.2 | . | 0 | 1 |
| chr8 | 51879577 | T | C | 55 | . | intergenic | . | . | . | 8qB1.3 | rs49563352 | 0 | 1 |
| chr8 | 51879626 | A | G | 124 | . | intergenic | . | . | . | 8qB1.3 | rs46409987 | 0 | 1 |
| chr8 | 51879658 | C | T | 158 | . | intergenic | . | . | . | 8qB1.3 | rs47233523 | 0 | 1 |
| chr8 | 51879765 | G | C | 204 | . | intergenic | . | . | . | 8qB1.3 | rs50137575 | 0 | 1 |
| chr8 | 51879796 | A | G | 188 | . | intergenic | . | . | . | 8qB1.3 | rs50227867 | 0 | 1 |
| chr8 | 51880070 | A | C | 221 | . | intergenic | . | . | . | 8qB1.3 | rs48835081 | 0 | 1 |
| chr8 | 52278393 | C | T | 106 | . | intergenic | . | . | . | 8qB1.3 | rs228577273 | 0 | 1 |
| chr8 | 52278415 | T | C | 140 | . | intergenic | . | . | . | 8qB1.3 | rs31274976 | 0 | 1 |
| chr8 | 52278469 | C | A | 222 | . | intergenic | . | . | . | 8qB1.3 | rs31274979 | 0 | 1 |
| chr8 | 52278834 | A | C | 212 | . | intergenic | . | . | . | 8qB1.3 | rs31276584 | 0 | 1 |
| chr8 | 52278839 | C | T | 215 | . | intergenic | . | . | . | 8qB1.3 | rs232584099 | 0 | 1 |
| chr8 | 52278917 | A | G | 214 | . | intergenic | . | . | . | 8qB1.3 | rs31276590 | 0 | 1 |
| chr8 | 52278937 | G | C | 222 | . | intergenic | . | . | . | 8qB1.3 | rs31277376 | 0 | 1 |
| chr8 | 52279034 | T | G | 222 | . | intergenic | . | . | . | 8qB1.3 | rs215757610 | 0 | 1 |
| chr8 | 52279036 | A | C | 222 | . | intergenic | . | . | . | 8qB1.3 | rs31277382 | 0 | 1 |
| chr8 | 52279182 | A | G | 131 | . | intergenic | . | . | . | 8qB1.3 | rs31278151 | 0 | 1 |
| chr8 | 52279339 | C | G | 37.9766 | . | intergenic | . | . | . | 8qB1.3 | rs31279676 | 0 | 1 |
| chr8 | 53511702 | G | A | 104 | Aga | UTR5 | . | . | . | 8qB1.3 | rs33041218 | 0 | 1 |
| chr8 | 53511726 | G | C | 103 | Aga | UTR5 | . | . | . | 8qB1.3 | rs13479764 | 0 | 1 |
| chr8 | 53513574 | C | A | 221 | Aga | exonic | missense SNV | Aga:NM_001005847:exon2:c.C160A:p.L54M,Aga:NM_001205054:exon2:c.C160A:p.L54M | . | 8qB1.3 | . | 0 | 1 |
| chr8 | 53515911 | G | T | 221 | Aga | intronic | . | . | . | 8qB1.3 | rs36852228 | 0 | 1 |
| chr8 | 53517704 | C | A | 204 | Aga | intronic | . | . | . | 8qB1.3 | rs265607984 | 0 | 1 |
| chr8 | 53517771 | C | T | 221 | Aga | exonic | synonymous SNV | Aga:NM_001005847:exon5:c.C525T:p.P175P,Aga:NM_001205054:exon5:c.C525T:p.P175P | . | 8qB1.3 | rs227874471 | 0 | 1 |
| chr8 | 53517823 | T | C | 221 | Aga | exonic | missense SNV | Aga:NM_001005847:exon5:c.T577C:p.S193P,Aga:NM_001205054:exon5:c.T577C:p.S193P | . | 8qB1.3 | rs217547607 | 0 | 1 |
| chr8 | 53517967 | C | T | 130 | Aga | intronic | . | . | . | 8qB1.3 | rs36819312 | 0 | 1 |
| chr8 | 53517999 | G | C | 117 | Aga | intronic | . | . | . | 8qB1.3 | rs218222058 | 0 | 1 |
| chr8 | 53518858 | A | G | 221 | Aga | intronic | . | . | . | 8qB1.3 | rs242867467 | 0 | 1 |
| chr8 | 53519046 | G | A | 222 | Aga | intronic | . | . | . | 8qB1.3 | rs256676815 | 0 | 1 |
| chr8 | 53519055 | C | G | 222 | Aga | intronic | . | . | . | 8qB1.3 | rs223727656 | 0 | 1 |
| chr8 | 53519122 | A | G | 123 | Aga | intronic | . | . | . | 8qB1.3 | rs242454343 | 0 | 1 |
| chr8 | 53520267 | C | T | 85 | Aga | exonic | synonymous SNV | Aga:NM_001005847:exon7:c.C729T:p.A243A,Aga:NM_001205054:exon7:c.C699T:p.A233A | . | 8qB1.3 | rs219691073 | 0 | 1 |
| chr8 | 53520373 | A | G | 187 | Aga | intronic | . | . | . | 8qB1.3 | rs36982833 | 0 | 1 |
| chr8 | 53520431 | T | C | 76 | Aga | intronic | . | . | . | 8qB1.3 | rs265905937 | 0 | 1 |
| chr8 | 53520432 | G | A | 76 | Aga | intronic | . | . | . | 8qB1.3 | rs233752479 | 0 | 1 |
| chr8 | 53523414 | C | G | 64 | Aga | UTR3 | . | . | . | 8qB1.3 | rs211789496 | 0 | 1 |
| chr8 | 53587213 | T | C | 221 | Neil3 | exonic | missense SNV | Neil3:NM_146208:exon10:c.A1753G:p.K585E | . | 8qB1.3 | rs36322259 | 0 | 1 |
| chr8 | 53587319 | G | A | 221 | Neil3 | exonic | synonymous SNV | Neil3:NM_146208:exon10:c.C1647T:p.D549D | . | 8qB1.3 | . | 0 | 1 |
| chr8 | 53587406 | C | T | 221 | Neil3 | intronic | . | . | . | 8qB1.3 | . | 0 | 1 |
| chr8 | 53589021 | A | G | 222 | Neil3 | exonic | synonymous SNV | Neil3:NM_146208:exon9:c.T1542C:p.R514R | . | 8qB1.3 | rs36664547 | 0 | 1 |
| chr8 | 53589035 | T | C | 222 | Neil3 | exonic | missense SNV | Neil3:NM_146208:exon9:c.A1528G:p.M510V | . | 8qB1.3 | . | 0 | 1 |
| chr8 | 53589134 | G | A | 221 | Neil3 | intronic | . | . | . | 8qB1.3 | . | 0 | 1 |
| chr8 | 53599620 | C | T | 130 | Neil3 | intronic | . | . | . | 8qB1.3 | rs37394893 | 0 | 1 |
| chr8 | 53601019 | A | G | 222 | Neil3 | exonic | missense SNV | Neil3:NM_146208:exon7:c.T974C:p.V325A | . | 8qB1.3 | rs37082574 | 0 | 1 |
| chr8 | 53605607 | A | G | 221 | Neil3 | intronic | . | . | . | 8qB1.3 | rs36670811 | 0 | 1 |
| chr8 | 53607375 | A | G | 222 | Neil3 | exonic | missense SNV | Neil3:NM_146208:exon5:c.T658C:p.C220R | . | 8qB1.3 | rs37214799 | 0 | 1 |
| chr8 | 53609554 | C | T | 222 | Neil3 | intronic | . | . | . | 8qB1.3 | rs252411658 | 0 | 1 |
| chr8 | 53623603 | A | G | 112 | Neil3 | intronic | . | . | . | 8qB1.3 | . | 0 | 1 |
| chr8 | 53623630 | G | T | 206 | Neil3 | exonic | missense SNV | Neil3:NM_146208:exon2:c.C269A:p.P90H | . | 8qB1.3 | rs242105085 | 0 | 1 |
| chr8 | 53623859 | G | A | 117 | Neil3 | intronic | . | . | . | 8qB1.3 | . | 0 | 1 |
| chr8 | 53638775 | A | G | 193 | Neil3 | exonic | missense SNV | Neil3:NM_146208:exon1:c.T137C:p.L46P | . | 8qB1.3 | rs37270056 | 0 | 1 |
| chr8 | 53638926 | C | G | 147 | Neil3 | UTR5 | . | . | . | 8qB1.3 | . | 0 | 1 |
| chr8 | 53911964 | A | C | 150 | . | intergenic | . | . | . | 8qB1.3 | rs37746374 | 0 | 1 |
| chr8 | 54157216 | C | T | 220 | Vegfc | intronic | . | . | . | 8qB1.3 | rs3661488 | 0 | 1 |
| chr8 | 54181042 | T | C | 222 | Vegfc | intronic | . | . | . | 8qB1.3 | rs36530873 | 0 | 1 |
| chr8 | 54185792 | T | G | 130 | Vegfc | intronic | . | . | . | 8qB1.3 | rs3693833 | 0 | 1 |
| chr8 | 54185890 | T | C | 221 | Vegfc | intronic | . | . | . | 8qB1.3 | rs37634675 | 0 | 1 |
| chr8 | 54186091 | C | T | 220 | Vegfc | UTR3 | . | . | . | 8qB1.3 | rs37399533 | 0 | 1 |
| chr8 | 54580806 | T | C | 222 | Asb5 | intronic | . | . | . | 8qB1.3 | rs33341763 | 0 | 1 |
| chr8 | 54583395 | T | G | 120 | Asb5 | intronic | . | . | . | 8qB1.3 | rs32662078 | 0 | 1 |
| chr8 | 54584823 | G | A | 221 | Asb5 | intronic | . | . | . | 8qB1.3 | rs31328837 | 0 | 1 |
| chr8 | 54600657 | C | T | 89 | . | upstream | . | . | . | 8qB1.3 | rs32962102 | 0 | 1 |
| chr8 | 54600890 | C | T | 222 | Spata4 | exonic | synonymous SNV | Spata4:NM_133711:exon1:c.C90T:p.P30P | . | 8qB1.3 | rs13479769 | 0 | 1 |
| chr8 | 54632288 | C | G | 221 | Wdr17 | intronic | . | . | . | 8qB1.3 | rs32709547 | 0 | 1 |
| chr8 | 54635337 | A | C | 58 | Wdr17 | intronic | . | . | . | 8qB1.3 | . | 0 | 1 |
| chr8 | 54635510 | T | A | 221 | Wdr17 | exonic | synonymous SNV | Wdr17:NM_001172152:exon28:c.A3504T:p.P1168P,Wdr17:NM_028220:exon29:c.A3525T:p.P1175P | . | 8qB1.3 | . | 0 | 1 |
| chr8 | 54651395 | G | T | 221 | Wdr17 | intronic | . | . | . | 8qB1.3 | rs31344471 | 0 | 1 |
| chr8 | 54660620 | T | C | 122 | Wdr17 | intronic | . | . | . | 8qB1.3 | rs31341737 | 0 | 1 |
| chr8 | 54661249 | C | T | 222 | Wdr17 | exonic | synonymous SNV | Wdr17:NM_001172152:exon15:c.G2145A:p.A715A,Wdr17:NM_028220:exon16:c.G2166A:p.A722A | . | 8qB1.3 | rs31343385 | 0 | 1 |
| chr8 | 54663114 | A | G | 221 | Wdr17 | intronic | . | . | . | 8qB1.3 | rs31346477 | 0 | 1 |
| chr8 | 54665096 | T | C | 221 | Wdr17 | exonic | missense SNV | Wdr17:NM_001172152:exon12:c.A1564G:p.I522V,Wdr17:NM_028220:exon13:c.A1585G:p.I529V | . | 8qB1.3 | rs31343316 | 0 | 1 |
| chr8 | 54672599 | G | A | 220 | Wdr17 | intronic | . | . | . | 8qB1.3 | rs31344763 | 0 | 1 |
| chr8 | 54681346 | C | T | 158 | Wdr17 | exonic | synonymous SNV | Wdr17:NM_001172152:exon7:c.G1098A:p.L366L,Wdr17:NM_028220:exon8:c.G1119A:p.L373L | . | 8qB1.3 | rs31347366 | 0 | 1 |
| chr8 | 54681691 | G | A | 106 | Wdr17 | intronic | . | . | . | 8qB1.3 | rs31347372 | 0 | 1 |
| chr8 | 54687344 | T | A | 65 | Wdr17 | intronic | . | . | . | 8qB1.3 | rs31347023 | 0 | 1 |
| chr8 | 54696476 | C | T | 222 | Wdr17 | intronic | . | . | . | 8qB1.3 | rs31350758 | 0 | 1 |
| chr8 | 55037611 | G | A | 173 | Gpm6a | intronic | . | . | . | 8qB1.3 | rs45820248 | 0 | 1 |
| chr8 | 55049966 | C | T | 81 | Gpm6a | intronic | . | . | . | 8qB1.3 | rs49398955 | 0 | 1 |
| chr8 | 55049990 | G | A | 116 | Gpm6a | intronic | . | . | . | 8qB1.3 | rs45948166 | 0 | 1 |
| chr8 | 55050035 | A | G | 187 | Gpm6a | intronic | . | . | . | 8qB1.3 | rs51697963 | 0 | 1 |
| chr8 | 55054858 | C | T | 220 | Gpm6a | intronic | . | . | . | 8qB1.3 | rs46931341 | 0 | 1 |
| chr8 | 55871460 | T | C | 222 | Adam29 | exonic | missense SNV | Adam29:NM_175939:exon2:c.A1958G:p.H653R | . | 8qB2 | rs36898217 | 0 | 1 |
| chr8 | 55871682 | A | T | 221 | Adam29 | exonic | missense SNV | Adam29:NM_175939:exon2:c.T1736A:p.F579Y | . | 8qB2 | . | 0 | 1 |
| chr8 | 55872930 | C | G | 222 | Adam29 | exonic | missense SNV | Adam29:NM_175939:exon2:c.G488C:p.S163T | . | 8qB2 | . | 0 | 1 |
| chr8 | 55973629 | C | T | 116 | Glra3 | intronic | . | . | . | 8qB2 | . | 0 | 1 |
| chr8 | 55973703 | G | A | 222 | Glra3 | intronic | . | . | . | 8qB2 | rs49827293 | 0 | 1 |

| | | | | | | | | | | | | | |
|---|---|---|---|---|---|---|---|---|---|---|---|---|---|
| chr8 | 55973726 | T | C | 222 | Glra3 | intronic | . | . | . | 8qB2 | rs37434436 | 0 | 1 |
| chr8 | 55973883 | T | C | 164 | Glra3 | intronic | . | . | . | 8qB2 | rs264698766 | 0 | 1 |
| chr8 | 55973898 | G | C | 130 | Glra3 | intronic | . | . | . | 8qB2 | rs37192627 | 0 | 1 |
| chr8 | 55973904 | T | C | 84 | Glra3 | intronic | . | . | . | 8qB2 | rs250285307 | 0 | 1 |
| chr8 | 55973906 | G | A | 121 | Glra3 | intronic | . | . | . | 8qB2 | . | 0 | 1 |
| chr8 | 55974010 | G | A | 222 | Glra3 | intronic | . | . | . | 8qB2 | . | 0 | 1 |
| chr8 | 55974061 | G | A | 222 | Glra3 | intronic | . | . | . | 8qB2 | rs239233852 | 0 | 1 |
| chr8 | 55974081 | T | G | 184 | Glra3 | intronic | . | . | . | 8qB2 | . | 0 | 1 |
| chr8 | 55990942 | A | C | 85 | Glra3 | intronic | . | . | . | 8qB2 | . | 0 | 1 |
| chr8 | 55991016 | A | G | 96 | Glra3 | intronic | . | . | . | 8qB2 | rs230452724 | 0 | 1 |
| chr8 | 55991034 | T | G | 58 | Glra3 | intronic | . | . | . | 8qB2 | rs256643978 | 0 | 1 |
| chr8 | 55991071 | T | G | 25.3307 | Glra3 | intronic | . | . | . | 8qB2 | . | 0 | 1 |
| chr8 | 55991072 | T | G | 35.6381 | Glra3 | intronic | . | . | . | 8qB2 | . | 0 | 1 |
| chr8 | 55991086 | C | T | 69 | Glra3 | intronic | . | . | . | 8qB2 | . | 0 | 1 |
| chr8 | 55991174 | A | T | 222 | Glra3 | exonic | synonymous SNV | Glra3:NM_080438:exon2:c.A168T:p.P56P | . | 8qB2 | . | 0 | 1 |
| chr8 | 55991195 | G | A | 222 | Glra3 | exonic | synonymous SNV | Glra3:NM_080438:exon2:c.G189A:p.L63L | . | 8qB2 | . | 0 | 1 |
| chr8 | 55991302 | A | G | 222 | Glra3 | intronic | . | . | . | 8qB2 | . | 0 | 1 |
| chr8 | 55991410 | G | A | 125 | Glra3 | intronic | . | . | . | 8qB2 | . | 0 | 1 |
| chr8 | 55991440 | T | A | 114 | Glra3 | intronic | . | . | . | 8qB2 | rs259072718 | 0 | 1 |
| chr8 | 56085043 | A | G | 168 | Glra3 | intronic | . | . | . | 8qB2 | rs226403854 | 0 | 1 |
| chr8 | 56088835 | A | G | 85 | Glra3 | intronic | . | . | . | 8qB2 | rs243545483 | 0 | 1 |
| chr8 | 56089250 | C | T | 221 | Glra3 | intronic | . | . | . | 8qB2 | . | 0 | 1 |
| chr8 | 56089360 | G | C | 46.7978 | Glra3 | intronic | . | . | . | 8qB2 | rs33326243 | 0 | 1 |
| chr8 | 56298210 | T | C | 61 | Hpgd | intronic | . | . | . | 8qB2 | rs36521301 | 0 | 1 |
| chr8 | 56298238 | T | C | 103 | Hpgd | intronic | . | . | . | 8qB2 | rs37327817 | 0 | 1 |
| chr8 | 56298315 | T | A | 124 | Hpgd | intronic | . | . | . | 8qB2 | rs49156741 | 0 | 1 |
| chr8 | 56298323 | C | T | 126 | Hpgd | intronic | . | . | . | 8qB2 | rs36927747 | 0 | 1 |
| chr8 | 56298411 | T | C | 222 | Hpgd | exonic | synonymous SNV | Hpgd:NM_008278:exon3:c.T273C:p.N91N | . | 8qB2 | rs38986052 | 0 | 1 |
| chr8 | 56298585 | T | C | 129 | Hpgd | intronic | . | . | . | 8qB2 | rs36548193 | 0 | 1 |
| chr8 | 56307370 | G | C | 72 | Hpgd | intronic | . | . | . | 8qB2 | rs32554294 | 0 | 1 |
| chr8 | 56307529 | G | A | 222 | Hpgd | intronic | . | . | . | 8qB2 | rs33554818 | 0 | 1 |
| chr8 | 56318886 | A | G | 228 | Hpgd | intronic | . | . | . | 8qB2 | rs32785829 | 1 | 0 |
| chr8 | 56319266 | A | C | 167 | Hpgd | intronic | . | . | . | 8qB2 | rs33224062 | 0 | 1 |
| chr8 | 56453831 | T | C | 148 | . | intergenic | . | . | . | 8qB2 | rs33236211 | 1 | 0 |
| chr8 | 56453991 | C | A | 222 | . | intergenic | . | . | . | 8qB2 | . | 0 | 1 |
| chr8 | 56454011 | T | C | 192 | . | intergenic | . | . | . | 8qB2 | . | 0 | 1 |
| chr8 | 56454044 | T | C | 222 | . | intergenic | . | . | . | 8qB2 | . | 0 | 1 |
| chr8 | 56454120 | G | A | 222 | . | intergenic | . | . | . | 8qB2 | . | 0 | 1 |
| chr8 | 56539940 | A | G | 222 | Cep44 | intronic | . | . | . | 8qB2 | . | 0 | 1 |
| chr8 | 56540804 | T | G | 132 | Cep44 | intronic | . | . | . | 8qB2 | rs37659878 | 0 | 1 |
| chr8 | 56543174 | A | G | 83 | Cep44 | intronic | . | . | . | 8qB2 | rs37486842 | 0 | 1 |
| chr8 | 56551106 | G | A | 97 | . | upstream | . | . | 712 | 8qB2 | rs33032281 | 0 | 1 |
| chr8 | 56561363 | T | A | 164 | Fbxo8 | intronic | . | . | . | 8qB2 | rs33544295 | 0 | 1 |
| chr8 | 56564931 | T | C | 222 | Fbxo8 | intronic | . | . | . | 8qB2 | rs33025156 | 0 | 1 |
| chr8 | 56589865 | G | A | 65 | Fbxo8 | intronic | . | . | . | 8qB2 | . | 0 | 1 |
| chr8 | 56589915 | T | C | 114 | Fbxo8 | intronic | . | . | . | 8qB2 | . | 0 | 1 |
| chr8 | 56590052 | T | C | 222 | Fbxo8 | intronic | . | . | . | 8qB2 | rs32593722 | 0 | 1 |
| chr8 | 57291495 | C | T | 119 | . | intergenic | . | . | . | 8qB2 | rs33536854 | 0 | 1 |
| chr8 | 57291666 | C | A | 221 | . | intergenic | . | . | . | 8qB2 | rs13479778 | 0 | 1 |
| chr8 | 57291780 | A | G | 222 | . | intergenic | . | . | . | 8qB2 | rs33219483 | 0 | 1 |
| chr8 | 57292094 | C | T | 56 | . | intergenic | . | . | . | 8qB2 | rs32955406 | 0 | 1 |
| chr8 | 57322493 | A | G | 222 | Hand2 | intronic | . | . | . | 8qB2 | rs33464531 | 0 | 1 |
| chr8 | 57322512 | G | A | 222 | Hand2 | intronic | . | . | . | 8qB2 | rs32856251 | 0 | 1 |
| chr8 | 57482771 | A | C | 116 | Sap30 | UTR3 | . | . | . | 8qB2 | . | 0 | 1 |
| chr8 | 57545293 | C | G | 222 | Galnt7 | intronic | . | . | . | 8qB2 | . | 0 | 1 |
| chr8 | 57777177 | C | T | 222 | Galnt16 | UTR3 | . | . | . | 8qB2 | rs31410869 | 0 | 1 |
| chr8 | 57794760 | C | A | 130 | Galnt16 | intronic | . | . | . | 8qB2 | rs32906518 | 0 | 1 |
| chr8 | 57837250 | A | G | 218 | Galnt16 | exonic | synonymous SNV | Galnt16:NM_175032:exon9:c.T1308C:p.A436A | . | 8qB2 | rs52035673 | 0 | 1 |
| chr8 | 57857658 | G | A | 222 | Galnt16 | intronic | . | . | . | 8qB2 | rs31416557 | 0 | 1 |
| chr8 | 57884201 | G | T | 120 | Galnt16 | intronic | . | . | . | 8qB2 | rs33035629 | 0 | 1 |
| chr8 | 57958316 | C | G | 221 | Galnt16 | exonic | synonymous SNV | Galnt16:NM_175032:exon6:c.G879C:p.P293P | . | 8qB2 | rs48572859 | 0 | 1 |
| chr8 | 60545252 | A | G | 31.4777 | Aadat | exonic | synonymous SNV | Aadat:NM_011834:exon13:c.A1266G:p.K422K | . | 8qB3.1 | . | 0 | 1 |
| chr8 | 61963603 | T | C | 222 | Ddx60 | exonic | synonymous SNV | Ddx60:NM_001293783:exon13:c.T1581C:p.H527H | . | 8qB3.1 | . | 0 | 1 |
| chr8 | 61974300 | G | T | 59 | Ddx60 | intronic | . | . | . | 8qB3.1 | . | 0 | 1 |
| chr8 | 62123026 | C | T | 222 | Anxa10 | exonic | missense SNV | Anxa10:NM_001136089:exon1:c.G3A:p.M1I,Anxa10:NM_011922:exon1:c.G3A:p.M1I | . | 8qB3.1 | . | 0 | 1 |
| chr8 | 63349076 | C | T | 222 | Spock3 | intronic | . | . | . | 8qB3.1 | . | 0 | 1 |
| chr8 | 64025331 | A | G | 143 | Tll1 | intronic | . | . | . | 8qB3.1 | . | 0 | 1 |
| chr8 | 66512390 | G | T | 222 | Tktl2 | exonic | missense SNV | Tktl2:NM_001271574:exon1:c.G599T:p.C200F,Tktl2:NM_028927:exon1:c.G599T:p.C200F | . | 8qB3.1 | . | 0 | 1 |
| chr8 | 67514913 | T | C | 221 | . | intergenic | . | . | . | 8qB3.2 | . | 0 | 1 |
| chr8 | 67547582 | G | A | 221 | Nat3 | exonic | missense SNV | Nat3:NM_008674:exon3:c.G112A:p.E38K | . | 8qB3.2 | . | 0 | 1 |
| chr8 | 69882179 | C | G | 52 | Cilp2 | exonic | missense SNV | Cilp2:NM_026818:exon8:c.G2168C:p.G723A | 2437 | 8qB3.3 | . | 0 | 1 |
| chr8 | 70100227 | A | T | 122 | Ncan | intronic | . | . | . | 8qB3.3 | . | 0 | 1 |
| chr8 | 70110099 | T | A | 222 | Ncan | exonic | missense SNV | Ncan:NM_007789:exon7:c.A1508T:p.D503V | . | 8qB3.3 | . | 0 | 1 |
| chr8 | 70186581 | C | T | 222 | Slc25a42 | exonic | missense SNV | Slc25a42:NM_001007570:exon8:c.G845A:p.R282Q | . | 8qB3.3 | . | 0 | 1 |
| chr8 | 70599835 | A | C | 228 | Ssbp4 | intronic | . | . | . | 8qB3.3 | rs32752001 | 1 | 0 |
| chr8 | 70749282 | C | G | 222 | Pde4c | exonic | synonymous SNV | Pde4c:NM_001310465:exon16:c.C1671G:p.L557L | . | 8qB3.3 | . | 0 | 1 |
| chr8 | 71378465 | C | T | 189 | Nr2f6 | exonic | synonymous SNV | Nr2f6:NM_010150:exon2:c.G300A:p.Q100Q | . | 8qB3.3 | . | 0 | 1 |
| chr8 | 71654997 | C | A | 222 | Unc13a | exonic | synonymous SNV | Unc13a:NM_001029873:exon15:c.G1650T:p.L550L | 356 | 8qB3.3 | . | 0 | 1 |
| chr8 | 71668592 | G | A | 115 | Unc13a | intronic | . | . | . | 8qB3.3 | . | 0 | 1 |
| chr8 | 72653542 | T | G | 222 | Nwd1 | exonic | missense SNV | Nwd1:NM_176940:exon2:c.T55G:p.F19V | . | 8qB3.3 | . | 0 | 1 |
| chr8 | 76908846 | C | A | 221 | Nr3c2 | exonic | missense SNV | Nr3c2:NM_001083906:exon2:c.C575A:p.P192H | . | 8qC1 | . | 0 | 1 |
| chr8 | 79489995 | C | T | 222 | . | intergenic | . | . | . | 8qC1 | . | 0 | 1 |
| chr8 | 79658905 | A | T | 222 | Otud4 | splicing | . | . | . | 8qC1 | . | 0 | 1 |
| chr8 | 79699210 | A | G | 119 | Abce1 | intronic | . | . | . | 8qC1 | rs33895928 | 0 | 1 |
| chr8 | 80615569 | A | G | 221 | Frem3 | exonic | missense SNV | Frem3:NM_001167898:exon1:c.A4490G:p.K1497R | . | 8qC2 | . | 0 | 1 |
| chr8 | 80774956 | T | C | 221 | Gab1 | exonic | missense SNV | Gab1:NM_021356:exon7:c.A1660G:p.I554V,Gab1:NM_001301298:exon8:c.A1750G:p.I584V | . | 8qC2 | . | 0 | 1 |
| chr8 | 82042038 | T | A | 221 | Inpp4b | intronic | . | . | . | 8qC2 | . | 0 | 1 |
| chr8 | 83295202 | T | G | 199 | Ucp1 | exonic | missense SNV | Ucp1:NM_009463:exon5:c.T662G:p.V221G | . | 8qC2 | . | 0 | 1 |
| chr8 | 83594440 | G | T | 155 | Tecr | UTR5 | . | . | 353 | 8qC2 | . | 0 | 1 |
| chr8 | 83926223 | T | C | 221 | Adgrl1 | intronic | . | . | . | 8qC2 | . | 0 | 1 |
| chr8 | 84691024 | G | T | 107 | Trmt1 | intronic | . | . | . | 8qC2 | . | 0 | 1 |
| chr8 | 84845941 | C | A | 221 | Calr | exonic | synonymous SNV | Calr:NM_007591:exon3:c.G318T:p.G106G | . | 8qC2 | . | 0 | 1 |
| chr8 | 84889511 | A | C | 222 | Gcdh | intronic | . | . | . | 8qC3 | . | 0 | 1 |
| chr8 | 84921052 | T | A | 221 | Mast1,Gm38426 | ncRNA_intronic | . | . | . | 8qC3 | . | 0 | 1 |
| chr8 | 84998286 | G | A | 215 | Hook2 | exonic | missense SNV | Hook2:NM_001167991:exon16:c.G1594A:p.E532K,Hook2:NM_133255:exon16:c.G1594A:p.E532K | . | 8qC3 | . | 0 | 1 |
| chr8 | 85275019 | G | A | 222 | Vps35 | exonic | missense SNV | Vps35:NM_022997:exon11:c.C1169T:p.T390I | . | 8qC3 | . | 0 | 1 |
| chr8 | 85365179 | A | T | 208 | Mylk3 | UTR5 | . | . | . | 8qC3 | rs245783224 | 0 | 1 |
| chr8 | 86019234 | T | A | 51 | Phkb | intronic | . | . | . | 8qC3 | . | 0 | 1 |
| chr8 | 88343714 | C | T | 153 | Brd7 | intronic | . | . | . | 8qC3 | rs219768878 | 0 | 1 |
| chr8 | 88681536 | T | A | 171 | Nod2 | intronic | . | . | . | 8qC3 | . | 0 | 1 |
| chr8 | 88709966 | A | C | 222 | Cyld | exonic | missense SNV | Cyld:NM_001128170:exon4:c.A883C:p.I295L,Cyld:NM_173369:exon4:c.A883C:p.I295L,Cyld:NM_00112817 | . | 8qC3 | . | 0 | 1 |
| chr8 | 89031071 | G | A | 222 | Sall1 | exonic | missense SNV | Sall1:NM_021390:exon3:c.C2404T:p.P802S | . | 8qC3 | . | 0 | 1 |
| chr8 | 89031105 | G | C | 222 | Sall1 | exonic | synonymous SNV | Sall1:NM_021390:exon3:c.C2370G:p.G790G | . | 8qC3 | . | 0 | 1 |
| chr8 | 90248372 | G | A | 222 | Tox3 | exonic | missense SNV | Tox3:NM_172913:exon7:c.C1630T:p.P544S | . | 8qC4 | . | 0 | 1 |
| chr8 | 91008589 | C | T | 98 | Chd9 | intronic | . | . | . | 8qC4 | rs256119857 | 0 | 1 |
| chr8 | 91034035 | A | T | 200 | Chd9 | exonic | missense SNV | Chd9:NM_001310530:exon30:c.A6407T:p.D2136V,Chd9:NM_177224:exon32:c.A6407T:p.D2136V | . | 8qC4 | . | 0 | 1 |
| chr8 | 92677394 | C | T | 164 | Irx6 | exonic | synonymous SNV | Irx6:NM_001301137:exon4:c.C588T:p.N196N,Irx6:NM_022428:exon4:c.C588T:p.N196N | . | 8qC5 | . | 0 | 1 |
| chr8 | 92873049 | T | C | 201 | Lpcat2 | exonic | missense SNV | Lpcat2:NM_173014:exon5:c.T644C:p.I215T | . | 8qC5 | . | 0 | 1 |
| chr8 | 92889548 | A | T | 20.3623 | Lpcat2 | intronic | . | . | . | 8qC5 | . | 0 | 1 |
| chr8 | 92918046 | A | G | 221 | Lpcat2 | exonic | missense SNV | Lpcat2:NM_173014:exon14:c.A1462G:p.S488G | . | 8qC5 | . | 0 | 1 |
| chr8 | 92961341 | G | C | 213 | Slc6a2 | exonic | missense SNV | Slc6a2:NM_009209:exon1:c.G94C:p.D32H | 1007 | 8qC5 | . | 0 | 1 |
| chr8 | 93057150 | T | G | 166 | Ces1b | intronic | . | . | . | 8qC5 | . | 0 | 1 |
| chr8 | 93067268 | C | T | 87 | Ces1b | intronic | . | . | . | 8qC5 | . | 0 | 1 |
| chr8 | 93203410 | G | A | 222 | Ces1e | intronic | . | . | . | 8qC5 | . | 0 | 1 |
| chr8 | 93256860 | T | G | 222 | Ces1f | splicing | . | . | . | 8qC5 | . | 0 | 1 |
| chr8 | 93268249 | A | G | 54 | Ces1f | intronic | . | . | . | 8qC5 | . | 0 | 1 |
| chr8 | 93336911 | G | C | 122 | Ces1g | intronic | . | . | . | 8qC5 | . | 0 | 1 |
| chr8 | 93528429 | A | C | 168 | Ces5a | intronic | . | . | . | 8qC5 | . | 0 | 1 |
| chr8 | 94138928 | G | A | 57 | Mt4 | UTR3 | . | . | . | 8qC5 | . | 0 | 1 |
| chr8 | 94392420 | G | C | 221 | Herpud1 | exonic | missense SNV | Herpud1:NM_022331:exon6:c.G801C:p.W267C | . | 8qC5 | . | 0 | 1 |
| chr8 | 94476356 | A | C | 176 | Nlrc5 | exonic | missense SNV | Nlrc5:NM_001033207:exon4:c.A1083C:p.E361D | . | 8qC5 | . | 0 | 1 |
| chr8 | 94937693 | C | A | 221 | Adgrg5 | exonic | stopgain | Adgrg5:NM_001033468:exon9:c.C927A:p.C309X,Adgrg5:NM_001145972:exon9:c.C930A:p.C310X | . | 8qC5 | . | 0 | 1 |
| chr8 | 95103553 | C | G | 21.464 | Kifc3 | intronic | . | . | . | 8qC5 | . | 0 | 1 |
| chr8 | 95733269 | A | T | 221 | Cnot1 | exonic | synonymous SNV | Cnot1:NM_001205226:exon39:c.T5448A:p.R1816R,Cnot1:NM_153164:exon39:c.T5454A:p.R1818R,Cnot1:NM | . | 8qD1 | . | 0 | 1 |
| chr8 | 99111836 | T | A | 222 | Cdh8 | intronic | . | . | . | 8qD1 | . | 0 | 1 |
| chr8 | 99190450 | G | T | 222 | Cdh8 | exonic | missense SNV | Cdh8:NM_001039154:exon7:c.C1032A:p.D344E,Cdh8:NM_001285913:exon7:c.C1032A:p.D344E,Cdh8:NM_001 | . | 8qD1 | . | 0 | 1 |
| chr8 | 100031075 | G | T | 221 | . | intergenic | . | . | . | 8qD1 | . | 0 | 1 |
| chr8 | 102865286 | C | T | 222 | . | intergenic | . | . | . | 8qD1 | . | 0 | 1 |
| chr8 | 104355354 | C | A | 99 | Cmtm4 | intronic | . | . | . | 8qD3 | rs33441846 | 0 | 1 |
| chr8 | 104952443 | C | A | 123 | Ces2f | intronic | . | . | . | 8qD3 | . | 0 | 1 |
| chr8 | 105285319 | C | T | 221 | 4931428F04Rik | exonic | synonymous SNV | 4931428F04Rik:NM_001166394:exon2:c.G495A:p.Q165Q,4931428F04Rik:NM_028888:exon3:c.G495A:p.Q165 | . | 8qD3 | . | 0 | 1 |
| chr8 | 105378375 | A | C | 222 | Plekhg4 | exonic | missense SNV | Plekhg4:NM_001081333:exon11:c.A1561C:p.K521Q | . | 8qD3 | . | 0 | 1 |
| chr8 | 105378377 | G | T | 222 | Plekhg4 | exonic | missense SNV | Plekhg4:NM_001081333:exon11:c.G1563T:p.K521N | . | 8qD3 | . | 0 | 1 |
| chr8 | 105468175 | G | A | 98 | Tppp3 | exonic | missense SNV | Tppp3:NM_026481:exon3:c.C323T:p.P108L | . | 8qD3 | . | 0 | 1 |
| chr8 | 105695768 | G | C | 221 | Rltpr | exonic | missense SNV | Rltpr:NM_001033320:exon12:c.G1689C:p.R563S | . | 8qD3 | . | 0 | 1 |
| chr8 | 105708313 | C | T | 173 | 4933405L10Rik | UTR5 | . | . | . | 8qD3 | . | 0 | 1 |
| chr8 | 105709600 | T | A | 222 | 4933405L10Rik | intronic | . | . | . | 8qD3 | . | 0 | 1 |
| chr8 | 105786594 | T | G | 221 | Ranbp10 | intronic | . | . | . | 8qD3 | . | 0 | 1 |
| chr8 | 106163198 | C | T | 222 | Pla2g15 | exonic | synonymous SNV | Pla2g15:NM_133792:exon6:c.C1101T:p.S367S | 243 | 8qD3 | . | 0 | 1 |
| chr8 | 106257610 | C | T | 221 | Smpd3 | exonic | missense SNV | Smpd3:NM_021491:exon6:c.G1615A:p.G539R | . | 8qD3 | . | 0 | 1 |
| chr8 | 106299088 | C | A | 158 | 4930506A18Rik,Smr | ncRNA_intronic | . | . | . | 8qD3 | . | 0 | 1 |
| chr8 | 106544378 | T | A | 151 | Cdh3 | intronic | . | . | . | 8qD3 | . | 0 | 1 |
| chr8 | 106900964 | G | T | 221 | Cirh1a | intronic | . | . | . | 8qD3 | . | 0 | 1 |
| chr8 | 107048352 | G | T | 97 | Pdf | exonic | missense SNV | Pdf:NM_026513:exon1:c.C247A:p.L83M | 763 | 8qD3 | . | 0 | 1 |
| chr8 | 109557058 | C | T | 73 | Dhx38 | intronic | . | . | . | 8qD3 | . | 0 | 1 |
| chr8 | 109954493 | T | C | 221 | Marveld3 | exonic | missense SNV | Marveld3:NM_028584:exon3:c.A769G:p.T257A | . | 8qD3 | . | 0 | 1 |
| chr8 | 110312350 | C | A | 222 | Hydin | intronic | . | . | . | 8qD3 | . | 0 | 1 |
| chr8 | 110572020 | A | G | 144 | Hydin | intronic | . | . | . | 8qD3 | . | 0 | 1 |
| chr8 | 110837384 | G | T | 221 | Sf3b3 | exonic | synonymous SNV | Sf3b3:NM_133953:exon8:c.C975A:p.I325I | . | 8qE1 | . | 0 | 1 |
| chr8 | 110886737 | C | A | 222 | Fuk | intronic | . | . | . | 8qE1 | . | 0 | 1 |
| chr8 | 111040743 | G | A | 195 | Aars | splicing | . | . | . | 8qE1 | . | 0 | 1 |
| chr8 | 111069711 | C | A | 100 | Clec18a | UTR3 | . | . | . | 8qE1 | . | 0 | 1 |
| chr8 | 112236604 | C | G | 222 | . | intergenic | . | . | . | 8qE1 | . | 0 | 1 |
| chr8 | 112815357 | T | A | 92 | Cntnap4 | intronic | . | . | . | 8qE1 | . | 0 | 1 |
| chr8 | 113652549 | G | A | 221 | Sycel1 | exonic | missense SNV | Sycel1:NM_001286494:exon4:c.G245A:p.R82H,Sycel1:NM_001048145:exon5:c.G308A:p.R103H,Sycel1:NM | . | 8qE1 | . | 0 | 1 |
| chr8 | 113845250 | G | A | 222 | Adamts18 | intronic | . | . | . | 8qE1 | rs236380914 | 0 | 1 |
| chr8 | 116997711 | A | T | 222 | Pkd1l2 | intronic | . | . | . | 8qE1 | . | 0 | 1 |
| chr8 | 117113432 | T | C | 228 | Bco1 | exonic | synonymous SNV | Bco1:NM_001163028:exon6:c.T688C:p.L230L,Bco1:NM_021486:exon6:c.T688C:p.L230L | . | 8qE1 | rs13480010 | 1 | 0 |
| chr8 | 117573834 | G | A | 222 | Plcg2 | intronic | . | . | . | 8qE1 | . | 0 | 1 |
| chr8 | 119345511 | G | C | 141 | Hsbp1 | intronic | . | . | . | 8qE1 | . | 0 | 1 |
| chr8 | 119596754 | A | G | 221 | Dnaaf1 | exonic | missense SNV | Dnaaf1:NM_026648:exon11:c.A1481G:p.E494G | . | 8qE1 | . | 0 | 1 |
| chr8 | 119956111 | T | G | 74 | Usp10 | intronic | . | . | . | 8qE1 | . | 0 | 1 |
| chr8 | 120145573 | C | A | 222 | 6430548M08Rik | exonic | missense SNV | 6430548M08Rik:NM_001163760:exon2:c.C239A:p.S80Y,6430548M08Rik:NM_001163761:exon2:c.C239A:p.S8 | . | 8qE1 | . | 0 | 1 |
| chr8 | 120727338 | C | T | 80 | . | intergenic | . | . | . | 8qE1 | . | 0 | 1 |
| chr8 | 121785399 | G | A | 140 | Jph3 | exonic | missense SNV | Jph3:NM_020605:exon4:c.G2125A:p.E709K | . | 8qE1 | . | 0 | 1 |
| chr8 | 122444029 | G | T | 52 | 9330133O14Rik | ncRNA_exonic | . | . | . | 8qE1 | . | 0 | 1 |
| chr8 | 123268007 | G | A | 222 | Zfp276 | exonic | missense SNV | Zfp276:NM_020497:exon11:c.G1812A:p.M604I | . | 8qE1 | . | 0 | 1 |
| chr8 | 123459316 | G | T | 102 | Def8 | intronic | . | . | . | 8qE1 | . | 0 | 1 |
| chr8 | 123806005 | A | G | 228 | Rab4a | UTR5 | . | . | 307 | 8qE2 | rs222410757 | 1 | 0 |
| chr8 | 124614802 | T | G | 117 | Capn9 | intronic | . | . | . | 8qE2 | rs49707042 | 1 | 0 |
| chr8 | 124823163 | C | G | 194 | Trim67 | exonic | missense SNV | Trim67:NM_198632:exon6:c.C1531G:p.R511G | . | 8qE2 | . | 0 | 1 |
| chr8 | 124903341 | A | T | 222 | Sprtn | exonic | missense SNV | Sprtn:NM_001111141:exon5:c.A1372T:p.S458C | . | 8qE2 | . | 0 | 1 |
| chr8 | 125924198 | G | T | 221 | BC021891 | exonic | missense SNV | BC021891:NM_145608:exon2:c.G938T:p.S313I | . | 8qE2 | . | 0 | 1 |
| chr8 | 125941891 | C | A | 221 | BC021891 | exonic | missense SNV | BC021891:NM_145608:exon9:c.C2215A:p.L739I | . | 8qE2 | . | 0 | 1 |
| chr8 | 127437018 | A | C | 221 | Pard3 | intronic | . | . | . | 8qE2 | . | 0 | 1 |
| chr8 | 128498470 | G | T | 221 | Nrp1 | intronic | . | . | . | 8qE2 | . | 0 | 1 |
| chr8 | 128988910 | T | C | 60 | Ccdc7a | intronic | . | . | . | 8qE2 | . | 0 | 1 |
| chr8 | 129027698 | A | G | 209 | Ccdc7a | intronic | . | . | . | 8qE2 | . | 0 | 1 |
| chr9 | 3865601 | T | C | 222 | Gucy1a2 | intronic | . | . | . | 9qA1 | . | 0 | 1 |
| chr9 | 5358185 | G | A | 116 | Casp12 | intronic | . | . | . | 9qA1 | . | 0 | 1 |
| chr9 | 6376707 | T | G | 76 | Pdgfd | intronic | . | . | . | 9qA1 | . | 0 | 1 |
| chr9 | 7124534 | T | G | 63 | Dync2h1 | intronic | . | . | . | 9qA1 | . | 0 | 1 |
| chr9 | 7447068 | T | C | 221 | Mmp3 | exonic | missense SNV | Mmp3:NM_010809:exon2:c.T247C:p.S83P | . | 9qA1 | . | 0 | 1 |
| chr9 | 7752205 | T | A | 148 | . | intergenic | . | . | . | 9qA1 | . | 0 | 1 |
| chr9 | 7752211 | A | T | 149 | . | intergenic | . | . | . | 9qA1 | . | 0 | 1 |

| | | | | | | | | | | | | |
|---|---|---|---|---|---|---|---|---|---|---|---|---|
| chr9 | 8246942 | G | T | 221 | . | downstream | . | . | . | 9qA1 | . | 0 | 1 |
| chr9 | 8678094 | G | A | 154 | Gm32014,Trpc6 | ncRNA_intronic | . | . | . | 9qA1 | . | 0 | 1 |
| chr9 | 12865424 | T | C | 158 | . | intergenic | . | . | . | 9qA1 | . | 0 | 1 |
| chr9 | 13789151 | G | T | 221 | Mtmr2 | splicing | . | . | . | 9qA1 | . | 0 | 1 |
| chr9 | 14357671 | G | A | 222 | Endod1 | exonic | stopgain | Endod1:NM_028013:exon2:c.C517T:p.R173X | . | 9qA1 | . | 0 | 1 |
| chr9 | 14380838 | C | T | 103 | Endod1 | exonic | missense SNV | Endod1:NM_028013:exon1:c.G133A:p.A45T | 534 | 9qA1 | . | 0 | 1 |
| chr9 | 14550694 | G | T | 98 | Amotl1 | intronic | . | . | . | 9qA2 | . | 0 | 1 |
| chr9 | 15084313 | G | T | 222 | Hephl1 | exonic | missense SNV | Hephl1:NM_001164797:exon8:c.C1418A:p.A473D | . | 9qA2 | . | 0 | 1 |
| chr9 | 15239256 | G | A | 107 | Vstm5 | UTR5 | . | . | . | 9qA2 | . | 0 | 1 |
| chr9 | 15914916 | G | T | 213 | Fat3 | UTR3 | . | . | . | 9qA2 | . | 0 | 1 |
| chr9 | 17030607 | G | A | 82 | . | intergenic | . | . | . | 9qA2 | rs52331843 | 0 | 1 |
| chr9 | 17030788 | C | T | 63 | . | intergenic | . | . | . | 9qA2 | rs108562337 | 0 | 1 |
| chr9 | 17030821 | C | T | 133 | . | intergenic | . | . | . | 9qA2 | rs51588497 | 0 | 1 |
| chr9 | 17031009 | A | C | 79 | . | intergenic | . | . | . | 9qA2 | . | 0 | 1 |
| chr9 | 17732696 | G | T | 192 | . | intergenic | . | . | . | 9qA2 | . | 0 | 1 |
| chr9 | 17732698 | G | T | 194 | . | intergenic | . | . | . | 9qA2 | . | 0 | 1 |
| chr9 | 17733643 | C | A | 222 | . | intergenic | . | . | . | 9qA2 | . | 0 | 1 |
| chr9 | 18755083 | A | C | 221 | Olfr24 | exonic | missense SNV | Olfr24:NM_146606:exon1:c.T551G:p.L184R | . | 9qA2 | . | 0 | 1 |
| chr9 | 18857703 | A | C | 37.7106 | . | downstream | . | . | . | 9qA2 | . | 0 | 1 |
| chr9 | 19360429 | A | C | 131 | Olfr846 | exonic | missense SNV | Olfr846:NM_146282:exon1:c.T925G:p.S309A | . | 9qA2 | . | 0 | 1 |
| chr9 | 19690463 | G | T | 49.2174 | . | intergenic | . | . | . | 9qA3 | . | 0 | 1 |
| chr9 | 19884124 | G | A | 221 | Olfr862 | exonic | synonymous SNV | Olfr862:NM_146562:exon1:c.C180T:p.Y60Y | . | 9qA3 | . | 0 | 1 |
| chr9 | 19928169 | A | T | 221 | . | intergenic | . | . | . | 9qA3 | . | 0 | 1 |
| chr9 | 20662145 | G | C | 126 | Pin1 | intronic | . | . | . | 9qA3 | . | 0 | 1 |
| chr9 | 20823368 | C | T | 221 | Rdh8 | exonic | synonymous SNV | Rdh8:NM_001030290:exon3:c.C330T:p.S110S | . | 9qA3 | rs219219688 | 0 | 1 |
| chr9 | 20878504 | G | C | 169 | Angptl6 | exonic | missense SNV | Angptl6:NM_145154:exon2:c.C91G:p.L31V | 572 | 9qA3 | . | 0 | 1 |
| chr9 | 21575504 | G | C | 221 | Carm1 | exonic | missense SNV | Carm1:NM_021531:exon4:c.G547C:p.D183H,Carm1:NM_153141:exon4:c.G547C:p.D183H | . | 9qA3 | . | 0 | 1 |
| chr9 | 21742818 | G | T | 221 | Ldlr | intronic | . | . | . | 9qA3 | . | 0 | 1 |
| chr9 | 22356102 | A | T | 221 | Mir680-2,Anln | exonic | synonymous SNV | Anln:NM_028390:exon15:c.T2544A:p.T848T | . | 9qA3 | . | 0 | 1 |
| chr9 | 24430684 | C | G | 43.9762 | Dpy19l1 | intronic | . | . | . | 9qA4 | . | 0 | 1 |
| chr9 | 24582663 | C | A | 123 | Dpy19l2 | intronic | . | . | . | 9qA4 | . | 0 | 1 |
| chr9 | 24658608 | C | T | 154 | Dpy19l2 | exonic | missense SNV | Dpy19l2:NM_001166207:exon9:c.G1048A:p.V350I | . | 9qA4 | . | 0 | 1 |
| chr9 | 25109401 | T | A | 222 | Herpud2 | intronic | . | . | . | 9qA4 | . | 0 | 1 |
| chr9 | 25264106 | G | C | 221 | 7-9月 | intronic | . | . | . | 9qA4 | . | 0 | 1 |
| chr9 | 26757867 | G | T | 90 | B3gat1 | intronic | . | . | . | 9qA4 | . | 0 | 1 |
| chr9 | 26767943 | A | G | 222 | Glb1l2 | intronic | . | . | . | 9qA4 | . | 0 | 1 |
| chr9 | 26771238 | A | G | 100 | Glb1l2 | intronic | . | . | . | 9qA4 | . | 0 | 1 |
| chr9 | 26827578 | C | A | 163 | Glb1l3 | intronic | . | . | . | 9qA4 | . | 0 | 1 |
| chr9 | 26859509 | C | A | 221 | Glb1l3 | exonic | missense SNV | Glb1l3:NM_001113323:exon3:c.G260T:p.R87L | . | 9qA4 | . | 0 | 1 |
| chr9 | 26896621 | G | T | 221 | Gm1110 | exonic | missense SNV | Gm1110:NM_001281475:exon10:c.C868A:p.H290N | . | 9qA4 | . | 0 | 1 |
| chr9 | 26897765 | C | T | 176 | Gm1110 | intronic | . | . | . | 9qA4 | . | 0 | 1 |
| chr9 | 30427644 | G | A | 222 | Snx19 | exonic | missense SNV | Snx19:NM_028874:exon1:c.G77A:p.S26N | . | 9qA4 | . | 0 | 1 |
| chr9 | 35646784 | A | T | 221 | Gm17365 | intronic | . | . | . | 9qA4 | . | 0 | 1 |
| chr9 | 35671020 | G | T | 222 | Pate2 | exonic | missense SNV | Pate2:NM_001033421:exon4:c.G293T:p.C98F | . | 9qA4 | . | 0 | 1 |
| chr9 | 35689747 | T | G | 83 | . | intergenic | . | . | . | 9qA4 | . | 0 | 1 |
| chr9 | 35742422 | A | T | 29.0705 | . | downstream | . | . | . | 9qA4 | . | 0 | 1 |
| chr9 | 36476830 | G | T | 110 | Gm9513 | intronic | . | . | . | 9qA4 | . | 0 | 1 |
| chr9 | 37239490 | C | A | 184 | Slc37a2 | intronic | . | . | . | 9qA4 | . | 0 | 1 |
| chr9 | 37418524 | T | G | 148 | Robo3 | exonic | missense SNV | Robo3:NM_001164767:exon23:c.A3495C:p.Q1165H | . | 9qA4 | . | 0 | 1 |
| chr9 | 37633560 | C | A | 221 | Siae | intronic | . | . | . | 9qA4 | . | 0 | 1 |
| chr9 | 37633565 | C | A | 213 | Siae | intronic | . | . | . | 9qA4 | . | 0 | 1 |
| chr9 | 37804879 | G | T | 221 | Olfr876 | UTR3 | . | . | . | 9qA4 | . | 0 | 1 |
| chr9 | 38062981 | T | G | 33.8515 | . | downstream | . | . | . | 9qA4 | . | 0 | 1 |
| chr9 | 38180110 | T | C | 210 | Olfr891 | exonic | missense SNV | Olfr891:NM_146478:exon1:c.A712G:p.S238G | . | 9qA5.1 | . | 0 | 1 |
| chr9 | 38185670 | A | G | 228 | . | intergenic | . | . | . | 9qA5.1 | rs51772077 | 1 | 0 |
| chr9 | 38189710 | G | T | 228 | . | intergenic | . | . | . | 9qA5.1 | rs230737141 | 1 | 0 |
| chr9 | 38349157 | G | T | 221 | Olfr898 | exonic | missense SNV | Olfr898:NM_146871:exon1:c.G73T:p.V25F | . | 9qA5.1 | . | 0 | 1 |
| chr9 | 38464308 | A | G | 178 | Olfr904 | exonic | missense SNV | Olfr904:NM_146801:exon1:c.A266G:p.K89R | . | 9qA5.1 | . | 0 | 1 |
| chr9 | 38581392 | T | C | 221 | Olfr912 | exonic | synonymous SNV | Olfr912:NM_146810:exon2:c.T114C:p.T38T | . | 9qA5.1 | . | 0 | 1 |
| chr9 | 38657421 | T | C | 75 | . | downstream | . | . | . | 9qA5.1 | . | 0 | 1 |
| chr9 | 38664840 | C | A | 77 | . | downstream | . | . | . | 9qA5.1 | . | 0 | 1 |
| chr9 | 38854980 | T | A | 73 | . | upstream | . | . | . | 9qA5.1 | . | 0 | 1 |
| chr9 | 39018503 | A | G | 64 | . | downstream | . | . | . | 9qA5.1 | . | 0 | 1 |
| chr9 | 39059862 | A | T | 221 | Olfr937 | exonic | missense SNV | Olfr937:NM_146439:exon1:c.T803A:p.M268K | . | 9qA5.1 | . | 0 | 1 |
| chr9 | 39237972 | A | C | 221 | Olfr1537 | exonic | missense SNV | Olfr1537:NM_207665:exon1:c.T460G:p.F154V | . | 9qA5.1 | . | 0 | 1 |
| chr9 | 39593850 | A | T | 222 | AW551984 | intronic | . | . | . | 9qA5.1 | . | 0 | 1 |
| chr9 | 39600486 | T | C | 222 | AW551984 | exonic | synonymous SNV | AW551984:NM_001199556:exon4:c.A228G:p.E76E,AW551984:NM_178737:exon4:c.A228G:p.E76E | . | 9qA5.1 | . | 0 | 1 |
| chr9 | 39613477 | G | C | 171 | . | upstream | . | . | . | 9qA5.1 | . | 0 | 1 |
| chr9 | 40040130 | G | T | 166 | . | intergenic | . | . | . | 9qA5.1 | . | 0 | 1 |
| chr9 | 42024607 | T | C | 59 | Sorl1 | intronic | . | . | . | 9qA5.1 | . | 0 | 1 |
| chr9 | 42046508 | C | G | 160 | Sorl1 | exonic | synonymous SNV | Sorl1:NM_011436:exon13:c.G1839C:p.L613L | . | 9qA5.1 | . | 0 | 1 |
| chr9 | 42966161 | C | A | 108 | Arhgef12 | UTR3 | . | . | . | 9qA5.1 | rs587139827 | 0 | 1 |
| chr9 | 43111672 | A | G | 222 | Tmem136 | exonic | missense SNV | Tmem136:NM_001034863:exon3:c.T386C:p.V129A | . | 9qA5.1 | . | 0 | 1 |
| chr9 | 45410253 | C | G | 222 | Fxyd2 | UTR3 | . | . | . | 9qA5.2 | . | 0 | 1 |
| chr9 | 45939179 | G | T | 222 | Sidt2 | UTR3 | . | . | . | 9qA5.2 | . | 0 | 1 |
| chr9 | 48450495 | C | T | 88 | . | intergenic | . | . | . | 9qA5.3 | . | 0 | 1 |
| chr9 | 48495371 | C | T | 85 | Gm5617 | UTR5 | . | . | 879 | 9qA5.3 | . | 0 | 1 |
| chr9 | 48831909 | A | G | 222 | Zbtb16 | exonic | missense SNV | Zbtb16:NM_001033324:exon2:c.T1102C:p.F368L | . | 9qA5.3 | . | 0 | 1 |
| chr9 | 48901124 | A | C | 222 | Htr3a | intronic | . | . | . | 9qA5.3 | . | 0 | 1 |
| chr9 | 48959333 | A | T | 164 | Htr3b | exonic | synonymous SNV | Htr3b:NM_020274:exon2:c.T69A:p.P23P | . | 9qA5.3 | . | 0 | 1 |
| chr9 | 49079161 | G | C | 146 | . | downstream | . | . | . | 9qA5.3 | . | 0 | 1 |
| chr9 | 49420552 | C | A | 222 | Ankk1 | intronic | . | . | . | 9qA5.3 | . | 0 | 1 |
| chr9 | 50455310 | C | T | 70 | . | intergenic | . | . | . | 9qA5.3 | . | 0 | 1 |
| chr9 | 50640183 | A | G | 228 | Dlat | intronic | . | . | . | 9qA5.3 | rs51381578 | 1 | 0 |
| chr9 | 53806743 | T | G | 222 | Slc35f2 | exonic | synonymous SNV | Slc35f2:NM_028060:exon4:c.T540G:p.G180G | . | 9qA5.3 | . | 0 | 1 |
| chr9 | 53934302 | T | C | 139 | Elmod1 | intronic | . | . | . | 9qA5.3 | . | 0 | 1 |
| chr9 | 54335841 | G | C | 22.5029 | Gldn | exonic | missense SNV | Gldn:NM_177350:exon9:c.G1106C:p.C369S | . | 9qA5.3 | . | 0 | 1 |
| chr9 | 54338471 | C | T | 222 | Gldn | exonic | synonymous SNV | Gldn:NM_177350:exon10:c.C1305T:p.Y435Y | . | 9qA5.3 | rs258769514 | 0 | 1 |
| chr9 | 54416444 | G | A | 222 | Dmxl2 | exonic | synonymous SNV | Dmxl2:NM_172771:exon18:c.C3655T:p.L1219L | . | 9qA5.3 | . | 0 | 1 |
| chr9 | 54461169 | G | T | 132 | Dmxl2 | intronic | . | . | . | 9qA5.3 | . | 0 | 1 |
| chr9 | 55214830 | G | T | 154 | Fbxo22 | intronic | . | . | . | 9qB | . | 0 | 1 |
| chr9 | 55259290 | G | T | 186 | Nrg4 | intronic | . | . | . | 9qB | . | 0 | 1 |
| chr9 | 55542034 | G | C | 90 | Isl2 | intronic | . | . | 1159 | 9qB | . | 0 | 1 |
| chr9 | 56123663 | C | G | 136 | Pstpip1 | intronic | . | . | . | 9qB | . | 0 | 1 |
| chr9 | 57434750 | C | T | 60 | Ppcdc | intronic | . | . | . | 9qB | rs220818179 | 0 | 1 |
| chr9 | 57679046 | G | A | 222 | Cyp1a2 | exonic | synonymous SNV | Cyp1a2:NM_009993:exon6:c.C1188T:p.G396G | . | 9qB | . | 0 | 1 |
| chr9 | 58537344 | T | C | 212 | Cd276 | exonic | missense SNV | Cd276:NM_133983:exon2:c.A343G:p.T115A | . | 9qB | . | 0 | 1 |
| chr9 | 59983006 | C | A | 127 | Thsd4 | intronic | . | . | . | 9qB | . | 0 | 1 |
| chr9 | 59983007 | T | A | 128 | Thsd4 | intronic | . | . | . | 9qB | . | 0 | 1 |
| chr9 | 61416522 | C | T | 115 | Tle3 | intronic | . | . | . | 9qB | . | 0 | 1 |
| chr9 | 62776973 | C | T | 128 | Itga11 | intronic | . | . | . | 9qB | . | 0 | 1 |
| chr9 | 63617667 | T | G | 222 | Aagab | intronic | . | . | . | 9qC | . | 0 | 1 |
| chr9 | 64935974 | C | A | 208 | Slc24a1 | exonic | missense SNV | Slc24a1:NM_144813:exon4:c.G1895T:p.S632I | . | 9qC | . | 0 | 1 |
| chr9 | 65123865 | C | T | 66 | Igdcc4 | exonic | missense SNV | Igdcc4:NM_001290315:exon7:c.C1084T:p.R362W,Igdcc4:NM_020043:exon7:c.C1084T:p.R362W | 305 | 9qC | . | 0 | 1 |
| chr9 | 65890470 | A | C | 221 | Trip4,2810417H13I | exonic | missense SNV | 2810417H13Rik:NM_026515:exon1:c.A42C:p.R14S | . | 9qC | . | 0 | 1 |
| chr9 | 66433668 | G | A | 222 | Herc1 | intronic | . | . | . | 9qC | . | 0 | 1 |
| chr9 | 66433669 | G | T | 222 | Herc1 | intronic | . | . | . | 9qC | . | 0 | 1 |
| chr9 | 66753726 | C | T | 222 | Car12 | exonic | missense SNV | Car12:NM_001306148:exon8:c.C782T:p.T261I,Car12:NM_178396:exon8:c.C782T:p.T261I | . | 9qC | . | 0 | 1 |
| chr9 | 66762693 | C | A | 82 | Car12 | intronic | . | . | . | 9qC | . | 0 | 1 |
| chr9 | 67902774 | A | G | 103 | Vps13c | intronic | . | . | . | 9qC | . | 0 | 1 |
| chr9 | 68769284 | C | A | 221 | 9530091C08Rik,Ror | ncRNA_exonic | . | . | . | 9qC | . | 0 | 1 |
| chr9 | 69491341 | G | A | 26.5073 | Anxa2 | intronic | . | . | . | 9qC | . | 0 | 1 |
| chr9 | 70034416 | G | A | 113 | Gcnt3 | exonic | missense SNV | Gcnt3:NM_028087:exon3:c.C869T:p.T290I | . | 9qD | . | 0 | 1 |
| chr9 | 71515237 | A | G | 54 | Myzap | intronic | . | . | . | 9qD | . | 0 | 1 |
| chr9 | 71552232 | C | A | 222 | Myzap | intronic | . | . | . | 9qD | . | 0 | 1 |
| chr9 | 72322368 | T | G | 196 | Zfp280d | intronic | . | . | . | 9qD | . | 0 | 1 |
| chr9 | 72739555 | C | A | 195 | Nedd4 | intronic | . | . | . | 9qD | . | 0 | 1 |
| chr9 | 72740283 | T | A | 221 | Nedd4 | intronic | . | . | . | 9qD | . | 0 | 1 |
| chr9 | 72944802 | T | A | 222 | Pygo1 | exonic | stopgain | Pygo1:NM_028116:exon3:c.T270A:p.Y90X | . | 9qD | . | 0 | 1 |
| chr9 | 73667566 | G | A | 81 | Unc13c | exonic | missense SNV | Unc13c:NM_001081153:exon14:c.C4564T:p.R1522W | . | 9qD | . | 0 | 1 |
| chr9 | 75448376 | C | T | 124 | Leo1 | exonic | missense SNV | Leo1:NM_001039522:exon4:c.C997T:p.P333S | . | 9qD | . | 0 | 1 |
| chr9 | 75450362 | T | A | 49.2542 | Leo1 | intronic | . | . | . | 9qD | . | 0 | 1 |
| chr9 | 76213360 | T | G | 196 | Gfra1 | intronic | . | . | . | 9qD | . | 0 | 1 |
| chr9 | 76999825 | T | A | 31.361 | Tinag | splicing | . | . | . | 9qD | . | 0 | 1 |
| chr9 | 77650070 | G | A | 138 | Klhl31 | exonic | missense SNV | Klhl31:NM_172925:exon2:c.G67A:p.E23K | . | 9qE1 | . | 0 | 1 |
| chr9 | 77793214 | C | T | 115 | Gclc | exonic | missense SNV | Gclc:NM_010295:exon16:c.C1877T:p.A626V | . | 9qE1 | . | 0 | 1 |
| chr9 | 78198407 | A | C | 173 | Gsta4 | intronic | . | . | . | 9qE1 | . | 0 | 1 |
| chr9 | 79651517 | A | C | 184 | Col12a1 | intronic | . | . | . | 9qE1 | . | 0 | 1 |
| chr9 | 79656733 | G | A | 114 | Col12a1 | exonic | synonymous SNV | Col12a1:NM_001290308:exon29:c.C5209T:p.L1737L | . | 9qE1 | . | 0 | 1 |
| chr9 | 79693297 | A | T | 217 | Col12a1 | intronic | . | . | . | 9qE1 | . | 0 | 1 |
| chr9 | 79699477 | C | T | 222 | Col12a1 | exonic | missense SNV | Col12a1:NM_001290308:exon10:c.G1663A:p.D555N | . | 9qE1 | . | 0 | 1 |
| chr9 | 80092279 | T | C | 70 | Senp6 | exonic | missense SNV | Senp6:NM_001311110:exon4:c.T214C:p.S72P,Senp6:NM_146003:exon4:c.T214C:p.S72P | . | 9qE1 | . | 0 | 1 |
| chr9 | 81890478 | C | A | 81 | Mei4 | intronic | . | . | . | 9qE1 | . | 0 | 1 |
| chr9 | 83788418 | G | T | 155 | Elovl4 | intronic | . | . | . | 9qE2 | . | 0 | 1 |
| chr9 | 83868184 | G | T | 222 | Ttk | exonic | stopgain | Ttk:NM_001284272:exon18:c.G2020T:p.E674X,Ttk:NM_001110265:exon19:c.G2191T:p.E731X,Ttk:NM_009[illegible] | . | 9qE2 | . | 0 | 1 |
| chr9 | 83869142 | C | T | 74 | Ttk | intronic | . | . | . | 9qE2 | . | 0 | 1 |
| chr9 | 84045721 | G | T | 81 | Bckdhb | intronic | . | . | . | 9qE2 | . | 0 | 1 |
| chr9 | 86513835 | C | T | 222 | Dopey1 | exonic | synonymous SNV | Dopey1:NM_177208:exon17:c.C2349T:p.H783H | . | 9qE3.1 | rs264571163 | 0 | 1 |
| chr9 | 86678055 | A | T | 222 | Me1 | intronic | . | . | . | 9qE3.1 | . | 0 | 1 |
| chr9 | 86755592 | C | T | 187 | Prss35 | exonic | synonymous SNV | Prss35:NM_178738:exon2:c.C414T:p.F138F | . | 9qE3.1 | . | 0 | 1 |
| chr9 | 86755740 | G | T | 132 | Prss35 | exonic | missense SNV | Prss35:NM_178738:exon2:c.G562T:p.V188L | . | 9qE3.1 | . | 0 | 1 |
| chr9 | 87394562 | C | G | 222 | . | intergenic | . | . | . | 9qE3.1 | . | 0 | 1 |
| chr9 | 88380425 | G | A | 105 | Snx14 | intronic | . | . | . | 9qE3.1 | rs48988651 | 1 | 0 |
| chr9 | 88380426 | G | A | 70 | Snx14 | intronic | . | . | . | 9qE3.1 | rs51295906 | 1 | 0 |
| chr9 | 88480024 | T | A | 123 | Syncrip | intronic | . | . | . | 9qE3.1 | . | 0 | 1 |
| chr9 | 89602916 | C | A | 222 | AF529169 | exonic | stopgain | AF529169:NM_153509:exon2:c.G427T:p.E143X | . | 9qE3.1 | . | 0 | 1 |
| chr9 | 89974734 | C | G | 77 | Rasgrf1 | intronic | . | . | . | 9qE3.1 | . | 0 | 1 |
| chr9 | 90250001 | A | G | 221 | Tbc1d2b | intronic | . | . | . | 9qE3.1 | . | 0 | 1 |
| chr9 | 91097550 | T | A | 152 | . | intergenic | . | . | . | 9qE3.1 | . | 0 | 1 |
| chr9 | 91379379 | A | C | 153 | Zic4 | exonic | missense SNV | Zic4:NM_009576:exon3:c.A665C:p.K222T | . | 9qE3.2 | . | 0 | 1 |
| chr9 | 94520441 | A | G | 221 | 1190002N15Rik | exonic | synonymous SNV | 1190002N15Rik:NM_001033145:exon3:c.T1167C:p.I389I | . | 9qE3.3 | . | 0 | 1 |
| chr9 | 95866816 | A | G | 184 | Atr | intronic | . | . | . | 9qE3.3 | . | 0 | 1 |
| chr9 | 96006661 | A | C | 222 | Xrn1 | intronic | . | . | . | 9qE3.3 | . | 0 | 1 |
| chr9 | 96051546 | G | T | 176 | Xrn1 | intronic | . | . | . | 9qE3.3 | . | 0 | 1 |
| chr9 | 96178912 | C | A | 21.8331 | Gk5 | intronic | . | . | . | 9qE3.3 | . | 0 | 1 |
| chr9 | 96437055 | G | T | 148 | . | intergenic | . | . | . | 9qE3.3 | . | 0 | 1 |
| chr9 | 96569822 | C | T | 160 | Rasa2 | intronic | . | . | . | 9qE3.3 | . | 0 | 1 |
| chr9 | 97532721 | C | T | 124 | Clstn2 | exonic | synonymous SNV | Clstn2:NM_022319:exon7:c.G1119A:p.V373V | . | 9qE3.3 | . | 0 | 1 |
| chr9 | 98585919 | T | G | 222 | Copb2 | intronic | . | . | . | 9qE3.3 | . | 0 | 1 |
| chr9 | 98857413 | G | T | 222 | . | downstream | . | . | . | 9qE3.3 | . | 0 | 1 |
| chr9 | 98904174 | C | A | 222 | 7420426K07Rik | UTR3 | . | . | . | 9qE3.3 | . | 0 | 1 |
| chr9 | 99006835 | G | A | 88 | . | downstream | . | . | . | 9qE3.3 | . | 0 | 1 |
| chr9 | 99468692 | A | G | 222 | Nme9 | exonic | synonymous SNV | Nme9:NM_001165957:exon6:c.A426G:p.Q142Q | . | 9qE3.3 | . | 0 | 1 |
| chr9 | 99699152 | A | C | 139 | Cldn18 | intronic | . | . | . | 9qE3.3 | . | 0 | 1 |
| chr9 | 100486097 | T | G | 115 | Il20rb | intronic | . | . | . | 9qE3.3 | . | 0 | 1 |
| chr9 | 100486290 | C | A | 88 | Il20rb | exonic | missense SNV | Il20rb:NM_001033543:exon1:c.G39T:p.W13C | . | 9qE3.3 | . | 0 | 1 |
| chr9 | 101929533 | A | G | 188 | Ephb1 | intronic | . | . | . | 9qF1 | . | 0 | 1 |
| chr9 | 101963882 | A | G | 222 | Ephb1 | intronic | . | . | . | 9qF1 | . | 0 | 1 |
| chr9 | 102552490 | G | C | 90 | . | intergenic | . | . | . | 9qF1 | . | 0 | 1 |
| chr9 | 103077123 | G | T | 222 | Slco2a1 | intronic | . | . | . | 9qF1 | . | 0 | 1 |
| chr9 | 103323276 | A | G | 222 | Topbp1 | exonic | missense SNV | Topbp1:NM_176979:exon11:c.A1507G:p.K503E | . | 9qF1 | . | 0 | 1 |
| chr9 | 104073753 | T | C | 228 | Acad11 | intronic | . | . | . | 9qF1 | rs46762584 | 1 | 0 |
| chr9 | 104170926 | T | C | 65 | Dnajc13 | intronic | . | . | . | 9qF1 | . | 0 | 1 |
| chr9 | 104238284 | G | A | 93 | Dnajc13 | intronic | . | . | . | 9qF1 | rs37261653 | 0 | 1 |
| chr9 | 105027041 | G | A | 148 | Cpne4 | intronic | . | . | . | 9qF1 | . | 0 | 1 |
| chr9 | 105218627 | G | T | 169 | Nek11 | intronic | . | . | . | 9qF1 | . | 0 | 1 |
| chr9 | 105298063 | C | T | 111 | Nek11 | intronic | . | . | . | 9qF1 | . | 0 | 1 |
| chr9 | 105347965 | A | T | 48.1248 | Nek11 | intronic | . | . | . | 9qF1 | . | 0 | 1 |
| chr9 | 105467436 | C | T | 111 | Atp2c1 | intronic | . | . | . | 9qF1 | rs212541373 | 0 | 1 |
| chr9 | 105467442 | C | T | 113 | Atp2c1 | intronic | . | . | . | 9qF1 | rs231270455 | 0 | 1 |
| chr9 | 105759903 | G | C | 192 | Col6a6 | intronic | . | . | . | 9qF1 | rs250490495 | 1 | 0 |
| chr9 | 105783810 | C | T | 190 | Col6a6 | exonic | missense SNV | Col6a6:NM_001102607:exon4:c.G1099A:p.E367K,Col6a6:NM_172927:exon4:c.G1099A:p.E367K | . | 9qF1 | . | 0 | 1 |
| chr9 | 106176824 | T | C | 44.1676 | Wdr82 | intronic | . | . | . | 9qF1 | . | 0 | 1 |
| chr9 | 106236538 | G | A | 222 | Alas1 | exonic | missense SNV | Alas1:NM_001291835:exon9:c.C1507T:p.R503C,Alas1:NM_020559:exon9:c.C1507T:p.R503C | . | 9qF1 | . | 0 | 1 |
| chr9 | 106435731 | G | T | 52 | Acy1 | intronic | . | . | . | 9qF1 | . | 0 | 1 |

| | | | | | | | | | | | | | |
|---|---|---|---|---|---|---|---|---|---|---|---|---|---|
| chr9 | 106460399 | G | T | 221 | Pcbp4 | exonic | missense SNV | Pcbp4:NM_021567:exon4:c.G108T:p.K36N | . | 9qF1 | . | 0 | 1 |
| chr9 | 106969830 | G | T | 207 | Dock3 | exonic | missense SNV | Dock3:NM_153413:exon25:c.C2599A:p.L867M | . | 9qF1 | . | 0 | 1 |
| chr9 | 107258358 | G | A | 222 | Mapkapk3 | exonic | synonymous SNV | Mapkapk3:NM_178907:exon8:c.C807T:p.N269N | . | 9qF1 | . | 0 | 1 |
| chr9 | 107329231 | G | T | 53 | Hemk1 | intronic | . | . | . | 9qF1 | . | 0 | 1 |
| chr9 | 107347891 | A | G | 34.1854 | 6430571L13Rik | intronic | . | . | . | 9qF1 | . | 0 | 1 |
| chr9 | 107465496 | G | A | 222 | Cacna2d2 | intronic | . | . | . | 9qF1 | . | 0 | 1 |
| chr9 | 107516998 | T | G | 85 | Cacna2d2 | intronic | . | . | . | 9qF1 | . | 0 | 1 |
| chr9 | 107517416 | C | A | 165 | Cacna2d2 | exonic | missense SNV | Cacna2d2:NM_001174047:exon20:c.C1786A:p.R596S,Cacna2d2:NM_001174048:exon20:c.C1786A:p.R596S,( | . | 9qF1 | . | 0 | 1 |
| chr9 | 107526950 | C | T | 137 | Cacna2d2 | exonic | stopgain | Cacna2d2:NM_001174048:exon36:c.C3124T:p.Q1042X,Cacna2d2:NM_001174049:exon36:c.C3121T:p.Q1041) | 394 | 9qF1 | . | 0 | 1 |
| chr9 | 107547508 | G | A | 199 | Zmynd10 | exonic | missense SNV | Zmynd10:NM_053253:exon1:c.G52A:p.G18S | . | 9qF1 | . | 0 | 1 |
| chr9 | 107655711 | G | T | 162 | Slc38a3 | intronic | . | . | . | 9qF1 | . | 0 | 1 |
| chr9 | 107898783 | G | A | 222 | Mon1a | exonic | synonymous SNV | Mon1a:NM_028369:exon2:c.G99A:p.T33T | . | 9qF1 | . | 0 | 1 |
| chr9 | 107978689 | A | G | 203 | Uba7 | exonic | missense SNV | Uba7:NM_023738:exon12:c.A1336G:p.R446G | . | 9qF1 | . | 0 | 1 |
| chr9 | 108093842 | C | T | 154 | Apeh | intronic | . | . | . | 9qF1 | . | 0 | 1 |
| chr9 | 108106805 | G | A | 221 | Bsn | exonic | missense SNV | Bsn:NM_007567:exon6:c.C10049T:p.P3350L | . | 9qF1 | . | 0 | 1 |
| chr9 | 108114761 | C | A | 222 | Bsn | exonic | missense SNV | Bsn:NM_007567:exon5:c.G3791T:p.G1264V | . | 9qF1 | . | 0 | 1 |
| chr9 | 108510206 | A | T | 133 | Qars | intronic | . | . | . | 9qF2 | . | 0 | 1 |
| chr9 | 108556021 | G | C | 72 | Qrich1 | exonic | missense SNV | Qrich1:NM_175143:exon7:c.G1849C:p.A617P,Qrich1:NM_001114119:exon8:c.G1849C:p.A617P | . | 9qF2 | . | 0 | 1 |
| chr9 | 108568320 | G | A | 103 | Mir191 | ncRNA exonic | . | . | . | 9qF2 | . | 0 | 1 |
| chr9 | 108580686 | C | T | 140 | P4htm | intronic | . | . | . | 9qF2 | . | 0 | 1 |
| chr9 | 108582828 | G | C | 24.1736 | P4htm | intronic | . | . | . | 9qF2 | . | 0 | 1 |
| chr9 | 108648916 | G | A | 77 | Arih2 | intronic | . | . | 847 | 9qF2 | . | 0 | 1 |
| chr9 | 108966075 | G | A | 25.6681 | Col7a1 | intronic | . | . | . | 9qF2 | . | 0 | 1 |
| chr9 | 109030596 | T | A | 103 | Pfkfb4 | UTR3 | . | . | . | 9qF2 | . | 0 | 1 |
| chr9 | 109054854 | G | A | 112 | Shisa5 | intronic | . | . | . | 9qF2 | . | 0 | 1 |
| chr9 | 109110296 | A | G | 165 | Plxnb1 | intronic | . | . | . | 9qF2 | . | 0 | 1 |
| chr9 | 109110338 | C | T | 222 | Plxnb1 | intronic | . | . | . | 9qF2 | . | 0 | 1 |
| chr9 | 109145537 | T | C | 228 | Fbxw21 | exonic | missense SNV | Fbxw21:NM_177069:exon7:c.A914G:p.H305R | . | 9qF2 | rs30173188 | 1 | 0 |
| chr9 | 109221833 | T | C | 202 | Fbxw20 | exonic | missense SNV | Fbxw20:NM_001008428:exon8:c.A1006G:p.S336G | . | 9qF2 | . | 0 | 1 |
| chr9 | 109224246 | T | A | 67 | Fbxw20 | intronic | . | . | . | 9qF2 | . | 0 | 1 |
| chr9 | 109663264 | G | T | 128 | . | intergenic | . | . | . | 9qF2 | . | 0 | 1 |
| chr9 | 109664512 | T | G | 24.8515 | . | intergenic | . | . | . | 9qF2 | . | 0 | 1 |
| chr9 | 110594485 | G | T | 112 | Setd2 | exonic | missense SNV | Setd2:NM_001081340:exon15:c.G6607T:p.A2203S | . | 9qF2 | . | 0 | 1 |
| chr9 | 110896015 | C | T | 78 | Als2cl | intronic | . | . | . | 9qF2 | . | 0 | 1 |
| chr9 | 111055812 | G | A | 205 | Ccrl2 | exonic | missense SNV | Ccrl2:NM_017466:exon2:c.C617T:p.S206L,Ccrl2:NM_001302376:exon3:c.C617T:p.S206L,Ccrl2:NM_0013( | . | 9qF2 | . | 0 | 1 |
| chr9 | 111174270 | T | C | 221 | Lrrfip2 | intronic | . | . | . | 9qF3 | . | 0 | 1 |
| chr9 | 111190482 | T | G | 109 | Lrrfip2 | intronic | . | . | . | 9qF3 | . | 0 | 1 |
| chr9 | 111389438 | G | T | 32.3896 | Trank1 | intronic | . | . | . | 9qF3 | . | 0 | 1 |
| chr9 | 111390716 | G | C | 221 | Trank1 | exonic | missense SNV | Trank1:NM_001164659:exon20:c.G6520C:p.D2174H | . | 9qF3 | . | 0 | 1 |
| chr9 | 111467727 | C | T | 176 | Dclk3 | exonic | missense SNV | Dclk3:NM_172928:exon2:c.C338T:p.T113I | . | 9qF3 | . | 0 | 1 |
| chr9 | 112177019 | A | T | 221 | Arpp21 | intronic | . | . | . | 9qF3 | . | 0 | 1 |
| chr9 | 112177067 | T | C | 130 | Arpp21 | intronic | . | . | . | 9qF3 | . | 0 | 1 |
| chr9 | 113531300 | A | T | 221 | AU023762 | ncRNA intronic | . | . | . | 9qF3 | . | 0 | 1 |
| chr9 | 113687742 | A | C | 81 | Pdcd6ip | intronic | . | . | . | 9qF3 | . | 0 | 1 |
| chr9 | 113697609 | T | C | 219 | Pdcd6ip | intronic | . | . | . | 9qF3 | . | 0 | 1 |
| chr9 | 113854092 | G | A | 55 | Clasp2 | intronic | . | . | . | 9qF3 | . | 0 | 1 |
| chr9 | 113958425 | G | A | 125 | Mir5128,Ubp1 | ncRNA intronic | . | . | . | 9qF3 | . | 0 | 1 |
| chr9 | 113959453 | C | T | 166 | Mir5128,Ubp1 | exonic | synonymous SNV | Ubp1:NM_001083319:exon9:c.C897T:p.D299D | . | 9qF3 | . | 0 | 1 |
| chr9 | 113977204 | G | A | 222 | Mir5128,Fbxl2 | ncRNA intronic | . | . | . | 9qF3 | . | 0 | 1 |
| chr9 | 114492533 | C | T | 146 | Ccr4 | exonic | missense SNV | Ccr4:NM_009916:exon2:c.G463A:p.V155I | . | 9qF3 | . | 0 | 1 |
| chr9 | 114615015 | T | G | 222 | Cnot10 | exonic | synonymous SNV | Cnot10:NM_153585:exon11:c.A1311C:p.S437S | . | 9qF3 | . | 0 | 1 |
| chr9 | 116131545 | C | A | 123 | Tgfbr2 | exonic | missense SNV | Tgfbr2:NM_029575:exon2:c.G263T:p.W88L,Tgfbr2:NM_009371:exon3:c.G338T:p.W113L | . | 9qF3 | . | 0 | 1 |
| chr9 | 116636561 | G | C | 116 | Rbms3 | intronic | . | . | . | 9qF3 | . | 0 | 1 |
| chr9 | 116695816 | T | G | 169 | Rbms3 | splicing | . | . | . | 9qF3 | . | 0 | 1 |
| chr9 | 118480448 | G | C | 222 | Eomes | intronic | . | . | . | 9qF3 | . | 0 | 1 |
| chr9 | 118485093 | T | G | 127 | Eomes | UTR3 | . | . | . | 9qF3 | . | 0 | 1 |
| chr9 | 118661582 | G | A | 95 | Itga9 | intronic | . | . | . | 9qF3 | . | 0 | 1 |
| chr9 | 118850715 | C | A | 202 | Itga9 | exonic | missense SNV | Itga9:NM_001113514:exon9:c.C695A:p.P232H,Itga9:NM_133721:exon23:c.C2453A:p.P818H | . | 9qF3 | . | 0 | 1 |
| chr9 | 119138187 | G | A | 143 | Dlec1 | exonic | missense SNV | Dlec1:NM_177117:exon25:c.G3640A:p.V1214M | . | 9qF3 | . | 0 | 1 |
| chr9 | 119154070 | T | G | 78 | Acaa1b | intronic | . | . | . | 9qF3 | . | 0 | 1 |
| chr9 | 119357714 | G | T | 190 | Xylb | intronic | . | . | 457 | 9qF3 | . | 0 | 1 |
| chr9 | 119486013 | G | C | 222 | Scn5a | exonic | missense SNV | Scn5a:NM_001253860:exon27:c.C5628G:p.I1876M,Scn5a:NM_021544:exon28:c.C5628G:p.I1876M | . | 9qF3 | . | 0 | 1 |
| chr9 | 119624050 | G | C | 128 | Scn10a | exonic | missense SNV | Scn10a:NM_001205321:exon22:c.C3778G:p.R1260G,Scn10a:NM_009134:exon22:c.C3775G:p.R1259G | . | 9qF3 | . | 0 | 1 |
| chr9 | 119661339 | G | A | 222 | Scn10a | exonic | synonymous SNV | Scn10a:NM_001205321:exon12:c.C1536T:p.D512D,Scn10a:NM_009134:exon12:c.C1536T:p.D512D | . | 9qF3 | . | 0 | 1 |
| chr9 | 119917189 | G | A | 55 | Wdr48 | exonic | missense SNV | Wdr48:NM_026236:exon13:c.G1360A:p.D454N | . | 9qF3 | . | 0 | 1 |
| chr9 | 119972864 | G | T | 37.8567 | Csrnp1 | exonic | missense SNV | Csrnp1:NM_153287:exon5:c.C1128A:p.S376R | . | 9qF4 | . | 0 | 1 |
| chr9 | 120016907 | T | G | 228 | Xirp1 | exonic | missense SNV | Xirp1:NM_011724:exon2:c.A2909C:p.Q970P | . | 9qF4 | rs49019729 | 1 | 0 |
| chr9 | 120135273 | G | T | 126 | . | intergenic | . | . | . | 9qF4 | . | 0 | 1 |
| chr9 | 120316646 | A | G | 44.092 | Myrip | intronic | . | . | . | 9qF4 | . | 0 | 1 |
| chr9 | 120388403 | G | A | 96 | Myrip | intronic | . | . | . | 9qF4 | . | 0 | 1 |
| chr9 | 120543932 | G | T | 44.4789 | Entpd3 | intronic | . | . | . | 9qF4 | . | 0 | 1 |
| chr9 | 120955270 | G | T | 70 | Ctnnb1 | intronic | . | . | . | 9qF4 | . | 0 | 1 |
| chr9 | 121141842 | A | C | 222 | Ulk4 | exonic | synonymous SNV | Ulk4:NM_177589:exon30:c.T3063G:p.A1021A | . | 9qF4 | rs51753015 | 0 | 1 |
| chr9 | 121141866 | G | T | 222 | Ulk4 | exonic | synonymous SNV | Ulk4:NM_177589:exon30:c.C3039A:p.V1013V | . | 9qF4 | rs49305715 | 0 | 1 |
| chr9 | 121144821 | A | G | 96 | Ulk4 | intronic | . | . | . | 9qF4 | rs50027962 | 0 | 1 |
| chr9 | 121152039 | G | A | 222 | Ulk4 | intronic | . | . | . | 9qF4 | rs46926320 | 0 | 1 |
| chr9 | 121156932 | C | A | 222 | Ulk4 | intronic | . | . | . | 9qF4 | rs30521176 | 0 | 1 |
| chr9 | 121157099 | T | C | 222 | Ulk4 | intronic | . | . | . | 9qF4 | rs29587211 | 0 | 1 |
| chr9 | 121157114 | T | G | 222 | Ulk4 | intronic | . | . | . | 9qF4 | rs29787979 | 0 | 1 |
| chr9 | 121157169 | T | C | 221 | Ulk4 | exonic | synonymous SNV | Ulk4:NM_177589:exon24:c.A2574G:p.S858S | . | 9qF4 | rs30432500 | 0 | 1 |
| chr9 | 121157181 | G | A | 221 | Ulk4 | exonic | synonymous SNV | Ulk4:NM_177589:exon24:c.C2562T:p.H854H | . | 9qF4 | rs29689473 | 0 | 1 |
| chr9 | 121168164 | G | T | 221 | Ulk4 | intronic | . | . | . | 9qF4 | rs247606318 | 0 | 1 |
| chr9 | 121168174 | G | A | 222 | Ulk4 | intronic | . | . | . | 9qF4 | rs50556903 | 0 | 1 |
| chr9 | 121168279 | A | G | 222 | Ulk4 | exonic | synonymous SNV | Ulk4:NM_177589:exon22:c.T2226C:p.D742D | . | 9qF4 | rs45908211 | 0 | 1 |
| chr9 | 121168326 | G | T | 222 | Ulk4 | intronic | . | . | . | 9qF4 | rs49762769 | 0 | 1 |
| chr9 | 121204599 | G | C | 184 | Ulk4 | intronic | . | . | . | 9qF4 | rs33721982 | 0 | 1 |
| chr9 | 121204851 | G | A | 162 | Ulk4 | intronic | . | . | . | 9qF4 | rs29980293 | 0 | 1 |
| chr9 | 121204855 | A | G | 155 | Ulk4 | intronic | . | . | . | 9qF4 | rs29694768 | 0 | 1 |
| chr9 | 121208048 | C | T | 129 | Ulk4 | intronic | . | . | . | 9qF4 | rs30374080 | 0 | 1 |
| chr9 | 121208154 | C | T | 222 | Ulk4 | splicing | . | . | . | 9qF4 | rs29837379 | 0 | 1 |
| chr9 | 121208169 | A | G | 222 | Ulk4 | exonic | synonymous SNV | Ulk4:NM_177589:exon18:c.T1752C:p.L584L | . | 9qF4 | rs29843675 | 0 | 1 |
| chr9 | 121208343 | G | A | 196 | Ulk4 | intronic | . | . | . | 9qF4 | rs47599891 | 0 | 1 |
| chr9 | 121244381 | A | G | 222 | Ulk4 | exonic | synonymous SNV | Ulk4:NM_177589:exon17:c.T1641C:p.N547N | . | 9qF4 | rs51867256 | 0 | 1 |
| chr9 | 121244432 | A | C | 222 | Ulk4 | exonic | synonymous SNV | Ulk4:NM_177589:exon17:c.T1590G:p.V530V | . | 9qF4 | rs47643585 | 0 | 1 |
| chr9 | 121244454 | G | C | 222 | Ulk4 | intronic | . | . | . | 9qF4 | rs49135537 | 0 | 1 |
| chr9 | 121248767 | G | T | 115 | Ulk4 | intronic | . | . | . | 9qF4 | rs30084319 | 0 | 1 |
| chr9 | 121248823 | G | A | 76 | Ulk4 | intronic | . | . | . | 9qF4 | rs30427992 | 0 | 1 |
| chr9 | 121248856 | T | C | 123 | Ulk4 | intronic | . | . | . | 9qF4 | rs29974478 | 0 | 1 |
| chr9 | 121248867 | A | G | 137 | Ulk4 | intronic | . | . | . | 9qF4 | rs33732866 | 0 | 1 |
| chr9 | 121248895 | A | T | 214 | Ulk4 | intronic | . | . | . | 9qF4 | rs29843775 | 0 | 1 |
| chr9 | 121250739 | C | G | 59 | Ulk4 | intronic | . | . | . | 9qF4 | rs30466330 | 0 | 1 |
| chr9 | 121250756 | A | C | 70 | Ulk4 | intronic | . | . | . | 9qF4 | rs48892020 | 0 | 1 |
| chr9 | 121250773 | T | C | 76 | Ulk4 | intronic | . | . | . | 9qF4 | rs46574214 | 0 | 1 |
| chr9 | 121250994 | C | T | 73 | Ulk4 | exonic | synonymous SNV | Ulk4:NM_177589:exon15:c.G1398A:p.Q466Q | . | 9qF4 | rs29930759 | 0 | 1 |
| chr9 | 121251006 | A | G | 142 | Ulk4 | exonic | synonymous SNV | Ulk4:NM_177589:exon15:c.T1386C:p.N462N | . | 9qF4 | rs30364145 | 0 | 1 |
| chr9 | 121252577 | C | T | 222 | Ulk4 | intronic | . | . | . | 9qF4 | rs51521978 | 0 | 1 |
| chr9 | 121252598 | G | T | 222 | Ulk4 | intronic | . | . | . | 9qF4 | rs49573946 | 0 | 1 |
| chr9 | 121252663 | T | C | 222 | Ulk4 | exonic | synonymous SNV | Ulk4:NM_177589:exon14:c.A1338G:p.P446P | . | 9qF4 | rs48173655 | 0 | 1 |
| chr9 | 121252791 | T | A | 222 | Ulk4 | intronic | . | . | . | 9qF4 | rs47014910 | 0 | 1 |
| chr9 | 121255277 | T | A | 222 | Ulk4 | intronic | . | . | . | 9qF4 | rs50887909 | 0 | 1 |
| chr9 | 121255287 | T | C | 161 | Ulk4 | intronic | . | . | . | 9qF4 | rs51427309 | 0 | 1 |
| chr9 | 121256442 | G | A | 222 | Ulk4 | intronic | . | . | . | 9qF4 | rs6308415 | 0 | 1 |
| chr9 | 121256474 | G | A | 157 | Ulk4 | intronic | . | . | . | 9qF4 | rs6308471 | 0 | 1 |
| chr9 | 121256570 | C | T | 39.4678 | Ulk4 | intronic | . | . | . | 9qF4 | rs6309010 | 0 | 1 |
| chr9 | 121256918 | G | A | 65 | Ulk4 | intronic | . | . | . | 9qF4 | rs6311043 | 0 | 1 |
| chr9 | 121256954 | G | A | 113 | Ulk4 | intronic | . | . | . | 9qF4 | rs50343838 | 0 | 1 |
| chr9 | 121257049 | C | T | 222 | Ulk4 | intronic | . | . | . | 9qF4 | rs51916119 | 0 | 1 |
| chr9 | 121257171 | A | T | 222 | Ulk4 | intronic | . | . | . | 9qF4 | rs48445124 | 0 | 1 |
| chr9 | 121257218 | G | A | 159 | Ulk4 | intronic | . | . | . | 9qF4 | rs48195362 | 0 | 1 |
| chr9 | 121257233 | A | G | 21.6957 | Ulk4 | intronic | . | . | . | 9qF4 | rs47570650 | 0 | 1 |
| chr9 | 121257248 | C | G | 34.0866 | Ulk4 | intronic | . | . | . | 9qF4 | rs46575179 | 0 | 1 |
| chr9 | 121257322 | C | T | 222 | Ulk4 | exonic | synonymous SNV | Ulk4:NM_177589:exon10:c.G993A:p.R331R | . | 9qF4 | rs51328154 | 0 | 1 |
| chr9 | 121257392 | G | A | 222 | Ulk4 | exonic | missense SNV | Ulk4:NM_177589:exon10:c.C923T:p.P308L | . | 9qF4 | rs46953710 | 0 | 1 |
| chr9 | 121257544 | C | T | 148 | Ulk4 | intronic | . | . | . | 9qF4 | rs50063287 | 0 | 1 |
| chr9 | 121257552 | T | C | 149 | Ulk4 | intronic | . | . | . | 9qF4 | rs47423462 | 0 | 1 |
| chr9 | 121257608 | A | G | 31.97 | Ulk4 | intronic | . | . | . | 9qF4 | rs51172011 | 0 | 1 |
| chr9 | 121258716 | T | C | 81 | Ulk4 | intronic | . | . | . | 9qF4 | rs221566742 | 0 | 1 |
| chr9 | 121259984 | C | A | 141 | Ulk4 | intronic | . | . | . | 9qF4 | rs219064152 | 0 | 1 |
| chr9 | 121260162 | G | A | 147 | Ulk4 | intronic | . | . | . | 9qF4 | rs33648224 | 0 | 1 |
| chr9 | 121260173 | T | A | 26.5127 | Ulk4 | intronic | . | . | . | 9qF4 | rs33679047 | 0 | 1 |
| chr9 | 121262100 | G | A | 222 | Ulk4 | intronic | . | . | . | 9qF4 | rs33897511 | 0 | 1 |
| chr9 | 121262619 | C | T | 222 | Ulk4 | exonic | missense SNV | Ulk4:NM_177589:exon6:c.G592A:p.V198I | . | 9qF4 | rs33897060 | 0 | 1 |
| chr9 | 121431396 | C | T | 212 | Trak1 | intronic | . | . | . | 9qF4 | rs29695924 | 0 | 1 |
| chr9 | 121431513 | G | A | 222 | Trak1 | exonic | synonymous SNV | Trak1:NM_175114:exon3:c.G318A:p.K106K | . | 9qF4 | rs29685754 | 0 | 1 |
| chr9 | 121445824 | C | T | 222 | Trak1 | exonic | synonymous SNV | Trak1:NM_175114:exon7:c.C697T:p.L233L | . | 9qF4 | rs29691368 | 0 | 1 |
| chr9 | 121493271 | T | C | 39.2511 | Cck | intronic | . | . | . | 9qF4 | rs36535739 | 0 | 1 |
| chr9 | 121493350 | A | C | 222 | Cck | intronic | . | . | . | 9qF4 | rs36999746 | 0 | 1 |
| chr9 | 121493387 | A | G | 222 | Cck | exonic | missense SNV | Cck:NM_001284508:exon3:c.T359C:p.V120A | . | 9qF4 | rs37300721 | 0 | 1 |
| chr9 | 121493547 | G | C | 222 | Cck | intronic | . | . | . | 9qF4 | rs38393603 | 0 | 1 |
| chr9 | 121493632 | T | G | 222 | Cck | intronic | . | . | . | 9qF4 | rs36259456 | 0 | 1 |
| chr9 | 121493640 | G | A | 186 | Cck | intronic | . | . | . | 9qF4 | rs49375075 | 0 | 1 |
| chr9 | 121493744 | C | A | 24.9269 | Cck | intronic | . | . | . | 9qF4 | rs38375748 | 0 | 1 |
| chr9 | 121493768 | C | T | 22.0166 | Cck | intronic | . | . | . | 9qF4 | rs37490393 | 0 | 1 |
| chr9 | 121584034 | A | G | 221 | Lyzl4 | intronic | . | . | . | 9qF4 | rs47636656 | 0 | 1 |
| chr9 | 121658114 | G | A | 217 | Vipr1 | exonic | synonymous SNV | Vipr1:NM_011703:exon3:c.G252A:p.L84L | . | 9qF4 | rs251378625 | 0 | 1 |
| chr9 | 121664562 | C | T | 222 | Vipr1 | exonic | synonymous SNV | Vipr1:NM_011703:exon7:c.C678T:p.C226C | . | 9qF4 | rs30425849 | 0 | 1 |
| chr9 | 121665634 | G | A | 222 | Vipr1 | intronic | . | . | . | 9qF4 | rs6412597 | 0 | 1 |
| chr9 | 121665941 | C | T | 222 | Vipr1 | intronic | . | . | . | 9qF4 | rs30331543 | 0 | 1 |
| chr9 | 121668359 | A | G | 144 | Vipr1 | intronic | . | . | . | 9qF4 | rs30087071 | 0 | 1 |
| chr9 | 121668373 | A | G | 151 | Vipr1 | intronic | . | . | . | 9qF4 | rs29596874 | 0 | 1 |
| chr9 | 121668391 | A | G | 170 | Vipr1 | intronic | . | . | . | 9qF4 | rs47119016 | 0 | 1 |
| chr9 | 121668473 | T | C | 222 | Vipr1 | exonic | synonymous SNV | Vipr1:NM_011703:exon11:c.T1056C:p.F352F | . | 9qF4 | rs46588209 | 0 | 1 |
| chr9 | 121668539 | T | C | 222 | Vipr1 | exonic | synonymous SNV | Vipr1:NM_011703:exon11:c.T1122C:p.F374F | . | 9qF4 | rs49140791 | 0 | 1 |
| chr9 | 121668594 | A | G | 222 | Vipr1 | intronic | . | . | . | 9qF4 | rs50405379 | 0 | 1 |
| chr9 | 121668828 | A | G | 148 | Vipr1 | intronic | . | . | . | 9qF4 | rs46220915 | 0 | 1 |
| chr9 | 121669445 | A | C | 28.9672 | Vipr1 | exonic | synonymous SNV | Vipr1:NM_011703:exon13:c.A1260C:p.S420S | 250 | 9qF4 | rs46824062 | 0 | 1 |
| chr9 | 121672623 | C | T | 222 | Vipr1 | UTR3 | . | . | . | 9qF4 | rs49606460 | 0 | 1 |
| chr9 | 121684823 | C | A | 222 | Sec22c | exonic | synonymous SNV | Sec22c:NM_001164562:exon7:c.G747T:p.T249T,Sec22c:NM_178677:exon8:c.G747T:p.T249T | . | 9qF4 | rs29942070 | 0 | 1 |
| chr9 | 121690075 | G | A | 36.8865 | Sec22c | intronic | . | . | . | 9qF4 | rs30335903 | 0 | 1 |
| chr9 | 121690184 | G | A | 192 | Sec22c | intronic | . | . | . | 9qF4 | rs29597286 | 0 | 1 |
| chr9 | 121690310 | G | T | 222 | Sec22c | exonic | missense SNV | Sec22c:NM_001164562:exon4:c.C452A:p.A151E,Sec22c:NM_178677:exon5:c.C452A:p.A151E | . | 9qF4 | rs30124189 | 0 | 1 |
| chr9 | 121692780 | A | G | 51 | Sec22c | intronic | . | . | . | 9qF4 | rs29877681 | 0 | 1 |
| chr9 | 121693112 | G | A | 189 | Sec22c | intronic | . | . | . | 9qF4 | rs30419166 | 0 | 1 |
| chr9 | 121693117 | A | G | 196 | Sec22c | intronic | . | . | . | 9qF4 | rs30383507 | 0 | 1 |
| chr9 | 121693123 | C | G | 150 | Sec22c | intronic | . | . | . | 9qF4 | rs30178635 | 0 | 1 |
| chr9 | 121695689 | G | A | 222 | Sec22c | exonic | synonymous SNV | Sec22c:NM_001164562:exon2:c.C27T:p.I9I,Sec22c:NM_178677:exon3:c.C27T:p.I9I | . | 9qF4 | rs33726720 | 0 | 1 |
| chr9 | 121695816 | T | C | 185 | Sec22c | intronic | . | . | . | 9qF4 | rs30189663 | 0 | 1 |
| chr9 | 121742563 | A | C | 65 | Nktr | splicing | . | . | . | 9qF4 | . | 0 | 1 |
| chr9 | 121742594 | T | C | 83 | Nktr | exonic | missense SNV | Nktr:NM_010918:exon9:c.T580C:p.C194R | . | 9qF4 | rs52463622 | 0 | 1 |
| chr9 | 121742686 | A | G | 222 | Nktr | exonic | synonymous SNV | Nktr:NM_010918:exon9:c.A672G:p.R224R | . | 9qF4 | rs49796365 | 0 | 1 |
| chr9 | 121742713 | A | G | 222 | Nktr | exonic | synonymous SNV | Nktr:NM_010918:exon9:c.A699G:p.R233R | . | 9qF4 | rs48800476 | 0 | 1 |
| chr9 | 121746615 | G | C | 222 | Nktr | intronic | . | . | . | 9qF4 | rs29984153 | 0 | 1 |
| chr9 | 121748301 | T | C | 113 | Nktr | exonic | synonymous SNV | Nktr:NM_010918:exon13:c.T1434C:p.S478S | . | 9qF4 | rs46763746 | 0 | 1 |
| chr9 | 121763269 | G | A | 222 | Zfp651 | exonic | synonymous SNV | Zfp651:NM_001166644:exon2:c.G654A:p.E218E | . | 9qF4 | rs30435446 | 0 | 1 |
| chr9 | 121763856 | G | A | 222 | Zfp651 | exonic | missense SNV | Zfp651:NM_001166644:exon2:c.G1241A:p.R414H | . | 9qF4 | rs51287661 | 0 | 1 |
| chr9 | 121764076 | T | C | 222 | Zfp651 | exonic | synonymous SNV | Zfp651:NM_001166644:exon2:c.T1461C:p.S487S | . | 9qF4 | rs30229525 | 0 | 1 |
| chr9 | 121764254 | C | T | 41.9188 | Zfp651 | intronic | . | . | . | 9qF4 | rs33756945 | 0 | 1 |
| chr9 | 121765670 | A | C | 222 | Zfp651 | intronic | . | . | . | 9qF4 | rs30230316 | 0 | 1 |
| chr9 | 121765693 | C | T | 222 | Zfp651 | intronic | . | . | . | 9qF4 | rs30034964 | 0 | 1 |
| chr9 | 121766373 | A | G | 120 | Zfp651 | intronic | . | . | . | 9qF4 | rs29704594 | 0 | 1 |
| chr9 | 121767213 | A | G | 222 | Zfp651 | intronic | . | . | . | 9qF4 | rs29744681 | 0 | 1 |
| chr9 | 121767386 | G | A | 222 | Zfp651 | splicing | . | . | . | 9qF4 | rs30388740 | 0 | 1 |
| chr9 | 121777907 | C | T | 124 | Klhl40 | exonic | synonymous SNV | Klhl40:NM_028202:exon1:c.C132T:p.F44F | 761 | 9qF4 | rs29795108 | 0 | 1 |
| chr9 | 121788962 | T | C | 222 | Hhatl | intronic | . | . | 242 | 9qF4 | rs237463107 | 0 | 1 |
| chr9 | 121789295 | C | T | 168 | Hhatl | intronic | . | . | . | 9qF4 | rs33630255 | 0 | 1 |

| | | | | | | | | | | | | | |
|---|---|---|---|---|---|---|---|---|---|---|---|---|---|
| chr9 | 121791322 | T | C | 73 | Hhatl | intronic | . | . | . | 9qF4 | rs29936094 | 0 | 1 |
| chr9 | 121824798 | T | C | 125 | Ccdc13 | intronic | . | . | . | 9qF4 | rs29928544 | 0 | 1 |
| chr9 | 121827401 | C | T | 222 | Ccdc13 | intronic | . | . | . | 9qF4 | rs46134568 | 0 | 1 |
| chr9 | 121827443 | C | A | 222 | Ccdc13 | intronic | . | . | . | 9qF4 | rs47477703 | 0 | 1 |
| chr9 | 121849403 | A | G | 115 | Higd1a | UTR3 | . | . | . | 9qF4 | rs29831823 | 0 | 1 |
| chr9 | 121849667 | C | T | 222 | Higd1a | exonic | synonymous SNV | Higd1a:NM_019814:exon4:c.G255A:p.Q85Q | . | 9qF4 | rs13473347 | 0 | 1 |
| chr9 | 121849796 | G | A | 169 | Higd1a | intronic | . | . | . | 9qF4 | rs29882228 | 0 | 1 |
| chr9 | 121850020 | T | C | 131 | Higd1a | intronic | . | . | . | 9qF4 | rs30470147 | 0 | 1 |
| chr9 | 121852336 | T | C | 154 | Higd1a | intronic | . | . | . | 9qF4 | rs29749919 | 0 | 1 |
| chr9 | 121915155 | T | C | 222 | Cyp8b1 | exonic | synonymous SNV | Cyp8b1:NM_010012:exon1:c.A1110G:p.G370G | . | 9qF4 | rs30280503 | 0 | 1 |
| chr9 | 121915254 | T | G | 222 | Cyp8b1 | exonic | synonymous SNV | Cyp8b1:NM_010012:exon1:c.A1011C:p.P337P | . | 9qF4 | rs30227435 | 0 | 1 |
| chr9 | 121915541 | T | C | 222 | Cyp8b1 | exonic | missense SNV | Cyp8b1:NM_010012:exon1:c.A724G:p.K242E | . | 9qF4 | rs29987659 | 0 | 1 |
| chr9 | 121915557 | A | G | 222 | Cyp8b1 | exonic | synonymous SNV | Cyp8b1:NM_010012:exon1:c.T708C:p.H236H | . | 9qF4 | rs30524312 | 0 | 1 |
| chr9 | 121915911 | A | G | 222 | Cyp8b1 | exonic | synonymous SNV | Cyp8b1:NM_010012:exon1:c.T354C:p.D118D | . | 9qF4 | rs13459114 | 0 | 1 |
| chr9 | 122818609 | A | G | 121 | Tcaim | intronic | . | . | . | 9qF4 | rs33525383 | 0 | 1 |
| chr9 | 122852517 | C | T | 222 | Zfp445 | exonic | synonymous SNV | Zfp445:NM_173364:exon7:c.G2358A:p.Q786Q | . | 9qF4 | rs29890772 | 0 | 1 |
| chr9 | 122888350 | C | T | 98 | . | upstream | . | . | . | 9qF4 | rs29699119 | 0 | 1 |
| chr9 | 122888377 | G | A | 116 | . | upstream | . | . | . | 9qF4 | rs30027394 | 0 | 1 |
| chr9 | 122888539 | A | T | 222 | Zkscan7 | UTR5 | . | . | . | 9qF4 | rs29785557 | 0 | 1 |
| chr9 | 122888612 | A | T | 222 | Zkscan7 | exonic | missense SNV | Zkscan7:NM_001177505:exon1:c.A71T:p.N24I | . | 9qF4 | rs33527703 | 0 | 1 |
| chr9 | 122888811 | A | G | 222 | Zkscan7 | exonic | synonymous SNV | Zkscan7:NM_001177505:exon1:c.A270G:p.E90E | . | 9qF4 | rs33528845 | 0 | 1 |
| chr9 | 122888918 | A | G | 222 | Zkscan7 | exonic | missense SNV | Zkscan7:NM_001177505:exon1:c.A377G:p.D126G | . | 9qF4 | rs33528847 | 0 | 1 |
| chr9 | 122890589 | T | C | 177 | Zkscan7 | intronic | . | . | . | 9qF4 | rs224336298 | 0 | 1 |
| chr9 | 122890640 | C | T | 222 | Zkscan7 | intronic | . | . | . | 9qF4 | rs30379977 | 0 | 1 |
| chr9 | 122892281 | G | C | 222 | Zkscan7 | intronic | . | . | . | 9qF4 | rs30179099 | 0 | 1 |
| chr9 | 122894897 | G | A | 222 | Zkscan7 | exonic | synonymous SNV | Zkscan7:NM_001177505:exon5:c.G930A:p.E310E | . | 9qF4 | rs30375414 | 0 | 1 |
| chr9 | 122894954 | G | A | 222 | Zkscan7 | exonic | synonymous SNV | Zkscan7:NM_001177505:exon5:c.G987A:p.V329V | . | 9qF4 | rs216913015 | 0 | 1 |
| chr9 | 122900565 | T | A | 148 | . | intergenic | . | . | . | 9qF4 | rs51048333 | 1 | 0 |
| chr9 | 122925223 | A | C | 222 | Zfp105 | exonic | missense SNV | Zfp105:NM_009544:exon2:c.A116C:p.Y39S | . | 9qF4 | rs6299531 | 0 | 1 |
| chr9 | 122926249 | T | A | 222 | Zfp105 | intronic | . | . | . | 9qF4 | rs33524902 | 0 | 1 |
| chr9 | 122929807 | G | T | 222 | Zfp105 | exonic | missense SNV | Zfp105:NM_009544:exon4:c.G542T:p.C181F | . | 9qF4 | rs29742668 | 0 | 1 |
| chr9 | 122948204 | G | A | 130 | 1110059G10Rik | intronic | . | . | . | 9qF4 | rs239649640 | 0 | 1 |
| chr9 | 122948222 | T | C | 96 | 1110059G10Rik | intronic | . | . | . | 9qF4 | rs33525453 | 0 | 1 |
| chr9 | 122949302 | C | T | 79 | 1110059G10Rik | intronic | . | . | . | 9qF4 | rs252206774 | 0 | 1 |
| chr9 | 122950950 | G | C | 222 | 1110059G10Rik | UTR5 | . | . | 617 | 9qF4 | rs33527950 | 0 | 1 |
| chr9 | 122950952 | A | C | 145 | 1110059G10Rik | UTR5 | . | . | 617 | 9qF4 | . | 0 | 1 |
| chr9 | 122958945 | T | C | 94 | Kif15 | intronic | . | . | . | 9qF4 | rs33526132 | 0 | 1 |
| chr9 | 122959001 | G | A | 116 | Kif15 | intronic | . | . | . | 9qF4 | . | 0 | 1 |
| chr9 | 122959064 | C | A | 169 | Kif15 | intronic | . | . | . | 9qF4 | . | 0 | 1 |
| chr9 | 122959611 | A | G | 89 | Kif15 | intronic | . | . | . | 9qF4 | rs242776089 | 0 | 1 |
| chr9 | 122959974 | G | A | 222 | Kif15 | exonic | missense SNV | Kif15:NM_010620:exon3:c.G230A:p.G77D | . | 9qF4 | . | 0 | 1 |
| chr9 | 122960350 | A | G | 222 | Kif15 | intronic | . | . | . | 9qF4 | . | 0 | 1 |
| chr9 | 122963279 | C | G | 94 | Kif15 | intronic | . | . | . | 9qF4 | rs33526978 | 0 | 1 |
| chr9 | 122963351 | G | C | 133 | Kif15 | intronic | . | . | . | 9qF4 | . | 0 | 1 |
| chr9 | 122963372 | T | C | 137 | Kif15 | intronic | . | . | . | 9qF4 | rs251469105 | 0 | 1 |
| chr9 | 122963574 | T | A | 222 | Kif15 | intronic | . | . | . | 9qF4 | rs246360210 | 0 | 1 |
| chr9 | 122963575 | C | T | 222 | Kif15 | intronic | . | . | . | 9qF4 | rs262968879 | 0 | 1 |
| chr9 | 122971635 | T | G | 97 | Kif15 | intronic | . | . | . | 9qF4 | . | 0 | 1 |
| chr9 | 122971675 | C | T | 174 | Kif15 | intronic | . | . | . | 9qF4 | rs33529577 | 0 | 1 |
| chr9 | 122975938 | G | C | 115 | Kif15 | intronic | . | . | . | 9qF4 | rs33530569 | 0 | 1 |
| chr9 | 122978055 | T | A | 222 | Kif15 | intronic | . | . | . | 9qF4 | . | 0 | 1 |
| chr9 | 122978212 | A | G | 109 | Kif15 | intronic | . | . | . | 9qF4 | . | 0 | 1 |
| chr9 | 122979342 | A | T | 153 | Kif15 | intronic | . | . | . | 9qF4 | . | 0 | 1 |
| chr9 | 122979869 | G | A | 221 | Kif15 | intronic | . | . | . | 9qF4 | rs254404229 | 0 | 1 |
| chr9 | 122984755 | T | C | 222 | Kif15 | intronic | . | . | . | 9qF4 | rs213673821 | 0 | 1 |
| chr9 | 122984972 | G | T | 222 | Kif15 | intronic | . | . | . | 9qF4 | rs221019121 | 0 | 1 |
| chr9 | 122985098 | G | A | 160 | Kif15 | intronic | . | . | . | 9qF4 | . | 0 | 1 |
| chr9 | 122986061 | C | G | 58 | Kif15 | intronic | . | . | . | 9qF4 | rs227581368 | 0 | 1 |
| chr9 | 122986218 | A | G | 217 | Kif15 | exonic | synonymous SNV | Kif15:NM_010620:exon12:c.A1296G:p.K432K | . | 9qF4 | rs254192821 | 0 | 1 |
| chr9 | 122986287 | A | T | 204 | Kif15 | intronic | . | . | . | 9qF4 | . | 0 | 1 |
| chr9 | 122986325 | C | G | 179 | Kif15 | intronic | . | . | . | 9qF4 | . | 0 | 1 |
| chr9 | 122986398 | A | G | 157 | Kif15 | intronic | . | . | . | 9qF4 | rs233761390 | 0 | 1 |
| chr9 | 122986564 | T | G | 222 | Kif15 | exonic | missense SNV | Kif15:NM_010620:exon13:c.T1422G:p.H474Q | . | 9qF4 | rs261768838 | 0 | 1 |
| chr9 | 122987698 | C | T | 126 | Kif15 | intronic | . | . | . | 9qF4 | rs215017476 | 0 | 1 |
| chr9 | 122987815 | G | A | 222 | Kif15 | intronic | . | . | . | 9qF4 | rs236584991 | 0 | 1 |
| chr9 | 122991972 | G | A | 222 | Kif15 | exonic | synonymous SNV | Kif15:NM_010620:exon16:c.G1941A:p.Q647Q | . | 9qF4 | . | 0 | 1 |
| chr9 | 122992157 | C | T | 69 | Kif15 | intronic | . | . | . | 9qF4 | . | 0 | 1 |
| chr9 | 122993748 | C | T | 124 | Kif15 | intronic | . | . | . | 9qF4 | rs214307747 | 0 | 1 |
| chr9 | 122993781 | G | C | 180 | Kif15 | intronic | . | . | . | 9qF4 | . | 0 | 1 |
| chr9 | 122994018 | C | T | 222 | Kif15 | exonic | missense SNV | Kif15:NM_010620:exon17:c.C2147T:p.S716L | . | 9qF4 | . | 0 | 1 |
| chr9 | 122994052 | T | A | 222 | Kif15 | intronic | . | . | . | 9qF4 | rs213114546 | 0 | 1 |
| chr9 | 122994126 | G | A | 104 | Kif15 | intronic | . | . | . | 9qF4 | rs234636791 | 0 | 1 |
| chr9 | 122994132 | G | A | 42.4259 | Kif15 | intronic | . | . | . | 9qF4 | rs253467792 | 0 | 1 |
| chr9 | 122995673 | A | T | 209 | Kif15 | intronic | . | . | . | 9qF4 | . | 0 | 1 |
| chr9 | 122995884 | G | A | 136 | Kif15 | intronic | . | . | . | 9qF4 | rs232938844 | 0 | 1 |
| chr9 | 122995961 | A | G | 78 | Kif15 | intronic | . | . | . | 9qF4 | rs33727382 | 0 | 1 |
| chr9 | 122996258 | T | C | 222 | Kif15 | intronic | . | . | . | 9qF4 | rs33532188 | 0 | 1 |
| chr9 | 122996371 | G | C | 222 | Kif15 | exonic | synonymous SNV | Kif15:NM_010620:exon19:c.G2358C:p.L786L | . | 9qF4 | rs33532192 | 0 | 1 |
| chr9 | 122996450 | G | A | 196 | Kif15 | intronic | . | . | . | 9qF4 | rs236111378 | 0 | 1 |
| chr9 | 122996586 | G | C | 83 | Kif15 | intronic | . | . | . | 9qF4 | rs224796385 | 0 | 1 |
| chr9 | 122996590 | A | G | 88 | Kif15 | intronic | . | . | . | 9qF4 | rs245796802 | 0 | 1 |
| chr9 | 122997716 | T | C | 120 | Kif15 | intronic | . | . | . | 9qF4 | rs582001057 | 0 | 1 |
| chr9 | 122997965 | C | T | 222 | Kif15 | intronic | . | . | . | 9qF4 | rs222639461 | 0 | 1 |
| chr9 | 122997988 | C | T | 222 | Kif15 | intronic | . | . | . | 9qF4 | rs239218401 | 0 | 1 |
| chr9 | 122998055 | T | C | 101 | Kif15 | intronic | . | . | . | 9qF4 | rs225050485 | 0 | 1 |
| chr9 | 122999029 | T | C | 74 | Kif15 | intronic | . | . | . | 9qF4 | rs223713860 | 0 | 1 |
| chr9 | 122999079 | A | G | 97 | Kif15 | intronic | . | . | . | 9qF4 | . | 0 | 1 |
| chr9 | 122999277 | T | C | 222 | Kif15 | exonic | synonymous SNV | Kif15:NM_010620:exon21:c.T2652C:p.N884N | . | 9qF4 | rs33533885 | 0 | 1 |
| chr9 | 122999455 | A | G | 196 | Kif15 | intronic | . | . | . | 9qF4 | rs29599761 | 0 | 1 |
| chr9 | 123046413 | T | G | 222 | Tgm4 | intronic | . | . | . | 9qF4 | rs252175429 | 0 | 1 |
| chr9 | 123061653 | C | A | 222 | Tgm4 | exonic | missense SNV | Tgm4:NM_177911:exon11:c.C1316A:p.A439E | . | 9qF4 | . | 0 | 1 |
| chr9 | 123061822 | C | T | 222 | Tgm4 | exonic | synonymous SNV | Tgm4:NM_177911:exon11:c.C1485T:p.S495S | . | 9qF4 | rs33534626 | 0 | 1 |
| chr9 | 123061915 | A | G | 222 | Tgm4 | intronic | . | . | . | 9qF4 | rs33534628 | 0 | 1 |
| chr9 | 123062011 | C | T | 68 | Tgm4 | intronic | . | . | . | 9qF4 | rs217404428 | 0 | 1 |
| chr9 | 123062074 | G | A | 36.9057 | Tgm4 | intronic | . | . | . | 9qF4 | . | 0 | 1 |
| chr9 | 123062276 | A | G | 125 | Tgm4 | intronic | . | . | . | 9qF4 | . | 0 | 1 |
| chr9 | 123062288 | C | T | 130 | Tgm4 | intronic | . | . | . | 9qF4 | rs249958757 | 0 | 1 |
| chr9 | 123062489 | A | G | 222 | Tgm4 | exonic | synonymous SNV | Tgm4:NM_177911:exon12:c.A1668G:p.V556V | . | 9qF4 | rs33535384 | 0 | 1 |
| chr9 | 123062563 | T | G | 191 | Tgm4 | intronic | . | . | . | 9qF4 | rs33535386 | 0 | 1 |
| chr9 | 123062570 | C | T | 172 | Tgm4 | intronic | . | . | . | 9qF4 | rs223823911 | 0 | 1 |
| chr9 | 123062585 | C | T | 168 | Tgm4 | intronic | . | . | . | 9qF4 | rs246036109 | 0 | 1 |
| chr9 | 123064534 | G | A | 138 | Tgm4 | intronic | . | . | . | 9qF4 | rs33529229 | 0 | 1 |
| chr9 | 123064672 | T | C | 222 | Tgm4 | intronic | . | . | . | 9qF4 | rs256944253 | 0 | 1 |
| chr9 | 123078106 | A | G | 98 | Zdhhc3 | UTR3 | . | . | . | 9qF4 | rs33531394 | 0 | 1 |
| chr9 | 123080535 | C | T | 78 | Zdhhc3 | intronic | . | . | . | 9qF4 | rs256812371 | 0 | 1 |
| chr9 | 123083312 | G | T | 65 | Zdhhc3 | intronic | . | . | . | 9qF4 | rs225663018 | 0 | 1 |
| chr9 | 123084019 | T | C | 158 | Zdhhc3 | intronic | . | . | . | 9qF4 | rs33533440 | 0 | 1 |
| chr9 | 123084166 | A | G | 222 | Zdhhc3 | intronic | . | . | . | 9qF4 | rs254009518 | 0 | 1 |
| chr9 | 123084461 | G | A | 106 | Zdhhc3 | intronic | . | . | . | 9qF4 | rs33534606 | 0 | 1 |
| chr9 | 123100304 | A | G | 222 | Zdhhc3 | exonic | synonymous SNV | Zdhhc3:NM_026917:exon2:c.T265C:p.L89L | . | 9qF4 | rs33533228 | 0 | 1 |
| chr9 | 123113339 | C | T | 171 | Exosc7 | intronic | . | . | 659 | 9qF4 | rs30485593 | 0 | 1 |
| chr9 | 123131856 | C | G | 222 | Exosc7 | intronic | . | . | . | 9qF4 | rs29985379 | 0 | 1 |
| chr9 | 123150993 | T | C | 147 | Clec3b | UTR5 | . | . | . | 9qF4 | rs29989859 | 0 | 1 |
| chr9 | 123151102 | T | C | 222 | Clec3b | exonic | missense SNV | Clec3b:NM_011606:exon1:c.T59C:p.I20T | . | 9qF4 | rs29844383 | 0 | 1 |
| chr9 | 123154259 | C | T | 140 | Clec3b | intronic | . | . | . | 9qF4 | . | 0 | 1 |
| chr9 | 123154364 | G | A | 222 | Clec3b | intronic | . | . | . | 9qF4 | rs30386203 | 0 | 1 |
| chr9 | 123156665 | G | A | 222 | Clec3b | intronic | . | . | . | 9qF4 | rs30211277 | 0 | 1 |
| chr9 | 123157250 | A | G | 67 | Clec3b | UTR3 | . | . | . | 9qF4 | rs240877433 | 0 | 1 |
| chr9 | 123173545 | G | A | 222 | Cdcp1 | exonic | synonymous SNV | Cdcp1:NM_133974:exon9:c.C2460T:p.I820I | . | 9qF4 | . | 0 | 1 |
| chr9 | 123175903 | T | C | 222 | Cdcp1 | intronic | . | . | . | 9qF4 | rs30329397 | 0 | 1 |
| chr9 | 123180205 | C | T | 222 | Cdcp1 | exonic | synonymous SNV | Cdcp1:NM_133974:exon6:c.G1407A:p.Q469Q | . | 9qF4 | rs30479541 | 0 | 1 |
| chr9 | 123183919 | C | T | 222 | Cdcp1 | intronic | . | . | . | 9qF4 | rs246829905 | 0 | 1 |
| chr9 | 123184966 | C | A | 104 | Cdcp1 | intronic | . | . | . | 9qF4 | rs243853114 | 0 | 1 |
| chr9 | 123185168 | A | G | 222 | Cdcp1 | exonic | synonymous SNV | Cdcp1:NM_133974:exon3:c.T540C:p.N180N | . | 9qF4 | rs29843515 | 0 | 1 |
| chr9 | 123185237 | G | A | 222 | Cdcp1 | exonic | synonymous SNV | Cdcp1:NM_133974:exon3:c.C471T:p.D157D | . | 9qF4 | . | 0 | 1 |
| chr9 | 123185247 | C | G | 222 | Cdcp1 | exonic | missense SNV | Cdcp1:NM_133974:exon3:c.G461C:p.S154T | . | 9qF4 | rs238413174 | 0 | 1 |
| chr9 | 123185288 | C | T | 222 | Cdcp1 | exonic | synonymous SNV | Cdcp1:NM_133974:exon3:c.G420A:p.Q140Q | . | 9qF4 | . | 0 | 1 |
| chr9 | 123185549 | T | G | 205 | Cdcp1 | intronic | . | . | . | 9qF4 | rs241580558 | 0 | 1 |
| chr9 | 123185588 | C | T | 111 | Cdcp1 | intronic | . | . | . | 9qF4 | rs260909603 | 0 | 1 |
| chr9 | 123189759 | G | A | 30.1672 | Cdcp1 | intronic | . | . | . | 9qF4 | rs231611188 | 0 | 1 |
| chr9 | 123189774 | G | A | 23.854 | Cdcp1 | intronic | . | . | . | 9qF4 | rs587527803 | 0 | 1 |
| chr9 | 123189844 | C | A | 64 | Cdcp1 | intronic | . | . | . | 9qF4 | rs231013935 | 0 | 1 |
| chr9 | 123190146 | G | A | 222 | Cdcp1 | intronic | . | . | . | 9qF4 | . | 0 | 1 |
| chr9 | 123190175 | G | A | 206 | Cdcp1 | intronic | . | . | . | 9qF4 | rs219364255 | 0 | 1 |
| chr9 | 123190181 | G | T | 192 | Cdcp1 | intronic | . | . | . | 9qF4 | . | 0 | 1 |
| chr9 | 123371787 | C | T | 120 | Lars2 | intronic | . | . | . | 9qF4 | rs38298820 | 0 | 1 |
| chr9 | 123371863 | C | T | 210 | Lars2 | UTR5 | . | . | . | 9qF4 | rs37621289 | 0 | 1 |
| chr9 | 123371921 | A | C | 222 | Lars2 | exonic | synonymous SNV | Lars2:NM_153168:exon2:c.A49C:p.R17R | . | 9qF4 | rs37364009 | 0 | 1 |
| chr9 | 123371936 | A | G | 222 | Lars2 | exonic | missense SNV | Lars2:NM_153168:exon2:c.A64G:p.R22G | . | 9qF4 | rs36883083 | 0 | 1 |
| chr9 | 123377548 | G | C | 96 | Lars2 | intronic | . | . | . | 9qF4 | rs254473629 | 0 | 1 |
| chr9 | 123377844 | T | C | 222 | Lars2 | intronic | . | . | . | 9qF4 | rs231320373 | 0 | 1 |
| chr9 | 123392517 | A | G | 97 | Lars2 | intronic | . | . | . | 9qF4 | rs38143024 | 0 | 1 |
| chr9 | 123392607 | G | A | 159 | Lars2 | intronic | . | . | . | 9qF4 | rs36926312 | 0 | 1 |
| chr9 | 123392778 | G | A | 222 | Lars2 | intronic | . | . | . | 9qF4 | rs37116614 | 0 | 1 |
| chr9 | 123392827 | A | G | 222 | Lars2 | exonic | synonymous SNV | Lars2:NM_153168:exon4:c.A405G:p.E135E | . | 9qF4 | rs38577299 | 0 | 1 |
| chr9 | 123392934 | T | A | 222 | Lars2 | intronic | . | . | . | 9qF4 | rs39480512 | 0 | 1 |
| chr9 | 123393065 | T | C | 99 | Lars2 | intronic | . | . | . | 9qF4 | rs36900729 | 0 | 1 |
[truncated: 415,002 more chars]
